# Supplementary material for: Evidence-guided approach to portfolio-guided teaching and assessing communications, ethics and professionalism for medical students and physicians: a systematic scoping review
Source: BMJ Open. 2023 Mar 28;13(3):e067048. doi: 10.1136/bmjopen-2022-067048 (PMC10069516; doi:10.1136/bmjopen-2022-067048)
Supplement: Supplementary data [file bmjopen-2022-067048supp001.pdf]

<u>Appendices</u>

Appendix A. SEBA Methodology

Theoretical lens

Krishna's Systematic Evidence-Based Approach (SEBA) is adopted to guide this systematic scoping review (SSR) (henceforth SSR in SEBA) (1-8). SEBA is composed of six stages- 1) Systematic Approach, 2) Split Approach, 3) Jigsaw Perspective, 4) Funnelling Process 5) Analysis of data and non-data driven literature, and 6) Discussion Synthesis (Figure 1). SEBA systematically map available data, structure the extraction of key characteristics of CEP education and its effects, synthesise and summarise actionable and applicable information across a diverse range of study formats and identify gaps in knowledge in current concepts. SEBA's constructivist approach (8-13) and relativist lens (14-17) acknowledges development of CEP competencies are individualised sociocultural constructs informed by the user's narratives, clinical competencies, contextual and environmental considerations.

Expert advice

An expert team consisting of a medical librarian from the Yong Loo Lin School of Medicine (YLLSoM) at the National University of Singapore (NUS), and local educational experts and clinicians at the National Cancer Centre Singapore (NCCS), the Palliative Care Unit, and the Institute of Population Health at the University of Liverpool, YLLSoM and Duke-NUS Medical School, ensured that the SEBA methodology was employed in a consistent manner within accepted practices.

Reflexivity

Personal reflexivity

Recognising that the research team are informed by their interests, narratives, clinical and research insights and contextual considerations, membership to the research and expert teams were made up of experienced physician-tutors, psychologists, methodologists, and educational scholars. Six members of these teams hold masters degrees in medical education, two hold masters degrees in ethics, two hold masters degrees in clinical research and two hold masters degrees in public health. Most of the expert and research team were experienced in quantitative research however five members of the team are experienced in qualitative methods. Six of the research and expert teams have published articles in peer-reviewed journals using the SEBA methodology. One member of the expert team is an experienced researcher at the Palliative Care Institute Liverpool and another is a member of the Health Data Science Department at the University of Liverpool. Both researchers from the University of Liverpool have

been collaborating with the research team on a number of studies pertaining to portfolio use, death and dying, moral distress, PIF and mentoring and are part of the team expanding the use of the SEBA methodology in medical education and palliative care.

Three senior members of the team are working on various projects on medical education and three others have already published articles in peer-reviewed journals in medical education. Six members of the research team were members of the PMI and have published SEBA guided reviews on mentoring, dignity and medical education. To ensure input from all members of the team, synchronous and asynchronous in-person and online meetings and Sandelowski and Barroso (18)'s approach to 'negotiated consensual validation' was used to reach consensus on the issues discussed.

Methodological reflexivity

Adopting a structured constructivist approach, we sought to build a holistic concept of current models of portfolios within medical education acknowledging however that we were limited by manpower and time constraints. We included articles featured in grey and bibliographic databases within the dates set in our selection criteria. Much of these theories required deeper consideration and discussions and we documented our discussions and decisions.

Iterative process

To ensure transparency and accountability, the expert team was involved in all stages of SEBA as part of the iterative process.

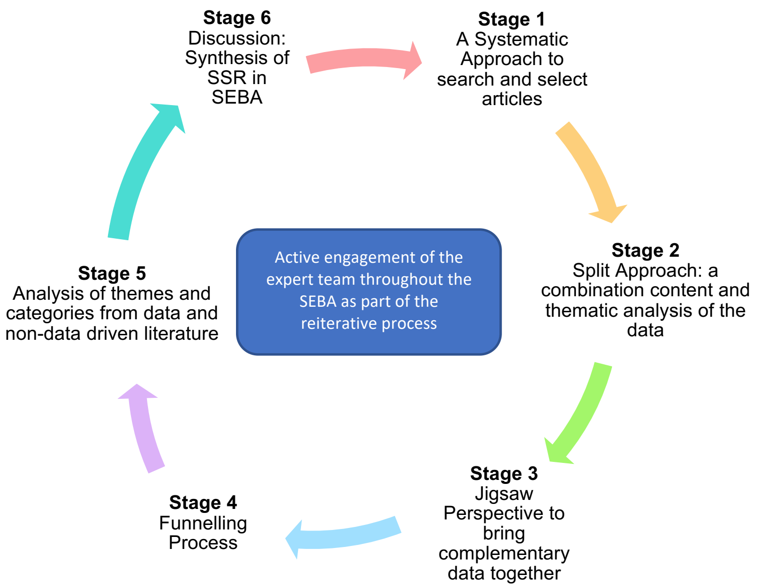

Figure 1. The SEBA Process

Stage 1 of SEBA: Systematic Approach

The SEBA methodology begins with the research and expert teams agreeing upon the research questions, the search terms and the databases to be scrutinised. In this case the research and expert team determined the primary research question: "*What is known about CEP portfolios?*". The secondary research questions were "*what role do CEP portfolios have in teaching and assessing CEP development?*".

Inclusion criteria

A PICOs (Population, Intervention, Comparison, Outcome, Study design) format was adopted to guide the research process (Table 1). There was no comparison group.

Table 1. *PICOS Inclusion and Exclusion Criteria*

PICOS	Inclusion criteria	Exclusion criteria
**Population**	• Undergraduate and postgraduate medical students<br>• Qualified medical doctors, physician or resident; medical officer, registrar, house officer, attending, consultant	• Allied health specialties such as Pharmacy, Dietetics, Chiropractic, Midwifery, Podiatry, Speech Therapy, Occupational and Physiotherapy<br>• Non-medical specialties such as Clinical and Translational Science, Alternative and Traditional Medicine, Veterinary, Dentistry
**Intervention**	• portfolios in undergraduate and postgraduate medical education for teaching and assessment of Communication, Ethics and Professionalism<br><br>Criteria of a portfolio:	Other documentation methods or learning tools that are:<br>• Not longitudinal or single timepoint<br>• Does not include personal intellectual engagement with the content and associated learning (for instance, curriculum vitae, logbooks

PICOS	Inclusion criteria	Exclusion criteria
	• longitudinal (more than a single timepoint) assessment data<br>• candidate's personal engagement with portfolio content and associated learning<br>• Interventions meeting the above criteria were included regardless of whether they were referred to as portfolios<br><br>All types of portfolios were included in the study:<br>• For instance: electronic & non-electronic; formative & summative or combined; clinical & non-clinical<br>• Portfolios with input from students and/or residents and/or doctors, and/or input from faculty members and other individuals<br>• Portfolios with different structures: extent by which the structure has been prescribed and/or left to individual discretion	and the use of personal digital assistants)
**Comparison/ Context**	NA	NA
**Outcome**	Papers that measured the following outcomes were included:<br>• Effectiveness of the use of portfolios to assess and teach Communication, Ethics and Professionalism<br>• Impact of the use of portfolios on medical students (both undergraduate and postgraduate)<br>• Impact of the use of portfolios on the faculty	NA
**Study design**	• Articles in English or translated to English<br>• Articles published from 1st January 2000 to 31st December 2020<br>• Databases: PsycINFO, EMBASE, PubMed, ERIC, Scopus, Google Scholar<br>• All study designs including:<br>• Mixed methods research, meta-analyses, systematic reviews, randomised controlled trials, cohort studies, case-control studies, cross-sectional studies, descriptive papers, grey literature, opinions, letters, commentaries and editorials	NA

Three members of the research team carried out independent ancestry searches of seven leading journals in medical education (Academic Medicine, Medical Education, Medical Teacher, Advances Health Sciences Education, BMC Medical Education, Teaching and Learning in Medicine and Perspectives on Medical Education) accessed through the NUS library portal. In keeping with Pham, Rajic (19)'s recommendations, the searches were restricted to articles published between 1st January 2000 and 31st December 2020 to accommodate to existing manpower and time constraints. Quantitative, mixed and qualitative research methodologies meeting the inclusion criteria were included.

Table 2. *Search Strategy for PubMed, Embase, PsycINFO, ERIC, Scopus, and Google Scholar databases.*

		**Mesh Terms**	**tiab**
		(1 OR 2 OR 3) AND 4	
**Population**	Medical Students OR Doctors	[1] "Physicians"[MeSH] OR "Students, Medical"[MeSH] OR "Clinical Clerkship"[MeSH] OR "Medicine"[Mesh] OR "Education, Medical"[Mesh] OR "Clinical Competence"[Mesh]	[2] Physician[tiab] OR Physicians[tiab] OR resident[tiab] OR residents[tiab] OR residency[tiab] OR residencies[tiab] OR practice[tiab] OR practitioner[tiab] OR practitioners[tiab] OR doctor[tiab] OR doctors[tiab] OR houseman[tiab] OR housemanship[tiab] OR housemen[tiab] OR medical[tiab] OR clinical[tiab] OR pre-clinical[tiab] OR preclinical[tiab] OR clinician*[tiab] OR surgery[tiab] OR surgical[tiab] OR surgeon*[tiab] OR clerkship*[tiab] OR specialist*[tiab]
			[3] ("Educational Measurement/methods"[Mesh] OR "Educational Measurement/standards"[Mesh] OR "Documentation/methods"[Mesh] OR "Benchmarking*"[MeSH] OR "Competency-based education/standards*"[MeSH] OR "Records*"[MeSH]) AND (medical[tiab] OR clinical[tiab] OR pre-clinical[tiab] OR preclinical[tiab] OR clinician*[tiab] OR surgery[tiab] OR surgical[tiab] OR surgeon*[tiab] OR clerkship*[tiab] OR specialist*[tiab])
**Intervention**	Portfolios		[4] Portfolio[tiab] OR portfolios[tiab] OR e-portfolio[tiab] OR e-portfolios[tiab] OR "curriculum vitae"[tiab] OR "personal statement"[tiab] OR "personal statements"[tiab]

Members of the research team carried out independent searches of databases. To facilitate this approach, the search process saw experienced senior researchers well-versed in carrying out systematic reviews and systematic scoping reviews each meet with a team of two to three medical students to guide them database searches. This approach was to enhance training of new researchers and to ensure that at least two teams were independently reviewing each database. Each team met regularly and discussed their findings. After a search of the first 100 articles in a particular database, the medical students and the senior researcher compared their findings at an online meeting.

Subsequently, the teams met at specific time points, often after reviewing a predetermined number of included articles to discuss their concerns, exchange opinions and advance their understanding of the research process and the area of study. Sandelowski and Barroso (18)'s 'negotiated consensual validation' was used to achieve consensus on the final list of titles to be reviewed. The teams repeated this process, independently studying all the

full text articles on the final list of titles, creating their own lists of articles to be included and discussing their findings at weekly online research meetings over the first 4 months of the project. Consensus on the final list of articles to be analysed was achieved following at least three online meetings when discrepancies were reviewed and discussed by each member of the research team.

As this was a training process for many of the participants who were medical students participating in the Palliative Medicine Initiative, senior mentors and peer-mentors were frequently involved in guiding and discussing individual findings over and above the online meetings especially at the start of each stage of the research process. Interrater reliability was not evaluated.

Stage 2 of SEBA: Split Approach

Krishna's 'Split Approach' (18-23) was employed to enhance the reliability of the data analyses. This saw three groups of researchers independently analysing the included articles.

The first team summarised and tabulated the included full-text articles in keeping with recommendations drawn from Wong, Greenhalgh (24)'s RAMESES publication standards: meta-narrative reviews and Popay, Roberts (25)'s "Guidance on the conduct of narrative synthesis in systematic reviews". The tabulated summaries served to ensure that key aspects of included articles were not lost.

Concurrently, the second team analysed the included articles using Braun and Clarke (26)'s approach to thematic analysis. In Phase 1, the research team carried out independent reviews, 'actively' reading the included articles to find meaning and patterns in the data. In Phase 2, 'codes' were constructed from the 'surface' meaning and collated into a code book to code and analyse the rest of the articles using an iterative step-by-step process. As new codes emerged, these were associated with previous codes and concepts. In Phase 3, the categories were organised into themes that best depict the data. An inductive approach allowed themes to be "defined from the raw data without any predetermined classification" (27). In Phase 4, the themes were refined to best represent the whole data set and discussed. In Phase 5, the research team discussed the results of their independent analysis online and at reviewer meetings. 'Negotiated consensual validation' was used to determine a final list of themes approach and ensure the final themes.

A third team of researchers employed Hsieh and Shannon (28)'s approach to directed content analysis to analyse the included articles. Analysis using the directed content analysis approach involved "identifying and operationalizing a priori coding categories". The categories employed in the content analysis for undergraduate communications were Rider et al. (29) 's *"A model for communication skills assessment across the undergraduate curriculum",* Goldie (30)'s *"Review of ethics curricula in undergraduate medical education"*, Duffy et al. (31)'s *"Assessing Competence in Communication and Interpersonal Skills: The Kalamazoo II Report"* and Hong et al. (32)'s *"Postgraduate Ethics Training Programs: A Systematic Scoping Review"*. Tay et al. (33)'s *"Assessing Professionalism in Medicine - A Scoping Review of Assessment Tools from 1990 to 2018"* was employed for codes for professionalism and David (34)'s article *"AMEE Medical Education Guide No. 24: Portfolios as a method of student assessment"* was then used to contextualise their use in portfolios. Any data not captured by these codes were assigned a new code.

By using directed content analysis, this "Split Approach" sought to address shortcomings int thematic analysis. This was done by ironing out disparities in data and increases the validity of the identified themes (35). It also limits inherent biases and condenses the interpretations of terminology used by each team's members. Consistency with existing literature is further offered by directed content analysis, by using existing data to identify codes and categories. The codes were used systematically and objectively, strengthening the validity and reliability of a positivist approach (35, 36). The transparency of this approach is enforced by clearly defined categories, together with references throughout the analytical process.

Stage 3 of SEBA: Jigsaw Perspective

The themes and categories identified in the Split Approach were viewed as pieces of a jigsaw puzzle where overlapping/complementary pieces were combined to create a bigger piece of the puzzle referred to as themes/categories. This process was guided by Phases 4 to 6 of France, Uny (37)'s adaptation of Noblit, Hare (38)'s seven phases of meta-ethnography. As per Phase 4, the themes and the categories identified in the Split Approach are grouped together according to their focus. These groupings of categories and themes were then contextualised through the review of the articles from which they were drawn from. Reciprocal translation was used to determine if the themes and categories could be used interchangeably. This allowed the themes and categories to be combined to form themes/categories.

France, Uny's adaptation:
Phase 1: Getting started, deciding on the focus of the synthesis.
Phase 2: Deciding what is relevant to the initial interest.
Phase 3: Reading the Studies.
Phase 4: Determining how the studies are related.
Phase 5: Translating the studies into one another.
Phase 6: Synthesising the translations.
Phase 7: Expressing the synthesis.

Codes derived from thematic analysis (TA): indications, characteristics and strengths and limitations

Codes derived from directed content analysis (DCA): indication for portfolio, portfolio content, portfolio design and strengths and limitations

Overlaps between indications and strengths and limitations allowed the combination of these categories and themes creating two themes/categories called indications and strengths and limitations. Careful review of the categories- portfolio content and portfolio design were subsets of the theme characteristics. For a more accurate presentation, the overlaps were conflated and presented together with the TA codes indicated under the DCA code. (Refer to Domain 4 table)

**Themes Identified**	**Categories Identified**	**Domains Created**

Indications	indications	Indications
characteristics	content	Characteristics
	Portfolio design	
Strengths and limitations	Strengths and limitations	Dignity-conserving Measures

Stage 4 of SEBA: Funnelling Process

The Funnelling Process employs Phases 3 to 5 of France, Uny's adaptation to juxtapose the themes/categories with key messages identified in the tabulated summaries to create domains. These domains form the basis for 'the line of argument' in Stage 6 of SEBA.

BMJ Publishing Group Limited (BMJ) disclaims all liability and responsibility arising from any reliance placed on this supplemental material which has been supplied by the author(s)

Domain 2. Relationship between Dignity, WTHD and Assisted Dying

Subdomains	Codes
**Increasing WTHD**	▪ Loss of dignity (39-81)<br>▪ Fear of loss of dignity (42, 44, 52, 59, 82, 83)<br>▪ Loss of autonomy (39, 40, 42, 45, 47, 50-53, 55, 59, 62, 65, 69, 70, 73, 74, 77, 79, 81, 82, 84-88)<br>▪ Fear of loss of autonomy (42, 54, 70, 89)
**Decreasing WTHD/protective factors**	▪ Addressing of dignity related issues improves health-related quality of life (46)<br>▪ Improving dignity reduces desire for euthanasia (50)
**Effect of assisted dying on dignity**	▪ Reclamation/preservation of dignity (45, 49, 58, 90-95)<br>▪ Lack of assisted dying interferes with dignity (50, 76, 96)<br>▪ Reclamation/preservation of autonomy (43, 45, 49, 51, 52, 67, 73, 74, 77, 85, 88, 92, 94, 97-100)<br>▪ Lack of assisted dying interferes with autonomy (49, 96)<br>▪ Violation of autonomy (100)

Domain 3. Stakeholder Perspectives on Dignity

Subdomains	Codes	
**Patient Perspectives**	**Positive Aspects**<br>▪ Respect for humanity (67)<br>▪ Right to die (99)<br>▪ Unaffected by fear of death (48)<br>▪ Good death (67, 73, 99, 101-103)<br>- Maintaining control of their death (67, 73, 99, 101-103)<br>- Ending suffering (67, 73)<br>▪ Perceived futility (73, 74)<br>▪ General support (73, 98)<br>▪ Wanting the option for themselves (98)	**Negative Aspects**<br>▪ Religious concerns (67, 73, 102)<br>▪ Ethical concerns (62, 67, 73)<br>- Inability to ascertain patient consent (62, 67)<br>- Equating to murder/suicide (67)<br>▪ Change in ideologies (50)<br>▪ Deterioration of patient-doctor relationship (58, 62, 74, 101)<br>- Loss of faith in palliative/hospice care (62, 101)<br>- Feeling of abandonment when WTHD is turned down (74)<br>- Poor communications (58)<br>▪ Against legislation (73)
**Healthcare Provider**<br><br>**Perspectives**	**Positive Aspects**<br>▪ Good death for patients (42, 47, 49, 50, 54, 56, 57, 61, 69, 70, 82, 87, 101, 103)<br>- Fulfilling patients' desire for control (47, 50, 56, 61, 82, 87, 101, 104)<br>- Ending suffering (42, 54, 57, 61, 69, 70, 103)<br>- Not requiring a long life (49)<br>▪ Feeling helpless (68)<br>▪ Non-abandonment of patients (90)<br>▪ Reducing patients' family burden (90)<br>▪ Avoiding unlawful hastening death (50)<br>▪ General support (40, 47, 62, 68-70, 75, 82, 90, 101, 103-105)<br>▪ Disregarding the need for alternatives (i.e. palliative care) (50)	**Negative Aspects**<br>▪ Religious concerns (106)<br>▪ Disrespecting patients' autonomy by not entertaining their wishes (104, 107)<br>▪ Emotional/moral distress (49, 50, 69, 91, 101, 108-113)<br>- Guilt (91, 112)<br>- Opposing medical principles (49, 69, 101, 108, 110, 111)<br>- Grief, avoidance after carrying out WTHD (113)<br>- Turning doctors into killers (111)<br>▪ No right to die (90)<br>▪ Unable to ascertain if patient's disease is incurable (109)<br>▪ Change in perceptions after disease (82)<br>▪ WTHD should not involve family members (113)<br>▪ "Slippery slope" (110)<br>▪ Societal pressure (55, 74)<br>▪ Disapproval of WTHD (62, 69, 75, 82, 101, 107, 113-115)<br>- Disagreement with the law (115)<br>- Reluctance to carry out WTHD (75, 101)<br>▪ Proposing alternatives (57, 62, 63, 90, 105, 111)<br>- Improving quality of life of dying patients (62, 111)<br>- Better palliative care (63, 90, 105)<br>- Healing (57)<br>▪ Death as an unknown (57)

		▪ Disapproval of 'tired of living' as a reason for WTHD (57)
**Lawmaker Perspectives**	▪ Belief in a right to die (76)<br>▪ Avoiding unlawful hastening death (96)<br>▪ Government's duty to consider patients' WTHD (74)<br>▪ Approval of WTHD (76, 100)	▪ "Slippery slope" (98, 114)<br>- Risk of abuse (114)<br>▪ Instigating social pressure (on the elderly) (74)<br>▪ Disapproval of WTHD (74, 83, 106, 114)

Domain 4. Dignity Conserving Measures

Subdomains	Codes
**Advantages to Dignity Conserving Measures**	▪ Palliative services<br>- Palliative care (40, 44, 47, 49, 50, 62, 63, 66, 73, 82, 90, 91, 103, 111, 116-118)<br>- Palliative sedation (47, 49, 91, 119)<br>- Palliative starvation (85, 109)<br>▪ Pain and symptom management<br>- Control of physical pain (58, 66, 75, 104, 107, 108, 119)<br>- Control of psychological symptoms (75, 102, 120)<br>▪ Hospice care (46, 47, 75, 100, 106, 107, 117)<br>▪ Holistic approach (43, 57, 105, 111, 112, 117, 121, 122)<br>▪ Ease of access to information (47, 57, 71, 87, 92, 100, 112, 117)<br>▪ Distributive justice (55)<br>▪ Social support<br>- Religious (75)<br>- Familial (45)<br>- Healthcare providers (47, 57, 58, 71, 87, 112)
**Limitations to Dignity Conserving Measures**	▪ Cannot completely address:<br>- Existential suffering (54)<br>- Pain (50)<br>- Complete loss of mobility (103)<br>- Extreme shortness of breath (103)<br>- Fear of sudden and rapid bleeding (103)<br>- Ongoing stress of losing autonomy and dignity (103)<br>▪ Side effects of pain medication (58)

Stage 5 of SEBA: Analysis of data and non-data driven literature

A novel aspect of the SEBA methodology has been its inclusion of position, perspective, conference, reflective and opinion papers, editorials, commentaries, letters, posters, oral presentations, forum discussions, interviews, blogs, governmental reports, policy statements and surveys from PubMed, Embase, PsycINFO, Cochrane Database of Systematic Reviews, CINAHL, Scopus databases and the Journal of Pain and Symptom Management, BMC Palliative Medicine, Death Studies, and Palliative Medicine. With many current survey and assessment tools not able to capture the intricate connections and personalised nature of wider concepts of dignity, assisted dying, WTHD, personhood and identity, these resources are a rich source of information. These resources also capture wider patient, HCP and lawmaker perspectives and offer information on ethical, existential, and societal considerations often excluded by traditional systematic reviews as evidenced by recent reviews into how physicians and patients deal with death and dying and moral distress (123-130)

The use of non-data driven articles such as position, perspective, conference, reflective and opinion papers, editorials, commentaries, letters, posters, oral presentations, forum discussions, interviews, blogs, governmental reports, policy statements and surveys is consistent with a constructivist approach that includes all forms of data. In addition, use of non-data driven articles is a valuable source of information for subject matter such as concepts of dignity which are notoriously difficult to study and open to sociocultural considerations.

However, with some of these sources neither peer-reviewed nor necessarily evidence-based, they were differentiated from primary research, compiled and thematically analysed. The themes identified from primary data sources and non-evidence based and/or tertiary data sources were then compared to enhance further the accountability and reproducibility.

With the themes from the two groups found to be similar, the expert and research teams were satisfied that their inclusion did not bias the overall data.

Stage 6 of SEBA: Synthesis of the Discussion

The Best Evidence Medical Education (BEME) Collaboration Guide (131) and the Structured approach to the Reporting In healthcare education of Evidence Synthesis (STORIES) (132) were used to guide the discussion.

References

1. Kow CS, Teo YH, Teo YN, Chua KZY, Quah ELY, Kamal NHBA, et al. A systematic scoping review of ethical issues in mentoring in medical schools. BMC Medical Education. 2020;20(1):1-10.
2. Ngiam LXL, Ong YT, Ng JX, Kuek JTY, Chia JL, Chan NPX, et al. Impact of Caring for Terminally Ill Children on Physicians: A Systematic Scoping Review. Am J Hosp Palliat Care. 2020:1049909120950301.
3. Krishna LKR, Tan LHE, Ong YT, Tay KT, Hee JM, Chiam M, et al. Enhancing Mentoring in Palliative Care: An Evidence Based Mentoring Framework. Journal of Medical Education and Curricular Development. 2020;7:2382120520957649.
4. Chia EWY, Huang H, Goh S, Peries MT, Lee CCY, Tan LHE, et al. A Systematic Scoping Review of Teaching and Evaluating Communications in The Intensive Care Unit. The Asia-Pacific Scholar. In Press.
5. Kuek JTY, Ngiam LXL, Kamal NHA, Chia JL, Chan NPX, Abdurrahman ABHM, et al. The impact of caring for dying patients in intensive care units on a physician's personhood: a systematic scoping review. 2020;15(1):1-16.
6. Hong DZ, Lim AJS, Tan R, Ong YT, Pisupati A, Chong EJX, et al. A Systematic Scoping Review on Portfolios of Medical Educators. Journal of Medical Education and Curricular Development. 2021;8:23821205211000356.
7. Goh S, Wong RSM, Quah ELY, Chua KZY, Lim WQ, Ng ADR, et al. Mentoring in palliative medicine in the time of covid-19: a systematic scoping review. BMC medical education. 2022;22(1):1-15.
8. Bok C, Ng CH, Koh JWH, Ong ZH, Ghazali HZB, Tan LHE, et al. Interprofessional communication (IPC) for medical students: a scoping review. BMC Med Educ. 2020;20(1):372.
9. Ng YX, Koh ZYK, Yap HW, Tay KT, Tan XH, Ong YT, et al. Assessing mentoring: A scoping review of mentoring assessment tools in internal medicine between 1990 and 2019. PloS one. 2020;15(5):e0232511.
10. Bousquet J, Schunemann HJ, Samolinski B, Demoly P, Baena-Cagnani CE, Bachert C, et al. Allergic Rhinitis and its Impact on Asthma (ARIA): achievements in 10 years and future needs. J Allergy Clin Immunol. 2012;130(5):1049-62.
11. Nur Haidah Ahmad Kamal LHET, Ruth Si Man Wong, Ryan Rui Song Ong, Ryan, Ern Wei Seow EKYL, Zheng Hui Mah, Min Chiam, Annelissa Mien Chew Chin, Jamie Xuelian Zhou, Gillian Li Gek Phua, Eng Koon Ong, Jin Wei Kwekc, Kiley Wei-Jen Loh and, Krishna LKR. Enhancing education in Palliative Medicine: the role of Systematic Scoping Reviews. Palliative Medicine & Care: Open Access. 2020;7(1):1-11.
12. Ryan Rui Song Ong REWS, Ruth Si Man Wong. A Systematic Scoping Review of Narrative Reviews in Palliative Medicine Education. Palliative Medicine & Care: Open Access. 2020;7(1):1-22.
13. Zheng Hui Mah RSMW, Ryan Ern Wei Seow Eleanor Kei Ying Loh, Nur Haidah, Ahmad Kamal RRSO, Lorraine Hui En Tan, Min Chiam, Annelissa Mien Chew Chin,, Jamie Xuelian Zhou GLGP, Yoke-Lim Soong, Jin Wei Kwek, and Lalit Kumar Radha Krishna. A Systematic Scoping Review of Systematic Reviews in Palliative Medicine Education. Palliative Medicine & Care: Open Access. 2020;7(1):1-12.
14. Pring R. The 'False Dualism' of Educational Research. Journal of Philosophy of Education. 2000;34(2):247-60.
15. Crotty M. The foundations of social research: Meaning and perspective in the research process: Sage; 1998 Oct 15.

16. Ford DW, Downey L, Engelberg R, Back AL, Curtis JR. Discussing religion and spirituality is an advanced communication skill: an exploratory structural equation model of physician trainee self-ratings. J Palliat Med. 2012;15(1):63-70.
17. Schick-Makaroff K, MacDonald M, Plummer M, Burgess J, Neander W. What synthesis methodology should I use? A review and analysis of approaches to research synthesis. AIMS public health. 2016;3(1):172.
18. Sandelowski M BJ. Handbook for synthesizing qualitative research. New York: Springer; 2007.
19. Pham MT, Rajic A, Greig JD, Sargeant JM, Papadopoulos A, McEwen SA. A scoping review of scoping reviews: advancing the approach and enhancing the consistency. Research synthesis methods. 2014;5(4):371-85.
20. Wen Jie Chua CWSC, Fion Qian Hui Lee, Eugene Yong Hian Koh, Ying Pin Toh, Stephen Mason, Lalit Kumar Radha Krishna. Structuring Mentoring in Medicine and Surgery. A Systematic Scoping Review of Mentoring Programs Between 2000 and 2019. Journal of Continuing Education in the Health Professions. 2020;40(3):158-68.
21. Yong Xiang Ng ZYKK, Hong Wei Yap, Kuang Teck Tay, Xiu Hui Tan, Yun Ting Ong, Lorraine Hui En Tan, Annelissa Mien Chew Chin, Ying Pin Toh, Sushma Shivananda, Scott Compton, Stephen Mason, Ravindran Kanesvaran, Lalit Krishna. Assessing mentoring: A scoping review of mentoring assessment tools in internal medicine between 1990 and 2019. . PLOS ONE. 2020;15(5):e0232511.
22. Peters MD, Godfrey CM, Khalil H, McInerney P, Parker D, Soares CB. Guidance for conducting systematic scoping reviews. Int J Evid Based Healthc. 2015;13(3):141-6.
23. Sambunjak D, Straus SE, Marusic A. A systematic review of qualitative research on the meaning and characteristics of mentoring in academic medicine. J Gen Intern Med. 2010;25(1):72-8.
24. Wong G, Greenhalgh T, Westhorp G, Buckingham J, Pawson R. RAMESES publication standards: meta-narrative reviews. BMC medicine. 2013;11(1):20.
25. Popay J, Roberts H, Sowden A, Petticrew M, Arai L, Rodgers M, et al. Guidance on the conduct of narrative synthesis in systematic reviews. A product from the ESRC methods programme Version. 2006;1:b92.
26. Braun V, Clarke V. Using thematic analysis in psychology. Qualitative research in psychology. 2006;3(2):77-101.
27. Cassol H, Pétré B, Degrange S, Martial C, Charland-Verville V, Lallier F, et al. Qualitative thematic analysis of the phenomenology of near-death experiences. PloS one. 2018;13(2):e0193001.
28. Hsieh H-F, Shannon SE. Three Approaches to Qualitative Content Analysis. Qualitative Health Research. 2005;15(9):1277-88.
29. Rider EA, Hinrichs MM, Lown BA. A model for communication skills assessment across the undergraduate curriculum. Med Teach. 2006;28(5):e127-34.
30. Goldie J. Review of ethics curricula in undergraduate medical education. Med Educ. 2000;34(2):108-19.
31. Duffy FD, Gordon GH, Whelan G, Cole-Kelly K, Frankel R, Buffone N, et al. Assessing competence in communication and interpersonal skills: the Kalamazoo II report. Acad Med. 2004;79(6):495-507.
32. Hong DZ, Goh JL, Ong ZY, Ting JJQ, Wong MK, Wu J, et al. Postgraduate ethics training programs: a systematic scoping review. BMC Medical Education. 2021;21(1):338.
33. Tay KT, Ng S, Hee JM, Chia EWY, Vythilingam D, Ong YT, et al. Assessing Professionalism in Medicine - A Scoping Review of Assessment Tools from 1990 to 2018. J Med Educ Curric Dev. 2020;7:2382120520955159.

34. David M, Davis M, Harden R, Howie P, Ker J, Pippard M. AMEE Medical Education Guide No. 24: Portfolios as a method of student assessment. Medical teacher. 2001;23:535-51.
35. Dixon-Woods M, Agarwal S, Young B, Jones D, Sutton A. Integrative Approaches to Qualitative and Quantitative Evidence. 2004.
36. Bryman A. Social Research Methods: Oxford University Press; 2016.
37. France EF, Uny I, Ring N, Turley RL, Maxwell M, Duncan EAS, et al. A methodological systematic review of meta-ethnography conduct to articulate the complex analytical phases. BMC Medical Research Methodology. 2019;19(1):35.
38. Noblit GW, Hare RD, Hare RD. Meta-ethnography: Synthesizing qualitative studies: sage; 1988.
39. Al Rabadi L, LeBlanc M, Bucy T, Ellis LM, Hershman DL, Meyskens FL, et al. Trends in Medical Aid in Dying in Oregon and Washington. JAMA Network Open. 2019;2(8):e198648-e.
40. Emanuel EJ, Onwuteaka-Philipsen BD, Urwin JW, Cohen J. Attitudes and practices of euthanasia and physician-assisted suicide in the United States, Canada, and Europe. JAMA: Journal of the American Medical Association. 2016;316(1):79-90.
41. Brinkman-Stoppelenburg A, Vergouwe Y, van der Heide A, Onwuteaka-Philipsen BD. Obligatory consultation of an independent physician on euthanasia requests in the Netherlands: What influences the SCEN physicians judgment of the legal requirements of due care? Health Policy. 2014;115(1):75-81.
42. Buiting H, van Delden J, Onwuteaka-Philpsen B, Rietjens J, Rurup M, van Tol D, et al. Reporting of euthanasia and physician-assisted suicide in the Netherlands: Descriptive study. BMC Medical Ethics Vol 10 2009, ArtID 18. 2009;10.
43. Konishi E, Davis A. The right-to-die and the duty-to-die: Perceptions of nurses in the West and in Japan. International Nursing Review. 2001;48(1):17-28.
44. Chapple A, Ziebland S, McPherson A, Herxheimer A. What People Close to Death Say About Euthanasia and Assisted Suicide: A Qualitative Study. Journal of Medical Ethics: Journal of the Institute of Medical Ethics. 2006;32(12):706-10.
45. Coyle N, Sculco L. Expressed desire for hastened death in seven patients living with advanced cancer: a phenomenologic inquiry. Oncol Nurs Forum. 2004;31(4):699-709.
46. Crespo I, Rodríguez-Prat A, Monforte-Royo C, Wilson KG, Porta-Sales J, Balaguer A. Health-related quality of life in patients with advanced cancer who express a wish to hasten death: A comparative study. Palliat Med. 2020;34(5):630-8.
47. Drum CE, White G, Taitano G, Horner-Johnson W. The Oregon Death with Dignity Act: Results of a literature review and naturalistic inquiry. Disability and Health Journal. 2010;3(1):3-15.
48. Ferrand E, Dreyfus JF, Chastrusse M, Ellien F, Lemaire F, Fischler M. Evolution of requests to hasten death among patients managed by palliative care teams in France: a multicentre cross-sectional survey (DemandE). Eur J Cancer. 2012;48(3):368-76.
49. Ramsey C. The right to die: beyond academia. Monash Bioeth Rev. 2016;34(1):70-87.
50. Reagan P, Hurst R, Cook L, Zylicz Z, Otlowski M, Veldink JH, et al. Physician-assisted death: Dying with dignity? Lancet Neurology. 2003;2(10):637-43.
51. Rodríguez-Prat A, Balaguer A, Booth A, Monforte-Royo C. Understanding patients' experiences of the wish to hasten death: an updated and expanded systematic review and meta-ethnography. BMJ Open. 2017;7(9):e016659.
52. Rodríguez-Prat A, van Leeuwen E. Assumptions and moral understanding of the wish to hasten death: a philosophical review of qualitative studies. Medicine, Health Care & Philosophy. 2018;21(1):63-75.

53. Wang L, Elliott M, Henson LJ, Gerena-Maldonado E, Strom S, Downing S, et al. Death with dignity in Washington and Oregon patients with amyotrophic lateral sclerosis. Neurology. 2016;86(16).
54. Fischer S, Huber CA, Furter M, Imhof L, Mahrer Imhof R, Schwarzenegger C, et al. Reasons why people in Switzerland seek assisted suicide: the view of patients and physicians. Swiss Med Wkly. 2009;139(23-24):333-8.
55. Freeman LM, Rose SL, Youngner SJ. Poverty: Not a Justification for Banning Physician-Assisted Death. Hastings Cent Rep. 2018;48(6):38-46.
56. Ganzini L, Goy ER, Dobscha SK. Why Oregon patients request assisted death: Family members' views. Journal of General Internal Medicine. 2008;23(2):154-7.
57. Goligher EC, Wesley Ely E, Sulmasy DP, Bakker J, Raphael J, Volandes AE, et al. Physician-Assisted Suicide and Euthanasia in the ICU: A Dialogue on Core Ethical Issues. Critical Care Medicine. 2017;45(2):149-55.
58. Hendry M, Pasterfield D, Lewis R, Carter B, Hodgson D, Wilkinson C. Why do we want the right to die? A systematic review of the international literature on the views of patients, carers and the public on assisted dying. Palliative Medicine. 2013;27(1):13-26.
59. Hiscox WE. Physician-assisted suicide in Oregon: The 'death with dignity' data. Medical Law International. 2007;8(3):197-220.
60. Kade WJ, Kade WJ. Death with dignity: a case study. Annals of Internal Medicine. 2000;132(6):504-6.
61. Kouwenhoven PS, van Thiel GJ, Raijmakers NJ, Rietjens JA, van der Heide A, van Delden JJ. Euthanasia or physician-assisted suicide? A survey from the Netherlands. Eur J Gen Pract. 2014;20(1):25-31.
62. Bailey F. "I am not afraid of dying. I just don't want to be there when it happens.". Medical Care. 2008;46(12):1195-7.
63. Barutta J, Vollmann J. Physician-assisted death with limited access to palliative care. Journal of Medical Ethics: Journal of the Institute of Medical Ethics. 2015;41(8):652-4.
64. Bolt EE, Pasman H, Deeg DJ, Onwuteaka-Philipsen BD. From advance euthanasia directive to euthanasia: Stable preference in older people? Journal of the American Geriatrics Society. 2016;64(8):1628-33.
65. Dees M, Vernooij-Dassen M, Dekkers W, van Weel C. Review unbearable suffering of patients with a request for euthanasia or physician-assisted suicide: An integrative review. Psycho-Oncology. 2010;19(4):339-52.
66. Duckett S. Pathos, death talk and palliative care in the assisted dying debate in Victoria, Australia. Mortality. 2020;25(2):151-66.
67. Eliott JA, Olver IN. Dying cancer patients talk about euthanasia. Social Science & Medicine. 2008;67(4):647-56.
68. McPherson T. My mum wanted assisted dying but we watched her die slowly and in pain. BMJ: British Medical Journal (Clinical Research Edition). 2012;344:e4007-e.
69. Mukhopadhyay S, Banerjee D. Physician assisted suicide in dementia: A critical review of global evidence and considerations from India. Asian J Psychiatr. 2021;64:102802.
70. Rowe Iii MJ. Beliefs. Chicago, Illinois: American Medical Association; 2015. p. 877-8.
71. Roy DJ. Palliative care and euthanasia: a continuing need to think again. J Palliat Care. 2002;18(1):3-5.
72. van Tol D, Rietjens J, van der Heide A. Judgment of unbearable suffering and willingness to grant a euthanasia request by Dutch general practitioners. Health Policy. 2010;97(2-3):166-72.

73. Wilson KG, Chochinov HM, McPherson CJ, Skirko MG, Allard P, Chary S, et al. Desire for euthanasia or physician-assisted suicide in palliative cancer care. Health Psychology. 2007;26(3):314-23.
74. Florijn BW. From Reciprocity to Autonomy in Physician-Assisted Death: An Ethical Analysis of the Dutch Supreme Court Ruling in the Albert Heringa Case. Am J Bioeth. 2022;22(2):51-8.
75. Ganzini L, Nelson HD, Schmidt TA, Kraemer DF, Delorit MA, Lee MA. Physicians' experiences with the Oregon Death with Dignity Act. N Engl J Med. 2000;342(8):557-63.
76. Kishore RR. Aruna Shanbaug and the right to die with dignity: the battle continues. Indian J Med Ethics. 2016;1(1):38-46.
77. Royo C, Villavicencio-Chávez C, Tomás-Sábado J, Mahtani V, Balaguer A. What Lies behind the Wish to Hasten Death? A Systematic Review and Meta-Ethnography from the Perspective of Patients. PloS one. 2012;7:e37117.
78. Bahník Š, Vranka MA, Trefná K. What makes euthanasia justifiable? The role of symptoms' characteristics and interindividual differences. Death Stud. 2021;45(3):226-37.
79. Hizo-Abes P, Siegel L, Schreier G. Exploring attitudes toward physician-assisted death in patients with life-limiting illnesses with varying experiences of palliative care: a pilot study. BMC Palliative Care. 2018;17(1):56.
80. Ohnsorge K, Gudat H, Rehmann-Sutter C. What a wish to die can mean: reasons, meanings and functions of wishes to die, reported from 30 qualitative case studies of terminally ill cancer patients in palliative care. BMC Palliat Care. 2014;13:38.
81. Ruijs CDM, van der Wal G, Kerkhof AJFM, Onwuteaka-Philipsen BD. Unbearable suffering and requests for euthanasia prospectively studied in end-of-life cancer patients in primary care. BMC Palliative Care. 2014;13(1):62.
82. Gómez-Vírseda C, Gastmans C. Euthanasia in persons with advanced dementia: a dignity-enhancing care approach. J Med Ethics. 2021.
83. Hale DB. A pretty pass: when is there a right to die? Common Law World Rev. 2003;32(1):1-14.
84. Cheung G, Sundram F. Who are the elderly who want to end their lives? [References]. Rational suicide in the elderly: Clinical, ethical, and sociocultural aspects. Cham, Switzerland: Springer International Publishing/Springer Nature; Switzerland; 2017. p. 113-28.
85. Dees MK, Vernooij-Dassen MJ, Dekkers WJ, Vissers KC, van Weel C. 'Unbearable suffering': A qualitative study on the perspectives of patients who request assistance in dying. Journal of Medical Ethics: Journal of the Institute of Medical Ethics. 2011;37(12):727-34.
86. Fontalis A, Prousali E, Kulkarni K. Euthanasia and assisted dying: what is the current position and what are the key arguments informing the debate? Journal of the Royal Society of Medicine. 2018;111(11):407-13.
87. Ganzini L, Dobscha SK, Heintz RT, Press N. Oregon physicians' perceptions of patients who request assisted suicide and their families. Journal of Palliative Medicine. 2003;6(3):381-90.
88. Karlsson M, Milberg A, Strang P. Dying cancer patients' own opinions on euthanasia: An expression of autonomy? A qualitative study. Palliative Medicine. 2012;26(1):34-42.
89. Gamester N, Van den Eynden B. The relationship between palliative care and legalized euthanasia in Belgium. Journal of Palliative Medicine. 2009;12(7):589-91.
90. Rodriquez E. The arguments for euthanasia and physician-assisted suicide: ethical reflection. Linacre Q. 2001;68(3):251-61.
91. Abrahm JL. Patient and family requests for hastened death. Hematology Am Soc Hematol Educ Program. 2008:475-80.

92. Gandsman A. Paradox of choice and the illusion of autonomy: The construction of ethical subjects in right-to-die activism. Death Studies. 2018;42(5):329-35.
93. Granda-Cameron C, Houldin A. Concept Analysis of Good Death in Terminally Ill Patients. American Journal of Hospice & Palliative Medicine. 2012;29(8):632-9.
94. Heintz AP. Euthanasia--or, death on request. J Psychosom Obstet Gynaecol. 2002;23(2):73-5.
95. Jiraphan A, Pitanupong J. General population-based study on preferences towards end-of-life care in Southern Thailand: a cross-sectional survey. BMC Palliative Care. 2022;21(1):36.
96. Dyer C. Two men plead with judges to let doctors end their life legally. BMJ: British Medical Journal (Clinical Research Edition). 2012;344:e4270-e.
97. Castelli Dransart DA, Lapierre S, Erlangsen A, Canetto SS, Heisel M, Draper B, et al. A systematic review of older adults' request for or attitude toward euthanasia or assisted-suicide. Aging & Mental Health. 2021;25(3):420-30.
98. Achille MA, Ogloff JR. Attitudes toward and desire for assisted suicide among persons with amyotrophic lateral sclerosis. Omega: Journal of Death and Dying. 2003;48(1):1-21.
99. Right to die--right or wrong? Lancet Neurol. 2003;2(10):583.
100. Friend ML. Physician-Assisted Suicide: Death With Dignity? Journal of Nursing Law. 2011;14(3/4):110-6.
101. Drury B. The doctors of mercy. Men's Health. 2006;21(8):180-7.
102. Wilson KG, Scott JF, Graham ID, Kozak JF, Chater S, Viola RA, et al. Attitudes of terminally ill patients toward euthanasia and physician-assisted suicide. Archives of Internal Medicine. 2000;160(16):2454-60.
103. Reid T. Reflections from a provider of medical assistance in dying. Canadian Family Physician. 2018;64(9):639-40.
104. Asai A, Ohnishi M, Nagata SK, Tanida N, Yamazaki Y. Doctors' and nurses' attitudes towards and experiences of voluntary euthanasia: survey of members of the Japanese Association of Palliative Medicine. J Med Ethics. 2001;27(5):324-30.
105. Eastaugh A. Choosing to die. Br J Gen Pract. 2012;62(595):96-7.
106. Salladay SA. Christian ethics. Death with dignity? Journal of Christian Nursing. 2010;27(3):232-.
107. Broom A. On euthanasia, resistance, and redemption: The moralities and politics of a hospice. Qualitative Health Research. 2012;22(2):226-37.
108. Mentzelopoulos SD, Haywood K, Cariou A, Mantzanas M, Bossaert L. Evolution of medical ethics in resuscitation and end of life. Trends in Anaesthesia and Critical Care. 2016;10:7-14.
109. Raus K, Sterckx S. Euthanasia for mental suffering. New directions in the ethics of assisted suicide and euthanasia. Cham, Switzerland: Springer International Publishing/Springer Nature; Switzerland; 2015. p. 79-96.
110. Robinson V, Clarke J, George R. Euthanasia: A caricature of care, not a cure for suffering. British Journal of Neuroscience Nursing. 2007;3(10):457-60.
111. Stephenson J. Assisted dying: a palliative care physician's view. Clin Med (Lond). 2006;6(4):374-7.
112. Finlay IG. Quality of life to the end. Commun Med. 2005;2(1):91-5.
113. Feigin S, Owens RG, Goodyear-Smith F. Helping a loved one die: the act of assisted dying in New Zealand. Mortality. 2019;24(1):95-110.
114. Dyer C. Dying woman loses her battle for assisted suicide. Bmj. 2002;324(7345):1055.

115. Fournier RR. Responses to life after death with dignity: the Oregon experience. Soc Work. 2000;45(5):467-8.
116. Comby M, Filbet M. The demand for euthanasia in palliative care units: A prospective study in seven units of the 'Rhone-Alpes' region. Palliative Medicine. 2005;19(8):587-93.
117. Simpson E. Harms to dignity, bioethics, and the scope of biolaw. Journal of Palliative Care. 2004;20(3):185-92.
118. Ganzini L, Back A. From the USA: Understanding requests for physician-assisted death. Palliative Medicine. 2003;17(2):113-4.
119. Gentzler J. What is a death with dignity? J Med Philos. 2003;28(4):461-87.
120. Robinson S, Kissane DW, Brooker J, Hempton C, Burney S. The Relationship Between Poor Quality of Life and Desire to Hasten Death: A Multiple Mediation Model Examining the Contributions of Depression, Demoralization, Loss of Control, and Low Self-worth. J Pain Symptom Manage. 2017;53(2):243-9.
121. Clark D. Cultural considerations in planning palliative and end of life care. Palliat Med. 2012;26(3):195-6.
122. Oczkowski SJW, Crawshaw DE, Austin P, Versluis D, Kalles-Chan G, Kekewich M, et al. How can we improve the experiences of patients and families who request medical assistance in dying? A multi-centre qualitative study. BMC Palliative Care. 2021;20(1):185.
123. Ong RSR, Wong RSM, Chee RCH, Quek CWN, Burla N, Loh CYL, et al. A systematic scoping review moral distress amongst medical students. BMC Medical Education. 2022;22(1):466.
124. Chua KZY, Quah ELY, Lim YX, Goh CK, Lim J, Wan DWJ, et al. A systematic scoping review on patients' perceptions of dignity. BMC Palliative Care. 2022;21(1):118.
125. Ho CY, Lim NA, Ong YT, Lee ASI, Chiam M, Gek GPL, et al. The impact of death and dying on the personhood of senior nurses at the National Cancer Centre Singapore (NCCS): a qualitative study. BMC Palliat Care. 2022;21(1):83.
126. Chiam M, Ho CY, Quah E, Chua KZY, Ng CWH, Lim EG, et al. Changing self-concept in the time of COVID-19: a close look at physician reflections on social media. Philos Ethics Humanit Med. 2022;17(1):1.
127. Chan NPX, Chia JL, Ho CY, Ngiam LXL, Kuek JTY, Ahmad Kamal NHB, et al. Extending the Ring Theory of Personhood to the Care of Dying Patients in Intensive Care Units. Asian Bioeth Rev. 2022;14(1):71-86.
128. Huang H, Toh RQE, Chiang CLL, Thenpandiyan AA, Vig PS, Lee RWL, et al. Impact of Dying Neonates on Doctors' and Nurses' Personhood: A Systematic Scoping Review. J Pain Symptom Manage. 2022;63(1):e59-e74.
129. Vig PS, Lim JY, Lee RWL, Huang H, Tan XH, Lim WQ, et al. Parental bereavement – impact of death of neonates and children under 12 years on personhood of parents: a systematic scoping review. BMC Palliative Care. 2021;20(1):136.
130. Ho CY, Kow CS, Chia CHJ, Low JY, Lai YHM, Lauw S-K, et al. The impact of death and dying on the personhood of medical students: a systematic scoping review. BMC Medical Education. 2020;20(1):516.
131. Haig A, Dozier M. BEME Guide no 3: systematic searching for evidence in medical education--Part 1: Sources of information. Medical teacher. 2003;25(4):352-63.
132. Gordon M, Gibbs T. STORIES statement: publication standards for healthcare education evidence synthesis. BMC medicine. 2014;12(1):143.
