# Supplementary material for: Evidence-guided approach to portfolio-guided teaching and assessing communications, ethics and professionalism for medical students and physicians: a systematic scoping review
Source: BMJ Open. 2023 Mar 28;13(3):e067048. doi: 10.1136/bmjopen-2022-067048 (PMC10069516; doi:10.1136/bmjopen-2022-067048)
Supplement: Supplementary data [file bmjopen-2022-067048supp002.pdf]

Appendix B. Search strategies
PubMed
Filters Applied: English, from 2000/1/1 – 2020/12/31

#1	("physicians"[MeSH Terms] OR "students, medical"[MeSH Terms] OR "physician*"[Title/Abstract] OR "doctor*"[Title/Abstract] OR "medical student*"[Title/Abstract] OR "medical trainee*"[Title/Abstract] OR "physician trainee"[Title/Abstract]) AND ((2000/1/1:2020/12/31[pdat]) AND (english[Filter]))	395,521
#2	("educational status"[MeSH Terms] OR "education"[MeSH Terms] OR "learning"[MeSH Terms] OR "educat*"[Title/Abstract] OR "learn*"[Title/Abstract] OR "teach*"[Title/Abstract] OR "mentor*"[Title/Abstract]) AND ((2000/1/1:2020/12/31[pdat]) AND (english[Filter]))	1,265,980
#3	("professional identity"[Title/Abstract] OR "portfolio*"[Title/Abstract] OR "appraisal*"[Title/Abstract] OR "assessment*"[Title/Abstract]) AND ((2000/1/1:2020/12/31[pdat]) AND (english[Filter]))	912,728
#4	("communication"[MeSH Terms] OR "communicat*"[Title/Abstract] OR "interpersonal skill*"[Title/Abstract]) AND ((2000/1/1:2020/12/31[pdat]) AND (english[Filter]))	418,990
#5	("ethics"[MeSH Terms] OR "morals"[MeSH Terms] OR "social values"[MeSH Terms] OR "ethic*"[Title/Abstract] OR "moral*"[Title/Abstract] OR "value*"[Title/Abstract] OR "Beliefs"[Title/Abstract] OR "Principles"[Title/Abstract] OR "ideals"[Title/Abstract]) AND ((2000/1/1:2020/12/31[pdat]) AND (english[Filter]))	1,628,161
#6	("Professionalism"[MeSH Terms] OR "Professionalism"[Title/Abstract] OR "Professional"[Title/Abstract]) AND ((2000/1/1:2020/12/31[pdat]) AND (english[Filter]))	130,203
#1 AND #2 AND #3 AND (#4 OR #5 OR #6)		7,617

Embase

#1	('physician'/exp OR 'medical student'/exp OR 'physician':ti,ab OR 'doctor':ti,ab OR 'medical student':ti,ab) AND [2000-2020]/py AND [embase]/lim NOT ([embase]/lim AND [medline]/lim) AND [english]/lim	482,177
#2	('education'/exp OR 'educat*':ti,ab OR 'learn*':ti,ab OR 'teach*':ti,ab OR 'mentor*':ti,ab) AND [2000-2020]/py AND [embase]/lim NOT ([embase]/lim AND [medline]/lim) AND [english]/lim	779,513
#3	('professional identity':ti,ab OR 'portfolio*':ti,ab OR 'appraisal*':ti,ab OR 'holistic assessment':ti,ab OR 'personal assessment':ti,ab OR 'feedback':ti,ab OR 'continuous assessment':ti,ab) AND [2000-2020]/py AND [embase]/lim NOT ([embase]/lim AND [medline]/lim) AND [english]/lim	73,761
#4	('interpersonal communication'/exp OR 'communication':ti,ab OR 'interpersonal skill':ti,ab) AND [2000-2020]/py AND [embase]/lim NOT ([embase]/lim AND [medline]/lim) AND [english]/lim	254,318
#5	('ethics'/exp OR 'morality'/exp OR 'social value'/exp OR 'ethic*':ti,ab OR 'moral*':ti,ab OR 'value*':ti,ab OR 'beliefs':ti,ab OR 'principles':ti,ab OR 'ideals':ti,ab) AND [2000-2020]/py AND [embase]/lim NOT ([embase]/lim AND [medline]/lim) AND [english]/lim	955,250
#6	('professionalism'/exp OR 'professionalism':ti,ab) AND [2000-2020]/py AND [embase]/lim NOT ([embase]/lim AND [medline]/lim) AND [english]/lim	3,740
#1 AND #2 AND #3 AND (#4 OR #5 OR #6)		3,751

PsycINFO

1	exp physicians/ or exp medical students/ or (physician* or doctor* or medical student* or physician trainee* or medical trainee*).ab,ti.	140,688
2	exp education/ or exp learning/ or (educat* or learn* or teach* or mentor*).ab,ti.	1,324,893
3	('professional identity' or portfolio* or appraisal* or assessment*).ab,ti.	410,796
4	1 and 2 and 3	6,780
5	exp communication/ or (communicat* or interpersonal skill*).ab,ti.	502,521
6	exp ethics/ or exp morality/ or exp social values/ or (ethic* or moral* or value* or beliefs or principles or ideals).ab,ti.	647,241
7	exp professionalism/ or (professionalism or professional).ab,ti.	130,203
8	5 or 6 or 7	1191406
9	4 and 8	3082
10	limit 9 to (english language and yr="2000 - 2020")	2507

ERIC
Limits applied: English Language, 01/01/2000-31/12/2020

S1	MAINSUBJECT.EXACT.EXPLODE("Physicians") OR MAINSUBJECT.EXACT.EXPLODE("Medical Students") OR title(physician* OR doctor* OR medical student* OR physician trainee* OR medical trainee*) AND abstract(physician* OR doctor* OR medical student* OR physician trainee* OR medical trainee*)	6,171
#2	(MAINSUBJECT.EXACT.EXPLODE("Informal Education") OR MAINSUBJECT.EXACT.EXPLODE("Adult Education") OR MAINSUBJECT.EXACT.EXPLODE("Medical Education") OR MAINSUBJECT.EXACT.EXPLODE("Individualized Education Programs")) OR title(educat* OR learn* OR teach* OR mentor*) OR abstract(educat* OR learn* OR teach* OR mentor*)	536,465
#3	(MAINSUBJECT.EXACT.EXPLODE("Portfolios (Background Materials)") OR MAINSUBJECT.EXACT.EXPLODE("Professional Identity") OR MAINSUBJECT.EXACT.EXPLODE("Portfolio Assessment")) OR abstract('professional identity' OR portfolio* OR appraisal* OR assessment*) OR title('professional identity' OR portfolio* OR appraisal* OR assessment*)	85,052
#4	(MAINSUBJECT.EXACT.EXPLODE("Communication Strategies") OR MAINSUBJECT.EXACT.EXPLODE("Communication (Thought Transfer)") OR MAINSUBJECT.EXACT.EXPLODE("Communication Skills")) OR abstract(communicat* OR 'interpersonal skill*') OR title(communicat* OR 'interpersonal skill*')	90,574
#5	(MAINSUBJECT.EXACT.EXPLODE("Social Values") OR MAINSUBJECT.EXACT.EXPLODE("Ethics")) OR abstract(ethic* OR moral* OR value* OR beliefs OR principles OR ideals) OR title(ethic* OR moral* OR value* OR beliefs OR principles OR ideals)	123,249
#6	MAINSUBJECT.EXACT.EXPLODE("Professionalism") OR abstract(professionalism OR professional) OR title(professionalism OR professional)	77,628
#1 AND #2 AND #3 AND (#4 OR #5 OR #6)		440

Google Scholar

(physicians OR medical students OR doctors) AND (education* OR learn* OR teach*) AND ("professional identity" OR portfolio*) AND ((communication* OR interpersonal skill*) OR (ethics OR values) OR professionalism)
