# Supplementary material for: Evidence-guided approach to portfolio-guided teaching and assessing communications, ethics and professionalism for medical students and physicians: a systematic scoping review
Source: BMJ Open. 2023 Mar 28;13(3):e067048. doi: 10.1136/bmjopen-2022-067048 (PMC10069516; doi:10.1136/bmjopen-2022-067048)
Supplement: Supplementary data [file bmjopen-2022-067048supp004.pdf]

Appendix C

Portfolio Content (Competencies Assessed and Assessment Modalities)

Subthemes	Content		
	**Communication**	**Ethics**	**Professionalism**
**Competencies Assessed**	• "Effective" communication (65, 67, 68, 81, 86, 107, 127, 131-133)<br>• Professional behaviour and moral reasoning (71)<br>• Systems awareness and team-based care (71)<br>• Continuous learning and quality improvement (71)<br>• Patient-centred medical care (71)<br>• Teamwork (70)<br><br>Oral communication skills<br>• Attentive, active listening (84, 139, 140)<br>• Explanation, information giving (127, 139)<br>• History taking (93)<br>• Consultation (141)<br>• Breaking bad news (24, 139)<br>• Shared decision making (142)<br>• Empathetic communication (142)<br>• Taking history and clinical examination (84, 93)<br>• Discerning appropriate language to ensure patient understands (24, 127)<br>• Conflict management (139)<br>• Adaptability to different settings (67)<br><br>Written communication skills<br>• Proper grammar, spelling, logical and coherent structure (103)<br>• Patient cases (93)<br>• Scientific research (93)<br><br>Communication with:<br>• Patients (24, 68, 84, 93, 127, 139, 143)<br>• Children (127)	• Ethics in interprofessional practice (24, 67, 86, 128, 130, 145, 146)<br>• Doctor-patient relationship (81, 141, 145)<br>• Values/ ethics (67, 70, 86, 146)<br>• Ethical and legal responsibilities (65, 86)<br>• Moral reasoning (89)<br>• Ethics is assessed as a component of professionalism (85, 87, 88)<br>• Empathy (84)<br>• Showing respect and commitment (84)<br>• Respect for privacy (84)<br>• How to express ethical views appropriately (82)<br>• Identifying unethical moments of practice and grey areas (85, 89, 90)<br><br>Reflection:<br>• Quality of student's reflections (82, 84, 87)<br>• Authenticity of reflection (87)	• Learning from clinical experience (24, 81)<br>• Working in groups/ teamwork (24, 67, 70, 81, 86, 128, 130, 134, 145)<br>• Doctor-patient relationship (81, 141, 145)<br>• Becoming a doctor/roles and responsibilities (81, 86, 128, 130, 134, 145, 146)<br>• Patient care (67, 70, 128, 130)<br>• Continuous learning (67, 70)<br>• Values/ ethics (67, 70, 86, 146)<br>• Interpersonal skills/ effective Communication, (24, 67, 128, 145, 146)<br><br>Reflection:<br>• Clinical experience (81)<br>• Teaching (81)<br>• Learning needs in becoming a doctor<br>• Strengths and developmental areas (65, 86)<br>• Understanding of concepts addressed (86)<br>• Dilemmas and doubts (133)

	• Family (68, 84, 139, 144)<br>• Colleagues, interprofessional team (24, 68, 84, 93, 130, 139)<br>• Public (68)		
**Assessment Modalities (Portfolio items)**	Formative only (67, 84, 87, 92, 119, 126, 130, 146)<br>Summative only (65, 70, 71, 81-83, 87, 104, 116, 131, 134)<br>Both formative and summative (24, 67, 83, 86, 87, 117, 122, 128, 130, 134)<br>MCQs (65, 68, 87, 95, 126, 141, 143, 144, 146)<br>OSCEs (65, 67, 70, 71, 87, 126, 127, 130, 141, 143, 144, 146)<br>Essays – topical (67, 81, 93, 126)<br>Mini clinical examination exercise (Mini-CEX) (65, 67, 70, 87, 93, 130, 139, 143, 146)<br>Medical/patient presentations/research (67, 68, 80, 86, 93, 121, 126, 129)<br>Workplace-based clinical (67, 70, 71, 80, 83, 93, 107, 112, 122, 126, 127, 130, 139, 143)<br>Case-based discussions, summaries (65, 68, 83, 85, 89, 92, 129)(19)(81, 129) (83, 126, 130)<br>Simulated patient assessment (65, 68, 84, 103, 112)<br>Team-based assessment (65, 83, 86, 93)<br>Logbooks/ Portfolio interview (65, 67, 68, 70, 81-85, 87, 88, 103, 121, 126) (67, 121, 126)<br>Peer evaluation (67, 70, 71, 80, 85, 93, 107, 126, 127)<br>Faculty evaluation (67, 68, 70, 71, 80, 81, 83-86, 117, 126, 131, 147)<br>Feedback reports (75, 77, 80, 86, 91, 93, 109, 112, 122, 126, 130, 134, 139, 143) – multisource feedback (75, 77, 81, 82, 85, 86, 91, 93, 103, 109, 127, 130)<br>Self-assessment/appraisal (67, 68, 80, 82, 93, 104, 126, 130, 134)<br>Self-reflections – written/video/diaries (65, 67, 68, 71, 80-83, 85, 87, 88, 90, 93, 103, 107, 121, 127-130)<br>Learning plans and contracts (67, 68, 71, 80-83, 86, 92, 93, 103, 133)<br>Letters of recommendation (65, 81, 103)<br>Curriculum vitae (65, 67, 70, 83, 86, 122, 126)		
