# Supplementary material for: Evidence-guided approach to portfolio-guided teaching and assessing communications, ethics and professionalism for medical students and physicians: a systematic scoping review
Source: BMJ Open. 2023 Mar 28;13(3):e067048. doi: 10.1136/bmjopen-2022-067048 (PMC10069516; doi:10.1136/bmjopen-2022-067048)
Supplement: Author's manuscript [file bmjopen-2022-067048.draft_revisions.pdf]

BMJ Open

BMJ Open is committed to open peer review. As part of this commitment we make the peer review history of every article we publish publicly available.

When an article is published we post the peer reviewers' comments and the authors' responses online. We also post the versions of the paper that were used during peer review. These are the versions that the peer review comments apply to.

The versions of the paper that follow are the versions that were submitted during the peer review process. They are not the versions of record or the final published versions. They should not be cited or distributed as the published version of this manuscript.

BMJ Open is an open access journal and the full, final, typeset and author-corrected version of record of the manuscript is available on our site with no access controls, subscription charges or pay-per-view fees (http://bmjopen.bmj.com).

If you have any questions on BMJ Open's open peer review process please email info.bmjopen@bmj.com

BMJ Open

An evidence-guided approach to Portfolio guided Teaching and Assessing Communications, Ethics and Professionalism for medical students and physicians:A Systematic Scoping Review

Journal:	*BMJ Open*
Manuscript ID	bmjopen-2022-067048
Article Type:	Original research
Date Submitted by the Author:	02-Aug-2022

Complete List of Authors:	Krishna, Lalit; National University of Singapore; Duke-NUS Medical School<br>Ting, Jacquelin Jia Qi; National University of Singapore, Yong Loo Lin School of Medicine; National Cancer Centre Singapore, Division of Supportive and Palliative Care<br>Hong, Daniel Zhihao; National University of Singapore, Yong Loo Lin School of Medicine; National Cancer Centre Singapore, Division of Supportive and Palliative Care<br>Lam, Bertrand Kai Yang; National Cancer Centre Singapore, Division of Supportive and Palliative Care<br>Lim, Annabelle Jia Sing ; National University of Singapore, Yong Loo Lin School of Medicine; National Cancer Centre Singapore, Department of Supportive and Palliative Care<br>Chong, Eleanor Jia Xin; National University of Singapore, Yong Loo Lin School of Medicine; National Cancer Centre Singapore, Division of Supportive and Palliative Care<br>Pisupati, Anushka; National University of Singapore, Yong Loo Lin School of Medicine; National Cancer Centre Singapore, Division of Supportive and Palliative Care<br>Tan, Rei; National University of Singapore, Yong Loo Lin School of Medicine; National Cancer Centre Singapore, Division of Supportive and Palliative Care<br>Yeo, Jocelyn Yi Huang; National University of Singapore, Yong Loo Lin School of Medicine; National Cancer Centre Singapore, Division of Supportive and Palliative Care<br>Koh, Yi Zhe; National University of Singapore, Yong Loo Lin School of Medicine; National Cancer Centre Singapore, Division of Supportive and Palliative Care<br>Quek, Chrystie; National University of Singapore, Yong Loo Lin School of Medicine; National Cancer Centre Singapore, Division of Supportive and Palliative Care<br>Lim, Jia Yin; National University of Singapore, Yong Loo Lin School of Medicine; National Cancer Centre Singapore, Division of Supportive and Palliative Care<br>Tay, Kuang Teck; National Cancer Centre Singapore, Division of Supportive and Palliative Care<br>Ong, Yun Ting; National University of Singapore, Yong Loo Lin School of Medicine; National Cancer Centre Singapore Division of Supportive and

	Palliative Care<br>Chiam, Min; National Cancer Centre Singapore, Division of Cancer Education<br>Zhou, Xuelian Jamie; National Cancer Centre Singapore, Division of Supportive and Palliative Care; Duke-NUS Medical School, Lien Centre for Palliative Care<br>Phua, Gillian Li Gek; National Cancer Centre Singapore, Division of Supportive and Palliative Care; Duke-NUS Medical School, Lien Centre for Palliative Care<br>Mason, Stephen; University of Liverpool, Palliative Care Institute Liverpool<br>Wijaya, Limin; National Cancer Centre Singapore, Division of Supportive and Palliative Care; Singapore General Hospital, Department of Infectious Diseases
Keywords:	MEDICAL EDUCATION & TRAINING, ETHICS (see Medical Ethics), EDUCATION & TRAINING (see Medical Education & Training)

SCHOLARONE™
Manuscripts

I, the Submitting Author has the right to grant and does grant on behalf of all authors of the Work (as defined in the below author licence), an exclusive licence and/or a non-exclusive licence for contributions from authors who are: i) UK Crown employees; ii) where BMJ has agreed a CC-BY licence shall apply, and/or iii) in accordance with the terms applicable for US Federal Government officers or employees acting as part of their official duties; on a worldwide, perpetual, irrevocable, royalty-free basis to BMJ Publishing Group Ltd ("BMJ") its licensees and where the relevant Journal is co-owned by BMJ to the co-owners of the Journal, to publish the Work in this journal and any other BMJ products and to exploit all rights, as set out in our licence.

The Submitting Author accepts and understands that any supply made under these terms is made by BMJ to the Submitting Author unless you are acting as an employee on behalf of your employer or a postgraduate student of an affiliated institution which is paying any applicable article publishing charge ("APC") for Open Access articles. Where the Submitting Author wishes to make the Work available on an Open Access basis (and intends to pay the relevant APC), the terms of reuse of such Open Access shall be governed by a Creative Commons licence – details of these licences and which Creative Commons licence will apply to this Work are set out in our licence referred to above.

Other than as permitted in any relevant BMJ Author's Self Archiving Policies, I confirm this Work has not been accepted for publication elsewhere, is not being considered for publication elsewhere and does not duplicate material already published. I confirm all authors consent to publication of this Work and authorise the granting of this licence.

For peer review only

Title: An evidence-guided approach to Portfolio guided Teaching and Assessing Communications, Ethics and Professionalism for medical students and physicians:

A Systematic Scoping Review

Short Title: A review of CEP Portfolios for physicians and medical students

Lalit Kumar Radha Krishna[1-8,#] Jacquelin Jia Qi Ting[1,2], Daniel Zhihao Hong [1,2], Bertrand Kai Yang Lam[2], Annabelle Jia Sing Lim[1,2], Eleanor Jia Xin Chong[1,2], Anushka Pisupati[1,2] , Rei Tan[1,2], Jocelyn Yi Huang Yeo[1,2], Yi Zhe Koh[1,2], Chrystie Wan Ning Quek[1,2], Jia Yin Lim[1,2], Kuang Teck Tay[2], Yun Ting Ong[1,2], Min Chiam[3,] , Jamie Zhou [2,5,8], Gillian Phua[2,5,8], Stephen Mason[7], Limin Wijaya[2,5,9]

[1] Yong Loo Lin School of Medicine, National University of Singapore, NUHS Tower Block, 1E Kent Ridge Road, Level 11, Singapore 119228

[2] Division of Supportive and Palliative Care, National Cancer Centre Singapore,11 Hospital Dr, Singapore 169610

[3] Division of Cancer Education, National Cancer Centre Singapore, 11 Hospital Dr, Singapore 169610

[4] PalC, The Palliative Care Centre for Excellence in Research and Education, PalC c/o Dover Park Hospice, 10 Jalan Tan Tock Seng, Singapore

[5] Duke-NUS Medical School, 8 College Rd, Singapore 169857

[6] Centre of Biomedical Ethics, National University of Singapore, 21 Lower Kent Ridge Rd, Singapore 119077

[7] Palliative Care Institute Liverpool, Academic Palliative & End of Life Care Centre, Cancer Research Centre, University of Liverpool, 200 London Rd, Liverpool L3 9TA, United Kingdom

[8] Lien Centre for Palliative Care, Duke-NUS Medical School, 8 College Rd, Singapore 169857

[9] Department of Infectious Diseases, Singapore General Hospital, Outram Road, Singapore 169608

[#]Corresponding Author:

Dr. Lalit Kumar Radha Krishna

Palliative Care Institute Liverpool, Academic Palliative & End of Life Care Centre,

University of Liverpool, 200 London Rd, Liverpool L3 9TA, United Kingdom

Email: lalit.radha-krishna@liverpool.ac.uk Phone number: 6436 8183

For peer review only

For peer review only - http://bmjopen.bmj.com/site/about/guidelines.xhtml

ABSTRACT

Objectives

Professional identity formation (PIF) or thinking, acting, and feeling like a physician involves the inculcation of a variety of professional values, beliefs, and principles, making shaping PIF difficult. One key aspect is communication, ethics, and professional (CEP). As efforts to structure longitudinal training and assessments of CEP to boost medical student and physician professionalism and patient interactions, combined CEP portfolios could achieve the desired longitudinal structured support mechanism and act as a surrogate marker for PIF. Yet in the absence of a consistent CEP portfolio, we evaluate current use, structure, assessment and effects of CEP portfolios and its support of PIF.

Design

This study is a systematic scoping review (SSR).

Methods

Krishna's Systematic Evidence Based Approach (SEBA) is employed to guide this SSR of portfolio use in CEP training and assessment in various online databases of publications between 2000 and 2020. The included articles were concurrently thematically, and content analysed. The categories and themes identified in The Split Approach were combined using the Funnelling Process to create domains that structured the discussion.

Results

12,300 abstracts were reviewed, and 82 articles were included. The domains identified are character, content, design, and strengths and limitations.

Conclusions

This SSR in SEBA affirms links between CEP's role in the longitudinal development of PIF and suggests that micro-competencies or CEP milestones employed in structured portfolios offer a consistent, and accessible framework maps CEP's developing competencies and PIF. However, to be effective CEP portfolios require the use of regular, contextually dependent multidimensional assessments.

For peer review only - http://bmjopen.bmj.com/site/about/guidelines.xhtml
For peer review only

Strengths and limitations of this study

- The SEBA methodology allows the study of data from diverse methodological sources, settings, physician populations and specialities.
- The SEBA methodology adopts the structure of systematic reviews and flexibility of narrative reviews to synthesise a reproducible and accountable evaluation of all sources of data.
- The clinically-evidenced Ring Theory of Personhood (RToP) allows the transparent, reproducible and personalised analysis of concepts of CEP amongst medical students and physicians.
- Use of the RToP and the SEBA methodology to study CEP is novel and require further study.

For peer review only

Abstract Word Count: 248 words

Manuscript Word Count: 3957 words

Number of Tables: 6

Number of Figures: 2

Number of References: 149

Keywords: Portfolios, Professional Identity Formation, Communication, Ethics, Professionalism, Medical Education

For peer review only - http://bmjopen.bmj.com/site/about/guidelines.xhtml

INTRODUCTION

The manner that medical students and physicians view and integrate their clinical, professional, academic, research, ethical, legal, moral, and social roles and responsibilities influences how they *'think, act, and feel like a physician'* or professional identity formation (PIF) (1-3). This longitudinal process involves internalising beliefs, principles, values, norms, characteristics, codes of practice, and expectations on roles, responsibilities, conduct and outcomes (henceforth new concepts) and integrating them with the individual's personal experiences, motivations, enthusiasm, idealism, abilities, competencies, virtues, expectations, knowledge, skills, emotions, and attitudes (henceforth narratives) and their values, beliefs, and principles (henceforth current concepts). Supporting the integration of new and current concepts is the mentoring umbrella which includes personalised, accessible, and longitudinal mentoring, coaching, supervision, feedback, role modelling, and guided reflections.

However, to support PIF effectively, mentoring umbrella-led interventions demands a structured stage-based framework that is assessed, overseen, and supported by the host organization. These requirements for a consistent, structured approach is emphasised by the growing notion that PIF evolves within "*persistent, sustaining social network of individuals who share and develop an overlapping knowledge base, set of beliefs, values, history and experiences focused on a common practice and/or enterprise*" or Communities of Practice (CoP)s. Whilst there are several ways of rendering a curated approach to nurturing PIF, one increasingly promising approach is use of portfolios focused on developing communication, ethics and professional (henceforth CEP) knowledge, skills, attitudes, and competencies (1, 4-7).

CEP is a key ingredient to linking PIF's intertwined influences. Understanding CEP's influence can be best contextualised when applied in a structured approach. A structured CEP based portfolio approach offers a longitudinal perspective and could enhance socialisation, reflective practice, and feedback, which are pivotal aspects of PIF.

METHODS

A Systematic Evidence-Based Approach (SEBA) guided Systematic Scoping Review (SSR) (henceforth SSR in SEBA) is proposed to explore what is known about portfolio use in CEP. Critically SEBA's constructivist perspective and relativist lens allows study of CEP as a sociocultural construct, an oversight that has thus far limited efforts to study and design CEP portfolios. This approach allows the effects of regnant social, cultural,

For peer review only - http://bmjopen.bmj.com/site/about/guidelines.xhtml

For peer review only

political, institutional and personal factors; social media (8), the hidden curriculum; practical factors and program, setting, speciality related factors and beliefs, codes of conduct, norms and expectations; team dynamics', the training approach; and intra- and interprofessional practice be evaluated longitudinally (9-13).

It is posited that this SSR in SEBA of portfolio will inform the design of a combined CEP portfolio for undergraduate and postgraduate medical education; improve training; enable consistent multi-sourced assessments; and encourage reflective practice amongst medical students and physicians (henceforth practitioners). In addition, in determining the viability, characteristics, contents, and assessments of CEP portfolios this SSR in SEBA will facilitate prompt, necessary, accessible, individualised, continuing, and comprehensive support as practitioners grapple with inevitable professional, moral, research, clinical and personal dilemmas and develop their professional identities.

SSRs in SEBA's six stages which consists of Systematic Approach, Split Approach, Jigsaw Perspective, Funnelling Process, Reiterative Process and Synthesis of SSR, as seen in *Figure 1* will facilitate the structured, transparent, accountable, reproducible and multiangled study of portfolio use in teaching and assessing CEP (14-23).

(Insert Figure 1. *The SEBA Process*)

Stage 1 of SEBA: Systematic Approach

A. Determining of Title and Background of the Review

To provide a balanced and accountable review of the proposed research process, SSRs in SEBA begin with consulting an expert team comprised of a medical librarian from the Yong Loo Lin School of Medicine (YLLSoM) at the National University of Singapore (24), and local education experts and clinicians at the National Cancer Centre Singapore (NCCS), the Palliative Care Institute Liverpool, YLLSoM and Duke-NUS Medical School (henceforth the expert team). By reviewing, questioning, and challenging the assumptions of the research team, the expert team ensures that a robust and evidence-based driven position is taken at all stages of this SSR in SEBA. Together the expert team and research teams determined the goals of the SSR in SEBA and the population, context, and concepts to be evaluated.

For peer review only - http://bmjopen.bmj.com/site/about/guidelines.xhtml
For peer review only

Both teams adopt an interpretive approach to immerse themselves in the data through repeated reading and/or analysis of qualitative data; reflexivity, and discussions to piece together data in a meaningful manner at every stage of SEBA.

B. Identification of Research Question

The research and expert team determined the primary research question: "*What is known about CEP portfolios?*". The secondary research questions were *"How portfolios to teach and assess CEP implemented?" What are the benefits of using portfolios to teach and assess CEP?"* This review will begin CEP training and assessment in medical school and proceed to evaluate CEP development throughout residency and further postgraduate training to include the training of consultants/attendings.

C. Inclusion Criteria

A Population, Intervention, Comparison and Outcome (PICOs) format, outlined in Table 1, was used to guide the research process (25, 26).

Table 1. *PICOS Inclusion and Exclusion Criteria*

PICOS	Inclusion criteria	Exclusion criteria
**Population**	• Undergraduate and postgraduate medical students<br>• Qualified medical doctors, physician or resident; medical officer, registrar, house officer, attending, consultant	• Allied health specialties such as Pharmacy, Dietetics, Chiropractic, Midwifery, Podiatry, Speech Therapy, Occupational and Physiotherapy<br>• Non-medical specialties such as Clinical and Translational Science, Alternative and Traditional Medicine, Veterinary, Dentistry
**Intervention**	• portfolios in undergraduate and postgraduate medical education for teaching and assessment of Communication, Ethics and Professionalism<br><br>Criteria of a portfolio:<br>• longitudinal (more than a single timepoint) assessment data<br>• candidate's personal engagement with portfolio content and associated learning<br>• Interventions meeting the above criteria were included regardless of whether they were referred to as portfolios	Other documentation methods or learning tools that are:<br>• Not longitudinal or single timepoint<br>• Does not include personal intellectual engagement with the content and associated learning (for instance, curriculum vitae, logbooks and the use of personal digital assistants)

For peer review only - http://bmjopen.bmj.com/site/about/guidelines.xhtml

PICOS	Inclusion criteria	Exclusion criteria
	All types of portfolios were included in the study:<br>• For instance: electronic & non-electronic; formative & summative or combined; clinical & non-clinical<br>• Portfolios with input from students and/or residents and/or doctors, and/or input from faculty members and other individuals<br>• Portfolios with different structures: extent by which the structure has been prescribed and/or left to individual discretion	
**Comparison/ Context**	NA	NA
**Outcome**	Papers that measured the following outcomes were included:<br>• Effectiveness of the use of portfolios to assess and teach Communication, Ethics and Professionalism<br>• Impact of the use of portfolios on medical students (both undergraduate and postgraduate)<br>• Impact of the use of portfolios on the faculty	NA
**Study design**	• Articles in English or translated to English<br>• Articles published from 1st January 2000 to 31st December 2020<br>• Databases: PsycINFO, EMBASE, PubMed, ERIC, Scopus, Google Scholar<br>• All study designs including:<br>• Mixed methods research, meta-analyses, systematic reviews, randomised controlled trials, cohort studies, case-control studies, cross-sectional studies, descriptive papers, grey literature, opinions, letters, commentaries and editorials	NA

D. Identification of Relevant Studies

Guided by the expert team and prevailing descriptions of CEP programs, the research team developed the search strategy for the PubMed, Embase, PsycINFO, ERIC, Scopus, and Google Scholar databases. Independent searches were carried out on the six databases for articles. All research methodologies (quantitative and qualitative) in articles published or translated into English were included.

Keeping with Pham, Rajić (27)'s approach of ensuring a feasible and sustainable research process, the team contained the search to articles published between 1st January 2000 and 31st December 2020 to accommodate existing human resource and time constraints and ensure a sustainable review process.

For peer review only - http://bmjopen.bmj.com/site/about/guidelines.xhtml
For peer review only

E. Selection of Studies Included in Review

Six members of the research team worked independently to identify, review, and create individual lists of all titles and abstracts meeting the inclusion criteria and created individual lists of titles to be included. Concurrently three other members of the research team carried out hand searches and ancestry searches of seven leading journals in medical education (Academic Medicine, Medical Education, Medical Teacher, Advances Health Sciences Education, BMC Medical Education, Teaching and Learning in Medicine and Perspectives on Medical Education) between 14th February 2021 and 18th April 2021. These individual lists of titles were discussed online where individual research team members adopted Sandelowski and Barroso (28)'s "negotiated consensual validation" approach to "*articulate, defend, and persuade others of the "cogency" or "incisiveness" of their points of view or show their willingness to abandon views that are no longer tenable*" to achieve consensus on the final list of titles to be scrutinised.

The research teams then independently reviewed all the full-text articles featured in the final list, created their lists of articles to be included and adopted Sandelowski and Barroso (28)'s "negotiated consensual validation" approach at research meetings to achieve consensus on the final list of articles to be analysed.

F. Assessing the Quality of Articles

Eight research team members individually appraised the quality of the quantitative and qualitative studies using the Medical Education Research Study Quality Instrument (MERSQI) (29) and Consolidated Criteria for Reporting Qualitative Studies (COREQ) (30). This allowed us to evaluate the methodology employed in included articles, and aid readers and reviewers to appraise the extent to which we reported the data, the weight we afforded the data in our analysis and assisted decision-makers to understand the transferability of the findings.

Stage 2 of SEBA: Split Approach

A' Split Approach' was employed to enhance the trustworthiness of the review (31, 32). This entailed concurrent analysis of all full-text articles on the final list of included articles by the three independent groups of reviewers. One group used Braun and Clarke (33)'s approach to thematic analysis, the second group employed Hsieh and

For peer review only - http://bmjopen.bmj.com/site/about/guidelines.xhtml

Shannon (34)'s approach to directed content analysis, whilst the third team created tabulated summaries in keeping with recommendations drawn from Wong, Greenhalgh (35)'s RAMESES publication standards: meta-narrative reviews and Popay, Roberts (36)'s *"Guidance on the conduct of narrative synthesis in systematic reviews"*. The tabulated summaries served to ensure that key aspects of included articles were not lost and be presented in tandem with the quality appraisals of the included articles to help readers evaluate the applicability of the data to their setting. The tabulated summaries also play a part in the Funnelling Process in Stage 4 of the SEBA process.

Using 'negotiated consensual validation' the independent members of the research team compared, discussed and achieved consensus on their individual group findings (28).

Thematic Analysis

In acknowledgement of medical education's socioculturally influenced, Braun and Clarke (33)'s approach to thematic analysis was adopted to allow for due consideration of contextual, individual, organisational, socio-cultural and healthcare, educational, and finance systems influence on the teaching and assessing of CEP. Braun and Clarke (33)'s approach to thematic analysis also allows circumnavigation of the limitations associated with the use of statistical pooling and analysis posed by the presence of a wide range of research methodologies amongst the included articles (33, 37-45).

A reiterative step-by-step thematic analysis was carried out in which 'codes' were constructed from the 'surface' meaning of the text. These were categorised and organised into themes deemed to represent the data best. They were reviewed individually and then as a group. 'Negotiated consensual validation' was used to derive the final list of themes (28, 33, 46).

Directed Content Analysis

Concurrently, the research team independently employed Hsieh and Shannon (34)'s approach to directed content analysis. This involved "*identifying and operationalizing a priori coding categories*" by classifying text of similar meaning into categories drawn from prevailing theories (34, 47-51).

Deductive category application was used to extract codes and categories from the following undergraduate communications and ethics articles: Rider et al. (52) 's *"A model for communication skills assessment across the undergraduate curriculum",* Goldie (53)'s *"Review of ethics curricula in undergraduate medical education"*, and

For peer review only - http://bmjopen.bmj.com/site/about/guidelines.xhtml

the following postgraduate communications and ethics articles: Duffy et al. (54)'s *"Assessing Competence in Communication and Interpersonal Skills: The Kalamazoo II Report"* and Hong et al. (55)'s *"Postgraduate Ethics Training Programs: A Systematic Scoping Review"*. Tay et al. (56)'s *"Assessing Professionalism in Medicine - A Scoping Review of Assessment Tools from 1990 to 2018"* was employed for codes for professionalism in the undergraduate and postgraduate setting. David (57)'s article *"AMEE Medical Education Guide No. 24: Portfolios as a method of student assessment"* was then used to contextualise the codes and ensure they are applicable to portfolio use.

Any data not captured by these codes were assigned a new code. In keeping with deductive category application, coding categories were reviewed and revised as required. The research team discussed their findings online and achieved a consensus on it.

Stage 3 of SEBA: The Jigsaw Perspective

The Jigsaw Perspective sees the themes identified using Braun and Clarke (33)'s approach to thematic analysis and categories identified through Hsieh and Shannon (34)'s approach to directed content analysis, compared to determine overlaps and similarities. These overlaps and similarities provide a holistic picture of available data on portfolio use for teaching and assessing CEP.

To do so, the Jigsaw Perspective adopts Phases 4 to 6 of France et al. (58, 59)'s adaptation of Noblit and Hare (60)'s seven phases of meta-ethnography. Here the themes and the categories identified in the Split Approach are grouped according to their focus. R*eciprocal translation* was used to determine their similarity to one another. This allowed the creation of themes/categories.

Stage 4 of SEBA: The Funnelling Process

The Funnelling Process sees the themes/categories created from the Jigsaw Approach compared with the tabulated summaries to determine their consistency. This process sees the creation of *funnelled domains* that will form 'the line of argument' in the synthesis of the discussion portion in Stage 6 of the SSR in SEBA. In keeping with France et al.'s (58, 59), the themes/categories identified in the Jigsaw Approach were juxtaposed with the key messages identified in the tabulated summaries created from the 82 included full-text articles to contextualise them and ensure that they are truly representative of the data.

For peer review only - http://bmjopen.bmj.com/site/about/guidelines.xhtml

Patient and Public Involvement

No patient involved.

RESULTS

A total of 12,300 abstracts were reviewed, 946 full-text articles were evaluated, and a total of 82 articles were included and analysed (Figure 2). The initial funnelled domains were sorted into three groups corresponding to communication, ethics, and professional portfolios. Within these three groups were four funnelled sub-domains, 1) definitions and functions, 2) portfolio content, 3) portfolio design and implementation and 4) strengths and limitations. These *funnelled sub-domains* were summarised, tabulated, and reviewed as part of SEBA's reiterative process. In keeping with its goals of transparency and accountability, these Tables are enclosed in Appendix A.

(Insert Figure 2. PRISMA Flowchart)

Following an independent review by the research team and the expert team, consensus on the funnelled domains of communication, ethics, and professional portfolios can be combined and tabulated, given similarities between their sub-funnelled domains. Promoting the funnelled sub-domains to funnelled domains allowed them to be tabulated for ease of review.

Funneled Domain 1: Definitions and Functions

Focused portfolios guided with clearly delineated goals (61), competencies (57, 61-77) allow for the curation, structuring and organisation of longitudinal multisource, multimodal assessment data taken at different settings and contexts (71, 78) (Table 2). These portfolios encourage self-directed learning (62, 79-81), self-improvements (71), reflective practice (62-64, 79, 81-92), motivate practitioners to achieve desired competencies (67, 84) and bolster overall professional and personal development (64, 67, 70, 82). Portfolios also assist identification of weak or underperforming students and guide the provision of a comprehensive, targeted feedback (64, 79, 93).

Table 2. *Reasons for Use of Portfolios to Teach CEP*

For peer review only - http://bmjopen.bmj.com/site/about/guidelines.xhtml

**Reasons for Use of Portfolios to Teach CEP**	
**Subthemes**	**Elaboration/Examples**
**Enhancing learning**	Promotion of life-long learning (73, 102)<br><br>Promotion of self-directed learning<br>o Formulation of learning plans (67, 84, 120)<br>o Facilitates Outcome-oriented learning (83, 84, 104, 114)<br>o Enhances self-evaluative capabilities (61, 62, 73, 82, 83, 89, 94, 96, 98, 101, 102, 120)<br>o Identification of strengths and weaknesses (57, 67, 71, 86, 94, 95, 98, 103, 108, 121, 122)<br>o Inculcates responsibility and ownership for one's learning (66, 71, 80, 81, 89, 93, 96, 104)<br>o Sets the pace (102)<br>o Personalised learning (108, 122)<br>o Self-improvement (57, 83, 84, 99)
**Personal and professional development**	Promotes self-understanding (69, 72, 92)<br>o Understanding feelings and thoughts (120, 122)<br>o Understanding of one's values (86)<br>o Understanding activity/experience (57, 82, 118)<br>o Understanding of how one functions in a team (116)<br>o Fears and stressors feelings of inadequacy (118, 123)<br><br>Curates evidence of skills (73)<br>o Skills to compile a portfolio (73, 75, 124-126)<br>o Reflective skills (57, 82, 94, 114, 118, 122, 127)<br>o Clinical skills (102)<br>Organizes information(114, 121)<br>o Documentation of progress (73, 74, 98, 103)<br>o Documentation of skills in different settings(71, 103, 108)<br>o Cross-sectional view of one's competence (57, 74, 103, 108)
**Personalisation**	Allows for personalisation (57, 66, 84, 102, 128)<br>o Personal experiences (57)<br>o Choice over the contents (57, 102, 128)
**Benefits to faculty**	Improvement of curriculum (76, 86)<br>o Identify areas of weakness in curriculum (76)<br>o Provide insights into mentoring, teaching etc (129)<br>Facilitates design of the program (76)<br><br>faculty development<br>o Understand more about how students acquire competencies (102)<br>o Insight into the impact of their interventions (129, 130)<br>o Insight into their professional conduct, role modelling and pastoral care (69)
**E-portfolios**	Ease of use:<br>o Easier use (84)<br>o Accessible (89)<br>o Dissemination and editing of information (66)

Whilst indications for portfolio use in the postgraduate setting are similar to those in the undergraduate setting, many accounts of postgraduate portfolios focus on curating assessments, evidencing attainment of required competencies (74, 92, 94-96) and reflective practice as well as professional identity formation (76, 90, 95, 97) for revalidation purposes (90) (Table 3).

For peer review only - http://bmjopen.bmj.com/site/about/guidelines.xhtml

Table 3. Reasons for Use of Portfolios to Assess CEP

Reasons for Use of Portfolios to Assess CEP
Balance of structure and freeform learning (57, 86, 94, 121, 124, 131)
Adaptable to goals (74, 100, 128)
Alignment of learning outcomes and assessment methods (61, 70, 84, 85, 104)
Allows personalised assessments (74)
Allows assessment over time (80, 86) and stages of development (57) and that facilitate evaluation of:<br>o Learning and progress over time (74, 103)<br>o Learning curve (121, 163)<br>o In different roles (92, 93)<br>o In different settings (78, 100)<br>o Complex behaviours (64, 74, 80, 84, 93, 102, 116, 132)<br>o Critical thinking (61, 70, 74, 128)<br>o Outcomes/competencies (121, 132)<br>o Self-assessment (61, 74, 128)<br>o Complex skills (100)<br>o Professionalism (128)<br>o Cultural competence (128)<br>o Communication skills (128)<br>o Clinical ethics (133)<br>o Problem solving abilities (74, 128)<br>o Reflective abilities (61, 74, 84, 108)
Longitudinal data<br>o Possess good predictive value of subsequent clinical competency (93, 134)<br>o Enhances validity of portfolio grading (57)<br>o Guides better decisions (93, 116, 130)<br>o Provides authentic assessment is a key principle of it (86, 102, 108)<br>o Provides insights in the various core competencies (73)<br>o Fair mode of assessment(102) and allows student to defend positions/work (57)<br>o Provides students with direction and clarity (124, 131)
Allows the combination of Summative and Formative assessment (57, 85) and multisource assessments (81, 87, 97, 108) (108) and triangulation (74, 108)
Facilitates personalised feedback (116) that can be informed by understanding of the individual setting and context (100, 102, 120)

David et al. (57), Davis et al. (64) and Ruiz et al. (89) advocate for the use of portfolios spanning the undergraduate and postgraduate settings.

Funneled Domain 2: Characteristics

Design and implementation principles for undergraduate portfolio structure, faculty involvement, curricular integration, assessors and the frequency of assessments, are delineated in Table 4. Postgraduate portfolios expand

For peer review only - http://bmjopen.bmj.com/site/about/guidelines.xhtml

on these features, adding characteristics of the teaching faculty, the size of the institution, associations with group practice, academic appointments and age of the adult practitioners (98).

In undergraduate and postgraduate settings, the contents of portfolios were shaped by the overall function of the portfolio (74, 76, 96, 99, 100). Clarity on the contents, assessment rubrics, guidance on the organization of the contents (76) facilitated individual practitioners' personalisation of the portfolio (74, 89, 98).

Table 4. Portfolio Design and Implementation

<table>
<tr><th rowspan="2">Themes</th><th rowspan="2">Subthemes</th><th colspan="3">Undergraduate</th></tr>
<tr><th>Communication</th><th>Ethics</th><th>Professionalism</th></tr>
<tr><td rowspan="4">Design and Implementation Principles</td><td>Structure</td><td colspan="3">Clear guidelines (57, 61, 63, 67, 70, 82, 122)
• Use of portfolio (57, 82)
• Portfolio content - determining competencies to assess (57, 85)
• Promotional decisions (57, 69, 71, 83, 135)
Standard-setting exercises (79)
Provision of sample portfolios for reference (61, 68, 115)
• Roles for assessors (57, 65, 69, 71, 72, 93, 135)
• Expectations of students (131)
• Duration spanning across preclinical and clinical years (126)
Well-defined structure with room for flexibility (57, 61, 81, 82, 84)
Student-centered (84)
Appropriate student to mentor ratio (71, 125)
Sufficient curricular experience for reflection, feedback (71)
Protected time for reflection, feedback and regular meetings with mentors (63, 71, 93, 122)</td></tr>
<tr><td>Faculty</td><td colspan="3">Good faculty support (69, 126)
Ensure culture is supportive of portfolio system (71, 126)
Flexible and enthusiastic tutors passionate and experienced mentors (62, 82)</td></tr>
<tr><td>Curricular Integration</td><td colspan="3">Early introduction of portfolio (66, 82, 122, 136)
Easing portfolio into curriculum (57, 64, 81, 82, 84) – including orientation, training and introductory sessions (57, 61, 64, 68, 69, 82, 84) for learners and staff training (57, 62, 64, 71, 79, 80, 82)
Implementation of portfolios throughout curriculum (69)
Portfolio content aligning with phase of medical journey and competencies required (57, 68, 70, 71, 84, 135)</td></tr>
<tr><td>Assessments</td><td colspan="3">Clear scoring rubrics (57, 126)
Alignment between assessments and outcomes (63, 81, 84)
Transparent assessment criteria and outcomes to maintain fairness (64, 66, 71, 115)
Standardisation of portfolio use and content (63, 81), assessors training (64) and increased number of assessors (63, 81, 136) to improve assessment reliability (63, 81, 136)
Separate processes and reviewers for summative and formative portfolio (71) – summative assessments to specify clear pass/fail requirements (57)
Incorporation of student feedback into portfolio development (57, 64, 84)</td></tr>
</table>

For peer review only

For peer review only - http://bmjopen.bmj.com/site/about/guidelines.xhtml

	**Assessors**	External examiners (57)<br>Faculty members (57, 65, 71, 72, 93, 135)<br>Mentors (121)<br><br>Senior staff to be paired with new examiners (57)<br>Mentors should not assess own student (81, 86)
	**Assessment Frequency**	Formative assessments to monitor and guide student learning – conducted throughout the year (81, 82, 85)<br><br>Summative assessments to evaluate learner performance – conducted annually (57, 62, 64, 68, 71, 79, 80, 84, 85, 93, 122, 135, 137-140), twice (71, 79, 116), thrice (82) or multiple times a year (74, 92)
	**Oversight and Delegation**	Establishment of portfolio oversight committee (71, 79)<br>Regular programme evaluation (71)<br>Clear division of support roles – e.g. pedagogical and technical support (66)

Funneled Domain 3: Assessed Content

Postgraduate CEP competencies built on those employed in undergraduate portfolios (Table 5). Postgraduate portfolios included assessment of advanced and longitudinal topics such as discussion of do-not-resuscitate orders (101), the resolution of ethically challenging clinical situations such as life-and-death decision-making with families of critically ill patients (95, 101, 102), dealing with and disclosure of medical errors (76, 103), attention to one's body language, defence mechanism, emotional intelligence (104), psychosocial awareness, the integration of the appropriate ethical principles behind these issues, health equity, global health and individual ethics consultations (105), multidisciplinary team collaboration (95), the execution/understanding of infection control (77), organ donation and withdrawal of care exercises (102), and strict compliance with institutional and departmental policies (75, 77, 103).

Undergraduate and postgraduate CEP portfolios employed similar assessment methods (Table 2) though postgraduate portfolios also added modified essay questions (MEQ)s (92), polypharmacy journal/audits (78, 91, 99, 102, 106), professional workshops and training sessions (4, 73, 100, 102, 107), health service meeting reports (92) and reviews by medical regulatory authorities (91).

Table 5. Portfolio Content (Competencies Assessed and Assessment Modalities)

Themes	Subthemes	Undergraduate		
		Communication	Ethics	Professionalism
**Portfolio Content**	**Competencies Assessed**	• "Effective" communication (57, 62, 64, 71, 84, 115, 124, 137-139)	• Ethics in interprofessional practice (56, 68,	• Learning from clinical experience (56, 62)

For peer review only - http://bmjopen.bmj.com/site/about/guidelines.xhtml

		• Professional behaviour and moral reasoning (79)<br>• Systems awareness and team-based care (79)<br>• Continuous learning and quality improvement (79)<br>• Patient-centred medical care (79)<br>• Teamwork (93)<br><br>Oral communication skills<br>• Attentive, active listening (82, 141, 142)<br>• Explanation, information giving (115, 141)<br>• History taking (70)<br>• Consultation (143)<br>• Breaking bad news (56, 141)<br>• Shared decision making (144)<br>• Empathetic communication (144)<br>• Taking history and clinical examination (70, 82)<br>• Discerning appropriate language to ensure patient understands (56, 115)<br>• Conflict management (141)<br>• Adaptability to different settings (71)<br><br>Written communication skills<br>• Proper grammar, spelling, logical and coherent structure (121)<br>• Patient cases (70)<br>• Scientific research (70)<br><br>Communication with:<br>• Patients (56, 64, 70, 82, 115, 141, 145)<br>• Children (115)<br>• Family (64, 82, 141, 146)	71, 84, 136, 147, 148)<br>• Doctor-patient relationship (62, 143, 147)<br>• Values/ ethics (71, 84, 93, 148)<br>• Ethical and legal responsibilities (57, 84)<br>• Moral reasoning (87)<br>• Ethics is assessed as a component of professionalism (83, 85, 86)<br>• Empathy (82)<br>• Showing respect and commitment (82)<br>• Respect for privacy (82)<br>• How to express ethical views appropriately (81)<br>• Identifying unethical moments of practice and grey areas (83, 87, 88)<br><br>Reflection:<br>• Quality of student's reflections (81, 82, 85)<br>• Authenticity of reflection (85)	• Working in groups/ teamwork (56, 62, 68, 71, 84, 93, 136, 140, 147)<br>• Doctor-patient relationship (62, 143, 147)<br>• Becoming a doctor/roles and responsibilities (62, 68, 84, 136, 140, 147, 148)<br>• Patient care (68, 71, 93, 136)<br>• Continuous learning (71, 93)<br>• Values/ ethics (71, 84, 93, 148)<br>• Interpersonal skills/ effective Communication, (56, 68, 71, 147, 148)<br><br>Reflection:<br>• Clinical experience (62)<br>• Teaching (62)<br>• Learning needs in becoming a doctor<br>• Strengths and developmental areas (57, 84)<br>• Understanding of concepts addressed (84)<br>• Dilemmas and doubts (139)

For peer review only

		• Colleagues, interprofessional team (56, 64, 70, 82, 136, 141)<br>• Public (64)		
	**Assessment Modalities (Portfolio items)**	Formative only (67, 71, 82, 85, 123, 135, 136, 148)<br>Summative only (57, 62, 63, 72, 79, 81, 85, 93, 122, 137, 140)<br>Both formative and summative (56, 63, 68, 69, 71, 84, 85, 126, 136, 140)<br>MCQs (57, 64, 85, 102, 135, 143, 145, 146, 148)<br>OSCEs (57, 71, 79, 85, 93, 115, 135, 136, 143, 145, 146, 148)<br>Essays – topical (62, 70, 71, 135)<br>Mini clinical examination exercise (Mini-CEX) (57, 70, 71, 85, 93, 136, 141, 145, 148)<br>Medical/patient presentations/research (64, 65, 70, 71, 80, 84, 125, 135)<br>Workplace-based clinical (63, 70, 71, 79, 80, 93, 115, 116, 124, 126, 135, 136, 141, 145)<br>Case-based discussions, summaries (57, 63-65, 67, 83, 87)(19)(62, 65)(63, 135, 136)<br>Simulated patient assessment (57, 64, 82, 116, 121)<br>Team-based assessment (57, 63, 70, 84)<br>Logbooks/ Portfolio interview (57, 62-64, 71, 81-83, 85, 86, 93, 121, 125, 135) (71, 125, 135)<br>Peer evaluation (70, 71, 79, 80, 83, 93, 115, 124, 135)<br>Faculty evaluation (62-64, 69, 71, 79, 80, 82-84, 93, 135, 137, 149)<br>Feedback reports (70, 80, 84, 90-92, 100, 116, 126, 135, 136, 140, 141, 145) – multisource feedback (62, 70, 81, 83, 84, 90-92, 100, 115, 121, 136)<br>Self-assessment/appraisal (64, 70, 71, 80, 81, 122, 135, 136, 140)<br>Self-reflections – written/video/diaries (57, 62-65, 68, 70, 71, 79-81, 83, 85, 86, 88, 115, 121, 124, 125, 136)<br>Learning plans and contracts (62-64, 67, 70, 71, 79-81, 84, 121, 139)<br>Letters of recommendation (57, 62, 121)<br>Curriculum vitae (57, 63, 71, 84, 93, 126, 135)		

Funneled Domain 4: Strengths and Limitations

The strengths and limitations of portfolios are outlined in Table 6. Postgraduate portfolios provide a deeper understanding of lifelong learning and continuous professional development (73, 74, 76, 90, 96, 100, 102, 103, 107, 108) and better recognise accomplishments, especially those that would have otherwise have gone undocumented (73, 92). Postgraduate portfolios were found to possess a good inter-rater reliability (96) through the triangulation of multiple instruments and observations based on multiple samples of behaviour (74, 108) and "authentic evidence" from the workplace (90, 102). Critically, portfolios allow for early detection of resident stress and burnout (107).

A key limitation to CEP portfolios is maintaining compliance with portfolio use (101). This issue is attributed to a lack of appreciation for its value (77, 106), struggles to balance both learning and work (94), an apparent lack

For peer review only

of relevance of portfolio content (94) and poor support of reflections and their role in the attainment of competencies (90, 108).

Table 6. Strengths and Limitations

Themes	Subthemes	Undergraduate		
		Communication	Ethics	Professionalism
**Strengths/ Limitations**	**Strengths**	For students<br>Identification of own strengths and weaknesses (57, 62, 63, 67, 79, 81, 82, 84, 86, 93, 120, 140)<br>Appreciation of effective communication (82, 136, 147), ethical issues (86), and fosters critical professional attitudes (63, 142)<br>Encourages self-regulation (140) through opportunities for reflection (81) and self-assessment (69, 82, 122)<br>Facilitates preparation for post-graduate medical training (82, 121, 124)<br><br>For faculty<br>Identification of areas of concern and allows for early, timely intervention (79, 93)<br>Enhances interaction between students and teachers (57, 82, 86) Facilitates reiterative reassessment of teaching strategies (57)<br><br>Assessments<br>Longitudinal (116)<br>Good validity (93)<br>Reliable assessment tool – allows for triangulation of information and evidence from multiple sources and contexts (71)<br>Ability to assess a wide range of competencies (70, 84)<br>Allows for more authentic assessments (93)<br>Gives faculty greater insight into students' achievement of behaviourally-oriented competencies that traditional methods do not (79)<br>Evaluates achievement during clinical attachment/clerkship (70)<br>Provides a basis for the judgement of a student's professional fitness to practice (64)		
	**Limitations**	Time-consuming and labour intensive (62, 64, 67, 79, 82-85, 121, 126, 136)<br>Issues with reliability (72, 81, 84, 136)<br>Inconsistent effects (81, 84, 122, 148)<br>Lack of honesty by students in reflections (64, 71, 81, 84, 85, 121, 122, 131)		

For peer review only

Stage 5 of SEBA: Analysis of data and non-data driven literature

Findings from the different stages of SEBA were discussed with members of the expert team and relevant stakeholders. There were concerns from the expert team about the impact of grey literature on the narrative, given that they were neither peer-reviewed nor necessarily evidence-based. As a result, the research team differentiated correspondence, letters, editorials and perspective pieces from academic databases and grey literature from data-driven or research-based peer-reviewed data. Both groups were then independently analysed. The themes/categories identified were then compared to enhance further the accountability and the reproducibility of

For peer review only - http://bmjopen.bmj.com/site/about/guidelines.xhtml

Stage 5 of SEBA. Evidence-based data from bibliographic databases (henceforth evidence-based publications) were separated from grey literature, perspectives, editorials, letters, and non-data-based articles drawn from bibliographic databases (henceforth non-data driven). These two groups were separately thematically analysed.

Stage 6 of SEBA: Synthesis of the Discussion

The Best Evidence Medical Education (BEME) Collaboration Guide (44) and the Structured approach to the Reporting In healthcare education of Evidence Synthesis (STORIES) (45) were used to guide the discussion.

DISCUSSION

On the surface in answering its research questions, this SSR in SEBA provides insights into two areas of interest. The first is insights into CEP portfolios. Acknowledging that CEP development is a highly personalised, socioculturally informed construct, this SSR in SEBA offer a consistent framework replete with clearly delineated goals (61) and competencies (57, 61-77), agreed upon endpoints and outcome measures, the curation, structuring and organisation of longitudinal multisource, multimodal assessment data (71, 78). This helps establish and align expectations and plot the developmental path for the individual. In addition, CEP portfolios bring together and integrates the closely intertwined aspects of communication, ethics and professionalism training, assessment and feedback allowing for holistic and longitudinal support for these competencies (62, 79-81) (104), (75, 77, 103, 105). Given the longitudinal trajectory of CEP competencies and the presence of overlaps in the assessment and oversight of CEP competencies, CEP portfolios also allow for the longitudinal, multi-source triangulation of assessment data and feedback (64, 79, 93). Similarly CEP portfolios facilitate reflection (62-64, 79, 81-92), self-improvement (71), and bolster overall efforts for professional and personal development (64, 67, 70, 82). Overall these findings echo the positions taken by the Top 10 medical schools featured on the QS World University Rankings 2020 that have CEP taught, role modelled, and assessed in tandem within a structured longitudinal program (109).

Perhaps just as significantly this SSR in SEBA provides insights into the design, structure, contents, assessments and the required support use of portfolios in programs. Here the flexibility of current concepts of CEP portfolios also affords them the luxury of adapting their contents to suit the setting and needs of the medical student or physician laying the foundation for their use across medical school to the continuing medical education stage.

For peer review only - http://bmjopen.bmj.com/site/about/guidelines.xhtml

It is this longitudinal, structured use that sees development across closely intertwined personal, cultural, professional, organizational and societal planes that promotes the idea that CEP portfolios could influence PIF. In addition by encouraging lifelong learning, boosting CEP knowledge, skills, and competencies, recognizing the accomplishments and the integration of reflections, insights and experiences into an individual's practice and conduct, CEP portfolios meet the basic description of PIF (73, 74, 76, 90, 96, 100, 102, 103, 107, 108). Guided by triangulated multisource workplace appraisals and practice related assessments, CEP portfolios help medical students and physicians build on their prevailing skills, knowledge, abilities, narratives, and competencies to grow and also detect distressed medical students and physicians. This underlines CEP portfolio's credentials as a user-specific, structured process that facilitates personal and professional growth over time.

Having affirmed the CEP portfolio's role in nurturing PIF, we shift our attention to operationalising the findings of the SSR in SEBA. To individualise support, guided reflections, mentoring, role modelling, timely feedback, remediation, and development Tan et al. (110) and Hong et al. (55) employ adaptations of Norcini (111)'s concept of micro-credentialling and micro-certification in medical education (112, 113). At its core are general and personalised milestones that allow for the establishment of general and personalised micro-competencies. 'General micro-competencies' are host organization-established milestones and timelines that facilitate appraisal of the practitioner's general progress (112, 113) whilst 'personalised micro-competencies' account for the practitioner's particular contextual considerations, knowledge, skills, experiences, and attitudes and facilitated more contextualised assessments of progress and development (112). The combination of general and personalised micro-competencies guide reflections, direct mentoring, shape role modelling and facilitate individualized feedback and support within a customized learning environment. It is here in bringing together elements of the mentoring-umbrella to shape PIF.

Operationalizing a CEP portfolio to nurture PIF requires a robust, accessible, and well supported electronic platform. Portfolio implementation must also be accompanied by clearly established goals, purpose, role, learning objectives, content, micro-competencies, and assessment guidelines (81, 114, 115). This will help structure and assess the portfolio yet ensure that it is sufficiently flexible to extend from medical school to the postgraduate setting (69, 72, 92). These features underline the need for a dedicated faculty (82) capable of effective role modelling (69), support (57, 70, 82, 86, 92) and providing feedback (116). The host organization must also structure and support the recruitment and training of enthusiastic faculty (92), provide them with support,

For peer review only - http://bmjopen.bmj.com/site/about/guidelines.xhtml

recognition (78) and oversight (100) and ensure they are afforded protected time for administrative duties, coordination and assessments (61, 74, 84, 103, 106, 117-119). Critically with CEP essential to all forms of training medical education, these findings may be of interests to healthcare professionals beyond the medical sphere.

There is another facet of CEP portfolios that bears further consideration. CEP portfolios allow PIF to be seen through the eyes of the individual medical student or physician rather than solely through the lens of organizational, clinical, research, professional and academic expectations. The CEP portfolio's longitudinal approach allows individual progress and development to be assessed on a personal basis considering the individual's current concepts, narratives, motivations, abilities and availabilities and context. This novel personalised approach allows for right siting of assessments and the direction of prompt, accessible, individualised, necessary, continuing, and comprehensive support.

LIMITATIONS

The generalisability of the results of this study is limited by the identified studies selected for review. Firstly, the majority of the papers included originate from Europe and North America. Given that CEP is a sociocultural construct, these geopolitical sociocultural differences raise questions as to the applicability of these findings beyond the European and North American medical education systems. Secondly, as the number of articles included is limited, the SSR in SEBA's ability to assess the long-term effectiveness of portfolios may be compromised.

CONCLUSIONS

In employing a CEP portfolio to lay the foundation for the nurturing of PIF, this SSR in SEBA has underlined the need for a longitudinal curation, assessment and support mechanism that begins in medical school and continues into the postgraduate training and continuing medical education settings. However, perhaps strikingly, the CEP portfolio delivers an individualised perspective of PIF that demands closer scrutiny. As we look forward to furthering efforts to develop PIF, we believe the specific impact of these potentially resource-heavy and financially costly interventions should be at the centre of PIF initiatives.

ABBREVIATIONS

CEP Communication, Ethics, Professionalism

For peer review only - http://bmjopen.bmj.com/site/about/guidelines.xhtml
For peer review only

**PIF**	Professional Identity Formation
**SSR**	Systematic Scoping Review
**SEBA**	Systematic Evidence Based Approach
**PICOS**	Population, Intervention, Comparison and Outcome
**BEME**	Best Evidence Medical Education
**STORIES**	Structured approach to the Reporting In healthcare education of Evidence Synthesis

STATEMENTS AND DECLARATIONS

Ethics Approval and Consent to Participate NA

Consent for Publication NA

Availability of Data and Materials All data generated or analysed during this review are included in this published article and its appendices.

Competing Interests LKRK, JJQT, DZH, BKYL, AJSL, EJXC, AP, RT, JYHY, YZK, CWNQ, JYL, KTT, YTO, MC, JZ, GP, SM, LW have no competing interests.

Funding No funding was received for this review

Author Contributions LKRK, JJQT, DZH, BKYL, AJSL, EJXC, AP, RT, JYHY, YZK, CWNQ, JYL, KTT, YTO, MC, JZ, GP, SM, LW were involved in data curation, formal analysis, investigation, preparing the original draft of the manuscript as well as reviewing and editing the manuscript. All authors have read and approved the manuscript.

ACKNOWLEDGEMENTS

The authors would like to dedicate this paper to the late Dr. S Radha Krishna and the late Dr. Cynthia Goh whose advice and ideas were integral to the success of this study. The authors would also like to thank Alexia Sze Inn Lee and Nur Diana Abdul Rahman~~, for t~~heir contributions.

REFERENCES

1. Cruess RL, Cruess SR, Boudreau JD, Snell L, Steinert Y. Reframing medical education to support professional identity formation. Acad Med. 2014;89(11):1446-51.
2. Cruess RL, Cruess SR, Boudreau JD, Snell L, Steinert Y. A schematic representation of the professional identity formation and socialization of medical students and residents: a guide for medical educators. Acad Med. 2015;90(6):718-25.
3. Sarraf-Yazdi S, Teo YN, How AEH, Teo YH, Goh S, Kow CS, et al. A Scoping Review of Professional Identity Formation in Undergraduate Medical Education. J Gen Intern Med. 2021;36(11):3511-21.

For peer review only - http://bmjopen.bmj.com/site/about/guidelines.xhtml

4. Yates J, James D. Risk factors at medical school for subsequent professional misconduct: multicentre retrospective case-control study. BMJ. 2010;340:c2040.
5. Papadakis MA, Hodgson CS, Teherani A, Kohatsu ND. Unprofessional behavior in medical school is associated with subsequent disciplinary action by a state medical board. Acad Med. 2004;79(3):244-9.
6. Papadakis MA, Teherani A, Banach MA, Knettler TR, Rattner SL, Stern DT, et al. Disciplinary action by medical boards and prior behavior in medical school. N Engl J Med. 2005;353(25):2673-82.
7. Papadakis MA, Loeser H, Healy K. Early detection and evaluation of professionalism deficiencies in medical students: one school's approach. Acad Med. 2001;76(11):1100-6.
8. O'Regan A, Smithson WH, Spain E. Social media and professional identity: Pitfalls and potential. Med Teach. 2018;40(2):112-6.
9. Cruess SR, Cruess RL, Steinert Y. Supporting the development of a professional identity: General principles. Med Teach. 2019;41(6):641-9.
10. Apker J, Eggly S. Communicating Professional Identity in Medical Socialization: Considering the Ideological Discourse of Morning Report. Qualitative Health Research. 2004;14(3):411-29.
11. Whitehead C, Kuper A, Freeman R, Grundland B, Webster F. Compassionate care? A critical discourse analysis of accreditation standards. Med Educ. 2014;48(6):632-43.
12. Kjaer NK, Kodal T, Qvesel D. The role of general practice in postgraduate basic training. Med Teach. 2010;32(10):e448-52.
13. Shubkin CD, Garrett JR, Lantos JD. When Residents Let Conscience Be Their Guide: Professional Development and Educational Opportunity.
. Acad Pediatr 2018;18(3):239-42.
14. Kow CS, Teo YH, Teo YN, Chua KZY, Quah ELY, Kamal NHBA, et al. A systematic scoping review of ethical issues in mentoring in medical schools. BMC Medical Education. 2020;20(1):1-10.
15. Bok C, Ng CH, Koh JWH, Ong ZH, Ghazali HZB, Tan LHE, et al. Interprofessional communication (IPC) for medical students: a scoping review. BMC Medical Education. 2020;20(1):372.
16. Ngiam LXL, Ong YT, Ng JX, Kuek JTY, Chia JL, Chan NPX, et al. Impact of Caring for Terminally Ill Children on Physicians: A Systematic Scoping Review. American Journal of Hospice and Palliative Medicine®. 2020:1049909120950301.
17. Krishna LKR, Tan LHE, Ong YT, Tay KT, Hee JM, Chiam M, et al. Enhancing Mentoring in Palliative Care: An Evidence Based Mentoring Framework. Journal of Medical Education and Curricular Development. 2020;7:2382120520957649.
18. Kamal NHA, Tan LHE, Wong RSM, Ong RRS, Seow REW, Loh EKY, et al. Enhancing education in Palliative Medicine: the role of Systematic Scoping Reviews. Palliative Medicine & Care: Open Access. 2020;7(1):1-11.
19. Ong RRS, Seow REW, Wong RSM, Loh EKY, Kamal NHA, Mah ZH, et al. A Systematic Scoping Review of Narrative Reviews in Palliative Medicine Education. Palliative Medicine & Care: Open Access. 2020;7(1):1-22.
20. Mah ZH, Wong RSM, Seow REW, Loh EKY, Kamal NHA, Ong RRS, et al. A Systematic Scoping Review of Systematic Reviews in Palliative Medicine Education. Palliative Medicine & Care: Open Access. 2020;7(1):1-12.
21. Kow CS, Teo YH, Teo YN, Chua KZY, Quah ELY, Kamal N, et al. A systematic scoping review of ethical issues in mentoring in medical schools. BMC Med Educ. 2020;20(1):246.

For peer review only - http://bmjopen.bmj.com/site/about/guidelines.xhtml

22. Bok C, Ng CH, Koh JWH, Ong ZH, Ghazali HZB, Tan LHE, et al. Interprofessional communication (IPC) for medical students: a scoping review. BMC Med Educ. 2020;20(1):372.
23. Krishna LKR, Tan LHE, Ong YT, Tay KT, Hee JM, Chiam M, et al. Enhancing Mentoring in Palliative Care: An Evidence Based Mentoring Framework. J Med Educ Curric Dev. 2020;7:2382120520957649.
24. Cleland J, McKimm J, Fuller R, Taylor D, Janczukowicz J, Gibbs T. Adapting to the impact of COVID-19: Sharing stories, sharing practice. Medical Teacher. 2020;42(7):772-5.
25. Peters M, Godfrey C, McInerney P, Soares C, Khalil H, Parker D. The Joanna Briggs Institute reviewers' manual 2015: methodology for JBI scoping reviews2015 April 29, 2019. Available from: http://joannabriggs.org/assets/docs/sumari/Reviewers-Manual_Methodology-for-JBI-Scoping-Reviews_2015_v1.pdf.
26. Peters MD, Godfrey CM, Khalil H, McInerney P, Parker D, Soares CB. Guidance for conducting systematic scoping reviews. Int J Evid Based Healthc. 2015;13(3):141-6.
27. Pham MT, Rajić A, Greig JD, Sargeant JM, Papadopoulos A, McEwen SA. A scoping review of scoping reviews: advancing the approach and enhancing the consistency. Res Synth Methods. 2014;5(4):371-85.
28. Sandelowski M, Barroso J. Handbook for synthesizing qualitative research: springer publishing company; 2006.
29. Reed DA, Beckman TJ, Wright SM, Levine RB, Kern DE, Cook DA. Predictive validity evidence for medical education research study quality instrument scores: quality of submissions to JGIM's Medical Education Special Issue. J Gen Intern Med. 2008;23(7):903-7.
30. Tong A, Sainsbury P, Craig J. Consolidated criteria for reporting qualitative research (COREQ): a 32-item checklist for interviews and focus groups. Int J Qual Health Care. 2007;19(6):349-57.
31. Chua WJ, Cheong CWS, Lee FQH, Koh EYH, Toh YP, Mason S, et al. Structuring Mentoring in Medicine and Surgery. A Systematic Scoping Review of Mentoring Programs Between 2000 and 2019. Journal of Continuing Education in the Health Professions. 2020;40(3):158-68.
32. Ng YX, Koh ZYK, Yap HW, Tay KT, Tan XH, Ong YT, et al. Assessing mentoring: A scoping review of mentoring assessment tools in internal medicine between 1990 and 2019. PLOS ONE. 2020;15(5):e0232511.
33. Braun V, Clarke V. Using thematic analysis in psychology. Qualitative research in psychology. 2006;3(2):77-101.
34. Hsieh H-F, Shannon SE. Three Approaches to Qualitative Content Analysis. Qualitative Health Research. 2005;15(9):1277-88.
35. Wong G, Greenhalgh T, Westhorp G, Buckingham J, Pawson R. RAMESES publication standards: meta-narrative reviews. BMC medicine. 2013;11(1):20.
36. Popay J, Roberts H, Sowden A, Petticrew M, Arai L, Rodgers M, et al. Guidance on the conduct of narrative synthesis in systematic reviews. A product from the ESRC methods programme Version. 2006;1:b92.
37. Sng JH, Pei Y, Toh YP, Peh TY, Neo SH, Krishna LKR. Mentoring relationships between senior physicians and junior doctors and/or medical students: A thematic review. Med Teach. 2017;39(8):866-75.
38. Wahab MT, Ikbal MFBM, Wu J, Loo WTW, Kanesvaran R, Krishna LKR. Creating Effective Interprofessional Mentoring Relationships in Palliative Care- Lessons from

For peer review only - http://bmjopen.bmj.com/site/about/guidelines.xhtml

Medicine, Nursing, Surgery and Social Work. Journal of Palliative Care & Medicine. 2016;06(06).
39. Toh YP, Lam B, Soo J, Chua KLL, Krishna L. Developing Palliative Care Physicians through Mentoring Relationships. Palliative Medicine & Care. 2017;4(1):1-6.
40. Wu J, Wahab MT, Ikbal MFBM, Loo TWW, Kanesvaran R, Krishna LKR. Toward an Interprofessional Mentoring Program in Palliative Care - A Review of Undergraduate and Postgraduate Mentoring in Medicine, Nursing, Surgery and Social Work. Journal of Palliative Care & Medicine. 2016;06(06):1-11.
41. Soemantri D, Herrera C, Riquelme A. Measuring the educational environment in health professions studies: a systematic review. Medical teacher. 2010;32(12):947-52.
42. Schönrock-Adema J, Heijne-Penninga M, van Hell EA, Cohen-Schotanus J. Necessary steps in factor analysis: enhancing validation studies of educational instruments. The PHEEM applied to clerks as an example. Medical teacher. 2009;31(6):e226-e32.
43. Riquelme A, Herrera C, Aranis C, Oporto J, Padilla O. Psychometric analyses and internal consistency of the PHEEM questionnaire to measure the clinical learning environment in the clerkship of a Medical School in Chile. Medical teacher. 2009;31(6):e221-e5.
44. Haig A, Dozier M. BEME Guide no 3: systematic searching for evidence in medical education--Part 1: Sources of information. Medical teacher. 2003;25(4):352-63.
45. Gordon M, Gibbs T. STORIES statement: publication standards for healthcare education evidence synthesis. BMC medicine. 2014;12(1):143.
46. Stenfors-Hayes T, Kalén S, Hult H, Dahlgren LO, Hindbeck H, Ponzer S. Being a mentor for undergraduate medical students enhances personal and professional development. Medical Teacher. 2010;32(2):148-53.
47. Neal JW, Neal ZP, Lawlor JA, Mills KJ, McAlindon KJA, Health PiM, et al. What makes research useful for public school educators? 2018;45(3):432-46.
48. Wagner-Menghin M, de Bruin A, van Merriënboer JJJAiHSE. Monitoring communication with patients: analyzing judgments of satisfaction (JOS). 2016;21(3):523-40.
49. Elo S, Kyngäs HJJoan. The qualitative content analysis process. 2008;62(1):107-15.
50. Mayring PJActqr. Qualitative content analysis. 2004;1:159-76.
51. Humble ÁMJIJoQM. Technique triangulation for validation in directed content analysis. 2009;8(3):34-51.
52. Rider EA, Hinrichs MM, Lown BA. A model for communication skills assessment across the undergraduate curriculum. Med Teach. 2006;28(5):e127-34.
53. Goldie J. Review of ethics curricula in undergraduate medical education. Med Educ. 2000;34(2):108-19.
54. Duffy FD, Gordon GH, Whelan G, Cole-Kelly K, Frankel R, Buffone N, et al. Assessing competence in communication and interpersonal skills: the Kalamazoo II report. Acad Med. 2004;79(6):495-507.
55. Hong DZ, Goh JL, Ong ZY, Ting JJQ, Wong MK, Wu J, et al. Postgraduate ethics training programs: a systematic scoping review. BMC Medical Education. 2021;21(1):338.
56. Tay KT, Ng S, Hee JM, Chia EWY, Vythilingam D, Ong YT, et al. Assessing Professionalism in Medicine - A Scoping Review of Assessment Tools from 1990 to 2018. J Med Educ Curric Dev. 2020;7:2382120520955159.
57. David M, Davis M, Harden R, Howie P, Ker J, Pippard M. AMEE Medical Education Guide No. 24: Portfolios as a method of student assessment. Medical teacher. 2001;23:535-51.

For peer review only - http://bmjopen.bmj.com/site/about/guidelines.xhtml

58. France EF, Wells M, Lang H, Williams B. Why, when and how to update a meta-ethnography qualitative synthesis. Syst Rev. 2016;5:44.
59. France EF, Uny I, Ring N, Turley RL, Maxwell M, Duncan EAS, et al. A methodological systematic review of meta-ethnography conduct to articulate the complex analytical phases. BMC Med Res Methodol. 2019;19(1):35.
60. Noblit GW, Hare RD. Meta-ethnography: synthesizing qualitative studies. California: Sage Publications; 1988.
61. Azer S. Use of Portfolios by Medical Students: Significance of Critical Thinking. The Kaohsiung journal of medical sciences. 2008;24:361-6.
62. O'Sullivan A, Howe A, Miles S, Harris P, Hughes C, Jones P, et al. Does a summative portfolio foster the development of capabilities such as reflective practice and understanding ethics? An evaluation from two medical schools. Medical teacher. 2012;34:e21-8.
63. Haffling A-C, Beckman A, Pahlmblad A, Edgren G. Students' reflections in a portfolio pilot: Highlighting professional issues. Medical teacher. 2010;32:e532-40.
64. Davis M, Ben-David M, Harden R, Howie P, Ker J, McGhee C, et al. Portfolio assessment in medical students' final examination. Medical teacher. 2001;23:357-66.
65. Michels N, Driessen E, Muijtjens A, Van Gaal L, Bossaert L, De Winter B. Portfolio assessment during medical internships: How to obtain a reliable and feasible assessment procedure? Education for health (Abingdon, England). 2009;22:313.
66. Moores A, Parks M. Twelve tips for introducing E-Portfolios with undergraduate students. Medical teacher. 2010;32:46-9.
67. Sambandam E, Jutti R, Lee L. Portfolio as a learning tool: Students' perspective. Annals of the Academy of Medicine, Singapore. 2005;34:511-4.
68. Kassab S, Bidmos M, Nomikos M, Daher-Nashif S, Kane T, Sarangi S, et al. Construct Validity of an Instrument for Assessment of Reflective Writing-Based Portfolios of Medical Students. Advances in Medical Education and Practice. 2020;Volume 11:397-404.
69. Hall P, Byszewski A, Sutherland S, Stodel E. Developing a Sustainable Electronic Portfolio (ePortfolio) Program That Fosters Reflective Practice and Incorporates CanMEDS Competencies Into the Undergraduate Medical Curriculum. Academic medicine : journal of the Association of American Medical Colleges. 2012;87:744-51.
70. Michels N, Avonts M, Peeraer G, Ulenaers K, Gaal L, Bossaert L, et al. Content validity of workplace-based portfolios: A multi-centre study. Medical Teacher. 2016;38:1-10.
71. Dannefer E, Henson L. The Portfolio Approach to Competency-Based Assessment at the Cleveland Clinic Lerner College of Medicine. Academic medicine : journal of the Association of American Medical Colleges. 2007;82:493-502.
72. Ekayanti F, Risahmawati R, Fadhilah M. Portfolio Assessment Implementation in Clinical Year of Community Medicine Module: Students Perspective2017.
73. Finlay K, Probyn L, Ho S. The CanMEDS Resume: A Useful Educational Portfolio Tool for Diagnostic Radiology Residents. Canadian Association of Radiologists journal = Journal l'Association canadienne des radiologistes. 2011;63:233-6.
74. Shumway J, Harden R. AMEE Guide No. 25: The assessment of learning outcomes for the competent and reflective physician. Medical teacher. 2003;25:569-84.
75. Fragneto R, Dilorenzo A, Schell R, Bowe E. Evaluating Practice-Based Learning and Improvement: Efforts to Improve Acceptance of Portfolios. Journal of graduate medical education. 2010;2:638-43.

For peer review only

76. O'Sullivan P, Reckase M, McClain T, Savidge M, Clardy J. Demonstration of Portfolios to Assess Competency of Residents. Advances in health sciences education : theory and practice. 2004;9:309-23.
77. Deitte L. Learning Portfolios in Radiology Residency Education: How Do I Get Started? Journal of the American College of Radiology : JACR. 2008;5:664-9.
78. McEwen L, Griffiths J, Schultz K. Developing and Successfully Implementing a Competency-Based Portfolio Assessment System in a Postgraduate Family Medicine Residency Program. Academic medicine : journal of the Association of American Medical Colleges. 2015;90.
79. O'Brien C, Sanguino S, Thomas J, Green M. Feasibility and Outcomes of Implementing a Portfolio Assessment System Alongside a Traditional Grading System. Academic medicine : journal of the Association of American Medical Colleges. 2016;91.
80. Yielder J, Moir F. Assessing the Development of Medical Students' Personal and Professional Skills by Portfolio. Journal of Medical Education and Curricular Development. 2016;2016:9.
81. Driessen E, Tartwijk J, Vermunt J, Van der Vleuten C. Use of Portfolios in early undergraduate medical training. Medical teacher. 2003;25:18-23.
82. Fida N, Hassanien M, Shamim M, Alafari R, Zaini R, Mufti S, et al. Students' perception of portfolio as a learning tool at King Abdulaziz University Medical School. Medical Teacher. 2018;40:1-10.
83. Goldie J, Dowie A, Cotton P, Morrison J. Teaching professionalism in the early years of a medical curriculum: A qualitative study. Medical education. 2007;41:610-7.
84. O'Sullivan A, Harris P, Hughes C, Toohey S, Balasooriya C, Velan G, et al. Linking assessment to undergraduate student capabilities through portfolio examination. Assessment & Evaluation in Higher Education. 2012;37:379-91.
85. Franco R, Franco C, Pestana O, Severo M, Ferreira M. The use of portfolios to foster professionalism: attributes, outcomes, and recommendations. Assessment & Evaluation in Higher Education. 2016;42:1-19.
86. Gordon J. Assessing students' personal and professional development using portfolios and interviews. Medical education. 2003;37:335-40.
87. Wong A, Trollope-Kumar K. Reflections: An inquiry into medical students' professional identity formation. Medical education. 2014;48:489-501.
88. Borgstrom E, Cohn S, Barclay S. Medical Professionalism: Conflicting Values for Tomorrow's Doctors. Journal of general internal medicine. 2010;25:1330-6.
89. Ruiz J, Qadri S, Karides M, Castillo C, Milanez M, Roos B. Fellows' Perceptions of a Mandatory Reflective Electronic Portfolio in a Geriatric Medicine Fellowship Program. Educational Gerontology - EDUC GERONTOL. 2009;35:634-52.
90. Wakeling J, Holmes S, Boyd A, Tredinnick-Rowe J, Cameron N, Marshall M, et al. Reflective Practice for Patient Benefit: An Analysis of Doctors' Appraisal Portfolios in Scotland. Journal of Continuing Education in the Health Professions. 2019;39:1.
91. Lockyer J, Sockalingam S, Campbell DC. Assessment and Change: An Exploration of Documented Assessment Activities and Outcomes by Canadian Psychiatrists. Journal of Continuing Education in the Health Professions. 2018;38:1.
92. Jenkins L, Mash B, Derese A. Development of a portfolio of learning for postgraduate family medicine training in South Africa: A Delphi study. BMC family practice. 2012;13:11.

For peer review only - http://bmjopen.bmj.com/site/about/guidelines.xhtml

93. O'Brien C, Thomas J, Green M. What Is the Relationship Between a Preclerkship Portfolio Review and Later Performance in Clerkships? Academic medicine : journal of the Association of American Medical Colleges. 2017;93.
94. Jenkins L, Mash B, Derese A. The national portfolio of learning for postgraduate family medicine training in South Africa: Experiences of registrars and supervisors in clinical practice. BMC medical education. 2013;13:149.
95. Daboval T, Moore G, Ferretti E. How we teach ethics and communication during a Canadian neonatal perinatal medicine residency: An interactive experience. Medical teacher. 2012;35.
96. Colbert C, Ownby A, Butler P. A Review of Portfolio Use in Residency Programs and Considerations before Implementation. Teaching and learning in medicine. 2008;20:340-5.
97. Torbeck L. A Method for Defining Competency-Based Promotion Criteria for Family Medicine Residents. Academic medicine : journal of the Association of American Medical Colleges. 2005;80:832-9.
98. Goliath C. Diffusion of an E-Portfolio to Assist in the Self-Directed Learning of Physicians: An Exploratory Study. 2009.
99. Singh R, Naughton B, Taylor J, Koenigsberg M, Anderson D, McCausland L, et al. A comprehensive collaborative patient safety residency curriculum to address the ACGME core competencies. Medical education. 2006;39:1195-204.
100. Pinsky L, Edwards K. Diving for PERLS: Working and performance portfolios for evaluation and reflection on learning. Journal of general internal medicine. 2004;19:582-7.
101. Watling C, Brown J. Education Research: Communication skills for neurology residents: Structured teaching and reflective practice. Neurology. 2007;69:E20-6.
102. Clay A, Petrusa E, Harker M, Andolsek K. Development of a web-based, specialty specific portfolio. Medical teacher. 2007;29:311-6.
103. Cheung R. ECLIPPx: An innovative model for reflective portfolios in life-long learning. The clinical teacher. 2011;8:27-30.
104. Bhattarai M. Study skills course in medical education for postgraduate residents. Kathmandu University medical journal (KUMJ). 2006;5:561-5.
105. Dawe R, Pike A, Kidd M, Janakiram P, Nicolle E, Allison J. Enhanced skills in global health and health equity: Guidelines for curriculum development. Canadian Medical Education Journal. 2017;8:e48-e60.
106. Vance G, Williamson A, Frearson R, O'Connor N, Davison J, Steele C, et al. Evaluation of an established learning portfolio. The clinical teacher. 2013;10:21-6.
107. Suraseranivongse S, Chinachoti T, Aroonpruksakul N, Halilamien P, Rushatamukayanunt P, Raksamani K, et al. Development of a learning portfolio to assess the competency of anesthesia residents in Thailand. Asian Biomedicine. 2011;5.
108. Gavriel J. Portfolios and 'the competent clinician': Past, present and future assessment. Education for Primary Care. 2016;27:1-4.
109. Rankings TU. QS World University Rankings 2020 [Available from: https://www.topuniversities.com/university-rankings/university-subject-rankings/2020/medicine.
110. Tan R, Qi Ting JJ, Zhihao Hong D, Sing Lim AJ, Ong YT, Pisupati A, et al. Medical Student Portfolios: A Systematic Scoping Review. Journal of Medical Education and Curricular Development. 2022;9:238212052210760.
111. Norcini J. Is it time for a new model of education in the health professions? Medical Education. 2020;54(8):687-90.

For peer review only - http://bmjopen.bmj.com/site/about/guidelines.xhtml

112. Honea H, Castro IA, Peter P. Evidence Items as Signals of Marketing Competencies and Workplace Readiness: A Practitioner Perspective. JME Outstanding Article of the Year Winners, 2012-. 2017;39 (3):145-61.
113. Association NE. Micro-credential guidance. 2018 [Available from: http://www.nea.org/home/microcredentials.html. .
114. Amsellem-Ouazana D, Pee D, Godin V. Use of portfolios as a learning and assessment tool in a surgical practical session of urology during undergraduate medical training. Medical teacher. 2006;28:356-9.
115. Rees C, Sheard C. The reliability of assessment criteria for undergraduate medical students' communication skills portfolios: The Nottingham experience. Medical education. 2004;38:138-44.
116. Dolan B, O'Brien C, Cameron K, Green M. A Qualitative Analysis of Narrative Preclerkship Assessment Data to Evaluate Teamwork Skills. Teaching and Learning in Medicine. 2018;30:1-9.
117. Sagasser M, Kramer A, Van der Vleuten C. How do postgraduate GP trainees regulate their learning and what helps and hinders them? A qualitative study. BMC medical education. 2012;12:67.
118. Menon V. Portfolios for post graduate psychiatry residents: Potentials and pitfalls. Asian Journal of Psychiatry. 2014;11.
119. Burch V. Portfolios for assessment and learning: Guide Supplement 45.1 – Viewpoint. Medical teacher. 2011;33:1029-31.
120. Ozcakar N, Mevsim V, Dilek G. Use of Portfolios in Undergraduate Medical Training: First Meeting With a Patient. Trakya Universitesi Tip Fakultesi Dergisi. 2009;26.
121. Rees C, Shepherd M, Chamberlain S. The utility of reflective portfolios as a method of assessing first year medical students' personal and professional development. Reflective Practice. 2005;6:3-14.
122. Driessen E, Tartwijk J, Overeem K, Vermunt J, Van der Vleuten C. Conditions for reflective use of portfolios in undergraduate medical education. Medical education. 2006;39:1230-5.
123. Prayson R, Bierer SB, Dannefer E. Medical student resilience strategies: A content analysis of medical students' portfolios. Perspectives on medical education. 2016;6.
124. Rees C, Sheard C. Undergraduate medical students' views about a reflective portfolio assessment of their communication skills. Medical education. 2004;38:125-8.
125. Austin C, Braidman I. Support for portfolio in the initial years of the undergraduate medical school curriculum: What do the tutors think? Medical teacher. 2008;30:265-71.
126. Arntfield S, Parlett B, Meston C, Apramian T, Lingard L. A model of engagement in reflective writing-based portfolios: Interactions between points of vulnerability and acts of adaptability. Medical teacher. 2015;38:1-10.
127. Pitkälä K, Mäntyranta T. Feelings related to first patient experiences in medical school: A qualitative study on students' personal portfolios. Patient education and counseling. 2004;54:171-7.
128. Gibson C, Chandratilake M, Hull A. Use of portfolios for assessment of global health residents: qualitative evaluation of design and implementation. Canadian medical education journal. 2018;9:e20-e32.
129. Tigelaar D, Dolmans D, Grave W, Wolfhagen I, Van der Vleuten C. Portfolio as a tool to stimulate teachers' reflections. Medical teacher. 2006;28:277-82.

For peer review only - http://bmjopen.bmj.com/site/about/guidelines.xhtml

130. Murphy D, Aitchison P, Hernandez Santiago V, Davey P, Mires G, Nathwani D. Insightful Practice: A robust measure of medical students' professional response to feedback on their performance. BMC Medical Education. 2015;15.
131. Chaffey L, de Leeuw E, Finnigan G. Facilitating Students' Reflective Practice in a Medical Course: Literature Review. Education for health (Abingdon, England). 2011;25:198-203.
132. Roberts C, Shadbolt N, Clark T, Simpson P. The reliability and validity of a portfolio designed as a programmatic assessment of performance in an integrated clinical placement. BMC Medical Education. 2014;14:197.
133. Fins J, Kodish E, Cohn F, Danis M, Derse A, Dubler N, et al. A Pilot Evaluation of Portfolios for Quality Attestation of Clinical Ethics Consultants. The American journal of bioethics : AJOB. 2016;16:15-24.
134. Naidu T, Ramlall S. Establishing psychiatric registrars' competence in psychotherapy: A portfolio based model. African journal of psychiatry. 2008;11:264-71.
135. Dannefer E, Bierer SB, Gladding S. Evidence within a portfolio-based assessment program: What do medical students select to document their performance? Medical teacher. 2012;34:215-20.
136. Susan E, Kinnair D. Integrating the assessment of interprofessional education into the health care curriculum. Journal of Taibah University Medical Sciences. 2016;11.
137. Bierer SB, Dannefer E. Does Students' Gender, Citizenship, or Verbal Ability Affect Fairness of Portfolio-Based Promotion Decisions? Results From One Medical School. Academic medicine : journal of the Association of American Medical Colleges. 2011;86:773-7.
138. Bierer SB, Dannefer E, Tetzlaff J. Time to Loosen the Apron Strings: Cohort-based Evaluation of a Learner-driven Remediation Model at One Medical School. Journal of General Internal Medicine. 2015.
139. Dannefer E. Beyond assessment of learning toward assessment for learning: Educating tomorrow's physicians. Medical teacher. 2013;35.
140. Dannefer E, Prayson R. Supporting students in self-regulation: Use of formative feedback and portfolios in a problem-based learning setting. Medical teacher. 2013;35.
141. Douglas AH, Acharya SP, Allery LA. Communication skills teaching and learning in Nepal; what are medical students' perceptions and experiences? A qualitative study. BMC Med Educ. 2020;20(1):391.
142. Remein CD, Childs E, Pasco JC, Trinquart L, Flynn DB, Wingerter SL, et al. Content and outcomes of narrative medicine programmes: a systematic review of the literature through 2019. BMJ Open. 2020;10(1):e031568.
143. Ament Giuliani Franco C, Franco RS, Cecilio-Fernandes D, Severo M, Ferreira MA, de Carvalho-Filho MA. Added value of assessing medical students' reflective writings in communication skills training: a longitudinal study in four academic centres. BMJ Open. 2020;10(11):e038898.
144. Cave J, Dacre J, Jones A. Educating Undergraduate Medical Students About Oncology: A Literature Review. Journal of clinical oncology : official journal of the American Society of Clinical Oncology. 2006;24:1932-9.
145. Brits H, Bezuidenhout J, Van der Merwe LJ, Joubert G. Assessment practices in undergraduate clinical medicine training: What do we do and how we can improve? Afr J Prim Health Care Fam Med. 2020;12(1):e1-e7.

For peer review only - http://bmjopen.bmj.com/site/about/guidelines.xhtml

146. Rajeh N, Grant J, Farsi J, Tekian A. Contextual Analysis of Stakeholder Opinion on Management and Leadership Competencies for Undergraduate Medical Education: Informing Course Design. J Med Educ Curric Dev. 2020;7:2382120520948866.
147. Peeters MJ, Sexton ME. Use of reflective writing within interprofessional education: a mixed-methods analysis. J Interprof Care. 2020;34(3):307-14.
148. Younas A, Ansar A. DEVELOPING A CURRICULUM FOR PROFESSIONAL ETHICS IN UNDERGRADUATE MEDICAL AND DENTAL EDUCATION: EVIDENCE FROM LITERATURE. 2020;70:1211-3.
149. García-Estañ J, Santonja F, Sanz M, Martínez F, Rueda D. Portfolio as a tool to evaluate clinical competences of traumatology in medical students. Advances in Medical Education and Practice. 2016;7:57.

Appendices

Appendix A. Tabulated Summaries

Figure Legend

Figure 2. The SEBA Process

Figure 2. PRISMA Flowchart

For peer review only

Figure 1. *The SEBA Process*

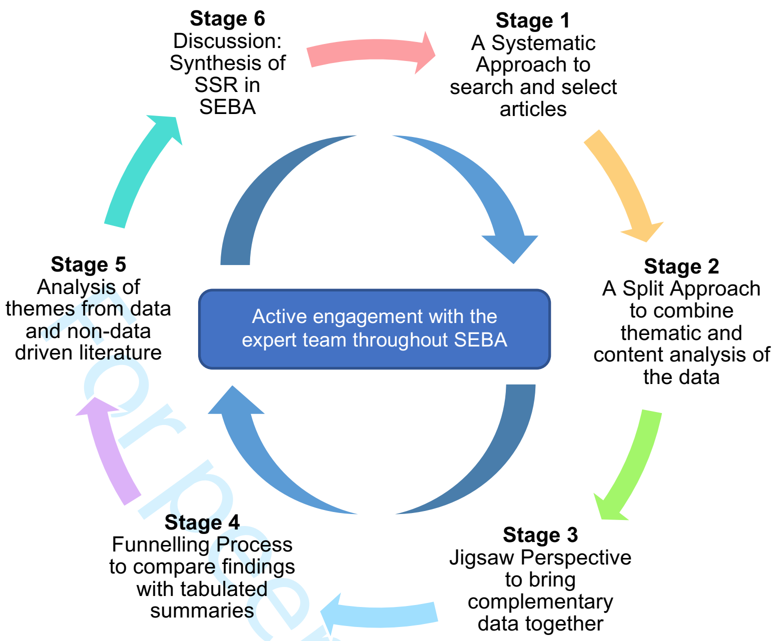

For peer review only

Figure 2. *PRISMA Flowchart*

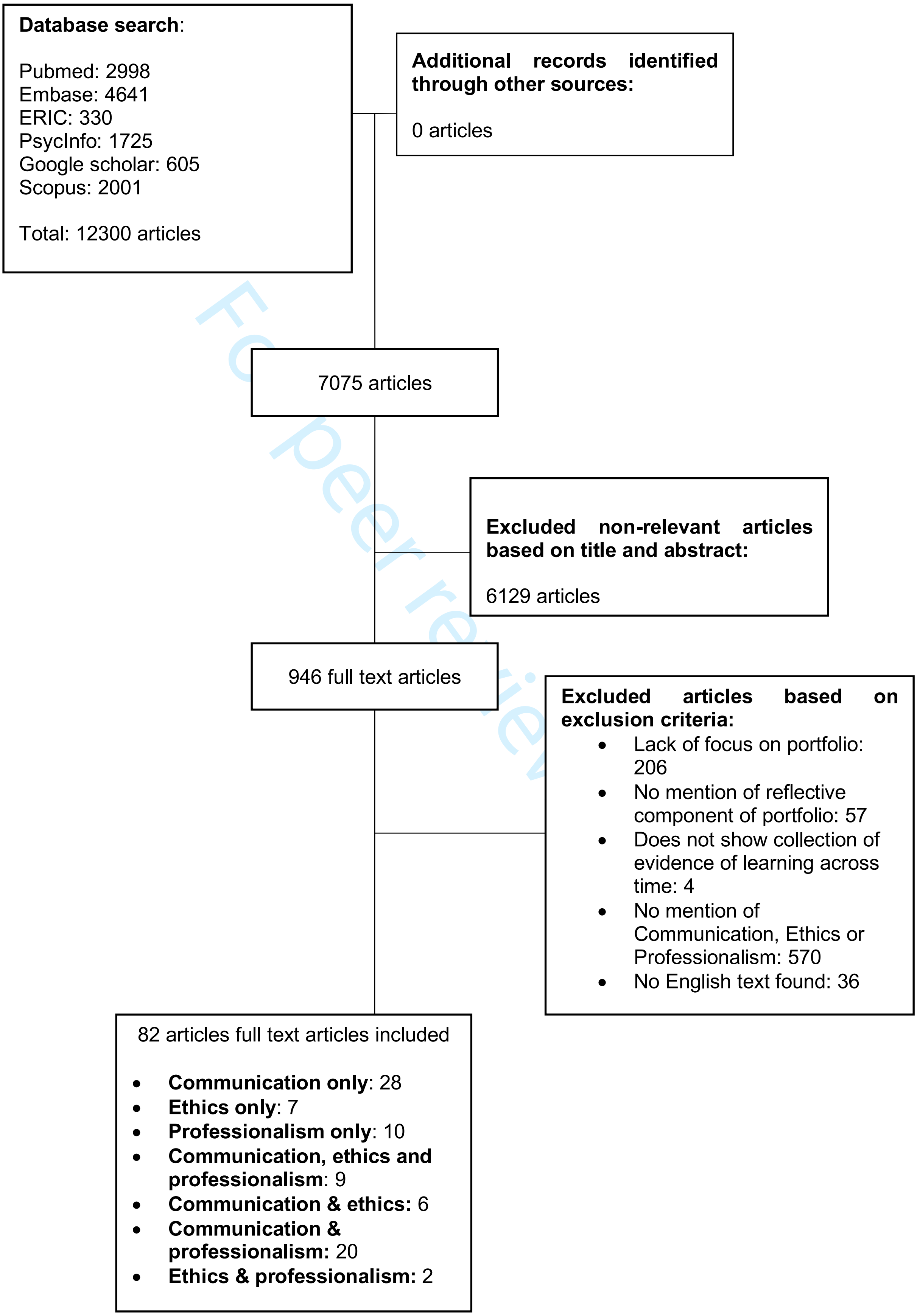

For peer review only

Appendices

Appendix A. Tabulated Summaries.

No.	Title	Author/year	MERSQI	COREQ	Reason for inclusion			Undergraduate (UG)/Postgraduate (PG)	Methodology	Purpose of study	Key findings
					Communication	Ethics	Professionalism				
1	The CanMEDS resume: a useful educational portfolio tool for diagnostic radiology residents	Finlay, K.<br>Probyn, L.<br>Ho, S.<br><br>2012			✓		✓	PG	We developed a comprehensive resident CanMEDS resume for ongoing documentation of resident participation, education, and developing competencies in CanMEDS Roles for diagnostic radiology.<br>This resume or portfolio summary is structured to high- light all 7 CanMEDS Roles. The resident is responsible for ongoing updates and submits this as part of his or her annual evaluation.	The objective of this article is to discuss the utility of this tool for inclusion in the educational portfolio for radiology residents.	The document increases awareness of the CanMEDS Roles and documents resident professional development, while also promoting reflection on the impor- tance of these Roles for radiology and future practice. The added advantage for program directors and assistants is up- to-date documentation of resident activities, which serves as a highly useful tool, particularly for program accreditation purposes.
2	A pilot evaluation of portfolios for quality attestation of clinical ethics consultants	Fins, Joseph J<br>Kodish, Eric<br>Cohn, Felicia<br>Danis, Marion<br>Derse, Arthur R<br>Dubler, Nancy<br>Neveloff<br>Goulden, Barbara<br>Kuczewski, Mark<br>Mercer, Mary Beth<br>Pearlman, Robert A<br><br>2016	7.5	14		✓		PG	The QAPTF committee decided on the key components of the portfolio, their respective weightages and scoring quality of each element.<br>ASBH issued an open call to individuals practicing CEC, publicized on the ASBH website, for let- ters of intent (LOI) to submit portfolios for the piloting of this first step in a quality attestation process. The QAPTF wanted to ensure a	We describe the results of a pilot project utilizing portfolios as an evaluation tool. The goals of this quality attestation pilot project were to assess the feasibility of a process to evaluate whether CE consultants are competent to perform ethics consultations as a lead or solo consultant and to develop quality metrics for this assessment process.	We found that this approach is feasible and resulted in a reasonably wide distribution of scores among the 23 submitted portfolios that we evaluated. We discuss limitations and implications of these results, and suggest that this is a significant step on the pathway to an eventual certification process for clinical ethics consultants.

For peer review only

No.	Title	Author/year	MERSQI	COREQ	Reason for inclusion			Undergraduate (UG)/Postgraduate (PG)	Methodology	Purpose of study	Key findings
					Communication	Ethics	Professionalism				
									representative distribution of candidates for the first portfolio review and adopted a method for select- ing portfolios that was intentionally designed to minimize bias.Candidates needed to have performed CEC, have at least a master's degree in a relevant discipline, and have sufficient CEC experience to be able to provide all ele- ments of the portfolio. 32 out of 82 individuals were invited to submit portfo- lios. After candidates submitted their portfolios, they were surveyed to obtain feedback on their portfolio develop- ment and submission experience. QAPTF members were also asked about their experi- ence and perceptions of whether the portfolio elements and approach to scoring functioned effectively in identify- ing candidates who should or should not proceed with the next step of		

For peer review only

No.	Title	Author/year	MERSQI	COREQ	Reason for inclusion			Undergraduate (UG)/Postgraduate (PG)	Methodology	Purpose of study	Key findings
					Communication	Ethics	Professionalism				
									quality attestation. They also were asked about ways to improve future attestation activities. This feedback is reflected in the limitations and discussion sections that follow.		
3	Portfolios and 'the competent clinician': past, present and future assessment	Gavriel, J.<br><br>2016	-	-			✓	PG	NA	To describe the evolution of portfolios, including its advantages, challenges and the future of portfolios	Portfolios have been a part of medical education since the early 1990s and many educators use them frequently, even habitually. Portfolios are primarily based on a constructivist approach to learning and there are also strong links with adult learning principles.Competence based education, once under fire for being too simplistic, now forms a significant part of medical education including the development of portfolios.<br><br>In the med- ical education arena there are some advantages that are particularly relevant: triangulation amongst those working alongside learners, developing wider professional skills and demonstrating the potential for lifelong learning and excellence.<br><br>For some learners and educators the portfolio is a burden and a frustration: the volume of work, a hoop jumping approach and a subjective nature all providing challenges [8] that are at least partially a result of some key complex- ities of the portfolio assessment.

For peer review only

No.	Title	Author/year	MERSQI	COREQ	Reason for inclusion			Undergraduate (UG)/Postgraduate (PG)	Methodology	Purpose of study	Key findings
					Communication	Ethics	Professionalism				
											Portfolios are evolving assessments; they are subject to continuing developments but the ongoing challenge for portfolio designers is the balance between reliability and validity on one hand and tick-boxing on the other.
4	Diffusion of an e-portfolio to assist in the self-directed learning of physicians: An exploratory study	Goliath, Cheryl Lynn 2010	13	21	✓			PG	A single case study comprised of nine physicians was used to examine the factors involved when the diffusion of an e-portfolio was introduced. The participants were studied using interviews, the Stages of Concern (SoCQ) and the Self-Directed Learning Readiness Scale (SDLRS) questionnaires, four-week trial period of I-Webfolio and field notes based upon observations during the study.	This study examined the impact of implementing an e-portfolio system within an Internal Medicine physician practice as a means of developing a process of life-long learning and professional development for the participants.	The results were analyzed to identify patterns or themes that appeared. One such pattern emerged, in that; all entries in the e-portfolio were almost exclusively in the Medical Knowledge component. A theme that became apparent was that none of the physicians in the study have developed a self-directed learning plan. The results of this study suggested that adoption of technology is multifactoral and there was no consistent individual profile that predicted optimal adoption of the innovation; however, increased scores on the SDLRS would indicate that the diffusion of I-Webfolio was effective in improving the self-directed learning of the participants.
5	The attributes of a good medical trainee: how to build your portfolio	Joseph, S. R. Sikkel, M. B. 2009	-	-	✓			PG	-	Review of the attributes of a good medical trainee based on the essential physician competencies, and how then to build a good portfolio	A comprehensive and contemporaneously completed procedural, operative, teaching and reflective practice logs should be included in the portfolio, and should be commenced as soon as possible. Compose your portfolio around the seven key roles of the Canadian Medical Education Directives for Specialists model, and be innovative when

For peer review only

No.	Title	Author/year	MERSQI	COREQ	Reason for inclusion			Undergraduate (UG)/Postgraduate (PG)	Methodology	Purpose of study	Key findings
					Communication	Ethics	Professionalism				
											filling each section with concrete examples. Use reflective practice of clinical cases also as further evidence.
6	Study skills course in medical education for postgraduate residents	Bhattarai, M. D. 2007	-	-	✓			PG	-	The learners have to take active parts in the teaching learning activities. To make them aware and to help them develop the skills required, the need of the study skills course in medical education early in the part of their training has been realized for the postgraduate residents	The important areas of the study skills course focusing in the requirement of clinical components of the postgraduate residents are 1.) Interpersonal and communications skills, 2.) Teaching, learning and presentation skills, 3.) Language, reading and computer use, 4.) Evidence based medicine and diagnosis and management, 5). Assessment principles and strategies, 6). Time management strategies to get the best out of the training, 7). Reflection, portfolio and self-directed lifelong learning, and 8). Follow-up presentation. The methodologies that could be used in such study skills course are interactive lectures, brainstorming, presentations by the trainees, demonstration to and by the trainees, small group discussion, group work and presentation, group and individual feedback, practice sessions, role play, short relevant video movies, video recording of the trainees and viewing with feedback. With their already tight training schedule and posting and other similar other mandatory courses required for the postgraduate residents, much time cannot be allocated for the study skills course in medical education alone. Similar study skills course in medical education

For peer review only

No.	Title	Author/year	MERSQI	COREQ	Reason for inclusion			Undergraduate (UG)/Postgraduate (PG)	Methodology	Purpose of study	Key findings
					Communication	Ethics	Professionalism				
											may need to be arranged for the undergraduate medical students as well.
7	Fellows' perceptions of a mandatory reflective electronic portfolio in a geriatric medicine fellowship program	Ruiz, Jorge G. Qadri, Syeda S. Karides, Marina Castillo, Carmen Milanez, Marcos Roos, Bernard A. 2009	9	15	✓		✓	PG	We conducted surveys and focus groups followed by quantitative and qualitative data analysis	Electronic portfolios (ePortfolios) can be useful for evaluating and documenting mastery of competencies. We investigated geriatric medicine fellows' perceptions of an ePortfolio	Our study revealed that fellows considered the ePortfolio acceptable and accessible. Fellows reflected on aspects relevant to postgraduate training and future practice and documented adherence to competencies. However, fellows expressed resentment about the self-reflection component, which interfered with the overall acceptance of the ePortfolio. Although ePortfolios can be powerful and reflective tools for competency assessment, care must be taken in their implementation.
8	Portfolios for assessment and learning: Guide Supplement 45.1 - Viewpoint	Burch, Vanessa 2011	-	-		✓		PG	NA	NA	The critical question to ask then is Should poorly resourced countries not find ways of adapting this valuable learning and assessment strategy to suit local realities? (Spandel 1997). Clearly, the 'Rolls Royce' version of portfolio implementation and assessment is beyond the reach of developing countries but a less resource-intensive strategy could be a feasible option. It was with this goal in mind that the University of Cape Town (UCT) in South Africa embarked on the task of adapting portfolios for use in less well-resourced settings (Burch & Seggie 2008). Medical students in a 6-year programme are introduced to portfolio-based learning and assessment in their third year of training and each subsequent year until graduation. Currently, clinical students write up,

For peer review only

No.	Title	Author/year	MERSQI	COREQ	Reason for inclusion			Undergraduate (UG)/Postgraduate (PG)	Methodology	Purpose of study	Key findings
					Communication	Ethics	Professionalism				
											and manage under supervision, between 18 and 32 cases in 6- to 10-week medicine clerkships These patients are presented to a specialist in training on admission and the attending consultant on the post-intake ward the following morning. This provides ample opportunity for students to look back on the action, reflect on their performance and receive feedback and mentoring from senior clinicians directly in the workplace setting. In this way, consultants and specialists in training act as mentors helping students identify alternative strategies and set goals to stimulate further learning. These distinct phases of the workplace-based mentoring process closely reflect the phases of spiral professional development described by Korthagen et al. (2001). The importance of this approach is that mentors provide ongoing guidance for students during the course of performing their routine clinical duties limiting the need for additional regular meetings between students and mentors. Students who are struggling are free to consult the course convener for assistance, but an introductory lecture clearly outlining the purpose, format and examination of portfolios, as well as a set of guidelines for completing portfolio tasks has significantly limited the need for further advice. In addition to writing up a series of patient case studies, students also

For peer review only

No.	Title	Author/year	MERSQI	COREQ	Reason for inclusion			Undergraduate (UG)/Postgraduate (PG)	Methodology	Purpose of study	Key findings
					Communication	Ethics	Professionalism				
											identify at least one clinical learning need based on each case discussion; these 'question and answer' tasks are also included in the portfolio. This provides a good opportunity for needs-driven, focused, self-directed learning. Students also complete a range of other written tasks using the index clinical cases in their portfolios. These case-based tasks (Harden et al. 2000) include written reflections on ethical issues relevant to selected patients, a bio-psycho-social analysis (mini-ethnography) (Kleinman & Benson 2006) of selected patients and the use of case material to explore the principles of Primary Health Care (Vivian et al. 2010) as they relate to patients in the portfolio. Finally, students also write up detailed treatment plans for a list of 32 common conditions as reflected by the cases in the portfolio. These tasks are all designed to promote self-directed, patient-centred learning.<br>At UCT it was recognised that marking of this volume of portfolio material was beyond the human resources capacity at departmental level and so a strategic decision was made to focus on an assessment strategy based on a series of structured interviews, focusing on different aspects of a sample of portfolio cases. Students rotate through four stations of 15 min each (OSCE style) at which single-examiner structured interviews are conducted based

For peer review only

No.	Title	Author/year	MERSQI	COREQ	Reason for inclusion			Undergraduate (UG)/Postgraduate (PG)	Methodology	Purpose of study	Key findings
					Communication	Ethics	Professionalism				
											on patient cases randomly selected from the portfolio. The case-based discussions focus on clinical reasoning, case management plans, bioethics, psychosocial aspects of illness and the principles of Primary Health Care. This approach assesses student performance on a ‘biopsy’ of at least 8–10 cases contained in the portfolio. Each case discussion is scored using a nine-point rubric (1–3 poor, 4–6 adequate, 7–9 good) and scores are totalled across all cases discussed to achieve an aggregate score for the examination.<br>The key reasons for adopting this assessment strategy at UCT included limited human resources but, more importantly, the observation that English second language speakers, more than 50% of university admissions nationally (Council on Higher Education 2009), are better able to express themselves in oral rather than written form. Indeed, it has been asked whether portfolios provide educators with real insight into practitioners’ clinical ability or simply show that they are good at writing about what they do? (Karlowicz 2000).<br>Having developed the strategy, the key question is whether any other university in sub-Saharan Africa has successfully adopted it. To date, two other South African universities have adopted and adapted the approach to suit local needs and two other African universities have

For peer review only

No.	Title	Author/year	MERSQI	COREQ	Reason for inclusion			Undergraduate (UG)/Postgraduate (PG)	Methodology	Purpose of study	Key findings
					Communication	Ethics	Professionalism				
											successfully adopted the strategy at Makerere University (Mubuuke et al. 2010) and Kampala International University (Ezeala et al. 2010) both in Uganda. Clearly, the data are limited but it does suggest that portfolio-based learning and assessment are within reach of countries attempting to improve the quality of health sciences education using available resources. This, I believe, is one of the most important issues to be addressed in the twenty-first century – the adaptation and implementation of better teaching and assessment strategies in developing countries so as to keep abreast of educational advances developed world countries enjoy.
9	AMEE Guide No. 25: The assessment of learning outcomes for the competent and reflective physician	Shumway, J. M. Harden, R. M. 2003	-	-	✓	✓	✓	PG	-	To emphasize on the 2 important features of contemporary medical education: first is an emphasis on assessment as a tool to ensure quality in training programmes, to motivate students and to direct what they learn. The second is a move to outcome-based education where the learning outcomes are defined and decisions about the curriculum are based on these.	Portfolio assessments have a positive impact on the learning environment because they document what the learner has done and ask the learner to reflect on what she/he has accomplished. They ask the learner to put together large amounts of disparate information into a whole that tells a story about the learner's accomplishments. Portfolios are a valuable instru- ment for inclusion in the examiner's toolkit, if for no other reason than that they assess learning outcomes such as critical thinking and self-assessment, which are not easily assessed using other instruments. They also provide a record of the

For peer review only

No.	Title	Author/year	MERSQI	COREQ	Reason for inclusion			Undergraduate (UG)/Postgraduate (PG)	Methodology	Purpose of study	Key findings
					Communication	Ethics	Professionalism				
											student's performance over a period of time and are not just a snapshot at one specific point in time. The reliability of portfolios is at least in part due to the ability of raters to agree on standards and criteria for the content and assessment of portfolios.
10	A comprehensive collaborative patient safety residency curriculum to address the ACGME core competencies	Singh, R.<br>Naughton, B.<br>Taylor, J. S.<br>Koenigsberg, M. R.<br>Anderson, D. R.<br>McCausland, L. L.<br>Wahler, R. G.<br>Robinson, A.<br>Singh, G.<br><br>2005	-	-	√			PG	-	To design and implement a new patient safety curriculum in collaboration with the Schools of Nursing and Pharmacy, in such a way as to address all 6 competencies.	Porfolios is used as one of the multiple assessment approaches in the patient safety programme. However, no evaluation made on this assessment mode.
11	ECLIPPx: an innovative model for reflective portfolios in life-long learning	Cheung, C. R.<br><br>2011	-	-			√	PG	-	For healthcare professionals, the educational portfolio is the most widely used component of lifelong learning – a vital aspect of modern medical practice. When used effectively, portfolios provide evidence of continuous learning and promote reflective practice. But traditional portfolio models are in danger of becoming outmoded, in the face of changing expectations of health- care provider competences today.<br><br>Portfolios in health care have generally focused on competencies in clinical skills. How- ever, many other domains of professional	A new model for categorising the health care port- folios of professionals is proposed. The ECLIPPx model is based on personal practice, and divides the evidence of ongoing professional learning into four categories: educational development; clinical practice; leadership, innovation and professionalism; and personal experience.<br><br>The ECLIPPx model offers a new approach for personal reflection and longitudinal learn- ing, one that gives flexibility to the user whilst simultaneously encompassing the many relatively new areas of competence and expertise that are now required of a modern doctor.

For peer review only

No.	Title	Author/year	MERSQI	COREQ	Reason for inclusion			Undergraduate (UG)/Postgraduate (PG)	Methodology	Purpose of study	Key findings
					Communication	Ethics	Professionalism				
										development, such as professionalism and leadership skills, are increasingly important for doctors and health care professionals, and must be addressed in amassing evidence for training and revalidation. There is a need for modern health care learning portfolios to reflect this sea change.	
12	Development of a web-based, specialty specific portfolio	Clay, A. S., Petrusa, E., Harker, M. and Andolsek, K.<br><br>2007	-	-	✓	✓	✓	PG	Three different disciplines (anesthesiology, surgery and medicine) worked together to create a critical care medicine portfolio. We began by reviewing the curriculum requirements for critical care medicine and organise<br><br>d these requirements into the six ACGME core competencies. We then developed learner led exercises in each core competency that were specific to critical care. Each exercise includes assessment of resident knowledge and application, an evaluation of the exercise, a learner self-	This article illustrates the creation of a specialty specific portfolio that can be used by several different residency programs to document resident competence during a given rotation	Creation of specialty specific portfolio reduces redundancy between disciplines, allows for increased time to be spent on the development of exercises specific to rotation objectives, and aids program directors in the collection of portfolio entries for each resident over the course of a residency.<br><br>We describe the creation of a multidisciplinary learning portfolio that can be used by different training programs within one institution to assess trainee performance in each respective area of expertise. The strengths of the specialty specific portfolio include the ability to be specific to the learning objectives of that rotation/specialty and uniform in content across multiple disciplines within that specialty. Commitment to the portfolio from multiple disciplines allows for careful refining of the portfolio and reduction of duplicative efforts. These specialty specific portfolios could potentially be improved upon if professional

For peer review only

No.	Title	Author/year	MERSQI	COREQ	Reason for inclusion			Undergraduate (UG)/Postgraduate (PG)	Methodology	Purpose of study	Key findings
					Communication	Ethics	Professionalism				
									assessment of skill, and a review of performance by a faculty member. Portfolio entries are highlighted in a multi-disciplinary weekly conference and posted on a critical care web site at our University.		organizations were committed to their adaptation, perhaps as a component of maintenance of certification. The portfolio entries as we have described are also useful for prompting frequent self reflection/evaluation and for providing trainees with feedback on their self-perceptions. Self assessment, a skill which is not often taught or evaluated in medical education, is notoriously inaccurate (Gordon 1991). However, self assessment may be improved with deliberate practice, and frequent self assessment with feedback on that self assessment (Gordon 1992; Norman & Schmidt 1992; Ericsson 2004). If other specialties were to create similar portfolios, the logistics of creating a portfolio for each resident would be simplified: the portfolio could be built a rotation at a time, meeting the objectives of that rotation monthly, and meeting the ACGME requirements over the course of a residency. Furthermore, by simply requiring a certain number entries from each core competency by the end of each year or the end of a residency, the learner is given the discretion to choose those exercise which he/she feels is most relevant given the cases he/she has encountered.
13	A review of portfolio use in residency programs and	Colbert, C. Y., Ownby, A. R. and Butler, P. M.	-	-			✓	PG	Two searches of PubMed, OVID, JSTOR, SCOPUS, and	Portfolios, often described as collections of evi-dence, are discussed	There is wide varia- tion in how portfolios are utilized within U.S. residency programs. The challenge for

For peer review only - http://bmjopen.bmj.com/site/about/guidelines.xhtml

No.	Title	Author/year	MERSQI	COREQ	Reason for inclusion			Undergraduate (UG)/Postgraduate (PG)	Methodology	Purpose of study	Key findings
					Communication	Ethics	Professionalism				
	considerations before implementation	2008							FirstSearch Wilson Select were conducted between October 2006 and April 2007 to identify studies and articles related to portfolio usage. Thirty-nine articles met criteria and were reviewed.	as a means of teaching or assessing the Ac- creditation Council for Graduate Medical Education competencies. Yet, it is unclear how many residency programs utilize portfolios. The purpose of this article is to (a) review the literature on portfolio use in graduate medical education; (b) examine efficacy of portfolio use, based upon studies in the field; and (c) offer a discussion of con- siderations for implementing portfolios.	graduate medical education is to create consensus on the definition and purpose of portfolios, such that best practices in portfolio implementation and assessment can be achieved.<br><br>The case for utilizing portfolios in residency education is compelling. Portfolios can offer a diverse palette of learners' achievements and experiences. When assessing competence from a holistic standpoint, the range of behaviors captured in a portfolio may render it superior to more traditional assessment practices. Self-awareness and self-directed learning, thought to be critical to the development of competent physicians, can be enhanced through the act of portfolio development,19 as port- folios can force one to reflect upon strengths, weaknesses, and opportunities for growth.<br><br>Before implementing a portfolio system in a residency pro- gram, several issues need to be considered. Learners and faculty should be trained on the portfolio process including the purpose, structure, and content of portfolios. Mentoring may be required when it comes to content assembly and interpretation. If portfolios are to be used for summative assessment pur- poses, an evaluation rubric—a scoring template—will need to be

For peer review only

No.	Title	Author/year	MERSQI	COREQ	Reason for inclusion			Undergraduate (UG)/Postgraduate (PG)	Methodology	Purpose of study	Key findings
					Communication	Ethics	Professionalism				
											created.<br><br>Last, the promise offered by portfolios is tempered by diffi-culties associated with their use. The lack of a standard defini- tion across the field and the dearth of outcome studies to assess efficacy are pressing concerns. The challenge now is to create consensus on the definition and purpose of portfolios within res- idency programs, such that collaboration within the field may begin to offer best practices in the areas of implementation and assessment.
14	How we teach ethics and communication during a Canadian neonatal perinatal medicine residency: an interactive experience	Daboval, T., Moore, G. P. and Ferretti, E.<br><br>2013	6.5		✓	✓	✓	PG	A revised ethics program implemented in 2009 identified competencies that should be demonstrated at the end of the Neonatal-Perinatal Medicine (NPM) residency. Several seminars were refined while new workshops, problem-based learning in ethics, and a personal portfolio were added.	This article describes the process by which a well-delineated, interactive program to teach ethical reasoning and skillful communication with parents was implemented at the University of Ottawa, Canada.	All teaching strategies were well received based on the average level of satisfaction (5.8 out of 7, SD 0.4). We are now moving forward by formally assessing our program including the impact on knowledge acquisition and behavior.<br><br>A dedicated, interactive competency-based neonatal ethics teaching program is vital to support NPM trainees in learning how to integrate ethical thinking with competencies in communication.<br><br>A dedicated, interactive, competency-based neonatal ethics teaching program with well-identified expectations and eval-uation is vital so that all graduating NPM trainees will not only have knowledge of key ethical precepts and ethical reasoning, but will also have learned how to integrate ethical thinking when they communicate and interact

For peer review only

No.	Title	Author/year	MERSQI	COREQ	Reason for inclusion			Undergraduate (UG)/Postgraduate (PG)	Methodology	Purpose of study	Key findings
					Communication	Ethics	Professionalism				
											professionally with parents during ethically and emotionally charged clinical situations. In this manner, our teaching is supporting a paradigm shift in neonatology from a paternalistic to a shared decision-making model. To advance our methods of education in this area, formal assessments of such teaching programs must occur.
15	Portfolio as a tool to stimulate teachers' reflections	Tigelaar, D. E.<br>Dolmans, D. H.<br>de Grave, W. S.<br>Wolfhagen, I. H.<br>van der Vleuten, C. P.<br><br>2006	-	15	✓			PG	In this study, all the written portfolio assignments were approached as reflections on teacher functioning. In order to gain insight into the portfolio process, we collected the teachers' final portfolio assignments as well as data about the process of portfolio construction. In this way, we obtained portfolio data comprising written portfolio assignments and final reflective portfolio assignments. Firstly, the teachers were asked to describe the course of their teaching career. They were also asked to describe critical incidents in their development as a teacher, set learning goals,	Portfolios are increasingly being used to stimulate teachers' reflections. Frameworks for reflection on teaching often emphasize competencies and behaviours. However, other aspects of teacher functioning are also important, such as the teaching environment and individual teachers' beliefs, professional identity and mission. In a study among five medical school teachers, we explored how a portfolio stimulated reflections on the various aspects of teaching functioning.	The analysis yielded examples of reflections on all aspects of Korthagen's model, although reflections on competencies proved easiest to identify.

For peer review only

No.	Title	Author/year	MERSQI	COREQ	Reason for inclusion			Undergraduate (UG)/Postgraduate (PG)	Methodology	Purpose of study	Key findings
					Communication	Ethics	Professionalism				
									select artefacts in evidence of their teaching activities, compose a profile of a good teacher and reflect on that. The final assign- ment required them to compose a reflective portfolio, using the previous assignments as points of departure. The assignments were intended to stimulate reflection on all aspects of teacher functioning as defined in Korthagen's model. However, the critical incidents assignment was focused on the environment, behaviour, and competencies. Table 1 presents brief descriptions of the assignments.		
16	A method for defining competency-based promotion criteria for family medicine residents	Torbeck, L. Wrightson, A. S. 2005	-	-	✓		✓	PG	In 2003, both of us began the task of developing promotion criteria by searching the literature for studies identifying standards or criteria for resident promotion. Few studies were found related to residents' progress and	The Accreditation Council for Graduate Medical Education (ACGME) has mandated a shift from a structure- and process-based educational system to a competency-based system. The ACGME has not provided criteria (standards), preferring to leave that to the discretion	The value of promotion criteria Training residents to become competent health care providers has been and always will be an important and challenging endeavor. With the ACGME's new mandate, many program directors and house office staff are looking for useful guidance to make their residency programs compliant. We believe that developing a

For peer review only

No.	Title	Author/year	MERSQI	COREQ	Reason for inclusion			Undergraduate (UG)/Postgraduate (PG)	Methodology	Purpose of study	Key findings
					Communication	Ethics	Professionalism				
									promotion.[2,7] We then reviewed the information contained within the last three Residency Assistance Program workshops of the American Academy of Family Physicians, 2001–2003. Frey et al.'s[8] 2003 outpatient core competency presentation notes were earmarked as having specific core competencies that could easily be rewritten as promotion criteria. Next, we reviewed the residency curriculum of the Department of Family Practice and Community Medicine at the University of Kentucky College of Medicine and listed promotion criteria that we thought were important for Family Medicine residents at each level of that curriculum to achieve. Taking this list and extrapolating from Frey et al.'s work, we composed a list of working promotion criteria.	of the individual training programs. Such criteria and an overall strong evaluation process are essential for residents to attain the appropriate knowledge, skills, and attitudes. With this need in mind, the authors describe an evaluation process in which they developed ACGME-competency-based promotion criteria for family medicine residents at the University of Kentucky College of Medicine in 2004.	set number of promotion criteria that are competency-based should be among the first tasks residency programs should tackle, for several reasons.<br><br>First, having defined criteria will make the three to seven years of residency performance assessment more educationally sound. The education literature reminds us that two components are mandatory to a performance assessment: the task and the criteria.[11,12] Since continuous performance assessment is carried out during the majority of a resident's training, the awareness of what is expected of the resident can be raised for both the resident and the faculty by making the task and criteria clearer to them. Residents then are better able to demonstrate that which is expected of them, and faculty are better prepared to evaluate them.<br><br>Second, having defined promotion criteria can better target remediation in those residents failing to meet the criteria in a timely manner. Intervention during a time early on in the resident's development is much more beneficial than it is just before making a decision to promote or not promote.<br><br>Third, having criteria can improve faculty development, especially in terms of how to assess competence, how to give feedback, and how to account for faculty

For peer review only

No.	Title	Author/year	MERSQI	COREQ	Reason for inclusion			Undergraduate (UG)/Postgraduate (PG)	Methodology	Purpose of study	Key findings
					Communication	Ethics	Professionalism				
											responsibility in training and evaluating competent physicians.
17	Enhanced skills in global health and health equity: Guidelines for curriculum development	Dawe, R., Pike, A., Kidd, M., Janakiram, P., Nicolle, E. and Allison, J.<br><br>2017	10	10		✓		PG	A panel comprised of 34 experts in global health education and practice completed three rounds of a Delphi small group process.	To generate consensus on the essential components of a Global Health/Health Equity Enhanced Skills Program in family medicine.	Consensus (defined as ≥ 75% agreement) was achieved on program length (12 months), inclusion of both domestic and international components, importance of mentorship, methods of learner assessment (in-training evaluation report, portfolio), four program objectives (advocacy, sustainability, social justice, and an inclusive view of global health), importance of core content, and six specific core topics (social determinants of health, principles and ethics of health equity/global health, cultural humility and competency, pre and post-departure training, health systems, policy, and advocacy for change, and community engagement).<br><br>Panellists agreed on a number of program components forming the initial foundation for an evidence- informed, competency-based Global Health/Health Equity Enhanced Skills Program in family medicine.
18	Learning portfolios in radiology residency education: how do I get started?	Deitte, L.<br><br>2008	-	-	✓		✓	PG	-	The purpose of this article is three-fold: (1) to famil- iarize the reader with learning portfolios in medical edu-cation, (2) to outline the new radiology ACGME re-quirements for a portfolio, and (53) to discuss strategies for the implementation of a portfolio as a learning and	As medical education and continuous professional devel- opment shift toward an outcomes-based model, there is increasing focus on documentation of physician self-as- sessment and a commitment to lifelong learning. The ACGME program requirements for diagnostic radiology graduate medical education effective July 1, 2008, in- clude learning portfolios as

For peer review only

No.	Title	Author/year	MERSQI	COREQ	Reason for inclusion			Undergraduate (UG)/Postgraduate (PG)	Methodology	Purpose of study	Key findings
					Communication	Ethics	Professionalism				
										assessment tool in a residency education program and continued professional development..	one method for this docu- mentation. Although there are some challenges associ- ated with the implementation of learning portfolios, it is likely that portfolios will prove to be a valuable learning and assessment tool not only in graduate medical educa- tion but also throughout a physician’s career and recerti- fication process.
19	Evaluation of an established learning portfolio	Vance, G. Williamson, A. Frearson, R. O'Connor, N. Davison, J. Steele, C. Burford, B. 2013	9	-	✓			PG	Deanery-wide trainee attitudes were surveyed by an electronic questionnaire in 2009 and compared with perceptions recorded during the pilot phase (2004–2005).	The trainee-held learning portfolio is integral to the foundation programme in the UK. In the Northern Deanery, portfolio assessment is standar- dised through the Annual Review of Competence Progression (ARCP) process. In this study we aimed to establish how current trainees evaluate portfolio-based learning and ARCP, and how these attitudes may have changed since the foundation programme was first introduced.	Many trainees continue to view the e-portfolio negatively. Indeed, significantly fewer train- ees in 2009 thought that the e-portfolio was a ‘good idea’ or a ‘worthwhile investment of time’ than in 2005. Trainees remain unconvinced about the educa- tional value of the e-portfolio: fewer trainees in 2009 regarded it as a tool that might help focus on training or recognise individual strengths and weaknesses. Issues around unnecessary bureaucracy persist. Current trainees tend to understand how to use the e- portfolio, but many did not know how much, or what evidence to collect. Few supervisors were re- ported to provide useful guidance on the portfolio. ARCP encouraged portfolio completion but did not give meaningful feedback to drive future learning.<br><br>Continued support is needed for both trainees and supervisors in portfolio-building skills and in using the e-portfolio as an educational tool. Trainee-tailored feedback is needed to ensure that portfolio-

No.	Title	Author/year	MERSQI	COREQ	Reason for inclusion			Undergraduate (UG)/Postgraduate (PG)	Methodology	Purpose of study	Key findings
					Communication	Ethics	Professionalism				
											based assessment promotes lifelong, self-directed and reflective learners. Trainees remain unconvinced about the educational value of the e-portfolio
20	Reflective Practice for Patient Benefit: An Analysis of Doctors' Appraisal Portfolios in Scotland	Wakeling, J. Holmes, S. Boyd, A. Tredinnick-Rowe, J. Cameron, N. Marshall, M. Bryce, M. Archer, J. 2019	-	16	✓			PG	Using an in-depth case study approach, 18 online portfolios in Scotland were examined with a template developed to record the types of supporting information submitted and how far these showed reflection and/or changes to practice. Data from semistructured interviews with the doctors (n = 17) and their appraisers (n = 9) were used to contextualize and broaden our understanding of the portfolios.	Reflective practice has become the cornerstone of continuing professional development for doctors, with the expectation that it helps to develop and sustain the workforce for patient benefit. Annual appraisal is mandatory for all practicing doctors in the United Kingdom as part of medical revalidation. Doctors submit a portfolio of supporting information forming the basis of their appraisal discussion where reflection on the information is mandated and evaluated by a colleague, acting as an appraiser.	Portfolios generally showed little written reflection, and most doctors were unenthusiastic about documenting reflective practice. Appraisals provided a forum for verbal reflection, which was often detailed in the appraisal summary. Portfolio examples showed that reflecting on continued professional development, audits, significant events, and colleague multisource feedback were sometimes considered to be useful. Reflecting on patient feedback was seen as less valuable because feedback tended to be uncritical. The written reflection element of educational portfolios needs to be carefully considered because it is clear that many doctors do not find it a helpful exercise. Instead, using the portfolio to record topics covered by a reflective discussion with a facilitator would not only prove more amenable to many doctors but would also allay fears of documentary evidence being used in litigation.
21	Developing an Evaluation Tool for Assessing Clinical Ethics Consultation Skills in Simulation Based	Wasson, K. Parsi, K. McCarthy, M. Siddall, V. J. Kuczewski, M. 2016	-	-		✓		PG	At the beginning of the project development, four of the authors (who are bioethicists with experience in	The American Society for Bioethics and Humanities has created a quality attestation (129) process for clinical ethics consultants;	Given the move toward quality attestation by ASBH and the need to develop a way of assessing the interpersonal skills of clinical ethics consultants ''live'' we have developed the ACES

For peer review only

No.	Title	Author/year	MERSQI	COREQ	Reason for inclusion			Undergraduate (UG)/Postgraduate (PG)	Methodology	Purpose of study	Key findings
					Communication	Ethics	Professionalism				
	Education: The ACES Project								clinical ethics consultation) agreed that any tool created should be grounded in the ASBH core competencies, as they are the articulated professional standards in the field. After a review of the limited assessment tools available, we agreed to draw from the VA's Ethics Consultant Proficiency Assessment Tool (2014). It draws on the ASBH core competencies, including the categories of Interpersonal Skills, Process Skills, Analytic Skills, and Core Knowledge, is quite comprehensive, and has been in use for a number of years. In addition, we followed Stufflebeam's (2000) guidelines for creating a checklist evaluation tool.<br><br>Given the focused usage of the ACES tool—clinical ethics simulation in an educational setting—we met regularly to	the pilot phase of reviewing portfolios has begun. One aspect of the QA process which is particularly challenging is assessing the interpersonal skills of individual clinical ethics con- sultants. We propose that using case simulation to evaluate clinical ethics consul- tants is an approach that can meet this need provided clear standards for assessment are identified. To this end, we developed the Assessing Clinical Ethics Skills (ACES) tool, which identifies and specifies specific behaviors that a clinical ethics consultant should demonstrate in an ethics case simulation. The aim is for the clinical ethics consultant or student to use a videotaped case simulation, along with the ACES tool scored by a trained rater, to demonstrate their competence as part of their QA portfolio.	tool. Our tool provides a clear structure for key domains, behaviors, and interpersonal skills that can be observed and scored by a trained rater during simulated ethics consultations. Individuals completing the ACES course or using the tool can opt to include their evaluation and video as part of their QA portfolio as a means of demonstrating their interpersonal skills in a simulated ethics consultation. Our tool also provides a framework and structure for the content of an ethics consultation and helps translate the core competencies into practical areas, behaviors, and skills. We spent a great deal of time thinking about and debating which items from the VA tool to include in the final ACES tool. It is not intended to be comprehensive for all aspects of clinical ethics consultation, e.g., for simplicity in the pilot stage we did not examine the clinical ethics consultant's ability to review and decipher the medical chart or write a note, though these elements could be added to the simulations in future. Instead, it focuses on those elements we thought could be captured in simulated clinical ethics consultations in an educational setting in this pilot phase. In the future, other elements of ethics consultation and levels of complexity can be added to the simulations as well as additional cases.

For peer review only

No.	Title	Author/year	MERSQI	COREQ	Reason for inclusion			Undergraduate (UG)/Postgraduate (PG)	Methodology	Purpose of study	Key findings
					Communication	Ethics	Professionalism				
									determine which items from the VA tool would be necessary for this purpose. Our tool is not meant to be a comprehensive tool to assess every element of an ethics consultation or CEC's role, thus it is briefer than the VA tool. It is also designed to be used in ''real time'' by trained bioethics raters, not retrospectively self-administered by the ethics consultant. Once we agreed upon the primary items, we met weekly for 3–4 months to identify and determine the specific behaviors and skills we would be evaluating under each main item. This was a time consuming process involving in-depth discussion and debate. Before each weekly meeting, we viewed videos of simulated ethics consultations from past graduate bioethics courses to refine our tool, its specified items, and behaviors. We discussed and		For clarity of assessment, we developed the ACES tool to be used for individual consultants. We believe each individual ethics consultant needs to have this set of skills, even if they work within a small team model in practice. If the ACES tool was adapted to assess a small team of ethics consultants, the raters would have to rate each consultant individually or rate them all as one ''ethics consultant'' to assess whether the team covered all the necessary items on the evaluation. This approach would be more complicated to assess and, we suspect, much less straightforward to score. At this stage, we do not recommend using the ACES tool in this way.<br>One of the most challenging areas in the ACES tool development was how to view and rate students in a similar way—or inter-rater agreement. We spent a great deal of time discussing what qualifies as ''Done,'' ''Not Done'' or ''Done Incorrectly'' for each and every item. Rater agreement is an ongoing process which needs to be practiced and refreshed, and is not unique to our setting. We plan to assess and recalibrate rater agreement prior to each time a course is offered (1–2 per annum). What makes it more challenging in clinical ethics is the nature of some parts of the consultation; e.g., identifying and clarifying the ethical issues. What counts as ''Done'' for certain items in

For peer review only

No.	Title	Author/year	MERSQI	COREQ	Reason for inclusion			Undergraduate (UG)/Postgraduate (PG)	Methodology	Purpose of study	Key findings
					Communication	Ethics	Professionalism				
									agreed on all of the behaviors and skills listed under each item in the ACES tool. In the end, we agreed on 12 main items and 35 corresponding behaviors to be evaluated and scored.		our tool can be interpreted differently depending on the rater's background and potential biases. This type of item— communication and interpersonal skills in ethics—is not always as neat and clear cut as in other clinical areas such as surgical preparation checklists or putting in a central line. Further work needs to be done to train additional raters if we are to use the tool with more people over time. We plan to recruit additional students to take the ACES course and are working on refining the cases used and plan to evaluate the tool in other settings to obtain feedback on its reliability. When we used the ACES tool in the basic clinical ethics consultation course the feedback was overwhelmingly positive. Students appreciated a clear articulation of what was expected in an ethics consult. While recognizing the tool was not designed to be comprehensive, they valued having a structure and framework for thinking about the elements of an ethics consultation and the behaviors they should be exhibiting. It gave them a clear standard by which to assess themselves and others.
22	Evaluating practice-based learning and improvement: efforts to improve acceptance of portfolios	Fragneto, R. Y., Dilorenzo, A. N., Schell, R. M. and Bowe, E. A. 2010	8		✓		✓	PG	Intensive education about the goals and importance of portfolios began in January 2006, including presentations at departmental conferences and	To find out whether educational efforts improved acceptance of the portfolio and retrospectively audited the portfolio evaluation forms completed by faculty advisors.	Portfolios are considered a best methods techni- que by the ACGME for evaluation of practice-based learning and improvment. We have found that intensive education about the goals and importance of portfolios can enhance acceptance of this evaluation tool, resulting

No.	Title	Author/year	MERSQI	COREQ	Reason for inclusion			Undergraduate (UG)/Postgraduate (PG)	Methodology	Purpose of study	Key findings
					Communication	Ethics	Professionalism				
									one-on-one education sessions. Faculty advisors were instructed to evaluate each resident's portfolio and complete a review form. We retrospectively collected data to determine the percentage of review forms completed by faculty. The portfolio reviews also assessed the percentage of 10 required portfolio components residents had completed.		in improved compliance in completion and evaluation of portfolios.
23	Use of portfolios for assessment of global health residents: qualitative evaluation of design and implementation	Gibson, C., Chandratilake, M. and Hull, A.<br>2018	-	22	✓			PG	A qualitative study was conducted with academic experts in Canadian residency training, as well as directors and residents involved in Global Health study in order to assess the validity and benefit of such a tool. Through an online survey, interviews, and focus groups, views on the portfolio and intended content were collected and coded thematically.	To determine the robustness of portfolios as an assessment tool for Global Health Residents.	This qualitative evaluation validated the use of portfolios for this cohort of students while yielding comments and suggestions that will further enhance the interactive and flexible nature of this seldom used assessment tool. These findings contribute to the understanding of how Global Health assessment can remain individualized yet rigorous.
24	Assessment and Change: An Exploration of	Jocelyn M. Lockyer, PhD; Sanjeev Sockalingam, MD,	-	16	✓			PG	A total of 5000 entries from 2195 psychiatrists were	Fellows of the Royal College of Physicians and	There were 3841 entries for analysis; 1159 entries did not meet the criteria for

For peer review only - http://bmjopen.bmj.com/site/about/guidelines.xhtml

No.	Title	Author/year	MERSQI	COREQ	Reason for inclusion: Communication	Ethics	Professionalism	Undergraduate (UG)/Postgraduate (PG)	Methodology	Purpose of study	Key findings
	Documented Assessment Activities and Outcomes by Canadian Psychiatrists	MHPE, FRCPC; Craig Campbell, MD, FRCPC<br><br>2018							examined. A thematic analysis drawing on the framework analysis was undertaken of the 2016 entries.	Surgeons of Canada are required to participate in assessment activities for all new 5-year cycles beginning on or after January 2014 to meet the maintenance of certification program requirements. This study examined the assessment activities which psychiatrists reported in their maintenance of certification e-portfolios to determine the types and frequency of activities reported; the resultant learning, planned learning, and/or changes to the practice they planned or implemented; and the interrelationship between the types of assessment activities, learning that was affirmed or planned, and changes planned or implemented.	assessment. The most commonly reported activities were self-assessment programs, feedback on teaching, regular performance reviews, and chart reviews. Less frequent were direct observation, peer supervision, and reviews by provincial medical regulatory authorities. In response to the data, psychiatrists affirmed that their practices were appropriate, identified gaps they intended to address, planned future learning, and/ or planned or implemented changes. The assessment activities were internally or externally initiated and resulted in no or small changes (accommodations and adjustments) or redirections.<br><br>Psychiatrists reported participating in a variety of assessment activities that resulted in variable impact on learning and change. The study underscores the need to ensure that assessments being undertaken are purposeful, relevant, and designed to enable identification of outcomes that impact practice.
25	Development of a portfolio of learning for postgraduate family medicine training in South Africa: a Delphi study	Jenkins, L., Mash, B. and Derese, A.<br><br>2012	-	10	✓	✓		PG	A workshop held at the WONCA Africa Regional Conference in 2009 explored the purpose and broad contents of the portfolio. The 85 training outcomes, ideas from the WONCA workshop, the literature, and	This study aimed to achieve a consensus on the contents and principles of the first national portfolio for use in family medicine training in South Africa.	A portfolio was developed and distributed to the eight departments of Family Medicine in South Africa, and the CMSA, to be further tested in implementation. This was the first attempt to reach consensus on the development of a national portfolio for family medicine training in South Africa. Consensus was reached on 50 items to include, and 26

For peer review only

No.	Title	Author/year	MERSQI	COREQ	Reason for inclusion			Undergraduate (UG)/Postgraduate (PG)	Methodology	Purpose of study	Key findings
					Communication	Ethics	Professionalism				
									existing portfolios in the various universities were used to develop a questionnaire that was tested for content validity by a panel of 31 experts in family medicine in South Africa, via the Delphi technique in four rounds. Eighty five content items (national learning outcomes) and 27 principles were tested. Consensus was defined as 70% agreement. For those items that the panel thought should be included, they were also asked how to provide evidence for the specific item in the portfolio, and how to assess that evidence.		principles relating to the port- folio. A draft national portfolio and portfolio guide have been developed and distributed to all the medical schools in the country. Further revision and testing with registrars in training is underway, with the aim to deli- ver a final portfolio in the following year.
26	The national portfolio of learning for postgraduate family medicine training in South Africa: experiences of registrars and supervisors in clinical practice	Jenkins, L., Mash, B. and Derese, A.<br><br>2013	-	15	✓			PG	Semi-structured interviews were conducted with 17 purposively selected registrars and supervisors from all eight South African training programmes.	The aim of this study was to explore registrars' and supervisors' experience regarding the portfolio's educational impact, acceptability, and perceived usefulness for assessment of competence.	The learning portfolio had a significant educational impact in shaping work-place based supervision and training and providing formative assessment. Its acceptability and usefulness as a learning tool should increase over time as supervisors and registrars become more competent in its use. There is a need to clarify how it will be used in summative assessment.
27	Demonstration of Portfolios to Assess	PATRICIA S. O'SULLIVAN1,* , MARK D. RECKASE2, TINA	8	12	✓		✓	PG	22 participants in this demonstration project were all	Describes assessment of the reliability and validity of portfolios in a	We must continue to emphasize some important qualities of portfolios: res- ident choice and reflection.

No.	Title	Author/year	MERSQI	COREQ	Reason for inclusion			Undergraduate (UG)/Postgraduate (PG)	Methodology	Purpose of study	Key findings
					Communication	Ethics	Professionalism				
	Competency of Residents	MCCLAIN3, MILDRED A. SAVIDGE1 and JAMES A. CLARDY3<br><br>2004							residents in the psychiatry residency education program at the primary affiliate of a state university medical school.	psychiatry residency program.	These are important to the life-long learner. Portfolio has been able to aid the faculty in meeting ACGME expectations and has oriented programe to the increasingly important role of evaluation.
28	Developing and Successfully Implementing a Competency-Based Portfolio Assessment System in a Postgraduate Family Medicine Residency Program	Laura A. McEwen, PhD, Jane Griffiths, MD, CCFP, FCFP, and Karen Schultz, MD, CCFP, FCFP<br><br>2015	-	-	✓			PG	-	The use of portfolios in postgraduate medical residency education to support competency development is increasing; however, the processes by which these assessment systems are designed, implemented, and maintained are emergent. The authors describe the needs assessment, development, implementation, and continuing quality improvement processes that have shaped the Portfolio Assessment Support System (PASS) used by the postgraduate family medicine program at Queen's University since 2009.	The authors discuss the identification of impact measures at the individual, programmatic, and institutional levels and the ways the department uses these to monitor how PASS supports competency development, scaffolds residents' self- regulated learning skills, and promotes professional identity formation. They describe the "academic advisor" role and provide an appendix covering the portfolio elements. Reflection elements include learning plans, clinical question logs, confidence surveys, and reflections about continuity of care and significant incidents. Learning module elements cover the required, online bioethics, global health, and consult- request modules. Assessment elements cover each resident's research project, clinical audits, presentations, objective structured clinical exam and simulated office oral exam results, field notes, entrustable professional activities, multisource feedback, and in-training evaluation reports. Document elements are the resident's continuing medical education activities including procedures log, attendance log, and patient demographic summaries.

For peer review only

No.	Title	Author/year	MERSQI	COREQ	Reason for inclusion			Undergraduate (UG)/Postgraduate (PG)	Methodology	Purpose of study	Key findings
					Communication	Ethics	Professionalism				
29	Diving for PERLS Diving for PERLS Working and Performance Portfolios for Evaluation and Reflection on Learning	Linda E. Pinsky, MD, Kelly Fryer-Edwards, PhD<br><br>2004	-	-	✓		✓	PG	-	Professional competence requires a commitment to lifelong learning, self-assessment, and excellence. Complex skills such as these require flexible and comprehensive teaching and assessment measures. We describe a combination of working and performance portfolios that both foster and evaluate the development of professional competence. We explain the con- ceptual and practical underpinnings that maximize the effec- tiveness of these tools. Drawing on experience with University of Washington residents, we identify 5 criteria that can help promote successful use of portfolios: separate working and performance functions of portfolios, developing a supportive climate, developing skills in faculty and residents, observing progress over time, and fostering mentorship opportunities.	To incorporate portfolios successfully into a residency program, we recommend including 5 elements (Table 1). We had success in our program by introducing the program slowly, and dedicating time to faculty education on men- toring, reflection, goal setting, and feedback skills, recog- nizing that the use of portfolios requires new skills and a cultural transformation. We are pleased by the residents' appreciation of the process, their honesty in sharing and evaluating personal areas of challenge, and their incorpora- tion of this approach into other areas of their education. For example, a current inpatient chief resident, based on his ex- perience of the importance of goal setting to learning, revised the structure of morning report to include weekly goal-setting sessions as well as a method to evaluate its effectiveness. Residents seeking employment report finding components of the portfolio helpful in creating their CVs, crafting their applications, and as display examples at job applications.<br><br>To date, the literature reports few trials of the portfolio method for assessing professional competence. The Post Graduate Education Accreditation (PGEA) in Britain illus- trates sharing responsibility for learning between teachers and learners. PGEA experimented with portfolios

For peer review only

No.	Title	Author/year	MERSQI	COREQ	Reason for inclusion			Undergraduate (UG)/Postgraduate (PG)	Methodology	Purpose of study	Key findings
					Communication	Ethics	Professionalism				
											as a flex- ible, targeted means of meeting continuing medical edu- 18 –20 cation (CME) requirements. In their pilot study, physicians were responsible for identifying their own learning objectives and outlining how they were going to achieve and document those objectives. The physicians each met with a mentor to review appro- priate objectives and realistic means for achieving them. Structured reflection was built into the process and was seen as strength, explicitly moving away from the passive approach of most traditional lecture-based CME courses. In the PGEA experience, the shortcomings identified were time and mentor availability. However, participants found the “deep learning” and personal mastery they achieved to be well worth it. In addition, accrediting bodies had something of substance to review in assessing the practitioners’ continuing education. Portfolios fill a need within training programs to foster 21–23 and document professional competencies. dents, training is a time of professional identity formation. The current format of most residencies — didactic confer- ences and time-consuming clinical work—emphasizes either a passive or unreflective learner role. Through portfolios, residents can experience

For peer review only

No.	Title	Author/year	MERSQI	COREQ	Reason for inclusion			Undergraduate (UG)/Postgraduate (PG)	Methodology	Purpose of study	Key findings
					Communication	Ethics	Professionalism				
											and develop the skills of lifelong learning, critical thinking, self- assessment, and excellence that can be carried forward into their future careers and practices. They can complete residency with a clearer sense of their own strengths and limitations, and a sense of who they are as physicians.
30	How do postgraduate GP trainees regulate their learning and what helps and hinders them? A qualitative study	Sagasser, M. H., Kramer, A. W. and van der Vleuten, C. P. 2012	-	18	✓			PG	In a qualitative study with a phenomenologic approach we interviewed first- and third-year GP trainees from two universities in the Netherlands. Twenty-one verbatim transcripts were coded. Through iterative discussion the researchers agreed on the interpretation of the data and saturation was reached.	To explore how postgraduate trainees regulate their learning in the workplace, how external regulation promotes self-regulation and which elements facilitate or impede self-regulation and learning.	Trainees used a short and a long self-regulation loop. The short loop took one week at most and was focused on problems that were easy to resolve and needed minor learning activities. The long loop was focused on complex or recurring problems needing multiple and planned longitudinal learning activities. External assessments and formal training affected the long but not the short loop. The supervisor had a facilitating role in both loops.
31	First year specialist trainees' engagement with reflective practice in the e-portfolio	Helen McNeill • Jeremy M. Brown • Nigel J. Shaw 2010	7.5	12	✓			PG	A modified Delphi technique was used to develop a grading system to indentify the level of reflection recorded by participants in the e-portfolio. Transcripts of the reflective accounts were then analysed using a qualitative approach which involved coding and categorising	This study aims specifically to explore how they have engaged in reflection on their practice and how they utilise their learning portfolio to document evidence of this.	Findings indicate there is variation is the extent to which doctors both engage in and document evidence of reflection. Further research is needed to explore factors that enable or inhibit the use of the e-portfolio for reflection and whether recorded reflection is a true picture of the cognitive process involved.

For peer review only

No.	Title	Author/year	MERSQI	COREQ	Reason for inclusion			Undergraduate (UG)/Postgraduate (PG)	Methodology	Purpose of study	Key findings
					Communication	Ethics	Professionalism				
									the data. This study demonstrated a wide variation in both the quantity and quality of reflection. Of particular note in the qualitative data analysis were themes relating to clinical knowledge and skills, learning in practice, communication, feelings, types of learning experience reflected on and wider aspects of medical practice.		
32	Development of a learning portfolio to assess the competency of anesthesia residents in Thailand	Suraseranivongse, S., Chinachoti, T., Aroonpruksakul, N., Halilamien, P., Rushatamukayanunt, P., Raksamani, K., Sirivanasandha, B. and Mandee, S.<br><br>2011	12.5	8			✓	PG	Learning portfolio was developed from Thai Medical Council general competencies, academic activities, and performance assessment in several modalities. Twenty-four first year anesthesia residents and eight mentors were enrolled for this study. One staff mentored three residents and rated their competencies in portfolios, twice, four-months apart. Content validity was assessed by six content experts. Concurrent validity of portfolio was determined	Develop a portfolio for learning improvement in first year anesthesia residents in Thailand, and validate this portfolio as a competency evaluation and to identify strength and weakness of implementation.	All content experts accepted that this portfolio could assess general competencies of the first year anesthesia residents. The majority of mentors and residents (>70%) agreed with the benefit of portfolio based on learning development and competency assessment. However, half of residents were not satisfied with the burden from portfolio

No.	Title	Author/year	MERSQI	COREQ	Reason for inclusion			Undergraduate (UG)/Postgraduate (PG)	Methodology	Purpose of study	Key findings
					Communication	Ethics	Professionalism				
									by agreement with faculty global rating and in-training examination. Inter-rater reliability of portfolio was evaluated by five faculties that rated 24 residents. Practicality was commented upon by all mentors and residents in the questionnaire and semi-structure, open-ended questions.		
33	Education Research: Communication skills for neurology residents - Structured teaching and reflective practice	Watling, C. J. and Brown, J. B.<br><br>2007	8.5	10	✓	✓		PG	A group of 12 neurology residents participated in a series of six case-based communi-cation skills workshops. Each workshop focused on a particular clinical scenario, including break-ing bad news, discussing do-not-resuscitate orders, communicating with "difficult" patients, disclosing medical errors, obtaining informed consent for neurologic tests and procedures, and discussing life-and-death decisions with families of critically ill patients.	Despite the importance of communication skills for neurologists, specific training in this area at the residency level is often lacking. This study aimed to enhance learning of these skills and to encourage reflective practice around communication skills.	The program was well accepted, and residents rated the workshops as effective and relevant to their practice. Analysis of residents' portfolios revealed three themes relevant to patient–physician communication: 1) communication is more successful when adequate time is allowed, 2) the ability to empathize with patients and their families is essential to successful interactions, and 3) the develop- ment of specific approaches to challenging scenarios can facilitate effective interactions. The portfo- lios also demonstrated that residents would engage in reflective practice.<br><br>Targeting of communication skills training around specific clinical scenarios using neurologic cases was well accepted and was deemed relevant to practice. The use of portfolios may

For peer review only

No.	Title	Author/year	MERSQI	COREQ	Reason for inclusion			Undergraduate (UG)/Postgraduate (PG)	Methodology	Purpose of study	Key findings
					Communication	Ethics	Professionalism				
									Residents also kept reflective portfolios in which real examples of these interactions were recorded.		promote lifelong learning in this area.
34	What the HEC-C? An Analysis of the Healthcare Ethics Consultant-Certified Program: One Year in	Horner, C. Childress, A. Fantus, S. Malek, J.<br><br>2020			✓	✓		PG	-	In this paper, we explore the history that has led to this certification process, and evaluate the ability of the HEC-C Program to meet the goals it has set forth for HCECs. We describe the benefits and weak- nesses of the program and offer constructive feedback on how the process might be strengthened, as well as share our team's experience in preparing for the exam.	Ultimately, while we are generally supportive of efforts to professionalize the field and ensure competence among HCECs, we are skeptical that a limited process such as this provides much assurance of such compe- tence either for HCECs or their employers. We pro- pose that a more robust or two-tiered evaluation process is needed before such a credential could reli- ably attest to the competence and expertise of an HCEC.<br>Despite shortcomings in the Certification Process as it is currently designed, in general there are benefits of having some kind of certification for individual consultants, as well as HCEC programs and the broader HCEC field. For consultants, achieving and maintaining certification requires continued review of core materials to refresh ethics knowledge and pro- vides external validation of the consultant's ability to engage in consultation, boosting confidence that the individual is successfully engaged in continuing educa- tion. For HCEC programs, having credentialed con- sultants supports the program's mission to engage in quality consultation, taking a step toward ensuring individual consultants are performing their job in a manner

For peer review only - http://bmjopen.bmj.com/site/about/guidelines.xhtml

No.	Title	Author/year	MERSQI	COREQ	Reason for inclusion			Undergraduate (UG)/Postgraduate (PG)	Methodology	Purpose of study	Key findings
					Communication	Ethics	Professionalism				
											consistent with established standards in the field. Finally, for the HCEC community as a whole, having a singular credential helps to standardize the practice and validate ethics consultation as a defined and important healthcare specialty. This effort to legit- imize the field as a profession will support the inclu- sion of clinical ethics across healthcare, hopefully expanding the practice from a few select academic or larger healthcare systems to all health care settings. Reaching a point where a health care ethics credential signifies not only competence but also excellence in the ability to conduct a clinical ethics consultation, however, may still be a long way off.
35	Assessing Professionalism in Medicine – A Scoping Review of Assessment Tools from 1990 to 2018	Tay, K. T.<br>Ng, S.<br>Hee, J. M.<br>Chia, E. W. Y.<br>Vythilingam, D.<br>Ong, Y. T.<br>Chiam, M.<br>Chin, A. M. C.<br>Fong, W.<br>Wijaya, L.<br>Toh, Y. P.<br>Mason, S.<br>Krishna, L. K. R.<br><br>2020			✓		✓	UG and PG	Arksey and O'Malley's (2005) approach to scoping reviews was used to identify appropriate publications featured in four data- bases published between 1 January 1990 and 31 December 2018. Seven members of the research team employed thematic analysis to evaluate the selected articles.	Medical professionalism enhances doctor-patient relationships and advances patient-centric care. However, despite its pivotal role, the concept of medical professionalism remains diversely understood, taught and thus poorly assessed with Singapore lacking a linguistically sensitive, context specific and culturally appropriate assessment tool. A scoping review of assessments of professionalism in medicine was thus carried out to better guide its understanding	Prevailing assessments of professionalism in medicine must contend with differences in setting, context and levels of pro- fessional development as these explicate variances found in existing assessment criteria and approaches. However, acknowledging the sig- nificance of context-specific competency-based stages in medical professionalism will allow the forwarding of guiding principles to aid the design of a culturally-sensitive and practical approach to assessing professionalism.

For peer review only

No.	Title	Author/year	MERSQI	COREQ	Reason for inclusion			Undergraduate (UG)/Postgraduate (PG)	Methodology	Purpose of study	Key findings
					Communication	Ethics	Professionalism				
36	Content and outcomes of narrative medicine programmes: a systematic review of the literature through 2019	Remein, Christy DiFrances<br>Childs, Ellen<br>Pasco, John Carlo<br>Trinquart, Ludovic<br>Flynn, David B.<br>Wingerter, Sarah L.<br>Bhasin, Robina M.<br>Demers, Lindsay B.<br>Benjamin, Emelia J.<br><br>2020	-	-	✓			PG	We conducted a systematic review of literature published through 2019 using five major databases: PubMed, Embase, PsycINFO, ERIC and MedEdPORTAL. Eligible NM programming included textual analysis/close reading of published literature and creative/reflective writing. Qualifying participants comprised individuals from academic medicine and health sciences disciplines. We reviewed and categorised programme goals, content and evaluation activities to assess participant satisfaction and programme efficacy. Two members of the research team assessed the risk of bias, independently screening records via a two-round, iterative process to reach consensus on eligibility.	Narrative medicine (NM) incorporates stories into health sciences paradigms as fundamental aspects of the human experience. The aim of this systematic review is to answer the research question: how effective is the implementation and evaluation of NM programmes in academic medicine and health sciences? We documented objectives, content and evaluation outcomes of NM programming to provide recommendations for future narrative-based education.	Of 1569 original citations identified, we selected 55 unique programmes (described in 61 records). In all,<br>41 (75%) programmes reported a form of evaluation; evaluation methods lacked consistency. Twenty-two programmes used quantitative evaluation (13 well described), and 33 programmes used qualitative evaluation (27 well described). Well-described quantitative evaluations relied on 32 different measures (7 validated) and showed evidence of high participant satisfaction and pre-post improvement in competencies such as relationship- building, empathy, confidence/personal accomplishment, pedagogical skills and clinical skills. An average of 88.3% of participants agreed or strongly agreed that the programme had positive outcomes. Qualitative evaluation identified high participant satisfaction and improvement<br>in competencies such as relationship-building, empathy, perspective-taking/reflection, resilience and burnout detection/mitigation, confidence/personal accomplishment, narrative competence, and ethical inquiry.<br><br>Evaluation suggests that NM programming leads to high participant satisfaction and positive outcomes

No.	Title	Author/year	MERSQI	COREQ	Reason for inclusion			Undergraduate (UG)/Postgraduate (PG)	Methodology	Purpose of study	Key findings
					Communication	Ethics	Professionalism				
											across various competencies. We suggest best practices and innovative future directions for programme implementation and evaluation.
37	Use of portfolios in early undergraduate medical training	Driessen, Erik Van Tartwijk, Jan Vermunt, Jan van der Vleuten, Cees 2003		10		✓		UG	During the academic year 2001–02 242 first-year medical students compiled a portfolio. Semi-structured interviews were held with a select group of students to explore the effect of the portfolio on reflection. Students from four random mentor groups, 39 in total, were approached and asked if they would be interviewed on the portfolio. With the exception of one student, all students were prepared to take part in the interview. The interview schedule focused on the process of compiling a portfolio and its possible effect on reflective ability. In addition to this, students were given the opportunity to offer suggestions for improvement.	This article describes the use of portfolios in **early** undergraduate medical training... In this article, we will describe the Maastricht portfolio as an illustration of an early undergraduate portfolio. We will discuss the reasons for the choices we made in designing the portfolio and report on a first evaluation, focusing on the effects on students' reflective ability.	The majority of students were of the opinion that analysing one's competences in a portfolio was instructive and meaningful. With regard to learning how to reflect and recognize learning needs, however, mentor coaching proved to be necessary. The results thus far show that the portfolio is a worthwhile addition to existing assessment and learning tools. Our preliminary findings show that the introduction of such a portfolio in the early stages of medical training seems to be an effective way for students to learn how to reflect on their learning and behaviour.
38	Conditions for reflective use of portfolios in	Driessen, E. W. van Tartwijk, J. Overeem, K.		18			✓	UG	We designed a portfolio that was aimed at	Portfolios are often used as an instrument with	The conditions for successful reflective use of portfolios that emerged from

For peer review only - http://bmjopen.bmj.com/site/about/guidelines.xhtml

No.	Title	Author/year	MERSQI	COREQ	Reason for inclusion			Undergraduate (UG)/Postgraduate (PG)	Methodology	Purpose of study	Key findings
					Communication	Ethics	Professionalism				
	undergraduate medical education	Vermunt, J. D. van der Vleuten, C. P. 2005							stimulating reflection in early undergraduate medical education, using experiences described in the medical educa- tion literature and elsewhere. Conditions for reflect- ive portfolio use were identified through interviews with 13 teachers (mentors), who were experienced in mentoring students in the process of developing their portfolios. The interviews were analysed according to the principles of grounded theory.	which to stimulate students to reflect on their experiences. Research has shown that working with portfolios does not automatically stimulate reflection. In this study we addressed the question: What are the conditions for successful reflective use of portfolios in undergraduate medical education?	the interviews fell into 4 categories: coaching; portfolio structure and guidelines; relevant experiences and materials, and summative assessment. According to the mentors, working with a portfolio designed to meet these conditions will stimulate students' reflective abilities...This study shows that portfolios are a potentially valuable method of assessing and develop- ing students' reflective skills in undergraduate med- ical training, provided certain conditions for effective portfolios are recognised and met. Portfolios have a strong potential for enhancing learning and assess- ment but they are very vulnerable and may easily lead to disappointment. Before implementing portfolios in education, one should first consider whether the necessary conditions can be fulfilled, including an appropriate portfolio structure, an appropriate assessment procedure, the provision of enough new experiences and materials, and sufficient teacher capacity for adequate coaching and assessment.
39	Portfolio Assessment Implementation in Clinical Year of Community Medicine Module: Students Perspective	Ekayanti, Fika Risahmawati, Risahmawati Fadhilah, Marita 2017	7				✓	UG	Nine portfolios were assigned to students within 5 weeks length module. The data was collected by questionnaires from 46 students after completing module in November 2016. Students were	This study aimed to identify the correlation of students' perspective for using portfolio and their portfolio scores.	Most students (36/78.3%) felt that the portfolio was not appropriate as their summative assessment. They preferred less portfolios, 30 (63%) students requested to decrease portfolios to 3-5, 28.3% to 6-8 and only 8.7% to <3. There was no significant correlation between students' satisfaction of using portfolio

For peer review only

No.	Title	Author/year	MERSQI	COREQ	Reason for inclusion: Communication	Reason for inclusion: Ethics	Reason for inclusion: Professionalism	Undergraduate (UG)/Postgraduate (PG)	Methodology	Purpose of study	Key findings
									asked about their satisfaction of using portfolio, then correlated to their portfolio scores. The reasons and suggestion for ideal portfolios were identified. Analysis was done by SPSS 20 using Rank Spearman correlation test. Students felt that creating portfolios were not comfortable.		to their portfolios score (p=0.262), while there was significant correlation between students' score to the number of portfolios to be submitted (p=0.017; r=0.349). Reasons for decreasing number of portfolios were inadequate time and many other tasks to be finished. Students in UIN Jakarta haven't used to retelling their experiences and reflecting them in writing as perceived in many students who were not used to portfolios. Doing portfolios need lots of work and time-consuming. Having lesser number of portfolios to be submitted would encourage students to create portfolios. Building familiarization, time management and good motivation for creating portfolios were important to successful portfolios. There was no significant correlation between students' satisfaction to their scores. Students should be encouraged to get comfortable in creating portfolios for the benefit as lifelong learning tool. Motivation to write and reflect should be nurtured to improve students' portfolio satisfaction and commitment.
40	Students' perception of portfolio as a learning tool at King Abdulaziz University Medical School	Fida, N. M. Hassanien, M. Shamim, M. S. Alafari, R. Zaini, R. Mufti, S. Al-Hayani, A. Farouq, M. Al-Zahrani, H. 2018	8	14	✓	✓	✓	UG	Portfolios were introduced in the 2nd through 5th years at King Abdulaziz University over a two-year period (2013–2015). At the end of each academic year, students completed a	Medical education has a longstanding tradition of using logbooks to record activities. The portfolio is an alter- native tool to document competence and promote reflective practice. This study assessed the	The results showed a difference in focus between basic and clinical years: in basic years students' focus was on acquiring practical skills, but in clinical years they focused more on acquiring complex skills, including identifying and man- aging problems. The questionnaire responses nonetheless revealed a

No.	Title	Author/year	MERSQI	COREQ	Reason for inclusion			Undergraduate (UG)/Postgraduate (PG)	Methodology	Purpose of study	Key findings
					Communication	Ethics	Professionalism				
									mixed questionnaire that included a self-assessment of skills learned through the use of portfolio.	acceptance of portfolio use among Saudi undergraduate medical students.	positive trend in acceptance (belief in the educational value) of portfolios among students and their mentors, across the years of the program. Using portfolios as a developmental learning and formative assessment tool in the early undergraduate years was found to contribute to students' ability to create their own clinical skills guidelines in later years, as well as to engage in and appreciate reflective learning.
41	The use of portfolios to foster professionalism: Attributes, outcomes, and recommendations	Renato Soleiman Franco, Camila Ament Giuliani dos Santos Franco,Olívia Pestana,Milton Severo &Maria Amélia Ferreira<br><br>2017		9		✓	✓	UG	A systematic review on the use of portfolios in teaching professionalism to medical students identified 1257 papers. Of these, 11 articles met all inclusion criteria	The main objective of this research was to review the characteristics of portfolios and their outcomes for teaching professionalism to undergraduate medical students	According to the papers, the use of portfolios for teaching professionalism shows versatility, supports learning strategies and has the potential to be used in different contexts, including for formative and summative purposes. The weaknesses were based on the artificiality of the reflections, deficient instructions, time-consuming processes and preference among students for other teaching methods. Students complained about feeling that the reflection was 'forced', and they tended to write based on social conventions rather than reveal their true thoughts. Reflection is a powerful component of the portfolio, but the method by which it is taught could easily ruin its potential to boost professionalism. Requiring reflection did not ensure its achievement; increased understanding by students regarding how and why they were doing it, the clarity of assessment methods and constructive feedback might strengthen the potential for success. A

No.	Title	Author/year	MERSQI	COREQ	Reason for inclusion			Undergraduate (UG)/Postgraduate (PG)	Methodology	Purpose of study	Key findings
					Communication	Ethics	Professionalism				
											framework was designed to support faculty members in developing and applying portfolios with a clear and broad view of this teaching strategy.
42	AMEE Medical Education Guide No. 24: Portfolios as a method of student assessment	Friedman Ben David, M.<br>Davis, M. H.<br>Harden, R. M.<br>Howie, P. W.<br>Ker, J.<br>Pippard, M. J.<br><br>2001	-	-	✓	✓	✓	UG	-	This guide is intended to inform medical teachers about the use of portfolios for student assessment. It provides a background to the topic, reviews the range of assessment purposes for which portfolios have been used, identifies possible portfolio contents and outlines the advantages of portfolio assess- ment with particular focus on assessing professionalism...The current state of understanding of the technical, psychometric issues relating to portfolio assessment is clarified. The final part of the paper provides a practical guide for those wishing to design and implement portfolio assessment in their own institutions.	-
43	Teaching professionalism in the early years of a medical curriculum: a qualitative study	Goldie, J.<br>Dowie, A.<br>Cotton, P.<br>Morrison, J.<br><br>2007		18	✓	✓		UG	A qualitative approach was adopted involving semistructured interviews, on a 1 in 6 sample of tutors involved in teaching in the early curricular years, and 3 student focus groups. The	This study investigated tutors' and students' perspectives of the delivery of profession- alism in the early years of Glasgow's learner-centred, problem-based learning (PBL), integrated medical curriculum.	Involvement in teaching raised students' and tutors' awareness of their professionalism. Learning activities promoting critical reflection were most effective. The integration of professionalism across the domains of Vocational Studies (135) was important for learning; however, it was not well integrated with the

For peer review only

No.	Title	Author/year	MERSQI	COREQ	Reason for inclusion			Undergraduate (UG)/Postgraduate (PG)	Methodology	Purpose of study	Key findings
					Communication	Ethics	Professionalism				
									findings were subjected to between-method triangulation.		PBL core. Integration was pro- moted by having the same tutor present throughout all VS sessions. Early patient contact experiences were found to be particularly important. The hidden curriculum provided both opportunities for, and threats to, learning. The small-group format provided a suitable environment for the examination of pre-existing perspectives. The portfolio was an effective learning tool, although its assessment should be formalised.<br><br>Reflection is integral to professional development. Early clinical contact is an important part of the process of socialisation, as it allows students to enter the community of practice that is the medical profession. Role models can contribute powerfully to students' learning and identity formation. As students move towards fuller participation, the clinical milieu should be controlled to maximise the influence of role models, and opportunities for guided reflection should be sustained.
44	Assessing students' personal and professional development using portfolios and interviews	Gordon, J.<br><br>2003	6.5			✓	✓	UG	The instruments used to assess Year 1 stu- dents in PPD are a portfolio and interview. This assessment format encourages students to explore ideas and values that are important to them	Medical schools are placing more emphasis on students' personal and professional development (PPD) and are seeking ways of assessing student progress towards meeting outcome goals in relation to professionalism. The	In 1997⁄98, 96% of students agreed that they had engaged in useful reflection on their approach to the course and 91% agreed that the experience was worthwhile. A further 76% of students agreed that they could see opportunities to modify their approach in some ways as result of this exercise.

For peer review only

No.	Title	Author/year	MERSQI	COREQ	Reason for inclusion			Undergraduate (UG)/Postgraduate (PG)	Methodology	Purpose of study	Key findings
					Communication	Ethics	Professionalism				
									and relevant to the PPD theme. A confidential interview, based on the PPD goals, is held with a faculty member who has read the student's portfolio.	Faculty of Medicine at the University of Sydney sought an assessment method that would demonstrate the value of reflection in attaining PPD, provide feedback and encourage stu- dents to take responsibility for setting and achieving high standards of performance.	
45	Developing a sustainable electronic portfolio (ePortfolio) program that fosters reflective practice and incorporates CanMEDS competencies into the undergraduate medical curriculum	Hall, P. Byszewski, A. Sutherland, S. Stodel, E. J. 2012	-	-	✓		✓	UG	-	In this article, the authors describe the development of an electronic Portfolio (ePortfolio) program that enables uOttawa medical students to document their activities and to demonstrate their development of competence in each of the eight roles	The authors reflect on the challenges they faced in the development and implementation of the ePortfolio program and share the lessons they have learned along the way to a successful and sustainable program. These lessons include switching from a complex information technology system to a user-friendly, Web- based blog platform; rethinking orientation sessions to ensure that faculty and students understand the value of the ePortfolio program; soliciting student input to improve the program and increase student buy-in; and providing faculty development opportunities and recognition.
46	The utility of reflective portfolios as a method of assessing first year medical students' personal and professional development	Rees, Charlotte E. Shepherd, Maggie Chamberlain, Suzanne 2005	8	17	✓			UG	The construct validity of the criteria was established by exploring its relationship with two other assessment methods and its reliability was determined using the	-	Themes emerging from the focus groups include students preferring the structured nature of the portfolios but assessors feel- ing that this reduced the uniqueness of the portfolios. Although students understood the importance of reflective practice, some disliked the process of reflection, particularly

For peer review only

No.	Title	Author/year	MERSQI	COREQ	Reason for inclusion			Undergraduate (UG)/Postgraduate (PG)	Methodology	Purpose of study	Key findings
					Communication	Ethics	Professionalism				
									generalizability (G) coefficient. Focus groups with assessors and students were convened to explore their views of the portfolios. Two portfolio analyses had a G coefficient of .42. Performance in the portfolio analyses was correlated with performance in personal and professional development judgements (r = −.512, p < .01) and scientific reports (r = .273, p = .002).		reflective writing. Educators should design assessment criteria that maximize reliability, validity and acceptability rather than simply focusing on reliability alone.<br><br>Despite these methodological limitations, this study provides support for the reli- ability and validity of the portfolio analyses assessment criteria at Peninsula Medical School.
47	Reflections: an inquiry into medical students' professional identity formation	Wong, A.<br>Trollope-Kumar, K.<br><br>2014		14	✓	✓		UG	Sixty-five medical students (46 women; 19 men) from a class of 194 consented to the study of their portfolios. In total, 604 reflections were analysed and coded using thematic narrative analysis. The codes were merged under subthemes and themes. Common or recurrent themes were identified in order to develop a descriptive framework of professional identity formation. Reflections were then analysed	The purpose of this study was to understand the major influences on medical students' professional identity formation.	Five major themes were associated with professional identity formation in medi- cal students: prior experiences, role models, patient encounters, curriculum (formal and hidden) and societal expectations. Our longi- tudinal analysis shows how these themes inter- act and shape pivotal moments, as well as the iterative nature of professional identity from the multiple ways in which individuals con- struct meaning from interactions with their environments.<br><br>Our study provides a window on the dynamic, discursive and constructed nature of professional identity formation. The five key themes associated with professional identity formation provide strategic

No.	Title	Author/year	MERSQI	COREQ	Reason for inclusion			Undergraduate (UG)/Postgraduate (PG)	Methodology	Purpose of study	Key findings
					Communication	Ethics	Professionalism				
									longitudinally within and across individual portfolios to examine the professional identity formation over time with respect to these themes.		opportuni- ties to enable positive development. This study also illustrates the power of reflective writing for students and tutors in the professional identity formation process.
48	Content validity of workplace-based portfolios: A multi-centre study	Nele R.M. Michels, Marijke Avonts, Griet Peeraer, Kris Ulenaers, Luc F. Van Gaal, Leo L. Bossaert, Erik W. Driessen, Arno M.M. Muijtjens & Benedicte Y. De Winter<br><br>2016	9.5		✓		✓	UG	We reviewed 120 workplace portfolios at three medical universities (Belgium and the Netherlands). To validate their content, we developed a Validity Inventory for Portfolio Assessment (VIPA) based on the CanMEDS roles. Two raters evaluated each portfolio and indicated for each VIPA item whether the portfolio provided sufficient information to enable satisfactory assessment of the item. We ran a descriptive analysis on the validation data and computed Cohen's Kappa to investigate interrater agreement.	This study sought to evaluate the content validity of portfolios as reflected in their capacity to adequately assess achieved competences of medical students during clerkships.	The portfolios adequately covered the items pertaining to the communicator (90%) and professional (87%) roles. Coverage of the medical expert, collaborator, scholar and manager roles ranged between 75% and 85%. The health advocate role, covering 59%, was clearly less well represented. This role also exhibited little interrater agreement (Kappa50.4).<br><br>This study lends further credence to the evidence that portfolios can indeed adequately assess the different CanMEDS roles during clerkships, the health advocate role, which was less well represented in the portfolio content, excepted.
49	Does a summative portfolio foster the development of capabilities such	Anthony J O'sullivan, Amanda C Howe, Susan Miles, Peter Harris, Chris S Hughes, Philip Jones, Helen	7.5		✓	✓	✓	UG	A questionnaire was designed to evaluate undergraduate medical students' experiences of	The aims of this study were (i) to determine whether preparing a portfolio helps promote students'	Students perceive portfolio preparation as an effective learning tool for the development of capabilities such as understanding ethical and legal principles

For peer review only

No.	Title	Author/year	MERSQI	COREQ	Reason for inclusion			Undergraduate (UG)/Postgraduate (PG)	Methodology	Purpose of study	Key findings
					Communication	Ethics	Professionalism				
	as reflective practice and understanding ethics? An evaluation from two medical schools	Scicluna & Sam J Leinster<br><br>2012							completing a portfolio at two medical schools. A total of 526 (45% response rate) students answered the on-line questionnaire. Students from both medical schools gave the highest ranking for the portfolio as a trigger for reflective practice. 63% of students agreed their portfolio helped them develop reflective practice skills (p < 0.001), whereas only 22% disagreed. 48% of students agreed portfolios helped them understand ethical and legal principles whereas 29% disagreed (p < 0.001). In contrast, only 34% of students thought the portfolio helped them to develop effective communication.	development in a range of capabilities including understanding ethical and legal principles, reflective practice and effective communication, and (ii) to determine to what extent the format of the portfolio affected the outcome by comparing the experiences of students at two different medical schools.<br><br>Therefore, the primary aim of this study was to determine whether the process of preparing a portfolio report or essay helped students appreciate and develop in a range of capabilities, such as understanding ethical and legal principles and reflective practice; capabilities which are not the focus of traditional assessments such as clinical or written examina- tions. The format of the portfolio assessment was different in each of the two medical schools in the study, and so a secondary aim was to determine to what extent the format of the assessment affected the outcome by comparing the student experiences with their portfolio	and reflective practice, whereas other capabilities such as effective communication require complementary techniques and other modes of assessment.

For peer review only

No.	Title	Author/year	MERSQI	COREQ	Reason for inclusion			Undergraduate (UG)/Postgraduate (PG)	Methodology	Purpose of study	Key findings
					Communication	Ethics	Professionalism				
										assessment in the two schools.	
50	Feasibility and Outcomes of Implementing a Portfolio Assessment System Alongside a Traditional Grading System	Celia Laird O'Brien, PhD, Sandra M. Sanguino, MD, MPH, John X. Thomas, PhD, and Marianne M. Green, MD<br><br>2016	7		✓		✓	UG	In 2009, the authors developed a portfolio system that served as a repository for all student assessments organized by competency domain. Five competencies were selected for a preclerkship summative portfolio review. Students submitted reflections on their performance. In 2014, four clinical faculty members participated in standard-setting activities and used expert judgment and holistic review to rate students' competency achievement as "progressing toward competence," "progressing toward competence with some concern," or "progressing toward competence pending remediation." Follow-up surveys measured students' and faculty members'	This study describes the development and implementation of a longitudinal competency-based electronic portfolio system alongside a graded curriculum at a relatively large U.S. medical school.	Faculty evaluated 156 portfolios and showed high levels of agreement in their ratings. The majority of students achieved the "progressing toward competence" benchmark in all competency areas. However, 31 students received at least one concerning rating, which was not reflected in their course grades. Students' perceptions of the system's ability to foster self-assessment were mixed.

For peer review only

No.	Title	Author/year	MERSQI	COREQ	Reason for inclusion			Undergraduate (UG)/Postgraduate (PG)	Methodology	Purpose of study	Key findings
					Communication	Ethics	Professionalism				
									perceptions of the process.		
51	Feelings related to first patient experiences in medical school A qualitative study on students' personal portfolios	Kaisu H. Pitkälä a,*, Taina Mäntyranta b<br><br>2003? 2004		14	✓		✓	UG	Twenty-two volunteer third and fourth year medical students (15 women and 7 men) of the University of Helsinki participated in a portfolio course for 1 year. Their reflective learning diaries and writings on specific themes were analyzed by qualitative content analysis	Feelings and thoughts of medical students related to first patient experiences during the first clinical year were examined	First patient encoun- ters were strong emotional experiences for medical students. The first patient examination was often described as an anxiety-provoking and confusing incident. Other emotionally significant encounters included helplessness when faced with serious illness and death, and role confusion when examining patients of one's own age but opposite sex. Students felt guilty for using patients for their own learn- ing purposes. Portfolios as learning tools may help in recognizing key experiences and support professional development of medical students.
52	Linking assessment to undergraduate student capabilities through portfolio examination	Anthony J. O'Sullivan , Peter Harris , Chris S. Hughes , Susan M. Toohey , Chinthaka Balasooriya , Gary Velan , Rakesh K. Kumar & H. Patrick McNeil<br><br>2012	8.5		✓	✓	✓	UG	Students are required to complete assessments linked to graduate capabilities. In Year 3, a portfolio review occurs (205–248 students per year), focusing on students' grades and feedback from assessments and a reflective essay is submitted. In the essay, students reflect on their progress, identify areas of weakness and detail plans for improvement. Progress in each capability is summatively graded against	This article describes an e-portfolio which closely aligns learning and reflection to graduate capabilities, incorporating features that address concerns about portfolios	Portfolios are an established method of assessment, although concerns do exist around their validity for capabilities such as reflection and self-direction

For peer review only

No.	Title	Author/year	MERSQI	COREQ	Reason for inclusion			Undergraduate (UG)/Postgraduate (PG)	Methodology	Purpose of study	Key findings
					Communication	Ethics	Professionalism				
									specific criteria and feedback is provided.		
53	Medical student resilience strategies: A content analysis of medical students' portfolios	Richard A. Prayson · S. Beth Bierer · Elaine F. Dannefer<br><br>2016		12	✓	✓	✓	UG	We retrospectively reviewed a sampling of year 1, 2 and 5 portfolio essays focused on the Personal Develop- ment competency and performance milestones, written by 49 students from three different classes in a 5-year pro- gramme devoted to training physician investigators. Two medical educators used a framework established by Jensen and colleagues (2008) to identify the nature and prevalence of various resilience strategies (valuing the physician role, self-awareness, personal arena, professional arena, profes- sional support and personal support) medical students re- ported in portfolio essays.	A student's ability to employ resilience strategies to self-regulate behaviour is critical to the student's future career as a physician. (The aim is to examine this??)	All students documented at least one strategy in their essays each year. In all years, the most commonly documented strategies were in the personal arena (95.7% of year 1, 98% of year 2 and 87.8% of year 5 portfolios). The least frequently documented strategy in all years was professional support (42.8% of year 1, 38.8% of year 2, and 28.6% of year 5 portfolios). Year 5 portfolios discussed personal support strategies (79.6%) more frequently than year 1 (53.1%) and year 2 (59.2%) portfolios.<br><br>The results suggest that medical students can identify stressors and articulate resilience strategies that can be employed to potentially address them.
54	Portfolio Assessment during Medical Internships: How to Obtain a Reliable and Feasible	NRM Michels, EW Driessen, AMM Muijtjens, LF Van Gaal, LL Bossaert, BY De Winter<br><br>2009		9.5			✓	UG	The domain-oriented reliability of 61 double-rated portfolios was measured, using a generalisability	In this study, we investigated whether assessment during medical internship by a portfolio can combine reliability and feasibility.	We obtained reliability (Φ coefficient) of 0.87 with this internship portfolio containing 15 double-rated tasks. The generalisability analysis showed that an acceptable level of reliability

No.	Title	Author/year	MERSQI	COREQ	Reason for inclusion			Undergraduate (UG)/Postgraduate (PG)	Methodology	Purpose of study	Key findings
					Communication	Ethics	Professionalism				
	Assessment Procedure?								analysis with portfolio tasks and raters as sources of variation in measuring the performance of a student.		(Φ = 0.80) was maintained when the amount of portfolio tasks was decreased to 13 or 9 using one and two raters, respectively.<br><br>Our study shows that a portfolio can be a reliable method for the assessment of workplace learning. The possibility of reducing the amount of tasks or raters while maintaining a sufficient level of reliability suggests an increase in feasibility of portfolio use for both students and raters.
55	Twelve tips for introducing E-Portfolios with undergraduate students	Alis Moores & Maria Parks<br><br>2010	-	-	✓			UG	-	-	-
56	Use of Portfolios in Undergraduate Medical Training: First Meeting With a Patient	Nilgün ÖZÇAKAR, Vildan MEVSİM, Dilek GÜLDAL<br><br>2009		10	✓		✓	UG	We designed a portfolio that was aimed at making reflection in early undergraduate medical education. Conditions for portfolio use were identified through interviews with six trainers who were experienced in mentoring students in the process of developing their portfolios. Analysis of writings was per- formed using qualitative content analysis.	Portfolios, as learning tools, are becoming increasingly important in medical education. Our aim was to evaluate the contents of portfolios used in medical education.	The conditions for successful reflective use of portfolios that emerged from the interviews fell into catego- ries: practice evaluation, patient perspective and primary prevention/health promotion. According to the students, working with a portfolio designed to meet these conditions is very useful and will stimulate students' abilities. The stu- dents shared the same opinion that analyzing one's com- petences in a portfolio was instructive and meaningful.<br><br>According to our students, portfolios are a potentially valuable method of developing students' skills in undergraduate medical education, provided that certain conditions for effective portfolios are recognized and met. The portfolio is a worthwhile addition to existing

For peer review only

No.	Title	Author/year	MERSQI	COREQ	Reason for inclusion			Undergraduate (UG)/Postgraduate (PG)	Methodology	Purpose of study	Key findings
					Communication	Ethics	Professionalism				
											assessment and learning tools.
57	What Is the Relationship Between a Preclerkship Portfolio Review and Later Performance in Clerkships?	Celia Laird O'Brien, PhD, John X. Thomas Jr, PhD, and Marianne M. Green, MD<br><br>2018	9.5		✓		✓	UG	The authors divided students into two groups based on a summative preclerkship portfolio review in 2014: students who had concerning behavior in one or more competencies and students progressing satisfactorily. They compared how students in these groups later performed on two clerkship outcomes as of October 2015: final grades in required clerkships, and performance on a clerkship clinical composite score. They used Mann–Whitney tests and multiple linear regression to examine the relationship between portfolio review results and clerkship outcomes. They used USMLE Step 1 to control for knowledge acquisition.	This study examines whether a preclerkship portfolio review is a valid method of identifying problematic student behavior affecting later performance in clerkships.	Students with concerning behavior preclerkship received significantly lower clerkship grades than students progressing satisfactorily (P = .002). They also scored significantly lower on the clinical composite score (P < .001). Regression analysis indicated concerning behavior was associated with lower clinical composite scores, even after controlling for knowledge acquisition.<br><br>The results show a preclerkship portfolio review can identify behaviors that impact clerkship performance. A comprehensive portfolio system is a valid way to measure behavioral competencies.
58	Educating undergraduate medical students about oncology: A literature review	Cave, Judith<br><br>2006	-	-	✓			UG	MEDLINE, Psychinfo, ERIC, TIMELIT, EMBASE, CINAHL and the Cochrane CENTRAL Register of	This article is a review of the literature regarding teaching oncology to undergraduate medical students.	The main findings can be summarized as follows: the involvement of patients in teaching is popular with students and portfolio learning is a successful way of involving patients; the use of standardized patients to

No.	Title	Author/year	MERSQI	COREQ	Reason for inclusion			Undergraduate (UG)/Postgraduate (PG)	Methodology	Purpose of study	Key findings
					Communication	Ethics	Professionalism				
									Controlled Trials (CENTRAL) were searched, using the search terms cancer, oncology, education, undergraduate, and teaching.		teach breast examination improves students' performance in clinical assessment; the use of silicone models to teach breast examination improves students' sensitivity for detecting breast lumps; computer aided learning modules have a role, but are not superior to other types of learning; learning about cancer screening and prevention increases students' knowledge, improves their self rated skills, and changes their behavior; and cancer patients have an important role to play in teaching undergraduate communication skills.<br><br>We have found 48 articles on undergraduate teaching in oncology. Oncology teachers should consider adopting the evidence based approaches outlined in this review, and there should be more emphasis on educational research within the field of oncology.
59	Integrating the assessment of interprofessional education into the health care curriculum	Anderson, E. S. and Kinnair, D.<br><br>2016	-	-	✓	✓	✓	UG	-	Reflection on some of the challenges associated with assessment in IPE by describing the experiences at one UK-based medical school	In regard to written reflections, reading 240 student entries per learning event is time-consuming, making<br>it challenging to offer individual feedback. Some students have challenged the formative outcomes that were graded for progression across broad boundaries of unsatisfactory, satisfactory or excellent. Checking for consistency across a small numbers of marker illuminates the subjective perspectives and interpretations of individual

No.	Title	Author/year	MERSQI	COREQ	Reason for inclusion			Undergraduate (UG)/Postgraduate (PG)	Methodology	Purpose of study	Key findings
					Communication	Ethics	Professionalism				
											markers on what constitutes a meaningful reflection. Nevertheless, offering this early writing platform has proved to be extremely helpful and enables educators to determine if students can reflect critically and apply meaning
60	A model of engagement in reflective writing-based portfolios: Interactions between points of vulnerability and acts of adaptability	Arntfield, S., Parlett, B., Meston, C. N., Apramian, T. and Lingard, L.<br><br>2015	7	21			✓	UG	Using mixed-methods rooted in grounded theory, 139 students and 13 mentors completed questionnaires, 23 students participated in four focus groups and 9 mentors in individual interviews	The aim of this study was to explore conditions affecting the experience of teaching and learning from the perspective of both students and mentors in a reflective writing-based portfolio initiative.	The overarching theme in our data was student–mentor engagement. Our results confirm previous literature describing portfolio as a vulnerable method of learning, extend this concept by identifying and categorizing specific points of vulnerability, and contribute new knowledge regarding acts of adaptability, which serve to strengthen the student–mentor relationship.<br><br>Engagement is central to the success of portfolio and is shaped by a dynamic interaction between points of vulnerability and acts of adaptability. We propose a model of engagement in portfolio that can be used for faculty development to optimize student–mentor engagement.
61	Support for portfolio in the initial years of the undergraduate medical school curriculum: what do the tutors think?	Austin, C. and Braidman, I.<br><br>2008	7	18	✓		✓	UG	A mixed method approach was used with data collected from both questionnaires and a focus group meeting.	The aims of this study were to investigate the views of these tutor facilitators on the delivery and support of portfolios in this way.	Tutor facilitators were generally positive about their roles in the support of portfolio development in group sessions and identified several advantages to this type of tutoring – namely the value of group discussion and discussion between peers to encourage reflection, and the practical ability to integrate portfolio sessions more closely with clinical experiences – which would not be possible in one to one tutor/student meetings.

For peer review only

No.	Title	Author/year	MERSQI	COREQ	Reason for inclusion			Undergraduate (UG)/Postgraduate (PG)	Methodology	Purpose of study	Key findings
					Communication	Ethics	Professionalism				
											With the training and guidance notes provided tutors were confident that they understood the support they should give students and felt that they could encourage reflection.<br><br>Thus, tutors perceived that the support of portfolio in groups is an effective way of developing portfolios for large numbers of students.
62	Use of portfolios by medical students: significance of critical thinking	Azer, S. A.<br><br>2008	-	-	✓		✓	UG	-	The aims of this paper are to: (1) understand the uses of portfolios and the rationale for using reflection in the early years of a PBL curriculum; (2) discuss how to introduce portfolios and encourage students' critical thinking skills, not just reflection; and (53) provide students with tips that could enhance their skills in constructing good portfolios.	-
63	Time to Loosen the Apron Strings: Cohort-based Evaluation of a Learner-driven Remediation Model at One Medical School	Bierer, S. B., Dannefer, E. F. and Tetzlaff, J. E.<br><br>2015	10		✓			UG	We used a retrospective, cohort-based approach to examine our learner-driven remediation model.<br>Data sources: We consulted MSPRC records to identify all CCLCM students from six class cohorts (2009–2014) placed in remediation during medical school, and compared remedial students	To examine a remediation model where students, rather than faculty, develop remedial plans to improve performance.<br>We present an innovative, systematic approach to remediation purposefully designed to engage learners with the development and implementation of remedial plans to improve their performance. We first describe our approach to	From abstract -- participants included 177 students from six classes (2009–2014). Twenty-six were placed in remediation, with more referrals occurring during Years 1 or 2 (n = 20, 76 %). Unprofessional behavior represented the most common reason for referral in Years 3–5. Remedial students did not differ from classmates (n = 151) on baseline characteristics (Age, Gender, US citizenship, MCAT) or willingness to recommend their medical school to future students ($p < 0.05$). Two remedial students did not graduate and three did

For peer review only

No.	Title	Author/year	MERSQI	COREQ	Reason for inclusion			Undergraduate (UG)/Postgraduate (PG)	Methodology	Purpose of study	Key findings
					Communication	Ethics	Professionalism				
									to their classmates not placed in remediation on variables collected for program evaluation purposes: Baseline characteristics (gender, age, US citizenship, MCAT score), USMLE licensure exam performance (pass-rate for each student), National Residency Matching Program data (clinical specialty for each student), Satisfaction with medical school (collected from graduating students using questionnaire). Then, we read MSPRC correspondence to each remedial student to extract referral mechanism (portfolio-based decision or faculty-initiated), unsatisfactory competency domain(s), remediation duration (days), and completion of students' remediation goals. Finally, we read students' remedial plans to identify strategies they proposed to	remediation and then report outcomes for students who were and were not placed in remediation during medical school. We conclude with lessons learned and future directions.	not pass USLME licensure exams on first attempt. Most remedial students (92 %) generated appropriate plans to address performance deficits. Students can successfully design remedial interventions. This learner-driven remediation model promotes greater autonomy and reinforces self-regulated learning. From results: Participants include 177 of 187 medical students from six class cohorts (2009–2014) who consented (95 %) to release program evaluation data for research purposes. Of these, the MSPRC placed 26 students (17 men and nine women) in formal remediation. Half (13) were identified by the MSPRC for not meeting year-end performance standards assessed via portfolios. The dean of student affairs referred the remaining 13 students based on faculty recommendation. We did not detect significant differences between remedial students and their classmates for baseline characteristics (Table 1). Overall, three-quarters of remediation decisions occurred during the first (10, 38 %) or second (10, 38 %) year of medical school. With regard to specific domains in which students failed to meet year-end standards or were otherwise referred, professionalism (18, 69 %), communication (10, 38 %), research (9, 35 %), and reflective practice (5, 19 %) represent frequent domains of unsatisfactory performance. Fewer students did not meet

For peer review only

No.	Title	Author/year	MERSQI	COREQ	Reason for inclusion			Undergraduate (UG)/Postgraduate (PG)	Methodology	Purpose of study	Key findings
					Communication	Ethics	Professionalism				
									the MSPRC to address performance deficits in specific competency areas. We manually recorded a subset of these strategies to provide examples of students' approaches to remediation. All performance decisions, referral mechanisms, and outcomes were manually entered into SPSS. Chi-square and ANOVA tests were used to compare baseline characteristics and satisfaction scores of the two student groups (remediation vs. other), while the Mann Whitney U test was used to examine if remediation duration (days) differed by students' gender. Statistical significance was established at $p < 0.05$ for all comparisons.		performance standards for clinical reasoning (4, 15 %), medical knowledge (3, 11 %), clinical skills (2, 8 %), personal development (2, 8 %), or health care systems (2, 8 %). Unprofessional behavior (e.g., not logging patient encounters, unexcused absences, chronic tardiness, etc.) represented the most common reason for student remediation during Years 3–5 of medical school. Ten students had deficient performance in one competency, while others had unsatisfactory performance in two (7, 27 %) or more (9, 35 %) competencies. Three students were placed in remediation twice, with one repeating for unprofessional behavior. Time in remediation (determined by dates in MSPRC correspondence to students) ranged from 39 to 1696 days (Median = 448 days) and did not differ by student gender ($p > 0.05$). Remediation plans revealed various student-proposed strategies to address TAFIs for multiple competency domains (Table 2). We observed that students' remedial strategies resembled approaches faculty would recommend. We also observed the MSRPC's correspondence to students consistently had an encouraging, supportive tone for students' remedial efforts (See Appendices for MSPRC's correspondence to one remedial student where committee asks student to develop a remediation plan, comments

No.	Title	Author/year	MERSQI	COREQ	Reason for inclusion			Undergraduate (UG)/Postgraduate (PG)	Methodology	Purpose of study	Key findings
					Communication	Ethics	Professionalism				
											on student's progress report, and releases student from remediation). The MSPRC "prescribed" remediation plans for two students only after these students did not provide adequate assessment evidence in progress reports. The MSPRC determined that most students (24, 92 %) generated sufficient evidence (predominately obtained from faculty and peers) to document their competence. Table 1 reveals that some remedial students experienced negative outcomes, as two did not graduate and three did not pass USMLE licensure exams on first attempt. Proportionally, more remedial students (11, 46 %) pursued surgical specialties than their classmates (50, 33 %). Table 1 also shows graduating remedial students were just as likely as their classmates to recommend the CCLCM program to future students, even though fewer were as satisfied with their overall experience at CCLCM as their classmates. From discussion -- Most programs struggle with how to identify and assist learners requiring remediation. We explored if students, rather than faculty, can take ownership for remedial plan design and implementation. We review key findings and conclude with future directions. The locus of control for selecting remedial interventions and assessment evidence typically rests with faculty. In

For peer review only

No.	Title	Author/year	MERSQI	COREQ	Reason for inclusion			Undergraduate (UG)/Postgraduate (PG)	Methodology	Purpose of study	Key findings
					Communication	Ethics	Professionalism				
											our model, the MSPRC charged students to develop remediation plans, with PA guidance as needed, and obtain assessment evidence to document progress and achievement. Most remediating students (92 %) successfully met these expectations and graduated from medical school. Three-quarters of remedial students were identified in Years 1–2 of medical school. Early identification provides opportunities for multiple, tailored interventions to occur. Professionalism, communication skills, and research performance were frequent reasons the MSPRC placed students in remediation. Few students were identified for gaps in medical knowledge or clinical reasoning. The literature suggests some competencies, like professionalism, require direct observation and ongoing assessment for learner success.1,18 Our students' remedial strategies (Table 2) ranged from specific to holistic approaches and often cut across courses and contexts, thereby providing them with more opportunities to improve and document their performance in complex domains. This emphasis on competence may explain why students' time in remediation was longer (Median = 448 days) than normally needed for "more of the same," such as retaking a test or course.2 Some learners may not know how to navigate performance obstacles,

For peer review only

No.	Title	Author/year	MERSQI	COREQ	Reason for inclusion			Undergraduate (UG)/Postgraduate (PG)	Methodology	Purpose of study	Key findings
					Communication	Ethics	Professionalism				
											making it important to offer guidance and oversight.19,20 For this reason, we train a core group of faculty to help students apply the self-regulation cycle.17 PAs coach students in reflection, self-assessment, and goal-setting.2,20,21 They also form long-term relationships with students and frequently serve as student advocates. The MSPRC encourages students to identify measurable outcomes to document competence, while offering positive support in written correspondence to students (See Appendices for series of letters to one student throughout the remediation process). Both faculty groups work in tandem to support this learner-driven remediation model while ensuring student accountability. Our evaluation is limited to one program, at one school, with a small class size. Other programs may foster remedial learners' autonomy more than conveyed in the literature.5,6,8 Our student satisfaction measures originate from two questionnaire items obtained at graduation and do not capture students' satisfaction with specific academic referral policies or remedial processes.22 Remediation is complex and involves multiple stakeholders and contexts. Future studies should capture learners' perceptions of remediation to complement the faculty point-of-view currently reported in the literature.

For peer review only

No.	Title	Author/year	MERSQI	COREQ	Reason for inclusion			Undergraduate (UG)/Postgraduate (PG)	Methodology	Purpose of study	Key findings
					Communication	Ethics	Professionalism				
											We discovered that more men than women were identified for remediation, yet we found no research exploring this observation in depth. Additionally, few longitudinal or multi-institutional studies currently exist, suggesting an area ripe for research. We discovered, in our 10-year experience with this remediation model, that medical students can select appropriate remedial interventions with the support of well-trained faculty. Our learner-driven approach to remediation may transfer to graduate medical education (GME), where Clinical Competence Committees (CCCs) meet regularly to discuss and review resident performance. Having struggling residents create remediation plans and submit progress reports to CCCs may present few barriers, given GME's competency-based assessment framework and emphasis on providing residents with frequent, formative feedback. Our approach requires a learning environment where faculty must trust students have the motivation and capabilities to identify and implement appropriate remedial strategies. We have created a systematic process where students must reflect on their performance, develop plans tailored to meet learning needs, and collect assessment evidence to document competence. To conclude, we believe educators who wish to

For peer review only

No.	Title	Author/year	MERSQI	COREQ	Reason for inclusion			Undergraduate (UG)/Postgraduate (PG)	Methodology	Purpose of study	Key findings
					Communication	Ethics	Professionalism				
											adopt a learner-driven approach to remediation should view remediation as a growth opportunity rather than as a punishment.
64	Medical professionalism: conflicting values for tomorrow's doctors	Borgstrom, E., Cohn, S. and Barclay, S.<br><br>2010		14		✓		UG	From abstract: Qualitative study using interpretative discourse analysis of anonymized student reflective portfolios. One hundred twenty-three final year undergraduate medical students (64 male and 59 female) from the University of Cambridge School of Clinical Medicine supplied 116 portfolios from general practice and 118 from hospital settings about patients receiving palliative or end of life care.<br>From introduction: This study investigates how professionalism is understood and experienced via one such initiative for medical students training in Cambridge, England, and highlights the conflicts between 'new' and 'old' values.<br>From methods: Final year medical students in Cambridge meet patients approaching the	To investigate how final year medical students experience and interpret new values of professionalism as they emerge in relation to confronting dying patients and as they potentially conflict with older values that emerge through hidden dimensions of the curriculum.	From abstract -- Results: Professional values were prevalent in all the portfolios. Students emphasised patient-centered, holistic care, synonymous with a more contemporary idea of professionalism, in conjunction with values associated with the 'old' model of professionalism that had not be directly taught to them. Integrating 'new' professional values was at times problematic. Three main areas of potential conflict were identified: ethical considerations, doctor-patient interaction and subjective boundaries. Students explicitly and implicitly discussed several tensions and described strategies to resolve them. Conclusions: The conflicts outlined arise from the mix of values associated with different models of professionalism. Analysis indicates that 'new' models are not simply replacing existing elements. Whilst this analysis is of accounts from students within one UK medical school, the experience of conflict between different notions of professionalism and the three broad domains in which this conflict arises are relevant in other areas of medicine and in different national contexts.<br>From discussion -- All of the students encountered challenges that required

No.	Title	Author/year	MERSQI	COREQ	Reason for inclusion			Undergraduate (UG)/Postgraduate (PG)	Methodology	Purpose of study	Key findings
					Communication	Ethics	Professionalism				
									end of life during general practice (GP) and hospital attachments. As required coursework they write portfolio items on two patients, one from each attachment. Key to this exercise is integrating issues arising from the interview with reflections of their own personal experiences. This study focuses on how different ideas are received and reproduced by a relatively young (typically early 20s) cohort of soon-to-be qualified doctors during the academic year 2007–2008. The University of Cambridge Psychology Research Ethics committee approved the study, and 86% of students (n = 123; 64 male, 59 female) gave informed consent for 234 reflective items: 116 from the GP and 118 from hospital settings. Each student was assigned a random identification number (ID), which is included		them to try and balance values characteristics of 'old' and 'new' forms of medical professionalism. Ethics, interaction and managing subjective boundaries all generated areas of conflict that they wrestled with in their assignments. Students addressed these tensions in a variety of ways, with strategies drawn from both formally taught skills and personal resources. In parallel with shifts from 'old' to 'new' values of professionalism, conflicts between the formal and the hidden curricula may also generate tensions44. In our study, the formal curricula is explicitly designed to embrace the 'new', leaving the 'old' to be communicated through more informal and hidden aspects of teaching. Yet for the students trying to personally reflect on their experiences and summatively draw from their entire educational experience for the exercise, no distinction is made between these two domains. Large organisations like the University of Cambridge Clinical School and the National Health Service have institutional memories45—collective experiences and concepts. These are communicated imperceptively through teaching elements as well as the very structures and policies that shape the educational environment. This feature serves to complicate understandings of medical education; it

For peer review only

No.	Title	Author/year	MERSQI	COREQ	Reason for inclusion			Undergraduate (UG)/Postgraduate (PG)	Methodology	Purpose of study	Key findings
					Communication	Ethics	Professionalism				
									with the quotes to demonstrate the breadth of the dataset presented. Analysis focused on the topic of 'professionalism', viewing the content of the portfolios as representative of general values imparted to students throughout their education. All items were coded and analysed in NVivo 8 using an interpretative approach. Following the social science traditions of two of the authors (EB and SC), the methodology employed a hermeneutic approach, striving to find meaning beyond straightforward discrete references35. Accordingly, rather than assuming the meaning of isolated sections of text in a reductive manner, they were always analysed in context and in relation to one another. Given the difficulty many students had in addressing the topic of end of life care, and		emphasises the extent to which values are embedded in the routines and practices of the organisation, and cannot therefore be simply addressed through redesigning curricula or individuals championing change. Our study suggests that tensions arise primarily because of different values that underlie the concept of professionalism throughout their education, rather than anything that is specific to end of life care. Whilst topics central to the everyday practice of medicine are particularly evident in palliative care46, they are made highly visible by students who see themselves on the cusp of becoming members of the profession themselves. Whilst it is likely that the concept of professionalism always has contained a wide variety of underlying values and principles that are not always commensurate with each other, we have argued that a more widespread shift in values over recent years has generated greater variance and hence more contradictory positions around what it means to be a good doctor. Our findings add to the literature on medical professionalism, which is rich in doctors' anecdotal experiences47,48, highlights the stresses and conflicts doctors face44,49,50 and illustrates a current amalgamation of definitions with conflicting values51. Specific national and local contexts are likely to generate differently

For peer review only

No.	Title	Author/year	MERSQI	COREQ	Reason for inclusion			Undergraduate (UG)/Postgraduate (PG)	Methodology	Purpose of study	Key findings
					Communication	Ethics	Professionalism				
									consequently the very discursive descriptions of their observations, the authors were committed to code more than just those phrases in which a theme was referred to explicitly, and so included concerns that were expressed less directly. A coding scheme was derived from initial independent item analysis (EB, SC and SB) and adjusted through discussion. It was initially applicable to explicit statements by drawing on key words and then was augmented to include the more implicit references that potentially alluded more to the hidden dimensions of their teaching (see Box 2). The inclusion of these required careful interpretative readings of the entire text but provided an essential contribution to our overall analysis. The first author (193) then led on-going analysis, with regular meetings to		nuanced versions of these issues, which might only be identified through comparative work. From conclusion: The integration of 'new' professional values taught in medical school is at times problematic for students in any health care system that maintains elements, whether overtly or not, of the 'old' paradigm. The areas of potential conflicts outlined—ethics, patient interaction and managing subjective boundaries—are not limited to medical school or end of life care. Our analysis suggests that 'old' variants of professionalism are not simply being replaced by 'new' ones delivered by a redesigned curriculum, but rather that values from each can emerge in a range of medical contexts. As a result, professionalism does not consist of a set of fixed or abstract concepts, but rather surfaces through medical practice. If individual reflection is now heralded as an essential component of the 'new' professionalism, as indicated by the compulsory student exercise we have drawn on here, it should be acknowledged that it demands a dynamic engagement with the wide range of often contradictory and shifting ideas and beliefs from both formal and more hidden aspects of their education. This study illustrates that overt commitment to more empathic and patient-centered approaches to medical care do not

For peer review only

No.	Title	Author/year	MERSQI	COREQ	Reason for inclusion			Undergraduate (UG)/Postgraduate (PG)	Methodology	Purpose of study	Key findings
					Communication	Ethics	Professionalism				
									discuss emerging themes.		necessarily replace other more prescribed values and behaviours that remain part of a hidden curriculum embedded in institutional practices. Integration of 'new' core values and skills into good medical practice is not a smooth or simple transition. Additionally, it seems any simple attempt to communicate them through formal teaching is unlikely to prepare students for the reality of medical encounters. Instead, the experience of tension and the individual desire to seek balance and resolution across a wide range of issues may themselves be key and lasting features of medical professionalism.s
65	Facilitating students' reflective practice in a medical course: literature review	Chaffey, L. J., de Leeuw, E. J. and Finnigan, G. A. 2012		12			✓	UG	The literature search was undertaken using the online databases of CINAHL and Medline. Keyword searching was conducted using MeSH headings, Boolean operators, and the terms, 'medical students', 'medical education', 'reflection', 'reflect*' and 'medicine'. The search was limited to peer-reviewed published material in English and between the years 2001 and 2011 to maintain currency of	The aim of this review is to summarise the literature published around facilitating reflection in a medical course, and to answer the question : What is the current evidence regarding learning and development moments across the medical curriculum in developing students' reflective practice?	From abstract -- Discussion: A variety of reflective purposes was found in this literature review. Evidence indicates that, if students are unclear as to the purpose of reflection and do not see educators modelling reflective behaviours, they are likely to undervalue this important skill regardless of the associated learning and development opportunities embedded in the curriculum. From results: Thirty-six relevant articles were found. These articles were classified by methodology (see [Table 1]. Relevant references identified). As can be seen in [Table 1], this review included many opinion pieces, reviews and descriptions of methods of facilitating reflection, but little in regards to primary data in the area. Qualitative methodology was the main research output, providing largely descriptive data of

For peer review only

No.	Title	Author/year	MERSQI	COREQ	Reason for inclusion			Undergraduate (UG)/Postgraduate (PG)	Methodology	Purpose of study	Key findings
					Communication	Ethics	Professionalism				
									practice. The primary search yielded 129 references of eligible papers, including research papers, reviews, formal reviews and opinion pieces. Abstracts were read by the first author, and only articles pertaining to facilitating or teaching reflection in undergraduate or postgraduate medical courses were included. The first author read and classed all included articles, categorising them into construct sub-categories. At several stages in this process all authors reviewed the validity and legitimacy of the classifications. This led to the sub-headings and analysis presented below.		the facilitation of reflection. The quantitative research was also largely descriptive, with survey methodology being the most popular in the articles found. This review found only two case-control studies comparing facilitation methods of reflection. The literature presented enhancing factors and barriers to effectively teaching reflective practice within medical curricula, related to: The breadth of the meaning of reflection; Facilitating reflection by medical educators; Using written or web-based portfolios to facilitate reflection; and Assessing the reflective work of students.<br>- The breadth of the meaning of reflection: The term 'reflection' is widely mentioned within medical education literature, but the purposes and goals described indicate a breadth of purposes of reflection.[14] Mann et al.[10] concluded from their systematic review of reflective practice in the health professions that the nature of reflection makes it difficult to quantify. From this current review, we determined that the breadth of reflection covers the purposes of reflecting to improve judgements, to personally develop and, and to contextualise practice, and are explained in [Table 2]. Reflection to improve judgement centres on improving students' clinical skills, problem solving, [11] flexible thinking, lifelong learning and the development of expertise.

For peer review only

No.	Title	Author/year	MERSQI	COREQ	Reason for inclusion			Undergraduate (UG)/Postgraduate (PG)	Methodology	Purpose of study	Key findings
					Communication	Ethics	Professionalism				
											[1],[10],[14],[17] Reflection for this purpose usually has a problem focus, involves critical thinking, and is commonly indicated by hypothesis testing. Reflection to personally develop is essential in client-centred practice, as doctors need to consider they own values and assumptions, and those of the patient, while making clinical decisions. [18] From reviewing the literature, Sandars [14] asserted that the knowledge of one's values and beliefs is important for a doctor to develop therapeutic relationships with patients, as understanding oneself enables empathy and caring. Aukes et al.[11] reviewed literature in preparation for creating an assessment tool for reflection, and noted that personal reflection has often been overlooked in medical education. They suggested that this has occurred as personal development and has been seen as outside the domain of the problem-solving culture of medical practice, and thus, outside the domain of medical education. Reflection to contextualise practice helps students integrate theory with their own experience. Several authors have hypothesised that this aspect of reflection is essential in developing a sound medical practice, [5] with a lack of reflective ability resulting in poor self-awareness, and thus poor medical practice. [19] Howe et al.[8] found, from their study of the development of

For peer review only

No.	Title	Author/year	MERSQI	COREQ	Reason for inclusion			Undergraduate (UG)/Postgraduate (PG)	Methodology	Purpose of study	Key findings
					Communication	Ethics	Professionalism				
											professionalism, that reflecting on practice and developing self-awareness are important mechanisms of resilience in the face of a demanding profession. Although this breadth of reflective practice makes it an essential skill in medical practice, this very strength can also be a barrier in teaching facilitating reflective practice as there is often ambiguity of the educator's goals in regard to the use of a particular reflective task. [20],[21] Aukes et al.[11] noted that purposes of a reflective tasks were rarely articulated, and students were often unaware of the perspective, reflective purpose or role, they were expected to take. Sanders [14] concluded from a literature review of reflection in medical education that, for students to reflect effectively, they required clarity in determining the appropriate purpose of a reflective task.<br>- Facilitation reflection by medical educators: Reflection is often provoked by uncertainty or unease resulting from an experience. [13],[22] According to Albanese, [22] in addition to this uncertainty or unease, a student requires knowledge of how to reflect, and time and motivation for reflection to occur. Supporting this opinion, Carr and Carmody [23] found in their qualitative study that some students could attain an acceptable level of reflection independently. However, reflection was greatly

No.	Title	Author/year	MERSQI	COREQ	Reason for inclusion			Undergraduate (UG)/Postgraduate (PG)	Methodology	Purpose of study	Key findings
					Communication	Ethics	Professionalism				
											enhanced through medical educators' facilitation. Medical educators' facilitation was found to be useful for reflection in one-on-one meetings with students and in small group tutorials. [9],[24] According to results of a study by Schaub-de Jong et al., [9] educators who wished to encourage reflection needed to model reflective practice by making their experiences more explicit, investigating emotions and stimulating interaction among students. Albanese [22] suggested that medical faculty staff should be trained to articulate their reflections to enhance modelling to students. - Using written or web-based portfolios to facilitate reflection: Traditionally, reflective tasks have been paper based. Recently, web-based methods and social media have been adopted to facilitate reflective skills. Cook [29] asserted that there has been little study of the efficacy of these methods. Fischer et al. [30] compared content, depth of reflection and student preference for web-based versus traditional essay-style reflections in two medical schools in the USA. Ninety-five students were quasi-randomly assigned to one of two study groups, where one group wrote a traditional reflective essay and attended a small-group discussion, while the other group posted two writings in a group blog and commented on a peer's post. The writings were

For peer review only

No.	Title	Author/year	MERSQI	COREQ	Reason for inclusion			Undergraduate (UG)/Postgraduate (PG)	Methodology	Purpose of study	Key findings
					Communication	Ethics	Professionalism				
											coded for theme, and level of reflection, with the authors finding no difference between groups for either variable. From discussion: Traditionally, paper-based methods of reflection have been popular with educators, [7],[8],[25] but recently, web-based methods have been adopted. Driessen et al. [36] found that the method of reflection appears to be less important than the guidelines provided, which should be structured initially, and become more fluid as students' reflective skills progress. Ideally, students should be able to revisit and edit the reflection, hence web-based methods may be suitable for this purpose. - Assessing the reflective work of students: ... (see relevant columns) From discussion: Several authors noted that reflection was an essential skill in medical practice, [2],[5],[7],[10],[14],[34] and advocated for training in reflective processes. [13],[22] Training in reflective practice can be significantly enhanced with the facilitation of skilled mentors, providing guidance and modelling of reflective behaviours, as found by Carr and Carmody. [23] Several studies and literature reviews report inconsistencies regarding the purpose of reflection, which was found to be a significant barrier to the facilitation of reflective practice. [11],[20],[21],[35] The implication for educators is that students

No.	Title	Author/year	MERSQI	COREQ	Reason for inclusion			Undergraduate (UG)/Postgraduate (PG)	Methodology	Purpose of study	Key findings
					Communication	Ethics	Professionalism				
											need to be given clear guidelines as to the purpose for each individual reflective task. Traditionally, paper-based methods of reflection have been popular with educators, [7],[8],[25] but recently, web-based methods have been adopted. Driessen et al. [36] found that the method of reflection appears to be less important than the guidelines provided, which should be structured initially, and become more fluid as students' reflective skills progress. Ideally, students should be able to revisit and edit the reflection, hence web-based methods may be suitable for this purpose. Students need to have the designated time and motivation for reflective practice. A common method of increasing task motivation in the education setting is to include the task as an assessment piece. There was some debate in the literature about the efficacy of this method. Some authors found that students' quality of reflection would be diminished if undertaken for an assessment task, [28] whereas others reported that reflection simply would not happen if not attached to an assessable piece of work. [22],[33] It is apparent from this review that the research surrounding the teaching and facilitation of reflection is generally weak. Although there are some descriptive data, there is very little empirical evidence of the efficacy of facilitation methods. Additionally, there

For peer review only

No.	Title	Author/year	MERSQI	COREQ	Reason for inclusion			Undergraduate (UG)/Postgraduate (PG)	Methodology	Purpose of study	Key findings
					Communication	Ethics	Professionalism				
											is a lack of rigorous research investigating the impact of reflection on practice skills or patient outcomes. A major limitation of literature is the breadth of the construct of reflection. A more defined construct of reflection, with clear outcomes, could lead to the development of benchmarks useful in tracking student progress and as research outcome measures. From conclusion: Most authors cited in this review suggested that the emphasis on reflection is increasing in medical curricula. The construct of reflection was broad in the reviewed literature. This suggests that medical educators must be clear on the purpose of reflection, the intended outcome for the individual student and the process necessary to achieve these aims for students. Evidence indicated that, if students are unclear as to the purpose of reflection and do not see educators modelling reflective behaviours, they are likely to undervalue this skill regardless of the associated learning and development opportunities embedded in the curriculum.[40]
66	Beyond assessment of learning toward assessment for learning: educating tomorrow's physicians	Dannefer, E. F. 2013	-	-	✓			UG	From abstract: Information-rich assessment data, structured opportunities for reflection, and facilitated self-assessment using a portfolio approach are designed to support	From abstract: We describe a program (based at Cleveland Clinic Lerner College of Medicine) in which explicit performance standards align these components and provide a roadmap for students to manage their learning.	From abstract: Preliminary evidence suggests that the program directs students towards learning, rather than on achieving a grade for grade's sake.

No.	Title	Author/year	MERSQI	COREQ	Reason for inclusion			Undergraduate (UG)/Postgraduate (PG)	Methodology	Purpose of study	Key findings
					Communication	Ethics	Professionalism				
									development of habits of reflective practice. Promotion depends on the achievement of competencies rather than grades. From the essential building blocks: All components and processes of a system need to be designed to interact in a way that accomplishes the goal of enhancing learning and habits of reflective practice. Key features of the CCLCM assessment system are now presented by addressing issues of competency standards, ongoing formative assessment, student responsibility, and purposeful support.		
67	Evidence within a portfolio-based assessment program: what do medical students select to document their performance?	Dannefer, E. F., Bierer, S. B. and Gladding, S. P. 2012	7		✓		✓	UG	From abstract: We reviewed portfolios to examine the number, type, and source of assessments selected by students (n = 32) to document their performance in seven competencies. The quality of assessment data selected for each	From abstract: In this article, we examine the nature of evidence selected by first-year medical students to include in a portfolio used to make promotion decisions. From introduction: This article attempts to address this gap in the literature by examining the types and sources of assessment	From abstract -- Results: Findings indicate that students cited multiple types and sources of available assessments. The promotion committee rated evidence quality highest for competencies where the program provided sufficient evidence for students to cite a broad range of assessments. When assessments were not provided by the program, students cited self-generated evidence.

For peer review only - http://bmjopen.bmj.com/site/about/guidelines.xhtml

No.	Title	Author/year	MERSQI	COREQ	Reason for inclusion			Undergraduate (UG)/Postgraduate (PG)	Methodology	Purpose of study	Key findings
					Communication	Ethics	Professionalism				
									competency was rated by promotion committee members (n = 14). From methods: We used two procedures to explore the assessment evidence that one cohort of medical students cited in their Year 1 summative portfolios. First, we examined the evidence cited in portfolio essays to ascertain what types and sources were selected. Second, we obtained feedback from promotion committee members concerning the quality of evidence via a questionnaire. - Citation coding procedures and data: A research assistant extracted essays from the e-Portfolio database for seven of the nine competencies included in the Year 1 summative portfolios that students submitted during the 2009–2010 academic year. Essays for the competencies of Personal Development and	evidence first-year medical students select from their performance database to construct portfolios used for promotion decisions. We describe our assessment program's evidence database before presenting the organizational elements designed to help students purposefully select data representative of their performance. We then describe the methods and results of the study before discussing implications about students' ability to select evidence within a centralized assessment program.	Conclusion: We found that when student-constructed portfolios are part of an overall assessment system, students generally select evidence in proportion to the number and types of assessments available.

For peer review only

No.	Title	Author/year	MERSQI	COREQ	Reason for inclusion			Undergraduate (UG)/Postgraduate (PG)	Methodology	Purpose of study	Key findings
					Communication	Ethics	Professionalism				
									Reflective Practice were excluded to ensure student confidentiality. Students' names on essays were replaced with a unique six-digit identifier. Overall, 224 essays for 32 students were reviewed for these seven competencies. Sophia Gladding sorted the essays by competency. She then read each set of essays nested within a given competency and manually recorded on a data extraction form all college-generated assessments, work samples and personal evidence students cited to document their performance of Year 1 standards. Extraction form data were entered into SPSS version 16.0, and descriptive statistics were computed after averaging across students' portfolios.<br>- Quality ratings: Promotions committee members involved with portfolio reviews (n = 18 faculty)		

For peer review only

No.	Title	Author/year	MERSQI	COREQ	Reason for inclusion			Undergraduate (UG)/Postgraduate (PG)	Methodology	Purpose of study	Key findings
					Communication	Ethics	Professionalism				
									completed a 21-item, web-based questionnaire after reading students' Year 1 summative portfolios. The questionnaire had been designed (Beth Bierer and Elaine Dannefer) and piloted earlier to monitor the Year 1 portfolio review process. This instrument included seven Likert-scaled items ranging from 1 "poor" to 4 "excellent" which asked promotion committee members to rate the overall quality of the assessment evidence they read for each competency after judging students' summative portfolios. Percentages were computed for each competency to summarize the committee's global ratings ("excellent" and "good/excellent") of evidence quality.		
68	The portfolio approach to competency-based assessment at the Cleveland Clinic Lerner College of Medicine	Dannefer, E. F. and Henson, L. C. 2007	-	-	✓		✓	UG	-	From abstract: This article describes how the portfolio was developed to provide both formative and summative assessment of student achievement in relation to the program's nine	Design of the portfolio assessment system: The search for an assessment system consistent with our principles led us to the literature on portfolios for medical student assessment14 –15,21–23 and to firsthand investigation of experiences

For peer review only - http://bmjopen.bmj.com/site/about/guidelines.xhtml

No.	Title	Author/year	MERSQI	COREQ	Reason for inclusion			Undergraduate (UG)/Postgraduate (PG)	Methodology	Purpose of study	Key findings
					Communication	Ethics	Professionalism				
										competencies.<br>From introduction: In this article we contribute to the literature on competency-based assessment by describing a portfolio assessment system developed as an integral component of the new Cleveland Clinic Lerner College of Medicine (CCLCM) of Case Western Reserve University<br>In this article, we describe the design of CCLCM's portfolio approach for a comprehensive, competency-based assessment system that is fully integrated with the curriculum. To provide context, we describe the program's competencies, performance standards, core assessment principles, and unique assessment instrument before addressing the design of the overall assessment system.	with portfolio assessment within and beyond national, disciplinary, and professional borders. At the time (2002– 2003), the portfolio assessment approach was being implemented in the United States in grades K–12 and at the undergraduate college level24 –26 and in the United Kingdom and Europe at a number of medical schools. For example, at the University of Dundee College of Medicine, as a key component of the final examination process, each final-year medical student was required to submit a portfolio documenting his or her progress in achieving the outcomes of the curriculum.14,15 The first-year medical student portfolio at the University of Maastricht Faculty of Medicine included students' reflective essays on their own development in four identified roles of the doctor as a method of encouraging integration of competencies.22<br>An Answer to the Challenges of Competency-Based Assessment: In our opinion, this system and its components should be applied in other settings across the medical education continuum because major themes in the current debate about assessment in medical education, such as integration across competencies, competency-based assessment methods, sampling from multiple contexts and multiple sources,

For peer review only

No.	Title	Author/year	MERSQI	COREQ	Reason for inclusion			Undergraduate (UG)/Postgraduate (PG)	Methodology	Purpose of study	Key findings
					Communication	Ethics	Professionalism				
											triangulation of information, and training of learners in reflective practice are addressed in this innovative method of student assessment.
69	Supporting students in self-regulation: use of formative feedback and portfolios in a problem-based learning setting	Dannefer, E. F. and Prayson, R. A.<br><br>2013		11	✓		✓	UG	From abstract: Formative feedback on works habits and interpersonal skills provided by peers and tutors to a Year 1 cohort (n ¼ 32) over the course of a year-long PBL experience (5 blocks) was examined for comments on targeted areas for improvement (TAFIs) and observed improvements. We examined congruence between PBL feedback and students' self-reported TAFIs and behavioral improvements in their assessment portfolios.<br>From study design and methods:<br>- Study design: This study presents an analysis of (1) narrative feedback on professionalism provided to Year 1 medical students from PBL peers, tutors, and self and (2) summative portfolio essays to	From abstract: This study examines the extent to which students self-regulate professionalism behaviors related to work habits and interpersonal skills in a PBL setting.<br>From introduction: In 2004, Cleveland Clinic Lerner College of Medicine (CCLCM) implemented a program designed specifically to promote the development of self-regulated learners (Dannefer & Henson 2007). ... We confront the question of whether or not students use CCLCM's learning environment to self-regulate performance by focusing on the core competency of professionalism.<br>We selected for this study of SR the content of PBL feedback on professionalism and its use by students to identify learning needs (self-assess) and document improvement (regulate). We asked these questions:<br>(1) To what extent did the content of peer and tutor PBL	From abstract -- Results: Both PBL peer and faculty feedback and portfolio self-assessments targeted Interpersonal Skills TAFIs more frequently than Work Habit-related issues. TAFIs were more frequently identified midway in PBL blocks versus the end. Students reported TAFIs in their portfolio essays, citing feedback from both peers and tutors, and provided evidence of improved performance over time.; Conclusions: Students utilized external formative feedback to document their portfolio self-assessment in a system designed to support self-regulation of PBL professionalism-related behaviors. A decrease in TAFIs identified at the end of PBL blocks suggests students made use of mid-block feedback to self-regulate behaviors.<br>From results:<br>- (1) To what extent did the content of peer and tutor PBL narrative feedback provide information relevant to the professionalism competency standards? -- Table 1 summarizes the content of aggregate PBL tutor and peer feedback as well as PBL self and portfolio self- assessments. Of all TAFIs identified by tutors, Interpersonal Skills account for 63%. Interpersonal Skills also dominated peer statements (57%). The majority of tutor

For peer review only

No.	Title	Author/year	MERSQI	COREQ	Reason for inclusion			Undergraduate (UG)/Postgraduate (PG)	Methodology	Purpose of study	Key findings
					Communication	Ethics	Professionalism				
									determine how students used PBL feedback to support their self-assessments. We limited the study to year one the time period reported by students, and supported by advisors' observations, as requiring significant adjustments in terms of taking responsibility for their own learning.<br>- Measures: Faculty defined measurable, behavioral competency standards developmentally appropriate for Year 1 students and observ- able across multiple settings. ... Self-regulation, as a process, consists of overlapping and recursive activities of which we targeted self-assessments that identified areas needing improvement and cited evidence that documented improved performance.<br>- Data sources: We collected peer and tutor PBL assessments electronically at mid and end of Block 1 problem-	narrative feedback provide information relevant to the professionalism competency standards?<br>(2) To what extent did students self-report and document professionalism learning needs and improvements consistent with peer and tutor PBL feedback in a portfolio used for promotion decisions?	and peer TAFI statements about Interpersonal Skills indicated a concern with ''quietness'' (e.g. lack of participation). In the area of Work Habits, peers more frequently identified ''preparedness'' (e.g. disorganized presentations) as a TAFI in fellow students as compared with tutors, who more frequently documented behaviors associated with being ''distracted'' (e.g. carrying on side conversations). Peers were also more likely to give feedback on TAFIs than tutors; of 2016 peer assessments, 41% contained feedback on TAFIs. Overall, tutor feedback tended to be less detailed and only 28% of the 288 possible assessments contained feedback on TAFIs. Similar to tutor and peer evaluations, students self- identified Interpersonal Skills issues as TAFIs more often than Work Habit-related issues (60% vs. 40% respectively). Also similar to peer assessments, students frequently self-identified ''ill-preparedness'' as the Work Habit most needing improvement. In contrast to peer and tutor feedback, however, students more frequently self-identified ''dominating'' behavior issues rather than ''quietness''. All students, except for one who did not complete self-assessments, self-identified TAFIs also reported by their peers and tutors. Interestingly, 95% of students at some point during the year also self-identified issues not documented by peers and

No.	Title	Author/year	MERSQI	COREQ	Reason for inclusion			Undergraduate (UG)/Postgraduate (PG)	Methodology	Purpose of study	Key findings
					Communication	Ethics	Professionalism				
									solving sessions, and from peers, tutors and self at mid and end of Blocks 2-4 and end of Block 5 case-based sessions. Because professionalism cannot be compartmen- talized, students address standards not only in the Professionalism Competency essay but also in the Communication, Personal Development, and Reflective Practice competency essays. Thus, data used in this study included essays and citations for these four competencies. - Coding and analysis of PBL narrative feedback: A trained research assistant extracted the electronic competency report of all PBL feedback for professionalism for all first-year students (n ¼ 32) for the 2009 academic year, and replaced students' names with a unique six-digit identifier. We		tutors. Table 1 shows that peer and tutor feedback and self-assessments documented improvements less frequently than TAFIs, and statements noting improvement in Interpersonal Skills were more fre- quent than statements of improvement in Work Habits. Figure 1 documents peer and tutor feedback statements for students at mid and end of the four PBL blocks. In aggregate, more TAFI statements were made by peers and tutors for mid-block assessments than at the end of the block with an overall decrease across the year. Regarding individual student data, 29 of the 32 students had more TAFIs documented at mid-block by peers as compared with end-of-block, and early in the year (Blocks 1–2) versus later in the year (Blocks 3–4). Similarly, more evidence with regard to improvements was documented for students by peers in end-of-block and late year feedback versus mid block and early year feedback, respectively. Similar trends were noted in the tutor feedback for mid- and end-of- block TAFIs and improvement statements. - (2) To what extent did students self-report and document professionalism learning needs and improvements consistent with peer and tutor PBL feedback in a portfolio used for promotion decisions? -- Student self-assessments presented in their summative portfolio essays are required to be a

No.	Title	Author/year	MERSQI	COREQ	Reason for inclusion			Undergraduate (UG)/Postgraduate (PG)	Methodology	Purpose of study	Key findings
					Communication	Ethics	Professionalism				
									developed a rubric using the PBL assessment form criteria for Interpersonal Skills and Work Habits to individually code five competency reports. The authors then met and reached consensus on coded professionalism statements about TAFIs and observed improvements. The refined Professionalism Rubric has three unprofessional behavior criteria each for Interpersonal Skills (Dominating, Quiet, Inappropriate) and for Work Habits (Late, Ill-prepared, Distracted). Each author coded the remaining reports before meeting to reach consen-sus and in rare instances of coding differences; these discrepancies were reconciled. The authors tallied type and number of professionalism statements for each student for each block from peers, tutors and self.		representative and balanced account of their professional behavior across the year. As an indicator of ''representative'', we identified behaviors that were observed by tutors in at least two different groups, which suggest stability of behavior across time and group dynamics. For the 16 students where two different tutors identified the same TAFI, peers identified the same TAFIs as the tutors in five PBL Blocks for seven students, four PBL blocks for six students, three PBL blocks for two students, two PBL blocks for one student. All 16 students reported the TAFI in their summative portfolio, citing both tutor and peer feedback. All cited evidence of improvement from peers and/or tutors. A strong majority (91%) of portfolio essays followed a pattern of citing feedback from PBL peers and/or faculty to identify at least one TAFI, followed by citations indicating improved performance. To illustrate student's use of PBL feedback to document performance, we provide the portfolio self-assessment of one student who received ten TAFIs during the first half of the year and three in the second half, indicating a tendency to dominate. The tendency to dominate was mentioned by peers in four of the five PBL groups but never mentioned by tutors. These comments included statements such as ''talking over people,'' ''can push ideas a little bit too hard ''sometime can seem

No.	Title	Author/year	MERSQI	COREQ	Reason for inclusion			Undergraduate (UG)/Postgraduate (PG)	Methodology	Purpose of study	Key findings
					Communication	Ethics	Professionalism				
									- Coding and analysis of summative portfolio essays: A research assistant extracted the summative portfolio essays from the database for the four competencies (Professionalism, Communication, Personal Development, and Reflective Practice) that 32 students submitted at the end of the 2009 academic year, for a total of 124 essays. The authors used the Professionalism Rubric to analyze the portfolio essays for the aforementioned competencies, where professionalism issues were likely to be addressed. The authors independently coded professionalism statements before meeting to reach consensus in the rare instances where there were differences in coding. All PBL citations used to document the coded statements were tallied by source (tutor, peer, self) and block time period.		argumentative/defensive'' and needs to ''listen to other viewpoints even though they might not coincide with personal views.'' Three other TAFIs were mentioned three or less times and only in the first half of the year. Table 2 provides quotes from this one student's cited PBL feedback, documenting a TAFI (tendency to dominate) and evidence of improvement over the course of year 1. This student acknowledged and reflected on this TAFI in the portfolio essay and provided evidence of improvement, noting that this behavior may need to be ''reestablished'' with each new working group. See Table 3 for excerpt from this student's portfolio. The essay provides evidence relevant to each of our questions. The student identified and acknowledged a prob- lematic pattern of behavior, reflected on the feedback, and used suggestions to ''amend'' behavior, which resulted in improved performance. Not every student described the self- regulation process in such detail, but all acknowledged a behavior identified by peers and/or tutors that was problem- atic and presented evidence of improvement.<br>From discussion: ... While this study suggests that students are using the supports of this educational program to self-regulate, we need to better understand how students process and use feedback, how external feedback triggers and shapes intern- ally

For peer review only

No.	Title	Author/year	MERSQI	COREQ	Reason for inclusion			Undergraduate (UG)/Postgraduate (PG)	Methodology	Purpose of study	Key findings
					Communication	Ethics	Professionalism				
											generated feedback, what role advisors play in helping students reflect on and use feedback, and what processes students use to make judgments and develop plans for improvement, questions being addressed by the work of Sargeant et al. (2008, 2009, 2011). As future physicians, today's students will need to be able to self-regulate their behaviors in settings where feedback may not be as structured or robust and facilitation of reflection lacking. To support development of SR skills, we need medical school programs designed to provide formative feedback and clear standards, opportunities to improve, and facilitated self-assessment. The PBL-portfolio model provides one strategy by which students can gain skills in self-regulation and develop habits useful for future practice.
70	Portfolio assessment in medical students' final examinations	Davis, MH, Ben-David, M Friedman, Harden, RM, Howie, Peter, Ker, Jean, McGhee, C, Pippard, MJ and Snadden, David<br><br>2009	7		✓	✓		UG	From abstract: Portfolio assessment has been developed for this purpose and has been adopted for the summative assessment of students in their final examination in Dundee. The contents of the portfolio and the assessment process have been defined and the first cohort of students to be assessed in this	This paper describes a new approach to the final examination based on portfolios, which has been piloted in Dundee Medical School. This paper describes our first year's experience with this new approach.	From abstract: The evalu-ation of the approach demonstrated strong staff support. Students were also positive although with some reservations. It is concluded that portfolio assessment is a powerful approach to assessing a range of curriculum outcomes not easily assessed by other methods and is worthy of inclusion in the assessor's toolkit.<br>Results: Of the 129 candidates for the portfolio assessment, three were withdrawn for the reasons given above. Of the 126 remaining, 108 (86%)

For peer review only

No.	Title	Author/year	MERSQI	COREQ	Reason for inclusion			Undergraduate (UG)/Postgraduate (PG)	Methodology	Purpose of study	Key findings
					Communication	Ethics	Professionalism				
									way has been studied. The final examination: The final examination was redesigned to meet the needs of the new curriculum, in particular the moves to an inte- grated, task-based approach and an outcome-based education model. The examination is in two parts. Part 1 is at the end of year 4 and comprises a multiple-choice paper of extended matching items (134) designed to assess knowledge and its application; a constructed response question (CRQ) paper to assess higher-order thinking such as problem solving and critical analysis as well as knowl- edge; and an OSCE to assess clinical skills. The use of an OSCE as part of the final examination has been reported (Preece et al., 1992). Part 2 of the final examination takes place towards the end of year 5 and adopts a more innova- tive		passed, 13 (10%) received a condi- tional pass and five (4%) failed. No students fell into the bad fail category. Individual student profiles, in terms of the 22 grades for the learning outcomes, showed variation. Three students (2%) were awarded 22 A grades. One student (0.8%) was awarded 22 B grades. No student was awarded 22 C, D, E or F grades. Forty-four students (35%) were awarded 22 grades at either A or B levels. Two students (1.5%) were awarded 22 grades at either C or D levels. No students were awarded 22 grades at either E or F levels. Over 60% of the students were not consistent in their ability across the learning outcomes, but had individual strengths and weaknesses. The student gradings awarded by both pairs of exam- iners in each outcome are given in Figure 1. Non-pass gradings are confined to outcomes 2, 7, 8, 11 and 12.<br>Evaluation of portfolio assessment:<br>- (1) Analysis of student results: Numeric values from one to six were allocated to the students' outcome grades in the portfolio assessment. An overall mark was then calculated for every student. This overall mark was correlated, using the Spearman correlation, with the mark for each of the three compo- nents of Part 1 of the final examination (EMI, 0.42; CRQ, 0.42; OSCE, 0.47) and with the mark allocated to students

For peer review only

No.	Title	Author/year	MERSQI	COREQ	Reason for inclusion			Undergraduate (UG)/Postgraduate (PG)	Methodology	Purpose of study	Key findings
					Communication	Ethics	Professionalism				
									approach – portfolio assessment. The approach was adopted as it offered the potential of assessing the students' achievement in each of the curriculum outcomes including those not easily assessed by more traditional approaches. It also offered the potential of reviewing the development of student competence over time and presenting feedback to students.<br>Implementation of the portfolio assessment:<br>Work for the portfolio assessment was carried out in three stages:<br>- Stage 1: Work carried out before the portfolio review -- Towards the end of year 5, before the PRHO preparation block, students submitted their completed portfolios to administrative staff in the medical school office. Following a check for completeness,		in the end-of-block examination for one phase 3 core block attachment (psychiatry, 0.34). These results show low/moderate correlation.<br>- (2) Observer documentation: Three observers, two internal and one external, were appointed to document any issues that arose during the portfolio assessment process. They reported that the port- folio submission procedures worked well. The countersigned receipt given to students was an essential step to avoid disputes over submitted material. Entering the pre-marked grades for various portfolio components into the PASS was a major task for administrative staff and required to be streamlined for future years. ... (see under issues related to implementation of portfolio) The PASS system worked well and provided signed sheets to be retained as a permanent record of the examination.<br>- (53) Examiners' evaluation questionnaire: The examiners' evaluation questionnaire was developed following discussion with members of the working group that introduced portfolio assessment and with both the external and internal examiners who participated in the portfolio examination. It was designed to gather the exam- iners' opinions regarding the: effectiveness of the portfolio approach to student assessment; usefulness of the components of the portfolio

For peer review only - http://bmjopen.bmj.com/site/about/guidelines.xhtml

No.	Title	Author/year	MERSQI	COREQ	Reason for inclusion			Undergraduate (UG)/Postgraduate (PG)	Methodology	Purpose of study	Key findings
					Communication	Ethics	Professionalism				
									they received and countersigned a receipt. The administrative staff transcribed from student records, onto an assessment summary sheet called the Port-folio Assessment and Summary Sheet (PASS), the grades awarded for the portfolio contents in respect of each of the curriculum outcomes. ... Three of the students were identified as ineligible to proceed to the portfolio review with the examiners at this stage, two for reasons of illness and non-completion of course work and one pending a disciplinary hearing. All three, however, were allowed to complete the portfolio review process for formative purposes.<br>- Stage 2: Portfolio review with the examiners -- ... (see under deciding how to mark/assess)<br>- Stage 3: Work completed after the portfolio review -- ... (see		in providing information about the students' abilities; conduct of the examination; standards achieved by the students; strengths and weaknesses of the portfolio approach to assessment; suggestions for improvement. Examiners were asked to rate a number of statements on a Likert-type five-point scale from 5—strongly agree to 1— strongly disagree. There were also free-response questions. The questionnaire was distributed and completed by the examiners at the end of the examiners' meeting on day 2. Thirty-three examiners' questionnaires were distributed and returned, giving a response rate of 100%. Responses were collated using the Excel computer software package by Microsoft. The percentage distribution of examiners' ratings with the number that responded to each statement (N) the calculated average score (AV) and the standard deviation (SD) are provided in Table 1. ... (see the rest under perspective of examiners)<br>- (4) Student evaluation questionnaire: The student evaluation questionnaire was developed following periodic feedback from the students during their phase 3 studies and from a student group discussion with the external observer that took place immediately after the portfolio review. Students were given the opportunity for free and frank expression of their opinions during this discussion. The

No.	Title	Author/year	MERSQI	COREQ	Reason for inclusion			Undergraduate (UG)/Postgraduate (PG)	Methodology	Purpose of study	Key findings
					Communication	Ethics	Professionalism				
									under deciding how to mark/assess) Evaluation of the portfolio assessment: Five methods were employed to evaluate the use of portfo- lios as the basis for the final examination: (1) analysis of student results; (2) observer documentation; (53) examiners' evaluation questionnaire; (4) student evaluation questionnaire; (5) verbal report from student representatives. (see details under results of study).; From discussion - evaluation methodology: Rossi & Freeman (1993a) (p. 13) define evaluation research as 'a robust arena of activity directed at collecting, analysing and interpreting information on the need for, implementa- tion of, and effectiveness and efficiency of intervention efforts'. Education evaluation is more specifically identified as 'the process of making judgement about		student questionnaire was designed to gather opinion about building the portfolio, about its focus around the curriculum outcomes and about events during the portfolio assessment. The questionnaire was distributed and completed 22 days after the examination, at a time when all students were aware of whether or not they had passed the examina- tion. Those students who were unsuccessful had received counselling. The rating scale, data entry and scoring system were similar to those used in the examiners' ques- tionnaire. A total of 107 student questionnaires were returned from 129 students who sat the examination—a response rate of 83%. The percentage distribution of students' ratings, the number that responded to each statement (N), the calcu- lated average score (AV) and the standard deviation (SD) are provided in Table 2 for building the portfolio and in Table 3 for the portfolio assessment. ... (see the rest under perspective of students) - (5) Student representatives' comments: ... (see the rest under perspective of students)

For peer review only

No.	Title	Author/year	MERSQI	COREQ	Reason for inclusion			Undergraduate (UG)/Postgraduate (PG)	Methodology	Purpose of study	Key findings
					Communication	Ethics	Professionalism				
									the merit, value or worth of educational programmes' (Gall et al., 1996). It is recognized that it is often impossible to conduct educational evaluations according to the best possible design and Rossi & Freeman (1993b) advocate the concept of the 'good enough' evaluation. The five methods employed to evaluate the use of portfolios as the basis for the final examination followed the 'good enough' approach.		
71	A Qualitative Analysis of Narrative Preclerkship Assessment Data to Evaluate Teamwork Skills	Dolan, B. M., O'Brien, C. L., Cameron, K. A. and Green, M. M.<br><br>2018		15	✓		✓	UG	From abstract: We performed a qualitative analysis of narrative data in 15 assessment portfolios. Student portfolios were randomly selected from 3 groups stratified by quantitative ratings of teamwork performance gathered from small-group and clinical preceptor assessment forms. Narrative data included peer and faculty feedback from these same forms. Data were	We sought to understand if and how teamwork behaviors are described in the narrative feedback given by faculty and peers in preclerkship years and how this feedback may vary based on students' competency achievement level. We expect that review of assessment data collected in an electronic portfolio and judged by experienced faculty may fill the gap in teamwork assessment by providing a method to evaluate	From abstract -- Results: Eight codes related to teamwork emerged: attitude and demeanor, information facilitation, leadership, preparation and dependability, professionalism, team orientation, values team member contributions, and nonspecific teamwork comments. The frequency of codes and valence varied across the 3 performance groups, with students in the low-performing group receiving more suggestions for improvement across all teamwork codes. Conclusions: Narrative data from assessment portfolios included specific descriptions of teamwork behavior, with important contributions provided by

For peer review only - http://bmjopen.bmj.com/site/about/guidelines.xhtml

No.	Title	Author/year	MERSQI	COREQ	Reason for inclusion			Undergraduate (UG)/Postgraduate (PG)	Methodology	Purpose of study	Key findings
					Communication	Ethics	Professionalism				
									coded for teamwork-related behaviors using a constant comparative approach combined with an identification of the valence of the coded statements as either “positive observation” or “suggestion for improvement.” Study approach and setting: We conducted a qualitative study using a constant comparative approach to better characterize the narrative assessments that students receive regarding teamwork.17 ... We excluded assessments that do not include students' teamwork skills, including assessments from observed structured clinical exams, assessments of written assignments, medical knowledge assessments, assessments of groups as a whole (where no individual feedback was given), ethics oral examinations, direct observations of	longitudinal, behaviorally based narrative feedback regarding student teamwork knowledge, skills, and attitudes.	both faculty and peers. A variety of teamwork domains were represented. Such feedback as collected in an assessment portfolio can be used for longitudinal assessment of preclerkship student teamwork skills and attitudes. From discussion: This analysis demonstrates that narrative feedback in assessment portfolios provides a longitudinal view of preclerkship students' teamwork behaviors. Although similarities exist between performance groups, we observed differences between groups in content, frequency, and valence of feedback. These differences could assist mentor/coaches and competency committee members in identifying both students who excel at teamwork and those needing additional support with teamwork behaviors prior to starting clerkships. Although our coded categories were identified without the use of a predetermined framework, they align with key teamwork and leadership attributes identified in the literature. Critical teamwork skills and attitudes have previously been described that share similarity with our codes: leadership, team orientation, preparation/dependability.21 Physician leadership competencies highlight the importance of showing integrity, listening actively, promoting team building, and nurturing/recognizing others' accomplishments.22–24

For peer review only

No.	Title	Author/year	MERSQI	COREQ	Reason for inclusion			Undergraduate (UG)/Postgraduate (PG)	Methodology	Purpose of study	Key findings
					Communication	Ethics	Professionalism				
									clinical skills, and scholarly project oral presentations (which focus solely on presentation skills). Sampling strategy: Students were notified of the study via e-mail and were encouraged to e-mail an author not involved in grading students (CLO) if they did not wish to participate. Students were given 1 week to respond. Four students chose to opt out of the study. We divided the remaining student portfolios into three groups. We first identified the students who received progressing towards competence with concern or progressing towards competence pending additional development for any of the three competencies most closely related to teamwork—teamwork, professionalism, and communication—during the competency review. We		These attributes arose in our analysis, lending credibility to the argument that narrative data in assessment portfolios can be used to understand students' teamwork behaviors in the preclerkship years and can help students reflect on how their behaviors promote or detract from their teams' overall performance. Review of the preclerkship narrative data provides mentors or coaches the ability to identify students in need of remediation before full-time immersion in clinical practice.25 Our analysis demonstrates that low-performing students share similar characteristics that reviewers can look for poor attitude in small groups, lack of preparation for group activities, or dismissive behavior toward others' contributions. It also demonstrates that the absence of certain comments may be telling, as evidenced by the sharp reduction in even nonspecific comments such as “great team member” or the lack of leadership observations in the low-performing student portfolios. When reviewing assessment data with their students, mentors may choose to highlight these absences to help students improve. Notably, peers provided the majority of feedback in the codes of team orientation and values' contributions of others. Due to their close working relationships during preclerkship years, peers may be in the best position

For peer review only

No.	Title	Author/year	MERSQI	COREQ	Reason for inclusion			Undergraduate (UG)/Postgraduate (PG)	Methodology	Purpose of study	Key findings
					Communication	Ethics	Professionalism				
									designated these 19 students as the low-performing group. As our competency review does not have a comparable high-performance designation, we employed an alternative strategy using the composite grading system. To identify a high-performing cohort of students for comparison, we identified the 19 students receiving the highest numerical ratings for the group of assessments included in Table 1. We designated this to be the high-performing group. The middle-performing group comprised all remaining students (n = 114) who did not meet criteria for the high- or low-performing groups. Four students opted out of the study. To protect privacy, each student portfolio received a random numerical identifier. A key was created linking each identifier to each student's ID number; an		to make such judgments. Furthermore, when peers are given formalized instruction in providing feedback, as they are at our institution, including their insight and feedback is critical when assessing student performance. Our results suggest that assessment systems that do not include peer feedback may miss the opportunity to identify students in need of support for developing teamwork skills. Prior studies demonstrate student acceptance of peer feedback, particularly when used for formative purposes.26 Additional studies that specifically compare faculty versus student feedback may further illuminate the best ways to employ the differing perspectives of peers and faculty when assessing student performance. The use of assessment portfolio data may continue to increase as institutions implement entrustable professional activities, competency committees, and other assessment strategies that require a systems-based approach.27 We believe that the findings in this analysis can assist both in teamwork assessment and as a resource to aid faculty and students in identifying preclerkship goals for teamwork and leadership skills development. Additional study is needed to determine if preclerkship assessment of teamwork correlates with later clinical performance. In conclusion, our analysis of narrative

For peer review only

No.	Title	Author/year	MERSQI	COREQ	Reason for inclusion			Undergraduate (UG)/Postgraduate (PG)	Methodology	Purpose of study	Key findings
					Communication	Ethics	Professionalism				
									author not involved in direct teaching of students (CLO) deidentified all data before analysis. We randomly selected three student portfolios from each group (high, middle, low) to analyze, then further intentionally sampled two additional portfolios in each group to allow for adequate gender inclusion and to confirm emergence of no new codes (see next); these 15 portfolios composed our sample. Data analysis: To better understand how teamwork performance is observed and described by peers and faculty, we used constant comparative analysis techniques. We selected this method as a framework to explore teamwork given that comparison between groups of students may better delineate the similarities and/or differences in how behaviors were described among variably		assessment data from across the preclerkship curriculum demonstrates that these data capture a number of key teamwork skills and attitudes with behaviorally based feedback, thus filling a gap in the longitudinal assessment of teamwork. Providing students and faculty the opportunity to critique and further reflect on behaviorally based narrative feedback may assist students in improving these skills prior to the beginning of clerkships.

No.	Title	Author/year	MERSQI	COREQ	Reason for inclusion			Undergraduate (UG)/Postgraduate (PG)	Methodology	Purpose of study	Key findings
					Communication	Ethics	Professionalism				
									performing students. Constant comparative methodology has been described as an ideal way to "conceptualise the variety that exists within the subject under study.17(p393) After using this methodology to complete the coding, we undertook a content analysis to better understand the prevalence and valence of identified themes across the three performance groups.19 Three research team members (BMD, CLO, MG) with expertise in medical student assessment performed the primary analysis of portfolio comments; two are practicing clinicians with experience on medical teams (BMD, MG). The fourth team member, an expert in qualitative research (KAC), provided guidance and oversight in methodology. Narrative data was uploaded for analysis into		

No.	Title	Author/year	MERSQI	COREQ	Reason for inclusion			Undergraduate (UG)/Postgraduate (PG)	Methodology	Purpose of study	Key findings
					Communication	Ethics	Professionalism				
									NVivo (QSR, Mac version 10.2.0) after deidentification.20 We selected the middle-performing group as the first group for analysis, as it represents the typical student who is neither struggling nor exceptionally high achieving. Consistent with conventional content analysis19 and to best explore what teamwork categories faculty and students choose to comment on in their narratives, we did not use an a priori framework for coding. However, one author (BMD) is familiar with medical teamwork literature,21,22 which has informed the way medical students approach teamwork in our institution and which may have influenced her approach to coding. The second coder has a background in education. She had no specific expertise with the teamwork literature and arrived at the same codes. Two		

For peer review only

No.	Title	Author/year	MERSQI	COREQ	Reason for inclusion			Undergraduate (UG)/Postgraduate (PG)	Methodology	Purpose of study	Key findings
					Communication	Ethics	Professionalism				
									authors (BMD and CLO) initially reviewed three portfolios from the middle-performing group, assigning a code to all narrative statements. These authors then met and achieved consensus regarding the coding framework. The remaining portfolios in the middle-performing group were analyzed using the identified codes; all statements were assigned a valence of either "positive observation" or "suggestion for improvement." Three authors (BMD, CLO, MG) met to review the statements' assigned codes and come to consensus on any differences, both with respect to code and valence. Differences were resolved via group discussion. Using the codes identified, we then analyzed the low- and high-performing groups, representing the extremes of performance.		

For peer review only

No.	Title	Author/year	MERSQI	COREQ	Reason for inclusion			Undergraduate (UG)/Postgraduate (PG)	Methodology	Purpose of study	Key findings
					Communication	Ethics	Professionalism				
									Although no new codes were identified in the high-performing or low-performing groups, the narrative from these groups helped to refine the coding set to reflect nuances that emerged from the other groups. For example, after reviewing the high-performing narrative data, the original code “information sharing” was revised to “information facilitation” to better reflect the variety of ways that students mediated the flow of information in small groups. The code “values contributions of team members” evolved from “values team members” after review of the additional suggestions for improvement found in both the high- and low-performing groups. We reviewed three portfolios in each performance group but identified few new codes after the second student's portfolio in each group. However,		

For peer review only

No.	Title	Author/year	MERSQI	COREQ	Reason for inclusion			Undergraduate (UG)/Postgraduate (PG)	Methodology	Purpose of study	Key findings
					Communication	Ethics	Professionalism				
									we continued with two additional portfolios per group for adequate gender inclusion and to confirm the emergence of no new codes; no new codes were found with the addition of these six portfolios. Upon completion of the coding process, NVivo software was used to quantify the number of comments per code and valence. Techniques to ensure trustworthiness of data included the use of two coders followed by additional review of the codes by a third author. Codes and examples were also discussed at a medical education group research meeting where additional faculty members with less connection to the portfolio process provided feedback to the research team.		
72	Portfolio as a learning tool: students' perspective	Elango, S., Jutti, R. C. and Lee, L. K.<br><br>2005	6.5		✓			UG	From abstract: A questionnaire survey was conducted among 143 medical students to find out their perceptions of the	This study was formulated to find out students' perspectives on portfolio learning.	From abstract: ... (see the rest under perspective of students) The study indicates that students need appropriate guidance from the academic staff for the system to succeed.<br>From introduction: In the

For peer review only - http://bmjopen.bmj.com/site/about/guidelines.xhtml

No.	Title	Author/year	MERSQI	COREQ	Reason for inclusion			Undergraduate (UG)/Postgraduate (PG)	Methodology	Purpose of study	Key findings
					Communication	Ethics	Professionalism				
									portfolio as a learning tool. From materials and methods: Feedback was obtained from an entire batch of Semester 8 students by asking them to write down what they felt about writing a portfolio. Based on their feedback, a questionnaire consisting of 23 questions was designed. The entire student population (259) in the clinical school (from Semester 6 to Semester 10) was given an information sheet with details about this survey and was given the option of participating in the study. The students who volunteered for the study were asked to sign a written consent form. The students who gave their consent for the survey were requested to complete the questionnaire anonymously. For each of the 23 questions, students had to respond on a 4-point Likert scale, ranging from		last decade, there have been numerous innovations in educational theory and practice. Education has moved from being a traditional teacher-centred process to one that is student-centred. Similarly, in the area of assessment, there has been a shift away from assessing knowledge towards a more competency/performance-based assessment. There is now a wide variety of methods of education and training to choose from and portfolio-based learning is an increasingly popular option among educationists.1<br>From results: One hundred and forty-three students completed and returned the questionnaire (Table1). There were 76 (53.1%) females and 64 (44.8%) males. In order to facilitate the analysis, the 4 levels of perception in the questionnaire – strongly agree, agree, disagree and strongly disagree – were regrouped; strongly agree and agree were grouped as one, and strongly disagree and disagree were grouped as the other. ... (see the rest under perspective of students)<br>From discussion: A study has shown that students using portfolios obtain higher marks in factual knowledge compared to the control group of students not using portfolios11<br>From conclusion: The study demonstrates that any medical school intending to introduce the portfolio as a new learning method must take into consideration an

For peer review only - http://bmjopen.bmj.com/site/about/guidelines.xhtml

No.	Title	Author/year	MERSQI	COREQ	Reason for inclusion			Undergraduate (UG)/Postgraduate (PG)	Methodology	Purpose of study	Key findings
					Communication	Ethics	Professionalism				
									strongly agree to strongly disagree. The responses to the questionnaire were analysed to determine the overall views of the students on the different aspects of portfolio writing.		effective orientation programme for both the learners and the teachers. Motivated students, and trained and motivated staff are essential for portfolio-based learning to succeed.
73	Students' reflections in a portfolio pilot: highlighting professional issues	Haffling, A. C., Beckman, A., Pahlmblad, A. and Edgren, G.<br><br>2010		14	✓	✓	✓	UG	From abstract: Thirty-five voluntary final-year medical students piloted a standardized portfolio in a general practice (GP) attachment at Lund University, Sweden. Students’ portfolio reflections were based upon documentary evidence from practice, and aimed to demonstrate students’ learning. The reflections were qualitatively analysed, using a framework approach. Students’ evaluations of the portfolio were subjected to quantitative and qualitative analysis.<br>Pilot: Students of two consecutive classes, of 76 and 71 students, respectively, were approached. Twenty students in each class	From abstract: To investigate whether students’ reflections include sufficient dimensions of professional competence, notwithstanding a standardized portfolio format, and to evaluate students’ satisfaction with the portfolio<br>From introduction: We aimed to develop a portfolio for the assessment of final-year students’ professional development during their general practice (GP) attachment. ... We designed a pilot study with the principal aim of investigating whether themes of students’ reflections would include satisfactory dimensions of professional competence, notwithstanding the standardized format. This article qualitatively explores the emergent themes of students’ reflections, and also reports on students’ evaluation of the portfolio.	From abstract -- Results: Among professional issues, an integration of cognitive, affective and practical dimensions in clinical practice was provided by students’ reflections. The findings suggested an emphasis on affective issues, particularly on self-awareness of feelings, attitudes and concerns. In addition, ethical problems, clinical reasoning strategies and future communication skills training were subjects of several reflective commentaries. Students’ reflections on their consultation skills demonstrated their endeavour to achieve structure in the medical interview by negotiation of an agenda for the consultation, keeping the interview on track, and using internal summarizing. The importance of active listening and exploration of patient's perspective was also emphasized. In students’ case summaries, illustrating characteristic attributes of GP, the dominating theme was ‘patient-centred care’, including the patient–doctor relationship, holistic modelling and longitudinal continuity. Students were satisfied with the portfolio,

For peer review only

No.	Title	Author/year	MERSQI	COREQ	Reason for inclusion			Undergraduate (UG)/Postgraduate (PG)	Methodology	Purpose of study	Key findings
					Communication	Ethics	Professionalism				
									were offered to voluntarily pilot a portfolio in GP as an addition to the written test, which for the participants would be reduced in the number of GP questions. A total of 35 students, 16 from the first and 19 from the second class, accepted. Twenty-four (69%) of them were women, as compared to 59% of all students. At the time of the study, consent from the University's ethics committee was not required for projects involving students' assessments. Participating students were provided with both verbal and written guidelines on the purpose, format and assessment of their portfolios. Three of the authors (ACH, AP, AB) and a fourth teacher, all involved in teaching, acted as mentors to support their students in compiling the portfolio. Qualitative analysis of students' reflections: To be		but improved instructions were needed.; Conclusions: A standardized portfolio in a defined course with a limited timeframe provided ample opportunities for reflections on professional issues. Support by mentors and a final examiner interview contributed to the success of the portfolio with students. The interview also allowed students to deepen their reflections and to receive feedback. Students' evaluation questionnaire: see under perspective of students From discussion: Firstly, students' reflections on their consultation skills showed that the most important learning issue in the theme of 'process skills' was how to achieve structure in the medical interview. Patient-centred communication can cause genuine concerns in students about losing control of the consultation and getting caught in a flow of less ordered information from the patient (Silverman et al. 2004). When students learned how to obtain structure, they also experienced better time management, enhanced self-confidence and an improved rapport with the patient. Secondly, the theme of 'patient-centred care' was emphasized in students' case summaries. Patient-centred care is a major principle in GP, but is of essential interest to several other medical disciplines as well. Achieving shared decision making, understanding the patient as

No.	Title	Author/year	MERSQI	COREQ	Reason for inclusion			Undergraduate (UG)/Postgraduate (PG)	Methodology	Purpose of study	Key findings
					Communication	Ethics	Professionalism				
									able to conduct the qualitative analysis, students' reflections were written verbatim into a word processor by one of the authors (173). Each student was allocated a code number to prevent identification of individual students' comments. The text of the reflections constructed two separate units of analysis; 'consultation skills' and 'case summaries'. The transcripts were read through several times to get a sense of the whole, and then analysed using a framework approach (Crabtree & Miller 1999). For 'consultation skills', the text was mapped against the Calgary Cambridge Guides (Silverman et al. 2004). For 'case summaries', the statement of WONCA Europe (2005), (Box 2), was used as a template for organizing the data. In each of		a whole person and exploring the patient's perspective are aspects shown to positively influence patients' satisfaction and enablement, reduce their symptom burden and discomfort, and increase efficiency of care (Stewart et al. 2000; Little et al. 2001). We believe that these central dimensions of patient-centred care ought to be highlighted earlier in hospital clerkships. Thirdly, the findings suggested integration of the cognitive, affective and practical dimensions of professional competence that can be acquired in a clinical learning environment (Mann et al. 2007; van Tartwijk & Driessen 2009; Passi et al. 2010). As expected, students reflected on their 'content skills' and provided evidence for their identification of deficits in knowledge and skills, their integration of new understanding, and their plans for development. However, 'perception skills' was a more prominent theme in both units of analysis. In their self-awareness of feelings, attitudes and concerns, students were unexpectedly open and frank about their deficiencies and about how they had tried to remedy them. They also acknowledged the importance of ethical issues and additional communication skills training, and openly reflected on their clinical reasoning. However, we found it remarkable that

For peer review only

No.	Title	Author/year	MERSQI	COREQ	Reason for inclusion			Undergraduate (UG)/Postgraduate (PG)	Methodology	Purpose of study	Key findings
					Communication	Ethics	Professionalism				
									the two units of analysis, related segments of text were identified and indexed to the appropriate part of the thematic framework. Some codes were modified and new ones added to reflect as many nuances in the data as possible. 'Chunks' of related text under each code were examined together and distilled summaries made; this process involved a considerable amount of abstraction. The chunk summaries provided an analytical tool from which connections were made and sub-themes and themes identified. ACH undertook the mapping of text and carried out the primary analysis of the data. The four teachers, involved in the pilot, examined in recurrent meetings whether the interpretation of the data was plausible. The codes and sub-themes were compared based on differences		several students, in their final year in medical school, reflected on their initial problems in transferring theory to practice; a skill supposed to be frequently trained during previous hospital clerkships.<br>From conclusion: In summary, this study demonstrated that a structured portfolio in a short timeframe provided abundant opportunities for students to reflect on personal and professional issues, notwithstanding the standardized format. We strongly recommend a final examiner interview to deepen students' reflections, to supply feedback and to raise the importance of reflective ability as a skill in future learning from practice.

For peer review only

No.	Title	Author/year	MERSQI	COREQ	Reason for inclusion			Undergraduate (UG)/Postgraduate (PG)	Methodology	Purpose of study	Key findings
					Communication	Ethics	Professionalism				
									and similarities, and some were revised during this process of corroboration. Students' evaluation questionnaire: The short student evaluation form was supplied directly after the examiner interview, asking five questions about the portfolio instructions, the mentor contact and the examiner interview, using a 3-point scale ('yes', 'to some extent', 'no'). Students were also requested to provide free comments on positive and negative experiences of the portfolio, and to give suggestions for improvements. The results were subjected to quantitative and qualitative (content) analyses.		
74	Undergraduate medical students' views about a reflective portfolio assessment of their communication skills learning	Rees, C. and Sheard, C.<br><br>2004	10		✓			UG	From abstract: 178 second-year medical students at the University of Nottingham completed the 18-item reflective portfolio questionnaire (RPQ) (α = 0.716) and a personal details questionnaire	this study explores second-year medical students' views about a reflective portfolio assessment of their communication skills. The research questions for this study are: 1) What are students' views of a reflective portfolio assessment	From abstract -- Results: Total scores on the RPQ ranged from 40 to 75 (mean 58.28, SD 7.08). Significant relationships existed between RPQ total scores and students' ratings of their reflection skills (r s = 0.322, P < 0.001), RPQ total scores and students' confidence building another portfolio (T = 4.381, d.f. = 176, P

For peer review only

No.	Title	Author/year	MERSQI	COREQ	Reason for inclusion			Undergraduate (UG)/Postgraduate (PG)	Methodology	Purpose of study	Key findings
					Communication	Ethics	Professionalism				
									three days before submitting their portfolio assessment for communication skills. Data were analysed using univariate and multivariate statistics on SPSS Version 10.0. From methods: Participants -- Second-year medical students at the University of Nottingham completed a personal details questionnaire (PDQ) and the reflective portfolio questionnaire (RPQ) three days before submitting their portfolio assessment for communication skills.; Measures -- The PDQ contains 13 demographic and education-related items. The RPQ was designed by CR to explore medical students' views of portfolios and reflection. Eighteen items were generated using previous research literature,1-4 e.g. ‘I have felt anxious about sharing my weaknesses in my reflective portfolio’ (item 16). Each item	of their communication skills? 2) Do relationships exist between students' views of reflective portfolios and their perceived and actual reflection skills and their confidence building portfolios?	< 0.001), and RPQ total scores and students' marks for their reflective portfolio assessment ($r_s = 0.167$, $P = 0.029$). Students with more positive views about reflective portfolios were more likely to rate their reflection skills as good, receive better marks for their portfolio assessment, and be more confident building another portfolio. Discussion: This study begins to highlight preclinical medical students' views about reflective portfolios. However, further research is required using qualitative studies to explore students' views in depth. Medical educators should be encouraged to consider introducing portfolios as a method of formative and summative assessment earlier in the medical curriculum. From results: - Participant characteristics: Of the 206 students invited to participate in this study, 178 (86.4%) completed the reflective portfolio questionnaires satisfactorily. Students ranged in age from 18 to 30 years (median = 20, interquartile range = 19–20). The majority were female ($n = 105$, 59%), white ($n = 130$, 73.4%) and came from nonmanual socio-economic classes ($n = 170$, 95.5%). Students' ratings of their communication skills ranged from 1 to 5 (median = 3, interquartile range = 3–3) and nearly all ($n = 173$, 97.2%) thought their communication skills needed improving. Students' ratings of their

For peer review only

No.	Title	Author/year	MERSQI	COREQ	Reason for inclusion			Undergraduate (UG)/Postgraduate (PG)	Methodology	Purpose of study	Key findings
					Communication	Ethics	Professionalism				
									was written in the form of a statement and was accompanied by a 5-point Likert scale from 1 (strongly disagree) to 5 (strongly agree). Six medical educators with a range of experience of reflective portfolios commented on the language used in the preliminary draft of the questionnaire, which resulted in some minor alterations in language. The RPQ is scored by reversing the scores for the negative items and then summing the scores for all 18 items. The total score ranges from 18 to 90, with higher scores indicating more positive views about reflective portfolios. This scale was found to have good internal consistency in this study (Cronbach's α= 0.716) and the study results presented in this paper support the construct validity of the tool (copies of the RPQ can		reflection skills ranged from 1 to 5 (median = 3, interquartile range = 2–3) and most (n = 155, 87.6%) thought their reflection skills needed improving. ... (see the rest under perspective of students) Students' scores for their reflective portfolio coursework ranged from 33 to 100% (median = 67, interquartile range = 60–73%).<br>- Students' views about reflective portfolios: see the rest under perspective of students<br>From discussion: ... (see the rest under perspective of students) These findings have implications for further research and educational practice. Qualitative studies are needed to explore in depth the reasons why students possess the views they have about reflective portfolios. Qualitative research may also shed some light on how medical educators could increase students' confidence in building reflective portfolios. This qualitative research could help medical educators improve educational programs using portfolios, so that they are more acceptable to students. In the meantime, this study indicates that the act of constructing a reflective portfolio could increase students' confidence in building another portfolio. Despite portfolios being seen to have weaknesses (e.g. lacking reliability4), medical educators should be encouraged to consider introducing portfolios as a

For peer review only

No.	Title	Author/year	MERSQI	COREQ	Reason for inclusion			Undergraduate (UG)/Postgraduate (PG)	Methodology	Purpose of study	Key findings
					Communication	Ethics	Professionalism				
									be requested by contacting the author). Students' percentage marks for their reflective portfolio assessments were collected at a later date.; Data analysis -- After checking the distribution of continuous variables, data were analysed using univariate (i.e. Spearman's correlation coefficients and T -tests) and multivariate statistics (i.e. stepwise multiple regression) on SPSS Version 10.0.		method of formative and summative assessment earlier in the medical curriculum. Students may then be better placed to negotiate this type of assessment in higher stakes examinations.
75	The reliability of assessment criteria for undergraduate medical students' communication skills portfolios: the Nottingham experience	Rees, C. E. and Sheard, C. E.<br><br>2004	8.5		✓			UG	From abstract: Two independent analysts assessed a random sample of portfolios (n = 100, 49.5%) using criterion-referenced assessment. Students' performances were examined against subjective items in five areas: 1) portfolio structure, 2) level of critical reflection, 3) level of skills development, 4) use of documentary evidence, and 5) use of relevant literature. These	From abstract: This study aims to determine the reliability of assessment criteria used for a portfolio at the University of Nottingham.	From abstract -- Results: The level of agreement between the two raters for the total percentage score was 0.771 (95% CI = 0.678, 0.840), as measured by an intraclass correlation coefficient. The levels of agreement between the two raters for the individual items of the assessment criteria ranged from κ = 0.359 (item 3) to κ = 0.693 (item 4). Discussion: This study provides some support for the summative assessment of portfolios. The findings suggest that discussion and negotiation between independent assessors can enhance the reliability of assessment criteria. Therefore, medical educators are encouraged to use such procedures in the summative assessment

No.	Title	Author/year	MERSQI	COREQ	Reason for inclusion			Undergraduate (UG)/Postgraduate (PG)	Methodology	Purpose of study	Key findings
					Communication	Ethics	Professionalism				
									subjective judgements were later converted into quantitative scales ranging from 0 to 3 so that interrater reliability could be established. The level of agreement between the two analysts for the total percentage score was established using an intraclass correlation coefficient and for the individual items using weighted kappa coefficients. From methods: Sample -- Of the 202 portfolios submitted in March 2002, a random sample of 100 (49.5%) were photocopied for second marking. This random sample of 100 portfolios (median = 66.7%, interquartile range = 53.3–73.3%) did not differ significantly from the portfolios (n = 102) not chosen for second marking (median = 66.7%, interquartile range = 53.3–73.3%) in terms of their total percentage scores (Z = −0.155, P = 0.877).; Assessment		of portfolios. From results: The level of agreement between the two raters for the total percentage scores, as measured by an intraclass correlation coefficient, was 0.771 (95% CI = 0.678, 0.840). The levels of agreement between the two raters for the individual items, as measured by weighted kappa coefficients, ranged from 0.359 (item 3) to 0.693 (item 4) (see Table 1).

For peer review only

No.	Title	Author/year	MERSQI	COREQ	Reason for inclusion			Undergraduate (UG)/Postgraduate (PG)	Methodology	Purpose of study	Key findings
					Communication	Ethics	Professionalism				
									procedures -- One hundred portfolios were assessed ... Although students were aware of these five areas, they were not give the full assessment criteria outlined in Appendix 1. ... (see the rest under deciding how to mark/assess); Statistical procedures: An intraclass correlation coefficient on SPSS (Version 10.0) was used to establish the level of agreement between the two raters for the total percentage scores. Weighted kappa coefficients on SAS (Release 6.12) were used to determine the levels of agreement between the two raters for the individual items of the assessment criteria.		
76	Portfolio as a tool to evaluate clinical competences of traumatology in medical students	Santonja-Medina, F., Garcia-Sanz, M. P., Martinez-Martinez, F., Bo, D. and Garcia-Estan, J. 2016	10.5		✓			UG	From abstract: A total of 131 students used the portfolio during their clinical rotation of traumatology. The students' portfolios were blind evaluated by four professors who annotated the existence	From abstract: This article investigates whether a reflexive portfolio is instrumental in determining the level of acquisition of clinical competences in traumatology, a subject in the 5th year of the degree of medicine. From introduction: ...	From abstract: The reliability of the portfolio was moderate, according to the kappa index (0.48), but the evaluation scores between evaluators were very similar. Considering the mean percentage, 59.8% of the students obtained all the competences established and only 13 of the 23 learning outcomes (56.5%) were fulfilled by >50% of the

For peer review only - http://bmjopen.bmj.com/site/about/guidelines.xhtml

No.	Title	Author/year	MERSQI	COREQ	Reason for inclusion			Undergraduate (UG)/Postgraduate (PG)	Methodology	Purpose of study	Key findings
					Communication	Ethics	Professionalism				
									(yes/no) of 23 learning outcomes. From methods: A portfolio was designed to assess the level of acquisition of clinical competences, ... - Academic structure: Traumatology is a compulsory subject in the 5th year of the degree, consisting of 55 hours of theoretical classes, of which 28 hours take place in the classroom, 14 hours are of a seminar type, 10 hours are about clinical cases, and 3 hours are about cooperative learning; 21 hours of classes on laboratory skills in seven sessions; and 2 weeks of hospital rotation (50 hours, 10 days). Of these 50 hours, students rotate in the traumatology service of the university hospitals between hospital ward, medical consultation, operating rooms, and emergency services. Only one student is assigned to one professor in the	Thus, in the current article, we report the application of a portfolio tool to assess the level of acquisition of clinical skills and competences in medical students of the Medical School of the University of Murcia (Spain), during the clinical rotations of traumatology and orthopedic surgery. An important part of the study is also devoted to the analysis of the reliability of the portfolio.29	students. Our study suggests that the portfolio may be an important tool to quantitatively analyze the acquisition of traumatology competences of medical students, thus allowing the implementation of methods to improve its teaching. From results: A total of 138 comparisons were made to obtain the kappa index among the four evaluators for each of the 23 learning outcomes. The distribution of frequencies and percentages of agreement, according to the classification of Landis and Koch,29 are shown in Table 3. As it can be seen, out of 138 comparisons, 83 had kappa indexes >0.4 and 44 of them had values >0.6, which indicates that in a 31.9% of the occasions, the agreements between evaluators have been very substantial. Table 4 shows the mean and standard deviation of the kappa index of the four evaluators. It can be seen that the global kappa is 0.48, indicating that the portfolio has a moderate reliability, according to Landis and Koch.29 As an additional way of assessing the reliability of the portfolio, 6 months after the initial evaluation, two of the evaluators revised again the portfolios (second revision or reference). Table 5 shows the level of agreement obtained by each evaluator when both revisions were analyzed. Globally, the differences among evaluators are not very important because kappa indexes show that

For peer review only

No.	Title	Author/year	MERSQI	COREQ	Reason for inclusion			Undergraduate (UG)/Postgraduate (PG)	Methodology	Purpose of study	Key findings
					Communication	Ethics	Professionalism				
									hospital. Table 2 shows the number of days students spent during these rotations. - Portfolio structure: The portfolio was given to all the students during the course (n=131) in a booklet format. After the identification of data, the students read about legal and ethical issues in relation to the need for keeping medical secret and signed conformity. Each day the students were expected to fill up their reflections before leaving the hospital, writing about the clinical activities observed during the day. In addition, the portfolio had to be signed by the assigned professor every day. The students were given enough space to include everything needed, but there were three main paragraphs: ... (see under content of portfolio) - Portfolio evaluation: The portfolios were evaluated by four		evaluators 1 and 4 have a substantial agreement in both revisions, whereas evaluators 2 and 3 have a moderate level of agreement. The level of achievement of the traumatology competences is shown in Table 1. The data show the mean percentage of the four evaluations as well as the number of times the procedures were performed. A total of 59.8% of the students have acquired the competences as expected during their hospital stay. In all, 13 of the 23 learning outcomes (56.5%) have been acquired by >50% of the students. Competence 5 (87.4%) and competence 1 (81.4%) show the greater values, whereas the rest of them show an acceptable level, except competence 7, showing only a value of 26%. From discussion: The current article shows the results obtained with the application of a portfolio to medical students as a tool to obtain information regarding the level of acquisition of the clinical competences of the topic traumatology. One of the important aspects of any portfolio is its reliability, the consistency and accuracy of the assessment tool in measuring students' performance. Our results quite agree with those found by other authors;30,31 although in some studies of nurse students, higher numbers (0.8) have been described.32 To prove that the results were not obtained by chance, the

For peer review only

No.	Title	Author/year	MERSQI	COREQ	Reason for inclusion			Undergraduate (UG)/Postgraduate (PG)	Methodology	Purpose of study	Key findings
					Communication	Ethics	Professionalism				
									professors, three of them professors of traumatology and one a colleague professor from a different area, that is, physiology; all of them were medical doctors. ... (see the rest under deciding how to mark/assess) All the portfolios underwent blind evaluation. All the students agreed and participated enthusiastically in the study.<br>- Statistical analysis: Data analysis was performed using the Statistical Package for the Social Sciences, Version 19. The Cohen's kappa coefficient was used as a statistical measure of interevaluator agreement for qualitative items.29 Frequencies, percentages, means, and standard deviations were also obtained to describe the results.		second review of the portfolios took place 6 months after the initial evaluation in order to see whether the reliability index could be improved. As observed in Table 5, the maximum level of agreement among evaluators is found in competence 6 (observe and assist in surgical treatments). A moderate agreement exists in competences 2, 3, and 4, whereas competences 1, 5, and 7 show a low level of agreement among evaluators. The results of Table 1 show important information regarding the level of acquisition of the competences analyzed. Two of them, competences 1 and 5, related to diagnostic and treatment obtained the greatest value, with an important frequency of occurrence. Next competence in terms of the level of acquisition is number 3 (70%), probably due to the high number of radiographic images viewed in contrast to magnetic resonance images, where it is likely that doctors rely more on the radiologist report, without actually interpreting them. Competence 6 has also a moderate level of acquisition (55.8%), in spite of the fact that almost all students (99.2%) report attendance to operating rooms (2.6 + 1.3 days). This is likely due to the fact that the surgeons do not invite all the students to wash and be prepared to help, an activity we recommend to all our professors. A similar

For peer review only

No.	Title	Author/year	MERSQI	COREQ	Reason for inclusion			Undergraduate (UG)/Postgraduate (PG)	Methodology	Purpose of study	Key findings
					Communication	Ethics	Professionalism				
											reason may be behind the moderate level achieved in competences 2 (47.1%) and 4 (51.5%), showing that not all the students are able to explore or perform maneuvers with the patients and that this may be due to a time limitation problem while in the ward or consultation. We believe that this should be corrected and doctors should allow more time for the students to perform, under the appropriate supervision, exploration, or treatment of the patients. Finally, competence 7 has the lowest level. It is likely related to the fact that students are not allowed to sign discharge reports or other forms, although it is important that they learn from their professors how to do it. In any case, this is the first time that we obtained this quantitative information regarding the clinical stays of the medical students and this will help us to analyze our teaching results and to develop new strategies to enhance the level of clinical competence the students should reach. A problem learned from the application of this portfolio is that many of the learning outcomes were checked without taking into account the number of times these were carried out. The rest of the learning outcomes were quantitative, so we could obtain the number of times the student performed them. For instance, they attended the operating rooms 5.5 + 3.3 times (range, 1–26), and viewed a mean of 2.3 magnetic resonance images

For peer review only

No.	Title	Author/year	MERSQI	COREQ	Reason for inclusion			Undergraduate (UG)/Postgraduate (PG)	Methodology	Purpose of study	Key findings
					Communication	Ethics	Professionalism				
											(range, 1–14). We believe that it is important that the portfolios are designed to collect also the quantitative information, since both quantitative and qualitative information give the real essence of the learning process. A drawback we have seen with the application of the portfolio to clinical students is that there is a random factor affecting the acquisition of the competences. This is due to the fact that the students rotate between different hospitals and at different times of the year. Thus, it depends on the type of medical problems available on the days they are assigned to the hospital; thus they may not see all the problems selected in the portfolio. Clearly, the greater the number of days of hospital stay, the better it is. However, this is not always possible. A better selection of the learning outcomes will also be helpful. Moreover, the portfolio is designed to be an evaluation tool that should be used in conjunction with others (written or oral exams, laboratory evaluations) because it is not possible to find just one tool that is able to assess all the competences.33,34<br>From conclusion: In conclusion, the portfolio is an important tool that has allowed to obtain qualitative and quantitative data regarding the degree of acquisition of traumatology competences by medical students. Although some factors need to be improved, such as the

No.	Title	Author/year	MERSQI	COREQ	Reason for inclusion			Undergraduate (UG)/Postgraduate (PG)	Methodology	Purpose of study	Key findings
					Communication	Ethics	Professionalism				
											inclusion of more quantitative elements, a more comprehensive selection of learning outcomes, and combination with other tools, we believe that the use of this kind of tool by clinical teachers will be useful to ascertain the degree of practical competence reached by their students.
77	Assessing the Development of Medical Students' Personal and Professional Skills by Portfolio	Yielder, J. and Moir, F.<br><br>2016		6			✓	UG	The implementation process employed a cyclical model that was designed to evaluate and improve the use of the portfolio, that could form the basis of the first cycle of action research that will be continued in the future to improve the initiative (Fig. 1). During 2013, the introduction of the portfolio was monitored, and changes were made in 2014. In 2014, feedback from students indicated the need for further modifications, which are currently being implemented, along with the introduction of an electronic platform. Student feedback was ascertained from the generic end-of-year course evaluation, which included	From abstract: We present the portfolio format along with the process for its introduction and appraise the challenges, strengths, and limitations of the approach within the context of the current literature. We then outline a cyclical model of evaluation used to monitor and fine-tune the portfolio tasks and implementation process, in response to student and assessor feedback.<br>From introduction: This document outlines the learning outcomes that are to be substantively completed by the end of the second postgraduate year in practice. ... This article presents the process for introducing the portfolio, including the challenges, strengths and limitations, evaluation of data to date, and the ways in which student	From abstract: The portfolios have illustrated the level of insight, maturity, and synthesis of personal and professional qualities that students are capable of achieving. The Auckland medical program strives to foster these qualities in its students, and the portfolio provides an opportunity for students to demonstrate their reflective abilities. Moreover, the creation of a Personal and Professional Skills domain with the portfolio as its key assessment emphasizes the importance of reflective practice and personal and professional development and gives a clear message that these are fundamental longitudinal elements of the program.<br>From conclusion:<br>The implementation of a portfolio for the assessment of the PPS domain in the medical program at the University of Auckland has proved to be an important part of the redeveloped curriculum. It has highlighted the importance of PPS topics and has provided an opportunity and incentive for students to reflect on aspects of their personal and professional development, with the aim

No.	Title	Author/year	MERSQI	COREQ	Reason for inclusion			Undergraduate (UG)/Postgraduate (PG)	Methodology	Purpose of study	Key findings
					Communication	Ethics	Professionalism				
									questions relating to the PPS domain. The number of students in each year varied from 199–260 and there was an average response rate of 50%. In addition, the Auckland University Medical Students Association conducted a student evaluation for the purpose of writing a report for the ongoing medical program accreditation, from which key issues were collated and forwarded to the PPS domain coordinators. The eight portfolio assessors also provided suggestions for improvement at the end of 2013 and 2014. Evaluation feedback, modifications, and qualitative data derived from the portfolios are presented in the Findings section. Due to the qualitative nature of the feedback, it was analyzed inductively, through a cross-sectional thematic analysis that sought to	feedback has been addressed.	of improving their practice as doctors. In the process, we have learned several aspects about students that previously may have remained unknown, which has led in some cases to conversations with students that will hopefully make a difference to their learning and development trajectories. We have also seen many high-quality and insightful portfolios that demonstrate a synthesis of personal and professional qualities that are inspiring and which we hope are an indication that these students will develop into excellent doctors. With the increasing use of, and requirement for, the compilation of professional portfolios in the medical profession internationally, especially at postgraduate level, there is real benefit for students in becoming familiar with this process of pregraduation.<br>It has also been a learning process for the domain coordinators introducing the assessment. In particular, we learned the value of a cyclic, flexible, and ongoing process of evaluation that has allowed us to continuously reflect on what is working and what needs to change in order to be responsive to student needs and at the same time create a curriculum change that aims to create better doctors. ... We hope that as students come to trust our responsiveness, they will become increasingly open to the importance of taking on the responsibility for their

For peer review only

No.	Title	Author/year	MERSQI	COREQ	Reason for inclusion			Undergraduate (UG)/Postgraduate (PG)	Methodology	Purpose of study	Key findings
					Communication	Ethics	Professionalism				
									ascertain the strongest themes from across the three sources of feedback.		own learning in the PPS domain.
78	Construct Validity of an Instrument for Assessment of Reflective Writing-Based Portfolios of Medical Students	Kassab, Salah Bidmos, Mubarak Nomikos, Michail Daher-Nashif, Suhad Kane, Tanya Sarangi, Srikant Abu-Hijleh, Marwan<br><br>2020	7		✓		✓	UG	After an extensive literature review and pilot testing of the instrument, two raters assessed the reflective writing-based portfolios from years 2 and 3 medical students (n=135) on three occasions. The instrument consists of three criteria: organization, description of an experience and reflection on the experience. We calculated the reliability of scores using generalizability theory with a fully crossed design and two facets (raters and occasions). In addition, we measured criterion validity by testing correlations with students' scores using other assessment methods.	Assessment of reflective writing for medical students is challenging, and there is lack of an available instrument with good psychometric properties. The authors developed a new instrument for assessment of reflective writing-based portfolios and examined the construct validity of this instrument.	The dependability (Φ) coefficient of the portfolio scores was 0.75 using two raters on three occasions. Students' portfolio scores represented 46.6% of the total variance across all score comparisons. The variance due to occasions was negligible, while the student– occasion interaction was small. The variance due to student–rater interaction represented 17.7%, and the remaining 27.7% of the variance was due to unexplained sources of error. The decision (D) study suggested that an acceptable dependability (Φ = 0.70 and 0.72) can be achieved by using two raters for one and two occasions, respectively. Finally, we found moderate to large effect-size correlations between students' scores in reflective writing-based portfolios and communication skills (r = 0.47) and PBL tutorials (r = 0.50).<br><br>We demonstrated the presence of different sources of evidence that support construct validity of the study instrument. Further studies are warranted before utilizing this instrument for summative assessment of students' reflective writing-based portfolios in other medical schools.
79	Communication skills teaching	Douglas, A. H. Acharya, S. P.		19	✓			UG	This study is a qualitative	Communication skills (194) are vital for	The two main themes: 1. Positivity

For peer review only - http://bmjopen.bmj.com/site/about/guidelines.xhtml

No.	Title	Author/year	MERSQI	COREQ	Reason for inclusion			Undergraduate (UG)/Postgraduate (PG)	Methodology	Purpose of study	Key findings
					Communication	Ethics	Professionalism				
	and learning in Nepal; what are medical students' perceptions and experiences? A qualitative study	Allery, L. A.<br><br>2020							evaluation of a CS course in Nepal, exploring the experiences and perceptions of participants. The study aims to also identify aspects that were helpful or not for student learning and areas for potential development.<br><br>A purposive sample of twenty: second, fourth and Intern year students was selected for interview. Data were collected through audio recorded semi-structured interviews following a piloted schedule. Interview transcripts were manually coded and thematically analysed. Codes were arranged into themes and subthemes.	doctors. Indeed, as the most important element of consultations, are highly valued by patients. CS are core, teachable skills, however, have not been widely taught in South Asian medical schools, unlike their western counterparts.<br><br>Patan Academy of Health Sciences, is one of the first in Nepal to have CS central to its' aims and curriculum. CS are taught from the first weeks of medical school and re-enforced during preclinical study (first 2 years). Our study seeks to explore students' perceptions and experiences of CS teaching in this South Asian, Nepal context.	2. Experiential learning. Results demonstrate participants' positive perceptions of CS teaching: believing it is important, effective, relevant and valuable for personal development. Participants identified experiential learning features as valuable for CS acquisition. Intern students recognised CS relevance and requested expanding teaching to clinical years, incorporating challenging communication scenarios.<br><br>This study shows that PAHS' CS course is well perceived and valuable to learners. Experiential learning is powerful for CS development. Expansion of formal, structured CS teaching through all years in a spiral curriculum, should be considered. Violence towards doctors in South Asia is increasing. Students recognised CS teaching's significance in addressing this.<br><br>CS teaching,still in its' infancy in South Asia, is a pressing issue for medical educators here. Our study provides evidence it is well perceived with positive impacts in this context, particularly when employing experiential learning. Medical schools in south Asia should be encouraged to incorporate and strengthen their CS teaching curriculum.
80	Assessment practices in undergraduate	Brits, H.<br>Bezuidenhout, J.<br>Van der Merwe, L. J.	5.5	12	✓			UG	A descriptive cross-sectional study design was	Assessment should form an integral part of curriculum design	All disciplines in the undergraduate medical programme are

For peer review only - http://bmjopen.bmj.com/site/about/guidelines.xhtml

No.	Title	Author/year	MERSQI	COREQ	Reason for inclusion			Undergraduate (UG)/Postgraduate (PG)	Methodology	Purpose of study	Key findings
					Communication	Ethics	Professionalism				
	clinical medicine training: What do we do and how can we improve?	Joubert, G.<br><br>2020							used. Qualitative and quantitative data were gathered by means of open- and closed-ended questions in a self-administered questionnaire, which was completed by teaching and learning coordinators in 13 disciplines.	in higher education and should be robust enough to ensure clinical competence.<br><br>This article reports on current assessment practices and makes recommendations to improve clinical assessment in the undergraduate medical programme at the University of the Free State.	represented. They used different assessment methods to assess the competencies required of entry-level healthcare professionals. Workplace-based assessment was performed by 30.1% of disciplines, while multiple-choice questions (MCQs) (76.9%) and objective structured clinical examinations (OSCEs) (53.6%) were the main methods used during formative assessment. Not all assessors were well prepared for assessment, with 38.5% never having received any formal training on assessment. Few disciplines (15.4%) made use of post-assessment moderation as a standard practice, and few disciplines always gave feedback after assessments.<br><br>The current assessment practices for clinical students in the undergraduate medical programme at the University of the Free State cover the spectrum that is necessary to assess all the different competencies required. Multiple-choice questions and OSCEs, which are valid and reliable assessment methods, are used frequently. Poor feedback and moderation practices should be addressed. More formative assessments, and less emphasis on summative assessment, should be considered. Workplace-based and continuous assessments may be good ways to assess clinical competence.

For peer review only

No.	Title	Author/year	MERSQI	COREQ	Reason for inclusion			Undergraduate (UG)/Postgraduate (PG)	Methodology	Purpose of study	Key findings
					Communication	Ethics	Professionalism				
81	**Added value of assessing medical students' reflective writings in communication skills training: a longitudinal study in four academic centres**	Ament Giuliani Franco, C.<br>Franco, R. S.<br>Cecilio-Fernandes, D.<br>Severo, M.<br>Ferreira, M. A.<br>de Carvalho-Filho, M. A.<br><br>2020	10.5		✓			UG	Third-year and fourth-year medical students enrolled in an elective course on clinical communication skills development were assessed using different assessment methods.<br><br>The communication skills course was offered at four universities (three in Brazil and one in Portugal) and included 69 students.<br><br>The students were assessed by a Multiple-Choice Questionnaire (MCQ), an objective structured clinical examination (OSCE) and reflective writing narratives. The Cronbach's alpha, dimensionality and the person's correlation were applied to evaluate the reliability of the assessment methods and their correlations. Reflective witting was assessed by applying the Reflection Evaluation for Enhanced Competencies Tool Rubric	This study describes the development and implementation of a model to assess students' communication skills highlighting the use of reflective writing. We aimed to evaluate the usefulness of the students' reflections in the assessment of communication skills.	The Cronbach alpha for the MCQ, OSCE global score, TS and RS were, respectively, 0.697, 0.633, 0.784 and 0.850. The interobserver correlation for the TS and RS were, respectively, 0.907 and 0.816. The assessment of reflection using the TS was significantly correlated with the MCQ (r=0.412; p=0.019), OSCE (0.439; p=0.012) and RS (0.410; p=0.020). The RS did not correlate with the MCQ and OSCE.<br><br>Assessing reflection through mapping the themes and analysing the depth of reflective writing expands the assessment of communication skills. While the assessment of reflective themes is related to the cognitive and behavioural domains of learning, the reflective depth seems to be a specific competence, not correlated with other assessment methods—possibly a metacognitive domain.

For peer review only

No.	Title	Author/year	MERSQI	COREQ	Reason for inclusion			Undergraduate (UG)/Postgraduate (PG)	Methodology	Purpose of study	Key findings
					Communication	Ethics	Professionalism				
									(Reflect Score (RS)) to measure reflections' depth, and the Thematic Score (TS) to map and grade reflections' themes.		
82	Contextual Analysis of Stakeholder Opinion on Management and Leadership Competencies for Undergraduate Medical Education: Informing Course Design	Rajeh, N.<br>Grant, J.<br>Farsi, J.<br>Tekian, A.<br><br>2020		16	✓			UG	This was a cross-sectional study conducted at King Abdulaziz University (KAU), Saudi Arabia, during 2019. An exploratory qualitative approach, utilizing systematic content analysis, was used. Data were collected using semi-structured interviews that were con- ducted with 10 leaders who were stakeholders at KAU, health service providers at KAU hospital, and stakeholders in the Ministry of Health.	The study aimed to conduct a contextual analysis of interviews intended to assist with the future design of a feasible and relevant leadership and management course for undergraduate medical students at King Abdulaziz University (KAU), Saudi Arabia.	This study revealed critical findings that highlighted the areas in which KAU could instill better and adequate leadership and management skills in their undergraduate medical students. Multiple core categories for a leadership and management curriculum emerged with many interrelated themes. Most participants mentioned that leadership can be taught and that early exposure is beneficial for develop- ing skills. Additionally, they stated that leaders should have a vision and the ability to articulate that vision.<br><br>Different implementation challenges were described in relation to the availability of human resources, the current short supply of suitable teachers, and program design. Teaching methods recommended included simulations, lectures, and a project-based approach. Assessment methods that were recommended included objective structured clinical examination (OSCE), formative and summa- tive assessments, self-assessments, and portfolios.

For peer review only

BMJ Open

An evidence-guided approach to Portfolio guided Teaching and Assessing Communications, Ethics and Professionalism for medical students and physicians:A Systematic Scoping Review

Journal:	*BMJ Open*
Manuscript ID	bmjopen-2022-067048.R1
Article Type:	Original research
Date Submitted by the Author:	11-Jan-2023

Complete List of Authors:	Ting, Jacquelin Jia Qi; National University of Singapore, Yong Loo Lin School of Medicine; National Cancer Centre Singapore, Division of Supportive and Palliative Care<br>Phua, Gillian Li Gek; National Cancer Centre Singapore, Division of Supportive and Palliative Care; Duke-NUS Medical School, Lien Centre for Palliative Care<br>Hong, Daniel Zhihao; National University of Singapore, Yong Loo Lin School of Medicine; National Cancer Centre Singapore, Division of Supportive and Palliative Care<br>Lam, Bertrand Kai Yang; National Cancer Centre Singapore, Division of Supportive and Palliative Care<br>Lim, Annabelle Jia Sing ; National University of Singapore, Yong Loo Lin School of Medicine; National Cancer Centre Singapore, Department of Supportive and Palliative Care<br>Chong, Eleanor Jia Xin; National University of Singapore, Yong Loo Lin School of Medicine; National Cancer Centre Singapore, Division of Supportive and Palliative Care<br>Pisupati, Anushka; National University of Singapore, Yong Loo Lin School of Medicine; National Cancer Centre Singapore, Division of Supportive and Palliative Care<br>Tan, Rei; National University of Singapore, Yong Loo Lin School of Medicine; National Cancer Centre Singapore, Division of Supportive and Palliative Care<br>Yeo, Jocelyn Yi Huang; National University of Singapore, Yong Loo Lin School of Medicine; National Cancer Centre Singapore, Division of Supportive and Palliative Care<br>Koh, Yi Zhe; National University of Singapore, Yong Loo Lin School of Medicine; National Cancer Centre Singapore, Division of Supportive and Palliative Care<br>Quek, Chrystie; National University of Singapore, Yong Loo Lin School of Medicine; National Cancer Centre Singapore, Division of Supportive and Palliative Care<br>Lim, Jia Yin; National University of Singapore, Yong Loo Lin School of Medicine; National Cancer Centre Singapore, Division of Supportive and Palliative Care<br>Tay, Kuang Teck; National Cancer Centre Singapore, Division of Supportive and Palliative Care<br>Ong, Yun Ting; National University of Singapore, Yong Loo Lin School of

	Medicine; National Cancer Centre Singapore Division of Supportive and Palliative Care<br>Chiam, Min; National Cancer Centre Singapore, Division of Cancer Education<br>Zhou, Xuelian Jamie; National Cancer Centre Singapore, Division of Supportive and Palliative Care; Duke-NUS Medical School, Lien Centre for Palliative Care<br>Mason, Stephen; University of Liverpool, Palliative Care Institute Liverpool<br>Wijaya, Limin; National Cancer Centre Singapore, Division of Supportive and Palliative Care; Duke-NUS Medical School<br>Krishna, Lalit; University of Liverpool,
**Primary Subject Heading**:	Communication
Secondary Subject Heading:	Communication, Ethics, Medical education and training
Keywords:	MEDICAL EDUCATION & TRAINING, ETHICS (see Medical Ethics), EDUCATION & TRAINING (see Medical Education & Training)

SCHOLARONE™ Manuscripts

I, the Submitting Author has the right to grant and does grant on behalf of all authors of the Work (as defined in the below author licence), an exclusive licence and/or a non-exclusive licence for contributions from authors who are: i) UK Crown employees; ii) where BMJ has agreed a CC-BY licence shall apply, and/or iii) in accordance with the terms applicable for US Federal Government officers or employees acting as part of their official duties; on a worldwide, perpetual, irrevocable, royalty-free basis to BMJ Publishing Group Ltd ("BMJ") its licensees and where the relevant Journal is co-owned by BMJ to the co-owners of the Journal, to publish the Work in this journal and any other BMJ products and to exploit all rights, as set out in our licence.

The Submitting Author accepts and understands that any supply made under these terms is made by BMJ to the Submitting Author unless you are acting as an employee on behalf of your employer or a postgraduate student of an affiliated institution which is paying any applicable article publishing charge ("APC") for Open Access articles. Where the Submitting Author wishes to make the Work available on an Open Access basis (and intends to pay the relevant APC), the terms of reuse of such Open Access shall be governed by a Creative Commons licence – details of these licences and which Creative Commons licence will apply to this Work are set out in our licence referred to above.

Other than as permitted in any relevant BMJ Author's Self Archiving Policies, I confirm this Work has not been accepted for publication elsewhere, is not being considered for publication elsewhere and does not duplicate material already published. I confirm all authors consent to publication of this Work and authorise the granting of this licence.

For peer review only

Title: **An evidence-guided approach to Portfolio guided Teaching and Assessing Communications, Ethics and Professionalism for medical students and physicians:**

A Systematic Scoping Review

Short Title: **A review of CEP Portfolios for physicians and medical students**

Jacquelin Jia Qi Ting[1,2], Gillian Phua[2,3,4], Daniel Zhihao Hong [1,2], Bertrand Kai Yang Lam[2], Annabelle Jia Sing Lim[1,2], Eleanor Jia Xin Chong[1,2], Anushka Pisupati[1,2] , Rei Tan[1,2], Jocelyn Yi Huang Yeo[1,2], Yi Zhe Koh[1,2], Chrystie Wan Ning Quek[1,2], Jia Yin Lim[1,2], Kuang Teck Tay[2], Yun Ting Ong[1,2], Min Chiam[5,] , Jamie Zhou [2,3,4], Stephen Mason[8], Limin Wijaya[2,3,9], Lalit Kumar Radha Krishna[1-8,#]

[1] Yong Loo Lin School of Medicine, National University of Singapore, NUHS Tower Block, 1E Kent Ridge Road, Level 11, Singapore 119228

[2] Division of Supportive and Palliative Care, National Cancer Centre Singapore,11 Hospital Dr, Singapore 169610

[3] Duke-NUS Medical School, 8 College Rd, Singapore 169857

[4] Lien Centre for Palliative Care, Duke-NUS Medical School, 8 College Rd, Singapore 169857

[5] Division of Cancer Education, National Cancer Centre Singapore, 11 Hospital Dr, Singapore 169610

[6] PalC, The Palliative Care Centre for Excellence in Research and Education, PalC c/o Dover Park Hospice, 10 Jalan Tan Tock Seng, Singapore

[7] Centre of Biomedical Ethics, National University of Singapore, 21 Lower Kent Ridge Rd, Singapore 119077

[8] Palliative Care Institute Liverpool, Academic Palliative & End of Life Care Centre, Cancer Research Centre, University of Liverpool, 200 London Rd, Liverpool L3 9TA, United Kingdom

[9] Department of Infectious Diseases, Singapore General Hospital, Outram Road, Singapore 169608

[#]Corresponding Author:

Dr. Lalit Kumar Radha Krishna

Palliative Care Institute Liverpool, Academic Palliative & End of Life Care Centre,

University of Liverpool, 200 London Rd, Liverpool L3 9TA, United Kingdom

Email: lalit.radha-krishna@liverpool.ac.uk Phone number: 6436 8183

For peer review only - http://bmjopen.bmj.com/site/about/guidelines.xhtml

ABSTRACT

Objectives

Guiding the development of longitudinal competencies in communication, ethics, and professionalism underlines the role of portfolios to capture and evaluate the multiple multi-source appraisals, and direct personalised support to clinicians. However a common approach to these combined portfolios continues to elude medical practice. A systematic scoping review is proposed to map portfolio use in training and assessments of ethics, communication, and professionalism competencies particularly as its inculcation of new values, beliefs and principles changes attitudes, thinking, practice, while nurturing Professional Identity Formation. It is posited that effective structuring of portfolios promote self-directed learning, personalised assessment and appropriate support of professional identity formation.

Design

Krishna's Systematic Evidence-Based Approach is employed to guide this systematic scoping review of portfolio use in communication, ethics and professionalism training and assessment published in PubMed, Embase, PsycINFO, ERIC, Scopus, and Google Scholar databases between 1st January 2000 and 30th June 2020. The included articles are concurrently content and thematically analysed using the Split Approach. Overlapping categories and themes identified are combined using the Jigsaw Perspective. The themes/categories are compared with the summaries of the included articles in the Funnelling Process to ensure their accuracy. The domains identified form the framework for the discussion

Results

12,300 abstracts were reviewed, 946 full-text articles were evaluated, and 82 articles were analysed and the four domains identified were indications; content; design; and strengths and limitations.

Conclusions

This review reveals that when using a consistent framework, accepted endpoints and outcome measures, longitudinal multisource, multimodal assessment data fashions professional and personal development and enhances identity construction. Future studies into effective assessment tools and support mechanisms are required if portfolio use is to be maximised.

For peer review only - http://bmjopen.bmj.com/site/about/guidelines.xhtml

Strengths and limitations of this study

- The SEBA methodology adopts the structure of systematic reviews and flexibility of narrative reviews to synthesise a reproducible and accountable evaluation of diverse methodological sources, settings, physician populations and specialities.
- This is the first review to map current practices and the longitudinal effects of communication, ethics and professionalism portfolios
- This review provides the first insights into the impact of longitudinal development of ethics, professionalism and communications competencies and their impact on professional identity formation

Abstract Word Count: 273 words

Manuscript Word Count: 1999 words

Number of Tables: 5

Number of Figures: 2

Number of References: 152

Keywords: Portfolios, Communication, Ethics, Professionalism, Professional Identity Formation, Medical Education, medical school, undergraduate, postgraduate training.

For peer review only

INTRODUCTION

Evidence for the effective embodiment of ethical and professional principles, communication skills and appropriate use of empathy in clinical practice requires a longitudinal, and often multi-source perspective. With ethics and professionalism sharing longitudinal developmental trajectories and intimately entwined with communication skills and competencies, the three competencies are increasingly considered together. In practice longitudinal CEP programs appear in the curricula of the Top 10 medical schools featured on the QS World University Rankings 2020 (1). Concurrent study of communication, ethics, and professionalism (henceforth CEP) is also underlined by their common sociocultural roots. As sociocultural constructs (2), CEP competencies are shaped by the individual's personal experiences, motivations, enthusiasm, idealism, abilities, competencies, virtues, expectations, knowledge, skills, emotions, and attitudes (henceforth narratives); their values, beliefs, and principles (henceforth belief systems)(3); their clinical experiences, competencies, training, insights, and confidence (henceforth clinical insights)(4-7) and their practice, clinical, social , cultural, academic, research, and personal considerations (henceforth contextual considerations)(8-12).

However, whilst previous reviews into the teaching of ethics (13-15), communication (16-22) and professionalism (23-25) suggest the use of portfolios could provide a personalised, holistic, and longitudinal perspective of CEP skills, knowledge, and attitudes, and support of developing competencies we are aware of little progress in designing such platforms. Impetus for mapping current use of CEP portfolios also arises from the notion that developing CEP competencies shape how medical students and physicians (henceforth clinician) "think, act and feel like a physician" (26) or their professional identity formation (henceforth PIF) (27). It is posited that the promise of CEP portfolios with better appreciation of evolving self-concepts of professional identity will better direct support and even remediation of professional, ethical, communication and interprofessional development and PIF. This is especially pertinent at a time of increasing reports of breaches in standards, codes of conduct, and social and practice expectations (25).

METHODS

A Systematic Evidence-Based Approach (SEBA) guided Systematic Scoping Review (SSR) (henceforth SSR in SEBA) is proposed to map CEP portfolio use in medicine to guide the design, structuring and support of a proposed program (13-17, 20-22, 24, 25, 27-48). Given space constraints, we briefly describe the six stages in the construction of SSRs in SEBA here (Figure 1) and advance more details of the Systematic Approach, Split

For peer review only - http://bmjopen.bmj.com/site/about/guidelines.xhtml

Approach, Jigsaw Perspective, Funnelling Process, Reiterative Process and Synthesis of SSR, in Appendix A (16, 34, 40, 49-52).

(Insert Figure 1. *The SEBA Process*)

Stage 1 of SEBA: Systematic Approach

A. Determining of Title and Background of the Review

An expert team comprised of a medical librarian from the Yong Loo Lin School of Medicine (YLLSoM) at the National University of Singapore (53), and local education experts and clinicians at the National Cancer Centre Singapore (NCCS), the Palliative Care Institute Liverpool, YLLSoM and Duke-NUS Medical School (henceforth the expert team) supported the research team in designing and overseeing the SEBA process.

B. Identification of Research Question

The research and expert team determined the primary research question: "*What is known about CEP portfolios?*". The secondary research questions were "*what role do CEP portfolios have in teaching and assessing CEP development?*".

C. Inclusion Criteria

A Population, Intervention, Comparison and Outcome (PICOs) format, outlined in Table 1, was used to guide the research process (54, 55).

Table 1. PICOS Inclusion and Exclusion Criteria

PICOS	Inclusion criteria	Exclusion criteria
**Population**	• Undergraduate and postgraduate medical students<br>• Qualified medical doctors, physician or resident; medical officer, registrar, house officer, attending, consultant	• Allied health specialties such as Pharmacy, Dietetics, Chiropractic, Midwifery, Podiatry, Speech Therapy, Occupational and Physiotherapy<br>• Non-medical specialties such as Clinical and Translational Science, Alternative and Traditional Medicine, Veterinary, Dentistry
**Intervention**	• portfolios in undergraduate and postgraduate medical education for teaching and assessment of Communication, Ethics and Professionalism<br><br>Criteria of a portfolio:	Other documentation methods or learning tools that are:<br>• Not longitudinal or single timepoint<br>• Does not include personal intellectual engagement with the content and associated learning (for

For peer review only - http://bmjopen.bmj.com/site/about/guidelines.xhtml

PICOS	Inclusion criteria	Exclusion criteria
	• longitudinal (more than a single timepoint) assessment data<br>• candidate's personal engagement with portfolio content and associated learning<br>• Interventions meeting the above criteria were included regardless of whether they were referred to as portfolios<br><br>All types of portfolios were included in the study:<br>• For instance: electronic & non-electronic; formative & summative or combined; clinical & non-clinical<br>• Portfolios with input from students and/or residents and/or doctors, and/or input from faculty members and other individuals<br>• Portfolios with different structures: extent by which the structure has been prescribed and/or left to individual discretion	instance, curriculum vitae, logbooks and the use of personal digital assistants)
**Comparison/ Context**	NA	NA
**Outcome**	Papers that measured the following outcomes were included:<br>• Effectiveness of the use of portfolios to assess and teach Communication, Ethics and Professionalism<br>• Impact of the use of portfolios on medical students (both undergraduate and postgraduate)<br>• Impact of the use of portfolios on the faculty	NA
**Study design**	• Articles in English or translated to English<br>• Articles published from 1st January 2000 to 31st December 2020<br>• Databases: PsycINFO, EMBASE, PubMed, ERIC, Scopus, Google Scholar<br>• All study designs including:<br>• Mixed methods research, meta-analyses, systematic reviews, randomised controlled trials, cohort studies, case-control studies, cross-sectional studies, descriptive papers, grey literature, opinions, letters, commentaries and editorials	NA

D. Identification of Relevant Studies

The research team developed search strategies and carried out independent reviews for relevant publications in the PubMed, Embase, PsycINFO, ERIC, Scopus, and Google Scholar databases. Keeping with Pham, Rajić (56)'s approach of ensuring a feasible and sustainable research process, the team contained the search to articles

For peer review only - http://bmjopen.bmj.com/site/about/guidelines.xhtml

published between 1st January 2000 and 31st December 2020 to accommodate existing human resource and time constraints and ensure a sustainable review process.

E. Selection of Studies Included in Review

Six members of the research team created individual lists of titles to be included whilst three other members of the research team carried out hand searches and ancestry searches of seven leading journals in medical education (Academic Medicine, Medical Education, Medical Teacher, Advances Health Sciences Education, BMC Medical Education, Teaching and Learning in Medicine and Perspectives on Medical Education) accessed through the NUS library portal. These individual lists of titles were discussed online and Sandelowski and Barroso (57)'s "negotiated consensual validation" approach to "*articulate, defend, and persuade others of the "cogency" or "incisiveness" of their points of view or show their willingness to abandon views that are no longer tenable*" was applied to achieve consensus on the final list of titles to be scrutinised.

F. Assessing the Quality of Articles

Eight research team members individually appraised the quality of the quantitative and qualitative studies using the Medical Education Research Study Quality Instrument (MERSQI) (58) and Consolidated Criteria for Reporting Qualitative Studies (COREQ) (59).

Stage 2 of SEBA: Split Approach

Two teams carried independent and concurrent thematic and content analysis of the included articles whilst a third team created tabulated summaries based on recommendations drawn from Wong, Greenhalgh (60)'s RAMESES publication standards: meta-narrative reviews and Popay, Roberts (61)'s *"Guidance on the conduct of narrative synthesis in systematic reviews".* The categories employed in the content analysis for undergraduate communications were Rider et al. (62) 's *"A model for communication skills assessment across the undergraduate curriculum",* Goldie (63)'s *"Review of ethics curricula in undergraduate medical education"*, Duffy et al. (64)'s *"Assessing Competence in Communication and Interpersonal Skills: The Kalamazoo II Report"* and Hong et al. (13)'s *"Postgraduate Ethics Training Programs: A Systematic Scoping Review"*. Tay et al. (24)'s *"Assessing Professionalism in Medicine - A Scoping Review of Assessment Tools from 1990 to 2018"* was employed for codes

For peer review only - http://bmjopen.bmj.com/site/about/guidelines.xhtml

for professionalism and David (65)'s article *"AMEE Medical Education Guide No. 24: Portfolios as a method of student assessment"* was then used to contextualise their use in portfolios.

Stage 3 of SEBA: The Jigsaw Perspective

The Jigsaw Perspective sees the themes and categories identified compared and combined where overlaps and similarities exist.

Stage 4 of SEBA: The Funnelling Process

The Funnelling Process sees the themes/categories created from the Jigsaw Approach compared with the tabulated summaries to determine their consistency.

Patient and public involvement

Patients or the public were not involved in the design, or conduct, or reporting, or dissemination plans of this study.

RESULTS

A total of 12,300 abstracts were reviewed, 946 full-text articles were evaluated, and 82 articles were analysed (Figure 2). The funnelled domains identified are 1) indications, 2) portfolio content, 3) portfolio design and implementation and 4) strengths and limitations. In keeping with its goals of transparency and accountability, these Tables are enclosed in Appendix B.

(Insert Figure 2. PRISMA Flowchart)

Funneled Domain 1: Indications

CEP portfolios curate longitudinal multisource, multimodal assessment data taken at different settings and contexts allowing it to serve two main roles- teaching and assessment (66, 67) (Table 2). However few span undergraduate and postgraduate education (65, 68, 69), . As assessment tool CEP portfolios assist in the identification of areas of weakness and guide the provision of a comprehensive, targeted feedback, support and remediation (68, 70, 71). Most CEP assessment portfolios in postgraduate education focus on evidencing the attainment of required competencies (72-76), capturing reflections and indications of professional identity

For peer review only - http://bmjopen.bmj.com/site/about/guidelines.xhtml

formation (74, 77-79). CEP portfolios in the postgraduate setting have also been used for revalidation purposes (77)

As a teaching tool, CEP portfolios encourage self-directed learning (71, 80-82), self-improvements (67), reflective practice (68, 69, 71, 75, 77, 81-91), motivate practitioners to achieve desired competencies (86, 92) and bolster overall professional and personal development (68, 84, 92, 93).

Table 2. Benefits of CEP Teaching and Assessment Portfolios

CEP teaching portfolios	CEP assessment portfolios
**Enhancing learning**<br>Promotion of life-long learning (94, 95)<br><br>Promotion of self-directed learning<br>○ Formulation of learning plans (86, 92, 96)<br>○ Facilitates Outcome-oriented learning (85, 86, 97, 98)<br>○ Enhances self-evaluative capabilities (69, 72, 76, 81, 84, 85, 94-96, 99-101)<br>○ Identification of strengths and weaknesses (65, 67, 72, 74, 88, 92, 100, 102-105)<br>○ Inculcates responsibility and ownership for one's learning (67, 69, 70, 76, 80, 82, 97, 106)<br>○ Sets the pace (95)<br>○ Personalised learning (104, 105)	Balance of structure and freeform learning (65, 72, 88, 103, 107, 108)<br><br>Adaptable to goals (73, 109, 110)<br><br>Alignment of learning outcomes and assessment methods (86, 87, 93, 97, 101)<br><br>Allows personalised assessments (73)<br><br>Allows assessment over time (80, 88) and stages of development (65) and that facilitate evaluation of:<br>○ Learning and progress over time (73, 102)<br>○ Learning curve (121, 163)<br>○ In different roles (92, 93)<br>○ In different settings (66, 109)<br>○ Complex behaviours (68, 70, 73, 80, 86, 95, 111, 112)<br>○ Critical thinking (73, 93, 101, 110)<br>○ Outcomes/competencies (103, 111)<br>○ Self-assessment (73, 101, 110)<br>○ Complex skills (109)<br>○ Professionalism (110)<br>○ Cultural competence (110)<br>○ Communication skills (110)<br>○ Clinical ethics (113)<br>○ Problem solving abilities (73, 110)<br>○ Reflective abilities (73, 86, 101, 105)<br><br>Longitudinal data<br>○ Possess good predictive value of subsequent clinical competency (70, 114)<br>○ Enhances validity of portfolio grading (65)<br>○ Guides better decisions (70, 112, 115)<br>○ Provides authentic assessment is a key principle of it (88, 95, 105)<br>○ Provides insights in the various core competencies (94)<br>○ Fair mode of assessment(95) and allows student to defend positions/work (65)<br>○ Provides students with direction and clarity (107, 108)
**Personal and professional development**<br>Promotes self-understanding (75, 116, 117)<br>○ Understanding feelings and thoughts (96, 104)<br>○ Understanding of one's values (88)<br>○ Understanding activity/experience (65, 84, 118)<br>○ Understanding of how one functions in a team (112)<br>○ Fears and stressors feelings of inadequacy (118, 119)<br><br>Curates evidence of skills (94)<br>○ Skills to compile a portfolio (94, 107, 120-122)<br>○ Reflective skills (65, 72, 84, 98, 104, 118, 123)<br>○ Clinical skills (95)<br>Organizes information(98, 103)<br>○ Documentation of progress (73, 94, 100, 102)<br>○ Documentation of skills in different settings(67, 102, 105)<br>Cross-sectional view of one's competence (65, 73, 102, 105)	
**Personalisation**<br>Allows for personalisation (65, 86, 95, 106, 110)	

For peer review only - http://bmjopen.bmj.com/site/about/guidelines.xhtml

○ Personal experiences (65)<br>Choice over the contents (65, 95, 110)	Allows the combination of Summative and Formative assessment (65, 87) and multisource assessments (81, 87, 97, 108) (105) and triangulation (73, 105)<br><br>Facilitates personalised feedback (112) that can be informed by understanding of the individual setting and context (95, 96, 109)
**Benefits to faculty**<br>Improvement of curriculum (79, 88)<br>○ Identify areas of weakness in curriculum (79)<br>○ Provide insights into mentoring, teaching etc (124)<br>Facilitates design of the program (79)<br><br>faculty development<br>○ Understand more about how students acquire competencies (95)<br>○ Insight into the impact of their interventions (115, 124)<br>Insight into their professional conduct, role modelling and pastoral care (117)	
**E-portfolios**<br>Ease of use:<br>○ Easier use (86)<br>○ Accessible (69)<br>Dissemination and editing of information (106)	

Funneled Domain 2: Characteristics

The contents of portfolios were shaped by their overall function (73, 76, 79, 109, 125) (Table 3). Expectations about the nature, and assessments of the contents (79) facilitates personalization of portfolios (69, 73, 100).

Table 3. Portfolio Design and Implementation

Themes	Subthemes	Contents
**Design and Implementation Principles**	**Structure**	Clear guidelines (65, 83, 84, 92, 93, 101, 104)<br>• Use of portfolio (65, 84)<br>• Portfolio content - determining competencies to assess (65, 87)<br>• Promotional decisions (65, 67, 85, 117, 126)<br>Standard-setting exercises (71)<br>Provision of sample portfolios for reference (101, 127, 128)<br>• Roles for assessors (65, 67, 70, 116, 117, 126, 129)<br>• Expectations of students (108)<br>• Duration spanning across preclinical and clinical years (122)<br>Well-defined structure with room for flexibility (65, 82, 84, 86, 101)<br>Student-centered (86)<br>Appropriate student to mentor ratio (67, 121)<br>Sufficient curricular experience for reflection, feedback (67)<br>Protected time for reflection, feedback, and regular meetings with mentors (67, 70, 83, 104)
	**Faculty**	Good faculty support (117, 122)<br>Ensure culture is supportive of portfolio system (67, 122)<br>Flexible and enthusiastic tutors passionate and experienced mentors (81, 84)
	**Curricular Integration**	Early introduction of portfolio (84, 104, 106, 130)

For peer review only - http://bmjopen.bmj.com/site/about/guidelines.xhtml

		Easing portfolio into curriculum (65, 68, 82, 84, 86) – including orientation, training and introductory sessions (65, 68, 84, 86, 101, 117, 128) for learners and staff training (65, 67, 68, 71, 80, 81, 84)<br>Implementation of portfolios throughout curriculum (117)<br>Portfolio content aligning with phase of medical journey and competencies required (65, 67, 86, 93, 126, 128)
	**Assessments**	Clear scoring rubrics (65, 122)<br>Alignment between assessments and outcomes (82, 83, 86)<br>Transparent assessment criteria and outcomes to maintain fairness (67, 68, 106, 127)<br>Standardisation of portfolio use and content (82, 83), assessors training (68) and increased number of assessors (82, 83, 130) to improve assessment reliability (82, 83, 130)<br>Separate processes and reviewers for summative and formative portfolio (67) – summative assessments to specify clear pass/fail requirements (65)<br>Incorporation of student feedback into portfolio development (65, 68, 86)
	**Assessors**	External examiners (65)<br>Faculty members (65, 67, 70, 116, 126, 129)<br>Mentors (103)<br>Senior staff to be paired with new examiners (65)<br>Mentors should not assess own student (82, 88)
	**Assessment Frequency**	Formative assessments to monitor and guide student learning – conducted throughout the year (82, 84, 87)<br>Summative assessments to evaluate learner performance – conducted annually (65, 67, 68, 70, 71, 80, 81, 86, 87, 104, 126, 128, 131-134), twice (67, 71, 112), thrice (84) or multiple times a year (73, 75)
	**Oversight and Delegation**	Establishment of portfolio oversight committee (67, 71)<br>Regular programme evaluation (67)<br>Clear division of support roles – e.g. pedagogical and technical support (106)

Postgraduate CEP assessment portfolios inject more details upon undergraduate portfolios (Table 4). These include communication skills, addressing ethical issues and compliance of professional, institutional and departmental policies (102, 120, 135). Greater detail in postgraduate CEP assessment portfolios sees the addition of modified essay questions (MEQ)s (75), polypharmacy journal/audits (66, 91, 95, 125, 136), professional workshops and training sessions (94, 95, 109, 137, 138), health service meeting reports (75) and reviews by medical regulatory authorities (91).

Table 4. Portfolio Content (Competencies Assessed and Assessment Modalities)

Themes	Subthemes	Content		
		**Communication**	**Ethics**	**Professionalism**
**Portfolio Content**	**Competencies Assessed**	• "Effective" communication (65, 67, 68, 81, 86, 107, 127, 131-133)<br>• Professional behaviour and moral reasoning (71)	• Ethics in interprofessional practice (24, 67, 86, 128, 130, 145, 146)	• Learning from clinical experience (24, 81)<br>• Working in groups/ teamwork (24, 67, 70, 81, 86, 128, 130, 134, 145)

For peer review only - http://bmjopen.bmj.com/site/about/guidelines.xhtml

		• Systems awareness and team-based care (71)<br>• Continuous learning and quality improvement (71)<br>• Patient-centred medical care (71)<br>• Teamwork (70)<br><br>Oral communication skills<br>• Attentive, active listening (84, 139, 140)<br>• Explanation, information giving (127, 139)<br>• History taking (93)<br>• Consultation (141)<br>• Breaking bad news (24, 139)<br>• Shared decision making (142)<br>• Empathetic communication (142)<br>• Taking history and clinical examination (84, 93)<br>• Discerning appropriate language to ensure patient understands (24, 127)<br>• Conflict management (139)<br>• Adaptability to different settings (67)<br><br>Written communication skills<br>• Proper grammar, spelling, logical and coherent structure (103)<br>• Patient cases (93)<br>• Scientific research (93)<br><br>Communication with:<br>• Patients (24, 68, 84, 93, 127, 139, 143)<br>• Children (127)<br>• Family (68, 84, 139, 144)<br>• Colleagues, interprofessional	• Doctor-patient relationship (81, 141, 145)<br>• Values/ ethics (67, 70, 86, 146)<br>• Ethical and legal responsibilities (65, 86)<br>• Moral reasoning (89)<br>• Ethics is assessed as a component of professionalism (85, 87, 88)<br>• Empathy (84)<br>• Showing respect and commitment (84)<br>• Respect for privacy (84)<br>• How to express ethical views appropriately (82)<br>• Identifying unethical moments of practice and grey areas (85, 89, 90)<br><br>Reflection:<br>• Quality of student's reflections (82, 84, 87)<br>• Authenticity of reflection (87)	• Doctor-patient relationship (81, 141, 145)<br>• Becoming a doctor/roles and responsibilities (81, 86, 128, 130, 134, 145, 146)<br>• Patient care (67, 70, 128, 130)<br>• Continuous learning (67, 70)<br>• Values/ ethics (67, 70, 86, 146)<br>• Interpersonal skills/ effective Communication, (24, 67, 128, 145, 146)<br><br>Reflection:<br>• Clinical experience (81)<br>• Teaching (81)<br>• Learning needs in becoming a doctor<br>• Strengths and developmental areas (65, 86)<br>• Understanding of concepts addressed (86)<br>• Dilemmas and doubts (133)

For peer review only - http://bmjopen.bmj.com/site/about/guidelines.xhtml

		team (24, 68, 84, 93, 130, 139)<br>• Public (68)		
	**Assessment Modalities (Portfolio items)**	Formative only (67, 84, 87, 92, 119, 126, 130, 146)<br>Summative only (65, 70, 71, 81-83, 87, 104, 116, 131, 134)<br>Both formative and summative (24, 67, 83, 86, 87, 117, 122, 128, 130, 134)<br>MCQs (65, 68, 87, 95, 126, 141, 143, 144, 146)<br>OSCEs (65, 67, 70, 71, 87, 126, 127, 130, 141, 143, 144, 146)<br>Essays – topical (67, 81, 93, 126)<br>Mini clinical examination exercise (Mini-CEX) (65, 67, 70, 87, 93, 130, 139, 143, 146)<br>Medical/patient presentations/research (67, 68, 80, 86, 93, 121, 126, 129)<br>Workplace-based clinical (67, 70, 71, 80, 83, 93, 107, 112, 122, 126, 127, 130, 139, 143)<br>Case-based discussions, summaries (65, 68, 83, 85, 89, 92, 129)(19)(81, 129) (83, 126, 130)<br>Simulated patient assessment (65, 68, 84, 103, 112)<br>Team-based assessment (65, 83, 86, 93)<br>Logbooks/ Portfolio interview (65, 67, 68, 70, 81-85, 87, 88, 103, 121, 126) (67, 121, 126)<br>Peer evaluation (67, 70, 71, 80, 85, 93, 107, 126, 127)<br>Faculty evaluation (67, 68, 70, 71, 80, 81, 83-86, 117, 126, 131, 147)<br>Feedback reports (75, 77, 80, 86, 91, 93, 109, 112, 122, 126, 130, 134, 139, 143) – multisource feedback (75, 77, 81, 82, 85, 86, 91, 93, 103, 109, 127, 130)<br>Self-assessment/appraisal (67, 68, 80, 82, 93, 104, 126, 130, 134)<br>Self-reflections – written/video/diaries (65, 67, 68, 71, 80-83, 85, 87, 88, 90, 93, 103, 107, 121, 127-130)<br>Learning plans and contracts (67, 68, 71, 80-83, 86, 92, 93, 103, 133)<br>Letters of recommendation (65, 81, 103)<br>Curriculum vitae (65, 67, 70, 83, 86, 122, 126)		

Funneled Domain 3: Strengths and Limitations

The strengths and limitations of portfolios are outlined in Table 5.

<u>Table 5. Strengths and Limitations</u>

Themes	Subthemes	Content
**Strengths/ Limitations**	**Strengths**	<u>For students</u><br>Identification of own strengths and weaknesses (65, 70, 71, 81-84, 86, 88, 92, 96, 134)<br>Appreciation of effective communication (84, 130, 145), ethical issues (88), and fosters critical professional attitudes (83, 140)<br>Encourages self-regulation (134) through opportunities for reflection (82) and self-assessment (84, 104, 117)<br>Facilitates preparation for post-graduate medical training (84, 103, 107)<br><br><u>For faculty</u><br>Identification of areas of concern and allows for early, timely intervention (70, 71)<br>Enhances interaction between students and teachers (65, 84, 88) Facilitates reiterative reassessment of teaching strategies (65)<br><br><u>Assessments</u><br>Longitudinal (112)

For peer review only - http://bmjopen.bmj.com/site/about/guidelines.xhtml

For peer review only

		Good validity (70)<br>Reliable assessment tool – allows for triangulation of information and evidence from multiple sources and contexts (67)<br>Ability to assess a wide range of competencies (86, 93)<br>Allows for more authentic assessments (70)<br>Gives faculty greater insight into students' achievement of behaviourally-oriented competencies that traditional methods do not (71)<br>Evaluates achievement during clinical attachment/clerkship (93)<br>Provides a basis for the judgement of a student's professional fitness to practice (68)
	**Limitations**	Time-consuming and labour intensive (68, 71, 81, 84-87, 92, 103, 122, 130)<br>Issues with reliability (82, 86, 116, 130)<br>Inconsistent effects (82, 86, 104, 146)<br>Lack of honesty by students in reflections (67, 68, 82, 86, 87, 103, 104, 108)

Postgraduate portfolios provide greater insights into lifelong learning and continuous professional development (73, 76, 77, 79, 94, 95, 102, 105, 109, 137). Use of multi-source assessments at multiple timepoints (76) in clinical settings (77, 95) see postgraduate portfolios provide good inter-rater reliability (73, 105). Postgraduate portfolios also detect stress and burnout (137).

Stage 5 of SEBA: Analysis of data and non-data driven literature

Findings from the different stages of SEBA were discussed with members of the expert team and relevant stakeholders. There were concerns from the expert team about the impact of grey literature on the narrative, given that they were neither peer-reviewed nor necessarily evidence-based. As a result, the research team differentiated correspondence, letters, editorials and perspective pieces from academic databases and grey literature from data-driven or research-based peer-reviewed data. Both groups were then independently analysed. The themes/categories identified were then compared to enhance further the accountability and the reproducibility of Stage 5 of SEBA. Evidence-based data from bibliographic databases (henceforth evidence-based publications) were separated from grey literature, perspectives, editorials, letters, and non-data-based articles drawn from bibliographic databases (henceforth non-data driven). These two groups were separately thematically analysed.

Stage 6 of SEBA: Synthesis of the Discussion

The Best Evidence Medical Education (BEME) Collaboration Guide (148) and the Structured approach to the Reporting In healthcare education of Evidence Synthesis (STORIES) (149) were used to guide the discussion.

DISCUSSION

For peer review only - http://bmjopen.bmj.com/site/about/guidelines.xhtml

This SSR in SEBA suggest that overlaps in ethics, professionalism, and communication skills, knowledge, attitudes and competencies reaffirm the concurrent training, and assessments of CEP competencies in portfolios (68, 70, 71). To be effective CEP teaching and longitudinal assessment portfolios require a consistent framework replete with clearly delineated goals (101), aligned expectations, predetermined assessment criteria for specified competencies (65, 67, 68, 73, 79, 81, 83, 92-94, 101, 106, 116, 117, 120, 128, 129, 135), agreed upon endpoints and outcome measures, and the curation, structuring and organisation of longitudinal multisource, multimodal assessment data (66, 67). The combination of this data facilitate training, assessment, feedback and holistic and longitudinal support (71, 80-82) (97) (102, 120, 135, 150), guide reflection (68, 69, 71, 75, 77, 81-91), and bolster professional and personal development (68, 84, 92, 93). Critically the employ of consistent and agreed upon structure, goals, purpose, role, learning objectives, content, and assessment guidelines also affords users with a personalised perspective of their CEP development upon which they can reflect on and glean insights and feedback (82, 98, 127).

Yet to be effective, CEP portfolios require policing and underscores the need for trained (66) and dedicated faculty (84) capable of effective role modelling (117), support (65, 75, 84, 88, 93) and providing feedback (112) and who appreciate the individual's current concepts, narratives, motivations, abilities and availabilities and contextual considerations. Furthermore, the efficacy of portfolios and their ability to meet the stated objectives stated in Table 2 and realise their stated benefits in Table 5 are reliant upon the appropriate mix of assessment methods employed, the frequency of assessments, the efficacy of analysis and the quality of the feedback, support and remediation provided should it be required. This in turn highlights the role of the host organization in ensuring effective oversight of the program and assessments and in ensuring that faculty are afforded protected time for administrative duties, coordination and assessments particularly given the longitudinal nature of CEP portfolios (73, 86, 101, 102, 118, 136, 151, 152). It should not be forgotten that CEP portfolios reflect the development of an individual and such change should be measured and contextualised within the wider education, training, practice, professional, research, clinical, interprofessional team based and organizational perspective. This further underlines the importance of the host organizations role in assessing and guiding development of CEP competencies.

With these requisites met, evidence of changes in CEP knowledge, skills and competencies suggests shift the thinking, attitudes and conduct of clinicians. The Krishna-Pisupati model posits that sustained changes in practice,

For peer review only

For peer review only - http://bmjopen.bmj.com/site/about/guidelines.xhtml

beliefs, values and principles will result in changes in the clinician's belief systems which in turn inform their self-concepts of identity and shape their PIF.

LIMITATIONS

The generalisability of the results of this study is limited by the identified studies selected for review. Firstly, the majority of the papers included originate from Europe and North America. Given that CEP is a sociocultural construct, these geopolitical sociocultural differences raise questions as to the applicability of these findings beyond the European and North American medical education systems. Secondly, as the number of articles included is limited, the SSR in SEBA's ability to assess the long-term effectiveness of portfolios may be compromised.

CONCLUSIONS

The suggestion that CEP portfolios can capture, instruct, and assess PIF requires further study. Whilst there have been posits on how such evaluations could be made, such tools remain unrealised and must be the focus of coming studies. One possible starting point for the design of such tools may be theories such as the Krishna-Pisupati model which attempts to link PIF to changes to belief systems and concepts of personhood. This may provide a grander view of PIF that spans the undergraduate and postgraduate settings and provide the basis for directing support and feedback for clinicians. As we look forward to furthering efforts to study the effects of CEP portfolios upon the PIF of clinicians, we also believe the specific impact of these potentially resource-heavy and financially costly intervention should be at the centre of future initiatives.

ABBREVIATIONS

**CEP**	Communication, Ethics, Professionalism
**PIF**	Professional Identity Formation
**SSR**	Systematic Scoping Review
**SEBA**	Systematic Evidence Based Approach
**PICOS**	Population, Intervention, Comparison and Outcome
**BEME**	Best Evidence Medical Education
**STORIES**	Structured approach to the Reporting In healthcare education of Evidence Synthesis

For peer review only - http://bmjopen.bmj.com/site/about/guidelines.xhtml
For peer review only

STATEMENTS AND DECLARATIONS

Ethics Approval and Consent to Participate NA

Consent for Publication NA

Availability of Data and Materials All data generated or analysed during this review are included in this published article and its appendices.

Competing Interests JJQT, GP, DZH, BKYL, AJSL, EJXC, AP, RT, JYHY, YZK, CWNQ, JYL, KTT, YTO, MC, JZ, SM, LW and LKRK have no competing interests.

Funding No funding was received for this review

Author Contributions JJQT, GP, DZH, BKYL, AJSL, EJXC, AP, RT, JYHY, YZK, CWNQ, JYL, KTT, YTO, MC, JZ, SM, LW and LKRK were involved in data curation, formal analysis, investigation, preparing the original draft of the manuscript as well as reviewing and editing the manuscript. All authors have read and approved the manuscript.

ACKNOWLEDGEMENTS

The authors would like to dedicate this paper to the late Dr. S Radha Krishna and A/Prof Cynthia Goh whose advice and ideas were integral to the success of this review and to Thondy, Maia Olivia and Raja Kamarul Ariffin whose lives continue to inspire us. The authors would also like to thank the anonymous reviewers, Dr Ruaraidh Hill and Dr Stephen Mason for their help comments which greatly enhanced this manuscript.

For peer review only

REFERENCES

1. Rankings TU. QS World University Rankings 2020 [Available from: https://www.topuniversities.com/university-rankings/university-subject-rankings/2020/medicine.
2. Deshpande AD. The Use of Sociocultural Constructs in Cancer Screening Research Among African Americans. Cancer Control. 2009;16(3):10.
3. MZJ H. Chinese Familial Tradition and Western Influences: A Case Study in Singapore on Decision Making at the end of life. . Journal of Pain and Symptom Management. 2010;40(6):6.
4. Foo WT, Zheng Y, Kwee AK, Yang GM, Krishna L. Factors considered in end-of-life care decision making by health care professionals. Am J Hosp Palliat Care. 2013;30(4):354-8.
5. Chong JA, Quah YL, Yang GM, Menon S, Radha Krishna LK. Patient and family involvement in decision making for management of cancer patients at a centre in Singapore. BMJ Support Palliat Care. 2015;5(4):420-6.
6. Chai HZ, Krishna LK, Wong VH. Feeding: what it means to patients and caregivers and how these views influence Singaporean Chinese caregivers' decisions to continue feeding at the end of life. Am J Hosp Palliat Care. 2014;31(2):166-71.
7. Marc Ho ZJ, Krishna LKR, Goh C, Alethea Yee CP. The physician–patient relationship in treatment decision making at the end of life: A pilot study of cancer patients in a Southeast Asian society. Palliative and Supportive Care. 2013;11(1):13-9.
8. Krishna LKR. Decision making at the end of life: A Singaporean Perspective. Asian Bioeth Rev. 2011;3(2):6.
9. Loh AZ, Tan JS, Jinxuan T, Lyn TY, Krishna LK, Goh CR. Place of Care at End of Life: What Factors Are Associated With Patients' and Their Family Members' Preferences? Am J Hosp Palliat Care. 2016;33(7):669-77.
10. Krishna LK, Watkinson DS, Beng NL. Limits to relational autonomy--the Singaporean experience. Nurs Ethics. 2015;22(3):331-40.
11. Krishna LK, Alsuwaigh R, Miti PT, Wei SS, Ling KH, Manoharan D. The influence of the family in conceptions of personhood in the palliative care setting in Singapore and its influence upon decision making. Am J Hosp Palliat Care. 2014;31(6):645-54.
12. Krishna L. The position of the family of palliative care patients within the decision-making process at the end of life in Singapore. Ethics & Medicine: An International Journal of Bioethics. 2011;27:183+.
13. Hong DZ, Goh JL, Ong ZY, Ting JJQ, Wong MK, Wu J, et al. Postgraduate ethics training programs: a systematic scoping review. BMC Med Educ. 2021;21(1):338.
14. Wong MK, Hong DZH, Wu J, Ting JJQ, Goh JL, Ong ZY, et al. A systematic scoping review of undergraduate medical ethics education programs from 1990 to 2020. Med Teach. 2022;44(2):167-86.
15. Yoon NYS, Ong YT, Yap HW, Tay KT, Lim EG, Cheong CWS, et al. Evaluating assessment tools of the quality of clinical ethics consultations: a systematic scoping review from 1992 to 2019. BMC Med Ethics. 2020;21(1):51.
16. Bok C, Ng CH, Koh JWH, Ong ZH, Ghazali HZB, Tan LHE, et al. Interprofessional communication (IPC) for medical students: a scoping review. BMC Med Educ. 2020;20(1):372.

For peer review only - http://bmjopen.bmj.com/site/about/guidelines.xhtml

17. Chia E, Huang H, Goh S, Peries M, Lee C, Tan L, et al. A systematic scoping review of teaching and evaluating communications in the intensive care unit. The Asia Pacific Scholar. 2021;6:3-29.
18. Malhotra C, Kanesvaran R, Krishna L, Xiang L, Kumarakulasinghe NB, Tan SH, et al. Oncologists' responses to patient and caregiver negative emotions and patient perception of quality of communication: results from a multi-ethnic Asian setting. Support Care Cancer. 2018;26(3):957-65.
19. Ng CH, Ong ZH, Koh JWH, Ang RZE, Tan LHS, Tay KT, et al. Enhancing Interprofessional Communications Training in Internal Medicine. Lessons Drawn From a Systematic Scoping Review From 2000 to 2018. J Contin Educ Health Prof. 2020;40(1):27-35.
20. Ong ZH, Tan LHE, Ghazali HZB, Ong YT, Koh JWH, Ang RZE, et al. A Systematic Scoping Review on Pedagogical Strategies of Interprofessional Communication for Physicians in Emergency Medicine. J Med Educ Curric Dev. 2021;8:23821205211041794.
21. Tan XH, Foo MA, Lim SLH, Lim M, Chin AMC, Zhou J, et al. Teaching and assessing communication skills in the postgraduate medical setting: a systematic scoping review. BMC Med Educ. 2021;21(1):483.
22. Venktaramana V, Loh EKY, Wong CJW, Yeo JW, Teo AYT, Chiam CSY, et al. A systematic scoping review of communication skills training in medical schools between 2000 and 2020. Med Teach. 2022;44(9):997-1006.
23. Ong YT, Kow CS, Teo YH, Tan LHE, Abdurrahman A, Quek NWS, et al. Nurturing professionalism in medical schools. A systematic scoping review of training curricula between 1990-2019. Med Teach. 2020;42(6):636-49.
24. Tay KT, Ng S, Hee JM, Chia EWY, Vythilingam D, Ong YT, et al. Assessing Professionalism in Medicine - A Scoping Review of Assessment Tools from 1990 to 2018. J Med Educ Curric Dev. 2020;7:2382120520955159.
25. Teo YH, Peh TY, Abdurrahman A, Lee ASI, Chiam M, Fong W, et al. A modified Delphi approach to enhance nurturing of professionalism in postgraduate medical education in Singapore. Singapore Med J. 2021.
26. The Student-Physician: Introductory Studies in the Sociology of Medical Education. Journal of the American Medical Association. 1958;166(1):106-7.
27. Teo KJH, Teo MYK, Pisupati A, Ong RSR, Goh CK, Seah CHX, et al. Assessing professional identity formation (PIF) amongst medical students in Oncology and Palliative Medicine postings: a SEBA guided scoping review. BMC Palliat Care. 2022;21(1):200.
28. Quah ELY, Chua KZY, Lua JK, Wan DWJ, Chong CS, Lim YX, et al. A Systematic Review of Stakeholder Perspectives of Dignity and Assisted Dying. J Pain Symptom Manage. 2022.
29. Toh RQE, Koh KK, Lua JK, Wong RSM, Quah ELY, Panda A, et al. The role of mentoring, supervision, coaching, teaching and instruction on professional identity formation: a systematic scoping review. BMC Med Educ. 2022;22(1):531.
30. Ho CY, Kow CS, Chia CHJ, Low JY, Lai YHM, Lauw SK, et al. The impact of death and dying on the personhood of medical students: a systematic scoping review. BMC Med Educ. 2020;20(1):516.
31. Sarraf-Yazdi S, Teo YN, How AEH, Teo YH, Goh S, Kow CS, et al. A Scoping Review of Professional Identity Formation in Undergraduate Medical Education. J Gen Intern Med. 2021;36(11):3511-21.
32. Vig PS, Lim JY, Lee RWL, Huang H, Tan XH, Lim WQ, et al. Parental bereavement - impact of death of neonates and children under 12 years on personhood of parents: a systematic scoping review. BMC Palliat Care. 2021;20(1):136.
33. Chan NPX, Chia JL, Ho CY, Ngiam LXL, Kuek JTY, Ahmad Kamal NHB, et al. Extending the Ring Theory of Personhood to the Care of Dying Patients in Intensive Care Units. Asian Bioeth Rev. 2022;14(1):71-86.
34. Ngiam LXL, Ong YT, Ng JX, Kuek JTY, Chia JL, Chan NPX, et al. Impact of Caring for Terminally Ill Children on Physicians: A Systematic Scoping Review. Am J Hosp Palliat Care. 2021;38(4):396-418.
35. Chia EWY, Tay KT, Xiao S, Teo YH, Ong YT, Chiam M, et al. The Pivotal Role of Host Organizations in Enhancing Mentoring in Internal Medicine: A Scoping Review. J Med Educ Curric Dev. 2020;7:2382120520956647.
36. Kuek JTY, Ngiam LXL, Kamal NHA, Chia JL, Chan NPX, Abdurrahman A, et al. The impact of caring for dying patients in intensive care units on a physician's personhood: a systematic scoping review. Philos Ethics Humanit Med. 2020;15(1):12.
37. Zhou YC, Tan SR, Tan CGH, Ng MSP, Lim KH, Tan LHE, et al. A systematic scoping review of approaches to teaching and assessing empathy in medicine. BMC Med Educ. 2021;21(1):292.
38. Tay KT, Tan XH, Tan LHE, Vythilingam D, Chin AMC, Loh V, et al. A systematic scoping review and thematic analysis of interprofessional mentoring in medicine from 2000 to 2019. J Interprof Care. 2021;35(6):927-39.
39. Tan R, Qi Ting JJ, Zhihao Hong D, Sing Lim AJ, Ong YT, Pisupati A, et al. Medical Student Portfolios: A Systematic Scoping Review. J Med Educ Curric Dev. 2022;9:23821205221076022.

For peer review only - http://bmjopen.bmj.com/site/about/guidelines.xhtml

40. Kow CS, Teo YH, Teo YN, Chua KZY, Quah ELY, Kamal N, et al. A systematic scoping review of ethical issues in mentoring in medical schools. BMC Med Educ. 2020;20(1):246.
41. Huang H, Toh RQE, Chiang CLL, Thenpandiyan AA, Vig PS, Lee RWL, et al. Impact of Dying Neonates on Doctors' and Nurses' Personhood: A Systematic Scoping Review. J Pain Symptom Manage. 2022;63(1):e59-e74.
42. Hong DZ, Lim AJS, Tan R, Ong YT, Pisupati A, Chong EJX, et al. A Systematic Scoping Review on Portfolios of Medical Educators. J Med Educ Curric Dev. 2021;8:23821205211000356.
43. Ho S, Tan YY, Neo SHS, Zhuang Q, Chiam M, Zhou JX, et al. COVID-19 - A Review of the Impact it has made on Supportive and Palliative Care Services Within a Tertiary Hospital and Cancer Centre in Singapore. Ann Acad Med Singap. 2020;49(7):489-95.
44. Hee J, Toh YL, Yap HW, Toh YP, Kanesvaran R, Mason S, et al. The development and design of a framework to match mentees and mentors through a systematic review and thematic analysis of mentoring programs between 2000 and 2015. Mentoring & Tutoring: Partnership in Learning. 2020;28:340-64.
45. Chua WJ, Cheong CWS, Lee FQH, Koh EYH, Toh YP, Mason S, et al. Structuring Mentoring in Medicine and Surgery. A Systematic Scoping Review of Mentoring Programs Between 2000 and 2019. J Contin Educ Health Prof. 2020;40(3):158-68.
46. Chua KZY, Quah ELY, Lim YX, Goh CK, Lim J, Wan DWJ, et al. A systematic scoping review on patients' perceptions of dignity. BMC Palliat Care. 2022;21(1):118.
47. Quek CWN, Ong RRS, Wong RSM, Chan SWK, Chok AK-L, Shen GS, et al. Systematic scoping review on moral distress among physicians. BMJ Open. 2022;12(9):e064029.
48. Ong RSR, Wong RSM, Chee RCH, Quek CWN, Burla N, Loh CYL, et al. A systematic scoping review moral distress amongst medical students. BMC Med Educ. 2022;22(1):466.
49. Krishna LKR, Tan LHE, Ong YT, Tay KT, Hee JM, Chiam M, et al. Enhancing Mentoring in Palliative Care: An Evidence Based Mentoring Framework. Journal of Medical Education and Curricular Development. 2020;7:2382120520957649.
50. Kamal NHA, Tan LHE, Wong RSM, Ong RRS, Seow REW, Loh EKY, et al. Enhancing education in Palliative Medicine: the role of Systematic Scoping Reviews. Palliative Medicine & Care: Open Access. 2020;7(1):1-11.
51. Ong RRS, Seow REW, Wong RSM, Loh EKY, Kamal NHA, Mah ZH, et al. A Systematic Scoping Review of Narrative Reviews in Palliative Medicine Education. Palliative Medicine & Care: Open Access. 2020;7(1):1-22.
52. Mah ZH, Wong RSM, Seow REW, Loh EKY, Kamal NHA, Ong RRS, et al. A Systematic Scoping Review of Systematic Reviews in Palliative Medicine Education. Palliative Medicine & Care: Open Access. 2020;7(1):1-12.
53. Cleland J, McKimm J, Fuller R, Taylor D, Janczukowicz J, Gibbs T. Adapting to the impact of COVID-19: Sharing stories, sharing practice. Medical Teacher. 2020;42(7):772-5.
54. Peters M, Godfrey C, McInerney P, Soares C, Khalil H, Parker D. The Joanna Briggs Institute reviewers' manual 2015: methodology for JBI scoping reviews2015 April 29, 2019. Available from: http://joannabriggs.org/assets/docs/sumari/Reviewers-Manual_Methodology-for-JBI-Scoping-Reviews_2015_v1.pdf.
55. Peters MD, Godfrey CM, Khalil H, McInerney P, Parker D, Soares CB. Guidance for conducting systematic scoping reviews. Int J Evid Based Healthc. 2015;13(3):141-6.
56. Pham MT, Rajić A, Greig JD, Sargeant JM, Papadopoulos A, McEwen SA. A scoping review of scoping reviews: advancing the approach and enhancing the consistency. Res Synth Methods. 2014;5(4):371-85.
57. Sandelowski M, Barroso J. Handbook for synthesizing qualitative research: springer publishing company; 2006.
58. Reed DA, Beckman TJ, Wright SM, Levine RB, Kern DE, Cook DA. Predictive validity evidence for medical education research study quality instrument scores: quality of submissions to JGIM's Medical Education Special Issue. J Gen Intern Med. 2008;23(7):903-7.
59. Tong A, Sainsbury P, Craig J. Consolidated criteria for reporting qualitative research (COREQ): a 32-item checklist for interviews and focus groups. Int J Qual Health Care. 2007;19(6):349-57.
60. Wong G, Greenhalgh T, Westhorp G, Buckingham J, Pawson R. RAMESES publication standards: meta-narrative reviews. BMC medicine. 2013;11(1):20.
61. Popay J, Roberts H, Sowden A, Petticrew M, Arai L, Rodgers M, et al. Guidance on the conduct of narrative synthesis in systematic reviews. A product from the ESRC methods programme Version. 2006;1:b92.
62. Rider EA, Hinrichs MM, Lown BA. A model for communication skills assessment across the undergraduate curriculum. Med Teach. 2006;28(5):e127-34.
63. Goldie J. Review of ethics curricula in undergraduate medical education. Med Educ. 2000;34(2):108-19.

For peer review only - http://bmjopen.bmj.com/site/about/guidelines.xhtml

64. Duffy FD, Gordon GH, Whelan G, Cole-Kelly K, Frankel R, Buffone N, et al. Assessing competence in communication and interpersonal skills: the Kalamazoo II report. Acad Med. 2004;79(6):495-507.
65. David M, Davis M, Harden R, Howie P, Ker J, Pippard M. AMEE Medical Education Guide No. 24: Portfolios as a method of student assessment. Medical teacher. 2001;23:535-51.
66. McEwen L, Griffiths J, Schultz K. Developing and Successfully Implementing a Competency-Based Portfolio Assessment System in a Postgraduate Family Medicine Residency Program. Academic medicine : journal of the Association of American Medical Colleges. 2015;90.
67. Dannefer E, Henson L. The Portfolio Approach to Competency-Based Assessment at the Cleveland Clinic Lerner College of Medicine. Academic medicine : journal of the Association of American Medical Colleges. 2007;82:493-502.
68. Davis M, Ben-David M, Harden R, Howie P, Ker J, McGhee C, et al. Portfolio assessment in medical students' final examination. Medical teacher. 2001;23:357-66.
69. Ruiz J, Qadri S, Karides M, Castillo C, Milanez M, Roos B. Fellows' Perceptions of a Mandatory Reflective Electronic Portfolio in a Geriatric Medicine Fellowship Program. Educational Gerontology - EDUC GERONTOL. 2009;35:634-52.
70. O'Brien C, Thomas J, Green M. What Is the Relationship Between a Preclerkship Portfolio Review and Later Performance in Clerkships? Academic medicine : journal of the Association of American Medical Colleges. 2017;93.
71. O'Brien C, Sanguino S, Thomas J, Green M. Feasibility and Outcomes of Implementing a Portfolio Assessment System Alongside a Traditional Grading System. Academic medicine : journal of the Association of American Medical Colleges. 2016;91.
72. Jenkins L, Mash B, Derese A. The national portfolio of learning for postgraduate family medicine training in South Africa: Experiences of registrars and supervisors in clinical practice. BMC medical education. 2013;13:149.
73. Shumway J, Harden R. AMEE Guide No. 25: The assessment of learning outcomes for the competent and reflective physician. Medical teacher. 2003;25:569-84.
74. Daboval T, Moore G, Ferretti E. How we teach ethics and communication during a Canadian neonatal perinatal medicine residency: An interactive experience. Medical teacher. 2012;35.
75. Jenkins L, Mash B, Derese A. Development of a portfolio of learning for postgraduate family medicine training in South Africa: A Delphi study. BMC family practice. 2012;13:11.
76. Colbert C, Ownby A, Butler P. A Review of Portfolio Use in Residency Programs and Considerations before Implementation. Teaching and learning in medicine. 2008;20:340-5.
77. Wakeling J, Holmes S, Boyd A, Tredinnick-Rowe J, Cameron N, Marshall M, et al. Reflective Practice for Patient Benefit: An Analysis of Doctors' Appraisal Portfolios in Scotland. Journal of Continuing Education in the Health Professions. 2019;39:1.
78. Torbeck L. A Method for Defining Competency-Based Promotion Criteria for Family Medicine Residents. Academic medicine : journal of the Association of American Medical Colleges. 2005;80:832-9.
79. O'Sullivan P, Reckase M, McClain T, Savidge M, Clardy J. Demonstration of Portfolios to Assess Competency of Residents. Advances in health sciences education : theory and practice. 2004;9:309-23.
80. Yielder J, Moir F. Assessing the Development of Medical Students' Personal and Professional Skills by Portfolio. Journal of Medical Education and Curricular Development. 2016;2016:9.
81. O'Sullivan A, Howe A, Miles S, Harris P, Hughes C, Jones P, et al. Does a summative portfolio foster the development of capabilities such as reflective practice and understanding ethics? An evaluation from two medical schools. Medical teacher. 2012;34:e21-8.
82. Driessen E, Tartwijk J, Vermunt J, Van der Vleuten C. Use of Portfolios in early undergraduate medical training. Medical teacher. 2003;25:18-23.
83. Haffling A-C, Beckman A, Pahlmblad A, Edgren G. Students' reflections in a portfolio pilot: Highlighting professional issues. Medical teacher. 2010;32:e532-40.
84. Fida N, Hassanien M, Shamim M, Alafari R, Zaini R, Mufti S, et al. Students' perception of portfolio as a learning tool at King Abdulaziz University Medical School. Medical Teacher. 2018;40:1-10.
85. Goldie J, Dowie A, Cotton P, Morrison J. Teaching professionalism in the early years of a medical curriculum: A qualitative study. Medical education. 2007;41:610-7.
86. O'Sullivan A, Harris P, Hughes C, Toohey S, Balasooriya C, Velan G, et al. Linking assessment to undergraduate student capabilities through portfolio examination. Assessment & Evaluation in Higher Education. 2012;37:379-91.
87. Franco R, Franco C, Pestana O, Severo M, Ferreira M. The use of portfolios to foster professionalism: attributes, outcomes, and recommendations. Assessment & Evaluation in Higher Education. 2016;42:1-19.
88. Gordon J. Assessing students' personal and professional development using portfolios and interviews. Medical education. 2003;37:335-40.

For peer review only - http://bmjopen.bmj.com/site/about/guidelines.xhtml

89. Wong A, Trollope-Kumar K. Reflections: An inquiry into medical students' professional identity formation. Medical education. 2014;48:489-501.
90. Borgstrom E, Cohn S, Barclay S. Medical Professionalism: Conflicting Values for Tomorrow's Doctors. Journal of general internal medicine. 2010;25:1330-6.
91. Lockyer J, Sockalingam S, Campbell DC. Assessment and Change: An Exploration of Documented Assessment Activities and Outcomes by Canadian Psychiatrists. Journal of Continuing Education in the Health Professions. 2018;38:1.
92. Sambandam E, Jutti R, Lee L. Portfolio as a learning tool: Students' perspective. Annals of the Academy of Medicine, Singapore. 2005;34:511-4.
93. Michels N, Avonts M, Peeraer G, Ulenaers K, Gaal L, Bossaert L, et al. Content validity of workplace-based portfolios: A multi-centre study. Medical Teacher. 2016;38:1-10.
94. Finlay K, Probyn L, Ho S. The CanMEDS Resume: A Useful Educational Portfolio Tool for Diagnostic Radiology Residents. Canadian Association of Radiologists journal = Journal l'Association canadienne des radiologistes. 2011;63:233-6.
95. Clay A, Petrusa E, Harker M, Andolsek K. Development of a web-based, specialty specific portfolio. Medical teacher. 2007;29:311-6.
96. Ozcakar N, Mevsim V, Dilek G. Use of Portfolios in Undergraduate Medical Training: First Meeting With a Patient. Trakya Universitesi Tip Fakultesi Dergisi. 2009;26.
97. Bhattarai M. Study skills course in medical education for postgraduate residents. Kathmandu University medical journal (KUMJ). 2006;5:561-5.
98. Amsellem-Ouazana D, Pee D, Godin V. Use of portfolios as a learning and assessment tool in a surgical practical session of urology during undergraduate medical training. Medical teacher. 2006;28:356-9.
99. Watling C, Brown J. Education Research: Communication skills for neurology residents: Structured teaching and reflective practice. Neurology. 2007;69:E20-6.
100. Goliath C. Diffusion of an E-Portfolio to Assist in the Self-Directed Learning of Physicians: An Exploratory Study. 2009.
101. Azer S. Use of Portfolios by Medical Students: Significance of Critical Thinking. The Kaohsiung journal of medical sciences. 2008;24:361-6.
102. Cheung R. ECLIPPx: An innovative model for reflective portfolios in life-long learning. The clinical teacher. 2011;8:27-30.
103. Rees C, Shepherd M, Chamberlain S. The utility of reflective portfolios as a method of assessing first year medical students' personal and professional development. Reflective Practice. 2005;6:3-14.
104. Driessen E, Tartwijk J, Overeem K, Vermunt J, Van der Vleuten C. Conditions for reflective use of portfolios in undergraduate medical education. Medical education. 2006;39:1230-5.
105. Gavriel J. Portfolios and 'the competent clinician': Past, present and future assessment. Education for Primary Care. 2016;27:1-4.
106. Moores A, Parks M. Twelve tips for introducing E-Portfolios with undergraduate students. Medical teacher. 2010;32:46-9.
107. Rees C, Sheard C. Undergraduate medical students' views about a reflective portfolio assessment of their communication skills. Medical education. 2004;38:125-8.
108. Chaffey L, de Leeuw E, Finnigan G. Facilitating Students' Reflective Practice in a Medical Course: Literature Review. Education for health (Abingdon, England). 2011;25:198-203.
109. Pinsky L, Edwards K. Diving for PERLS: Working and performance portfolios for evaluation and reflection on learning. Journal of general internal medicine. 2004;19:582-7.
110. Gibson C, Chandratilake M, Hull A. Use of portfolios for assessment of global health residents: qualitative evaluation of design and implementation. Canadian medical education journal. 2018;9:e20-e32.
111. Roberts C, Shadbolt N, Clark T, Simpson P. The reliability and validity of a portfolio designed as a programmatic assessment of performance in an integrated clinical placement. BMC Medical Education. 2014;14:197.
112. Dolan B, O'Brien C, Cameron K, Green M. A Qualitative Analysis of Narrative Preclerkship Assessment Data to Evaluate Teamwork Skills. Teaching and Learning in Medicine. 2018;30:1-9.
113. Fins J, Kodish E, Cohn F, Danis M, Derse A, Dubler N, et al. A Pilot Evaluation of Portfolios for Quality Attestation of Clinical Ethics Consultants. The American journal of bioethics : AJOB. 2016;16:15-24.
114. Naidu T, Ramlall S. Establishing psychiatric registrars' competence in psychotherapy: A portfolio based model. African journal of psychiatry. 2008;11:264-71.
115. Murphy D, Aitchison P, Hernandez Santiago V, Davey P, Mires G, Nathwani D. Insightful Practice: A robust measure of medical students' professional response to feedback on their performance. BMC Medical Education. 2015;15.
116. Ekayanti F, Risahmawati R, Fadhilah M. Portfolio Assessment Implementation in Clinical Year of Community Medicine Module: Students Perspective2017.

For peer review only - http://bmjopen.bmj.com/site/about/guidelines.xhtml

117. Hall P, Byszewski A, Sutherland S, Stodel E. Developing a Sustainable Electronic Portfolio (ePortfolio) Program That Fosters Reflective Practice and Incorporates CanMEDS Competencies Into the Undergraduate Medical Curriculum. Academic medicine : journal of the Association of American Medical Colleges. 2012;87:744-51.
118. Menon V. Portfolios for post graduate psychiatry residents: Potentials and pitfalls. Asian Journal of Psychiatry. 2014;11.
119. Prayson R, Bierer SB, Dannefer E. Medical student resilience strategies: A content analysis of medical students' portfolios. Perspectives on medical education. 2016;6.
120. Fragneto R, Dilorenzo A, Schell R, Bowe E. Evaluating Practice-Based Learning and Improvement: Efforts to Improve Acceptance of Portfolios. Journal of graduate medical education. 2010;2:638-43.
121. Austin C, Braidman I. Support for portfolio in the initial years of the undergraduate medical school curriculum: What do the tutors think? Medical teacher. 2008;30:265-71.
122. Arntfield S, Parlett B, Meston C, Apramian T, Lingard L. A model of engagement in reflective writing-based portfolios: Interactions between points of vulnerability and acts of adaptability. Medical teacher. 2015;38:1-10.
123. Pitkälä K, Mäntyranta T. Feelings related to first patient experiences in medical school: A qualitative study on students' personal portfolios. Patient education and counseling. 2004;54:171-7.
124. Tigelaar D, Dolmans D, Grave W, Wolfhagen I, Van der Vleuten C. Portfolio as a tool to stimulate teachers' reflections. Medical teacher. 2006;28:277-82.
125. Singh R, Naughton B, Taylor J, Koenigsberg M, Anderson D, McCausland L, et al. A comprehensive collaborative patient safety residency curriculum to address the ACGME core competencies. Medical education. 2006;39:1195-204.
126. Dannefer E, Bierer SB, Gladding S. Evidence within a portfolio-based assessment program: What do medical students select to document their performance? Medical teacher. 2012;34:215-20.
127. Rees C, Sheard C. The reliability of assessment criteria for undergraduate medical students' communication skills portfolios: The Nottingham experience. Medical education. 2004;38:138-44.
128. Kassab S, Bidmos M, Nomikos M, Daher-Nashif S, Kane T, Sarangi S, et al. Construct Validity of an Instrument for Assessment of Reflective Writing-Based Portfolios of Medical Students. Advances in Medical Education and Practice. 2020;Volume 11:397-404.
129. Michels N, Driessen E, Muijtjens A, Van Gaal L, Bossaert L, De Winter B. Portfolio assessment during medical internships: How to obtain a reliable and feasible assessment procedure? Education for health (Abingdon, England). 2009;22:313.
130. Susan E, Kinnair D. Integrating the assessment of interprofessional education into the health care curriculum. Journal of Taibah University Medical Sciences. 2016;11.
131. Bierer SB, Dannefer E. Does Students' Gender, Citizenship, or Verbal Ability Affect Fairness of Portfolio-Based Promotion Decisions? Results From One Medical School. Academic medicine : journal of the Association of American Medical Colleges. 2011;86:773-7.
132. Bierer SB, Dannefer E, Tetzlaff J. Time to Loosen the Apron Strings: Cohort-based Evaluation of a Learner-driven Remediation Model at One Medical School. Journal of General Internal Medicine. 2015.
133. Dannefer E. Beyond assessment of learning toward assessment for learning: Educating tomorrow's physicians. Medical teacher. 2013;35.
134. Dannefer E, Prayson R. Supporting students in self-regulation: Use of formative feedback and portfolios in a problem-based learning setting. Medical teacher. 2013;35.
135. Deitte L. Learning Portfolios in Radiology Residency Education: How Do I Get Started? Journal of the American College of Radiology : JACR. 2008;5:664-9.
136. Vance G, Williamson A, Frearson R, O'Connor N, Davison J, Steele C, et al. Evaluation of an established learning portfolio. The clinical teacher. 2013;10:21-6.
137. Suraseranivongse S, Chinachoti T, Aroonpruksakul N, Halilamien P, Rushatamukayanunt P, Raksamani K, et al. Development of a learning portfolio to assess the competency of anesthesia residents in Thailand. Asian Biomedicine. 2011;5.
138. Yates J, James D. Risk factors at medical school for subsequent professional misconduct: multicentre retrospective case-control study. BMJ. 2010;340:c2040.
139. Douglas AH, Acharya SP, Allery LA. Communication skills teaching and learning in Nepal; what are medical students' perceptions and experiences? A qualitative study. BMC Med Educ. 2020;20(1):391.
140. Remein CD, Childs E, Pasco JC, Trinquart L, Flynn DB, Wingerter SL, et al. Content and outcomes of narrative medicine programmes: a systematic review of the literature through 2019. BMJ Open. 2020;10(1):e031568.
141. Ament Giuliani Franco C, Franco RS, Cecilio-Fernandes D, Severo M, Ferreira MA, de Carvalho-Filho MA. Added value of assessing medical students' reflective writings in communication skills training: a longitudinal study in four academic centres. BMJ Open. 2020;10(11):e038898.

For peer review only - http://bmjopen.bmj.com/site/about/guidelines.xhtml

142. Cave J, Dacre J, Jones A. Educating Undergraduate Medical Students About Oncology: A Literature Review. Journal of clinical oncology : official journal of the American Society of Clinical Oncology. 2006;24:1932-9.
143. Brits H, Bezuidenhout J, Van der Merwe LJ, Joubert G. Assessment practices in undergraduate clinical medicine training: What do we do and how we can improve? Afr J Prim Health Care Fam Med. 2020;12(1):e1-e7.
144. Rajeh N, Grant J, Farsi J, Tekian A. Contextual Analysis of Stakeholder Opinion on Management and Leadership Competencies for Undergraduate Medical Education: Informing Course Design. J Med Educ Curric Dev. 2020;7:2382120520948866.
145. Peeters MJ, Sexton ME. Use of reflective writing within interprofessional education: a mixed-methods analysis. J Interprof Care. 2020;34(3):307-14.
146. Younas A, Ansar A. DEVELOPING A CURRICULUM FOR PROFESSIONAL ETHICS IN UNDERGRADUATE MEDICAL AND DENTAL EDUCATION: EVIDENCE FROM LITERATURE. 2020;70:1211-3.
147. García-Estañ J, Santonja F, Sanz M, Martínez F, Rueda D. Portfolio as a tool to evaluate clinical competences of traumatology in medical students. Advances in Medical Education and Practice. 2016;7:57.
148. Haig A, Dozier M. BEME Guide no 3: systematic searching for evidence in medical education--Part 1: Sources of information. Medical teacher. 2003;25(4):352-63.
149. Gordon M, Gibbs T. STORIES statement: publication standards for healthcare education evidence synthesis. BMC medicine. 2014;12(1):143.
150. Dawe R, Pike A, Kidd M, Janakiram P, Nicolle E, Allison J. Enhanced skills in global health and health equity: Guidelines for curriculum development. Canadian Medical Education Journal. 2017;8:e48-e60.
151. Sagasser M, Kramer A, Van der Vleuten C. How do postgraduate GP trainees regulate their learning and what helps and hinders them? A qualitative study. BMC medical education. 2012;12:67.
152. Burch V. Portfolios for assessment and learning: Guide Supplement 45.1 – Viewpoint. Medical teacher. 2011;33:1029-31.

Appendices

Appendix A. SEBA Methodology

Appendix B. Tabulated Summaries

Figure Legend

Figure 2. The SEBA Process

Figure 2. PRISMA Flowchart

For peer review only

For peer review only - http://bmjopen.bmj.com/site/about/guidelines.xhtml

Figure 1. *The SEBA Process*

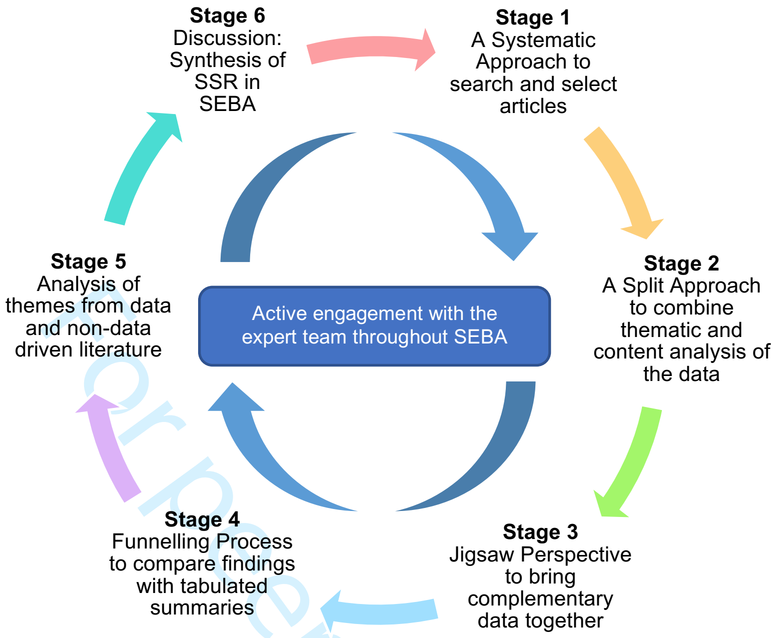

Figure 2. *PRISMA Flowchart*

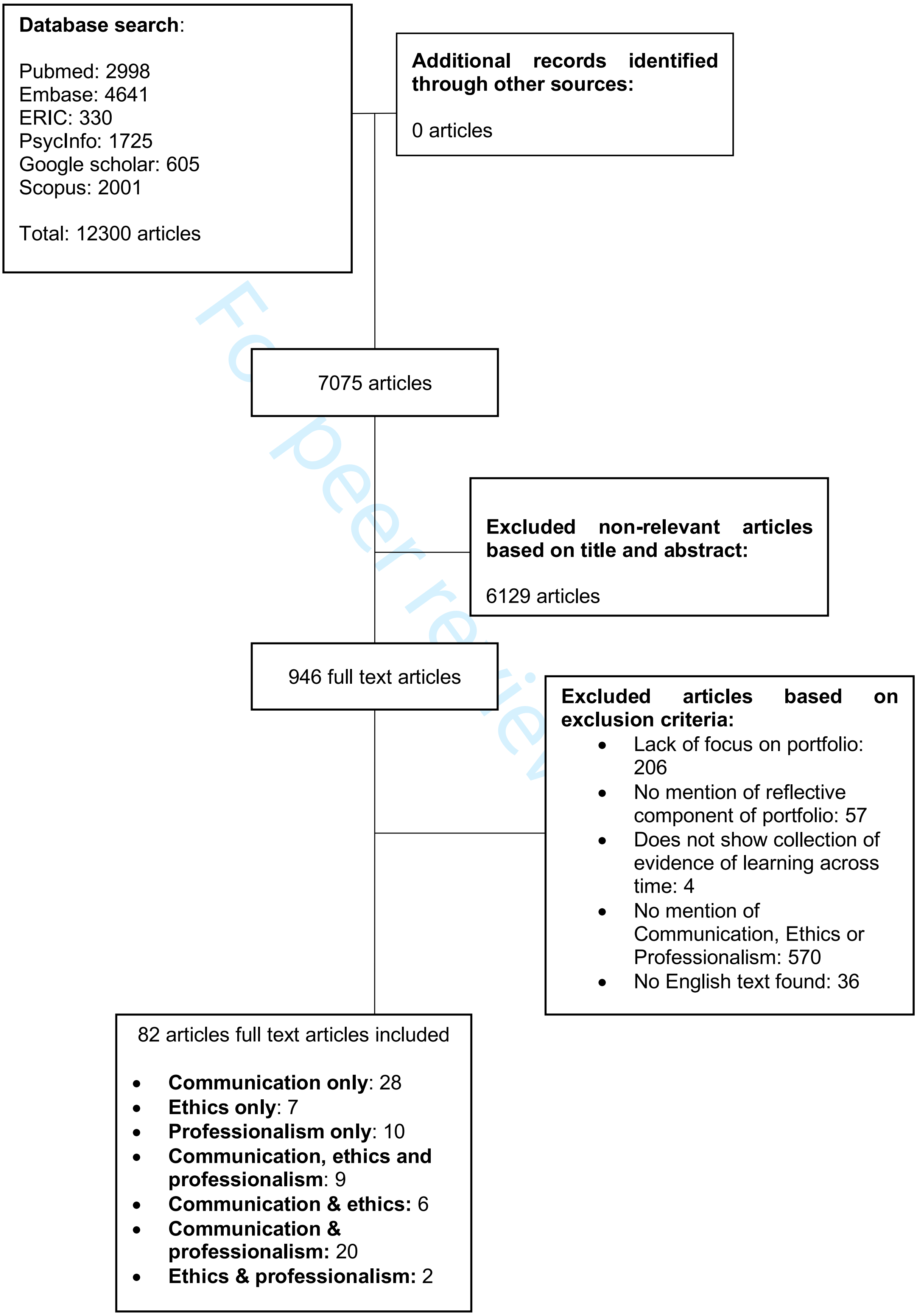

For peer review

Appendices

Appendix A. SEBA Methodology

Theoretical lens

Krishna's Systematic Evidence-Based Approach (SEBA) is adopted to guide this systematic scoping review (SSR) (henceforth SSR in SEBA) (1-8). SEBA is composed of six stages- 1) Systematic Approach, 2) Split Approach, 3) Jigsaw Perspective, 4) Funnelling Process 5) Analysis of data and non-data driven literature, and 6) Discussion Synthesis (Figure 1). SEBA systematically map available data, structure the extraction of key characteristics of CEP education and its effects, synthesise and summarise actionable and applicable information across a diverse range of study formats and identify gaps in knowledge in current concepts. SEBA's constructivist approach (8-13) and relativist lens (14-17) acknowledges development of CEP competencies are individualised sociocultural constructs informed by the user's narratives, clinical competencies, contextual and environmental considerations.

Expert advice

An expert team consisting of a medical librarian from the Yong Loo Lin School of Medicine (YLLSoM) at the National University of Singapore (NUS), and local educational experts and clinicians at the National Cancer Centre Singapore (NCCS), the Palliative Care Unit, and the Institute of Population Health at the University of Liverpool, YLLSoM and Duke-NUS Medical School, ensured that the SEBA methodology was employed in a consistent manner within accepted practices.

Reflexivity

Personal reflexivity

Recognising that the research team are informed by their interests, narratives, clinical and research insights and contextual considerations, membership to the research and expert teams were made up of experienced physician-tutors, psychologists, methodologists, and educational scholars. Six members of these teams hold masters degrees in medical education, two hold masters degrees in ethics, two hold masters degrees in clinical research and two hold masters degrees in public health. Most of the expert and research team were experienced in quantitative research however five members of the team are experienced in qualitative methods. Six of the research and expert teams have published articles in peer-reviewed journals using the SEBA methodology. One member of the expert team is an experienced researcher at the Palliative Care Institute Liverpool and another is a member of the Health Data Science Department at the University of Liverpool. Both researchers from the University of Liverpool have

For peer review only

been collaborating with the research team on a number of studies pertaining to portfolio use, death and dying, moral distress, PIF and mentoring and are part of the team expanding the use of the SEBA methodology in medical education and palliative care.

Three senior members of the team are working on various projects on medical education and three others have already published articles in peer-reviewed journals in medical education. Six members of the research team were members of the PMI and have published SEBA guided reviews on mentoring, dignity and medical education. To ensure input from all members of the team, synchronous and asynchronous in-person and online meetings and Sandelowski and Barroso (18)'s approach to 'negotiated consensual validation' was used to reach consensus on the issues discussed.

Methodological reflexivity

Adopting a structured constructivist approach, we sought to build a holistic concept of current models of portfolios within medical education acknowledging however that we were limited by manpower and time constraints. We included articles featured in grey and bibliographic databases within the dates set in our selection criteria. Much of these theories required deeper consideration and discussions and we documented our discussions and decisions.

Iterative process

To ensure transparency and accountability, the expert team was involved in all stages of SEBA as part of the iterative process.

For peer review only

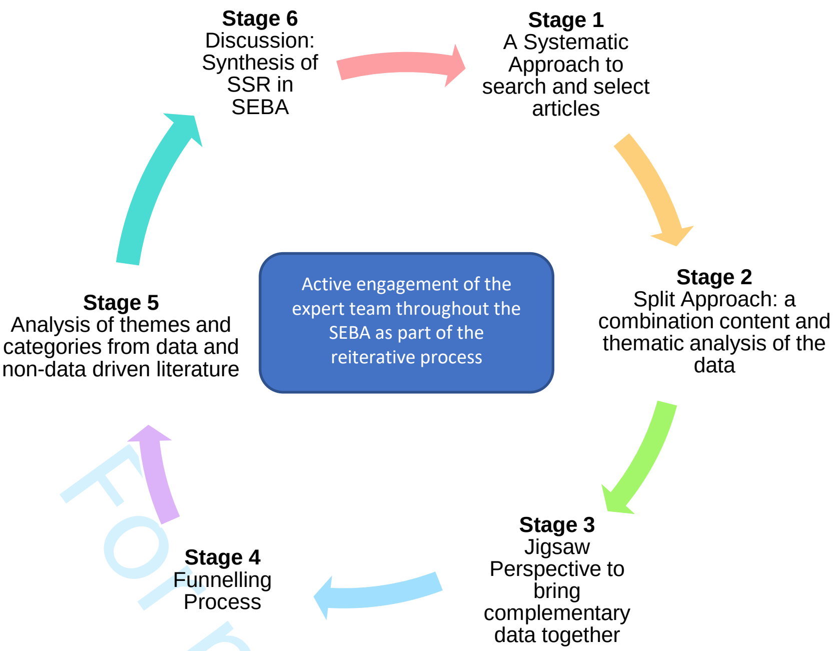

Figure 1. The SEBA Process

For peer review only

Stage 1 of SEBA: Systematic Approach

The SEBA methodology begins with the research and expert teams agreeing upon the research questions, the search terms and the databases to be scrutinised. In this case the research and expert team determined the primary research question: "*What is known about CEP portfolios?*". The secondary research questions were "*what role do CEP portfolios have in teaching and assessing CEP development?*".

Inclusion criteria

A PICOs (Population, Intervention, Comparison, Outcome, Study design) format was adopted to guide the research process (Table 1). There was no comparison group.

Table 1. PICOS, inclusion and exclusion criteria applied to the database search

<u>Table 1. *PICOS Inclusion and Exclusion Criteria*</u>

PICOS	Inclusion criteria	Exclusion criteria
**Population**	• Undergraduate and postgraduate medical students<br>• Qualified medical doctors, physician or resident; medical officer, registrar, house officer, attending, consultant	• Allied health specialties such as Pharmacy, Dietetics, Chiropractic, Midwifery, Podiatry, Speech Therapy, Occupational and Physiotherapy<br>• Non-medical specialties such as Clinical and Translational Science, Alternative and Traditional Medicine, Veterinary, Dentistry
**Intervention**	• portfolios in undergraduate and postgraduate medical education for teaching and assessment of Communication, Ethics and Professionalism	Other documentation methods or learning tools that are:<br>• Not longitudinal or single timepoint<br>• Does not include personal intellectual engagement with the

PICOS	Inclusion criteria	Exclusion criteria
	Criteria of a portfolio:<br>• longitudinal (more than a single timepoint) assessment data<br>• candidate's personal engagement with portfolio content and associated learning<br>• Interventions meeting the above criteria were included regardless of whether they were referred to as portfolios<br><br>All types of portfolios were included in the study:<br>• For instance: electronic & non-electronic; formative & summative or combined; clinical & non-clinical<br>• Portfolios with input from students and/or residents and/or doctors, and/or input from faculty members and other individuals<br>• Portfolios with different structures: extent by which the structure has been prescribed and/or left to individual discretion	content and associated learning (for instance, curriculum vitae, logbooks and the use of personal digital assistants)
**Comparison/ Context**	NA	NA
**Outcome**	Papers that measured the following outcomes were included:<br>• Effectiveness of the use of portfolios to assess and teach Communication, Ethics and Professionalism<br>• Impact of the use of portfolios on medical students (both undergraduate and postgraduate)<br>• Impact of the use of portfolios on the faculty	NA
**Study design**	• Articles in English or translated to English<br>• Articles published from 1st January 2000 to 31st December 2020<br>• Databases: PsycINFO, EMBASE, PubMed, ERIC, Scopus, Google Scholar<br>• All study designs including:<br>• Mixed methods research, meta-analyses, systematic reviews, randomised controlled trials, cohort studies, case-control studies, cross-sectional studies, descriptive papers, grey literature, opinions, letters, commentaries and editorials	NA

Three members of the research team carried out independent ancestry searches of seven leading journals in medical education (Academic Medicine, Medical Education, Medical Teacher, Advances Health Sciences Education, BMC Medical Education, Teaching and Learning in Medicine and Perspectives on Medical Education) accessed through the NUS library portal. In keeping with Pham, Rajic (19)'s recommendations, the searches were restricted to articles published between 1st January 2000 and 31st December 2020 to accommodate to existing

For peer review only

manpower and time constraints. Quantitative, mixed and qualitative research methodologies meeting the inclusion criteria were included.

Members of the research team carried out independent searches of databases. To facilitate this approach, the search process saw experienced senior researchers well-versed in carrying out systematic reviews and systematic scoping reviews each meet with a team of two to three medical students to guide them database searches. This approach was to enhance training of new researchers and to ensure that at least two teams were independently reviewing each database. Each team met regularly and discussed their findings. After a search of the first 100 articles in a particular database, the medical students and the senior researcher compared their findings at an online meeting.

Subsequently, the teams met at specific time points, often after reviewing a predetermined number of included articles to discuss their concerns, exchange opinions and advance their understanding of the research process and the area of study. Sandelowski and Barroso (18)'s 'negotiated consensual validation' was used to achieve consensus on the final list of titles to be reviewed. The teams repeated this process, independently studying all the full text articles on the final list of titles, creating their own lists of articles to be included and discussing their findings at weekly online research meetings over the first 4 months of the project. Consensus on the final list of articles to be analysed was achieved following at least three online meetings when discrepancies were reviewed and discussed by each member of the research team.

As this was a training process for many of the participants who were medical students participating in the Palliative Medicine Initiative, senior mentors and peer-mentors were frequently involved in guiding and discussing individual findings over and above the online meetings especially at the start of each stage of the research process. Interrater reliability was not evaluated.

Stage 2 of SEBA: Split Approach

Krishna's 'Split Approach' (18-23) was employed to enhance the reliability of the data analyses. This saw three groups of researchers independently analysing the included articles.

The first team summarised and tabulated the included full-text articles in keeping with recommendations drawn from Wong, Greenhalgh (24)'s RAMESES publication standards: meta-narrative reviews and Popay, Roberts (25)'s "Guidance on the conduct of narrative synthesis in systematic reviews". The tabulated summaries served to ensure that key aspects of included articles were not lost.

Concurrently, the second team analysed the included articles using Braun and Clarke (26)'s approach to thematic analysis. In Phase 1, the research team carried out independent reviews, 'actively' reading the included articles to find meaning and patterns in the data. In Phase 2, 'codes' were constructed from the 'surface' meaning and collated into a code book to code and analyse the rest of the articles using an iterative step-by-step process. As new codes emerged, these were associated with previous codes and concepts. In Phase 3, the categories were organised into themes that best depict the data. An inductive approach allowed themes to be "defined from the raw data without any predetermined classification" (27). In Phase 4, the themes were refined to best represent the

For peer review only

whole data set and discussed. In Phase 5, the research team discussed the results of their independent analysis online and at reviewer meetings. ‘Negotiated consensual validation’ was used to determine a final list of themes approach and ensure the final themes.

A third team of researchers employed Hsieh and Shannon (28)’s approach to directed content analysis to analyse the included articles. Analysis using the directed content analysis approach involved “identifying and operationalizing a priori coding categories”. The categories employed in the content analysis for undergraduate communications were Rider et al. (29) ’s *“A model for communication skills assessment across the undergraduate curriculum”,* Goldie (30)’s *“Review of ethics curricula in undergraduate medical education”*, Duffy et al. (31)’s *“Assessing Competence in Communication and Interpersonal Skills: The Kalamazoo II Report”* and Hong et al. (32)’s *“Postgraduate Ethics Training Programs: A Systematic Scoping Review”*. Tay et al. (33)’s *“Assessing Professionalism in Medicine - A Scoping Review of Assessment Tools from 1990 to 2018”* was employed for codes for professionalism and David (34)’s article *“AMEE Medical Education Guide No. 24: Portfolios as a method of student assessment”* was then used to contextualise their use in portfolios. Any data not captured by these codes were assigned a new code.

By using directed content analysis, this “Split Approach” sought to address shortcomings int thematic analysis. This was done by ironing out disparities in data and increases the validity of the identified themes (35). It also limits inherent biases and condenses the interpretations of terminology used by each team’s members. Consistency with existing literature is further offered by directed content analysis, by using existing data to identify codes and categories. The codes were used systematically and objectively, strengthening the validity and reliability of a positivist approach (35, 36). The transparency of this approach is enforced by clearly defined categories, together with references throughout the analytical process.

Stage 3 of SEBA: Jigsaw Perspective

The themes and categories identified in the Split Approach were viewed as pieces of a jigsaw puzzle where overlapping/complementary pieces were combined to create a bigger piece of the puzzle referred to as themes/categories. This process was guided by Phases 4 to 6 of France, Uny (37)’s adaptation of Noblit, Hare (38)’s seven phases of meta-ethnography. As per Phase 4, the themes and the categories identified in the Split Approach are grouped together according to their focus. These groupings of categories and themes were then contextualised through the review of the articles from which they were drawn from. Reciprocal translation was used to determine if the themes and categories could be used interchangeably. This allowed the themes and categories to be combined to form themes/categories.

France, Uny’s adaptation:
Phase 1: Getting started, deciding on the focus of the synthesis.
Phase 2: Deciding what is relevant to the initial interest.
Phase 3: Reading the Studies.
Phase 4: Determining how the studies are related.
Phase 5: Translating the studies into one another.
Phase 6: Synthesising the translations.

For peer review only

Phase 7: Expressing the synthesis.

Codes derived from thematic analysis (TA): indications, characteristics and strengths and limitations

Codes derived from directed content analysis (DCA): indication for portfolio, portfolio content, portfolio design and strengths and limitations

Overlaps between indications and strengths and limitations allowed the combination of these categories and themes creating two themes/categories called indications and strengths and limitations. Careful review of the categories- portfolio content and portfolio design were subsets of the theme characteristics. For a more accurate presentation, the overlaps were conflated and presented together with the TA codes indicated under the DCA code. (Refer to Domain 4 table)

**Themes Identified**	**Categories Identified**	**Domains Created**
Indications	indications	Indications
characteristics	content	Characteristics
	Portfolio design	
Strengths and limitations	Strengths and limitations	Dignity-conserving Measures

For peer review only

Stage 4 of SEBA: Funnelling Process

The Funnelling Process employs Phases 3 to 5 of France, Uny's adaptation to juxtapose the themes/categories with key messages identified in the tabulated summaries to create domains. These domains form the basis for 'the line of argument' in Stage 6 of SEBA.

For peer review only

For peer review only

For peer review only

Domain 2. Relationship between Dignity, WTHD and Assisted Dying

Subdomains	Codes
**Increasing WTHD**	▪ Loss of dignity (39-81)<br>▪ Fear of loss of dignity (42, 44, 52, 59, 82, 83)<br>▪ Loss of autonomy (39, 40, 42, 45, 47, 50-53, 55, 59, 62, 65, 69, 70, 73, 74, 77, 79, 81, 82, 84-88)<br>▪ Fear of loss of autonomy (42, 54, 70, 89)
**Decreasing WTHD/protective factors**	▪ Addressing of dignity related issues improves health-related quality of life (46)<br>▪ Improving dignity reduces desire for euthanasia (50)
**Effect of assisted dying on dignity**	▪ Reclamation/preservation of dignity (45, 49, 58, 90-95)<br>▪ Lack of assisted dying interferes with dignity (50, 76, 96)<br>▪ Reclamation/preservation of autonomy (43, 45, 49, 51, 52, 67, 73, 74, 77, 85, 88, 92, 94, 97-100)<br>▪ Lack of assisted dying interferes with autonomy (49, 96)<br>▪ Violation of autonomy (100)

For peer review only

Domain 3. Stakeholder Perspectives on Dignity

**Subdomains**	**Codes**	
**Patient Perspectives**	**Positive Aspects**<br>▪ Respect for humanity (67)<br>▪ Right to die (99)<br>▪ Unaffected by fear of death (48)<br>▪ Good death (67, 73, 99, 101-103)<br>- Maintaining control of their death (67, 73, 99, 101-103)<br>- Ending suffering (67, 73)<br>▪ Perceived futility (73, 74)<br>▪ General support (73, 98)<br>▪ Wanting the option for themselves (98)	**Negative Aspects**<br>▪ Religious concerns (67, 73, 102)<br>▪ Ethical concerns (62, 67, 73)<br>- Inability to ascertain patient consent (62, 67)<br>- Equating to murder/suicide (67)<br>▪ Change in ideologies (50)<br>▪ Deterioration of patient-doctor relationship (58, 62, 74, 101)<br>- Loss of faith in palliative/hospice care (62, 101)<br>- Feeling of abandonment when WTHD is turned down (74)<br>- Poor communications (58)<br>▪ Against legislation (73)
**Healthcare Provider Perspectives**	**Positive Aspects**<br>▪ Good death for patients (42, 47, 49, 50, 54, 56, 57, 61, 69, 70, 82, 87, 101, 103)<br>- Fulfilling patients' desire for control (47, 50, 56, 61, 82, 87, 101, 104)<br>- Ending suffering (42, 54, 57, 61, 69, 70, 103)<br>- Not requiring a long life (49)<br>▪ Feeling helpless (68)<br>▪ Non-abandonment of patients (90)<br>▪ Reducing patients' family burden (90)<br>▪ Avoiding unlawful hastening death (50)<br>▪ General support (40, 47, 62, 68-70, 75, 82, 90, 101, 103-105)<br>▪ Disregarding the need for alternatives (i.e. palliative care) (50)	**Negative Aspects**<br>▪ Religious concerns (106)<br>▪ Disrespecting patients' autonomy by not entertaining their wishes (104, 107)<br>▪ Emotional/moral distress (49, 50, 69, 91, 101, 108-113)<br>- Guilt (91, 112)<br>- Opposing medical principles (49, 69, 101, 108, 110, 111)<br>- Grief, avoidance after carrying out WTHD (113)<br>- Turning doctors into killers (111)<br>▪ No right to die (90)<br>▪ Unable to ascertain if patient's disease is incurable (109)<br>▪ Change in perceptions after disease (82)<br>▪ WTHD should not involve family members (113)<br>▪ "Slippery slope" (110)<br>▪ Societal pressure (55, 74)<br>▪ Disapproval of WTHD (62, 69, 75, 82, 101, 107, 113-115)<br>- Disagreement with the law (115)<br>- Reluctance to carry out WTHD (75, 101)<br>▪ Proposing alternatives (57, 62, 63, 90, 105, 111)<br>- Improving quality of life of dying patients (62, 111)<br>- Better palliative care (63, 90, 105)<br>- Healing (57)<br>▪ Death as an unknown (57)

For peer review only

		▪ Disapproval of 'tired of living' as a reason for WTHD (57)
**Lawmaker Perspectives**	▪ Belief in a right to die (76)<br>▪ Avoiding unlawful hastening death (96)<br>▪ Government's duty to consider patients' WTHD (74)<br>▪ Approval of WTHD (76, 100)	▪ "Slippery slope" (98, 114)<br>- Risk of abuse (114)<br>▪ Instigating social pressure (on the elderly) (74)<br>▪ Disapproval of WTHD (74, 83, 106, 114)

For peer review only

Domain 4. Dignity Conserving Measures

Subdomains	Codes
**Advantages to Dignity Conserving Measures**	▪ Palliative services<br>- Palliative care (40, 44, 47, 49, 50, 62, 63, 66, 73, 82, 90, 91, 103, 111, 116-118)<br>- Palliative sedation (47, 49, 91, 119)<br>- Palliative starvation (85, 109)<br>▪ Pain and symptom management<br>- Control of physical pain (58, 66, 75, 104, 107, 108, 119)<br>- Control of psychological symptoms (75, 102, 120)<br>▪ Hospice care (46, 47, 75, 100, 106, 107, 117)<br>▪ Holistic approach (43, 57, 105, 111, 112, 117, 121, 122)<br>▪ Ease of access to information (47, 57, 71, 87, 92, 100, 112, 117)<br>▪ Distributive justice (55)<br>▪ Social support<br>- Religious (75)<br>- Familial (45)<br>- Healthcare providers (47, 57, 58, 71, 87, 112)
**Limitations to Dignity Conserving Measures**	▪ Cannot completely address:<br>- Existential suffering (54)<br>- Pain (50)<br>- Complete loss of mobility (103)<br>- Extreme shortness of breath (103)<br>- Fear of sudden and rapid bleeding (103)<br>- Ongoing stress of losing autonomy and dignity (103)<br>▪ Side effects of pain medication (58)

For peer review only

Stage 5 of SEBA: Analysis of data and non-data driven literature

A novel aspect of the SEBA methodology has been its inclusion of position, perspective, conference, reflective and opinion papers, editorials, commentaries, letters, posters, oral presentations, forum discussions, interviews, blogs, governmental reports, policy statements and surveys from PubMed, Embase, PsycINFO, Cochrane Database of Systematic Reviews, CINAHL, Scopus databases and the Journal of Pain and Symptom Management, BMC Palliative Medicine, Death Studies, and Palliative Medicine. With many current survey and assessment tools not able to capture the intricate connections and personalised nature of wider concepts of dignity, assisted dying, WTHD, personhood and identity, these resources are a rich source of information. These resources also capture wider patient, HCP and lawmaker perspectives and offer information on ethical, existential, and societal considerations often excluded by traditional systematic reviews as evidenced by recent reviews into how physicians and patients deal with death and dying and moral distress (123-130)

The use of non-data driven articles such as position, perspective, conference, reflective and opinion papers, editorials, commentaries, letters, posters, oral presentations, forum discussions, interviews, blogs, governmental reports, policy statements and surveys is consistent with a constructivist approach that includes all forms of data. In addition, use of non-data driven articles is a valuable source of information for subject matter such as concepts of dignity which are notoriously difficult to study and open to sociocultural considerations.

However, with some of these sources neither peer-reviewed nor necessarily evidence-based, they were differentiated from primary research, compiled and thematically analysed. The themes identified from primary data sources and non-evidence based and/or tertiary data sources were then compared to enhance further the accountability and reproducibility.

With the themes from the two groups found to be similar, the expert and research teams were satisfied that their inclusion did not bias the overall data.

Stage 6 of SEBA: Synthesis of the Discussion

The Best Evidence Medical Education (BEME) Collaboration Guide (131) and the Structured approach to the Reporting In healthcare education of Evidence Synthesis (STORIES) (132) were used to guide the discussion.

For peer review only

References

1. Kow CS, Teo YH, Teo YN, Chua KZY, Quah ELY, Kamal NHBA, et al. A systematic scoping review of ethical issues in mentoring in medical schools. BMC Medical Education. 2020;20(1):1-10.
2. Ngiam LXL, Ong YT, Ng JX, Kuek JTY, Chia JL, Chan NPX, et al. Impact of Caring for Terminally Ill Children on Physicians: A Systematic Scoping Review. Am J Hosp Palliat Care. 2020:1049909120950301.
3. Krishna LKR, Tan LHE, Ong YT, Tay KT, Hee JM, Chiam M, et al. Enhancing Mentoring in Palliative Care: An Evidence Based Mentoring Framework. Journal of Medical Education and Curricular Development. 2020;7:2382120520957649.
4. Chia EWY, Huang H, Goh S, Peries MT, Lee CCY, Tan LHE, et al. A Systematic Scoping Review of Teaching and Evaluating Communications in The Intensive Care Unit. The Asia-Pacific Scholar. In Press.
5. Kuek JTY, Ngiam LXL, Kamal NHA, Chia JL, Chan NPX, Abdurrahman ABHM, et al. The impact of caring for dying patients in intensive care units on a physician's personhood: a systematic scoping review. 2020;15(1):1-16.
6. Hong DZ, Lim AJS, Tan R, Ong YT, Pisupati A, Chong EJX, et al. A Systematic Scoping Review on Portfolios of Medical Educators. Journal of Medical Education and Curricular Development. 2021;8:23821205211000356.
7. Goh S, Wong RSM, Quah ELY, Chua KZY, Lim WQ, Ng ADR, et al. Mentoring in palliative medicine in the time of covid-19: a systematic scoping review. BMC medical education. 2022;22(1):1-15.
8. Bok C, Ng CH, Koh JWH, Ong ZH, Ghazali HZB, Tan LHE, et al. Interprofessional communication (IPC) for medical students: a scoping review. BMC Med Educ. 2020;20(1):372.
9. Ng YX, Koh ZYK, Yap HW, Tay KT, Tan XH, Ong YT, et al. Assessing mentoring: A scoping review of mentoring assessment tools in internal medicine between 1990 and 2019. PloS one. 2020;15(5):e0232511.
10. Bousquet J, Schunemann HJ, Samolinski B, Demoly P, Baena-Cagnani CE, Bachert C, et al. Allergic Rhinitis and its Impact on Asthma (ARIA): achievements in 10 years and future needs. J Allergy Clin Immunol. 2012;130(5):1049-62.
11. Nur Haidah Ahmad Kamal LHET, Ruth Si Man Wong, Ryan Rui Song Ong, Ryan, Ern Wei Seow EKYL, Zheng Hui Mah, Min Chiam, Annelissa Mien Chew Chin, Jamie Xuelian Zhou, Gillian Li Gek Phua, Eng Koon Ong, Jin Wei Kwekc, Kiley Wei-Jen Loh and, Krishna LKR. Enhancing education in Palliative Medicine: the role of Systematic Scoping Reviews. Palliative Medicine & Care: Open Access. 2020;7(1):1-11.
12. Ryan Rui Song Ong REWS, Ruth Si Man Wong. A Systematic Scoping Review of Narrative Reviews in Palliative Medicine Education. Palliative Medicine & Care: Open Access. 2020;7(1):1-22.
13. Zheng Hui Mah RSMW, Ryan Ern Wei Seow Eleanor Kei Ying Loh, Nur Haidah, Ahmad Kamal RRSO, Lorraine Hui En Tan, Min Chiam, Annelissa Mien Chew Chin,, Jamie Xuelian Zhou GLGP, Yoke-Lim Soong, Jin Wei Kwek, and Lalit Kumar Radha Krishna. A Systematic Scoping Review of Systematic Reviews in Palliative Medicine Education. Palliative Medicine & Care: Open Access. 2020;7(1):1-12.
14. Pring R. The 'False Dualism' of Educational Research. Journal of Philosophy of Education. 2000;34(2):247-60.
15. Crotty M. The foundations of social research: Meaning and perspective in the research process: Sage; 1998 Oct 15.

For peer review only

16. Ford DW, Downey L, Engelberg R, Back AL, Curtis JR. Discussing religion and spirituality is an advanced communication skill: an exploratory structural equation model of physician trainee self-ratings. J Palliat Med. 2012;15(1):63-70.
17. Schick-Makaroff K, MacDonald M, Plummer M, Burgess J, Neander W. What synthesis methodology should I use? A review and analysis of approaches to research synthesis. AIMS public health. 2016;3(1):172.
18. Sandelowski M BJ. Handbook for synthesizing qualitative research. New York: Springer; 2007.
19. Pham MT, Rajic A, Greig JD, Sargeant JM, Papadopoulos A, McEwen SA. A scoping review of scoping reviews: advancing the approach and enhancing the consistency. Research synthesis methods. 2014;5(4):371-85.
20. Wen Jie Chua CWSC, Fion Qian Hui Lee, Eugene Yong Hian Koh, Ying Pin Toh, Stephen Mason, Lalit Kumar Radha Krishna. Structuring Mentoring in Medicine and Surgery. A Systematic Scoping Review of Mentoring Programs Between 2000 and 2019. Journal of Continuing Education in the Health Professions. 2020;40(3):158-68.
21. Yong Xiang Ng ZYKK, Hong Wei Yap, Kuang Teck Tay, Xiu Hui Tan, Yun Ting Ong, Lorraine Hui En Tan, Annelissa Mien Chew Chin, Ying Pin Toh, Sushma Shivananda, Scott Compton, Stephen Mason, Ravindran Kanesvaran, Lalit Krishna. Assessing mentoring: A scoping review of mentoring assessment tools in internal medicine between 1990 and 2019. . PLOS ONE. 2020;15(5):e0232511.
22. Peters MD, Godfrey CM, Khalil H, McInerney P, Parker D, Soares CB. Guidance for conducting systematic scoping reviews. Int J Evid Based Healthc. 2015;13(3):141-6.
23. Sambunjak D, Straus SE, Marusic A. A systematic review of qualitative research on the meaning and characteristics of mentoring in academic medicine. J Gen Intern Med. 2010;25(1):72-8.
24. Wong G, Greenhalgh T, Westhorp G, Buckingham J, Pawson R. RAMESES publication standards: meta-narrative reviews. BMC medicine. 2013;11(1):20.
25. Popay J, Roberts H, Sowden A, Petticrew M, Arai L, Rodgers M, et al. Guidance on the conduct of narrative synthesis in systematic reviews. A product from the ESRC methods programme Version. 2006;1:b92.
26. Braun V, Clarke V. Using thematic analysis in psychology. Qualitative research in psychology. 2006;3(2):77-101.
27. Cassol H, Pétré B, Degrange S, Martial C, Charland-Verville V, Lallier F, et al. Qualitative thematic analysis of the phenomenology of near-death experiences. PloS one. 2018;13(2):e0193001.
28. Hsieh H-F, Shannon SE. Three Approaches to Qualitative Content Analysis. Qualitative Health Research. 2005;15(9):1277-88.
29. Rider EA, Hinrichs MM, Lown BA. A model for communication skills assessment across the undergraduate curriculum. Med Teach. 2006;28(5):e127-34.
30. Goldie J. Review of ethics curricula in undergraduate medical education. Med Educ. 2000;34(2):108-19.
31. Duffy FD, Gordon GH, Whelan G, Cole-Kelly K, Frankel R, Buffone N, et al. Assessing competence in communication and interpersonal skills: the Kalamazoo II report. Acad Med. 2004;79(6):495-507.
32. Hong DZ, Goh JL, Ong ZY, Ting JJQ, Wong MK, Wu J, et al. Postgraduate ethics training programs: a systematic scoping review. BMC Medical Education. 2021;21(1):338.
33. Tay KT, Ng S, Hee JM, Chia EWY, Vythilingam D, Ong YT, et al. Assessing Professionalism in Medicine - A Scoping Review of Assessment Tools from 1990 to 2018. J Med Educ Curric Dev. 2020;7:2382120520955159.

For peer review only

34. David M, Davis M, Harden R, Howie P, Ker J, Pippard M. AMEE Medical Education Guide No. 24: Portfolios as a method of student assessment. Medical teacher. 2001;23:535-51.
35. Dixon-Woods M, Agarwal S, Young B, Jones D, Sutton A. Integrative Approaches to Qualitative and Quantitative Evidence. 2004.
36. Bryman A. Social Research Methods: Oxford University Press; 2016.
37. France EF, Uny I, Ring N, Turley RL, Maxwell M, Duncan EAS, et al. A methodological systematic review of meta-ethnography conduct to articulate the complex analytical phases. BMC Medical Research Methodology. 2019;19(1):35.
38. Noblit GW, Hare RD, Hare RD. Meta-ethnography: Synthesizing qualitative studies: sage; 1988.
39. Al Rabadi L, LeBlanc M, Bucy T, Ellis LM, Hershman DL, Meyskens FL, et al. Trends in Medical Aid in Dying in Oregon and Washington. JAMA Network Open. 2019;2(8):e198648-e.
40. Emanuel EJ, Onwuteaka-Philipsen BD, Urwin JW, Cohen J. Attitudes and practices of euthanasia and physician-assisted suicide in the United States, Canada, and Europe. JAMA: Journal of the American Medical Association. 2016;316(1):79-90.
41. Brinkman-Stoppelenburg A, Vergouwe Y, van der Heide A, Onwuteaka-Philipsen BD. Obligatory consultation of an independent physician on euthanasia requests in the Netherlands: What influences the SCEN physicians judgment of the legal requirements of due care? Health Policy. 2014;115(1):75-81.
42. Buiting H, van Delden J, Onwuteaka-Philpsen B, Rietjens J, Rurup M, van Tol D, et al. Reporting of euthanasia and physician-assisted suicide in the Netherlands: Descriptive study. BMC Medical Ethics Vol 10 2009, ArtID 18. 2009;10.
43. Konishi E, Davis A. The right-to-die and the duty-to-die: Perceptions of nurses in the West and in Japan. International Nursing Review. 2001;48(1):17-28.
44. Chapple A, Ziebland S, McPherson A, Herxheimer A. What People Close to Death Say About Euthanasia and Assisted Suicide: A Qualitative Study. Journal of Medical Ethics: Journal of the Institute of Medical Ethics. 2006;32(12):706-10.
45. Coyle N, Sculco L. Expressed desire for hastened death in seven patients living with advanced cancer: a phenomenologic inquiry. Oncol Nurs Forum. 2004;31(4):699-709.
46. Crespo I, Rodríguez-Prat A, Monforte-Royo C, Wilson KG, Porta-Sales J, Balaguer A. Health-related quality of life in patients with advanced cancer who express a wish to hasten death: A comparative study. Palliat Med. 2020;34(5):630-8.
47. Drum CE, White G, Taitano G, Horner-Johnson W. The Oregon Death with Dignity Act: Results of a literature review and naturalistic inquiry. Disability and Health Journal. 2010;3(1):3-15.
48. Ferrand E, Dreyfus JF, Chastrusse M, Ellien F, Lemaire F, Fischler M. Evolution of requests to hasten death among patients managed by palliative care teams in France: a multicentre cross-sectional survey (DemandE). Eur J Cancer. 2012;48(3):368-76.
49. Ramsey C. The right to die: beyond academia. Monash Bioeth Rev. 2016;34(1):70-87.
50. Reagan P, Hurst R, Cook L, Zylicz Z, Otlowski M, Veldink JH, et al. Physician-assisted death: Dying with dignity? Lancet Neurology. 2003;2(10):637-43.
51. Rodríguez-Prat A, Balaguer A, Booth A, Monforte-Royo C. Understanding patients' experiences of the wish to hasten death: an updated and expanded systematic review and meta-ethnography. BMJ Open. 2017;7(9):e016659.
52. Rodríguez-Prat A, van Leeuwen E. Assumptions and moral understanding of the wish to hasten death: a philosophical review of qualitative studies. Medicine, Health Care & Philosophy. 2018;21(1):63-75.

For peer review only

53. Wang L, Elliott M, Henson LJ, Gerena-Maldonado E, Strom S, Downing S, et al. Death with dignity in Washington and Oregon patients with amyotrophic lateral sclerosis. Neurology. 2016;86(16).
54. Fischer S, Huber CA, Furter M, Imhof L, Mahrer Imhof R, Schwarzenegger C, et al. Reasons why people in Switzerland seek assisted suicide: the view of patients and physicians. Swiss Med Wkly. 2009;139(23-24):333-8.
55. Freeman LM, Rose SL, Youngner SJ. Poverty: Not a Justification for Banning Physician-Assisted Death. Hastings Cent Rep. 2018;48(6):38-46.
56. Ganzini L, Goy ER, Dobscha SK. Why Oregon patients request assisted death: Family members' views. Journal of General Internal Medicine. 2008;23(2):154-7.
57. Goligher EC, Wesley Ely E, Sulmasy DP, Bakker J, Raphael J, Volandes AE, et al. Physician-Assisted Suicide and Euthanasia in the ICU: A Dialogue on Core Ethical Issues. Critical Care Medicine. 2017;45(2):149-55.
58. Hendry M, Pasterfield D, Lewis R, Carter B, Hodgson D, Wilkinson C. Why do we want the right to die? A systematic review of the international literature on the views of patients, carers and the public on assisted dying. Palliative Medicine. 2013;27(1):13-26.
59. Hiscox WE. Physician-assisted suicide in Oregon: The 'death with dignity' data. Medical Law International. 2007;8(3):197-220.
60. Kade WJ, Kade WJ. Death with dignity: a case study. Annals of Internal Medicine. 2000;132(6):504-6.
61. Kouwenhoven PS, van Thiel GJ, Raijmakers NJ, Rietjens JA, van der Heide A, van Delden JJ. Euthanasia or physician-assisted suicide? A survey from the Netherlands. Eur J Gen Pract. 2014;20(1):25-31.
62. Bailey F. "I am not afraid of dying. I just don't want to be there when it happens.". Medical Care. 2008;46(12):1195-7.
63. Barutta J, Vollmann J. Physician-assisted death with limited access to palliative care. Journal of Medical Ethics: Journal of the Institute of Medical Ethics. 2015;41(8):652-4.
64. Bolt EE, Pasman H, Deeg DJ, Onwuteaka-Philipsen BD. From advance euthanasia directive to euthanasia: Stable preference in older people? Journal of the American Geriatrics Society. 2016;64(8):1628-33.
65. Dees M, Vernooij-Dassen M, Dekkers W, van Weel C. Review unbearable suffering of patients with a request for euthanasia or physician-assisted suicide: An integrative review. Psycho-Oncology. 2010;19(4):339-52.
66. Duckett S. Pathos, death talk and palliative care in the assisted dying debate in Victoria, Australia. Mortality. 2020;25(2):151-66.
67. Eliott JA, Olver IN. Dying cancer patients talk about euthanasia. Social Science & Medicine. 2008;67(4):647-56.
68. McPherson T. My mum wanted assisted dying but we watched her die slowly and in pain. BMJ: British Medical Journal (Clinical Research Edition). 2012;344:e4007-e.
69. Mukhopadhyay S, Banerjee D. Physician assisted suicide in dementia: A critical review of global evidence and considerations from India. Asian J Psychiatr. 2021;64:102802.
70. Rowe Iii MJ. Beliefs. Chicago, Illinois: American Medical Association; 2015. p. 877-8.
71. Roy DJ. Palliative care and euthanasia: a continuing need to think again. J Palliat Care. 2002;18(1):3-5.
72. van Tol D, Rietjens J, van der Heide A. Judgment of unbearable suffering and willingness to grant a euthanasia request by Dutch general practitioners. Health Policy. 2010;97(2-3):166-72.

For peer review only

73. Wilson KG, Chochinov HM, McPherson CJ, Skirko MG, Allard P, Chary S, et al. Desire for euthanasia or physician-assisted suicide in palliative cancer care. Health Psychology. 2007;26(3):314-23.
74. Florijn BW. From Reciprocity to Autonomy in Physician-Assisted Death: An Ethical Analysis of the Dutch Supreme Court Ruling in the Albert Heringa Case. Am J Bioeth. 2022;22(2):51-8.
75. Ganzini L, Nelson HD, Schmidt TA, Kraemer DF, Delorit MA, Lee MA. Physicians' experiences with the Oregon Death with Dignity Act. N Engl J Med. 2000;342(8):557-63.
76. Kishore RR. Aruna Shanbaug and the right to die with dignity: the battle continues. Indian J Med Ethics. 2016;1(1):38-46.
77. Royo C, Villavicencio-Chávez C, Tomás-Sábado J, Mahtani V, Balaguer A. What Lies behind the Wish to Hasten Death? A Systematic Review and Meta-Ethnography from the Perspective of Patients. PloS one. 2012;7:e37117.
78. Bahník Š, Vranka MA, Trefná K. What makes euthanasia justifiable? The role of symptoms' characteristics and interindividual differences. Death Stud. 2021;45(3):226-37.
79. Hizo-Abes P, Siegel L, Schreier G. Exploring attitudes toward physician-assisted death in patients with life-limiting illnesses with varying experiences of palliative care: a pilot study. BMC Palliative Care. 2018;17(1):56.
80. Ohnsorge K, Gudat H, Rehmann-Sutter C. What a wish to die can mean: reasons, meanings and functions of wishes to die, reported from 30 qualitative case studies of terminally ill cancer patients in palliative care. BMC Palliat Care. 2014;13:38.
81. Ruijs CDM, van der Wal G, Kerkhof AJFM, Onwuteaka-Philipsen BD. Unbearable suffering and requests for euthanasia prospectively studied in end-of-life cancer patients in primary care. BMC Palliative Care. 2014;13(1):62.
82. Gómez-Vírseda C, Gastmans C. Euthanasia in persons with advanced dementia: a dignity-enhancing care approach. J Med Ethics. 2021.
83. Hale DB. A pretty pass: when is there a right to die? Common Law World Rev. 2003;32(1):1-14.
84. Cheung G, Sundram F. Who are the elderly who want to end their lives? [References]. Rational suicide in the elderly: Clinical, ethical, and sociocultural aspects. Cham, Switzerland: Springer International Publishing/Springer Nature; Switzerland; 2017. p. 113-28.
85. Dees MK, Vernooij-Dassen MJ, Dekkers WJ, Vissers KC, van Weel C. 'Unbearable suffering': A qualitative study on the perspectives of patients who request assistance in dying. Journal of Medical Ethics: Journal of the Institute of Medical Ethics. 2011;37(12):727-34.
86. Fontalis A, Prousali E, Kulkarni K. Euthanasia and assisted dying: what is the current position and what are the key arguments informing the debate? Journal of the Royal Society of Medicine. 2018;111(11):407-13.
87. Ganzini L, Dobscha SK, Heintz RT, Press N. Oregon physicians' perceptions of patients who request assisted suicide and their families. Journal of Palliative Medicine. 2003;6(3):381-90.
88. Karlsson M, Milberg A, Strang P. Dying cancer patients' own opinions on euthanasia: An expression of autonomy? A qualitative study. Palliative Medicine. 2012;26(1):34-42.
89. Gamester N, Van den Eynden B. The relationship between palliative care and legalized euthanasia in Belgium. Journal of Palliative Medicine. 2009;12(7):589-91.
90. Rodriquez E. The arguments for euthanasia and physician-assisted suicide: ethical reflection. Linacre Q. 2001;68(3):251-61.
91. Abrahm JL. Patient and family requests for hastened death. Hematology Am Soc Hematol Educ Program. 2008:475-80.

For peer review only

92. Gandsman A. Paradox of choice and the illusion of autonomy: The construction of ethical subjects in right-to-die activism. Death Studies. 2018;42(5):329-35.
93. Granda-Cameron C, Houldin A. Concept Analysis of Good Death in Terminally Ill Patients. American Journal of Hospice & Palliative Medicine. 2012;29(8):632-9.
94. Heintz AP. Euthanasia--or, death on request. J Psychosom Obstet Gynaecol. 2002;23(2):73-5.
95. Jiraphan A, Pitanupong J. General population-based study on preferences towards end-of-life care in Southern Thailand: a cross-sectional survey. BMC Palliative Care. 2022;21(1):36.
96. Dyer C. Two men plead with judges to let doctors end their life legally. BMJ: British Medical Journal (Clinical Research Edition). 2012;344:e4270-e.
97. Castelli Dransart DA, Lapierre S, Erlangsen A, Canetto SS, Heisel M, Draper B, et al. A systematic review of older adults' request for or attitude toward euthanasia or assisted-suicide. Aging & Mental Health. 2021;25(3):420-30.
98. Achille MA, Ogloff JR. Attitudes toward and desire for assisted suicide among persons with amyotrophic lateral sclerosis. Omega: Journal of Death and Dying. 2003;48(1):1-21.
99. Right to die--right or wrong? Lancet Neurol. 2003;2(10):583.
100. Friend ML. Physician-Assisted Suicide: Death With Dignity? Journal of Nursing Law. 2011;14(3/4):110-6.
101. Drury B. The doctors of mercy. Men's Health. 2006;21(8):180-7.
102. Wilson KG, Scott JF, Graham ID, Kozak JF, Chater S, Viola RA, et al. Attitudes of terminally ill patients toward euthanasia and physician-assisted suicide. Archives of Internal Medicine. 2000;160(16):2454-60.
103. Reid T. Reflections from a provider of medical assistance in dying. Canadian Family Physician. 2018;64(9):639-40.
104. Asai A, Ohnishi M, Nagata SK, Tanida N, Yamazaki Y. Doctors' and nurses' attitudes towards and experiences of voluntary euthanasia: survey of members of the Japanese Association of Palliative Medicine. J Med Ethics. 2001;27(5):324-30.
105. Eastaugh A. Choosing to die. Br J Gen Pract. 2012;62(595):96-7.
106. Salladay SA. Christian ethics. Death with dignity? Journal of Christian Nursing. 2010;27(3):232-.
107. Broom A. On euthanasia, resistance, and redemption: The moralities and politics of a hospice. Qualitative Health Research. 2012;22(2):226-37.
108. Mentzelopoulos SD, Haywood K, Cariou A, Mantzanas M, Bossaert L. Evolution of medical ethics in resuscitation and end of life. Trends in Anaesthesia and Critical Care. 2016;10:7-14.
109. Raus K, Sterckx S. Euthanasia for mental suffering. New directions in the ethics of assisted suicide and euthanasia. Cham, Switzerland: Springer International Publishing/Springer Nature; Switzerland; 2015. p. 79-96.
110. Robinson V, Clarke J, George R. Euthanasia: A caricature of care, not a cure for suffering. British Journal of Neuroscience Nursing. 2007;3(10):457-60.
111. Stephenson J. Assisted dying: a palliative care physician's view. Clin Med (Lond). 2006;6(4):374-7.
112. Finlay IG. Quality of life to the end. Commun Med. 2005;2(1):91-5.
113. Feigin S, Owens RG, Goodyear-Smith F. Helping a loved one die: the act of assisted dying in New Zealand. Mortality. 2019;24(1):95-110.
114. Dyer C. Dying woman loses her battle for assisted suicide. Bmj. 2002;324(7345):1055.

For peer review only

115. Fournier RR. Responses to life after death with dignity: the Oregon experience. Soc Work. 2000;45(5):467-8.
116. Comby M, Filbet M. The demand for euthanasia in palliative care units: A prospective study in seven units of the 'Rhone-Alpes' region. Palliative Medicine. 2005;19(8):587-93.
117. Simpson E. Harms to dignity, bioethics, and the scope of biolaw. Journal of Palliative Care. 2004;20(3):185-92.
118. Ganzini L, Back A. From the USA: Understanding requests for physician-assisted death. Palliative Medicine. 2003;17(2):113-4.
119. Gentzler J. What is a death with dignity? J Med Philos. 2003;28(4):461-87.
120. Robinson S, Kissane DW, Brooker J, Hempton C, Burney S. The Relationship Between Poor Quality of Life and Desire to Hasten Death: A Multiple Mediation Model Examining the Contributions of Depression, Demoralization, Loss of Control, and Low Self-worth. J Pain Symptom Manage. 2017;53(2):243-9.
121. Clark D. Cultural considerations in planning palliative and end of life care. Palliat Med. 2012;26(3):195-6.
122. Oczkowski SJW, Crawshaw DE, Austin P, Versluis D, Kalles-Chan G, Kekewich M, et al. How can we improve the experiences of patients and families who request medical assistance in dying? A multi-centre qualitative study. BMC Palliative Care. 2021;20(1):185.
123. Ong RSR, Wong RSM, Chee RCH, Quek CWN, Burla N, Loh CYL, et al. A systematic scoping review moral distress amongst medical students. BMC Medical Education. 2022;22(1):466.
124. Chua KZY, Quah ELY, Lim YX, Goh CK, Lim J, Wan DWJ, et al. A systematic scoping review on patients’ perceptions of dignity. BMC Palliative Care. 2022;21(1):118.
125. Ho CY, Lim NA, Ong YT, Lee ASI, Chiam M, Gek GPL, et al. The impact of death and dying on the personhood of senior nurses at the National Cancer Centre Singapore (NCCS): a qualitative study. BMC Palliat Care. 2022;21(1):83.
126. Chiam M, Ho CY, Quah E, Chua KZY, Ng CWH, Lim EG, et al. Changing self-concept in the time of COVID-19: a close look at physician reflections on social media. Philos Ethics Humanit Med. 2022;17(1):1.
127. Chan NPX, Chia JL, Ho CY, Ngiam LXL, Kuek JTY, Ahmad Kamal NHB, et al. Extending the Ring Theory of Personhood to the Care of Dying Patients in Intensive Care Units. Asian Bioeth Rev. 2022;14(1):71-86.
128. Huang H, Toh RQE, Chiang CLL, Thenpandiyan AA, Vig PS, Lee RWL, et al. Impact of Dying Neonates on Doctors' and Nurses' Personhood: A Systematic Scoping Review. J Pain Symptom Manage. 2022;63(1):e59-e74.
129. Vig PS, Lim JY, Lee RWL, Huang H, Tan XH, Lim WQ, et al. Parental bereavement – impact of death of neonates and children under 12 years on personhood of parents: a systematic scoping review. BMC Palliative Care. 2021;20(1):136.
130. Ho CY, Kow CS, Chia CHJ, Low JY, Lai YHM, Lauw S-K, et al. The impact of death and dying on the personhood of medical students: a systematic scoping review. BMC Medical Education. 2020;20(1):516.
131. Haig A, Dozier M. BEME Guide no 3: systematic searching for evidence in medical education--Part 1: Sources of information. Medical teacher. 2003;25(4):352-63.
132. Gordon M, Gibbs T. STORIES statement: publication standards for healthcare education evidence synthesis. BMC medicine. 2014;12(1):143.

For peer review only

Appendices

Appendix B. Tabulated Summaries.

No.	Title	Author/year	MERSQI	COREQ	Reason for inclusion			Undergraduate (UG)/Postgraduate (PG)	Methodology	Purpose of study	Key findings
					Communication	Ethics	Professionalism				
1	The CanMEDS resume: a useful educational portfolio tool for diagnostic radiology residents	Finlay, K.<br>Probyn, L.<br>Ho, S.<br><br>2012			✓		✓	PG	We developed a comprehensive resident CanMEDS resume for ongoing documentation of resident participation, education, and developing competencies in CanMEDS Roles for diagnostic radiology.<br>This resume or portfolio summary is structured to high- light all 7 CanMEDS Roles. The resident is responsible for ongoing updates and submits this as part of his or her annual evaluation.	The objective of this article is to discuss the utility of this tool for inclusion in the educational portfolio for radiology residents.	The document increases awareness of the CanMEDS Roles and documents resident professional development, while also promoting reflection on the impor- tance of these Roles for radiology and future practice. The added advantage for program directors and assistants is up- to-date documentation of resident activities, which serves as a highly useful tool, particularly for program accreditation purposes.
2	A pilot evaluation of portfolios for quality attestation of clinical ethics consultants	Fins, Joseph J<br>Kodish, Eric<br>Cohn, Felicia<br>Danis, Marion<br>Derse, Arthur R<br>Dubler, Nancy<br>Neveloff<br>Goulden, Barbara<br>Kuczewski, Mark<br>Mercer, Mary Beth<br>Pearlman, Robert A<br><br>2016	7.5	14		✓		PG	The QAPTF committee decided on the key components of the portfolio, their respective weightages and scoring quality of each element.<br>ASBH issued an open call to individuals practicing CEC, publicized on the ASBH website, for let- ters of intent (LOI) to submit portfolios for the piloting of this first step in a quality attestation process. The QAPTF wanted to ensure a	We describe the results of a pilot project utilizing portfolios as an evaluation tool. The goals of this quality attestation pilot project were to assess the feasibility of a process to evaluate whether CE consultants are competent to perform ethics consultations as a lead or solo consultant and to develop quality metrics for this assessment process.	We found that this approach is feasible and resulted in a reasonably wide distribution of scores among the 23 submitted portfolios that we evaluated. We discuss limitations and implications of these results, and suggest that this is a significant step on the pathway to an eventual certification process for clinical ethics consultants.

For peer review only

No.	Title	Author/year	MERSQI	COREQ	Reason for inclusion			Undergraduate (UG)/Postgraduate (PG)	Methodology	Purpose of study	Key findings
					Communication	Ethics	Professionalism				
									representative distribution of candidates for the first portfolio review and adopted a method for select- ing portfolios that was intentionally designed to minimize bias.Candidates needed to have performed CEC, have at least a master's degree in a relevant discipline, and have sufficient CEC experience to be able to provide all ele- ments of the portfolio. 32 out of 82 individuals were invited to submit portfo- lios. After candidates submitted their portfolios, they were surveyed to obtain feedback on their portfolio develop- ment and submission experience. QAPTF members were also asked about their experi- ence and perceptions of whether the portfolio elements and approach to scoring functioned effectively in identify- ing candidates who should or should not proceed with the next step of		

For peer review only

No.	Title	Author/year	MERSQI	COREQ	Reason for inclusion			Undergraduate (UG)/Postgraduate (PG)	Methodology	Purpose of study	Key findings
					Communication	Ethics	Professionalism				
									quality attestation. They also were asked about ways to improve future attestation activities. This feedback is reflected in the limitations and discussion sections that follow.		
3	Portfolios and 'the competent clinician': past, present and future assessment	Gavriel, J. 2016	-	-			✓	PG	NA	To describe the evolution of portfolios, including its advantages, challenges and the future of portfolios	Portfolios have been a part of medical education since the early 1990s and many educators use them frequently, even habitually. Portfolios are primarily based on a constructivist approach to learning and there are also strong links with adult learning principles.Competence based education, once under fire for being too simplistic, now forms a significant part of medical education including the development of portfolios. In the med- ical education arena there are some advantages that are particularly relevant: triangulation amongst those working alongside learners, developing wider professional skills and demonstrating the potential for lifelong learning and excellence. For some learners and educators the portfolio is a burden and a frustration: the volume of work, a hoop jumping approach and a subjective nature all providing challenges [8] that are at least partially a result of some key complex- ities of the portfolio assessment.

For peer review only

No.	Title	Author/year	MERSQI	COREQ	Reason for inclusion			Undergraduate (UG)/Postgraduate (PG)	Methodology	Purpose of study	Key findings
					Communication	Ethics	Professionalism				
											Portfolios are evolving assessments; they are subject to continuing developments but the ongoing challenge for portfolio designers is the balance between reliability and validity on one hand and tick-boxing on the other.
4	Diffusion of an e-portfolio to assist in the self-directed learning of physicians: An exploratory study	Goliath, Cheryl Lynn<br><br>2010	13	21	✓			PG	A single case study comprised of nine physicians was used to examine the factors involved when the diffusion of an e-portfolio was introduced. The participants were studied using interviews, the Stages of Concern (SoCQ) and the Self-Directed Learning Readiness Scale (SDLRS) questionnaires, four-week trial period of I-Webfolio and field notes based upon observations during the study.	This study examined the impact of implementing an e-portfolio system within an Internal Medicine physician practice as a means of developing a process of life-long learning and professional development for the participants.	The results were analyzed to identify patterns or themes that appeared. One such pattern emerged, in that; all entries in the e-portfolio were almost exclusively in the Medical Knowledge component. A theme that became apparent was that none of the physicians in the study have developed a self-directed learning plan. The results of this study suggested that adoption of technology is multifactoral and there was no consistent individual profile that predicted optimal adoption of the innovation; however, increased scores on the SDLRS would indicate that the diffusion of I-Webfolio was effective in improving the self-directed learning of the participants.
5	The attributes of a good medical trainee: how to build your portfolio	Joseph, S. R.<br>Sikkel, M. B.<br><br>2009	-	-	✓			PG	-	Review of the attributes of a good medical trainee based on the essential physician competencies, and how then to build a good portfolio	A comprehensive and contemporaneously completed procedural, operative, teaching and reflective practice logs should be included in the portfolio, and should be commenced as soon as possible. Compose your portfolio around the seven key roles of the Canadian Medical Education Directives for Specialists model, and be innovative when

For peer review only

No.	Title	Author/year	MERSQI	COREQ	Reason for inclusion			Undergraduate (UG)/Postgraduate (PG)	Methodology	Purpose of study	Key findings
					Communication	Ethics	Professionalism				
											filling each section with concrete examples. Use reflective practice of clinical cases also as further evidence.
6	Study skills course in medical education for postgraduate residents	Bhattarai, M. D. 2007	-	-	✓			PG	-	The learners have to take active parts in the teaching learning activities. To make them aware and to help them develop the skills required, the need of the study skills course in medical education early in the part of their training has been realized for the postgraduate residents	The important areas of the study skills course focusing in the requirement of clinical components of the postgraduate residents are 1.) Interpersonal and communications skills, 2.) Teaching, learning and presentation skills, 3.) Language, reading and computer use, 4.) Evidence based medicine and diagnosis and management, 5). Assessment principles and strategies, 6). Time management strategies to get the best out of the training, 7). Reflection, portfolio and self-directed lifelong learning, and 8). Follow-up presentation. The methodologies that could be used in such study skills course are interactive lectures, brainstorming, presentations by the trainees, demonstration to and by the trainees, small group discussion, group work and presentation, group and individual feedback, practice sessions, role play, short relevant video movies, video recording of the trainees and viewing with feedback. With their already tight training schedule and posting and other similar other mandatory courses required for the postgraduate residents, much time cannot be allocated for the study skills course in medical education alone. Similar study skills course in medical education

For peer review only

No.	Title	Author/year	MERSQI	COREQ	Reason for inclusion			Undergraduate (UG)/Postgraduate (PG)	Methodology	Purpose of study	Key findings
					Communication	Ethics	Professionalism				
											may need to be arranged for the undergraduate medical students as well.
7	Fellows' perceptions of a mandatory reflective electronic portfolio in a geriatric medicine fellowship program	Ruiz, Jorge G. Qadri, Syeda S. Karides, Marina Castillo, Carmen Milanez, Marcos Roos, Bernard A. 2009	9	15	✓		✓	PG	We conducted surveys and focus groups followed by quantitative and qualitative data analysis	Electronic portfolios (ePortfolios) can be useful for evaluating and documenting mastery of competencies. We investigated geriatric medicine fellows' perceptions of an ePortfolio	Our study revealed that fellows considered the ePortfolio acceptable and accessible. Fellows reflected on aspects relevant to postgraduate training and future practice and documented adherence to competencies. However, fellows expressed resentment about the self-reflection component, which interfered with the overall acceptance of the ePortfolio. Although ePortfolios can be powerful and reflective tools for competency assessment, care must be taken in their implementation.
8	Portfolios for assessment and learning: Guide Supplement 45.1 - Viewpoint	Burch, Vanessa 2011	-	-		✓		PG	NA	NA	The critical question to ask then is Should poorly resourced countries not find ways of adapting this valuable learning and assessment strategy to suit local realities? (Spandel 1997). Clearly, the ‘Rolls Royce’ version of portfolio implementation and assessment is beyond the reach of developing countries but a less resource-intensive strategy could be a feasible option. It was with this goal in mind that the University of Cape Town (UCT) in South Africa embarked on the task of adapting portfolios for use in less well-resourced settings (Burch & Seggie 2008). Medical students in a 6-year programme are introduced to portfolio-based learning and assessment in their third year of training and each subsequent year until graduation. Currently, clinical students write up,

For peer review only

No.	Title	Author/year	MERSQI	COREQ	Reason for inclusion			Undergraduate (UG)/Postgraduate (PG)	Methodology	Purpose of study	Key findings
					Communication	Ethics	Professionalism				
											and manage under supervision, between 18 and 32 cases in 6- to 10-week medicine clerkships These patients are presented to a specialist in training on admission and the attending consultant on the post-intake ward the following morning. This provides ample opportunity for students to look back on the action, reflect on their performance and receive feedback and mentoring from senior clinicians directly in the workplace setting. In this way, consultants and specialists in training act as mentors helping students identify alternative strategies and set goals to stimulate further learning. These distinct phases of the workplace-based mentoring process closely reflect the phases of spiral professional development described by Korthagen et al. (2001). The importance of this approach is that mentors provide ongoing guidance for students during the course of performing their routine clinical duties limiting the need for additional regular meetings between students and mentors. Students who are struggling are free to consult the course convener for assistance, but an introductory lecture clearly outlining the purpose, format and examination of portfolios, as well as a set of guidelines for completing portfolio tasks has significantly limited the need for further advice. In addition to writing up a series of patient case studies, students also

For peer review only

No.	Title	Author/year	MERSQI	COREQ	Reason for inclusion			Undergraduate (UG)/Postgraduate (PG)	Methodology	Purpose of study	Key findings
					Communication	Ethics	Professionalism				
											identify at least one clinical learning need based on each case discussion; these ‘question and answer’ tasks are also included in the portfolio. This provides a good opportunity for needs-driven, focused, self-directed learning. Students also complete a range of other written tasks using the index clinical cases in their portfolios. These case-based tasks (Harden et al. 2000) include written reflections on ethical issues relevant to selected patients, a bio-psycho-social analysis (mini-ethnography) (Kleinman & Benson 2006) of selected patients and the use of case material to explore the principles of Primary Health Care (Vivian et al. 2010) as they relate to patients in the portfolio. Finally, students also write up detailed treatment plans for a list of 32 common conditions as reflected by the cases in the portfolio. These tasks are all designed to promote self-directed, patient-centred learning. At UCT it was recognised that marking of this volume of portfolio material was beyond the human resources capacity at departmental level and so a strategic decision was made to focus on an assessment strategy based on a series of structured interviews, focusing on different aspects of a sample of portfolio cases. Students rotate through four stations of 15 min each (OSCE style) at which single-examiner structured interviews are conducted based

For peer review only

No.	Title	Author/year	MERSQI	COREQ	Reason for inclusion			Undergraduate (UG)/Postgraduate (PG)	Methodology	Purpose of study	Key findings
					Communication	Ethics	Professionalism				
											on patient cases randomly selected from the portfolio. The case-based discussions focus on clinical reasoning, case management plans, bioethics, psychosocial aspects of illness and the principles of Primary Health Care. This approach assesses student performance on a 'biopsy' of at least 8–10 cases contained in the portfolio. Each case discussion is scored using a nine-point rubric (1–3 poor, 4–6 adequate, 7–9 good) and scores are totalled across all cases discussed to achieve an aggregate score for the examination. The key reasons for adopting this assessment strategy at UCT included limited human resources but, more importantly, the observation that English second language speakers, more than 50% of university admissions nationally (Council on Higher Education 2009), are better able to express themselves in oral rather than written form. Indeed, it has been asked whether portfolios provide educators with real insight into practitioners' clinical ability or simply show that they are good at writing about what they do? (Karlowicz 2000). Having developed the strategy, the key question is whether any other university in sub-Saharan Africa has successfully adopted it. To date, two other South African universities have adopted and adapted the approach to suit local needs and two other African universities have

For peer review only

No.	Title	Author/year	MERSQI	COREQ	Reason for inclusion			Undergraduate (UG)/Postgraduate (PG)	Methodology	Purpose of study	Key findings
					Communication	Ethics	Professionalism				
											successfully adopted the strategy at Makerere University (Mubuuke et al. 2010) and Kampala International University (Ezeala et al. 2010) both in Uganda. Clearly, the data are limited but it does suggest that portfolio-based learning and assessment are within reach of countries attempting to improve the quality of health sciences education using available resources. This, I believe, is one of the most important issues to be addressed in the twenty-first century – the adaptation and implementation of better teaching and assessment strategies in developing countries so as to keep abreast of educational advances developed world countries enjoy.
9	AMEE Guide No. 25: The assessment of learning outcomes for the competent and reflective physician	Shumway, J. M. Harden, R. M. 2003	-	-	✓	✓	✓	PG	-	To emphasize on the 2 important features of contemporary medical education: first is an emphasis on assessment as a tool to ensure quality in training programmes, to motivate students and to direct what they learn. The second is a move to outcome-based education where the learning outcomes are defined and decisions about the curriculum are based on these.	Portfolio assessments have a positive impact on the learning environment because they document what the learner has done and ask the learner to reflect on what she/he has accomplished. They ask the learner to put together large amounts of disparate information into a whole that tells a story about the learner's accomplishments. Portfolios are a valuable instru- ment for inclusion in the examiner's toolkit, if for no other reason than that they assess learning outcomes such as critical thinking and self-assessment, which are not easily assessed using other instruments. They also provide a record of the

For peer review only - http://bmjopen.bmj.com/site/about/guidelines.xhtml

No.	Title	Author/year	MERSQI	COREQ	Reason for inclusion			Undergraduate (UG)/Postgraduate (PG)	Methodology	Purpose of study	Key findings
					Communication	Ethics	Professionalism				
											student's performance over a period of time and are not just a snapshot at one specific point in time. The reliability of portfolios is at least in part due to the ability of raters to agree on standards and criteria for the content and assessment of portfolios.
10	A comprehensive collaborative patient safety residency curriculum to address the ACGME core competencies	Singh, R.<br>Naughton, B.<br>Taylor, J. S.<br>Koenigsberg, M. R.<br>Anderson, D. R.<br>McCausland, L. L.<br>Wahler, R. G.<br>Robinson, A.<br>Singh, G.<br><br>2005	-	-	√			PG	-	To design and implement a new patient safety curriculum in collaboration with the Schools of Nursing and Pharmacy, in such a way as to address all 6 competencies.	Porfolios is used as one of the multiple assessment approaches in the patient safety programme. However, no evaluation made on this assessment mode.
11	ECLIPPx: an innovative model for reflective portfolios in life-long learning	Cheung, C. R.<br><br>2011	-	-			√	PG	-	For healthcare professionals, the educational portfolio is the most widely used component of lifelong learning – a vital aspect of modern medical practice. When used effectively, portfolios provide evidence of continuous learning and promote reflective practice. But traditional portfolio models are in danger of becoming outmoded, in the face of changing expectations of health- care provider competences today.<br><br>Portfolios in health care have generally focused on competencies in clinical skills. How- ever, many other domains of professional	A new model for categorising the health care port- folios of professionals is proposed. The ECLIPPx model is based on personal practice, and divides the evidence of ongoing professional learning into four categories: educational development; clinical practice; leadership, innovation and professionalism; and personal experience.<br><br>The ECLIPPx model offers a new approach for personal reflection and longitudinal learn- ing, one that gives flexibility to the user whilst simultaneously encompassing the many relatively new areas of competence and expertise that are now required of a modern doctor.

For peer review only

No.	Title	Author/year	MERSQI	COREQ	Reason for inclusion			Undergraduate (UG)/Postgraduate (PG)	Methodology	Purpose of study	Key findings
					Communication	Ethics	Professionalism				
										development, such as professionalism and leadership skills, are increasingly important for doctors and health care professionals, and must be addressed in amassing evidence for training and revalidation. There is a need for modern health care learning portfolios to reflect this sea change.	
12	Development of a web-based, specialty specific portfolio	Clay, A. S., Petrusa, E., Harker, M. and Andolsek, K.<br><br>2007	-	-	✓	✓	✓	PG	Three different disciplines (anesthesiology, surgery and medicine) worked together to create a critical care medicine portfolio. We began by reviewing the curriculum requirements for critical care medicine and organise<br><br>d these requirements into the six ACGME core competencies. We then developed learner led exercises in each core competency that were specific to critical care. Each exercise includes assessment of resident knowledge and application, an evaluation of the exercise, a learner self-	This article illustrates the creation of a specialty specific portfolio that can be used by several different residency programs to document resident competence during a given rotation	Creation of specialty specific portfolio reduces redundancy between disciplines, allows for increased time to be spent on the development of exercises specific to rotation objectives, and aids program directors in the collection of portfolio entries for each resident over the course of a residency.<br><br>We describe the creation of a multidisciplinary learning portfolio that can be used by different training programs within one institution to assess trainee performance in each respective area of expertise. The strengths of the specialty specific portfolio include the ability to be specific to the learning objectives of that rotation/specialty and uniform in content across multiple disciplines within that specialty. Commitment to the portfolio from multiple disciplines allows for careful refining of the portfolio and reduction of duplicative efforts. These specialty specific portfolios could potentially be improved upon if professional

For peer review only

No.	Title	Author/year	MERSQI	COREQ	Reason for inclusion			Undergraduate (UG)/Postgraduate (PG)	Methodology	Purpose of study	Key findings
					Communication	Ethics	Professionalism				
									assessment of skill, and a review of performance by a faculty member. Portfolio entries are highlighted in a multi-disciplinary weekly conference and posted on a critical care web site at our University.		organizations were committed to their adaptation, perhaps as a component of maintenance of certification.<br>The portfolio entries as we have described are also useful for prompting frequent self reflection/evaluation and for providing trainees with feedback on their self-perceptions. Self assessment, a skill which is not often taught or evaluated in medical education, is notoriously inaccurate (Gordon 1991). However, self assessment may be improved with deliberate practice, and frequent self assessment with feedback on that self assessment (Gordon 1992; Norman & Schmidt 1992; Ericsson 2004).<br>If other specialties were to create similar portfolios, the logistics of creating a portfolio for each resident would be simplified: the portfolio could be built a rotation at a time, meeting the objectives of that rotation monthly, and meeting the ACGME requirements over the course of a residency. Furthermore, by simply requiring a certain number entries from each core competency by the end of each year or the end of a residency, the learner is given the discretion to choose those exercise which he/she feels is most relevant given the cases he/she has encountered.
13	A review of portfolio use in residency programs and	Colbert, C. Y., Ownby, A. R. and Butler, P. M.	-	-			✓	PG	Two searches of PubMed, OVID, JSTOR, SCOPUS, and	Portfolios, often described as collections of evi-dence, are discussed	There is wide varia- tion in how portfolios are utilized within U.S. residency programs. The challenge for

For peer review only

No.	Title	Author/year	MERSQI	COREQ	Reason for inclusion			Undergraduate (UG)/Postgraduate (PG)	Methodology	Purpose of study	Key findings
					Communication	Ethics	Professionalism				
	considerations before implementation	2008							FirstSearch Wilson Select were conducted between October 2006 and April 2007 to identify studies and articles related to portfolio usage. Thirty-nine articles met criteria and were reviewed.	as a means of teaching or assessing the Ac- creditation Council for Graduate Medical Education competencies. Yet, it is unclear how many residency programs utilize portfolios. The purpose of this article is to (a) review the literature on portfolio use in graduate medical education; (b) examine efficacy of portfolio use, based upon studies in the field; and (c) offer a discussion of con- siderations for implementing portfolios.	graduate medical education is to create consensus on the definition and purpose of portfolios, such that best practices in portfolio implementation and assessment can be achieved.<br><br>The case for utilizing portfolios in residency education is compelling. Portfolios can offer a diverse palette of learners' achievements and experiences. When assessing competence from a holistic standpoint, the range of behaviors captured in a portfolio may render it superior to more traditional assessment practices. Self-awareness and self-directed learning, thought to be critical to the development of competent physicians, can be enhanced through the act of portfolio development,19 as port- folios can force one to reflect upon strengths, weaknesses, and opportunities for growth.<br><br>Before implementing a portfolio system in a residency pro- gram, several issues need to be considered. Learners and faculty should be trained on the portfolio process including the purpose, structure, and content of portfolios. Mentoring may be required when it comes to content assembly and interpretation. If portfolios are to be used for summative assessment pur- poses, an evaluation rubric—a scoring template—will need to be

For peer review only

No.	Title	Author/year	MERSQI	COREQ	Reason for inclusion			Undergraduate (UG)/Postgraduate (PG)	Methodology	Purpose of study	Key findings
					Communication	Ethics	Professionalism				
											created.<br><br>Last, the promise offered by portfolios is tempered by diffi-culties associated with their use. The lack of a standard defini- tion across the field and the dearth of outcome studies to assess efficacy are pressing concerns. The challenge now is to create consensus on the definition and purpose of portfolios within res- idency programs, such that collaboration within the field may begin to offer best practices in the areas of implementation and assessment.
14	How we teach ethics and communication during a Canadian neonatal perinatal medicine residency: an interactive experience	Daboval, T., Moore, G. P. and Ferretti, E.<br><br>2013	6.5		✓	✓	✓	PG	A revised ethics program implemented in 2009 identified competencies that should be demonstrated at the end of the Neonatal-Perinatal Medicine (NPM) residency. Several seminars were refined while new workshops, problem-based learning in ethics, and a personal portfolio were added.	This article describes the process by which a well-delineated, interactive program to teach ethical reasoning and skillful communication with parents was implemented at the University of Ottawa, Canada.	All teaching strategies were well received based on the average level of satisfaction (5.8 out of 7, SD 0.4). We are now moving forward by formally assessing our program including the impact on knowledge acquisition and behavior.<br><br>A dedicated, interactive competency-based neonatal ethics teaching program is vital to support NPM trainees in learning how to integrate ethical thinking with competencies in communication.<br><br>A dedicated, interactive, competency-based neonatal ethics teaching program with well-identified expectations and eval-uation is vital so that all graduating NPM trainees will not only have knowledge of key ethical precepts and ethical reasoning, but will also have learned how to integrate ethical thinking when they communicate and interact

For peer review only

No.	Title	Author/year	MERSQI	COREQ	Reason for inclusion			Undergraduate (UG)/Postgraduate (PG)	Methodology	Purpose of study	Key findings
					Communication	Ethics	Professionalism				
											professionally with parents during ethically and emotionally charged clinical situations. In this manner, our teaching is supporting a paradigm shift in neonatology from a paternalistic to a shared decision-making model. To advance our methods of education in this area, formal assessments of such teaching programs must occur.
15	Portfolio as a tool to stimulate teachers' reflections	Tigelaar, D. E. Dolmans, D. H. de Grave, W. S. Wolfhagen, I. H. van der Vleuten, C. P.<br><br>2006	-	15	✓			PG	In this study, all the written portfolio assignments were approached as reflections on teacher functioning. In order to gain insight into the portfolio process, we collected the teachers' final portfolio assignments as well as data about the process of portfolio construction. In this way, we obtained portfolio data comprising written portfolio assignments and final reflective portfolio assignments. Firstly, the teachers were asked to describe the course of their teaching career. They were also asked to describe critical incidents in their development as a teacher, set learning goals,	Portfolios are increasingly being used to stimulate teachers' reflections. Frameworks for reflection on teaching often emphasize competencies and behaviours. However, other aspects of teacher functioning are also important, such as the teaching environment and individual teachers' beliefs, professional identity and mission. In a study among five medical school teachers, we explored how a portfolio stimulated reflections on the various aspects of teaching functioning.	The analysis yielded examples of reflections on all aspects of Korthagen's model, although reflections on competencies proved easiest to identify.

For peer review only

No.	Title	Author/year	MERSQI	COREQ	Reason for inclusion			Undergraduate (UG)/Postgraduate (PG)	Methodology	Purpose of study	Key findings
					Communication	Ethics	Professionalism				
									select artefacts in evidence of their teaching activities, compose a profile of a good teacher and reflect on that. The final assign- ment required them to compose a reflective portfolio, using the previous assignments as points of departure. The assignments were intended to stimulate reflection on all aspects of teacher functioning as defined in Korthagen's model. However, the critical incidents assignment was focused on the environment, behaviour, and competencies. Table 1 presents brief descriptions of the assignments.		
16	A method for defining competency-based promotion criteria for family medicine residents	Torbeck, L. Wrightson, A. S. 2005	-	-	✓		✓	PG	In 2003, both of us began the task of developing promotion criteria by searching the literature for studies identifying standards or criteria for resident promotion. Few studies were found related to residents' progress and	The Accreditation Council for Graduate Medical Education (ACGME) has mandated a shift from a structure- and process-based educational system to a competency-based system. The ACGME has not provided criteria (standards), preferring to leave that to the discretion	The value of promotion criteria Training residents to become competent health care providers has been and always will be an important and challenging endeavor. With the ACGME's new mandate, many program directors and house office staff are looking for useful guidance to make their residency programs compliant. We believe that developing a

For peer review only

No.	Title	Author/year	MERSQI	COREQ	Reason for inclusion			Undergraduate (UG)/Postgraduate (PG)	Methodology	Purpose of study	Key findings
					Communication	Ethics	Professionalism				
									promotion.[2,7] We then reviewed the information contained within the last three Residency Assistance Program workshops of the American Academy of Family Physicians, 2001–2003. Frey et al.'s[8] 2003 outpatient core competency presentation notes were earmarked as having specific core competencies that could easily be rewritten as promotion criteria. Next, we reviewed the residency curriculum of the Department of Family Practice and Community Medicine at the University of Kentucky College of Medicine and listed promotion criteria that we thought were important for Family Medicine residents at each level of that curriculum to achieve. Taking this list and extrapolating from Frey et al.'s work, we composed a list of working promotion criteria.	of the individual training programs. Such criteria and an overall strong evaluation process are essential for residents to attain the appropriate knowledge, skills, and attitudes. With this need in mind, the authors describe an evaluation process in which they developed ACGME-competency-based promotion criteria for family medicine residents at the University of Kentucky College of Medicine in 2004.	set number of promotion criteria that are competency-based should be among the first tasks residency programs should tackle, for several reasons.<br><br>First, having defined criteria will make the three to seven years of residency performance assessment more educationally sound. The education literature reminds us that two components are mandatory to a performance assessment: the task and the criteria.[11,12] Since continuous performance assessment is carried out during the majority of a resident's training, the awareness of what is expected of the resident can be raised for both the resident and the faculty by making the task and criteria clearer to them. Residents then are better able to demonstrate that which is expected of them, and faculty are better prepared to evaluate them.<br><br>Second, having defined promotion criteria can better target remediation in those residents failing to meet the criteria in a timely manner. Intervention during a time early on in the resident's development is much more beneficial than it is just before making a decision to promote or not promote.<br><br>Third, having criteria can improve faculty development, especially in terms of how to assess competence, how to give feedback, and how to account for faculty

For peer review only

No.	Title	Author/year	MERSQI	COREQ	Reason for inclusion			Undergraduate (UG)/Postgraduate (PG)	Methodology	Purpose of study	Key findings
					Communication	Ethics	Professionalism				
											responsibility in training and evaluating competent physicians.
17	Enhanced skills in global health and health equity: Guidelines for curriculum development	Dawe, R., Pike, A., Kidd, M., Janakiram, P., Nicolle, E. and Allison, J.<br><br>2017	10	10		✓		PG	A panel comprised of 34 experts in global health education and practice completed three rounds of a Delphi small group process.	To generate consensus on the essential components of a Global Health/Health Equity Enhanced Skills Program in family medicine.	Consensus (defined as ≥ 75% agreement) was achieved on program length (12 months), inclusion of both domestic and international components, importance of mentorship, methods of learner assessment (in-training evaluation report, portfolio), four program objectives (advocacy, sustainability, social justice, and an inclusive view of global health), importance of core content, and six specific core topics (social determinants of health, principles and ethics of health equity/global health, cultural humility and competency, pre and post-departure training, health systems, policy, and advocacy for change, and community engagement).<br><br>Panellists agreed on a number of program components forming the initial foundation for an evidence- informed, competency-based Global Health/Health Equity Enhanced Skills Program in family medicine.
18	Learning portfolios in radiology residency education: how do I get started?	Deitte, L.<br><br>2008	-	-	✓		✓	PG	-	The purpose of this article is three-fold: (1) to famil- iarize the reader with learning portfolios in medical edu-cation, (2) to outline the new radiology ACGME re- quirements for a portfolio, and (53) to discuss strategies for the implementation of a portfolio as a learning and	As medical education and continuous professional devel- opment shift toward an outcomes-based model, there is increasing focus on documentation of physician self-as- sessment and a commitment to lifelong learning. The ACGME program requirements for diagnostic radiology graduate medical education effective July 1, 2008, in- clude learning portfolios as

For peer review only

No.	Title	Author/year	MERSQI	COREQ	Reason for inclusion			Undergraduate (UG)/Postgraduate (PG)	Methodology	Purpose of study	Key findings
					Communication	Ethics	Professionalism				
										assessment tool in a residency education program and continued professional development..	one method for this docu- mentation. Although there are some challenges associ- ated with the implementation of learning portfolios, it is likely that portfolios will prove to be a valuable learning and assessment tool not only in graduate medical educa- tion but also throughout a physician's career and recerti- fication process.
19	Evaluation of an established learning portfolio	Vance, G. Williamson, A. Frearson, R. O'Connor, N. Davison, J. Steele, C. Burford, B.<br><br>2013	9	-	✓			PG	Deanery-wide trainee attitudes were surveyed by an electronic questionnaire in 2009 and compared with perceptions recorded during the pilot phase (2004–2005).	The trainee-held learning portfolio is integral to the foundation programme in the UK. In the Northern Deanery, portfolio assessment is standar- dised through the Annual Review of Competence Progression (ARCP) process. In this study we aimed to establish how current trainees evaluate portfolio-based learning and ARCP, and how these attitudes may have changed since the foundation programme was first introduced.	Many trainees continue to view the e-portfolio negatively. Indeed, significantly fewer train- ees in 2009 thought that the e-portfolio was a 'good idea' or a 'worthwhile investment of time' than in 2005. Trainees remain unconvinced about the educa- tional value of the e-portfolio: fewer trainees in 2009 regarded it as a tool that might help focus on training or recognise individual strengths and weaknesses. Issues around unnecessary bureaucracy persist. Current trainees tend to understand how to use the e- portfolio, but many did not know how much, or what evidence to collect. Few supervisors were re- ported to provide useful guidance on the portfolio. ARCP encouraged portfolio completion but did not give meaningful feedback to drive future learning.<br><br>Continued support is needed for both trainees and supervisors in portfolio-building skills and in using the e-portfolio as an educational tool. Trainee-tailored feedback is needed to ensure that portfolio-

No.	Title	Author/year	MERSQI	COREQ	Reason for inclusion			Undergraduate (UG)/Postgraduate (PG)	Methodology	Purpose of study	Key findings
					Communication	Ethics	Professionalism				
											based assessment promotes lifelong, self-directed and reflective learners.<br>Trainees remain unconvinced about the educational value of the e-portfolio
20	Reflective Practice for Patient Benefit: An Analysis of Doctors' Appraisal Portfolios in Scotland	Wakeling, J.<br>Holmes, S.<br>Boyd, A.<br>Tredinnick-Rowe, J.<br>Cameron, N.<br>Marshall, M.<br>Bryce, M.<br>Archer, J.<br><br>2019	-	16	✓			PG	Using an in-depth case study approach, 18 online portfolios in Scotland were examined with a template developed to record the types of supporting information submitted and how far these showed reflection and/or changes to practice. Data from semistructured interviews with the doctors (n = 17) and their appraisers (n = 9) were used to contextualize and broaden our understanding of the portfolios.	Reflective practice has become the cornerstone of continuing professional development for doctors, with the expectation that it helps to develop and sustain the workforce for patient benefit. Annual appraisal is mandatory for all practicing doctors in the United Kingdom as part of medical revalidation. Doctors submit a portfolio of supporting information forming the basis of their appraisal discussion where reflection on the information is mandated and evaluated by a colleague, acting as an appraiser.	Portfolios generally showed little written reflection, and most doctors were unenthusiastic about documenting reflective practice. Appraisals provided a forum for verbal reflection, which was often detailed in the appraisal summary. Portfolio examples showed that reflecting on continued professional development, audits, significant events, and colleague multisource feedback were sometimes considered to be useful. Reflecting on patient feedback was seen as less valuable because feedback tended to be uncritical.<br><br>The written reflection element of educational portfolios needs to be carefully considered because it is clear that many doctors do not find it a helpful exercise. Instead, using the portfolio to record topics covered by a reflective discussion with a facilitator would not only prove more amenable to many doctors but would also allay fears of documentary evidence being used in litigation.
21	Developing an Evaluation Tool for Assessing Clinical Ethics Consultation Skills in Simulation Based	Wasson, K.<br>Parsi, K.<br>McCarthy, M.<br>Siddall, V. J.<br>Kuczewski, M.<br><br>2016	-	-		✓		PG	At the beginning of the project development, four of the authors (who are bioethicists with experience in	The American Society for Bioethics and Humanities has created a quality attestation (129) process for clinical ethics consultants;	Given the move toward quality attestation by ASBH and the need to develop a way of assessing the interpersonal skills of clinical ethics consultants ‘‘live’’ we have developed the ACES

For peer review only

No.	Title	Author/year	MERSQI	COREQ	Reason for inclusion			Undergraduate (UG)/Postgraduate (PG)	Methodology	Purpose of study	Key findings
					Communication	Ethics	Professionalism				
	Education: The ACES Project								clinical ethics consultation) agreed that any tool created should be grounded in the ASBH core competencies, as they are the articulated professional standards in the field. After a review of the limited assessment tools available, we agreed to draw from the VA's Ethics Consultant Proficiency Assessment Tool (2014). It draws on the ASBH core competencies, including the categories of Interpersonal Skills, Process Skills, Analytic Skills, and Core Knowledge, is quite comprehensive, and has been in use for a number of years. In addition, we followed Stufflebeam's (2000) guidelines for creating a checklist evaluation tool.<br><br>Given the focused usage of the ACES tool—clinical ethics simulation in an educational setting—we met regularly to	the pilot phase of reviewing portfolios has begun. One aspect of the QA process which is particularly challenging is assessing the interpersonal skills of individual clinical ethics con- sultants. We propose that using case simulation to evaluate clinical ethics consul- tants is an approach that can meet this need provided clear standards for assessment are identified. To this end, we developed the Assessing Clinical Ethics Skills (ACES) tool, which identifies and specifies specific behaviors that a clinical ethics consultant should demonstrate in an ethics case simulation. The aim is for the clinical ethics consultant or student to use a videotaped case simulation, along with the ACES tool scored by a trained rater, to demonstrate their competence as part of their QA portfolio.	tool. Our tool provides a clear structure for key domains, behaviors, and interpersonal skills that can be observed and scored by a trained rater during simulated ethics consultations. Individuals completing the ACES course or using the tool can opt to include their evaluation and video as part of their QA portfolio as a means of demonstrating their interpersonal skills in a simulated ethics consultation. Our tool also provides a framework and structure for the content of an ethics consultation and helps translate the core competencies into practical areas, behaviors, and skills. We spent a great deal of time thinking about and debating which items from the VA tool to include in the final ACES tool. It is not intended to be comprehensive for all aspects of clinical ethics consultation, e.g., for simplicity in the pilot stage we did not examine the clinical ethics consultant's ability to review and decipher the medical chart or write a note, though these elements could be added to the simulations in future. Instead, it focuses on those elements we thought could be captured in simulated clinical ethics consultations in an educational setting in this pilot phase. In the future, other elements of ethics consultation and levels of complexity can be added to the simulations as well as additional cases.

For peer review only

No.	Title	Author/year	MERSQI	COREQ	Reason for inclusion			Undergraduate (UG)/Postgraduate (PG)	Methodology	Purpose of study	Key findings
					Communication	Ethics	Professionalism				
									determine which items from the VA tool would be necessary for this purpose. Our tool is not meant to be a comprehensive tool to assess every element of an ethics consultation or CEC's role, thus it is briefer than the VA tool. It is also designed to be used in ''real time'' by trained bioethics raters, not retrospectively self-administered by the ethics consultant. Once we agreed upon the primary items, we met weekly for 3–4 months to identify and determine the specific behaviors and skills we would be evaluating under each main item. This was a time consuming process involving in-depth discussion and debate. Before each weekly meeting, we viewed videos of simulated ethics consultations from past graduate bioethics courses to refine our tool, its specified items, and behaviors. We discussed and		For clarity of assessment, we developed the ACES tool to be used for individual consultants. We believe each individual ethics consultant needs to have this set of skills, even if they work within a small team model in practice. If the ACES tool was adapted to assess a small team of ethics consultants, the raters would have to rate each consultant individually or rate them all as one ''ethics consultant'' to assess whether the team covered all the necessary items on the evaluation. This approach would be more complicated to assess and, we suspect, much less straightforward to score. At this stage, we do not recommend using the ACES tool in this way. One of the most challenging areas in the ACES tool development was how to view and rate students in a similar way—or inter-rater agreement. We spent a great deal of time discussing what qualifies as ''Done,'' ''Not Done'' or ''Done Incorrectly'' for each and every item. Rater agreement is an ongoing process which needs to be practiced and refreshed, and is not unique to our setting. We plan to assess and recalibrate rater agreement prior to each time a course is offered (1–2 per annum). What makes it more challenging in clinical ethics is the nature of some parts of the consultation; e.g., identifying and clarifying the ethical issues. What counts as ''Done'' for certain items in

For peer review only - http://bmjopen.bmj.com/site/about/guidelines.xhtml

No.	Title	Author/year	MERSQI	COREQ	Reason for inclusion			Undergraduate (UG)/Postgraduate (PG)	Methodology	Purpose of study	Key findings
					Communication	Ethics	Professionalism				
									agreed on all of the behaviors and skills listed under each item in the ACES tool. In the end, we agreed on 12 main items and 35 corresponding behaviors to be evaluated and scored.		our tool can be interpreted differently depending on the rater's background and potential biases. This type of item— communication and interpersonal skills in ethics—is not always as neat and clear cut as in other clinical areas such as surgical preparation checklists or putting in a central line. Further work needs to be done to train additional raters if we are to use the tool with more people over time. We plan to recruit additional students to take the ACES course and are working on refining the cases used and plan to evaluate the tool in other settings to obtain feedback on its reliability.<br>When we used the ACES tool in the basic clinical ethics consultation course the feedback was overwhelmingly positive. Students appreciated a clear articulation of what was expected in an ethics consult. While recognizing the tool was not designed to be comprehensive, they valued having a structure and framework for thinking about the elements of an ethics consultation and the behaviors they should be exhibiting. It gave them a clear standard by which to assess themselves and others.
22	Evaluating practice-based learning and improvement: efforts to improve acceptance of portfolios	Fragneto, R. Y., Dilorenzo, A. N., Schell, R. M. and Bowe, E. A.<br><br>2010	8		✓		✓	PG	Intensive education about the goals and importance of portfolios began in January 2006, including presentations at departmental conferences and	To find out whether educational efforts improved acceptance of the portfolio and retrospectively audited the portfolio evaluation forms completed by faculty advisors.	Portfolios are considered a best methods techni- que by the ACGME for evaluation of practice-based learning and improvment. We have found that intensive education about the goals and importance of portfolios can enhance acceptance of this evaluation tool, resulting

For peer review only

No.	Title	Author/year	MERSQI	COREQ	Reason for inclusion			Undergraduate (UG)/Postgraduate (PG)	Methodology	Purpose of study	Key findings
					Communication	Ethics	Professionalism				
									one-on-one education sessions. Faculty advisors were instructed to evaluate each resident's portfolio and complete a review form. We retrospectively collected data to determine the percentage of review forms completed by faculty. The portfolio reviews also assessed the percentage of 10 required portfolio components residents had completed.		in improved compliance in completion and evaluation of portfolios.
23	Use of portfolios for assessment of global health residents: qualitative evaluation of design and implementation	Gibson, C., Chandratilake, M. and Hull, A.<br><br>2018	-	22	✓			PG	A qualitative study was conducted with academic experts in Canadian residency training, as well as directors and residents involved in Global Health study in order to assess the validity and benefit of such a tool. Through an online survey, interviews, and focus groups, views on the portfolio and intended content were collected and coded thematically.	To determine the robustness of portfolios as an assessment tool for Global Health Residents.	This qualitative evaluation validated the use of portfolios for this cohort of students while yielding comments and suggestions that will further enhance the interactive and flexible nature of this seldom used assessment tool. These findings contribute to the understanding of how Global Health assessment can remain individualized yet rigorous.
24	Assessment and Change: An Exploration of	Jocelyn M. Lockyer, PhD; Sanjeev Sockalingam, MD,	-	16	✓			PG	A total of 5000 entries from 2195 psychiatrists were	Fellows of the Royal College of Physicians and	There were 3841 entries for analysis; 1159 entries did not meet the criteria for

For peer review only

No.	Title	Author/year	MERSQI	COREQ	Reason for inclusion			Undergraduate (UG)/Postgraduate (PG)	Methodology	Purpose of study	Key findings
					Communication	Ethics	Professionalism				
	Documented Assessment Activities and Outcomes by Canadian Psychiatrists	MHPE, FRCPC; Craig Campbell, MD, FRCPC<br><br>2018							examined. A thematic analysis drawing on the framework analysis was undertaken of the 2016 entries.	Surgeons of Canada are required to participate in assessment activities for all new 5-year cycles beginning on or after January 2014 to meet the maintenance of certification program requirements. This study examined the assessment activities which psychiatrists reported in their maintenance of certification e-portfolios to determine the types and frequency of activities reported; the resultant learning, planned learning, and/or changes to the practice they planned or implemented; and the interrelationship between the types of assessment activities, learning that was affirmed or planned, and changes planned or implemented.	assessment. The most commonly reported activities were self-assessment programs, feedback on teaching, regular performance reviews, and chart reviews. Less frequent were direct observation, peer supervision, and reviews by provincial medical regulatory authorities. In response to the data, psychiatrists affirmed that their practices were appropriate, identified gaps they intended to address, planned future learning, and/ or planned or implemented changes. The assessment activities were internally or externally initiated and resulted in no or small changes (accommodations and adjustments) or redirections.<br><br>Psychiatrists reported participating in a variety of assessment activities that resulted in variable impact on learning and change. The study underscores the need to ensure that assessments being undertaken are purposeful, relevant, and designed to enable identification of outcomes that impact practice.
25	Development of a portfolio of learning for postgraduate family medicine training in South Africa: a Delphi study	Jenkins, L., Mash, B. and Derese, A.<br><br>2012	-	10	✓	✓		PG	A workshop held at the WONCA Africa Regional Conference in 2009 explored the purpose and broad contents of the portfolio. The 85 training outcomes, ideas from the WONCA workshop, the literature, and	This study aimed to achieve a consensus on the contents and principles of the first national portfolio for use in family medicine training in South Africa.	A portfolio was developed and distributed to the eight departments of Family Medicine in South Africa, and the CMSA, to be further tested in implementation. This was the first attempt to reach consensus on the development of a national portfolio for family medicine training in South Africa. Consensus was reached on 50 items to include, and 26

No.	Title	Author/year	MERSQI	COREQ	Reason for inclusion			Undergraduate (UG)/Postgraduate (PG)	Methodology	Purpose of study	Key findings
					Communication	Ethics	Professionalism				
									existing portfolios in the various universities were used to develop a questionnaire that was tested for content validity by a panel of 31 experts in family medicine in South Africa, via the Delphi technique in four rounds. Eighty five content items (national learning outcomes) and 27 principles were tested. Consensus was defined as 70% agreement. For those items that the panel thought should be included, they were also asked how to provide evidence for the specific item in the portfolio, and how to assess that evidence.		principles relating to the port- folio. A draft national portfolio and portfolio guide have been developed and distributed to all the medical schools in the country. Further revision and testing with registrars in training is underway, with the aim to deli- ver a final portfolio in the following year.
26	The national portfolio of learning for postgraduate family medicine training in South Africa: experiences of registrars and supervisors in clinical practice	Jenkins, L., Mash, B. and Derese, A.<br><br>2013	-	15	✓			PG	Semi-structured interviews were conducted with 17 purposively selected registrars and supervisors from all eight South African training programmes.	The aim of this study was to explore registrars' and supervisors' experience regarding the portfolio's educational impact, acceptability, and perceived usefulness for assessment of competence.	The learning portfolio had a significant educational impact in shaping work- place based supervision and training and providing formative assessment. Its acceptability and usefulness as a learning tool should increase over time as supervisors and registrars become more competent in its use. There is a need to clarify how it will be used in summative assessment.
27	Demonstration of Portfolios to Assess	PATRICIA S. O'SULLIVAN1,* , MARK D. RECKASE2, TINA	8	12	✓		✓	PG	22 participants in this demonstration project were all	Describes assessment of the reliability and validity of portfolios in a	We must continue to emphasize some important qualities of portfolios: res- ident choice and reflection.

For peer review only

No.	Title	Author/year	MERSQI	COREQ	Reason for inclusion			Undergraduate (UG)/Postgraduate (PG)	Methodology	Purpose of study	Key findings
					Communication	Ethics	Professionalism				
	Competency of Residents	MCCLAIN3, MILDRED A. SAVIDGE1 and JAMES A. CLARDY3<br><br>2004							residents in the psychiatry residency education program at the primary affiliate of a state university medical school.	psychiatry residency program.	These are important to the life-long learner. Portfolio has been able to aid the faculty in meeting ACGME expectations and has oriented programe to the increasingly important role of evaluation.
28	Developing and Successfully Implementing a Competency-Based Portfolio Assessment System in a Postgraduate Family Medicine Residency Program	Laura A. McEwen, PhD, Jane Griffiths, MD, CCFP, FCFP, and Karen Schultz, MD, CCFP, FCFP<br><br>2015	-	-	✓			PG	-	The use of portfolios in postgraduate medical residency education to support competency development is increasing; however, the processes by which these assessment systems are designed, implemented, and maintained are emergent. The authors describe the needs assessment, development, implementation, and continuing quality improvement processes that have shaped the Portfolio Assessment Support System (PASS) used by the postgraduate family medicine program at Queen's University since 2009.	The authors discuss the identification of impact measures at the individual, programmatic, and institutional levels and the ways the department uses these to monitor how PASS supports competency development, scaffolds residents' self- regulated learning skills, and promotes professional identity formation. They describe the "academic advisor" role and provide an appendix covering the portfolio elements. Reflection elements include learning plans, clinical question logs, confidence surveys, and reflections about continuity of care and significant incidents. Learning module elements cover the required, online bioethics, global health, and consult- request modules. Assessment elements cover each resident's research project, clinical audits, presentations, objective structured clinical exam and simulated office oral exam results, field notes, entrustable professional activities, multisource feedback, and in-training evaluation reports. Document elements are the resident's continuing medical education activities including procedures log, attendance log, and patient demographic summaries.

For peer review only

No.	Title	Author/year	MERSQI	COREQ	Reason for inclusion			Undergraduate (UG)/Postgraduate (PG)	Methodology	Purpose of study	Key findings
					Communication	Ethics	Professionalism				
29	Diving for PERLS Diving for PERLS Working and Performance Portfolios for Evaluation and Reflection on Learning	Linda E. Pinsky, MD, Kelly Fryer-Edwards, PhD<br><br>2004	-	-	✓		✓	PG	-	Professional competence requires a commitment to lifelong learning, self-assessment, and excellence. Complex skills such as these require flexible and comprehensive teaching and assessment measures. We describe a combination of working and performance portfolios that both foster and evaluate the development of professional competence. We explain the con- ceptual and practical underpinnings that maximize the effec- tiveness of these tools. Drawing on experience with University of Washington residents, we identify 5 criteria that can help promote successful use of portfolios: separate working and performance functions of portfolios, developing a supportive climate, developing skills in faculty and residents, observing progress over time, and fostering mentorship opportunities.	To incorporate portfolios successfully into a residency program, we recommend including 5 elements (Table 1). We had success in our program by introducing the program slowly, and dedicating time to faculty education on men- toring, reflection, goal setting, and feedback skills, recog- nizing that the use of portfolios requires new skills and a cultural transformation. We are pleased by the residents' appreciation of the process, their honesty in sharing and evaluating personal areas of challenge, and their incorpora- tion of this approach into other areas of their education. For example, a current inpatient chief resident, based on his ex- perience of the importance of goal setting to learning, revised the structure of morning report to include weekly goal-setting sessions as well as a method to evaluate its effectiveness. Residents seeking employment report finding components of the portfolio helpful in creating their CVs, crafting their applications, and as display examples at job applications.<br><br>To date, the literature reports few trials of the portfolio method for assessing professional competence. The Post Graduate Education Accreditation (PGEA) in Britain illus- trates sharing responsibility for learning between teachers and learners. PGEA experimented with portfolios

No.	Title	Author/year	MERSQI	COREQ	Reason for inclusion			Undergraduate (UG)/Postgraduate (PG)	Methodology	Purpose of study	Key findings
					Communication	Ethics	Professionalism				
											as a flex- ible, targeted means of meeting continuing medical edu- 18 –20 cation (CME) requirements. In their pilot study, physicians were responsible for identifying their own learning objectives and outlining how they were going to achieve and document those objectives. The physicians each met with a mentor to review appro- priate objectives and realistic means for achieving them. Structured reflection was built into the process and was seen as strength, explicitly moving away from the passive approach of most traditional lecture-based CME courses. In the PGEA experience, the shortcomings identified were time and mentor availability. However, participants found the “deep learning” and personal mastery they achieved to be well worth it. In addition, accrediting bodies had something of substance to review in assessing the practitioners’ continuing education. Portfolios fill a need within training programs to foster 21–23 and document professional competencies. dents, training is a time of professional identity formation. The current format of most residencies — didactic confer- ences and time-consuming clinical work—emphasizes either a passive or unreflective learner role. Through portfolios, residents can experience

No.	Title	Author/year	MERSQI	COREQ	Reason for inclusion			Undergraduate (UG)/Postgraduate (PG)	Methodology	Purpose of study	Key findings
					Communication	Ethics	Professionalism				
											and develop the skills of lifelong learning, critical thinking, self- assessment, and excellence that can be carried forward into their future careers and practices. They can complete residency with a clearer sense of their own strengths and limitations, and a sense of who they are as physicians.
30	How do postgraduate GP trainees regulate their learning and what helps and hinders them? A qualitative study	Sagasser, M. H., Kramer, A. W. and van der Vleuten, C. P. 2012	-	18	✓			PG	In a qualitative study with a phenomenologic approach we interviewed first- and third-year GP trainees from two universities in the Netherlands. Twenty-one verbatim transcripts were coded. Through iterative discussion the researchers agreed on the interpretation of the data and saturation was reached.	To explore how postgraduate trainees regulate their learning in the workplace, how external regulation promotes self-regulation and which elements facilitate or impede self-regulation and learning.	Trainees used a short and a long self-regulation loop. The short loop took one week at most and was focused on problems that were easy to resolve and needed minor learning activities. The long loop was focused on complex or recurring problems needing multiple and planned longitudinal learning activities. External assessments and formal training affected the long but not the short loop. The supervisor had a facilitating role in both loops.
31	First year specialist trainees' engagement with reflective practice in the e-portfolio	Helen McNeill • Jeremy M. Brown • Nigel J. Shaw 2010	7.5	12	✓			PG	A modified Delphi technique was used to develop a grading system to indentify the level of reflection recorded by participants in the e-portfolio. Transcripts of the reflective accounts were then analysed using a qualitative approach which involved coding and categorising	This study aims specifically to explore how they have engaged in reflection on their practice and how they utilise their learning portfolio to document evidence of this.	Findings indicate there is variation is the extent to which doctors both engage in and document evidence of reflection. Further research is needed to explore factors that enable or inhibit the use of the e-portfolio for reflection and whether recorded reflection is a true picture of the cognitive process involved.

For peer review only

No.	Title	Author/year	MERSQI	COREQ	Reason for inclusion			Undergraduate (UG)/Postgraduate (PG)	Methodology	Purpose of study	Key findings
					Communication	Ethics	Professionalism				
									the data. This study demonstrated a wide variation in both the quantity and quality of reflection. Of particular note in the qualitative data analysis were themes relating to clinical knowledge and skills, learning in practice, communication, feelings, types of learning experience reflected on and wider aspects of medical practice.		
32	Development of a learning portfolio to assess the competency of anesthesia residents in Thailand	Suraseranivongse, S., Chinachoti, T., Aroonpruksakul, N., Halilamien, P., Rushatamukayanunt, P., Raksamani, K., Sirivanasandha, B. and Mandee, S.<br><br>2011	12.5	8			✓	PG	Learning portfolio was developed from Thai Medical Council general competencies, academic activities, and performance assessment in several modalities. Twenty-four first year anesthesia residents and eight mentors were enrolled for this study. One staff mentored three residents and rated their competencies in portfolios, twice, four-months apart. Content validity was assessed by six content experts. Concurrent validity of portfolio was determined	Develop a portfolio for learning improvement in first year anesthesia residents in Thailand, and validate this portfolio as a competency evaluation and to identify strength and weakness of implementation.	All content experts accepted that this portfolio could assess general competencies of the first year anesthesia residents. The majority of mentors and residents (>70%) agreed with the benefit of portfolio based on learning development and competency assessment. However, half of residents were not satisfied with the burden from portfolio

For peer review only

No.	Title	Author/year	MERSQI	COREQ	Reason for inclusion			Undergraduate (UG)/Postgraduate (PG)	Methodology	Purpose of study	Key findings
					Communication	Ethics	Professionalism				
									by agreement with faculty global rating and in-training examination. Inter-rater reliability of portfolio was evaluated by five faculties that rated 24 residents. Practicality was commented upon by all mentors and residents in the questionnaire and semi-structure, open-ended questions.		
33	Education Research: Communication skills for neurology residents - Structured teaching and reflective practice	Watling, C. J. and Brown, J. B.<br><br>2007	8.5	10	✓	✓		PG	A group of 12 neurology residents participated in a series of six case-based communi-cation skills workshops. Each workshop focused on a particular clinical scenario, including break-ing bad news, discussing do-not-resuscitate orders, communicating with “difficult” patients, disclosing medical errors, obtaining informed consent for neurologic tests and procedures, and discussing life-and-death decisions with families of critically ill patients.	Despite the importance of communication skills for neurologists, specific training in this area at the residency level is often lacking. This study aimed to enhance learning of these skills and to encourage reflective practice around communication skills.	The program was well accepted, and residents rated the workshops as effective and relevant to their practice. Analysis of residents’ portfolios revealed three themes relevant to patient–physician communication: 1) communication is more successful when adequate time is allowed, 2) the ability to empathize with patients and their families is essential to successful interactions, and 3) the develop- ment of specific approaches to challenging scenarios can facilitate effective interactions. The portfo- lios also demonstrated that residents would engage in reflective practice.<br><br>Targeting of communication skills training around specific clinical scenarios using neurologic cases was well accepted and was deemed relevant to practice. The use of portfolios may

For peer review only

No.	Title	Author/year	MERSQI	COREQ	Reason for inclusion			Undergraduate (UG)/Postgraduate (PG)	Methodology	Purpose of study	Key findings
					Communication	Ethics	Professionalism				
									Residents also kept reflective portfolios in which real examples of these interactions were recorded.		promote lifelong learning in this area.
34	What the HEC-C? An Analysis of the Healthcare Ethics Consultant-Certified Program: One Year in	Horner, C. Childress, A. Fantus, S. Malek, J.<br><br>2020			✓	✓		PG	-	In this paper, we explore the history that has led to this certification process, and evaluate the ability of the HEC-C Program to meet the goals it has set forth for HCECs. We describe the benefits and weak- nesses of the program and offer constructive feedback on how the process might be strengthened, as well as share our team's experience in preparing for the exam.	Ultimately, while we are generally supportive of efforts to professionalize the field and ensure competence among HCECs, we are skeptical that a limited process such as this provides much assurance of such compe- tence either for HCECs or their employers. We pro- pose that a more robust or two-tiered evaluation process is needed before such a credential could reli- ably attest to the competence and expertise of an HCEC.<br>Despite shortcomings in the Certification Process as it is currently designed, in general there are benefits of having some kind of certification for individual consultants, as well as HCEC programs and the broader HCEC field. For consultants, achieving and maintaining certification requires continued review of core materials to refresh ethics knowledge and pro- vides external validation of the consultant's ability to engage in consultation, boosting confidence that the individual is successfully engaged in continuing educa- tion. For HCEC programs, having credentialed con- sultants supports the program's mission to engage in quality consultation, taking a step toward ensuring individual consultants are performing their job in a manner

For peer review only

No.	Title	Author/year	MERSQI	COREQ	Reason for inclusion			Undergraduate (UG)/Postgraduate (PG)	Methodology	Purpose of study	Key findings
					Communication	Ethics	Professionalism				
											consistent with established standards in the field. Finally, for the HCEC community as a whole, having a singular credential helps to standardize the practice and validate ethics consultation as a defined and important healthcare specialty. This effort to legit- imize the field as a profession will support the inclu- sion of clinical ethics across healthcare, hopefully expanding the practice from a few select academic or larger healthcare systems to all health care settings. Reaching a point where a health care ethics credential signifies not only competence but also excellence in the ability to conduct a clinical ethics consultation, however, may still be a long way off.
35	Assessing Professionalism in Medicine – A Scoping Review of Assessment Tools from 1990 to 2018	Tay, K. T.<br>Ng, S.<br>Hee, J. M.<br>Chia, E. W. Y.<br>Vythilingam, D.<br>Ong, Y. T.<br>Chiam, M.<br>Chin, A. M. C.<br>Fong, W.<br>Wijaya, L.<br>Toh, Y. P.<br>Mason, S.<br>Krishna, L. K. R.<br><br>2020			✓		✓	UG and PG	Arksey and O'Malley's (2005) approach to scoping reviews was used to identify appropriate publications featured in four data- bases published between 1 January 1990 and 31 December 2018. Seven members of the research team employed thematic analysis to evaluate the selected articles.	Medical professionalism enhances doctor-patient relationships and advances patient-centric care. However, despite its pivotal role, the concept of medical professionalism remains diversely understood, taught and thus poorly assessed with Singapore lacking a linguistically sensitive, context specific and culturally appropriate assessment tool. A scoping review of assessments of professionalism in medicine was thus carried out to better guide its understanding	Prevailing assessments of professionalism in medicine must contend with differences in setting, context and levels of pro- fessional development as these explicate variances found in existing assessment criteria and approaches. However, acknowledging the sig- nificance of context-specific competency-based stages in medical professionalism will allow the forwarding of guiding principles to aid the design of a culturally-sensitive and practical approach to assessing professionalism.

For peer review only

No.	Title	Author/year	MERSQI	COREQ	Reason for inclusion			Undergraduate (UG)/Postgraduate (PG)	Methodology	Purpose of study	Key findings
					Communication	Ethics	Professionalism				
36	Content and outcomes of narrative medicine programmes: a systematic review of the literature through 2019	Remein, Christy DiFrances<br>Childs, Ellen<br>Pasco, John Carlo<br>Trinquart, Ludovic<br>Flynn, David B.<br>Wingerter, Sarah L.<br>Bhasin, Robina M.<br>Demers, Lindsay B.<br>Benjamin, Emelia J.<br><br>2020	-	-	✓			PG	We conducted a systematic review of literature published through 2019 using five major databases: PubMed, Embase, PsycINFO, ERIC and MedEdPORTAL. Eligible NM programming included textual analysis/close reading of published literature and creative/reflective writing. Qualifying participants comprised individuals from academic medicine and health sciences disciplines. We reviewed and categorised programme goals, content and evaluation activities to assess participant satisfaction and programme efficacy. Two members of the research team assessed the risk of bias, independently screening records via a two-round, iterative process to reach consensus on eligibility.	Narrative medicine (NM) incorporates stories into health sciences paradigms as fundamental aspects of the human experience. The aim of this systematic review is to answer the research question: how effective is the implementation and evaluation of NM programmes in academic medicine and health sciences? We documented objectives, content and evaluation outcomes of NM programming to provide recommendations for future narrative-based education.	Of 1569 original citations identified, we selected 55 unique programmes (described in 61 records). In all,<br>41 (75%) programmes reported a form of evaluation; evaluation methods lacked consistency. Twenty-two programmes used quantitative evaluation (13 well described), and 33 programmes used qualitative evaluation (27 well described). Well-described quantitative evaluations relied on 32 different measures (7 validated) and showed evidence of high participant satisfaction and pre-post improvement in competencies such as relationship- building, empathy, confidence/personal accomplishment, pedagogical skills and clinical skills. An average of 88.3% of participants agreed or strongly agreed that the programme had positive outcomes. Qualitative evaluation identified high participant satisfaction and improvement<br>in competencies such as relationship-building, empathy, perspective-taking/reflection, resilience and burnout detection/mitigation, confidence/personal accomplishment, narrative competence, and ethical inquiry.<br><br>Evaluation suggests that NM programming leads to high participant satisfaction and positive outcomes

No.	Title	Author/year	MERSQI	COREQ	Reason for inclusion			Undergraduate (UG)/Postgraduate (PG)	Methodology	Purpose of study	Key findings
					Communication	Ethics	Professionalism				
											across various competencies. We suggest best practices and innovative future directions for programme implementation and evaluation.
37	Use of portfolios in early undergraduate medical training	Driessen, Erik<br>Van Tartwijk, Jan<br>Vermunt, Jan<br>van der Vleuten, Cees<br><br>2003		10		✓		UG	During the academic year 2001–02 242 first-year medical students compiled a portfolio. Semi-structured interviews were held with a select group of students to explore the effect of the portfolio on reflection. Students from four random mentor groups, 39 in total, were approached and asked if they would be interviewed on the portfolio. With the exception of one student, all students were prepared to take part in the interview. The interview sched-ule focused on the process of compiling a portfolio and its possible effect on reflective ability. In addition to this, students were given the opportunity to offer suggestions for improvement.	This article describes the use of portfolios in **early** undergraduate medical training... In this article, we will describe the Maastricht portfolio as an illustration of an early undergraduate portfolio. We will discuss the reasons for the choices we made in designing the portfolio and report on a first evaluation, focusing on the effects on students' reflective ability.	The majority of students were of the opinion that analysing one's competences in a portfolio was instructive and meaningful. With regard to learning how to reflect and recognize learning needs, however, mentor coaching proved to be necessary. The results thus far show that the portfolio is a worthwhile addition to existing assessment and learning tools. Our preliminary findings show that the introduction of such a portfolio in the early stages of medical training seems to be an effective way for students to learn how to reflect on their learning and behaviour.
38	Conditions for reflective use of portfolios in	Driessen, E. W.<br>van Tartwijk, J.<br>Overeem, K.		18			✓	UG	We designed a portfolio that was aimed at	Portfolios are often used as an instrument with	The conditions for successful reflective use of portfolios that emerged from

For peer review only

No.	Title	Author/year	MERSQI	COREQ	Reason for inclusion			Undergraduate (UG)/Postgraduate (PG)	Methodology	Purpose of study	Key findings
					Communication	Ethics	Professionalism				
	undergraduate medical education	Vermunt, J. D. van der Vleuten, C. P. 2005							stimulating reflection in early undergraduate medical education, using experiences described in the medical educa- tion literature and elsewhere. Conditions for reflect- ive portfolio use were identified through interviews with 13 teachers (mentors), who were experienced in mentoring students in the process of developing their portfolios. The interviews were analysed according to the principles of grounded theory.	which to stimulate students to reflect on their experiences. Research has shown that working with portfolios does not automatically stimulate reflection. In this study we addressed the question: What are the conditions for successful reflective use of portfolios in undergraduate medical education?	the interviews fell into 4 categories: coaching; portfolio structure and guidelines; relevant experiences and materials, and summative assessment. According to the mentors, working with a portfolio designed to meet these conditions will stimulate students' reflective abilities...This study shows that portfolios are a potentially valuable method of assessing and develop- ing students' reflective skills in undergraduate med- ical training, provided certain conditions for effective portfolios are recognised and met. Portfolios have a strong potential for enhancing learning and assess- ment but they are very vulnerable and may easily lead to disappointment. Before implementing portfolios in education, one should first consider whether the necessary conditions can be fulfilled, including an appropriate portfolio structure, an appropriate assessment procedure, the provision of enough new experiences and materials, and sufficient teacher capacity for adequate coaching and assessment.
39	Portfolio Assessment Implementation in Clinical Year of Community Medicine Module: Students Perspective	Ekayanti, Fika Risahmawati, Risahmawati Fadhilah, Marita 2017	7				✓	UG	Nine portfolios were assigned to students within 5 weeks length module. The data was collected by questionnaires from 46 students after completing module in November 2016. Students were	This study aimed to identify the correlation of students' perspective for using portfolio and their portfolio scores.	Most students (36/78.3%) felt that the portfolio was not appropriate as their summative assessment. They preferred less portfolios, 30 (63%) students requested to decrease portfolios to 3-5, 28.3% to 6-8 and only 8.7% to <3. There was no significant correlation between students' satisfaction of using portfolio

No.	Title	Author/year	MERSQI	COREQ	Reason for inclusion			Undergraduate (UG)/Postgraduate (PG)	Methodology	Purpose of study	Key findings
					Communication	Ethics	Professionalism				
									asked about their satisfaction of using portfolio, then correlated to their portfolio scores. The reasons and suggestion for ideal portfolios were identified. Analysis was done by SPSS 20 using Rank Spearman correlation test. Students felt that creating portfolios were not comfortable.		to their portfolios score (p=0.262), while there was significant correlation between students' score to the number of portfolios to be submitted (p=0.017; r=0.349). Reasons for decreasing number of portfolios were inadequate time and many other tasks to be finished. Students in UIN Jakarta haven't used to retelling their experiences and reflecting them in writing as perceived in many students who were not used to portfolios. Doing portfolios need lots of work and time-consuming. Having lesser number of portfolios to be submitted would encourage students to create portfolios. Building familiarization, time management and good motivation for creating portfolios were important to successful portfolios. There was no significant correlation between students' satisfaction to their scores. Students should be encouraged to get comfortable in creating portfolios for the benefit as lifelong learning tool. Motivation to write and reflect should be nurtured to improve students' portfolio satisfaction and commitment.
40	Students' perception of portfolio as a learning tool at King Abdulaziz University Medical School	Fida, N. M.<br>Hassanien, M.<br>Shamim, M. S.<br>Alafari, R.<br>Zaini, R.<br>Mufti, S.<br>Al-Hayani, A.<br>Farouq, M.<br>Al-Zahrani, H.<br><br>2018	8	14	✓	✓	✓	UG	Portfolios were introduced in the 2nd through 5th years at King Abdulaziz University over a two-year period (2013–2015). At the end of each academic year, students completed a	Medical education has a longstanding tradition of using logbooks to record activities. The portfolio is an alter-native tool to document competence and promote reflective practice. This study assessed the	The results showed a difference in focus between basic and clinical years: in basic years students' focus was on acquiring practical skills, but in clinical years they focused more on acquiring complex skills, including identifying and man- aging problems. The questionnaire responses nonetheless revealed a

For peer review only - http://bmjopen.bmj.com/site/about/guidelines.xhtml

No.	Title	Author/year	MERSQI	COREQ	Reason for inclusion			Undergraduate (UG)/Postgraduate (PG)	Methodology	Purpose of study	Key findings
					Communication	Ethics	Professionalism				
									mixed questionnaire that included a self-assessment of skills learned through the use of portfolio.	acceptance of portfolio use among Saudi undergraduate medical students.	positive trend in acceptance (belief in the educational value) of portfolios among students and their mentors, across the years of the program. Using portfolios as a developmental learning and formative assessment tool in the early undergraduate years was found to contribute to students' ability to create their own clinical skills guidelines in later years, as well as to engage in and appreciate reflective learning.
41	The use of portfolios to foster professionalism: Attributes, outcomes, and recommendations	Renato Soleiman Franco, Camila Ament Giuliani dos Santos Franco,Olívia Pestana,Milton Severo &Maria Amélia Ferreira<br><br>2017		9		✓	✓	UG	A systematic review on the use of portfolios in teaching professionalism to medical students identified 1257 papers. Of these, 11 articles met all inclusion criteria	The main objective of this research was to review the characteristics of portfolios and their outcomes for teaching professionalism to undergraduate medical students	According to the papers, the use of portfolios for teaching professionalism shows versatility, supports learning strategies and has the potential to be used in different contexts, including for formative and summative purposes. The weaknesses were based on the artificiality of the reflections, deficient instructions, time-consuming processes and preference among students for other teaching methods. Students complained about feeling that the reflection was 'forced', and they tended to write based on social conventions rather than reveal their true thoughts. Reflection is a powerful component of the portfolio, but the method by which it is taught could easily ruin its potential to boost professionalism. Requiring reflection did not ensure its achievement; increased understanding by students regarding how and why they were doing it, the clarity of assessment methods and constructive feedback might strengthen the potential for success. A

No.	Title	Author/year	MERSQI	COREQ	Reason for inclusion			Undergraduate (UG)/Postgraduate (PG)	Methodology	Purpose of study	Key findings
					Communication	Ethics	Professionalism				
											framework was designed to support faculty members in developing and applying portfolios with a clear and broad view of this teaching strategy.
42	AMEE Medical Education Guide No. 24: Portfolios as a method of student assessment	Friedman Ben David, M.<br>Davis, M. H.<br>Harden, R. M.<br>Howie, P. W.<br>Ker, J.<br>Pippard, M. J.<br><br>2001	-	-	✓	✓	✓	UG	-	This guide is intended to inform medical teachers about the use of portfolios for student assessment. It provides a background to the topic, reviews the range of assessment purposes for which portfolios have been used, identifies possible portfolio contents and outlines the advantages of portfolio assess-ment with particular focus on assessing professionalism...The current state of understanding of the technical, psychometric issues relating to portfolio assessment is clarified. The final part of the paper provides a practical guide for those wishing to design and implement portfolio assessment in their own institutions.	-
43	Teaching professionalism in the early years of a medical curriculum: a qualitative study	Goldie, J.<br>Dowie, A.<br>Cotton, P.<br>Morrison, J.<br><br>2007		18	✓	✓		UG	A qualitative approach was adopted involving semistructured interviews, on a 1 in 6 sample of tutors involved in teaching in the early curricular years, and 3 student focus groups. The	This study investigated tutors' and students' perspectives of the delivery of profession- alism in the early years of Glasgow's learner-centred, problem-based learning (PBL), integrated medical curriculum.	Involvement in teaching raised students' and tutors' awareness of their professionalism. Learning activities promoting critical reflection were most effective. The integration of professionalism across the domains of Vocational Studies (135) was important for learning; however, it was not well integrated with the

For peer review only

For peer review only - http://bmjopen.bmj.com/site/about/guidelines.xhtml

No.	Title	Author/year	MERSQI	COREQ	Reason for inclusion			Undergraduate (UG)/Postgraduate (PG)	Methodology	Purpose of study	Key findings
					Communication	Ethics	Professionalism				
									findings were subjected to between-method triangulation.		PBL core. Integration was pro- moted by having the same tutor present throughout all VS sessions. Early patient contact experiences were found to be particularly important. The hidden curriculum provided both opportunities for, and threats to, learning. The small-group format provided a suitable environment for the examination of pre-existing perspectives. The portfolio was an effective learning tool, although its assessment should be formalised.<br><br>Reflection is integral to professional development. Early clinical contact is an important part of the process of socialisation, as it allows students to enter the community of practice that is the medical profession. Role models can contribute powerfully to students' learning and identity formation. As students move towards fuller participation, the clinical milieu should be controlled to maximise the influence of role models, and opportunities for guided reflection should be sustained.
44	Assessing students' personal and professional development using portfolios and interviews	Gordon, J.<br><br>2003	6.5			✓	✓	UG	The instruments used to assess Year 1 stu- dents in PPD are a portfolio and interview. This assessment format encourages students to explore ideas and values that are important to them	Medical schools are placing more emphasis on students' personal and professional development (PPD) and are seeking ways of assessing student progress towards meeting outcome goals in relation to professionalism. The	In 1997⁄98, 96% of students agreed that they had engaged in useful reflection on their approach to the course and 91% agreed that the experience was worthwhile. A further 76% of students agreed that they could see opportunities to modify their approach in some ways as result of this exercise.

For peer review only - http://bmjopen.bmj.com/site/about/guidelines.xhtml

No.	Title	Author/year	MERSQI	COREQ	Reason for inclusion			Undergraduate (UG)/Postgraduate (PG)	Methodology	Purpose of study	Key findings
					Communication	Ethics	Professionalism				
									and relevant to the PPD theme. A confidential interview, based on the PPD goals, is held with a faculty member who has read the student's portfolio.	Faculty of Medicine at the University of Sydney sought an assessment method that would demonstrate the value of reflection in attaining PPD, provide feedback and encourage stu- dents to take responsibility for setting and achieving high standards of performance.	
45	Developing a sustainable electronic portfolio (ePortfolio) program that fosters reflective practice and incorporates CanMEDS competencies into the undergraduate medical curriculum	Hall, P. Byszewski, A. Sutherland, S. Stodel, E. J.<br><br>2012	-	-	✓		✓	UG	-	In this article, the authors describe the development of an electronic Portfolio (ePortfolio) program that enables uOttawa medical students to document their activities and to demonstrate their development of competence in each of the eight roles	The authors reflect on the challenges they faced in the development and implementation of the ePortfolio program and share the lessons they have learned along the way to a successful and sustainable program. These lessons include switching from a complex information technology system to a user-friendly, Web- based blog platform; rethinking orientation sessions to ensure that faculty and students understand the value of the ePortfolio program; soliciting student input to improve the program and increase student buy-in; and providing faculty development opportunities and recognition.
46	The utility of reflective portfolios as a method of assessing first year medical students' personal and professional development	Rees, Charlotte E. Shepherd, Maggie Chamberlain, Suzanne<br><br>2005	8	17	✓			UG	The construct validity of the criteria was established by exploring its relationship with two other assessment methods and its reliability was determined using the	-	Themes emerging from the focus groups include students preferring the structured nature of the portfolios but assessors feel- ing that this reduced the uniqueness of the portfolios. Although students understood the importance of reflective practice, some disliked the process of reflection, particularly

No.	Title	Author/year	MERSQI	COREQ	Reason for inclusion			Undergraduate (UG)/Postgraduate (PG)	Methodology	Purpose of study	Key findings
					Communication	Ethics	Professionalism				
									generalizability (G) coefficient. Focus groups with assessors and students were convened to explore their views of the portfolios. Two portfolio analyses had a G coefficient of .42. Performance in the portfolio analyses was correlated with performance in personal and professional development judgements (r = −.512, p < .01) and scientific reports (r = .273, p = .002).		reflective writing. Educators should design assessment criteria that maximize reliability, validity and acceptability rather than simply focusing on reliability alone.<br><br>Despite these methodological limitations, this study provides support for the reli- ability and validity of the portfolio analyses assessment criteria at Peninsula Medical School.
47	Reflections: an inquiry into medical students' professional identity formation	Wong, A.<br>Trollope-Kumar, K.<br><br>2014		14	✓	✓		UG	Sixty-five medical students (46 women; 19 men) from a class of 194 consented to the study of their portfolios. In total, 604 reflections were analysed and coded using thematic narrative analysis. The codes were merged under subthemes and themes. Common or recurrent themes were identified in order to develop a descriptive framework of professional identity formation. Reflections were then analysed	The purpose of this study was to understand the major influences on medical students' professional identity formation.	Five major themes were associated with professional identity formation in medi- cal students: prior experiences, role models, patient encounters, curriculum (formal and hidden) and societal expectations. Our longi- tudinal analysis shows how these themes inter- act and shape pivotal moments, as well as the iterative nature of professional identity from the multiple ways in which individuals con- struct meaning from interactions with their environments.<br><br>Our study provides a window on the dynamic, discursive and constructed nature of professional identity formation. The five key themes associated with professional identity formation provide strategic

For peer review only

No.	Title	Author/year	MERSQI	COREQ	Reason for inclusion			Undergraduate (UG)/Postgraduate (PG)	Methodology	Purpose of study	Key findings
					Communication	Ethics	Professionalism				
									longitudinally within and across individual portfolios to examine the professional identity formation over time with respect to these themes.		opportuni- ties to enable positive development. This study also illustrates the power of reflective writing for students and tutors in the professional identity formation process.
48	Content validity of workplace-based portfolios: A multi-centre study	Nele R.M. Michels, Marijke Avonts, Griet Peeraer, Kris Ulenaers, Luc F. Van Gaal, Leo L. Bossaert, Erik W. Driessen, Arno M.M. Muijtjens & Benedicte Y. De Winter<br><br>2016	9.5		✓		✓	UG	We reviewed 120 workplace portfolios at three medical universities (Belgium and the Netherlands). To validate their content, we developed a Validity Inventory for Portfolio Assessment (VIPA) based on the CanMEDS roles. Two raters evaluated each portfolio and indicated for each VIPA item whether the portfolio provided sufficient information to enable satisfactory assessment of the item. We ran a descriptive analysis on the validation data and computed Cohen's Kappa to investigate interrater agreement.	This study sought to evaluate the content validity of portfolios as reflected in their capacity to adequately assess achieved competences of medical students during clerkships.	The portfolios adequately covered the items pertaining to the communicator (90%) and professional (87%) roles. Coverage of the medical expert, collaborator, scholar and manager roles ranged between 75% and 85%. The health advocate role, covering 59%, was clearly less well represented. This role also exhibited little interrater agreement (Kappa50.4).<br><br>This study lends further credence to the evidence that portfolios can indeed adequately assess the different CanMEDS roles during clerkships, the health advocate role, which was less well represented in the portfolio content, excepted.
49	Does a summative portfolio foster the development of capabilities such	Anthony J O'sullivan, Amanda C Howe, Susan Miles, Peter Harris, Chris S Hughes, Philip Jones, Helen	7.5		✓	✓	✓	UG	A questionnaire was designed to evaluate undergraduate medical students' experiences of	The aims of this study were (i) to determine whether preparing a portfolio helps promote students'	Students perceive portfolio preparation as an effective learning tool for the development of capabilities such as understanding ethical and legal principles

For peer review only

No.	Title	Author/year	MERSQI	COREQ	Reason for inclusion			Undergraduate (UG)/Postgraduate (PG)	Methodology	Purpose of study	Key findings
					Communication	Ethics	Professionalism				
	as reflective practice and understanding ethics? An evaluation from two medical schools	Scicluna & Sam J Leinster<br><br>2012							completing a portfolio at two medical schools. A total of 526 (45% response rate) students answered the on-line questionnaire. Students from both medical schools gave the highest ranking for the portfolio as a trigger for reflective practice. 63% of students agreed their portfolio helped them develop reflective practice skills (p < 0.001), whereas only 22% disagreed. 48% of students agreed portfolios helped them understand ethical and legal principles whereas 29% disagreed (p < 0.001). In contrast, only 34% of students thought the portfolio helped them to develop effective communication.	development in a range of capabilities including understanding ethical and legal principles, reflective practice and effective communication, and (ii) to determine to what extent the format of the portfolio affected the outcome by comparing the experiences of students at two different medical schools.<br><br>Therefore, the primary aim of this study was to determine whether the process of preparing a portfolio report or essay helped students appreciate and develop in a range of capabilities, such as understanding ethical and legal principles and reflective practice; capabilities which are not the focus of traditional assessments such as clinical or written examina- tions. The format of the portfolio assessment was different in each of the two medical schools in the study, and so a secondary aim was to determine to what extent the format of the assessment affected the outcome by comparing the student experiences with their portfolio	and reflective practice, whereas other capabilities such as effective communication require complementary techniques and other modes of assessment.

For peer review only

No.	Title	Author/year	MERSQI	COREQ	Reason for inclusion			Undergraduate (UG)/Postgraduate (PG)	Methodology	Purpose of study	Key findings
					Communication	Ethics	Professionalism				
										assessment in the two schools.	
50	Feasibility and Outcomes of Implementing a Portfolio Assessment System Alongside a Traditional Grading System	Celia Laird O'Brien, PhD, Sandra M. Sanguino, MD, MPH, John X. Thomas, PhD, and Marianne M. Green, MD<br><br>2016	7		✓		✓	UG	In 2009, the authors developed a portfolio system that served as a repository for all student assessments organized by competency domain. Five competencies were selected for a preclerkship summative portfolio review. Students submitted reflections on their performance. In 2014, four clinical faculty members participated in standard-setting activities and used expert judgment and holistic review to rate students' competency achievement as "progressing toward competence," "progressing toward competence with some concern," or "progressing toward competence pending remediation." Follow-up surveys measured students' and faculty members'	This study describes the development and implementation of a longitudinal competency-based electronic portfolio system alongside a graded curriculum at a relatively large U.S. medical school.	Faculty evaluated 156 portfolios and showed high levels of agreement in their ratings. The majority of students achieved the "progressing toward competence" benchmark in all competency areas. However, 31 students received at least one concerning rating, which was not reflected in their course grades. Students' perceptions of the system's ability to foster self-assessment were mixed.

For peer review only

No.	Title	Author/year	MERSQI	COREQ	Reason for inclusion			Undergraduate (UG)/Postgraduate (PG)	Methodology	Purpose of study	Key findings
					Communication	Ethics	Professionalism				
									perceptions of the process.		
51	Feelings related to first patient experiences in medical school A qualitative study on students' personal portfolios	Kaisu H. Pitkälä a,⁎, Taina Mäntyranta b<br><br>2003? 2004		14	✓		✓	UG	Twenty-two volunteer third and fourth year medical students (15 women and 7 men) of the University of Helsinki participated in a portfolio course for 1 year. Their reflective learning diaries and writings on specific themes were analyzed by qualitative content analysis	Feelings and thoughts of medical students related to first patient experiences during the first clinical year were examined	First patient encoun- ters were strong emotional experiences for medical students. The first patient examination was often described as an anxiety-provoking and confusing incident. Other emotionally significant encounters included helplessness when faced with serious illness and death, and role confusion when examining patients of one's own age but opposite sex. Students felt guilty for using patients for their own learn- ing purposes. Portfolios as learning tools may help in recognizing key experiences and support professional development of medical students.
52	Linking assessment to undergraduate student capabilities through portfolio examination	Anthony J. O'Sullivan , Peter Harris , Chris S. Hughes , Susan M. Toohey , Chinthaka Balasooriya , Gary Velan , Rakesh K. Kumar & H. Patrick McNeil<br><br>2012	8.5		✓	✓	✓	UG	Students are required to complete assessments linked to graduate capabilities. In Year 3, a portfolio review occurs (205–248 students per year), focusing on students' grades and feedback from assessments and a reflective essay is submitted. In the essay, students reflect on their progress, identify areas of weakness and detail plans for improvement. Progress in each capability is summatively graded against	This article describes an e-portfolio which closely aligns learning and reflection to graduate capabilities, incorporating features that address concerns about portfolios	Portfolios are an established method of assessment, although concerns do exist around their validity for capabilities such as reflection and self-direction

No.	Title	Author/year	MERSQI	COREQ	Reason for inclusion			Undergraduate (UG)/Postgraduate (PG)	Methodology	Purpose of study	Key findings
					Communication	Ethics	Professionalism				
									specific criteria and feedback is provided.		
53	Medical student resilience strategies: A content analysis of medical students' portfolios	Richard A. Prayson · S. Beth Bierer · Elaine F. Dannefer<br><br>2016		12	✓	✓	✓	UG	We retrospectively reviewed a sampling of year 1, 2 and 5 portfolio essays focused on the Personal Develop- ment competency and performance milestones, written by 49 students from three different classes in a 5-year pro- gramme devoted to training physician investigators. Two medical educators used a framework established by Jensen and colleagues (2008) to identify the nature and prevalence of various resilience strategies (valuing the physician role, self-awareness, personal arena, professional arena, profes- sional support and personal support) medical students re- ported in portfolio essays.	A student's ability to employ resilience strategies to self-regulate behaviour is critical to the student's future career as a physician. (The aim is to examine this??)	All students documented at least one strategy in their essays each year. In all years, the most commonly documented strategies were in the personal arena (95.7% of year 1, 98% of year 2 and 87.8% of year 5 portfolios). The least frequently documented strategy in all years was professional support (42.8% of year 1, 38.8% of year 2, and 28.6% of year 5 portfolios). Year 5 portfolios discussed personal support strategies (79.6%) more frequently than year 1 (53.1%) and year 2 (59.2%) portfolios.<br><br>The results suggest that medical students can identify stressors and articulate resilience strategies that can be employed to potentially address them.
54	Portfolio Assessment during Medical Internships: How to Obtain a Reliable and Feasible	NRM Michels, EW Driessen, AMM Muijtjens, LF Van Gaal, LL Bossaert, BY De Winter<br><br>2009		9.5			✓	UG	The domain-oriented reliability of 61 double-rated portfolios was measured, using a generalisability	In this study, we investigated whether assessment during medical internship by a portfolio can combine reliability and feasibility.	We obtained reliability (Φ coefficient) of 0.87 with this internship portfolio containing 15 double-rated tasks. The generalisability analysis showed that an acceptable level of reliability

No.	Title	Author/year	MERSQI	COREQ	Reason for inclusion			Undergraduate (UG)/Postgraduate (PG)	Methodology	Purpose of study	Key findings
					Communication	Ethics	Professionalism				
	Assessment Procedure?								analysis with portfolio tasks and raters as sources of variation in measuring the performance of a student.		(Φ = 0.80) was maintained when the amount of portfolio tasks was decreased to 13 or 9 using one and two raters, respectively.<br><br>Our study shows that a portfolio can be a reliable method for the assessment of workplace learning. The possibility of reducing the amount of tasks or raters while maintaining a sufficient level of reliability suggests an increase in feasibility of portfolio use for both students and raters.
55	Twelve tips for introducing E-Portfolios with undergraduate students	Alis Moores & Maria Parks<br><br>2010	-	-	✓			UG	-	-	-
56	Use of Portfolios in Undergraduate Medical Training: First Meeting With a Patient	Nilgün ÖZÇAKAR, Vildan MEVSİM, Dilek GÜLDAL<br><br>2009		10	✓		✓	UG	We designed a portfolio that was aimed at making reflection in early undergraduate medical education. Conditions for portfolio use were identified through interviews with six trainers who were experienced in mentoring students in the process of developing their portfolios. Analysis of writings was per- formed using qualitative content analysis.	Portfolios, as learning tools, are becoming increasingly important in medical education. Our aim was to evaluate the contents of portfolios used in medical education.	The conditions for successful reflective use of portfolios that emerged from the interviews fell into catego- ries: practice evaluation, patient perspective and primary prevention/health promotion. According to the students, working with a portfolio designed to meet these conditions is very useful and will stimulate students' abilities. The stu- dents shared the same opinion that analyzing one's com- petences in a portfolio was instructive and meaningful.<br><br>According to our students, portfolios are a potentially valuable method of developing students' skills in undergraduate medical education, provided that certain conditions for effective portfolios are recognized and met. The portfolio is a worthwhile addition to existing

For peer review only

No.	Title	Author/year	MERSQI	COREQ	Reason for inclusion			Undergraduate (UG)/Postgraduate (PG)	Methodology	Purpose of study	Key findings
					Communication	Ethics	Professionalism				
											assessment and learning tools.
57	What Is the Relationship Between a Preclerkship Portfolio Review and Later Performance in Clerkships?	Celia Laird O'Brien, PhD, John X. Thomas Jr, PhD, and Marianne M. Green, MD<br><br>2018	9.5		✓		✓	UG	The authors divided students into two groups based on a summative preclerkship portfolio review in 2014: students who had concerning behavior in one or more competencies and students progressing satisfactorily. They compared how students in these groups later performed on two clerkship outcomes as of October 2015: final grades in required clerkships, and performance on a clerkship clinical composite score. They used Mann–Whitney tests and multiple linear regression to examine the relationship between portfolio review results and clerkship outcomes. They used USMLE Step 1 to control for knowledge acquisition.	This study examines whether a preclerkship portfolio review is a valid method of identifying problematic student behavior affecting later performance in clerkships.	Students with concerning behavior preclerkship received significantly lower clerkship grades than students progressing satisfactorily ($P = .002$). They also scored significantly lower on the clinical composite score ($P < .001$). Regression analysis indicated concerning behavior was associated with lower clinical composite scores, even after controlling for knowledge acquisition.<br><br>The results show a preclerkship portfolio review can identify behaviors that impact clerkship performance. A comprehensive portfolio system is a valid way to measure behavioral competencies.
58	Educating undergraduate medical students about oncology: A literature review	Cave, Judith<br><br>2006	-	-	✓			UG	MEDLINE, Psychinfo, ERIC, TIMELIT, EMBASE, CINAHL and the Cochrane CENTRAL Register of	This article is a review of the literature regarding teaching oncology to undergraduate medical students.	The main findings can be summarized as follows: the involvement of patients in teaching is popular with students and portfolio learning is a successful way of involving patients; the use of standardized patients to

No.	Title	Author/year	MERSQI	COREQ	Reason for inclusion			Undergraduate (UG)/Postgraduate (PG)	Methodology	Purpose of study	Key findings
					Communication	Ethics	Professionalism				
									Controlled Trials (CENTRAL) were searched, using the search terms cancer, oncology, education, undergraduate, and teaching.		teach breast examination improves students' performance in clinical assessment; the use of silicone models to teach breast examination improves students' sensitivity for detecting breast lumps; computer aided learning modules have a role, but are not superior to other types of learning; learning about cancer screening and prevention increases students' knowledge, improves their self rated skills, and changes their behavior; and cancer patients have an important role to play in teaching undergraduate communication skills.<br><br>We have found 48 articles on undergraduate teaching in oncology. Oncology teachers should consider adopting the evidence based approaches outlined in this review, and there should be more emphasis on educational research within the field of oncology.
59	Integrating the assessment of interprofessional education into the health care curriculum	Anderson, E. S. and Kinnair, D.<br><br>2016	-	-	✓	✓	✓	UG	-	Reflection on some of the challenges associated with assessment in IPE by describing the experiences at one UK-based medical school	In regard to written reflections, reading 240 student entries per learning event is time-consuming, making<br>it challenging to offer individual feedback. Some students have challenged the formative outcomes that were graded for progression across broad boundaries of unsatisfactory, satisfactory or excellent. Checking for consistency across a small numbers of marker illuminates the subjective perspectives and interpretations of individual

For peer review only

No.	Title	Author/year	MERSQI	COREQ	Reason for inclusion			Undergraduate (UG)/Postgraduate (PG)	Methodology	Purpose of study	Key findings
					Communication	Ethics	Professionalism				
											markers on what constitutes a meaningful reflection. Nevertheless, offering this early writing platform has proved to be extremely helpful and enables educators to determine if students can reflect critically and apply meaning
60	A model of engagement in reflective writing-based portfolios: Interactions between points of vulnerability and acts of adaptability	Arntfield, S., Parlett, B., Meston, C. N., Apramian, T. and Lingard, L. 2015	7	21			✓	UG	Using mixed-methods rooted in grounded theory, 139 students and 13 mentors completed questionnaires, 23 students participated in four focus groups and 9 mentors in individual interviews	The aim of this study was to explore conditions affecting the experience of teaching and learning from the perspective of both students and mentors in a reflective writing-based portfolio initiative.	The overarching theme in our data was student–mentor engagement. Our results confirm previous literature describing portfolio as a vulnerable method of learning, extend this concept by identifying and categorizing specific points of vulnerability, and contribute new knowledge regarding acts of adaptability, which serve to strengthen the student–mentor relationship. Engagement is central to the success of portfolio and is shaped by a dynamic interaction between points of vulnerability and acts of adaptability. We propose a model of engagement in portfolio that can be used for faculty development to optimize student–mentor engagement.
61	Support for portfolio in the initial years of the undergraduate medical school curriculum: what do the tutors think?	Austin, C. and Braidman, I. 2008	7	18	✓		✓	UG	A mixed method approach was used with data collected from both questionnaires and a focus group meeting.	The aims of this study were to investigate the views of these tutor facilitators on the delivery and support of portfolios in this way.	Tutor facilitators were generally positive about their roles in the support of portfolio development in group sessions and identified several advantages to this type of tutoring – namely the value of group discussion and discussion between peers to encourage reflection, and the practical ability to integrate portfolio sessions more closely with clinical experiences – which would not be possible in one to one tutor/student meetings.

For peer review only

No.	Title	Author/year	MERSQI	COREQ	Reason for inclusion			Undergraduate (UG)/Postgraduate (PG)	Methodology	Purpose of study	Key findings
					Communication	Ethics	Professionalism				
											With the training and guidance notes provided tutors were confident that they understood the support they should give students and felt that they could encourage reflection.<br><br>Thus, tutors perceived that the support of portfolio in groups is an effective way of developing portfolios for large numbers of students.
62	Use of portfolios by medical students: significance of critical thinking	Azer, S. A.<br><br>2008	-	-	✓		✓	UG	-	The aims of this paper are to: (1) understand the uses of portfolios and the rationale for using reflection in the early years of a PBL curriculum; (2) discuss how to introduce portfolios and encourage students' critical thinking skills, not just reflection; and (53) provide students with tips that could enhance their skills in constructing good portfolios.	-
63	Time to Loosen the Apron Strings: Cohort-based Evaluation of a Learner-driven Remediation Model at One Medical School	Bierer, S. B., Dannefer, E. F. and Tetzlaff, J. E.<br><br>2015	10		✓			UG	We used a retrospective, cohort-based approach to examine our learner-driven remediation model.<br>Data sources: We consulted MSPRC records to identify all CCLCM students from six class cohorts (2009–2014) placed in remediation during medical school, and compared remedial students	To examine a remediation model where students, rather than faculty, develop remedial plans to improve performance.<br>We present an innovative, systematic approach to remediation purposefully designed to engage learners with the development and implementation of remedial plans to improve their performance. We first describe our approach to	From abstract -- participants included 177 students from six classes (2009–2014). Twenty-six were placed in remediation, with more referrals occurring during Years 1 or 2 ($n = 20$, 76 %). Unprofessional behavior represented the most common reason for referral in Years 3–5. Remedial students did not differ from classmates ($n = 151$) on baseline characteristics (Age, Gender, US citizenship, MCAT) or willingness to recommend their medical school to future students ($p < 0.05$). Two remedial students did not graduate and three did

For peer review only

No.	Title	Author/year	MERSQI	COREQ	Reason for inclusion			Undergraduate (UG)/Postgraduate (PG)	Methodology	Purpose of study	Key findings
					Communication	Ethics	Professionalism				
									to their classmates not placed in remediation on variables collected for program evaluation purposes: Baseline characteristics (gender, age, US citizenship, MCAT score), USMLE licensure exam performance (pass-rate for each student), National Residency Matching Program data (clinical specialty for each student), Satisfaction with medical school (collected from graduating students using questionnaire). Then, we read MSPRC correspondence to each remedial student to extract referral mechanism (portfolio-based decision or faculty-initiated), unsatisfactory competency domain(s), remediation duration (days), and completion of students' remediation goals. Finally, we read students' remedial plans to identify strategies they proposed to	remediation and then report outcomes for students who were and were not placed in remediation during medical school. We conclude with lessons learned and future directions.	not pass USLME licensure exams on first attempt. Most remedial students (92 %) generated appropriate plans to address performance deficits. Students can successfully design remedial interventions. This learner-driven remediation model promotes greater autonomy and reinforces self-regulated learning. From results: Participants include 177 of 187 medical students from six class cohorts (2009–2014) who consented (95 %) to release program evaluation data for research purposes. Of these, the MSPRC placed 26 students (17 men and nine women) in formal remediation. Half (13) were identified by the MSPRC for not meeting year-end performance standards assessed via portfolios. The dean of student affairs referred the remaining 13 students based on faculty recommendation. We did not detect significant differences between remedial students and their classmates for baseline characteristics (Table 1). Overall, three-quarters of remediation decisions occurred during the first (10, 38 %) or second (10, 38 %) year of medical school. With regard to specific domains in which students failed to meet year-end standards or were otherwise referred, professionalism (18, 69 %), communication (10, 38 %), research (9, 35 %), and reflective practice (5, 19 %) represent frequent domains of unsatisfactory performance. Fewer students did not meet

For peer review only

No.	Title	Author/year	MERSQI	COREQ	Reason for inclusion			Undergraduate (UG)/Postgraduate (PG)	Methodology	Purpose of study	Key findings
					Communication	Ethics	Professionalism				
									the MSPRC to address performance deficits in specific competency areas. We manually recorded a subset of these strategies to provide examples of students' approaches to remediation. All performance decisions, referral mechanisms, and outcomes were manually entered into SPSS. Chi-square and ANOVA tests were used to compare baseline characteristics and satisfaction scores of the two student groups (remediation vs. other), while the Mann Whitney U test was used to examine if remediation duration (days) differed by students' gender. Statistical significance was established at p < 0.05 for all comparisons.		performance standards for clinical reasoning (4, 15 %), medical knowledge (3, 11 %), clinical skills (2, 8 %), personal development (2, 8 %), or health care systems (2, 8 %). Unprofessional behavior (e.g., not logging patient encounters, unexcused absences, chronic tardiness, etc.) represented the most common reason for student remediation during Years 3–5 of medical school. Ten students had deficient performance in one competency, while others had unsatisfactory performance in two (7, 27 %) or more (9, 35 %) competencies. Three students were placed in remediation twice, with one repeating for unprofessional behavior. Time in remediation (determined by dates in MSPRC correspondence to students) ranged from 39 to 1696 days (Median = 448 days) and did not differ by student gender (p > 0.05). Remediation plans revealed various student-proposed strategies to address TAFIs for multiple competency domains (Table 2). We observed that students' remedial strategies resembled approaches faculty would recommend. We also observed the MSRPC's correspondence to students consistently had an encouraging, supportive tone for students' remedial efforts (See Appendices for MSPRC's correspondence to one remedial student where committee asks student to develop a remediation plan, comments

No.	Title	Author/year	MERSQI	COREQ	Reason for inclusion			Undergraduate (UG)/Postgraduate (PG)	Methodology	Purpose of study	Key findings
					Communication	Ethics	Professionalism				
											on student's progress report, and releases student from remediation). The MSPRC "prescribed" remediation plans for two students only after these students did not provide adequate assessment evidence in progress reports. The MSPRC determined that most students (24, 92 %) generated sufficient evidence (predominately obtained from faculty and peers) to document their competence. Table 1 reveals that some remedial students experienced negative outcomes, as two did not graduate and three did not pass USMLE licensure exams on first attempt. Proportionally, more remedial students (11, 46 %) pursued surgical specialties than their classmates (50, 33 %). Table 1 also shows graduating remedial students were just as likely as their classmates to recommend the CCLCM program to future students, even though fewer were as satisfied with their overall experience at CCLCM as their classmates. From discussion -- Most programs struggle with how to identify and assist learners requiring remediation. We explored if students, rather than faculty, can take ownership for remedial plan design and implementation. We review key findings and conclude with future directions. The locus of control for selecting remedial interventions and assessment evidence typically rests with faculty. In

For peer review only

No.	Title	Author/year	MERSQI	COREQ	Reason for inclusion			Undergraduate (UG)/Postgraduate (PG)	Methodology	Purpose of study	Key findings
					Communication	Ethics	Professionalism				
											our model, the MSPRC charged students to develop remediation plans, with PA guidance as needed, and obtain assessment evidence to document progress and achievement. Most remediating students (92 %) successfully met these expectations and graduated from medical school. Three-quarters of remedial students were identified in Years 1–2 of medical school. Early identification provides opportunities for multiple, tailored interventions to occur. Professionalism, communication skills, and research performance were frequent reasons the MSPRC placed students in remediation. Few students were identified for gaps in medical knowledge or clinical reasoning. The literature suggests some competencies, like professionalism, require direct observation and ongoing assessment for learner success.1,18 Our students' remedial strategies (Table 2) ranged from specific to holistic approaches and often cut across courses and contexts, thereby providing them with more opportunities to improve and document their performance in complex domains. This emphasis on competence may explain why students' time in remediation was longer (Median = 448 days) than normally needed for "more of the same," such as retaking a test or course.2 Some learners may not know how to navigate performance obstacles,

For peer review only

No.	Title	Author/year	MERSQI	COREQ	Reason for inclusion			Undergraduate (UG)/Postgraduate (PG)	Methodology	Purpose of study	Key findings
					Communication	Ethics	Professionalism				
											making it important to offer guidance and oversight.19,20 For this reason, we train a core group of faculty to help students apply the self-regulation cycle.17 PAs coach students in reflection, self-assessment, and goal-setting.2,20,21 They also form long-term relationships with students and frequently serve as student advocates. The MSPRC encourages students to identify measurable outcomes to document competence, while offering positive support in written correspondence to students (See Appendices for series of letters to one student throughout the remediation process). Both faculty groups work in tandem to support this learner-driven remediation model while ensuring student accountability. Our evaluation is limited to one program, at one school, with a small class size. Other programs may foster remedial learners' autonomy more than conveyed in the literature.5,6,8 Our student satisfaction measures originate from two questionnaire items obtained at graduation and do not capture students' satisfaction with specific academic referral policies or remedial processes.22 Remediation is complex and involves multiple stakeholders and contexts. Future studies should capture learners' perceptions of remediation to complement the faculty point-of-view currently reported in the literature.

For peer review only

No.	Title	Author/year	MERSQI	COREQ	Reason for inclusion			Undergraduate (UG)/Postgraduate (PG)	Methodology	Purpose of study	Key findings
					Communication	Ethics	Professionalism				
											We discovered that more men than women were identified for remediation, yet we found no research exploring this observation in depth. Additionally, few longitudinal or multi-institutional studies currently exist, suggesting an area ripe for research. We discovered, in our 10-year experience with this remediation model, that medical students can select appropriate remedial interventions with the support of well-trained faculty. Our learner-driven approach to remediation may transfer to graduate medical education (GME), where Clinical Competence Committees (CCCs) meet regularly to discuss and review resident performance. Having struggling residents create remediation plans and submit progress reports to CCCs may present few barriers, given GME's competency-based assessment framework and emphasis on providing residents with frequent, formative feedback. Our approach requires a learning environment where faculty must trust students have the motivation and capabilities to identify and implement appropriate remedial strategies. We have created a systematic process where students must reflect on their performance, develop plans tailored to meet learning needs, and collect assessment evidence to document competence. To conclude, we believe educators who wish to

For peer review only

No.	Title	Author/year	MERSQI	COREQ	Reason for inclusion			Undergraduate (UG)/Postgraduate (PG)	Methodology	Purpose of study	Key findings
					Communication	Ethics	Professionalism				
64											adopt a learner-driven approach to remediation should view remediation as a growth opportunity rather than as a punishment.
64	Medical professionalism: conflicting values for tomorrow's doctors	Borgstrom, E., Cohn, S. and Barclay, S. 2010		14		✓		UG	From abstract: Qualitative study using interpretative discourse analysis of anonymized student reflective portfolios. One hundred twenty-three final year undergraduate medical students (64 male and 59 female) from the University of Cambridge School of Clinical Medicine supplied 116 portfolios from general practice and 118 from hospital settings about patients receiving palliative or end of life care. From introduction: This study investigates how professionalism is understood and experienced via one such initiative for medical students training in Cambridge, England, and highlights the conflicts between 'new' and 'old' values. From methods: Final year medical students in Cambridge meet patients approaching the	To investigate how final year medical students experience and interpret new values of professionalism as they emerge in relation to confronting dying patients and as they potentially conflict with older values that emerge through hidden dimensions of the curriculum.	From abstract -- Results: Professional values were prevalent in all the portfolios. Students emphasised patient-centered, holistic care, synonymous with a more contemporary idea of professionalism, in conjunction with values associated with the 'old' model of professionalism that had not be directly taught to them. Integrating 'new' professional values was at times problematic. Three main areas of potential conflict were identified: ethical considerations, doctor-patient interaction and subjective boundaries. Students explicitly and implicitly discussed several tensions and described strategies to resolve them. Conclusions: The conflicts outlined arise from the mix of values associated with different models of professionalism. Analysis indicates that 'new' models are not simply replacing existing elements. Whilst this analysis is of accounts from students within one UK medical school, the experience of conflict between different notions of professionalism and the three broad domains in which this conflict arises are relevant in other areas of medicine and in different national contexts. From discussion -- All of the students encountered challenges that required

For peer review only

No.	Title	Author/year	MERSQI	COREQ	Reason for inclusion			Undergraduate (UG)/Postgraduate (PG)	Methodology	Purpose of study	Key findings
					Communication	Ethics	Professionalism				
									end of life during general practice (GP) and hospital attachments. As required coursework they write portfolio items on two patients, one from each attachment. Key to this exercise is integrating issues arising from the interview with reflections of their own personal experiences. This study focuses on how different ideas are received and reproduced by a relatively young (typically early 20s) cohort of soon-to-be qualified doctors during the academic year 2007–2008. The University of Cambridge Psychology Research Ethics committee approved the study, and 86% of students (n = 123; 64 male, 59 female) gave informed consent for 234 reflective items: 116 from the GP and 118 from hospital settings. Each student was assigned a random identification number (ID), which is included		them to try and balance values characteristics of 'old' and 'new' forms of medical professionalism. Ethics, interaction and managing subjective boundaries all generated areas of conflict that they wrestled with in their assignments. Students addressed these tensions in a variety of ways, with strategies drawn from both formally taught skills and personal resources. In parallel with shifts from 'old' to 'new' values of professionalism, conflicts between the formal and the hidden curricula may also generate tensions44. In our study, the formal curricula is explicitly designed to embrace the 'new', leaving the 'old' to be communicated through more informal and hidden aspects of teaching. Yet for the students trying to personally reflect on their experiences and summatively draw from their entire educational experience for the exercise, no distinction is made between these two domains. Large organisations like the University of Cambridge Clinical School and the National Health Service have institutional memories45—collective experiences and concepts. These are communicated imperceptively through teaching elements as well as the very structures and policies that shape the educational environment. This feature serves to complicate understandings of medical education; it

No.	Title	Author/year	MERSQI	COREQ	Reason for inclusion			Undergraduate (UG)/Postgraduate (PG)	Methodology	Purpose of study	Key findings
					Communication	Ethics	Professionalism				
									with the quotes to demonstrate the breadth of the dataset presented. Analysis focused on the topic of ‘professionalism’, viewing the content of the portfolios as representative of general values imparted to students throughout their education. All items were coded and analysed in NVivo 8 using an interpretative approach. Following the social science traditions of two of the authors (EB and SC), the methodology employed a hermeneutic approach, striving to find meaning beyond straightforward discrete references35. Accordingly, rather than assuming the meaning of isolated sections of text in a reductive manner, they were always analysed in context and in relation to one another. Given the difficulty many students had in addressing the topic of end of life care, and		emphasises the extent to which values are embedded in the routines and practices of the organisation, and cannot therefore be simply addressed through redesigning curricula or individuals championing change. Our study suggests that tensions arise primarily because of different values that underlie the concept of professionalism throughout their education, rather than anything that is specific to end of life care. Whilst topics central to the everyday practice of medicine are particularly evident in palliative care46, they are made highly visible by students who see themselves on the cusp of becoming members of the profession themselves. Whilst it is likely that the concept of professionalism always has contained a wide variety of underlying values and principles that are not always commensurate with each other, we have argued that a more widespread shift in values over recent years has generated greater variance and hence more contradictory positions around what it means to be a good doctor. Our findings add to the literature on medical professionalism, which is rich in doctors’ anecdotal experiences47,48, highlights the stresses and conflicts doctors face44,49,50 and illustrates a current amalgamation of definitions with conflicting values51. Specific national and local contexts are likely to generate differently

For peer review only

No.	Title	Author/year	MERSQI	COREQ	Reason for inclusion			Undergraduate (UG)/Postgraduate (PG)	Methodology	Purpose of study	Key findings
					Communication	Ethics	Professionalism				
									consequently the very discursive descriptions of their observations, the authors were committed to code more than just those phrases in which a theme was referred to explicitly, and so included concerns that were expressed less directly. A coding scheme was derived from initial independent item analysis (EB, SC and SB) and adjusted through discussion. It was initially applicable to explicit statements by drawing on key words and then was augmented to include the more implicit references that potentially alluded more to the hidden dimensions of their teaching (see Box 2). The inclusion of these required careful interpretative readings of the entire text but provided an essential contribution to our overall analysis. The first author (193) then led on-going analysis, with regular meetings to		nuanced versions of these issues, which might only be identified through comparative work. From conclusion: The integration of 'new' professional values taught in medical school is at times problematic for students in any health care system that maintains elements, whether overtly or not, of the 'old' paradigm. The areas of potential conflicts outlined—ethics, patient interaction and managing subjective boundaries—are not limited to medical school or end of life care. Our analysis suggests that 'old' variants of professionalism are not simply being replaced by 'new' ones delivered by a redesigned curriculum, but rather that values from each can emerge in a range of medical contexts. As a result, professionalism does not consist of a set of fixed or abstract concepts, but rather surfaces through medical practice. If individual reflection is now heralded as an essential component of the 'new' professionalism, as indicated by the compulsory student exercise we have drawn on here, it should be acknowledged that it demands a dynamic engagement with the wide range of often contradictory and shifting ideas and beliefs from both formal and more hidden aspects of their education. This study illustrates that overt commitment to more empathic and patient-centered approaches to medical care do not

For peer review only

No.	Title	Author/year	MERSQI	COREQ	Reason for inclusion			Undergraduate (UG)/Postgraduate (PG)	Methodology	Purpose of study	Key findings
					Communication	Ethics	Professionalism				
									discuss emerging themes.		necessarily replace other more prescribed values and behaviours that remain part of a hidden curriculum embedded in institutional practices. Integration of 'new' core values and skills into good medical practice is not a smooth or simple transition. Additionally, it seems any simple attempt to communicate them through formal teaching is unlikely to prepare students for the reality of medical encounters. Instead, the experience of tension and the individual desire to seek balance and resolution across a wide range of issues may themselves be key and lasting features of medical professionalism.s
65	Facilitating students' reflective practice in a medical course: literature review	Chaffey, L. J., de Leeuw, E. J. and Finnigan, G. A.<br><br>2012		12			✓	UG	The literature search was undertaken using the online databases of CINAHL and Medline. Keyword searching was conducted using MeSH headings, Boolean operators, and the terms, 'medical students', 'medical education', 'reflection', 'reflect*' and 'medicine'. The search was limited to peer-reviewed published material in English and between the years 2001 and 2011 to maintain currency of	The aim of this review is to summarise the literature published around facilitating reflection in a medical course, and to answer the question : What is the current evidence regarding learning and development moments across the medical curriculum in developing students' reflective practice?	From abstract -- Discussion: A variety of reflective purposes was found in this literature review. Evidence indicates that, if students are unclear as to the purpose of reflection and do not see educators modelling reflective behaviours, they are likely to undervalue this important skill regardless of the associated learning and development opportunities embedded in the curriculum. From results: Thirty-six relevant articles were found. These articles were classified by methodology (see [Table 1]. Relevant references identified). As can be seen in [Table 1], this review included many opinion pieces, reviews and descriptions of methods of facilitating reflection, but little in regards to primary data in the area. Qualitative methodology was the main research output, providing largely descriptive data of

For peer review only

No.	Title	Author/year	MERSQI	COREQ	Reason for inclusion			Undergraduate (UG)/Postgraduate (PG)	Methodology	Purpose of study	Key findings
					Communication	Ethics	Professionalism				
									practice. The primary search yielded 129 references of eligible papers, including research papers, reviews, formal reviews and opinion pieces. Abstracts were read by the first author, and only articles pertaining to facilitating or teaching reflection in undergraduate or postgraduate medical courses were included. The first author read and classed all included articles, categorising them into construct sub-categories. At several stages in this process all authors reviewed the validity and legitimacy of the classifications. This led to the sub-headings and analysis presented below.		the facilitation of reflection. The quantitative research was also largely descriptive, with survey methodology being the most popular in the articles found. This review found only two case-control studies comparing facilitation methods of reflection. The literature presented enhancing factors and barriers to effectively teaching reflective practice within medical curricula, related to: The breadth of the meaning of reflection; Facilitating reflection by medical educators; Using written or web-based portfolios to facilitate reflection; and Assessing the reflective work of students.<br>- The breadth of the meaning of reflection: The term 'reflection' is widely mentioned within medical education literature, but the purposes and goals described indicate a breadth of purposes of reflection. [14] Mann et al.[10] concluded from their systematic review of reflective practice in the health professions that the nature of reflection makes it difficult to quantify. From this current review, we determined that the breadth of reflection covers the purposes of reflecting to improve judgements, to personally develop and, and to contextualise practice, and are explained in [Table 2]. Reflection to improve judgement centres on improving students' clinical skills, problem solving, [11] flexible thinking, lifelong learning and the development of expertise.

For peer review only

No.	Title	Author/year	MERSQI	COREQ	Reason for inclusion			Undergraduate (UG)/Postgraduate (PG)	Methodology	Purpose of study	Key findings
					Communication	Ethics	Professionalism				
											[1],[10],[14],[17] Reflection for this purpose usually has a problem focus, involves critical thinking, and is commonly indicated by hypothesis testing. Reflection to personally develop is essential in client-centred practice, as doctors need to consider they own values and assumptions, and those of the patient, while making clinical decisions. [18] From reviewing the literature, Sandars [14] asserted that the knowledge of one's values and beliefs is important for a doctor to develop therapeutic relationships with patients, as understanding oneself enables empathy and caring. Aukes et al.[11] reviewed literature in preparation for creating an assessment tool for reflection, and noted that personal reflection has often been overlooked in medical education. They suggested that this has occurred as personal development and has been seen as outside the domain of the problem-solving culture of medical practice, and thus, outside the domain of medical education. Reflection to contextualise practice helps students integrate theory with their own experience. Several authors have hypothesised that this aspect of reflection is essential in developing a sound medical practice, [5] with a lack of reflective ability resulting in poor self-awareness, and thus poor medical practice. [19] Howe et al.[8] found, from their study of the development of

For peer review only

No.	Title	Author/year	MERSQI	COREQ	Reason for inclusion			Undergraduate (UG)/Postgraduate (PG)	Methodology	Purpose of study	Key findings
					Communication	Ethics	Professionalism				
											professionalism, that reflecting on practice and developing self-awareness are important mechanisms of resilience in the face of a demanding profession. Although this breadth of reflective practice makes it an essential skill in medical practice, this very strength can also be a barrier in teaching facilitating reflective practice as there is often ambiguity of the educator's goals in regard to the use of a particular reflective task. [20],[21] Aukes et al.[11] noted that purposes of a reflective tasks were rarely articulated, and students were often unaware of the perspective, reflective purpose or role, they were expected to take. Sanders [14] concluded from a literature review of reflection in medical education that, for students to reflect effectively, they required clarity in determining the appropriate purpose of a reflective task.<br>- Facilitation reflection by medical educators:<br>Reflection is often provoked by uncertainty or unease resulting from an experience. [13],[22] According to Albanese, [22] in addition to this uncertainty or unease, a student requires knowledge of how to reflect, and time and motivation for reflection to occur. Supporting this opinion, Carr and Carmody [23] found in their qualitative study that some students could attain an acceptable level of reflection independently. However, reflection was greatly

For peer review only

No.	Title	Author/year	MERSQI	COREQ	Reason for inclusion			Undergraduate (UG)/Postgraduate (PG)	Methodology	Purpose of study	Key findings
					Communication	Ethics	Professionalism				
											enhanced through medical educators' facilitation. Medical educators' facilitation was found to be useful for reflection in one-on-one meetings with students and in small group tutorials. [9],[24] According to results of a study by Schaub-de Jong et al., [9] educators who wished to encourage reflection needed to model reflective practice by making their experiences more explicit, investigating emotions and stimulating interaction among students. Albanese [22] suggested that medical faculty staff should be trained to articulate their reflections to enhance modelling to students.<br>- Using written or web-based portfolios to facilitate reflection: Traditionally, reflective tasks have been paper based. Recently, web-based methods and social media have been adopted to facilitate reflective skills. Cook [29] asserted that there has been little study of the efficacy of these methods. Fischer et al. [30] compared content, depth of reflection and student preference for web-based versus traditional essay-style reflections in two medical schools in the USA. Ninety-five students were quasi-randomly assigned to one of two study groups, where one group wrote a traditional reflective essay and attended a small-group discussion, while the other group posted two writings in a group blog and commented on a peer's post. The writings were

No.	Title	Author/year	MERSQI	COREQ	Reason for inclusion			Undergraduate (UG)/Postgraduate (PG)	Methodology	Purpose of study	Key findings
					Communication	Ethics	Professionalism				
											coded for theme, and level of reflection, with the authors finding no difference between groups for either variable.<br>From discussion:<br>Traditionally, paper-based methods of reflection have been popular with educators, [7],[8],[25] but recently, web-based methods have been adopted. Driessen et al. [36] found that the method of reflection appears to be less important than the guidelines provided, which should be structured initially, and become more fluid as students' reflective skills progress. Ideally, students should be able to revisit and edit the reflection, hence web-based methods may be suitable for this purpose.<br>- Assessing the reflective work of students: ... (see relevant columns)<br>From discussion:<br>Several authors noted that reflection was an essential skill in medical practice, [2],[5],[7],[10],[14],[34] and advocated for training in reflective processes. [13],[22] Training in reflective practice can be significantly enhanced with the facilitation of skilled mentors, providing guidance and modelling of reflective behaviours, as found by Carr and Carmody. [23] Several studies and literature reviews report inconsistencies regarding the purpose of reflection, which was found to be a significant barrier to the facilitation of reflective practice. [11],[20],[21],[35] The implication for educators is that students

For peer review only

No.	Title	Author/year	MERSQI	COREQ	Reason for inclusion			Undergraduate (UG)/Postgraduate (PG)	Methodology	Purpose of study	Key findings
					Communication	Ethics	Professionalism				
											need to be given clear guidelines as to the purpose for each individual reflective task. Traditionally, paper-based methods of reflection have been popular with educators, [7],[8],[25] but recently, web-based methods have been adopted. Driessen et al. [36] found that the method of reflection appears to be less important than the guidelines provided, which should be structured initially, and become more fluid as students' reflective skills progress. Ideally, students should be able to revisit and edit the reflection, hence web-based methods may be suitable for this purpose. Students need to have the designated time and motivation for reflective practice. A common method of increasing task motivation in the education setting is to include the task as an assessment piece. There was some debate in the literature about the efficacy of this method. Some authors found that students' quality of reflection would be diminished if undertaken for an assessment task, [28] whereas others reported that reflection simply would not happen if not attached to an assessable piece of work. [22],[33] It is apparent from this review that the research surrounding the teaching and facilitation of reflection is generally weak. Although there are some descriptive data, there is very little empirical evidence of the efficacy of facilitation methods. Additionally, there

For peer review only

No.	Title	Author/year	MERSQI	COREQ	Reason for inclusion			Undergraduate (UG)/Postgraduate (PG)	Methodology	Purpose of study	Key findings
					Communication	Ethics	Professionalism				
											is a lack of rigorous research investigating the impact of reflection on practice skills or patient outcomes. A major limitation of literature is the breadth of the construct of reflection. A more defined construct of reflection, with clear outcomes, could lead to the development of benchmarks useful in tracking student progress and as research outcome measures. From conclusion: Most authors cited in this review suggested that the emphasis on reflection is increasing in medical curricula. The construct of reflection was broad in the reviewed literature. This suggests that medical educators must be clear on the purpose of reflection, the intended outcome for the individual student and the process necessary to achieve these aims for students. Evidence indicated that, if students are unclear as to the purpose of reflection and do not see educators modelling reflective behaviours, they are likely to undervalue this skill regardless of the associated learning and development opportunities embedded in the curriculum.[40]
66	Beyond assessment of learning toward assessment for learning: educating tomorrow's physicians	Dannefer, E. F. 2013	-	-	✓			UG	From abstract: Information-rich assessment data, structured opportunities for reflection, and facilitated self-assessment using a portfolio approach are designed to support	From abstract: We describe a program (based at Cleveland Clinic Lerner College of Medicine) in which explicit performance standards align these components and provide a roadmap for students to manage their learning.	From abstract: Preliminary evidence suggests that the program directs students towards learning, rather than on achieving a grade for grade's sake.

For peer review only

No.	Title	Author/year	MERSQI	COREQ	Reason for inclusion			Undergraduate (UG)/Postgraduate (PG)	Methodology	Purpose of study	Key findings
					Communication	Ethics	Professionalism				
									development of habits of reflective practice. Promotion depends on the achievement of competencies rather than grades. From the essential building blocks: All components and processes of a system need to be designed to interact in a way that accomplishes the goal of enhancing learning and habits of reflective practice. Key features of the CCLCM assessment system are now presented by addressing issues of competency standards, ongoing formative assessment, student responsibility, and purposeful support.		
67	Evidence within a portfolio-based assessment program: what do medical students select to document their performance?	Dannefer, E. F., Bierer, S. B. and Gladding, S. P.<br><br>2012	7		✓		✓	UG	From abstract: We reviewed portfolios to examine the number, type, and source of assessments selected by students (n = 32) to document their performance in seven competencies. The quality of assessment data selected for each	From abstract: In this article, we examine the nature of evidence selected by first-year medical students to include in a portfolio used to make promotion decisions. From introduction: This article attempts to address this gap in the literature by examining the types and sources of assessment	From abstract -- Results: Findings indicate that students cited multiple types and sources of available assessments. The promotion committee rated evidence quality highest for competencies where the program provided sufficient evidence for students to cite a broad range of assessments. When assessments were not provided by the program, students cited self-generated evidence.

For peer review only - http://bmjopen.bmj.com/site/about/guidelines.xhtml

No.	Title	Author/year	MERSQI	COREQ	Reason for inclusion			Undergraduate (UG)/Postgraduate (PG)	Methodology	Purpose of study	Key findings
					Communication	Ethics	Professionalism				
									competency was rated by promotion committee members (n = 14). From methods: We used two procedures to explore the assessment evidence that one cohort of medical students cited in their Year 1 summative portfolios. First, we examined the evidence cited in portfolio essays to ascertain what types and sources were selected. Second, we obtained feedback from promotion committee members concerning the quality of evidence via a questionnaire. - Citation coding procedures and data: A research assistant extracted essays from the e-Portfolio database for seven of the nine competencies included in the Year 1 summative portfolios that students submitted during the 2009–2010 academic year. Essays for the competencies of Personal Development and	evidence first-year medical students select from their performance database to construct portfolios used for promotion decisions. We describe our assessment program's evidence database before presenting the organizational elements designed to help students purposefully select data representative of their performance. We then describe the methods and results of the study before discussing implications about students' ability to select evidence within a centralized assessment program.	Conclusion: We found that when student-constructed portfolios are part of an overall assessment system, students generally select evidence in proportion to the number and types of assessments available.

For peer review only

No.	Title	Author/year	MERSQI	COREQ	Reason for inclusion			Undergraduate (UG)/Postgraduate (PG)	Methodology	Purpose of study	Key findings
					Communication	Ethics	Professionalism				
									Reflective Practice were excluded to ensure student confidentiality. Students' names on essays were replaced with a unique six-digit identifier. Overall, 224 essays for 32 students were reviewed for these seven competencies. Sophia Gladding sorted the essays by competency. She then read each set of essays nested within a given competency and manually recorded on a data extraction form all college-generated assessments, work samples and personal evidence students cited to document their performance of Year 1 standards. Extraction form data were entered into SPSS version 16.0, and descriptive statistics were computed after averaging across students' portfolios.<br>- Quality ratings: Promotions committee members involved with portfolio reviews (n = 18 faculty)		

For peer review only

No.	Title	Author/year	MERSQI	COREQ	Reason for inclusion			Undergraduate (UG)/Postgraduate (PG)	Methodology	Purpose of study	Key findings
					Communication	Ethics	Professionalism				
									completed a 21-item, web-based questionnaire after reading students' Year 1 summative portfolios. The questionnaire had been designed (Beth Bierer and Elaine Dannefer) and piloted earlier to monitor the Year 1 portfolio review process. This instrument included seven Likert-scaled items ranging from 1 "poor" to 4 "excellent" which asked promotion committee members to rate the overall quality of the assessment evidence they read for each competency after judging students' summative portfolios. Percentages were computed for each competency to summarize the committee's global ratings ("excellent" and "good/excellent") of evidence quality.		
68	The portfolio approach to competency-based assessment at the Cleveland Clinic Lerner College of Medicine	Dannefer, E. F. and Henson, L. C.<br><br>2007	-	-	✓		✓	UG	-	From abstract: This article describes how the portfolio was developed to provide both formative and summative assessment of student achievement in relation to the program's nine	Design of the portfolio assessment system: The search for an assessment system consistent with our principles led us to the literature on portfolios for medical student assessment14 –15,21–23 and to firsthand investigation of experiences

For peer review only

No.	Title	Author/year	MERSQI	COREQ	Reason for inclusion			Undergraduate (UG)/Postgraduate (PG)	Methodology	Purpose of study	Key findings
					Communication	Ethics	Professionalism				
										competencies. From introduction: In this article we contribute to the literature on competency-based assessment by describing a portfolio assessment system developed as an integral component of the new Cleveland Clinic Lerner College of Medicine (CCLCM) of Case Western Reserve University In this article, we describe the design of CCLCM's portfolio approach for a comprehensive, competency-based assessment system that is fully integrated with the curriculum. To provide context, we describe the program's competencies, performance standards, core assessment principles, and unique assessment instrument before addressing the design of the overall assessment system.	with portfolio assessment within and beyond national, disciplinary, and professional borders. At the time (2002– 2003), the portfolio assessment approach was being implemented in the United States in grades K–12 and at the undergraduate college level24 –26 and in the United Kingdom and Europe at a number of medical schools. For example, at the University of Dundee College of Medicine, as a key component of the final examination process, each final-year medical student was required to submit a portfolio documenting his or her progress in achieving the outcomes of the curriculum.14,15 The first-year medical student portfolio at the University of Maastricht Faculty of Medicine included students' reflective essays on their own development in four identified roles of the doctor as a method of encouraging integration of competencies.22 An Answer to the Challenges of Competency-Based Assessment: In our opinion, this system and its components should be applied in other settings across the medical education continuum because major themes in the current debate about assessment in medical education, such as integration across competencies, competency-based assessment methods, sampling from multiple contexts and multiple sources,

For peer review only

No.	Title	Author/year	MERSQI	COREQ	Reason for inclusion			Undergraduate (UG)/Postgraduate (PG)	Methodology	Purpose of study	Key findings
					Communication	Ethics	Professionalism				
											triangulation of information, and training of learners in reflective practice are addressed in this innovative method of student assessment.
69	Supporting students in self-regulation: use of formative feedback and portfolios in a problem-based learning setting	Dannefer, E. F. and Prayson, R. A.<br><br>2013		11	✓		✓	UG	From abstract: Formative feedback on works habits and interpersonal skills provided by peers and tutors to a Year 1 cohort (n ¼ 32) over the course of a year-long PBL experience (5 blocks) was examined for comments on targeted areas for improvement (TAFIs) and observed improvements. We examined congruence between PBL feedback and students' self-reported TAFIs and behavioral improvements in their assessment portfolios.<br>From study design and methods:<br>- Study design: This study presents an analysis of (1) narrative feedback on professionalism provided to Year 1 medical students from PBL peers, tutors, and self and (2) summative portfolio essays to	From abstract: This study examines the extent to which students self-regulate professionalism behaviors related to work habits and interpersonal skills in a PBL setting.<br>From introduction: In 2004, Cleveland Clinic Lerner College of Medicine (CCLCM) implemented a program designed specifically to promote the development of self-regulated learners (Dannefer & Henson 2007). ... We confront the question of whether or not students use CCLCM's learning environment to self-regulate performance by focusing on the core competency of professionalism.<br>We selected for this study of SR the content of PBL feedback on professionalism and its use by students to identify learning needs (self-assess) and document improvement (regulate). We asked these questions:<br>(1) To what extent did the content of peer and tutor PBL	From abstract -- Results: Both PBL peer and faculty feedback and portfolio self-assessments targeted Interpersonal Skills TAFIs more frequently than Work Habit-related issues. TAFIs were more frequently identified midway in PBL blocks versus the end. Students reported TAFIs in their portfolio essays, citing feedback from both peers and tutors, and provided evidence of improved performance over time.; Conclusions: Students utilized external formative feedback to document their portfolio self-assessment in a system designed to support self-regulation of PBL professionalism-related behaviors. A decrease in TAFIs identified at the end of PBL blocks suggests students made use of mid-block feedback to self-regulate behaviors.<br>From results:<br>- (1) To what extent did the content of peer and tutor PBL narrative feedback provide information relevant to the professionalism competency standards? -- Table 1 summarizes the content of aggregate PBL tutor and peer feedback as well as PBL self and portfolio self- assessments. Of all TAFIs identified by tutors, Interpersonal Skills account for 63%. Interpersonal Skills also dominated peer statements (57%). The majority of tutor

For peer review only

No.	Title	Author/year	MERSQI	COREQ	Reason for inclusion			Undergraduate (UG)/Postgraduate (PG)	Methodology	Purpose of study	Key findings
					Communication	Ethics	Professionalism				
									determine how students used PBL feedback to support their self-assessments. We limited the study to year one the time period reported by students, and supported by advisors' observations, as requiring significant adjustments in terms of taking responsibility for their own learning. - Measures: Faculty defined measurable, behavioral competency standards developmentally appropriate for Year 1 students and observ- able across multiple settings. ... Self-regulation, as a process, consists of overlapping and recursive activities of which we targeted self-assessments that identified areas needing improvement and cited evidence that documented improved performance. - Data sources: We collected peer and tutor PBL assessments electronically at mid and end of Block 1 problem-	narrative feedback provide information relevant to the professionalism competency standards? (2) To what extent did students self-report and document professionalism learning needs and improvements consistent with peer and tutor PBL feedback in a portfolio used for promotion decisions?	and peer TAFI statements about Interpersonal Skills indicated a concern with ''quietness'' (e.g. lack of participation). In the area of Work Habits, peers more frequently identified ''preparedness'' (e.g. disorganized presentations) as a TAFI in fellow students as compared with tutors, who more frequently documented behaviors associated with being ''distracted'' (e.g. carrying on side conversations). Peers were also more likely to give feedback on TAFIs than tutors; of 2016 peer assessments, 41% contained feedback on TAFIs. Overall, tutor feedback tended to be less detailed and only 28% of the 288 possible assessments contained feedback on TAFIs. Similar to tutor and peer evaluations, students self- identified Interpersonal Skills issues as TAFIs more often than Work Habit-related issues (60% vs. 40% respectively). Also similar to peer assessments, students frequently self-identified ''ill-preparedness'' as the Work Habit most needing improvement. In contrast to peer and tutor feedback, however, students more frequently self-identified ''dominating'' behavior issues rather than ''quietness''. All students, except for one who did not complete self-assessments, self-identified TAFIs also reported by their peers and tutors. Interestingly, 95% of students at some point during the year also self-identified issues not documented by peers and

No.	Title	Author/year	MERSQI	COREQ	Reason for inclusion			Undergraduate (UG)/Postgraduate (PG)	Methodology	Purpose of study	Key findings
					Communication	Ethics	Professionalism				
									solving sessions, and from peers, tutors and self at mid and end of Blocks 2-4 and end of Block 5 case-based sessions. Because professionalism cannot be compartmen-talized, students address standards not only in the Professionalism Competency essay but also in the Communication, Personal Development, and Reflective Practice competency essays. Thus, data used in this study included essays and citations for these four competencies.<br>- Coding and analysis of PBL narrative feedback: A trained research assistant extracted the electronic competency report of all PBL feedback for professionalism for all first-year students (n 1⁄4 32) for the 2009 academic year, and replaced students' names with a unique six-digit identifier. We		tutors. Table 1 shows that peer and tutor feedback and self-assessments documented improvements less frequently than TAFIs, and statements noting improvement in Interpersonal Skills were more fre- quent than statements of improvement in Work Habits. Figure 1 documents peer and tutor feedback statements for students at mid and end of the four PBL blocks. In aggregate, more TAFI statements were made by peers and tutors for mid-block assessments than at the end of the block with an overall decrease across the year. Regarding individual student data, 29 of the 32 students had more TAFIs documented at mid-block by peers as compared with end-of-block, and early in the year (Blocks 1–2) versus later in the year (Blocks 3–4). Similarly, more evidence with regard to improvements was documented for students by peers in end-of-block and late year feedback versus mid block and early year feedback, respectively. Similar trends were noted in the tutor feedback for mid- and end-of- block TAFIs and improvement statements.<br>- (2) To what extent did students self-report and document professionalism learning needs and improvements consistent with peer and tutor PBL feedback in a portfolio used for promotion decisions? --Student self-assessments presented in their summative portfolio essays are required to be a

For peer review only - http://bmjopen.bmj.com/site/about/guidelines.xhtml

No.	Title	Author/year	MERSQI	COREQ	Reason for inclusion			Undergraduate (UG)/Postgraduate (PG)	Methodology	Purpose of study	Key findings
					Communication	Ethics	Professionalism				
									developed a rubric using the PBL assessment form criteria for Interpersonal Skills and Work Habits to individually code five competency reports. The authors then met and reached consensus on coded professionalism statements about TAFIs and observed improvements. The refined Professionalism Rubric has three unprofessional behavior criteria each for Interpersonal Skills (Dominating, Quiet, Inappropriate) and for Work Habits (Late, Ill-prepared, Distracted). Each author coded the remaining reports before meeting to reach consen-sus and in rare instances of coding differences; these discrepancies were reconciled. The authors tallied type and number of professionalism statements for each student for each block from peers, tutors and self.		representative and balanced account of their professional behavior across the year. As an indicator of ‘‘representative’’, we identified behaviors that were observed by tutors in at least two different groups, which suggest stability of behavior across time and group dynamics. For the 16 students where two different tutors identified the same TAFI, peers identified the same TAFIs as the tutors in five PBL Blocks for seven students, four PBL blocks for six students, three PBL blocks for two students, two PBL blocks for one student. All 16 students reported the TAFI in their summative portfolio, citing both tutor and peer feedback. All cited evidence of improvement from peers and/or tutors. A strong majority (91%) of portfolio essays followed a pattern of citing feedback from PBL peers and/or faculty to identify at least one TAFI, followed by citations indicating improved performance. To illustrate student’s use of PBL feedback to document performance, we provide the portfolio self-assessment of one student who received ten TAFIs during the first half of the year and three in the second half, indicating a tendency to dominate. The tendency to dominate was mentioned by peers in four of the five PBL groups but never mentioned by tutors. These comments included statements such as ‘‘talking over people,’’ ‘‘can push ideas a little bit too hard ‘‘sometime can seem

For peer review only

No.	Title	Author/year	MERSQI	COREQ	Reason for inclusion			Undergraduate (UG)/Postgraduate (PG)	Methodology	Purpose of study	Key findings
					Communication	Ethics	Professionalism				
									- Coding and analysis of summative portfolio essays: A research assistant extracted the summative portfolio essays from the database for the four competencies (Professionalism, Communication, Personal Development, and Reflective Practice) that 32 students submitted at the end of the 2009 academic year, for a total of 124 essays. The authors used the Professionalism Rubric to analyze the portfolio essays for the aforementioned competencies, where professionalism issues were likely to be addressed. The authors independently coded professionalism statements before meeting to reach consensus in the rare instances where there were differences in coding. All PBL citations used to document the coded statements were tallied by source (tutor, peer, self) and block time period.		argumentative/defensive'' and needs to ''listen to other viewpoints even though they might not coincide with personal views.'' Three other TAFIs were mentioned three or less times and only in the first half of the year. Table 2 provides quotes from this one student's cited PBL feedback, documenting a TAFI (tendency to dominate) and evidence of improvement over the course of year 1. This student acknowledged and reflected on this TAFI in the portfolio essay and provided evidence of improvement, noting that this behavior may need to be ''reestablished'' with each new working group. See Table 3 for excerpt from this student's portfolio. The essay provides evidence relevant to each of our questions. The student identified and acknowledged a prob- lematic pattern of behavior, reflected on the feedback, and used suggestions to ''amend'' behavior, which resulted in improved performance. Not every student described the self- regulation process in such detail, but all acknowledged a behavior identified by peers and/or tutors that was problem- atic and presented evidence of improvement.<br>From discussion: ... While this study suggests that students are using the supports of this educational program to self-regulate, we need to better understand how students process and use feedback, how external feedback triggers and shapes intern- ally

No.	Title	Author/year	MERSQI	COREQ	Reason for inclusion			Undergraduate (UG)/Postgraduate (PG)	Methodology	Purpose of study	Key findings
					Communication	Ethics	Professionalism				
											generated feedback, what role advisors play in helping students reflect on and use feedback, and what processes students use to make judgments and develop plans for improvement, questions being addressed by the work of Sargeant et al. (2008, 2009, 2011). As future physicians, today's students will need to be able to self-regulate their behaviors in settings where feedback may not be as structured or robust and facilitation of reflection lacking. To support development of SR skills, we need medical school programs designed to provide formative feedback and clear standards, opportunities to improve, and facilitated self-assessment. The PBL-portfolio model provides one strategy by which students can gain skills in self-regulation and develop habits useful for future practice.
70	Portfolio assessment in medical students' final examinations	Davis, MH, Ben-David, M Friedman, Harden, RM, Howie, Peter, Ker, Jean, McGhee, C, Pippard, MJ and Snadden, David<br><br>2009	7		✓	✓		UG	From abstract: Portfolio assessment has been developed for this purpose and has been adopted for the summative assessment of students in their final examination in Dundee. The contents of the portfolio and the assessment process have been defined and the first cohort of students to be assessed in this	This paper describes a new approach to the final examination based on portfolios, which has been piloted in Dundee Medical School. This paper describes our first year's experience with this new approach.	From abstract: The evaluation of the approach demonstrated strong staff support. Students were also positive although with some reservations. It is concluded that portfolio assessment is a powerful approach to assessing a range of curriculum outcomes not easily assessed by other methods and is worthy of inclusion in the assessor's toolkit.<br>Results: Of the 129 candidates for the portfolio assessment, three were withdrawn for the reasons given above. Of the 126 remaining, 108 (86%)

For peer review only

No.	Title	Author/year	MERSQI	COREQ	Reason for inclusion			Undergraduate (UG)/Postgraduate (PG)	Methodology	Purpose of study	Key findings
					Communication	Ethics	Professionalism				
									way has been studied. The final examination: The final examination was redesigned to meet the needs of the new curriculum, in particular the moves to an inte- grated, task-based approach and an outcome-based education model. The examination is in two parts. Part 1 is at the end of year 4 and comprises a multiple-choice paper of extended matching items (134) designed to assess knowledge and its application; a constructed response question (CRQ) paper to assess higher-order thinking such as problem solving and critical analysis as well as knowl- edge; and an OSCE to assess clinical skills. The use of an OSCE as part of the final examination has been reported (Preece et al., 1992). Part 2 of the final examination takes place towards the end of year 5 and adopts a more innova- tive		passed, 13 (10%) received a condi- tional pass and five (4%) failed. No students fell into the bad fail category. Individual student profiles, in terms of the 22 grades for the learning outcomes, showed variation. Three students (2%) were awarded 22 A grades. One student (0.8%) was awarded 22 B grades. No student was awarded 22 C, D, E or F grades. Forty-four students (35%) were awarded 22 grades at either A or B levels. Two students (1.5%) were awarded 22 grades at either C or D levels. No students were awarded 22 grades at either E or F levels. Over 60% of the students were not consistent in their ability across the learning outcomes, but had individual strengths and weaknesses. The student gradings awarded by both pairs of exam- iners in each outcome are given in Figure 1. Non-pass gradings are confined to outcomes 2, 7, 8, 11 and 12. Evaluation of portfolio assessment: - (1) Analysis of student results: Numeric values from one to six were allocated to the students' outcome grades in the portfolio assessment. An overall mark was then calculated for every student. This overall mark was correlated, using the Spearman correlation, with the mark for each of the three compo- nents of Part 1 of the final examination (EMI, 0.42; CRQ, 0.42; OSCE, 0.47) and with the mark allocated to students

For peer review only

No.	Title	Author/year	MERSQI	COREQ	Reason for inclusion			Undergraduate (UG)/Postgraduate (PG)	Methodology	Purpose of study	Key findings
					Communication	Ethics	Professionalism				
									approach – portfolio assessment. The approach was adopted as it offered the potential of assessing the students' achievement in each of the curriculum outcomes including those not easily assessed by more traditional approaches. It also offered the potential of reviewing the development of student competence over time and presenting feedback to students.<br>Implementation of the portfolio assessment: Work for the portfolio assessment was carried out in three stages:<br>- Stage 1: Work carried out before the portfolio review -- Towards the end of year 5, before the PRHO preparation block, students submitted their completed portfolios to administrative staff in the medical school office. Following a check for completeness,		in the end-of-block examination for one phase 3 core block attachment (psychiatry, 0.34). These results show low/moderate correlation.<br>- (2) Observer documentation: Three observers, two internal and one external, were appointed to document any issues that arose during the portfolio assessment process. They reported that the port- folio submission procedures worked well. The countersigned receipt given to students was an essential step to avoid disputes over submitted material. Entering the pre-marked grades for various portfolio components into the PASS was a major task for administrative staff and required to be streamlined for future years. ... (see under issues related to implementation of portfolio) The PASS system worked well and provided signed sheets to be retained as a permanent record of the examination.<br>- (53) Examiners' evaluation questionnaire: The examiners' evaluation questionnaire was developed following discussion with members of the working group that introduced portfolio assessment and with both the external and internal examiners who participated in the portfolio examination. It was designed to gather the exam- iners' opinions regarding the: effectiveness of the portfolio approach to student assessment; usefulness of the components of the portfolio

No.	Title	Author/year	MERSQI	COREQ	Reason for inclusion			Undergraduate (UG)/Postgraduate (PG)	Methodology	Purpose of study	Key findings
					Communication	Ethics	Professionalism				
									they received and countersigned a receipt. The administrative staff transcribed from student records, onto an assessment summary sheet called the Port-folio Assessment and Summary Sheet (PASS), the grades awarded for the portfolio contents in respect of each of the curriculum outcomes. ... Three of the students were identified as ineligible to proceed to the portfolio review with the examiners at this stage, two for reasons of illness and non-completion of course work and one pending a disciplinary hearing. All three, however, were allowed to complete the portfolio review process for formative purposes.<br>- Stage 2: Portfolio review with the examiners -- ... (see under deciding how to mark/assess)<br>- Stage 3: Work completed after the portfolio review -- ... (see		in providing information about the students' abilities; conduct of the examination; standards achieved by the students; strengths and weaknesses of the portfolio approach to assessment; suggestions for improvement. Examiners were asked to rate a number of statements on a Likert-type five-point scale from 5—strongly agree to 1— strongly disagree. There were also free-response questions. The questionnaire was distributed and completed by the examiners at the end of the examiners' meeting on day 2. Thirty-three examiners' questionnaires were distributed and returned, giving a response rate of 100%. Responses were collated using the Excel computer software package by Microsoft. The percentage distribution of examiners' ratings with the number that responded to each statement (N) the calculated average score (AV) and the standard deviation (SD) are provided in Table 1. ... (see the rest under perspective of examiners)<br>- (4) Student evaluation questionnaire: The student evaluation questionnaire was developed following periodic feedback from the students during their phase 3 studies and from a student group discussion with the external observer that took place immediately after the portfolio review. Students were given the opportunity for free and frank expression of their opinions during this discussion. The

For peer review only

No.	Title	Author/year	MERSQI	COREQ	Reason for inclusion			Undergraduate (UG)/Postgraduate (PG)	Methodology	Purpose of study	Key findings
					Communication	Ethics	Professionalism				
									under deciding how to mark/assess) Evaluation of the portfolio assessment: Five methods were employed to evaluate the use of portfo- lios as the basis for the final examination: (1) analysis of student results; (2) observer documentation; (53) examiners' evaluation questionnaire; (4) student evaluation questionnaire; (5) verbal report from student representatives. (see details under results of study).; From discussion - evaluation methodology: Rossi & Freeman (1993a) (p. 13) define evaluation research as 'a robust arena of activity directed at collecting, analysing and interpreting information on the need for, implementa- tion of, and effectiveness and efficiency of intervention efforts'. Education evaluation is more specifically identified as 'the process of making judgement about		student questionnaire was designed to gather opinion about building the portfolio, about its focus around the curriculum outcomes and about events during the portfolio assessment. The questionnaire was distributed and completed 22 days after the examination, at a time when all students were aware of whether or not they had passed the examina- tion. Those students who were unsuccessful had received counselling. The rating scale, data entry and scoring system were similar to those used in the examiners' ques- tionnaire. A total of 107 student questionnaires were returned from 129 students who sat the examination—a response rate of 83%. The percentage distribution of students' ratings, the number that responded to each statement (N), the calcu- lated average score (AV) and the standard deviation (SD) are provided in Table 2 for building the portfolio and in Table 3 for the portfolio assessment. ... (see the rest under perspective of students)<br>- (5) Student representatives' comments: ... (see the rest under perspective of students)

For peer review only

No.	Title	Author/year	MERSQI	COREQ	Reason for inclusion			Undergraduate (UG)/Postgraduate (PG)	Methodology	Purpose of study	Key findings
					Communication	Ethics	Professionalism				
									the merit, value or worth of educational programmes' (Gall et al., 1996). It is recognized that it is often impossible to conduct educational evaluations according to the best possible design and Rossi & Freeman (1993b) advocate the concept of the 'good enough' evaluation. The five methods employed to evaluate the use of portfolios as the basis for the final examination followed the 'good enough' approach.		
71	A Qualitative Analysis of Narrative Preclerkship Assessment Data to Evaluate Teamwork Skills	Dolan, B. M., O'Brien, C. L., Cameron, K. A. and Green, M. M.<br><br>2018		15	✓		✓	UG	From abstract: We performed a qualitative analysis of narrative data in 15 assessment portfolios. Student portfolios were randomly selected from 3 groups stratified by quantitative ratings of teamwork performance gathered from small-group and clinical preceptor assessment forms. Narrative data included peer and faculty feedback from these same forms. Data were	We sought to understand if and how teamwork behaviors are described in the narrative feedback given by faculty and peers in preclerkship years and how this feedback may vary based on students' competency achievement level. We expect that review of assessment data collected in an electronic portfolio and judged by experienced faculty may fill the gap in teamwork assessment by providing a method to evaluate	From abstract -- Results: Eight codes related to teamwork emerged: attitude and demeanor, information facilitation, leadership, preparation and dependability, professionalism, team orientation, values team member contributions, and nonspecific teamwork comments. The frequency of codes and valence varied across the 3 performance groups, with students in the low-performing group receiving more suggestions for improvement across all teamwork codes. Conclusions: Narrative data from assessment portfolios included specific descriptions of teamwork behavior, with important contributions provided by

For peer review only

No.	Title	Author/year	MERSQI	COREQ	Reason for inclusion			Undergraduate (UG)/Postgraduate (PG)	Methodology	Purpose of study	Key findings
					Communication	Ethics	Professionalism				
									coded for teamwork-related behaviors using a constant comparative approach combined with an identification of the valence of the coded statements as either “positive observation” or “suggestion for improvement.” Study approach and setting: We conducted a qualitative study using a constant comparative approach to better characterize the narrative assessments that students receive regarding teamwork.17 ... We excluded assessments that do not include students' teamwork skills, including assessments from observed structured clinical exams, assessments of written assignments, medical knowledge assessments, assessments of groups as a whole (where no individual feedback was given), ethics oral examinations, direct observations of	longitudinal, behaviorally based narrative feedback regarding student teamwork knowledge, skills, and attitudes.	both faculty and peers. A variety of teamwork domains were represented. Such feedback as collected in an assessment portfolio can be used for longitudinal assessment of preclerkship student teamwork skills and attitudes. From discussion: This analysis demonstrates that narrative feedback in assessment portfolios provides a longitudinal view of preclerkship students' teamwork behaviors. Although similarities exist between performance groups, we observed differences between groups in content, frequency, and valence of feedback. These differences could assist mentor/coaches and competency committee members in identifying both students who excel at teamwork and those needing additional support with teamwork behaviors prior to starting clerkships. Although our coded categories were identified without the use of a predetermined framework, they align with key teamwork and leadership attributes identified in the literature. Critical teamwork skills and attitudes have previously been described that share similarity with our codes: leadership, team orientation, preparation/dependability.21 Physician leadership competencies highlight the importance of showing integrity, listening actively, promoting team building, and nurturing/recognizing others' accomplishments.22–24

For peer review only

No.	Title	Author/year	MERSQI	COREQ	Reason for inclusion			Undergraduate (UG)/Postgraduate (PG)	Methodology	Purpose of study	Key findings
					Communication	Ethics	Professionalism				
									clinical skills, and scholarly project oral presentations (which focus solely on presentation skills). Sampling strategy: Students were notified of the study via e-mail and were encouraged to e-mail an author not involved in grading students (CLO) if they did not wish to participate. Students were given 1 week to respond. Four students chose to opt out of the study. We divided the remaining student portfolios into three groups. We first identified the students who received progressing towards competence with concern or progressing towards competence pending additional development for any of the three competencies most closely related to teamwork—teamwork, professionalism, and communication—during the competency review. We		These attributes arose in our analysis, lending credibility to the argument that narrative data in assessment portfolios can be used to understand students' teamwork behaviors in the preclerkship years and can help students reflect on how their behaviors promote or detract from their teams' overall performance. Review of the preclerkship narrative data provides mentors or coaches the ability to identify students in need of remediation before full-time immersion in clinical practice.25 Our analysis demonstrates that low-performing students share similar characteristics that reviewers can look for poor attitude in small groups, lack of preparation for group activities, or dismissive behavior toward others' contributions. It also demonstrates that the absence of certain comments may be telling, as evidenced by the sharp reduction in even nonspecific comments such as “great team member” or the lack of leadership observations in the low-performing student portfolios. When reviewing assessment data with their students, mentors may choose to highlight these absences to help students improve. Notably, peers provided the majority of feedback in the codes of team orientation and values' contributions of others. Due to their close working relationships during preclerkship years, peers may be in the best position

For peer review only

No.	Title	Author/year	MERSQI	COREQ	Reason for inclusion			Undergraduate (UG)/Postgraduate (PG)	Methodology	Purpose of study	Key findings
					Communication	Ethics	Professionalism				
									designated these 19 students as the low-performing group. As our competency review does not have a comparable high-performance designation, we employed an alternative strategy using the composite grading system. To identify a high-performing cohort of students for comparison, we identified the 19 students receiving the highest numerical ratings for the group of assessments included in Table 1. We designated this to be the high-performing group. The middle-performing group comprised all remaining students (n = 114) who did not meet criteria for the high- or low-performing groups. Four students opted out of the study. To protect privacy, each student portfolio received a random numerical identifier. A key was created linking each identifier to each student's ID number; an		to make such judgments. Furthermore, when peers are given formalized instruction in providing feedback, as they are at our institution, including their insight and feedback is critical when assessing student performance. Our results suggest that assessment systems that do not include peer feedback may miss the opportunity to identify students in need of support for developing teamwork skills. Prior studies demonstrate student acceptance of peer feedback, particularly when used for formative purposes.26 Additional studies that specifically compare faculty versus student feedback may further illuminate the best ways to employ the differing perspectives of peers and faculty when assessing student performance. The use of assessment portfolio data may continue to increase as institutions implement entrustable professional activities, competency committees, and other assessment strategies that require a systems-based approach.27 We believe that the findings in this analysis can assist both in teamwork assessment and as a resource to aid faculty and students in identifying preclerkship goals for teamwork and leadership skills development. Additional study is needed to determine if preclerkship assessment of teamwork correlates with later clinical performance. In conclusion, our analysis of narrative

No.	Title	Author/year	MERSQI	COREQ	Reason for inclusion			Undergraduate (UG)/Postgraduate (PG)	Methodology	Purpose of study	Key findings
					Communication	Ethics	Professionalism				
									author not involved in direct teaching of students (CLO) deidentified all data before analysis. We randomly selected three student portfolios from each group (high, middle, low) to analyze, then further intentionally sampled two additional portfolios in each group to allow for adequate gender inclusion and to confirm emergence of no new codes (see next); these 15 portfolios composed our sample. Data analysis: To better understand how teamwork performance is observed and described by peers and faculty, we used constant comparative analysis techniques. We selected this method as a framework to explore teamwork given that comparison between groups of students may better delineate the similarities and/or differences in how behaviors were described among variably		assessment data from across the preclerkship curriculum demonstrates that these data capture a number of key teamwork skills and attitudes with behaviorally based feedback, thus filling a gap in the longitudinal assessment of teamwork. Providing students and faculty the opportunity to critique and further reflect on behaviorally based narrative feedback may assist students in improving these skills prior to the beginning of clerkships.

For peer review only

No.	Title	Author/year	MERSQI	COREQ	Reason for inclusion			Undergraduate (UG)/Postgraduate (PG)	Methodology	Purpose of study	Key findings
					Communication	Ethics	Professionalism				
									performing students. Constant comparative methodology has been described as an ideal way to "conceptualise the variety that exists within the subject under study.17(p393) After using this methodology to complete the coding, we undertook a content analysis to better understand the prevalence and valence of identified themes across the three performance groups.19 Three research team members (BMD, CLO, MG) with expertise in medical student assessment performed the primary analysis of portfolio comments; two are practicing clinicians with experience on medical teams (BMD, MG). The fourth team member, an expert in qualitative research (KAC), provided guidance and oversight in methodology. Narrative data was uploaded for analysis into		

For peer review only

No.	Title	Author/year	MERSQI	COREQ	Reason for inclusion			Undergraduate (UG)/Postgraduate (PG)	Methodology	Purpose of study	Key findings
					Communication	Ethics	Professionalism				
									NVivo (QSR, Mac version 10.2.0) after deidentification.20 We selected the middle-performing group as the first group for analysis, as it represents the typical student who is neither struggling nor exceptionally high achieving. Consistent with conventional content analysis19 and to best explore what teamwork categories faculty and students choose to comment on in their narratives, we did not use an a priori framework for coding. However, one author (BMD) is familiar with medical teamwork literature,21,22 which has informed the way medical students approach teamwork in our institution and which may have influenced her approach to coding. The second coder has a background in education. She had no specific expertise with the teamwork literature and arrived at the same codes. Two		

For peer review only

No.	Title	Author/year	MERSQI	COREQ	Reason for inclusion			Undergraduate (UG)/Postgraduate (PG)	Methodology	Purpose of study	Key findings
					Communication	Ethics	Professionalism				
									authors (BMD and CLO) initially reviewed three portfolios from the middle-performing group, assigning a code to all narrative statements. These authors then met and achieved consensus regarding the coding framework. The remaining portfolios in the middle-performing group were analyzed using the identified codes; all statements were assigned a valence of either “positive observation” or “suggestion for improvement.” Three authors (BMD, CLO, MG) met to review the statements' assigned codes and come to consensus on any differences, both with respect to code and valence. Differences were resolved via group discussion. Using the codes identified, we then analyzed the low- and high-performing groups, representing the extremes of performance.		

For peer review only

No.	Title	Author/year	MERSQI	COREQ	Reason for inclusion			Undergraduate (UG)/Postgraduate (PG)	Methodology	Purpose of study	Key findings
					Communication	Ethics	Professionalism				
									Although no new codes were identified in the high-performing or low-performing groups, the narrative from these groups helped to refine the coding set to reflect nuances that emerged from the other groups. For example, after reviewing the high-performing narrative data, the original code “information sharing” was revised to “information facilitation” to better reflect the variety of ways that students mediated the flow of information in small groups. The code “values contributions of team members” evolved from “values team members” after review of the additional suggestions for improvement found in both the high- and low-performing groups. We reviewed three portfolios in each performance group but identified few new codes after the second student's portfolio in each group. However,		

For peer review only

No.	Title	Author/year	MERSQI	COREQ	Reason for inclusion			Undergraduate (UG)/Postgraduate (PG)	Methodology	Purpose of study	Key findings
					Communication	Ethics	Professionalism				
									we continued with two additional portfolios per group for adequate gender inclusion and to confirm the emergence of no new codes; no new codes were found with the addition of these six portfolios. Upon completion of the coding process, NVivo software was used to quantify the number of comments per code and valence. Techniques to ensure trustworthiness of data included the use of two coders followed by additional review of the codes by a third author. Codes and examples were also discussed at a medical education group research meeting where additional faculty members with less connection to the portfolio process provided feedback to the research team.		
72	Portfolio as a learning tool: students' perspective	Elango, S., Jutti, R. C. and Lee, L. K.<br><br>2005	6.5		✓			UG	From abstract: A questionnaire survey was conducted among 143 medical students to find out their perceptions of the	This study was formulated to find out students' perspectives on portfolio learning.	From abstract: ... (see the rest under perspective of students) The study indicates that students need appropriate guidance from the academic staff for the system to succeed.<br>From introduction: In the

For peer review only - http://bmjopen.bmj.com/site/about/guidelines.xhtml

No.	Title	Author/year	MERSQI	COREQ	Reason for inclusion			Undergraduate (UG)/Postgraduate (PG)	Methodology	Purpose of study	Key findings
					Communication	Ethics	Professionalism				
									portfolio as a learning tool. From materials and methods: Feedback was obtained from an entire batch of Semester 8 students by asking them to write down what they felt about writing a portfolio. Based on their feedback, a questionnaire consisting of 23 questions was designed. The entire student population (259) in the clinical school (from Semester 6 to Semester 10) was given an information sheet with details about this survey and was given the option of participating in the study. The students who volunteered for the study were asked to sign a written consent form. The students who gave their consent for the survey were requested to complete the questionnaire anonymously. For each of the 23 questions, students had to respond on a 4-point Likert scale, ranging from		last decade, there have been numerous innovations in educational theory and practice. Education has moved from being a traditional teacher-centred process to one that is student-centred. Similarly, in the area of assessment, there has been a shift away from assessing knowledge towards a more competency/performance-based assessment. There is now a wide variety of methods of education and training to choose from and portfolio-based learning is an increasingly popular option among educationists.1<br>From results: One hundred and forty-three students completed and returned the questionnaire (Table1). There were 76 (53.1%) females and 64 (44.8%) males. In order to facilitate the analysis, the 4 levels of perception in the questionnaire – strongly agree, agree, disagree and strongly disagree – were regrouped; strongly agree and agree were grouped as one, and strongly disagree and disagree were grouped as the other. ... (see the rest under perspective of students)<br>From discussion: A study has shown that students using portfolios obtain higher marks in factual knowledge compared to the control group of students not using portfolios11<br>From conclusion: The study demonstrates that any medical school intending to introduce the portfolio as a new learning method must take into consideration an

No.	Title	Author/year	MERSQI	COREQ	Reason for inclusion			Undergraduate (UG)/Postgraduate (PG)	Methodology	Purpose of study	Key findings
					Communication	Ethics	Professionalism				
									strongly agree to strongly disagree. The responses to the questionnaire were analysed to determine the overall views of the students on the different aspects of portfolio writing.		effective orientation programme for both the learners and the teachers. Motivated students, and trained and motivated staff are essential for portfolio-based learning to succeed.
73	Students' reflections in a portfolio pilot: highlighting professional issues	Haffling, A. C., Beckman, A., Pahlmblad, A. and Edgren, G. 2010		14	✓	✓	✓	UG	From abstract: Thirty-five voluntary final-year medical students piloted a standardized portfolio in a general practice (GP) attachment at Lund University, Sweden. Students' portfolio reflections were based upon documentary evidence from practice, and aimed to demonstrate students' learning. The reflections were qualitatively analysed, using a framework approach. Students' evaluations of the portfolio were subjected to quantitative and qualitative analysis. Pilot: Students of two consecutive classes, of 76 and 71 students, respectively, were approached. Twenty students in each class	From abstract: To investigate whether students' reflections include sufficient dimensions of professional competence, notwithstanding a standardized portfolio format, and to evaluate students' satisfaction with the portfolio From introduction: We aimed to develop a portfolio for the assessment of final-year students' professional development during their general practice (GP) attachment. ... We designed a pilot study with the principal aim of investigating whether themes of students' reflections would include satisfactory dimensions of professional competence, notwithstanding the standardized format. This article qualitatively explores the emergent themes of students' reflections, and also reports on students' evaluation of the portfolio.	From abstract -- Results: Among professional issues, an integration of cognitive, affective and practical dimensions in clinical practice was provided by students' reflections. The findings suggested an emphasis on affective issues, particularly on self-awareness of feelings, attitudes and concerns. In addition, ethical problems, clinical reasoning strategies and future communication skills training were subjects of several reflective commentaries. Students' reflections on their consultation skills demonstrated their endeavour to achieve structure in the medical interview by negotiation of an agenda for the consultation, keeping the interview on track, and using internal summarizing. The importance of active listening and exploration of patient's perspective was also emphasized. In students' case summaries, illustrating characteristic attributes of GP, the dominating theme was 'patient-centred care', including the patient–doctor relationship, holistic modelling and longitudinal continuity. Students were satisfied with the portfolio,

For peer review only

No.	Title	Author/year	MERSQI	COREQ	Reason for inclusion			Undergraduate (UG)/Postgraduate (PG)	Methodology	Purpose of study	Key findings
					Communication	Ethics	Professionalism				
									were offered to voluntarily pilot a portfolio in GP as an addition to the written test, which for the participants would be reduced in the number of GP questions. A total of 35 students, 16 from the first and 19 from the second class, accepted. Twenty-four (69%) of them were women, as compared to 59% of all students. At the time of the study, consent from the University's ethics committee was not required for projects involving students' assessments. Participating students were provided with both verbal and written guidelines on the purpose, format and assessment of their portfolios. Three of the authors (ACH, AP, AB) and a fourth teacher, all involved in teaching, acted as mentors to support their students in compiling the portfolio. Qualitative analysis of students' reflections: To be		but improved instructions were needed.; Conclusions: A standardized portfolio in a defined course with a limited timeframe provided ample opportunities for reflections on professional issues. Support by mentors and a final examiner interview contributed to the success of the portfolio with students. The interview also allowed students to deepen their reflections and to receive feedback. Students' evaluation questionnaire: see under perspective of students From discussion: Firstly, students' reflections on their consultation skills showed that the most important learning issue in the theme of 'process skills' was how to achieve structure in the medical interview. Patient-centred communication can cause genuine concerns in students about losing control of the consultation and getting caught in a flow of less ordered information from the patient (Silverman et al. 2004). When students learned how to obtain structure, they also experienced better time management, enhanced self-confidence and an improved rapport with the patient. Secondly, the theme of 'patient-centred care' was emphasized in students' case summaries. Patient-centred care is a major principle in GP, but is of essential interest to several other medical disciplines as well. Achieving shared decision making, understanding the patient as

For peer review only

No.	Title	Author/year	MERSQI	COREQ	Reason for inclusion			Undergraduate (UG)/Postgraduate (PG)	Methodology	Purpose of study	Key findings
					Communication	Ethics	Professionalism				
									able to conduct the qualitative analysis, students' reflections were written verbatim into a word processor by one of the authors (173). Each student was allocated a code number to prevent identification of individual students' comments. The text of the reflections constructed two separate units of analysis; 'consultation skills' and 'case summaries'. The transcripts were read through several times to get a sense of the whole, and then analysed using a framework approach (Crabtree & Miller 1999). For 'consultation skills', the text was mapped against the Calgary Cambridge Guides (Silverman et al. 2004). For 'case summaries', the statement of WONCA Europe (2005), (Box 2), was used as a template for organizing the data. In each of		a whole person and exploring the patient's perspective are aspects shown to positively influence patients' satisfaction and enablement, reduce their symptom burden and discomfort, and increase efficiency of care (Stewart et al. 2000; Little et al. 2001). We believe that these central dimensions of patient-centred care ought to be highlighted earlier in hospital clerkships. Thirdly, the findings suggested integration of the cognitive, affective and practical dimensions of professional competence that can be acquired in a clinical learning environment (Mann et al. 2007; van Tartwijk & Driessen 2009; Passi et al. 2010). As expected, students reflected on their 'content skills' and provided evidence for their identification of deficits in knowledge and skills, their integration of new understanding, and their plans for development. However, 'perception skills' was a more prominent theme in both units of analysis. In their self-awareness of feelings, attitudes and concerns, students were unexpectedly open and frank about their deficiencies and about how they had tried to remedy them. They also acknowledged the importance of ethical issues and additional communication skills training, and openly reflected on their clinical reasoning. However, we found it remarkable that

For peer review only

No.	Title	Author/year	MERSQI	COREQ	Reason for inclusion			Undergraduate (UG)/Postgraduate (PG)	Methodology	Purpose of study	Key findings
					Communication	Ethics	Professionalism				
									the two units of analysis, related segments of text were identified and indexed to the appropriate part of the thematic framework. Some codes were modified and new ones added to reflect as many nuances in the data as possible. 'Chunks' of related text under each code were examined together and distilled summaries made; this process involved a considerable amount of abstraction. The chunk summaries provided an analytical tool from which connections were made and sub-themes and themes identified. ACH undertook the mapping of text and carried out the primary analysis of the data. The four teachers, involved in the pilot, examined in recurrent meetings whether the interpretation of the data was plausible. The codes and sub-themes were compared based on differences		several students, in their final year in medical school, reflected on their initial problems in transferring theory to practice; a skill supposed to be frequently trained during previous hospital clerkships. From conclusion: In summary, this study demonstrated that a structured portfolio in a short timeframe provided abundant opportunities for students to reflect on personal and professional issues, notwithstanding the standardized format. We strongly recommend a final examiner interview to deepen students' reflections, to supply feedback and to raise the importance of reflective ability as a skill in future learning from practice.

No.	Title	Author/year	MERSQI	COREQ	Reason for inclusion			Undergraduate (UG)/Postgraduate (PG)	Methodology	Purpose of study	Key findings
					Communication	Ethics	Professionalism				
									and similarities, and some were revised during this process of corroboration. Students' evaluation questionnaire: The short student evaluation form was supplied directly after the examiner interview, asking five questions about the portfolio instructions, the mentor contact and the examiner interview, using a 3-point scale ('yes', 'to some extent', 'no'). Students were also requested to provide free comments on positive and negative experiences of the portfolio, and to give suggestions for improvements. The results were subjected to quantitative and qualitative (content) analyses.		
74	Undergraduate medical students' views about a reflective portfolio assessment of their communication skills learning	Rees, C. and Sheard, C. 2004	10		✓			UG	From abstract: 178 second-year medical students at the University of Nottingham completed the 18-item reflective portfolio questionnaire (RPQ) (α = 0.716) and a personal details questionnaire	this study explores second-year medical students' views about a reflective portfolio assessment of their communication skills. The research questions for this study are: 1) What are students' views of a reflective portfolio assessment	From abstract -- Results: Total scores on the RPQ ranged from 40 to 75 (mean 58.28, SD 7.08). Significant relationships existed between RPQ total scores and students' ratings of their reflection skills (r s = 0.322, P < 0.001), RPQ total scores and students' confidence building another portfolio (T = 4.381, d.f. = 176, P

For peer review only

No.	Title	Author/year	MERSQI	COREQ	Reason for inclusion			Undergraduate (UG)/Postgraduate (PG)	Methodology	Purpose of study	Key findings
					Communication	Ethics	Professionalism				
									three days before submitting their portfolio assessment for communication skills. Data were analysed using univariate and multivariate statistics on SPSS Version 10.0. From methods: Participants -- Second-year medical students at the University of Nottingham completed a personal details questionnaire (PDQ) and the reflective portfolio questionnaire (RPQ) three days before submitting their portfolio assessment for communication skills.; Measures -- The PDQ contains 13 demographic and education-related items. The RPQ was designed by CR to explore medical students' views of portfolios and reflection. Eighteen items were generated using previous research literature,1-4 e.g. ‘I have felt anxious about sharing my weaknesses in my reflective portfolio’ (item 16). Each item	of their communication skills? 2) Do relationships exist between students' views of reflective portfolios and their perceived and actual reflection skills and their confidence building portfolios?	< 0.001), and RPQ total scores and students' marks for their reflective portfolio assessment (r s = 0.167, P = 0.029). Students with more positive views about reflective portfolios were more likely to rate their reflection skills as good, receive better marks for their portfolio assessment, and be more confident building another portfolio. Discussion: This study begins to highlight preclinical medical students' views about reflective portfolios. However, further research is required using qualitative studies to explore students' views in depth. Medical educators should be encouraged to consider introducing portfolios as a method of formative and summative assessment earlier in the medical curriculum. From results: - Participant characteristics: Of the 206 students invited to participate in this study, 178 (86.4%) completed the reflective portfolio questionnaires satisfactorily. Students ranged in age from 18 to 30 years (median = 20, interquartile range = 19–20). The majority were female (n = 105, 59%), white (n = 130, 73.4%) and came from nonmanual socio-economic classes (n = 170, 95.5%). Students' ratings of their communication skills ranged from 1 to 5 (median = 3, interquartile range = 3–3) and nearly all (n = 173, 97.2%) thought their communication skills needed improving. Students' ratings of their

For peer review only

No.	Title	Author/year	MERSQI	COREQ	Reason for inclusion			Undergraduate (UG)/Postgraduate (PG)	Methodology	Purpose of study	Key findings
					Communication	Ethics	Professionalism				
									was written in the form of a statement and was accompanied by a 5-point Likert scale from 1 (strongly disagree) to 5 (strongly agree). Six medical educators with a range of experience of reflective portfolios commented on the language used in the preliminary draft of the questionnaire, which resulted in some minor alterations in language. The RPQ is scored by reversing the scores for the negative items and then summing the scores for all 18 items. The total score ranges from 18 to 90, with higher scores indicating more positive views about reflective portfolios. This scale was found to have good internal consistency in this study (Cronbach's α= 0.716) and the study results presented in this paper support the construct validity of the tool (copies of the RPQ can		reflection skills ranged from 1 to 5 (median = 3, interquartile range = 2–3) and most (n = 155, 87.6%) thought their reflection skills needed improving. ... (see the rest under perspective of students) Students' scores for their reflective portfolio coursework ranged from 33 to 100% (median = 67, interquartile range = 60–73%).<br>- Students' views about reflective portfolios: see the rest under perspective of students<br>From discussion: ... (see the rest under perspective of students) These findings have implications for further research and educational practice. Qualitative studies are needed to explore in depth the reasons why students possess the views they have about reflective portfolios. Qualitative research may also shed some light on how medical educators could increase students' confidence in building reflective portfolios. This qualitative research could help medical educators improve educational programs using portfolios, so that they are more acceptable to students. In the meantime, this study indicates that the act of constructing a reflective portfolio could increase students' confidence in building another portfolio. Despite portfolios being seen to have weaknesses (e.g. lacking reliability4), medical educators should be encouraged to consider introducing portfolios as a

No.	Title	Author/year	MERSQI	COREQ	Reason for inclusion			Undergraduate (UG)/Postgraduate (PG)	Methodology	Purpose of study	Key findings
					Communication	Ethics	Professionalism				
									be requested by contacting the author). Students' percentage marks for their reflective portfolio assessments were collected at a later date.; Data analysis -- After checking the distribution of continuous variables, data were analysed using univariate (i.e. Spearman's correlation coefficients and T -tests) and multivariate statistics (i.e. stepwise multiple regression) on SPSS Version 10.0.		method of formative and summative assessment earlier in the medical curriculum. Students may then be better placed to negotiate this type of assessment in higher stakes examinations.
75	The reliability of assessment criteria for undergraduate medical students' communication skills portfolios: the Nottingham experience	Rees, C. E. and Sheard, C. E.<br><br>2004	8.5		✓			UG	From abstract: Two independent analysts assessed a random sample of portfolios (n = 100, 49.5%) using criterion-referenced assessment. Students' performances were examined against subjective items in five areas: 1) portfolio structure, 2) level of critical reflection, 3) level of skills development, 4) use of documentary evidence, and 5) use of relevant literature. These	From abstract: This study aims to determine the reliability of assessment criteria used for a portfolio at the University of Nottingham.	From abstract -- Results: The level of agreement between the two raters for the total percentage score was 0.771 (95% CI = 0.678, 0.840), as measured by an intraclass correlation coefficient. The levels of agreement between the two raters for the individual items of the assessment criteria ranged from κ = 0.359 (item 3) to κ = 0.693 (item 4). Discussion: This study provides some support for the summative assessment of portfolios. The findings suggest that discussion and negotiation between independent assessors can enhance the reliability of assessment criteria. Therefore, medical educators are encouraged to use such procedures in the summative assessment

For peer review only

No.	Title	Author/year	MERSQI	COREQ	Reason for inclusion			Undergraduate (UG)/Postgraduate (PG)	Methodology	Purpose of study	Key findings
					Communication	Ethics	Professionalism				
									subjective judgements were later converted into quantitative scales ranging from 0 to 3 so that interrater reliability could be established. The level of agreement between the two analysts for the total percentage score was established using an intraclass correlation coefficient and for the individual items using weighted kappa coefficients. From methods: Sample -- Of the 202 portfolios submitted in March 2002, a random sample of 100 (49.5%) were photocopied for second marking. This random sample of 100 portfolios (median = 66.7%, interquartile range = 53.3–73.3%) did not differ significantly from the portfolios (n = 102) not chosen for second marking (median = 66.7%, interquartile range = 53.3–73.3%) in terms of their total percentage scores (Z = − 0.155, P = 0.877).; Assessment		of portfolios. From results: The level of agreement between the two raters for the total percentage scores, as measured by an intraclass correlation coefficient, was 0.771 (95% CI = 0.678, 0.840). The levels of agreement between the two raters for the individual items, as measured by weighted kappa coefficients, ranged from 0.359 (item 3) to 0.693 (item 4) (see Table 1).

No.	Title	Author/year	MERSQI	COREQ	Reason for inclusion			Undergraduate (UG)/Postgraduate (PG)	Methodology	Purpose of study	Key findings
					Communication	Ethics	Professionalism				
									procedures -- One hundred portfolios were assessed ... Although students were aware of these five areas, they were not give the full assessment criteria outlined in Appendix 1. ... (see the rest under deciding how to mark/assess); Statistical procedures: An intraclass correlation coefficient on SPSS (Version 10.0) was used to establish the level of agreement between the two raters for the total percentage scores. Weighted kappa coefficients on SAS (Release 6.12) were used to determine the levels of agreement between the two raters for the individual items of the assessment criteria.		
76	Portfolio as a tool to evaluate clinical competences of traumatology in medical students	Santonja-Medina, F., Garcia-Sanz, M. P., Martinez-Martinez, F., Bo, D. and Garcia-Estan, J.<br><br>2016	10.5		✓			UG	From abstract: A total of 131 students used the portfolio during their clinical rotation of traumatology. The students' portfolios were blind evaluated by four professors who annotated the existence	From abstract: This article investigates whether a reflexive portfolio is instrumental in determining the level of acquisition of clinical competences in traumatology, a subject in the 5th year of the degree of medicine.<br>From introduction: ...	From abstract: The reliability of the portfolio was moderate, according to the kappa index (0.48), but the evaluation scores between evaluators were very similar. Considering the mean percentage, 59.8% of the students obtained all the competences established and only 13 of the 23 learning outcomes (56.5%) were fulfilled by >50% of the

For peer review only

No.	Title	Author/year	MERSQI	COREQ	Reason for inclusion			Undergraduate (UG)/Postgraduate (PG)	Methodology	Purpose of study	Key findings
					Communication	Ethics	Professionalism				
									(yes/no) of 23 learning outcomes. From methods: A portfolio was designed to assess the level of acquisition of clinical competences, ... - Academic structure: Traumatology is a compulsory subject in the 5th year of the degree, consisting of 55 hours of theoretical classes, of which 28 hours take place in the classroom, 14 hours are of a seminar type, 10 hours are about clinical cases, and 3 hours are about cooperative learning; 21 hours of classes on laboratory skills in seven sessions; and 2 weeks of hospital rotation (50 hours, 10 days). Of these 50 hours, students rotate in the traumatology service of the university hospitals between hospital ward, medical consultation, operating rooms, and emergency services. Only one student is assigned to one professor in the	Thus, in the current article, we report the application of a portfolio tool to assess the level of acquisition of clinical skills and competences in medical students of the Medical School of the University of Murcia (Spain), during the clinical rotations of traumatology and orthopedic surgery. An important part of the study is also devoted to the analysis of the reliability of the portfolio.29	students. Our study suggests that the portfolio may be an important tool to quantitatively analyze the acquisition of traumatology competences of medical students, thus allowing the implementation of methods to improve its teaching. From results: A total of 138 comparisons were made to obtain the kappa index among the four evaluators for each of the 23 learning outcomes. The distribution of frequencies and percentages of agreement, according to the classification of Landis and Koch,29 are shown in Table 3. As it can be seen, out of 138 comparisons, 83 had kappa indexes >0.4 and 44 of them had values >0.6, which indicates that in a 31.9% of the occasions, the agreements between evaluators have been very substantial. Table 4 shows the mean and standard deviation of the kappa index of the four evaluators. It can be seen that the global kappa is 0.48, indicating that the portfolio has a moderate reliability, according to Landis and Koch.29 As an additional way of assessing the reliability of the portfolio, 6 months after the initial evaluation, two of the evaluators revised again the portfolios (second revision or reference). Table 5 shows the level of agreement obtained by each evaluator when both revisions were analyzed. Globally, the differences among evaluators are not very important because kappa indexes show that

For peer review only

No.	Title	Author/year	MERSQI	COREQ	Reason for inclusion			Undergraduate (UG)/Postgraduate (PG)	Methodology	Purpose of study	Key findings
					Communication	Ethics	Professionalism				
									hospital. Table 2 shows the number of days students spent during these rotations.<br>- Portfolio structure: The portfolio was given to all the students during the course (n=131) in a booklet format. After the identification of data, the students read about legal and ethical issues in relation to the need for keeping medical secret and signed conformity. Each day the students were expected to fill up their reflections before leaving the hospital, writing about the clinical activities observed during the day. In addition, the portfolio had to be signed by the assigned professor every day. The students were given enough space to include everything needed, but there were three main paragraphs: ... (see under content of portfolio)<br>- Portfolio evaluation: The portfolios were evaluated by four		evaluators 1 and 4 have a substantial agreement in both revisions, whereas evaluators 2 and 3 have a moderate level of agreement. The level of achievement of the traumatology competences is shown in Table 1. The data show the mean percentage of the four evaluations as well as the number of times the procedures were performed. A total of 59.8% of the students have acquired the competences as expected during their hospital stay. In all, 13 of the 23 learning outcomes (56.5%) have been acquired by >50% of the students. Competence 5 (87.4%) and competence 1 (81.4%) show the greater values, whereas the rest of them show an acceptable level, except competence 7, showing only a value of 26%.<br>From discussion: The current article shows the results obtained with the application of a portfolio to medical students as a tool to obtain information regarding the level of acquisition of the clinical competences of the topic traumatology. One of the important aspects of any portfolio is its reliability, the consistency and accuracy of the assessment tool in measuring students' performance. Our results quite agree with those found by other authors;30,31 although in some studies of nurse students, higher numbers (0.8) have been described.32 To prove that the results were not obtained by chance, the

For peer review only

No.	Title	Author/year	MERSQI	COREQ	Reason for inclusion			Undergraduate (UG)/Postgraduate (PG)	Methodology	Purpose of study	Key findings
					Communication	Ethics	Professionalism				
									professors, three of them professors of traumatology and one a colleague professor from a different area, that is, physiology; all of them were medical doctors. ... (see the rest under deciding how to mark/assess) All the portfolios underwent blind evaluation. All the students agreed and participated enthusiastically in the study.<br>- Statistical analysis: Data analysis was performed using the Statistical Package for the Social Sciences, Version 19. The Cohen's kappa coefficient was used as a statistical measure of interevaluator agreement for qualitative items.29 Frequencies, percentages, means, and standard deviations were also obtained to describe the results.		second review of the portfolios took place 6 months after the initial evaluation in order to see whether the reliability index could be improved. As observed in Table 5, the maximum level of agreement among evaluators is found in competence 6 (observe and assist in surgical treatments). A moderate agreement exists in competences 2, 3, and 4, whereas competences 1, 5, and 7 show a low level of agreement among evaluators. The results of Table 1 show important information regarding the level of acquisition of the competences analyzed. Two of them, competences 1 and 5, related to diagnostic and treatment obtained the greatest value, with an important frequency of occurrence. Next competence in terms of the level of acquisition is number 3 (70%), probably due to the high number of radiographic images viewed in contrast to magnetic resonance images, where it is likely that doctors rely more on the radiologist report, without actually interpreting them. Competence 6 has also a moderate level of acquisition (55.8%), in spite of the fact that almost all students (99.2%) report attendance to operating rooms (2.6 + 1.3 days). This is likely due to the fact that the surgeons do not invite all the students to wash and be prepared to help, an activity we recommend to all our professors. A similar

For peer review only

No.	Title	Author/year	MERSQI	COREQ	Reason for inclusion			Undergraduate (UG)/Postgraduate (PG)	Methodology	Purpose of study	Key findings
					Communication	Ethics	Professionalism				
											reason may be behind the moderate level achieved in competences 2 (47.1%) and 4 (51.5%), showing that not all the students are able to explore or perform maneuvers with the patients and that this may be due to a time limitation problem while in the ward or consultation. We believe that this should be corrected and doctors should allow more time for the students to perform, under the appropriate supervision, exploration, or treatment of the patients. Finally, competence 7 has the lowest level. It is likely related to the fact that students are not allowed to sign discharge reports or other forms, although it is important that they learn from their professors how to do it. In any case, this is the first time that we obtained this quantitative information regarding the clinical stays of the medical students and this will help us to analyze our teaching results and to develop new strategies to enhance the level of clinical competence the students should reach. A problem learned from the application of this portfolio is that many of the learning outcomes were checked without taking into account the number of times these were carried out. The rest of the learning outcomes were quantitative, so we could obtain the number of times the student performed them. For instance, they attended the operating rooms 5.5 + 3.3 times (range, 1–26), and viewed a mean of 2.3 magnetic resonance images

For peer review only

No.	Title	Author/year	MERSQI	COREQ	Reason for inclusion			Undergraduate (UG)/Postgraduate (PG)	Methodology	Purpose of study	Key findings
					Communication	Ethics	Professionalism				
											(range, 1–14). We believe that it is important that the portfolios are designed to collect also the quantitative information, since both quantitative and qualitative information give the real essence of the learning process. A drawback we have seen with the application of the portfolio to clinical students is that there is a random factor affecting the acquisition of the competences. This is due to the fact that the students rotate between different hospitals and at different times of the year. Thus, it depends on the type of medical problems available on the days they are assigned to the hospital; thus they may not see all the problems selected in the portfolio. Clearly, the greater the number of days of hospital stay, the better it is. However, this is not always possible. A better selection of the learning outcomes will also be helpful. Moreover, the portfolio is designed to be an evaluation tool that should be used in conjunction with others (written or oral exams, laboratory evaluations) because it is not possible to find just one tool that is able to assess all the competences.33,34<br>From conclusion: In conclusion, the portfolio is an important tool that has allowed to obtain qualitative and quantitative data regarding the degree of acquisition of traumatology competences by medical students. Although some factors need to be improved, such as the

For peer review only

No.	Title	Author/year	MERSQI	COREQ	Reason for inclusion			Undergraduate (UG)/Postgraduate (PG)	Methodology	Purpose of study	Key findings
					Communication	Ethics	Professionalism				
											inclusion of more quantitative elements, a more comprehensive selection of learning outcomes, and combination with other tools, we believe that the use of this kind of tool by clinical teachers will be useful to ascertain the degree of practical competence reached by their students.
77	Assessing the Development of Medical Students' Personal and Professional Skills by Portfolio	Yielder, J. and Moir, F.<br><br>2016		6			✓	UG	The implementation process employed a cyclical model that was designed to evaluate and improve the use of the portfolio, that could form the basis of the first cycle of action research that will be continued in the future to improve the initiative (Fig. 1). During 2013, the introduction of the portfolio was monitored, and changes were made in 2014. In 2014, feedback from students indicated the need for further modifications, which are currently being implemented, along with the introduction of an electronic platform. Student feedback was ascertained from the generic end-of-year course evaluation, which included	From abstract: We present the portfolio format along with the process for its introduction and appraise the challenges, strengths, and limitations of the approach within the context of the current literature. We then outline a cyclical model of evaluation used to monitor and fine-tune the portfolio tasks and implementation process, in response to student and assessor feedback.<br>From introduction: This document outlines the learning outcomes that are to be substantively completed by the end of the second postgraduate year in practice. ... This article presents the process for introducing the portfolio, including the challenges, strengths and limitations, evaluation of data to date, and the ways in which student	From abstract: The portfolios have illustrated the level of insight, maturity, and synthesis of personal and professional qualities that students are capable of achieving. The Auckland medical program strives to foster these qualities in its students, and the portfolio provides an opportunity for students to demonstrate their reflective abilities. Moreover, the creation of a Personal and Professional Skills domain with the portfolio as its key assessment emphasizes the importance of reflective practice and personal and professional development and gives a clear message that these are fundamental longitudinal elements of the program.<br>From conclusion:<br>The implementation of a portfolio for the assessment of the PPS domain in the medical program at the University of Auckland has proved to be an important part of the redeveloped curriculum. It has highlighted the importance of PPS topics and has provided an opportunity and incentive for students to reflect on aspects of their personal and professional development, with the aim

For peer review only

No.	Title	Author/year	MERSQI	COREQ	Reason for inclusion			Undergraduate (UG)/Postgraduate (PG)	Methodology	Purpose of study	Key findings
					Communication	Ethics	Professionalism				
									questions relating to the PPS domain. The number of students in each year varied from 199–260 and there was an average response rate of 50%. In addition, the Auckland University Medical Students Association conducted a student evaluation for the purpose of writing a report for the ongoing medical program accreditation, from which key issues were collated and forwarded to the PPS domain coordinators. The eight portfolio assessors also provided suggestions for improvement at the end of 2013 and 2014. Evaluation feedback, modifications, and qualitative data derived from the portfolios are presented in the Findings section. Due to the qualitative nature of the feedback, it was analyzed inductively, through a cross-sectional thematic analysis that sought to	feedback has been addressed.	of improving their practice as doctors. In the process, we have learned several aspects about students that previously may have remained unknown, which has led in some cases to conversations with students that will hopefully make a difference to their learning and development trajectories. We have also seen many high-quality and insightful portfolios that demonstrate a synthesis of personal and professional qualities that are inspiring and which we hope are an indication that these students will develop into excellent doctors. With the increasing use of, and requirement for, the compilation of professional portfolios in the medical profession internationally, especially at postgraduate level, there is real benefit for students in becoming familiar with this process of pregraduation.<br>It has also been a learning process for the domain coordinators introducing the assessment. In particular, we learned the value of a cyclic, flexible, and ongoing process of evaluation that has allowed us to continuously reflect on what is working and what needs to change in order to be responsive to student needs and at the same time create a curriculum change that aims to create better doctors. ... We hope that as students come to trust our responsiveness, they will become increasingly open to the importance of taking on the responsibility for their

For peer review only

No.	Title	Author/year	MERSQI	COREQ	Reason for inclusion			Undergraduate (UG)/Postgraduate (PG)	Methodology	Purpose of study	Key findings
					Communication	Ethics	Professionalism				
									ascertain the strongest themes from across the three sources of feedback.		own learning in the PPS domain.
78	Construct Validity of an Instrument for Assessment of Reflective Writing-Based Portfolios of Medical Students	Kassab, Salah Bidmos, Mubarak Nomikos, Michail Daher-Nashif, Suhad Kane, Tanya Sarangi, Srikant Abu-Hijleh, Marwan<br><br>2020	7		✓		✓	UG	After an extensive literature review and pilot testing of the instrument, two raters assessed the reflective writing-based portfolios from years 2 and 3 medical students (n=135) on three occasions. The instrument consists of three criteria: organization, description of an experience and reflection on the experience. We calculated the reliability of scores using generalizability theory with a fully crossed design and two facets (raters and occasions). In addition, we measured criterion validity by testing correlations with students' scores using other assessment methods.	Assessment of reflective writing for medical students is challenging, and there is lack of an available instrument with good psychometric properties. The authors developed a new instrument for assessment of reflective writing-based portfolios and examined the construct validity of this instrument.	The dependability (Φ) coefficient of the portfolio scores was 0.75 using two raters on three occasions. Students' portfolio scores represented 46.6% of the total variance across all score comparisons. The variance due to occasions was negligible, while the student– occasion interaction was small. The variance due to student–rater interaction represented 17.7%, and the remaining 27.7% of the variance was due to unexplained sources of error. The decision (D) study suggested that an acceptable dependability (Φ = 0.70 and 0.72) can be achieved by using two raters for one and two occasions, respectively. Finally, we found moderate to large effect-size correlations between students' scores in reflective writing-based portfolios and communication skills (r = 0.47) and PBL tutorials (r = 0.50).<br><br>We demonstrated the presence of different sources of evidence that support construct validity of the study instrument. Further studies are warranted before utilizing this instrument for summative assessment of students' reflective writing-based portfolios in other medical schools.
79	Communication skills teaching	Douglas, A. H. Acharya, S. P.		19	✓			UG	This study is a qualitative	Communication skills (194) are vital for	The two main themes: 1. Positivity

No.	Title	Author/year	MERSQI	COREQ	Reason for inclusion			Undergraduate (UG)/Postgraduate (PG)	Methodology	Purpose of study	Key findings
					Communication	Ethics	Professionalism				
	and learning in Nepal; what are medical students' perceptions and experiences? A qualitative study	Allery, L. A.<br><br>2020							evaluation of a CS course in Nepal, exploring the experiences and perceptions of participants. The study aims to also identify aspects that were helpful or not for student learning and areas for potential development.<br><br>A purposive sample of twenty: second, fourth and Intern year students was selected for interview. Data were collected through audio recorded semi-structured interviews following a piloted schedule. Interview transcripts were manually coded and thematically analysed. Codes were arranged into themes and subthemes.	doctors. Indeed, as the most important element of consultations, are highly valued by patients. CS are core, teachable skills, however, have not been widely taught in South Asian medical schools, unlike their western counterparts.<br><br>Patan Academy of Health Sciences, is one of the first in Nepal to have CS central to its' aims and curriculum. CS are taught from the first weeks of medical school and re-enforced during preclinical study (first 2 years). Our study seeks to explore students' perceptions and experiences of CS teaching in this South Asian, Nepal context.	2. Experiential learning. Results demonstrate participants' positive perceptions of CS teaching: believing it is important, effective, relevant and valuable for personal development. Participants identified experiential learning features as valuable for CS acquisition. Intern students recognised CS relevance and requested expanding teaching to clinical years, incorporating challenging communication scenarios.<br><br>This study shows that PAHS' CS course is well perceived and valuable to learners. Experiential learning is powerful for CS development. Expansion of formal, structured CS teaching through all years in a spiral curriculum, should be considered. Violence towards doctors in South Asia is increasing. Students recognised CS teaching's significance in addressing this.<br><br>CS teaching,still in its' infancy in South Asia, is a pressing issue for medical educators here. Our study provides evidence it is well perceived with positive impacts in this context, particularly when employing experiential learning. Medical schools in south Asia should be encouraged to incorporate and strengthen their CS teaching curriculum.
80	Assessment practices in undergraduate	Brits, H.<br>Bezuidenhout, J.<br>Van der Merwe, L. J.	5.5	12	✓			UG	A descriptive cross-sectional study design was	Assessment should form an integral part of curriculum design	All disciplines in the undergraduate medical programme are

No.	Title	Author/year	MERSQI	COREQ	Reason for inclusion			Undergraduate (UG)/Postgraduate (PG)	Methodology	Purpose of study	Key findings
					Communication	Ethics	Professionalism				
	clinical medicine training: What do we do and how can we improve?	Joubert, G.<br><br>2020							used. Qualitative and quantitative data were gathered by means of open- and closed-ended questions in a self-administered questionnaire, which was completed by teaching and learning coordinators in 13 disciplines.	in higher education and should be robust enough to ensure clinical competence.<br><br>This article reports on current assessment practices and makes recommendations to improve clinical assessment in the undergraduate medical programme at the University of the Free State.	represented. They used different assessment methods to assess the competencies required of entry-level healthcare professionals. Workplace-based assessment was performed by 30.1% of disciplines, while multiple-choice questions (MCQs) (76.9%) and objective structured clinical examinations (OSCEs) (53.6%) were the main methods used during formative assessment. Not all assessors were well prepared for assessment, with 38.5% never having received any formal training on assessment. Few disciplines (15.4%) made use of post-assessment moderation as a standard practice, and few disciplines always gave feedback after assessments.<br><br>The current assessment practices for clinical students in the undergraduate medical programme at the University of the Free State cover the spectrum that is necessary to assess all the different competencies required. Multiple-choice questions and OSCEs, which are valid and reliable assessment methods, are used frequently. Poor feedback and moderation practices should be addressed. More formative assessments, and less emphasis on summative assessment, should be considered. Workplace-based and continuous assessments may be good ways to assess clinical competence.

For peer review only

No.	Title	Author/year	MERSQI	COREQ	Reason for inclusion			Undergraduate (UG)/Postgraduate (PG)	Methodology	Purpose of study	Key findings
					Communication	Ethics	Professionalism				
81	**Added value of assessing medical students' reflective writings in communication skills training: a longitudinal study in four academic centres**	Ament Giuliani Franco, C.<br>Franco, R. S.<br>Cecilio-Fernandes, D.<br>Severo, M.<br>Ferreira, M. A.<br>de Carvalho-Filho, M. A.<br><br>2020	10.5		✓			UG	Third-year and fourth-year medical students enrolled in an elective course on clinical communication skills development were assessed using different assessment methods.<br><br>The communication skills course was offered at four universities (three in Brazil and one in Portugal) and included 69 students.<br><br>The students were assessed by a Multiple-Choice Questionnaire (MCQ), an objective structured clinical examination (OSCE) and reflective writing narratives. The Cronbach's alpha, dimensionality and the person's correlation were applied to evaluate the reliability of the assessment methods and their correlations. Reflective witting was assessed by applying the Reflection Evaluation for Enhanced Competencies Tool Rubric	This study describes the development and implementation of a model to assess students' communication skills highlighting the use of reflective writing. We aimed to evaluate the usefulness of the students' reflections in the assessment of communication skills.	The Cronbach alpha for the MCQ, OSCE global score, TS and RS were, respectively, 0.697, 0.633, 0.784 and 0.850. The interobserver correlation for the TS and RS were, respectively, 0.907 and 0.816. The assessment of reflection using the TS was significantly correlated with the MCQ (r=0.412; p=0.019), OSCE (0.439; p=0.012) and RS (0.410; p=0.020). The RS did not correlate with the MCQ and OSCE.<br><br>Assessing reflection through mapping the themes and analysing the depth of reflective writing expands the assessment of communication skills. While the assessment of reflective themes is related to the cognitive and behavioural domains of learning, the reflective depth seems to be a specific competence, not correlated with other assessment methods—possibly a metacognitive domain.

No.	Title	Author/year	MERSQI	COREQ	Reason for inclusion			Undergraduate (UG)/Postgraduate (PG)	Methodology	Purpose of study	Key findings
					Communication	Ethics	Professionalism				
									(Reflect Score (RS)) to measure reflections' depth, and the Thematic Score (TS) to map and grade reflections' themes.		
82	Contextual Analysis of Stakeholder Opinion on Management and Leadership Competencies for Undergraduate Medical Education: Informing Course Design	Rajeh, N. Grant, J. Farsi, J. Tekian, A.<br><br>2020		16	✓			UG	This was a cross-sectional study conducted at King Abdulaziz University (KAU), Saudi Arabia, during 2019. An exploratory qualitative approach, utilizing systematic content analysis, was used. Data were collected using semi-structured interviews that were con- ducted with 10 leaders who were stakeholders at KAU, health service providers at KAU hospital, and stakeholders in the Ministry of Health.	The study aimed to conduct a contextual analysis of interviews intended to assist with the future design of a feasible and relevant leadership and management course for undergraduate medical students at King Abdulaziz University (KAU), Saudi Arabia.	This study revealed critical findings that highlighted the areas in which KAU could instill better and adequate leadership and management skills in their undergraduate medical students. Multiple core categories for a leadership and management curriculum emerged with many interrelated themes. Most participants mentioned that leadership can be taught and that early exposure is beneficial for develop- ing skills. Additionally, they stated that leaders should have a vision and the ability to articulate that vision.<br><br>Different implementation challenges were described in relation to the availability of human resources, the current short supply of suitable teachers, and program design. Teaching methods recommended included simulations, lectures, and a project-based approach. Assessment methods that were recommended included objective structured clinical examination (OSCE), formative and summa- tive assessments, self-assessments, and portfolios.

For peer review only

BMJ Open

An evidence-guided approach to Portfolio guided Teaching and Assessing Communications, Ethics and Professionalism for medical students and physicians:A Systematic Scoping Review

Journal:	*BMJ Open*
Manuscript ID	bmjopen-2022-067048.R2
Article Type:	Original research
Date Submitted by the Author:	27-Feb-2023

Complete List of Authors:	Ting, Jacquelin Jia Qi; National University of Singapore, Yong Loo Lin School of Medicine; National Cancer Centre Singapore, Division of Supportive and Palliative Care<br>Phua, Gillian Li Gek; National Cancer Centre Singapore, Division of Supportive and Palliative Care; Duke-NUS Medical School, Lien Centre for Palliative Care<br>Hong, Daniel Zhihao; National University of Singapore, Yong Loo Lin School of Medicine; National Cancer Centre Singapore, Division of Supportive and Palliative Care<br>Lam, Bertrand Kai Yang; National Cancer Centre Singapore, Division of Supportive and Palliative Care<br>Lim, Annabelle Jia Sing ; National University of Singapore, Yong Loo Lin School of Medicine; National Cancer Centre Singapore, Department of Supportive and Palliative Care<br>Chong, Eleanor Jia Xin; National University of Singapore, Yong Loo Lin School of Medicine; National Cancer Centre Singapore, Division of Supportive and Palliative Care<br>Pisupati, Anushka; National University of Singapore, Yong Loo Lin School of Medicine; National Cancer Centre Singapore, Division of Supportive and Palliative Care<br>Tan, Rei; National University of Singapore, Yong Loo Lin School of Medicine; National Cancer Centre Singapore, Division of Supportive and Palliative Care<br>Yeo, Jocelyn Yi Huang; National University of Singapore, Yong Loo Lin School of Medicine; National Cancer Centre Singapore, Division of Supportive and Palliative Care<br>Koh, Yi Zhe; National University of Singapore, Yong Loo Lin School of Medicine; National Cancer Centre Singapore, Division of Supportive and Palliative Care<br>Quek, Chrystie; National University of Singapore, Yong Loo Lin School of Medicine; National Cancer Centre Singapore, Division of Supportive and Palliative Care<br>Lim, Jia Yin; National University of Singapore, Yong Loo Lin School of Medicine; National Cancer Centre Singapore, Division of Supportive and Palliative Care<br>Tay, Kuang Teck; National Cancer Centre Singapore, Division of Supportive and Palliative Care<br>Ong, Yun Ting; National University of Singapore, Yong Loo Lin School of

Medicine; National Cancer Centre Singapore Division of Supportive and Palliative Care
Chiam, Min; National Cancer Centre Singapore, Division of Cancer Education
Zhou, Xuelian Jamie; National Cancer Centre Singapore, Division of Supportive and Palliative Care; Duke-NUS Medical School, Lien Centre for Palliative Care
Mason, Stephen; University of Liverpool, Palliative Care Institute Liverpool
Wijaya, Limin; National Cancer Centre Singapore, Division of Supportive and Palliative Care; Duke-NUS Medical School
Krishna, Lalit; University of Liverpool,

**Primary Subject Heading**:	Communication
Secondary Subject Heading:	Communication, Ethics, Medical education and training
Keywords:	MEDICAL EDUCATION & TRAINING, ETHICS (see Medical Ethics), EDUCATION & TRAINING (see Medical Education & Training)

SCHOLARONE™
Manuscripts

I, the Submitting Author has the right to grant and does grant on behalf of all authors of the Work (as defined in the below author licence), an exclusive licence and/or a non-exclusive licence for contributions from authors who are: i) UK Crown employees; ii) where BMJ has agreed a CC-BY licence shall apply, and/or iii) in accordance with the terms applicable for US Federal Government officers or employees acting as part of their official duties; on a worldwide, perpetual, irrevocable, royalty-free basis to BMJ Publishing Group Ltd ("BMJ") its licensees and where the relevant Journal is co-owned by BMJ to the co-owners of the Journal, to publish the Work in this journal and any other BMJ products and to exploit all rights, as set out in our licence.

The Submitting Author accepts and understands that any supply made under these terms is made by BMJ to the Submitting Author unless you are acting as an employee on behalf of your employer or a postgraduate student of an affiliated institution which is paying any applicable article publishing charge ("APC") for Open Access articles. Where the Submitting Author wishes to make the Work available on an Open Access basis (and intends to pay the relevant APC), the terms of reuse of such Open Access shall be governed by a Creative Commons licence – details of these licences and which Creative Commons licence will apply to this Work are set out in our licence referred to above.

Other than as permitted in any relevant BMJ Author's Self Archiving Policies, I confirm this Work has not been accepted for publication elsewhere, is not being considered for publication elsewhere and does not duplicate material already published. I confirm all authors consent to publication of this Work and authorise the granting of this licence.

For peer review only

Title: An evidence-guided approach to Portfolio guided Teaching and Assessing Communications, Ethics and Professionalism for medical students and physicians:

A Systematic Scoping Review

Short Title: A review of CEP Portfolios for physicians and medical students

Jacquelin Jia Qi Ting[1,2], Gillian Phua[2,3,4], Daniel Zhihao Hong [1,2], Bertrand Kai Yang Lam[2], Annabelle Jia Sing Lim[1,2], Eleanor Jia Xin Chong[1,2], Anushka Pisupati[1,2] , Rei Tan[1,2], Jocelyn Yi Huang Yeo[1,2], Yi Zhe Koh[1,2], Chrystie Wan Ning Quek[1,2], Jia Yin Lim[1,2], Kuang Teck Tay[2], Yun Ting Ong[1,2], Min Chiam[5,] , Jamie Zhou [2,3,4], Stephen Mason[8], Limin Wijaya[2,3,9], Lalit Kumar Radha Krishna[1-8,#]

[1] Yong Loo Lin School of Medicine, National University of Singapore, NUHS Tower Block, 1E Kent Ridge Road, Level 11, Singapore 119228

[2] Division of Supportive and Palliative Care, National Cancer Centre Singapore,11 Hospital Dr, Singapore 169610

[3] Duke-NUS Medical School, 8 College Rd, Singapore 169857

[4] Lien Centre for Palliative Care, Duke-NUS Medical School, 8 College Rd, Singapore 169857

[5] Division of Cancer Education, National Cancer Centre Singapore, 11 Hospital Dr, Singapore 169610

[6] PalC, The Palliative Care Centre for Excellence in Research and Education, PalC c/o Dover Park Hospice, 10 Jalan Tan Tock Seng, Singapore

[7] Centre of Biomedical Ethics, National University of Singapore, 21 Lower Kent Ridge Rd, Singapore 119077

[8] Palliative Care Institute Liverpool, Academic Palliative & End of Life Care Centre, Cancer Research Centre, University of Liverpool, 200 London Rd, Liverpool L3 9TA, United Kingdom

[9] Department of Infectious Diseases, Singapore General Hospital, Outram Road, Singapore 169608

[#]Corresponding Author:

Dr. Lalit Kumar Radha Krishna

Palliative Care Institute Liverpool, Academic Palliative & End of Life Care Centre,

University of Liverpool, 200 London Rd, Liverpool L3 9TA, United Kingdom

Email: lalit.radha-krishna@liverpool.ac.uk Phone number: 6436 8183

For peer review only

ABSTRACT

Objectives

Guiding the development of longitudinal competencies in communication, ethics, and professionalism underlines the role of portfolios to capture and evaluate the multiple multi-source appraisals, and direct personalised support to clinicians. However a common approach to these combined portfolios continues to elude medical practice. A systematic scoping review is proposed to map portfolio use in training and assessments of ethics, communication, and professionalism competencies particularly as its inculcation of new values, beliefs and principles changes attitudes, thinking, practice, while nurturing Professional Identity Formation. It is posited that effective structuring of portfolios promote self-directed learning, personalised assessment and appropriate support of professional identity formation.

Design

Krishna's Systematic Evidence-Based Approach is employed to guide this systematic scoping review of portfolio use in communication, ethics and professionalism training and assessment published in PubMed, Embase, PsycINFO, ERIC, Scopus, and Google Scholar databases between 1st January 2000 and 30th June 2020. The included articles are concurrently content and thematically analysed using the Split Approach. Overlapping categories and themes identified are combined using the Jigsaw Perspective. The themes/categories are compared with the summaries of the included articles in the Funnelling Process to ensure their accuracy. The domains identified form the framework for the discussion

Results

12,300 abstracts were reviewed, 946 full-text articles were evaluated, and 82 articles were analysed and the four domains identified were indications; content; design; and strengths and limitations.

Conclusions

This review reveals that when using a consistent framework, accepted endpoints and outcome measures, longitudinal multisource, multimodal assessment data fashions professional and personal development and enhances identity construction. Future studies into effective assessment tools and support mechanisms are required if portfolio use is to be maximised.

For peer review only - http://bmjopen.bmj.com/site/about/guidelines.xhtml

Strengths and limitations of this study

- The SEBA methodology adopts the structure of systematic reviews and flexibility of narrative reviews to synthesise a reproducible and accountable evaluation of diverse methodological sources, settings, physician populations and specialities.
- This review provides the first insights into the impact of longitudinal development of ethics, professionalism and communications competencies and their impact on professional identity formation

Abstract Word Count: 273 words

Manuscript Word Count: 1999 words

Number of Tables: 5

Number of Figures: 2

Number of References: 152

Keywords: Portfolios, Communication, Ethics, Professionalism, Professional Identity Formation, Medical Education, medical school, undergraduate, postgraduate training.

For peer review only

For peer review only - http://bmjopen.bmj.com/site/about/guidelines.xhtml

INTRODUCTION

Evidence for the effective embodiment of ethical and professional principles, communication skills and appropriate use of empathy in clinical practice requires a longitudinal, and often multi-source perspective. With ethics and professionalism sharing longitudinal developmental trajectories and intimately entwined with communication skills and competencies, the three competencies are increasingly considered together. In practice longitudinal CEP programs appear in the curricula of the Top 10 medical schools featured on the QS World University Rankings 2020 (1). Concurrent study of communication, ethics, and professionalism (henceforth CEP) is also underlined by their common sociocultural roots. As sociocultural constructs (2), CEP competencies are shaped by the individual's personal experiences, motivations, enthusiasm, idealism, abilities, competencies, virtues, expectations, knowledge, skills, emotions, and attitudes (henceforth narratives); their values, beliefs, and principles (henceforth belief systems)(3); their clinical experiences, competencies, training, insights, and confidence (henceforth clinical insights)(4, 5, 6, 7) and their practice, clinical, social , cultural, academic, research, and personal considerations (henceforth contextual considerations)(8, 9, 10, 11, 12).

However, whilst previous reviews into the teaching of ethics (13, 14, 15), communication (16, 17, 18, 19, 20, 21, 22) and professionalism (23, 24, 25) suggest the use of portfolios could provide a personalised, holistic, and longitudinal perspective of CEP skills, knowledge, and attitudes, and support of developing competencies we are aware of little progress in designing such platforms. Impetus for mapping current use of CEP portfolios also arises from the notion that developing CEP competencies shape how medical students and physicians (henceforth clinician) "think, act and feel like a physician" (26) or their professional identity formation (henceforth PIF) (27). It is posited that the promise of CEP portfolios with better appreciation of evolving self-concepts of professional identity will better direct support and even remediation of professional, ethical, communication and interprofessional development and PIF. This is especially pertinent at a time of increasing reports of breaches in standards, codes of conduct, and social and practice expectations (25).

METHODS

A Systematic Evidence-Based Approach (SEBA) guided Systematic Scoping Review (SSR) (henceforth SSR in SEBA) is proposed to map CEP portfolio use in medicine to guide the design, structuring and support of a proposed program (13, 14, 15, 16, 17, 20, 21, 22, 24, 25, 27, 28, 29, 30, 31, 32, 33, 34, 35, 36, 37, 38, 39, 40, 41, 42, 43, 44, 45, 46, 47, 48, 49, 50). Given space constraints, we briefly describe the six stages in the construction

For peer review only - http://bmjopen.bmj.com/site/about/guidelines.xhtml

of SSRs in SEBA here (Figure 1) and advance more details of the Systematic Approach, Split Approach, Jigsaw Perspective, Funnelling Process, Reiterative Process and Synthesis of SSR, in Appendix A (16, 27, 28, 35, 41, 48, 51, 52, 53, 54, 55, 56).

(Insert Figure 1. *The SEBA Process*)

Stage 1 of SEBA: Systematic Approach

A. Determining of Title and Background of the Review

An expert team comprised of a medical librarian from the Yong Loo Lin School of Medicine (YLLSoM) at the National University of Singapore (57), and local education experts and clinicians at the National Cancer Centre Singapore (NCCS), the Palliative Care Institute Liverpool, YLLSoM and Duke-NUS Medical School (henceforth the expert team) supported the research team in designing and overseeing the SEBA process.

B. Identification of Research Question

The research and expert team determined the primary research question: "*What is known about CEP portfolios?*". The secondary research questions were "*what role do CEP portfolios have in teaching and assessing CEP development?*".

C. Inclusion Criteria

A Population, Intervention, Comparison and Outcome (PICOs) format, outlined in Table 1, was used to guide the research process (58, 59).

Table 1. PICOS Inclusion and Exclusion Criteria

PICOS	Inclusion criteria	Exclusion criteria
**Population**	• Undergraduate and postgraduate medical students<br>• Qualified medical doctors, physician or resident; medical officer, registrar, house officer, attending, consultant	• Allied health specialties such as Pharmacy, Dietetics, Chiropractic, Midwifery, Podiatry, Speech Therapy, Occupational and Physiotherapy<br>• Non-medical specialties such as Clinical and Translational Science, Alternative and Traditional Medicine, Veterinary, Dentistry
**Intervention**	• portfolios in undergraduate and postgraduate medical education for teaching and assessment of Communication, Ethics and Professionalism	Other documentation methods or learning tools that are:<br>• Not longitudinal or single timepoint

For peer review only - http://bmjopen.bmj.com/site/about/guidelines.xhtml

PICOS	Inclusion criteria	Exclusion criteria
	Criteria of a portfolio:<br>• longitudinal (more than a single timepoint) assessment data<br>• candidate's personal engagement with portfolio content and associated learning<br>• Interventions meeting the above criteria were included regardless of whether they were referred to as portfolios<br><br>All types of portfolios were included in the study:<br>• For instance: electronic & non-electronic; formative & summative or combined; clinical & non-clinical<br>• Portfolios with input from students and/or residents and/or doctors, and/or input from faculty members and other individuals<br>• Portfolios with different structures: extent by which the structure has been prescribed and/or left to individual discretion	• Does not include personal intellectual engagement with the content and associated learning (for instance, curriculum vitae, logbooks and the use of personal digital assistants)
**Comparison/ Context**	NA	NA
**Outcome**	Papers that measured the following outcomes were included:<br>• Effectiveness of the use of portfolios to assess and teach Communication, Ethics and Professionalism<br>• Impact of the use of portfolios on medical students (both undergraduate and postgraduate)<br>• Impact of the use of portfolios on the faculty	NA
**Study design**	• Articles in English or translated to English<br>• Articles published from 1st January 2000 to 31st December 2020<br>• Databases: PsycINFO, EMBASE, PubMed, ERIC, Scopus, Google Scholar<br>• All study designs including:<br>• Mixed methods research, meta-analyses, systematic reviews, randomised controlled trials, cohort studies, case-control studies, cross-sectional studies, descriptive papers, grey literature, opinions, letters, commentaries and editorials	NA

D. Identification of Relevant Studies

The research team developed search strategies and carried out independent reviews for relevant publications in the PubMed, Embase, PsycINFO, ERIC, Scopus, and Google Scholar databases. Keeping with Pham, Rajić (60)'s approach of ensuring a feasible and sustainable research process, the team contained the search to articles

For peer review only - http://bmjopen.bmj.com/site/about/guidelines.xhtml

published between 1st January 2000 and 31st December 2020 to accommodate existing human resource and time constraints and ensure a sustainable review process.

E. Selection of Studies Included in Review

Six members of the research team created individual lists of titles to be included whilst three other members of the research team carried out hand searches and ancestry searches of seven leading journals in medical education (Academic Medicine, Medical Education, Medical Teacher, Advances Health Sciences Education, BMC Medical Education, Teaching and Learning in Medicine and Perspectives on Medical Education) accessed through the NUS library portal. These individual lists of titles were discussed online and Sandelowski and Barroso (61)'s "negotiated consensual validation" approach to "*articulate, defend, and persuade others of the "cogency" or "incisiveness" of their points of view or show their willingness to abandon views that are no longer tenable*" was applied to achieve consensus on the final list of titles to be scrutinised.

F. Assessing the Quality of Articles

Eight research team members individually appraised the quality of the quantitative and qualitative studies using the Medical Education Research Study Quality Instrument (MERSQI) (62) and Consolidated Criteria for Reporting Qualitative Studies (COREQ) (63).

Stage 2 of SEBA: Split Approach

Two teams carried independent and concurrent thematic and content analysis of the included articles whilst a third team created tabulated summaries based on recommendations drawn from Wong, Greenhalgh (64)'s RAMESES publication standards: meta-narrative reviews and Popay, Roberts (65)'s *"Guidance on the conduct of narrative synthesis in systematic reviews"*. The categories employed in the content analysis for undergraduate communications were Rider et al. (66) 's *"A model for communication skills assessment across the undergraduate curriculum",* Goldie (67)'s *"Review of ethics curricula in undergraduate medical education"*, Duffy et al. (68)'s *"Assessing Competence in Communication and Interpersonal Skills: The Kalamazoo II Report"* and Hong et al. (13)'s *"Postgraduate Ethics Training Programs: A Systematic Scoping Review"*. Tay et al. (24)'s *"Assessing Professionalism in Medicine - A Scoping Review of Assessment Tools from 1990 to 2018"* was employed for codes

For peer review only - http://bmjopen.bmj.com/site/about/guidelines.xhtml

for professionalism and David (69)'s article *"AMEE Medical Education Guide No. 24: Portfolios as a method of student assessment"* was then used to contextualise their use in portfolios.

Stage 3 of SEBA: The Jigsaw Perspective

The Jigsaw Perspective sees the themes and categories identified compared and combined where overlaps and similarities exist.

Stage 4 of SEBA: The Funnelling Process

The Funnelling Process sees the themes/categories created from the Jigsaw Approach compared with the tabulated summaries to determine their consistency.

Patient and Public Involvement

Patients or the public were not involved in the design, or conduct, or reporting, or dissemination plans of our research.

RESULTS

A total of 12,300 abstracts were reviewed, 946 full-text articles were evaluated, and 82 articles were analysed (Figure 2). The funnelled domains identified are 1) indications, 2) portfolio content, 3) portfolio design and implementation and 4) strengths and limitations. In keeping with its goals of transparency and accountability, these Tables are enclosed in Appendix B.

(Insert Figure 2. PRISMA Flowchart)

Funneled Domain 1: Indications

CEP portfolios curate longitudinal multisource, multimodal assessment data taken at different settings and contexts allowing it to serve two main roles- teaching and assessment (70, 71) (Table 2). However few span undergraduate and postgraduate education (69, 72, 73), . As assessment tool CEP portfolios assist in the identification of areas of weakness and guide the provision of a comprehensive, targeted feedback, support and remediation (72, 74, 75). Most CEP assessment portfolios in postgraduate education focus on evidencing the attainment of required competencies (76, 77, 78, 79, 80), capturing reflections and indications of professional

For peer review only - http://bmjopen.bmj.com/site/about/guidelines.xhtml

identity formation (78, 81, 82, 83). CEP portfolios in the postgraduate setting have also been used for revalidation purposes (81)

As a teaching tool, CEP portfolios encourage self-directed learning (75, 84, 85, 86), self-improvements (71), reflective practice (72, 73, 75, 79, 81, 85, 86, 87, 88, 89, 90, 91, 92, 93, 94, 95), motivate practitioners to achieve desired competencies (90, 96) and bolster overall professional and personal development (72, 88, 96, 97).

Table 2. Benefits of CEP Teaching and Assessment Portfolios

CEP teaching portfolios	CEP assessment portfolios
**Enhancing learning**<br>Promotion of life-long learning (98, 99)<br><br>Promotion of self-directed learning<br>○ Formulation of learning plans (90, 96, 100)<br>○ Facilitates Outcome-oriented learning (89, 90, 101, 102)<br>○ Enhances self-evaluative capabilities (73, 76, 80, 85, 88, 89, 98, 99, 100, 103, 104, 105)<br>○ Identification of strengths and weaknesses (69, 71, 76, 78, 92, 96, 104, 106, 107, 108, 109)<br>○ Inculcates responsibility and ownership for one's learning (71, 73, 74, 80, 84, 86, 101, 110)<br>○ Sets the pace (99)<br>○ Personalised learning (108, 109)<br><br>**Personal and professional development**<br>Promotes self-understanding (79, 120, 121)<br>○ Understanding feelings and thoughts (100, 108)<br>○ Understanding of one's values (92)<br>○ Understanding activity/experience (69, 88, 122)<br>○ Understanding of how one functions in a team (116)<br>○ Fears and stressors feelings of inadequacy (122, 123)<br><br>Curates evidence of skills (98)<br>○ Skills to compile a portfolio (98, 111, 124, 125, 126)<br>○ Reflective skills (69, 76, 88, 102, 108, 122, 127)<br>○ Clinical skills (99)<br>Organizes information(102, 107)<br>○ Documentation of progress (77, 98, 104, 106)<br>○ Documentation of skills in different settings(71, 106, 109)<br>Cross-sectional view of one's competence (69, 77, 106, 109)	Balance of structure and freeform learning (69, 76, 92, 107, 111, 112)<br><br>Adaptable to goals (77, 113, 114)<br><br>Alignment of learning outcomes and assessment methods (90, 91, 97, 101, 105)<br><br>Allows personalised assessments (77)<br><br>Allows assessment over time (84, 92) and stages of development (69) and that facilitate evaluation of:<br>○ Learning and progress over time (77, 106)<br>○ Learning curve (121)<br>○ In different roles (92, 93)<br>○ In different settings (70, 113)<br>○ Complex behaviours (72, 74, 77, 84, 90, 99, 115, 116)<br>○ Critical thinking (77, 97, 105, 114)<br>○ Outcomes/competencies (107, 115)<br>○ Self-assessment (77, 105, 114)<br>○ Complex skills (113)<br>○ Professionalism (114)<br>○ Cultural competence (114)<br>○ Communication skills (114)<br>○ Clinical ethics (117)<br>○ Problem solving abilities (77, 114)<br>○ Reflective abilities (77, 90, 105, 109)<br><br>Longitudinal data<br>○ Possess good predictive value of subsequent clinical competency (74, 118)<br>○ Enhances validity of portfolio grading (69)<br>○ Guides better decisions (74, 116, 119)<br>○ Provides authentic assessment is a key principle of it (92, 99, 109)<br>○ Provides insights in the various core competencies (98)<br>○ Fair mode of assessment(99) and allows student to defend positions/work (69)<br>○ Provides students with direction and clarity (111, 112)

For peer review only

For peer review only - http://bmjopen.bmj.com/site/about/guidelines.xhtml

**Personalisation**<br>Allows for personalisation (69, 90, 99, 110, 114)<br>○ Personal experiences (69)<br>Choice over the contents (69, 99, 114)	Allows the combination of Summative and Formative assessment (69, 91) and multisource assessments (81, 87, 97, 108) (109) and triangulation (77, 109)<br><br>Facilitates personalised feedback (116) that can be informed by understanding of the individual setting and context (99, 100, 113)
**Benefits to faculty**<br>Improvement of curriculum (83, 92)<br>○ Identify areas of weakness in curriculum (83)<br>○ Provide insights into mentoring, teaching etc (128)<br>Facilitates design of the program (83)<br><br>faculty development<br>○ Understand more about how students acquire competencies (99)<br>○ Insight into the impact of their interventions (119, 128)<br>Insight into their professional conduct, role modelling and pastoral care (121)	
**E-portfolios**<br>Ease of use:<br>○ Easier use (90)<br>○ Accessible (73)<br>Dissemination and editing of information (110)	

Funneled Domain 2: Characteristics

The contents of portfolios were shaped by their overall function (77, 80, 83, 113, 129) (Table 3). Expectations about the nature, and assessments of the contents (83) facilitates personalization of portfolios (73, 77, 104).

Table 3. Portfolio Design and Implementation

Themes	Subthemes	Contents
**Design and Implementation Principles**	**Structure**	Clear guidelines (69, 87, 88, 96, 97, 105, 108)<br>• Use of portfolio (69, 88)<br>• Portfolio content - determining competencies to assess (69, 91)<br>• Promotional decisions (69, 71, 89, 121, 130)<br>Standard-setting exercises (75)<br>Provision of sample portfolios for reference (105, 131, 132)<br>• Roles for assessors (69, 71, 74, 120, 121, 130, 133)<br>• Expectations of students (112)<br>• Duration spanning across preclinical and clinical years (126)<br>Well-defined structure with room for flexibility (69, 86, 88, 90, 105)<br>Student-centered (90)<br>Appropriate student to mentor ratio (71, 125)<br>Sufficient curricular experience for reflection, feedback (71)<br>Protected time for reflection, feedback, and regular meetings with mentors (71, 74, 87, 108)
	**Faculty**	Good faculty support (121, 126)<br>Ensure culture is supportive of portfolio system (71, 126)<br>Flexible and enthusiastic tutors passionate and experienced mentors (85, 88)

For peer review only

	**Curricular Integration**	Early introduction of portfolio (88, 108, 110, 134)<br>Easing portfolio into curriculum (69, 72, 86, 88, 90) – including orientation, training and introductory sessions (69, 72, 88, 90, 105, 121, 132) for learners and staff training (69, 71, 72, 75, 84, 85, 88)<br>Implementation of portfolios throughout curriculum (121)<br>Portfolio content aligning with phase of medical journey and competencies required (69, 71, 90, 97, 130, 132)
	**Assessments**	Clear scoring rubrics (69, 126)<br>Alignment between assessments and outcomes (86, 87, 90)<br>Transparent assessment criteria and outcomes to maintain fairness (71, 72, 110, 131)<br>Standardisation of portfolio use and content (86, 87), assessors training (72) and increased number of assessors (86, 87, 134) to improve assessment reliability (86, 87, 134)<br>Separate processes and reviewers for summative and formative portfolio (71) – summative assessments to specify clear pass/fail requirements (69)<br>Incorporation of student feedback into portfolio development (69, 72, 90)
	**Assessors**	External examiners (69)<br>Faculty members (69, 71, 74, 120, 130, 133)<br>Mentors (107)<br>Senior staff to be paired with new examiners (69)<br>Mentors should not assess own student (86, 92)
	**Assessment Frequency**	Formative assessments to monitor and guide student learning – conducted throughout the year (86, 88, 91)<br>Summative assessments to evaluate learner performance – conducted annually (69, 71, 72, 74, 75, 84, 85, 90, 91, 108, 130, 132, 135, 136, 137, 138), twice (71, 75, 116), thrice (88) or multiple times a year (77, 79)
	**Oversight and Delegation**	Establishment of portfolio oversight committee (71, 75)<br>Regular programme evaluation (71)<br>Clear division of support roles – e.g. pedagogical and technical support (110)

Postgraduate CEP assessment portfolios inject more details upon undergraduate portfolios. Table 4 summarises these elements include communication skills, addressing ethical issues and compliance of professional, institutional and departmental policies (106, 124, 139). Greater detail in postgraduate CEP assessment portfolios sees the addition of modified essay questions (MEQ)s (79), polypharmacy journal/audits (70, 95, 99, 129, 140), professional workshops and training sessions (98, 99, 113, 141, 142), health service meeting reports (79) and reviews by medical regulatory authorities (95). A detailed summary is provided in Appendix C.

Table 4. Portfolio Content (Competencies Assessed and Assessment Modalities)

	Communication	Ethics	Professionalism
Common competencies assessed	1. Values/ethics – appreciation of professional, legal and societal values to guide practice<br>2. Professional responsibilities- appreciation and manifesting these responsibilities		

For peer review only - http://bmjopen.bmj.com/site/about/guidelines.xhtml

	3. Doctor- patient relationships- appreciation of the guiding principles, expectations and ability to nurture these relationships<br>4. Interprofessional team working<br>5. Ethical and legal responsibilities<br>6. Continuous learning and quality improvement		
Specific competencies	1. Active listening<br>2. Clear explanations<br>3. Empathetic communication<br>4. History taking<br>5. Breaking bad news<br>6. Conflict management<br>7. Shared decision making<br>8. Written communication	1. Professionalism<br>2. Respect<br>3. Privacy<br>4. Identifying key ethical issues<br>5. Balancing competing ethical responsibilities<br>6. Consent	1. Interpersonal communication
Reflection	1. Content<br>2. Context<br>3. Depth<br>4. Authenticity<br>5. Developmental areas<br>6. Dilemma and doubts		
Assessment (formative/ summative/ mixed)	MCQ<br>Essays<br>OSCE<br>Mini-CEX<br>Case presentations<br>Case based discussion<br>Simulated patient assessment<br>Work based assessment<br>Team based assessment<br>Peer evaluation<br>Self-evaluation<br>Faculty evaluations<br>Feedback<br>Reflections<br>Letters of commendations<br>Learning and or improvement plans<br>Curriculum Vitae<br>Logbook review/interview		

Funneled Domain 3: Strengths and Limitations

The strengths and limitations of portfolios are outlined in Table 5.

Table 5. Strengths and Limitations

Themes	Subthemes	Content
**Strengths/ Limitations**	**Strengths**	For students<br>Identification of own strengths and weaknesses (69, 74, 75, 85, 86, 87, 88, 90, 92, 96, 100, 138)<br>Appreciation of effective communication (88, 134, 143), ethical issues (92), and fosters critical professional attitudes (87, 144)<br>Encourages self-regulation (138) through opportunities for reflection (86) and self-assessment (88, 108, 121)<br>Facilitates preparation for post-graduate medical training (88, 107, 111)<br><br>For faculty<br>Identification of areas of concern and allows for early, timely intervention (74, 75)

For peer review only

		Enhances interaction between students and teachers (69, 88, 92) Facilitates reiterative reassessment of teaching strategies (69)<br><br>Assessments<br>Longitudinal (116)<br>Good validity (74)<br>Reliable assessment tool – allows for triangulation of information and evidence from multiple sources and contexts (71)<br>Ability to assess a wide range of competencies (90, 97)<br>Allows for more authentic assessments (74)<br>Gives faculty greater insight into students' achievement of behaviourally-oriented competencies that traditional methods do not (75)<br>Evaluates achievement during clinical attachment/clerkship (97)<br>Provides a basis for the judgement of a student's professional fitness to practice (72)
	**Limitations**	Time-consuming and labour intensive (72, 75, 85, 88, 89, 90, 91, 96, 107, 126, 134)<br>Issues with reliability (86, 90, 120, 134)<br>Inconsistent effects (86, 90, 108, 145)<br>Lack of honesty by students in reflections (71, 72, 86, 90, 91, 107, 108, 112)

Postgraduate portfolios provide greater insights into lifelong learning and continuous professional development (77, 80, 81, 83, 98, 99, 106, 109, 113, 141). Use of multi-source assessments at multiple timepoints (80) in clinical settings (81, 99) see postgraduate portfolios provide good inter-rater reliability (77, 109). Postgraduate portfolios also detect stress and burnout (141).

Stage 5 of SEBA: Analysis of data and non-data driven literature

Findings from the different stages of SEBA were discussed with members of the expert team and relevant stakeholders. There were concerns from the expert team about the impact of grey literature on the narrative, given that they were neither peer-reviewed nor necessarily evidence-based. As a result, the research team differentiated correspondence, letters, editorials and perspective pieces from academic databases and grey literature from data-driven or research-based peer-reviewed data. Both groups were then independently analysed. The themes/categories identified were then compared to enhance further the accountability and the reproducibility of Stage 5 of SEBA. Evidence-based data from bibliographic databases (henceforth evidence-based publications) were separated from grey literature, perspectives, editorials, letters, and non-data-based articles drawn from bibliographic databases (henceforth non-data driven). These two groups were separately thematically analysed.

Stage 6 of SEBA: Synthesis of the Discussion

The Best Evidence Medical Education (BEME) Collaboration Guide (146) and the Structured approach to the Reporting In healthcare education of Evidence Synthesis (STORIES) (147) were used to guide the discussion.

For peer review only - http://bmjopen.bmj.com/site/about/guidelines.xhtml

DISCUSSION

This SSR in SEBA suggest that overlaps in ethics, professionalism, and communication skills, knowledge, attitudes and competencies reaffirm the concurrent training, and assessments of CEP competencies in portfolios (72, 74, 75). To be effective CEP teaching and longitudinal assessment portfolios require a consistent framework replete with clearly delineated goals (105), aligned expectations, predetermined assessment criteria for specified competencies (69, 71, 72, 77, 83, 85, 87, 96, 97, 98, 105, 110, 120, 121, 124, 132, 133, 139), agreed upon endpoints and outcome measures, and the curation, structuring and organisation of longitudinal multisource, multimodal assessment data (70, 71). The combination of this data facilitate training, assessment, feedback and holistic and longitudinal support (75, 84, 85, 86) (101) (106, 124, 139, 148), guide reflection (72, 73, 75, 79, 81, 85, 86, 87, 88, 89, 90, 91, 92, 93, 94, 95), and bolster professional and personal development (72, 88, 96, 97). Critically the employ of consistent and agreed upon structure, goals, purpose, role, learning objectives, content, and assessment guidelines also affords users with a personalised perspective of their CEP development upon which they can reflect on and glean insights and feedback (86, 102, 131).

Yet to be effective, CEP portfolios require policing and underscores the need for trained (70) and dedicated faculty (88) capable of effective role modelling (121), support (69, 79, 88, 92, 97) and providing feedback (116) and who appreciate the individual's current concepts, narratives, motivations, abilities and availabilities and contextual considerations. Furthermore, the efficacy of portfolios and their ability to meet the stated objectives stated in Table 2 and realise their stated benefits in Table 5 are reliant upon the appropriate mix of assessment methods employed, the frequency of assessments, the efficacy of analysis and the quality of the feedback, support and remediation provided should it be required. This in turn highlights the role of the host organization in ensuring effective oversight of the program and assessments and in ensuring that faculty are afforded protected time for administrative duties, coordination and assessments particularly given the longitudinal nature of CEP portfolios (77, 90, 105, 106, 122, 140, 149, 150). It should not be forgotten that CEP portfolios reflect the development of an individual and such change should be measured and contextualised within the wider education, training, practice, professional, research, clinical, interprofessional team based and organizational perspective. This further underlines the importance of the host organizations role in assessing and guiding development of CEP competencies.

For peer review only - http://bmjopen.bmj.com/site/about/guidelines.xhtml

With these requisites met, evidence of changes in CEP knowledge, skills and competencies suggests shift the thinking, attitudes and conduct of clinicians. The Krishna-Pisupati model posits that sustained changes in practice, beliefs, values and principles will result in changes in the clinician's belief systems which in turn inform their self-concepts of identity and shape their PIF.

LIMITATIONS

The generalisability of the results of this study is limited by the identified studies selected for review. Firstly, the majority of the papers included originate from Europe and North America. Given that CEP is a sociocultural construct, these geopolitical sociocultural differences raise questions as to the applicability of these findings beyond the European and North American medical education systems. Secondly, as the number of articles included is limited, the SSR in SEBA's ability to assess the long-term effectiveness of portfolios may be compromised.

CONCLUSIONS

The suggestion that CEP portfolios can capture, instruct, and assess PIF requires further study. Whilst there have been posits on how such evaluations could be made, such tools remain unrealised and must be the focus of coming studies. One possible starting point for the design of such tools may be theories such as the Krishna-Pisupati model which attempts to link PIF to changes to belief systems and concepts of personhood. This may provide a grander view of PIF that spans the undergraduate and postgraduate settings and provide the basis for directing support and feedback for clinicians. As we look forward to furthering efforts to study the effects of CEP portfolios upon the PIF of clinicians, we also believe the specific impact of these potentially resource-heavy and financially costly intervention should be at the centre of future initiatives.

ABBREVIATIONS

**CEP**	Communication, Ethics, Professionalism
**PIF**	Professional Identity Formation
**SSR**	Systematic Scoping Review
**SEBA**	Systematic Evidence Based Approach
**PICOS**	Population, Intervention, Comparison and Outcome
**BEME**	Best Evidence Medical Education

For peer review only - http://bmjopen.bmj.com/site/about/guidelines.xhtml
For peer review only

STORIES Structured approach to the Reporting In healthcare education of Evidence Synthesis

STATEMENTS AND DECLARATIONS

Ethics Approval and Consent to Participate NA

Consent for Publication NA

Availability of Data and Materials All data generated or analysed during this review are included in this published article and its appendices.

Competing Interests JJQT, GP, DZH, BKYL, AJSL, EJXC, AP, RT, JYHY, YZK, CWNQ, JYL, KTT, YTO, MC, JZ, SM, LW and LKRK have no competing interests.

Funding No funding was received for this review

Author Contributions JJQT, GP, DZH, BKYL, AJSL, EJXC, AP, RT, JYHY, YZK, CWNQ, JYL, KTT, YTO, MC, JZ, SM, LW and LKRK were involved in data curation, formal analysis, investigation, preparing the original draft of the manuscript as well as reviewing and editing the manuscript. All authors have read and approved the manuscript.

ACKNOWLEDGEMENTS

The authors would like to dedicate this paper to the late Dr. S Radha Krishna and A/Prof Cynthia Goh whose advice and ideas were integral to the success of this review and to Thondy, Maia Olivia and Raja Kamarul Ariffin whose lives continue to inspire us. The authors would also like to thank the anonymous reviewers, Dr Ruaraidh Hill and Dr Stephen Mason for their help comments which greatly enhanced this manuscript.

For peer review only

For peer review only

For peer review only - http://bmjopen.bmj.com/site/about/guidelines.xhtml

REFERENCES

1. Rankings TU. QS World University Rankings 2020 [Available from: https://www.topuniversities.com/university-rankings/university-subject-rankings/2020/medicine.

2. Deshpande AD. The Use of Sociocultural Constructs in Cancer Screening Research Among African Americans. Cancer Control. 2009;16(3):10.

3. MZJ H. Chinese Familial Tradition and Western Influences: A Case Study in Singapore on Decision Making at the end of life. Journal of Pain and Symptom Management. 2010;40(6):6.

4. Foo WT, Zheng Y, Kwee AK, Yang GM, Krishna L. Factors considered in end-of-life care decision making by health care professionals. Am J Hosp Palliat Care. 2013;30(4):354-8.

5. Chong JA, Quah YL, Yang GM, Menon S, Radha Krishna LK. Patient and family involvement in decision making for management of cancer patients at a centre in Singapore. BMJ Support Palliat Care. 2015;5(4):420-6.

6. Chai HZ, Krishna LK, Wong VH. Feeding: what it means to patients and caregivers and how these views influence Singaporean Chinese caregivers' decisions to continue feeding at the end of life. Am J Hosp Palliat Care. 2014;31(2):166-71.

7. Marc Ho ZJ, Krishna LKR, Goh C, Alethea Yee CP. The physician–patient relationship in treatment decision making at the end of life: A pilot study of cancer patients in a Southeast Asian society. Palliative and Supportive Care. 2013;11(1):13-9.

8. Krishna LKR. Decision making at the end of life: A Singaporean Perspective. Asian Bioeth Rev. 2011;3(2):6.

9. Loh AZ, Tan JS, Jinxuan T, Lyn TY, Krishna LK, Goh CR. Place of Care at End of Life: What Factors Are Associated With Patients' and Their Family Members' Preferences? Am J Hosp Palliat Care. 2016;33(7):669-77.

10. Krishna LK, Watkinson DS, Beng NL. Limits to relational autonomy--the Singaporean experience. Nurs Ethics. 2015;22(3):331-40.

11. Krishna LK, Alsuwaigh R, Miti PT, Wei SS, Ling KH, Manoharan D. The influence of the family in conceptions of personhood in the palliative care setting in Singapore and its influence upon decision making. Am J Hosp Palliat Care. 2014;31(6):645-54.

12. Krishna L. The position of the family of palliative care patients within the decision-making process at the end of life in Singapore. Ethics & Medicine: An International Journal of Bioethics. 2011;27:183+.

13. Hong DZ, Goh JL, Ong ZY, Ting JJQ, Wong MK, Wu J, et al. Postgraduate ethics training programs: a systematic scoping review. BMC Med Educ. 2021;21(1):338.

14. Wong MK, Hong DZH, Wu J, Ting JJQ, Goh JL, Ong ZY, et al. A systematic scoping review of undergraduate medical ethics education programs from 1990 to 2020. Med Teach. 2022;44(2):167-86.

15. Yoon NYS, Ong YT, Yap HW, Tay KT, Lim EG, Cheong CWS, et al. Evaluating assessment tools of the quality of clinical ethics consultations: a systematic scoping review from 1992 to 2019. BMC Med Ethics. 2020;21(1):51.

16. Bok C, Ng CH, Koh JWH, Ong ZH, Ghazali HZB, Tan LHE, et al. Interprofessional communication (IPC) for medical students: a scoping review. BMC Med Educ. 2020;20(1):372.

17. Chia E, Huang H, Goh S, Peries M, Lee C, Tan L, et al. A systematic scoping review of teaching and evaluating communications in the intensive care unit. The Asia Pacific Scholar. 2021;6:3-29.

For peer review only - http://bmjopen.bmj.com/site/about/guidelines.xhtml

18. Malhotra C, Kanesvaran R, Krishna L, Xiang L, Kumarakulasinghe NB, Tan SH, et al. Oncologists' responses to patient and caregiver negative emotions and patient perception of quality of communication: results from a multi-ethnic Asian setting. Support Care Cancer. 2018;26(3):957-65.
19. Ng CH, Ong ZH, Koh JWH, Ang RZE, Tan LHS, Tay KT, et al. Enhancing Interprofessional Communications Training in Internal Medicine. Lessons Drawn From a Systematic Scoping Review From 2000 to 2018. J Contin Educ Health Prof. 2020;40(1):27-35.
20. Ong ZH, Tan LHE, Ghazali HZB, Ong YT, Koh JWH, Ang RZE, et al. A Systematic Scoping Review on Pedagogical Strategies of Interprofessional Communication for Physicians in Emergency Medicine. J Med Educ Curric Dev. 2021;8:23821205211041794.
21. Tan XH, Foo MA, Lim SLH, Lim M, Chin AMC, Zhou J, et al. Teaching and assessing communication skills in the postgraduate medical setting: a systematic scoping review. BMC Med Educ. 2021;21(1):483.
22. Venktaramana V, Loh EKY, Wong CJW, Yeo JW, Teo AYT, Chiam CSY, et al. A systematic scoping review of communication skills training in medical schools between 2000 and 2020. Med Teach. 2022;44(9):997-1006.
23. Ong YT, Kow CS, Teo YH, Tan LHE, Abdurrahman A, Quek NWS, et al. Nurturing professionalism in medical schools. A systematic scoping review of training curricula between 1990-2019. Med Teach. 2020;42(6):636-49.
24. Tay KT, Ng S, Hee JM, Chia EWY, Vythilingam D, Ong YT, et al. Assessing Professionalism in Medicine - A Scoping Review of Assessment Tools from 1990 to 2018. J Med Educ Curric Dev. 2020;7:2382120520955159.
25. Teo YH, Peh TY, Abdurrahman A, Lee ASI, Chiam M, Fong W, et al. A modified Delphi approach to enhance nurturing of professionalism in postgraduate medical education in Singapore. Singapore Med J. 2021.
26. The Student-Physician: Introductory Studies in the Sociology of Medical Education. Journal of the American Medical Association. 1958;166(1):106-7.
27. Teo KJH, Teo MYK, Pisupati A, Ong RSR, Goh CK, Seah CHX, et al. Assessing professional identity formation (PIF) amongst medical students in Oncology and Palliative Medicine postings: a SEBA guided scoping review. BMC Palliat Care. 2022;21(1):200.
28. Quah ELY, Chua KZY, Lua JK, Wan DWJ, Chong CS, Lim YX, et al. A Systematic Review of Stakeholder Perspectives of Dignity and Assisted Dying. J Pain Symptom Manage. 2022.
29. Venktaramana V. Understanding Mentoring Relationships between Mentees, Peer and Senior Mentors. BMC Med Educ. 2023.
30. Toh RQE, Koh KK, Lua JK, Wong RSM, Quah ELY, Panda A, et al. The role of mentoring, supervision, coaching, teaching and instruction on professional identity formation: a systematic scoping review. BMC Med Educ. 2022;22(1):531.
31. Ho CY, Kow CS, Chia CHJ, Low JY, Lai YHM, Lauw SK, et al. The impact of death and dying on the personhood of medical students: a systematic scoping review. BMC Med Educ. 2020;20(1):516.
32. Sarraf-Yazdi S, Teo YN, How AEH, Teo YH, Goh S, Kow CS, et al. A Scoping Review of Professional Identity Formation in Undergraduate Medical Education. J Gen Intern Med. 2021;36(11):3511-21.
33. Vig PS, Lim JY, Lee RWL, Huang H, Tan XH, Lim WQ, et al. Parental bereavement - impact of death of neonates and children under 12 years on personhood of parents: a systematic scoping review. BMC Palliat Care. 2021;20(1):136.

For peer review only - http://bmjopen.bmj.com/site/about/guidelines.xhtml

34. Chan NPX, Chia JL, Ho CY, Ngiam LXL, Kuek JTY, Ahmad Kamal NHB, et al. Extending the Ring Theory of Personhood to the Care of Dying Patients in Intensive Care Units. Asian Bioeth Rev. 2022;14(1):71-86.
35. Ngiam LXL, Ong YT, Ng JX, Kuek JTY, Chia JL, Chan NPX, et al. Impact of Caring for Terminally Ill Children on Physicians: A Systematic Scoping Review. Am J Hosp Palliat Care. 2021;38(4):396-418.
36. Chia EWY, Tay KT, Xiao S, Teo YH, Ong YT, Chiam M, et al. The Pivotal Role of Host Organizations in Enhancing Mentoring in Internal Medicine: A Scoping Review. J Med Educ Curric Dev. 2020;7:2382120520956647.
37. Kuek JTY, Ngiam LXL, Kamal NHA, Chia JL, Chan NPX, Abdurrahman A, et al. The impact of caring for dying patients in intensive care units on a physician's personhood: a systematic scoping review. Philos Ethics Humanit Med. 2020;15(1):12.
38. Zhou YC, Tan SR, Tan CGH, Ng MSP, Lim KH, Tan LHE, et al. A systematic scoping review of approaches to teaching and assessing empathy in medicine. BMC Med Educ. 2021;21(1):292.
39. Tay KT, Tan XH, Tan LHE, Vythilingam D, Chin AMC, Loh V, et al. A systematic scoping review and thematic analysis of interprofessional mentoring in medicine from 2000 to 2019. J Interprof Care. 2021;35(6):927-39.
40. Tan R, Qi Ting JJ, Zhihao Hong D, Sing Lim AJ, Ong YT, Pisupati A, et al. Medical Student Portfolios: A Systematic Scoping Review. J Med Educ Curric Dev. 2022;9:23821205221076022.
41. Kow CS, Teo YH, Teo YN, Chua KZY, Quah ELY, Kamal N, et al. A systematic scoping review of ethical issues in mentoring in medical schools. BMC Med Educ. 2020;20(1):246.
42. Huang H, Toh RQE, Chiang CLL, Thenpandiyan AA, Vig PS, Lee RWL, et al. Impact of Dying Neonates on Doctors' and Nurses' Personhood: A Systematic Scoping Review. J Pain Symptom Manage. 2022;63(1):e59-e74.
43. Hong DZ, Lim AJS, Tan R, Ong YT, Pisupati A, Chong EJX, et al. A Systematic Scoping Review on Portfolios of Medical Educators. J Med Educ Curric Dev. 2021;8:23821205211000356.
44. Ho S, Tan YY, Neo SHS, Zhuang Q, Chiam M, Zhou JX, et al. COVID-19 - A Review of the Impact it has made on Supportive and Palliative Care Services Within a Tertiary Hospital and Cancer Centre in Singapore. Ann Acad Med Singap. 2020;49(7):489-95.
45. Hee J, Toh YL, Yap HW, Toh YP, Kanesvaran R, Mason S, et al. The development and design of a framework to match mentees and mentors through a systematic review and thematic analysis of mentoring programs between 2000 and 2015. Mentoring & Tutoring: Partnership in Learning. 2020;28:340-64.
46. Chua WJ, Cheong CWS, Lee FQH, Koh EYH, Toh YP, Mason S, et al. Structuring Mentoring in Medicine and Surgery. A Systematic Scoping Review of Mentoring Programs Between 2000 and 2019. J Contin Educ Health Prof. 2020;40(3):158-68.
47. Chua KZY, Quah ELY, Lim YX, Goh CK, Lim J, Wan DWJ, et al. A systematic scoping review on patients' perceptions of dignity. BMC Palliat Care. 2022;21(1):118.
48. Quek CWN, Ong RRS, Wong RSM, Chan SWK, Chok AK-L, Shen GS, et al. Systematic scoping review on moral distress among physicians. BMJ Open. 2022;12(9):e064029.
49. Ong RSR, Wong RSM, Chee RCH, Quek CWN, Burla N, Loh CYL, et al. A systematic scoping review moral distress amongst medical students. BMC Med Educ. 2022;22(1):466.
50. Yang LH, Kleinman A, Link BG, Phelan JC, Lee S, Good B. Culture and stigma: adding moral experience to stigma theory. Soc Sci Med. 2007;64(7):1524-35.

For peer review only - http://bmjopen.bmj.com/site/about/guidelines.xhtml

51. Krishna LKR, Tan LHE, Ong YT, Tay KT, Hee JM, Chiam M, et al. Enhancing Mentoring in Palliative Care: An Evidence Based Mentoring Framework. Journal of Medical Education and Curricular Development. 2020;7:2382120520957649.
52. Ong YT, Quek CWN, Pisupati A, Loh EKY, Venktaramana V, Chiam M, et al. Mentoring future mentors in undergraduate medical education. PLoS One. 2022;17(9):e0273358.
53. Lim JY, Ong SYK, Ng CYH, Chan KLE, Wu S, So WZ, et al. A systematic scoping review of reflective writing in medical education. BMC Med Educ. 2023;23(1):12.
54. Kamal NHA, Tan LHE, Wong RSM, Ong RRS, Seow REW, Loh EKY, et al. Enhancing education in Palliative Medicine: the role of Systematic Scoping Reviews. Palliative Medicine & Care: Open Access. 2020;7(1):1-11.
55. Ong RRS, Seow REW, Wong RSM, Loh EKY, Kamal NHA, Mah ZH, et al. A Systematic Scoping Review of Narrative Reviews in Palliative Medicine Education. Palliative Medicine & Care: Open Access. 2020;7(1):1-22.
56. Mah ZH, Wong RSM, Seow REW, Loh EKY, Kamal NHA, Ong RRS, et al. A Systematic Scoping Review of Systematic Reviews in Palliative Medicine Education. Palliative Medicine & Care: Open Access. 2020;7(1):1-12.
57. Cleland J, McKimm J, Fuller R, Taylor D, Janczukowicz J, Gibbs T. Adapting to the impact of COVID-19: Sharing stories, sharing practice. Medical Teacher. 2020;42(7):772-5.
58. Peters M, Godfrey C, McInerney P, Soares C, Khalil H, Parker D. The Joanna Briggs Institute reviewers' manual 2015: methodology for JBI scoping reviews2015 April 29, 2019. Available from: http://joannabriggs.org/assets/docs/sumari/Reviewers-Manual_Methodology-for-JBI-Scoping-Reviews_2015_v1.pdf.
59. Peters MD, Godfrey CM, Khalil H, McInerney P, Parker D, Soares CB. Guidance for conducting systematic scoping reviews. Int J Evid Based Healthc. 2015;13(3):141-6.
60. Pham MT, Rajić A, Greig JD, Sargeant JM, Papadopoulos A, McEwen SA. A scoping review of scoping reviews: advancing the approach and enhancing the consistency. Res Synth Methods. 2014;5(4):371-85.
61. Sandelowski M, Barroso J. Handbook for synthesizing qualitative research: springer publishing company; 2006.
62. Reed DA, Beckman TJ, Wright SM, Levine RB, Kern DE, Cook DA. Predictive validity evidence for medical education research study quality instrument scores: quality of submissions to JGIM's Medical Education Special Issue. J Gen Intern Med. 2008;23(7):903-7.
63. Tong A, Sainsbury P, Craig J. Consolidated criteria for reporting qualitative research (COREQ): a 32-item checklist for interviews and focus groups. Int J Qual Health Care. 2007;19(6):349-57.
64. Wong G, Greenhalgh T, Westhorp G, Buckingham J, Pawson R. RAMESES publication standards: meta-narrative reviews. BMC medicine. 2013;11(1):20.
65. Popay J, Roberts H, Sowden A, Petticrew M, Arai L, Rodgers M, et al. Guidance on the conduct of narrative synthesis in systematic reviews. A product from the ESRC methods programme Version. 2006;1:b92.
66. Rider EA, Hinrichs MM, Lown BA. A model for communication skills assessment across the undergraduate curriculum. Med Teach. 2006;28(5):e127-34.
67. Goldie J. Review of ethics curricula in undergraduate medical education. Med Educ. 2000;34(2):108-19.
68. Duffy FD, Gordon GH, Whelan G, Cole-Kelly K, Frankel R, Buffone N, et al. Assessing competence in communication and interpersonal skills: the Kalamazoo II report. Acad Med. 2004;79(6):495-507.

For peer review only - http://bmjopen.bmj.com/site/about/guidelines.xhtml

69. David M, Davis M, Harden R, Howie P, Ker J, Pippard M. AMEE Medical Education Guide No. 24: Portfolios as a method of student assessment. Medical teacher. 2001;23:535-51.
70. McEwen L, Griffiths J, Schultz K. Developing and Successfully Implementing a Competency-Based Portfolio Assessment System in a Postgraduate Family Medicine Residency Program. Academic medicine : journal of the Association of American Medical Colleges. 2015;90.
71. Dannefer E, Henson L. The Portfolio Approach to Competency-Based Assessment at the Cleveland Clinic Lerner College of Medicine. Academic medicine : journal of the Association of American Medical Colleges. 2007;82:493-502.
72. Davis M, Ben-David M, Harden R, Howie P, Ker J, McGhee C, et al. Portfolio assessment in medical students' final examination. Medical teacher. 2001;23:357-66.
73. Ruiz J, Qadri S, Karides M, Castillo C, Milanez M, Roos B. Fellows' Perceptions of a Mandatory Reflective Electronic Portfolio in a Geriatric Medicine Fellowship Program. Educational Gerontology - EDUC GERONTOL. 2009;35:634-52.
74. O'Brien C, Thomas J, Green M. What Is the Relationship Between a Preclerkship Portfolio Review and Later Performance in Clerkships? Academic medicine : journal of the Association of American Medical Colleges. 2017;93.
75. O'Brien C, Sanguino S, Thomas J, Green M. Feasibility and Outcomes of Implementing a Portfolio Assessment System Alongside a Traditional Grading System. Academic medicine : journal of the Association of American Medical Colleges. 2016;91.
76. Jenkins L, Mash B, Derese A. The national portfolio of learning for postgraduate family medicine training in South Africa: Experiences of registrars and supervisors in clinical practice. BMC medical education. 2013;13:149.
77. Shumway J, Harden R. AMEE Guide No. 25: The assessment of learning outcomes for the competent and reflective physician. Medical teacher. 2003;25:569-84.
78. Daboval T, Moore G, Ferretti E. How we teach ethics and communication during a Canadian neonatal perinatal medicine residency: An interactive experience. Medical teacher. 2012;35.
79. Jenkins L, Mash B, Derese A. Development of a portfolio of learning for postgraduate family medicine training in South Africa: A Delphi study. BMC family practice. 2012;13:11.
80. Colbert C, Ownby A, Butler P. A Review of Portfolio Use in Residency Programs and Considerations before Implementation. Teaching and learning in medicine. 2008;20:340-5.
81. Wakeling J, Holmes S, Boyd A, Tredinnick-Rowe J, Cameron N, Marshall M, et al. Reflective Practice for Patient Benefit: An Analysis of Doctors' Appraisal Portfolios in Scotland. Journal of Continuing Education in the Health Professions. 2019;39:1.
82. Torbeck L. A Method for Defining Competency-Based Promotion Criteria for Family Medicine Residents. Academic medicine : journal of the Association of American Medical Colleges. 2005;80:832-9.
83. O'Sullivan P, Reckase M, McClain T, Savidge M, Clardy J. Demonstration of Portfolios to Assess Competency of Residents. Advances in health sciences education : theory and practice. 2004;9:309-23.
84. Yielder J, Moir F. Assessing the Development of Medical Students' Personal and Professional Skills by Portfolio. Journal of Medical Education and Curricular Development. 2016;2016:9.
85. O'Sullivan A, Howe A, Miles S, Harris P, Hughes C, Jones P, et al. Does a summative portfolio foster the development of capabilities such as reflective practice and

For peer review only - http://bmjopen.bmj.com/site/about/guidelines.xhtml

understanding ethics? An evaluation from two medical schools. Medical teacher. 2012;34:e21-8.
86. Driessen E, Tartwijk J, Vermunt J, Van der Vleuten C. Use of Portfolios in early undergraduate medical training. Medical teacher. 2003;25:18-23.
87. Haffling A-C, Beckman A, Pahlmblad A, Edgren G. Students' reflections in a portfolio pilot: Highlighting professional issues. Medical teacher. 2010;32:e532-40.
88. Fida N, Hassanien M, Shamim M, Alafari R, Zaini R, Mufti S, et al. Students' perception of portfolio as a learning tool at King Abdulaziz University Medical School. Medical Teacher. 2018;40:1-10.
89. Goldie J, Dowie A, Cotton P, Morrison J. Teaching professionalism in the early years of a medical curriculum: A qualitative study. Medical education. 2007;41:610-7.
90. O'Sullivan A, Harris P, Hughes C, Toohey S, Balasooriya C, Velan G, et al. Linking assessment to undergraduate student capabilities through portfolio examination. Assessment & Evaluation in Higher Education. 2012;37:379-91.
91. Franco R, Franco C, Pestana O, Severo M, Ferreira M. The use of portfolios to foster professionalism: attributes, outcomes, and recommendations. Assessment & Evaluation in Higher Education. 2016;42:1-19.
92. Gordon J. Assessing students' personal and professional development using portfolios and interviews. Medical education. 2003;37:335-40.
93. Wong A, Trollope-Kumar K. Reflections: An inquiry into medical students' professional identity formation. Medical education. 2014;48:489-501.
94. Borgstrom E, Cohn S, Barclay S. Medical Professionalism: Conflicting Values for Tomorrow's Doctors. Journal of general internal medicine. 2010;25:1330-6.
95. Lockyer J, Sockalingam S, Campbell DC. Assessment and Change: An Exploration of Documented Assessment Activities and Outcomes by Canadian Psychiatrists. Journal of Continuing Education in the Health Professions. 2018;38:1.
96. Sambandam E, Jutti R, Lee L. Portfolio as a learning tool: Students' perspective. Annals of the Academy of Medicine, Singapore. 2005;34:511-4.
97. Michels N, Avonts M, Peeraer G, Ulenaers K, Gaal L, Bossaert L, et al. Content validity of workplace-based portfolios: A multi-centre study. Medical Teacher. 2016;38:1-10.
98. Finlay K, Probyn L, Ho S. The CanMEDS Resume: A Useful Educational Portfolio Tool for Diagnostic Radiology Residents. Canadian Association of Radiologists journal = Journal l'Association canadienne des radiologistes. 2011;63:233-6.
99. Clay A, Petrusa E, Harker M, Andolsek K. Development of a web-based, specialty specific portfolio. Medical teacher. 2007;29:311-6.
100. Ozcakar N, Mevsim V, Dilek G. Use of Portfolios in Undergraduate Medical Training: First Meeting With a Patient. Trakya Universitesi Tip Fakultesi Dergisi. 2009;26.
101. Bhattarai M. Study skills course in medical education for postgraduate residents. Kathmandu University medical journal (KUMJ). 2006;5:561-5.
102. Amsellem-Ouazana D, Pee D, Godin V. Use of portfolios as a learning and assessment tool in a surgical practical session of urology during undergraduate medical training. Medical teacher. 2006;28:356-9.
103. Watling C, Brown J. Education Research: Communication skills for neurology residents: Structured teaching and reflective practice. Neurology. 2007;69:E20-6.
104. Goliath C. Diffusion of an E-Portfolio to Assist in the Self-Directed Learning of Physicians: An Exploratory Study. 2009.

For peer review only - http://bmjopen.bmj.com/site/about/guidelines.xhtml

105. Azer S. Use of Portfolios by Medical Students: Significance of Critical Thinking. The Kaohsiung journal of medical sciences. 2008;24:361-6.
106. Cheung R. ECLIPPx: An innovative model for reflective portfolios in life-long learning. The clinical teacher. 2011;8:27-30.
107. Rees C, Shepherd M, Chamberlain S. The utility of reflective portfolios as a method of assessing first year medical students' personal and professional development. Reflective Practice. 2005;6:3-14.
108. Driessen E, Tartwijk J, Overeem K, Vermunt J, Van der Vleuten C. Conditions for reflective use of portfolios in undergraduate medical education. Medical education. 2006;39:1230-5.
109. Gavriel J. Portfolios and 'the competent clinician': Past, present and future assessment. Education for Primary Care. 2016;27:1-4.
110. Moores A, Parks M. Twelve tips for introducing E-Portfolios with undergraduate students. Medical teacher. 2010;32:46-9.
111. Rees C, Sheard C. Undergraduate medical students' views about a reflective portfolio assessment of their communication skills. Medical education. 2004;38:125-8.
112. Chaffey L, de Leeuw E, Finnigan G. Facilitating Students' Reflective Practice in a Medical Course: Literature Review. Education for health (Abingdon, England). 2011;25:198-203.
113. Pinsky L, Edwards K. Diving for PERLS: Working and performance portfolios for evaluation and reflection on learning. Journal of general internal medicine. 2004;19:582-7.
114. Gibson C, Chandratilake M, Hull A. Use of portfolios for assessment of global health residents: qualitative evaluation of design and implementation. Canadian medical education journal. 2018;9:e20-e32.
115. Roberts C, Shadbolt N, Clark T, Simpson P. The reliability and validity of a portfolio designed as a programmatic assessment of performance in an integrated clinical placement. BMC Medical Education. 2014;14:197.
116. Dolan B, O'Brien C, Cameron K, Green M. A Qualitative Analysis of Narrative Preclerkship Assessment Data to Evaluate Teamwork Skills. Teaching and Learning in Medicine. 2018;30:1-9.
117. Fins J, Kodish E, Cohn F, Danis M, Derse A, Dubler N, et al. A Pilot Evaluation of Portfolios for Quality Attestation of Clinical Ethics Consultants. The American journal of bioethics : AJOB. 2016;16:15-24.
118. Naidu T, Ramlall S. Establishing psychiatric registrars' competence in psychotherapy: A portfolio based model. African journal of psychiatry. 2008;11:264-71.
119. Murphy D, Aitchison P, Hernandez Santiago V, Davey P, Mires G, Nathwani D. Insightful Practice: A robust measure of medical students' professional response to feedback on their performance. BMC Medical Education. 2015;15.
120. Ekayanti F, Risahmawati R, Fadhilah M. Portfolio Assessment Implementation in Clinical Year of Community Medicine Module: Students Perspective2017.
121. Hall P, Byszewski A, Sutherland S, Stodel E. Developing a Sustainable Electronic Portfolio (ePortfolio) Program That Fosters Reflective Practice and Incorporates CanMEDS Competencies Into the Undergraduate Medical Curriculum. Academic medicine : journal of the Association of American Medical Colleges. 2012;87:744-51.
122. Menon V. Portfolios for post graduate psychiatry residents: Potentials and pitfalls. Asian Journal of Psychiatry. 2014;11.

For peer review only

For peer review only - http://bmjopen.bmj.com/site/about/guidelines.xhtml

123. Prayson R, Bierer SB, Dannefer E. Medical student resilience strategies: A content analysis of medical students' portfolios. Perspectives on medical education. 2016;6.
124. Fragneto R, Dilorenzo A, Schell R, Bowe E. Evaluating Practice-Based Learning and Improvement: Efforts to Improve Acceptance of Portfolios. Journal of graduate medical education. 2010;2:638-43.
125. Austin C, Braidman I. Support for portfolio in the initial years of the undergraduate medical school curriculum: What do the tutors think? Medical teacher. 2008;30:265-71.
126. Arntfield S, Parlett B, Meston C, Apramian T, Lingard L. A model of engagement in reflective writing-based portfolios: Interactions between points of vulnerability and acts of adaptability. Medical teacher. 2015;38:1-10.
127. Pitkälä K, Mäntyranta T. Feelings related to first patient experiences in medical school: A qualitative study on students' personal portfolios. Patient education and counseling. 2004;54:171-7.
128. Tigelaar D, Dolmans D, Grave W, Wolfhagen I, Van der Vleuten C. Portfolio as a tool to stimulate teachers' reflections. Medical teacher. 2006;28:277-82.
129. Singh R, Naughton B, Taylor J, Koenigsberg M, Anderson D, McCausland L, et al. A comprehensive collaborative patient safety residency curriculum to address the ACGME core competencies. Medical education. 2006;39:1195-204.
130. Dannefer E, Bierer SB, Gladding S. Evidence within a portfolio-based assessment program: What do medical students select to document their performance? Medical teacher. 2012;34:215-20.
131. Rees C, Sheard C. The reliability of assessment criteria for undergraduate medical students' communication skills portfolios: The Nottingham experience. Medical education. 2004;38:138-44.
132. Kassab S, Bidmos M, Nomikos M, Daher-Nashif S, Kane T, Sarangi S, et al. Construct Validity of an Instrument for Assessment of Reflective Writing-Based Portfolios of Medical Students. Advances in Medical Education and Practice. 2020;Volume 11:397-404.
133. Michels N, Driessen E, Muijtjens A, Van Gaal L, Bossaert L, De Winter B. Portfolio assessment during medical internships: How to obtain a reliable and feasible assessment procedure? Education for health (Abingdon, England). 2009;22:313.
134. Susan E, Kinnair D. Integrating the assessment of interprofessional education into the health care curriculum. Journal of Taibah University Medical Sciences. 2016;11.
135. Bierer SB, Dannefer E. Does Students' Gender, Citizenship, or Verbal Ability Affect Fairness of Portfolio-Based Promotion Decisions? Results From One Medical School. Academic medicine : journal of the Association of American Medical Colleges. 2011;86:773-7.
136. Bierer SB, Dannefer E, Tetzlaff J. Time to Loosen the Apron Strings: Cohort-based Evaluation of a Learner-driven Remediation Model at One Medical School. Journal of General Internal Medicine. 2015.
137. Dannefer E. Beyond assessment of learning toward assessment for learning: Educating tomorrow's physicians. Medical teacher. 2013;35.
138. Dannefer E, Prayson R. Supporting students in self-regulation: Use of formative feedback and portfolios in a problem-based learning setting. Medical teacher. 2013;35.
139. Deitte L. Learning Portfolios in Radiology Residency Education: How Do I Get Started? Journal of the American College of Radiology : JACR. 2008;5:664-9.
140. Vance G, Williamson A, Frearson R, O'Connor N, Davison J, Steele C, et al. Evaluation of an established learning portfolio. The clinical teacher. 2013;10:21-6.

For peer review only - http://bmjopen.bmj.com/site/about/guidelines.xhtml

141. Suraseranivongse S, Chinachoti T, Aroonpruksakul N, Halilamien P, Rushatamukayanunt P, Raksamani K, et al. Development of a learning portfolio to assess the competency of anesthesia residents in Thailand. Asian Biomedicine. 2011;5.
142. Yates J, James D. Risk factors at medical school for subsequent professional misconduct: multicentre retrospective case-control study. BMJ. 2010;340:c2040.
143. Peeters MJ, Sexton ME. Use of reflective writing within interprofessional education: a mixed-methods analysis. J Interprof Care. 2020;34(3):307-14.
144. Remein CD, Childs E, Pasco JC, Trinquart L, Flynn DB, Wingerter SL, et al. Content and outcomes of narrative medicine programmes: a systematic review of the literature through 2019. BMJ Open. 2020;10(1):e031568.
145. Younas A, Ansar A. DEVELOPING A CURRICULUM FOR PROFESSIONAL ETHICS IN UNDERGRADUATE MEDICAL AND DENTAL EDUCATION: EVIDENCE FROM LITERATURE. 2020;70:1211-3.
146. Haig A, Dozier M. BEME Guide no 3: systematic searching for evidence in medical education--Part 1: Sources of information. Medical teacher. 2003;25(4):352-63.
147. Gordon M, Gibbs T. STORIES statement: publication standards for healthcare education evidence synthesis. BMC medicine. 2014;12(1):143.
148. Dawe R, Pike A, Kidd M, Janakiram P, Nicolle E, Allison J. Enhanced skills in global health and health equity: Guidelines for curriculum development. Canadian Medical Education Journal. 2017;8:e48-e60.
149. Sagasser M, Kramer A, Van der Vleuten C. How do postgraduate GP trainees regulate their learning and what helps and hinders them? A qualitative study. BMC medical education. 2012;12:67.
150. Burch V. Portfolios for assessment and learning: Guide Supplement 45.1 – Viewpoint. Medical teacher. 2011;33:1029-31.

Appendices

Appendix A. SEBA Methodology

Appendix B. Tabulated Summaries

Appendix C. Portfolio Content (Competencies Assessed and Assessment Modalities)

Figure Legend

Figure 2. The SEBA Process

Figure 2. PRISMA Flowchart

For peer review only

Figure 1. *The SEBA Process*

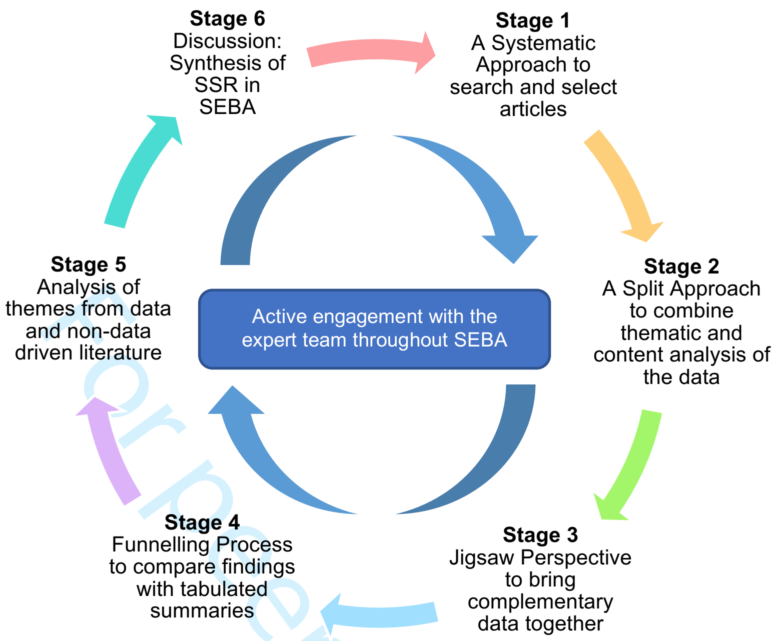

For peer review only

Figure 2. *PRISMA Flowchart*

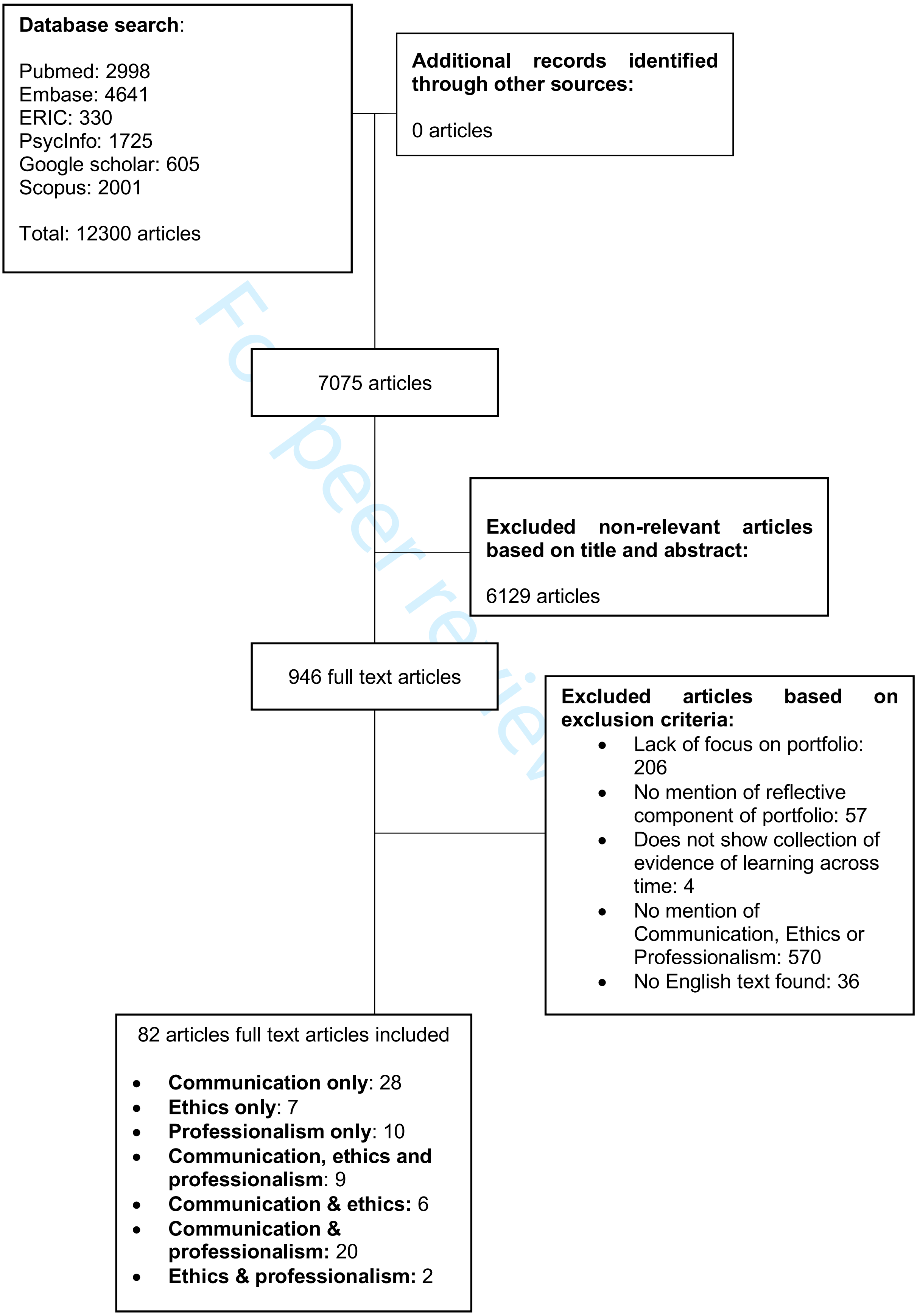

Appendices

Appendix A. SEBA Methodology

Theoretical lens

Krishna's Systematic Evidence-Based Approach (SEBA) is adopted to guide this systematic scoping review (SSR) (henceforth SSR in SEBA) (1-8). SEBA is composed of six stages- 1) Systematic Approach, 2) Split Approach, 3) Jigsaw Perspective, 4) Funnelling Process 5) Analysis of data and non-data driven literature, and 6) Discussion Synthesis (Figure 1). SEBA systematically map available data, structure the extraction of key characteristics of CEP education and its effects, synthesise and summarise actionable and applicable information across a diverse range of study formats and identify gaps in knowledge in current concepts. SEBA's constructivist approach (8-13) and relativist lens (14-17) acknowledges development of CEP competencies are individualised sociocultural constructs informed by the user's narratives, clinical competencies, contextual and environmental considerations.

Expert advice

An expert team consisting of a medical librarian from the Yong Loo Lin School of Medicine (YLLSoM) at the National University of Singapore (NUS), and local educational experts and clinicians at the National Cancer Centre Singapore (NCCS), the Palliative Care Unit, and the Institute of Population Health at the University of Liverpool, YLLSoM and Duke-NUS Medical School, ensured that the SEBA methodology was employed in a consistent manner within accepted practices.

Reflexivity

Personal reflexivity

Recognising that the research team are informed by their interests, narratives, clinical and research insights and contextual considerations, membership to the research and expert teams were made up of experienced physician-tutors, psychologists, methodologists, and educational scholars. Six members of these teams hold masters degrees in medical education, two hold masters degrees in ethics, two hold masters degrees in clinical research and two hold masters degrees in public health. Most of the expert and research team were experienced in quantitative research however five members of the team are experienced in qualitative methods. Six of the research and expert teams have published articles in peer-reviewed journals using the SEBA methodology. One member of the expert team is an experienced researcher at the Palliative Care Institute Liverpool and another is a member of the Health Data Science Department at the University of Liverpool. Both researchers from the University of Liverpool have

For peer review only

been collaborating with the research team on a number of studies pertaining to portfolio use, death and dying, moral distress, PIF and mentoring and are part of the team expanding the use of the SEBA methodology in medical education and palliative care.

Three senior members of the team are working on various projects on medical education and three others have already published articles in peer-reviewed journals in medical education. Six members of the research team were members of the PMI and have published SEBA guided reviews on mentoring, dignity and medical education. To ensure input from all members of the team, synchronous and asynchronous in-person and online meetings and Sandelowski and Barroso (18)'s approach to 'negotiated consensual validation' was used to reach consensus on the issues discussed.

Methodological reflexivity

Adopting a structured constructivist approach, we sought to build a holistic concept of current models of portfolios within medical education acknowledging however that we were limited by manpower and time constraints. We included articles featured in grey and bibliographic databases within the dates set in our selection criteria. Much of these theories required deeper consideration and discussions and we documented our discussions and decisions.

Iterative process

To ensure transparency and accountability, the expert team was involved in all stages of SEBA as part of the iterative process.

For peer review only

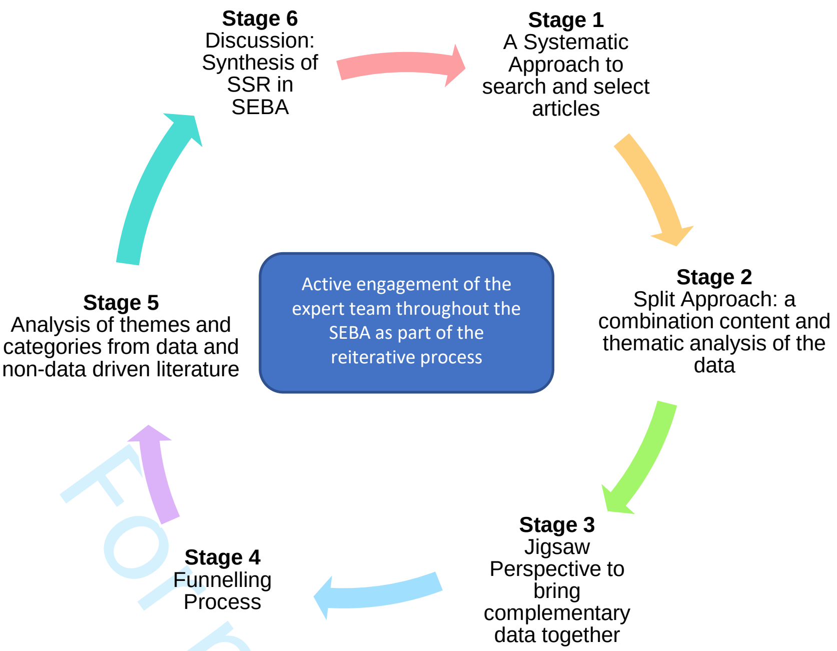

Figure 1. The SEBA Process

Stage 1 of SEBA: Systematic Approach

The SEBA methodology begins with the research and expert teams agreeing upon the research questions, the search terms and the databases to be scrutinised. In this case the research and expert team determined the primary research question: "*What is known about CEP portfolios?*". The secondary research questions were "*what role do CEP portfolios have in teaching and assessing CEP development?*".

Inclusion criteria

A PICOs (Population, Intervention, Comparison, Outcome, Study design) format was adopted to guide the research process (Table 1). There was no comparison group.

Table 1. PICOS, inclusion and exclusion criteria applied to the database search

Table 1. *PICOS Inclusion and Exclusion Criteria*

PICOS	Inclusion criteria	Exclusion criteria
**Population**	• Undergraduate and postgraduate medical students<br>• Qualified medical doctors, physician or resident; medical officer, registrar, house officer, attending, consultant	• Allied health specialties such as Pharmacy, Dietetics, Chiropractic, Midwifery, Podiatry, Speech Therapy, Occupational and Physiotherapy<br>• Non-medical specialties such as Clinical and Translational Science, Alternative and Traditional Medicine, Veterinary, Dentistry
**Intervention**	• portfolios in undergraduate and postgraduate medical education for teaching and assessment of Communication, Ethics and Professionalism	Other documentation methods or learning tools that are:<br>• Not longitudinal or single timepoint<br>• Does not include personal intellectual engagement with the

For peer review only

PICOS	Inclusion criteria	Exclusion criteria
	Criteria of a portfolio:<br>• longitudinal (more than a single timepoint) assessment data<br>• candidate's personal engagement with portfolio content and associated learning<br>• Interventions meeting the above criteria were included regardless of whether they were referred to as portfolios<br><br>All types of portfolios were included in the study:<br>• For instance: electronic & non-electronic; formative & summative or combined; clinical & non-clinical<br>• Portfolios with input from students and/or residents and/or doctors, and/or input from faculty members and other individuals<br>• Portfolios with different structures: extent by which the structure has been prescribed and/or left to individual discretion	content and associated learning (for instance, curriculum vitae, logbooks and the use of personal digital assistants)
**Comparison/ Context**	NA	NA
**Outcome**	Papers that measured the following outcomes were included:<br>• Effectiveness of the use of portfolios to assess and teach Communication, Ethics and Professionalism<br>• Impact of the use of portfolios on medical students (both undergraduate and postgraduate)<br>• Impact of the use of portfolios on the faculty	NA
**Study design**	• Articles in English or translated to English<br>• Articles published from 1st January 2000 to 31st December 2020<br>• Databases: PsycINFO, EMBASE, PubMed, ERIC, Scopus, Google Scholar<br>• All study designs including:<br>• Mixed methods research, meta-analyses, systematic reviews, randomised controlled trials, cohort studies, case-control studies, cross-sectional studies, descriptive papers, grey literature, opinions, letters, commentaries and editorials	NA

Three members of the research team carried out independent ancestry searches of seven leading journals in medical education (Academic Medicine, Medical Education, Medical Teacher, Advances Health Sciences Education, BMC Medical Education, Teaching and Learning in Medicine and Perspectives on Medical Education) accessed through the NUS library portal. In keeping with Pham, Rajic (19)'s recommendations, the searches were restricted to articles published between 1st January 2000 and 31st December 2020 to accommodate to existing

For peer review only

manpower and time constraints. Quantitative, mixed and qualitative research methodologies meeting the inclusion criteria were included.

Members of the research team carried out independent searches of databases. To facilitate this approach, the search process saw experienced senior researchers well-versed in carrying out systematic reviews and systematic scoping reviews each meet with a team of two to three medical students to guide them database searches. This approach was to enhance training of new researchers and to ensure that at least two teams were independently reviewing each database. Each team met regularly and discussed their findings. After a search of the first 100 articles in a particular database, the medical students and the senior researcher compared their findings at an online meeting.

Subsequently, the teams met at specific time points, often after reviewing a predetermined number of included articles to discuss their concerns, exchange opinions and advance their understanding of the research process and the area of study. Sandelowski and Barroso (18)'s 'negotiated consensual validation' was used to achieve consensus on the final list of titles to be reviewed. The teams repeated this process, independently studying all the full text articles on the final list of titles, creating their own lists of articles to be included and discussing their findings at weekly online research meetings over the first 4 months of the project. Consensus on the final list of articles to be analysed was achieved following at least three online meetings when discrepancies were reviewed and discussed by each member of the research team.

As this was a training process for many of the participants who were medical students participating in the Palliative Medicine Initiative, senior mentors and peer-mentors were frequently involved in guiding and discussing individual findings over and above the online meetings especially at the start of each stage of the research process. Interrater reliability was not evaluated.

Stage 2 of SEBA: Split Approach

Krishna's 'Split Approach' (18-23) was employed to enhance the reliability of the data analyses. This saw three groups of researchers independently analysing the included articles.

The first team summarised and tabulated the included full-text articles in keeping with recommendations drawn from Wong, Greenhalgh (24)'s RAMESES publication standards: meta-narrative reviews and Popay, Roberts (25)'s "Guidance on the conduct of narrative synthesis in systematic reviews". The tabulated summaries served to ensure that key aspects of included articles were not lost.

Concurrently, the second team analysed the included articles using Braun and Clarke (26)'s approach to thematic analysis. In Phase 1, the research team carried out independent reviews, 'actively' reading the included articles to find meaning and patterns in the data. In Phase 2, 'codes' were constructed from the 'surface' meaning and collated into a code book to code and analyse the rest of the articles using an iterative step-by-step process. As new codes emerged, these were associated with previous codes and concepts. In Phase 3, the categories were organised into themes that best depict the data. An inductive approach allowed themes to be "defined from the raw data without any predetermined classification" (27). In Phase 4, the themes were refined to best represent the

For peer review only

whole data set and discussed. In Phase 5, the research team discussed the results of their independent analysis online and at reviewer meetings. 'Negotiated consensual validation' was used to determine a final list of themes approach and ensure the final themes.

A third team of researchers employed Hsieh and Shannon (28)'s approach to directed content analysis to analyse the included articles. Analysis using the directed content analysis approach involved "identifying and operationalizing a priori coding categories". The categories employed in the content analysis for undergraduate communications were Rider et al. (29) 's *"A model for communication skills assessment across the undergraduate curriculum",* Goldie (30)'s *"Review of ethics curricula in undergraduate medical education"*, Duffy et al. (31)'s *"Assessing Competence in Communication and Interpersonal Skills: The Kalamazoo II Report"* and Hong et al. (32)'s *"Postgraduate Ethics Training Programs: A Systematic Scoping Review"*. Tay et al. (33)'s *"Assessing Professionalism in Medicine - A Scoping Review of Assessment Tools from 1990 to 2018"* was employed for codes for professionalism and David (34)'s article *"AMEE Medical Education Guide No. 24: Portfolios as a method of student assessment"* was then used to contextualise their use in portfolios. Any data not captured by these codes were assigned a new code.

By using directed content analysis, this "Split Approach" sought to address shortcomings int thematic analysis. This was done by ironing out disparities in data and increases the validity of the identified themes (35). It also limits inherent biases and condenses the interpretations of terminology used by each team's members. Consistency with existing literature is further offered by directed content analysis, by using existing data to identify codes and categories. The codes were used systematically and objectively, strengthening the validity and reliability of a positivist approach (35, 36). The transparency of this approach is enforced by clearly defined categories, together with references throughout the analytical process.

Stage 3 of SEBA: Jigsaw Perspective

The themes and categories identified in the Split Approach were viewed as pieces of a jigsaw puzzle where overlapping/complementary pieces were combined to create a bigger piece of the puzzle referred to as themes/categories. This process was guided by Phases 4 to 6 of France, Uny (37)'s adaptation of Noblit, Hare (38)'s seven phases of meta-ethnography. As per Phase 4, the themes and the categories identified in the Split Approach are grouped together according to their focus. These groupings of categories and themes were then contextualised through the review of the articles from which they were drawn from. Reciprocal translation was used to determine if the themes and categories could be used interchangeably. This allowed the themes and categories to be combined to form themes/categories.

France, Uny's adaptation:
Phase 1: Getting started, deciding on the focus of the synthesis.
Phase 2: Deciding what is relevant to the initial interest.
Phase 3: Reading the Studies.
Phase 4: Determining how the studies are related.
Phase 5: Translating the studies into one another.
Phase 6: Synthesising the translations.

For peer review only

Phase 7: Expressing the synthesis.

Codes derived from thematic analysis (TA): indications, characteristics and strengths and limitations

Codes derived from directed content analysis (DCA): indication for portfolio, portfolio content, portfolio design and strengths and limitations

Overlaps between indications and strengths and limitations allowed the combination of these categories and themes creating two themes/categories called indications and strengths and limitations. Careful review of the categories- portfolio content and portfolio design were subsets of the theme characteristics. For a more accurate presentation, the overlaps were conflated and presented together with the TA codes indicated under the DCA code. (Refer to Domain 4 table)

**Themes Identified**	**Categories Identified**	**Domains Created**
Indications	indications	Indications
characteristics	content	Characteristics
	Portfolio design	
Strengths and limitations	Strengths and limitations	Dignity-conserving Measures

For peer review only

Stage 4 of SEBA: Funnelling Process

The Funnelling Process employs Phases 3 to 5 of France, Uny's adaptation to juxtapose the themes/categories with key messages identified in the tabulated summaries to create domains. These domains form the basis for 'the line of argument' in Stage 6 of SEBA.

For peer review only

For peer review only

For peer review only

Domain 2. Relationship between Dignity, WTHD and Assisted Dying

Subdomains	Codes
**Increasing WTHD**	▪ Loss of dignity (39-81)<br>▪ Fear of loss of dignity (42, 44, 52, 59, 82, 83)<br>▪ Loss of autonomy (39, 40, 42, 45, 47, 50-53, 55, 59, 62, 65, 69, 70, 73, 74, 77, 79, 81, 82, 84-88)<br>▪ Fear of loss of autonomy (42, 54, 70, 89)
**Decreasing WTHD/protective factors**	▪ Addressing of dignity related issues improves health-related quality of life (46)<br>▪ Improving dignity reduces desire for euthanasia (50)
**Effect of assisted dying on dignity**	▪ Reclamation/preservation of dignity (45, 49, 58, 90-95)<br>▪ Lack of assisted dying interferes with dignity (50, 76, 96)<br>▪ Reclamation/preservation of autonomy (43, 45, 49, 51, 52, 67, 73, 74, 77, 85, 88, 92, 94, 97-100)<br>▪ Lack of assisted dying interferes with autonomy (49, 96)<br>▪ Violation of autonomy (100)

For peer review only

Domain 3. Stakeholder Perspectives on Dignity

**Subdomains**	**Codes**	
**Patient Perspectives**	**Positive Aspects**<br>▪ Respect for humanity (67)<br>▪ Right to die (99)<br>▪ Unaffected by fear of death (48)<br>▪ Good death (67, 73, 99, 101-103)<br>- Maintaining control of their death (67, 73, 99, 101-103)<br>- Ending suffering (67, 73)<br>▪ Perceived futility (73, 74)<br>▪ General support (73, 98)<br>▪ Wanting the option for themselves (98)	**Negative Aspects**<br>▪ Religious concerns (67, 73, 102)<br>▪ Ethical concerns (62, 67, 73)<br>- Inability to ascertain patient consent (62, 67)<br>- Equating to murder/suicide (67)<br>▪ Change in ideologies (50)<br>▪ Deterioration of patient-doctor relationship (58, 62, 74, 101)<br>- Loss of faith in palliative/hospice care (62, 101)<br>- Feeling of abandonment when WTHD is turned down (74)<br>- Poor communications (58)<br>▪ Against legislation (73)
**Healthcare Provider**<br><br>**Perspectives**	**Positive Aspects**<br>▪ Good death for patients (42, 47, 49, 50, 54, 56, 57, 61, 69, 70, 82, 87, 101, 103)<br>- Fulfilling patients' desire for control (47, 50, 56, 61, 82, 87, 101, 104)<br>- Ending suffering (42, 54, 57, 61, 69, 70, 103)<br>- Not requiring a long life (49)<br>▪ Feeling helpless (68)<br>▪ Non-abandonment of patients (90)<br>▪ Reducing patients' family burden (90)<br>▪ Avoiding unlawful hastening death (50)<br>▪ General support (40, 47, 62, 68-70, 75, 82, 90, 101, 103-105)<br>▪ Disregarding the need for alternatives (i.e. palliative care) (50)	**Negative Aspects**<br>▪ Religious concerns (106)<br>▪ Disrespecting patients' autonomy by not entertaining their wishes (104, 107)<br>▪ Emotional/moral distress (49, 50, 69, 91, 101, 108-113)<br>- Guilt (91, 112)<br>- Opposing medical principles (49, 69, 101, 108, 110, 111)<br>- Grief, avoidance after carrying out WTHD (113)<br>- Turning doctors into killers (111)<br>▪ No right to die (90)<br>▪ Unable to ascertain if patient's disease is incurable (109)<br>▪ Change in perceptions after disease (82)<br>▪ WTHD should not involve family members (113)<br>▪ "Slippery slope" (110)<br>▪ Societal pressure (55, 74)<br>▪ Disapproval of WTHD (62, 69, 75, 82, 101, 107, 113-115)<br>- Disagreement with the law (115)<br>- Reluctance to carry out WTHD (75, 101)<br>▪ Proposing alternatives (57, 62, 63, 90, 105, 111)<br>- Improving quality of life of dying patients (62, 111)<br>- Better palliative care (63, 90, 105)<br>- Healing (57)<br>▪ Death as an unknown (57)

For peer review only

		▪ Disapproval of 'tired of living' as a reason for WTHD (57)
**Lawmaker Perspectives**	▪ Belief in a right to die (76)<br>▪ Avoiding unlawful hastening death (96)<br>▪ Government's duty to consider patients' WTHD (74)<br>▪ Approval of WTHD (76, 100)	▪ "Slippery slope" (98, 114)<br>- Risk of abuse (114)<br>▪ Instigating social pressure (on the elderly) (74)<br>▪ Disapproval of WTHD (74, 83, 106, 114)

For peer review only

Domain 4. Dignity Conserving Measures

Subdomains	Codes
**Advantages to Dignity Conserving Measures**	▪ Palliative services<br>- Palliative care (40, 44, 47, 49, 50, 62, 63, 66, 73, 82, 90, 91, 103, 111, 116-118)<br>- Palliative sedation (47, 49, 91, 119)<br>- Palliative starvation (85, 109)<br>▪ Pain and symptom management<br>- Control of physical pain (58, 66, 75, 104, 107, 108, 119)<br>- Control of psychological symptoms (75, 102, 120)<br>▪ Hospice care (46, 47, 75, 100, 106, 107, 117)<br>▪ Holistic approach (43, 57, 105, 111, 112, 117, 121, 122)<br>▪ Ease of access to information (47, 57, 71, 87, 92, 100, 112, 117)<br>▪ Distributive justice (55)<br>▪ Social support<br>- Religious (75)<br>- Familial (45)<br>- Healthcare providers (47, 57, 58, 71, 87, 112)
**Limitations to Dignity Conserving Measures**	▪ Cannot completely address:<br>- Existential suffering (54)<br>- Pain (50)<br>- Complete loss of mobility (103)<br>- Extreme shortness of breath (103)<br>- Fear of sudden and rapid bleeding (103)<br>- Ongoing stress of losing autonomy and dignity (103)<br>▪ Side effects of pain medication (58)

For peer review only

Stage 5 of SEBA: Analysis of data and non-data driven literature

A novel aspect of the SEBA methodology has been its inclusion of position, perspective, conference, reflective and opinion papers, editorials, commentaries, letters, posters, oral presentations, forum discussions, interviews, blogs, governmental reports, policy statements and surveys from PubMed, Embase, PsycINFO, Cochrane Database of Systematic Reviews, CINAHL, Scopus databases and the Journal of Pain and Symptom Management, BMC Palliative Medicine, Death Studies, and Palliative Medicine. With many current survey and assessment tools not able to capture the intricate connections and personalised nature of wider concepts of dignity, assisted dying, WTHD, personhood and identity, these resources are a rich source of information. These resources also capture wider patient, HCP and lawmaker perspectives and offer information on ethical, existential, and societal considerations often excluded by traditional systematic reviews as evidenced by recent reviews into how physicians and patients deal with death and dying and moral distress (123-130)

The use of non-data driven articles such as position, perspective, conference, reflective and opinion papers, editorials, commentaries, letters, posters, oral presentations, forum discussions, interviews, blogs, governmental reports, policy statements and surveys is consistent with a constructivist approach that includes all forms of data. In addition, use of non-data driven articles is a valuable source of information for subject matter such as concepts of dignity which are notoriously difficult to study and open to sociocultural considerations.

However, with some of these sources neither peer-reviewed nor necessarily evidence-based, they were differentiated from primary research, compiled and thematically analysed. The themes identified from primary data sources and non-evidence based and/or tertiary data sources were then compared to enhance further the accountability and reproducibility.

With the themes from the two groups found to be similar, the expert and research teams were satisfied that their inclusion did not bias the overall data.

Stage 6 of SEBA: Synthesis of the Discussion

The Best Evidence Medical Education (BEME) Collaboration Guide (131) and the Structured approach to the Reporting In healthcare education of Evidence Synthesis (STORIES) (132) were used to guide the discussion.

For peer review only

References

1. Kow CS, Teo YH, Teo YN, Chua KZY, Quah ELY, Kamal NHBA, et al. A systematic scoping review of ethical issues in mentoring in medical schools. BMC Medical Education. 2020;20(1):1-10.
2. Ngiam LXL, Ong YT, Ng JX, Kuek JTY, Chia JL, Chan NPX, et al. Impact of Caring for Terminally Ill Children on Physicians: A Systematic Scoping Review. Am J Hosp Palliat Care. 2020:1049909120950301.
3. Krishna LKR, Tan LHE, Ong YT, Tay KT, Hee JM, Chiam M, et al. Enhancing Mentoring in Palliative Care: An Evidence Based Mentoring Framework. Journal of Medical Education and Curricular Development. 2020;7:2382120520957649.
4. Chia EWY, Huang H, Goh S, Peries MT, Lee CCY, Tan LHE, et al. A Systematic Scoping Review of Teaching and Evaluating Communications in The Intensive Care Unit. The Asia-Pacific Scholar. In Press.
5. Kuek JTY, Ngiam LXL, Kamal NHA, Chia JL, Chan NPX, Abdurrahman ABHM, et al. The impact of caring for dying patients in intensive care units on a physician's personhood: a systematic scoping review. 2020;15(1):1-16.
6. Hong DZ, Lim AJS, Tan R, Ong YT, Pisupati A, Chong EJX, et al. A Systematic Scoping Review on Portfolios of Medical Educators. Journal of Medical Education and Curricular Development. 2021;8:23821205211000356.
7. Goh S, Wong RSM, Quah ELY, Chua KZY, Lim WQ, Ng ADR, et al. Mentoring in palliative medicine in the time of covid-19: a systematic scoping review. BMC medical education. 2022;22(1):1-15.
8. Bok C, Ng CH, Koh JWH, Ong ZH, Ghazali HZB, Tan LHE, et al. Interprofessional communication (IPC) for medical students: a scoping review. BMC Med Educ. 2020;20(1):372.
9. Ng YX, Koh ZYK, Yap HW, Tay KT, Tan XH, Ong YT, et al. Assessing mentoring: A scoping review of mentoring assessment tools in internal medicine between 1990 and 2019. PloS one. 2020;15(5):e0232511.
10. Bousquet J, Schunemann HJ, Samolinski B, Demoly P, Baena-Cagnani CE, Bachert C, et al. Allergic Rhinitis and its Impact on Asthma (ARIA): achievements in 10 years and future needs. J Allergy Clin Immunol. 2012;130(5):1049-62.
11. Nur Haidah Ahmad Kamal LHET, Ruth Si Man Wong, Ryan Rui Song Ong, Ryan, Ern Wei Seow EKYL, Zheng Hui Mah, Min Chiam, Annelissa Mien Chew Chin, Jamie Xuelian Zhou, Gillian Li Gek Phua, Eng Koon Ong, Jin Wei Kwekc, Kiley Wei-Jen Loh and, Krishna LKR. Enhancing education in Palliative Medicine: the role of Systematic Scoping Reviews. Palliative Medicine & Care: Open Access. 2020;7(1):1-11.
12. Ryan Rui Song Ong REWS, Ruth Si Man Wong. A Systematic Scoping Review of Narrative Reviews in Palliative Medicine Education. Palliative Medicine & Care: Open Access. 2020;7(1):1-22.
13. Zheng Hui Mah RSMW, Ryan Ern Wei Seow Eleanor Kei Ying Loh, Nur Haidah, Ahmad Kamal RRSO, Lorraine Hui En Tan, Min Chiam, Annelissa Mien Chew Chin,, Jamie Xuelian Zhou GLGP, Yoke-Lim Soong, Jin Wei Kwek, and Lalit Kumar Radha Krishna. A Systematic Scoping Review of Systematic Reviews in Palliative Medicine Education. Palliative Medicine & Care: Open Access. 2020;7(1):1-12.
14. Pring R. The 'False Dualism' of Educational Research. Journal of Philosophy of Education. 2000;34(2):247-60.
15. Crotty M. The foundations of social research: Meaning and perspective in the research process: Sage; 1998 Oct 15.

16. Ford DW, Downey L, Engelberg R, Back AL, Curtis JR. Discussing religion and spirituality is an advanced communication skill: an exploratory structural equation model of physician trainee self-ratings. J Palliat Med. 2012;15(1):63-70.
17. Schick-Makaroff K, MacDonald M, Plummer M, Burgess J, Neander W. What synthesis methodology should I use? A review and analysis of approaches to research synthesis. AIMS public health. 2016;3(1):172.
18. Sandelowski M BJ. Handbook for synthesizing qualitative research. New York: Springer; 2007.
19. Pham MT, Rajic A, Greig JD, Sargeant JM, Papadopoulos A, McEwen SA. A scoping review of scoping reviews: advancing the approach and enhancing the consistency. Research synthesis methods. 2014;5(4):371-85.
20. Wen Jie Chua CWSC, Fion Qian Hui Lee, Eugene Yong Hian Koh, Ying Pin Toh, Stephen Mason, Lalit Kumar Radha Krishna. Structuring Mentoring in Medicine and Surgery. A Systematic Scoping Review of Mentoring Programs Between 2000 and 2019. Journal of Continuing Education in the Health Professions. 2020;40(3):158-68.
21. Yong Xiang Ng ZYKK, Hong Wei Yap, Kuang Teck Tay, Xiu Hui Tan, Yun Ting Ong, Lorraine Hui En Tan, Annelissa Mien Chew Chin, Ying Pin Toh, Sushma Shivananda, Scott Compton, Stephen Mason, Ravindran Kanesvaran, Lalit Krishna. Assessing mentoring: A scoping review of mentoring assessment tools in internal medicine between 1990 and 2019. . PLOS ONE. 2020;15(5):e0232511.
22. Peters MD, Godfrey CM, Khalil H, McInerney P, Parker D, Soares CB. Guidance for conducting systematic scoping reviews. Int J Evid Based Healthc. 2015;13(3):141-6.
23. Sambunjak D, Straus SE, Marusic A. A systematic review of qualitative research on the meaning and characteristics of mentoring in academic medicine. J Gen Intern Med. 2010;25(1):72-8.
24. Wong G, Greenhalgh T, Westhorp G, Buckingham J, Pawson R. RAMESES publication standards: meta-narrative reviews. BMC medicine. 2013;11(1):20.
25. Popay J, Roberts H, Sowden A, Petticrew M, Arai L, Rodgers M, et al. Guidance on the conduct of narrative synthesis in systematic reviews. A product from the ESRC methods programme Version. 2006;1:b92.
26. Braun V, Clarke V. Using thematic analysis in psychology. Qualitative research in psychology. 2006;3(2):77-101.
27. Cassol H, Pétré B, Degrange S, Martial C, Charland-Verville V, Lallier F, et al. Qualitative thematic analysis of the phenomenology of near-death experiences. PloS one. 2018;13(2):e0193001.
28. Hsieh H-F, Shannon SE. Three Approaches to Qualitative Content Analysis. Qualitative Health Research. 2005;15(9):1277-88.
29. Rider EA, Hinrichs MM, Lown BA. A model for communication skills assessment across the undergraduate curriculum. Med Teach. 2006;28(5):e127-34.
30. Goldie J. Review of ethics curricula in undergraduate medical education. Med Educ. 2000;34(2):108-19.
31. Duffy FD, Gordon GH, Whelan G, Cole-Kelly K, Frankel R, Buffone N, et al. Assessing competence in communication and interpersonal skills: the Kalamazoo II report. Acad Med. 2004;79(6):495-507.
32. Hong DZ, Goh JL, Ong ZY, Ting JJQ, Wong MK, Wu J, et al. Postgraduate ethics training programs: a systematic scoping review. BMC Medical Education. 2021;21(1):338.
33. Tay KT, Ng S, Hee JM, Chia EWY, Vythilingam D, Ong YT, et al. Assessing Professionalism in Medicine - A Scoping Review of Assessment Tools from 1990 to 2018. J Med Educ Curric Dev. 2020;7:2382120520955159.

For peer review only

34. David M, Davis M, Harden R, Howie P, Ker J, Pippard M. AMEE Medical Education Guide No. 24: Portfolios as a method of student assessment. Medical teacher. 2001;23:535-51.
35. Dixon-Woods M, Agarwal S, Young B, Jones D, Sutton A. Integrative Approaches to Qualitative and Quantitative Evidence. 2004.
36. Bryman A. Social Research Methods: Oxford University Press; 2016.
37. France EF, Uny I, Ring N, Turley RL, Maxwell M, Duncan EAS, et al. A methodological systematic review of meta-ethnography conduct to articulate the complex analytical phases. BMC Medical Research Methodology. 2019;19(1):35.
38. Noblit GW, Hare RD, Hare RD. Meta-ethnography: Synthesizing qualitative studies: sage; 1988.
39. Al Rabadi L, LeBlanc M, Bucy T, Ellis LM, Hershman DL, Meyskens FL, et al. Trends in Medical Aid in Dying in Oregon and Washington. JAMA Network Open. 2019;2(8):e198648-e.
40. Emanuel EJ, Onwuteaka-Philipsen BD, Urwin JW, Cohen J. Attitudes and practices of euthanasia and physician-assisted suicide in the United States, Canada, and Europe. JAMA: Journal of the American Medical Association. 2016;316(1):79-90.
41. Brinkman-Stoppelenburg A, Vergouwe Y, van der Heide A, Onwuteaka-Philipsen BD. Obligatory consultation of an independent physician on euthanasia requests in the Netherlands: What influences the SCEN physicians judgment of the legal requirements of due care? Health Policy. 2014;115(1):75-81.
42. Buiting H, van Delden J, Onwuteaka-Philpsen B, Rietjens J, Rurup M, van Tol D, et al. Reporting of euthanasia and physician-assisted suicide in the Netherlands: Descriptive study. BMC Medical Ethics Vol 10 2009, ArtID 18. 2009;10.
43. Konishi E, Davis A. The right-to-die and the duty-to-die: Perceptions of nurses in the West and in Japan. International Nursing Review. 2001;48(1):17-28.
44. Chapple A, Ziebland S, McPherson A, Herxheimer A. What People Close to Death Say About Euthanasia and Assisted Suicide: A Qualitative Study. Journal of Medical Ethics: Journal of the Institute of Medical Ethics. 2006;32(12):706-10.
45. Coyle N, Sculco L. Expressed desire for hastened death in seven patients living with advanced cancer: a phenomenologic inquiry. Oncol Nurs Forum. 2004;31(4):699-709.
46. Crespo I, Rodríguez-Prat A, Monforte-Royo C, Wilson KG, Porta-Sales J, Balaguer A. Health-related quality of life in patients with advanced cancer who express a wish to hasten death: A comparative study. Palliat Med. 2020;34(5):630-8.
47. Drum CE, White G, Taitano G, Horner-Johnson W. The Oregon Death with Dignity Act: Results of a literature review and naturalistic inquiry. Disability and Health Journal. 2010;3(1):3-15.
48. Ferrand E, Dreyfus JF, Chastrusse M, Ellien F, Lemaire F, Fischler M. Evolution of requests to hasten death among patients managed by palliative care teams in France: a multicentre cross-sectional survey (DemandE). Eur J Cancer. 2012;48(3):368-76.
49. Ramsey C. The right to die: beyond academia. Monash Bioeth Rev. 2016;34(1):70-87.
50. Reagan P, Hurst R, Cook L, Zylicz Z, Otlowski M, Veldink JH, et al. Physician-assisted death: Dying with dignity? Lancet Neurology. 2003;2(10):637-43.
51. Rodríguez-Prat A, Balaguer A, Booth A, Monforte-Royo C. Understanding patients' experiences of the wish to hasten death: an updated and expanded systematic review and meta-ethnography. BMJ Open. 2017;7(9):e016659.
52. Rodríguez-Prat A, van Leeuwen E. Assumptions and moral understanding of the wish to hasten death: a philosophical review of qualitative studies. Medicine, Health Care & Philosophy. 2018;21(1):63-75.

For peer review only

53. Wang L, Elliott M, Henson LJ, Gerena-Maldonado E, Strom S, Downing S, et al. Death with dignity in Washington and Oregon patients with amyotrophic lateral sclerosis. Neurology. 2016;86(16).
54. Fischer S, Huber CA, Furter M, Imhof L, Mahrer Imhof R, Schwarzenegger C, et al. Reasons why people in Switzerland seek assisted suicide: the view of patients and physicians. Swiss Med Wkly. 2009;139(23-24):333-8.
55. Freeman LM, Rose SL, Youngner SJ. Poverty: Not a Justification for Banning Physician-Assisted Death. Hastings Cent Rep. 2018;48(6):38-46.
56. Ganzini L, Goy ER, Dobscha SK. Why Oregon patients request assisted death: Family members' views. Journal of General Internal Medicine. 2008;23(2):154-7.
57. Goligher EC, Wesley Ely E, Sulmasy DP, Bakker J, Raphael J, Volandes AE, et al. Physician-Assisted Suicide and Euthanasia in the ICU: A Dialogue on Core Ethical Issues. Critical Care Medicine. 2017;45(2):149-55.
58. Hendry M, Pasterfield D, Lewis R, Carter B, Hodgson D, Wilkinson C. Why do we want the right to die? A systematic review of the international literature on the views of patients, carers and the public on assisted dying. Palliative Medicine. 2013;27(1):13-26.
59. Hiscox WE. Physician-assisted suicide in Oregon: The 'death with dignity' data. Medical Law International. 2007;8(3):197-220.
60. Kade WJ, Kade WJ. Death with dignity: a case study. Annals of Internal Medicine. 2000;132(6):504-6.
61. Kouwenhoven PS, van Thiel GJ, Raijmakers NJ, Rietjens JA, van der Heide A, van Delden JJ. Euthanasia or physician-assisted suicide? A survey from the Netherlands. Eur J Gen Pract. 2014;20(1):25-31.
62. Bailey F. "I am not afraid of dying. I just don't want to be there when it happens.". Medical Care. 2008;46(12):1195-7.
63. Barutta J, Vollmann J. Physician-assisted death with limited access to palliative care. Journal of Medical Ethics: Journal of the Institute of Medical Ethics. 2015;41(8):652-4.
64. Bolt EE, Pasman H, Deeg DJ, Onwuteaka-Philipsen BD. From advance euthanasia directive to euthanasia: Stable preference in older people? Journal of the American Geriatrics Society. 2016;64(8):1628-33.
65. Dees M, Vernooij-Dassen M, Dekkers W, van Weel C. Review unbearable suffering of patients with a request for euthanasia or physician-assisted suicide: An integrative review. Psycho-Oncology. 2010;19(4):339-52.
66. Duckett S. Pathos, death talk and palliative care in the assisted dying debate in Victoria, Australia. Mortality. 2020;25(2):151-66.
67. Eliott JA, Olver IN. Dying cancer patients talk about euthanasia. Social Science & Medicine. 2008;67(4):647-56.
68. McPherson T. My mum wanted assisted dying but we watched her die slowly and in pain. BMJ: British Medical Journal (Clinical Research Edition). 2012;344:e4007-e.
69. Mukhopadhyay S, Banerjee D. Physician assisted suicide in dementia: A critical review of global evidence and considerations from India. Asian J Psychiatr. 2021;64:102802.
70. Rowe Iii MJ. Beliefs. Chicago, Illinois: American Medical Association; 2015. p. 877-8.
71. Roy DJ. Palliative care and euthanasia: a continuing need to think again. J Palliat Care. 2002;18(1):3-5.
72. van Tol D, Rietjens J, van der Heide A. Judgment of unbearable suffering and willingness to grant a euthanasia request by Dutch general practitioners. Health Policy. 2010;97(2-3):166-72.

For peer review only

73. Wilson KG, Chochinov HM, McPherson CJ, Skirko MG, Allard P, Chary S, et al. Desire for euthanasia or physician-assisted suicide in palliative cancer care. Health Psychology. 2007;26(3):314-23.
74. Florijn BW. From Reciprocity to Autonomy in Physician-Assisted Death: An Ethical Analysis of the Dutch Supreme Court Ruling in the Albert Heringa Case. Am J Bioeth. 2022;22(2):51-8.
75. Ganzini L, Nelson HD, Schmidt TA, Kraemer DF, Delorit MA, Lee MA. Physicians' experiences with the Oregon Death with Dignity Act. N Engl J Med. 2000;342(8):557-63.
76. Kishore RR. Aruna Shanbaug and the right to die with dignity: the battle continues. Indian J Med Ethics. 2016;1(1):38-46.
77. Royo C, Villavicencio-Chávez C, Tomás-Sábado J, Mahtani V, Balaguer A. What Lies behind the Wish to Hasten Death? A Systematic Review and Meta-Ethnography from the Perspective of Patients. PloS one. 2012;7:e37117.
78. Bahník Š, Vranka MA, Trefná K. What makes euthanasia justifiable? The role of symptoms' characteristics and interindividual differences. Death Stud. 2021;45(3):226-37.
79. Hizo-Abes P, Siegel L, Schreier G. Exploring attitudes toward physician-assisted death in patients with life-limiting illnesses with varying experiences of palliative care: a pilot study. BMC Palliative Care. 2018;17(1):56.
80. Ohnsorge K, Gudat H, Rehmann-Sutter C. What a wish to die can mean: reasons, meanings and functions of wishes to die, reported from 30 qualitative case studies of terminally ill cancer patients in palliative care. BMC Palliat Care. 2014;13:38.
81. Ruijs CDM, van der Wal G, Kerkhof AJFM, Onwuteaka-Philipsen BD. Unbearable suffering and requests for euthanasia prospectively studied in end-of-life cancer patients in primary care. BMC Palliative Care. 2014;13(1):62.
82. Gómez-Vírseda C, Gastmans C. Euthanasia in persons with advanced dementia: a dignity-enhancing care approach. J Med Ethics. 2021.
83. Hale DB. A pretty pass: when is there a right to die? Common Law World Rev. 2003;32(1):1-14.
84. Cheung G, Sundram F. Who are the elderly who want to end their lives? [References]. Rational suicide in the elderly: Clinical, ethical, and sociocultural aspects. Cham, Switzerland: Springer International Publishing/Springer Nature; Switzerland; 2017. p. 113-28.
85. Dees MK, Vernooij-Dassen MJ, Dekkers WJ, Vissers KC, van Weel C. 'Unbearable suffering': A qualitative study on the perspectives of patients who request assistance in dying. Journal of Medical Ethics: Journal of the Institute of Medical Ethics. 2011;37(12):727-34.
86. Fontalis A, Prousali E, Kulkarni K. Euthanasia and assisted dying: what is the current position and what are the key arguments informing the debate? Journal of the Royal Society of Medicine. 2018;111(11):407-13.
87. Ganzini L, Dobscha SK, Heintz RT, Press N. Oregon physicians' perceptions of patients who request assisted suicide and their families. Journal of Palliative Medicine. 2003;6(3):381-90.
88. Karlsson M, Milberg A, Strang P. Dying cancer patients' own opinions on euthanasia: An expression of autonomy? A qualitative study. Palliative Medicine. 2012;26(1):34-42.
89. Gamester N, Van den Eynden B. The relationship between palliative care and legalized euthanasia in Belgium. Journal of Palliative Medicine. 2009;12(7):589-91.
90. Rodriquez E. The arguments for euthanasia and physician-assisted suicide: ethical reflection. Linacre Q. 2001;68(3):251-61.
91. Abrahm JL. Patient and family requests for hastened death. Hematology Am Soc Hematol Educ Program. 2008:475-80.

For peer review only

92. Gandsman A. Paradox of choice and the illusion of autonomy: The construction of ethical subjects in right-to-die activism. Death Studies. 2018;42(5):329-35.
93. Granda-Cameron C, Houldin A. Concept Analysis of Good Death in Terminally Ill Patients. American Journal of Hospice & Palliative Medicine. 2012;29(8):632-9.
94. Heintz AP. Euthanasia--or, death on request. J Psychosom Obstet Gynaecol. 2002;23(2):73-5.
95. Jiraphan A, Pitanupong J. General population-based study on preferences towards end-of-life care in Southern Thailand: a cross-sectional survey. BMC Palliative Care. 2022;21(1):36.
96. Dyer C. Two men plead with judges to let doctors end their life legally. BMJ: British Medical Journal (Clinical Research Edition). 2012;344:e4270-e.
97. Castelli Dransart DA, Lapierre S, Erlangsen A, Canetto SS, Heisel M, Draper B, et al. A systematic review of older adults' request for or attitude toward euthanasia or assisted-suicide. Aging & Mental Health. 2021;25(3):420-30.
98. Achille MA, Ogloff JR. Attitudes toward and desire for assisted suicide among persons with amyotrophic lateral sclerosis. Omega: Journal of Death and Dying. 2003;48(1):1-21.
99. Right to die--right or wrong? Lancet Neurol. 2003;2(10):583.
100. Friend ML. Physician-Assisted Suicide: Death With Dignity? Journal of Nursing Law. 2011;14(3/4):110-6.
101. Drury B. The doctors of mercy. Men's Health. 2006;21(8):180-7.
102. Wilson KG, Scott JF, Graham ID, Kozak JF, Chater S, Viola RA, et al. Attitudes of terminally ill patients toward euthanasia and physician-assisted suicide. Archives of Internal Medicine. 2000;160(16):2454-60.
103. Reid T. Reflections from a provider of medical assistance in dying. Canadian Family Physician. 2018;64(9):639-40.
104. Asai A, Ohnishi M, Nagata SK, Tanida N, Yamazaki Y. Doctors' and nurses' attitudes towards and experiences of voluntary euthanasia: survey of members of the Japanese Association of Palliative Medicine. J Med Ethics. 2001;27(5):324-30.
105. Eastaugh A. Choosing to die. Br J Gen Pract. 2012;62(595):96-7.
106. Salladay SA. Christian ethics. Death with dignity? Journal of Christian Nursing. 2010;27(3):232-.
107. Broom A. On euthanasia, resistance, and redemption: The moralities and politics of a hospice. Qualitative Health Research. 2012;22(2):226-37.
108. Mentzelopoulos SD, Haywood K, Cariou A, Mantzanas M, Bossaert L. Evolution of medical ethics in resuscitation and end of life. Trends in Anaesthesia and Critical Care. 2016;10:7-14.
109. Raus K, Sterckx S. Euthanasia for mental suffering. New directions in the ethics of assisted suicide and euthanasia. Cham, Switzerland: Springer International Publishing/Springer Nature; Switzerland; 2015. p. 79-96.
110. Robinson V, Clarke J, George R. Euthanasia: A caricature of care, not a cure for suffering. British Journal of Neuroscience Nursing. 2007;3(10):457-60.
111. Stephenson J. Assisted dying: a palliative care physician's view. Clin Med (Lond). 2006;6(4):374-7.
112. Finlay IG. Quality of life to the end. Commun Med. 2005;2(1):91-5.
113. Feigin S, Owens RG, Goodyear-Smith F. Helping a loved one die: the act of assisted dying in New Zealand. Mortality. 2019;24(1):95-110.
114. Dyer C. Dying woman loses her battle for assisted suicide. Bmj. 2002;324(7345):1055.

For peer review only

115. Fournier RR. Responses to life after death with dignity: the Oregon experience. Soc Work. 2000;45(5):467-8.
116. Comby M, Filbet M. The demand for euthanasia in palliative care units: A prospective study in seven units of the 'Rhone-Alpes' region. Palliative Medicine. 2005;19(8):587-93.
117. Simpson E. Harms to dignity, bioethics, and the scope of biolaw. Journal of Palliative Care. 2004;20(3):185-92.
118. Ganzini L, Back A. From the USA: Understanding requests for physician-assisted death. Palliative Medicine. 2003;17(2):113-4.
119. Gentzler J. What is a death with dignity? J Med Philos. 2003;28(4):461-87.
120. Robinson S, Kissane DW, Brooker J, Hempton C, Burney S. The Relationship Between Poor Quality of Life and Desire to Hasten Death: A Multiple Mediation Model Examining the Contributions of Depression, Demoralization, Loss of Control, and Low Self-worth. J Pain Symptom Manage. 2017;53(2):243-9.
121. Clark D. Cultural considerations in planning palliative and end of life care. Palliat Med. 2012;26(3):195-6.
122. Oczkowski SJW, Crawshaw DE, Austin P, Versluis D, Kalles-Chan G, Kekewich M, et al. How can we improve the experiences of patients and families who request medical assistance in dying? A multi-centre qualitative study. BMC Palliative Care. 2021;20(1):185.
123. Ong RSR, Wong RSM, Chee RCH, Quek CWN, Burla N, Loh CYL, et al. A systematic scoping review moral distress amongst medical students. BMC Medical Education. 2022;22(1):466.
124. Chua KZY, Quah ELY, Lim YX, Goh CK, Lim J, Wan DWJ, et al. A systematic scoping review on patients' perceptions of dignity. BMC Palliative Care. 2022;21(1):118.
125. Ho CY, Lim NA, Ong YT, Lee ASI, Chiam M, Gek GPL, et al. The impact of death and dying on the personhood of senior nurses at the National Cancer Centre Singapore (NCCS): a qualitative study. BMC Palliat Care. 2022;21(1):83.
126. Chiam M, Ho CY, Quah E, Chua KZY, Ng CWH, Lim EG, et al. Changing self-concept in the time of COVID-19: a close look at physician reflections on social media. Philos Ethics Humanit Med. 2022;17(1):1.
127. Chan NPX, Chia JL, Ho CY, Ngiam LXL, Kuek JTY, Ahmad Kamal NHB, et al. Extending the Ring Theory of Personhood to the Care of Dying Patients in Intensive Care Units. Asian Bioeth Rev. 2022;14(1):71-86.
128. Huang H, Toh RQE, Chiang CLL, Thenpandiyan AA, Vig PS, Lee RWL, et al. Impact of Dying Neonates on Doctors' and Nurses' Personhood: A Systematic Scoping Review. J Pain Symptom Manage. 2022;63(1):e59-e74.
129. Vig PS, Lim JY, Lee RWL, Huang H, Tan XH, Lim WQ, et al. Parental bereavement – impact of death of neonates and children under 12 years on personhood of parents: a systematic scoping review. BMC Palliative Care. 2021;20(1):136.
130. Ho CY, Kow CS, Chia CHJ, Low JY, Lai YHM, Lauw S-K, et al. The impact of death and dying on the personhood of medical students: a systematic scoping review. BMC Medical Education. 2020;20(1):516.
131. Haig A, Dozier M. BEME Guide no 3: systematic searching for evidence in medical education--Part 1: Sources of information. Medical teacher. 2003;25(4):352-63.
132. Gordon M, Gibbs T. STORIES statement: publication standards for healthcare education evidence synthesis. BMC medicine. 2014;12(1):143.

For peer review only

Appendices

Appendix B. Tabulated Summaries.

No.	Title	Author/year	MERSQI	COREQ	Reason for inclusion			Undergraduate (UG)/Postgraduate (PG)	Methodology	Purpose of study	Key findings
					Communication	Ethics	Professionalism				
1	The CanMEDS resume: a useful educational portfolio tool for diagnostic radiology residents	Finlay, K.<br>Probyn, L.<br>Ho, S.<br><br>2012			✓		✓	PG	We developed a comprehensive resident CanMEDS resume for ongoing documentation of resident participation, education, and developing competencies in CanMEDS Roles for diagnostic radiology.<br>This resume or portfolio summary is structured to high- light all 7 CanMEDS Roles. The resident is responsible for ongoing updates and submits this as part of his or her annual evaluation.	The objective of this article is to discuss the utility of this tool for inclusion in the educational portfolio for radiology residents.	The document increases awareness of the CanMEDS Roles and documents resident professional development, while also promoting reflection on the impor- tance of these Roles for radiology and future practice. The added advantage for program directors and assistants is up- to-date documentation of resident activities, which serves as a highly useful tool, particularly for program accreditation purposes.
2	A pilot evaluation of portfolios for quality attestation of clinical ethics consultants	Fins, Joseph J<br>Kodish, Eric<br>Cohn, Felicia<br>Danis, Marion<br>Derse, Arthur R<br>Dubler, Nancy Neveloff<br>Goulden, Barbara<br>Kuczewski, Mark<br>Mercer, Mary Beth<br>Pearlman, Robert A<br><br>2016	7.5	14		✓		PG	The QAPTF committee decided on the key components of the portfolio, their respective weightages and scoring quality of each element.<br>ASBH issued an open call to individuals practicing CEC, publicized on the ASBH website, for let- ters of intent (LOI) to submit portfolios for the piloting of this first step in a quality attestation process. The QAPTF wanted to ensure a	We describe the results of a pilot project utilizing portfolios as an evaluation tool. The goals of this quality attestation pilot project were to assess the feasibility of a process to evaluate whether CE consultants are competent to perform ethics consultations as a lead or solo consultant and to develop quality metrics for this assessment process.	We found that this approach is feasible and resulted in a reasonably wide distribution of scores among the 23 submitted portfolios that we evaluated. We discuss limitations and implications of these results, and suggest that this is a significant step on the pathway to an eventual certification process for clinical ethics consultants.

For peer review only

No.	Title	Author/year	MERSQI	COREQ	Reason for inclusion			Undergraduate (UG)/Postgraduate (PG)	Methodology	Purpose of study	Key findings
					Communication	Ethics	Professionalism				
									representative distribution of candidates for the first portfolio review and adopted a method for select- ing portfolios that was intentionally designed to minimize bias.Candidates needed to have performed CEC, have at least a master's degree in a relevant discipline, and have sufficient CEC experience to be able to provide all ele- ments of the portfolio. 32 out of 82 individuals were invited to submit portfo- lios. After candidates submitted their portfolios, they were surveyed to obtain feedback on their portfolio develop- ment and submission experience. QAPTF members were also asked about their experi- ence and perceptions of whether the portfolio elements and approach to scoring functioned effectively in identify- ing candidates who should or should not proceed with the next step of		

For peer review only

No.	Title	Author/year	MERSQI	COREQ	Reason for inclusion			Undergraduate (UG)/Postgraduate (PG)	Methodology	Purpose of study	Key findings
					Communication	Ethics	Professionalism				
									quality attestation. They also were asked about ways to improve future attestation activities. This feedback is reflected in the limitations and discussion sections that follow.		
3	Portfolios and 'the competent clinician': past, present and future assessment	Gavriel, J.<br><br>2016	-	-			✓	PG	NA	To describe the evolution of portfolios, including its advantages, challenges and the future of portfolios	Portfolios have been a part of medical education since the early 1990s and many educators use them frequently, even habitually. Portfolios are primarily based on a constructivist approach to learning and there are also strong links with adult learning principles.Competence based education, once under fire for being too simplistic, now forms a significant part of medical education including the development of portfolios.<br><br>In the med- ical education arena there are some advantages that are particularly relevant: triangulation amongst those working alongside learners, developing wider professional skills and demonstrating the potential for lifelong learning and excellence.<br><br>For some learners and educators the portfolio is a burden and a frustration: the volume of work, a hoop jumping approach and a subjective nature all providing challenges [8] that are at least partially a result of some key complex- ities of the portfolio assessment.

For peer review only

No.	Title	Author/year	MERSQI	COREQ	Reason for inclusion			Undergraduate (UG)/Postgraduate (PG)	Methodology	Purpose of study	Key findings
					Communication	Ethics	Professionalism				
											Portfolios are evolving assessments; they are subject to continuing developments but the ongoing challenge for portfolio designers is the balance between reliability and validity on one hand and tick-boxing on the other.
4	Diffusion of an e-portfolio to assist in the self-directed learning of physicians: An exploratory study	Goliath, Cheryl Lynn<br><br>2010	13	21	✓			PG	A single case study comprised of nine physicians was used to examine the factors involved when the diffusion of an e-portfolio was introduced. The participants were studied using interviews, the Stages of Concern (SoCQ) and the Self-Directed Learning Readiness Scale (SDLRS) questionnaires, four-week trial period of I-Webfolio and field notes based upon observations during the study.	This study examined the impact of implementing an e-portfolio system within an Internal Medicine physician practice as a means of developing a process of life-long learning and professional development for the participants.	The results were analyzed to identify patterns or themes that appeared. One such pattern emerged, in that; all entries in the e-portfolio were almost exclusively in the Medical Knowledge component. A theme that became apparent was that none of the physicians in the study have developed a self-directed learning plan. The results of this study suggested that adoption of technology is multifactoral and there was no consistent individual profile that predicted optimal adoption of the innovation; however, increased scores on the SDLRS would indicate that the diffusion of I-Webfolio was effective in improving the self-directed learning of the participants.
5	The attributes of a good medical trainee: how to build your portfolio	Joseph, S. R.<br>Sikkel, M. B.<br><br>2009	-	-	✓			PG	-	Review of the attributes of a good medical trainee based on the essential physician competencies, and how then to build a good portfolio	A comprehensive and contemporaneously completed procedural, operative, teaching and reflective practice logs should be included in the portfolio, and should be commenced as soon as possible. Compose your portfolio around the seven key roles of the Canadian Medical Education Directives for Specialists model, and be innovative when

For peer review only

No.	Title	Author/year	MERSQI	COREQ	Reason for inclusion			Undergraduate (UG)/Postgraduate (PG)	Methodology	Purpose of study	Key findings
					Communication	Ethics	Professionalism				
											filling each section with concrete examples. Use reflective practice of clinical cases also as further evidence.
6	Study skills course in medical education for postgraduate residents	Bhattarai, M. D.<br>2007	-	-	✓			PG	-	The learners have to take active parts in the teaching learning activities. To make them aware and to help them develop the skills required, the need of the study skills course in medical education early in the part of their training has been realized for the postgraduate residents	The important areas of the study skills course focusing in the requirement of clinical components of the postgraduate residents are 1.) Interpersonal and communications skills, 2.) Teaching, learning and presentation skills, 3.) Language, reading and computer use, 4.) Evidence based medicine and diagnosis and management, 5). Assessment principles and strategies, 6). Time management strategies to get the best out of the training, 7). Reflection, portfolio and self-directed lifelong learning, and 8). Follow-up presentation. The methodologies that could be used in such study skills course are interactive lectures, brainstorming, presentations by the trainees, demonstration to and by the trainees, small group discussion, group work and presentation, group and individual feedback, practice sessions, role play, short relevant video movies, video recording of the trainees and viewing with feedback. With their already tight training schedule and posting and other similar other mandatory courses required for the postgraduate residents, much time cannot be allocated for the study skills course in medical education alone. Similar study skills course in medical education

For peer review only

No.	Title	Author/year	MERSQI	COREQ	Reason for inclusion			Undergraduate (UG)/Postgraduate (PG)	Methodology	Purpose of study	Key findings
					Communication	Ethics	Professionalism				
											may need to be arranged for the undergraduate medical students as well.
7	Fellows' perceptions of a mandatory reflective electronic portfolio in a geriatric medicine fellowship program	Ruiz, Jorge G. Qadri, Syeda S. Karides, Marina Castillo, Carmen Milanez, Marcos Roos, Bernard A. 2009	9	15	✓		✓	PG	We conducted surveys and focus groups followed by quantitative and qualitative data analysis	Electronic portfolios (ePortfolios) can be useful for evaluating and documenting mastery of competencies. We investigated geriatric medicine fellows' perceptions of an ePortfolio	Our study revealed that fellows considered the ePortfolio acceptable and accessible. Fellows reflected on aspects relevant to postgraduate training and future practice and documented adherence to competencies. However, fellows expressed resentment about the self-reflection component, which interfered with the overall acceptance of the ePortfolio. Although ePortfolios can be powerful and reflective tools for competency assessment, care must be taken in their implementation.
8	Portfolios for assessment and learning: Guide Supplement 45.1 - Viewpoint	Burch, Vanessa 2011	-	-		✓		PG	NA	NA	The critical question to ask then is Should poorly resourced countries not find ways of adapting this valuable learning and assessment strategy to suit local realities? (Spandel 1997). Clearly, the 'Rolls Royce' version of portfolio implementation and assessment is beyond the reach of developing countries but a less resource-intensive strategy could be a feasible option. It was with this goal in mind that the University of Cape Town (UCT) in South Africa embarked on the task of adapting portfolios for use in less well-resourced settings (Burch & Seggie 2008). Medical students in a 6-year programme are introduced to portfolio-based learning and assessment in their third year of training and each subsequent year until graduation. Currently, clinical students write up,

For peer review only

No.	Title	Author/year	MERSQI	COREQ	Reason for inclusion			Undergraduate (UG)/Postgraduate (PG)	Methodology	Purpose of study	Key findings
					Communication	Ethics	Professionalism				
											and manage under supervision, between 18 and 32 cases in 6- to 10-week medicine clerkships These patients are presented to a specialist in training on admission and the attending consultant on the post-intake ward the following morning. This provides ample opportunity for students to look back on the action, reflect on their performance and receive feedback and mentoring from senior clinicians directly in the workplace setting. In this way, consultants and specialists in training act as mentors helping students identify alternative strategies and set goals to stimulate further learning. These distinct phases of the workplace-based mentoring process closely reflect the phases of spiral professional development described by Korthagen et al. (2001). The importance of this approach is that mentors provide ongoing guidance for students during the course of performing their routine clinical duties limiting the need for additional regular meetings between students and mentors. Students who are struggling are free to consult the course convener for assistance, but an introductory lecture clearly outlining the purpose, format and examination of portfolios, as well as a set of guidelines for completing portfolio tasks has significantly limited the need for further advice. In addition to writing up a series of patient case studies, students also

For peer review only

No.	Title	Author/year	MERSQI	COREQ	Reason for inclusion			Undergraduate (UG)/Postgraduate (PG)	Methodology	Purpose of study	Key findings
					Communication	Ethics	Professionalism				
											identify at least one clinical learning need based on each case discussion; these ‘question and answer’ tasks are also included in the portfolio. This provides a good opportunity for needs-driven, focused, self-directed learning. Students also complete a range of other written tasks using the index clinical cases in their portfolios. These case-based tasks (Harden et al. 2000) include written reflections on ethical issues relevant to selected patients, a bio-psycho-social analysis (mini-ethnography) (Kleinman & Benson 2006) of selected patients and the use of case material to explore the principles of Primary Health Care (Vivian et al. 2010) as they relate to patients in the portfolio. Finally, students also write up detailed treatment plans for a list of 32 common conditions as reflected by the cases in the portfolio. These tasks are all designed to promote self-directed, patient-centred learning.<br>At UCT it was recognised that marking of this volume of portfolio material was beyond the human resources capacity at departmental level and so a strategic decision was made to focus on an assessment strategy based on a series of structured interviews, focusing on different aspects of a sample of portfolio cases. Students rotate through four stations of 15 min each (OSCE style) at which single-examiner structured interviews are conducted based

No.	Title	Author/year	MERSQI	COREQ	Reason for inclusion			Undergraduate (UG)/Postgraduate (PG)	Methodology	Purpose of study	Key findings
					Communication	Ethics	Professionalism				
											on patient cases randomly selected from the portfolio. The case-based discussions focus on clinical reasoning, case management plans, bioethics, psychosocial aspects of illness and the principles of Primary Health Care. This approach assesses student performance on a 'biopsy' of at least 8–10 cases contained in the portfolio. Each case discussion is scored using a nine-point rubric (1–3 poor, 4–6 adequate, 7–9 good) and scores are totalled across all cases discussed to achieve an aggregate score for the examination. The key reasons for adopting this assessment strategy at UCT included limited human resources but, more importantly, the observation that English second language speakers, more than 50% of university admissions nationally (Council on Higher Education 2009), are better able to express themselves in oral rather than written form. Indeed, it has been asked whether portfolios provide educators with real insight into practitioners' clinical ability or simply show that they are good at writing about what they do? (Karlowicz 2000). Having developed the strategy, the key question is whether any other university in sub-Saharan Africa has successfully adopted it. To date, two other South African universities have adopted and adapted the approach to suit local needs and two other African universities have

For peer review only

No.	Title	Author/year	MERSQI	COREQ	Reason for inclusion			Undergraduate (UG)/Postgraduate (PG)	Methodology	Purpose of study	Key findings
					Communication	Ethics	Professionalism				
											successfully adopted the strategy at Makerere University (Mubuuke et al. 2010) and Kampala International University (Ezeala et al. 2010) both in Uganda. Clearly, the data are limited but it does suggest that portfolio-based learning and assessment are within reach of countries attempting to improve the quality of health sciences education using available resources. This, I believe, is one of the most important issues to be addressed in the twenty-first century – the adaptation and implementation of better teaching and assessment strategies in developing countries so as to keep abreast of educational advances developed world countries enjoy.
9	AMEE Guide No. 25: The assessment of learning outcomes for the competent and reflective physician	Shumway, J. M. Harden, R. M. 2003	-	-	✓	✓	✓	PG	-	To emphasize on the 2 important features of contemporary medical education: first is an emphasis on assessment as a tool to ensure quality in training programmes, to motivate students and to direct what they learn. The second is a move to outcome-based education where the learning outcomes are defined and decisions about the curriculum are based on these.	Portfolio assessments have a positive impact on the learning environment because they document what the learner has done and ask the learner to reflect on what she/he has accomplished. They ask the learner to put together large amounts of disparate information into a whole that tells a story about the learner's accomplishments. Portfolios are a valuable instru- ment for inclusion in the examiner's toolkit, if for no other reason than that they assess learning outcomes such as critical thinking and self-assessment, which are not easily assessed using other instruments. They also provide a record of the

For peer review only

No.	Title	Author/year	MERSQI	COREQ	Reason for inclusion			Undergraduate (UG)/Postgraduate (PG)	Methodology	Purpose of study	Key findings
					Communication	Ethics	Professionalism				
											student's performance over a period of time and are not just a snapshot at one specific point in time. The reliability of portfolios is at least in part due to the ability of raters to agree on standards and criteria for the content and assessment of portfolios.
10	A comprehensive collaborative patient safety residency curriculum to address the ACGME core competencies	Singh, R.<br>Naughton, B.<br>Taylor, J. S.<br>Koenigsberg, M. R.<br>Anderson, D. R.<br>McCausland, L. L.<br>Wahler, R. G.<br>Robinson, A.<br>Singh, G.<br><br>2005	-	-	√			PG	-	To design and implement a new patient safety curriculum in collaboration with the Schools of Nursing and Pharmacy, in such a way as to address all 6 competencies.	Porfolios is used as one of the multiple assessment approaches in the patient safety programme. However, no evaluation made on this assessment mode.
11	ECLIPPx: an innovative model for reflective portfolios in life-long learning	Cheung, C. R.<br><br>2011	-	-			√	PG	-	For healthcare professionals, the educational portfolio is the most widely used component of lifelong learning – a vital aspect of modern medical practice. When used effectively, portfolios provide evidence of continuous learning and promote reflective practice. But traditional portfolio models are in danger of becoming outmoded, in the face of changing expectations of health- care provider competences today.<br><br>Portfolios in health care have generally focused on competencies in clinical skills. How- ever, many other domains of professional	A new model for categorising the health care port- folios of professionals is proposed. The ECLIPPx model is based on personal practice, and divides the evidence of ongoing professional learning into four categories: educational development; clinical practice; leadership, innovation and professionalism; and personal experience.<br><br>The ECLIPPx model offers a new approach for personal reflection and longitudinal learn- ing, one that gives flexibility to the user whilst simultaneously encompassing the many relatively new areas of competence and expertise that are now required of a modern doctor.

For peer review only

No.	Title	Author/year	MERSQI	COREQ	Reason for inclusion			Undergraduate (UG)/Postgraduate (PG)	Methodology	Purpose of study	Key findings
					Communication	Ethics	Professionalism				
										development, such as professionalism and leadership skills, are increasingly important for doctors and health care professionals, and must be addressed in amassing evidence for training and revalidation. There is a need for modern health care learning portfolios to reflect this sea change.	
12	Development of a web-based, specialty specific portfolio	Clay, A. S., Petrusa, E., Harker, M. and Andolsek, K.<br><br>2007	-	-	✓	✓	✓	PG	Three different disciplines (anesthesiology, surgery and medicine) worked together to create a critical care medicine portfolio. We began by reviewing the curriculum requirements for critical care medicine and organise<br><br>d these requirements into the six ACGME core competencies. We then developed learner led exercises in each core competency that were specific to critical care. Each exercise includes assessment of resident knowledge and application, an evaluation of the exercise, a learner self-	This article illustrates the creation of a specialty specific portfolio that can be used by several different residency programs to document resident competence during a given rotation	Creation of specialty specific portfolio reduces redundancy between disciplines, allows for increased time to be spent on the development of exercises specific to rotation objectives, and aids program directors in the collection of portfolio entries for each resident over the course of a residency.<br><br>We describe the creation of a multidisciplinary learning portfolio that can be used by different training programs within one institution to assess trainee performance in each respective area of expertise. The strengths of the specialty specific portfolio include the ability to be specific to the learning objectives of that rotation/specialty and uniform in content across multiple disciplines within that specialty. Commitment to the portfolio from multiple disciplines allows for careful refining of the portfolio and reduction of duplicative efforts. These specialty specific portfolios could potentially be improved upon if professional

No.	Title	Author/year	MERSQI	COREQ	Reason for inclusion			Undergraduate (UG)/Postgraduate (PG)	Methodology	Purpose of study	Key findings
					Communication	Ethics	Professionalism				
									assessment of skill, and a review of performance by a faculty member. Portfolio entries are highlighted in a multi-disciplinary weekly conference and posted on a critical care web site at our University.		organizations were committed to their adaptation, perhaps as a component of maintenance of certification. The portfolio entries as we have described are also useful for prompting frequent self reflection/evaluation and for providing trainees with feedback on their self-perceptions. Self assessment, a skill which is not often taught or evaluated in medical education, is notoriously inaccurate (Gordon 1991). However, self assessment may be improved with deliberate practice, and frequent self assessment with feedback on that self assessment (Gordon 1992; Norman & Schmidt 1992; Ericsson 2004). If other specialties were to create similar portfolios, the logistics of creating a portfolio for each resident would be simplified: the portfolio could be built a rotation at a time, meeting the objectives of that rotation monthly, and meeting the ACGME requirements over the course of a residency. Furthermore, by simply requiring a certain number entries from each core competency by the end of each year or the end of a residency, the learner is given the discretion to choose those exercise which he/she feels is most relevant given the cases he/she has encountered.
13	A review of portfolio use in residency programs and	Colbert, C. Y., Ownby, A. R. and Butler, P. M.	-	-			✓	PG	Two searches of PubMed, OVID, JSTOR, SCOPUS, and	Portfolios, often described as collections of evi-dence, are discussed	There is wide varia- tion in how portfolios are utilized within U.S. residency programs. The challenge for

For peer review only

No.	Title	Author/year	MERSQI	COREQ	Reason for inclusion			Undergraduate (UG)/Postgraduate (PG)	Methodology	Purpose of study	Key findings
					Communication	Ethics	Professionalism				
	considerations before implementation	2008							FirstSearch Wilson Select were conducted between October 2006 and April 2007 to identify studies and articles related to portfolio usage. Thirty-nine articles met criteria and were reviewed.	as a means of teaching or assessing the Ac- creditation Council for Graduate Medical Education competencies. Yet, it is unclear how many residency programs utilize portfolios. The purpose of this article is to (a) review the literature on portfolio use in graduate medical education; (b) examine efficacy of portfolio use, based upon studies in the field; and (c) offer a discussion of con- siderations for implementing portfolios.	graduate medical education is to create consensus on the definition and purpose of portfolios, such that best practices in portfolio implementation and assessment can be achieved.<br><br>The case for utilizing portfolios in residency education is compelling. Portfolios can offer a diverse palette of learners' achievements and experiences. When assessing competence from a holistic standpoint, the range of behaviors captured in a portfolio may render it superior to more traditional assessment practices. Self-awareness and self-directed learning, thought to be critical to the development of competent physicians, can be enhanced through the act of portfolio development,19 as port- folios can force one to reflect upon strengths, weaknesses, and opportunities for growth.<br><br>Before implementing a portfolio system in a residency pro- gram, several issues need to be considered. Learners and faculty should be trained on the portfolio process including the purpose, structure, and content of portfolios. Mentoring may be required when it comes to content assembly and interpretation. If portfolios are to be used for summative assessment pur- poses, an evaluation rubric—a scoring template—will need to be

For peer review only

No.	Title	Author/year	MERSQI	COREQ	Reason for inclusion			Undergraduate (UG)/Postgraduate (PG)	Methodology	Purpose of study	Key findings
					Communication	Ethics	Professionalism				
											created.<br><br>Last, the promise offered by portfolios is tempered by diffi-culties associated with their use. The lack of a standard defini- tion across the field and the dearth of outcome studies to assess efficacy are pressing concerns. The challenge now is to create consensus on the definition and purpose of portfolios within res- idency programs, such that collaboration within the field may begin to offer best practices in the areas of implementation and assessment.
14	How we teach ethics and communication during a Canadian neonatal perinatal medicine residency: an interactive experience	Daboval, T., Moore, G. P. and Ferretti, E.<br><br>2013	6.5		✓	✓	✓	PG	A revised ethics program implemented in 2009 identified competencies that should be demonstrated at the end of the Neonatal-Perinatal Medicine (NPM) residency. Several seminars were refined while new workshops, problem-based learning in ethics, and a personal portfolio were added.	This article describes the process by which a well-delineated, interactive program to teach ethical reasoning and skillful communication with parents was implemented at the University of Ottawa, Canada.	All teaching strategies were well received based on the average level of satisfaction (5.8 out of 7, SD 0.4). We are now moving forward by formally assessing our program including the impact on knowledge acquisition and behavior.<br><br>A dedicated, interactive competency-based neonatal ethics teaching program is vital to support NPM trainees in learning how to integrate ethical thinking with competencies in communication.<br><br>A dedicated, interactive, competency-based neonatal ethics teaching program with well-identified expectations and eval- uation is vital so that all graduating NPM trainees will not only have knowledge of key ethical precepts and ethical reasoning, but will also have learned how to integrate ethical thinking when they communicate and interact

For peer review only

No.	Title	Author/year	MERSQI	COREQ	Reason for inclusion			Undergraduate (UG)/Postgraduate (PG)	Methodology	Purpose of study	Key findings
					Communication	Ethics	Professionalism				
											professionally with parents during ethically and emotionally charged clinical situations. In this manner, our teaching is supporting a paradigm shift in neonatology from a paternalistic to a shared decision-making model. To advance our methods of education in this area, formal assessments of such teaching programs must occur.
15	Portfolio as a tool to stimulate teachers' reflections	Tigelaar, D. E. Dolmans, D. H. de Grave, W. S. Wolfhagen, I. H. van der Vleuten, C. P.<br><br>2006	-	15	✓			PG	In this study, all the written portfolio assignments were approached as reflections on teacher functioning. In order to gain insight into the portfolio process, we collected the teachers' final portfolio assignments as well as data about the process of portfolio construction. In this way, we obtained portfolio data comprising written portfolio assignments and final reflective portfolio assignments. Firstly, the teachers were asked to describe the course of their teaching career. They were also asked to describe critical incidents in their development as a teacher, set learning goals,	Portfolios are increasingly being used to stimulate teachers' reflections. Frameworks for reflection on teaching often emphasize competencies and behaviours. However, other aspects of teacher functioning are also important, such as the teaching environment and individual teachers' beliefs, professional identity and mission. In a study among five medical school teachers, we explored how a portfolio stimulated reflections on the various aspects of teaching functioning.	The analysis yielded examples of reflections on all aspects of Korthagen's model, although reflections on competencies proved easiest to identify.

For peer review only

No.	Title	Author/year	MERSQI	COREQ	Reason for inclusion			Undergraduate (UG)/Postgraduate (PG)	Methodology	Purpose of study	Key findings
					Communication	Ethics	Professionalism				
									select artefacts in evidence of their teaching activities, compose a profile of a good teacher and reflect on that. The final assign- ment required them to compose a reflective portfolio, using the previous assignments as points of departure. The assignments were intended to stimulate reflection on all aspects of teacher functioning as defined in Korthagen's model. However, the critical incidents assignment was focused on the environment, behaviour, and competencies. Table 1 presents brief descriptions of the assignments.		
16	A method for defining competency-based promotion criteria for family medicine residents	Torbeck, L. Wrightson, A. S.<br><br>2005	-	-	✓		✓	PG	In 2003, both of us began the task of developing promotion criteria by searching the literature for studies identifying standards or criteria for resident promotion. Few studies were found related to residents' progress and	The Accreditation Council for Graduate Medical Education (ACGME) has mandated a shift from a structure- and process-based educational system to a competency-based system. The ACGME has not provided criteria (standards), preferring to leave that to the discretion	The value of promotion criteria<br>Training residents to become competent health care providers has been and always will be an important and challenging endeavor. With the ACGME's new mandate, many program directors and house office staff are looking for useful guidance to make their residency programs compliant. We believe that developing a

For peer review only

No.	Title	Author/year	MERSQI	COREQ	Reason for inclusion			Undergraduate (UG)/Postgraduate (PG)	Methodology	Purpose of study	Key findings
					Communication	Ethics	Professionalism				
									promotion.[2,7] We then reviewed the information contained within the last three Residency Assistance Program workshops of the American Academy of Family Physicians, 2001–2003. Frey et al.'s[8] 2003 outpatient core competency presentation notes were earmarked as having specific core competencies that could easily be rewritten as promotion criteria. Next, we reviewed the residency curriculum of the Department of Family Practice and Community Medicine at the University of Kentucky College of Medicine and listed promotion criteria that we thought were important for Family Medicine residents at each level of that curriculum to achieve. Taking this list and extrapolating from Frey et al.'s work, we composed a list of working promotion criteria.	of the individual training programs. Such criteria and an overall strong evaluation process are essential for residents to attain the appropriate knowledge, skills, and attitudes. With this need in mind, the authors describe an evaluation process in which they developed ACGME-competency-based promotion criteria for family medicine residents at the University of Kentucky College of Medicine in 2004.	set number of promotion criteria that are competency-based should be among the first tasks residency programs should tackle, for several reasons.<br><br>First, having defined criteria will make the three to seven years of residency performance assessment more educationally sound. The education literature reminds us that two components are mandatory to a performance assessment: the task and the criteria.[11,12] Since continuous performance assessment is carried out during the majority of a resident's training, the awareness of what is expected of the resident can be raised for both the resident and the faculty by making the task and criteria clearer to them. Residents then are better able to demonstrate that which is expected of them, and faculty are better prepared to evaluate them.<br><br>Second, having defined promotion criteria can better target remediation in those residents failing to meet the criteria in a timely manner. Intervention during a time early on in the resident's development is much more beneficial than it is just before making a decision to promote or not promote.<br><br>Third, having criteria can improve faculty development, especially in terms of how to assess competence, how to give feedback, and how to account for faculty

For peer review only

No.	Title	Author/year	MERSQI	COREQ	Reason for inclusion			Undergraduate (UG)/Postgraduate (PG)	Methodology	Purpose of study	Key findings
					Communication	Ethics	Professionalism				
											responsibility in training and evaluating competent physicians.
17	Enhanced skills in global health and health equity: Guidelines for curriculum development	Dawe, R., Pike, A., Kidd, M., Janakiram, P., Nicolle, E. and Allison, J.<br><br>2017	10	10		✓		PG	A panel comprised of 34 experts in global health education and practice completed three rounds of a Delphi small group process.	To generate consensus on the essential components of a Global Health/Health Equity Enhanced Skills Program in family medicine.	Consensus (defined as ≥ 75% agreement) was achieved on program length (12 months), inclusion of both domestic and international components, importance of mentorship, methods of learner assessment (in-training evaluation report, portfolio), four program objectives (advocacy, sustainability, social justice, and an inclusive view of global health), importance of core content, and six specific core topics (social determinants of health, principles and ethics of health equity/global health, cultural humility and competency, pre and post-departure training, health systems, policy, and advocacy for change, and community engagement).<br><br>Panellists agreed on a number of program components forming the initial foundation for an evidence- informed, competency-based Global Health/Health Equity Enhanced Skills Program in family medicine.
18	Learning portfolios in radiology residency education: how do I get started?	Deitte, L.<br><br>2008	-	-	✓		✓	PG	-	The purpose of this article is three-fold: (1) to famil- iarize the reader with learning portfolios in medical edu-cation, (2) to outline the new radiology ACGME re- quirements for a portfolio, and (53) to discuss strategies for the implementation of a portfolio as a learning and	As medical education and continuous professional devel- opment shift toward an outcomes-based model, there is increasing focus on documentation of physician self-as- sessment and a commitment to lifelong learning. The ACGME program requirements for diagnostic radiology graduate medical education effective July 1, 2008, in- clude learning portfolios as

For peer review only

No.	Title	Author/year	MERSQI	COREQ	Reason for inclusion: Communication	Ethics	Professionalism	Undergraduate (UG)/Postgraduate (PG)	Methodology	Purpose of study	Key findings
										assessment tool in a residency education program and continued professional development..	one method for this docu- mentation. Although there are some challenges associ- ated with the implementation of learning portfolios, it is likely that portfolios will prove to be a valuable learning and assessment tool not only in graduate medical educa- tion but also throughout a physician's career and recerti- fication process.
19	Evaluation of an established learning portfolio	Vance, G. Williamson, A. Frearson, R. O'Connor, N. Davison, J. Steele, C. Burford, B. 2013	9	-	✓			PG	Deanery-wide trainee attitudes were surveyed by an electronic questionnaire in 2009 and compared with perceptions recorded during the pilot phase (2004–2005).	The trainee-held learning portfolio is integral to the foundation programme in the UK. In the Northern Deanery, portfolio assessment is standar- dised through the Annual Review of Competence Progression (ARCP) process. In this study we aimed to establish how current trainees evaluate portfolio-based learning and ARCP, and how these attitudes may have changed since the foundation programme was first introduced.	Many trainees continue to view the e-portfolio negatively. Indeed, significantly fewer train- ees in 2009 thought that the e-portfolio was a 'good idea' or a 'worthwhile investment of time' than in 2005. Trainees remain unconvinced about the educa- tional value of the e-portfolio: fewer trainees in 2009 regarded it as a tool that might help focus on training or recognise individual strengths and weaknesses. Issues around unnecessary bureaucracy persist. Current trainees tend to understand how to use the e- portfolio, but many did not know how much, or what evidence to collect. Few supervisors were re- ported to provide useful guidance on the portfolio. ARCP encouraged portfolio completion but did not give meaningful feedback to drive future learning. Continued support is needed for both trainees and supervisors in portfolio-building skills and in using the e-portfolio as an educational tool. Trainee-tailored feedback is needed to ensure that portfolio-

For peer review only

No.	Title	Author/year	MERSQI	COREQ	Reason for inclusion			Undergraduate (UG)/Postgraduate (PG)	Methodology	Purpose of study	Key findings
					Communication	Ethics	Professionalism				
											based assessment promotes lifelong, self-directed and reflective learners. Trainees remain unconvinced about the educational value of the e-portfolio
20	Reflective Practice for Patient Benefit: An Analysis of Doctors' Appraisal Portfolios in Scotland	Wakeling, J. Holmes, S. Boyd, A. Tredinnick-Rowe, J. Cameron, N. Marshall, M. Bryce, M. Archer, J. 2019	-	16	✓			PG	Using an in-depth case study approach, 18 online portfolios in Scotland were examined with a template developed to record the types of supporting information submitted and how far these showed reflection and/or changes to practice. Data from semistructured interviews with the doctors (n = 17) and their appraisers (n = 9) were used to contextualize and broaden our understanding of the portfolios.	Reflective practice has become the cornerstone of continuing professional development for doctors, with the expectation that it helps to develop and sustain the workforce for patient benefit. Annual appraisal is mandatory for all practicing doctors in the United Kingdom as part of medical revalidation. Doctors submit a portfolio of supporting information forming the basis of their appraisal discussion where reflection on the information is mandated and evaluated by a colleague, acting as an appraiser.	Portfolios generally showed little written reflection, and most doctors were unenthusiastic about documenting reflective practice. Appraisals provided a forum for verbal reflection, which was often detailed in the appraisal summary. Portfolio examples showed that reflecting on continued professional development, audits, significant events, and colleague multisource feedback were sometimes considered to be useful. Reflecting on patient feedback was seen as less valuable because feedback tended to be uncritical. <br>The written reflection element of educational portfolios needs to be carefully considered because it is clear that many doctors do not find it a helpful exercise. Instead, using the portfolio to record topics covered by a reflective discussion with a facilitator would not only prove more amenable to many doctors but would also allay fears of documentary evidence being used in litigation.
21	Developing an Evaluation Tool for Assessing Clinical Ethics Consultation Skills in Simulation Based	Wasson, K. Parsi, K. McCarthy, M. Siddall, V. J. Kuczewski, M. 2016	-	-		✓		PG	At the beginning of the project development, four of the authors (who are bioethicists with experience in	The American Society for Bioethics and Humanities has created a quality attestation (129) process for clinical ethics consultants;	Given the move toward quality attestation by ASBH and the need to develop a way of assessing the interpersonal skills of clinical ethics consultants "live" we have developed the ACES

For peer review only

No.	Title	Author/year	MERSQI	COREQ	Reason for inclusion			Undergraduate (UG)/Postgraduate (PG)	Methodology	Purpose of study	Key findings
					Communication	Ethics	Professionalism				
	Education: The ACES Project								clinical ethics consultation) agreed that any tool created should be grounded in the ASBH core competencies, as they are the articulated professional standards in the field. After a review of the limited assessment tools available, we agreed to draw from the VA's Ethics Consultant Proficiency Assessment Tool (2014). It draws on the ASBH core competencies, including the categories of Interpersonal Skills, Process Skills, Analytic Skills, and Core Knowledge, is quite comprehensive, and has been in use for a number of years. In addition, we followed Stufflebeam's (2000) guidelines for creating a checklist evaluation tool.<br><br>Given the focused usage of the ACES tool—clinical ethics simulation in an educational setting—we met regularly to	the pilot phase of reviewing portfolios has begun. One aspect of the QA process which is particularly challenging is assessing the interpersonal skills of individual clinical ethics con- sultants. We propose that using case simulation to evaluate clinical ethics consul- tants is an approach that can meet this need provided clear standards for assessment are identified. To this end, we developed the Assessing Clinical Ethics Skills (ACES) tool, which identifies and specifies specific behaviors that a clinical ethics consultant should demonstrate in an ethics case simulation. The aim is for the clinical ethics consultant or student to use a videotaped case simulation, along with the ACES tool scored by a trained rater, to demonstrate their competence as part of their QA portfolio.	tool. Our tool provides a clear structure for key domains, behaviors, and interpersonal skills that can be observed and scored by a trained rater during simulated ethics consultations. Individuals completing the ACES course or using the tool can opt to include their evaluation and video as part of their QA portfolio as a means of demonstrating their interpersonal skills in a simulated ethics consultation. Our tool also provides a framework and structure for the content of an ethics consultation and helps translate the core competencies into practical areas, behaviors, and skills. We spent a great deal of time thinking about and debating which items from the VA tool to include in the final ACES tool. It is not intended to be comprehensive for all aspects of clinical ethics consultation, e.g., for simplicity in the pilot stage we did not examine the clinical ethics consultant's ability to review and decipher the medical chart or write a note, though these elements could be added to the simulations in future. Instead, it focuses on those elements we thought could be captured in simulated clinical ethics consultations in an educational setting in this pilot phase. In the future, other elements of ethics consultation and levels of complexity can be added to the simulations as well as additional cases.

For peer review only

No.	Title	Author/year	MERSQI	COREQ	Reason for inclusion			Undergraduate (UG)/Postgraduate (PG)	Methodology	Purpose of study	Key findings
					Communication	Ethics	Professionalism				
									determine which items from the VA tool would be necessary for this purpose. Our tool is not meant to be a comprehensive tool to assess every element of an ethics consultation or CEC's role, thus it is briefer than the VA tool. It is also designed to be used in ''real time'' by trained bioethics raters, not retrospectively self-administered by the ethics consultant. Once we agreed upon the primary items, we met weekly for 3–4 months to identify and determine the specific behaviors and skills we would be evaluating under each main item. This was a time consuming process involving in-depth discussion and debate. Before each weekly meeting, we viewed videos of simulated ethics consultations from past graduate bioethics courses to refine our tool, its specified items, and behaviors. We discussed and		For clarity of assessment, we developed the ACES tool to be used for individual consultants. We believe each individual ethics consultant needs to have this set of skills, even if they work within a small team model in practice. If the ACES tool was adapted to assess a small team of ethics consultants, the raters would have to rate each consultant individually or rate them all as one ''ethics consultant'' to assess whether the team covered all the necessary items on the evaluation. This approach would be more complicated to assess and, we suspect, much less straightforward to score. At this stage, we do not recommend using the ACES tool in this way.<br>One of the most challenging areas in the ACES tool development was how to view and rate students in a similar way—or inter-rater agreement. We spent a great deal of time discussing what qualifies as ''Done,'' ''Not Done'' or ''Done Incorrectly'' for each and every item. Rater agreement is an ongoing process which needs to be practiced and refreshed, and is not unique to our setting. We plan to assess and recalibrate rater agreement prior to each time a course is offered (1–2 per annum). What makes it more challenging in clinical ethics is the nature of some parts of the consultation; e.g., identifying and clarifying the ethical issues. What counts as ''Done'' for certain items in

For peer review only

No.	Title	Author/year	MERSQI	COREQ	Reason for inclusion			Undergraduate (UG)/Postgraduate (PG)	Methodology	Purpose of study	Key findings
					Communication	Ethics	Professionalism				
									agreed on all of the behaviors and skills listed under each item in the ACES tool. In the end, we agreed on 12 main items and 35 corresponding behaviors to be evaluated and scored.		our tool can be interpreted differently depending on the rater's background and potential biases. This type of item— communication and interpersonal skills in ethics—is not always as neat and clear cut as in other clinical areas such as surgical preparation checklists or putting in a central line. Further work needs to be done to train additional raters if we are to use the tool with more people over time. We plan to recruit additional students to take the ACES course and are working on refining the cases used and plan to evaluate the tool in other settings to obtain feedback on its reliability. When we used the ACES tool in the basic clinical ethics consultation course the feedback was overwhelmingly positive. Students appreciated a clear articulation of what was expected in an ethics consult. While recognizing the tool was not designed to be comprehensive, they valued having a structure and framework for thinking about the elements of an ethics consultation and the behaviors they should be exhibiting. It gave them a clear standard by which to assess themselves and others.
22	Evaluating practice-based learning and improvement: efforts to improve acceptance of portfolios	Fragneto, R. Y., Dilorenzo, A. N., Schell, R. M. and Bowe, E. A. 2010	8		✓		✓	PG	Intensive education about the goals and importance of portfolios began in January 2006, including presentations at departmental conferences and	To find out whether educational efforts improved acceptance of the portfolio and retrospectively audited the portfolio evaluation forms completed by faculty advisors.	Portfolios are considered a best methods techni- que by the ACGME for evaluation of practice-based learning and improvment. We have found that intensive education about the goals and importance of portfolios can enhance acceptance of this evaluation tool, resulting

For peer review only - http://bmjopen.bmj.com/site/about/guidelines.xhtml

No.	Title	Author/year	MERSQI	COREQ	Reason for inclusion: Communication	Ethics	Professionalism	Undergraduate (UG)/Postgraduate (PG)	Methodology	Purpose of study	Key findings
									one-on-one education sessions. Faculty advisors were instructed to evaluate each resident's portfolio and complete a review form. We retrospectively collected data to determine the percentage of review forms completed by faculty. The portfolio reviews also assessed the percentage of 10 required portfolio components residents had completed.		in improved compliance in completion and evaluation of portfolios.
23	Use of portfolios for assessment of global health residents: qualitative evaluation of design and implementation	Gibson, C., Chandratilake, M. and Hull, A. 2018	-	22	✓			PG	A qualitative study was conducted with academic experts in Canadian residency training, as well as directors and residents involved in Global Health study in order to assess the validity and benefit of such a tool. Through an online survey, interviews, and focus groups, views on the portfolio and intended content were collected and coded thematically.	To determine the robustness of portfolios as an assessment tool for Global Health Residents.	This qualitative evaluation validated the use of portfolios for this cohort of students while yielding comments and suggestions that will further enhance the interactive and flexible nature of this seldom used assessment tool. These findings contribute to the understanding of how Global Health assessment can remain individualized yet rigorous.
24	Assessment and Change: An Exploration of	Jocelyn M. Lockyer, PhD; Sanjeev Sockalingam, MD,	-	16	✓			PG	A total of 5000 entries from 2195 psychiatrists were	Fellows of the Royal College of Physicians and	There were 3841 entries for analysis; 1159 entries did not meet the criteria for

For peer review only - http://bmjopen.bmj.com/site/about/guidelines.xhtml

No.	Title	Author/year	MERSQI	COREQ	Reason for inclusion			Undergraduate (UG)/Postgraduate (PG)	Methodology	Purpose of study	Key findings
					Communication	Ethics	Professionalism				
	Documented Assessment Activities and Outcomes by Canadian Psychiatrists	MHPE, FRCPC; Craig Campbell, MD, FRCPC<br><br>2018							examined. A thematic analysis drawing on the framework analysis was undertaken of the 2016 entries.	Surgeons of Canada are required to participate in assessment activities for all new 5-year cycles beginning on or after January 2014 to meet the maintenance of certification program requirements. This study examined the assessment activities which psychiatrists reported in their maintenance of certification e-portfolios to determine the types and frequency of activities reported; the resultant learning, planned learning, and/or changes to the practice they planned or implemented; and the interrelationship between the types of assessment activities, learning that was affirmed or planned, and changes planned or implemented.	assessment. The most commonly reported activities were self-assessment programs, feedback on teaching, regular performance reviews, and chart reviews. Less frequent were direct observation, peer supervision, and reviews by provincial medical regulatory authorities. In response to the data, psychiatrists affirmed that their practices were appropriate, identified gaps they intended to address, planned future learning, and/ or planned or implemented changes. The assessment activities were internally or externally initiated and resulted in no or small changes (accommodations and adjustments) or redirections.<br><br>Psychiatrists reported participating in a variety of assessment activities that resulted in variable impact on learning and change. The study underscores the need to ensure that assessments being undertaken are purposeful, relevant, and designed to enable identification of outcomes that impact practice.
25	Development of a portfolio of learning for postgraduate family medicine training in South Africa: a Delphi study	Jenkins, L., Mash, B. and Derese, A.<br><br>2012	-	10	✓	✓		PG	A workshop held at the WONCA Africa Regional Conference in 2009 explored the purpose and broad contents of the portfolio. The 85 training outcomes, ideas from the WONCA workshop, the literature, and	This study aimed to achieve a consensus on the contents and principles of the first national portfolio for use in family medicine training in South Africa.	A portfolio was developed and distributed to the eight departments of Family Medicine in South Africa, and the CMSA, to be further tested in implementation. This was the first attempt to reach consensus on the development of a national portfolio for family medicine training in South Africa. Consensus was reached on 50 items to include, and 26

For peer review only

No.	Title	Author/year	MERSQI	COREQ	Reason for inclusion			Undergraduate (UG)/Postgraduate (PG)	Methodology	Purpose of study	Key findings
					Communication	Ethics	Professionalism				
									existing portfolios in the various universities were used to develop a questionnaire that was tested for content validity by a panel of 31 experts in family medicine in South Africa, via the Delphi technique in four rounds. Eighty five content items (national learning outcomes) and 27 principles were tested. Consensus was defined as 70% agreement. For those items that the panel thought should be included, they were also asked how to provide evidence for the specific item in the portfolio, and how to assess that evidence.		principles relating to the port- folio. A draft national portfolio and portfolio guide have been developed and distributed to all the medical schools in the country. Further revision and testing with registrars in training is underway, with the aim to deli- ver a final portfolio in the following year.
26	The national portfolio of learning for postgraduate family medicine training in South Africa: experiences of registrars and supervisors in clinical practice	Jenkins, L., Mash, B. and Derese, A.<br><br>2013	-	15	✓			PG	Semi-structured interviews were conducted with 17 purposively selected registrars and supervisors from all eight South African training programmes.	The aim of this study was to explore registrars' and supervisors' experience regarding the portfolio's educational impact, acceptability, and perceived usefulness for assessment of competence.	The learning portfolio had a significant educational impact in shaping work- place based supervision and training and providing formative assessment. Its acceptability and usefulness as a learning tool should increase over time as supervisors and registrars become more competent in its use. There is a need to clarify how it will be used in summative assessment.
27	Demonstration of Portfolios to Assess	PATRICIA S. O'SULLIVAN1,* , MARK D. RECKASE2, TINA	8	12	✓		✓	PG	22 participants in this demonstration project were all	Describes assessment of the reliability and validity of portfolios in a	We must continue to emphasize some important qualities of portfolios: res- ident choice and reflection.

For peer review only

No.	Title	Author/year	MERSQI	COREQ	Reason for inclusion			Undergraduate (UG)/Postgraduate (PG)	Methodology	Purpose of study	Key findings
					Communication	Ethics	Professionalism				
	Competency of Residents	MCCLAIN3, MILDRED A. SAVIDGE1 and JAMES A. CLARDY3<br><br>2004							residents in the psychiatry residency education program at the primary affiliate of a state university medical school.	psychiatry residency program.	These are important to the life-long learner. Portfolio has been able to aid the faculty in meeting ACGME expectations and has oriented programe to the increasingly important role of evaluation.
28	Developing and Successfully Implementing a Competency-Based Portfolio Assessment System in a Postgraduate Family Medicine Residency Program	Laura A. McEwen, PhD, Jane Griffiths, MD, CCFP, FCFP, and Karen Schultz, MD, CCFP, FCFP<br><br>2015	-	-	✓			PG	-	The use of portfolios in postgraduate medical residency education to support competency development is increasing; however, the processes by which these assessment systems are designed, implemented, and maintained are emergent. The authors describe the needs assessment, development, implementation, and continuing quality improvement processes that have shaped the Portfolio Assessment Support System (PASS) used by the postgraduate family medicine program at Queen's University since 2009.	The authors discuss the identification of impact measures at the individual, programmatic, and institutional levels and the ways the department uses these to monitor how PASS supports competency development, scaffolds residents' self- regulated learning skills, and promotes professional identity formation. They describe the "academic advisor" role and provide an appendix covering the portfolio elements. Reflection elements include learning plans, clinical question logs, confidence surveys, and reflections about continuity of care and significant incidents. Learning module elements cover the required, online bioethics, global health, and consult- request modules. Assessment elements cover each resident's research project, clinical audits, presentations, objective structured clinical exam and simulated office oral exam results, field notes, entrustable professional activities, multisource feedback, and in-training evaluation reports. Document elements are the resident's continuing medical education activities including procedures log, attendance log, and patient demographic summaries.

For peer review only

No.	Title	Author/year	MERSQI	COREQ	Reason for inclusion			Undergraduate (UG)/Postgraduate (PG)	Methodology	Purpose of study	Key findings
					Communication	Ethics	Professionalism				
29	Diving for PERLS Diving for PERLS Working and Performance Portfolios for Evaluation and Reflection on Learning	Linda E. Pinsky, MD, Kelly Fryer-Edwards, PhD<br><br>2004	-	-	✓		✓	PG	-	Professional competence requires a commitment to lifelong learning, self-assessment, and excellence. Complex skills such as these require flexible and comprehensive teaching and assessment measures. We describe a combination of working and performance portfolios that both foster and evaluate the development of professional competence. We explain the con- ceptual and practical underpinnings that maximize the effec- tiveness of these tools. Drawing on experience with University of Washington residents, we identify 5 criteria that can help promote successful use of portfolios: separate working and performance functions of portfolios, developing a supportive climate, developing skills in faculty and residents, observing progress over time, and fostering mentorship opportunities.	To incorporate portfolios successfully into a residency program, we recommend including 5 elements (Table 1). We had success in our program by introducing the program slowly, and dedicating time to faculty education on men- toring, reflection, goal setting, and feedback skills, recog- nizing that the use of portfolios requires new skills and a cultural transformation. We are pleased by the residents' appreciation of the process, their honesty in sharing and evaluating personal areas of challenge, and their incorpora- tion of this approach into other areas of their education. For example, a current inpatient chief resident, based on his ex- perience of the importance of goal setting to learning, revised the structure of morning report to include weekly goal-setting sessions as well as a method to evaluate its effectiveness. Residents seeking employment report finding components of the portfolio helpful in creating their CVs, crafting their applications, and as display examples at job applications.<br><br>To date, the literature reports few trials of the portfolio method for assessing professional competence. The Post Graduate Education Accreditation (PGEA) in Britain illus- trates sharing responsibility for learning between teachers and learners. PGEA experimented with portfolios

For peer review only

No.	Title	Author/year	MERSQI	COREQ	Reason for inclusion			Undergraduate (UG)/Postgraduate (PG)	Methodology	Purpose of study	Key findings
					Communication	Ethics	Professionalism				
											as a flex- ible, targeted means of meeting continuing medical edu- 18 –20 cation (CME) requirements. In their pilot study, physicians were responsible for identifying their own learning objectives and outlining how they were going to achieve and document those objectives. The physicians each met with a mentor to review appro- priate objectives and realistic means for achieving them. Structured reflection was built into the process and was seen as strength, explicitly moving away from the passive approach of most traditional lecture-based CME courses.<br>In the PGEA experience, the shortcomings identified were time and mentor availability. However, participants found the “deep learning” and personal mastery they achieved to be well worth it. In addition, accrediting bodies had something of substance to review in assessing the practitioners’ continuing education.<br>Portfolios fill a need within training programs to foster 21–23 and document professional competencies.<br>dents, training is a time of professional identity formation. The current format of most residencies — didactic confer- ences and time-consuming clinical work—emphasizes either a passive or unreflective learner role.<br>Through portfolios, residents can experience

For peer review only

No.	Title	Author/year	MERSQI	COREQ	Reason for inclusion			Undergraduate (UG)/Postgraduate (PG)	Methodology	Purpose of study	Key findings
					Communication	Ethics	Professionalism				
											and develop the skills of lifelong learning, critical thinking, self- assessment, and excellence that can be carried forward into their future careers and practices. They can complete residency with a clearer sense of their own strengths and limitations, and a sense of who they are as physicians.
30	How do postgraduate GP trainees regulate their learning and what helps and hinders them? A qualitative study	Sagasser, M. H., Kramer, A. W. and van der Vleuten, C. P.<br><br>2012	-	18	✓			PG	In a qualitative study with a phenomenologic approach we interviewed first- and third-year GP trainees from two universities in the Netherlands. Twenty-one verbatim transcripts were coded. Through iterative discussion the researchers agreed on the interpretation of the data and saturation was reached.	To explore how postgraduate trainees regulate their learning in the workplace, how external regulation promotes self-regulation and which elements facilitate or impede self-regulation and learning.	Trainees used a short and a long self-regulation loop. The short loop took one week at most and was focused on problems that were easy to resolve and needed minor learning activities. The long loop was focused on complex or recurring problems needing multiple and planned longitudinal learning activities. External assessments and formal training affected the long but not the short loop. The supervisor had a facilitating role in both loops.
31	First year specialist trainees' engagement with reflective practice in the e-portfolio	Helen McNeill • Jeremy M. Brown • Nigel J. Shaw<br><br>2010	7.5	12	✓			PG	A modified Delphi technique was used to develop a grading system to indentify the level of reflection recorded by participants in the e-portfolio. Transcripts of the reflective accounts were then analysed using a qualitative approach which involved coding and categorising	This study aims specifically to explore how they have engaged in reflection on their practice and how they utilise their learning portfolio to document evidence of this.	Findings indicate there is variation is the extent to which doctors both engage in and document evidence of reflection. Further research is needed to explore factors that enable or inhibit the use of the e-portfolio for reflection and whether recorded reflection is a true picture of the cognitive process involved.

For peer review only

No.	Title	Author/year	MERSQI	COREQ	Reason for inclusion			Undergraduate (UG)/Postgraduate (PG)	Methodology	Purpose of study	Key findings
					Communication	Ethics	Professionalism				
									the data. This study demonstrated a wide variation in both the quantity and quality of reflection. Of particular note in the qualitative data analysis were themes relating to clinical knowledge and skills, learning in practice, communication, feelings, types of learning experience reflected on and wider aspects of medical practice.		
32	Development of a learning portfolio to assess the competency of anesthesia residents in Thailand	Suraseranivongse, S., Chinachoti, T., Aroonpruksakul, N., Halilamien, P., Rushatamukayanunt, P., Raksamani, K., Sirivanasandha, B. and Mandee, S.<br><br>2011	12.5	8			✓	PG	Learning portfolio was developed from Thai Medical Council general competencies, academic activities, and performance assessment in several modalities. Twenty-four first year anesthesia residents and eight mentors were enrolled for this study. One staff mentored three residents and rated their competencies in portfolios, twice, four-months apart. Content validity was assessed by six content experts. Concurrent validity of portfolio was determined	Develop a portfolio for learning improvement in first year anesthesia residents in Thailand, and validate this portfolio as a competency evaluation and to identify strength and weakness of implementation.	All content experts accepted that this portfolio could assess general competencies of the first year anesthesia residents. The majority of mentors and residents (>70%) agreed with the benefit of portfolio based on learning development and competency assessment. However, half of residents were not satisfied with the burden from portfolio

For peer review only - http://bmjopen.bmj.com/site/about/guidelines.xhtml

No.	Title	Author/year	MERSQI	COREQ	Reason for inclusion			Undergraduate (UG)/Postgraduate (PG)	Methodology	Purpose of study	Key findings
					Communication	Ethics	Professionalism				
									by agreement with faculty global rating and in-training examination. Inter-rater reliability of portfolio was evaluated by five faculties that rated 24 residents. Practicality was commented upon by all mentors and residents in the questionnaire and semi-structure, open-ended questions.		
33	Education Research: Communication skills for neurology residents - Structured teaching and reflective practice	Watling, C. J. and Brown, J. B. 2007	8.5	10	✓	✓		PG	A group of 12 neurology residents participated in a series of six case-based communi-cation skills workshops. Each workshop focused on a particular clinical scenario, including break-ing bad news, discussing do-not-resuscitate orders, communicating with "difficult" patients, disclosing medical errors, obtaining informed consent for neurologic tests and procedures, and discussing life-and-death decisions with families of critically ill patients.	Despite the importance of communication skills for neurologists, specific training in this area at the residency level is often lacking. This study aimed to enhance learning of these skills and to encourage reflective practice around communication skills.	The program was well accepted, and residents rated the workshops as effective and relevant to their practice. Analysis of residents' portfolios revealed three themes relevant to patient–physician communication: 1) communication is more successful when adequate time is allowed, 2) the ability to empathize with patients and their families is essential to successful interactions, and 3) the develop- ment of specific approaches to challenging scenarios can facilitate effective interactions. The portfo- lios also demonstrated that residents would engage in reflective practice.<br><br>Targeting of communication skills training around specific clinical scenarios using neurologic cases was well accepted and was deemed relevant to practice. The use of portfolios may

For peer review only

No.	Title	Author/year	MERSQI	COREQ	Reason for inclusion			Undergraduate (UG)/Postgraduate (PG)	Methodology	Purpose of study	Key findings
					Communication	Ethics	Professionalism				
									Residents also kept reflective portfolios in which real examples of these interactions were recorded.		promote lifelong learning in this area.
34	What the HEC-C? An Analysis of the Healthcare Ethics Consultant-Certified Program: One Year in	Horner, C. Childress, A. Fantus, S. Malek, J. 2020			✓	✓		PG	-	In this paper, we explore the history that has led to this certification process, and evaluate the ability of the HEC-C Program to meet the goals it has set forth for HCECs. We describe the benefits and weak- nesses of the program and offer constructive feedback on how the process might be strengthened, as well as share our team's experience in preparing for the exam.	Ultimately, while we are generally supportive of efforts to professionalize the field and ensure competence among HCECs, we are skeptical that a limited process such as this provides much assurance of such compe- tence either for HCECs or their employers. We pro- pose that a more robust or two-tiered evaluation process is needed before such a credential could reli- ably attest to the competence and expertise of an HCEC. Despite shortcomings in the Certification Process as it is currently designed, in general there are benefits of having some kind of certification for individual consultants, as well as HCEC programs and the broader HCEC field. For consultants, achieving and maintaining certification requires continued review of core materials to refresh ethics knowledge and pro- vides external validation of the consultant's ability to engage in consultation, boosting confidence that the individual is successfully engaged in continuing educa- tion. For HCEC programs, having credentialed con- sultants supports the program's mission to engage in quality consultation, taking a step toward ensuring individual consultants are performing their job in a manner

For peer review only

No.	Title	Author/year	MERSQI	COREQ	Reason for inclusion			Undergraduate (UG)/Postgraduate (PG)	Methodology	Purpose of study	Key findings
					Communication	Ethics	Professionalism				
											consistent with established standards in the field. Finally, for the HCEC community as a whole, having a singular credential helps to standardize the practice and validate ethics consultation as a defined and important healthcare specialty. This effort to legit- imize the field as a profession will support the inclu- sion of clinical ethics across healthcare, hopefully expanding the practice from a few select academic or larger healthcare systems to all health care settings. Reaching a point where a health care ethics credential signifies not only competence but also excellence in the ability to conduct a clinical ethics consultation, however, may still be a long way off.
35	Assessing Professionalism in Medicine – A Scoping Review of Assessment Tools from 1990 to 2018	Tay, K. T.<br>Ng, S.<br>Hee, J. M.<br>Chia, E. W. Y.<br>Vythilingam, D.<br>Ong, Y. T.<br>Chiam, M.<br>Chin, A. M. C.<br>Fong, W.<br>Wijaya, L.<br>Toh, Y. P.<br>Mason, S.<br>Krishna, L. K. R.<br><br>2020			✓		✓	UG and PG	Arksey and O'Malley's (2005) approach to scoping reviews was used to identify appropriate publications featured in four data- bases published between 1 January 1990 and 31 December 2018. Seven members of the research team employed thematic analysis to evaluate the selected articles.	Medical professionalism enhances doctor-patient relationships and advances patient-centric care. However, despite its pivotal role, the concept of medical professionalism remains diversely understood, taught and thus poorly assessed with Singapore lacking a linguistically sensitive, context specific and culturally appropriate assessment tool. A scoping review of assessments of professionalism in medicine was thus carried out to better guide its understanding	Prevailing assessments of professionalism in medicine must contend with differences in setting, context and levels of pro- fessional development as these explicate variances found in existing assessment criteria and approaches. However, acknowledging the sig- nificance of context-specific competency-based stages in medical professionalism will allow the forwarding of guiding principles to aid the design of a culturally-sensitive and practical approach to assessing professionalism.

For peer review only

No.	Title	Author/year	MERSQI	COREQ	Reason for inclusion			Undergraduate (UG)/Postgraduate (PG)	Methodology	Purpose of study	Key findings
					Communication	Ethics	Professionalism				
36	Content and outcomes of narrative medicine programmes: a systematic review of the literature through 2019	Remein, Christy DiFrances Childs, Ellen Pasco, John Carlo Trinquart, Ludovic Flynn, David B. Wingerter, Sarah L. Bhasin, Robina M. Demers, Lindsay B. Benjamin, Emelia J.<br><br>2020	-	-	✓			PG	We conducted a systematic review of literature published through 2019 using five major databases: PubMed, Embase, PsycINFO, ERIC and MedEdPORTAL. Eligible NM programming included textual analysis/close reading of published literature and creative/reflective writing. Qualifying participants comprised individuals from academic medicine and health sciences disciplines. We reviewed and categorised programme goals, content and evaluation activities to assess participant satisfaction and programme efficacy. Two members of the research team assessed the risk of bias, independently screening records via a two-round, iterative process to reach consensus on eligibility.	Narrative medicine (NM) incorporates stories into health sciences paradigms as fundamental aspects of the human experience. The aim of this systematic review is to answer the research question: how effective is the implementation and evaluation of NM programmes in academic medicine and health sciences? We documented objectives, content and evaluation outcomes of NM programming to provide recommendations for future narrative-based education.	Of 1569 original citations identified, we selected 55 unique programmes (described in 61 records). In all,<br>41 (75%) programmes reported a form of evaluation; evaluation methods lacked consistency. Twenty-two programmes used quantitative evaluation (13 well described), and 33 programmes used qualitative evaluation (27 well described). Well-described quantitative evaluations relied on 32 different measures (7 validated) and showed evidence of high participant satisfaction and pre-post improvement in competencies such as relationship- building, empathy, confidence/personal accomplishment, pedagogical skills and clinical skills. An average of 88.3% of participants agreed or strongly agreed that the programme had positive outcomes. Qualitative evaluation identified high participant satisfaction and improvement<br>in competencies such as relationship-building, empathy, perspective-taking/reflection, resilience and burnout detection/mitigation, confidence/personal accomplishment, narrative competence, and ethical inquiry.<br><br>Evaluation suggests that NM programming leads to high participant satisfaction and positive outcomes

No.	Title	Author/year	MERSQI	COREQ	Reason for inclusion			Undergraduate (UG)/Postgraduate (PG)	Methodology	Purpose of study	Key findings
					Communication	Ethics	Professionalism				
											across various competencies. We suggest best practices and innovative future directions for programme implementation and evaluation.
37	Use of portfolios in early undergraduate medical training	Driessen, Erik<br>Van Tartwijk, Jan<br>Vermunt, Jan<br>van der Vleuten, Cees<br><br>2003		10		✓		UG	During the academic year 2001–02 242 first-year medical students compiled a portfolio. Semi-structured interviews were held with a select group of students to explore the effect of the portfolio on reflection. Students from four random mentor groups, 39 in total, were approached and asked if they would be interviewed on the portfolio. With the exception of one student, all students were prepared to take part in the interview. The interview sched-ule focused on the process of compiling a portfolio and its possible effect on reflective ability. In addition to this, students were given the opportunity to offer suggestions for improvement.	This article describes the use of portfolios in **early** undergraduate medical training... In this article, we will describe the Maastricht portfolio as an illustration of an early undergraduate portfolio. We will discuss the reasons for the choices we made in designing the portfolio and report on a first evaluation, focusing on the effects on students' reflective ability.	The majority of students were of the opinion that analysing one's competences in a portfolio was instructive and meaningful. With regard to learning how to reflect and recognize learning needs, however, mentor coaching proved to be necessary. The results thus far show that the portfolio is a worthwhile addition to existing assessment and learning tools. Our preliminary findings show that the introduction of such a portfolio in the early stages of medical training seems to be an effective way for students to learn how to reflect on their learning and behaviour.
38	Conditions for reflective use of portfolios in	Driessen, E. W.<br>van Tartwijk, J.<br>Overeem, K.		18			✓	UG	We designed a portfolio that was aimed at	Portfolios are often used as an instrument with	The conditions for successful reflective use of portfolios that emerged from

No.	Title	Author/year	MERSQI	COREQ	Reason for inclusion			Undergraduate (UG)/Postgraduate (PG)	Methodology	Purpose of study	Key findings
					Communication	Ethics	Professionalism				
	undergraduate medical education	Vermunt, J. D. van der Vleuten, C. P.<br><br>2005							stimulating reflection in early undergraduate medical education, using experiences described in the medical educa- tion literature and elsewhere. Conditions for reflect- ive portfolio use were identified through interviews with 13 teachers (mentors), who were experienced in mentoring students in the process of developing their portfolios. The interviews were analysed according to the principles of grounded theory.	which to stimulate students to reflect on their experiences. Research has shown that working with portfolios does not automatically stimulate reflection. In this study we addressed the question: What are the conditions for successful reflective use of portfolios in undergraduate medical education?	the interviews fell into 4 categories: coaching; portfolio structure and guidelines; relevant experiences and materials, and summative assessment. According to the mentors, working with a portfolio designed to meet these conditions will stimulate students' reflective abilities...This study shows that portfolios are a potentially valuable method of assessing and develop- ing students' reflective skills in undergraduate med- ical training, provided certain conditions for effective portfolios are recognised and met. Portfolios have a strong potential for enhancing learning and assess- ment but they are very vulnerable and may easily lead to disappointment. Before implementing portfolios in education, one should first consider whether the necessary conditions can be fulfilled, including an appropriate portfolio structure, an appropriate assessment procedure, the provision of enough new experiences and materials, and sufficient teacher capacity for adequate coaching and assessment.
39	Portfolio Assessment Implementation in Clinical Year of Community Medicine Module: Students Perspective	Ekayanti, Fika Risahmawati, Risahmawati Fadhilah, Marita<br><br>2017	7				✓	UG	Nine portfolios were assigned to students within 5 weeks length module. The data was collected by questionnaires from 46 students after completing module in November 2016. Students were	This study aimed to identify the correlation of students' perspective for using portfolio and their portfolio scores.	Most students (36/78.3%) felt that the portfolio was not appropriate as their summative assessment. They preferred less portfolios, 30 (63%) students requested to decrease portfolios to 3-5, 28.3% to 6-8 and only 8.7% to <3. There was no significant correlation between students' satisfaction of using portfolio

No.	Title	Author/year	MERSQI	COREQ	Reason for inclusion			Undergraduate (UG)/Postgraduate (PG)	Methodology	Purpose of study	Key findings
					Communication	Ethics	Professionalism				
									asked about their satisfaction of using portfolio, then correlated to their portfolio scores. The reasons and suggestion for ideal portfolios were identified. Analysis was done by SPSS 20 using Rank Spearman correlation test. Students felt that creating portfolios were not comfortable.		to their portfolios score (p=0.262), while there was significant correlation between students' score to the number of portfolios to be submitted (p=0.017; r=0.349). Reasons for decreasing number of portfolios were inadequate time and many other tasks to be finished. Students in UIN Jakarta haven't used to retelling their experiences and reflecting them in writing as perceived in many students who were not used to portfolios. Doing portfolios need lots of work and time-consuming. Having lesser number of portfolios to be submitted would encourage students to create portfolios. Building familiarization, time management and good motivation for creating portfolios were important to successful portfolios. There was no significant correlation between students' satisfaction to their scores. Students should be encouraged to get comfortable in creating portfolios for the benefit as lifelong learning tool. Motivation to write and reflect should be nurtured to improve students' portfolio satisfaction and commitment.
40	Students' perception of portfolio as a learning tool at King Abdulaziz University Medical School	Fida, N. M. Hassanien, M. Shamim, M. S. Alafari, R. Zaini, R. Mufti, S. Al-Hayani, A. Farouq, M. Al-Zahrani, H. 2018	8	14	✓	✓	✓	UG	Portfolios were introduced in the 2nd through 5th years at King Abdulaziz University over a two-year period (2013–2015). At the end of each academic year, students completed a	Medical education has a longstanding tradition of using logbooks to record activities. The portfolio is an alter- native tool to document competence and promote reflective practice. This study assessed the	The results showed a difference in focus between basic and clinical years: in basic years students' focus was on acquiring practical skills, but in clinical years they focused more on acquiring complex skills, including identifying and man- aging problems. The questionnaire responses nonetheless revealed a

For peer review only

No.	Title	Author/year	MERSQI	COREQ	Reason for inclusion			Undergraduate (UG)/Postgraduate (PG)	Methodology	Purpose of study	Key findings
					Communication	Ethics	Professionalism				
									mixed questionnaire that included a self-assessment of skills learned through the use of portfolio.	acceptance of portfolio use among Saudi undergraduate medical students.	positive trend in acceptance (belief in the educational value) of portfolios among students and their mentors, across the years of the program. Using portfolios as a developmental learning and formative assessment tool in the early undergraduate years was found to contribute to students' ability to create their own clinical skills guidelines in later years, as well as to engage in and appreciate reflective learning.
41	The use of portfolios to foster professionalism: Attributes, outcomes, and recommendations	Renato Soleiman Franco, Camila Ament Giuliani dos Santos Franco,Olívia Pestana,Milton Severo &Maria Amélia Ferreira<br><br>2017		9		✓	✓	UG	A systematic review on the use of portfolios in teaching professionalism to medical students identified 1257 papers. Of these, 11 articles met all inclusion criteria	The main objective of this research was to review the characteristics of portfolios and their outcomes for teaching professionalism to undergraduate medical students	According to the papers, the use of portfolios for teaching professionalism shows versatility, supports learning strategies and has the potential to be used in different contexts, including for formative and summative purposes. The weaknesses were based on the artificiality of the reflections, deficient instructions, time-consuming processes and preference among students for other teaching methods. Students complained about feeling that the reflection was 'forced', and they tended to write based on social conventions rather than reveal their true thoughts. Reflection is a powerful component of the portfolio, but the method by which it is taught could easily ruin its potential to boost professionalism. Requiring reflection did not ensure its achievement; increased understanding by students regarding how and why they were doing it, the clarity of assessment methods and constructive feedback might strengthen the potential for success. A

For peer review only

No.	Title	Author/year	MERSQI	COREQ	Reason for inclusion			Undergraduate (UG)/Postgraduate (PG)	Methodology	Purpose of study	Key findings
					Communication	Ethics	Professionalism				
											framework was designed to support faculty members in developing and applying portfolios with a clear and broad view of this teaching strategy.
42	AMEE Medical Education Guide No. 24: Portfolios as a method of student assessment	Friedman Ben David, M.<br>Davis, M. H.<br>Harden, R. M.<br>Howie, P. W.<br>Ker, J.<br>Pippard, M. J.<br><br>2001	-	-	✓	✓	✓	UG	-	This guide is intended to inform medical teachers about the use of portfolios for student assessment. It provides a background to the topic, reviews the range of assessment purposes for which portfolios have been used, identifies possible portfolio contents and outlines the advantages of portfolio assess- ment with particular focus on assessing professionalism...The current state of understanding of the technical, psychometric issues relating to portfolio assessment is clarified. The final part of the paper provides a practical guide for those wishing to design and implement portfolio assessment in their own institutions.	-
43	Teaching professionalism in the early years of a medical curriculum: a qualitative study	Goldie, J.<br>Dowie, A.<br>Cotton, P.<br>Morrison, J.<br><br>2007		18	✓	✓		UG	A qualitative approach was adopted involving semistructured interviews, on a 1 in 6 sample of tutors involved in teaching in the early curricular years, and 3 student focus groups. The	This study investigated tutors' and students' perspectives of the delivery of profession- alism in the early years of Glasgow's learner-centred, problem-based learning (PBL), integrated medical curriculum.	Involvement in teaching raised students' and tutors' awareness of their professionalism. Learning activities promoting critical reflection were most effective. The integration of professionalism across the domains of Vocational Studies (135) was important for learning; however, it was not well integrated with the

For peer review only

No.	Title	Author/year	MERSQI	COREQ	Reason for inclusion			Undergraduate (UG)/Postgraduate (PG)	Methodology	Purpose of study	Key findings
					Communication	Ethics	Professionalism				
									findings were subjected to between-method triangulation.		PBL core. Integration was pro- moted by having the same tutor present throughout all VS sessions. Early patient contact experiences were found to be particularly important. The hidden curriculum provided both opportunities for, and threats to, learning. The small-group format provided a suitable environment for the examination of pre-existing perspectives. The portfolio was an effective learning tool, although its assessment should be formalised.<br><br>Reflection is integral to professional development. Early clinical contact is an important part of the process of socialisation, as it allows students to enter the community of practice that is the medical profession. Role models can contribute powerfully to students' learning and identity formation. As students move towards fuller participation, the clinical milieu should be controlled to maximise the influence of role models, and opportunities for guided reflection should be sustained.
44	Assessing students' personal and professional development using portfolios and interviews	Gordon, J.<br><br>2003	6.5			✓	✓	UG	The instruments used to assess Year 1 stu- dents in PPD are a portfolio and interview. This assessment format encourages students to explore ideas and values that are important to them	Medical schools are placing more emphasis on students' personal and professional development (PPD) and are seeking ways of assessing student progress towards meeting outcome goals in relation to professionalism. The	In 1997⁄98, 96% of students agreed that they had engaged in useful reflection on their approach to the course and 91% agreed that the experience was worthwhile. A further 76% of students agreed that they could see opportunities to modify their approach in some ways as result of this exercise.

For peer review only

No.	Title	Author/year	MERSQI	COREQ	Reason for inclusion			Undergraduate (UG)/Postgraduate (PG)	Methodology	Purpose of study	Key findings
					Communication	Ethics	Professionalism				
									and relevant to the PPD theme. A confidential interview, based on the PPD goals, is held with a faculty member who has read the student's portfolio.	Faculty of Medicine at the University of Sydney sought an assessment method that would demonstrate the value of reflection in attaining PPD, provide feedback and encourage stu- dents to take responsibility for setting and achieving high standards of performance.	
45	Developing a sustainable electronic portfolio (ePortfolio) program that fosters reflective practice and incorporates CanMEDS competencies into the undergraduate medical curriculum	Hall, P.<br>Byszewski, A.<br>Sutherland, S.<br>Stodel, E. J.<br><br>2012	-	-	✓		✓	UG	-	In this article, the authors describe the development of an electronic Portfolio (ePortfolio) program that enables uOttawa medical students to document their activities and to demonstrate their development of competence in each of the eight roles	The authors reflect on the challenges<br>they faced in the development and implementation of the ePortfolio program and share the lessons they have learned along the way to a successful and sustainable program. These lessons include switching from a complex information technology system to a user-friendly, Web- based blog platform; rethinking orientation sessions to ensure that faculty and students understand the value of the ePortfolio program; soliciting student input to improve the program and increase student buy-in; and providing faculty development opportunities and recognition.
46	The utility of reflective portfolios as a method of assessing first year medical students' personal and professional development	Rees, Charlotte E.<br>Shepherd, Maggie<br>Chamberlain, Suzanne<br><br>2005	8	17	✓			UG	The construct validity of the criteria was established by exploring its relationship with two other assessment methods and its reliability was determined using the	-	Themes emerging from the focus groups include students preferring the structured nature of the portfolios but assessors feel- ing that this reduced the uniqueness of the portfolios. Although students understood the importance of reflective practice, some disliked the process of reflection, particularly

For peer review only

No.	Title	Author/year	MERSQI	COREQ	Reason for inclusion			Undergraduate (UG)/Postgraduate (PG)	Methodology	Purpose of study	Key findings
					Communication	Ethics	Professionalism				
									generalizability (G) coefficient. Focus groups with assessors and students were convened to explore their views of the portfolios. Two portfolio analyses had a G coefficient of .42. Performance in the portfolio analyses was correlated with performance in personal and professional development judgements (r = −.512, p < .01) and scientific reports (r = .273, p = .002).		reflective writing. Educators should design assessment criteria that maximize reliability, validity and acceptability rather than simply focusing on reliability alone.<br><br>Despite these methodological limitations, this study provides support for the reli- ability and validity of the portfolio analyses assessment criteria at Peninsula Medical School.
47	Reflections: an inquiry into medical students' professional identity formation	Wong, A.<br>Trollope-Kumar, K.<br><br>2014		14	✓	✓		UG	Sixty-five medical students (46 women; 19 men) from a class of 194 consented to the study of their portfolios. In total, 604 reflections were analysed and coded using thematic narrative analysis. The codes were merged under subthemes and themes. Common or recurrent themes were identified in order to develop a descriptive framework of professional identity formation. Reflections were then analysed	The purpose of this study was to understand the major influences on medical students' professional identity formation.	Five major themes were associated with professional identity formation in medi- cal students: prior experiences, role models, patient encounters, curriculum (formal and hidden) and societal expectations. Our longi- tudinal analysis shows how these themes inter- act and shape pivotal moments, as well as the iterative nature of professional identity from the multiple ways in which individuals con- struct meaning from interactions with their environments.<br><br>Our study provides a window on the dynamic, discursive and constructed nature of professional identity formation. The five key themes associated with professional identity formation provide strategic

No.	Title	Author/year	MERSQI	COREQ	Reason for inclusion			Undergraduate (UG)/Postgraduate (PG)	Methodology	Purpose of study	Key findings
					Communication	Ethics	Professionalism				
									longitudinally within and across individual portfolios to examine the professional identity formation over time with respect to these themes.		opportuni- ties to enable positive development. This study also illustrates the power of reflective writing for students and tutors in the professional identity formation process.
48	Content validity of workplace-based portfolios: A multi-centre study	Nele R.M. Michels, Marijke Avonts, Griet Peeraer, Kris Ulenaers, Luc F. Van Gaal, Leo L. Bossaert, Erik W. Driessen, Arno M.M. Muijtjens & Benedicte Y. De Winter<br><br>2016	9.5		✓		✓	UG	We reviewed 120 workplace portfolios at three medical universities (Belgium and the Netherlands). To validate their content, we developed a Validity Inventory for Portfolio Assessment (VIPA) based on the CanMEDS roles. Two raters evaluated each portfolio and indicated for each VIPA item whether the portfolio provided sufficient information to enable satisfactory assessment of the item. We ran a descriptive analysis on the validation data and computed Cohen's Kappa to investigate interrater agreement.	This study sought to evaluate the content validity of portfolios as reflected in their capacity to adequately assess achieved competences of medical students during clerkships.	The portfolios adequately covered the items pertaining to the communicator (90%) and professional (87%) roles. Coverage of the medical expert, collaborator, scholar and manager roles ranged between 75% and 85%. The health advocate role, covering 59%, was clearly less well represented. This role also exhibited little interrater agreement (Kappa50.4).<br><br>This study lends further credence to the evidence that portfolios can indeed adequately assess the different CanMEDS roles during clerkships, the health advocate role, which was less well represented in the portfolio content, excepted.
49	Does a summative portfolio foster the development of capabilities such	Anthony J O'sullivan, Amanda C Howe, Susan Miles, Peter Harris, Chris S Hughes, Philip Jones, Helen	7.5		✓	✓	✓	UG	A questionnaire was designed to evaluate undergraduate medical students' experiences of	The aims of this study were (i) to determine whether preparing a portfolio helps promote students'	Students perceive portfolio preparation as an effective learning tool for the development of capabilities such as understanding ethical and legal principles

For peer review only

No.	Title	Author/year	MERSQI	COREQ	Reason for inclusion			Undergraduate (UG)/Postgraduate (PG)	Methodology	Purpose of study	Key findings
					Communication	Ethics	Professionalism				
	as reflective practice and understanding ethics? An evaluation from two medical schools	Scicluna & Sam J Leinster<br><br>2012							completing a portfolio at two medical schools. A total of 526 (45% response rate) students answered the on-line questionnaire. Students from both medical schools gave the highest ranking for the portfolio as a trigger for reflective practice. 63% of students agreed their portfolio helped them develop reflective practice skills (p < 0.001), whereas only 22% disagreed. 48% of students agreed portfolios helped them understand ethical and legal principles whereas 29% disagreed (p < 0.001). In contrast, only 34% of students thought the portfolio helped them to develop effective communication.	development in a range of capabilities including understanding ethical and legal principles, reflective practice and effective communication, and (ii) to determine to what extent the format of the portfolio affected the outcome by comparing the experiences of students at two different medical schools.<br><br>Therefore, the primary aim of this study was to determine whether the process of preparing a portfolio report or essay helped students appreciate and develop in a range of capabilities, such as understanding ethical and legal principles and reflective practice; capabilities which are not the focus of traditional assessments such as clinical or written examina- tions. The format of the portfolio assessment was different in each of the two medical schools in the study, and so a secondary aim was to determine to what extent the format of the assessment affected the outcome by comparing the student experiences with their portfolio	and reflective practice, whereas other capabilities such as effective communication require complementary techniques and other modes of assessment.

For peer review only

No.	Title	Author/year	MERSQI	COREQ	Reason for inclusion			Undergraduate (UG)/Postgraduate (PG)	Methodology	Purpose of study	Key findings
					Communication	Ethics	Professionalism				
										assessment in the two schools.	
50	Feasibility and Outcomes of Implementing a Portfolio Assessment System Alongside a Traditional Grading System	Celia Laird O'Brien, PhD, Sandra M. Sanguino, MD, MPH, John X. Thomas, PhD, and Marianne M. Green, MD<br><br>2016	7		✓		✓	UG	In 2009, the authors developed a portfolio system that served as a repository for all student assessments organized by competency domain. Five competencies were selected for a preclerkship summative portfolio review. Students submitted reflections on their performance. In 2014, four clinical faculty members participated in standard-setting activities and used expert judgment and holistic review to rate students' competency achievement as "progressing toward competence," "progressing toward competence with some concern," or "progressing toward competence pending remediation." Follow-up surveys measured students' and faculty members'	This study describes the development and implementation of a longitudinal competency-based electronic portfolio system alongside a graded curriculum at a relatively large U.S. medical school.	Faculty evaluated 156 portfolios and showed high levels of agreement in their ratings. The majority of students achieved the "progressing toward competence" benchmark in all competency areas. However, 31 students received at least one concerning rating, which was not reflected in their course grades. Students' perceptions of the system's ability to foster self-assessment were mixed.

For peer review only

No.	Title	Author/year	MERSQI	COREQ	Reason for inclusion			Undergraduate (UG)/Postgraduate (PG)	Methodology	Purpose of study	Key findings
					Communication	Ethics	Professionalism				
									perceptions of the process.		
51	Feelings related to first patient experiences in medical school A qualitative study on students' personal portfolios	Kaisu H. Pitkälä a,⁎, Taina Mäntyranta b<br><br>2003? 2004		14	✓		✓	UG	Twenty-two volunteer third and fourth year medical students (15 women and 7 men) of the University of Helsinki participated in a portfolio course for 1 year. Their reflective learning diaries and writings on specific themes were analyzed by qualitative content analysis	Feelings and thoughts of medical students related to first patient experiences during the first clinical year were examined	First patient encoun- ters were strong emotional experiences for medical students. The first patient examination was often described as an anxiety-provoking and confusing incident. Other emotionally significant encounters included helplessness when faced with serious illness and death, and role confusion when examining patients of one's own age but opposite sex. Students felt guilty for using patients for their own learn- ing purposes. Portfolios as learning tools may help in recognizing key experiences and support professional development of medical students.
52	Linking assessment to undergraduate student capabilities through portfolio examination	Anthony J. O'Sullivan , Peter Harris , Chris S. Hughes , Susan M. Toohey , Chinthaka Balasooriya , Gary Velan , Rakesh K. Kumar & H. Patrick McNeil<br><br>2012	8.5		✓	✓	✓	UG	Students are required to complete assessments linked to graduate capabilities. In Year 3, a portfolio review occurs (205–248 students per year), focusing on students' grades and feedback from assessments and a reflective essay is submitted. In the essay, students reflect on their progress, identify areas of weakness and detail plans for improvement. Progress in each capability is summatively graded against	This article describes an e-portfolio which closely aligns learning and reflection to graduate capabilities, incorporating features that address concerns about portfolios	Portfolios are an established method of assessment, although concerns do exist around their validity for capabilities such as reflection and self-direction

For peer review only

No.	Title	Author/year	MERSQI	COREQ	Reason for inclusion			Undergraduate (UG)/Postgraduate (PG)	Methodology	Purpose of study	Key findings
					Communication	Ethics	Professionalism				
									specific criteria and feedback is provided.		
53	Medical student resilience strategies: A content analysis of medical students' portfolios	Richard A. Prayson · S. Beth Bierer · Elaine F. Dannefer<br><br>2016		12	✓	✓	✓	UG	We retrospectively reviewed a sampling of year 1, 2 and 5 portfolio essays focused on the Personal Develop- ment competency and performance milestones, written by 49 students from three different classes in a 5-year pro- gramme devoted to training physician investigators. Two medical educators used a framework established by Jensen and colleagues (2008) to identify the nature and prevalence of various resilience strategies (valuing the physician role, self-awareness, personal arena, professional arena, profes- sional support and personal support) medical students re- ported in portfolio essays.	A student's ability to employ resilience strategies to self-regulate behaviour is critical to the student's future career as a physician. (The aim is to examine this??)	All students documented at least one strategy in their essays each year. In all years, the most commonly documented strategies were in the personal arena (95.7% of year 1, 98% of year 2 and 87.8% of year 5 portfolios). The least frequently documented strategy in all years was professional support (42.8% of year 1, 38.8% of year 2, and 28.6% of year 5 portfolios). Year 5 portfolios discussed personal support strategies (79.6%) more frequently than year 1 (53.1%) and year 2 (59.2%) portfolios.<br><br>The results suggest that medical students can identify stressors and articulate resilience strategies that can be employed to potentially address them.
54	Portfolio Assessment during Medical Internships: How to Obtain a Reliable and Feasible	NRM Michels, EW Driessen, AMM Muijtjens, LF Van Gaal, LL Bossaert, BY De Winter<br><br>2009		9.5			✓	UG	The domain-oriented reliability of 61 double-rated portfolios was measured, using a generalisability	In this study, we investigated whether assessment during medical internship by a portfolio can combine reliability and feasibility.	We obtained reliability (Φ coefficient) of 0.87 with this internship portfolio containing 15 double-rated tasks. The generalisability analysis showed that an acceptable level of reliability

For peer review only

No.	Title	Author/year	MERSQI	COREQ	Reason for inclusion: Communication	Reason for inclusion: Ethics	Reason for inclusion: Professionalism	Undergraduate (UG)/Postgraduate (PG)	Methodology	Purpose of study	Key findings
	Assessment Procedure?								analysis with portfolio tasks and raters as sources of variation in measuring the performance of a student.		(Φ = 0.80) was maintained when the amount of portfolio tasks was decreased to 13 or 9 using one and two raters, respectively.<br><br>Our study shows that a portfolio can be a reliable method for the assessment of workplace learning. The possibility of reducing the amount of tasks or raters while maintaining a sufficient level of reliability suggests an increase in feasibility of portfolio use for both students and raters.
55	Twelve tips for introducing E-Portfolios with undergraduate students	Alis Moores & Maria Parks<br><br>2010	-	-	✓			UG	-	-	-
56	Use of Portfolios in Undergraduate Medical Training: First Meeting With a Patient	Nilgün ÖZÇAKAR, Vildan MEVSİM, Dilek GÜLDAL<br><br>2009		10	✓		✓	UG	We designed a portfolio that was aimed at making reflection in early undergraduate medical education. Conditions for portfolio use were identified through interviews with six trainers who were experienced in mentoring students in the process of developing their portfolios. Analysis of writings was per- formed using qualitative content analysis.	Portfolios, as learning tools, are becoming increasingly important in medical education. Our aim was to evaluate the contents of portfolios used in medical education.	The conditions for successful reflective use of portfolios that emerged from the interviews fell into catego- ries: practice evaluation, patient perspective and primary prevention/health promotion. According to the students, working with a portfolio designed to meet these conditions is very useful and will stimulate students' abilities. The stu- dents shared the same opinion that analyzing one's com- petences in a portfolio was instructive and meaningful.<br><br>According to our students, portfolios are a potentially valuable method of developing students' skills in undergraduate medical education, provided that certain conditions for effective portfolios are recognized and met. The portfolio is a worthwhile addition to existing

For peer review only

No.	Title	Author/year	MERSQI	COREQ	Reason for inclusion			Undergraduate (UG)/Postgraduate (PG)	Methodology	Purpose of study	Key findings
					Communication	Ethics	Professionalism				
											assessment and learning tools.
57	What Is the Relationship Between a Preclerkship Portfolio Review and Later Performance in Clerkships?	Celia Laird O'Brien, PhD, John X. Thomas Jr, PhD, and Marianne M. Green, MD<br><br>2018	9.5		✓		✓	UG	The authors divided students into two groups based on a summative preclerkship portfolio review in 2014: students who had concerning behavior in one or more competencies and students progressing satisfactorily. They compared how students in these groups later performed on two clerkship outcomes as of October 2015: final grades in required clerkships, and performance on a clerkship clinical composite score. They used Mann–Whitney tests and multiple linear regression to examine the relationship between portfolio review results and clerkship outcomes. They used USMLE Step 1 to control for knowledge acquisition.	This study examines whether a preclerkship portfolio review is a valid method of identifying problematic student behavior affecting later performance in clerkships.	Students with concerning behavior preclerkship received significantly lower clerkship grades than students progressing satisfactorily ($P = .002$). They also scored significantly lower on the clinical composite score ($P < .001$). Regression analysis indicated concerning behavior was associated with lower clinical composite scores, even after controlling for knowledge acquisition.<br><br>The results show a preclerkship portfolio review can identify behaviors that impact clerkship performance. A comprehensive portfolio system is a valid way to measure behavioral competencies.
58	Educating undergraduate medical students about oncology: A literature review	Cave, Judith<br><br>2006	-	-	✓			UG	MEDLINE, Psychinfo, ERIC, TIMELIT, EMBASE, CINAHL and the Cochrane CENTRAL Register of	This article is a review of the literature regarding teaching oncology to undergraduate medical students.	The main findings can be summarized as follows: the involvement of patients in teaching is popular with students and portfolio learning is a successful way of involving patients; the use of standardized patients to

No.	Title	Author/year	MERSQI	COREQ	Reason for inclusion			Undergraduate (UG)/Postgraduate (PG)	Methodology	Purpose of study	Key findings
					Communication	Ethics	Professionalism				
									Controlled Trials (CENTRAL) were searched, using the search terms cancer, oncology, education, undergraduate, and teaching.		teach breast examination improves students' performance in clinical assessment; the use of silicone models to teach breast examination improves students' sensitivity for detecting breast lumps; computer aided learning modules have a role, but are not superior to other types of learning; learning about cancer screening and prevention increases students' knowledge, improves their self rated skills, and changes their behavior; and cancer patients have an important role to play in teaching undergraduate communication skills.<br><br>We have found 48 articles on undergraduate teaching in oncology. Oncology teachers should consider adopting the evidence based approaches outlined in this review, and there should be more emphasis on educational research within the field of oncology.
59	Integrating the assessment of interprofessional education into the health care curriculum	Anderson, E. S. and Kinnair, D.<br><br>2016	-	-	✓	✓	✓	UG	-	Reflection on some of the challenges associated with assessment in IPE by describing the experiences at one UK-based medical school	In regard to written reflections, reading 240 student entries per learning event is time-consuming, making<br>it challenging to offer individual feedback. Some students have challenged the formative outcomes that were graded for progression across broad boundaries of unsatisfactory, satisfactory or excellent. Checking for consistency across a small numbers of marker illuminates the subjective perspectives and interpretations of individual

For peer review only

No.	Title	Author/year	MERSQI	COREQ	Reason for inclusion			Undergraduate (UG)/Postgraduate (PG)	Methodology	Purpose of study	Key findings
					Communication	Ethics	Professionalism				
											markers on what constitutes a meaningful reflection. Nevertheless, offering this early writing platform has proved to be extremely helpful and enables educators to determine if students can reflect critically and apply meaning
60	A model of engagement in reflective writing-based portfolios: Interactions between points of vulnerability and acts of adaptability	Arntfield, S., Parlett, B., Meston, C. N., Apramian, T. and Lingard, L.<br><br>2015	7	21			✓	UG	Using mixed-methods rooted in grounded theory, 139 students and 13 mentors completed questionnaires, 23 students participated in four focus groups and 9 mentors in individual interviews	The aim of this study was to explore conditions affecting the experience of teaching and learning from the perspective of both students and mentors in a reflective writing-based portfolio initiative.	The overarching theme in our data was student–mentor engagement. Our results confirm previous literature describing portfolio as a vulnerable method of learning, extend this concept by identifying and categorizing specific points of vulnerability, and contribute new knowledge regarding acts of adaptability, which serve to strengthen the student–mentor relationship.<br><br>Engagement is central to the success of portfolio and is shaped by a dynamic interaction between points of vulnerability and acts of adaptability. We propose a model of engagement in portfolio that can be used for faculty development to optimize student–mentor engagement.
61	Support for portfolio in the initial years of the undergraduate medical school curriculum: what do the tutors think?	Austin, C. and Braidman, I.<br><br>2008	7	18	✓		✓	UG	A mixed method approach was used with data collected from both questionnaires and a focus group meeting.	The aims of this study were to investigate the views of these tutor facilitators on the delivery and support of portfolios in this way.	Tutor facilitators were generally positive about their roles in the support of portfolio development in group sessions and identified several advantages to this type of tutoring – namely the value of group discussion and discussion between peers to encourage reflection, and the practical ability to integrate portfolio sessions more closely with clinical experiences – which would not be possible in one to one tutor/student meetings.

For peer review only

No.	Title	Author/year	MERSQI	COREQ	Reason for inclusion			Undergraduate (UG)/Postgraduate (PG)	Methodology	Purpose of study	Key findings
					Communication	Ethics	Professionalism				
											With the training and guidance notes provided tutors were confident that they understood the support they should give students and felt that they could encourage reflection.<br><br>Thus, tutors perceived that the support of portfolio in groups is an effective way of developing portfolios for large numbers of students.
62	Use of portfolios by medical students: significance of critical thinking	Azer, S. A.<br><br>2008	-	-	✓		✓	UG	-	The aims of this paper are to: (1) understand the uses of portfolios and the rationale for using reflection in the early years of a PBL curriculum; (2) discuss how to introduce portfolios and encourage students' critical thinking skills, not just reflection; and (53) provide students with tips that could enhance their skills in constructing good portfolios.	-
63	Time to Loosen the Apron Strings: Cohort-based Evaluation of a Learner-driven Remediation Model at One Medical School	Bierer, S. B., Dannefer, E. F. and Tetzlaff, J. E.<br><br>2015	10		✓			UG	We used a retrospective, cohort-based approach to examine our learner-driven remediation model.<br>Data sources: We consulted MSPRC records to identify all CCLCM students from six class cohorts (2009–2014) placed in remediation during medical school, and compared remedial students	To examine a remediation model where students, rather than faculty, develop remedial plans to improve performance.<br>We present an innovative, systematic approach to remediation purposefully designed to engage learners with the development and implementation of remedial plans to improve their performance. We first describe our approach to	From abstract -- participants included 177 students from six classes (2009–2014). Twenty-six were placed in remediation, with more referrals occurring during Years 1 or 2 (n = 20, 76 %). Unprofessional behavior represented the most common reason for referral in Years 3–5. Remedial students did not differ from classmates (n = 151) on baseline characteristics (Age, Gender, US citizenship, MCAT) or willingness to recommend their medical school to future students ($p < 0.05$). Two remedial students did not graduate and three did

For peer review only

No.	Title	Author/year	MERSQI	COREQ	Reason for inclusion			Undergraduate (UG)/Postgraduate (PG)	Methodology	Purpose of study	Key findings
					Communication	Ethics	Professionalism				
									to their classmates not placed in remediation on variables collected for program evaluation purposes: Baseline characteristics (gender, age, US citizenship, MCAT score), USMLE licensure exam performance (pass-rate for each student), National Residency Matching Program data (clinical specialty for each student), Satisfaction with medical school (collected from graduating students using questionnaire). Then, we read MSPRC correspondence to each remedial student to extract referral mechanism (portfolio-based decision or faculty-initiated), unsatisfactory competency domain(s), remediation duration (days), and completion of students' remediation goals. Finally, we read students' remedial plans to identify strategies they proposed to	remediation and then report outcomes for students who were and were not placed in remediation during medical school. We conclude with lessons learned and future directions.	not pass USLME licensure exams on first attempt. Most remedial students (92 %) generated appropriate plans to address performance deficits. Students can successfully design remedial interventions. This learner-driven remediation model promotes greater autonomy and reinforces self-regulated learning. From results: Participants include 177 of 187 medical students from six class cohorts (2009–2014) who consented (95 %) to release program evaluation data for research purposes. Of these, the MSPRC placed 26 students (17 men and nine women) in formal remediation. Half (13) were identified by the MSPRC for not meeting year-end performance standards assessed via portfolios. The dean of student affairs referred the remaining 13 students based on faculty recommendation. We did not detect significant differences between remedial students and their classmates for baseline characteristics (Table 1). Overall, three-quarters of remediation decisions occurred during the first (10, 38 %) or second (10, 38 %) year of medical school. With regard to specific domains in which students failed to meet year-end standards or were otherwise referred, professionalism (18, 69 %), communication (10, 38 %), research (9, 35 %), and reflective practice (5, 19 %) represent frequent domains of unsatisfactory performance. Fewer students did not meet

For peer review only

No.	Title	Author/year	MERSQI	COREQ	Reason for inclusion			Undergraduate (UG)/Postgraduate (PG)	Methodology	Purpose of study	Key findings
					Communication	Ethics	Professionalism				
									the MSPRC to address performance deficits in specific competency areas. We manually recorded a subset of these strategies to provide examples of students' approaches to remediation. All performance decisions, referral mechanisms, and outcomes were manually entered into SPSS. Chi-square and ANOVA tests were used to compare baseline characteristics and satisfaction scores of the two student groups (remediation vs. other), while the Mann Whitney U test was used to examine if remediation duration (days) differed by students' gender. Statistical significance was established at p < 0.05 for all comparisons.		performance standards for clinical reasoning (4, 15 %), medical knowledge (3, 11 %), clinical skills (2, 8 %), personal development (2, 8 %), or health care systems (2, 8 %). Unprofessional behavior (e.g., not logging patient encounters, unexcused absences, chronic tardiness, etc.) represented the most common reason for student remediation during Years 3–5 of medical school. Ten students had deficient performance in one competency, while others had unsatisfactory performance in two (7, 27 %) or more (9, 35 %) competencies. Three students were placed in remediation twice, with one repeating for unprofessional behavior. Time in remediation (determined by dates in MSPRC correspondence to students) ranged from 39 to 1696 days (Median = 448 days) and did not differ by student gender (p > 0.05). Remediation plans revealed various student-proposed strategies to address TAFIs for multiple competency domains (Table 2). We observed that students' remedial strategies resembled approaches faculty would recommend. We also observed the MSRPC's correspondence to students consistently had an encouraging, supportive tone for students' remedial efforts (See Appendices for MSPRC's correspondence to one remedial student where committee asks student to develop a remediation plan, comments

For peer review only

No.	Title	Author/year	MERSQI	COREQ	Reason for inclusion			Undergraduate (UG)/Postgraduate (PG)	Methodology	Purpose of study	Key findings
					Communication	Ethics	Professionalism				
											on student's progress report, and releases student from remediation). The MSPRC "prescribed" remediation plans for two students only after these students did not provide adequate assessment evidence in progress reports. The MSPRC determined that most students (24, 92 %) generated sufficient evidence (predominately obtained from faculty and peers) to document their competence. Table 1 reveals that some remedial students experienced negative outcomes, as two did not graduate and three did not pass USMLE licensure exams on first attempt. Proportionally, more remedial students (11, 46 %) pursued surgical specialties than their classmates (50, 33 %). Table 1 also shows graduating remedial students were just as likely as their classmates to recommend the CCLCM program to future students, even though fewer were as satisfied with their overall experience at CCLCM as their classmates. From discussion -- Most programs struggle with how to identify and assist learners requiring remediation. We explored if students, rather than faculty, can take ownership for remedial plan design and implementation. We review key findings and conclude with future directions. The locus of control for selecting remedial interventions and assessment evidence typically rests with faculty. In

No.	Title	Author/year	MERSQI	COREQ	Reason for inclusion			Undergraduate (UG)/Postgraduate (PG)	Methodology	Purpose of study	Key findings
					Communication	Ethics	Professionalism				
											our model, the MSPRC charged students to develop remediation plans, with PA guidance as needed, and obtain assessment evidence to document progress and achievement. Most remediating students (92 %) successfully met these expectations and graduated from medical school. Three-quarters of remedial students were identified in Years 1–2 of medical school. Early identification provides opportunities for multiple, tailored interventions to occur. Professionalism, communication skills, and research performance were frequent reasons the MSPRC placed students in remediation. Few students were identified for gaps in medical knowledge or clinical reasoning. The literature suggests some competencies, like professionalism, require direct observation and ongoing assessment for learner success.1,18 Our students' remedial strategies (Table 2) ranged from specific to holistic approaches and often cut across courses and contexts, thereby providing them with more opportunities to improve and document their performance in complex domains. This emphasis on competence may explain why students' time in remediation was longer (Median = 448 days) than normally needed for "more of the same," such as retaking a test or course.2 Some learners may not know how to navigate performance obstacles,

For peer review only

No.	Title	Author/year	MERSQI	COREQ	Reason for inclusion			Undergraduate (UG)/Postgraduate (PG)	Methodology	Purpose of study	Key findings
					Communication	Ethics	Professionalism				
											making it important to offer guidance and oversight.19,20 For this reason, we train a core group of faculty to help students apply the self-regulation cycle.17 PAs coach students in reflection, self-assessment, and goal-setting.2,20,21 They also form long-term relationships with students and frequently serve as student advocates. The MSPRC encourages students to identify measurable outcomes to document competence, while offering positive support in written correspondence to students (See Appendices for series of letters to one student throughout the remediation process). Both faculty groups work in tandem to support this learner-driven remediation model while ensuring student accountability. Our evaluation is limited to one program, at one school, with a small class size. Other programs may foster remedial learners' autonomy more than conveyed in the literature.5,6,8 Our student satisfaction measures originate from two questionnaire items obtained at graduation and do not capture students' satisfaction with specific academic referral policies or remedial processes.22 Remediation is complex and involves multiple stakeholders and contexts. Future studies should capture learners' perceptions of remediation to complement the faculty point-of-view currently reported in the literature.

For peer review only

No.	Title	Author/year	MERSQI	COREQ	Reason for inclusion			Undergraduate (UG)/Postgraduate (PG)	Methodology	Purpose of study	Key findings
					Communication	Ethics	Professionalism				
											We discovered that more men than women were identified for remediation, yet we found no research exploring this observation in depth. Additionally, few longitudinal or multi-institutional studies currently exist, suggesting an area ripe for research. We discovered, in our 10-year experience with this remediation model, that medical students can select appropriate remedial interventions with the support of well-trained faculty. Our learner-driven approach to remediation may transfer to graduate medical education (GME), where Clinical Competence Committees (CCCs) meet regularly to discuss and review resident performance. Having struggling residents create remediation plans and submit progress reports to CCCs may present few barriers, given GME's competency-based assessment framework and emphasis on providing residents with frequent, formative feedback. Our approach requires a learning environment where faculty must trust students have the motivation and capabilities to identify and implement appropriate remedial strategies. We have created a systematic process where students must reflect on their performance, develop plans tailored to meet learning needs, and collect assessment evidence to document competence. To conclude, we believe educators who wish to

For peer review only

No.	Title	Author/year	MERSQI	COREQ	Reason for inclusion			Undergraduate (UG)/Postgraduate (PG)	Methodology	Purpose of study	Key findings
					Communication	Ethics	Professionalism				
64											adopt a learner-driven approach to remediation should view remediation as a growth opportunity rather than as a punishment.
64	Medical professionalism: conflicting values for tomorrow's doctors	Borgstrom, E., Cohn, S. and Barclay, S.<br><br>2010		14		✓		UG	From abstract: Qualitative study using interpretative discourse analysis of anonymized student reflective portfolios. One hundred twenty-three final year undergraduate medical students (64 male and 59 female) from the University of Cambridge School of Clinical Medicine supplied 116 portfolios from general practice and 118 from hospital settings about patients receiving palliative or end of life care.<br>From introduction: This study investigates how professionalism is understood and experienced via one such initiative for medical students training in Cambridge, England, and highlights the conflicts between 'new' and 'old' values.<br>From methods: Final year medical students in Cambridge meet patients approaching the	To investigate how final year medical students experience and interpret new values of professionalism as they emerge in relation to confronting dying patients and as they potentially conflict with older values that emerge through hidden dimensions of the curriculum.	From abstract -- Results: Professional values were prevalent in all the portfolios. Students emphasised patient-centered, holistic care, synonymous with a more contemporary idea of professionalism, in conjunction with values associated with the 'old' model of professionalism that had not be directly taught to them. Integrating 'new' professional values was at times problematic. Three main areas of potential conflict were identified: ethical considerations, doctor-patient interaction and subjective boundaries. Students explicitly and implicitly discussed several tensions and described strategies to resolve them. Conclusions: The conflicts outlined arise from the mix of values associated with different models of professionalism. Analysis indicates that 'new' models are not simply replacing existing elements. Whilst this analysis is of accounts from students within one UK medical school, the experience of conflict between different notions of professionalism and the three broad domains in which this conflict arises are relevant in other areas of medicine and in different national contexts.<br>From discussion -- All of the students encountered challenges that required

For peer review only

No.	Title	Author/year	MERSQI	COREQ	Reason for inclusion			Undergraduate (UG)/Postgraduate (PG)	Methodology	Purpose of study	Key findings
					Communication	Ethics	Professionalism				
									end of life during general practice (GP) and hospital attachments. As required coursework they write portfolio items on two patients, one from each attachment. Key to this exercise is integrating issues arising from the interview with reflections of their own personal experiences. This study focuses on how different ideas are received and reproduced by a relatively young (typically early 20s) cohort of soon-to-be qualified doctors during the academic year 2007–2008. The University of Cambridge Psychology Research Ethics committee approved the study, and 86% of students (n = 123; 64 male, 59 female) gave informed consent for 234 reflective items: 116 from the GP and 118 from hospital settings. Each student was assigned a random identification number (ID), which is included		them to try and balance values characteristics of 'old' and 'new' forms of medical professionalism. Ethics, interaction and managing subjective boundaries all generated areas of conflict that they wrestled with in their assignments. Students addressed these tensions in a variety of ways, with strategies drawn from both formally taught skills and personal resources. In parallel with shifts from 'old' to 'new' values of professionalism, conflicts between the formal and the hidden curricula may also generate tensions44. In our study, the formal curricula is explicitly designed to embrace the 'new', leaving the 'old' to be communicated through more informal and hidden aspects of teaching. Yet for the students trying to personally reflect on their experiences and summatively draw from their entire educational experience for the exercise, no distinction is made between these two domains. Large organisations like the University of Cambridge Clinical School and the National Health Service have institutional memories45—collective experiences and concepts. These are communicated imperceptibly through teaching elements as well as the very structures and policies that shape the educational environment. This feature serves to complicate understandings of medical education; it

For peer review only

No.	Title	Author/year	MERSQI	COREQ	Reason for inclusion			Undergraduate (UG)/Postgraduate (PG)	Methodology	Purpose of study	Key findings
					Communication	Ethics	Professionalism				
									with the quotes to demonstrate the breadth of the dataset presented. Analysis focused on the topic of 'professionalism', viewing the content of the portfolios as representative of general values imparted to students throughout their education. All items were coded and analysed in NVivo 8 using an interpretative approach. Following the social science traditions of two of the authors (EB and SC), the methodology employed a hermeneutic approach, striving to find meaning beyond straightforward discrete references35. Accordingly, rather than assuming the meaning of isolated sections of text in a reductive manner, they were always analysed in context and in relation to one another. Given the difficulty many students had in addressing the topic of end of life care, and		emphasises the extent to which values are embedded in the routines and practices of the organisation, and cannot therefore be simply addressed through redesigning curricula or individuals championing change. Our study suggests that tensions arise primarily because of different values that underlie the concept of professionalism throughout their education, rather than anything that is specific to end of life care. Whilst topics central to the everyday practice of medicine are particularly evident in palliative care46, they are made highly visible by students who see themselves on the cusp of becoming members of the profession themselves. Whilst it is likely that the concept of professionalism always has contained a wide variety of underlying values and principles that are not always commensurate with each other, we have argued that a more widespread shift in values over recent years has generated greater variance and hence more contradictory positions around what it means to be a good doctor. Our findings add to the literature on medical professionalism, which is rich in doctors' anecdotal experiences47,48, highlights the stresses and conflicts doctors face44,49,50 and illustrates a current amalgamation of definitions with conflicting values51. Specific national and local contexts are likely to generate differently

No.	Title	Author/year	MERSQI	COREQ	Reason for inclusion			Undergraduate (UG)/Postgraduate (PG)	Methodology	Purpose of study	Key findings
					Communication	Ethics	Professionalism				
									consequently the very discursive descriptions of their observations, the authors were committed to code more than just those phrases in which a theme was referred to explicitly, and so included concerns that were expressed less directly. A coding scheme was derived from initial independent item analysis (EB, SC and SB) and adjusted through discussion. It was initially applicable to explicit statements by drawing on key words and then was augmented to include the more implicit references that potentially alluded more to the hidden dimensions of their teaching (see Box 2). The inclusion of these required careful interpretative readings of the entire text but provided an essential contribution to our overall analysis. The first author (193) then led on-going analysis, with regular meetings to		nuanced versions of these issues, which might only be identified through comparative work. From conclusion: The integration of 'new' professional values taught in medical school is at times problematic for students in any health care system that maintains elements, whether overtly or not, of the 'old' paradigm. The areas of potential conflicts outlined—ethics, patient interaction and managing subjective boundaries—are not limited to medical school or end of life care. Our analysis suggests that 'old' variants of professionalism are not simply being replaced by 'new' ones delivered by a redesigned curriculum, but rather that values from each can emerge in a range of medical contexts. As a result, professionalism does not consist of a set of fixed or abstract concepts, but rather surfaces through medical practice. If individual reflection is now heralded as an essential component of the 'new' professionalism, as indicated by the compulsory student exercise we have drawn on here, it should be acknowledged that it demands a dynamic engagement with the wide range of often contradictory and shifting ideas and beliefs from both formal and more hidden aspects of their education. This study illustrates that overt commitment to more empathic and patient-centered approaches to medical care do not

For peer review only

No.	Title	Author/year	MERSQI	COREQ	Reason for inclusion			Undergraduate (UG)/Postgraduate (PG)	Methodology	Purpose of study	Key findings
					Communication	Ethics	Professionalism				
									discuss emerging themes.		necessarily replace other more prescribed values and behaviours that remain part of a hidden curriculum embedded in institutional practices. Integration of 'new' core values and skills into good medical practice is not a smooth or simple transition. Additionally, it seems any simple attempt to communicate them through formal teaching is unlikely to prepare students for the reality of medical encounters. Instead, the experience of tension and the individual desire to seek balance and resolution across a wide range of issues may themselves be key and lasting features of medical professionalism.s
65	Facilitating students' reflective practice in a medical course: literature review	Chaffey, L. J., de Leeuw, E. J. and Finnigan, G. A.<br><br>2012		12			✓	UG	The literature search was undertaken using the online databases of CINAHL and Medline. Keyword searching was conducted using MeSH headings, Boolean operators, and the terms, 'medical students', 'medical education', 'reflection', 'reflect*' and 'medicine'. The search was limited to peer-reviewed published material in English and between the years 2001 and 2011 to maintain currency of	The aim of this review is to summarise the literature published around facilitating reflection in a medical course, and to answer the question : What is the current evidence regarding learning and development moments across the medical curriculum in developing students' reflective practice?	From abstract -- Discussion: A variety of reflective purposes was found in this literature review. Evidence indicates that, if students are unclear as to the purpose of reflection and do not see educators modelling reflective behaviours, they are likely to undervalue this important skill regardless of the associated learning and development opportunities embedded in the curriculum. From results: Thirty-six relevant articles were found. These articles were classified by methodology (see [Table 1]. Relevant references identified). As can be seen in [Table 1], this review included many opinion pieces, reviews and descriptions of methods of facilitating reflection, but little in regards to primary data in the area. Qualitative methodology was the main research output, providing largely descriptive data of

For peer review only

No.	Title	Author/year	MERSQI	COREQ	Reason for inclusion			Undergraduate (UG)/Postgraduate (PG)	Methodology	Purpose of study	Key findings
					Communication	Ethics	Professionalism				
									practice. The primary search yielded 129 references of eligible papers, including research papers, reviews, formal reviews and opinion pieces. Abstracts were read by the first author, and only articles pertaining to facilitating or teaching reflection in undergraduate or postgraduate medical courses were included. The first author read and classed all included articles, categorising them into construct sub-categories. At several stages in this process all authors reviewed the validity and legitimacy of the classifications. This led to the sub-headings and analysis presented below.		the facilitation of reflection. The quantitative research was also largely descriptive, with survey methodology being the most popular in the articles found. This review found only two case-control studies comparing facilitation methods of reflection. The literature presented enhancing factors and barriers to effectively teaching reflective practice within medical curricula, related to: The breadth of the meaning of reflection; Facilitating reflection by medical educators; Using written or web-based portfolios to facilitate reflection; and Assessing the reflective work of students.<br>- The breadth of the meaning of reflection: The term 'reflection' is widely mentioned within medical education literature, but the purposes and goals described indicate a breadth of purposes of reflection. [14] Mann et al.[10] concluded from their systematic review of reflective practice in the health professions that the nature of reflection makes it difficult to quantify. From this current review, we determined that the breadth of reflection covers the purposes of reflecting to improve judgements, to personally develop and, and to contextualise practice, and are explained in [Table 2]. Reflection to improve judgement centres on improving students' clinical skills, problem solving, [11] flexible thinking, lifelong learning and the development of expertise.

No.	Title	Author/year	MERSQI	COREQ	Reason for inclusion			Undergraduate (UG)/Postgraduate (PG)	Methodology	Purpose of study	Key findings
					Communication	Ethics	Professionalism				
											[1],[10],[14],[17] Reflection for this purpose usually has a problem focus, involves critical thinking, and is commonly indicated by hypothesis testing. Reflection to personally develop is essential in client-centred practice, as doctors need to consider they own values and assumptions, and those of the patient, while making clinical decisions. [18] From reviewing the literature, Sandars [14] asserted that the knowledge of one's values and beliefs is important for a doctor to develop therapeutic relationships with patients, as understanding oneself enables empathy and caring. Aukes et al.[11] reviewed literature in preparation for creating an assessment tool for reflection, and noted that personal reflection has often been overlooked in medical education. They suggested that this has occurred as personal development and has been seen as outside the domain of the problem-solving culture of medical practice, and thus, outside the domain of medical education. Reflection to contextualise practice helps students integrate theory with their own experience. Several authors have hypothesised that this aspect of reflection is essential in developing a sound medical practice, [5] with a lack of reflective ability resulting in poor self-awareness, and thus poor medical practice. [19] Howe et al.[8] found, from their study of the development of

For peer review only

No.	Title	Author/year	MERSQI	COREQ	Reason for inclusion			Undergraduate (UG)/Postgraduate (PG)	Methodology	Purpose of study	Key findings
					Communication	Ethics	Professionalism				
											professionalism, that reflecting on practice and developing self-awareness are important mechanisms of resilience in the face of a demanding profession. Although this breadth of reflective practice makes it an essential skill in medical practice, this very strength can also be a barrier in teaching facilitating reflective practice as there is often ambiguity of the educator's goals in regard to the use of a particular reflective task. [20],[21] Aukes et al.[11] noted that purposes of a reflective tasks were rarely articulated, and students were often unaware of the perspective, reflective purpose or role, they were expected to take. Sanders [14] concluded from a literature review of reflection in medical education that, for students to reflect effectively, they required clarity in determining the appropriate purpose of a reflective task.<br>- Facilitation reflection by medical educators:<br>Reflection is often provoked by uncertainty or unease resulting from an experience. [13],[22] According to Albanese, [22] in addition to this uncertainty or unease, a student requires knowledge of how to reflect, and time and motivation for reflection to occur. Supporting this opinion, Carr and Carmody [23] found in their qualitative study that some students could attain an acceptable level of reflection independently. However, reflection was greatly

For peer review only

No.	Title	Author/year	MERSQI	COREQ	Reason for inclusion			Undergraduate (UG)/Postgraduate (PG)	Methodology	Purpose of study	Key findings
					Communication	Ethics	Professionalism				
											enhanced through medical educators' facilitation. Medical educators' facilitation was found to be useful for reflection in one-on-one meetings with students and in small group tutorials. [9],[24] According to results of a study by Schaub-de Jong et al., [9] educators who wished to encourage reflection needed to model reflective practice by making their experiences more explicit, investigating emotions and stimulating interaction among students. Albanese [22] suggested that medical faculty staff should be trained to articulate their reflections to enhance modelling to students. - Using written or web-based portfolios to facilitate reflection: Traditionally, reflective tasks have been paper based. Recently, web-based methods and social media have been adopted to facilitate reflective skills. Cook [29] asserted that there has been little study of the efficacy of these methods. Fischer et al. [30] compared content, depth of reflection and student preference for web-based versus traditional essay-style reflections in two medical schools in the USA. Ninety-five students were quasi-randomly assigned to one of two study groups, where one group wrote a traditional reflective essay and attended a small-group discussion, while the other group posted two writings in a group blog and commented on a peer's post. The writings were

For peer review only

No.	Title	Author/year	MERSQI	COREQ	Reason for inclusion			Undergraduate (UG)/Postgraduate (PG)	Methodology	Purpose of study	Key findings
					Communication	Ethics	Professionalism				
											coded for theme, and level of reflection, with the authors finding no difference between groups for either variable.<br>From discussion:<br>Traditionally, paper-based methods of reflection have been popular with educators, [7],[8],[25] but recently, web-based methods have been adopted. Driessen et al. [36] found that the method of reflection appears to be less important than the guidelines provided, which should be structured initially, and become more fluid as students' reflective skills progress. Ideally, students should be able to revisit and edit the reflection, hence web-based methods may be suitable for this purpose.<br>- Assessing the reflective work of students: ... (see relevant columns)<br>From discussion:<br>Several authors noted that reflection was an essential skill in medical practice, [2],[5],[7],[10],[14],[34] and advocated for training in reflective processes. [13],[22] Training in reflective practice can be significantly enhanced with the facilitation of skilled mentors, providing guidance and modelling of reflective behaviours, as found by Carr and Carmody. [23] Several studies and literature reviews report inconsistencies regarding the purpose of reflection, which was found to be a significant barrier to the facilitation of reflective practice. [11],[20],[21],[35] The implication for educators is that students

For peer review only

No.	Title	Author/year	MERSQI	COREQ	Reason for inclusion			Undergraduate (UG)/Postgraduate (PG)	Methodology	Purpose of study	Key findings
					Communication	Ethics	Professionalism				
											need to be given clear guidelines as to the purpose for each individual reflective task. Traditionally, paper-based methods of reflection have been popular with educators, [7],[8],[25] but recently, web-based methods have been adopted. Driessen et al. [36] found that the method of reflection appears to be less important than the guidelines provided, which should be structured initially, and become more fluid as students' reflective skills progress. Ideally, students should be able to revisit and edit the reflection, hence web-based methods may be suitable for this purpose. Students need to have the designated time and motivation for reflective practice. A common method of increasing task motivation in the education setting is to include the task as an assessment piece. There was some debate in the literature about the efficacy of this method. Some authors found that students' quality of reflection would be diminished if undertaken for an assessment task, [28] whereas others reported that reflection simply would not happen if not attached to an assessable piece of work. [22],[33] It is apparent from this review that the research surrounding the teaching and facilitation of reflection is generally weak. Although there are some descriptive data, there is very little empirical evidence of the efficacy of facilitation methods. Additionally, there

For peer review only

No.	Title	Author/year	MERSQI	COREQ	Reason for inclusion			Undergraduate (UG)/Postgraduate (PG)	Methodology	Purpose of study	Key findings
					Communication	Ethics	Professionalism				
											is a lack of rigorous research investigating the impact of reflection on practice skills or patient outcomes. A major limitation of literature is the breadth of the construct of reflection. A more defined construct of reflection, with clear outcomes, could lead to the development of benchmarks useful in tracking student progress and as research outcome measures. From conclusion: Most authors cited in this review suggested that the emphasis on reflection is increasing in medical curricula. The construct of reflection was broad in the reviewed literature. This suggests that medical educators must be clear on the purpose of reflection, the intended outcome for the individual student and the process necessary to achieve these aims for students. Evidence indicated that, if students are unclear as to the purpose of reflection and do not see educators modelling reflective behaviours, they are likely to undervalue this skill regardless of the associated learning and development opportunities embedded in the curriculum.[40]
66	Beyond assessment of learning toward assessment for learning: educating tomorrow's physicians	Dannefer, E. F. 2013	-	-	✓			UG	From abstract: Information-rich assessment data, structured opportunities for reflection, and facilitated self-assessment using a portfolio approach are designed to support	From abstract: We describe a program (based at Cleveland Clinic Lerner College of Medicine) in which explicit performance standards align these components and provide a roadmap for students to manage their learning.	From abstract: Preliminary evidence suggests that the program directs students towards learning, rather than on achieving a grade for grade's sake.

For peer review only

No.	Title	Author/year	MERSQI	COREQ	Reason for inclusion			Undergraduate (UG)/Postgraduate (PG)	Methodology	Purpose of study	Key findings
					Communication	Ethics	Professionalism				
									development of habits of reflective practice. Promotion depends on the achievement of competencies rather than grades. From the essential building blocks: All components and processes of a system need to be designed to interact in a way that accomplishes the goal of enhancing learning and habits of reflective practice. Key features of the CCLCM assessment system are now presented by addressing issues of competency standards, ongoing formative assessment, student responsibility, and purposeful support.		
67	Evidence within a portfolio-based assessment program: what do medical students select to document their performance?	Dannefer, E. F., Bierer, S. B. and Gladding, S. P.<br><br>2012	7		✓		✓	UG	From abstract: We reviewed portfolios to examine the number, type, and source of assessments selected by students (n = 32) to document their performance in seven competencies. The quality of assessment data selected for each	From abstract: In this article, we examine the nature of evidence selected by first-year medical students to include in a portfolio used to make promotion decisions. From introduction: This article attempts to address this gap in the literature by examining the types and sources of assessment	From abstract -- Results: Findings indicate that students cited multiple types and sources of available assessments. The promotion committee rated evidence quality highest for competencies where the program provided sufficient evidence for students to cite a broad range of assessments. When assessments were not provided by the program, students cited self-generated evidence.

For peer review only

No.	Title	Author/year	MERSQI	COREQ	Reason for inclusion			Undergraduate (UG)/Postgraduate (PG)	Methodology	Purpose of study	Key findings
					Communication	Ethics	Professionalism				
									competency was rated by promotion committee members (n = 14). From methods: We used two procedures to explore the assessment evidence that one cohort of medical students cited in their Year 1 summative portfolios. First, we examined the evidence cited in portfolio essays to ascertain what types and sources were selected. Second, we obtained feedback from promotion committee members concerning the quality of evidence via a questionnaire. - Citation coding procedures and data: A research assistant extracted essays from the e-Portfolio database for seven of the nine competencies included in the Year 1 summative portfolios that students submitted during the 2009–2010 academic year. Essays for the competencies of Personal Development and	evidence first-year medical students select from their performance database to construct portfolios used for promotion decisions. We describe our assessment program's evidence database before presenting the organizational elements designed to help students purposefully select data representative of their performance. We then describe the methods and results of the study before discussing implications about students' ability to select evidence within a centralized assessment program.	Conclusion: We found that when student-constructed portfolios are part of an overall assessment system, students generally select evidence in proportion to the number and types of assessments available.

No.	Title	Author/year	MERSQI	COREQ	Reason for inclusion			Undergraduate (UG)/Postgraduate (PG)	Methodology	Purpose of study	Key findings
					Communication	Ethics	Professionalism				
									Reflective Practice were excluded to ensure student confidentiality. Students' names on essays were replaced with a unique six-digit identifier. Overall, 224 essays for 32 students were reviewed for these seven competencies. Sophia Gladding sorted the essays by competency. She then read each set of essays nested within a given competency and manually recorded on a data extraction form all college-generated assessments, work samples and personal evidence students cited to document their performance of Year 1 standards. Extraction form data were entered into SPSS version 16.0, and descriptive statistics were computed after averaging across students' portfolios.<br>- Quality ratings: Promotions committee members involved with portfolio reviews (n = 18 faculty)		

No.	Title	Author/year	MERSQI	COREQ	Reason for inclusion			Undergraduate (UG)/Postgraduate (PG)	Methodology	Purpose of study	Key findings
					Communication	Ethics	Professionalism				
									completed a 21-item, web-based questionnaire after reading students' Year 1 summative portfolios. The questionnaire had been designed (Beth Bierer and Elaine Dannefer) and piloted earlier to monitor the Year 1 portfolio review process. This instrument included seven Likert-scaled items ranging from 1 "poor" to 4 "excellent" which asked promotion committee members to rate the overall quality of the assessment evidence they read for each competency after judging students' summative portfolios. Percentages were computed for each competency to summarize the committee's global ratings ("excellent" and "good/excellent") of evidence quality.		
68	The portfolio approach to competency-based assessment at the Cleveland Clinic Lerner College of Medicine	Dannefer, E. F. and Henson, L. C.<br><br>2007	-	-	✓		✓	UG	-	From abstract: This article describes how the portfolio was developed to provide both formative and summative assessment of student achievement in relation to the program's nine	Design of the portfolio assessment system: The search for an assessment system consistent with our principles led us to the literature on portfolios for medical student assessment14 –15,21–23 and to firsthand investigation of experiences

For peer review only

No.	Title	Author/year	MERSQI	COREQ	Reason for inclusion			Undergraduate (UG)/Postgraduate (PG)	Methodology	Purpose of study	Key findings
					Communication	Ethics	Professionalism				
										competencies. From introduction: In this article we contribute to the literature on competency-based assessment by describing a portfolio assessment system developed as an integral component of the new Cleveland Clinic Lerner College of Medicine (CCLCM) of Case Western Reserve University In this article, we describe the design of CCLCM's portfolio approach for a comprehensive, competency-based assessment system that is fully integrated with the curriculum. To provide context, we describe the program's competencies, performance standards, core assessment principles, and unique assessment instrument before addressing the design of the overall assessment system.	with portfolio assessment within and beyond national, disciplinary, and professional borders. At the time (2002– 2003), the portfolio assessment approach was being implemented in the United States in grades K–12 and at the undergraduate college level24 –26 and in the United Kingdom and Europe at a number of medical schools. For example, at the University of Dundee College of Medicine, as a key component of the final examination process, each final-year medical student was required to submit a portfolio documenting his or her progress in achieving the outcomes of the curriculum.14,15 The first-year medical student portfolio at the University of Maastricht Faculty of Medicine included students' reflective essays on their own development in four identified roles of the doctor as a method of encouraging integration of competencies.22 An Answer to the Challenges of Competency-Based Assessment: In our opinion, this system and its components should be applied in other settings across the medical education continuum because major themes in the current debate about assessment in medical education, such as integration across competencies, competency-based assessment methods, sampling from multiple contexts and multiple sources,

For peer review only

No.	Title	Author/year	MERSQI	COREQ	Reason for inclusion			Undergraduate (UG)/Postgraduate (PG)	Methodology	Purpose of study	Key findings
					Communication	Ethics	Professionalism				
											triangulation of information, and training of learners in reflective practice are addressed in this innovative method of student assessment.
69	Supporting students in self-regulation: use of formative feedback and portfolios in a problem-based learning setting	Dannefer, E. F. and Prayson, R. A.<br><br>2013		11	✓		✓	UG	From abstract: Formative feedback on works habits and interpersonal skills provided by peers and tutors to a Year 1 cohort (n ¼ 32) over the course of a year-long PBL experience (5 blocks) was examined for comments on targeted areas for improvement (TAFIs) and observed improvements. We examined congruence between PBL feedback and students' self-reported TAFIs and behavioral improvements in their assessment portfolios.<br>From study design and methods:<br>- Study design: This study presents an analysis of (1) narrative feedback on professionalism provided to Year 1 medical students from PBL peers, tutors, and self and (2) summative portfolio essays to	From abstract: This study examines the extent to which students self-regulate professionalism behaviors related to work habits and interpersonal skills in a PBL setting.<br>From introduction: In 2004, Cleveland Clinic Lerner College of Medicine (CCLCM) implemented a program designed specifically to promote the development of self-regulated learners (Dannefer & Henson 2007). ... We confront the question of whether or not students use CCLCM's learning environment to self-regulate performance by focusing on the core competency of professionalism.<br>We selected for this study of SR the content of PBL feedback on professionalism and its use by students to identify learning needs (self-assess) and document improvement (regulate). We asked these questions:<br>(1) To what extent did the content of peer and tutor PBL	From abstract -- Results: Both PBL peer and faculty feedback and portfolio self-assessments targeted Interpersonal Skills TAFIs more frequently than Work Habit-related issues. TAFIs were more frequently identified midway in PBL blocks versus the end. Students reported TAFIs in their portfolio essays, citing feedback from both peers and tutors, and provided evidence of improved performance over time.; Conclusions: Students utilized external formative feedback to document their portfolio self-assessment in a system designed to support self-regulation of PBL professionalism-related behaviors. A decrease in TAFIs identified at the end of PBL blocks suggests students made use of mid-block feedback to self-regulate behaviors.<br>From results:<br>- (1) To what extent did the content of peer and tutor PBL narrative feedback provide information relevant to the professionalism competency standards? -- Table 1 summarizes the content of aggregate PBL tutor and peer feedback as well as PBL self and portfolio self- assessments. Of all TAFIs identified by tutors, Interpersonal Skills account for 63%. Interpersonal Skills also dominated peer statements (57%). The majority of tutor

For peer review only

No.	Title	Author/year	MERSQI	COREQ	Reason for inclusion			Undergraduate (UG)/Postgraduate (PG)	Methodology	Purpose of study	Key findings
					Communication	Ethics	Professionalism				
									determine how students used PBL feedback to support their self-assessments. We limited the study to year one the time period reported by students, and supported by advisors' observations, as requiring significant adjustments in terms of taking responsibility for their own learning.<br>- Measures: Faculty defined measurable, behavioral competency standards developmentally appropriate for Year 1 students and observ- able across multiple settings. ... Self-regulation, as a process, consists of overlapping and recursive activities of which we targeted self-assessments that identified areas needing improvement and cited evidence that documented improved performance.<br>- Data sources: We collected peer and tutor PBL assessments electronically at mid and end of Block 1 problem-	narrative feedback provide information relevant to the professionalism competency standards?<br>(2) To what extent did students self-report and document professionalism learning needs and improvements consistent with peer and tutor PBL feedback in a portfolio used for promotion decisions?	and peer TAFI statements about Interpersonal Skills indicated a concern with ''quietness'' (e.g. lack of participation). In the area of Work Habits, peers more frequently identified ''preparedness'' (e.g. disorganized presentations) as a TAFI in fellow students as compared with tutors, who more frequently documented behaviors associated with being ''distracted'' (e.g. carrying on side conversations). Peers were also more likely to give feedback on TAFIs than tutors; of 2016 peer assessments, 41% contained feedback on TAFIs. Overall, tutor feedback tended to be less detailed and only 28% of the 288 possible assessments contained feedback on TAFIs. Similar to tutor and peer evaluations, students self- identified Interpersonal Skills issues as TAFIs more often than Work Habit-related issues (60% vs. 40% respectively). Also similar to peer assessments, students frequently self-identified ''ill-preparedness'' as the Work Habit most needing improvement. In contrast to peer and tutor feedback, however, students more frequently self-identified ''dominating'' behavior issues rather than ''quietness''. All students, except for one who did not complete self-assessments, self-identified TAFIs also reported by their peers and tutors. Interestingly, 95% of students at some point during the year also self-identified issues not documented by peers and

For peer review only

No.	Title	Author/year	MERSQI	COREQ	Reason for inclusion			Undergraduate (UG)/Postgraduate (PG)	Methodology	Purpose of study	Key findings
					Communication	Ethics	Professionalism				
									solving sessions, and from peers, tutors and self at mid and end of Blocks 2-4 and end of Block 5 case-based sessions. Because professionalism cannot be compartmen- talized, students address standards not only in the Professionalism Competency essay but also in the Communication, Personal Development, and Reflective Practice competency essays. Thus, data used in this study included essays and citations for these four competencies. - Coding and analysis of PBL narrative feedback: A trained research assistant extracted the electronic competency report of all PBL feedback for professionalism for all first-year students (n ¼ 32) for the 2009 academic year, and replaced students' names with a unique six-digit identifier. We		tutors. Table 1 shows that peer and tutor feedback and self-assessments documented improvements less frequently than TAFIs, and statements noting improvement in Interpersonal Skills were more fre- quent than statements of improvement in Work Habits. Figure 1 documents peer and tutor feedback statements for students at mid and end of the four PBL blocks. In aggregate, more TAFI statements were made by peers and tutors for mid-block assessments than at the end of the block with an overall decrease across the year. Regarding individual student data, 29 of the 32 students had more TAFIs documented at mid-block by peers as compared with end-of-block, and early in the year (Blocks 1–2) versus later in the year (Blocks 3–4). Similarly, more evidence with regard to improvements was documented for students by peers in end-of-block and late year feedback versus mid block and early year feedback, respectively. Similar trends were noted in the tutor feedback for mid- and end-of- block TAFIs and improvement statements. - (2) To what extent did students self-report and document professionalism learning needs and improvements consistent with peer and tutor PBL feedback in a portfolio used for promotion decisions? -- Student self-assessments presented in their summative portfolio essays are required to be a

For peer review only

No.	Title	Author/year	MERSQI	COREQ	Reason for inclusion			Undergraduate (UG)/Postgraduate (PG)	Methodology	Purpose of study	Key findings
					Communication	Ethics	Professionalism				
									developed a rubric using the PBL assessment form criteria for Interpersonal Skills and Work Habits to individually code five competency reports. The authors then met and reached consensus on coded professionalism statements about TAFIs and observed improvements. The refined Professionalism Rubric has three unprofessional behavior criteria each for Interpersonal Skills (Dominating, Quiet, Inappropriate) and for Work Habits (Late, Ill-prepared, Distracted). Each author coded the remaining reports before meeting to reach consen-sus and in rare instances of coding differences; these discrepancies were reconciled. The authors tallied type and number of professionalism statements for each student for each block from peers, tutors and self.		representative and balanced account of their professional behavior across the year. As an indicator of ‘‘representative’’, we identified behaviors that were observed by tutors in at least two different groups, which suggest stability of behavior across time and group dynamics. For the 16 students where two different tutors identified the same TAFI, peers identified the same TAFIs as the tutors in five PBL Blocks for seven students, four PBL blocks for six students, three PBL blocks for two students, two PBL blocks for one student. All 16 students reported the TAFI in their summative portfolio, citing both tutor and peer feedback. All cited evidence of improvement from peers and/or tutors. A strong majority (91%) of portfolio essays followed a pattern of citing feedback from PBL peers and/or faculty to identify at least one TAFI, followed by citations indicating improved performance. To illustrate student’s use of PBL feedback to document performance, we provide the portfolio self-assessment of one student who received ten TAFIs during the first half of the year and three in the second half, indicating a tendency to dominate. The tendency to dominate was mentioned by peers in four of the five PBL groups but never mentioned by tutors. These comments included statements such as ‘‘talking over people,’’ ‘‘can push ideas a little bit too hard ‘‘sometime can seem

For peer review only - http://bmjopen.bmj.com/site/about/guidelines.xhtml

No.	Title	Author/year	MERSQI	COREQ	Reason for inclusion			Undergraduate (UG)/Postgraduate (PG)	Methodology	Purpose of study	Key findings
					Communication	Ethics	Professionalism				
									- Coding and analysis of summative portfolio essays: A research assistant extracted the summative portfolio essays from the database for the four competencies (Professionalism, Communication, Personal Development, and Reflective Practice) that 32 students submitted at the end of the 2009 academic year, for a total of 124 essays. The authors used the Professionalism Rubric to analyze the portfolio essays for the aforementioned competencies, where professionalism issues were likely to be addressed. The authors independently coded professionalism statements before meeting to reach consensus in the rare instances where there were differences in coding. All PBL citations used to document the coded statements were tallied by source (tutor, peer, self) and block time period.		argumentative/defensive'' and needs to ''listen to other viewpoints even though they might not coincide with personal views.'' Three other TAFIs were mentioned three or less times and only in the first half of the year. Table 2 provides quotes from this one student's cited PBL feedback, documenting a TAFI (tendency to dominate) and evidence of improvement over the course of year 1. This student acknowledged and reflected on this TAFI in the portfolio essay and provided evidence of improvement, noting that this behavior may need to be ''reestablished'' with each new working group. See Table 3 for excerpt from this student's portfolio. The essay provides evidence relevant to each of our questions. The student identified and acknowledged a prob- lematic pattern of behavior, reflected on the feedback, and used suggestions to ''amend'' behavior, which resulted in improved performance. Not every student described the self- regulation process in such detail, but all acknowledged a behavior identified by peers and/or tutors that was problem- atic and presented evidence of improvement.<br>From discussion: ... While this study suggests that students are using the supports of this educational program to self-regulate, we need to better understand how students process and use feedback, how external feedback triggers and shapes intern- ally

No.	Title	Author/year	MERSQI	COREQ	Reason for inclusion			Undergraduate (UG)/Postgraduate (PG)	Methodology	Purpose of study	Key findings
					Communication	Ethics	Professionalism				
											generated feedback, what role advisors play in helping students reflect on and use feedback, and what processes students use to make judgments and develop plans for improvement, questions being addressed by the work of Sargeant et al. (2008, 2009, 2011). As future physicians, today's students will need to be able to self-regulate their behaviors in settings where feedback may not be as structured or robust and facilitation of reflection lacking. To support development of SR skills, we need medical school programs designed to provide formative feedback and clear standards, opportunities to improve, and facilitated self-assessment. The PBL-portfolio model provides one strategy by which students can gain skills in self-regulation and develop habits useful for future practice.
70	Portfolio assessment in medical students' final examinations	Davis, MH, Ben-David, M Friedman, Harden, RM, Howie, Peter, Ker, Jean, McGhee, C, Pippard, MJ and Snadden, David<br><br>2009	7		✓	✓		UG	From abstract: Portfolio assessment has been developed for this purpose and has been adopted for the summative assessment of students in their final examination in Dundee. The contents of the portfolio and the assessment process have been defined and the first cohort of students to be assessed in this	This paper describes a new approach to the final examination based on portfolios, which has been piloted in Dundee Medical School. This paper describes our first year's experience with this new approach.	From abstract: The evaluation of the approach demonstrated strong staff support. Students were also positive although with some reservations. It is concluded that portfolio assessment is a powerful approach to assessing a range of curriculum outcomes not easily assessed by other methods and is worthy of inclusion in the assessor's toolkit.<br>Results: Of the 129 candidates for the portfolio assessment, three were withdrawn for the reasons given above. Of the 126 remaining, 108 (86%)

For peer review only - http://bmjopen.bmj.com/site/about/guidelines.xhtml

No.	Title	Author/year	MERSQI	COREQ	Reason for inclusion			Undergraduate (UG)/Postgraduate (PG)	Methodology	Purpose of study	Key findings
					Communication	Ethics	Professionalism				
									way has been studied. The final examination: The final examination was redesigned to meet the needs of the new curriculum, in particular the moves to an inte- grated, task-based approach and an outcome-based education model. The examination is in two parts. Part 1 is at the end of year 4 and comprises a multiple-choice paper of extended matching items (134) designed to assess knowledge and its application; a constructed response question (CRQ) paper to assess higher-order thinking such as problem solving and critical analysis as well as knowl- edge; and an OSCE to assess clinical skills. The use of an OSCE as part of the final examination has been reported (Preece et al., 1992). Part 2 of the final examination takes place towards the end of year 5 and adopts a more innova- tive		passed, 13 (10%) received a condi- tional pass and five (4%) failed. No students fell into the bad fail category. Individual student profiles, in terms of the 22 grades for the learning outcomes, showed variation. Three students (2%) were awarded 22 A grades. One student (0.8%) was awarded 22 B grades. No student was awarded 22 C, D, E or F grades. Forty-four students (35%) were awarded 22 grades at either A or B levels. Two students (1.5%) were awarded 22 grades at either C or D levels. No students were awarded 22 grades at either E or F levels. Over 60% of the students were not consistent in their ability across the learning outcomes, but had individual strengths and weaknesses. The student gradings awarded by both pairs of exam- iners in each outcome are given in Figure 1. Non-pass gradings are confined to outcomes 2, 7, 8, 11 and 12. Evaluation of portfolio assessment: - (1) Analysis of student results: Numeric values from one to six were allocated to the students' outcome grades in the portfolio assessment. An overall mark was then calculated for every student. This overall mark was correlated, using the Spearman correlation, with the mark for each of the three compo- nents of Part 1 of the final examination (EMI, 0.42; CRQ, 0.42; OSCE, 0.47) and with the mark allocated to students

No.	Title	Author/year	MERSQI	COREQ	Reason for inclusion			Undergraduate (UG)/Postgraduate (PG)	Methodology	Purpose of study	Key findings
					Communication	Ethics	Professionalism				
									approach – portfolio assessment. The approach was adopted as it offered the potential of assessing the students' achievement in each of the curriculum outcomes including those not easily assessed by more traditional approaches. It also offered the potential of reviewing the development of student competence over time and presenting feedback to students. Implementation of the portfolio assessment: Work for the portfolio assessment was carried out in three stages: - Stage 1: Work carried out before the portfolio review -- Towards the end of year 5, before the PRHO preparation block, students submitted their completed portfolios to administrative staff in the medical school office. Following a check for completeness,		in the end-of-block examination for one phase 3 core block attachment (psychiatry, 0.34). These results show low/moderate correlation. - (2) Observer documentation: Three observers, two internal and one external, were appointed to document any issues that arose during the portfolio assessment process. They reported that the port- folio submission procedures worked well. The countersigned receipt given to students was an essential step to avoid disputes over submitted material. Entering the pre-marked grades for various portfolio components into the PASS was a major task for administrative staff and required to be streamlined for future years. ... (see under issues related to implementation of portfolio) The PASS system worked well and provided signed sheets to be retained as a permanent record of the examination. - (53) Examiners' evaluation questionnaire: The examiners' evaluation questionnaire was developed following discussion with members of the working group that introduced portfolio assessment and with both the external and internal examiners who participated in the portfolio examination. It was designed to gather the exam- iners' opinions regarding the: effectiveness of the portfolio approach to student assessment; usefulness of the components of the portfolio

For peer review only

No.	Title	Author/year	MERSQI	COREQ	Reason for inclusion			Undergraduate (UG)/Postgraduate (PG)	Methodology	Purpose of study	Key findings
					Communication	Ethics	Professionalism				
									they received and countersigned a receipt. The administrative staff transcribed from student records, onto an assessment summary sheet called the Port-folio Assessment and Summary Sheet (PASS), the grades awarded for the portfolio contents in respect of each of the curriculum outcomes. ... Three of the students were identified as ineligible to proceed to the portfolio review with the examiners at this stage, two for reasons of illness and non-completion of course work and one pending a disciplinary hearing. All three, however, were allowed to complete the portfolio review process for formative purposes.<br>- Stage 2: Portfolio review with the examiners -- ... (see under deciding how to mark/assess)<br>- Stage 3: Work completed after the portfolio review -- ... (see		in providing information about the students' abilities; conduct of the examination; standards achieved by the students; strengths and weaknesses of the portfolio approach to assessment; suggestions for improvement. Examiners were asked to rate a number of statements on a Likert-type five-point scale from 5—strongly agree to 1— strongly disagree. There were also free-response questions. The questionnaire was distributed and completed by the examiners at the end of the examiners' meeting on day 2. Thirty-three examiners' questionnaires were distributed and returned, giving a response rate of 100%. Responses were collated using the Excel computer software package by Microsoft. The percentage distribution of examiners' ratings with the number that responded to each statement (N) the calculated average score (AV) and the standard deviation (SD) are provided in Table 1. ... (see the rest under perspective of examiners)<br>- (4) Student evaluation questionnaire: The student evaluation questionnaire was developed following periodic feedback from the students during their phase 3 studies and from a student group discussion with the external observer that took place immediately after the portfolio review. Students were given the opportunity for free and frank expression of their opinions during this discussion. The

For peer review only

No.	Title	Author/year	MERSQI	COREQ	Reason for inclusion			Undergraduate (UG)/Postgraduate (PG)	Methodology	Purpose of study	Key findings
					Communication	Ethics	Professionalism				
									under deciding how to mark/assess) Evaluation of the portfolio assessment: Five methods were employed to evaluate the use of portfo- lios as the basis for the final examination: (1) analysis of student results; (2) observer documentation; (53) examiners' evaluation questionnaire; (4) student evaluation questionnaire; (5) verbal report from student representatives. (see details under results of study).; From discussion - evaluation methodology: Rossi & Freeman (1993a) (p. 13) define evaluation research as 'a robust arena of activity directed at collecting, analysing and interpreting information on the need for, implementa- tion of, and effectiveness and efficiency of intervention efforts'. Education evaluation is more specifically identified as 'the process of making judgement about		student questionnaire was designed to gather opinion about building the portfolio, about its focus around the curriculum outcomes and about events during the portfolio assessment. The questionnaire was distributed and completed 22 days after the examination, at a time when all students were aware of whether or not they had passed the examina- tion. Those students who were unsuccessful had received counselling. The rating scale, data entry and scoring system were similar to those used in the examiners' ques- tionnaire. A total of 107 student questionnaires were returned from 129 students who sat the examination—a response rate of 83%. The percentage distribution of students' ratings, the number that responded to each statement (N), the calcu- lated average score (AV) and the standard deviation (SD) are provided in Table 2 for building the portfolio and in Table 3 for the portfolio assessment. ... (see the rest under perspective of students) - (5) Student representatives' comments: ... (see the rest under perspective of students)

For peer review only

No.	Title	Author/year	MERSQI	COREQ	Reason for inclusion			Undergraduate (UG)/Postgraduate (PG)	Methodology	Purpose of study	Key findings
					Communication	Ethics	Professionalism				
									the merit, value or worth of educational programmes' (Gall et al., 1996). It is recognized that it is often impossible to conduct educational evaluations according to the best possible design and Rossi & Freeman (1993b) advocate the concept of the 'good enough' evaluation. The five methods employed to evaluate the use of portfolios as the basis for the final examination followed the 'good enough' approach.		
71	A Qualitative Analysis of Narrative Preclerkship Assessment Data to Evaluate Teamwork Skills	Dolan, B. M., O'Brien, C. L., Cameron, K. A. and Green, M. M.<br><br>2018		15	✓		✓	UG	From abstract: We performed a qualitative analysis of narrative data in 15 assessment portfolios. Student portfolios were randomly selected from 3 groups stratified by quantitative ratings of teamwork performance gathered from small-group and clinical preceptor assessment forms. Narrative data included peer and faculty feedback from these same forms. Data were	We sought to understand if and how teamwork behaviors are described in the narrative feedback given by faculty and peers in preclerkship years and how this feedback may vary based on students' competency achievement level. We expect that review of assessment data collected in an electronic portfolio and judged by experienced faculty may fill the gap in teamwork assessment by providing a method to evaluate	From abstract -- Results: Eight codes related to teamwork emerged: attitude and demeanor, information facilitation, leadership, preparation and dependability, professionalism, team orientation, values team member contributions, and nonspecific teamwork comments. The frequency of codes and valence varied across the 3 performance groups, with students in the low-performing group receiving more suggestions for improvement across all teamwork codes. Conclusions: Narrative data from assessment portfolios included specific descriptions of teamwork behavior, with important contributions provided by

For peer review only

No.	Title	Author/year	MERSQI	COREQ	Reason for inclusion			Undergraduate (UG)/Postgraduate (PG)	Methodology	Purpose of study	Key findings
					Communication	Ethics	Professionalism				
									coded for teamwork-related behaviors using a constant comparative approach combined with an identification of the valence of the coded statements as either “positive observation” or “suggestion for improvement.” Study approach and setting: We conducted a qualitative study using a constant comparative approach to better characterize the narrative assessments that students receive regarding teamwork.17 ... We excluded assessments that do not include students' teamwork skills, including assessments from observed structured clinical exams, assessments of written assignments, medical knowledge assessments, assessments of groups as a whole (where no individual feedback was given), ethics oral examinations, direct observations of	longitudinal, behaviorally based narrative feedback regarding student teamwork knowledge, skills, and attitudes.	both faculty and peers. A variety of teamwork domains were represented. Such feedback as collected in an assessment portfolio can be used for longitudinal assessment of preclerkship student teamwork skills and attitudes. From discussion: This analysis demonstrates that narrative feedback in assessment portfolios provides a longitudinal view of preclerkship students' teamwork behaviors. Although similarities exist between performance groups, we observed differences between groups in content, frequency, and valence of feedback. These differences could assist mentor/coaches and competency committee members in identifying both students who excel at teamwork and those needing additional support with teamwork behaviors prior to starting clerkships. Although our coded categories were identified without the use of a predetermined framework, they align with key teamwork and leadership attributes identified in the literature. Critical teamwork skills and attitudes have previously been described that share similarity with our codes: leadership, team orientation, preparation/dependability.21 Physician leadership competencies highlight the importance of showing integrity, listening actively, promoting team building, and nurturing/recognizing others' accomplishments.22–24

For peer review only

No.	Title	Author/year	MERSQI	COREQ	Reason for inclusion			Undergraduate (UG)/Postgraduate (PG)	Methodology	Purpose of study	Key findings
					Communication	Ethics	Professionalism				
									clinical skills, and scholarly project oral presentations (which focus solely on presentation skills). Sampling strategy: Students were notified of the study via e-mail and were encouraged to e-mail an author not involved in grading students (CLO) if they did not wish to participate. Students were given 1 week to respond. Four students chose to opt out of the study. We divided the remaining student portfolios into three groups. We first identified the students who received progressing towards competence with concern or progressing towards competence pending additional development for any of the three competencies most closely related to teamwork—teamwork, professionalism, and communication—during the competency review. We		These attributes arose in our analysis, lending credibility to the argument that narrative data in assessment portfolios can be used to understand students' teamwork behaviors in the preclerkship years and can help students reflect on how their behaviors promote or detract from their teams' overall performance. Review of the preclerkship narrative data provides mentors or coaches the ability to identify students in need of remediation before full-time immersion in clinical practice.25 Our analysis demonstrates that low-performing students share similar characteristics that reviewers can look for poor attitude in small groups, lack of preparation for group activities, or dismissive behavior toward others' contributions. It also demonstrates that the absence of certain comments may be telling, as evidenced by the sharp reduction in even nonspecific comments such as “great team member” or the lack of leadership observations in the low-performing student portfolios. When reviewing assessment data with their students, mentors may choose to highlight these absences to help students improve. Notably, peers provided the majority of feedback in the codes of team orientation and values' contributions of others. Due to their close working relationships during preclerkship years, peers may be in the best position

For peer review only

No.	Title	Author/year	MERSQI	COREQ	Reason for inclusion			Undergraduate (UG)/Postgraduate (PG)	Methodology	Purpose of study	Key findings
					Communication	Ethics	Professionalism				
									designated these 19 students as the low-performing group. As our competency review does not have a comparable high-performance designation, we employed an alternative strategy using the composite grading system. To identify a high-performing cohort of students for comparison, we identified the 19 students receiving the highest numerical ratings for the group of assessments included in Table 1. We designated this to be the high-performing group. The middle-performing group comprised all remaining students (n = 114) who did not meet criteria for the high- or low-performing groups. Four students opted out of the study. To protect privacy, each student portfolio received a random numerical identifier. A key was created linking each identifier to each student's ID number; an		to make such judgments. Furthermore, when peers are given formalized instruction in providing feedback, as they are at our institution, including their insight and feedback is critical when assessing student performance. Our results suggest that assessment systems that do not include peer feedback may miss the opportunity to identify students in need of support for developing teamwork skills. Prior studies demonstrate student acceptance of peer feedback, particularly when used for formative purposes.26 Additional studies that specifically compare faculty versus student feedback may further illuminate the best ways to employ the differing perspectives of peers and faculty when assessing student performance. The use of assessment portfolio data may continue to increase as institutions implement entrustable professional activities, competency committees, and other assessment strategies that require a systems-based approach.27 We believe that the findings in this analysis can assist both in teamwork assessment and as a resource to aid faculty and students in identifying preclerkship goals for teamwork and leadership skills development. Additional study is needed to determine if preclerkship assessment of teamwork correlates with later clinical performance. In conclusion, our analysis of narrative

For peer review only

No.	Title	Author/year	MERSQI	COREQ	Reason for inclusion			Undergraduate (UG)/Postgraduate (PG)	Methodology	Purpose of study	Key findings
					Communication	Ethics	Professionalism				
									author not involved in direct teaching of students (CLO) deidentified all data before analysis. We randomly selected three student portfolios from each group (high, middle, low) to analyze, then further intentionally sampled two additional portfolios in each group to allow for adequate gender inclusion and to confirm emergence of no new codes (see next); these 15 portfolios composed our sample. Data analysis: To better understand how teamwork performance is observed and described by peers and faculty, we used constant comparative analysis techniques. We selected this method as a framework to explore teamwork given that comparison between groups of students may better delineate the similarities and/or differences in how behaviors were described among variably		assessment data from across the preclerkship curriculum demonstrates that these data capture a number of key teamwork skills and attitudes with behaviorally based feedback, thus filling a gap in the longitudinal assessment of teamwork. Providing students and faculty the opportunity to critique and further reflect on behaviorally based narrative feedback may assist students in improving these skills prior to the beginning of clerkships.

For peer review only

No.	Title	Author/year	MERSQI	COREQ	Reason for inclusion			Undergraduate (UG)/Postgraduate (PG)	Methodology	Purpose of study	Key findings
					Communication	Ethics	Professionalism				
									performing students. Constant comparative methodology has been described as an ideal way to "conceptualise the variety that exists within the subject under study.17(p393) After using this methodology to complete the coding, we undertook a content analysis to better understand the prevalence and valence of identified themes across the three performance groups.19 Three research team members (BMD, CLO, MG) with expertise in medical student assessment performed the primary analysis of portfolio comments; two are practicing clinicians with experience on medical teams (BMD, MG). The fourth team member, an expert in qualitative research (KAC), provided guidance and oversight in methodology. Narrative data was uploaded for analysis into		

For peer review only

No.	Title	Author/year	MERSQI	COREQ	Reason for inclusion			Undergraduate (UG)/Postgraduate (PG)	Methodology	Purpose of study	Key findings
					Communication	Ethics	Professionalism				
									NVivo (QSR, Mac version 10.2.0) after deidentification.20 We selected the middle-performing group as the first group for analysis, as it represents the typical student who is neither struggling nor exceptionally high achieving. Consistent with conventional content analysis19 and to best explore what teamwork categories faculty and students choose to comment on in their narratives, we did not use an a priori framework for coding. However, one author (BMD) is familiar with medical teamwork literature,21,22 which has informed the way medical students approach teamwork in our institution and which may have influenced her approach to coding. The second coder has a background in education. She had no specific expertise with the teamwork literature and arrived at the same codes. Two		

No.	Title	Author/year	MERSQI	COREQ	Reason for inclusion			Undergraduate (UG)/Postgraduate (PG)	Methodology	Purpose of study	Key findings
					Communication	Ethics	Professionalism				
									authors (BMD and CLO) initially reviewed three portfolios from the middle-performing group, assigning a code to all narrative statements. These authors then met and achieved consensus regarding the coding framework. The remaining portfolios in the middle-performing group were analyzed using the identified codes; all statements were assigned a valence of either "positive observation" or "suggestion for improvement." Three authors (BMD, CLO, MG) met to review the statements' assigned codes and come to consensus on any differences, both with respect to code and valence. Differences were resolved via group discussion. Using the codes identified, we then analyzed the low- and high-performing groups, representing the extremes of performance.		

For peer review only - http://bmjopen.bmj.com/site/about/guidelines.xhtml

No.	Title	Author/year	MERSQI	COREQ	Reason for inclusion			Undergraduate (UG)/Postgraduate (PG)	Methodology	Purpose of study	Key findings
					Communication	Ethics	Professionalism				
									Although no new codes were identified in the high-performing or low-performing groups, the narrative from these groups helped to refine the coding set to reflect nuances that emerged from the other groups. For example, after reviewing the high-performing narrative data, the original code “information sharing” was revised to “information facilitation” to better reflect the variety of ways that students mediated the flow of information in small groups. The code “values contributions of team members” evolved from “values team members” after review of the additional suggestions for improvement found in both the high- and low-performing groups. We reviewed three portfolios in each performance group but identified few new codes after the second student's portfolio in each group. However,		

For peer review only

No.	Title	Author/year	MERSQI	COREQ	Reason for inclusion			Undergraduate (UG)/Postgraduate (PG)	Methodology	Purpose of study	Key findings
					Communication	Ethics	Professionalism				
									we continued with two additional portfolios per group for adequate gender inclusion and to confirm the emergence of no new codes; no new codes were found with the addition of these six portfolios. Upon completion of the coding process, NVivo software was used to quantify the number of comments per code and valence. Techniques to ensure trustworthiness of data included the use of two coders followed by additional review of the codes by a third author. Codes and examples were also discussed at a medical education group research meeting where additional faculty members with less connection to the portfolio process provided feedback to the research team.		
72	Portfolio as a learning tool: students' perspective	Elango, S., Jutti, R. C. and Lee, L. K.<br><br>2005	6.5		✓			UG	From abstract: A questionnaire survey was conducted among 143 medical students to find out their perceptions of the	This study was formulated to find out students' perspectives on portfolio learning.	From abstract: ... (see the rest under perspective of students) The study indicates that students need appropriate guidance from the academic staff for the system to succeed.<br>From introduction: In the

For peer review only

No.	Title	Author/year	MERSQI	COREQ	Reason for inclusion			Undergraduate (UG)/Postgraduate (PG)	Methodology	Purpose of study	Key findings
					Communication	Ethics	Professionalism				
									portfolio as a learning tool. From materials and methods: Feedback was obtained from an entire batch of Semester 8 students by asking them to write down what they felt about writing a portfolio. Based on their feedback, a questionnaire consisting of 23 questions was designed. The entire student population (259) in the clinical school (from Semester 6 to Semester 10) was given an information sheet with details about this survey and was given the option of participating in the study. The students who volunteered for the study were asked to sign a written consent form. The students who gave their consent for the survey were requested to complete the questionnaire anonymously. For each of the 23 questions, students had to respond on a 4-point Likert scale, ranging from		last decade, there have been numerous innovations in educational theory and practice. Education has moved from being a traditional teacher-centred process to one that is student-centred. Similarly, in the area of assessment, there has been a shift away from assessing knowledge towards a more competency/performance-based assessment. There is now a wide variety of methods of education and training to choose from and portfolio-based learning is an increasingly popular option among educationists.1<br>From results: One hundred and forty-three students completed and returned the questionnaire (Table1). There were 76 (53.1%) females and 64 (44.8%) males. In order to facilitate the analysis, the 4 levels of perception in the questionnaire – strongly agree, agree, disagree and strongly disagree – were regrouped; strongly agree and agree were grouped as one, and strongly disagree and disagree were grouped as the other. ... (see the rest under perspective of students)<br>From discussion: A study has shown that students using portfolios obtain higher marks in factual knowledge compared to the control group of students not using portfolios11<br>From conclusion: The study demonstrates that any medical school intending to introduce the portfolio as a new learning method must take into consideration an

For peer review only

No.	Title	Author/year	MERSQI	COREQ	Reason for inclusion			Undergraduate (UG)/Postgraduate (PG)	Methodology	Purpose of study	Key findings
					Communication	Ethics	Professionalism				
									strongly agree to strongly disagree. The responses to the questionnaire were analysed to determine the overall views of the students on the different aspects of portfolio writing.		effective orientation programme for both the learners and the teachers. Motivated students, and trained and motivated staff are essential for portfolio-based learning to succeed.
73	Students' reflections in a portfolio pilot: highlighting professional issues	Haffling, A. C., Beckman, A., Pahlmblad, A. and Edgren, G.<br><br>2010		14	✓	✓	✓	UG	From abstract: Thirty-five voluntary final-year medical students piloted a standardized portfolio in a general practice (GP) attachment at Lund University, Sweden. Students' portfolio reflections were based upon documentary evidence from practice, and aimed to demonstrate students' learning. The reflections were qualitatively analysed, using a framework approach. Students' evaluations of the portfolio were subjected to quantitative and qualitative analysis.<br>Pilot: Students of two consecutive classes, of 76 and 71 students, respectively, were approached. Twenty students in each class	From abstract: To investigate whether students' reflections include sufficient dimensions of professional competence, notwithstanding a standardized portfolio format, and to evaluate students' satisfaction with the portfolio<br>From introduction: We aimed to develop a portfolio for the assessment of final-year students' professional development during their general practice (GP) attachment. ... We designed a pilot study with the principal aim of investigating whether themes of students' reflections would include satisfactory dimensions of professional competence, notwithstanding the standardized format. This article qualitatively explores the emergent themes of students' reflections, and also reports on students' evaluation of the portfolio.	From abstract -- Results: Among professional issues, an integration of cognitive, affective and practical dimensions in clinical practice was provided by students' reflections. The findings suggested an emphasis on affective issues, particularly on self-awareness of feelings, attitudes and concerns. In addition, ethical problems, clinical reasoning strategies and future communication skills training were subjects of several reflective commentaries. Students' reflections on their consultation skills demonstrated their endeavour to achieve structure in the medical interview by negotiation of an agenda for the consultation, keeping the interview on track, and using internal summarizing. The importance of active listening and exploration of patient's perspective was also emphasized. In students' case summaries, illustrating characteristic attributes of GP, the dominating theme was 'patient-centred care', including the patient–doctor relationship, holistic modelling and longitudinal continuity. Students were satisfied with the portfolio,

For peer review only - http://bmjopen.bmj.com/site/about/guidelines.xhtml

No.	Title	Author/year	MERSQI	COREQ	Reason for inclusion			Undergraduate (UG)/Postgraduate (PG)	Methodology	Purpose of study	Key findings
					Communication	Ethics	Professionalism				
									were offered to voluntarily pilot a portfolio in GP as an addition to the written test, which for the participants would be reduced in the number of GP questions. A total of 35 students, 16 from the first and 19 from the second class, accepted. Twenty-four (69%) of them were women, as compared to 59% of all students. At the time of the study, consent from the University's ethics committee was not required for projects involving students' assessments. Participating students were provided with both verbal and written guidelines on the purpose, format and assessment of their portfolios. Three of the authors (ACH, AP, AB) and a fourth teacher, all involved in teaching, acted as mentors to support their students in compiling the portfolio. Qualitative analysis of students' reflections: To be		but improved instructions were needed.; Conclusions: A standardized portfolio in a defined course with a limited timeframe provided ample opportunities for reflections on professional issues. Support by mentors and a final examiner interview contributed to the success of the portfolio with students. The interview also allowed students to deepen their reflections and to receive feedback. Students' evaluation questionnaire: see under perspective of students From discussion: Firstly, students' reflections on their consultation skills showed that the most important learning issue in the theme of 'process skills' was how to achieve structure in the medical interview. Patient-centred communication can cause genuine concerns in students about losing control of the consultation and getting caught in a flow of less ordered information from the patient (Silverman et al. 2004). When students learned how to obtain structure, they also experienced better time management, enhanced self-confidence and an improved rapport with the patient. Secondly, the theme of 'patient-centred care' was emphasized in students' case summaries. Patient-centred care is a major principle in GP, but is of essential interest to several other medical disciplines as well. Achieving shared decision making, understanding the patient as

No.	Title	Author/year	MERSQI	COREQ	Reason for inclusion			Undergraduate (UG)/Postgraduate (PG)	Methodology	Purpose of study	Key findings
					Communication	Ethics	Professionalism				
									able to conduct the qualitative analysis, students' reflections were written verbatim into a word processor by one of the authors (173). Each student was allocated a code number to prevent identification of individual students' comments. The text of the reflections constructed two separate units of analysis; 'consultation skills' and 'case summaries'. The transcripts were read through several times to get a sense of the whole, and then analysed using a framework approach (Crabtree & Miller 1999). For 'consultation skills', the text was mapped against the Calgary Cambridge Guides (Silverman et al. 2004). For 'case summaries', the statement of WONCA Europe (2005), (Box 2), was used as a template for organizing the data. In each of		a whole person and exploring the patient's perspective are aspects shown to positively influence patients' satisfaction and enablement, reduce their symptom burden and discomfort, and increase efficiency of care (Stewart et al. 2000; Little et al. 2001). We believe that these central dimensions of patient-centred care ought to be highlighted earlier in hospital clerkships. Thirdly, the findings suggested integration of the cognitive, affective and practical dimensions of professional competence that can be acquired in a clinical learning environment (Mann et al. 2007; van Tartwijk & Driessen 2009; Passi et al. 2010). As expected, students reflected on their 'content skills' and provided evidence for their identification of deficits in knowledge and skills, their integration of new understanding, and their plans for development. However, 'perception skills' was a more prominent theme in both units of analysis. In their self-awareness of feelings, attitudes and concerns, students were unexpectedly open and frank about their deficiencies and about how they had tried to remedy them. They also acknowledged the importance of ethical issues and additional communication skills training, and openly reflected on their clinical reasoning. However, we found it remarkable that

No.	Title	Author/year	MERSQI	COREQ	Reason for inclusion			Undergraduate (UG)/Postgraduate (PG)	Methodology	Purpose of study	Key findings
					Communication	Ethics	Professionalism				
									the two units of analysis, related segments of text were identified and indexed to the appropriate part of the thematic framework. Some codes were modified and new ones added to reflect as many nuances in the data as possible. 'Chunks' of related text under each code were examined together and distilled summaries made; this process involved a considerable amount of abstraction. The chunk summaries provided an analytical tool from which connections were made and sub-themes and themes identified. ACH undertook the mapping of text and carried out the primary analysis of the data. The four teachers, involved in the pilot, examined in recurrent meetings whether the interpretation of the data was plausible. The codes and sub-themes were compared based on differences		several students, in their final year in medical school, reflected on their initial problems in transferring theory to practice; a skill supposed to be frequently trained during previous hospital clerkships. From conclusion: In summary, this study demonstrated that a structured portfolio in a short timeframe provided abundant opportunities for students to reflect on personal and professional issues, notwithstanding the standardized format. We strongly recommend a final examiner interview to deepen students' reflections, to supply feedback and to raise the importance of reflective ability as a skill in future learning from practice.

For peer review only

No.	Title	Author/year	MERSQI	COREQ	Reason for inclusion			Undergraduate (UG)/Postgraduate (PG)	Methodology	Purpose of study	Key findings
					Communication	Ethics	Professionalism				
									and similarities, and some were revised during this process of corroboration. Students' evaluation questionnaire: The short student evaluation form was supplied directly after the examiner interview, asking five questions about the portfolio instructions, the mentor contact and the examiner interview, using a 3-point scale ('yes', 'to some extent', 'no'). Students were also requested to provide free comments on positive and negative experiences of the portfolio, and to give suggestions for improvements. The results were subjected to quantitative and qualitative (content) analyses.		
74	Undergraduate medical students' views about a reflective portfolio assessment of their communication skills learning	Rees, C. and Sheard, C.<br><br>2004	10		✓			UG	From abstract: 178 second-year medical students at the University of Nottingham completed the 18-item reflective portfolio questionnaire (RPQ) (α = 0.716) and a personal details questionnaire	this study explores second-year medical students' views about a reflective portfolio assessment of their communication skills. The research questions for this study are: 1) What are students' views of a reflective portfolio assessment	From abstract -- Results: Total scores on the RPQ ranged from 40 to 75 (mean 58.28, SD 7.08). Significant relationships existed between RPQ total scores and students' ratings of their reflection skills (r s = 0.322, P < 0.001), RPQ total scores and students' confidence building another portfolio (T = 4.381, d.f. = 176, P

For peer review only

No.	Title	Author/year	MERSQI	COREQ	Reason for inclusion			Undergraduate (UG)/Postgraduate (PG)	Methodology	Purpose of study	Key findings
					Communication	Ethics	Professionalism				
									three days before submitting their portfolio assessment for communication skills. Data were analysed using univariate and multivariate statistics on SPSS Version 10.0. From methods: Participants -- Second-year medical students at the University of Nottingham completed a personal details questionnaire (PDQ) and the reflective portfolio questionnaire (RPQ) three days before submitting their portfolio assessment for communication skills.; Measures -- The PDQ contains 13 demographic and education-related items. The RPQ was designed by CR to explore medical students' views of portfolios and reflection. Eighteen items were generated using previous research literature,1-4 e.g. 'I have felt anxious about sharing my weaknesses in my reflective portfolio' (item 16). Each item	of their communication skills? 2) Do relationships exist between students' views of reflective portfolios and their perceived and actual reflection skills and their confidence building portfolios?	< 0.001), and RPQ total scores and students' marks for their reflective portfolio assessment (r s = 0.167, P = 0.029). Students with more positive views about reflective portfolios were more likely to rate their reflection skills as good, receive better marks for their portfolio assessment, and be more confident building another portfolio. Discussion: This study begins to highlight preclinical medical students' views about reflective portfolios. However, further research is required using qualitative studies to explore students' views in depth. Medical educators should be encouraged to consider introducing portfolios as a method of formative and summative assessment earlier in the medical curriculum. From results: - Participant characteristics: Of the 206 students invited to participate in this study, 178 (86.4%) completed the reflective portfolio questionnaires satisfactorily. Students ranged in age from 18 to 30 years (median = 20, interquartile range = 19–20). The majority were female (n = 105, 59%), white (n = 130, 73.4%) and came from nonmanual socio-economic classes (n = 170, 95.5%). Students' ratings of their communication skills ranged from 1 to 5 (median = 3, interquartile range = 3–3) and nearly all (n = 173, 97.2%) thought their communication skills needed improving. Students' ratings of their

For peer review only

No.	Title	Author/year	MERSQI	COREQ	Reason for inclusion			Undergraduate (UG)/Postgraduate (PG)	Methodology	Purpose of study	Key findings
					Communication	Ethics	Professionalism				
									was written in the form of a statement and was accompanied by a 5-point Likert scale from 1 (strongly disagree) to 5 (strongly agree). Six medical educators with a range of experience of reflective portfolios commented on the language used in the preliminary draft of the questionnaire, which resulted in some minor alterations in language. The RPQ is scored by reversing the scores for the negative items and then summing the scores for all 18 items. The total score ranges from 18 to 90, with higher scores indicating more positive views about reflective portfolios. This scale was found to have good internal consistency in this study (Cronbach's α= 0.716) and the study results presented in this paper support the construct validity of the tool (copies of the RPQ can		reflection skills ranged from 1 to 5 (median = 3, interquartile range = 2–3) and most (n = 155, 87.6%) thought their reflection skills needed improving. ... (see the rest under perspective of students) Students' scores for their reflective portfolio coursework ranged from 33 to 100% (median = 67, interquartile range = 60–73%).<br>- Students' views about reflective portfolios: see the rest under perspective of students<br>From discussion: ... (see the rest under perspective of students) These findings have implications for further research and educational practice. Qualitative studies are needed to explore in depth the reasons why students possess the views they have about reflective portfolios. Qualitative research may also shed some light on how medical educators could increase students' confidence in building reflective portfolios. This qualitative research could help medical educators improve educational programs using portfolios, so that they are more acceptable to students. In the meantime, this study indicates that the act of constructing a reflective portfolio could increase students' confidence in building another portfolio. Despite portfolios being seen to have weaknesses (e.g. lacking reliability4), medical educators should be encouraged to consider introducing portfolios as a

No.	Title	Author/year	MERSQI	COREQ	Reason for inclusion			Undergraduate (UG)/Postgraduate (PG)	Methodology	Purpose of study	Key findings
					Communication	Ethics	Professionalism				
									be requested by contacting the author). Students' percentage marks for their reflective portfolio assessments were collected at a later date.; Data analysis -- After checking the distribution of continuous variables, data were analysed using univariate (i.e. Spearman's correlation coefficients and T -tests) and multivariate statistics (i.e. stepwise multiple regression) on SPSS Version 10.0.		method of formative and summative assessment earlier in the medical curriculum. Students may then be better placed to negotiate this type of assessment in higher stakes examinations.
75	The reliability of assessment criteria for undergraduate medical students' communication skills portfolios: the Nottingham experience	Rees, C. E. and Sheard, C. E.<br><br>2004	8.5		✓			UG	From abstract: Two independent analysts assessed a random sample of portfolios (n = 100, 49.5%) using criterion-referenced assessment. Students' performances were examined against subjective items in five areas: 1) portfolio structure, 2) level of critical reflection, 3) level of skills development, 4) use of documentary evidence, and 5) use of relevant literature. These	From abstract: This study aims to determine the reliability of assessment criteria used for a portfolio at the University of Nottingham.	From abstract -- Results: The level of agreement between the two raters for the total percentage score was 0.771 (95% CI = 0.678, 0.840), as measured by an intraclass correlation coefficient. The levels of agreement between the two raters for the individual items of the assessment criteria ranged from κ = 0.359 (item 3) to κ = 0.693 (item 4). Discussion: This study provides some support for the summative assessment of portfolios. The findings suggest that discussion and negotiation between independent assessors can enhance the reliability of assessment criteria. Therefore, medical educators are encouraged to use such procedures in the summative assessment

No.	Title	Author/year	MERSQI	COREQ	Reason for inclusion			Undergraduate (UG)/Postgraduate (PG)	Methodology	Purpose of study	Key findings
					Communication	Ethics	Professionalism				
									subjective judgements were later converted into quantitative scales ranging from 0 to 3 so that interrater reliability could be established. The level of agreement between the two analysts for the total percentage score was established using an intraclass correlation coefficient and for the individual items using weighted kappa coefficients. From methods: Sample -- Of the 202 portfolios submitted in March 2002, a random sample of 100 (49.5%) were photocopied for second marking. This random sample of 100 portfolios (median = 66.7%, interquartile range = 53.3–73.3%) did not differ significantly from the portfolios (n = 102) not chosen for second marking (median = 66.7%, interquartile range = 53.3–73.3%) in terms of their total percentage scores (Z = − 0.155, P = 0.877).; Assessment		of portfolios. From results: The level of agreement between the two raters for the total percentage scores, as measured by an intraclass correlation coefficient, was 0.771 (95% CI = 0.678, 0.840). The levels of agreement between the two raters for the individual items, as measured by weighted kappa coefficients, ranged from 0.359 (item 3) to 0.693 (item 4) (see Table 1).

For peer review only - http://bmjopen.bmj.com/site/about/guidelines.xhtml

No.	Title	Author/year	MERSQI	COREQ	Reason for inclusion: Communication	Reason for inclusion: Ethics	Reason for inclusion: Professionalism	Undergraduate (UG)/Postgraduate (PG)	Methodology	Purpose of study	Key findings
									procedures -- One hundred portfolios were assessed ... Although students were aware of these five areas, they were not give the full assessment criteria outlined in Appendix 1. ... (see the rest under deciding how to mark/assess); Statistical procedures: An intraclass correlation coefficient on SPSS (Version 10.0) was used to establish the level of agreement between the two raters for the total percentage scores. Weighted kappa coefficients on SAS (Release 6.12) were used to determine the levels of agreement between the two raters for the individual items of the assessment criteria.		
76	Portfolio as a tool to evaluate clinical competences of traumatology in medical students	Santonja-Medina, F., Garcia-Sanz, M. P., Martinez-Martinez, F., Bo, D. and Garcia-Estan, J.<br><br>2016	10.5		✓			UG	From abstract: A total of 131 students used the portfolio during their clinical rotation of traumatology. The students' portfolios were blind evaluated by four professors who annotated the existence	From abstract: This article investigates whether a reflexive portfolio is instrumental in determining the level of acquisition of clinical competences in traumatology, a subject in the 5th year of the degree of medicine.<br>From introduction: ...	From abstract: The reliability of the portfolio was moderate, according to the kappa index (0.48), but the evaluation scores between evaluators were very similar. Considering the mean percentage, 59.8% of the students obtained all the competences established and only 13 of the 23 learning outcomes (56.5%) were fulfilled by >50% of the

No.	Title	Author/year	MERSQI	COREQ	Reason for inclusion			Undergraduate (UG)/Postgraduate (PG)	Methodology	Purpose of study	Key findings
					Communication	Ethics	Professionalism				
									(yes/no) of 23 learning outcomes. From methods: A portfolio was designed to assess the level of acquisition of clinical competences, ... - Academic structure: Traumatology is a compulsory subject in the 5th year of the degree, consisting of 55 hours of theoretical classes, of which 28 hours take place in the classroom, 14 hours are of a seminar type, 10 hours are about clinical cases, and 3 hours are about cooperative learning; 21 hours of classes on laboratory skills in seven sessions; and 2 weeks of hospital rotation (50 hours, 10 days). Of these 50 hours, students rotate in the traumatology service of the university hospitals between hospital ward, medical consultation, operating rooms, and emergency services. Only one student is assigned to one professor in the	Thus, in the current article, we report the application of a portfolio tool to assess the level of acquisition of clinical skills and competences in medical students of the Medical School of the University of Murcia (Spain), during the clinical rotations of traumatology and orthopedic surgery. An important part of the study is also devoted to the analysis of the reliability of the portfolio.29	students. Our study suggests that the portfolio may be an important tool to quantitatively analyze the acquisition of traumatology competences of medical students, thus allowing the implementation of methods to improve its teaching. From results: A total of 138 comparisons were made to obtain the kappa index among the four evaluators for each of the 23 learning outcomes. The distribution of frequencies and percentages of agreement, according to the classification of Landis and Koch,29 are shown in Table 3. As it can be seen, out of 138 comparisons, 83 had kappa indexes >0.4 and 44 of them had values >0.6, which indicates that in a 31.9% of the occasions, the agreements between evaluators have been very substantial. Table 4 shows the mean and standard deviation of the kappa index of the four evaluators. It can be seen that the global kappa is 0.48, indicating that the portfolio has a moderate reliability, according to Landis and Koch.29 As an additional way of assessing the reliability of the portfolio, 6 months after the initial evaluation, two of the evaluators revised again the portfolios (second revision or reference). Table 5 shows the level of agreement obtained by each evaluator when both revisions were analyzed. Globally, the differences among evaluators are not very important because kappa indexes show that

For peer review only

No.	Title	Author/year	MERSQI	COREQ	Reason for inclusion			Undergraduate (UG)/Postgraduate (PG)	Methodology	Purpose of study	Key findings
					Communication	Ethics	Professionalism				
									hospital. Table 2 shows the number of days students spent during these rotations.<br>- Portfolio structure: The portfolio was given to all the students during the course (n=131) in a booklet format. After the identification of data, the students read about legal and ethical issues in relation to the need for keeping medical secret and signed conformity. Each day the students were expected to fill up their reflections before leaving the hospital, writing about the clinical activities observed during the day. In addition, the portfolio had to be signed by the assigned professor every day. The students were given enough space to include everything needed, but there were three main paragraphs: ... (see under content of portfolio)<br>- Portfolio evaluation: The portfolios were evaluated by four		evaluators 1 and 4 have a substantial agreement in both revisions, whereas evaluators 2 and 3 have a moderate level of agreement. The level of achievement of the traumatology competences is shown in Table 1. The data show the mean percentage of the four evaluations as well as the number of times the procedures were performed. A total of 59.8% of the students have acquired the competences as expected during their hospital stay. In all, 13 of the 23 learning outcomes (56.5%) have been acquired by >50% of the students. Competence 5 (87.4%) and competence 1 (81.4%) show the greater values, whereas the rest of them show an acceptable level, except competence 7, showing only a value of 26%.<br>From discussion: The current article shows the results obtained with the application of a portfolio to medical students as a tool to obtain information regarding the level of acquisition of the clinical competences of the topic traumatology. One of the important aspects of any portfolio is its reliability, the consistency and accuracy of the assessment tool in measuring students' performance. Our results quite agree with those found by other authors;30,31 although in some studies of nurse students, higher numbers (0.8) have been described.32 To prove that the results were not obtained by chance, the

No.	Title	Author/year	MERSQI	COREQ	Reason for inclusion			Undergraduate (UG)/Postgraduate (PG)	Methodology	Purpose of study	Key findings
					Communication	Ethics	Professionalism				
									professors, three of them professors of traumatology and one a colleague professor from a different area, that is, physiology; all of them were medical doctors. ... (see the rest under deciding how to mark/assess) All the portfolios underwent blind evaluation. All the students agreed and participated enthusiastically in the study.<br>- Statistical analysis: Data analysis was performed using the Statistical Package for the Social Sciences, Version 19. The Cohen's kappa coefficient was used as a statistical measure of interevaluator agreement for qualitative items.29 Frequencies, percentages, means, and standard deviations were also obtained to describe the results.		second review of the portfolios took place 6 months after the initial evaluation in order to see whether the reliability index could be improved. As observed in Table 5, the maximum level of agreement among evaluators is found in competence 6 (observe and assist in surgical treatments). A moderate agreement exists in competences 2, 3, and 4, whereas competences 1, 5, and 7 show a low level of agreement among evaluators. The results of Table 1 show important information regarding the level of acquisition of the competences analyzed. Two of them, competences 1 and 5, related to diagnostic and treatment obtained the greatest value, with an important frequency of occurrence. Next competence in terms of the level of acquisition is number 3 (70%), probably due to the high number of radiographic images viewed in contrast to magnetic resonance images, where it is likely that doctors rely more on the radiologist report, without actually interpreting them. Competence 6 has also a moderate level of acquisition (55.8%), in spite of the fact that almost all students (99.2%) report attendance to operating rooms (2.6 + 1.3 days). This is likely due to the fact that the surgeons do not invite all the students to wash and be prepared to help, an activity we recommend to all our professors. A similar

For peer review only

No.	Title	Author/year	MERSQI	COREQ	Reason for inclusion			Undergraduate (UG)/Postgraduate (PG)	Methodology	Purpose of study	Key findings
					Communication	Ethics	Professionalism				
											reason may be behind the moderate level achieved in competences 2 (47.1%) and 4 (51.5%), showing that not all the students are able to explore or perform maneuvers with the patients and that this may be due to a time limitation problem while in the ward or consultation. We believe that this should be corrected and doctors should allow more time for the students to perform, under the appropriate supervision, exploration, or treatment of the patients. Finally, competence 7 has the lowest level. It is likely related to the fact that students are not allowed to sign discharge reports or other forms, although it is important that they learn from their professors how to do it. In any case, this is the first time that we obtained this quantitative information regarding the clinical stays of the medical students and this will help us to analyze our teaching results and to develop new strategies to enhance the level of clinical competence the students should reach. A problem learned from the application of this portfolio is that many of the learning outcomes were checked without taking into account the number of times these were carried out. The rest of the learning outcomes were quantitative, so we could obtain the number of times the student performed them. For instance, they attended the operating rooms 5.5 + 3.3 times (range, 1–26), and viewed a mean of 2.3 magnetic resonance images

For peer review only

No.	Title	Author/year	MERSQI	COREQ	Reason for inclusion			Undergraduate (UG)/Postgraduate (PG)	Methodology	Purpose of study	Key findings
					Communication	Ethics	Professionalism				
											(range, 1–14). We believe that it is important that the portfolios are designed to collect also the quantitative information, since both quantitative and qualitative information give the real essence of the learning process. A drawback we have seen with the application of the portfolio to clinical students is that there is a random factor affecting the acquisition of the competences. This is due to the fact that the students rotate between different hospitals and at different times of the year. Thus, it depends on the type of medical problems available on the days they are assigned to the hospital; thus they may not see all the problems selected in the portfolio. Clearly, the greater the number of days of hospital stay, the better it is. However, this is not always possible. A better selection of the learning outcomes will also be helpful. Moreover, the portfolio is designed to be an evaluation tool that should be used in conjunction with others (written or oral exams, laboratory evaluations) because it is not possible to find just one tool that is able to assess all the competences.33,34<br>From conclusion: In conclusion, the portfolio is an important tool that has allowed to obtain qualitative and quantitative data regarding the degree of acquisition of traumatology competences by medical students. Although some factors need to be improved, such as the

For peer review only

No.	Title	Author/year	MERSQI	COREQ	Reason for inclusion			Undergraduate (UG)/Postgraduate (PG)	Methodology	Purpose of study	Key findings
					Communication	Ethics	Professionalism				
											inclusion of more quantitative elements, a more comprehensive selection of learning outcomes, and combination with other tools, we believe that the use of this kind of tool by clinical teachers will be useful to ascertain the degree of practical competence reached by their students.
77	Assessing the Development of Medical Students' Personal and Professional Skills by Portfolio	Yielder, J. and Moir, F.<br><br>2016		6			✓	UG	The implementation process employed a cyclical model that was designed to evaluate and improve the use of the portfolio, that could form the basis of the first cycle of action research that will be continued in the future to improve the initiative (Fig. 1). During 2013, the introduction of the portfolio was monitored, and changes were made in 2014. In 2014, feedback from students indicated the need for further modifications, which are currently being implemented, along with the introduction of an electronic platform. Student feedback was ascertained from the generic end-of-year course evaluation, which included	From abstract: We present the portfolio format along with the process for its introduction and appraise the challenges, strengths, and limitations of the approach within the context of the current literature. We then outline a cyclical model of evaluation used to monitor and fine-tune the portfolio tasks and implementation process, in response to student and assessor feedback.<br>From introduction: This document outlines the learning outcomes that are to be substantively completed by the end of the second postgraduate year in practice. ... This article presents the process for introducing the portfolio, including the challenges, strengths and limitations, evaluation of data to date, and the ways in which student	From abstract: The portfolios have illustrated the level of insight, maturity, and synthesis of personal and professional qualities that students are capable of achieving. The Auckland medical program strives to foster these qualities in its students, and the portfolio provides an opportunity for students to demonstrate their reflective abilities. Moreover, the creation of a Personal and Professional Skills domain with the portfolio as its key assessment emphasizes the importance of reflective practice and personal and professional development and gives a clear message that these are fundamental longitudinal elements of the program.<br>From conclusion:<br>The implementation of a portfolio for the assessment of the PPS domain in the medical program at the University of Auckland has proved to be an important part of the redeveloped curriculum. It has highlighted the importance of PPS topics and has provided an opportunity and incentive for students to reflect on aspects of their personal and professional development, with the aim

For peer review only

No.	Title	Author/year	MERSQI	COREQ	Reason for inclusion			Undergraduate (UG)/Postgraduate (PG)	Methodology	Purpose of study	Key findings
					Communication	Ethics	Professionalism				
									questions relating to the PPS domain. The number of students in each year varied from 199–260 and there was an average response rate of 50%. In addition, the Auckland University Medical Students Association conducted a student evaluation for the purpose of writing a report for the ongoing medical program accreditation, from which key issues were collated and forwarded to the PPS domain coordinators. The eight portfolio assessors also provided suggestions for improvement at the end of 2013 and 2014. Evaluation feedback, modifications, and qualitative data derived from the portfolios are presented in the Findings section. Due to the qualitative nature of the feedback, it was analyzed inductively, through a cross-sectional thematic analysis that sought to	feedback has been addressed.	of improving their practice as doctors. In the process, we have learned several aspects about students that previously may have remained unknown, which has led in some cases to conversations with students that will hopefully make a difference to their learning and development trajectories. We have also seen many high-quality and insightful portfolios that demonstrate a synthesis of personal and professional qualities that are inspiring and which we hope are an indication that these students will develop into excellent doctors. With the increasing use of, and requirement for, the compilation of professional portfolios in the medical profession internationally, especially at postgraduate level, there is real benefit for students in becoming familiar with this process of pregraduation.<br>It has also been a learning process for the domain coordinators introducing the assessment. In particular, we learned the value of a cyclic, flexible, and ongoing process of evaluation that has allowed us to continuously reflect on what is working and what needs to change in order to be responsive to student needs and at the same time create a curriculum change that aims to create better doctors. ... We hope that as students come to trust our responsiveness, they will become increasingly open to the importance of taking on the responsibility for their

For peer review only

No.	Title	Author/year	MERSQI	COREQ	Reason for inclusion			Undergraduate (UG)/Postgraduate (PG)	Methodology	Purpose of study	Key findings
					Communication	Ethics	Professionalism				
									ascertain the strongest themes from across the three sources of feedback.		own learning in the PPS domain.
78	Construct Validity of an Instrument for Assessment of Reflective Writing-Based Portfolios of Medical Students	Kassab, Salah Bidmos, Mubarak Nomikos, Michail Daher-Nashif, Suhad Kane, Tanya Sarangi, Srikant Abu-Hijleh, Marwan<br><br>2020	7		✓		✓	UG	After an extensive literature review and pilot testing of the instrument, two raters assessed the reflective writing-based portfolios from years 2 and 3 medical students (n=135) on three occasions. The instrument consists of three criteria: organization, description of an experience and reflection on the experience. We calculated the reliability of scores using generalizability theory with a fully crossed design and two facets (raters and occasions). In addition, we measured criterion validity by testing correlations with students' scores using other assessment methods.	Assessment of reflective writing for medical students is challenging, and there is lack of an available instrument with good psychometric properties. The authors developed a new instrument for assessment of reflective writing-based portfolios and examined the construct validity of this instrument.	The dependability (Φ) coefficient of the portfolio scores was 0.75 using two raters on three occasions. Students' portfolio scores represented 46.6% of the total variance across all score comparisons. The variance due to occasions was negligible, while the student– occasion interaction was small. The variance due to student–rater interaction represented 17.7%, and the remaining 27.7% of the variance was due to unexplained sources of error. The decision (D) study suggested that an acceptable dependability (Φ = 0.70 and 0.72) can be achieved by using two raters for one and two occasions, respectively. Finally, we found moderate to large effect-size correlations between students' scores in reflective writing-based portfolios and communication skills (r = 0.47) and PBL tutorials (r = 0.50).<br><br>We demonstrated the presence of different sources of evidence that support construct validity of the study instrument. Further studies are warranted before utilizing this instrument for summative assessment of students' reflective writing-based portfolios in other medical schools.
79	Communication skills teaching	Douglas, A. H. Acharya, S. P.		19	✓			UG	This study is a qualitative	Communication skills (194) are vital for	The two main themes: 1. Positivity

For peer review only

No.	Title	Author/year	MERSQI	COREQ	Reason for inclusion			Undergraduate (UG)/Postgraduate (PG)	Methodology	Purpose of study	Key findings
					Communication	Ethics	Professionalism				
	and learning in Nepal; what are medical students' perceptions and experiences? A qualitative study	Allery, L. A. 2020							evaluation of a CS course in Nepal, exploring the experiences and perceptions of participants. The study aims to also identify aspects that were helpful or not for student learning and areas for potential development. A purposive sample of twenty: second, fourth and Intern year students was selected for interview. Data were collected through audio recorded semi-structured interviews following a piloted schedule. Interview transcripts were manually coded and thematically analysed. Codes were arranged into themes and subthemes.	doctors. Indeed, as the most important element of consultations, are highly valued by patients. CS are core, teachable skills, however, have not been widely taught in South Asian medical schools, unlike their western counterparts. Patan Academy of Health Sciences, is one of the first in Nepal to have CS central to its' aims and curriculum. CS are taught from the first weeks of medical school and re-enforced during preclinical study (first 2 years). Our study seeks to explore students' perceptions and experiences of CS teaching in this South Asian, Nepal context.	2. Experiential learning. Results demonstrate participants' positive perceptions of CS teaching: believing it is important, effective, relevant and valuable for personal development. Participants identified experiential learning features as valuable for CS acquisition. Intern students recognised CS relevance and requested expanding teaching to clinical years, incorporating challenging communication scenarios. This study shows that PAHS' CS course is well perceived and valuable to learners. Experiential learning is powerful for CS development. Expansion of formal, structured CS teaching through all years in a spiral curriculum, should be considered. Violence towards doctors in South Asia is increasing. Students recognised CS teaching's significance in addressing this. CS teaching,still in its' infancy in South Asia, is a pressing issue for medical educators here. Our study provides evidence it is well perceived with positive impacts in this context, particularly when employing experiential learning. Medical schools in south Asia should be encouraged to incorporate and strengthen their CS teaching curriculum.
80	Assessment practices in undergraduate	Brits, H. Bezuidenhout, J. Van der Merwe, L. J.	5.5	12	✓			UG	A descriptive cross-sectional study design was	Assessment should form an integral part of curriculum design	All disciplines in the undergraduate medical programme are

For peer review only - http://bmjopen.bmj.com/site/about/guidelines.xhtml

No.	Title	Author/year	MERSQI	COREQ	Reason for inclusion			Undergraduate (UG)/Postgraduate (PG)	Methodology	Purpose of study	Key findings
					Communication	Ethics	Professionalism				
	clinical medicine training: What do we do and how can we improve?	Joubert, G.<br><br>2020							used. Qualitative and quantitative data were gathered by means of open- and closed-ended questions in a self-administered questionnaire, which was completed by teaching and learning coordinators in 13 disciplines.	in higher education and should be robust enough to ensure clinical competence.<br><br>This article reports on current assessment practices and makes recommendations to improve clinical assessment in the undergraduate medical programme at the University of the Free State.	represented. They used different assessment methods to assess the competencies required of entry-level healthcare professionals. Workplace-based assessment was performed by 30.1% of disciplines, while multiple-choice questions (MCQs) (76.9%) and objective structured clinical examinations (OSCEs) (53.6%) were the main methods used during formative assessment. Not all assessors were well prepared for assessment, with 38.5% never having received any formal training on assessment. Few disciplines (15.4%) made use of post-assessment moderation as a standard practice, and few disciplines always gave feedback after assessments.<br><br>The current assessment practices for clinical students in the undergraduate medical programme at the University of the Free State cover the spectrum that is necessary to assess all the different competencies required. Multiple-choice questions and OSCEs, which are valid and reliable assessment methods, are used frequently. Poor feedback and moderation practices should be addressed. More formative assessments, and less emphasis on summative assessment, should be considered. Workplace-based and continuous assessments may be good ways to assess clinical competence.

For peer review only

No.	Title	Author/year	MERSQI	COREQ	Reason for inclusion			Undergraduate (UG)/Postgraduate (PG)	Methodology	Purpose of study	Key findings
					Communication	Ethics	Professionalism				
81	**Added value of assessing medical students' reflective writings in communication skills training: a longitudinal study in four academic centres**	Ament Giuliani Franco, C.<br>Franco, R. S.<br>Cecilio-Fernandes, D.<br>Severo, M.<br>Ferreira, M. A.<br>de Carvalho-Filho, M. A.<br><br>2020	10.5		✓			UG	Third-year and fourth-year medical students enrolled in an elective course on clinical communication skills development were assessed using different assessment methods.<br><br>The communication skills course was offered at four universities (three in Brazil and one in Portugal) and included 69 students.<br><br>The students were assessed by a Multiple-Choice Questionnaire (MCQ), an objective structured clinical examination (OSCE) and reflective writing narratives. The Cronbach's alpha, dimensionality and the person's correlation were applied to evaluate the reliability of the assessment methods and their correlations. Reflective witting was assessed by applying the Reflection Evaluation for Enhanced Competencies Tool Rubric	This study describes the development and implementation of a model to assess students' communication skills highlighting the use of reflective writing. We aimed to evaluate the usefulness of the students' reflections in the assessment of communication skills.	The Cronbach alpha for the MCQ, OSCE global score, TS and RS were, respectively, 0.697, 0.633, 0.784 and 0.850. The interobserver correlation for the TS and RS were, respectively, 0.907 and 0.816. The assessment of reflection using the TS was significantly correlated with the MCQ (r=0.412; p=0.019), OSCE (0.439; p=0.012) and RS (0.410; p=0.020). The RS did not correlate with the MCQ and OSCE.<br><br>Assessing reflection through mapping the themes and analysing the depth of reflective writing expands the assessment of communication skills. While the assessment of reflective themes is related to the cognitive and behavioural domains of learning, the reflective depth seems to be a specific competence, not correlated with other assessment methods—possibly a metacognitive domain.

For peer review only

No.	Title	Author/year	MERSQI	COREQ	Reason for inclusion			Undergraduate (UG)/Postgraduate (PG)	Methodology	Purpose of study	Key findings
					Communication	Ethics	Professionalism				
									(Reflect Score (RS)) to measure reflections' depth, and the Thematic Score (TS) to map and grade reflections' themes.		
82	Contextual Analysis of Stakeholder Opinion on Management and Leadership Competencies for Undergraduate Medical Education: Informing Course Design	Rajeh, N. Grant, J. Farsi, J. Tekian, A.<br><br>2020		16	✓			UG	This was a cross-sectional study conducted at King Abdulaziz University (KAU), Saudi Arabia, during 2019. An exploratory qualitative approach, utilizing systematic content analysis, was used. Data were collected using semi-structured interviews that were con- ducted with 10 leaders who were stakeholders at KAU, health service providers at KAU hospital, and stakeholders in the Ministry of Health.	The study aimed to conduct a contextual analysis of interviews intended to assist with the future design of a feasible and relevant leadership and management course for undergraduate medical students at King Abdulaziz University (KAU), Saudi Arabia.	This study revealed critical findings that highlighted the areas in which KAU could instill better and adequate leadership and management skills in their undergraduate medical students. Multiple core categories for a leadership and management curriculum emerged with many interrelated themes. Most participants mentioned that leadership can be taught and that early exposure is beneficial for develop- ing skills. Additionally, they stated that leaders should have a vision and the ability to articulate that vision.<br><br>Different implementation challenges were described in relation to the availability of human resources, the current short supply of suitable teachers, and program design. Teaching methods recommended included simulations, lectures, and a project-based approach. Assessment methods that were recommended included objective structured clinical examination (OSCE), formative and summa- tive assessments, self-assessments, and portfolios.

For peer review only

Appendix C

Portfolio Content (Competencies Assessed and Assessment Modalities)

**Subthemes**	**Content**		
	**Communication**	**Ethics**	**Professionalism**
**Competencies Assessed**	• "Effective" communication (65, 67, 68, 81, 86, 107, 127, 131-133)<br>• Professional behaviour and moral reasoning (71)<br>• Systems awareness and team-based care (71)<br>• Continuous learning and quality improvement (71)<br>• Patient-centred medical care (71)<br>• Teamwork (70)<br><br>Oral communication skills<br>• Attentive, active listening (84, 139, 140)<br>• Explanation, information giving (127, 139)<br>• History taking (93)<br>• Consultation (141)<br>• Breaking bad news (24, 139)<br>• Shared decision making (142)<br>• Empathetic communication (142)<br>• Taking history and clinical examination (84, 93)<br>• Discerning appropriate language to ensure patient understands (24, 127)<br>• Conflict management (139)<br>• Adaptability to different settings (67)<br><br>Written communication skills<br>• Proper grammar, spelling, logical and coherent structure (103)<br>• Patient cases (93)<br>• Scientific research (93)<br><br>Communication with:<br>• Patients (24, 68, 84, 93, 127, 139, 143)<br>• Children (127)<br>• Family (68, 84, 139, 144)<br>• Colleagues, interprofessional team (24, 68, 84, 93, 130, 139)	• Ethics in interprofessional practice (24, 67, 86, 128, 130, 145, 146)<br>• Doctor-patient relationship (81, 141, 145)<br>• Values/ ethics (67, 70, 86, 146)<br>• Ethical and legal responsibilities (65, 86)<br>• Moral reasoning (89)<br>• Ethics is assessed as a component of professionalism (85, 87, 88)<br>• Empathy (84)<br>• Showing respect and commitment (84)<br>• Respect for privacy (84)<br>• How to express ethical views appropriately (82)<br>• Identifying unethical moments of practice and grey areas (85, 89, 90)<br><br>Reflection:<br>• Quality of student's reflections (82, 84, 87)<br>• Authenticity of reflection (87)	• Learning from clinical experience (24, 81)<br>• Working in groups/ teamwork (24, 67, 70, 81, 86, 128, 130, 134, 145)<br>• Doctor-patient relationship (81, 141, 145)<br>• Becoming a doctor/roles and responsibilities (81, 86, 128, 130, 134, 145, 146)<br>• Patient care (67, 70, 128, 130)<br>• Continuous learning (67, 70)<br>• Values/ ethics (67, 70, 86, 146)<br>• Interpersonal skills/ effective Communication, (24, 67, 128, 145, 146)<br><br>Reflection:<br>• Clinical experience (81)<br>• Teaching (81)<br>• Learning needs in becoming a doctor<br>• Strengths and developmental areas (65, 86)<br>• Understanding of concepts addressed (86)<br>• Dilemmas and doubts (133)

	• Public (68)		
**Assessment Modalities (Portfolio items)**	Formative only (67, 84, 87, 92, 119, 126, 130, 146)<br>Summative only (65, 70, 71, 81-83, 87, 104, 116, 131, 134)<br>Both formative and summative (24, 67, 83, 86, 87, 117, 122, 128, 130, 134)<br>MCQs (65, 68, 87, 95, 126, 141, 143, 144, 146)<br>OSCEs (65, 67, 70, 71, 87, 126, 127, 130, 141, 143, 144, 146)<br>Essays – topical (67, 81, 93, 126)<br>Mini clinical examination exercise (Mini-CEX) (65, 67, 70, 87, 93, 130, 139, 143, 146)<br>Medical/patient presentations/research (67, 68, 80, 86, 93, 121, 126, 129)<br>Workplace-based clinical (67, 70, 71, 80, 83, 93, 107, 112, 122, 126, 127, 130, 139, 143)<br>Case-based discussions, summaries (65, 68, 83, 85, 89, 92, 129)(19)(81, 129) (83, 126, 130)<br>Simulated patient assessment (65, 68, 84, 103, 112)<br>Team-based assessment (65, 83, 86, 93)<br>Logbooks/ Portfolio interview (65, 67, 68, 70, 81-85, 87, 88, 103, 121, 126) (67, 121, 126)<br>Peer evaluation (67, 70, 71, 80, 85, 93, 107, 126, 127)<br>Faculty evaluation (67, 68, 70, 71, 80, 81, 83-86, 117, 126, 131, 147)<br>Feedback reports (75, 77, 80, 86, 91, 93, 109, 112, 122, 126, 130, 134, 139, 143) – multisource feedback (75, 77, 81, 82, 85, 86, 91, 93, 103, 109, 127, 130)<br>Self-assessment/appraisal (67, 68, 80, 82, 93, 104, 126, 130, 134)<br>Self-reflections – written/video/diaries (65, 67, 68, 71, 80-83, 85, 87, 88, 90, 93, 103, 107, 121, 127-130)<br>Learning plans and contracts (67, 68, 71, 80-83, 86, 92, 93, 103, 133)<br>Letters of recommendation (65, 81, 103)<br>Curriculum vitae (65, 67, 70, 83, 86, 122, 126)		

For peer review only

Preferred Reporting Items for Systematic reviews and Meta-Analyses extension for Scoping Reviews (PRISMA-ScR) Checklist

SECTION	ITEM	PRISMA-ScR CHECKLIST ITEM	REPORTED ON PAGE #
**TITLE**			
Title	1	Identify the report as a scoping review.	1
**ABSTRACT**			
Structured summary	2	Provide a structured summary that includes (as applicable): background, objectives, eligibility criteria, sources of evidence, charting methods, results, and conclusions that relate to the review questions and objectives.	2
**INTRODUCTION**			
Rationale	3	Describe the rationale for the review in the context of what is already known. Explain why the review questions/objectives lend themselves to a scoping review approach.	4
Objectives	4	Provide an explicit statement of the questions and objectives being addressed with reference to their key elements (e.g., population or participants, concepts, and context) or other relevant key elements used to conceptualize the review questions and/or objectives.	4
**METHODS**			
Protocol and registration	5	Indicate whether a review protocol exists; state if and where it can be accessed (e.g., a Web address); and if available, provide registration information, including the registration number.	5
Eligibility criteria	6	Specify characteristics of the sources of evidence used as eligibility criteria (e.g., years considered, language, and publication status), and provide a rationale.	5
Information sources*	7	Describe all information sources in the search (e.g., databases with dates of coverage and contact with authors to identify additional sources), as well as the date the most recent search was executed.	5
Search	8	Present the full electronic search strategy for at least 1 database, including any limits used, such that it could be repeated.	5
Selection of sources of evidence†	9	State the process for selecting sources of evidence (i.e., screening and eligibility) included in the scoping review.	5
Data charting process‡	10	Describe the methods of charting data from the included sources of evidence (e.g., calibrated forms or forms that have been tested by the team before their use, and whether data charting was done independently or in duplicate) and any processes for obtaining and confirming data from investigators.	6
Data items	11	List and define all variables for which data were sought and any assumptions and simplifications made.	7
Critical appraisal of individual sources of evidence§	12	If done, provide a rationale for conducting a critical appraisal of included sources of evidence; describe the methods used and how this information was used in any data synthesis (if appropriate).	7

St. Michael's
Inspired Care. Inspiring Science.

For peer review only - http://bmjopen.bmj.com/site/about/guidelines.xhtml

SECTION	ITEM	PRISMA-ScR CHECKLIST ITEM	REPORTED ON PAGE #
Synthesis of results	13	Describe the methods of handling and summarizing the data that were charted.	8
**RESULTS**			
Selection of sources of evidence	14	Give numbers of sources of evidence screened, assessed for eligibility, and included in the review, with reasons for exclusions at each stage, ideally using a flow diagram.	8
Characteristics of sources of evidence	15	For each source of evidence, present characteristics for which data were charted and provide the citations.	8
Critical appraisal within sources of evidence	16	If done, present data on critical appraisal of included sources of evidence (see item 12).	Appendix B
Results of individual sources of evidence	17	For each included source of evidence, present the relevant data that were charted that relate to the review questions and objectives.	Appendix B
Synthesis of results	18	Summarize and/or present the charting results as they relate to the review questions and objectives.	8
**DISCUSSION**			
Summary of evidence	19	Summarize the main results (including an overview of concepts, themes, and types of evidence available), link to the review questions and objectives, and consider the relevance to key groups.	9-13
Limitations	20	Discuss the limitations of the scoping review process.	15
Conclusions	21	Provide a general interpretation of the results with respect to the review questions and objectives, as well as potential implications and/or next steps.	15
**FUNDING**			
Funding	22	Describe sources of funding for the included sources of evidence, as well as sources of funding for the scoping review. Describe the role of the funders of the scoping review.	None

JBI = Joanna Briggs Institute; PRISMA-ScR = Preferred Reporting Items for Systematic reviews and Meta-Analyses extension for Scoping Reviews.
* Where *sources of evidence* (see second footnote) are compiled from, such as bibliographic databases, social media platforms, and Web sites.
† A more inclusive/heterogeneous term used to account for the different types of evidence or data sources (e.g., quantitative and/or qualitative research, expert opinion, and policy documents) that may be eligible in a scoping review as opposed to only studies. This is not to be confused with *information sources* (see first footnote).
‡ The frameworks by Arksey and O'Malley (6) and Levac and colleagues (7) and the JBI guidance (4, 5) refer to the process of data extraction in a scoping review as data charting.
§ The process of systematically examining research evidence to assess its validity, results, and relevance before using it to inform a decision. This term is used for items 12 and 19 instead of "risk of bias" (which is more applicable to systematic reviews of interventions) to include and acknowledge the various sources of evidence that may be used in a scoping review (e.g., quantitative and/or qualitative research, expert opinion, and policy document).

From: Tricco AC, Lillie E, Zarin W, O'Brien KK, Colquhoun H, Levac D, et al. PRISMA Extension for Scoping Reviews (PRISMAScR): Checklist and Explanation. Ann Intern Med. 2018;169:467–473. doi: 10.7326/M18-0850.

For peer review only - http://bmjopen.bmj.com/site/about/guidelines.xhtml

BMJ Open

An evidence-guided approach to Portfolio guided Teaching and Assessing Communications, Ethics and Professionalism for medical students and physicians:A Systematic Scoping Review

Journal:	*BMJ Open*
Manuscript ID	bmjopen-2022-067048.R3
Article Type:	Original research
Date Submitted by the Author:	09-Mar-2023

Complete List of Authors:	Ting, Jacquelin Jia Qi; National University of Singapore, Yong Loo Lin School of Medicine; National Cancer Centre Singapore, Division of Supportive and Palliative Care<br>Phua, Gillian Li Gek; National Cancer Centre Singapore, Division of Supportive and Palliative Care; Duke-NUS Medical School, Lien Centre for Palliative Care<br>Hong, Daniel Zhihao; National University of Singapore, Yong Loo Lin School of Medicine; National Cancer Centre Singapore, Division of Supportive and Palliative Care<br>Lam, Bertrand Kai Yang; National Cancer Centre Singapore, Division of Supportive and Palliative Care<br>Lim, Annabelle Jia Sing ; National University of Singapore, Yong Loo Lin School of Medicine; National Cancer Centre Singapore, Department of Supportive and Palliative Care<br>Chong, Eleanor Jia Xin; National University of Singapore, Yong Loo Lin School of Medicine; National Cancer Centre Singapore, Division of Supportive and Palliative Care<br>Pisupati, Anushka; National University of Singapore, Yong Loo Lin School of Medicine; National Cancer Centre Singapore, Division of Supportive and Palliative Care<br>Tan, Rei; National University of Singapore, Yong Loo Lin School of Medicine; National Cancer Centre Singapore, Division of Supportive and Palliative Care<br>Yeo, Jocelyn Yi Huang; National University of Singapore, Yong Loo Lin School of Medicine; National Cancer Centre Singapore, Division of Supportive and Palliative Care<br>Koh, Yi Zhe; National University of Singapore, Yong Loo Lin School of Medicine; National Cancer Centre Singapore, Division of Supportive and Palliative Care<br>Quek, Chrystie; National University of Singapore, Yong Loo Lin School of Medicine; National Cancer Centre Singapore, Division of Supportive and Palliative Care<br>Lim, Jia Yin; National University of Singapore, Yong Loo Lin School of Medicine; National Cancer Centre Singapore, Division of Supportive and Palliative Care<br>Tay, Kuang Teck; National Cancer Centre Singapore, Division of Supportive and Palliative Care<br>Ong, Yun Ting; National University of Singapore, Yong Loo Lin School of

	Medicine; National Cancer Centre Singapore Division of Supportive and Palliative Care<br>Chiam, Min; National Cancer Centre Singapore, Division of Cancer Education<br>Zhou, Xuelian Jamie; National Cancer Centre Singapore, Division of Supportive and Palliative Care; Duke-NUS Medical School, Lien Centre for Palliative Care<br>Mason, Stephen; University of Liverpool, Palliative Care Institute Liverpool<br>Wijaya, Limin; National Cancer Centre Singapore, Division of Supportive and Palliative Care; Duke-NUS Medical School<br>Krishna, Lalit; University of Liverpool,

**Primary Subject Heading**:	Communication
Secondary Subject Heading:	Communication, Ethics, Medical education and training
Keywords:	MEDICAL EDUCATION & TRAINING, ETHICS (see Medical Ethics), EDUCATION & TRAINING (see Medical Education & Training)

SCHOLARONE™
Manuscripts

I, the Submitting Author has the right to grant and does grant on behalf of all authors of the Work (as defined in the below author licence), an exclusive licence and/or a non-exclusive licence for contributions from authors who are: i) UK Crown employees; ii) where BMJ has agreed a CC-BY licence shall apply, and/or iii) in accordance with the terms applicable for US Federal Government officers or employees acting as part of their official duties; on a worldwide, perpetual, irrevocable, royalty-free basis to BMJ Publishing Group Ltd ("BMJ") its licensees and where the relevant Journal is co-owned by BMJ to the co-owners of the Journal, to publish the Work in this journal and any other BMJ products and to exploit all rights, as set out in our licence.

The Submitting Author accepts and understands that any supply made under these terms is made by BMJ to the Submitting Author unless you are acting as an employee on behalf of your employer or a postgraduate student of an affiliated institution which is paying any applicable article publishing charge ("APC") for Open Access articles. Where the Submitting Author wishes to make the Work available on an Open Access basis (and intends to pay the relevant APC), the terms of reuse of such Open Access shall be governed by a Creative Commons licence – details of these licences and which Creative Commons licence will apply to this Work are set out in our licence referred to above.

Other than as permitted in any relevant BMJ Author's Self Archiving Policies, I confirm this Work has not been accepted for publication elsewhere, is not being considered for publication elsewhere and does not duplicate material already published. I confirm all authors consent to publication of this Work and authorise the granting of this licence.

For peer review only

Title: An evidence-guided approach to Portfolio guided Teaching and Assessing Communications, Ethics and Professionalism for medical students and physicians: A Systematic Scoping Review

Short Title: A review of CEP Portfolios for physicians and medical students

Jacquelin Jia Qi Ting[1,2], Gillian Phua[2,3,4], Daniel Zhihao Hong [1,2], Bertrand Kai Yang Lam[2], Annabelle Jia Sing Lim[1,2], Eleanor Jia Xin Chong[1,2], Anushka Pisupati[1,2] , Rei Tan[1,2], Jocelyn Yi Huang Yeo[1,2], Yi Zhe Koh[1,2], Chrystie Wan Ning Quek[1,2], Jia Yin Lim[1,2], Kuang Teck Tay[2], Yun Ting Ong[1,2], Min Chiam[5,] , Jamie Zhou [2,3,4], Stephen Mason[8], Limin Wijaya[2,3,9], Lalit Kumar Radha Krishna[1-8,#]

[1] Yong Loo Lin School of Medicine, National University of Singapore, NUHS Tower Block, 1E Kent Ridge Road, Level 11, Singapore 119228

[2] Division of Supportive and Palliative Care, National Cancer Centre Singapore,11 Hospital Dr, Singapore 169610

[3] Duke-NUS Medical School, 8 College Rd, Singapore 169857

[4] Lien Centre for Palliative Care, Duke-NUS Medical School, 8 College Rd, Singapore 169857

[5] Division of Cancer Education, National Cancer Centre Singapore, 11 Hospital Dr, Singapore 169610

[6] PalC, The Palliative Care Centre for Excellence in Research and Education, PalC c/o Dover Park Hospice, 10 Jalan Tan Tock Seng, Singapore

[7] Centre of Biomedical Ethics, National University of Singapore, 21 Lower Kent Ridge Rd, Singapore 119077

[8] Palliative Care Institute Liverpool, Academic Palliative & End of Life Care Centre, Cancer Research Centre, University of Liverpool, 200 London Rd, Liverpool L3 9TA, United Kingdom

[9] Department of Infectious Diseases, Singapore General Hospital, Outram Road, Singapore 169608

[#]Corresponding Author:

Dr. Lalit Kumar Radha Krishna

Palliative Care Institute Liverpool, Academic Palliative & End of Life Care Centre,

University of Liverpool, 200 London Rd, Liverpool L3 9TA, United Kingdom

Email: lalit.radha-krishna@liverpool.ac.uk Phone number: 6436 8183

For peer review only

For peer review only - http://bmjopen.bmj.com/site/about/guidelines.xhtml

ABSTRACT

Objectives

Guiding the development of longitudinal competencies in communication, ethics, and professionalism underlines the role of portfolios to capture and evaluate the multiple multi-source appraisals, and direct personalised support to clinicians. However a common approach to these combined portfolios continues to elude medical practice. A systematic scoping review is proposed to map portfolio use in training and assessments of ethics, communication, and professionalism competencies particularly as its inculcation of new values, beliefs and principles changes attitudes, thinking, practice, while nurturing Professional Identity Formation. It is posited that effective structuring of portfolios promote self-directed learning, personalised assessment and appropriate support of professional identity formation.

Design

Krishna's Systematic Evidence-Based Approach is employed to guide this systematic scoping review of portfolio use in communication, ethics and professionalism training and assessment published in PubMed, Embase, PsycINFO, ERIC, Scopus, and Google Scholar databases between 1st January 2000 and 30th June 2020. The included articles are concurrently content and thematically analysed using the Split Approach. Overlapping categories and themes identified are combined using the Jigsaw Perspective. The themes/categories are compared with the summaries of the included articles in the Funnelling Process to ensure their accuracy. The domains identified form the framework for the discussion

Results

12,300 abstracts were reviewed, 946 full-text articles were evaluated, and 82 articles were analysed and the four domains identified were indications; content; design; and strengths and limitations.

Conclusions

This review reveals that when using a consistent framework, accepted endpoints and outcome measures, longitudinal multisource, multimodal assessment data fashions professional and personal development and enhances identity construction. Future studies into effective assessment tools and support mechanisms are required if portfolio use is to be maximised.

For peer review only

Strengths and limitations of this study

- The SEBA methodology adopts the structure of systematic reviews and flexibility of narrative reviews to synthesise a reproducible and accountable evaluation of diverse methodological sources, settings, physician populations and specialities.
- This review provides first insights into the impact of longitudinal development of communications, ethics and professionalism competencies and their impact on professional identity formation.
- Given that communications, ethics and professionalism are sociocultural constructs, geopolitical sociocultural differences may raise questions as to the applicability of these findings beyond the European and North American medical education systems.
- As the number of articles included is limited, the review's ability to assess the long-term effectiveness of portfolios may be compromised.

For peer review only

Abstract Word Count: 273 words

Manuscript Word Count: 1999 words

Number of Tables: 5

Number of Figures: 2

Number of References: 152

Keywords: Portfolios, Communication, Ethics, Professionalism, Professional Identity Formation, Medical Education, medical school, undergraduate, postgraduate training.

For peer review only - http://bmjopen.bmj.com/site/about/guidelines.xhtml

INTRODUCTION

Evidence for the effective embodiment of ethical and professional principles, communication skills and appropriate use of empathy in clinical practice requires a longitudinal, and often multi-source perspective. With ethics and professionalism sharing longitudinal developmental trajectories and intimately entwined with communication skills and competencies, the three competencies are increasingly considered together. In practice longitudinal CEP programs appear in the curricula of the Top 10 medical schools featured on the QS World University Rankings 2020 (1). Concurrent study of communication, ethics, and professionalism (henceforth CEP) is also underlined by their common sociocultural roots. As sociocultural constructs (2), CEP competencies are shaped by the individual's personal experiences, motivations, enthusiasm, idealism, abilities, competencies, virtues, expectations, knowledge, skills, emotions, and attitudes (henceforth narratives); their values, beliefs, and principles (henceforth belief systems)(3); their clinical experiences, competencies, training, insights, and confidence (henceforth clinical insights)(4, 5, 6, 7) and their practice, clinical, social , cultural, academic, research, and personal considerations (henceforth contextual considerations)(8, 9, 10, 11, 12).

However, whilst previous reviews into the teaching of ethics (13, 14, 15), communication (16, 17, 18, 19, 20, 21, 22) and professionalism (23, 24, 25) suggest the use of portfolios could provide a personalised, holistic, and longitudinal perspective of CEP skills, knowledge, and attitudes, and support of developing competencies we are aware of little progress in designing such platforms. Impetus for mapping current use of CEP portfolios also arises from the notion that developing CEP competencies shape how medical students and physicians (henceforth clinician) "think, act and feel like a physician" (26) or their professional identity formation (henceforth PIF) (27). It is posited that the promise of CEP portfolios with better appreciation of evolving self-concepts of professional identity will better direct support and even remediation of professional, ethical, communication and interprofessional development and PIF. This is especially pertinent at a time of increasing reports of breaches in standards, codes of conduct, and social and practice expectations (25).

METHODS

A Systematic Evidence-Based Approach (SEBA) guided Systematic Scoping Review (SSR) (henceforth SSR in SEBA) is proposed to map CEP portfolio use in medicine to guide the design, structuring and support of a proposed program (13, 14, 15, 16, 17, 20, 21, 22, 24, 25, 27, 28, 29, 30, 31, 32, 33, 34, 35, 36, 37, 38, 39, 40, 41, 42, 43, 44, 45, 46, 47, 48, 49, 50). Given space constraints, we briefly describe the six stages in the construction

For peer review only - http://bmjopen.bmj.com/site/about/guidelines.xhtml

of SSRs in SEBA here (Figure 1) and advance more details of the Systematic Approach, Split Approach, Jigsaw Perspective, Funnelling Process, Reiterative Process and Synthesis of SSR, in Appendix A (16, 27, 28, 35, 41, 48, 51, 52, 53, 54, 55, 56).

(Insert Figure 1. *The SEBA Process*)

Stage 1 of SEBA: Systematic Approach

A. Determining of Title and Background of the Review

An expert team comprised of a medical librarian from the Yong Loo Lin School of Medicine (YLLSoM) at the National University of Singapore (57), and local education experts and clinicians at the National Cancer Centre Singapore (NCCS), the Palliative Care Institute Liverpool, YLLSoM and Duke-NUS Medical School (henceforth the expert team) supported the research team in designing and overseeing the SEBA process.

For peer review only

B. Identification of Research Question

The research and expert team determined the primary research question: "*What is known about CEP portfolios?*". The secondary research questions were "*what role do CEP portfolios have in teaching and assessing CEP development?*".

C. Inclusion Criteria

A Population, Intervention, Comparison and Outcome (PICOs) format, outlined in Table 1, was used to guide the research process (58, 59).

Table 1. PICOS Inclusion and Exclusion Criteria

PICOS	Inclusion criteria	Exclusion criteria
**Population**	• Undergraduate and postgraduate medical students<br>• Qualified medical doctors, physician or resident; medical officer, registrar, house officer, attending, consultant	• Allied health specialties such as Pharmacy, Dietetics, Chiropractic, Midwifery, Podiatry, Speech Therapy, Occupational and Physiotherapy<br>• Non-medical specialties such as Clinical and Translational Science, Alternative and Traditional Medicine, Veterinary, Dentistry
**Intervention**	• portfolios in undergraduate and postgraduate medical education for teaching and assessment of Communication, Ethics and Professionalism	Other documentation methods or learning tools that are:<br>• Not longitudinal or single timepoint

For peer review only - http://bmjopen.bmj.com/site/about/guidelines.xhtml

PICOS	Inclusion criteria	Exclusion criteria
	Criteria of a portfolio:<br>• longitudinal (more than a single timepoint) assessment data<br>• candidate's personal engagement with portfolio content and associated learning<br>• Interventions meeting the above criteria were included regardless of whether they were referred to as portfolios<br><br>All types of portfolios were included in the study:<br>• For instance: electronic & non-electronic; formative & summative or combined; clinical & non-clinical<br>• Portfolios with input from students and/or residents and/or doctors, and/or input from faculty members and other individuals<br>• Portfolios with different structures: extent by which the structure has been prescribed and/or left to individual discretion	• Does not include personal intellectual engagement with the content and associated learning (for instance, curriculum vitae, logbooks and the use of personal digital assistants)
**Comparison/ Context**	NA	NA
**Outcome**	Papers that measured the following outcomes were included:<br>• Effectiveness of the use of portfolios to assess and teach Communication, Ethics and Professionalism<br>• Impact of the use of portfolios on medical students (both undergraduate and postgraduate)<br>• Impact of the use of portfolios on the faculty	NA
**Study design**	• Articles in English or translated to English<br>• Articles published from 1st January 2000 to 31st December 2020<br>• Databases: PsycINFO, EMBASE, PubMed, ERIC, Scopus, Google Scholar<br>• All study designs including:<br>• Mixed methods research, meta-analyses, systematic reviews, randomised controlled trials, cohort studies, case-control studies, cross-sectional studies, descriptive papers, grey literature, opinions, letters, commentaries and editorials	NA

D. Identification of Relevant Studies

The research team developed search strategies and carried out independent reviews for relevant publications in the PubMed, Embase, PsycINFO, ERIC, Scopus, and Google Scholar databases. Keeping with Pham, Rajić (60)'s approach of ensuring a feasible and sustainable research process, the team contained the search to articles

For peer review only - http://bmjopen.bmj.com/site/about/guidelines.xhtml

published between 1st January 2000 and 31st December 2020 to accommodate existing human resource and time constraints and ensure a sustainable review process.

E. Selection of Studies Included in Review

Six members of the research team created individual lists of titles to be included whilst three other members of the research team carried out hand searches and ancestry searches of seven leading journals in medical education (Academic Medicine, Medical Education, Medical Teacher, Advances Health Sciences Education, BMC Medical Education, Teaching and Learning in Medicine and Perspectives on Medical Education) accessed through the NUS library portal. These individual lists of titles were discussed online and Sandelowski and Barroso (61)'s "negotiated consensual validation" approach to "*articulate, defend, and persuade others of the "cogency" or "incisiveness" of their points of view or show their willingness to abandon views that are no longer tenable*" was applied to achieve consensus on the final list of titles to be scrutinised.

F. Assessing the Quality of Articles

Eight research team members individually appraised the quality of the quantitative and qualitative studies using the Medical Education Research Study Quality Instrument (MERSQI) (62) and Consolidated Criteria for Reporting Qualitative Studies (COREQ) (63).

Stage 2 of SEBA: Split Approach

Two teams carried independent and concurrent thematic and content analysis of the included articles whilst a third team created tabulated summaries based on recommendations drawn from Wong, Greenhalgh (64)'s RAMESES publication standards: meta-narrative reviews and Popay, Roberts (65)'s *"Guidance on the conduct of narrative synthesis in systematic reviews".* The categories employed in the content analysis for undergraduate communications were Rider et al. (66) 's *"A model for communication skills assessment across the undergraduate curriculum",* Goldie (67)'s *"Review of ethics curricula in undergraduate medical education"*, Duffy et al. (68)'s *"Assessing Competence in Communication and Interpersonal Skills: The Kalamazoo II Report"* and Hong et al. (13)'s *"Postgraduate Ethics Training Programs: A Systematic Scoping Review"*. Tay et al. (24)'s *"Assessing Professionalism in Medicine - A Scoping Review of Assessment Tools from 1990 to 2018"* was employed for codes

For peer review only - http://bmjopen.bmj.com/site/about/guidelines.xhtml

for professionalism and David (69)'s article *"AMEE Medical Education Guide No. 24: Portfolios as a method of student assessment"* was then used to contextualise their use in portfolios.

Stage 3 of SEBA: The Jigsaw Perspective

The Jigsaw Perspective sees the themes and categories identified compared and combined where overlaps and similarities exist.

Stage 4 of SEBA: The Funnelling Process

The Funnelling Process sees the themes/categories created from the Jigsaw Approach compared with the tabulated summaries to determine their consistency.

Patient and Public Involvement

Patients or the public were not involved in the design, or conduct, or reporting, or dissemination plans of our research.

RESULTS

A total of 12,300 abstracts were reviewed, 946 full-text articles were evaluated, and 82 articles were analysed (Figure 2). The funnelled domains identified are 1) indications, 2) portfolio content, 3) portfolio design and implementation and 4) strengths and limitations. In keeping with its goals of transparency and accountability, these Tables are enclosed in Appendix B.

(Insert Figure 2. PRISMA Flowchart)

Funneled Domain 1: Indications

CEP portfolios curate longitudinal multisource, multimodal assessment data taken at different settings and contexts allowing it to serve two main roles- teaching and assessment (70, 71) (Table 2). However few span undergraduate and postgraduate education (69, 72, 73), . As assessment tool CEP portfolios assist in the identification of areas of weakness and guide the provision of a comprehensive, targeted feedback, support and remediation (72, 74, 75). Most CEP assessment portfolios in postgraduate education focus on evidencing the attainment of required competencies (76, 77, 78, 79, 80), capturing reflections and indications of professional

For peer review only

identity formation (78, 81, 82, 83). CEP portfolios in the postgraduate setting have also been used for revalidation purposes (81)

As a teaching tool, CEP portfolios encourage self-directed learning (75, 84, 85, 86), self-improvements (71), reflective practice (72, 73, 75, 79, 81, 85, 86, 87, 88, 89, 90, 91, 92, 93, 94, 95), motivate practitioners to achieve desired competencies (90, 96) and bolster overall professional and personal development (72, 88, 96, 97).

Table 2. Benefits of CEP Teaching and Assessment Portfolios

CEP teaching portfolios	CEP assessment portfolios
**Enhancing learning**<br>Promotion of life-long learning (98, 99)<br><br>Promotion of self-directed learning<br>○ Formulation of learning plans (90, 96, 100)<br>○ Facilitates Outcome-oriented learning (89, 90, 101, 102)<br>○ Enhances self-evaluative capabilities (73, 76, 80, 85, 88, 89, 98, 99, 100, 103, 104, 105)<br>○ Identification of strengths and weaknesses (69, 71, 76, 78, 92, 96, 104, 106, 107, 108, 109)<br>○ Inculcates responsibility and ownership for one's learning (71, 73, 74, 80, 84, 86, 101, 110)<br>○ Sets the pace (99)<br>○ Personalised learning (108, 109)<br><br>**Personal and professional development**<br>Promotes self-understanding (79, 120, 121)<br>○ Understanding feelings and thoughts (100, 108)<br>○ Understanding of one's values (92)<br>○ Understanding activity/experience (69, 88, 122)<br>○ Understanding of how one functions in a team (116)<br>○ Fears and stressors feelings of inadequacy (122, 123)<br><br>Curates evidence of skills (98)<br>○ Skills to compile a portfolio (98, 111, 124, 125, 126)<br>○ Reflective skills (69, 76, 88, 102, 108, 122, 127)<br>○ Clinical skills (99)<br>Organizes information(102, 107)<br>○ Documentation of progress (77, 98, 104, 106)<br>○ Documentation of skills in different settings(71, 106, 109)<br>Cross-sectional view of one's competence (69, 77, 106, 109)	Balance of structure and freeform learning (69, 76, 92, 107, 111, 112)<br><br>Adaptable to goals (77, 113, 114)<br><br>Alignment of learning outcomes and assessment methods (90, 91, 97, 101, 105)<br><br>Allows personalised assessments (77)<br><br>Allows assessment over time (84, 92) and stages of development (69) and that facilitate evaluation of:<br>○ Learning and progress over time (77, 106)<br>○ Learning curve (121)<br>○ In different roles (92, 93)<br>○ In different settings (70, 113)<br>○ Complex behaviours (72, 74, 77, 84, 90, 99, 115, 116)<br>○ Critical thinking (77, 97, 105, 114)<br>○ Outcomes/competencies (107, 115)<br>○ Self-assessment (77, 105, 114)<br>○ Complex skills (113)<br>○ Professionalism (114)<br>○ Cultural competence (114)<br>○ Communication skills (114)<br>○ Clinical ethics (117)<br>○ Problem solving abilities (77, 114)<br>○ Reflective abilities (77, 90, 105, 109)<br><br>Longitudinal data<br>○ Possess good predictive value of subsequent clinical competency (74, 118)<br>○ Enhances validity of portfolio grading (69)<br>○ Guides better decisions (74, 116, 119)<br>○ Provides authentic assessment is a key principle of it (92, 99, 109)<br>○ Provides insights in the various core competencies (98)<br>○ Fair mode of assessment(99) and allows student to defend positions/work (69)<br>○ Provides students with direction and clarity (111, 112)

For peer review only - http://bmjopen.bmj.com/site/about/guidelines.xhtml

**Personalisation**<br>Allows for personalisation (69, 90, 99, 110, 114)<br>o Personal experiences (69)<br>Choice over the contents (69, 99, 114)	Allows the combination of Summative and Formative assessment (69, 91) and multisource assessments (81, 87, 97, 108) (109) and triangulation (77, 109)<br><br>Facilitates personalised feedback (116) that can be informed by understanding of the individual setting and context (99, 100, 113)
**Benefits to faculty**<br>Improvement of curriculum (83, 92)<br>o Identify areas of weakness in curriculum (83)<br>o Provide insights into mentoring, teaching etc (128)<br>Facilitates design of the program (83)<br><br>faculty development<br>o Understand more about how students acquire competencies (99)<br>o Insight into the impact of their interventions (119, 128)<br>Insight into their professional conduct, role modelling and pastoral care (121)	
**E-portfolios**<br>Ease of use:<br>o Easier use (90)<br>o Accessible (73)<br>Dissemination and editing of information (110)	

Funneled Domain 2: Characteristics

The contents of portfolios were shaped by their overall function (77, 80, 83, 113, 129) (Table 3). Expectations about the nature, and assessments of the contents (83) facilitates personalization of portfolios (73, 77, 104).

Table 3. Portfolio Design and Implementation

Themes	Subthemes	Contents
**Design and Implementation Principles**	**Structure**	Clear guidelines (69, 87, 88, 96, 97, 105, 108)<br>• Use of portfolio (69, 88)<br>• Portfolio content - determining competencies to assess (69, 91)<br>• Promotional decisions (69, 71, 89, 121, 130)<br>Standard-setting exercises (75)<br>Provision of sample portfolios for reference (105, 131, 132)<br>• Roles for assessors (69, 71, 74, 120, 121, 130, 133)<br>• Expectations of students (112)<br>• Duration spanning across preclinical and clinical years (126)<br>Well-defined structure with room for flexibility (69, 86, 88, 90, 105)<br>Student-centered (90)<br>Appropriate student to mentor ratio (71, 125)<br>Sufficient curricular experience for reflection, feedback (71)<br>Protected time for reflection, feedback, and regular meetings with mentors (71, 74, 87, 108)
	**Faculty**	Good faculty support (121, 126)<br>Ensure culture is supportive of portfolio system (71, 126)<br>Flexible and enthusiastic tutors passionate and experienced mentors (85, 88)

For peer review only - http://bmjopen.bmj.com/site/about/guidelines.xhtml

	**Curricular Integration**	Early introduction of portfolio (88, 108, 110, 134)<br>Easing portfolio into curriculum (69, 72, 86, 88, 90) – including orientation, training and introductory sessions (69, 72, 88, 90, 105, 121, 132) for learners and staff training (69, 71, 72, 75, 84, 85, 88)<br>Implementation of portfolios throughout curriculum (121)<br>Portfolio content aligning with phase of medical journey and competencies required (69, 71, 90, 97, 130, 132)
	**Assessments**	Clear scoring rubrics (69, 126)<br>Alignment between assessments and outcomes (86, 87, 90)<br>Transparent assessment criteria and outcomes to maintain fairness (71, 72, 110, 131)<br>Standardisation of portfolio use and content (86, 87), assessors training (72) and increased number of assessors (86, 87, 134) to improve assessment reliability (86, 87, 134)<br>Separate processes and reviewers for summative and formative portfolio (71) – summative assessments to specify clear pass/fail requirements (69)<br>Incorporation of student feedback into portfolio development (69, 72, 90)
	**Assessors**	External examiners (69)<br>Faculty members (69, 71, 74, 120, 130, 133)<br>Mentors (107)<br>Senior staff to be paired with new examiners (69)<br>Mentors should not assess own student (86, 92)
	**Assessment Frequency**	Formative assessments to monitor and guide student learning – conducted throughout the year (86, 88, 91)<br>Summative assessments to evaluate learner performance – conducted annually (69, 71, 72, 74, 75, 84, 85, 90, 91, 108, 130, 132, 135, 136, 137, 138), twice (71, 75, 116), thrice (88) or multiple times a year (77, 79)
	**Oversight and Delegation**	Establishment of portfolio oversight committee (71, 75)<br>Regular programme evaluation (71)<br>Clear division of support roles – e.g. pedagogical and technical support (110)

Postgraduate CEP assessment portfolios inject more details upon undergraduate portfolios. Table 4 summarises these elements include communication skills, addressing ethical issues and compliance of professional, institutional and departmental policies (106, 124, 139). Greater detail in postgraduate CEP assessment portfolios sees the addition of modified essay questions (MEQ)s (79), polypharmacy journal/audits (70, 95, 99, 129, 140), professional workshops and training sessions (98, 99, 113, 141, 142), health service meeting reports (79) and reviews by medical regulatory authorities (95). A detailed summary is provided in Appendix C.

Table 4. Portfolio Content (Competencies Assessed and Assessment Modalities)

	Communication	Ethics	Professionalism
Common competencies assessed	1. Values/ethics – appreciation of professional, legal and societal values to guide practice<br>2. Professional responsibilities- appreciation and manifesting these responsibilities		

For peer review only - http://bmjopen.bmj.com/site/about/guidelines.xhtml

	3. Doctor- patient relationships- appreciation of the guiding principles, expectations and ability to nurture these relationships<br>4. Interprofessional team working<br>5. Ethical and legal responsibilities<br>6. Continuous learning and quality improvement		
Specific competencies	1. Active listening<br>2. Clear explanations<br>3. Empathetic communication<br>4. History taking<br>5. Breaking bad news<br>6. Conflict management<br>7. Shared decision making<br>8. Written communication	1. Professionalism<br>2. Respect<br>3. Privacy<br>4. Identifying key ethical issues<br>5. Balancing competing ethical responsibilities<br>6. Consent	1. Interpersonal communication
Reflection	1. Content<br>2. Context<br>3. Depth<br>4. Authenticity<br>5. Developmental areas<br>6. Dilemma and doubts		
Assessment (formative/ summative/ mixed)	MCQ<br>Essays<br>OSCE<br>Mini-CEX<br>Case presentations<br>Case based discussion<br>Simulated patient assessment<br>Work based assessment<br>Team based assessment<br>Peer evaluation<br>Self-evaluation<br>Faculty evaluations<br>Feedback<br>Reflections<br>Letters of commendations<br>Learning and or improvement plans<br>Curriculum Vitae<br>Logbook review/interview		

Funneled Domain 3: Strengths and Limitations

The strengths and limitations of portfolios are outlined in Table 5.

Table 5. Strengths and Limitations

**Themes**	**Subthemes**	**Content**
**Strengths/ Limitations**	**Strengths**	For students<br>Identification of own strengths and weaknesses (69, 74, 75, 85, 86, 87, 88, 90, 92, 96, 100, 138)<br>Appreciation of effective communication (88, 134, 143), ethical issues (92), and fosters critical professional attitudes (87, 144)<br>Encourages self-regulation (138) through opportunities for reflection (86) and self-assessment (88, 108, 121)<br>Facilitates preparation for post-graduate medical training (88, 107, 111)<br><br>For faculty<br>Identification of areas of concern and allows for early, timely intervention (74, 75)

For peer review only - http://bmjopen.bmj.com/site/about/guidelines.xhtml

		Enhances interaction between students and teachers (69, 88, 92) Facilitates reiterative reassessment of teaching strategies (69)<br><br>Assessments<br>Longitudinal (116)<br>Good validity (74)<br>Reliable assessment tool – allows for triangulation of information and evidence from multiple sources and contexts (71)<br>Ability to assess a wide range of competencies (90, 97)<br>Allows for more authentic assessments (74)<br>Gives faculty greater insight into students' achievement of behaviourally-oriented competencies that traditional methods do not (75)<br>Evaluates achievement during clinical attachment/clerkship (97)<br>Provides a basis for the judgement of a student's professional fitness to practice (72)
	**Limitations**	Time-consuming and labour intensive (72, 75, 85, 88, 89, 90, 91, 96, 107, 126, 134)<br>Issues with reliability (86, 90, 120, 134)<br>Inconsistent effects (86, 90, 108, 145)<br>Lack of honesty by students in reflections (71, 72, 86, 90, 91, 107, 108, 112)

Postgraduate portfolios provide greater insights into lifelong learning and continuous professional development (77, 80, 81, 83, 98, 99, 106, 109, 113, 141). Use of multi-source assessments at multiple timepoints (80) in clinical settings (81, 99) see postgraduate portfolios provide good inter-rater reliability (77, 109). Postgraduate portfolios also detect stress and burnout (141).

Stage 5 of SEBA: Analysis of data and non-data driven literature

Findings from the different stages of SEBA were discussed with members of the expert team and relevant stakeholders. There were concerns from the expert team about the impact of grey literature on the narrative, given that they were neither peer-reviewed nor necessarily evidence-based. As a result, the research team differentiated correspondence, letters, editorials and perspective pieces from academic databases and grey literature from data-driven or research-based peer-reviewed data. Both groups were then independently analysed. The themes/categories identified were then compared to enhance further the accountability and the reproducibility of Stage 5 of SEBA. Evidence-based data from bibliographic databases (henceforth evidence-based publications) were separated from grey literature, perspectives, editorials, letters, and non-data-based articles drawn from bibliographic databases (henceforth non-data driven). These two groups were separately thematically analysed.

Stage 6 of SEBA: Synthesis of the Discussion

The Best Evidence Medical Education (BEME) Collaboration Guide (146) and the Structured approach to the Reporting In healthcare education of Evidence Synthesis (STORIES) (147) were used to guide the discussion.

For peer review only - http://bmjopen.bmj.com/site/about/guidelines.xhtml

DISCUSSION

This SSR in SEBA suggest that overlaps in ethics, professionalism, and communication skills, knowledge, attitudes and competencies reaffirm the concurrent training, and assessments of CEP competencies in portfolios (72, 74, 75). To be effective CEP teaching and longitudinal assessment portfolios require a consistent framework replete with clearly delineated goals (105), aligned expectations, predetermined assessment criteria for specified competencies (69, 71, 72, 77, 83, 85, 87, 96, 97, 98, 105, 110, 120, 121, 124, 132, 133, 139), agreed upon endpoints and outcome measures, and the curation, structuring and organisation of longitudinal multisource, multimodal assessment data (70, 71). The combination of this data facilitate training, assessment, feedback and holistic and longitudinal support (75, 84, 85, 86) (101) (106, 124, 139, 148), guide reflection (72, 73, 75, 79, 81, 85, 86, 87, 88, 89, 90, 91, 92, 93, 94, 95), and bolster professional and personal development (72, 88, 96, 97). Critically the employ of consistent and agreed upon structure, goals, purpose, role, learning objectives, content, and assessment guidelines also affords users with a personalised perspective of their CEP development upon which they can reflect on and glean insights and feedback (86, 102, 131).

Yet to be effective, CEP portfolios require policing and underscores the need for trained (70) and dedicated faculty (88) capable of effective role modelling (121), support (69, 79, 88, 92, 97) and providing feedback (116) and who appreciate the individual's current concepts, narratives, motivations, abilities and availabilities and contextual considerations. Furthermore, the efficacy of portfolios and their ability to meet the stated objectives stated in Table 2 and realise their stated benefits in Table 5 are reliant upon the appropriate mix of assessment methods employed, the frequency of assessments, the efficacy of analysis and the quality of the feedback, support and remediation provided should it be required. This in turn highlights the role of the host organization in ensuring effective oversight of the program and assessments and in ensuring that faculty are afforded protected time for administrative duties, coordination and assessments particularly given the longitudinal nature of CEP portfolios (77, 90, 105, 106, 122, 140, 149, 150). It should not be forgotten that CEP portfolios reflect the development of an individual and such change should be measured and contextualised within the wider education, training, practice, professional, research, clinical, interprofessional team based and organizational perspective. This further underlines the importance of the host organizations role in assessing and guiding development of CEP competencies.

For peer review only - http://bmjopen.bmj.com/site/about/guidelines.xhtml

With these requisites met, evidence of changes in CEP knowledge, skills and competencies suggests shift the thinking, attitudes and conduct of clinicians. The Krishna-Pisupati model posits that sustained changes in practice, beliefs, values and principles will result in changes in the clinician's belief systems which in turn inform their self-concepts of identity and shape their PIF.

LIMITATIONS

The generalisability of the results of this study is limited by the identified studies selected for review. Firstly, the majority of the papers included originate from Europe and North America. Given that CEP is a sociocultural construct, these geopolitical sociocultural differences raise questions as to the applicability of these findings beyond the European and North American medical education systems. Secondly, as the number of articles included is limited, the SSR in SEBA's ability to assess the long-term effectiveness of portfolios may be compromised.

CONCLUSIONS

The suggestion that CEP portfolios can capture, instruct, and assess PIF requires further study. Whilst there have been posits on how such evaluations could be made, such tools remain unrealised and must be the focus of coming studies. One possible starting point for the design of such tools may be theories such as the Krishna-Pisupati model which attempts to link PIF to changes to belief systems and concepts of personhood. This may provide a grander view of PIF that spans the undergraduate and postgraduate settings and provide the basis for directing support and feedback for clinicians. As we look forward to furthering efforts to study the effects of CEP portfolios upon the PIF of clinicians, we also believe the specific impact of these potentially resource-heavy and financially costly intervention should be at the centre of future initiatives.

ABBREVIATIONS

**CEP**	Communication, Ethics, Professionalism
**PIF**	Professional Identity Formation
**SSR**	Systematic Scoping Review
**SEBA**	Systematic Evidence Based Approach
**PICOS**	Population, Intervention, Comparison and Outcome
**BEME**	Best Evidence Medical Education

For peer review only - http://bmjopen.bmj.com/site/about/guidelines.xhtml

STORIES Structured approach to the Reporting In healthcare education of Evidence Synthesis

STATEMENTS AND DECLARATIONS

Ethics Approval and Consent to Participate NA

Consent for Publication NA

Availability of Data and Materials All data generated or analysed during this review are included in this published article and its appendices.

Competing Interests JJQT, GP, DZH, BKYL, AJSL, EJXC, AP, RT, JYHY, YZK, CWNQ, JYL, KTT, YTO, MC, JZ, SM, LW and LKRK have no competing interests.

Funding No funding was received for this review

Author Contributions JJQT, GP, DZH, BKYL, AJSL, EJXC, AP, RT, JYHY, YZK, CWNQ, JYL, KTT, YTO, MC, JZ, SM, LW and LKRK were involved in data curation, formal analysis, investigation, preparing the original draft of the manuscript as well as reviewing and editing the manuscript. All authors have read and approved the manuscript.

ACKNOWLEDGEMENTS

The authors would like to dedicate this paper to the late Dr. S Radha Krishna and A/Prof Cynthia Goh whose advice and ideas were integral to the success of this review and to Thondy, Maia Olivia and Raja Kamarul Ariffin whose lives continue to inspire us. The authors would also like to thank the anonymous reviewers, Dr Ruaraidh Hill and Dr Stephen Mason for their help comments which greatly enhanced this manuscript.

For peer review only - http://bmjopen.bmj.com/site/about/guidelines.xhtml

For peer review only

For peer review only - http://bmjopen.bmj.com/site/about/guidelines.xhtml

REFERENCES

1. Rankings TU. QS World University Rankings 2020 [Available from: https://www.topuniversities.com/university-rankings/university-subject-rankings/2020/medicine.
2. Deshpande AD. The Use of Sociocultural Constructs in Cancer Screening Research Among African Americans. Cancer Control. 2009;16(3):10.
3. MZJ H. Chinese Familial Tradition and Western Influences: A Case Study in Singapore on Decision Making at the end of life. Journal of Pain and Symptom Management. 2010;40(6):6.
4. Foo WT, Zheng Y, Kwee AK, Yang GM, Krishna L. Factors considered in end-of-life care decision making by health care professionals. Am J Hosp Palliat Care. 2013;30(4):354-8.
5. Chong JA, Quah YL, Yang GM, Menon S, Radha Krishna LK. Patient and family involvement in decision making for management of cancer patients at a centre in Singapore. BMJ Support Palliat Care. 2015;5(4):420-6.
6. Chai HZ, Krishna LK, Wong VH. Feeding: what it means to patients and caregivers and how these views influence Singaporean Chinese caregivers' decisions to continue feeding at the end of life. Am J Hosp Palliat Care. 2014;31(2):166-71.
7. Marc Ho ZJ, Krishna LKR, Goh C, Alethea Yee CP. The physician–patient relationship in treatment decision making at the end of life: A pilot study of cancer patients in a Southeast Asian society. Palliative and Supportive Care. 2013;11(1):13-9.
8. Krishna LKR. Decision making at the end of life: A Singaporean Perspective. Asian Bioeth Rev. 2011;3(2):6.
9. Loh AZ, Tan JS, Jinxuan T, Lyn TY, Krishna LK, Goh CR. Place of Care at End of Life: What Factors Are Associated With Patients' and Their Family Members' Preferences? Am J Hosp Palliat Care. 2016;33(7):669-77.
10. Krishna LK, Watkinson DS, Beng NL. Limits to relational autonomy--the Singaporean experience. Nurs Ethics. 2015;22(3):331-40.
11. Krishna LK, Alsuwaigh R, Miti PT, Wei SS, Ling KH, Manoharan D. The influence of the family in conceptions of personhood in the palliative care setting in Singapore and its influence upon decision making. Am J Hosp Palliat Care. 2014;31(6):645-54.
12. Krishna L. The position of the family of palliative care patients within the decision-making process at the end of life in Singapore. Ethics & Medicine: An International Journal of Bioethics. 2011;27:183+.
13. Hong DZ, Goh JL, Ong ZY, Ting JJQ, Wong MK, Wu J, et al. Postgraduate ethics training programs: a systematic scoping review. BMC Med Educ. 2021;21(1):338.
14. Wong MK, Hong DZH, Wu J, Ting JJQ, Goh JL, Ong ZY, et al. A systematic scoping review of undergraduate medical ethics education programs from 1990 to 2020. Med Teach. 2022;44(2):167-86.
15. Yoon NYS, Ong YT, Yap HW, Tay KT, Lim EG, Cheong CWS, et al. Evaluating assessment tools of the quality of clinical ethics consultations: a systematic scoping review from 1992 to 2019. BMC Med Ethics. 2020;21(1):51.
16. Bok C, Ng CH, Koh JWH, Ong ZH, Ghazali HZB, Tan LHE, et al. Interprofessional communication (IPC) for medical students: a scoping review. BMC Med Educ. 2020;20(1):372.
17. Chia E, Huang H, Goh S, Peries M, Lee C, Tan L, et al. A systematic scoping review of teaching and evaluating communications in the intensive care unit. The Asia Pacific Scholar. 2021;6:3-29.

For peer review only - http://bmjopen.bmj.com/site/about/guidelines.xhtml

18. Malhotra C, Kanesvaran R, Krishna L, Xiang L, Kumarakulasinghe NB, Tan SH, et al. Oncologists' responses to patient and caregiver negative emotions and patient perception of quality of communication: results from a multi-ethnic Asian setting. Support Care Cancer. 2018;26(3):957-65.
19. Ng CH, Ong ZH, Koh JWH, Ang RZE, Tan LHS, Tay KT, et al. Enhancing Interprofessional Communications Training in Internal Medicine. Lessons Drawn From a Systematic Scoping Review From 2000 to 2018. J Contin Educ Health Prof. 2020;40(1):27-35.
20. Ong ZH, Tan LHE, Ghazali HZB, Ong YT, Koh JWH, Ang RZE, et al. A Systematic Scoping Review on Pedagogical Strategies of Interprofessional Communication for Physicians in Emergency Medicine. J Med Educ Curric Dev. 2021;8:23821205211041794.
21. Tan XH, Foo MA, Lim SLH, Lim M, Chin AMC, Zhou J, et al. Teaching and assessing communication skills in the postgraduate medical setting: a systematic scoping review. BMC Med Educ. 2021;21(1):483.
22. Venktaramana V, Loh EKY, Wong CJW, Yeo JW, Teo AYT, Chiam CSY, et al. A systematic scoping review of communication skills training in medical schools between 2000 and 2020. Med Teach. 2022;44(9):997-1006.
23. Ong YT, Kow CS, Teo YH, Tan LHE, Abdurrahman A, Quek NWS, et al. Nurturing professionalism in medical schools. A systematic scoping review of training curricula between 1990-2019. Med Teach. 2020;42(6):636-49.
24. Tay KT, Ng S, Hee JM, Chia EWY, Vythilingam D, Ong YT, et al. Assessing Professionalism in Medicine - A Scoping Review of Assessment Tools from 1990 to 2018. J Med Educ Curric Dev. 2020;7:2382120520955159.
25. Teo YH, Peh TY, Abdurrahman A, Lee ASI, Chiam M, Fong W, et al. A modified Delphi approach to enhance nurturing of professionalism in postgraduate medical education in Singapore. Singapore Med J. 2021.
26. The Student-Physician: Introductory Studies in the Sociology of Medical Education. Journal of the American Medical Association. 1958;166(1):106-7.
27. Teo KJH, Teo MYK, Pisupati A, Ong RSR, Goh CK, Seah CHX, et al. Assessing professional identity formation (PIF) amongst medical students in Oncology and Palliative Medicine postings: a SEBA guided scoping review. BMC Palliat Care. 2022;21(1):200.
28. Quah ELY, Chua KZY, Lua JK, Wan DWJ, Chong CS, Lim YX, et al. A Systematic Review of Stakeholder Perspectives of Dignity and Assisted Dying. J Pain Symptom Manage. 2022.
29. Venktaramana V. Understanding Mentoring Relationships between Mentees, Peer and Senior Mentors. BMC Med Educ. 2023.
30. Toh RQE, Koh KK, Lua JK, Wong RSM, Quah ELY, Panda A, et al. The role of mentoring, supervision, coaching, teaching and instruction on professional identity formation: a systematic scoping review. BMC Med Educ. 2022;22(1):531.
31. Ho CY, Kow CS, Chia CHJ, Low JY, Lai YHM, Lauw SK, et al. The impact of death and dying on the personhood of medical students: a systematic scoping review. BMC Med Educ. 2020;20(1):516.
32. Sarraf-Yazdi S, Teo YN, How AEH, Teo YH, Goh S, Kow CS, et al. A Scoping Review of Professional Identity Formation in Undergraduate Medical Education. J Gen Intern Med. 2021;36(11):3511-21.
33. Vig PS, Lim JY, Lee RWL, Huang H, Tan XH, Lim WQ, et al. Parental bereavement - impact of death of neonates and children under 12 years on personhood of parents: a systematic scoping review. BMC Palliat Care. 2021;20(1):136.

For peer review only - http://bmjopen.bmj.com/site/about/guidelines.xhtml

34. Chan NPX, Chia JL, Ho CY, Ngiam LXL, Kuek JTY, Ahmad Kamal NHB, et al. Extending the Ring Theory of Personhood to the Care of Dying Patients in Intensive Care Units. Asian Bioeth Rev. 2022;14(1):71-86.
35. Ngiam LXL, Ong YT, Ng JX, Kuek JTY, Chia JL, Chan NPX, et al. Impact of Caring for Terminally Ill Children on Physicians: A Systematic Scoping Review. Am J Hosp Palliat Care. 2021;38(4):396-418.
36. Chia EWY, Tay KT, Xiao S, Teo YH, Ong YT, Chiam M, et al. The Pivotal Role of Host Organizations in Enhancing Mentoring in Internal Medicine: A Scoping Review. J Med Educ Curric Dev. 2020;7:2382120520956647.
37. Kuek JTY, Ngiam LXL, Kamal NHA, Chia JL, Chan NPX, Abdurrahman A, et al. The impact of caring for dying patients in intensive care units on a physician's personhood: a systematic scoping review. Philos Ethics Humanit Med. 2020;15(1):12.
38. Zhou YC, Tan SR, Tan CGH, Ng MSP, Lim KH, Tan LHE, et al. A systematic scoping review of approaches to teaching and assessing empathy in medicine. BMC Med Educ. 2021;21(1):292.
39. Tay KT, Tan XH, Tan LHE, Vythilingam D, Chin AMC, Loh V, et al. A systematic scoping review and thematic analysis of interprofessional mentoring in medicine from 2000 to 2019. J Interprof Care. 2021;35(6):927-39.
40. Tan R, Qi Ting JJ, Zhihao Hong D, Sing Lim AJ, Ong YT, Pisupati A, et al. Medical Student Portfolios: A Systematic Scoping Review. J Med Educ Curric Dev. 2022;9:23821205221076022.
41. Kow CS, Teo YH, Teo YN, Chua KZY, Quah ELY, Kamal N, et al. A systematic scoping review of ethical issues in mentoring in medical schools. BMC Med Educ. 2020;20(1):246.
42. Huang H, Toh RQE, Chiang CLL, Thenpandiyan AA, Vig PS, Lee RWL, et al. Impact of Dying Neonates on Doctors' and Nurses' Personhood: A Systematic Scoping Review. J Pain Symptom Manage. 2022;63(1):e59-e74.
43. Hong DZ, Lim AJS, Tan R, Ong YT, Pisupati A, Chong EJX, et al. A Systematic Scoping Review on Portfolios of Medical Educators. J Med Educ Curric Dev. 2021;8:23821205211000356.
44. Ho S, Tan YY, Neo SHS, Zhuang Q, Chiam M, Zhou JX, et al. COVID-19 - A Review of the Impact it has made on Supportive and Palliative Care Services Within a Tertiary Hospital and Cancer Centre in Singapore. Ann Acad Med Singap. 2020;49(7):489-95.
45. Hee J, Toh YL, Yap HW, Toh YP, Kanesvaran R, Mason S, et al. The development and design of a framework to match mentees and mentors through a systematic review and thematic analysis of mentoring programs between 2000 and 2015. Mentoring & Tutoring: Partnership in Learning. 2020;28:340-64.
46. Chua WJ, Cheong CWS, Lee FQH, Koh EYH, Toh YP, Mason S, et al. Structuring Mentoring in Medicine and Surgery. A Systematic Scoping Review of Mentoring Programs Between 2000 and 2019. J Contin Educ Health Prof. 2020;40(3):158-68.
47. Chua KZY, Quah ELY, Lim YX, Goh CK, Lim J, Wan DWJ, et al. A systematic scoping review on patients' perceptions of dignity. BMC Palliat Care. 2022;21(1):118.
48. Quek CWN, Ong RRS, Wong RSM, Chan SWK, Chok AK-L, Shen GS, et al. Systematic scoping review on moral distress among physicians. BMJ Open. 2022;12(9):e064029.
49. Ong RSR, Wong RSM, Chee RCH, Quek CWN, Burla N, Loh CYL, et al. A systematic scoping review moral distress amongst medical students. BMC Med Educ. 2022;22(1):466.
50. Yang LH, Kleinman A, Link BG, Phelan JC, Lee S, Good B. Culture and stigma: adding moral experience to stigma theory. Soc Sci Med. 2007;64(7):1524-35.

For peer review only - http://bmjopen.bmj.com/site/about/guidelines.xhtml

51. Krishna LKR, Tan LHE, Ong YT, Tay KT, Hee JM, Chiam M, et al. Enhancing Mentoring in Palliative Care: An Evidence Based Mentoring Framework. Journal of Medical Education and Curricular Development. 2020;7:2382120520957649.
52. Ong YT, Quek CWN, Pisupati A, Loh EKY, Venktaramana V, Chiam M, et al. Mentoring future mentors in undergraduate medical education. PLoS One. 2022;17(9):e0273358.
53. Lim JY, Ong SYK, Ng CYH, Chan KLE, Wu S, So WZ, et al. A systematic scoping review of reflective writing in medical education. BMC Med Educ. 2023;23(1):12.
54. Kamal NHA, Tan LHE, Wong RSM, Ong RRS, Seow REW, Loh EKY, et al. Enhancing education in Palliative Medicine: the role of Systematic Scoping Reviews. Palliative Medicine & Care: Open Access. 2020;7(1):1-11.
55. Ong RRS, Seow REW, Wong RSM, Loh EKY, Kamal NHA, Mah ZH, et al. A Systematic Scoping Review of Narrative Reviews in Palliative Medicine Education. Palliative Medicine & Care: Open Access. 2020;7(1):1-22.
56. Mah ZH, Wong RSM, Seow REW, Loh EKY, Kamal NHA, Ong RRS, et al. A Systematic Scoping Review of Systematic Reviews in Palliative Medicine Education. Palliative Medicine & Care: Open Access. 2020;7(1):1-12.
57. Cleland J, McKimm J, Fuller R, Taylor D, Janczukowicz J, Gibbs T. Adapting to the impact of COVID-19: Sharing stories, sharing practice. Medical Teacher. 2020;42(7):772-5.
58. Peters M, Godfrey C, McInerney P, Soares C, Khalil H, Parker D. The Joanna Briggs Institute reviewers' manual 2015: methodology for JBI scoping reviews2015 April 29, 2019. Available from: http://joannabriggs.org/assets/docs/sumari/Reviewers-Manual_Methodology-for-JBI-Scoping-Reviews_2015_v1.pdf.
59. Peters MD, Godfrey CM, Khalil H, McInerney P, Parker D, Soares CB. Guidance for conducting systematic scoping reviews. Int J Evid Based Healthc. 2015;13(3):141-6.
60. Pham MT, Rajić A, Greig JD, Sargeant JM, Papadopoulos A, McEwen SA. A scoping review of scoping reviews: advancing the approach and enhancing the consistency. Res Synth Methods. 2014;5(4):371-85.
61. Sandelowski M, Barroso J. Handbook for synthesizing qualitative research: springer publishing company; 2006.
62. Reed DA, Beckman TJ, Wright SM, Levine RB, Kern DE, Cook DA. Predictive validity evidence for medical education research study quality instrument scores: quality of submissions to JGIM's Medical Education Special Issue. J Gen Intern Med. 2008;23(7):903-7.
63. Tong A, Sainsbury P, Craig J. Consolidated criteria for reporting qualitative research (COREQ): a 32-item checklist for interviews and focus groups. Int J Qual Health Care. 2007;19(6):349-57.
64. Wong G, Greenhalgh T, Westhorp G, Buckingham J, Pawson R. RAMESES publication standards: meta-narrative reviews. BMC medicine. 2013;11(1):20.
65. Popay J, Roberts H, Sowden A, Petticrew M, Arai L, Rodgers M, et al. Guidance on the conduct of narrative synthesis in systematic reviews. A product from the ESRC methods programme Version. 2006;1:b92.
66. Rider EA, Hinrichs MM, Lown BA. A model for communication skills assessment across the undergraduate curriculum. Med Teach. 2006;28(5):e127-34.
67. Goldie J. Review of ethics curricula in undergraduate medical education. Med Educ. 2000;34(2):108-19.
68. Duffy FD, Gordon GH, Whelan G, Cole-Kelly K, Frankel R, Buffone N, et al. Assessing competence in communication and interpersonal skills: the Kalamazoo II report. Acad Med. 2004;79(6):495-507.

For peer review only

69. David M, Davis M, Harden R, Howie P, Ker J, Pippard M. AMEE Medical Education Guide No. 24: Portfolios as a method of student assessment. Medical teacher. 2001;23:535-51.
70. McEwen L, Griffiths J, Schultz K. Developing and Successfully Implementing a Competency-Based Portfolio Assessment System in a Postgraduate Family Medicine Residency Program. Academic medicine : journal of the Association of American Medical Colleges. 2015;90.
71. Dannefer E, Henson L. The Portfolio Approach to Competency-Based Assessment at the Cleveland Clinic Lerner College of Medicine. Academic medicine : journal of the Association of American Medical Colleges. 2007;82:493-502.
72. Davis M, Ben-David M, Harden R, Howie P, Ker J, McGhee C, et al. Portfolio assessment in medical students' final examination. Medical teacher. 2001;23:357-66.
73. Ruiz J, Qadri S, Karides M, Castillo C, Milanez M, Roos B. Fellows' Perceptions of a Mandatory Reflective Electronic Portfolio in a Geriatric Medicine Fellowship Program. Educational Gerontology - EDUC GERONTOL. 2009;35:634-52.
74. O'Brien C, Thomas J, Green M. What Is the Relationship Between a Preclerkship Portfolio Review and Later Performance in Clerkships? Academic medicine : journal of the Association of American Medical Colleges. 2017;93.
75. O'Brien C, Sanguino S, Thomas J, Green M. Feasibility and Outcomes of Implementing a Portfolio Assessment System Alongside a Traditional Grading System. Academic medicine : journal of the Association of American Medical Colleges. 2016;91.
76. Jenkins L, Mash B, Derese A. The national portfolio of learning for postgraduate family medicine training in South Africa: Experiences of registrars and supervisors in clinical practice. BMC medical education. 2013;13:149.
77. Shumway J, Harden R. AMEE Guide No. 25: The assessment of learning outcomes for the competent and reflective physician. Medical teacher. 2003;25:569-84.
78. Daboval T, Moore G, Ferretti E. How we teach ethics and communication during a Canadian neonatal perinatal medicine residency: An interactive experience. Medical teacher. 2012;35.
79. Jenkins L, Mash B, Derese A. Development of a portfolio of learning for postgraduate family medicine training in South Africa: A Delphi study. BMC family practice. 2012;13:11.
80. Colbert C, Ownby A, Butler P. A Review of Portfolio Use in Residency Programs and Considerations before Implementation. Teaching and learning in medicine. 2008;20:340-5.
81. Wakeling J, Holmes S, Boyd A, Tredinnick-Rowe J, Cameron N, Marshall M, et al. Reflective Practice for Patient Benefit: An Analysis of Doctors' Appraisal Portfolios in Scotland. Journal of Continuing Education in the Health Professions. 2019;39:1.
82. Torbeck L. A Method for Defining Competency-Based Promotion Criteria for Family Medicine Residents. Academic medicine : journal of the Association of American Medical Colleges. 2005;80:832-9.
83. O'Sullivan P, Reckase M, McClain T, Savidge M, Clardy J. Demonstration of Portfolios to Assess Competency of Residents. Advances in health sciences education : theory and practice. 2004;9:309-23.
84. Yielder J, Moir F. Assessing the Development of Medical Students' Personal and Professional Skills by Portfolio. Journal of Medical Education and Curricular Development. 2016;2016:9.
85. O'Sullivan A, Howe A, Miles S, Harris P, Hughes C, Jones P, et al. Does a summative portfolio foster the development of capabilities such as reflective practice and

For peer review only - http://bmjopen.bmj.com/site/about/guidelines.xhtml

understanding ethics? An evaluation from two medical schools. Medical teacher. 2012;34:e21-8.
86. Driessen E, Tartwijk J, Vermunt J, Van der Vleuten C. Use of Portfolios in early undergraduate medical training. Medical teacher. 2003;25:18-23.
87. Haffling A-C, Beckman A, Pahlmblad A, Edgren G. Students' reflections in a portfolio pilot: Highlighting professional issues. Medical teacher. 2010;32:e532-40.
88. Fida N, Hassanien M, Shamim M, Alafari R, Zaini R, Mufti S, et al. Students' perception of portfolio as a learning tool at King Abdulaziz University Medical School. Medical Teacher. 2018;40:1-10.
89. Goldie J, Dowie A, Cotton P, Morrison J. Teaching professionalism in the early years of a medical curriculum: A qualitative study. Medical education. 2007;41:610-7.
90. O'Sullivan A, Harris P, Hughes C, Toohey S, Balasooriya C, Velan G, et al. Linking assessment to undergraduate student capabilities through portfolio examination. Assessment & Evaluation in Higher Education. 2012;37:379-91.
91. Franco R, Franco C, Pestana O, Severo M, Ferreira M. The use of portfolios to foster professionalism: attributes, outcomes, and recommendations. Assessment & Evaluation in Higher Education. 2016;42:1-19.
92. Gordon J. Assessing students' personal and professional development using portfolios and interviews. Medical education. 2003;37:335-40.
93. Wong A, Trollope-Kumar K. Reflections: An inquiry into medical students' professional identity formation. Medical education. 2014;48:489-501.
94. Borgstrom E, Cohn S, Barclay S. Medical Professionalism: Conflicting Values for Tomorrow's Doctors. Journal of general internal medicine. 2010;25:1330-6.
95. Lockyer J, Sockalingam S, Campbell DC. Assessment and Change: An Exploration of Documented Assessment Activities and Outcomes by Canadian Psychiatrists. Journal of Continuing Education in the Health Professions. 2018;38:1.
96. Sambandam E, Jutti R, Lee L. Portfolio as a learning tool: Students' perspective. Annals of the Academy of Medicine, Singapore. 2005;34:511-4.
97. Michels N, Avonts M, Peeraer G, Ulenaers K, Gaal L, Bossaert L, et al. Content validity of workplace-based portfolios: A multi-centre study. Medical Teacher. 2016;38:1-10.
98. Finlay K, Probyn L, Ho S. The CanMEDS Resume: A Useful Educational Portfolio Tool for Diagnostic Radiology Residents. Canadian Association of Radiologists journal = Journal l'Association canadienne des radiologistes. 2011;63:233-6.
99. Clay A, Petrusa E, Harker M, Andolsek K. Development of a web-based, specialty specific portfolio. Medical teacher. 2007;29:311-6.
100. Ozcakar N, Mevsim V, Dilek G. Use of Portfolios in Undergraduate Medical Training: First Meeting With a Patient. Trakya Universitesi Tip Fakultesi Dergisi. 2009;26.
101. Bhattarai M. Study skills course in medical education for postgraduate residents. Kathmandu University medical journal (KUMJ). 2006;5:561-5.
102. Amsellem-Ouazana D, Pee D, Godin V. Use of portfolios as a learning and assessment tool in a surgical practical session of urology during undergraduate medical training. Medical teacher. 2006;28:356-9.
103. Watling C, Brown J. Education Research: Communication skills for neurology residents: Structured teaching and reflective practice. Neurology. 2007;69:E20-6.
104. Goliath C. Diffusion of an E-Portfolio to Assist in the Self-Directed Learning of Physicians: An Exploratory Study. 2009.

For peer review only - http://bmjopen.bmj.com/site/about/guidelines.xhtml

105. Azer S. Use of Portfolios by Medical Students: Significance of Critical Thinking. The Kaohsiung journal of medical sciences. 2008;24:361-6.
106. Cheung R. ECLIPPx: An innovative model for reflective portfolios in life-long learning. The clinical teacher. 2011;8:27-30.
107. Rees C, Shepherd M, Chamberlain S. The utility of reflective portfolios as a method of assessing first year medical students' personal and professional development. Reflective Practice. 2005;6:3-14.
108. Driessen E, Tartwijk J, Overeem K, Vermunt J, Van der Vleuten C. Conditions for reflective use of portfolios in undergraduate medical education. Medical education. 2006;39:1230-5.
109. Gavriel J. Portfolios and 'the competent clinician': Past, present and future assessment. Education for Primary Care. 2016;27:1-4.
110. Moores A, Parks M. Twelve tips for introducing E-Portfolios with undergraduate students. Medical teacher. 2010;32:46-9.
111. Rees C, Sheard C. Undergraduate medical students' views about a reflective portfolio assessment of their communication skills. Medical education. 2004;38:125-8.
112. Chaffey L, de Leeuw E, Finnigan G. Facilitating Students' Reflective Practice in a Medical Course: Literature Review. Education for health (Abingdon, England). 2011;25:198-203.
113. Pinsky L, Edwards K. Diving for PERLS: Working and performance portfolios for evaluation and reflection on learning. Journal of general internal medicine. 2004;19:582-7.
114. Gibson C, Chandratilake M, Hull A. Use of portfolios for assessment of global health residents: qualitative evaluation of design and implementation. Canadian medical education journal. 2018;9:e20-e32.
115. Roberts C, Shadbolt N, Clark T, Simpson P. The reliability and validity of a portfolio designed as a programmatic assessment of performance in an integrated clinical placement. BMC Medical Education. 2014;14:197.
116. Dolan B, O'Brien C, Cameron K, Green M. A Qualitative Analysis of Narrative Preclerkship Assessment Data to Evaluate Teamwork Skills. Teaching and Learning in Medicine. 2018;30:1-9.
117. Fins J, Kodish E, Cohn F, Danis M, Derse A, Dubler N, et al. A Pilot Evaluation of Portfolios for Quality Attestation of Clinical Ethics Consultants. The American journal of bioethics : AJOB. 2016;16:15-24.
118. Naidu T, Ramlall S. Establishing psychiatric registrars' competence in psychotherapy: A portfolio based model. African journal of psychiatry. 2008;11:264-71.
119. Murphy D, Aitchison P, Hernandez Santiago V, Davey P, Mires G, Nathwani D. Insightful Practice: A robust measure of medical students' professional response to feedback on their performance. BMC Medical Education. 2015;15.
120. Ekayanti F, Risahmawati R, Fadhilah M. Portfolio Assessment Implementation in Clinical Year of Community Medicine Module: Students Perspective2017.
121. Hall P, Byszewski A, Sutherland S, Stodel E. Developing a Sustainable Electronic Portfolio (ePortfolio) Program That Fosters Reflective Practice and Incorporates CanMEDS Competencies Into the Undergraduate Medical Curriculum. Academic medicine : journal of the Association of American Medical Colleges. 2012;87:744-51.
122. Menon V. Portfolios for post graduate psychiatry residents: Potentials and pitfalls. Asian Journal of Psychiatry. 2014;11.

For peer review only

123. Prayson R, Bierer SB, Dannefer E. Medical student resilience strategies: A content analysis of medical students' portfolios. Perspectives on medical education. 2016;6.
124. Fragneto R, Dilorenzo A, Schell R, Bowe E. Evaluating Practice-Based Learning and Improvement: Efforts to Improve Acceptance of Portfolios. Journal of graduate medical education. 2010;2:638-43.
125. Austin C, Braidman I. Support for portfolio in the initial years of the undergraduate medical school curriculum: What do the tutors think? Medical teacher. 2008;30:265-71.
126. Arntfield S, Parlett B, Meston C, Apramian T, Lingard L. A model of engagement in reflective writing-based portfolios: Interactions between points of vulnerability and acts of adaptability. Medical teacher. 2015;38:1-10.
127. Pitkälä K, Mäntyranta T. Feelings related to first patient experiences in medical school: A qualitative study on students' personal portfolios. Patient education and counseling. 2004;54:171-7.
128. Tigelaar D, Dolmans D, Grave W, Wolfhagen I, Van der Vleuten C. Portfolio as a tool to stimulate teachers' reflections. Medical teacher. 2006;28:277-82.
129. Singh R, Naughton B, Taylor J, Koenigsberg M, Anderson D, McCausland L, et al. A comprehensive collaborative patient safety residency curriculum to address the ACGME core competencies. Medical education. 2006;39:1195-204.
130. Dannefer E, Bierer SB, Gladding S. Evidence within a portfolio-based assessment program: What do medical students select to document their performance? Medical teacher. 2012;34:215-20.
131. Rees C, Sheard C. The reliability of assessment criteria for undergraduate medical students' communication skills portfolios: The Nottingham experience. Medical education. 2004;38:138-44.
132. Kassab S, Bidmos M, Nomikos M, Daher-Nashif S, Kane T, Sarangi S, et al. Construct Validity of an Instrument for Assessment of Reflective Writing-Based Portfolios of Medical Students. Advances in Medical Education and Practice. 2020;Volume 11:397-404.
133. Michels N, Driessen E, Muijtjens A, Van Gaal L, Bossaert L, De Winter B. Portfolio assessment during medical internships: How to obtain a reliable and feasible assessment procedure? Education for health (Abingdon, England). 2009;22:313.
134. Susan E, Kinnair D. Integrating the assessment of interprofessional education into the health care curriculum. Journal of Taibah University Medical Sciences. 2016;11.
135. Bierer SB, Dannefer E. Does Students' Gender, Citizenship, or Verbal Ability Affect Fairness of Portfolio-Based Promotion Decisions? Results From One Medical School. Academic medicine : journal of the Association of American Medical Colleges. 2011;86:773-7.
136. Bierer SB, Dannefer E, Tetzlaff J. Time to Loosen the Apron Strings: Cohort-based Evaluation of a Learner-driven Remediation Model at One Medical School. Journal of General Internal Medicine. 2015.
137. Dannefer E. Beyond assessment of learning toward assessment for learning: Educating tomorrow's physicians. Medical teacher. 2013;35.
138. Dannefer E, Prayson R. Supporting students in self-regulation: Use of formative feedback and portfolios in a problem-based learning setting. Medical teacher. 2013;35.
139. Deitte L. Learning Portfolios in Radiology Residency Education: How Do I Get Started? Journal of the American College of Radiology : JACR. 2008;5:664-9.
140. Vance G, Williamson A, Frearson R, O'Connor N, Davison J, Steele C, et al. Evaluation of an established learning portfolio. The clinical teacher. 2013;10:21-6.

For peer review only

141. Suraseranivongse S, Chinachoti T, Aroonpruksakul N, Halilamien P, Rushatamukayanunt P, Raksamani K, et al. Development of a learning portfolio to assess the competency of anesthesia residents in Thailand. Asian Biomedicine. 2011;5.
142. Yates J, James D. Risk factors at medical school for subsequent professional misconduct: multicentre retrospective case-control study. BMJ. 2010;340:c2040.
143. Peeters MJ, Sexton ME. Use of reflective writing within interprofessional education: a mixed-methods analysis. J Interprof Care. 2020;34(3):307-14.
144. Remein CD, Childs E, Pasco JC, Trinquart L, Flynn DB, Wingerter SL, et al. Content and outcomes of narrative medicine programmes: a systematic review of the literature through 2019. BMJ Open. 2020;10(1):e031568.
145. Younas A, Ansar A. DEVELOPING A CURRICULUM FOR PROFESSIONAL ETHICS IN UNDERGRADUATE MEDICAL AND DENTAL EDUCATION: EVIDENCE FROM LITERATURE. 2020;70:1211-3.
146. Haig A, Dozier M. BEME Guide no 3: systematic searching for evidence in medical education--Part 1: Sources of information. Medical teacher. 2003;25(4):352-63.
147. Gordon M, Gibbs T. STORIES statement: publication standards for healthcare education evidence synthesis. BMC medicine. 2014;12(1):143.
148. Dawe R, Pike A, Kidd M, Janakiram P, Nicolle E, Allison J. Enhanced skills in global health and health equity: Guidelines for curriculum development. Canadian Medical Education Journal. 2017;8:e48-e60.
149. Sagasser M, Kramer A, Van der Vleuten C. How do postgraduate GP trainees regulate their learning and what helps and hinders them? A qualitative study. BMC medical education. 2012;12:67.
150. Burch V. Portfolios for assessment and learning: Guide Supplement 45.1 – Viewpoint. Medical teacher. 2011;33:1029-31.

Appendices

Appendix A. SEBA Methodology

Appendix B. Tabulated Summaries

Appendix C. Portfolio Content (Competencies Assessed and Assessment Modalities)

Figure Legend

Figure 2. The SEBA Process

Figure 2. PRISMA Flowchart

For peer review only

Figure 1. *The SEBA Process*

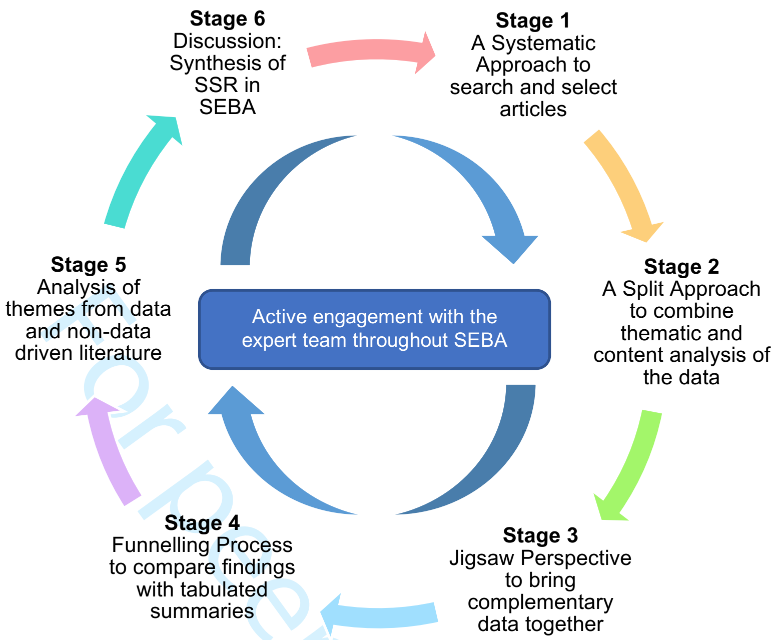

For peer review only

Figure 2. *PRISMA Flowchart*

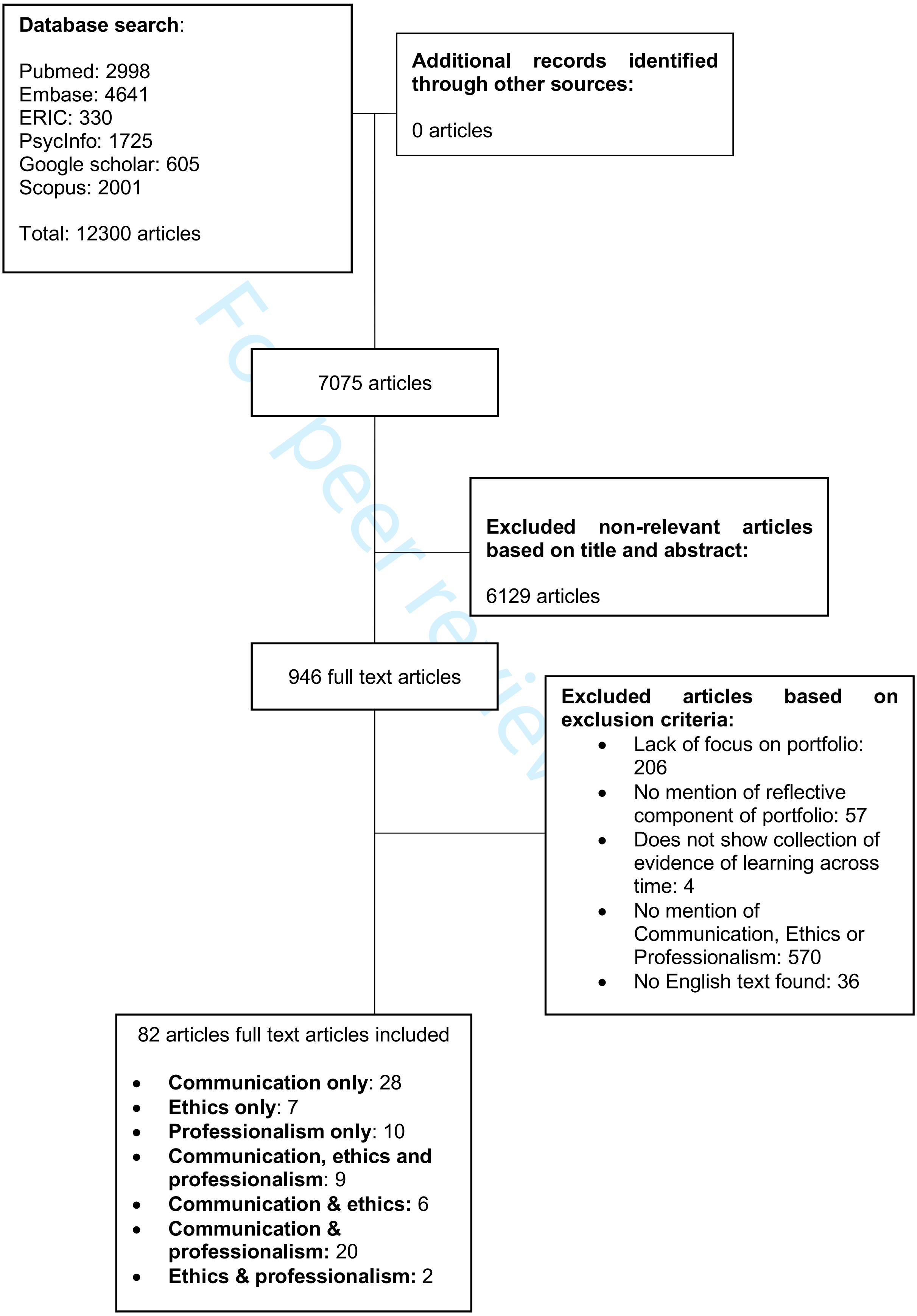

For peer review only - http://bmjopen.bmj.com/site/about/guidelines.xhtml

Appendices

Appendix A. SEBA Methodology

Theoretical lens

Krishna's Systematic Evidence-Based Approach (SEBA) is adopted to guide this systematic scoping review (SSR) (henceforth SSR in SEBA) (1-8). SEBA is composed of six stages- 1) Systematic Approach, 2) Split Approach, 3) Jigsaw Perspective, 4) Funnelling Process 5) Analysis of data and non-data driven literature, and 6) Discussion Synthesis (Figure 1). SEBA systematically map available data, structure the extraction of key characteristics of CEP education and its effects, synthesise and summarise actionable and applicable information across a diverse range of study formats and identify gaps in knowledge in current concepts. SEBA's constructivist approach (8-13) and relativist lens (14-17) acknowledges development of CEP competencies are individualised sociocultural constructs informed by the user's narratives, clinical competencies, contextual and environmental considerations.

Expert advice

An expert team consisting of a medical librarian from the Yong Loo Lin School of Medicine (YLLSoM) at the National University of Singapore (NUS), and local educational experts and clinicians at the National Cancer Centre Singapore (NCCS), the Palliative Care Unit, and the Institute of Population Health at the University of Liverpool, YLLSoM and Duke-NUS Medical School, ensured that the SEBA methodology was employed in a consistent manner within accepted practices.

Reflexivity

Personal reflexivity

Recognising that the research team are informed by their interests, narratives, clinical and research insights and contextual considerations, membership to the research and expert teams were made up of experienced physician-tutors, psychologists, methodologists, and educational scholars. Six members of these teams hold masters degrees in medical education, two hold masters degrees in ethics, two hold masters degrees in clinical research and two hold masters degrees in public health. Most of the expert and research team were experienced in quantitative research however five members of the team are experienced in qualitative methods. Six of the research and expert teams have published articles in peer-reviewed journals using the SEBA methodology. One member of the expert team is an experienced researcher at the Palliative Care Institute Liverpool and another is a member of the Health Data Science Department at the University of Liverpool. Both researchers from the University of Liverpool have

been collaborating with the research team on a number of studies pertaining to portfolio use, death and dying, moral distress, PIF and mentoring and are part of the team expanding the use of the SEBA methodology in medical education and palliative care.

Three senior members of the team are working on various projects on medical education and three others have already published articles in peer-reviewed journals in medical education. Six members of the research team were members of the PMI and have published SEBA guided reviews on mentoring, dignity and medical education. To ensure input from all members of the team, synchronous and asynchronous in-person and online meetings and Sandelowski and Barroso (18)'s approach to 'negotiated consensual validation' was used to reach consensus on the issues discussed.

Methodological reflexivity

Adopting a structured constructivist approach, we sought to build a holistic concept of current models of portfolios within medical education acknowledging however that we were limited by manpower and time constraints. We included articles featured in grey and bibliographic databases within the dates set in our selection criteria. Much of these theories required deeper consideration and discussions and we documented our discussions and decisions.

Iterative process

To ensure transparency and accountability, the expert team was involved in all stages of SEBA as part of the iterative process.

For peer review only

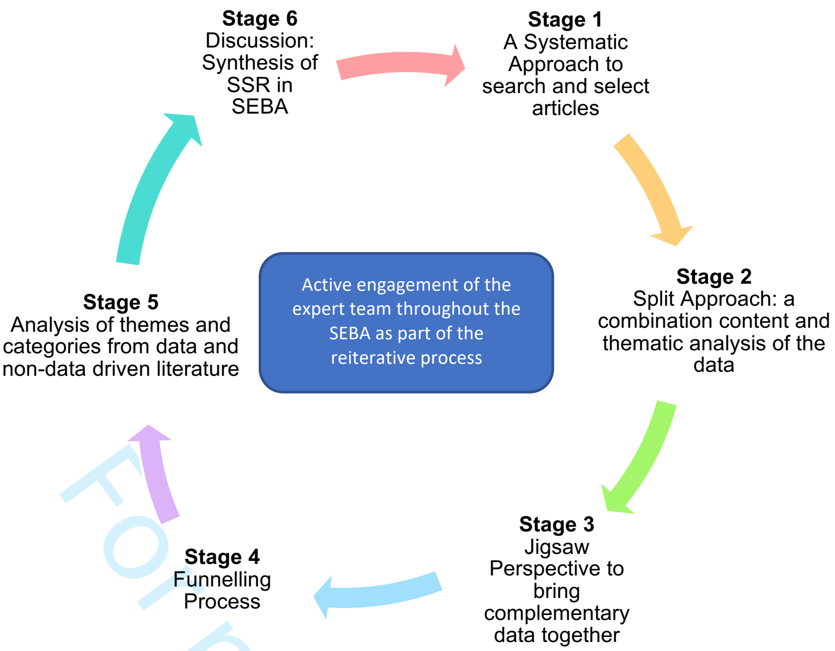

Figure 1. The SEBA Process

Stage 1 of SEBA: Systematic Approach

The SEBA methodology begins with the research and expert teams agreeing upon the research questions, the search terms and the databases to be scrutinised. In this case the research and expert team determined the primary research question: "*What is known about CEP portfolios?*". The secondary research questions were "*what role do CEP portfolios have in teaching and assessing CEP development?*".

Inclusion criteria

A PICOs (Population, Intervention, Comparison, Outcome, Study design) format was adopted to guide the research process (Table 1). There was no comparison group.

Table 1. *PICOS Inclusion and Exclusion Criteria*

PICOS	Inclusion criteria	Exclusion criteria
**Population**	• Undergraduate and postgraduate medical students<br>• Qualified medical doctors, physician or resident; medical officer, registrar, house officer, attending, consultant	• Allied health specialties such as Pharmacy, Dietetics, Chiropractic, Midwifery, Podiatry, Speech Therapy, Occupational and Physiotherapy<br>• Non-medical specialties such as Clinical and Translational Science, Alternative and Traditional Medicine, Veterinary, Dentistry
**Intervention**	• portfolios in undergraduate and postgraduate medical education for teaching and assessment of Communication, Ethics and Professionalism<br><br>Criteria of a portfolio:	Other documentation methods or learning tools that are:<br>• Not longitudinal or single timepoint<br>• Does not include personal intellectual engagement with the content and associated learning (for instance, curriculum vitae, logbooks

For peer review only

PICOS	Inclusion criteria	Exclusion criteria
	• longitudinal (more than a single timepoint) assessment data<br>• candidate's personal engagement with portfolio content and associated learning<br>• Interventions meeting the above criteria were included regardless of whether they were referred to as portfolios<br><br>All types of portfolios were included in the study:<br>• For instance: electronic & non-electronic; formative & summative or combined; clinical & non-clinical<br>• Portfolios with input from students and/or residents and/or doctors, and/or input from faculty members and other individuals<br>• Portfolios with different structures: extent by which the structure has been prescribed and/or left to individual discretion	and the use of personal digital assistants)
**Comparison/ Context**	NA	NA
**Outcome**	Papers that measured the following outcomes were included:<br>• Effectiveness of the use of portfolios to assess and teach Communication, Ethics and Professionalism<br>• Impact of the use of portfolios on medical students (both undergraduate and postgraduate)<br>• Impact of the use of portfolios on the faculty	NA
**Study design**	• Articles in English or translated to English<br>• Articles published from 1st January 2000 to 31st December 2020<br>• Databases: PsycINFO, EMBASE, PubMed, ERIC, Scopus, Google Scholar<br>• All study designs including:<br>• Mixed methods research, meta-analyses, systematic reviews, randomised controlled trials, cohort studies, case-control studies, cross-sectional studies, descriptive papers, grey literature, opinions, letters, commentaries and editorials	NA

Three members of the research team carried out independent ancestry searches of seven leading journals in medical education (Academic Medicine, Medical Education, Medical Teacher, Advances Health Sciences Education, BMC Medical Education, Teaching and Learning in Medicine and Perspectives on Medical Education) accessed through the NUS library portal. In keeping with Pham, Rajic (19)'s recommendations, the searches were restricted to articles published between 1st January 2000 and 31st December 2020 to accommodate to existing manpower and time constraints. Quantitative, mixed and qualitative research methodologies meeting the inclusion criteria were included.

For peer review only

Table 2. *Search Strategy for PubMed, Embase, PsycINFO, ERIC, Scopus, and Google Scholar databases.*

		Mesh Terms	tiab
		(1 OR 2 OR 3) AND 4	
**Population**	Medical Students OR Doctors	[1] "Physicians"[MeSH] OR "Students, Medical"[MeSH] OR "Clinical Clerkship"[MeSH] OR "Medicine"[Mesh] OR "Education, Medical"[Mesh] OR "Clinical Competence"[Mesh]	[2] Physician[tiab] OR Physicians[tiab] OR resident[tiab] OR residents[tiab] OR residency[tiab] OR residencies[tiab] OR practice[tiab] OR practitioner[tiab] OR practitioners[tiab] OR doctor[tiab] OR doctors[tiab] OR houseman[tiab] OR housemanship[tiab] OR housemen[tiab] OR medical[tiab] OR clinical[tiab] OR pre-clinical[tiab] OR preclinical[tiab] OR clinician*[tiab] OR surgery[tiab] OR surgical[tiab] OR surgeon*[tiab] OR clerkship*[tiab] OR specialist*[tiab]
			[3] ("Educational Measurement/methods"[Mesh] OR "Educational Measurement/standards"[Mesh] OR "Documentation/methods"[Mesh] OR "Benchmarking*"[MeSH] OR "Competency-based education/standards*"[MeSH] OR "Records*"[MeSH]) AND (medical[tiab] OR clinical[tiab] OR pre-clinical[tiab] OR preclinical[tiab] OR clinician*[tiab] OR surgery[tiab] OR surgical[tiab] OR surgeon*[tiab] OR clerkship*[tiab] OR specialist*[tiab])
**Intervention**	Portfolios		[4] Portfolio[tiab] OR portfolios[tiab] OR e-portfolio[tiab] OR e-portfolios[tiab] OR "curriculum vitae"[tiab] OR "personal statement"[tiab] OR "personal statements"[tiab]

For peer review only

Members of the research team carried out independent searches of databases. To facilitate this approach, the search process saw experienced senior researchers well-versed in carrying out systematic reviews and systematic scoping reviews each meet with a team of two to three medical students to guide them database searches. This approach was to enhance training of new researchers and to ensure that at least two teams were independently reviewing each database. Each team met regularly and discussed their findings. After a search of the first 100 articles in a particular database, the medical students and the senior researcher compared their findings at an online meeting.

Subsequently, the teams met at specific time points, often after reviewing a predetermined number of included articles to discuss their concerns, exchange opinions and advance their understanding of the research process and the area of study. Sandelowski and Barroso (18)'s 'negotiated consensual validation' was used to achieve consensus on the final list of titles to be reviewed. The teams repeated this process, independently studying all the

full text articles on the final list of titles, creating their own lists of articles to be included and discussing their findings at weekly online research meetings over the first 4 months of the project. Consensus on the final list of articles to be analysed was achieved following at least three online meetings when discrepancies were reviewed and discussed by each member of the research team.

As this was a training process for many of the participants who were medical students participating in the Palliative Medicine Initiative, senior mentors and peer-mentors were frequently involved in guiding and discussing individual findings over and above the online meetings especially at the start of each stage of the research process. Interrater reliability was not evaluated.

Stage 2 of SEBA: Split Approach

Krishna's 'Split Approach' (18-23) was employed to enhance the reliability of the data analyses. This saw three groups of researchers independently analysing the included articles.

The first team summarised and tabulated the included full-text articles in keeping with recommendations drawn from Wong, Greenhalgh (24)'s RAMESES publication standards: meta-narrative reviews and Popay, Roberts (25)'s "Guidance on the conduct of narrative synthesis in systematic reviews". The tabulated summaries served to ensure that key aspects of included articles were not lost.

Concurrently, the second team analysed the included articles using Braun and Clarke (26)'s approach to thematic analysis. In Phase 1, the research team carried out independent reviews, 'actively' reading the included articles to find meaning and patterns in the data. In Phase 2, 'codes' were constructed from the 'surface' meaning and collated into a code book to code and analyse the rest of the articles using an iterative step-by-step process. As new codes emerged, these were associated with previous codes and concepts. In Phase 3, the categories were organised into themes that best depict the data. An inductive approach allowed themes to be "defined from the raw data without any predetermined classification" (27). In Phase 4, the themes were refined to best represent the whole data set and discussed. In Phase 5, the research team discussed the results of their independent analysis online and at reviewer meetings. 'Negotiated consensual validation' was used to determine a final list of themes approach and ensure the final themes.

A third team of researchers employed Hsieh and Shannon (28)'s approach to directed content analysis to analyse the included articles. Analysis using the directed content analysis approach involved "identifying and operationalizing a priori coding categories". The categories employed in the content analysis for undergraduate communications were Rider et al. (29) 's *"A model for communication skills assessment across the undergraduate curriculum",* Goldie (30)'s *"Review of ethics curricula in undergraduate medical education"*, Duffy et al. (31)'s *"Assessing Competence in Communication and Interpersonal Skills: The Kalamazoo II Report"* and Hong et al. (32)'s *"Postgraduate Ethics Training Programs: A Systematic Scoping Review"*. Tay et al. (33)'s *"Assessing Professionalism in Medicine - A Scoping Review of Assessment Tools from 1990 to 2018"* was employed for codes for professionalism and David (34)'s article *"AMEE Medical Education Guide No. 24: Portfolios as a method of student assessment"* was then used to contextualise their use in portfolios. Any data not captured by these codes were assigned a new code.

By using directed content analysis, this "Split Approach" sought to address shortcomings int thematic analysis. This was done by ironing out disparities in data and increases the validity of the identified themes (35). It also limits inherent biases and condenses the interpretations of terminology used by each team's members. Consistency with existing literature is further offered by directed content analysis, by using existing data to identify codes and categories. The codes were used systematically and objectively, strengthening the validity and reliability of a positivist approach (35, 36). The transparency of this approach is enforced by clearly defined categories, together with references throughout the analytical process.

Stage 3 of SEBA: Jigsaw Perspective

The themes and categories identified in the Split Approach were viewed as pieces of a jigsaw puzzle where overlapping/complementary pieces were combined to create a bigger piece of the puzzle referred to as themes/categories. This process was guided by Phases 4 to 6 of France, Uny (37)'s adaptation of Noblit, Hare (38)'s seven phases of meta-ethnography. As per Phase 4, the themes and the categories identified in the Split Approach are grouped together according to their focus. These groupings of categories and themes were then contextualised through the review of the articles from which they were drawn from. Reciprocal translation was used to determine if the themes and categories could be used interchangeably. This allowed the themes and categories to be combined to form themes/categories.

France, Uny's adaptation:
Phase 1: Getting started, deciding on the focus of the synthesis.
Phase 2: Deciding what is relevant to the initial interest.
Phase 3: Reading the Studies.
Phase 4: Determining how the studies are related.
Phase 5: Translating the studies into one another.
Phase 6: Synthesising the translations.
Phase 7: Expressing the synthesis.

Codes derived from thematic analysis (TA): indications, characteristics and strengths and limitations

Codes derived from directed content analysis (DCA): indication for portfolio, portfolio content, portfolio design and strengths and limitations

Overlaps between indications and strengths and limitations allowed the combination of these categories and themes creating two themes/categories called indications and strengths and limitations. Careful review of the categories- portfolio content and portfolio design were subsets of the theme characteristics. For a more accurate presentation, the overlaps were conflated and presented together with the TA codes indicated under the DCA code. (Refer to Domain 4 table)

Themes Identified	Categories Identified	Domains Created

For peer review only

Indications	indications	Indications
characteristics	content	Characteristics
	Portfolio design	
Strengths and limitations	Strengths and limitations	Dignity-conserving Measures

For peer review only

Stage 4 of SEBA: Funnelling Process

The Funnelling Process employs Phases 3 to 5 of France, Uny's adaptation to juxtapose the themes/categories with key messages identified in the tabulated summaries to create domains. These domains form the basis for 'the line of argument' in Stage 6 of SEBA.

For peer review only

For peer review only

For peer review only

Domain 2. Relationship between Dignity, WTHD and Assisted Dying

Subdomains	Codes
**Increasing WTHD**	▪ Loss of dignity (39-81)<br>▪ Fear of loss of dignity (42, 44, 52, 59, 82, 83)<br>▪ Loss of autonomy (39, 40, 42, 45, 47, 50-53, 55, 59, 62, 65, 69, 70, 73, 74, 77, 79, 81, 82, 84-88)<br>▪ Fear of loss of autonomy (42, 54, 70, 89)
**Decreasing WTHD/protective factors**	▪ Addressing of dignity related issues improves health-related quality of life (46)<br>▪ Improving dignity reduces desire for euthanasia (50)
**Effect of assisted dying on dignity**	▪ Reclamation/preservation of dignity (45, 49, 58, 90-95)<br>▪ Lack of assisted dying interferes with dignity (50, 76, 96)<br>▪ Reclamation/preservation of autonomy (43, 45, 49, 51, 52, 67, 73, 74, 77, 85, 88, 92, 94, 97-100)<br>▪ Lack of assisted dying interferes with autonomy (49, 96)<br>▪ Violation of autonomy (100)

For peer review only

Domain 3. Stakeholder Perspectives on Dignity

**Subdomains**	**Codes**	
**Patient Perspectives**	**Positive Aspects**<br>▪ Respect for humanity (67)<br>▪ Right to die (99)<br>▪ Unaffected by fear of death (48)<br>▪ Good death (67, 73, 99, 101-103)<br>- Maintaining control of their death (67, 73, 99, 101-103)<br>- Ending suffering (67, 73)<br>▪ Perceived futility (73, 74)<br>▪ General support (73, 98)<br>▪ Wanting the option for themselves (98)	**Negative Aspects**<br>▪ Religious concerns (67, 73, 102)<br>▪ Ethical concerns (62, 67, 73)<br>- Inability to ascertain patient consent (62, 67)<br>- Equating to murder/suicide (67)<br>▪ Change in ideologies (50)<br>▪ Deterioration of patient-doctor relationship (58, 62, 74, 101)<br>- Loss of faith in palliative/hospice care (62, 101)<br>- Feeling of abandonment when WTHD is turned down (74)<br>- Poor communications (58)<br>▪ Against legislation (73)
**Healthcare Provider Perspectives**	**Positive Aspects**<br>▪ Good death for patients (42, 47, 49, 50, 54, 56, 57, 61, 69, 70, 82, 87, 101, 103)<br>- Fulfilling patients' desire for control (47, 50, 56, 61, 82, 87, 101, 104)<br>- Ending suffering (42, 54, 57, 61, 69, 70, 103)<br>- Not requiring a long life (49)<br>▪ Feeling helpless (68)<br>▪ Non-abandonment of patients (90)<br>▪ Reducing patients' family burden (90)<br>▪ Avoiding unlawful hastening death (50)<br>▪ General support (40, 47, 62, 68-70, 75, 82, 90, 101, 103-105)<br>▪ Disregarding the need for alternatives (i.e. palliative care) (50)	**Negative Aspects**<br>▪ Religious concerns (106)<br>▪ Disrespecting patients' autonomy by not entertaining their wishes (104, 107)<br>▪ Emotional/moral distress (49, 50, 69, 91, 101, 108-113)<br>- Guilt (91, 112)<br>- Opposing medical principles (49, 69, 101, 108, 110, 111)<br>- Grief, avoidance after carrying out WTHD (113)<br>- Turning doctors into killers (111)<br>▪ No right to die (90)<br>▪ Unable to ascertain if patient's disease is incurable (109)<br>▪ Change in perceptions after disease (82)<br>▪ WTHD should not involve family members (113)<br>▪ "Slippery slope" (110)<br>▪ Societal pressure (55, 74)<br>▪ Disapproval of WTHD (62, 69, 75, 82, 101, 107, 113-115)<br>- Disagreement with the law (115)<br>- Reluctance to carry out WTHD (75, 101)<br>▪ Proposing alternatives (57, 62, 63, 90, 105, 111)<br>- Improving quality of life of dying patients (62, 111)<br>- Better palliative care (63, 90, 105)<br>- Healing (57)<br>▪ Death as an unknown (57)

For peer review only

For peer review only - http://bmjopen.bmj.com/site/about/guidelines.xhtml

		▪ Disapproval of 'tired of living' as a reason for WTHD (57)
**Lawmaker Perspectives**	▪ Belief in a right to die (76)<br>▪ Avoiding unlawful hastening death (96)<br>▪ Government's duty to consider patients' WTHD (74)<br>▪ Approval of WTHD (76, 100)	▪ "Slippery slope" (98, 114)<br>- Risk of abuse (114)<br>▪ Instigating social pressure (on the elderly) (74)<br>▪ Disapproval of WTHD (74, 83, 106, 114)

For peer review only

Domain 4. Dignity Conserving Measures

Subdomains	Codes
**Advantages to Dignity Conserving Measures**	▪ Palliative services<br>- Palliative care (40, 44, 47, 49, 50, 62, 63, 66, 73, 82, 90, 91, 103, 111, 116-118)<br>- Palliative sedation (47, 49, 91, 119)<br>- Palliative starvation (85, 109)<br>▪ Pain and symptom management<br>- Control of physical pain (58, 66, 75, 104, 107, 108, 119)<br>- Control of psychological symptoms (75, 102, 120)<br>▪ Hospice care (46, 47, 75, 100, 106, 107, 117)<br>▪ Holistic approach (43, 57, 105, 111, 112, 117, 121, 122)<br>▪ Ease of access to information (47, 57, 71, 87, 92, 100, 112, 117)<br>▪ Distributive justice (55)<br>▪ Social support<br>- Religious (75)<br>- Familial (45)<br>- Healthcare providers (47, 57, 58, 71, 87, 112)
**Limitations to Dignity Conserving Measures**	▪ Cannot completely address:<br>- Existential suffering (54)<br>- Pain (50)<br>- Complete loss of mobility (103)<br>- Extreme shortness of breath (103)<br>- Fear of sudden and rapid bleeding (103)<br>- Ongoing stress of losing autonomy and dignity (103)<br>▪ Side effects of pain medication (58)

For peer review only

Stage 5 of SEBA: Analysis of data and non-data driven literature

A novel aspect of the SEBA methodology has been its inclusion of position, perspective, conference, reflective and opinion papers, editorials, commentaries, letters, posters, oral presentations, forum discussions, interviews, blogs, governmental reports, policy statements and surveys from PubMed, Embase, PsycINFO, Cochrane Database of Systematic Reviews, CINAHL, Scopus databases and the Journal of Pain and Symptom Management, BMC Palliative Medicine, Death Studies, and Palliative Medicine. With many current survey and assessment tools not able to capture the intricate connections and personalised nature of wider concepts of dignity, assisted dying, WTHD, personhood and identity, these resources are a rich source of information. These resources also capture wider patient, HCP and lawmaker perspectives and offer information on ethical, existential, and societal considerations often excluded by traditional systematic reviews as evidenced by recent reviews into how physicians and patients deal with death and dying and moral distress (123-130)

The use of non-data driven articles such as position, perspective, conference, reflective and opinion papers, editorials, commentaries, letters, posters, oral presentations, forum discussions, interviews, blogs, governmental reports, policy statements and surveys is consistent with a constructivist approach that includes all forms of data. In addition, use of non-data driven articles is a valuable source of information for subject matter such as concepts of dignity which are notoriously difficult to study and open to sociocultural considerations.

However, with some of these sources neither peer-reviewed nor necessarily evidence-based, they were differentiated from primary research, compiled and thematically analysed. The themes identified from primary data sources and non-evidence based and/or tertiary data sources were then compared to enhance further the accountability and reproducibility.

With the themes from the two groups found to be similar, the expert and research teams were satisfied that their inclusion did not bias the overall data.

Stage 6 of SEBA: Synthesis of the Discussion

The Best Evidence Medical Education (BEME) Collaboration Guide (131) and the Structured approach to the Reporting In healthcare education of Evidence Synthesis (STORIES) (132) were used to guide the discussion.

For peer review only

References

1. Kow CS, Teo YH, Teo YN, Chua KZY, Quah ELY, Kamal NHBA, et al. A systematic scoping review of ethical issues in mentoring in medical schools. BMC Medical Education. 2020;20(1):1-10.
2. Ngiam LXL, Ong YT, Ng JX, Kuek JTY, Chia JL, Chan NPX, et al. Impact of Caring for Terminally Ill Children on Physicians: A Systematic Scoping Review. Am J Hosp Palliat Care. 2020:1049909120950301.
3. Krishna LKR, Tan LHE, Ong YT, Tay KT, Hee JM, Chiam M, et al. Enhancing Mentoring in Palliative Care: An Evidence Based Mentoring Framework. Journal of Medical Education and Curricular Development. 2020;7:2382120520957649.
4. Chia EWY, Huang H, Goh S, Peries MT, Lee CCY, Tan LHE, et al. A Systematic Scoping Review of Teaching and Evaluating Communications in The Intensive Care Unit. The Asia-Pacific Scholar. In Press.
5. Kuek JTY, Ngiam LXL, Kamal NHA, Chia JL, Chan NPX, Abdurrahman ABHM, et al. The impact of caring for dying patients in intensive care units on a physician's personhood: a systematic scoping review. 2020;15(1):1-16.
6. Hong DZ, Lim AJS, Tan R, Ong YT, Pisupati A, Chong EJX, et al. A Systematic Scoping Review on Portfolios of Medical Educators. Journal of Medical Education and Curricular Development. 2021;8:23821205211000356.
7. Goh S, Wong RSM, Quah ELY, Chua KZY, Lim WQ, Ng ADR, et al. Mentoring in palliative medicine in the time of covid-19: a systematic scoping review. BMC medical education. 2022;22(1):1-15.
8. Bok C, Ng CH, Koh JWH, Ong ZH, Ghazali HZB, Tan LHE, et al. Interprofessional communication (IPC) for medical students: a scoping review. BMC Med Educ. 2020;20(1):372.
9. Ng YX, Koh ZYK, Yap HW, Tay KT, Tan XH, Ong YT, et al. Assessing mentoring: A scoping review of mentoring assessment tools in internal medicine between 1990 and 2019. PloS one. 2020;15(5):e0232511.
10. Bousquet J, Schunemann HJ, Samolinski B, Demoly P, Baena-Cagnani CE, Bachert C, et al. Allergic Rhinitis and its Impact on Asthma (ARIA): achievements in 10 years and future needs. J Allergy Clin Immunol. 2012;130(5):1049-62.
11. Nur Haidah Ahmad Kamal LHET, Ruth Si Man Wong, Ryan Rui Song Ong, Ryan, Ern Wei Seow EKYL, Zheng Hui Mah, Min Chiam, Annelissa Mien Chew Chin, Jamie Xuelian Zhou, Gillian Li Gek Phua, Eng Koon Ong, Jin Wei Kwekc, Kiley Wei-Jen Loh and, Krishna LKR. Enhancing education in Palliative Medicine: the role of Systematic Scoping Reviews. Palliative Medicine & Care: Open Access. 2020;7(1):1-11.
12. Ryan Rui Song Ong REWS, Ruth Si Man Wong. A Systematic Scoping Review of Narrative Reviews in Palliative Medicine Education. Palliative Medicine & Care: Open Access. 2020;7(1):1-22.
13. Zheng Hui Mah RSMW, Ryan Ern Wei Seow Eleanor Kei Ying Loh, Nur Haidah, Ahmad Kamal RRSO, Lorraine Hui En Tan, Min Chiam, Annelissa Mien Chew Chin,, Jamie Xuelian Zhou GLGP, Yoke-Lim Soong, Jin Wei Kwek, and Lalit Kumar Radha Krishna. A Systematic Scoping Review of Systematic Reviews in Palliative Medicine Education. Palliative Medicine & Care: Open Access. 2020;7(1):1-12.
14. Pring R. The 'False Dualism' of Educational Research. Journal of Philosophy of Education. 2000;34(2):247-60.
15. Crotty M. The foundations of social research: Meaning and perspective in the research process: Sage; 1998 Oct 15.

For peer review only

16. Ford DW, Downey L, Engelberg R, Back AL, Curtis JR. Discussing religion and spirituality is an advanced communication skill: an exploratory structural equation model of physician trainee self-ratings. J Palliat Med. 2012;15(1):63-70.
17. Schick-Makaroff K, MacDonald M, Plummer M, Burgess J, Neander W. What synthesis methodology should I use? A review and analysis of approaches to research synthesis. AIMS public health. 2016;3(1):172.
18. Sandelowski M BJ. Handbook for synthesizing qualitative research. New York: Springer; 2007.
19. Pham MT, Rajic A, Greig JD, Sargeant JM, Papadopoulos A, McEwen SA. A scoping review of scoping reviews: advancing the approach and enhancing the consistency. Research synthesis methods. 2014;5(4):371-85.
20. Wen Jie Chua CWSC, Fion Qian Hui Lee, Eugene Yong Hian Koh, Ying Pin Toh, Stephen Mason, Lalit Kumar Radha Krishna. Structuring Mentoring in Medicine and Surgery. A Systematic Scoping Review of Mentoring Programs Between 2000 and 2019. Journal of Continuing Education in the Health Professions. 2020;40(3):158-68.
21. Yong Xiang Ng ZYKK, Hong Wei Yap, Kuang Teck Tay, Xiu Hui Tan, Yun Ting Ong, Lorraine Hui En Tan, Annelissa Mien Chew Chin, Ying Pin Toh, Sushma Shivananda, Scott Compton, Stephen Mason, Ravindran Kanesvaran, Lalit Krishna. Assessing mentoring: A scoping review of mentoring assessment tools in internal medicine between 1990 and 2019. . PLOS ONE. 2020;15(5):e0232511.
22. Peters MD, Godfrey CM, Khalil H, McInerney P, Parker D, Soares CB. Guidance for conducting systematic scoping reviews. Int J Evid Based Healthc. 2015;13(3):141-6.
23. Sambunjak D, Straus SE, Marusic A. A systematic review of qualitative research on the meaning and characteristics of mentoring in academic medicine. J Gen Intern Med. 2010;25(1):72-8.
24. Wong G, Greenhalgh T, Westhorp G, Buckingham J, Pawson R. RAMESES publication standards: meta-narrative reviews. BMC medicine. 2013;11(1):20.
25. Popay J, Roberts H, Sowden A, Petticrew M, Arai L, Rodgers M, et al. Guidance on the conduct of narrative synthesis in systematic reviews. A product from the ESRC methods programme Version. 2006;1:b92.
26. Braun V, Clarke V. Using thematic analysis in psychology. Qualitative research in psychology. 2006;3(2):77-101.
27. Cassol H, Pétré B, Degrange S, Martial C, Charland-Verville V, Lallier F, et al. Qualitative thematic analysis of the phenomenology of near-death experiences. PloS one. 2018;13(2):e0193001.
28. Hsieh H-F, Shannon SE. Three Approaches to Qualitative Content Analysis. Qualitative Health Research. 2005;15(9):1277-88.
29. Rider EA, Hinrichs MM, Lown BA. A model for communication skills assessment across the undergraduate curriculum. Med Teach. 2006;28(5):e127-34.
30. Goldie J. Review of ethics curricula in undergraduate medical education. Med Educ. 2000;34(2):108-19.
31. Duffy FD, Gordon GH, Whelan G, Cole-Kelly K, Frankel R, Buffone N, et al. Assessing competence in communication and interpersonal skills: the Kalamazoo II report. Acad Med. 2004;79(6):495-507.
32. Hong DZ, Goh JL, Ong ZY, Ting JJQ, Wong MK, Wu J, et al. Postgraduate ethics training programs: a systematic scoping review. BMC Medical Education. 2021;21(1):338.
33. Tay KT, Ng S, Hee JM, Chia EWY, Vythilingam D, Ong YT, et al. Assessing Professionalism in Medicine - A Scoping Review of Assessment Tools from 1990 to 2018. J Med Educ Curric Dev. 2020;7:2382120520955159.

For peer review only

34. David M, Davis M, Harden R, Howie P, Ker J, Pippard M. AMEE Medical Education Guide No. 24: Portfolios as a method of student assessment. Medical teacher. 2001;23:535-51.
35. Dixon-Woods M, Agarwal S, Young B, Jones D, Sutton A. Integrative Approaches to Qualitative and Quantitative Evidence. 2004.
36. Bryman A. Social Research Methods: Oxford University Press; 2016.
37. France EF, Uny I, Ring N, Turley RL, Maxwell M, Duncan EAS, et al. A methodological systematic review of meta-ethnography conduct to articulate the complex analytical phases. BMC Medical Research Methodology. 2019;19(1):35.
38. Noblit GW, Hare RD, Hare RD. Meta-ethnography: Synthesizing qualitative studies: sage; 1988.
39. Al Rabadi L, LeBlanc M, Bucy T, Ellis LM, Hershman DL, Meyskens FL, et al. Trends in Medical Aid in Dying in Oregon and Washington. JAMA Network Open. 2019;2(8):e198648-e.
40. Emanuel EJ, Onwuteaka-Philipsen BD, Urwin JW, Cohen J. Attitudes and practices of euthanasia and physician-assisted suicide in the United States, Canada, and Europe. JAMA: Journal of the American Medical Association. 2016;316(1):79-90.
41. Brinkman-Stoppelenburg A, Vergouwe Y, van der Heide A, Onwuteaka-Philipsen BD. Obligatory consultation of an independent physician on euthanasia requests in the Netherlands: What influences the SCEN physicians judgment of the legal requirements of due care? Health Policy. 2014;115(1):75-81.
42. Buiting H, van Delden J, Onwuteaka-Philpsen B, Rietjens J, Rurup M, van Tol D, et al. Reporting of euthanasia and physician-assisted suicide in the Netherlands: Descriptive study. BMC Medical Ethics Vol 10 2009, ArtID 18. 2009;10.
43. Konishi E, Davis A. The right-to-die and the duty-to-die: Perceptions of nurses in the West and in Japan. International Nursing Review. 2001;48(1):17-28.
44. Chapple A, Ziebland S, McPherson A, Herxheimer A. What People Close to Death Say About Euthanasia and Assisted Suicide: A Qualitative Study. Journal of Medical Ethics: Journal of the Institute of Medical Ethics. 2006;32(12):706-10.
45. Coyle N, Sculco L. Expressed desire for hastened death in seven patients living with advanced cancer: a phenomenologic inquiry. Oncol Nurs Forum. 2004;31(4):699-709.
46. Crespo I, Rodríguez-Prat A, Monforte-Royo C, Wilson KG, Porta-Sales J, Balaguer A. Health-related quality of life in patients with advanced cancer who express a wish to hasten death: A comparative study. Palliat Med. 2020;34(5):630-8.
47. Drum CE, White G, Taitano G, Horner-Johnson W. The Oregon Death with Dignity Act: Results of a literature review and naturalistic inquiry. Disability and Health Journal. 2010;3(1):3-15.
48. Ferrand E, Dreyfus JF, Chastrusse M, Ellien F, Lemaire F, Fischler M. Evolution of requests to hasten death among patients managed by palliative care teams in France: a multicentre cross-sectional survey (DemandE). Eur J Cancer. 2012;48(3):368-76.
49. Ramsey C. The right to die: beyond academia. Monash Bioeth Rev. 2016;34(1):70-87.
50. Reagan P, Hurst R, Cook L, Zylicz Z, Otlowski M, Veldink JH, et al. Physician-assisted death: Dying with dignity? Lancet Neurology. 2003;2(10):637-43.
51. Rodríguez-Prat A, Balaguer A, Booth A, Monforte-Royo C. Understanding patients' experiences of the wish to hasten death: an updated and expanded systematic review and meta-ethnography. BMJ Open. 2017;7(9):e016659.
52. Rodríguez-Prat A, van Leeuwen E. Assumptions and moral understanding of the wish to hasten death: a philosophical review of qualitative studies. Medicine, Health Care & Philosophy. 2018;21(1):63-75.

For peer review only

53. Wang L, Elliott M, Henson LJ, Gerena-Maldonado E, Strom S, Downing S, et al. Death with dignity in Washington and Oregon patients with amyotrophic lateral sclerosis. Neurology. 2016;86(16).
54. Fischer S, Huber CA, Furter M, Imhof L, Mahrer Imhof R, Schwarzenegger C, et al. Reasons why people in Switzerland seek assisted suicide: the view of patients and physicians. Swiss Med Wkly. 2009;139(23-24):333-8.
55. Freeman LM, Rose SL, Youngner SJ. Poverty: Not a Justification for Banning Physician-Assisted Death. Hastings Cent Rep. 2018;48(6):38-46.
56. Ganzini L, Goy ER, Dobscha SK. Why Oregon patients request assisted death: Family members' views. Journal of General Internal Medicine. 2008;23(2):154-7.
57. Goligher EC, Wesley Ely E, Sulmasy DP, Bakker J, Raphael J, Volandes AE, et al. Physician-Assisted Suicide and Euthanasia in the ICU: A Dialogue on Core Ethical Issues. Critical Care Medicine. 2017;45(2):149-55.
58. Hendry M, Pasterfield D, Lewis R, Carter B, Hodgson D, Wilkinson C. Why do we want the right to die? A systematic review of the international literature on the views of patients, carers and the public on assisted dying. Palliative Medicine. 2013;27(1):13-26.
59. Hiscox WE. Physician-assisted suicide in Oregon: The 'death with dignity' data. Medical Law International. 2007;8(3):197-220.
60. Kade WJ, Kade WJ. Death with dignity: a case study. Annals of Internal Medicine. 2000;132(6):504-6.
61. Kouwenhoven PS, van Thiel GJ, Raijmakers NJ, Rietjens JA, van der Heide A, van Delden JJ. Euthanasia or physician-assisted suicide? A survey from the Netherlands. Eur J Gen Pract. 2014;20(1):25-31.
62. Bailey F. "I am not afraid of dying. I just don't want to be there when it happens.". Medical Care. 2008;46(12):1195-7.
63. Barutta J, Vollmann J. Physician-assisted death with limited access to palliative care. Journal of Medical Ethics: Journal of the Institute of Medical Ethics. 2015;41(8):652-4.
64. Bolt EE, Pasman H, Deeg DJ, Onwuteaka-Philipsen BD. From advance euthanasia directive to euthanasia: Stable preference in older people? Journal of the American Geriatrics Society. 2016;64(8):1628-33.
65. Dees M, Vernooij-Dassen M, Dekkers W, van Weel C. Review unbearable suffering of patients with a request for euthanasia or physician-assisted suicide: An integrative review. Psycho-Oncology. 2010;19(4):339-52.
66. Duckett S. Pathos, death talk and palliative care in the assisted dying debate in Victoria, Australia. Mortality. 2020;25(2):151-66.
67. Eliott JA, Olver IN. Dying cancer patients talk about euthanasia. Social Science & Medicine. 2008;67(4):647-56.
68. McPherson T. My mum wanted assisted dying but we watched her die slowly and in pain. BMJ: British Medical Journal (Clinical Research Edition). 2012;344:e4007-e.
69. Mukhopadhyay S, Banerjee D. Physician assisted suicide in dementia: A critical review of global evidence and considerations from India. Asian J Psychiatr. 2021;64:102802.
70. Rowe Iii MJ. Beliefs. Chicago, Illinois: American Medical Association; 2015. p. 877-8.
71. Roy DJ. Palliative care and euthanasia: a continuing need to think again. J Palliat Care. 2002;18(1):3-5.
72. van Tol D, Rietjens J, van der Heide A. Judgment of unbearable suffering and willingness to grant a euthanasia request by Dutch general practitioners. Health Policy. 2010;97(2-3):166-72.

For peer review only

73. Wilson KG, Chochinov HM, McPherson CJ, Skirko MG, Allard P, Chary S, et al. Desire for euthanasia or physician-assisted suicide in palliative cancer care. Health Psychology. 2007;26(3):314-23.
74. Florijn BW. From Reciprocity to Autonomy in Physician-Assisted Death: An Ethical Analysis of the Dutch Supreme Court Ruling in the Albert Heringa Case. Am J Bioeth. 2022;22(2):51-8.
75. Ganzini L, Nelson HD, Schmidt TA, Kraemer DF, Delorit MA, Lee MA. Physicians' experiences with the Oregon Death with Dignity Act. N Engl J Med. 2000;342(8):557-63.
76. Kishore RR. Aruna Shanbaug and the right to die with dignity: the battle continues. Indian J Med Ethics. 2016;1(1):38-46.
77. Royo C, Villavicencio-Chávez C, Tomás-Sábado J, Mahtani V, Balaguer A. What Lies behind the Wish to Hasten Death? A Systematic Review and Meta-Ethnography from the Perspective of Patients. PloS one. 2012;7:e37117.
78. Bahník Š, Vranka MA, Trefná K. What makes euthanasia justifiable? The role of symptoms' characteristics and interindividual differences. Death Stud. 2021;45(3):226-37.
79. Hizo-Abes P, Siegel L, Schreier G. Exploring attitudes toward physician-assisted death in patients with life-limiting illnesses with varying experiences of palliative care: a pilot study. BMC Palliative Care. 2018;17(1):56.
80. Ohnsorge K, Gudat H, Rehmann-Sutter C. What a wish to die can mean: reasons, meanings and functions of wishes to die, reported from 30 qualitative case studies of terminally ill cancer patients in palliative care. BMC Palliat Care. 2014;13:38.
81. Ruijs CDM, van der Wal G, Kerkhof AJFM, Onwuteaka-Philipsen BD. Unbearable suffering and requests for euthanasia prospectively studied in end-of-life cancer patients in primary care. BMC Palliative Care. 2014;13(1):62.
82. Gómez-Vírseda C, Gastmans C. Euthanasia in persons with advanced dementia: a dignity-enhancing care approach. J Med Ethics. 2021.
83. Hale DB. A pretty pass: when is there a right to die? Common Law World Rev. 2003;32(1):1-14.
84. Cheung G, Sundram F. Who are the elderly who want to end their lives? [References]. Rational suicide in the elderly: Clinical, ethical, and sociocultural aspects. Cham, Switzerland: Springer International Publishing/Springer Nature; Switzerland; 2017. p. 113-28.
85. Dees MK, Vernooij-Dassen MJ, Dekkers WJ, Vissers KC, van Weel C. 'Unbearable suffering': A qualitative study on the perspectives of patients who request assistance in dying. Journal of Medical Ethics: Journal of the Institute of Medical Ethics. 2011;37(12):727-34.
86. Fontalis A, Prousali E, Kulkarni K. Euthanasia and assisted dying: what is the current position and what are the key arguments informing the debate? Journal of the Royal Society of Medicine. 2018;111(11):407-13.
87. Ganzini L, Dobscha SK, Heintz RT, Press N. Oregon physicians' perceptions of patients who request assisted suicide and their families. Journal of Palliative Medicine. 2003;6(3):381-90.
88. Karlsson M, Milberg A, Strang P. Dying cancer patients' own opinions on euthanasia: An expression of autonomy? A qualitative study. Palliative Medicine. 2012;26(1):34-42.
89. Gamester N, Van den Eynden B. The relationship between palliative care and legalized euthanasia in Belgium. Journal of Palliative Medicine. 2009;12(7):589-91.
90. Rodriquez E. The arguments for euthanasia and physician-assisted suicide: ethical reflection. Linacre Q. 2001;68(3):251-61.
91. Abrahm JL. Patient and family requests for hastened death. Hematology Am Soc Hematol Educ Program. 2008:475-80.

For peer review only

92. Gandsman A. Paradox of choice and the illusion of autonomy: The construction of ethical subjects in right-to-die activism. Death Studies. 2018;42(5):329-35.
93. Granda-Cameron C, Houldin A. Concept Analysis of Good Death in Terminally Ill Patients. American Journal of Hospice & Palliative Medicine. 2012;29(8):632-9.
94. Heintz AP. Euthanasia--or, death on request. J Psychosom Obstet Gynaecol. 2002;23(2):73-5.
95. Jiraphan A, Pitanupong J. General population-based study on preferences towards end-of-life care in Southern Thailand: a cross-sectional survey. BMC Palliative Care. 2022;21(1):36.
96. Dyer C. Two men plead with judges to let doctors end their life legally. BMJ: British Medical Journal (Clinical Research Edition). 2012;344:e4270-e.
97. Castelli Dransart DA, Lapierre S, Erlangsen A, Canetto SS, Heisel M, Draper B, et al. A systematic review of older adults' request for or attitude toward euthanasia or assisted-suicide. Aging & Mental Health. 2021;25(3):420-30.
98. Achille MA, Ogloff JR. Attitudes toward and desire for assisted suicide among persons with amyotrophic lateral sclerosis. Omega: Journal of Death and Dying. 2003;48(1):1-21.
99. Right to die--right or wrong? Lancet Neurol. 2003;2(10):583.
100. Friend ML. Physician-Assisted Suicide: Death With Dignity? Journal of Nursing Law. 2011;14(3/4):110-6.
101. Drury B. The doctors of mercy. Men's Health. 2006;21(8):180-7.
102. Wilson KG, Scott JF, Graham ID, Kozak JF, Chater S, Viola RA, et al. Attitudes of terminally ill patients toward euthanasia and physician-assisted suicide. Archives of Internal Medicine. 2000;160(16):2454-60.
103. Reid T. Reflections from a provider of medical assistance in dying. Canadian Family Physician. 2018;64(9):639-40.
104. Asai A, Ohnishi M, Nagata SK, Tanida N, Yamazaki Y. Doctors' and nurses' attitudes towards and experiences of voluntary euthanasia: survey of members of the Japanese Association of Palliative Medicine. J Med Ethics. 2001;27(5):324-30.
105. Eastaugh A. Choosing to die. Br J Gen Pract. 2012;62(595):96-7.
106. Salladay SA. Christian ethics. Death with dignity? Journal of Christian Nursing. 2010;27(3):232-.
107. Broom A. On euthanasia, resistance, and redemption: The moralities and politics of a hospice. Qualitative Health Research. 2012;22(2):226-37.
108. Mentzelopoulos SD, Haywood K, Cariou A, Mantzanas M, Bossaert L. Evolution of medical ethics in resuscitation and end of life. Trends in Anaesthesia and Critical Care. 2016;10:7-14.
109. Raus K, Sterckx S. Euthanasia for mental suffering. New directions in the ethics of assisted suicide and euthanasia. Cham, Switzerland: Springer International Publishing/Springer Nature; Switzerland; 2015. p. 79-96.
110. Robinson V, Clarke J, George R. Euthanasia: A caricature of care, not a cure for suffering. British Journal of Neuroscience Nursing. 2007;3(10):457-60.
111. Stephenson J. Assisted dying: a palliative care physician's view. Clin Med (Lond). 2006;6(4):374-7.
112. Finlay IG. Quality of life to the end. Commun Med. 2005;2(1):91-5.
113. Feigin S, Owens RG, Goodyear-Smith F. Helping a loved one die: the act of assisted dying in New Zealand. Mortality. 2019;24(1):95-110.
114. Dyer C. Dying woman loses her battle for assisted suicide. Bmj. 2002;324(7345):1055.

For peer review only

115. Fournier RR. Responses to life after death with dignity: the Oregon experience. Soc Work. 2000;45(5):467-8.
116. Comby M, Filbet M. The demand for euthanasia in palliative care units: A prospective study in seven units of the 'Rhone-Alpes' region. Palliative Medicine. 2005;19(8):587-93.
117. Simpson E. Harms to dignity, bioethics, and the scope of biolaw. Journal of Palliative Care. 2004;20(3):185-92.
118. Ganzini L, Back A. From the USA: Understanding requests for physician-assisted death. Palliative Medicine. 2003;17(2):113-4.
119. Gentzler J. What is a death with dignity? J Med Philos. 2003;28(4):461-87.
120. Robinson S, Kissane DW, Brooker J, Hempton C, Burney S. The Relationship Between Poor Quality of Life and Desire to Hasten Death: A Multiple Mediation Model Examining the Contributions of Depression, Demoralization, Loss of Control, and Low Self-worth. J Pain Symptom Manage. 2017;53(2):243-9.
121. Clark D. Cultural considerations in planning palliative and end of life care. Palliat Med. 2012;26(3):195-6.
122. Oczkowski SJW, Crawshaw DE, Austin P, Versluis D, Kalles-Chan G, Kekewich M, et al. How can we improve the experiences of patients and families who request medical assistance in dying? A multi-centre qualitative study. BMC Palliative Care. 2021;20(1):185.
123. Ong RSR, Wong RSM, Chee RCH, Quek CWN, Burla N, Loh CYL, et al. A systematic scoping review moral distress amongst medical students. BMC Medical Education. 2022;22(1):466.
124. Chua KZY, Quah ELY, Lim YX, Goh CK, Lim J, Wan DWJ, et al. A systematic scoping review on patients' perceptions of dignity. BMC Palliative Care. 2022;21(1):118.
125. Ho CY, Lim NA, Ong YT, Lee ASI, Chiam M, Gek GPL, et al. The impact of death and dying on the personhood of senior nurses at the National Cancer Centre Singapore (NCCS): a qualitative study. BMC Palliat Care. 2022;21(1):83.
126. Chiam M, Ho CY, Quah E, Chua KZY, Ng CWH, Lim EG, et al. Changing self-concept in the time of COVID-19: a close look at physician reflections on social media. Philos Ethics Humanit Med. 2022;17(1):1.
127. Chan NPX, Chia JL, Ho CY, Ngiam LXL, Kuek JTY, Ahmad Kamal NHB, et al. Extending the Ring Theory of Personhood to the Care of Dying Patients in Intensive Care Units. Asian Bioeth Rev. 2022;14(1):71-86.
128. Huang H, Toh RQE, Chiang CLL, Thenpandiyan AA, Vig PS, Lee RWL, et al. Impact of Dying Neonates on Doctors' and Nurses' Personhood: A Systematic Scoping Review. J Pain Symptom Manage. 2022;63(1):e59-e74.
129. Vig PS, Lim JY, Lee RWL, Huang H, Tan XH, Lim WQ, et al. Parental bereavement – impact of death of neonates and children under 12 years on personhood of parents: a systematic scoping review. BMC Palliative Care. 2021;20(1):136.
130. Ho CY, Kow CS, Chia CHJ, Low JY, Lai YHM, Lauw S-K, et al. The impact of death and dying on the personhood of medical students: a systematic scoping review. BMC Medical Education. 2020;20(1):516.
131. Haig A, Dozier M. BEME Guide no 3: systematic searching for evidence in medical education--Part 1: Sources of information. Medical teacher. 2003;25(4):352-63.
132. Gordon M, Gibbs T. STORIES statement: publication standards for healthcare education evidence synthesis. BMC medicine. 2014;12(1):143.

For peer review only

Appendices

Appendix B. Tabulated Summaries.

No.	Title	Author/year	MERSQI	COREQ	Reason for inclusion			Undergraduate (UG)/Postgraduate (PG)	Methodology	Purpose of study	Key findings
					Communication	Ethics	Professionalism				
1	The CanMEDS resume: a useful educational portfolio tool for diagnostic radiology residents	Finlay, K.<br>Probyn, L.<br>Ho, S.<br><br>2012			✓		✓	PG	We developed a comprehensive resident CanMEDS resume for ongoing documentation of resident participation, education, and developing competencies in CanMEDS Roles for diagnostic radiology.<br>This resume or portfolio summary is structured to high- light all 7 CanMEDS Roles. The resident is responsible for ongoing updates and submits this as part of his or her annual evaluation.	The objective of this article is to discuss the utility of this tool for inclusion in the educational portfolio for radiology residents.	The document increases awareness of the CanMEDS Roles and documents resident professional development, while also promoting reflection on the impor- tance of these Roles for radiology and future practice. The added advantage for program directors and assistants is up- to-date documentation of resident activities, which serves as a highly useful tool, particularly for program accreditation purposes.
2	A pilot evaluation of portfolios for quality attestation of clinical ethics consultants	Fins, Joseph J<br>Kodish, Eric<br>Cohn, Felicia<br>Danis, Marion<br>Derse, Arthur R<br>Dubler, Nancy<br>Neveloff<br>Goulden, Barbara<br>Kuczewski, Mark<br>Mercer, Mary Beth<br>Pearlman, Robert A<br><br>2016	7.5	14		✓		PG	The QAPTF committee decided on the key components of the portfolio, their respective weightages and scoring quality of each element.<br>ASBH issued an open call to individuals practicing CEC, publicized on the ASBH website, for let- ters of intent (LOI) to submit portfolios for the piloting of this first step in a quality attestation process. The QAPTF wanted to ensure a	We describe the results of a pilot project utilizing portfolios as an evaluation tool. The goals of this quality attestation pilot project were to assess the feasibility of a process to evaluate whether CE consultants are competent to perform ethics consultations as a lead or solo consultant and to develop quality metrics for this assessment process.	We found that this approach is feasible and resulted in a reasonably wide distribution of scores among the 23 submitted portfolios that we evaluated. We discuss limitations and implications of these results, and suggest that this is a significant step on the pathway to an eventual certification process for clinical ethics consultants.

For peer review only

No.	Title	Author/year	MERSQI	COREQ	Reason for inclusion			Undergraduate (UG)/Postgraduate (PG)	Methodology	Purpose of study	Key findings
					Communication	Ethics	Professionalism				
									representative distribution of candidates for the first portfolio review and adopted a method for select- ing portfolios that was intentionally designed to minimize bias.Candidates needed to have performed CEC, have at least a master's degree in a relevant discipline, and have sufficient CEC experience to be able to provide all ele- ments of the portfolio. 32 out of 82 individuals were invited to submit portfo- lios. After candidates submitted their portfolios, they were surveyed to obtain feedback on their portfolio develop- ment and submission experience. QAPTF members were also asked about their experi- ence and perceptions of whether the portfolio elements and approach to scoring functioned effectively in identify- ing candidates who should or should not proceed with the next step of		

For peer review only

No.	Title	Author/year	MERSQI	COREQ	Reason for inclusion			Undergraduate (UG)/Postgraduate (PG)	Methodology	Purpose of study	Key findings
					Communication	Ethics	Professionalism				
									quality attestation. They also were asked about ways to improve future attestation activities. This feedback is reflected in the limitations and discussion sections that follow.		
3	Portfolios and 'the competent clinician': past, present and future assessment	Gavriel, J.<br>2016	-	-			✓	PG	NA	To describe the evolution of portfolios, including its advantages, challenges and the future of portfolios	Portfolios have been a part of medical education since the early 1990s and many educators use them frequently, even habitually. Portfolios are primarily based on a constructivist approach to learning and there are also strong links with adult learning principles.Competence based education, once under fire for being too simplistic, now forms a significant part of medical education including the development of portfolios.<br>In the med- ical education arena there are some advantages that are particularly relevant: triangulation amongst those working alongside learners, developing wider professional skills and demonstrating the potential for lifelong learning and excellence.<br>For some learners and educators the portfolio is a burden and a frustration: the volume of work, a hoop jumping approach and a subjective nature all providing challenges [8] that are at least partially a result of some key complex- ities of the portfolio assessment.

For peer review only

No.	Title	Author/year	MERSQI	COREQ	Reason for inclusion			Undergraduate (UG)/Postgraduate (PG)	Methodology	Purpose of study	Key findings
					Communication	Ethics	Professionalism				
											Portfolios are evolving assessments; they are subject to continuing developments but the ongoing challenge for portfolio designers is the balance between reliability and validity on one hand and tick-boxing on the other.
4	Diffusion of an e-portfolio to assist in the self-directed learning of physicians: An exploratory study	Goliath, Cheryl Lynn<br><br>2010	13	21	✓			PG	A single case study comprised of nine physicians was used to examine the factors involved when the diffusion of an e-portfolio was introduced. The participants were studied using interviews, the Stages of Concern (SoCQ) and the Self-Directed Learning Readiness Scale (SDLRS) questionnaires, four-week trial period of I-Webfolio and field notes based upon observations during the study.	This study examined the impact of implementing an e-portfolio system within an Internal Medicine physician practice as a means of developing a process of life-long learning and professional development for the participants.	The results were analyzed to identify patterns or themes that appeared. One such pattern emerged, in that; all entries in the e-portfolio were almost exclusively in the Medical Knowledge component. A theme that became apparent was that none of the physicians in the study have developed a self-directed learning plan. The results of this study suggested that adoption of technology is multifactoral and there was no consistent individual profile that predicted optimal adoption of the innovation; however, increased scores on the SDLRS would indicate that the diffusion of I-Webfolio was effective in improving the self-directed learning of the participants.
5	The attributes of a good medical trainee: how to build your portfolio	Joseph, S. R.<br>Sikkel, M. B.<br><br>2009	-	-	✓			PG	-	Review of the attributes of a good medical trainee based on the essential physician competencies, and how then to build a good portfolio	A comprehensive and contemporaneously completed procedural, operative, teaching and reflective<br>practice logs should be included in the portfolio, and should be commenced as soon as possible. Compose your portfolio around the seven<br>key roles of the Canadian Medical<br>Education Directives for Specialists model,<br>and be innovative when

For peer review only

No.	Title	Author/year	MERSQI	COREQ	Reason for inclusion			Undergraduate (UG)/Postgraduate (PG)	Methodology	Purpose of study	Key findings
					Communication	Ethics	Professionalism				
											filling each section with concrete examples. Use reflective practice of clinical cases also as further evidence.
6	Study skills course in medical education for postgraduate residents	Bhattarai, M. D. 2007	-	-	✓			PG	-	The learners have to take active parts in the teaching learning activities. To make them aware and to help them develop the skills required, the need of the study skills course in medical education early in the part of their training has been realized for the postgraduate residents	The important areas of the study skills course focusing in the requirement of clinical components of the postgraduate residents are 1.) Interpersonal and communications skills, 2.) Teaching, learning and presentation skills, 3.) Language, reading and computer use, 4.) Evidence based medicine and diagnosis and management, 5). Assessment principles and strategies, 6). Time management strategies to get the best out of the training, 7). Reflection, portfolio and self-directed lifelong learning, and 8). Follow-up presentation. The methodologies that could be used in such study skills course are interactive lectures, brainstorming, presentations by the trainees, demonstration to and by the trainees, small group discussion, group work and presentation, group and individual feedback, practice sessions, role play, short relevant video movies, video recording of the trainees and viewing with feedback. With their already tight training schedule and posting and other similar other mandatory courses required for the postgraduate residents, much time cannot be allocated for the study skills course in medical education alone. Similar study skills course in medical education

For peer review only

No.	Title	Author/year	MERSQI	COREQ	Reason for inclusion			Undergraduate (UG)/Postgraduate (PG)	Methodology	Purpose of study	Key findings
					Communication	Ethics	Professionalism				
											may need to be arranged for the undergraduate medical students as well.
7	Fellows' perceptions of a mandatory reflective electronic portfolio in a geriatric medicine fellowship program	Ruiz, Jorge G. Qadri, Syeda S. Karides, Marina Castillo, Carmen Milanez, Marcos Roos, Bernard A. 2009	9	15	✓		✓	PG	We conducted surveys and focus groups followed by quantitative and qualitative data analysis	Electronic portfolios (ePortfolios) can be useful for evaluating and documenting mastery of competencies. We investigated geriatric medicine fellows' perceptions of an ePortfolio	Our study revealed that fellows considered the ePortfolio acceptable and accessible. Fellows reflected on aspects relevant to postgraduate training and future practice and documented adherence to competencies. However, fellows expressed resentment about the self-reflection component, which interfered with the overall acceptance of the ePortfolio. Although ePortfolios can be powerful and reflective tools for competency assessment, care must be taken in their implementation.
8	Portfolios for assessment and learning: Guide Supplement 45.1 - Viewpoint	Burch, Vanessa 2011	-	-		✓		PG	NA	NA	The critical question to ask then is Should poorly resourced countries not find ways of adapting this valuable learning and assessment strategy to suit local realities? (Spandel 1997). Clearly, the 'Rolls Royce' version of portfolio implementation and assessment is beyond the reach of developing countries but a less resource-intensive strategy could be a feasible option. It was with this goal in mind that the University of Cape Town (UCT) in South Africa embarked on the task of adapting portfolios for use in less well-resourced settings (Burch & Seggie 2008). Medical students in a 6-year programme are introduced to portfolio-based learning and assessment in their third year of training and each subsequent year until graduation. Currently, clinical students write up,

For peer review only - http://bmjopen.bmj.com/site/about/guidelines.xhtml

No.	Title	Author/year	MERSQI	COREQ	Reason for inclusion			Undergraduate (UG)/Postgraduate (PG)	Methodology	Purpose of study	Key findings
					Communication	Ethics	Professionalism				
											and manage under supervision, between 18 and 32 cases in 6- to 10-week medicine clerkships These patients are presented to a specialist in training on admission and the attending consultant on the post-intake ward the following morning. This provides ample opportunity for students to look back on the action, reflect on their performance and receive feedback and mentoring from senior clinicians directly in the workplace setting. In this way, consultants and specialists in training act as mentors helping students identify alternative strategies and set goals to stimulate further learning. These distinct phases of the workplace-based mentoring process closely reflect the phases of spiral professional development described by Korthagen et al. (2001). The importance of this approach is that mentors provide ongoing guidance for students during the course of performing their routine clinical duties limiting the need for additional regular meetings between students and mentors. Students who are struggling are free to consult the course convener for assistance, but an introductory lecture clearly outlining the purpose, format and examination of portfolios, as well as a set of guidelines for completing portfolio tasks has significantly limited the need for further advice. In addition to writing up a series of patient case studies, students also

For peer review only

No.	Title	Author/year	MERSQI	COREQ	Reason for inclusion			Undergraduate (UG)/Postgraduate (PG)	Methodology	Purpose of study	Key findings
					Communication	Ethics	Professionalism				
											identify at least one clinical learning need based on each case discussion; these ‘question and answer’ tasks are also included in the portfolio. This provides a good opportunity for needs-driven, focused, self-directed learning. Students also complete a range of other written tasks using the index clinical cases in their portfolios. These case-based tasks (Harden et al. 2000) include written reflections on ethical issues relevant to selected patients, a bio-psycho-social analysis (mini-ethnography) (Kleinman & Benson 2006) of selected patients and the use of case material to explore the principles of Primary Health Care (Vivian et al. 2010) as they relate to patients in the portfolio. Finally, students also write up detailed treatment plans for a list of 32 common conditions as reflected by the cases in the portfolio. These tasks are all designed to promote self-directed, patient-centred learning. At UCT it was recognised that marking of this volume of portfolio material was beyond the human resources capacity at departmental level and so a strategic decision was made to focus on an assessment strategy based on a series of structured interviews, focusing on different aspects of a sample of portfolio cases. Students rotate through four stations of 15 min each (OSCE style) at which single-examiner structured interviews are conducted based

For peer review only

No.	Title	Author/year	MERSQI	COREQ	Reason for inclusion			Undergraduate (UG)/Postgraduate (PG)	Methodology	Purpose of study	Key findings
					Communication	Ethics	Professionalism				
											on patient cases randomly selected from the portfolio. The case-based discussions focus on clinical reasoning, case management plans, bioethics, psychosocial aspects of illness and the principles of Primary Health Care. This approach assesses student performance on a 'biopsy' of at least 8–10 cases contained in the portfolio. Each case discussion is scored using a nine-point rubric (1–3 poor, 4–6 adequate, 7–9 good) and scores are totalled across all cases discussed to achieve an aggregate score for the examination. The key reasons for adopting this assessment strategy at UCT included limited human resources but, more importantly, the observation that English second language speakers, more than 50% of university admissions nationally (Council on Higher Education 2009), are better able to express themselves in oral rather than written form. Indeed, it has been asked whether portfolios provide educators with real insight into practitioners' clinical ability or simply show that they are good at writing about what they do? (Karlowicz 2000). Having developed the strategy, the key question is whether any other university in sub-Saharan Africa has successfully adopted it. To date, two other South African universities have adopted and adapted the approach to suit local needs and two other African universities have

For peer review only

No.	Title	Author/year	MERSQI	COREQ	Reason for inclusion			Undergraduate (UG)/Postgraduate (PG)	Methodology	Purpose of study	Key findings
					Communication	Ethics	Professionalism				
											successfully adopted the strategy at Makerere University (Mubuuke et al. 2010) and Kampala International University (Ezeala et al. 2010) both in Uganda.<br>Clearly, the data are limited but it does suggest that portfolio-based learning and assessment are within reach of countries attempting to improve the quality of health sciences education using available resources. This, I believe, is one of the most important issues to be addressed in the twenty-first century – the adaptation and implementation of better teaching and assessment strategies in developing countries so as to keep abreast of educational advances developed world countries enjoy.
9	AMEE Guide No. 25: The assessment of learning outcomes for the competent and reflective physician	Shumway, J. M. Harden, R. M.<br><br>2003	-	-	✓	✓	✓	PG	-	To emphasize on the 2 important features of contemporary medical education: first is an emphasis on assessment as a tool to ensure quality in training programmes, to motivate students and to direct what they learn. The second is a move to outcome-based education where the learning outcomes are defined and decisions about the curriculum are based on these.	Portfolio assessments have a positive impact on the learning environment because they document what the learner has done and ask the learner to reflect on what she/he has accomplished. They ask the learner to put together large amounts of disparate information into a whole that tells a story about the learner's accomplishments. Portfolios are a valuable instru- ment for inclusion in the examiner's toolkit, if for no other reason than that they assess learning outcomes such as critical thinking and self-assessment, which are not easily assessed using other instruments. They also provide a record of the

For peer review only

No.	Title	Author/year	MERSQI	COREQ	Reason for inclusion			Undergraduate (UG)/Postgraduate (PG)	Methodology	Purpose of study	Key findings
					Communication	Ethics	Professionalism				
											student's performance over a period of time and are not just a snapshot at one specific point in time. The reliability of portfolios is at least in part due to the ability of raters to agree on standards and criteria for the content and assessment of portfolios.
10	A comprehensive collaborative patient safety residency curriculum to address the ACGME core competencies	Singh, R.<br>Naughton, B.<br>Taylor, J. S.<br>Koenigsberg, M. R.<br>Anderson, D. R.<br>McCausland, L. L.<br>Wahler, R. G.<br>Robinson, A.<br>Singh, G.<br><br>2005	-	-	✓			PG	-	To design and implement a new patient safety curriculum in collaboration with the Schools of Nursing and Pharmacy, in such a way as to address all 6 competencies.	Porfolios is used as one of the multiple assessment approaches in the patient safety programme. However, no evaluation made on this assessment mode.
11	ECLIPPx: an innovative model for reflective portfolios in life-long learning	Cheung, C. R.<br><br>2011	-	-			✓	PG	-	For healthcare professionals, the educational portfolio is the most widely used component of lifelong learning – a vital aspect of modern medical practice. When used effectively, portfolios provide evidence of continuous learning and promote reflective practice. But traditional portfolio models are in danger of becoming outmoded, in the face of changing expectations of health- care provider competences today.<br><br>Portfolios in health care have generally focused on competencies in clinical skills. How-ever, many other domains of professional	A new model for categorising the health care port- folios of professionals is proposed. The ECLIPPx model is based on personal practice, and divides the evidence of ongoing professional learning into four categories: educational development; clinical practice; leadership, innovation and professionalism; and personal experience.<br><br>The ECLIPPx model offers a new approach for personal reflection and longitudinal learn- ing, one that gives flexibility to the user whilst simultaneously encompassing the many relatively new areas of competence and expertise that are now required of a modern doctor.

For peer review only

No.	Title	Author/year	MERSQI	COREQ	Reason for inclusion			Undergraduate (UG)/Postgraduate (PG)	Methodology	Purpose of study	Key findings
					Communication	Ethics	Professionalism				
										development, such as professionalism and leadership skills, are increasingly important for doctors and health care professionals, and must be addressed in amassing evidence for training and revalidation. There is a need for modern health care learning portfolios to reflect this sea change.	
12	Development of a web-based, specialty specific portfolio	Clay, A. S., Petrusa, E., Harker, M. and Andolsek, K.<br><br>2007	-	-	✓	✓	✓	PG	Three different disciplines (anesthesiology, surgery and medicine) worked together to create a critical care medicine portfolio. We began by reviewing the curriculum requirements for critical care medicine and organise<br><br>d these requirements into the six ACGME core competencies. We then developed learner led exercises in each core competency that were specific to critical care. Each exercise includes assessment of resident knowledge and application, an evaluation of the exercise, a learner self-	This article illustrates the creation of a specialty specific portfolio that can be used by several different residency programs to document resident competence during a given rotation	Creation of specialty specific portfolio reduces redundancy between disciplines, allows for increased time to be spent on the development of exercises specific to rotation objectives, and aids program directors in the collection of portfolio entries for each resident over the course of a residency.<br><br>We describe the creation of a multidisciplinary learning portfolio that can be used by different training programs within one institution to assess trainee performance in each respective area of expertise. The strengths of the specialty specific portfolio include the ability to be specific to the learning objectives of that rotation/specialty and uniform in content across multiple disciplines within that specialty. Commitment to the portfolio from multiple disciplines allows for careful refining of the portfolio and reduction of duplicative efforts. These specialty specific portfolios could potentially be improved upon if professional

No.	Title	Author/year	MERSQI	COREQ	Reason for inclusion			Undergraduate (UG)/Postgraduate (PG)	Methodology	Purpose of study	Key findings
					Communication	Ethics	Professionalism				
									assessment of skill, and a review of performance by a faculty member. Portfolio entries are highlighted in a multi-disciplinary weekly conference and posted on a critical care web site at our University.		organizations were committed to their adaptation, perhaps as a component of maintenance of certification. The portfolio entries as we have described are also useful for prompting frequent self reflection/evaluation and for providing trainees with feedback on their self-perceptions. Self assessment, a skill which is not often taught or evaluated in medical education, is notoriously inaccurate (Gordon 1991). However, self assessment may be improved with deliberate practice, and frequent self assessment with feedback on that self assessment (Gordon 1992; Norman & Schmidt 1992; Ericsson 2004). If other specialties were to create similar portfolios, the logistics of creating a portfolio for each resident would be simplified: the portfolio could be built a rotation at a time, meeting the objectives of that rotation monthly, and meeting the ACGME requirements over the course of a residency. Furthermore, by simply requiring a certain number entries from each core competency by the end of each year or the end of a residency, the learner is given the discretion to choose those exercise which he/she feels is most relevant given the cases he/she has encountered.
13	A review of portfolio use in residency programs and	Colbert, C. Y., Ownby, A. R. and Butler, P. M.	-	-			✓	PG	Two searches of PubMed, OVID, JSTOR, SCOPUS, and	Portfolios, often described as collections of evi-dence, are discussed	There is wide varia- tion in how portfolios are utilized within U.S. residency programs. The challenge for

For peer review only

No.	Title	Author/year	MERSQI	COREQ	Reason for inclusion			Undergraduate (UG)/Postgraduate (PG)	Methodology	Purpose of study	Key findings
					Communication	Ethics	Professionalism				
	considerations before implementation	2008							FirstSearch Wilson Select were conducted between October 2006 and April 2007 to identify studies and articles related to portfolio usage. Thirty-nine articles met criteria and were reviewed.	as a means of teaching or assessing the Ac- creditation Council for Graduate Medical Education competencies. Yet, it is unclear how many residency programs utilize portfolios. The purpose of this article is to (a) review the literature on portfolio use in graduate medical education; (b) examine efficacy of portfolio use, based upon studies in the field; and (c) offer a discussion of con- siderations for implementing portfolios.	graduate medical education is to create consensus on the definition and purpose of portfolios, such that best practices in portfolio implementation and assessment can be achieved.<br><br>The case for utilizing portfolios in residency education is compelling. Portfolios can offer a diverse palette of learners' achievements and experiences. When assessing competence from a holistic standpoint, the range of behaviors captured in a portfolio may render it superior to more traditional assessment practices. Self-awareness and self-directed learning, thought to be critical to the development of competent physicians, can be enhanced through the act of portfolio development,19 as port- folios can force one to reflect upon strengths, weaknesses, and opportunities for growth.<br><br>Before implementing a portfolio system in a residency pro- gram, several issues need to be considered. Learners and faculty should be trained on the portfolio process including the purpose, structure, and content of portfolios. Mentoring may be required when it comes to content assembly and interpretation. If portfolios are to be used for summative assessment pur- poses, an evaluation rubric—a scoring template—will need to be

For peer review only

No.	Title	Author/year	MERSQI	COREQ	Reason for inclusion			Undergraduate (UG)/Postgraduate (PG)	Methodology	Purpose of study	Key findings
					Communication	Ethics	Professionalism				
											created.<br><br>Last, the promise offered by portfolios is tempered by diffi-culties associated with their use. The lack of a standard defini- tion across the field and the dearth of outcome studies to assess efficacy are pressing concerns. The challenge now is to create consensus on the definition and purpose of portfolios within res- idency programs, such that collaboration within the field may begin to offer best practices in the areas of implementation and assessment.
14	How we teach ethics and communication during a Canadian neonatal perinatal medicine residency: an interactive experience	Daboval, T., Moore, G. P. and Ferretti, E.<br><br>2013	6.5		✓	✓	✓	PG	A revised ethics program implemented in 2009 identified competencies that should be demonstrated at the end of the Neonatal-Perinatal Medicine (NPM) residency. Several seminars were refined while new workshops, problem-based learning in ethics, and a personal portfolio were added.	This article describes the process by which a well-delineated, interactive program to teach ethical reasoning and skillful communication with parents was implemented at the University of Ottawa, Canada.	All teaching strategies were well received based on the average level of satisfaction (5.8 out of 7, SD 0.4). We are now moving forward by formally assessing our program including the impact on knowledge acquisition and behavior.<br><br>A dedicated, interactive competency-based neonatal ethics teaching program is vital to support NPM trainees in learning how to integrate ethical thinking with competencies in communication.<br><br>A dedicated, interactive, competency-based neonatal ethics teaching program with well-identified expectations and eval- uation is vital so that all graduating NPM trainees will not only have knowledge of key ethical precepts and ethical reasoning, but will also have learned how to integrate ethical thinking when they communicate and interact

For peer review only

No.	Title	Author/year	MERSQI	COREQ	Reason for inclusion			Undergraduate (UG)/Postgraduate (PG)	Methodology	Purpose of study	Key findings
					Communication	Ethics	Professionalism				
											professionally with parents during ethically and emotionally charged clinical situations. In this manner, our teaching is supporting a paradigm shift in neonatology from a paternalistic to a shared decision-making model. To advance our methods of education in this area, formal assessments of such teaching programs must occur.
15	Portfolio as a tool to stimulate teachers' reflections	Tigelaar, D. E.<br>Dolmans, D. H.<br>de Grave, W. S.<br>Wolfhagen, I. H.<br>van der Vleuten, C. P.<br><br>2006	-	15	✓			PG	In this study, all the written portfolio assignments were approached as reflections on teacher functioning. In order to gain insight into the portfolio process, we collected the teachers' final portfolio assignments as well as data about the process of portfolio construction. In this way, we obtained portfolio data comprising written portfolio assignments and final reflective portfolio assignments. Firstly, the teachers were asked to describe the course of their teaching career. They were also asked to describe critical incidents in their development as a teacher, set learning goals,	Portfolios are increasingly being used to stimulate teachers' reflections. Frameworks for reflection on teaching often emphasize competencies and behaviours. However, other aspects of teacher functioning are also important, such as the teaching environment and individual teachers' beliefs, professional identity and mission. In a study among five medical school teachers, we explored how a portfolio stimulated reflections on the various aspects of teaching functioning.	The analysis yielded examples of reflections on all aspects of Korthagen's model, although reflections on competencies proved easiest to identify.

For peer review only - http://bmjopen.bmj.com/site/about/guidelines.xhtml

No.	Title	Author/year	MERSQI	COREQ	Reason for inclusion			Undergraduate (UG)/Postgraduate (PG)	Methodology	Purpose of study	Key findings
					Communication	Ethics	Professionalism				
									select artefacts in evidence of their teaching activities, compose a profile of a good teacher and reflect on that. The final assign- ment required them to compose a reflective portfolio, using the previous assignments as points of departure. The assignments were intended to stimulate reflection on all aspects of teacher functioning as defined in Korthagen's model. However, the critical incidents assignment was focused on the environment, behaviour, and competencies. Table 1 presents brief descriptions of the assignments.		
16	A method for defining competency-based promotion criteria for family medicine residents	Torbeck, L. Wrightson, A. S.<br><br>2005	-	-	✓		✓	PG	In 2003, both of us began the task of developing promotion criteria by searching the literature for studies identifying standards or criteria for resident promotion. Few studies were found related to residents' progress and	The Accreditation Council for Graduate Medical Education (ACGME) has mandated a shift from a structure- and process-based educational system to a competency-based system. The ACGME has not provided criteria (standards), preferring to leave that to the discretion	The value of promotion criteria<br>Training residents to become competent health care providers has been and always will be an important and challenging endeavor. With the ACGME's new mandate, many program directors and house office staff are looking for useful guidance to make their residency programs compliant. We believe that developing a

For peer review only

No.	Title	Author/year	MERSQI	COREQ	Reason for inclusion			Undergraduate (UG)/Postgraduate (PG)	Methodology	Purpose of study	Key findings
					Communication	Ethics	Professionalism				
									promotion.[2,7] We then reviewed the information contained within the last three Residency Assistance Program workshops of the American Academy of Family Physicians, 2001–2003. Frey et al.'s[8] 2003 outpatient core competency presentation notes were earmarked as having specific core competencies that could easily be rewritten as promotion criteria. Next, we reviewed the residency curriculum of the Department of Family Practice and Community Medicine at the University of Kentucky College of Medicine and listed promotion criteria that we thought were important for Family Medicine residents at each level of that curriculum to achieve. Taking this list and extrapolating from Frey et al.'s work, we composed a list of working promotion criteria.	of the individual training programs. Such criteria and an overall strong evaluation process are essential for residents to attain the appropriate knowledge, skills, and attitudes. With this need in mind, the authors describe an evaluation process in which they developed ACGME-competency-based promotion criteria for family medicine residents at the University of Kentucky College of Medicine in 2004.	set number of promotion criteria that are competency-based should be among the first tasks residency programs should tackle, for several reasons.<br><br>First, having defined criteria will make the three to seven years of residency performance assessment more educationally sound. The education literature reminds us that two components are mandatory to a performance assessment: the task and the criteria.[11,12] Since continuous performance assessment is carried out during the majority of a resident's training, the awareness of what is expected of the resident can be raised for both the resident and the faculty by making the task and criteria clearer to them. Residents then are better able to demonstrate that which is expected of them, and faculty are better prepared to evaluate them.<br><br>Second, having defined promotion criteria can better target remediation in those residents failing to meet the criteria in a timely manner. Intervention during a time early on in the resident's development is much more beneficial than it is just before making a decision to promote or not promote.<br><br>Third, having criteria can improve faculty development, especially in terms of how to assess competence, how to give feedback, and how to account for faculty

For peer review only

No.	Title	Author/year	MERSQI	COREQ	Reason for inclusion			Undergraduate (UG)/Postgraduate (PG)	Methodology	Purpose of study	Key findings
					Communication	Ethics	Professionalism				
											responsibility in training and evaluating competent physicians.
17	Enhanced skills in global health and health equity: Guidelines for curriculum development	Dawe, R., Pike, A., Kidd, M., Janakiram, P., Nicolle, E. and Allison, J.<br><br>2017	10	10		✓		PG	A panel comprised of 34 experts in global health education and practice completed three rounds of a Delphi small group process.	To generate consensus on the essential components of a Global Health/Health Equity Enhanced Skills Program in family medicine.	Consensus (defined as ≥ 75% agreement) was achieved on program length (12 months), inclusion of both domestic and international components, importance of mentorship, methods of learner assessment (in-training evaluation report, portfolio), four program objectives (advocacy, sustainability, social justice, and an inclusive view of global health), importance of core content, and six specific core topics (social determinants of health, principles and ethics of health equity/global health, cultural humility and competency, pre and post-departure training, health systems, policy, and advocacy for change, and community engagement).<br><br>Panellists agreed on a number of program components forming the initial foundation for an evidence- informed, competency-based Global Health/Health Equity Enhanced Skills Program in family medicine.
18	Learning portfolios in radiology residency education: how do I get started?	Deitte, L.<br><br>2008	-	-	✓		✓	PG	-	The purpose of this article is three-fold: (1) to famil- iarize the reader with learning portfolios in medical edu-cation, (2) to outline the new radiology ACGME re-quirements for a portfolio, and (53) to discuss strategies for the implementation of a portfolio as a learning and	As medical education and continuous professional devel- opment shift toward an outcomes-based model, there is increasing focus on documentation of physician self-as- sessment and a commitment to lifelong learning. The ACGME program requirements for diagnostic radiology graduate medical education effective July 1, 2008, in-clude learning portfolios as

For peer review only

No.	Title	Author/year	MERSQI	COREQ	Reason for inclusion			Undergraduate (UG)/Postgraduate (PG)	Methodology	Purpose of study	Key findings
					Communication	Ethics	Professionalism				
										assessment tool in a residency education program and continued professional development..	one method for this docu- mentation. Although there are some challenges associ- ated with the implementation of learning portfolios, it is likely that portfolios will prove to be a valuable learning and assessment tool not only in graduate medical educa- tion but also throughout a physician's career and recerti- fication process.
19	Evaluation of an established learning portfolio	Vance, G. Williamson, A. Frearson, R. O'Connor, N. Davison, J. Steele, C. Burford, B. 2013	9	-	✓			PG	Deanery-wide trainee attitudes were surveyed by an electronic questionnaire in 2009 and compared with perceptions recorded during the pilot phase (2004–2005).	The trainee-held learning portfolio is integral to the foundation programme in the UK. In the Northern Deanery, portfolio assessment is standar- dised through the Annual Review of Competence Progression (ARCP) process. In this study we aimed to establish how current trainees evaluate portfolio-based learning and ARCP, and how these attitudes may have changed since the foundation programme was first introduced.	Many trainees continue to view the e-portfolio negatively. Indeed, significantly fewer train- ees in 2009 thought that the e-portfolio was a 'good idea' or a 'worthwhile investment of time' than in 2005. Trainees remain unconvinced about the educa- tional value of the e-portfolio: fewer trainees in 2009 regarded it as a tool that might help focus on training or recognise individual strengths and weaknesses. Issues around unnecessary bureaucracy persist. Current trainees tend to understand how to use the e- portfolio, but many did not know how much, or what evidence to collect. Few supervisors were re- ported to provide useful guidance on the portfolio. ARCP encouraged portfolio completion but did not give meaningful feedback to drive future learning. Continued support is needed for both trainees and supervisors in portfolio-building skills and in using the e-portfolio as an educational tool. Trainee-tailored feedback is needed to ensure that portfolio-

For peer review only

No.	Title	Author/year	MERSQI	COREQ	Reason for inclusion			Undergraduate (UG)/Postgraduate (PG)	Methodology	Purpose of study	Key findings
					Communication	Ethics	Professionalism				
											based assessment promotes lifelong, self-directed and reflective learners. Trainees remain unconvinced about the educational value of the e-portfolio
20	Reflective Practice for Patient Benefit: An Analysis of Doctors' Appraisal Portfolios in Scotland	Wakeling, J. Holmes, S. Boyd, A. Tredinnick-Rowe, J. Cameron, N. Marshall, M. Bryce, M. Archer, J. 2019	-	16	✓			PG	Using an in-depth case study approach, 18 online portfolios in Scotland were examined with a template developed to record the types of supporting information submitted and how far these showed reflection and/or changes to practice. Data from semistructured interviews with the doctors (n = 17) and their appraisers (n = 9) were used to contextualize and broaden our understanding of the portfolios.	Reflective practice has become the cornerstone of continuing professional development for doctors, with the expectation that it helps to develop and sustain the workforce for patient benefit. Annual appraisal is mandatory for all practicing doctors in the United Kingdom as part of medical revalidation. Doctors submit a portfolio of supporting information forming the basis of their appraisal discussion where reflection on the information is mandated and evaluated by a colleague, acting as an appraiser.	Portfolios generally showed little written reflection, and most doctors were unenthusiastic about documenting reflective practice. Appraisals provided a forum for verbal reflection, which was often detailed in the appraisal summary. Portfolio examples showed that reflecting on continued professional development, audits, significant events, and colleague multisource feedback were sometimes considered to be useful. Reflecting on patient feedback was seen as less valuable because feedback tended to be uncritical. The written reflection element of educational portfolios needs to be carefully considered because it is clear that many doctors do not find it a helpful exercise. Instead, using the portfolio to record topics covered by a reflective discussion with a facilitator would not only prove more amenable to many doctors but would also allay fears of documentary evidence being used in litigation.
21	Developing an Evaluation Tool for Assessing Clinical Ethics Consultation Skills in Simulation Based	Wasson, K. Parsi, K. McCarthy, M. Siddall, V. J. Kuczewski, M. 2016	-	-		✓		PG	At the beginning of the project development, four of the authors (who are bioethicists with experience in	The American Society for Bioethics and Humanities has created a quality attestation (129) process for clinical ethics consultants;	Given the move toward quality attestation by ASBH and the need to develop a way of assessing the interpersonal skills of clinical ethics consultants ''live'' we have developed the ACES

For peer review only - http://bmjopen.bmj.com/site/about/guidelines.xhtml

No.	Title	Author/year	MERSQI	COREQ	Reason for inclusion			Undergraduate (UG)/Postgraduate (PG)	Methodology	Purpose of study	Key findings
					Communication	Ethics	Professionalism				
	Education: The ACES Project								clinical ethics consultation) agreed that any tool created should be grounded in the ASBH core competencies, as they are the articulated professional standards in the field. After a review of the limited assessment tools available, we agreed to draw from the VA's Ethics Consultant Proficiency Assessment Tool (2014). It draws on the ASBH core competencies, including the categories of Interpersonal Skills, Process Skills, Analytic Skills, and Core Knowledge, is quite comprehensive, and has been in use for a number of years. In addition, we followed Stufflebeam's (2000) guidelines for creating a checklist evaluation tool.<br><br>Given the focused usage of the ACES tool—clinical ethics simulation in an educational setting—we met regularly to	the pilot phase of reviewing portfolios has begun. One aspect of the QA process which is particularly challenging is assessing the interpersonal skills of individual clinical ethics con- sultants. We propose that using case simulation to evaluate clinical ethics consul- tants is an approach that can meet this need provided clear standards for assessment are identified. To this end, we developed the Assessing Clinical Ethics Skills (ACES) tool, which identifies and specifies specific behaviors that a clinical ethics consultant should demonstrate in an ethics case simulation. The aim is for the clinical ethics consultant or student to use a videotaped case simulation, along with the ACES tool scored by a trained rater, to demonstrate their competence as part of their QA portfolio.	tool. Our tool provides a clear structure for key domains, behaviors, and interpersonal skills that can be observed and scored by a trained rater during simulated ethics consultations. Individuals completing the ACES course or using the tool can opt to include their evaluation and video as part of their QA portfolio as a means of demonstrating their interpersonal skills in a simulated ethics consultation. Our tool also provides a framework and structure for the content of an ethics consultation and helps translate the core competencies into practical areas, behaviors, and skills. We spent a great deal of time thinking about and debating which items from the VA tool to include in the final ACES tool. It is not intended to be comprehensive for all aspects of clinical ethics consultation, e.g., for simplicity in the pilot stage we did not examine the clinical ethics consultant's ability to review and decipher the medical chart or write a note, though these elements could be added to the simulations in future. Instead, it focuses on those elements we thought could be captured in simulated clinical ethics consultations in an educational setting in this pilot phase. In the future, other elements of ethics consultation and levels of complexity can be added to the simulations as well as additional cases.

No.	Title	Author/year	MERSQI	COREQ	Reason for inclusion			Undergraduate (UG)/Postgraduate (PG)	Methodology	Purpose of study	Key findings
					Communication	Ethics	Professionalism				
									determine which items from the VA tool would be necessary for this purpose. Our tool is not meant to be a comprehensive tool to assess every element of an ethics consultation or CEC's role, thus it is briefer than the VA tool. It is also designed to be used in ''real time'' by trained bioethics raters, not retrospectively self-administered by the ethics consultant. Once we agreed upon the primary items, we met weekly for 3–4 months to identify and determine the specific behaviors and skills we would be evaluating under each main item. This was a time consuming process involving in-depth discussion and debate. Before each weekly meeting, we viewed videos of simulated ethics consultations from past graduate bioethics courses to refine our tool, its specified items, and behaviors. We discussed and		For clarity of assessment, we developed the ACES tool to be used for individual consultants. We believe each individual ethics consultant needs to have this set of skills, even if they work within a small team model in practice. If the ACES tool was adapted to assess a small team of ethics consultants, the raters would have to rate each consultant individually or rate them all as one ''ethics consultant'' to assess whether the team covered all the necessary items on the evaluation. This approach would be more complicated to assess and, we suspect, much less straightforward to score. At this stage, we do not recommend using the ACES tool in this way.<br>One of the most challenging areas in the ACES tool development was how to view and rate students in a similar way—or inter-rater agreement. We spent a great deal of time discussing what qualifies as ''Done,'' ''Not Done'' or ''Done Incorrectly'' for each and every item. Rater agreement is an ongoing process which needs to be practiced and refreshed, and is not unique to our setting. We plan to assess and recalibrate rater agreement prior to each time a course is offered (1–2 per annum). What makes it more challenging in clinical ethics is the nature of some parts of the consultation; e.g., identifying and clarifying the ethical issues. What counts as ''Done'' for certain items in

For peer review only

No.	Title	Author/year	MERSQI	COREQ	Reason for inclusion			Undergraduate (UG)/Postgraduate (PG)	Methodology	Purpose of study	Key findings
					Communication	Ethics	Professionalism				
									agreed on all of the behaviors and skills listed under each item in the ACES tool. In the end, we agreed on 12 main items and 35 corresponding behaviors to be evaluated and scored.		our tool can be interpreted differently depending on the rater's background and potential biases. This type of item— communication and interpersonal skills in ethics—is not always as neat and clear cut as in other clinical areas such as surgical preparation checklists or putting in a central line. Further work needs to be done to train additional raters if we are to use the tool with more people over time. We plan to recruit additional students to take the ACES course and are working on refining the cases used and plan to evaluate the tool in other settings to obtain feedback on its reliability. When we used the ACES tool in the basic clinical ethics consultation course the feedback was overwhelmingly positive. Students appreciated a clear articulation of what was expected in an ethics consult. While recognizing the tool was not designed to be comprehensive, they valued having a structure and framework for thinking about the elements of an ethics consultation and the behaviors they should be exhibiting. It gave them a clear standard by which to assess themselves and others.
22	Evaluating practice-based learning and improvement: efforts to improve acceptance of portfolios	Fragneto, R. Y., Dilorenzo, A. N., Schell, R. M. and Bowe, E. A.<br><br>2010	8		✓		✓	PG	Intensive education about the goals and importance of portfolios began in January 2006, including presentations at departmental conferences and	To find out whether educational efforts improved acceptance of the portfolio and retrospectively audited the portfolio evaluation forms completed by faculty advisors.	Portfolios are considered a best methods techni- que by the ACGME for evaluation of practice-based learning and improvment. We have found that intensive education about the goals and importance of portfolios can enhance acceptance of this evaluation tool, resulting

No.	Title	Author/year	MERSQI	COREQ	Reason for inclusion			Undergraduate (UG)/Postgraduate (PG)	Methodology	Purpose of study	Key findings
					Communication	Ethics	Professionalism				
									one-on-one education sessions. Faculty advisors were instructed to evaluate each resident's portfolio and complete a review form. We retrospectively collected data to determine the percentage of review forms completed by faculty. The portfolio reviews also assessed the percentage of 10 required portfolio components residents had completed.		in improved compliance in completion and evaluation of portfolios.
23	Use of portfolios for assessment of global health residents: qualitative evaluation of design and implementation	Gibson, C., Chandratilake, M. and Hull, A.<br><br>2018	-	22	✓			PG	A qualitative study was conducted with academic experts in Canadian residency training, as well as directors and residents involved in Global Health study in order to assess the validity and benefit of such a tool. Through an online survey, interviews, and focus groups, views on the portfolio and intended content were collected and coded thematically.	To determine the robustness of portfolios as an assessment tool for Global Health Residents.	This qualitative evaluation validated the use of portfolios for this cohort of students while yielding comments and suggestions that will further enhance the interactive and flexible nature of this seldom used assessment tool. These findings contribute to the understanding of how Global Health assessment can remain individualized yet rigorous.
24	Assessment and Change: An Exploration of	Jocelyn M. Lockyer, PhD; Sanjeev Sockalingam, MD,	-	16	✓			PG	A total of 5000 entries from 2195 psychiatrists were	Fellows of the Royal College of Physicians and	There were 3841 entries for analysis; 1159 entries did not meet the criteria for

For peer review only - http://bmjopen.bmj.com/site/about/guidelines.xhtml

No.	Title	Author/year	MERSQI	COREQ	Reason for inclusion			Undergraduate (UG)/Postgraduate (PG)	Methodology	Purpose of study	Key findings
					Communication	Ethics	Professionalism				
	Documented Assessment Activities and Outcomes by Canadian Psychiatrists	MHPE, FRCPC; Craig Campbell, MD, FRCPC<br><br>2018							examined. A thematic analysis drawing on the framework analysis was undertaken of the 2016 entries.	Surgeons of Canada are required to participate in assessment activities for all new 5-year cycles beginning on or after January 2014 to meet the maintenance of certification program requirements. This study examined the assessment activities which psychiatrists reported in their maintenance of certification e-portfolios to determine the types and frequency of activities reported; the resultant learning, planned learning, and/or changes to the practice they planned or implemented; and the interrelationship between the types of assessment activities, learning that was affirmed or planned, and changes planned or implemented.	assessment. The most commonly reported activities were self-assessment programs, feedback on teaching, regular performance reviews, and chart reviews. Less frequent were direct observation, peer supervision, and reviews by provincial medical regulatory authorities. In response to the data, psychiatrists affirmed that their practices were appropriate, identified gaps they intended to address, planned future learning, and/ or planned or implemented changes. The assessment activities were internally or externally initiated and resulted in no or small changes (accommodations and adjustments) or redirections.<br><br>Psychiatrists reported participating in a variety of assessment activities that resulted in variable impact on learning and change. The study underscores the need to ensure that assessments being undertaken are purposeful, relevant, and designed to enable identification of outcomes that impact practice.
25	Development of a portfolio of learning for postgraduate family medicine training in South Africa: a Delphi study	Jenkins, L., Mash, B. and Derese, A.<br><br>2012	-	10	✓	✓		PG	A workshop held at the WONCA Africa Regional Conference in 2009 explored the purpose and broad contents of the portfolio. The 85 training outcomes, ideas from the WONCA workshop, the literature, and	This study aimed to achieve a consensus on the contents and principles of the first national portfolio for use in family medicine training in South Africa.	A portfolio was developed and distributed to the eight departments of Family Medicine in South Africa, and the CMSA, to be further tested in implementation. This was the first attempt to reach consensus on the development of a national portfolio for family medicine training in South Africa. Consensus was reached on 50 items to include, and 26

For peer review only

No.	Title	Author/year	MERSQI	COREQ	Reason for inclusion			Undergraduate (UG)/Postgraduate (PG)	Methodology	Purpose of study	Key findings
					Communication	Ethics	Professionalism				
									existing portfolios in the various universities were used to develop a questionnaire that was tested for content validity by a panel of 31 experts in family medicine in South Africa, via the Delphi technique in four rounds. Eighty five content items (national learning outcomes) and 27 principles were tested. Consensus was defined as 70% agreement. For those items that the panel thought should be included, they were also asked how to provide evidence for the specific item in the portfolio, and how to assess that evidence.		principles relating to the port- folio. A draft national portfolio and portfolio guide have been developed and distributed to all the medical schools in the country. Further revision and testing with registrars in training is underway, with the aim to deli- ver a final portfolio in the following year.
26	The national portfolio of learning for postgraduate family medicine training in South Africa: experiences of registrars and supervisors in clinical practice	Jenkins, L., Mash, B. and Derese, A.<br><br>2013	-	15	✓			PG	Semi-structured interviews were conducted with 17 purposively selected registrars and supervisors from all eight South African training programmes.	The aim of this study was to explore registrars' and supervisors' experience regarding the portfolio's educational impact, acceptability, and perceived usefulness for assessment of competence.	The learning portfolio had a significant educational impact in shaping work-place based supervision and training and providing formative assessment. Its acceptability and usefulness as a learning tool should increase over time as supervisors and registrars become more competent in its use. There is a need to clarify how it will be used in summative assessment.
27	Demonstration of Portfolios to Assess	PATRICIA S. O'SULLIVAN1,* , MARK D. RECKASE2, TINA	8	12	✓		✓	PG	22 participants in this demonstration project were all	Describes assessment of the reliability and validity of portfolios in a	We must continue to emphasize some important qualities of portfolios: res- ident choice and reflection.

For peer review only

No.	Title	Author/year	MERSQI	COREQ	Reason for inclusion			Undergraduate (UG)/Postgraduate (PG)	Methodology	Purpose of study	Key findings
					Communication	Ethics	Professionalism				
	Competency of Residents	MCCLAIN3, MILDRED A. SAVIDGE1 and JAMES A. CLARDY3<br><br>2004							residents in the psychiatry residency education program at the primary affiliate of a state university medical school.	psychiatry residency program.	These are important to the life-long learner. Portfolio has been able to aid the faculty in meeting ACGME expectations and has oriented programe to the increasingly important role of evaluation.
28	Developing and Successfully Implementing a Competency-Based Portfolio Assessment System in a Postgraduate Family Medicine Residency Program	Laura A. McEwen, PhD, Jane Griffiths, MD, CCFP, FCFP, and Karen Schultz, MD, CCFP, FCFP<br><br>2015	-	-	✓			PG	-	The use of portfolios in postgraduate medical residency education to support competency development is increasing; however, the processes by which these assessment systems are designed, implemented, and maintained are emergent. The authors describe the needs assessment, development, implementation, and continuing quality improvement processes that have shaped the Portfolio Assessment Support System (PASS) used by the postgraduate family medicine program at Queen's University since 2009.	The authors discuss the identification of impact measures at the individual, programmatic, and institutional levels and the ways the department uses these to monitor how PASS supports competency development, scaffolds residents' self- regulated learning skills, and promotes professional identity formation. They describe the "academic advisor" role and provide an appendix covering the portfolio elements. Reflection elements include learning plans, clinical question logs, confidence surveys, and reflections about continuity of care and significant incidents. Learning module elements cover the required, online bioethics, global health, and consult- request modules. Assessment elements cover each resident's research project, clinical audits, presentations, objective structured clinical exam and simulated office oral exam results, field notes, entrustable professional activities, multisource feedback, and in-training evaluation reports. Document elements are the resident's continuing medical education activities including procedures log, attendance log, and patient demographic summaries.

For peer review only

No.	Title	Author/year	MERSQI	COREQ	Reason for inclusion			Undergraduate (UG)/Postgraduate (PG)	Methodology	Purpose of study	Key findings
					Communication	Ethics	Professionalism				
29	Diving for PERLS Diving for PERLS Working and Performance Portfolios for Evaluation and Reflection on Learning	Linda E. Pinsky, MD, Kelly Fryer-Edwards, PhD<br><br>2004	-	-	✓		✓	PG	-	Professional competence requires a commitment to lifelong learning, self-assessment, and excellence. Complex skills such as these require flexible and comprehensive teaching and assessment measures. We describe a combination of working and performance portfolios that both foster and evaluate the development of professional competence. We explain the con- ceptual and practical underpinnings that maximize the effec- tiveness of these tools. Drawing on experience with University of Washington residents, we identify 5 criteria that can help promote successful use of portfolios: separate working and performance functions of portfolios, developing a supportive climate, developing skills in faculty and residents, observing progress over time, and fostering mentorship opportunities.	To incorporate portfolios successfully into a residency program, we recommend including 5 elements (Table 1). We had success in our program by introducing the program slowly, and dedicating time to faculty education on men- toring, reflection, goal setting, and feedback skills, recog- nizing that the use of portfolios requires new skills and a cultural transformation. We are pleased by the residents' appreciation of the process, their honesty in sharing and evaluating personal areas of challenge, and their incorpora- tion of this approach into other areas of their education. For example, a current inpatient chief resident, based on his ex- perience of the importance of goal setting to learning, revised the structure of morning report to include weekly goal-setting sessions as well as a method to evaluate its effectiveness. Residents seeking employment report finding components of the portfolio helpful in creating their CVs, crafting their applications, and as display examples at job applications.<br><br>To date, the literature reports few trials of the portfolio method for assessing professional competence. The Post Graduate Education Accreditation (PGEA) in Britain illus- trates sharing responsibility for learning between teachers and learners. PGEA experimented with portfolios

For peer review only

No.	Title	Author/year	MERSQI	COREQ	Reason for inclusion			Undergraduate (UG)/Postgraduate (PG)	Methodology	Purpose of study	Key findings
					Communication	Ethics	Professionalism				
											as a flex- ible, targeted means of meeting continuing medical edu- 18 –20 cation (CME) requirements. In their pilot study, physicians were responsible for identifying their own learning objectives and outlining how they were going to achieve and document those objectives. The physicians each met with a mentor to review appro- priate objectives and realistic means for achieving them. Structured reflection was built into the process and was seen as strength, explicitly moving away from the passive approach of most traditional lecture-based CME courses. In the PGEA experience, the shortcomings identified were time and mentor availability. However, participants found the “deep learning” and personal mastery they achieved to be well worth it. In addition, accrediting bodies had something of substance to review in assessing the practitioners’ continuing education. Portfolios fill a need within training programs to foster 21–23 and document professional competencies. dents, training is a time of professional identity formation. The current format of most residencies — didactic confer- ences and time-consuming clinical work—emphasizes either a passive or unreflective learner role. Through portfolios, residents can experience

For peer review only

No.	Title	Author/year	MERSQI	COREQ	Reason for inclusion			Undergraduate (UG)/Postgraduate (PG)	Methodology	Purpose of study	Key findings
					Communication	Ethics	Professionalism				
											and develop the skills of lifelong learning, critical thinking, self- assessment, and excellence that can be carried forward into their future careers and practices. They can complete residency with a clearer sense of their own strengths and limitations, and a sense of who they are as physicians.
30	How do postgraduate GP trainees regulate their learning and what helps and hinders them? A qualitative study	Sagasser, M. H., Kramer, A. W. and van der Vleuten, C. P. 2012	-	18	✓			PG	In a qualitative study with a phenomenologic approach we interviewed first- and third-year GP trainees from two universities in the Netherlands. Twenty-one verbatim transcripts were coded. Through iterative discussion the researchers agreed on the interpretation of the data and saturation was reached.	To explore how postgraduate trainees regulate their learning in the workplace, how external regulation promotes self-regulation and which elements facilitate or impede self-regulation and learning.	Trainees used a short and a long self-regulation loop. The short loop took one week at most and was focused on problems that were easy to resolve and needed minor learning activities. The long loop was focused on complex or recurring problems needing multiple and planned longitudinal learning activities. External assessments and formal training affected the long but not the short loop. The supervisor had a facilitating role in both loops.
31	First year specialist trainees' engagement with reflective practice in the e-portfolio	Helen McNeill • Jeremy M. Brown • Nigel J. Shaw 2010	7.5	12	✓			PG	A modified Delphi technique was used to develop a grading system to indentify the level of reflection recorded by participants in the e-portfolio. Transcripts of the reflective accounts were then analysed using a qualitative approach which involved coding and categorising	This study aims specifically to explore how they have engaged in reflection on their practice and how they utilise their learning portfolio to document evidence of this.	Findings indicate there is variation is the extent to which doctors both engage in and document evidence of reflection. Further research is needed to explore factors that enable or inhibit the use of the e-portfolio for reflection and whether recorded reflection is a true picture of the cognitive process involved.

For peer review only

No.	Title	Author/year	MERSQI	COREQ	Reason for inclusion			Undergraduate (UG)/Postgraduate (PG)	Methodology	Purpose of study	Key findings
					Communication	Ethics	Professionalism				
									the data. This study demonstrated a wide variation in both the quantity and quality of reflection. Of particular note in the qualitative data analysis were themes relating to clinical knowledge and skills, learning in practice, communication, feelings, types of learning experience reflected on and wider aspects of medical practice.		
32	Development of a learning portfolio to assess the competency of anesthesia residents in Thailand	Suraseranivongse, S., Chinachoti, T., Aroonpruksakul, N., Halilamien, P., Rushatamukayanunt, P., Raksamani, K., Sirivanasandha, B. and Mandee, S.<br><br>2011	12.5	8			✓	PG	Learning portfolio was developed from Thai Medical Council general competencies, academic activities, and performance assessment in several modalities. Twenty-four first year anesthesia residents and eight mentors were enrolled for this study. One staff mentored three residents and rated their competencies in portfolios, twice, four-months apart. Content validity was assessed by six content experts. Concurrent validity of portfolio was determined	Develop a portfolio for learning improvement in first year anesthesia residents in Thailand, and validate this portfolio as a competency evaluation and to identify strength and weakness of implementation.	All content experts accepted that this portfolio could assess general competencies of the first year anesthesia residents. The majority of mentors and residents (>70%) agreed with the benefit of portfolio based on learning development and competency assessment. However, half of residents were not satisfied with the burden from portfolio

No.	Title	Author/year	MERSQI	COREQ	Reason for inclusion			Undergraduate (UG)/Postgraduate (PG)	Methodology	Purpose of study	Key findings
					Communication	Ethics	Professionalism				
									by agreement with faculty global rating and in-training examination. Inter-rater reliability of portfolio was evaluated by five faculties that rated 24 residents. Practicality was commented upon by all mentors and residents in the questionnaire and semi-structure, open-ended questions.		
33	Education Research: Communication skills for neurology residents - Structured teaching and reflective practice	Watling, C. J. and Brown, J. B.<br><br>2007	8.5	10	✓	✓		PG	A group of 12 neurology residents participated in a series of six case-based communi-cation skills workshops. Each workshop focused on a particular clinical scenario, including break-ing bad news, discussing do-not-resuscitate orders, communicating with “difficult” patients, disclosing medical errors, obtaining informed consent for neurologic tests and procedures, and discussing life-and-death decisions with families of critically ill patients.	Despite the importance of communication skills for neurologists, specific training in this area at the residency level is often lacking. This study aimed to enhance learning of these skills and to encourage reflective practice around communication skills.	The program was well accepted, and residents rated the workshops as effective and relevant to their practice. Analysis of residents’ portfolios revealed three themes relevant to patient–physician communication: 1) communication is more successful when adequate time is allowed, 2) the ability to empathize with patients and their families is essential to successful interactions, and 3) the develop- ment of specific approaches to challenging scenarios can facilitate effective interactions. The portfo- lios also demonstrated that residents would engage in reflective practice.<br><br>Targeting of communication skills training around specific clinical scenarios using neurologic cases was well accepted and was deemed relevant to practice. The use of portfolios may

For peer review only

No.	Title	Author/year	MERSQI	COREQ	Reason for inclusion			Undergraduate (UG)/Postgraduate (PG)	Methodology	Purpose of study	Key findings
					Communication	Ethics	Professionalism				
									Residents also kept reflective portfolios in which real examples of these interactions were recorded.		promote lifelong learning in this area.
34	What the HEC-C? An Analysis of the Healthcare Ethics Consultant-Certified Program: One Year in	Horner, C. Childress, A. Fantus, S. Malek, J. 2020			✓	✓		PG	-	In this paper, we explore the history that has led to this certification process, and evaluate the ability of the HEC-C Program to meet the goals it has set forth for HCECs. We describe the benefits and weak- nesses of the program and offer constructive feedback on how the process might be strengthened, as well as share our team's experience in preparing for the exam.	Ultimately, while we are generally supportive of efforts to professionalize the field and ensure competence among HCECs, we are skeptical that a limited process such as this provides much assurance of such compe- tence either for HCECs or their employers. We pro- pose that a more robust or two-tiered evaluation process is needed before such a credential could reli- ably attest to the competence and expertise of an HCEC. Despite shortcomings in the Certification Process as it is currently designed, in general there are benefits of having some kind of certification for individual consultants, as well as HCEC programs and the broader HCEC field. For consultants, achieving and maintaining certification requires continued review of core materials to refresh ethics knowledge and pro- vides external validation of the consultant's ability to engage in consultation, boosting confidence that the individual is successfully engaged in continuing educa- tion. For HCEC programs, having credentialed con- sultants supports the program's mission to engage in quality consultation, taking a step toward ensuring individual consultants are performing their job in a manner

For peer review only

No.	Title	Author/year	MERSQI	COREQ	Reason for inclusion			Undergraduate (UG)/Postgraduate (PG)	Methodology	Purpose of study	Key findings
					Communication	Ethics	Professionalism				
											consistent with established standards in the field. Finally, for the HCEC community as a whole, having a singular credential helps to standardize the practice and validate ethics consultation as a defined and important healthcare specialty. This effort to legit- imize the field as a profession will support the inclu- sion of clinical ethics across healthcare, hopefully expanding the practice from a few select academic or larger healthcare systems to all health care settings. Reaching a point where a health care ethics credential signifies not only competence but also excellence in the ability to conduct a clinical ethics consultation, however, may still be a long way off.
35	Assessing Professionalism in Medicine – A Scoping Review of Assessment Tools from 1990 to 2018	Tay, K. T.<br>Ng, S.<br>Hee, J. M.<br>Chia, E. W. Y.<br>Vythilingam, D.<br>Ong, Y. T.<br>Chiam, M.<br>Chin, A. M. C.<br>Fong, W.<br>Wijaya, L.<br>Toh, Y. P.<br>Mason, S.<br>Krishna, L. K. R.<br><br>2020			✓		✓	UG and PG	Arksey and O'Malley's (2005) approach to scoping reviews was used to identify appropriate publications featured in four data- bases published between 1 January 1990 and 31 December 2018. Seven members of the research team employed thematic analysis to evaluate the selected articles.	Medical professionalism enhances doctor-patient relationships and advances patient-centric care. However, despite its pivotal role, the concept of medical professionalism remains diversely understood, taught and thus poorly assessed with Singapore lacking a linguistically sensitive, context specific and culturally appropriate assessment tool. A scoping review of assessments of professionalism in medicine was thus carried out to better guide its understanding	Prevailing assessments of professionalism in medicine must contend with differences in setting, context and levels of pro- fessional development as these explicate variances found in existing assessment criteria and approaches. However, acknowledging the sig- nificance of context-specific competency-based stages in medical professionalism will allow the forwarding of guiding principles to aid the design of a culturally-sensitive and practical approach to assessing professionalism.

For peer review only

No.	Title	Author/year	MERSQI	COREQ	Reason for inclusion			Undergraduate (UG)/Postgraduate (PG)	Methodology	Purpose of study	Key findings
					Communication	Ethics	Professionalism				
36	Content and outcomes of narrative medicine programmes: a systematic review of the literature through 2019	Remein, Christy DiFrances<br>Childs, Ellen<br>Pasco, John Carlo<br>Trinquart, Ludovic<br>Flynn, David B.<br>Wingerter, Sarah L.<br>Bhasin, Robina M.<br>Demers, Lindsay B.<br>Benjamin, Emelia J.<br><br>2020	-	-	✓			PG	We conducted a systematic review of literature published through 2019 using five major databases: PubMed, Embase, PsycINFO, ERIC and MedEdPORTAL. Eligible NM programming included textual analysis/close reading of published literature and creative/reflective writing. Qualifying participants comprised individuals from academic medicine and health sciences disciplines. We reviewed and categorised programme goals, content and evaluation activities to assess participant satisfaction and programme efficacy. Two members of the research team assessed the risk of bias, independently screening records via a two-round, iterative process to reach consensus on eligibility.	Narrative medicine (NM) incorporates stories into health sciences paradigms as fundamental aspects of the human experience. The aim of this systematic review is to answer the research question: how effective is the implementation and evaluation of NM programmes in academic medicine and health sciences? We documented objectives, content and evaluation outcomes of NM programming to provide recommendations for future narrative-based education.	Of 1569 original citations identified, we selected 55 unique programmes (described in 61 records). In all,<br>41 (75%) programmes reported a form of evaluation; evaluation methods lacked consistency. Twenty-two programmes used quantitative evaluation (13 well described), and 33 programmes used qualitative evaluation (27 well described). Well-described quantitative evaluations relied on 32 different measures (7 validated) and showed evidence of high participant satisfaction and pre-post improvement in competencies such as relationship- building, empathy, confidence/personal accomplishment, pedagogical skills and clinical skills. An average of 88.3% of participants agreed or strongly agreed that the programme had positive outcomes. Qualitative evaluation identified high participant satisfaction and improvement<br>in competencies such as relationship-building, empathy, perspective-taking/reflection, resilience and burnout detection/mitigation, confidence/personal accomplishment, narrative competence, and ethical inquiry.<br><br>Evaluation suggests that NM programming leads to high participant satisfaction and positive outcomes

For peer review only

For peer review only - http://bmjopen.bmj.com/site/about/guidelines.xhtml

No.	Title	Author/year	MERSQI	COREQ	Reason for inclusion			Undergraduate (UG)/Postgraduate (PG)	Methodology	Purpose of study	Key findings
					Communication	Ethics	Professionalism				
											across various competencies. We suggest best practices and innovative future directions for programme implementation and evaluation.
37	Use of portfolios in early undergraduate medical training	Driessen, Erik<br>Van Tartwijk, Jan<br>Vermunt, Jan<br>van der Vleuten, Cees<br><br>2003		10		✓		UG	During the academic year 2001–02 242 first-year medical students compiled a portfolio. Semi-structured interviews were held with a select group of students to explore the effect of the portfolio on reflection. Students from four random mentor groups, 39 in total, were approached and asked if they would be interviewed on the portfolio. With the exception of one student, all students were prepared to take part in the interview. The interview sched-ule focused on the process of compiling a portfolio and its possible effect on reflective ability. In addition to this, students were given the opportunity to offer suggestions for improvement.	This article describes the use of portfolios in **early** undergraduate medical training... In this article, we will describe the Maastricht portfolio as an illustration of an early undergraduate portfolio. We will discuss the reasons for the choices we made in designing the portfolio and report on a first evaluation, focusing on the effects on students' reflective ability.	The majority of students were of the opinion that analysing one's competences in a portfolio was instructive and meaningful. With regard to learning how to reflect and recognize learning needs, however, mentor coaching proved to be necessary. The results thus far show that the portfolio is a worthwhile addition to existing assessment and learning tools. Our preliminary findings show that the introduction of such a portfolio in the early stages of medical training seems to be an effective way for students to learn how to reflect on their learning and behaviour.
38	Conditions for reflective use of portfolios in	Driessen, E. W.<br>van Tartwijk, J.<br>Overeem, K.		18			✓	UG	We designed a portfolio that was aimed at	Portfolios are often used as an instrument with	The conditions for successful reflective use of portfolios that emerged from

No.	Title	Author/year	MERSQI	COREQ	Reason for inclusion			Undergraduate (UG)/Postgraduate (PG)	Methodology	Purpose of study	Key findings
					Communication	Ethics	Professionalism				
	undergraduate medical education	Vermunt, J. D. van der Vleuten, C. P. 2005							stimulating reflection in early undergraduate medical education, using experiences described in the medical educa- tion literature and elsewhere. Conditions for reflect- ive portfolio use were identified through interviews with 13 teachers (mentors), who were experienced in mentoring students in the process of developing their portfolios. The interviews were analysed according to the principles of grounded theory.	which to stimulate students to reflect on their experiences. Research has shown that working with portfolios does not automatically stimulate reflection. In this study we addressed the question: What are the conditions for successful reflective use of portfolios in undergraduate medical education?	the interviews fell into 4 categories: coaching; portfolio structure and guidelines; relevant experiences and materials, and summative assessment. According to the mentors, working with a portfolio designed to meet these conditions will stimulate students' reflective abilities...This study shows that portfolios are a potentially valuable method of assessing and develop- ing students' reflective skills in undergraduate med- ical training, provided certain conditions for effective portfolios are recognised and met. Portfolios have a strong potential for enhancing learning and assess- ment but they are very vulnerable and may easily lead to disappointment. Before implementing portfolios in education, one should first consider whether the necessary conditions can be fulfilled, including an appropriate portfolio structure, an appropriate assessment procedure, the provision of enough new experiences and materials, and sufficient teacher capacity for adequate coaching and assessment.
39	Portfolio Assessment Implementation in Clinical Year of Community Medicine Module: Students Perspective	Ekayanti, Fika Risahmawati, Risahmawati Fadhilah, Marita 2017	7				✓	UG	Nine portfolios were assigned to students within 5 weeks length module. The data was collected by questionnaires from 46 students after completing module in November 2016. Students were	This study aimed to identify the correlation of students' perspective for using portfolio and their portfolio scores.	Most students (36/78.3%) felt that the portfolio was not appropriate as their summative assessment. They preferred less portfolios, 30 (63%) students requested to decrease portfolios to 3-5, 28.3% to 6-8 and only 8.7% to <3. There was no significant correlation between students' satisfaction of using portfolio

For peer review only

No.	Title	Author/year	MERSQI	COREQ	Reason for inclusion			Undergraduate (UG)/Postgraduate (PG)	Methodology	Purpose of study	Key findings
					Communication	Ethics	Professionalism				
									asked about their satisfaction of using portfolio, then correlated to their portfolio scores. The reasons and suggestion for ideal portfolios were identified. Analysis was done by SPSS 20 using Rank Spearman correlation test. Students felt that creating portfolios were not comfortable.		to their portfolios score (p=0.262), while there was significant correlation between students' score to the number of portfolios to be submitted (p=0.017; r=0.349). Reasons for decreasing number of portfolios were inadequate time and many other tasks to be finished. Students in UIN Jakarta haven't used to retelling their experiences and reflecting them in writing as perceived in many students who were not used to portfolios. Doing portfolios need lots of work and time-consuming. Having lesser number of portfolios to be submitted would encourage students to create portfolios. Building familiarization, time management and good motivation for creating portfolios were important to successful portfolios. There was no significant correlation between students' satisfaction to their scores. Students should be encouraged to get comfortable in creating portfolios for the benefit as lifelong learning tool. Motivation to write and reflect should be nurtured to improve students' portfolio satisfaction and commitment.
40	Students' perception of portfolio as a learning tool at King Abdulaziz University Medical School	Fida, N. M.<br>Hassanien, M.<br>Shamim, M. S.<br>Alafari, R.<br>Zaini, R.<br>Mufti, S.<br>Al-Hayani, A.<br>Farouq, M.<br>Al-Zahrani, H.<br><br>2018	8	14	✓	✓	✓	UG	Portfolios were introduced in the 2nd through 5th years at King Abdulaziz University over a two-year period (2013–2015). At the end of each academic year, students completed a	Medical education has a longstanding tradition of using logbooks to record activities. The portfolio is an alter-native tool to document competence and promote reflective practice. This study assessed the	The results showed a difference in focus between basic and clinical years: in basic years students' focus was on acquiring practical skills, but in clinical years they focused more on acquiring complex skills, including identifying and man- aging problems. The questionnaire responses nonetheless revealed a

For peer review only

No.	Title	Author/year	MERSQI	COREQ	Reason for inclusion			Undergraduate (UG)/Postgraduate (PG)	Methodology	Purpose of study	Key findings
					Communication	Ethics	Professionalism				
									mixed questionnaire that included a self-assessment of skills learned through the use of portfolio.	acceptance of portfolio use among Saudi undergraduate medical students.	positive trend in acceptance (belief in the educational value) of portfolios among students and their mentors, across the years of the program. Using portfolios as a developmental learning and formative assessment tool in the early undergraduate years was found to contribute to students' ability to create their own clinical skills guidelines in later years, as well as to engage in and appreciate reflective learning.
41	The use of portfolios to foster professionalism: Attributes, outcomes, and recommendations	Renato Soleiman Franco, Camila Ament Giuliani dos Santos Franco,Olívia Pestana,Milton Severo &Maria Amélia Ferreira<br><br>2017		9		✓	✓	UG	A systematic review on the use of portfolios in teaching professionalism to medical students identified 1257 papers. Of these, 11 articles met all inclusion criteria	The main objective of this research was to review the characteristics of portfolios and their outcomes for teaching professionalism to undergraduate medical students	According to the papers, the use of portfolios for teaching professionalism shows versatility, supports learning strategies and has the potential to be used in different contexts, including for formative and summative purposes. The weaknesses were based on the artificiality of the reflections, deficient instructions, time-consuming processes and preference among students for other teaching methods. Students complained about feeling that the reflection was 'forced', and they tended to write based on social conventions rather than reveal their true thoughts. Reflection is a powerful component of the portfolio, but the method by which it is taught could easily ruin its potential to boost professionalism. Requiring reflection did not ensure its achievement; increased understanding by students regarding how and why they were doing it, the clarity of assessment methods and constructive feedback might strengthen the potential for success. A

For peer review only

No.	Title	Author/year	MERSQI	COREQ	Reason for inclusion			Undergraduate (UG)/Postgraduate (PG)	Methodology	Purpose of study	Key findings
					Communication	Ethics	Professionalism				
											framework was designed to support faculty members in developing and applying portfolios with a clear and broad view of this teaching strategy.
42	AMEE Medical Education Guide No. 24: Portfolios as a method of student assessment	Friedman Ben David, M.<br>Davis, M. H.<br>Harden, R. M.<br>Howie, P. W.<br>Ker, J.<br>Pippard, M. J.<br><br>2001	-	-	✓	✓	✓	UG	-	This guide is intended to inform medical teachers about the use of portfolios for student assessment. It provides a background to the topic, reviews the range of assessment purposes for which portfolios have been used, identifies possible portfolio contents and outlines the advantages of portfolio assess-ment with particular focus on assessing professionalism...The current state of understanding of the technical, psychometric issues relating to portfolio assessment is clarified. The final part of the paper provides a practical guide for those wishing to design and implement portfolio assessment in their own institutions.	-
43	Teaching professionalism in the early years of a medical curriculum: a qualitative study	Goldie, J.<br>Dowie, A.<br>Cotton, P.<br>Morrison, J.<br><br>2007		18	✓	✓		UG	A qualitative approach was adopted involving semistructured interviews, on a 1 in 6 sample of tutors involved in teaching in the early curricular years, and 3 student focus groups. The	This study investigated tutors' and students' perspectives of the delivery of profession- alism in the early years of Glasgow's learner-centred, problem-based learning (PBL), integrated medical curriculum.	Involvement in teaching raised students' and tutors' awareness of their professionalism. Learning activities promoting critical reflection were most effective. The integration of professionalism across the domains of Vocational Studies (135) was important for learning; however, it was not well integrated with the

For peer review only

No.	Title	Author/year	MERSQI	COREQ	Reason for inclusion			Undergraduate (UG)/Postgraduate (PG)	Methodology	Purpose of study	Key findings
					Communication	Ethics	Professionalism				
									findings were subjected to between-method triangulation.		PBL core. Integration was pro- moted by having the same tutor present throughout all VS sessions. Early patient contact experiences were found to be particularly important. The hidden curriculum provided both opportunities for, and threats to, learning. The small-group format provided a suitable environment for the examination of pre-existing perspectives. The portfolio was an effective learning tool, although its assessment should be formalised.<br><br>Reflection is integral to professional development. Early clinical contact is an important part of the process of socialisation, as it allows students to enter the community of practice that is the medical profession. Role models can contribute powerfully to students' learning and identity formation. As students move towards fuller participation, the clinical milieu should be controlled to maximise the influence of role models, and opportunities for guided reflection should be sustained.
44	Assessing students' personal and professional development using portfolios and interviews	Gordon, J.<br><br>2003	6.5			✓	✓	UG	The instruments used to assess Year 1 stu- dents in PPD are a portfolio and interview. This assessment format encourages students to explore ideas and values that are important to them	Medical schools are placing more emphasis on students' personal and professional development (PPD) and are seeking ways of assessing student progress towards meeting outcome goals in relation to professionalism. The	In 1997⁄98, 96% of students agreed that they had engaged in useful reflection on their approach to the course and 91% agreed that the experience was worthwhile. A further 76% of students agreed that they could see opportunities to modify their approach in some ways as result of this exercise.

No.	Title	Author/year	MERSQI	COREQ	Reason for inclusion			Undergraduate (UG)/Postgraduate (PG)	Methodology	Purpose of study	Key findings
					Communication	Ethics	Professionalism				
									and relevant to the PPD theme. A confidential interview, based on the PPD goals, is held with a faculty member who has read the student's portfolio.	Faculty of Medicine at the University of Sydney sought an assessment method that would demonstrate the value of reflection in attaining PPD, provide feedback and encourage stu- dents to take responsibility for setting and achieving high standards of performance.	
45	Developing a sustainable electronic portfolio (ePortfolio) program that fosters reflective practice and incorporates CanMEDS competencies into the undergraduate medical curriculum	Hall, P. Byszewski, A. Sutherland, S. Stodel, E. J. 2012	-	-	✓		✓	UG	-	In this article, the authors describe the development of an electronic Portfolio (ePortfolio) program that enables uOttawa medical students to document their activities and to demonstrate their development of competence in each of the eight roles	The authors reflect on the challenges they faced in the development and implementation of the ePortfolio program and share the lessons they have learned along the way to a successful and sustainable program. These lessons include switching from a complex information technology system to a user-friendly, Web- based blog platform; rethinking orientation sessions to ensure that faculty and students understand the value of the ePortfolio program; soliciting student input to improve the program and increase student buy-in; and providing faculty development opportunities and recognition.
46	The utility of reflective portfolios as a method of assessing first year medical students' personal and professional development	Rees, Charlotte E. Shepherd, Maggie Chamberlain, Suzanne 2005	8	17	✓			UG	The construct validity of the criteria was established by exploring its relationship with two other assessment methods and its reliability was determined using the	-	Themes emerging from the focus groups include students preferring the structured nature of the portfolios but assessors feel- ing that this reduced the uniqueness of the portfolios. Although students understood the importance of reflective practice, some disliked the process of reflection, particularly

No.	Title	Author/year	MERSQI	COREQ	Reason for inclusion			Undergraduate (UG)/Postgraduate (PG)	Methodology	Purpose of study	Key findings
					Communication	Ethics	Professionalism				
									generalizability (G) coefficient. Focus groups with assessors and students were convened to explore their views of the portfolios. Two portfolio analyses had a G coefficient of .42. Performance in the portfolio analyses was correlated with performance in personal and professional development judgements (r = −.512, p < .01) and scientific reports (r = .273, p = .002).		reflective writing. Educators should design assessment criteria that maximize reliability, validity and acceptability rather than simply focusing on reliability alone.<br><br>Despite these methodological limitations, this study provides support for the reli- ability and validity of the portfolio analyses assessment criteria at Peninsula Medical School.
47	Reflections: an inquiry into medical students' professional identity formation	Wong, A.<br>Trollope-Kumar, K.<br><br>2014		14	✓	✓		UG	Sixty-five medical students (46 women; 19 men) from a class of 194 consented to the study of their portfolios. In total, 604 reflections were analysed and coded using thematic narrative analysis. The codes were merged under subthemes and themes. Common or recurrent themes were identified in order to develop a descriptive framework of professional identity formation. Reflections were then analysed	The purpose of this study was to understand the major influences on medical students' professional identity formation.	Five major themes were associated with professional identity formation in medi- cal students: prior experiences, role models, patient encounters, curriculum (formal and hidden) and societal expectations. Our longi- tudinal analysis shows how these themes inter- act and shape pivotal moments, as well as the iterative nature of professional identity from the multiple ways in which individuals con- struct meaning from interactions with their environments.<br><br>Our study provides a window on the dynamic, discursive and constructed nature of professional identity formation. The five key themes associated with professional identity formation provide strategic

For peer review only

No.	Title	Author/year	MERSQI	COREQ	Reason for inclusion			Undergraduate (UG)/Postgraduate (PG)	Methodology	Purpose of study	Key findings
					Communication	Ethics	Professionalism				
									longitudinally within and across individual portfolios to examine the professional identity formation over time with respect to these themes.		opportuni- ties to enable positive development. This study also illustrates the power of reflective writing for students and tutors in the professional identity formation process.
48	Content validity of workplace-based portfolios: A multi-centre study	Nele R.M. Michels, Marijke Avonts, Griet Peeraer, Kris Ulenaers, Luc F. Van Gaal, Leo L. Bossaert, Erik W. Driessen, Arno M.M. Muijtjens & Benedicte Y. De Winter<br><br>2016	9.5		✓		✓	UG	We reviewed 120 workplace portfolios at three medical universities (Belgium and the Netherlands). To validate their content, we developed a Validity Inventory for Portfolio Assessment (VIPA) based on the CanMEDS roles. Two raters evaluated each portfolio and indicated for each VIPA item whether the portfolio provided sufficient information to enable satisfactory assessment of the item. We ran a descriptive analysis on the validation data and computed Cohen's Kappa to investigate interrater agreement.	This study sought to evaluate the content validity of portfolios as reflected in their capacity to adequately assess achieved competences of medical students during clerkships.	The portfolios adequately covered the items pertaining to the communicator (90%) and professional (87%) roles. Coverage of the medical expert, collaborator, scholar and manager roles ranged between 75% and 85%. The health advocate role, covering 59%, was clearly less well represented. This role also exhibited little interrater agreement (Kappa50.4).<br><br>This study lends further credence to the evidence that portfolios can indeed adequately assess the different CanMEDS roles during clerkships, the health advocate role, which was less well represented in the portfolio content, excepted.
49	Does a summative portfolio foster the development of capabilities such	Anthony J O'sullivan, Amanda C Howe, Susan Miles, Peter Harris, Chris S Hughes, Philip Jones, Helen	7.5		✓	✓	✓	UG	A questionnaire was designed to evaluate undergraduate medical students' experiences of	The aims of this study were (i) to determine whether preparing a portfolio helps promote students'	Students perceive portfolio preparation as an effective learning tool for the development of capabilities such as understanding ethical and legal principles

For peer review only

No.	Title	Author/year	MERSQI	COREQ	Reason for inclusion			Undergraduate (UG)/Postgraduate (PG)	Methodology	Purpose of study	Key findings
					Communication	Ethics	Professionalism				
	as reflective practice and understanding ethics? An evaluation from two medical schools	Scicluna & Sam J Leinster<br><br>2012							completing a portfolio at two medical schools. A total of 526 (45% response rate) students answered the on-line questionnaire. Students from both medical schools gave the highest ranking for the portfolio as a trigger for reflective practice. 63% of students agreed their portfolio helped them develop reflective practice skills (p < 0.001), whereas only 22% disagreed. 48% of students agreed portfolios helped them understand ethical and legal principles whereas 29% disagreed (p < 0.001). In contrast, only 34% of students thought the portfolio helped them to develop effective communication.	development in a range of capabilities including understanding ethical and legal principles, reflective practice and effective communication, and (ii) to determine to what extent the format of the portfolio affected the outcome by comparing the experiences of students at two different medical schools.<br><br>Therefore, the primary aim of this study was to determine whether the process of preparing a portfolio report or essay helped students appreciate and develop in a range of capabilities, such as understanding ethical and legal principles and reflective practice; capabilities which are not the focus of traditional assessments such as clinical or written examina- tions. The format of the portfolio assessment was different in each of the two medical schools in the study, and so a secondary aim was to determine to what extent the format of the assessment affected the outcome by comparing the student experiences with their portfolio	and reflective practice, whereas other capabilities such as effective communication require complementary techniques and other modes of assessment.

No.	Title	Author/year	MERSQI	COREQ	Reason for inclusion			Undergraduate (UG)/Postgraduate (PG)	Methodology	Purpose of study	Key findings
					Communication	Ethics	Professionalism				
										assessment in the two schools.	
50	Feasibility and Outcomes of Implementing a Portfolio Assessment System Alongside a Traditional Grading System	Celia Laird O'Brien, PhD, Sandra M. Sanguino, MD, MPH, John X. Thomas, PhD, and Marianne M. Green, MD<br><br>2016	7		✓		✓	UG	In 2009, the authors developed a portfolio system that served as a repository for all student assessments organized by competency domain. Five competencies were selected for a preclerkship summative portfolio review. Students submitted reflections on their performance. In 2014, four clinical faculty members participated in standard-setting activities and used expert judgment and holistic review to rate students' competency achievement as "progressing toward competence," "progressing toward competence with some concern," or "progressing toward competence pending remediation." Follow-up surveys measured students' and faculty members'	This study describes the development and implementation of a longitudinal competency-based electronic portfolio system alongside a graded curriculum at a relatively large U.S. medical school.	Faculty evaluated 156 portfolios and showed high levels of agreement in their ratings. The majority of students achieved the "progressing toward competence" benchmark in all competency areas. However, 31 students received at least one concerning rating, which was not reflected in their course grades. Students' perceptions of the system's ability to foster self-assessment were mixed.

No.	Title	Author/year	MERSQI	COREQ	Reason for inclusion			Undergraduate (UG)/Postgraduate (PG)	Methodology	Purpose of study	Key findings
					Communication	Ethics	Professionalism				
									perceptions of the process.		
51	Feelings related to first patient experiences in medical school A qualitative study on students' personal portfolios	Kaisu H. Pitkälä a,⁎, Taina Mäntyranta b<br><br>2003? 2004		14	✓		✓	UG	Twenty-two volunteer third and fourth year medical students (15 women and 7 men) of the University of Helsinki participated in a portfolio course for 1 year. Their reflective learning diaries and writings on specific themes were analyzed by qualitative content analysis	Feelings and thoughts of medical students related to first patient experiences during the first clinical year were examined	First patient encoun- ters were strong emotional experiences for medical students. The first patient examination was often described as an anxiety-provoking and confusing incident. Other emotionally significant encounters included helplessness when faced with serious illness and death, and role confusion when examining patients of one's own age but opposite sex. Students felt guilty for using patients for their own learn- ing purposes. Portfolios as learning tools may help in recognizing key experiences and support professional development of medical students.
52	Linking assessment to undergraduate student capabilities through portfolio examination	Anthony J. O'Sullivan , Peter Harris , Chris S. Hughes , Susan M. Toohey , Chinthaka Balasooriya , Gary Velan , Rakesh K. Kumar & H. Patrick McNeil<br><br>2012	8.5		✓	✓	✓	UG	Students are required to complete assessments linked to graduate capabilities. In Year 3, a portfolio review occurs (205–248 students per year), focusing on students' grades and feedback from assessments and a reflective essay is submitted. In the essay, students reflect on their progress, identify areas of weakness and detail plans for improvement. Progress in each capability is summatively graded against	This article describes an e-portfolio which closely aligns learning and reflection to graduate capabilities, incorporating features that address concerns about portfolios	Portfolios are an established method of assessment, although concerns do exist around their validity for capabilities such as reflection and self-direction

For peer review only

No.	Title	Author/year	MERSQI	COREQ	Reason for inclusion			Undergraduate (UG)/Postgraduate (PG)	Methodology	Purpose of study	Key findings
					Communication	Ethics	Professionalism				
									specific criteria and feedback is provided.		
53	Medical student resilience strategies: A content analysis of medical students' portfolios	Richard A. Prayson · S. Beth Bierer · Elaine F. Dannefer<br><br>2016		12	✓	✓	✓	UG	We retrospectively reviewed a sampling of year 1, 2 and 5 portfolio essays focused on the Personal Develop- ment competency and performance milestones, written by 49 students from three different classes in a 5-year pro- gramme devoted to training physician investigators. Two medical educators used a framework established by Jensen and colleagues (2008) to identify the nature and prevalence of various resilience strategies (valuing the physician role, self-awareness, personal arena, professional arena, profes- sional support and personal support) medical students re- ported in portfolio essays.	A student's ability to employ resilience strategies to self-regulate behaviour is critical to the student's future career as a physician. (The aim is to examine this??)	All students documented at least one strategy in their essays each year. In all years, the most commonly documented strategies were in the personal arena (95.7% of year 1, 98% of year 2 and 87.8% of year 5 portfolios). The least frequently documented strategy in all years was professional support (42.8% of year 1, 38.8% of year 2, and 28.6% of year 5 portfolios). Year 5 portfolios discussed personal support strategies (79.6%) more frequently than year 1 (53.1%) and year 2 (59.2%) portfolios.<br><br>The results suggest that medical students can identify stressors and articulate resilience strategies that can be employed to potentially address them.
54	Portfolio Assessment during Medical Internships: How to Obtain a Reliable and Feasible	NRM Michels, EW Driessen, AMM Muijtjens, LF Van Gaal, LL Bossaert, BY De Winter<br><br>2009		9.5			✓	UG	The domain-oriented reliability of 61 double-rated portfolios was measured, using a generalisability	In this study, we investigated whether assessment during medical internship by a portfolio can combine reliability and feasibility.	We obtained reliability (Φ coefficient) of 0.87 with this internship portfolio containing 15 double-rated tasks. The generalisability analysis showed that an acceptable level of reliability

For peer review only - http://bmjopen.bmj.com/site/about/guidelines.xhtml

No.	Title	Author/year	MERSQI	COREQ	Reason for inclusion			Undergraduate (UG)/Postgraduate (PG)	Methodology	Purpose of study	Key findings
					Communication	Ethics	Professionalism				
	Assessment Procedure?								analysis with portfolio tasks and raters as sources of variation in measuring the performance of a student.		(Φ = 0.80) was maintained when the amount of portfolio tasks was decreased to 13 or 9 using one and two raters, respectively.<br><br>Our study shows that a portfolio can be a reliable method for the assessment of workplace learning. The possibility of reducing the amount of tasks or raters while maintaining a sufficient level of reliability suggests an increase in feasibility of portfolio use for both students and raters.
55	Twelve tips for introducing E-Portfolios with undergraduate students	Alis Moores & Maria Parks<br><br>2010	-	-	✓			UG	-	-	-
56	Use of Portfolios in Undergraduate Medical Training: First Meeting With a Patient	Nilgün ÖZÇAKAR, Vildan MEVSİM, Dilek GÜLDAL<br><br>2009		10	✓		✓	UG	We designed a portfolio that was aimed at making reflection in early undergraduate medical education. Conditions for portfolio use were identified through interviews with six trainers who were experienced in mentoring students in the process of developing their portfolios. Analysis of writings was per- formed using qualitative content analysis.	Portfolios, as learning tools, are becoming increasingly important in medical education. Our aim was to evaluate the contents of portfolios used in medical education.	The conditions for successful reflective use of portfolios that emerged from the interviews fell into catego- ries: practice evaluation, patient perspective and primary prevention/health promotion. According to the students, working with a portfolio designed to meet these conditions is very useful and will stimulate students' abilities. The stu- dents shared the same opinion that analyzing one's com- petences in a portfolio was instructive and meaningful.<br><br>According to our students, portfolios are a potentially valuable method of developing students' skills in undergraduate medical education, provided that certain conditions for effective portfolios are recognized and met. The portfolio is a worthwhile addition to existing

For peer review only

No.	Title	Author/year	MERSQI	COREQ	Reason for inclusion			Undergraduate (UG)/Postgraduate (PG)	Methodology	Purpose of study	Key findings
					Communication	Ethics	Professionalism				
											assessment and learning tools.
57	What Is the Relationship Between a Preclerkship Portfolio Review and Later Performance in Clerkships?	Celia Laird O'Brien, PhD, John X. Thomas Jr, PhD, and Marianne M. Green, MD<br><br>2018	9.5		✓		✓	UG	The authors divided students into two groups based on a summative preclerkship portfolio review in 2014: students who had concerning behavior in one or more competencies and students progressing satisfactorily. They compared how students in these groups later performed on two clerkship outcomes as of October 2015: final grades in required clerkships, and performance on a clerkship clinical composite score. They used Mann–Whitney tests and multiple linear regression to examine the relationship between portfolio review results and clerkship outcomes. They used USMLE Step 1 to control for knowledge acquisition.	This study examines whether a preclerkship portfolio review is a valid method of identifying problematic student behavior affecting later performance in clerkships.	Students with concerning behavior preclerkship received significantly lower clerkship grades than students progressing satisfactorily ($P = .002$). They also scored significantly lower on the clinical composite score ($P < .001$). Regression analysis indicated concerning behavior was associated with lower clinical composite scores, even after controlling for knowledge acquisition.<br><br>The results show a preclerkship portfolio review can identify behaviors that impact clerkship performance. A comprehensive portfolio system is a valid way to measure behavioral competencies.
58	Educating undergraduate medical students about oncology: A literature review	Cave, Judith<br><br>2006	-	-	✓			UG	MEDLINE, Psychinfo, ERIC, TIMELIT, EMBASE, CINAHL and the Cochrane CENTRAL Register of	This article is a review of the literature regarding teaching oncology to undergraduate medical students.	The main findings can be summarized as follows: the involvement of patients in teaching is popular with students and portfolio learning is a successful way of involving patients; the use of standardized patients to

For peer review only

No.	Title	Author/year	MERSQI	COREQ	Reason for inclusion			Undergraduate (UG)/Postgraduate (PG)	Methodology	Purpose of study	Key findings
					Communication	Ethics	Professionalism				
									Controlled Trials (CENTRAL) were searched, using the search terms cancer, oncology, education, undergraduate, and teaching.		teach breast examination improves students' performance in clinical assessment; the use of silicone models to teach breast examination improves students' sensitivity for detecting breast lumps; computer aided learning modules have a role, but are not superior to other types of learning; learning about cancer screening and prevention increases students' knowledge, improves their self rated skills, and changes their behavior; and cancer patients have an important role to play in teaching undergraduate communication skills.<br><br>We have found 48 articles on undergraduate teaching in oncology. Oncology teachers should consider adopting the evidence based approaches outlined in this review, and there should be more emphasis on educational research within the field of oncology.
59	Integrating the assessment of interprofessional education into the health care curriculum	Anderson, E. S. and Kinnair, D.<br><br>2016	-	-	✓	✓	✓	UG	-	Reflection on some of the challenges associated with assessment in IPE by describing the experiences at one UK-based medical school	In regard to written reflections, reading 240 student entries per learning event is time-consuming, making it challenging to offer individual feedback. Some students have challenged the formative outcomes that were graded for progression across broad boundaries of unsatisfactory, satisfactory or excellent. Checking for consistency across a small numbers of marker illuminates the subjective perspectives and interpretations of individual

For peer review only

No.	Title	Author/year	MERSQI	COREQ	Reason for inclusion			Undergraduate (UG)/Postgraduate (PG)	Methodology	Purpose of study	Key findings
					Communication	Ethics	Professionalism				
											markers on what constitutes a meaningful reflection. Nevertheless, offering this early writing platform has proved to be extremely helpful and enables educators to determine if students can reflect critically and apply meaning
60	A model of engagement in reflective writing-based portfolios: Interactions between points of vulnerability and acts of adaptability	Arntfield, S., Parlett, B., Meston, C. N., Apramian, T. and Lingard, L.<br><br>2015	7	21			✓	UG	Using mixed-methods rooted in grounded theory, 139 students and 13 mentors completed questionnaires, 23 students participated in four focus groups and 9 mentors in individual interviews	The aim of this study was to explore conditions affecting the experience of teaching and learning from the perspective of both students and mentors in a reflective writing-based portfolio initiative.	The overarching theme in our data was student–mentor engagement. Our results confirm previous literature describing portfolio as a vulnerable method of learning, extend this concept by identifying and categorizing specific points of vulnerability, and contribute new knowledge regarding acts of adaptability, which serve to strengthen the student–mentor relationship.<br><br>Engagement is central to the success of portfolio and is shaped by a dynamic interaction between points of vulnerability and acts of adaptability. We propose a model of engagement in portfolio that can be used for faculty development to optimize student–mentor engagement.
61	Support for portfolio in the initial years of the undergraduate medical school curriculum: what do the tutors think?	Austin, C. and Braidman, I.<br><br>2008	7	18	✓		✓	UG	A mixed method approach was used with data collected from both questionnaires and a focus group meeting.	The aims of this study were to investigate the views of these tutor facilitators on the delivery and support of portfolios in this way.	Tutor facilitators were generally positive about their roles in the support of portfolio development in group sessions and identified several advantages to this type of tutoring – namely the value of group discussion and discussion between peers to encourage reflection, and the practical ability to integrate portfolio sessions more closely with clinical experiences – which would not be possible in one to one tutor/student meetings.

For peer review only

No.	Title	Author/year	MERSQI	COREQ	Reason for inclusion			Undergraduate (UG)/Postgraduate (PG)	Methodology	Purpose of study	Key findings
					Communication	Ethics	Professionalism				
											With the training and guidance notes provided tutors were confident that they understood the support they should give students and felt that they could encourage reflection.<br><br>Thus, tutors perceived that the support of portfolio in groups is an effective way of developing portfolios for large numbers of students.
62	Use of portfolios by medical students: significance of critical thinking	Azer, S. A.<br><br>2008	-	-	✓		✓	UG	-	The aims of this paper are to: (1) understand the uses of portfolios and the rationale for using reflection in the early years of a PBL curriculum; (2) discuss how to introduce portfolios and encourage students' critical thinking skills, not just reflection; and (53) provide students with tips that could enhance their skills in constructing good portfolios.	-
63	Time to Loosen the Apron Strings: Cohort-based Evaluation of a Learner-driven Remediation Model at One Medical School	Bierer, S. B., Dannefer, E. F. and Tetzlaff, J. E.<br><br>2015	10		✓			UG	We used a retrospective, cohort-based approach to examine our learner-driven remediation model.<br>Data sources: We consulted MSPRC records to identify all CCLCM students from six class cohorts (2009–2014) placed in remediation during medical school, and compared remedial students	To examine a remediation model where students, rather than faculty, develop remedial plans to improve performance.<br>We present an innovative, systematic approach to remediation purposefully designed to engage learners with the development and implementation of remedial plans to improve their performance. We first describe our approach to	From abstract -- participants included 177 students from six classes (2009–2014). Twenty-six were placed in remediation, with more referrals occurring during Years 1 or 2 (n = 20, 76 %). Unprofessional behavior represented the most common reason for referral in Years 3–5. Remedial students did not differ from classmates (n = 151) on baseline characteristics (Age, Gender, US citizenship, MCAT) or willingness to recommend their medical school to future students ($p < 0.05$). Two remedial students did not graduate and three did

For peer review only - http://bmjopen.bmj.com/site/about/guidelines.xhtml

No.	Title	Author/year	MERSQI	COREQ	Reason for inclusion			Undergraduate (UG)/Postgraduate (PG)	Methodology	Purpose of study	Key findings
					Communication	Ethics	Professionalism				
									to their classmates not placed in remediation on variables collected for program evaluation purposes: Baseline characteristics (gender, age, US citizenship, MCAT score), USMLE licensure exam performance (pass-rate for each student), National Residency Matching Program data (clinical specialty for each student), Satisfaction with medical school (collected from graduating students using questionnaire). Then, we read MSPRC correspondence to each remedial student to extract referral mechanism (portfolio-based decision or faculty-initiated), unsatisfactory competency domain(s), remediation duration (days), and completion of students' remediation goals. Finally, we read students' remedial plans to identify strategies they proposed to	remediation and then report outcomes for students who were and were not placed in remediation during medical school. We conclude with lessons learned and future directions.	not pass USLME licensure exams on first attempt. Most remedial students (92 %) generated appropriate plans to address performance deficits. Students can successfully design remedial interventions. This learner-driven remediation model promotes greater autonomy and reinforces self-regulated learning. From results: Participants include 177 of 187 medical students from six class cohorts (2009–2014) who consented (95 %) to release program evaluation data for research purposes. Of these, the MSPRC placed 26 students (17 men and nine women) in formal remediation. Half (13) were identified by the MSPRC for not meeting year-end performance standards assessed via portfolios. The dean of student affairs referred the remaining 13 students based on faculty recommendation. We did not detect significant differences between remedial students and their classmates for baseline characteristics (Table 1). Overall, three-quarters of remediation decisions occurred during the first (10, 38 %) or second (10, 38 %) year of medical school. With regard to specific domains in which students failed to meet year-end standards or were otherwise referred, professionalism (18, 69 %), communication (10, 38 %), research (9, 35 %), and reflective practice (5, 19 %) represent frequent domains of unsatisfactory performance. Fewer students did not meet

For peer review only - http://bmjopen.bmj.com/site/about/guidelines.xhtml

No.	Title	Author/year	MERSQI	COREQ	Reason for inclusion			Undergraduate (UG)/Postgraduate (PG)	Methodology	Purpose of study	Key findings
					Communication	Ethics	Professionalism				
									the MSPRC to address performance deficits in specific competency areas. We manually recorded a subset of these strategies to provide examples of students' approaches to remediation. All performance decisions, referral mechanisms, and outcomes were manually entered into SPSS. Chi-square and ANOVA tests were used to compare baseline characteristics and satisfaction scores of the two student groups (remediation vs. other), while the Mann Whitney U test was used to examine if remediation duration (days) differed by students' gender. Statistical significance was established at p < 0.05 for all comparisons.		performance standards for clinical reasoning (4, 15 %), medical knowledge (3, 11 %), clinical skills (2, 8 %), personal development (2, 8 %), or health care systems (2, 8 %). Unprofessional behavior (e.g., not logging patient encounters, unexcused absences, chronic tardiness, etc.) represented the most common reason for student remediation during Years 3–5 of medical school. Ten students had deficient performance in one competency, while others had unsatisfactory performance in two (7, 27 %) or more (9, 35 %) competencies. Three students were placed in remediation twice, with one repeating for unprofessional behavior. Time in remediation (determined by dates in MSPRC correspondence to students) ranged from 39 to 1696 days (Median = 448 days) and did not differ by student gender (p > 0.05). Remediation plans revealed various student-proposed strategies to address TAFIs for multiple competency domains (Table 2). We observed that students' remedial strategies resembled approaches faculty would recommend. We also observed the MSRPC's correspondence to students consistently had an encouraging, supportive tone for students' remedial efforts (See Appendices for MSPRC's correspondence to one remedial student where committee asks student to develop a remediation plan, comments

No.	Title	Author/year	MERSQI	COREQ	Reason for inclusion			Undergraduate (UG)/Postgraduate (PG)	Methodology	Purpose of study	Key findings
					Communication	Ethics	Professionalism				
											on student's progress report, and releases student from remediation). The MSPRC "prescribed" remediation plans for two students only after these students did not provide adequate assessment evidence in progress reports. The MSPRC determined that most students (24, 92 %) generated sufficient evidence (predominately obtained from faculty and peers) to document their competence. Table 1 reveals that some remedial students experienced negative outcomes, as two did not graduate and three did not pass USMLE licensure exams on first attempt. Proportionally, more remedial students (11, 46 %) pursued surgical specialties than their classmates (50, 33 %). Table 1 also shows graduating remedial students were just as likely as their classmates to recommend the CCLCM program to future students, even though fewer were as satisfied with their overall experience at CCLCM as their classmates. From discussion -- Most programs struggle with how to identify and assist learners requiring remediation. We explored if students, rather than faculty, can take ownership for remedial plan design and implementation. We review key findings and conclude with future directions. The locus of control for selecting remedial interventions and assessment evidence typically rests with faculty. In

For peer review only

No.	Title	Author/year	MERSQI	COREQ	Reason for inclusion			Undergraduate (UG)/Postgraduate (PG)	Methodology	Purpose of study	Key findings
					Communication	Ethics	Professionalism				
											our model, the MSPRC charged students to develop remediation plans, with PA guidance as needed, and obtain assessment evidence to document progress and achievement. Most remediating students (92 %) successfully met these expectations and graduated from medical school. Three-quarters of remedial students were identified in Years 1–2 of medical school. Early identification provides opportunities for multiple, tailored interventions to occur. Professionalism, communication skills, and research performance were frequent reasons the MSPRC placed students in remediation. Few students were identified for gaps in medical knowledge or clinical reasoning. The literature suggests some competencies, like professionalism, require direct observation and ongoing assessment for learner success.1,18 Our students' remedial strategies (Table 2) ranged from specific to holistic approaches and often cut across courses and contexts, thereby providing them with more opportunities to improve and document their performance in complex domains. This emphasis on competence may explain why students' time in remediation was longer (Median = 448 days) than normally needed for "more of the same," such as retaking a test or course.2 Some learners may not know how to navigate performance obstacles,

For peer review only

No.	Title	Author/year	MERSQI	COREQ	Reason for inclusion			Undergraduate (UG)/Postgraduate (PG)	Methodology	Purpose of study	Key findings
					Communication	Ethics	Professionalism				
											making it important to offer guidance and oversight.19,20 For this reason, we train a core group of faculty to help students apply the self-regulation cycle.17 PAs coach students in reflection, self-assessment, and goal-setting.2,20,21 They also form long-term relationships with students and frequently serve as student advocates. The MSPRC encourages students to identify measurable outcomes to document competence, while offering positive support in written correspondence to students (See Appendices for series of letters to one student throughout the remediation process). Both faculty groups work in tandem to support this learner-driven remediation model while ensuring student accountability. Our evaluation is limited to one program, at one school, with a small class size. Other programs may foster remedial learners' autonomy more than conveyed in the literature.5,6,8 Our student satisfaction measures originate from two questionnaire items obtained at graduation and do not capture students' satisfaction with specific academic referral policies or remedial processes.22 Remediation is complex and involves multiple stakeholders and contexts. Future studies should capture learners' perceptions of remediation to complement the faculty point-of-view currently reported in the literature.

For peer review only

No.	Title	Author/year	MERSQI	COREQ	Reason for inclusion			Undergraduate (UG)/Postgraduate (PG)	Methodology	Purpose of study	Key findings
					Communication	Ethics	Professionalism				
											We discovered that more men than women were identified for remediation, yet we found no research exploring this observation in depth. Additionally, few longitudinal or multi-institutional studies currently exist, suggesting an area ripe for research. We discovered, in our 10-year experience with this remediation model, that medical students can select appropriate remedial interventions with the support of well-trained faculty. Our learner-driven approach to remediation may transfer to graduate medical education (GME), where Clinical Competence Committees (CCCs) meet regularly to discuss and review resident performance. Having struggling residents create remediation plans and submit progress reports to CCCs may present few barriers, given GME's competency-based assessment framework and emphasis on providing residents with frequent, formative feedback. Our approach requires a learning environment where faculty must trust students have the motivation and capabilities to identify and implement appropriate remedial strategies. We have created a systematic process where students must reflect on their performance, develop plans tailored to meet learning needs, and collect assessment evidence to document competence. To conclude, we believe educators who wish to

For peer review only

No.	Title	Author/year	MERSQI	COREQ	Reason for inclusion			Undergraduate (UG)/Postgraduate (PG)	Methodology	Purpose of study	Key findings
					Communication	Ethics	Professionalism				
											adopt a learner-driven approach to remediation should view remediation as a growth opportunity rather than as a punishment.
64	Medical professionalism: conflicting values for tomorrow's doctors	Borgstrom, E., Cohn, S. and Barclay, S.<br><br>2010		14		✓		UG	From abstract: Qualitative study using interpretative discourse analysis of anonymized student reflective portfolios. One hundred twenty-three final year undergraduate medical students (64 male and 59 female) from the University of Cambridge School of Clinical Medicine supplied 116 portfolios from general practice and 118 from hospital settings about patients receiving palliative or end of life care.<br>From introduction: This study investigates how professionalism is understood and experienced via one such initiative for medical students training in Cambridge, England, and highlights the conflicts between 'new' and 'old' values.<br>From methods: Final year medical students in Cambridge meet patients approaching the	To investigate how final year medical students experience and interpret new values of professionalism as they emerge in relation to confronting dying patients and as they potentially conflict with older values that emerge through hidden dimensions of the curriculum.	From abstract -- Results: Professional values were prevalent in all the portfolios. Students emphasised patient-centered, holistic care, synonymous with a more contemporary idea of professionalism, in conjunction with values associated with the 'old' model of professionalism that had not be directly taught to them. Integrating 'new' professional values was at times problematic. Three main areas of potential conflict were identified: ethical considerations, doctor-patient interaction and subjective boundaries. Students explicitly and implicitly discussed several tensions and described strategies to resolve them. Conclusions: The conflicts outlined arise from the mix of values associated with different models of professionalism. Analysis indicates that 'new' models are not simply replacing existing elements. Whilst this analysis is of accounts from students within one UK medical school, the experience of conflict between different notions of professionalism and the three broad domains in which this conflict arises are relevant in other areas of medicine and in different national contexts.<br>From discussion -- All of the students encountered challenges that required

For peer review only

No.	Title	Author/year	MERSQI	COREQ	Reason for inclusion			Undergraduate (UG)/Postgraduate (PG)	Methodology	Purpose of study	Key findings
					Communication	Ethics	Professionalism				
									end of life during general practice (GP) and hospital attachments. As required coursework they write portfolio items on two patients, one from each attachment. Key to this exercise is integrating issues arising from the interview with reflections of their own personal experiences. This study focuses on how different ideas are received and reproduced by a relatively young (typically early 20s) cohort of soon-to-be qualified doctors during the academic year 2007–2008. The University of Cambridge Psychology Research Ethics committee approved the study, and 86% of students (n = 123; 64 male, 59 female) gave informed consent for 234 reflective items: 116 from the GP and 118 from hospital settings. Each student was assigned a random identification number (ID), which is included		them to try and balance values characteristics of 'old' and 'new' forms of medical professionalism. Ethics, interaction and managing subjective boundaries all generated areas of conflict that they wrestled with in their assignments. Students addressed these tensions in a variety of ways, with strategies drawn from both formally taught skills and personal resources. In parallel with shifts from 'old' to 'new' values of professionalism, conflicts between the formal and the hidden curricula may also generate tensions44. In our study, the formal curricula is explicitly designed to embrace the 'new', leaving the 'old' to be communicated through more informal and hidden aspects of teaching. Yet for the students trying to personally reflect on their experiences and summatively draw from their entire educational experience for the exercise, no distinction is made between these two domains. Large organisations like the University of Cambridge Clinical School and the National Health Service have institutional memories45—collective experiences and concepts. These are communicated imperceptively through teaching elements as well as the very structures and policies that shape the educational environment. This feature serves to complicate understandings of medical education; it

For peer review only

No.	Title	Author/year	MERSQI	COREQ	Reason for inclusion			Undergraduate (UG)/Postgraduate (PG)	Methodology	Purpose of study	Key findings
					Communication	Ethics	Professionalism				
									with the quotes to demonstrate the breadth of the dataset presented. Analysis focused on the topic of 'professionalism', viewing the content of the portfolios as representative of general values imparted to students throughout their education. All items were coded and analysed in NVivo 8 using an interpretative approach. Following the social science traditions of two of the authors (EB and SC), the methodology employed a hermeneutic approach, striving to find meaning beyond straightforward discrete references35. Accordingly, rather than assuming the meaning of isolated sections of text in a reductive manner, they were always analysed in context and in relation to one another. Given the difficulty many students had in addressing the topic of end of life care, and		emphasises the extent to which values are embedded in the routines and practices of the organisation, and cannot therefore be simply addressed through redesigning curricula or individuals championing change. Our study suggests that tensions arise primarily because of different values that underlie the concept of professionalism throughout their education, rather than anything that is specific to end of life care. Whilst topics central to the everyday practice of medicine are particularly evident in palliative care46, they are made highly visible by students who see themselves on the cusp of becoming members of the profession themselves. Whilst it is likely that the concept of professionalism always has contained a wide variety of underlying values and principles that are not always commensurate with each other, we have argued that a more widespread shift in values over recent years has generated greater variance and hence more contradictory positions around what it means to be a good doctor. Our findings add to the literature on medical professionalism, which is rich in doctors' anecdotal experiences47,48, highlights the stresses and conflicts doctors face44,49,50 and illustrates a current amalgamation of definitions with conflicting values51. Specific national and local contexts are likely to generate differently

For peer review only

No.	Title	Author/year	MERSQI	COREQ	Reason for inclusion			Undergraduate (UG)/Postgraduate (PG)	Methodology	Purpose of study	Key findings
					Communication	Ethics	Professionalism				
									consequently the very discursive descriptions of their observations, the authors were committed to code more than just those phrases in which a theme was referred to explicitly, and so included concerns that were expressed less directly. A coding scheme was derived from initial independent item analysis (EB, SC and SB) and adjusted through discussion. It was initially applicable to explicit statements by drawing on key words and then was augmented to include the more implicit references that potentially alluded more to the hidden dimensions of their teaching (see Box 2). The inclusion of these required careful interpretative readings of the entire text but provided an essential contribution to our overall analysis. The first author (193) then led on-going analysis, with regular meetings to		nuanced versions of these issues, which might only be identified through comparative work. From conclusion: The integration of 'new' professional values taught in medical school is at times problematic for students in any health care system that maintains elements, whether overtly or not, of the 'old' paradigm. The areas of potential conflicts outlined—ethics, patient interaction and managing subjective boundaries—are not limited to medical school or end of life care. Our analysis suggests that 'old' variants of professionalism are not simply being replaced by 'new' ones delivered by a redesigned curriculum, but rather that values from each can emerge in a range of medical contexts. As a result, professionalism does not consist of a set of fixed or abstract concepts, but rather surfaces through medical practice. If individual reflection is now heralded as an essential component of the 'new' professionalism, as indicated by the compulsory student exercise we have drawn on here, it should be acknowledged that it demands a dynamic engagement with the wide range of often contradictory and shifting ideas and beliefs from both formal and more hidden aspects of their education. This study illustrates that overt commitment to more empathic and patient-centered approaches to medical care do not

For peer review only

No.	Title	Author/year	MERSQI	COREQ	Reason for inclusion: Communication	Ethics	Professionalism	Undergraduate (UG)/Postgraduate (PG)	Methodology	Purpose of study	Key findings
									discuss emerging themes.		necessarily replace other more prescribed values and behaviours that remain part of a hidden curriculum embedded in institutional practices. Integration of 'new' core values and skills into good medical practice is not a smooth or simple transition. Additionally, it seems any simple attempt to communicate them through formal teaching is unlikely to prepare students for the reality of medical encounters. Instead, the experience of tension and the individual desire to seek balance and resolution across a wide range of issues may themselves be key and lasting features of medical professionalism.s
65	Facilitating students' reflective practice in a medical course: literature review	Chaffey, L. J., de Leeuw, E. J. and Finnigan, G. A.<br><br>2012		12			✓	UG	The literature search was undertaken using the online databases of CINAHL and Medline. Keyword searching was conducted using MeSH headings, Boolean operators, and the terms, 'medical students', 'medical education', 'reflection', 'reflect*' and 'medicine'. The search was limited to peer-reviewed published material in English and between the years 2001 and 2011 to maintain currency of	The aim of this review is to summarise the literature published around facilitating reflection in a medical course, and to answer the question : What is the current evidence regarding learning and development moments across the medical curriculum in developing students' reflective practice?	From abstract -- Discussion: A variety of reflective purposes was found in this literature review. Evidence indicates that, if students are unclear as to the purpose of reflection and do not see educators modelling reflective behaviours, they are likely to undervalue this important skill regardless of the associated learning and development opportunities embedded in the curriculum. From results: Thirty-six relevant articles were found. These articles were classified by methodology (see [Table 1]. Relevant references identified). As can be seen in [Table 1], this review included many opinion pieces, reviews and descriptions of methods of facilitating reflection, but little in regards to primary data in the area. Qualitative methodology was the main research output, providing largely descriptive data of

For peer review only

No.	Title	Author/year	MERSQI	COREQ	Reason for inclusion			Undergraduate (UG)/Postgraduate (PG)	Methodology	Purpose of study	Key findings
					Communication	Ethics	Professionalism				
									practice. The primary search yielded 129 references of eligible papers, including research papers, reviews, formal reviews and opinion pieces. Abstracts were read by the first author, and only articles pertaining to facilitating or teaching reflection in undergraduate or postgraduate medical courses were included. The first author read and classed all included articles, categorising them into construct sub-categories. At several stages in this process all authors reviewed the validity and legitimacy of the classifications. This led to the sub-headings and analysis presented below.		the facilitation of reflection. The quantitative research was also largely descriptive, with survey methodology being the most popular in the articles found. This review found only two case-control studies comparing facilitation methods of reflection. The literature presented enhancing factors and barriers to effectively teaching reflective practice within medical curricula, related to: The breadth of the meaning of reflection; Facilitating reflection by medical educators; Using written or web-based portfolios to facilitate reflection; and Assessing the reflective work of students. - The breadth of the meaning of reflection: The term 'reflection' is widely mentioned within medical education literature, but the purposes and goals described indicate a breadth of purposes of reflection. [14] Mann et al.[10] concluded from their systematic review of reflective practice in the health professions that the nature of reflection makes it difficult to quantify. From this current review, we determined that the breadth of reflection covers the purposes of reflecting to improve judgements, to personally develop and, and to contextualise practice, and are explained in [Table 2]. Reflection to improve judgement centres on improving students' clinical skills, problem solving, [11] flexible thinking, lifelong learning and the development of expertise.

For peer review only

No.	Title	Author/year	MERSQI	COREQ	Reason for inclusion			Undergraduate (UG)/Postgraduate (PG)	Methodology	Purpose of study	Key findings
					Communication	Ethics	Professionalism				
											[1],[10],[14],[17] Reflection for this purpose usually has a problem focus, involves critical thinking, and is commonly indicated by hypothesis testing. Reflection to personally develop is essential in client-centred practice, as doctors need to consider they own values and assumptions, and those of the patient, while making clinical decisions. [18] From reviewing the literature, Sandars [14] asserted that the knowledge of one's values and beliefs is important for a doctor to develop therapeutic relationships with patients, as understanding oneself enables empathy and caring. Aukes et al.[11] reviewed literature in preparation for creating an assessment tool for reflection, and noted that personal reflection has often been overlooked in medical education. They suggested that this has occurred as personal development and has been seen as outside the domain of the problem-solving culture of medical practice, and thus, outside the domain of medical education. Reflection to contextualise practice helps students integrate theory with their own experience. Several authors have hypothesised that this aspect of reflection is essential in developing a sound medical practice, [5] with a lack of reflective ability resulting in poor self-awareness, and thus poor medical practice. [19] Howe et al.[8] found, from their study of the development of

For peer review only

No.	Title	Author/year	MERSQI	COREQ	Reason for inclusion			Undergraduate (UG)/Postgraduate (PG)	Methodology	Purpose of study	Key findings
					Communication	Ethics	Professionalism				
											professionalism, that reflecting on practice and developing self-awareness are important mechanisms of resilience in the face of a demanding profession. Although this breadth of reflective practice makes it an essential skill in medical practice, this very strength can also be a barrier in teaching facilitating reflective practice as there is often ambiguity of the educator's goals in regard to the use of a particular reflective task. [20],[21] Aukes et al.[11] noted that purposes of a reflective tasks were rarely articulated, and students were often unaware of the perspective, reflective purpose or role, they were expected to take. Sanders [14] concluded from a literature review of reflection in medical education that, for students to reflect effectively, they required clarity in determining the appropriate purpose of a reflective task.<br>- Facilitation reflection by medical educators:<br>Reflection is often provoked by uncertainty or unease resulting from an experience. [13],[22] According to Albanese, [22] in addition to this uncertainty or unease, a student requires knowledge of how to reflect, and time and motivation for reflection to occur. Supporting this opinion, Carr and Carmody [23] found in their qualitative study that some students could attain an acceptable level of reflection independently. However, reflection was greatly

For peer review only

No.	Title	Author/year	MERSQI	COREQ	Reason for inclusion			Undergraduate (UG)/Postgraduate (PG)	Methodology	Purpose of study	Key findings
					Communication	Ethics	Professionalism				
											enhanced through medical educators' facilitation. Medical educators' facilitation was found to be useful for reflection in one-on-one meetings with students and in small group tutorials. [9],[24] According to results of a study by Schaub-de Jong et al., [9] educators who wished to encourage reflection needed to model reflective practice by making their experiences more explicit, investigating emotions and stimulating interaction among students. Albanese [22] suggested that medical faculty staff should be trained to articulate their reflections to enhance modelling to students. - Using written or web-based portfolios to facilitate reflection: Traditionally, reflective tasks have been paper based. Recently, web-based methods and social media have been adopted to facilitate reflective skills. Cook [29] asserted that there has been little study of the efficacy of these methods. Fischer et al. [30] compared content, depth of reflection and student preference for web-based versus traditional essay-style reflections in two medical schools in the USA. Ninety-five students were quasi-randomly assigned to one of two study groups, where one group wrote a traditional reflective essay and attended a small-group discussion, while the other group posted two writings in a group blog and commented on a peer's post. The writings were

For peer review only

No.	Title	Author/year	MERSQI	COREQ	Reason for inclusion			Undergraduate (UG)/Postgraduate (PG)	Methodology	Purpose of study	Key findings
					Communication	Ethics	Professionalism				
											coded for theme, and level of reflection, with the authors finding no difference between groups for either variable.<br>From discussion:<br>Traditionally, paper-based methods of reflection have been popular with educators, [7],[8],[25] but recently, web-based methods have been adopted. Driessen et al. [36] found that the method of reflection appears to be less important than the guidelines provided, which should be structured initially, and become more fluid as students' reflective skills progress. Ideally, students should be able to revisit and edit the reflection, hence web-based methods may be suitable for this purpose.<br>- Assessing the reflective work of students: ... (see relevant columns)<br>From discussion:<br>Several authors noted that reflection was an essential skill in medical practice, [2],[5],[7],[10],[14],[34] and advocated for training in reflective processes. [13],[22] Training in reflective practice can be significantly enhanced with the facilitation of skilled mentors, providing guidance and modelling of reflective behaviours, as found by Carr and Carmody. [23] Several studies and literature reviews report inconsistencies regarding the purpose of reflection, which was found to be a significant barrier to the facilitation of reflective practice. [11],[20],[21],[35] The implication for educators is that students

For peer review only

No.	Title	Author/year	MERSQI	COREQ	Reason for inclusion			Undergraduate (UG)/Postgraduate (PG)	Methodology	Purpose of study	Key findings
					Communication	Ethics	Professionalism				
											need to be given clear guidelines as to the purpose for each individual reflective task. Traditionally, paper-based methods of reflection have been popular with educators, [7],[8],[25] but recently, web-based methods have been adopted. Driessen et al. [36] found that the method of reflection appears to be less important than the guidelines provided, which should be structured initially, and become more fluid as students' reflective skills progress. Ideally, students should be able to revisit and edit the reflection, hence web-based methods may be suitable for this purpose. Students need to have the designated time and motivation for reflective practice. A common method of increasing task motivation in the education setting is to include the task as an assessment piece. There was some debate in the literature about the efficacy of this method. Some authors found that students' quality of reflection would be diminished if undertaken for an assessment task, [28] whereas others reported that reflection simply would not happen if not attached to an assessable piece of work. [22],[33] It is apparent from this review that the research surrounding the teaching and facilitation of reflection is generally weak. Although there are some descriptive data, there is very little empirical evidence of the efficacy of facilitation methods. Additionally, there

For peer review only

No.	Title	Author/year	MERSQI	COREQ	Reason for inclusion			Undergraduate (UG)/Postgraduate (PG)	Methodology	Purpose of study	Key findings
					Communication	Ethics	Professionalism				
											is a lack of rigorous research investigating the impact of reflection on practice skills or patient outcomes. A major limitation of literature is the breadth of the construct of reflection. A more defined construct of reflection, with clear outcomes, could lead to the development of benchmarks useful in tracking student progress and as research outcome measures. From conclusion: Most authors cited in this review suggested that the emphasis on reflection is increasing in medical curricula. The construct of reflection was broad in the reviewed literature. This suggests that medical educators must be clear on the purpose of reflection, the intended outcome for the individual student and the process necessary to achieve these aims for students. Evidence indicated that, if students are unclear as to the purpose of reflection and do not see educators modelling reflective behaviours, they are likely to undervalue this skill regardless of the associated learning and development opportunities embedded in the curriculum.[40]
66	Beyond assessment of learning toward assessment for learning: educating tomorrow's physicians	Dannefer, E. F. 2013	-	-	✓			UG	From abstract: Information-rich assessment data, structured opportunities for reflection, and facilitated self-assessment using a portfolio approach are designed to support	From abstract: We describe a program (based at Cleveland Clinic Lerner College of Medicine) in which explicit performance standards align these components and provide a roadmap for students to manage their learning.	From abstract: Preliminary evidence suggests that the program directs students towards learning, rather than on achieving a grade for grade's sake.

No.	Title	Author/year	MERSQI	COREQ	Reason for inclusion			Undergraduate (UG)/Postgraduate (PG)	Methodology	Purpose of study	Key findings
					Communication	Ethics	Professionalism				
									development of habits of reflective practice. Promotion depends on the achievement of competencies rather than grades. From the essential building blocks: All components and processes of a system need to be designed to interact in a way that accomplishes the goal of enhancing learning and habits of reflective practice. Key features of the CCLCM assessment system are now presented by addressing issues of competency standards, ongoing formative assessment, student responsibility, and purposeful support.		
67	Evidence within a portfolio-based assessment program: what do medical students select to document their performance?	Dannefer, E. F., Bierer, S. B. and Gladding, S. P. 2012	7		✓		✓	UG	From abstract: We reviewed portfolios to examine the number, type, and source of assessments selected by students (n = 32) to document their performance in seven competencies. The quality of assessment data selected for each	From abstract: In this article, we examine the nature of evidence selected by first-year medical students to include in a portfolio used to make promotion decisions. From introduction: This article attempts to address this gap in the literature by examining the types and sources of assessment	From abstract -- Results: Findings indicate that students cited multiple types and sources of available assessments. The promotion committee rated evidence quality highest for competencies where the program provided sufficient evidence for students to cite a broad range of assessments. When assessments were not provided by the program, students cited self-generated evidence.

For peer review only

No.	Title	Author/year	MERSQI	COREQ	Reason for inclusion			Undergraduate (UG)/Postgraduate (PG)	Methodology	Purpose of study	Key findings
					Communication	Ethics	Professionalism				
									competency was rated by promotion committee members (n = 14). From methods: We used two procedures to explore the assessment evidence that one cohort of medical students cited in their Year 1 summative portfolios. First, we examined the evidence cited in portfolio essays to ascertain what types and sources were selected. Second, we obtained feedback from promotion committee members concerning the quality of evidence via a questionnaire. - Citation coding procedures and data: A research assistant extracted essays from the e-Portfolio database for seven of the nine competencies included in the Year 1 summative portfolios that students submitted during the 2009–2010 academic year. Essays for the competencies of Personal Development and	evidence first-year medical students select from their performance database to construct portfolios used for promotion decisions. We describe our assessment program's evidence database before presenting the organizational elements designed to help students purposefully select data representative of their performance. We then describe the methods and results of the study before discussing implications about students' ability to select evidence within a centralized assessment program.	Conclusion: We found that when student-constructed portfolios are part of an overall assessment system, students generally select evidence in proportion to the number and types of assessments available.

No.	Title	Author/year	MERSQI	COREQ	Reason for inclusion			Undergraduate (UG)/Postgraduate (PG)	Methodology	Purpose of study	Key findings
					Communication	Ethics	Professionalism				
									Reflective Practice were excluded to ensure student confidentiality. Students' names on essays were replaced with a unique six-digit identifier. Overall, 224 essays for 32 students were reviewed for these seven competencies. Sophia Gladding sorted the essays by competency. She then read each set of essays nested within a given competency and manually recorded on a data extraction form all college-generated assessments, work samples and personal evidence students cited to document their performance of Year 1 standards. Extraction form data were entered into SPSS version 16.0, and descriptive statistics were computed after averaging across students' portfolios.<br>- Quality ratings: Promotions committee members involved with portfolio reviews (n = 18 faculty)		

For peer review only

No.	Title	Author/year	MERSQI	COREQ	Reason for inclusion			Undergraduate (UG)/Postgraduate (PG)	Methodology	Purpose of study	Key findings
					Communication	Ethics	Professionalism				
									completed a 21-item, web-based questionnaire after reading students' Year 1 summative portfolios. The questionnaire had been designed (Beth Bierer and Elaine Dannefer) and piloted earlier to monitor the Year 1 portfolio review process. This instrument included seven Likert-scaled items ranging from 1 "poor" to 4 "excellent" which asked promotion committee members to rate the overall quality of the assessment evidence they read for each competency after judging students' summative portfolios. Percentages were computed for each competency to summarize the committee's global ratings ("excellent" and "good/excellent") of evidence quality.		
68	The portfolio approach to competency-based assessment at the Cleveland Clinic Lerner College of Medicine	Dannefer, E. F. and Henson, L. C.<br><br>2007	-	-	✓		✓	UG	-	From abstract: This article describes how the portfolio was developed to provide both formative and summative assessment of student achievement in relation to the program's nine	Design of the portfolio assessment system: The search for an assessment system consistent with our principles led us to the literature on portfolios for medical student assessment14 –15,21–23 and to firsthand investigation of experiences

For peer review only - http://bmjopen.bmj.com/site/about/guidelines.xhtml

For peer review only

No.	Title	Author/year	MERSQI	COREQ	Reason for inclusion			Undergraduate (UG)/Postgraduate (PG)	Methodology	Purpose of study	Key findings
					Communication	Ethics	Professionalism				
										competencies. From introduction: In this article we contribute to the literature on competency-based assessment by describing a portfolio assessment system developed as an integral component of the new Cleveland Clinic Lerner College of Medicine (CCLCM) of Case Western Reserve University In this article, we describe the design of CCLCM's portfolio approach for a comprehensive, competency-based assessment system that is fully integrated with the curriculum. To provide context, we describe the program's competencies, performance standards, core assessment principles, and unique assessment instrument before addressing the design of the overall assessment system.	with portfolio assessment within and beyond national, disciplinary, and professional borders. At the time (2002– 2003), the portfolio assessment approach was being implemented in the United States in grades K–12 and at the undergraduate college level24 –26 and in the United Kingdom and Europe at a number of medical schools. For example, at the University of Dundee College of Medicine, as a key component of the final examination process, each final-year medical student was required to submit a portfolio documenting his or her progress in achieving the outcomes of the curriculum.14,15 The first-year medical student portfolio at the University of Maastricht Faculty of Medicine included students' reflective essays on their own development in four identified roles of the doctor as a method of encouraging integration of competencies.22 An Answer to the Challenges of Competency-Based Assessment: In our opinion, this system and its components should be applied in other settings across the medical education continuum because major themes in the current debate about assessment in medical education, such as integration across competencies, competency-based assessment methods, sampling from multiple contexts and multiple sources,

For peer review only

No.	Title	Author/year	MERSQI	COREQ	Reason for inclusion			Undergraduate (UG)/Postgraduate (PG)	Methodology	Purpose of study	Key findings
					Communication	Ethics	Professionalism				
											triangulation of information, and training of learners in reflective practice are addressed in this innovative method of student assessment.
69	Supporting students in self-regulation: use of formative feedback and portfolios in a problem-based learning setting	Dannefer, E. F. and Prayson, R. A.<br><br>2013		11	✓		✓	UG	From abstract: Formative feedback on works habits and interpersonal skills provided by peers and tutors to a Year 1 cohort (n 1⁄4 32) over the course of a year-long PBL experience (5 blocks) was examined for comments on targeted areas for improvement (TAFIs) and observed improvements. We examined congruence between PBL feedback and students' self-reported TAFIs and behavioral improvements in their assessment portfolios.<br>From study design and methods:<br>- Study design: This study presents an analysis of (1) narrative feedback on professionalism provided to Year 1 medical students from PBL peers, tutors, and self and (2) summative portfolio essays to	From abstract: This study examines the extent to which students self-regulate professionalism behaviors related to work habits and interpersonal skills in a PBL setting.<br>From introduction: In 2004, Cleveland Clinic Lerner College of Medicine (CCLCM) implemented a program designed specifically to promote the development of self-regulated learners (Dannefer & Henson 2007). ... We confront the question of whether or not students use CCLCM's learning environment to self-regulate performance by focusing on the core competency of professionalism.<br>We selected for this study of SR the content of PBL feedback on professionalism and its use by students to identify learning needs (self-assess) and document improvement (regulate). We asked these questions:<br>(1) To what extent did the content of peer and tutor PBL	From abstract -- Results: Both PBL peer and faculty feedback and portfolio self-assessments targeted Interpersonal Skills TAFIs more frequently than Work Habit-related issues. TAFIs were more frequently identified midway in PBL blocks versus the end. Students reported TAFIs in their portfolio essays, citing feedback from both peers and tutors, and provided evidence of improved performance over time.; Conclusions: Students utilized external formative feedback to document their portfolio self-assessment in a system designed to support self-regulation of PBL professionalism-related behaviors. A decrease in TAFIs identified at the end of PBL blocks suggests students made use of mid-block feedback to self-regulate behaviors.<br>From results:<br>- (1) To what extent did the content of peer and tutor PBL narrative feedback provide information relevant to the professionalism competency standards? -- Table 1 summarizes the content of aggregate PBL tutor and peer feedback as well as PBL self and portfolio self- assessments. Of all TAFIs identified by tutors, Interpersonal Skills account for 63%. Interpersonal Skills also dominated peer statements (57%). The majority of tutor

For peer review only

No.	Title	Author/year	MERSQI	COREQ	Reason for inclusion			Undergraduate (UG)/Postgraduate (PG)	Methodology	Purpose of study	Key findings
					Communication	Ethics	Professionalism				
									determine how students used PBL feedback to support their self-assessments. We limited the study to year one the time period reported by students, and supported by advisors' observations, as requiring significant adjustments in terms of taking responsibility for their own learning.<br>- Measures: Faculty defined measurable, behavioral competency standards developmentally appropriate for Year 1 students and observ- able across multiple settings. ... Self-regulation, as a process, consists of overlapping and recursive activities of which we targeted self-assessments that identified areas needing improvement and cited evidence that documented improved performance.<br>- Data sources: We collected peer and tutor PBL assessments electronically at mid and end of Block 1 problem-	narrative feedback provide information relevant to the professionalism competency standards?<br>(2) To what extent did students self-report and document professionalism learning needs and improvements consistent with peer and tutor PBL feedback in a portfolio used for promotion decisions?	and peer TAFI statements about Interpersonal Skills indicated a concern with ''quietness'' (e.g. lack of participation). In the area of Work Habits, peers more frequently identified ''preparedness'' (e.g. disorganized presentations) as a TAFI in fellow students as compared with tutors, who more frequently documented behaviors associated with being ''distracted'' (e.g. carrying on side conversations). Peers were also more likely to give feedback on TAFIs than tutors; of 2016 peer assessments, 41% contained feedback on TAFIs. Overall, tutor feedback tended to be less detailed and only 28% of the 288 possible assessments contained feedback on TAFIs. Similar to tutor and peer evaluations, students self- identified Interpersonal Skills issues as TAFIs more often than Work Habit-related issues (60% vs. 40% respectively). Also similar to peer assessments, students frequently self-identified ''ill-preparedness'' as the Work Habit most needing improvement. In contrast to peer and tutor feedback, however, students more frequently self-identified ''dominating'' behavior issues rather than ''quietness''. All students, except for one who did not complete self-assessments, self-identified TAFIs also reported by their peers and tutors. Interestingly, 95% of students at some point during the year also self-identified issues not documented by peers and

For peer review only

No.	Title	Author/year	MERSQI	COREQ	Reason for inclusion			Undergraduate (UG)/Postgraduate (PG)	Methodology	Purpose of study	Key findings
					Communication	Ethics	Professionalism				
									solving sessions, and from peers, tutors and self at mid and end of Blocks 2-4 and end of Block 5 case-based sessions. Because professionalism cannot be compartmen- talized, students address standards not only in the Professionalism Competency essay but also in the Communication, Personal Development, and Reflective Practice competency essays. Thus, data used in this study included essays and citations for these four competencies. - Coding and analysis of PBL narrative feedback: A trained research assistant extracted the electronic competency report of all PBL feedback for professionalism for all first-year students (n 1⁄4 32) for the 2009 academic year, and replaced students' names with a unique six-digit identifier. We		tutors. Table 1 shows that peer and tutor feedback and self-assessments documented improvements less frequently than TAFIs, and statements noting improvement in Interpersonal Skills were more fre- quent than statements of improvement in Work Habits. Figure 1 documents peer and tutor feedback statements for students at mid and end of the four PBL blocks. In aggregate, more TAFI statements were made by peers and tutors for mid-block assessments than at the end of the block with an overall decrease across the year. Regarding individual student data, 29 of the 32 students had more TAFIs documented at mid-block by peers as compared with end-of-block, and early in the year (Blocks 1–2) versus later in the year (Blocks 3–4). Similarly, more evidence with regard to improvements was documented for students by peers in end-of-block and late year feedback versus mid block and early year feedback, respectively. Similar trends were noted in the tutor feedback for mid- and end-of- block TAFIs and improvement statements. - (2) To what extent did students self-report and document professionalism learning needs and improvements consistent with peer and tutor PBL feedback in a portfolio used for promotion decisions? -- Student self-assessments presented in their summative portfolio essays are required to be a

For peer review only

No.	Title	Author/year	MERSQI	COREQ	Reason for inclusion			Undergraduate (UG)/Postgraduate (PG)	Methodology	Purpose of study	Key findings
					Communication	Ethics	Professionalism				
									developed a rubric using the PBL assessment form criteria for Interpersonal Skills and Work Habits to individually code five competency reports. The authors then met and reached consensus on coded professionalism statements about TAFIs and observed improvements. The refined Professionalism Rubric has three unprofessional behavior criteria each for Interpersonal Skills (Dominating, Quiet, Inappropriate) and for Work Habits (Late, Ill-prepared, Distracted). Each author coded the remaining reports before meeting to reach consen-sus and in rare instances of coding differences; these discrepancies were reconciled. The authors tallied type and number of professionalism statements for each student for each block from peers, tutors and self.		representative and balanced account of their professional behavior across the year. As an indicator of ‘‘representative’’, we identified behaviors that were observed by tutors in at least two different groups, which suggest stability of behavior across time and group dynamics. For the 16 students where two different tutors identified the same TAFI, peers identified the same TAFIs as the tutors in five PBL Blocks for seven students, four PBL blocks for six students, three PBL blocks for two students, two PBL blocks for one student. All 16 students reported the TAFI in their summative portfolio, citing both tutor and peer feedback. All cited evidence of improvement from peers and/or tutors. A strong majority (91%) of portfolio essays followed a pattern of citing feedback from PBL peers and/or faculty to identify at least one TAFI, followed by citations indicating improved performance. To illustrate student’s use of PBL feedback to document performance, we provide the portfolio self-assessment of one student who received ten TAFIs during the first half of the year and three in the second half, indicating a tendency to dominate. The tendency to dominate was mentioned by peers in four of the five PBL groups but never mentioned by tutors. These comments included statements such as ‘‘talking over people,’’ ‘‘can push ideas a little bit too hard ‘‘sometime can seem

No.	Title	Author/year	MERSQI	COREQ	Reason for inclusion			Undergraduate (UG)/Postgraduate (PG)	Methodology	Purpose of study	Key findings
					Communication	Ethics	Professionalism				
									- Coding and analysis of summative portfolio essays: A research assistant extracted the summative portfolio essays from the database for the four competencies (Professionalism, Communication, Personal Development, and Reflective Practice) that 32 students submitted at the end of the 2009 academic year, for a total of 124 essays. The authors used the Professionalism Rubric to analyze the portfolio essays for the aforementioned competencies, where professionalism issues were likely to be addressed. The authors independently coded professionalism statements before meeting to reach consensus in the rare instances where there were differences in coding. All PBL citations used to document the coded statements were tallied by source (tutor, peer, self) and block time period.		argumentative/defensive'' and needs to ''listen to other viewpoints even though they might not coincide with personal views.'' Three other TAFIs were mentioned three or less times and only in the first half of the year. Table 2 provides quotes from this one student's cited PBL feedback, documenting a TAFI (tendency to dominate) and evidence of improvement over the course of year 1. This student acknowledged and reflected on this TAFI in the portfolio essay and provided evidence of improvement, noting that this behavior may need to be ''reestablished'' with each new working group. See Table 3 for excerpt from this student's portfolio. The essay provides evidence relevant to each of our questions. The student identified and acknowledged a prob- lematic pattern of behavior, reflected on the feedback, and used suggestions to ''amend'' behavior, which resulted in improved performance. Not every student described the self- regulation process in such detail, but all acknowledged a behavior identified by peers and/or tutors that was problem- atic and presented evidence of improvement.<br>From discussion: ... While this study suggests that students are using the supports of this educational program to self-regulate, we need to better understand how students process and use feedback, how external feedback triggers and shapes intern- ally

For peer review only

No.	Title	Author/year	MERSQI	COREQ	Reason for inclusion			Undergraduate (UG)/Postgraduate (PG)	Methodology	Purpose of study	Key findings
					Communication	Ethics	Professionalism				
											generated feedback, what role advisors play in helping students reflect on and use feedback, and what processes students use to make judgments and develop plans for improvement, questions being addressed by the work of Sargeant et al. (2008, 2009, 2011). As future physicians, today's students will need to be able to self-regulate their behaviors in settings where feedback may not be as structured or robust and facilitation of reflection lacking. To support development of SR skills, we need medical school programs designed to provide formative feedback and clear standards, opportunities to improve, and facilitated self-assessment. The PBL-portfolio model provides one strategy by which students can gain skills in self-regulation and develop habits useful for future practice.
70	Portfolio assessment in medical students' final examinations	Davis, MH, Ben-David, M Friedman, Harden, RM, Howie, Peter, Ker, Jean, McGhee, C, Pippard, MJ and Snadden, David<br><br>2009	7		✓	✓		UG	From abstract: Portfolio assessment has been developed for this purpose and has been adopted for the summative assessment of students in their final examination in Dundee. The contents of the portfolio and the assessment process have been defined and the first cohort of students to be assessed in this	This paper describes a new approach to the final examination based on portfolios, which has been piloted in Dundee Medical School. This paper describes our first year's experience with this new approach.	From abstract: The evaluation of the approach demonstrated strong staff support. Students were also positive although with some reservations. It is concluded that portfolio assessment is a powerful approach to assessing a range of curriculum outcomes not easily assessed by other methods and is worthy of inclusion in the assessor's toolkit.<br>Results: Of the 129 candidates for the portfolio assessment, three were withdrawn for the reasons given above. Of the 126 remaining, 108 (86%)

No.	Title	Author/year	MERSQI	COREQ	Reason for inclusion			Undergraduate (UG)/Postgraduate (PG)	Methodology	Purpose of study	Key findings
					Communication	Ethics	Professionalism				
									way has been studied. The final examination: The final examination was redesigned to meet the needs of the new curriculum, in particular the moves to an inte- grated, task-based approach and an outcome-based education model. The examination is in two parts. Part 1 is at the end of year 4 and comprises a multiple-choice paper of extended matching items (134) designed to assess knowledge and its application; a constructed response question (CRQ) paper to assess higher-order thinking such as problem solving and critical analysis as well as knowl- edge; and an OSCE to assess clinical skills. The use of an OSCE as part of the final examination has been reported (Preece et al., 1992). Part 2 of the final examination takes place towards the end of year 5 and adopts a more innova- tive		passed, 13 (10%) received a condi- tional pass and five (4%) failed. No students fell into the bad fail category. Individual student profiles, in terms of the 22 grades for the learning outcomes, showed variation. Three students (2%) were awarded 22 A grades. One student (0.8%) was awarded 22 B grades. No student was awarded 22 C, D, E or F grades. Forty-four students (35%) were awarded 22 grades at either A or B levels. Two students (1.5%) were awarded 22 grades at either C or D levels. No students were awarded 22 grades at either E or F levels. Over 60% of the students were not consistent in their ability across the learning outcomes, but had individual strengths and weaknesses. The student gradings awarded by both pairs of exam- iners in each outcome are given in Figure 1. Non-pass gradings are confined to outcomes 2, 7, 8, 11 and 12.<br>Evaluation of portfolio assessment:<br>- (1) Analysis of student results: Numeric values from one to six were allocated to the students' outcome grades in the portfolio assessment. An overall mark was then calculated for every student. This overall mark was correlated, using the Spearman correlation, with the mark for each of the three compo- nents of Part 1 of the final examination (EMI, 0.42; CRQ, 0.42; OSCE, 0.47) and with the mark allocated to students

No.	Title	Author/year	MERSQI	COREQ	Reason for inclusion			Undergraduate (UG)/Postgraduate (PG)	Methodology	Purpose of study	Key findings
					Communication	Ethics	Professionalism				
									approach – portfolio assessment. The approach was adopted as it offered the potential of assessing the students' achievement in each of the curriculum outcomes including those not easily assessed by more traditional approaches. It also offered the potential of reviewing the development of student competence over time and presenting feedback to students. Implementation of the portfolio assessment: Work for the portfolio assessment was carried out in three stages: - Stage 1: Work carried out before the portfolio review -- Towards the end of year 5, before the PRHO preparation block, students submitted their completed portfolios to administrative staff in the medical school office. Following a check for completeness,		in the end-of-block examination for one phase 3 core block attachment (psychiatry, 0.34). These results show low/moderate correlation. - (2) Observer documentation: Three observers, two internal and one external, were appointed to document any issues that arose during the portfolio assessment process. They reported that the port- folio submission procedures worked well. The countersigned receipt given to students was an essential step to avoid disputes over submitted material. Entering the pre-marked grades for various portfolio components into the PASS was a major task for administrative staff and required to be streamlined for future years. ... (see under issues related to implementation of portfolio) The PASS system worked well and provided signed sheets to be retained as a permanent record of the examination. - (53) Examiners' evaluation questionnaire: The examiners' evaluation questionnaire was developed following discussion with members of the working group that introduced portfolio assessment and with both the external and internal examiners who participated in the portfolio examination. It was designed to gather the exam- iners' opinions regarding the: effectiveness of the portfolio approach to student assessment; usefulness of the components of the portfolio

For peer review only - http://bmjopen.bmj.com/site/about/guidelines.xhtml

No.	Title	Author/year	MERSQI	COREQ	Reason for inclusion			Undergraduate (UG)/Postgraduate (PG)	Methodology	Purpose of study	Key findings
					Communication	Ethics	Professionalism				
									they received and countersigned a receipt. The administrative staff transcribed from student records, onto an assessment summary sheet called the Port-folio Assessment and Summary Sheet (PASS), the grades awarded for the portfolio contents in respect of each of the curriculum outcomes. ... Three of the students were identified as ineligible to proceed to the portfolio review with the examiners at this stage, two for reasons of illness and non-completion of course work and one pending a disciplinary hearing. All three, however, were allowed to complete the portfolio review process for formative purposes.<br>- Stage 2: Portfolio review with the examiners -- ... (see under deciding how to mark/assess)<br>- Stage 3: Work completed after the portfolio review -- ... (see		in providing information about the students' abilities; conduct of the examination; standards achieved by the students; strengths and weaknesses of the portfolio approach to assessment; suggestions for improvement. Examiners were asked to rate a number of statements on a Likert-type five-point scale from 5—strongly agree to 1— strongly disagree. There were also free-response questions. The questionnaire was distributed and completed by the examiners at the end of the examiners' meeting on day 2. Thirty-three examiners' questionnaires were distributed and returned, giving a response rate of 100%. Responses were collated using the Excel computer software package by Microsoft. The percentage distribution of examiners' ratings with the number that responded to each statement (N) the calculated average score (AV) and the standard deviation (SD) are provided in Table 1. ... (see the rest under perspective of examiners)<br>- (4) Student evaluation questionnaire: The student evaluation questionnaire was developed following periodic feedback from the students during their phase 3 studies and from a student group discussion with the external observer that took place immediately after the portfolio review. Students were given the opportunity for free and frank expression of their opinions during this discussion. The

For peer review only

No.	Title	Author/year	MERSQI	COREQ	Reason for inclusion			Undergraduate (UG)/Postgraduate (PG)	Methodology	Purpose of study	Key findings
					Communication	Ethics	Professionalism				
									under deciding how to mark/assess) Evaluation of the portfolio assessment: Five methods were employed to evaluate the use of portfo- lios as the basis for the final examination: (1) analysis of student results; (2) observer documentation; (53) examiners' evaluation questionnaire; (4) student evaluation questionnaire; (5) verbal report from student representatives. (see details under results of study).; From discussion - evaluation methodology: Rossi & Freeman (1993a) (p. 13) define evaluation research as 'a robust arena of activity directed at collecting, analysing and interpreting information on the need for, implementa- tion of, and effectiveness and efficiency of intervention efforts'. Education evaluation is more specifically identified as 'the process of making judgement about		student questionnaire was designed to gather opinion about building the portfolio, about its focus around the curriculum outcomes and about events during the portfolio assessment. The questionnaire was distributed and completed 22 days after the examination, at a time when all students were aware of whether or not they had passed the examina- tion. Those students who were unsuccessful had received counselling. The rating scale, data entry and scoring system were similar to those used in the examiners' ques- tionnaire. A total of 107 student questionnaires were returned from 129 students who sat the examination—a response rate of 83%. The percentage distribution of students' ratings, the number that responded to each statement (N), the calcu- lated average score (AV) and the standard deviation (SD) are provided in Table 2 for building the portfolio and in Table 3 for the portfolio assessment. ... (see the rest under perspective of students) - (5) Student representatives' comments: ... (see the rest under perspective of students)

For peer review only

No.	Title	Author/year	MERSQI	COREQ	Reason for inclusion			Undergraduate (UG)/Postgraduate (PG)	Methodology	Purpose of study	Key findings
					Communication	Ethics	Professionalism				
									the merit, value or worth of educational programmes' (Gall et al., 1996). It is recognized that it is often impossible to conduct educational evaluations according to the best possible design and Rossi & Freeman (1993b) advocate the concept of the 'good enough' evaluation. The five methods employed to evaluate the use of portfolios as the basis for the final examination followed the 'good enough' approach.		
71	A Qualitative Analysis of Narrative Preclerkship Assessment Data to Evaluate Teamwork Skills	Dolan, B. M., O'Brien, C. L., Cameron, K. A. and Green, M. M.<br><br>2018		15	✓		✓	UG	From abstract: We performed a qualitative analysis of narrative data in 15 assessment portfolios. Student portfolios were randomly selected from 3 groups stratified by quantitative ratings of teamwork performance gathered from small-group and clinical preceptor assessment forms. Narrative data included peer and faculty feedback from these same forms. Data were	We sought to understand if and how teamwork behaviors are described in the narrative feedback given by faculty and peers in preclerkship years and how this feedback may vary based on students' competency achievement level. We expect that review of assessment data collected in an electronic portfolio and judged by experienced faculty may fill the gap in teamwork assessment by providing a method to evaluate	From abstract -- Results: Eight codes related to teamwork emerged: attitude and demeanor, information facilitation, leadership, preparation and dependability, professionalism, team orientation, values team member contributions, and nonspecific teamwork comments. The frequency of codes and valence varied across the 3 performance groups, with students in the low-performing group receiving more suggestions for improvement across all teamwork codes. Conclusions: Narrative data from assessment portfolios included specific descriptions of teamwork behavior, with important contributions provided by

For peer review only - http://bmjopen.bmj.com/site/about/guidelines.xhtml

No.	Title	Author/year	MERSQI	COREQ	Reason for inclusion			Undergraduate (UG)/Postgraduate (PG)	Methodology	Purpose of study	Key findings
					Communication	Ethics	Professionalism				
									coded for teamwork-related behaviors using a constant comparative approach combined with an identification of the valence of the coded statements as either “positive observation” or “suggestion for improvement.” Study approach and setting: We conducted a qualitative study using a constant comparative approach to better characterize the narrative assessments that students receive regarding teamwork.17 ... We excluded assessments that do not include students' teamwork skills, including assessments from observed structured clinical exams, assessments of written assignments, medical knowledge assessments, assessments of groups as a whole (where no individual feedback was given), ethics oral examinations, direct observations of	longitudinal, behaviorally based narrative feedback regarding student teamwork knowledge, skills, and attitudes.	both faculty and peers. A variety of teamwork domains were represented. Such feedback as collected in an assessment portfolio can be used for longitudinal assessment of preclerkship student teamwork skills and attitudes. From discussion: This analysis demonstrates that narrative feedback in assessment portfolios provides a longitudinal view of preclerkship students' teamwork behaviors. Although similarities exist between performance groups, we observed differences between groups in content, frequency, and valence of feedback. These differences could assist mentor/coaches and competency committee members in identifying both students who excel at teamwork and those needing additional support with teamwork behaviors prior to starting clerkships. Although our coded categories were identified without the use of a predetermined framework, they align with key teamwork and leadership attributes identified in the literature. Critical teamwork skills and attitudes have previously been described that share similarity with our codes: leadership, team orientation, preparation/dependability.21 Physician leadership competencies highlight the importance of showing integrity, listening actively, promoting team building, and nurturing/recognizing others' accomplishments.22–24

For peer review only

No.	Title	Author/year	MERSQI	COREQ	Reason for inclusion			Undergraduate (UG)/Postgraduate (PG)	Methodology	Purpose of study	Key findings
					Communication	Ethics	Professionalism				
									clinical skills, and scholarly project oral presentations (which focus solely on presentation skills). Sampling strategy: Students were notified of the study via e-mail and were encouraged to e-mail an author not involved in grading students (CLO) if they did not wish to participate. Students were given 1 week to respond. Four students chose to opt out of the study. We divided the remaining student portfolios into three groups. We first identified the students who received progressing towards competence with concern or progressing towards competence pending additional development for any of the three competencies most closely related to teamwork—teamwork, professionalism, and communication—during the competency review. We		These attributes arose in our analysis, lending credibility to the argument that narrative data in assessment portfolios can be used to understand students' teamwork behaviors in the preclerkship years and can help students reflect on how their behaviors promote or detract from their teams' overall performance. Review of the preclerkship narrative data provides mentors or coaches the ability to identify students in need of remediation before full-time immersion in clinical practice.25 Our analysis demonstrates that low-performing students share similar characteristics that reviewers can look for poor attitude in small groups, lack of preparation for group activities, or dismissive behavior toward others' contributions. It also demonstrates that the absence of certain comments may be telling, as evidenced by the sharp reduction in even nonspecific comments such as "great team member" or the lack of leadership observations in the low-performing student portfolios. When reviewing assessment data with their students, mentors may choose to highlight these absences to help students improve. Notably, peers provided the majority of feedback in the codes of team orientation and values' contributions of others. Due to their close working relationships during preclerkship years, peers may be in the best position

For peer review only

No.	Title	Author/year	MERSQI	COREQ	Reason for inclusion			Undergraduate (UG)/Postgraduate (PG)	Methodology	Purpose of study	Key findings
					Communication	Ethics	Professionalism				
									designated these 19 students as the low-performing group. As our competency review does not have a comparable high-performance designation, we employed an alternative strategy using the composite grading system. To identify a high-performing cohort of students for comparison, we identified the 19 students receiving the highest numerical ratings for the group of assessments included in Table 1. We designated this to be the high-performing group. The middle-performing group comprised all remaining students (n = 114) who did not meet criteria for the high- or low-performing groups. Four students opted out of the study. To protect privacy, each student portfolio received a random numerical identifier. A key was created linking each identifier to each student's ID number; an		to make such judgments. Furthermore, when peers are given formalized instruction in providing feedback, as they are at our institution, including their insight and feedback is critical when assessing student performance. Our results suggest that assessment systems that do not include peer feedback may miss the opportunity to identify students in need of support for developing teamwork skills. Prior studies demonstrate student acceptance of peer feedback, particularly when used for formative purposes.26 Additional studies that specifically compare faculty versus student feedback may further illuminate the best ways to employ the differing perspectives of peers and faculty when assessing student performance. The use of assessment portfolio data may continue to increase as institutions implement entrustable professional activities, competency committees, and other assessment strategies that require a systems-based approach.27 We believe that the findings in this analysis can assist both in teamwork assessment and as a resource to aid faculty and students in identifying preclerkship goals for teamwork and leadership skills development. Additional study is needed to determine if preclerkship assessment of teamwork correlates with later clinical performance. In conclusion, our analysis of narrative

For peer review only

No.	Title	Author/year	MERSQI	COREQ	Reason for inclusion			Undergraduate (UG)/Postgraduate (PG)	Methodology	Purpose of study	Key findings
					Communication	Ethics	Professionalism				
									author not involved in direct teaching of students (CLO) deidentified all data before analysis. We randomly selected three student portfolios from each group (high, middle, low) to analyze, then further intentionally sampled two additional portfolios in each group to allow for adequate gender inclusion and to confirm emergence of no new codes (see next); these 15 portfolios composed our sample. Data analysis: To better understand how teamwork performance is observed and described by peers and faculty, we used constant comparative analysis techniques. We selected this method as a framework to explore teamwork given that comparison between groups of students may better delineate the similarities and/or differences in how behaviors were described among variably		assessment data from across the preclerkship curriculum demonstrates that these data capture a number of key teamwork skills and attitudes with behaviorally based feedback, thus filling a gap in the longitudinal assessment of teamwork. Providing students and faculty the opportunity to critique and further reflect on behaviorally based narrative feedback may assist students in improving these skills prior to the beginning of clerkships.

For peer review only

No.	Title	Author/year	MERSQI	COREQ	Reason for inclusion			Undergraduate (UG)/Postgraduate (PG)	Methodology	Purpose of study	Key findings
					Communication	Ethics	Professionalism				
									performing students. Constant comparative methodology has been described as an ideal way to "conceptualise the variety that exists within the subject under study.17(p393) After using this methodology to complete the coding, we undertook a content analysis to better understand the prevalence and valence of identified themes across the three performance groups.19 Three research team members (BMD, CLO, MG) with expertise in medical student assessment performed the primary analysis of portfolio comments; two are practicing clinicians with experience on medical teams (BMD, MG). The fourth team member, an expert in qualitative research (KAC), provided guidance and oversight in methodology. Narrative data was uploaded for analysis into		

For peer review only

No.	Title	Author/year	MERSQI	COREQ	Reason for inclusion			Undergraduate (UG)/Postgraduate (PG)	Methodology	Purpose of study	Key findings
					Communication	Ethics	Professionalism				
									NVivo (QSR, Mac version 10.2.0) after deidentification.20 We selected the middle-performing group as the first group for analysis, as it represents the typical student who is neither struggling nor exceptionally high achieving. Consistent with conventional content analysis19 and to best explore what teamwork categories faculty and students choose to comment on in their narratives, we did not use an a priori framework for coding. However, one author (BMD) is familiar with medical teamwork literature,21,22 which has informed the way medical students approach teamwork in our institution and which may have influenced her approach to coding. The second coder has a background in education. She had no specific expertise with the teamwork literature and arrived at the same codes. Two		

No.	Title	Author/year	MERSQI	COREQ	Reason for inclusion			Undergraduate (UG)/Postgraduate (PG)	Methodology	Purpose of study	Key findings
					Communication	Ethics	Professionalism				
									authors (BMD and CLO) initially reviewed three portfolios from the middle-performing group, assigning a code to all narrative statements. These authors then met and achieved consensus regarding the coding framework. The remaining portfolios in the middle-performing group were analyzed using the identified codes; all statements were assigned a valence of either "positive observation" or "suggestion for improvement." Three authors (BMD, CLO, MG) met to review the statements' assigned codes and come to consensus on any differences, both with respect to code and valence. Differences were resolved via group discussion. Using the codes identified, we then analyzed the low- and high-performing groups, representing the extremes of performance.		

No.	Title	Author/year	MERSQI	COREQ	Reason for inclusion			Undergraduate (UG)/Postgraduate (PG)	Methodology	Purpose of study	Key findings
					Communication	Ethics	Professionalism				
									Although no new codes were identified in the high-performing or low-performing groups, the narrative from these groups helped to refine the coding set to reflect nuances that emerged from the other groups. For example, after reviewing the high-performing narrative data, the original code “information sharing” was revised to “information facilitation” to better reflect the variety of ways that students mediated the flow of information in small groups. The code “values contributions of team members” evolved from “values team members” after review of the additional suggestions for improvement found in both the high- and low-performing groups. We reviewed three portfolios in each performance group but identified few new codes after the second student's portfolio in each group. However,		

For peer review only

No.	Title	Author/year	MERSQI	COREQ	Reason for inclusion			Undergraduate (UG)/Postgraduate (PG)	Methodology	Purpose of study	Key findings
					Communication	Ethics	Professionalism				
									we continued with two additional portfolios per group for adequate gender inclusion and to confirm the emergence of no new codes; no new codes were found with the addition of these six portfolios. Upon completion of the coding process, NVivo software was used to quantify the number of comments per code and valence. Techniques to ensure trustworthiness of data included the use of two coders followed by additional review of the codes by a third author. Codes and examples were also discussed at a medical education group research meeting where additional faculty members with less connection to the portfolio process provided feedback to the research team.		
72	Portfolio as a learning tool: students' perspective	Elango, S., Jutti, R. C. and Lee, L. K.<br><br>2005	6.5		✓			UG	From abstract: A questionnaire survey was conducted among 143 medical students to find out their perceptions of the	This study was formulated to find out students' perspectives on portfolio learning.	From abstract: ... (see the rest under perspective of students) The study indicates that students need appropriate guidance from the academic staff for the system to succeed.<br>From introduction: In the

For peer review only

No.	Title	Author/year	MERSQI	COREQ	Reason for inclusion			Undergraduate (UG)/Postgraduate (PG)	Methodology	Purpose of study	Key findings
					Communication	Ethics	Professionalism				
									portfolio as a learning tool. From materials and methods: Feedback was obtained from an entire batch of Semester 8 students by asking them to write down what they felt about writing a portfolio. Based on their feedback, a questionnaire consisting of 23 questions was designed. The entire student population (259) in the clinical school (from Semester 6 to Semester 10) was given an information sheet with details about this survey and was given the option of participating in the study. The students who volunteered for the study were asked to sign a written consent form. The students who gave their consent for the survey were requested to complete the questionnaire anonymously. For each of the 23 questions, students had to respond on a 4-point Likert scale, ranging from		last decade, there have been numerous innovations in educational theory and practice. Education has moved from being a traditional teacher-centred process to one that is student-centred. Similarly, in the area of assessment, there has been a shift away from assessing knowledge towards a more competency/performance-based assessment. There is now a wide variety of methods of education and training to choose from and portfolio-based learning is an increasingly popular option among educationists.1<br>From results: One hundred and forty-three students completed and returned the questionnaire (Table1). There were 76 (53.1%) females and 64 (44.8%) males. In order to facilitate the analysis, the 4 levels of perception in the questionnaire – strongly agree, agree, disagree and strongly disagree – were regrouped; strongly agree and agree were grouped as one, and strongly disagree and disagree were grouped as the other. ... (see the rest under perspective of students)<br>From discussion: A study has shown that students using portfolios obtain higher marks in factual knowledge compared to the control group of students not using portfolios11<br>From conclusion: The study demonstrates that any medical school intending to introduce the portfolio as a new learning method must take into consideration an

No.	Title	Author/year	MERSQI	COREQ	Reason for inclusion			Undergraduate (UG)/Postgraduate (PG)	Methodology	Purpose of study	Key findings
					Communication	Ethics	Professionalism				
									strongly agree to strongly disagree. The responses to the questionnaire were analysed to determine the overall views of the students on the different aspects of portfolio writing.		effective orientation programme for both the learners and the teachers. Motivated students, and trained and motivated staff are essential for portfolio-based learning to succeed.
73	Students' reflections in a portfolio pilot: highlighting professional issues	Haffling, A. C., Beckman, A., Pahlmblad, A. and Edgren, G. 2010		14	✓	✓	✓	UG	From abstract: Thirty-five voluntary final-year medical students piloted a standardized portfolio in a general practice (GP) attachment at Lund University, Sweden. Students' portfolio reflections were based upon documentary evidence from practice, and aimed to demonstrate students' learning. The reflections were qualitatively analysed, using a framework approach. Students' evaluations of the portfolio were subjected to quantitative and qualitative analysis. Pilot: Students of two consecutive classes, of 76 and 71 students, respectively, were approached. Twenty students in each class	From abstract: To investigate whether students' reflections include sufficient dimensions of professional competence, notwithstanding a standardized portfolio format, and to evaluate students' satisfaction with the portfolio From introduction: We aimed to develop a portfolio for the assessment of final-year students' professional development during their general practice (GP) attachment. ... We designed a pilot study with the principal aim of investigating whether themes of students' reflections would include satisfactory dimensions of professional competence, notwithstanding the standardized format. This article qualitatively explores the emergent themes of students' reflections, and also reports on students' evaluation of the portfolio.	From abstract -- Results: Among professional issues, an integration of cognitive, affective and practical dimensions in clinical practice was provided by students' reflections. The findings suggested an emphasis on affective issues, particularly on self-awareness of feelings, attitudes and concerns. In addition, ethical problems, clinical reasoning strategies and future communication skills training were subjects of several reflective commentaries. Students' reflections on their consultation skills demonstrated their endeavour to achieve structure in the medical interview by negotiation of an agenda for the consultation, keeping the interview on track, and using internal summarizing. The importance of active listening and exploration of patient's perspective was also emphasized. In students' case summaries, illustrating characteristic attributes of GP, the dominating theme was 'patient-centred care', including the patient–doctor relationship, holistic modelling and longitudinal continuity. Students were satisfied with the portfolio,

For peer review only

No.	Title	Author/year	MERSQI	COREQ	Reason for inclusion			Undergraduate (UG)/Postgraduate (PG)	Methodology	Purpose of study	Key findings
					Communication	Ethics	Professionalism				
									were offered to voluntarily pilot a portfolio in GP as an addition to the written test, which for the participants would be reduced in the number of GP questions. A total of 35 students, 16 from the first and 19 from the second class, accepted. Twenty-four (69%) of them were women, as compared to 59% of all students. At the time of the study, consent from the University's ethics committee was not required for projects involving students' assessments. Participating students were provided with both verbal and written guidelines on the purpose, format and assessment of their portfolios. Three of the authors (ACH, AP, AB) and a fourth teacher, all involved in teaching, acted as mentors to support their students in compiling the portfolio. Qualitative analysis of students' reflections: To be		but improved instructions were needed.; Conclusions: A standardized portfolio in a defined course with a limited timeframe provided ample opportunities for reflections on professional issues. Support by mentors and a final examiner interview contributed to the success of the portfolio with students. The interview also allowed students to deepen their reflections and to receive feedback. Students' evaluation questionnaire: see under perspective of students From discussion: Firstly, students' reflections on their consultation skills showed that the most important learning issue in the theme of 'process skills' was how to achieve structure in the medical interview. Patient-centred communication can cause genuine concerns in students about losing control of the consultation and getting caught in a flow of less ordered information from the patient (Silverman et al. 2004). When students learned how to obtain structure, they also experienced better time management, enhanced self-confidence and an improved rapport with the patient. Secondly, the theme of 'patient-centred care' was emphasized in students' case summaries. Patient-centred care is a major principle in GP, but is of essential interest to several other medical disciplines as well. Achieving shared decision making, understanding the patient as

No.	Title	Author/year	MERSQI	COREQ	Reason for inclusion			Undergraduate (UG)/Postgraduate (PG)	Methodology	Purpose of study	Key findings
					Communication	Ethics	Professionalism				
									able to conduct the qualitative analysis, students' reflections were written verbatim into a word processor by one of the authors (173). Each student was allocated a code number to prevent identification of individual students' comments. The text of the reflections constructed two separate units of analysis; 'consultation skills' and 'case summaries'. The transcripts were read through several times to get a sense of the whole, and then analysed using a framework approach (Crabtree & Miller 1999). For 'consultation skills', the text was mapped against the Calgary Cambridge Guides (Silverman et al. 2004). For 'case summaries', the statement of WONCA Europe (2005), (Box 2), was used as a template for organizing the data. In each of		a whole person and exploring the patient's perspective are aspects shown to positively influence patients' satisfaction and enablement, reduce their symptom burden and discomfort, and increase efficiency of care (Stewart et al. 2000; Little et al. 2001). We believe that these central dimensions of patient-centred care ought to be highlighted earlier in hospital clerkships. Thirdly, the findings suggested integration of the cognitive, affective and practical dimensions of professional competence that can be acquired in a clinical learning environment (Mann et al. 2007; van Tartwijk & Driessen 2009; Passi et al. 2010). As expected, students reflected on their 'content skills' and provided evidence for their identification of deficits in knowledge and skills, their integration of new understanding, and their plans for development. However, 'perception skills' was a more prominent theme in both units of analysis. In their self-awareness of feelings, attitudes and concerns, students were unexpectedly open and frank about their deficiencies and about how they had tried to remedy them. They also acknowledged the importance of ethical issues and additional communication skills training, and openly reflected on their clinical reasoning. However, we found it remarkable that

For peer review only - http://bmjopen.bmj.com/site/about/guidelines.xhtml

No.	Title	Author/year	MERSQI	COREQ	Reason for inclusion			Undergraduate (UG)/Postgraduate (PG)	Methodology	Purpose of study	Key findings
					Communication	Ethics	Professionalism				
									the two units of analysis, related segments of text were identified and indexed to the appropriate part of the thematic framework. Some codes were modified and new ones added to reflect as many nuances in the data as possible. 'Chunks' of related text under each code were examined together and distilled summaries made; this process involved a considerable amount of abstraction. The chunk summaries provided an analytical tool from which connections were made and sub-themes and themes identified. ACH undertook the mapping of text and carried out the primary analysis of the data. The four teachers, involved in the pilot, examined in recurrent meetings whether the interpretation of the data was plausible. The codes and sub-themes were compared based on differences		several students, in their final year in medical school, reflected on their initial problems in transferring theory to practice; a skill supposed to be frequently trained during previous hospital clerkships. From conclusion: In summary, this study demonstrated that a structured portfolio in a short timeframe provided abundant opportunities for students to reflect on personal and professional issues, notwithstanding the standardized format. We strongly recommend a final examiner interview to deepen students' reflections, to supply feedback and to raise the importance of reflective ability as a skill in future learning from practice.

For peer review only

No.	Title	Author/year	MERSQI	COREQ	Reason for inclusion			Undergraduate (UG)/Postgraduate (PG)	Methodology	Purpose of study	Key findings
					Communication	Ethics	Professionalism				
									and similarities, and some were revised during this process of corroboration. Students' evaluation questionnaire: The short student evaluation form was supplied directly after the examiner interview, asking five questions about the portfolio instructions, the mentor contact and the examiner interview, using a 3-point scale ('yes', 'to some extent', 'no'). Students were also requested to provide free comments on positive and negative experiences of the portfolio, and to give suggestions for improvements. The results were subjected to quantitative and qualitative (content) analyses.		
74	Undergraduate medical students' views about a reflective portfolio assessment of their communication skills learning	Rees, C. and Sheard, C.<br><br>2004	10		✓			UG	From abstract: 178 second-year medical students at the University of Nottingham completed the 18-item reflective portfolio questionnaire (RPQ) (α = 0.716) and a personal details questionnaire	this study explores second-year medical students' views about a reflective portfolio assessment of their communication skills. The research questions for this study are: 1) What are students' views of a reflective portfolio assessment	From abstract -- Results: Total scores on the RPQ ranged from 40 to 75 (mean 58.28, SD 7.08). Significant relationships existed between RPQ total scores and students' ratings of their reflection skills (r s = 0.322, P < 0.001), RPQ total scores and students' confidence building another portfolio (T = 4.381, d.f. = 176, P

For peer review only - http://bmjopen.bmj.com/site/about/guidelines.xhtml

No.	Title	Author/year	MERSQI	COREQ	Reason for inclusion			Undergraduate (UG)/Postgraduate (PG)	Methodology	Purpose of study	Key findings
					Communication	Ethics	Professionalism				
									three days before submitting their portfolio assessment for communication skills. Data were analysed using univariate and multivariate statistics on SPSS Version 10.0. From methods: Participants -- Second-year medical students at the University of Nottingham completed a personal details questionnaire (PDQ) and the reflective portfolio questionnaire (RPQ) three days before submitting their portfolio assessment for communication skills.; Measures - - The PDQ contains 13 demographic and education-related items. The RPQ was designed by CR to explore medical students' views of portfolios and reflection. Eighteen items were generated using previous research literature,1-4 e.g. 'I have felt anxious about sharing my weaknesses in my reflective portfolio' (item 16). Each item	of their communication skills? 2) Do relationships exist between students' views of reflective portfolios and their perceived and actual reflection skills and their confidence building portfolios?	< 0.001), and RPQ total scores and students' marks for their reflective portfolio assessment (r s = 0.167, P = 0.029). Students with more positive views about reflective portfolios were more likely to rate their reflection skills as good, receive better marks for their portfolio assessment, and be more confident building another portfolio. Discussion: This study begins to highlight preclinical medical students' views about reflective portfolios. However, further research is required using qualitative studies to explore students' views in depth. Medical educators should be encouraged to consider introducing portfolios as a method of formative and summative assessment earlier in the medical curriculum. From results: - Participant characteristics: Of the 206 students invited to participate in this study, 178 (86.4%) completed the reflective portfolio questionnaires satisfactorily. Students ranged in age from 18 to 30 years (median = 20, interquartile range = 19–20). The majority were female (n = 105, 59%), white (n = 130, 73.4%) and came from nonmanual socio-economic classes (n = 170, 95.5%). Students' ratings of their communication skills ranged from 1 to 5 (median = 3, interquartile range = 3–3) and nearly all (n = 173, 97.2%) thought their communication skills needed improving. Students' ratings of their

For peer review only

No.	Title	Author/year	MERSQI	COREQ	Reason for inclusion			Undergraduate (UG)/Postgraduate (PG)	Methodology	Purpose of study	Key findings
					Communication	Ethics	Professionalism				
									was written in the form of a statement and was accompanied by a 5-point Likert scale from 1 (strongly disagree) to 5 (strongly agree). Six medical educators with a range of experience of reflective portfolios commented on the language used in the preliminary draft of the questionnaire, which resulted in some minor alterations in language. The RPQ is scored by reversing the scores for the negative items and then summing the scores for all 18 items. The total score ranges from 18 to 90, with higher scores indicating more positive views about reflective portfolios. This scale was found to have good internal consistency in this study (Cronbach's α= 0.716) and the study results presented in this paper support the construct validity of the tool (copies of the RPQ can		reflection skills ranged from 1 to 5 (median = 3, interquartile range = 2–3) and most (n = 155, 87.6%) thought their reflection skills needed improving. ... (see the rest under perspective of students) Students' scores for their reflective portfolio coursework ranged from 33 to 100% (median = 67, interquartile range = 60–73%).<br>- Students' views about reflective portfolios: see the rest under perspective of students<br>From discussion: ... (see the rest under perspective of students) These findings have implications for further research and educational practice. Qualitative studies are needed to explore in depth the reasons why students possess the views they have about reflective portfolios. Qualitative research may also shed some light on how medical educators could increase students' confidence in building reflective portfolios. This qualitative research could help medical educators improve educational programs using portfolios, so that they are more acceptable to students. In the meantime, this study indicates that the act of constructing a reflective portfolio could increase students' confidence in building another portfolio. Despite portfolios being seen to have weaknesses (e.g. lacking reliability4), medical educators should be encouraged to consider introducing portfolios as a

For peer review only

No.	Title	Author/year	MERSQI	COREQ	Reason for inclusion			Undergraduate (UG)/Postgraduate (PG)	Methodology	Purpose of study	Key findings
					Communication	Ethics	Professionalism				
									be requested by contacting the author). Students' percentage marks for their reflective portfolio assessments were collected at a later date.; Data analysis -- After checking the distribution of continuous variables, data were analysed using univariate (i.e. Spearman's correlation coefficients and T -tests) and multivariate statistics (i.e. stepwise multiple regression) on SPSS Version 10.0.		method of formative and summative assessment earlier in the medical curriculum. Students may then be better placed to negotiate this type of assessment in higher stakes examinations.
75	The reliability of assessment criteria for undergraduate medical students' communication skills portfolios: the Nottingham experience	Rees, C. E. and Sheard, C. E.<br><br>2004	8.5		✓			UG	From abstract: Two independent analysts assessed a random sample of portfolios (n = 100, 49.5%) using criterion-referenced assessment. Students' performances were examined against subjective items in five areas: 1) portfolio structure, 2) level of critical reflection, 3) level of skills development, 4) use of documentary evidence, and 5) use of relevant literature. These	From abstract: This study aims to determine the reliability of assessment criteria used for a portfolio at the University of Nottingham.	From abstract -- Results: The level of agreement between the two raters for the total percentage score was 0.771 (95% CI = 0.678, 0.840), as measured by an intraclass correlation coefficient. The levels of agreement between the two raters for the individual items of the assessment criteria ranged from κ = 0.359 (item 3) to κ = 0.693 (item 4). Discussion: This study provides some support for the summative assessment of portfolios. The findings suggest that discussion and negotiation between independent assessors can enhance the reliability of assessment criteria. Therefore, medical educators are encouraged to use such procedures in the summative assessment

For peer review only - http://bmjopen.bmj.com/site/about/guidelines.xhtml

No.	Title	Author/year	MERSQI	COREQ	Reason for inclusion			Undergraduate (UG)/Postgraduate (PG)	Methodology	Purpose of study	Key findings
					Communication	Ethics	Professionalism				
									subjective judgements were later converted into quantitative scales ranging from 0 to 3 so that interrater reliability could be established. The level of agreement between the two analysts for the total percentage score was established using an intraclass correlation coefficient and for the individual items using weighted kappa coefficients. From methods: Sample -- Of the 202 portfolios submitted in March 2002, a random sample of 100 (49.5%) were photocopied for second marking. This random sample of 100 portfolios (median = 66.7%, interquartile range = 53.3–73.3%) did not differ significantly from the portfolios (n = 102) not chosen for second marking (median = 66.7%, interquartile range = 53.3–73.3%) in terms of their total percentage scores (Z = −0.155, P = 0.877).; Assessment		of portfolios. From results: The level of agreement between the two raters for the total percentage scores, as measured by an intraclass correlation coefficient, was 0.771 (95% CI = 0.678, 0.840). The levels of agreement between the two raters for the individual items, as measured by weighted kappa coefficients, ranged from 0.359 (item 3) to 0.693 (item 4) (see Table 1).

For peer review only

No.	Title	Author/year	MERSQI	COREQ	Reason for inclusion			Undergraduate (UG)/Postgraduate (PG)	Methodology	Purpose of study	Key findings
					Communication	Ethics	Professionalism				
									procedures -- One hundred portfolios were assessed ... Although students were aware of these five areas, they were not give the full assessment criteria outlined in Appendix 1. ... (see the rest under deciding how to mark/assess); Statistical procedures: An intraclass correlation coefficient on SPSS (Version 10.0) was used to establish the level of agreement between the two raters for the total percentage scores. Weighted kappa coefficients on SAS (Release 6.12) were used to determine the levels of agreement between the two raters for the individual items of the assessment criteria.		
76	Portfolio as a tool to evaluate clinical competences of traumatology in medical students	Santonja-Medina, F., Garcia-Sanz, M. P., Martinez-Martinez, F., Bo, D. and Garcia-Estan, J.<br><br>2016	10.5		✓			UG	From abstract: A total of 131 students used the portfolio during their clinical rotation of traumatology. The students' portfolios were blind evaluated by four professors who annotated the existence	From abstract: This article investigates whether a reflexive portfolio is instrumental in determining the level of acquisition of clinical competences in traumatology, a subject in the 5th year of the degree of medicine.<br>From introduction: ...	From abstract: The reliability of the portfolio was moderate, according to the kappa index (0.48), but the evaluation scores between evaluators were very similar. Considering the mean percentage, 59.8% of the students obtained all the competences established and only 13 of the 23 learning outcomes (56.5%) were fulfilled by >50% of the

No.	Title	Author/year	MERSQI	COREQ	Reason for inclusion			Undergraduate (UG)/Postgraduate (PG)	Methodology	Purpose of study	Key findings
					Communication	Ethics	Professionalism				
									(yes/no) of 23 learning outcomes. From methods: A portfolio was designed to assess the level of acquisition of clinical competences, ... - Academic structure: Traumatology is a compulsory subject in the 5th year of the degree, consisting of 55 hours of theoretical classes, of which 28 hours take place in the classroom, 14 hours are of a seminar type, 10 hours are about clinical cases, and 3 hours are about cooperative learning; 21 hours of classes on laboratory skills in seven sessions; and 2 weeks of hospital rotation (50 hours, 10 days). Of these 50 hours, students rotate in the traumatology service of the university hospitals between hospital ward, medical consultation, operating rooms, and emergency services. Only one student is assigned to one professor in the	Thus, in the current article, we report the application of a portfolio tool to assess the level of acquisition of clinical skills and competences in medical students of the Medical School of the University of Murcia (Spain), during the clinical rotations of traumatology and orthopedic surgery. An important part of the study is also devoted to the analysis of the reliability of the portfolio.29	students. Our study suggests that the portfolio may be an important tool to quantitatively analyze the acquisition of traumatology competences of medical students, thus allowing the implementation of methods to improve its teaching. From results: A total of 138 comparisons were made to obtain the kappa index among the four evaluators for each of the 23 learning outcomes. The distribution of frequencies and percentages of agreement, according to the classification of Landis and Koch,29 are shown in Table 3. As it can be seen, out of 138 comparisons, 83 had kappa indexes >0.4 and 44 of them had values >0.6, which indicates that in a 31.9% of the occasions, the agreements between evaluators have been very substantial. Table 4 shows the mean and standard deviation of the kappa index of the four evaluators. It can be seen that the global kappa is 0.48, indicating that the portfolio has a moderate reliability, according to Landis and Koch.29 As an additional way of assessing the reliability of the portfolio, 6 months after the initial evaluation, two of the evaluators revised again the portfolios (second revision or reference). Table 5 shows the level of agreement obtained by each evaluator when both revisions were analyzed. Globally, the differences among evaluators are not very important because kappa indexes show that

For peer review only

No.	Title	Author/year	MERSQI	COREQ	Reason for inclusion			Undergraduate (UG)/Postgraduate (PG)	Methodology	Purpose of study	Key findings
					Communication	Ethics	Professionalism				
									hospital. Table 2 shows the number of days students spent during these rotations.<br>- Portfolio structure: The portfolio was given to all the students during the course (n=131) in a booklet format. After the identification of data, the students read about legal and ethical issues in relation to the need for keeping medical secret and signed conformity. Each day the students were expected to fill up their reflections before leaving the hospital, writing about the clinical activities observed during the day. In addition, the portfolio had to be signed by the assigned professor every day. The students were given enough space to include everything needed, but there were three main paragraphs: ... (see under content of portfolio)<br>- Portfolio evaluation: The portfolios were evaluated by four		evaluators 1 and 4 have a substantial agreement in both revisions, whereas evaluators 2 and 3 have a moderate level of agreement. The level of achievement of the traumatology competences is shown in Table 1. The data show the mean percentage of the four evaluations as well as the number of times the procedures were performed. A total of 59.8% of the students have acquired the competences as expected during their hospital stay. In all, 13 of the 23 learning outcomes (56.5%) have been acquired by >50% of the students. Competence 5 (87.4%) and competence 1 (81.4%) show the greater values, whereas the rest of them show an acceptable level, except competence 7, showing only a value of 26%.<br>From discussion: The current article shows the results obtained with the application of a portfolio to medical students as a tool to obtain information regarding the level of acquisition of the clinical competences of the topic traumatology. One of the important aspects of any portfolio is its reliability, the consistency and accuracy of the assessment tool in measuring students' performance. Our results quite agree with those found by other authors;30,31 although in some studies of nurse students, higher numbers (0.8) have been described.32 To prove that the results were not obtained by chance, the

For peer review only

No.	Title	Author/year	MERSQI	COREQ	Reason for inclusion			Undergraduate (UG)/Postgraduate (PG)	Methodology	Purpose of study	Key findings
					Communication	Ethics	Professionalism				
									professors, three of them professors of traumatology and one a colleague professor from a different area, that is, physiology; all of them were medical doctors. ... (see the rest under deciding how to mark/assess) All the portfolios underwent blind evaluation. All the students agreed and participated enthusiastically in the study.<br>- Statistical analysis: Data analysis was performed using the Statistical Package for the Social Sciences, Version 19. The Cohen's kappa coefficient was used as a statistical measure of interevaluator agreement for qualitative items.29 Frequencies, percentages, means, and standard deviations were also obtained to describe the results.		second review of the portfolios took place 6 months after the initial evaluation in order to see whether the reliability index could be improved. As observed in Table 5, the maximum level of agreement among evaluators is found in competence 6 (observe and assist in surgical treatments). A moderate agreement exists in competences 2, 3, and 4, whereas competences 1, 5, and 7 show a low level of agreement among evaluators. The results of Table 1 show important information regarding the level of acquisition of the competences analyzed. Two of them, competences 1 and 5, related to diagnostic and treatment obtained the greatest value, with an important frequency of occurrence. Next competence in terms of the level of acquisition is number 3 (70%), probably due to the high number of radiographic images viewed in contrast to magnetic resonance images, where it is likely that doctors rely more on the radiologist report, without actually interpreting them. Competence 6 has also a moderate level of acquisition (55.8%), in spite of the fact that almost all students (99.2%) report attendance to operating rooms (2.6 + 1.3 days). This is likely due to the fact that the surgeons do not invite all the students to wash and be prepared to help, an activity we recommend to all our professors. A similar

For peer review only

No.	Title	Author/year	MERSQI	COREQ	Reason for inclusion			Undergraduate (UG)/Postgraduate (PG)	Methodology	Purpose of study	Key findings
					Communication	Ethics	Professionalism				
											reason may be behind the moderate level achieved in competences 2 (47.1%) and 4 (51.5%), showing that not all the students are able to explore or perform maneuvers with the patients and that this may be due to a time limitation problem while in the ward or consultation. We believe that this should be corrected and doctors should allow more time for the students to perform, under the appropriate supervision, exploration, or treatment of the patients. Finally, competence 7 has the lowest level. It is likely related to the fact that students are not allowed to sign discharge reports or other forms, although it is important that they learn from their professors how to do it. In any case, this is the first time that we obtained this quantitative information regarding the clinical stays of the medical students and this will help us to analyze our teaching results and to develop new strategies to enhance the level of clinical competence the students should reach. A problem learned from the application of this portfolio is that many of the learning outcomes were checked without taking into account the number of times these were carried out. The rest of the learning outcomes were quantitative, so we could obtain the number of times the student performed them. For instance, they attended the operating rooms 5.5 + 3.3 times (range, 1–26), and viewed a mean of 2.3 magnetic resonance images

For peer review only

No.	Title	Author/year	MERSQI	COREQ	Reason for inclusion			Undergraduate (UG)/Postgraduate (PG)	Methodology	Purpose of study	Key findings
					Communication	Ethics	Professionalism				
											(range, 1–14). We believe that it is important that the portfolios are designed to collect also the quantitative information, since both quantitative and qualitative information give the real essence of the learning process. A drawback we have seen with the application of the portfolio to clinical students is that there is a random factor affecting the acquisition of the competences. This is due to the fact that the students rotate between different hospitals and at different times of the year. Thus, it depends on the type of medical problems available on the days they are assigned to the hospital; thus they may not see all the problems selected in the portfolio. Clearly, the greater the number of days of hospital stay, the better it is. However, this is not always possible. A better selection of the learning outcomes will also be helpful. Moreover, the portfolio is designed to be an evaluation tool that should be used in conjunction with others (written or oral exams, laboratory evaluations) because it is not possible to find just one tool that is able to assess all the competences.33,34<br>From conclusion: In conclusion, the portfolio is an important tool that has allowed to obtain qualitative and quantitative data regarding the degree of acquisition of traumatology competences by medical students. Although some factors need to be improved, such as the

For peer review only

No.	Title	Author/year	MERSQI	COREQ	Reason for inclusion			Undergraduate (UG)/Postgraduate (PG)	Methodology	Purpose of study	Key findings
					Communication	Ethics	Professionalism				
											inclusion of more quantitative elements, a more comprehensive selection of learning outcomes, and combination with other tools, we believe that the use of this kind of tool by clinical teachers will be useful to ascertain the degree of practical competence reached by their students.
77	Assessing the Development of Medical Students' Personal and Professional Skills by Portfolio	Yielder, J. and Moir, F.<br>2016		6			✓	UG	The implementation process employed a cyclical model that was designed to evaluate and improve the use of the portfolio, that could form the basis of the first cycle of action research that will be continued in the future to improve the initiative (Fig. 1). During 2013, the introduction of the portfolio was monitored, and changes were made in 2014. In 2014, feedback from students indicated the need for further modifications, which are currently being implemented, along with the introduction of an electronic platform. Student feedback was ascertained from the generic end-of-year course evaluation, which included	From abstract: We present the portfolio format along with the process for its introduction and appraise the challenges, strengths, and limitations of the approach within the context of the current literature. We then outline a cyclical model of evaluation used to monitor and fine-tune the portfolio tasks and implementation process, in response to student and assessor feedback.<br>From introduction: This document outlines the learning outcomes that are to be substantively completed by the end of the second postgraduate year in practice. ... This article presents the process for introducing the portfolio, including the challenges, strengths and limitations, evaluation of data to date, and the ways in which student	From abstract: The portfolios have illustrated the level of insight, maturity, and synthesis of personal and professional qualities that students are capable of achieving. The Auckland medical program strives to foster these qualities in its students, and the portfolio provides an opportunity for students to demonstrate their reflective abilities. Moreover, the creation of a Personal and Professional Skills domain with the portfolio as its key assessment emphasizes the importance of reflective practice and personal and professional development and gives a clear message that these are fundamental longitudinal elements of the program.<br>From conclusion:<br>The implementation of a portfolio for the assessment of the PPS domain in the medical program at the University of Auckland has proved to be an important part of the redeveloped curriculum. It has highlighted the importance of PPS topics and has provided an opportunity and incentive for students to reflect on aspects of their personal and professional development, with the aim

For peer review only

No.	Title	Author/year	MERSQI	COREQ	Reason for inclusion			Undergraduate (UG)/Postgraduate (PG)	Methodology	Purpose of study	Key findings
					Communication	Ethics	Professionalism				
									questions relating to the PPS domain. The number of students in each year varied from 199–260 and there was an average response rate of 50%. In addition, the Auckland University Medical Students Association conducted a student evaluation for the purpose of writing a report for the ongoing medical program accreditation, from which key issues were collated and forwarded to the PPS domain coordinators. The eight portfolio assessors also provided suggestions for improvement at the end of 2013 and 2014. Evaluation feedback, modifications, and qualitative data derived from the portfolios are presented in the Findings section. Due to the qualitative nature of the feedback, it was analyzed inductively, through a cross-sectional thematic analysis that sought to	feedback has been addressed.	of improving their practice as doctors. In the process, we have learned several aspects about students that previously may have remained unknown, which has led in some cases to conversations with students that will hopefully make a difference to their learning and development trajectories. We have also seen many high-quality and insightful portfolios that demonstrate a synthesis of personal and professional qualities that are inspiring and which we hope are an indication that these students will develop into excellent doctors. With the increasing use of, and requirement for, the compilation of professional portfolios in the medical profession internationally, especially at postgraduate level, there is real benefit for students in becoming familiar with this process of pregraduation.<br>It has also been a learning process for the domain coordinators introducing the assessment. In particular, we learned the value of a cyclic, flexible, and ongoing process of evaluation that has allowed us to continuously reflect on what is working and what needs to change in order to be responsive to student needs and at the same time create a curriculum change that aims to create better doctors. ... We hope that as students come to trust our responsiveness, they will become increasingly open to the importance of taking on the responsibility for their

For peer review only

No.	Title	Author/year	MERSQI	COREQ	Reason for inclusion			Undergraduate (UG)/Postgraduate (PG)	Methodology	Purpose of study	Key findings
					Communication	Ethics	Professionalism				
									ascertain the strongest themes from across the three sources of feedback.		own learning in the PPS domain.
78	Construct Validity of an Instrument for Assessment of Reflective Writing-Based Portfolios of Medical Students	Kassab, Salah Bidmos, Mubarak Nomikos, Michail Daher-Nashif, Suhad Kane, Tanya Sarangi, Srikant Abu-Hijleh, Marwan<br><br>2020	7		✓		✓	UG	After an extensive literature review and pilot testing of the instrument, two raters assessed the reflective writing-based portfolios from years 2 and 3 medical students (n=135) on three occasions. The instrument consists of three criteria: organization, description of an experience and reflection on the experience. We calculated the reliability of scores using generalizability theory with a fully crossed design and two facets (raters and occasions). In addition, we measured criterion validity by testing correlations with students' scores using other assessment methods.	Assessment of reflective writing for medical students is challenging, and there is lack of an available instrument with good psychometric properties. The authors developed a new instrument for assessment of reflective writing-based portfolios and examined the construct validity of this instrument.	The dependability (Φ) coefficient of the portfolio scores was 0.75 using two raters on three occasions. Students' portfolio scores represented 46.6% of the total variance across all score comparisons. The variance due to occasions was negligible, while the student– occasion interaction was small. The variance due to student–rater interaction represented 17.7%, and the remaining 27.7% of the variance was due to unexplained sources of error. The decision (D) study suggested that an acceptable dependability (Φ = 0.70 and 0.72) can be achieved by using two raters for one and two occasions, respectively. Finally, we found moderate to large effect-size correlations between students' scores in reflective writing-based portfolios and communication skills (r = 0.47) and PBL tutorials (r = 0.50).<br><br>We demonstrated the presence of different sources of evidence that support construct validity of the study instrument. Further studies are warranted before utilizing this instrument for summative assessment of students' reflective writing-based portfolios in other medical schools.
79	Communication skills teaching	Douglas, A. H. Acharya, S. P.		19	✓			UG	This study is a qualitative	Communication skills (194) are vital for	The two main themes: 1. Positivity

No.	Title	Author/year	MERSQI	COREQ	Reason for inclusion			Undergraduate (UG)/Postgraduate (PG)	Methodology	Purpose of study	Key findings
					Communication	Ethics	Professionalism				
	and learning in Nepal; what are medical students' perceptions and experiences? A qualitative study	Allery, L. A.<br><br>2020							evaluation of a CS course in Nepal, exploring the experiences and perceptions of participants. The study aims to also identify aspects that were helpful or not for student learning and areas for potential development.<br><br>A purposive sample of twenty: second, fourth and Intern year students was selected for interview. Data were collected through audio recorded semi-structured interviews following a piloted schedule. Interview transcripts were manually coded and thematically analysed. Codes were arranged into themes and subthemes.	doctors. Indeed, as the most important element of consultations, are highly valued by patients. CS are core, teachable skills, however, have not been widely taught in South Asian medical schools, unlike their western counterparts.<br><br>Patan Academy of Health Sciences, is one of the first in Nepal to have CS central to its' aims and curriculum. CS are taught from the first weeks of medical school and re-enforced during preclinical study (first 2 years). Our study seeks to explore students' perceptions and experiences of CS teaching in this South Asian, Nepal context.	2. Experiential learning. Results demonstrate participants' positive perceptions of CS teaching: believing it is important, effective, relevant and valuable for personal development. Participants identified experiential learning features as valuable for CS acquisition. Intern students recognised CS relevance and requested expanding teaching to clinical years, incorporating challenging communication scenarios.<br><br>This study shows that PAHS' CS course is well perceived and valuable to learners. Experiential learning is powerful for CS development. Expansion of formal, structured CS teaching through all years in a spiral curriculum, should be considered. Violence towards doctors in South Asia is increasing. Students recognised CS teaching's significance in addressing this.<br><br>CS teaching,still in its' infancy in South Asia, is a pressing issue for medical educators here. Our study provides evidence it is well perceived with positive impacts in this context, particularly when employing experiential learning. Medical schools in south Asia should be encouraged to incorporate and strengthen their CS teaching curriculum.
80	Assessment practices in undergraduate	Brits, H.<br>Bezuidenhout, J.<br>Van der Merwe, L. J.	5.5	12	✓			UG	A descriptive cross-sectional study design was	Assessment should form an integral part of curriculum design	All disciplines in the undergraduate medical programme are

For peer review only

No.	Title	Author/year	MERSQI	COREQ	Reason for inclusion			Undergraduate (UG)/Postgraduate (PG)	Methodology	Purpose of study	Key findings
					Communication	Ethics	Professionalism				
	clinical medicine training: What do we do and how can we improve?	Joubert, G.<br><br>2020							used. Qualitative and quantitative data were gathered by means of open- and closed-ended questions in a self-administered questionnaire, which was completed by teaching and learning coordinators in 13 disciplines.	in higher education and should be robust enough to ensure clinical competence.<br><br>This article reports on current assessment practices and makes recommendations to improve clinical assessment in the undergraduate medical programme at the University of the Free State.	represented. They used different assessment methods to assess the competencies required of entry-level healthcare professionals. Workplace-based assessment was performed by 30.1% of disciplines, while multiple-choice questions (MCQs) (76.9%) and objective structured clinical examinations (OSCEs) (53.6%) were the main methods used during formative assessment. Not all assessors were well prepared for assessment, with 38.5% never having received any formal training on assessment. Few disciplines (15.4%) made use of post-assessment moderation as a standard practice, and few disciplines always gave feedback after assessments.<br><br>The current assessment practices for clinical students in the undergraduate medical programme at the University of the Free State cover the spectrum that is necessary to assess all the different competencies required. Multiple-choice questions and OSCEs, which are valid and reliable assessment methods, are used frequently. Poor feedback and moderation practices should be addressed. More formative assessments, and less emphasis on summative assessment, should be considered. Workplace-based and continuous assessments may be good ways to assess clinical competence.

For peer review only

No.	Title	Author/year	MERSQI	COREQ	Reason for inclusion			Undergraduate (UG)/Postgraduate (PG)	Methodology	Purpose of study	Key findings
					Communication	Ethics	Professionalism				
81	**Added value of assessing medical students' reflective writings in communication skills training: a longitudinal study in four academic centres**	Ament Giuliani Franco, C.<br>Franco, R. S.<br>Cecilio-Fernandes, D.<br>Severo, M.<br>Ferreira, M. A.<br>de Carvalho-Filho, M. A.<br><br>2020	10.5		✓			UG	Third-year and fourth-year medical students enrolled in an elective course on clinical communication skills development were assessed using different assessment methods.<br><br>The communication skills course was offered at four universities (three in Brazil and one in Portugal) and included 69 students.<br><br>The students were assessed by a Multiple-Choice Questionnaire (MCQ), an objective structured clinical examination (OSCE) and reflective writing narratives. The Cronbach's alpha, dimensionality and the person's correlation were applied to evaluate the reliability of the assessment methods and their correlations. Reflective witting was assessed by applying the Reflection Evaluation for Enhanced Competencies Tool Rubric	This study describes the development and implementation of a model to assess students' communication skills highlighting the use of reflective writing. We aimed to evaluate the usefulness of the students' reflections in the assessment of communication skills.	The Cronbach alpha for the MCQ, OSCE global score, TS and RS were, respectively, 0.697, 0.633, 0.784 and 0.850. The interobserver correlation for the TS and RS were, respectively, 0.907 and 0.816. The assessment of reflection using the TS was significantly correlated with the MCQ (r=0.412; p=0.019), OSCE (0.439; p=0.012) and RS (0.410; p=0.020). The RS did not correlate with the MCQ and OSCE.<br><br>Assessing reflection through mapping the themes and analysing the depth of reflective writing expands the assessment of communication skills. While the assessment of reflective themes is related to the cognitive and behavioural domains of learning, the reflective depth seems to be a specific competence, not correlated with other assessment methods—possibly a metacognitive domain.

For peer review only

No.	Title	Author/year	MERSQI	COREQ	Reason for inclusion			Undergraduate (UG)/Postgraduate (PG)	Methodology	Purpose of study	Key findings
					Communication	Ethics	Professionalism				
									(Reflect Score (RS)) to measure reflections' depth, and the Thematic Score (TS) to map and grade reflections' themes.		
82	Contextual Analysis of Stakeholder Opinion on Management and Leadership Competencies for Undergraduate Medical Education: Informing Course Design	Rajeh, N. Grant, J. Farsi, J. Tekian, A.<br><br>2020		16	✓			UG	This was a cross-sectional study conducted at King Abdulaziz University (KAU), Saudi Arabia, during 2019. An exploratory qualitative approach, utilizing systematic content analysis, was used. Data were collected using semi-structured interviews that were con- ducted with 10 leaders who were stakeholders at KAU, health service providers at KAU hospital, and stakeholders in the Ministry of Health.	The study aimed to conduct a contextual analysis of interviews intended to assist with the future design of a feasible and relevant leadership and management course for undergraduate medical students at King Abdulaziz University (KAU), Saudi Arabia.	This study revealed critical findings that highlighted the areas in which KAU could instill better and adequate leadership and management skills in their undergraduate medical students. Multiple core categories for a leadership and management curriculum emerged with many interrelated themes. Most participants mentioned that leadership can be taught and that early exposure is beneficial for develop- ing skills. Additionally, they stated that leaders should have a vision and the ability to articulate that vision.<br><br>Different implementation challenges were described in relation to the availability of human resources, the current short supply of suitable teachers, and program design. Teaching methods recommended included simulations, lectures, and a project-based approach. Assessment methods that were recommended included objective structured clinical examination (OSCE), formative and summa- tive assessments, self-assessments, and portfolios.

For peer review only

Appendix C

Portfolio Content (Competencies Assessed and Assessment Modalities)

**Subthemes**	**Content**		
	**Communication**	**Ethics**	**Professionalism**
**Competencies Assessed**	• "Effective" communication (65, 67, 68, 81, 86, 107, 127, 131-133)<br>• Professional behaviour and moral reasoning (71)<br>• Systems awareness and team-based care (71)<br>• Continuous learning and quality improvement (71)<br>• Patient-centred medical care (71)<br>• Teamwork (70)<br><br>Oral communication skills<br>• Attentive, active listening (84, 139, 140)<br>• Explanation, information giving (127, 139)<br>• History taking (93)<br>• Consultation (141)<br>• Breaking bad news (24, 139)<br>• Shared decision making (142)<br>• Empathetic communication (142)<br>• Taking history and clinical examination (84, 93)<br>• Discerning appropriate language to ensure patient understands (24, 127)<br>• Conflict management (139)<br>• Adaptability to different settings (67)<br><br>Written communication skills<br>• Proper grammar, spelling, logical and coherent structure (103)<br>• Patient cases (93)<br>• Scientific research (93)<br><br>Communication with:<br>• Patients (24, 68, 84, 93, 127, 139, 143)<br>• Children (127)<br>• Family (68, 84, 139, 144)<br>• Colleagues, interprofessional team (24, 68, 84, 93, 130, 139)	• Ethics in interprofessional practice (24, 67, 86, 128, 130, 145, 146)<br>• Doctor-patient relationship (81, 141, 145)<br>• Values/ ethics (67, 70, 86, 146)<br>• Ethical and legal responsibilities (65, 86)<br>• Moral reasoning (89)<br>• Ethics is assessed as a component of professionalism (85, 87, 88)<br>• Empathy (84)<br>• Showing respect and commitment (84)<br>• Respect for privacy (84)<br>• How to express ethical views appropriately (82)<br>• Identifying unethical moments of practice and grey areas (85, 89, 90)<br><br>Reflection:<br>• Quality of student's reflections (82, 84, 87)<br>• Authenticity of reflection (87)	• Learning from clinical experience (24, 81)<br>• Working in groups/ teamwork (24, 67, 70, 81, 86, 128, 130, 134, 145)<br>• Doctor-patient relationship (81, 141, 145)<br>• Becoming a doctor/roles and responsibilities (81, 86, 128, 130, 134, 145, 146)<br>• Patient care (67, 70, 128, 130)<br>• Continuous learning (67, 70)<br>• Values/ ethics (67, 70, 86, 146)<br>• Interpersonal skills/ effective Communication, (24, 67, 128, 145, 146)<br><br>Reflection:<br>• Clinical experience (81)<br>• Teaching (81)<br>• Learning needs in becoming a doctor<br>• Strengths and developmental areas (65, 86)<br>• Understanding of concepts addressed (86)<br>• Dilemmas and doubts (133)

For peer review only

	• Public (68)		
**Assessment Modalities (Portfolio items)**	Formative only (67, 84, 87, 92, 119, 126, 130, 146)<br>Summative only (65, 70, 71, 81-83, 87, 104, 116, 131, 134)<br>Both formative and summative (24, 67, 83, 86, 87, 117, 122, 128, 130, 134)<br>MCQs (65, 68, 87, 95, 126, 141, 143, 144, 146)<br>OSCEs (65, 67, 70, 71, 87, 126, 127, 130, 141, 143, 144, 146)<br>Essays – topical (67, 81, 93, 126)<br>Mini clinical examination exercise (Mini-CEX) (65, 67, 70, 87, 93, 130, 139, 143, 146)<br>Medical/patient presentations/research (67, 68, 80, 86, 93, 121, 126, 129)<br>Workplace-based clinical (67, 70, 71, 80, 83, 93, 107, 112, 122, 126, 127, 130, 139, 143)<br>Case-based discussions, summaries (65, 68, 83, 85, 89, 92, 129)(19)(81, 129) (83, 126, 130)<br>Simulated patient assessment (65, 68, 84, 103, 112)<br>Team-based assessment (65, 83, 86, 93)<br>Logbooks/ Portfolio interview (65, 67, 68, 70, 81-85, 87, 88, 103, 121, 126) (67, 121, 126)<br>Peer evaluation (67, 70, 71, 80, 85, 93, 107, 126, 127)<br>Faculty evaluation (67, 68, 70, 71, 80, 81, 83-86, 117, 126, 131, 147)<br>Feedback reports (75, 77, 80, 86, 91, 93, 109, 112, 122, 126, 130, 134, 139, 143) – multisource feedback (75, 77, 81, 82, 85, 86, 91, 93, 103, 109, 127, 130)<br>Self-assessment/appraisal (67, 68, 80, 82, 93, 104, 126, 130, 134)<br>Self-reflections – written/video/diaries (65, 67, 68, 71, 80-83, 85, 87, 88, 90, 93, 103, 107, 121, 127-130)<br>Learning plans and contracts (67, 68, 71, 80-83, 86, 92, 93, 103, 133)<br>Letters of recommendation (65, 81, 103)<br>Curriculum vitae (65, 67, 70, 83, 86, 122, 126)		

For peer review only

Preferred Reporting Items for Systematic reviews and Meta-Analyses extension for Scoping Reviews (PRISMA-ScR) Checklist

SECTION	ITEM	PRISMA-ScR CHECKLIST ITEM	REPORTED ON PAGE #
**TITLE**			
Title	1	Identify the report as a scoping review.	1
**ABSTRACT**			
Structured summary	2	Provide a structured summary that includes (as applicable): background, objectives, eligibility criteria, sources of evidence, charting methods, results, and conclusions that relate to the review questions and objectives.	2
**INTRODUCTION**			
Rationale	3	Describe the rationale for the review in the context of what is already known. Explain why the review questions/objectives lend themselves to a scoping review approach.	4
Objectives	4	Provide an explicit statement of the questions and objectives being addressed with reference to their key elements (e.g., population or participants, concepts, and context) or other relevant key elements used to conceptualize the review questions and/or objectives.	4
**METHODS**			
Protocol and registration	5	Indicate whether a review protocol exists; state if and where it can be accessed (e.g., a Web address); and if available, provide registration information, including the registration number.	5
Eligibility criteria	6	Specify characteristics of the sources of evidence used as eligibility criteria (e.g., years considered, language, and publication status), and provide a rationale.	5
Information sources*	7	Describe all information sources in the search (e.g., databases with dates of coverage and contact with authors to identify additional sources), as well as the date the most recent search was executed.	5
Search	8	Present the full electronic search strategy for at least 1 database, including any limits used, such that it could be repeated.	5
Selection of sources of evidence†	9	State the process for selecting sources of evidence (i.e., screening and eligibility) included in the scoping review.	5
Data charting process‡	10	Describe the methods of charting data from the included sources of evidence (e.g., calibrated forms or forms that have been tested by the team before their use, and whether data charting was done independently or in duplicate) and any processes for obtaining and confirming data from investigators.	6
Data items	11	List and define all variables for which data were sought and any assumptions and simplifications made.	7
Critical appraisal of individual sources of evidence§	12	If done, provide a rationale for conducting a critical appraisal of included sources of evidence; describe the methods used and how this information was used in any data synthesis (if appropriate).	7

St. Michael's
Inspired Care. Inspiring Science.

For peer review only - http://bmjopen.bmj.com/site/about/guidelines.xhtml

SECTION	ITEM	PRISMA-ScR CHECKLIST ITEM	REPORTED ON PAGE #
Synthesis of results	13	Describe the methods of handling and summarizing the data that were charted.	8
**RESULTS**			
Selection of sources of evidence	14	Give numbers of sources of evidence screened, assessed for eligibility, and included in the review, with reasons for exclusions at each stage, ideally using a flow diagram.	8
Characteristics of sources of evidence	15	For each source of evidence, present characteristics for which data were charted and provide the citations.	8
Critical appraisal within sources of evidence	16	If done, present data on critical appraisal of included sources of evidence (see item 12).	Appendix B
Results of individual sources of evidence	17	For each included source of evidence, present the relevant data that were charted that relate to the review questions and objectives.	Appendix B
Synthesis of results	18	Summarize and/or present the charting results as they relate to the review questions and objectives.	8
**DISCUSSION**			
Summary of evidence	19	Summarize the main results (including an overview of concepts, themes, and types of evidence available), link to the review questions and objectives, and consider the relevance to key groups.	9-13
Limitations	20	Discuss the limitations of the scoping review process.	15
Conclusions	21	Provide a general interpretation of the results with respect to the review questions and objectives, as well as potential implications and/or next steps.	15
**FUNDING**			
Funding	22	Describe sources of funding for the included sources of evidence, as well as sources of funding for the scoping review. Describe the role of the funders of the scoping review.	None

JBI = Joanna Briggs Institute; PRISMA-ScR = Preferred Reporting Items for Systematic reviews and Meta-Analyses extension for Scoping Reviews.

* Where *sources of evidence* (see second footnote) are compiled from, such as bibliographic databases, social media platforms, and Web sites.

† A more inclusive/heterogeneous term used to account for the different types of evidence or data sources (e.g., quantitative and/or qualitative research, expert opinion, and policy documents) that may be eligible in a scoping review as opposed to only studies. This is not to be confused with *information sources* (see first footnote).

‡ The frameworks by Arksey and O'Malley (6) and Levac and colleagues (7) and the JBI guidance (4, 5) refer to the process of data extraction in a scoping review as data charting.

§ The process of systematically examining research evidence to assess its validity, results, and relevance before using it to inform a decision. This term is used for items 12 and 19 instead of "risk of bias" (which is more applicable to systematic reviews of interventions) to include and acknowledge the various sources of evidence that may be used in a scoping review (e.g., quantitative and/or qualitative research, expert opinion, and policy document).

From: Tricco AC, Lillie E, Zarin W, O'Brien KK, Colquhoun H, Levac D, et al. PRISMA Extension for Scoping Reviews (PRISMAScR): Checklist and Explanation. Ann Intern Med. 2018;169:467–473. doi: 10.7326/M18-0850.

BMJ Open

An evidence-guided approach to Portfolio guided Teaching and Assessing Communications, Ethics and Professionalism for medical students and physicians:A Systematic Scoping Review

Journal:	*BMJ Open*
Manuscript ID	bmjopen-2022-067048.R4
Article Type:	Original research
Date Submitted by the Author:	14-Mar-2023

Complete List of Authors:	Ting, Jacquelin Jia Qi; National University of Singapore, Yong Loo Lin School of Medicine; National Cancer Centre Singapore, Division of Supportive and Palliative Care<br>Phua, Gillian Li Gek; National Cancer Centre Singapore, Division of Supportive and Palliative Care; Duke-NUS Medical School, Lien Centre for Palliative Care<br>Hong, Daniel Zhihao; National University of Singapore, Yong Loo Lin School of Medicine; National Cancer Centre Singapore, Division of Supportive and Palliative Care<br>Lam, Bertrand Kai Yang; National Cancer Centre Singapore, Division of Supportive and Palliative Care<br>Lim, Annabelle Jia Sing ; National University of Singapore, Yong Loo Lin School of Medicine; National Cancer Centre Singapore, Department of Supportive and Palliative Care<br>Chong, Eleanor Jia Xin; National University of Singapore, Yong Loo Lin School of Medicine; National Cancer Centre Singapore, Division of Supportive and Palliative Care<br>Pisupati, Anushka; National University of Singapore, Yong Loo Lin School of Medicine; National Cancer Centre Singapore, Division of Supportive and Palliative Care<br>Tan, Rei; National University of Singapore, Yong Loo Lin School of Medicine; National Cancer Centre Singapore, Division of Supportive and Palliative Care<br>Yeo, Jocelyn Yi Huang; National University of Singapore, Yong Loo Lin School of Medicine; National Cancer Centre Singapore, Division of Supportive and Palliative Care<br>Koh, Yi Zhe; National University of Singapore, Yong Loo Lin School of Medicine; National Cancer Centre Singapore, Division of Supportive and Palliative Care<br>Quek, Chrystie; National University of Singapore, Yong Loo Lin School of Medicine; National Cancer Centre Singapore, Division of Supportive and Palliative Care<br>Lim, Jia Yin; National University of Singapore, Yong Loo Lin School of Medicine; National Cancer Centre Singapore, Division of Supportive and Palliative Care<br>Tay, Kuang Teck; National Cancer Centre Singapore, Division of Supportive and Palliative Care<br>Ong, Yun Ting; National University of Singapore, Yong Loo Lin School of

	Medicine; National Cancer Centre Singapore Division of Supportive and Palliative Care<br>Chiam, Min; National Cancer Centre Singapore, Division of Cancer Education<br>Zhou, Xuelian Jamie; National Cancer Centre Singapore, Division of Supportive and Palliative Care; Duke-NUS Medical School, Lien Centre for Palliative Care<br>Mason, Stephen; University of Liverpool, Palliative Care Institute Liverpool<br>Wijaya, Limin; National Cancer Centre Singapore, Division of Supportive and Palliative Care; Duke-NUS Medical School<br>Krishna, Lalit; University of Liverpool,

**Primary Subject Heading**:	Communication
Secondary Subject Heading:	Communication, Ethics, Medical education and training
Keywords:	MEDICAL EDUCATION & TRAINING, ETHICS (see Medical Ethics), EDUCATION & TRAINING (see Medical Education & Training)

SCHOLARONE™
Manuscripts

I, the Submitting Author has the right to grant and does grant on behalf of all authors of the Work (as defined in the below author licence), an exclusive licence and/or a non-exclusive licence for contributions from authors who are: i) UK Crown employees; ii) where BMJ has agreed a CC-BY licence shall apply, and/or iii) in accordance with the terms applicable for US Federal Government officers or employees acting as part of their official duties; on a worldwide, perpetual, irrevocable, royalty-free basis to BMJ Publishing Group Ltd ("BMJ") its licensees and where the relevant Journal is co-owned by BMJ to the co-owners of the Journal, to publish the Work in this journal and any other BMJ products and to exploit all rights, as set out in our licence.

The Submitting Author accepts and understands that any supply made under these terms is made by BMJ to the Submitting Author unless you are acting as an employee on behalf of your employer or a postgraduate student of an affiliated institution which is paying any applicable article publishing charge ("APC") for Open Access articles. Where the Submitting Author wishes to make the Work available on an Open Access basis (and intends to pay the relevant APC), the terms of reuse of such Open Access shall be governed by a Creative Commons licence – details of these licences and which Creative Commons licence will apply to this Work are set out in our licence referred to above.

Other than as permitted in any relevant BMJ Author's Self Archiving Policies, I confirm this Work has not been accepted for publication elsewhere, is not being considered for publication elsewhere and does not duplicate material already published. I confirm all authors consent to publication of this Work and authorise the granting of this licence.

For peer review only

Title: An evidence-guided approach to Portfolio guided Teaching and Assessing Communications, Ethics and Professionalism for medical students and physicians: A Systematic Scoping Review

Short Title: A review of CEP Portfolios for physicians and medical students

Jacquelin Jia Qi Ting[1,2], Gillian Phua[2,3,4], Daniel Zhihao Hong [1,2], Bertrand Kai Yang Lam[2], Annabelle Jia Sing Lim[1,2], Eleanor Jia Xin Chong[1,2], Anushka Pisupati[1,2] , Rei Tan[1,2], Jocelyn Yi Huang Yeo[1,2], Yi Zhe Koh[1,2], Chrystie Wan Ning Quek[1,2], Jia Yin Lim[1,2], Kuang Teck Tay[2], Yun Ting Ong[1,2], Min Chiam[5,] , Jamie Zhou [2,3,4], Stephen Mason[8], Limin Wijaya[2,3,9], Lalit Kumar Radha Krishna[1-8,#]

[1] Yong Loo Lin School of Medicine, National University of Singapore, NUHS Tower Block, 1E Kent Ridge Road, Level 11, Singapore 119228

[2] Division of Supportive and Palliative Care, National Cancer Centre Singapore,11 Hospital Dr, Singapore 169610

[3] Duke-NUS Medical School, 8 College Rd, Singapore 169857

[4] Lien Centre for Palliative Care, Duke-NUS Medical School, 8 College Rd, Singapore 169857

[5] Division of Cancer Education, National Cancer Centre Singapore, 11 Hospital Dr, Singapore 169610

[6] PalC, The Palliative Care Centre for Excellence in Research and Education, PalC c/o Dover Park Hospice, 10 Jalan Tan Tock Seng, Singapore

[7] Centre of Biomedical Ethics, National University of Singapore, 21 Lower Kent Ridge Rd, Singapore 119077

[8] Palliative Care Institute Liverpool, Academic Palliative & End of Life Care Centre, Cancer Research Centre, University of Liverpool, 200 London Rd, Liverpool L3 9TA, United Kingdom

[9] Department of Infectious Diseases, Singapore General Hospital, Outram Road, Singapore 169608

[#]Corresponding Author:

Dr. Lalit Kumar Radha Krishna

Palliative Care Institute Liverpool, Academic Palliative & End of Life Care Centre,

University of Liverpool, 200 London Rd, Liverpool L3 9TA, United Kingdom

Email: lalit.radha-krishna@liverpool.ac.uk Phone number: 6436 8183

For peer review only

For peer review only - http://bmjopen.bmj.com/site/about/guidelines.xhtml

ABSTRACT

Objectives

Guiding the development of longitudinal competencies in communication, ethics, and professionalism underlines the role of portfolios to capture and evaluate the multiple multi-source appraisals, and direct personalised support to clinicians. However, a common approach to these combined portfolios continues to elude medical practice. A systematic scoping review is proposed to map portfolio use in training and assessments of ethics, communication, and professionalism competencies particularly as its inculcation of new values, beliefs and principles changes attitudes, thinking, practice, while nurturing Professional Identity Formation. It is posited that effective structuring of portfolios promote self-directed learning, personalised assessment and appropriate support of professional identity formation.

Design

Krishna's Systematic Evidence-Based Approach (SEBA) is employed to guide this systematic scoping review of portfolio use in communication, ethics and professionalism training and assessment.

Data sources

PubMed, Embase, PsycINFO, ERIC, Scopus, and Google Scholar databases.

Eligibility Criteria

Articles published between 1st January 2000 and 30th June 2020 were included.

Data extraction and synthesis

The included articles are concurrently content and thematically analysed using the Split Approach. Overlapping categories and themes identified are combined using the Jigsaw Perspective. The themes/categories are compared with the summaries of the included articles in the Funnelling Process to ensure their accuracy. The domains identified form the framework for the discussion

Results

12,300 abstracts were reviewed, 946 full-text articles were evaluated, and 82 articles were analysed and the four domains identified were indications; content; design; and strengths and limitations.

For peer review only

For peer review only - http://bmjopen.bmj.com/site/about/guidelines.xhtml

Conclusions

This review reveals that when using a consistent framework, accepted endpoints and outcome measures, longitudinal multisource, multimodal assessment data fashions professional and personal development and enhances identity construction. Future studies into effective assessment tools and support mechanisms are required if portfolio use is to be maximised.

Strengths and limitations of this study

- The SEBA methodology adopts the structure of systematic reviews and flexibility of narrative reviews to synthesise a reproducible and accountable evaluation of diverse methodological sources, settings, physician populations and specialities.
- This review evaluates the impact of longitudinal development of communications, ethics and professionalism competencies and their impact on professional identity formation.
- Given that communications, ethics and professionalism are sociocultural constructs, geopolitical sociocultural differences may raise questions as to the applicability of these findings beyond the European and North American medical education systems.
- As the number of articles included is limited, the review's ability to assess the long-term effectiveness of portfolios may be compromised.

For peer review only

Abstract Word Count: 284 words

Manuscript Word Count: 2346 words

Number of Tables: 5

Number of Figures: 2

Number of References: 150

Keywords: Portfolios, Communication, Ethics, Professionalism, Professional Identity Formation, Medical Education, medical school, undergraduate, postgraduate training.

For peer review only - http://bmjopen.bmj.com/site/about/guidelines.xhtml

INTRODUCTION

Evidence for the effective embodiment of ethical and professional principles, communication skills and appropriate use of empathy in clinical practice requires a longitudinal, and often multi-source perspective. With ethics and professionalism sharing longitudinal developmental trajectories and intimately entwined with communication skills and competencies, the three competencies are increasingly considered together. In practice longitudinal CEP programs appear in the curricula of the Top 10 medical schools featured on the QS World University Rankings 2020 (1). Concurrent study of communication, ethics, and professionalism (henceforth CEP) is also underlined by their common sociocultural roots. As sociocultural constructs (2), CEP competencies are shaped by the individual's personal experiences, motivations, enthusiasm, idealism, abilities, competencies, virtues, expectations, knowledge, skills, emotions, and attitudes (henceforth narratives); their values, beliefs, and principles (henceforth belief systems)(3); their clinical experiences, competencies, training, insights, and confidence (henceforth clinical insights)(4, 5, 6, 7) and their practice, clinical, social , cultural, academic, research, and personal considerations (henceforth contextual considerations)(8, 9, 10, 11, 12).

However, whilst previous reviews into the teaching of ethics (13, 14, 15), communication (16, 17, 18, 19, 20, 21, 22) and professionalism (23, 24, 25) suggest the use of portfolios could provide a personalised, holistic, and longitudinal perspective of CEP skills, knowledge, and attitudes, and support of developing competencies we are aware of little progress in designing such platforms. Impetus for mapping current use of CEP portfolios also arises from the notion that developing CEP competencies shape how medical students and physicians (henceforth clinician) "think, act and feel like a physician" (26) or their professional identity formation (henceforth PIF) (27). It is posited that the promise of CEP portfolios with better appreciation of evolving self-concepts of professional identity will better direct support and even remediation of professional, ethical, communication and interprofessional development and PIF. This is especially pertinent at a time of increasing reports of breaches in standards, codes of conduct, and social and practice expectations (25).

METHODS

A Systematic Evidence-Based Approach (SEBA) guided Systematic Scoping Review (SSR) (henceforth SSR in SEBA) is proposed to map CEP portfolio use in medicine to guide the design, structuring and support of a proposed program (13, 14, 15, 16, 17, 20, 21, 22, 24, 25, 27, 28, 29, 30, 31, 32, 33, 34, 35, 36, 37, 38, 39, 40, 41, 42, 43, 44, 45, 46, 47, 48, 49, 50). Given space constraints, we briefly describe the six stages in the construction

For peer review only - http://bmjopen.bmj.com/site/about/guidelines.xhtml

of SSRs in SEBA here (Figure 1) and advance more details of the Systematic Approach, Split Approach, Jigsaw Perspective, Funnelling Process, Reiterative Process and Synthesis of SSR, in Appendix A (16, 27, 28, 35, 41, 48, 51, 52, 53, 54, 55, 56).

(Insert Figure 1. *The SEBA Process*)

Stage 1 of SEBA: Systematic Approach

A. Determining of Title and Background of the Review

An expert team comprised of a medical librarian from the Yong Loo Lin School of Medicine (YLLSoM) at the National University of Singapore (57), and local education experts and clinicians at the National Cancer Centre Singapore (NCCS), the Palliative Care Institute Liverpool, YLLSoM and Duke-NUS Medical School (henceforth the expert team) supported the research team in designing and overseeing the SEBA process.

B. Identification of Research Question

The research and expert team determined the primary research question: "*What is known about CEP portfolios?"*. The secondary research questions were "*what role do CEP portfolios have in teaching and assessing CEP development?"*.

C. Inclusion Criteria

A Population, Intervention, Comparison and Outcome (PICOs) format, outlined in Table 1, was used to guide the research process (58, 59).

Table 1. PICOS Inclusion and Exclusion Criteria

PICOS	Inclusion criteria	Exclusion criteria
**Population**	• Undergraduate and postgraduate medical students<br>• Qualified medical doctors, physician or resident; medical officer, registrar, house officer, attending, consultant	• Allied health specialties such as Pharmacy, Dietetics, Chiropractic, Midwifery, Podiatry, Speech Therapy, Occupational and Physiotherapy<br>• Non-medical specialties such as Clinical and Translational Science, Alternative and Traditional Medicine, Veterinary, Dentistry
**Intervention**	• portfolios in undergraduate and postgraduate medical education for teaching and assessment of Communication, Ethics and Professionalism	Other documentation methods or learning tools that are:<br>• Not longitudinal or single timepoint

For peer review only - http://bmjopen.bmj.com/site/about/guidelines.xhtml

PICOS	Inclusion criteria	Exclusion criteria
	Criteria of a portfolio:<br>• longitudinal (more than a single timepoint) assessment data<br>• candidate's personal engagement with portfolio content and associated learning<br>• Interventions meeting the above criteria were included regardless of whether they were referred to as portfolios<br><br>All types of portfolios were included in the study:<br>• For instance: electronic & non-electronic; formative & summative or combined; clinical & non-clinical<br>• Portfolios with input from students and/or residents and/or doctors, and/or input from faculty members and other individuals<br>• Portfolios with different structures: extent by which the structure has been prescribed and/or left to individual discretion	• Does not include personal intellectual engagement with the content and associated learning (for instance, curriculum vitae, logbooks and the use of personal digital assistants)
**Comparison/ Context**	NA	NA
**Outcome**	Papers that measured the following outcomes were included:<br>• Effectiveness of the use of portfolios to assess and teach Communication, Ethics and Professionalism<br>• Impact of the use of portfolios on medical students (both undergraduate and postgraduate)<br>• Impact of the use of portfolios on the faculty	NA
**Study design**	• Articles in English or translated to English<br>• Articles published from 1st January 2000 to 31st December 2020<br>• Databases: PsycINFO, EMBASE, PubMed, ERIC, Scopus, Google Scholar<br>• All study designs including:<br>• Mixed methods research, meta-analyses, systematic reviews, randomised controlled trials, cohort studies, case-control studies, cross-sectional studies, descriptive papers, grey literature, opinions, letters, commentaries and editorials	NA

D. Identification of Relevant Studies

The research team developed search strategies and carried out independent reviews for relevant publications in the PubMed, Embase, PsycINFO, ERIC, Scopus, and Google Scholar databases (search strategy enclosed in Appendix B). Keeping with Pham, Rajić (60)'s approach of ensuring a feasible and sustainable research process,

For peer review only - http://bmjopen.bmj.com/site/about/guidelines.xhtml

the team contained the search to articles published between 1st January 2000 and 31st December 2020 to accommodate existing human resource and time constraints and ensure a sustainable review process.

E. Selection of Studies Included in Review

Six members of the research team created individual lists of titles to be included whilst three other members of the research team carried out hand searches and ancestry searches of seven leading journals in medical education (Academic Medicine, Medical Education, Medical Teacher, Advances Health Sciences Education, BMC Medical Education, Teaching and Learning in Medicine and Perspectives on Medical Education) accessed through the NUS library portal. These individual lists of titles were discussed online and Sandelowski and Barroso (61)'s "negotiated consensual validation" approach to "*articulate, defend, and persuade others of the "cogency" or "incisiveness" of their points of view or show their willingness to abandon views that are no longer tenable*" was applied to achieve consensus on the final list of titles to be scrutinised.

F. Assessing the Quality of Articles

Eight research team members individually appraised the quality of the quantitative and qualitative studies using the Medical Education Research Study Quality Instrument (MERSQI) (62) and Consolidated Criteria for Reporting Qualitative Studies (COREQ) (63).

Stage 2 of SEBA: Split Approach

Two teams carried independent and concurrent thematic and content analysis of the included articles whilst a third team created tabulated summaries based on recommendations drawn from Wong, Greenhalgh (64)'s RAMESES publication standards: meta-narrative reviews and Popay, Roberts (65)'s *"Guidance on the conduct of narrative synthesis in systematic reviews".* The categories employed in the content analysis for undergraduate communications were Rider et al. (66) 's *"A model for communication skills assessment across the undergraduate curriculum",* Goldie (67)'s *"Review of ethics curricula in undergraduate medical education"*, Duffy et al. (68)'s *"Assessing Competence in Communication and Interpersonal Skills: The Kalamazoo II Report"* and Hong et al. (13)'s *"Postgraduate Ethics Training Programs: A Systematic Scoping Review"*. Tay et al. (24)'s *"Assessing Professionalism in Medicine - A Scoping Review of Assessment Tools from 1990 to 2018"* was employed for codes

For peer review only - http://bmjopen.bmj.com/site/about/guidelines.xhtml

for professionalism and David (69)'s article *"AMEE Medical Education Guide No. 24: Portfolios as a method of student assessment"* was then used to contextualise their use in portfolios.

Stage 3 of SEBA: The Jigsaw Perspective

The Jigsaw Perspective sees the themes and categories identified compared and combined where overlaps and similarities exist.

Stage 4 of SEBA: The Funnelling Process

The Funnelling Process sees the themes/categories created from the Jigsaw Approach compared with the tabulated summaries to determine their consistency.

Patient and Public Involvement

Patients or the public were not involved in the design, or conduct, or reporting, or dissemination plans of our research.

RESULTS

A total of 12,300 abstracts were reviewed, 946 full-text articles were evaluated, and 82 articles were analysed (Figure 2). The funnelled domains identified are 1) indications, 2) portfolio content, 3) portfolio design and implementation and 4) strengths and limitations. In keeping with its goals of transparency and accountability, these Tables are enclosed in Appendix C.

(Insert Figure 2. PRISMA Flowchart)

Funneled Domain 1: Indications

CEP portfolios curate longitudinal multisource, multimodal assessment data taken at different settings and contexts allowing it to serve two main roles- teaching and assessment (70, 71) (Table 2). However few span undergraduate and postgraduate education (69, 72, 73), . As assessment tool CEP portfolios assist in the identification of areas of weakness and guide the provision of a comprehensive, targeted feedback, support and remediation (72, 74, 75). Most CEP assessment portfolios in postgraduate education focus on evidencing the attainment of required competencies (76, 77, 78, 79, 80), capturing reflections and indications of professional

For peer review only - http://bmjopen.bmj.com/site/about/guidelines.xhtml

identity formation (78, 81, 82, 83). CEP portfolios in the postgraduate setting have also been used for revalidation purposes (81)

As a teaching tool, CEP portfolios encourage self-directed learning (75, 84, 85, 86), self-improvements (71), reflective practice (72, 73, 75, 79, 81, 85, 86, 87, 88, 89, 90, 91, 92, 93, 94, 95), motivate practitioners to achieve desired competencies (90, 96) and bolster overall professional and personal development (72, 88, 96, 97).

Table 2. Benefits of CEP Teaching and Assessment Portfolios

<table>
<tr><th>CEP teaching portfolios</th><th>CEP assessment portfolios</th></tr>
<tr><td>Enhancing learning
Promotion of life-long learning (98, 99)

Promotion of self-directed learning
o Formulation of learning plans (90, 96, 100)
o Facilitates Outcome-oriented learning (89, 90, 101, 102)
o Enhances self-evaluative capabilities (73, 76, 80, 85, 88, 89, 98, 99, 100, 103, 104, 105)
o Identification of strengths and weaknesses (69, 71, 76, 78, 92, 96, 104, 106, 107, 108, 109)
o Inculcates responsibility and ownership for one’s learning (71, 73, 74, 80, 84, 86, 101, 110)
o Sets the pace (99)
o Personalised learning (108, 109)</td><td rowspan="2">Balance of structure and freeform learning (69, 76, 92, 107, 111, 112)

Adaptable to goals (77, 113, 114)

Alignment of learning outcomes and assessment methods (90, 91, 97, 101, 105)

Allows personalised assessments (77)

Allows assessment over time (84, 92) and stages of development (69) and that facilitate evaluation of:
o Learning and progress over time (77, 106)
o Learning curve (121)
o In different roles (92, 93)
o In different settings (70, 113)
o Complex behaviours (72, 74, 77, 84, 90, 99, 115, 116)
o Critical thinking (77, 97, 105, 114)
o Outcomes/competencies (107, 115)
o Self-assessment (77, 105, 114)
o Complex skills (113)
o Professionalism (114)
o Cultural competence (114)
o Communication skills (114)
o Clinical ethics (117)
o Problem solving abilities (77, 114)
o Reflective abilities (77, 90, 105, 109)

Longitudinal data
o Possess good predictive value of subsequent clinical competency (74, 118)
o Enhances validity of portfolio grading (69)
o Guides better decisions (74, 116, 119)
o Provides authentic assessment is a key principle of it (92, 99, 109)
o Provides insights in the various core competencies (98)
o Fair mode of assessment(99) and allows student to defend positions/work (69)
o Provides students with direction and clarity (111, 112)</td></tr>
<tr><td>Personal and professional development
Promotes self-understanding (79, 120, 121)
o Understanding feelings and thoughts (100, 108)
o Understanding of one’s values (92)
o Understanding activity/experience (69, 88, 122)
o Understanding of how one functions in a team (116)
o Fears and stressors feelings of inadequacy (122, 123)

Curates evidence of skills (98)
o Skills to compile a portfolio (98, 111, 124, 125, 126)
o Reflective skills (69, 76, 88, 102, 108, 122, 127)
o Clinical skills (99)
Organizes information(102, 107)
o Documentation of progress (77, 98, 104, 106)
o Documentation of skills in different settings(71, 106, 109)
Cross-sectional view of one’s competence (69, 77, 106, 109)</td></tr>
</table>

For peer review only - http://bmjopen.bmj.com/site/about/guidelines.xhtml

**Personalisation**<br>Allows for personalisation (69, 90, 99, 110, 114)<br>○ Personal experiences (69)<br>Choice over the contents (69, 99, 114)	Allows the combination of Summative and Formative assessment (69, 91) and multisource assessments (81, 87, 97, 108) (109) and triangulation (77, 109)<br><br>Facilitates personalised feedback (116) that can be informed by understanding of the individual setting and context (99, 100, 113)
**Benefits to faculty**<br>Improvement of curriculum (83, 92)<br>○ Identify areas of weakness in curriculum (83)<br>○ Provide insights into mentoring, teaching etc (128)<br>Facilitates design of the program (83)<br><br>faculty development<br>○ Understand more about how students acquire competencies (99)<br>○ Insight into the impact of their interventions (119, 128)<br>Insight into their professional conduct, role modelling and pastoral care (121)	
**E-portfolios**<br>Ease of use:<br>○ Easier use (90)<br>○ Accessible (73)<br>Dissemination and editing of information (110)	

Funneled Domain 2: Characteristics

The contents of portfolios were shaped by their overall function (77, 80, 83, 113, 129) (Table 3). Expectations about the nature, and assessments of the contents (83) facilitates personalization of portfolios (73, 77, 104).

Table 3. Portfolio Design and Implementation

Themes	Subthemes	Contents
**Design and Implementation Principles**	**Structure**	Clear guidelines (69, 87, 88, 96, 97, 105, 108)<br>• Use of portfolio (69, 88)<br>• Portfolio content - determining competencies to assess (69, 91)<br>• Promotional decisions (69, 71, 89, 121, 130)<br>Standard-setting exercises (75)<br>Provision of sample portfolios for reference (105, 131, 132)<br>• Roles for assessors (69, 71, 74, 120, 121, 130, 133)<br>• Expectations of students (112)<br>• Duration spanning across preclinical and clinical years (126)<br>Well-defined structure with room for flexibility (69, 86, 88, 90, 105)<br>Student-centered (90)<br>Appropriate student to mentor ratio (71, 125)<br>Sufficient curricular experience for reflection, feedback (71)<br>Protected time for reflection, feedback, and regular meetings with mentors (71, 74, 87, 108)
	**Faculty**	Good faculty support (121, 126)<br>Ensure culture is supportive of portfolio system (71, 126)<br>Flexible and enthusiastic tutors passionate and experienced mentors (85, 88)

For peer review only - http://bmjopen.bmj.com/site/about/guidelines.xhtml

	**Curricular Integration**	Early introduction of portfolio (88, 108, 110, 134)<br>Easing portfolio into curriculum (69, 72, 86, 88, 90) – including orientation, training and introductory sessions (69, 72, 88, 90, 105, 121, 132) for learners and staff training (69, 71, 72, 75, 84, 85, 88)<br>Implementation of portfolios throughout curriculum (121)<br>Portfolio content aligning with phase of medical journey and competencies required (69, 71, 90, 97, 130, 132)
	**Assessments**	Clear scoring rubrics (69, 126)<br>Alignment between assessments and outcomes (86, 87, 90)<br>Transparent assessment criteria and outcomes to maintain fairness (71, 72, 110, 131)<br>Standardisation of portfolio use and content (86, 87), assessors training (72) and increased number of assessors (86, 87, 134) to improve assessment reliability (86, 87, 134)<br>Separate processes and reviewers for summative and formative portfolio (71) – summative assessments to specify clear pass/fail requirements (69)<br>Incorporation of student feedback into portfolio development (69, 72, 90)
	**Assessors**	External examiners (69)<br>Faculty members (69, 71, 74, 120, 130, 133)<br>Mentors (107)<br>Senior staff to be paired with new examiners (69)<br>Mentors should not assess own student (86, 92)
	**Assessment Frequency**	Formative assessments to monitor and guide student learning – conducted throughout the year (86, 88, 91)<br>Summative assessments to evaluate learner performance – conducted annually (69, 71, 72, 74, 75, 84, 85, 90, 91, 108, 130, 132, 135, 136, 137, 138), twice (71, 75, 116), thrice (88) or multiple times a year (77, 79)
	**Oversight and Delegation**	Establishment of portfolio oversight committee (71, 75)<br>Regular programme evaluation (71)<br>Clear division of support roles – e.g. pedagogical and technical support (110)

Postgraduate CEP assessment portfolios inject more details upon undergraduate portfolios. Table 4 summarises these elements include communication skills, addressing ethical issues and compliance of professional, institutional and departmental policies (106, 124, 139). Greater detail in postgraduate CEP assessment portfolios sees the addition of modified essay questions (MEQ)s (79), polypharmacy journal/audits (70, 95, 99, 129, 140), professional workshops and training sessions (98, 99, 113, 141, 142), health service meeting reports (79) and reviews by medical regulatory authorities (95). A detailed summary is provided in Appendix D.

Table 4. Portfolio Content (Competencies Assessed and Assessment Modalities)

	Communication	Ethics	Professionalism
Common competencies assessed	1. Values/ethics – appreciation of professional, legal and societal values to guide practice<br>2. Professional responsibilities- appreciation and manifesting these responsibilities		

For peer review only

	3. Doctor- patient relationships- appreciation of the guiding principles, expectations and ability to nurture these relationships<br>4. Interprofessional team working<br>5. Ethical and legal responsibilities<br>6. Continuous learning and quality improvement		
Specific competencies	1. Active listening<br>2. Clear explanations<br>3. Empathetic communication<br>4. History taking<br>5. Breaking bad news<br>6. Conflict management<br>7. Shared decision making<br>8. Written communication	1. Professionalism<br>2. Respect<br>3. Privacy<br>4. Identifying key ethical issues<br>5. Balancing competing ethical responsibilities<br>6. Consent	1. Interpersonal communication
Reflection	1. Content<br>2. Context<br>3. Depth<br>4. Authenticity<br>5. Developmental areas<br>6. Dilemma and doubts		
Assessment (formative/ summative/ mixed)	MCQ<br>Essays<br>OSCE<br>Mini-CEX<br>Case presentations<br>Case based discussion<br>Simulated patient assessment<br>Work based assessment<br>Team based assessment<br>Peer evaluation<br>Self-evaluation<br>Faculty evaluations<br>Feedback<br>Reflections<br>Letters of commendations<br>Learning and or improvement plans<br>Curriculum Vitae<br>Logbook review/interview		

For peer review only

Funneled Domain 3: Strengths and Limitations

The strengths and limitations of portfolios are outlined in Table 5.

Table 5. Strengths and Limitations

Themes	Subthemes	Content
**Strengths/ Limitations**	**Strengths**	For students<br>Identification of own strengths and weaknesses (69, 74, 75, 85, 86, 87, 88, 90, 92, 96, 100, 138)<br>Appreciation of effective communication (88, 134, 143), ethical issues (92), and fosters critical professional attitudes (87, 144)<br>Encourages self-regulation (138) through opportunities for reflection (86) and self-assessment (88, 108, 121)<br>Facilitates preparation for post-graduate medical training (88, 107, 111)<br><br>For faculty<br>Identification of areas of concern and allows for early, timely intervention (74, 75)

For peer review only - http://bmjopen.bmj.com/site/about/guidelines.xhtml

		Enhances interaction between students and teachers (69, 88, 92) Facilitates reiterative reassessment of teaching strategies (69)<br><br>Assessments<br>Longitudinal (116)<br>Good validity (74)<br>Reliable assessment tool – allows for triangulation of information and evidence from multiple sources and contexts (71)<br>Ability to assess a wide range of competencies (90, 97)<br>Allows for more authentic assessments (74)<br>Gives faculty greater insight into students' achievement of behaviourally-oriented competencies that traditional methods do not (75)<br>Evaluates achievement during clinical attachment/clerkship (97)<br>Provides a basis for the judgement of a student's professional fitness to practice (72)
	**Limitations**	Time-consuming and labour intensive (72, 75, 85, 88, 89, 90, 91, 96, 107, 126, 134)<br>Issues with reliability (86, 90, 120, 134)<br>Inconsistent effects (86, 90, 108, 145)<br>Lack of honesty by students in reflections (71, 72, 86, 90, 91, 107, 108, 112)

Postgraduate portfolios provide greater insights into lifelong learning and continuous professional development (77, 80, 81, 83, 98, 99, 106, 109, 113, 141). Use of multi-source assessments at multiple timepoints (80) in clinical settings (81, 99) see postgraduate portfolios provide good inter-rater reliability (77, 109). Postgraduate portfolios also detect stress and burnout (141).

Stage 5 of SEBA: Analysis of data and non-data driven literature

Findings from the different stages of SEBA were discussed with members of the expert team and relevant stakeholders. There were concerns from the expert team about the impact of grey literature on the narrative, given that they were neither peer-reviewed nor necessarily evidence-based. As a result, the research team differentiated correspondence, letters, editorials and perspective pieces from academic databases and grey literature from data-driven or research-based peer-reviewed data. Both groups were then independently analysed. The themes/categories identified were then compared to enhance further the accountability and the reproducibility of Stage 5 of SEBA. Evidence-based data from bibliographic databases (henceforth evidence-based publications) were separated from grey literature, perspectives, editorials, letters, and non-data-based articles drawn from bibliographic databases (henceforth non-data driven). These two groups were separately thematically analysed.

Stage 6 of SEBA: Synthesis of the Discussion

The Best Evidence Medical Education (BEME) Collaboration Guide (146) and the Structured approach to the Reporting In healthcare education of Evidence Synthesis (STORIES) (147) were used to guide the discussion.

For peer review only - http://bmjopen.bmj.com/site/about/guidelines.xhtml

DISCUSSION

This SSR in SEBA suggest that overlaps in ethics, professionalism, and communication skills, knowledge, attitudes and competencies reaffirm the concurrent training, and assessments of CEP competencies in portfolios (72, 74, 75). To be effective CEP teaching and longitudinal assessment portfolios require a consistent framework replete with clearly delineated goals (105), aligned expectations, predetermined assessment criteria for specified competencies (69, 71, 72, 77, 83, 85, 87, 96, 97, 98, 105, 110, 120, 121, 124, 132, 133, 139), agreed upon endpoints and outcome measures, and the curation, structuring and organisation of longitudinal multisource, multimodal assessment data (70, 71). The combination of this data facilitate training, assessment, feedback and holistic and longitudinal support (75, 84, 85, 86) (101) (106, 124, 139, 148), guide reflection (72, 73, 75, 79, 81, 85, 86, 87, 88, 89, 90, 91, 92, 93, 94, 95), and bolster professional and personal development (72, 88, 96, 97). Critically the employ of consistent and agreed upon structure, goals, purpose, role, learning objectives, content, and assessment guidelines also affords users with a personalised perspective of their CEP development upon which they can reflect on and glean insights and feedback (86, 102, 131).

Yet to be effective, CEP portfolios require policing and underscores the need for trained (70) and dedicated faculty (88) capable of effective role modelling (121), support (69, 79, 88, 92, 97) and providing feedback (116) and who appreciate the individual's current concepts, narratives, motivations, abilities and availabilities and contextual considerations. Furthermore, the efficacy of portfolios and their ability to meet the stated objectives stated in Table 2 and realise their stated benefits in Table 5 are reliant upon the appropriate mix of assessment methods employed, the frequency of assessments, the efficacy of analysis and the quality of the feedback, support and remediation provided should it be required. This in turn highlights the role of the host organization in ensuring effective oversight of the program and assessments and in ensuring that faculty are afforded protected time for administrative duties, coordination and assessments particularly given the longitudinal nature of CEP portfolios (77, 90, 105, 106, 122, 140, 149, 150). It should not be forgotten that CEP portfolios reflect the development of an individual and such change should be measured and contextualised within the wider education, training, practice, professional, research, clinical, interprofessional team based and organizational perspective. This further underlines the importance of the host organizations role in assessing and guiding development of CEP competencies.

For peer review only

With these requisites met, evidence of changes in CEP knowledge, skills and competencies suggests shift the thinking, attitudes and conduct of clinicians. The Krishna-Pisupati model posits that sustained changes in practice, beliefs, values and principles will result in changes in the clinician's belief systems which in turn inform their self-concepts of identity and shape their PIF.

LIMITATIONS

The generalisability of the results of this study is limited by the identified studies selected for review. Firstly, the majority of the papers included originate from Europe and North America. Given that CEP is a sociocultural construct, these geopolitical sociocultural differences raise questions as to the applicability of these findings beyond the European and North American medical education systems. Secondly, as the number of articles included is limited, the SSR in SEBA's ability to assess the long-term effectiveness of portfolios may be compromised.

CONCLUSIONS

The suggestion that CEP portfolios can capture, instruct, and assess PIF requires further study. Whilst there have been posits on how such evaluations could be made, such tools remain unrealised and must be the focus of coming studies. One possible starting point for the design of such tools may be theories such as the Krishna-Pisupati model which attempts to link PIF to changes to belief systems and concepts of personhood. This may provide a grander view of PIF that spans the undergraduate and postgraduate settings and provide the basis for directing support and feedback for clinicians. As we look forward to furthering efforts to study the effects of CEP portfolios upon the PIF of clinicians, we also believe the specific impact of these potentially resource-heavy and financially costly intervention should be at the centre of future initiatives.

ABBREVIATIONS

**CEP**	Communication, Ethics, Professionalism
**PIF**	Professional Identity Formation
**SSR**	Systematic Scoping Review
**SEBA**	Systematic Evidence Based Approach
**PICOS**	Population, Intervention, Comparison and Outcome
**BEME**	Best Evidence Medical Education

For peer review only - http://bmjopen.bmj.com/site/about/guidelines.xhtml
For peer review only

STORIES Structured approach to the Reporting In healthcare education of Evidence Synthesis

STATEMENTS AND DECLARATIONS

Ethics Approval and Consent to Participate NA

Consent for Publication NA

Availability of Data and Materials All data generated or analysed during this review are included in this published article and its appendices.

Competing Interests JJQT, GP, DZH, BKYL, AJSL, EJXC, AP, RT, JYHY, YZK, CWNQ, JYL, KTT, YTO, MC, JZ, SM, LW and LKRK have no competing interests.

Funding No funding was received for this review

Author Contributions JJQT, GP, DZH, BKYL, AJSL, EJXC, AP, RT, JYHY, YZK, CWNQ, JYL, KTT, YTO, MC, JZ, SM, LW and LKRK were involved in data curation, formal analysis, investigation, preparing the original draft of the manuscript as well as reviewing and editing the manuscript. All authors have read and approved the manuscript.

ACKNOWLEDGEMENTS

The authors would like to dedicate this paper to the late Dr. S Radha Krishna and A/Prof Cynthia Goh whose advice and ideas were integral to the success of this review and to Thondy, Maia Olivia and Raja Kamarul Ariffin whose lives continue to inspire us. The authors would also like to thank the anonymous reviewers, Dr Ruaraidh Hill and Dr Stephen Mason for their help comments which greatly enhanced this manuscript.

For peer review only

REFERENCES

1. Rankings TU. QS World University Rankings 2020 [Available from: https://www.topuniversities.com/university-rankings/university-subject-rankings/2020/medicine.
2. Deshpande AD. The Use of Sociocultural Constructs in Cancer Screening Research Among African Americans. Cancer Control. 2009;16(3):10.
3. MZJ H. Chinese Familial Tradition and Western Influences: A Case Study in Singapore on Decision Making at the end of life. Journal of Pain and Symptom Management. 2010;40(6):6.
4. Foo WT, Zheng Y, Kwee AK, Yang GM, Krishna L. Factors considered in end-of-life care decision making by health care professionals. Am J Hosp Palliat Care. 2013;30(4):354-8.
5. Chong JA, Quah YL, Yang GM, Menon S, Radha Krishna LK. Patient and family involvement in decision making for management of cancer patients at a centre in Singapore. BMJ Support Palliat Care. 2015;5(4):420-6.
6. Chai HZ, Krishna LK, Wong VH. Feeding: what it means to patients and caregivers and how these views influence Singaporean Chinese caregivers' decisions to continue feeding at the end of life. Am J Hosp Palliat Care. 2014;31(2):166-71.
7. Marc Ho ZJ, Krishna LKR, Goh C, Alethea Yee CP. The physician–patient relationship in treatment decision making at the end of life: A pilot study of cancer patients in a Southeast Asian society. Palliative and Supportive Care. 2013;11(1):13-9.
8. Krishna LKR. Decision making at the end of life: A Singaporean Perspective. Asian Bioeth Rev. 2011;3(2):6.
9. Loh AZ, Tan JS, Jinxuan T, Lyn TY, Krishna LK, Goh CR. Place of Care at End of Life: What Factors Are Associated With Patients' and Their Family Members' Preferences? Am J Hosp Palliat Care. 2016;33(7):669-77.
10. Krishna LK, Watkinson DS, Beng NL. Limits to relational autonomy--the Singaporean experience. Nurs Ethics. 2015;22(3):331-40.
11. Krishna LK, Alsuwaigh R, Miti PT, Wei SS, Ling KH, Manoharan D. The influence of the family in conceptions of personhood in the palliative care setting in Singapore and its influence upon decision making. Am J Hosp Palliat Care. 2014;31(6):645-54.
12. Krishna L. The position of the family of palliative care patients within the decision-making process at the end of life in Singapore. Ethics & Medicine: An International Journal of Bioethics. 2011;27:183+.
13. Hong DZ, Goh JL, Ong ZY, Ting JJQ, Wong MK, Wu J, et al. Postgraduate ethics training programs: a systematic scoping review. BMC Med Educ. 2021;21(1):338.
14. Wong MK, Hong DZH, Wu J, Ting JJQ, Goh JL, Ong ZY, et al. A systematic scoping review of undergraduate medical ethics education programs from 1990 to 2020. Med Teach. 2022;44(2):167-86.
15. Yoon NYS, Ong YT, Yap HW, Tay KT, Lim EG, Cheong CWS, et al. Evaluating assessment tools of the quality of clinical ethics consultations: a systematic scoping review from 1992 to 2019. BMC Med Ethics. 2020;21(1):51.
16. Bok C, Ng CH, Koh JWH, Ong ZH, Ghazali HZB, Tan LHE, et al. Interprofessional communication (IPC) for medical students: a scoping review. BMC Med Educ. 2020;20(1):372.
17. Chia E, Huang H, Goh S, Peries M, Lee C, Tan L, et al. A systematic scoping review of teaching and evaluating communications in the intensive care unit. The Asia Pacific Scholar. 2021;6:3-29.
18. Malhotra C, Kanesvaran R, Krishna L, Xiang L, Kumarakulasinghe NB, Tan SH, et al. Oncologists' responses to patient and caregiver negative emotions and patient perception of quality of communication: results from a multi-ethnic Asian setting. Support Care Cancer. 2018;26(3):957-65.
19. Ng CH, Ong ZH, Koh JWH, Ang RZE, Tan LHS, Tay KT, et al. Enhancing Interprofessional Communications Training in Internal Medicine. Lessons Drawn From a Systematic Scoping Review From 2000 to 2018. J Contin Educ Health Prof. 2020;40(1):27-35.
20. Ong ZH, Tan LHE, Ghazali HZB, Ong YT, Koh JWH, Ang RZE, et al. A Systematic Scoping Review on Pedagogical Strategies of Interprofessional Communication for Physicians in Emergency Medicine. J Med Educ Curric Dev. 2021;8:23821205211041794.
21. Tan XH, Foo MA, Lim SLH, Lim M, Chin AMC, Zhou J, et al. Teaching and assessing communication skills in the postgraduate medical setting: a systematic scoping review. BMC Med Educ. 2021;21(1):483.
22. Venktaramana V, Loh EKY, Wong CJW, Yeo JW, Teo AYT, Chiam CSY, et al. A systematic scoping review of communication skills training in medical schools between 2000 and 2020. Med Teach. 2022;44(9):997-1006.
23. Ong YT, Kow CS, Teo YH, Tan LHE, Abdurrahman A, Quek NWS, et al. Nurturing professionalism in medical schools. A systematic scoping review of training curricula between 1990-2019. Med Teach. 2020;42(6):636-49.

For peer review only - http://bmjopen.bmj.com/site/about/guidelines.xhtml

24. Tay KT, Ng S, Hee JM, Chia EWY, Vythilingam D, Ong YT, et al. Assessing Professionalism in Medicine - A Scoping Review of Assessment Tools from 1990 to 2018. J Med Educ Curric Dev. 2020;7:2382120520955159.
25. Teo YH, Peh TY, Abdurrahman A, Lee ASI, Chiam M, Fong W, et al. A modified Delphi approach to enhance nurturing of professionalism in postgraduate medical education in Singapore. Singapore Med J. 2021.
26. The Student-Physician: Introductory Studies in the Sociology of Medical Education. Journal of the American Medical Association. 1958;166(1):106-7.
27. Teo KJH, Teo MYK, Pisupati A, Ong RSR, Goh CK, Seah CHX, et al. Assessing professional identity formation (PIF) amongst medical students in Oncology and Palliative Medicine postings: a SEBA guided scoping review. BMC Palliat Care. 2022;21(1):200.
28. Quah ELY, Chua KZY, Lua JK, Wan DWJ, Chong CS, Lim YX, et al. A Systematic Review of Stakeholder Perspectives of Dignity and Assisted Dying. J Pain Symptom Manage. 2022.
29. Venktaramana V. Understanding Mentoring Relationships between Mentees, Peer and Senior Mentors. BMC Med Educ. 2023.
30. Toh RQE, Koh KK, Lua JK, Wong RSM, Quah ELY, Panda A, et al. The role of mentoring, supervision, coaching, teaching and instruction on professional identity formation: a systematic scoping review. BMC Med Educ. 2022;22(1):531.
31. Ho CY, Kow CS, Chia CHJ, Low JY, Lai YHM, Lauw SK, et al. The impact of death and dying on the personhood of medical students: a systematic scoping review. BMC Med Educ. 2020;20(1):516.
32. Sarraf-Yazdi S, Teo YN, How AEH, Teo YH, Goh S, Kow CS, et al. A Scoping Review of Professional Identity Formation in Undergraduate Medical Education. J Gen Intern Med. 2021;36(11):3511-21.
33. Vig PS, Lim JY, Lee RWL, Huang H, Tan XH, Lim WQ, et al. Parental bereavement - impact of death of neonates and children under 12 years on personhood of parents: a systematic scoping review. BMC Palliat Care. 2021;20(1):136.
34. Chan NPX, Chia JL, Ho CY, Ngiam LXL, Kuek JTY, Ahmad Kamal NHB, et al. Extending the Ring Theory of Personhood to the Care of Dying Patients in Intensive Care Units. Asian Bioeth Rev. 2022;14(1):71-86.
35. Ngiam LXL, Ong YT, Ng JX, Kuek JTY, Chia JL, Chan NPX, et al. Impact of Caring for Terminally Ill Children on Physicians: A Systematic Scoping Review. Am J Hosp Palliat Care. 2021;38(4):396-418.
36. Chia EWY, Tay KT, Xiao S, Teo YH, Ong YT, Chiam M, et al. The Pivotal Role of Host Organizations in Enhancing Mentoring in Internal Medicine: A Scoping Review. J Med Educ Curric Dev. 2020;7:2382120520956647.
37. Kuek JTY, Ngiam LXL, Kamal NHA, Chia JL, Chan NPX, Abdurrahman A, et al. The impact of caring for dying patients in intensive care units on a physician's personhood: a systematic scoping review. Philos Ethics Humanit Med. 2020;15(1):12.
38. Zhou YC, Tan SR, Tan CGH, Ng MSP, Lim KH, Tan LHE, et al. A systematic scoping review of approaches to teaching and assessing empathy in medicine. BMC Med Educ. 2021;21(1):292.
39. Tay KT, Tan XH, Tan LHE, Vythilingam D, Chin AMC, Loh V, et al. A systematic scoping review and thematic analysis of interprofessional mentoring in medicine from 2000 to 2019. J Interprof Care. 2021;35(6):927-39.
40. Tan R, Qi Ting JJ, Zhihao Hong D, Sing Lim AJ, Ong YT, Pisupati A, et al. Medical Student Portfolios: A Systematic Scoping Review. J Med Educ Curric Dev. 2022;9:23821205221076022.
41. Kow CS, Teo YH, Teo YN, Chua KZY, Quah ELY, Kamal N, et al. A systematic scoping review of ethical issues in mentoring in medical schools. BMC Med Educ. 2020;20(1):246.
42. Huang H, Toh RQE, Chiang CLL, Thenpandiyan AA, Vig PS, Lee RWL, et al. Impact of Dying Neonates on Doctors' and Nurses' Personhood: A Systematic Scoping Review. J Pain Symptom Manage. 2022;63(1):e59-e74.
43. Hong DZ, Lim AJS, Tan R, Ong YT, Pisupati A, Chong EJX, et al. A Systematic Scoping Review on Portfolios of Medical Educators. J Med Educ Curric Dev. 2021;8:23821205211000356.
44. Ho S, Tan YY, Neo SHS, Zhuang Q, Chiam M, Zhou JX, et al. COVID-19 - A Review of the Impact it has made on Supportive and Palliative Care Services Within a Tertiary Hospital and Cancer Centre in Singapore. Ann Acad Med Singap. 2020;49(7):489-95.
45. Hee J, Toh YL, Yap HW, Toh YP, Kanesvaran R, Mason S, et al. The development and design of a framework to match mentees and mentors through a systematic review and thematic analysis of mentoring programs between 2000 and 2015. Mentoring & Tutoring: Partnership in Learning. 2020;28:340-64.
46. Chua WJ, Cheong CWS, Lee FQH, Koh EYH, Toh YP, Mason S, et al. Structuring Mentoring in Medicine and Surgery. A Systematic Scoping Review of Mentoring Programs Between 2000 and 2019. J Contin Educ Health Prof. 2020;40(3):158-68.
47. Chua KZY, Quah ELY, Lim YX, Goh CK, Lim J, Wan DWJ, et al. A systematic scoping review on patients' perceptions of dignity. BMC Palliat Care. 2022;21(1):118.

For peer review only - http://bmjopen.bmj.com/site/about/guidelines.xhtml

48. Quek CWN, Ong RRS, Wong RSM, Chan SWK, Chok AK-L, Shen GS, et al. Systematic scoping review on moral distress among physicians. BMJ Open. 2022;12(9):e064029.
49. Ong RSR, Wong RSM, Chee RCH, Quek CWN, Burla N, Loh CYL, et al. A systematic scoping review moral distress amongst medical students. BMC Med Educ. 2022;22(1):466.
50. Yang LH, Kleinman A, Link BG, Phelan JC, Lee S, Good B. Culture and stigma: adding moral experience to stigma theory. Soc Sci Med. 2007;64(7):1524-35.
51. Krishna LKR, Tan LHE, Ong YT, Tay KT, Hee JM, Chiam M, et al. Enhancing Mentoring in Palliative Care: An Evidence Based Mentoring Framework. Journal of Medical Education and Curricular Development. 2020;7:2382120520957649.
52. Ong YT, Quek CWN, Pisupati A, Loh EKY, Venktaramana V, Chiam M, et al. Mentoring future mentors in undergraduate medical education. PLoS One. 2022;17(9):e0273358.
53. Lim JY, Ong SYK, Ng CYH, Chan KLE, Wu S, So WZ, et al. A systematic scoping review of reflective writing in medical education. BMC Med Educ. 2023;23(1):12.
54. Kamal NHA, Tan LHE, Wong RSM, Ong RRS, Seow REW, Loh EKY, et al. Enhancing education in Palliative Medicine: the role of Systematic Scoping Reviews. Palliative Medicine & Care: Open Access. 2020;7(1):1-11.
55. Ong RRS, Seow REW, Wong RSM, Loh EKY, Kamal NHA, Mah ZH, et al. A Systematic Scoping Review of Narrative Reviews in Palliative Medicine Education. Palliative Medicine & Care: Open Access. 2020;7(1):1-22.
56. Mah ZH, Wong RSM, Seow REW, Loh EKY, Kamal NHA, Ong RRS, et al. A Systematic Scoping Review of Systematic Reviews in Palliative Medicine Education. Palliative Medicine & Care: Open Access. 2020;7(1):1-12.
57. Cleland J, McKimm J, Fuller R, Taylor D, Janczukowicz J, Gibbs T. Adapting to the impact of COVID-19: Sharing stories, sharing practice. Medical Teacher. 2020;42(7):772-5.
58. Peters M, Godfrey C, McInerney P, Soares C, Khalil H, Parker D. The Joanna Briggs Institute reviewers' manual 2015: methodology for JBI scoping reviews2015 April 29, 2019. Available from: http://joannabriggs.org/assets/docs/sumari/Reviewers-Manual_Methodology-for-JBI-Scoping-Reviews_2015_v1.pdf.
59. Peters MD, Godfrey CM, Khalil H, McInerney P, Parker D, Soares CB. Guidance for conducting systematic scoping reviews. Int J Evid Based Healthc. 2015;13(3):141-6.
60. Pham MT, Rajić A, Greig JD, Sargeant JM, Papadopoulos A, McEwen SA. A scoping review of scoping reviews: advancing the approach and enhancing the consistency. Res Synth Methods. 2014;5(4):371-85.
61. Sandelowski M, Barroso J. Handbook for synthesizing qualitative research: springer publishing company; 2006.
62. Reed DA, Beckman TJ, Wright SM, Levine RB, Kern DE, Cook DA. Predictive validity evidence for medical education research study quality instrument scores: quality of submissions to JGIM's Medical Education Special Issue. J Gen Intern Med. 2008;23(7):903-7.
63. Tong A, Sainsbury P, Craig J. Consolidated criteria for reporting qualitative research (COREQ): a 32-item checklist for interviews and focus groups. Int J Qual Health Care. 2007;19(6):349-57.
64. Wong G, Greenhalgh T, Westhorp G, Buckingham J, Pawson R. RAMESES publication standards: meta-narrative reviews. BMC medicine. 2013;11(1):20.
65. Popay J, Roberts H, Sowden A, Petticrew M, Arai L, Rodgers M, et al. Guidance on the conduct of narrative synthesis in systematic reviews. A product from the ESRC methods programme Version. 2006;1:b92.
66. Rider EA, Hinrichs MM, Lown BA. A model for communication skills assessment across the undergraduate curriculum. Med Teach. 2006;28(5):e127-34.
67. Goldie J. Review of ethics curricula in undergraduate medical education. Med Educ. 2000;34(2):108-19.
68. Duffy FD, Gordon GH, Whelan G, Cole-Kelly K, Frankel R, Buffone N, et al. Assessing competence in communication and interpersonal skills: the Kalamazoo II report. Acad Med. 2004;79(6):495-507.
69. David M, Davis M, Harden R, Howie P, Ker J, Pippard M. AMEE Medical Education Guide No. 24: Portfolios as a method of student assessment. Medical teacher. 2001;23:535-51.
70. McEwen L, Griffiths J, Schultz K. Developing and Successfully Implementing a Competency-Based Portfolio Assessment System in a Postgraduate Family Medicine Residency Program. Academic medicine : journal of the Association of American Medical Colleges. 2015;90.
71. Dannefer E, Henson L. The Portfolio Approach to Competency-Based Assessment at the Cleveland Clinic Lerner College of Medicine. Academic medicine : journal of the Association of American Medical Colleges. 2007;82:493-502.
72. Davis M, Ben-David M, Harden R, Howie P, Ker J, McGhee C, et al. Portfolio assessment in medical students' final examination. Medical teacher. 2001;23:357-66.

For peer review only - http://bmjopen.bmj.com/site/about/guidelines.xhtml

73. Ruiz J, Qadri S, Karides M, Castillo C, Milanez M, Roos B. Fellows' Perceptions of a Mandatory Reflective Electronic Portfolio in a Geriatric Medicine Fellowship Program. Educational Gerontology - EDUC GERONTOL. 2009;35:634-52.
74. O'Brien C, Thomas J, Green M. What Is the Relationship Between a Preclerkship Portfolio Review and Later Performance in Clerkships? Academic medicine : journal of the Association of American Medical Colleges. 2017;93.
75. O'Brien C, Sanguino S, Thomas J, Green M. Feasibility and Outcomes of Implementing a Portfolio Assessment System Alongside a Traditional Grading System. Academic medicine : journal of the Association of American Medical Colleges. 2016;91.
76. Jenkins L, Mash B, Derese A. The national portfolio of learning for postgraduate family medicine training in South Africa: Experiences of registrars and supervisors in clinical practice. BMC medical education. 2013;13:149.
77. Shumway J, Harden R. AMEE Guide No. 25: The assessment of learning outcomes for the competent and reflective physician. Medical teacher. 2003;25:569-84.
78. Daboval T, Moore G, Ferretti E. How we teach ethics and communication during a Canadian neonatal perinatal medicine residency: An interactive experience. Medical teacher. 2012;35.
79. Jenkins L, Mash B, Derese A. Development of a portfolio of learning for postgraduate family medicine training in South Africa: A Delphi study. BMC family practice. 2012;13:11.
80. Colbert C, Ownby A, Butler P. A Review of Portfolio Use in Residency Programs and Considerations before Implementation. Teaching and learning in medicine. 2008;20:340-5.
81. Wakeling J, Holmes S, Boyd A, Tredinnick-Rowe J, Cameron N, Marshall M, et al. Reflective Practice for Patient Benefit: An Analysis of Doctors' Appraisal Portfolios in Scotland. Journal of Continuing Education in the Health Professions. 2019;39:1.
82. Torbeck L. A Method for Defining Competency-Based Promotion Criteria for Family Medicine Residents. Academic medicine : journal of the Association of American Medical Colleges. 2005;80:832-9.
83. O'Sullivan P, Reckase M, McClain T, Savidge M, Clardy J. Demonstration of Portfolios to Assess Competency of Residents. Advances in health sciences education : theory and practice. 2004;9:309-23.
84. Yielder J, Moir F. Assessing the Development of Medical Students' Personal and Professional Skills by Portfolio. Journal of Medical Education and Curricular Development. 2016;2016:9.
85. O'Sullivan A, Howe A, Miles S, Harris P, Hughes C, Jones P, et al. Does a summative portfolio foster the development of capabilities such as reflective practice and understanding ethics? An evaluation from two medical schools. Medical teacher. 2012;34:e21-8.
86. Driessen E, Tartwijk J, Vermunt J, Van der Vleuten C. Use of Portfolios in early undergraduate medical training. Medical teacher. 2003;25:18-23.
87. Haffling A-C, Beckman A, Pahlmblad A, Edgren G. Students' reflections in a portfolio pilot: Highlighting professional issues. Medical teacher. 2010;32:e532-40.
88. Fida N, Hassanien M, Shamim M, Alafari R, Zaini R, Mufti S, et al. Students' perception of portfolio as a learning tool at King Abdulaziz University Medical School. Medical Teacher. 2018;40:1-10.
89. Goldie J, Dowie A, Cotton P, Morrison J. Teaching professionalism in the early years of a medical curriculum: A qualitative study. Medical education. 2007;41:610-7.
90. O'Sullivan A, Harris P, Hughes C, Toohey S, Balasooriya C, Velan G, et al. Linking assessment to undergraduate student capabilities through portfolio examination. Assessment & Evaluation in Higher Education. 2012;37:379-91.
91. Franco R, Franco C, Pestana O, Severo M, Ferreira M. The use of portfolios to foster professionalism: attributes, outcomes, and recommendations. Assessment & Evaluation in Higher Education. 2016;42:1-19.
92. Gordon J. Assessing students' personal and professional development using portfolios and interviews. Medical education. 2003;37:335-40.
93. Wong A, Trollope-Kumar K. Reflections: An inquiry into medical students' professional identity formation. Medical education. 2014;48:489-501.
94. Borgstrom E, Cohn S, Barclay S. Medical Professionalism: Conflicting Values for Tomorrow's Doctors. Journal of general internal medicine. 2010;25:1330-6.
95. Lockyer J, Sockalingam S, Campbell DC. Assessment and Change: An Exploration of Documented Assessment Activities and Outcomes by Canadian Psychiatrists. Journal of Continuing Education in the Health Professions. 2018;38:1.
96. Sambandam E, Jutti R, Lee L. Portfolio as a learning tool: Students' perspective. Annals of the Academy of Medicine, Singapore. 2005;34:511-4.
97. Michels N, Avonts M, Peeraer G, Ulenaers K, Gaal L, Bossaert L, et al. Content validity of workplace-based portfolios: A multi-centre study. Medical Teacher. 2016;38:1-10.

For peer review only - http://bmjopen.bmj.com/site/about/guidelines.xhtml

98. Finlay K, Probyn L, Ho S. The CanMEDS Resume: A Useful Educational Portfolio Tool for Diagnostic Radiology Residents. Canadian Association of Radiologists journal = Journal l'Association canadienne des radiologistes. 2011;63:233-6.
99. Clay A, Petrusa E, Harker M, Andolsek K. Development of a web-based, specialty specific portfolio. Medical teacher. 2007;29:311-6.
100. Ozcakar N, Mevsim V, Dilek G. Use of Portfolios in Undergraduate Medical Training: First Meeting With a Patient. Trakya Universitesi Tip Fakultesi Dergisi. 2009;26.
101. Bhattarai M. Study skills course in medical education for postgraduate residents. Kathmandu University medical journal (KUMJ). 2006;5:561-5.
102. Amsellem-Ouazana D, Pee D, Godin V. Use of portfolios as a learning and assessment tool in a surgical practical session of urology during undergraduate medical training. Medical teacher. 2006;28:356-9.
103. Watling C, Brown J. Education Research: Communication skills for neurology residents: Structured teaching and reflective practice. Neurology. 2007;69:E20-6.
104. Goliath C. Diffusion of an E-Portfolio to Assist in the Self-Directed Learning of Physicians: An Exploratory Study. 2009.
105. Azer S. Use of Portfolios by Medical Students: Significance of Critical Thinking. The Kaohsiung journal of medical sciences. 2008;24:361-6.
106. Cheung R. ECLIPPx: An innovative model for reflective portfolios in life-long learning. The clinical teacher. 2011;8:27-30.
107. Rees C, Shepherd M, Chamberlain S. The utility of reflective portfolios as a method of assessing first year medical students' personal and professional development. Reflective Practice. 2005;6:3-14.
108. Driessen E, Tartwijk J, Overeem K, Vermunt J, Van der Vleuten C. Conditions for reflective use of portfolios in undergraduate medical education. Medical education. 2006;39:1230-5.
109. Gavriel J. Portfolios and 'the competent clinician': Past, present and future assessment. Education for Primary Care. 2016;27:1-4.
110. Moores A, Parks M. Twelve tips for introducing E-Portfolios with undergraduate students. Medical teacher. 2010;32:46-9.
111. Rees C, Sheard C. Undergraduate medical students' views about a reflective portfolio assessment of their communication skills. Medical education. 2004;38:125-8.
112. Chaffey L, de Leeuw E, Finnigan G. Facilitating Students' Reflective Practice in a Medical Course: Literature Review. Education for health (Abingdon, England). 2011;25:198-203.
113. Pinsky L, Edwards K. Diving for PERLS: Working and performance portfolios for evaluation and reflection on learning. Journal of general internal medicine. 2004;19:582-7.
114. Gibson C, Chandratilake M, Hull A. Use of portfolios for assessment of global health residents: qualitative evaluation of design and implementation. Canadian medical education journal. 2018;9:e20-e32.
115. Roberts C, Shadbolt N, Clark T, Simpson P. The reliability and validity of a portfolio designed as a programmatic assessment of performance in an integrated clinical placement. BMC Medical Education. 2014;14:197.
116. Dolan B, O'Brien C, Cameron K, Green M. A Qualitative Analysis of Narrative Preclerkship Assessment Data to Evaluate Teamwork Skills. Teaching and Learning in Medicine. 2018;30:1-9.
117. Fins J, Kodish E, Cohn F, Danis M, Derse A, Dubler N, et al. A Pilot Evaluation of Portfolios for Quality Attestation of Clinical Ethics Consultants. The American journal of bioethics : AJOB. 2016;16:15-24.
118. Naidu T, Ramlall S. Establishing psychiatric registrars' competence in psychotherapy: A portfolio based model. African journal of psychiatry. 2008;11:264-71.
119. Murphy D, Aitchison P, Hernandez Santiago V, Davey P, Mires G, Nathwani D. Insightful Practice: A robust measure of medical students' professional response to feedback on their performance. BMC Medical Education. 2015;15.
120. Ekayanti F, Risahmawati R, Fadhilah M. Portfolio Assessment Implementation in Clinical Year of Community Medicine Module: Students Perspective2017.
121. Hall P, Byszewski A, Sutherland S, Stodel E. Developing a Sustainable Electronic Portfolio (ePortfolio) Program That Fosters Reflective Practice and Incorporates CanMEDS Competencies Into the Undergraduate Medical Curriculum. Academic medicine : journal of the Association of American Medical Colleges. 2012;87:744-51.
122. Menon V. Portfolios for post graduate psychiatry residents: Potentials and pitfalls. Asian Journal of Psychiatry. 2014;11.
123. Prayson R, Bierer SB, Dannefer E. Medical student resilience strategies: A content analysis of medical students' portfolios. Perspectives on medical education. 2016;6.
124. Fragneto R, Dilorenzo A, Schell R, Bowe E. Evaluating Practice-Based Learning and Improvement: Efforts to Improve Acceptance of Portfolios. Journal of graduate medical education. 2010;2:638-43.

For peer review only

125. Austin C, Braidman I. Support for portfolio in the initial years of the undergraduate medical school curriculum: What do the tutors think? Medical teacher. 2008;30:265-71.
126. Arntfield S, Parlett B, Meston C, Apramian T, Lingard L. A model of engagement in reflective writing-based portfolios: Interactions between points of vulnerability and acts of adaptability. Medical teacher. 2015;38:1-10.
127. Pitkälä K, Mäntyranta T. Feelings related to first patient experiences in medical school: A qualitative study on students' personal portfolios. Patient education and counseling. 2004;54:171-7.
128. Tigelaar D, Dolmans D, Grave W, Wolfhagen I, Van der Vleuten C. Portfolio as a tool to stimulate teachers' reflections. Medical teacher. 2006;28:277-82.
129. Singh R, Naughton B, Taylor J, Koenigsberg M, Anderson D, McCausland L, et al. A comprehensive collaborative patient safety residency curriculum to address the ACGME core competencies. Medical education. 2006;39:1195-204.
130. Dannefer E, Bierer SB, Gladding S. Evidence within a portfolio-based assessment program: What do medical students select to document their performance? Medical teacher. 2012;34:215-20.
131. Rees C, Sheard C. The reliability of assessment criteria for undergraduate medical students' communication skills portfolios: The Nottingham experience. Medical education. 2004;38:138-44.
132. Kassab S, Bidmos M, Nomikos M, Daher-Nashif S, Kane T, Sarangi S, et al. Construct Validity of an Instrument for Assessment of Reflective Writing-Based Portfolios of Medical Students. Advances in Medical Education and Practice. 2020;Volume 11:397-404.
133. Michels N, Driessen E, Muijtjens A, Van Gaal L, Bossaert L, De Winter B. Portfolio assessment during medical internships: How to obtain a reliable and feasible assessment procedure? Education for health (Abingdon, England). 2009;22:313.
134. Susan E, Kinnair D. Integrating the assessment of interprofessional education into the health care curriculum. Journal of Taibah University Medical Sciences. 2016;11.
135. Bierer SB, Dannefer E. Does Students' Gender, Citizenship, or Verbal Ability Affect Fairness of Portfolio-Based Promotion Decisions? Results From One Medical School. Academic medicine : journal of the Association of American Medical Colleges. 2011;86:773-7.
136. Bierer SB, Dannefer E, Tetzlaff J. Time to Loosen the Apron Strings: Cohort-based Evaluation of a Learner-driven Remediation Model at One Medical School. Journal of General Internal Medicine. 2015.
137. Dannefer E. Beyond assessment of learning toward assessment for learning: Educating tomorrow's physicians. Medical teacher. 2013;35.
138. Dannefer E, Prayson R. Supporting students in self-regulation: Use of formative feedback and portfolios in a problem-based learning setting. Medical teacher. 2013;35.
139. Deitte L. Learning Portfolios in Radiology Residency Education: How Do I Get Started? Journal of the American College of Radiology : JACR. 2008;5:664-9.
140. Vance G, Williamson A, Frearson R, O'Connor N, Davison J, Steele C, et al. Evaluation of an established learning portfolio. The clinical teacher. 2013;10:21-6.
141. Suraseranivongse S, Chinachoti T, Aroonpruksakul N, Halilamien P, Rushatamukayanunt P, Raksamani K, et al. Development of a learning portfolio to assess the competency of anesthesia residents in Thailand. Asian Biomedicine. 2011;5.
142. Yates J, James D. Risk factors at medical school for subsequent professional misconduct: multicentre retrospective case-control study. BMJ. 2010;340:c2040.
143. Peeters MJ, Sexton ME. Use of reflective writing within interprofessional education: a mixed-methods analysis. J Interprof Care. 2020;34(3):307-14.
144. Remein CD, Childs E, Pasco JC, Trinquart L, Flynn DB, Wingerter SL, et al. Content and outcomes of narrative medicine programmes: a systematic review of the literature through 2019. BMJ Open. 2020;10(1):e031568.
145. Younas A, Ansar A. DEVELOPING A CURRICULUM FOR PROFESSIONAL ETHICS IN UNDERGRADUATE MEDICAL AND DENTAL EDUCATION: EVIDENCE FROM LITERATURE. 2020;70:1211-3.
146. Haig A, Dozier M. BEME Guide no 3: systematic searching for evidence in medical education--Part 1: Sources of information. Medical teacher. 2003;25(4):352-63.
147. Gordon M, Gibbs T. STORIES statement: publication standards for healthcare education evidence synthesis. BMC medicine. 2014;12(1):143.
148. Dawe R, Pike A, Kidd M, Janakiram P, Nicolle E, Allison J. Enhanced skills in global health and health equity: Guidelines for curriculum development. Canadian Medical Education Journal. 2017;8:e48-e60.
149. Sagasser M, Kramer A, Van der Vleuten C. How do postgraduate GP trainees regulate their learning and what helps and hinders them? A qualitative study. BMC medical education. 2012;12:67.
150. Burch V. Portfolios for assessment and learning: Guide Supplement 45.1 – Viewpoint. Medical teacher. 2011;33:1029-31.

For peer review only - http://bmjopen.bmj.com/site/about/guidelines.xhtml

Appendices

Appendix A. SEBA Methodology

Appendix B. Search Strategies

Appendix C. Tabulated Summaries

Appendix D. Portfolio Content (Competencies Assessed and Assessment Modalities)

Figure Legend

Figure 2. The SEBA Process

Figure 2. PRISMA Flowchart

For peer review only

For peer review only - http://bmjopen.bmj.com/site/about/guidelines.xhtml

Figure 1. *The SEBA Process*

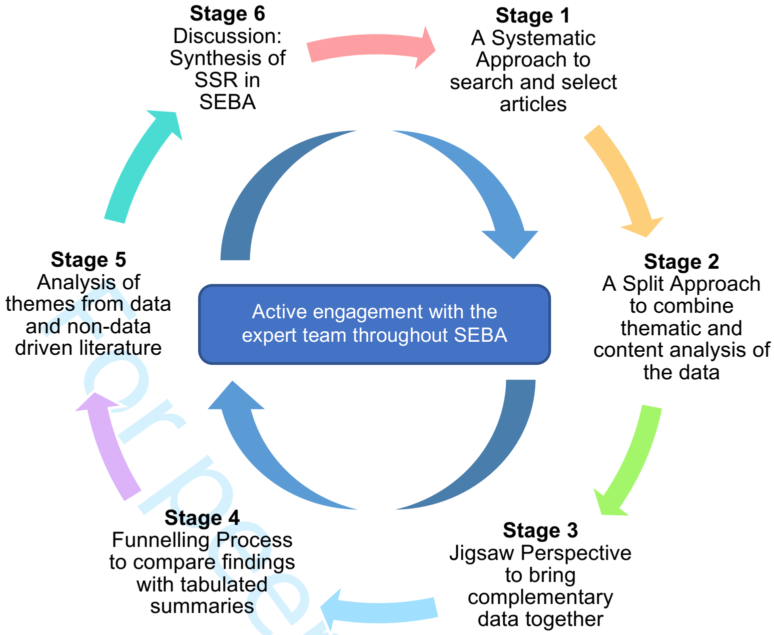

For peer review only

Figure 2. *PRISMA Flowchart*

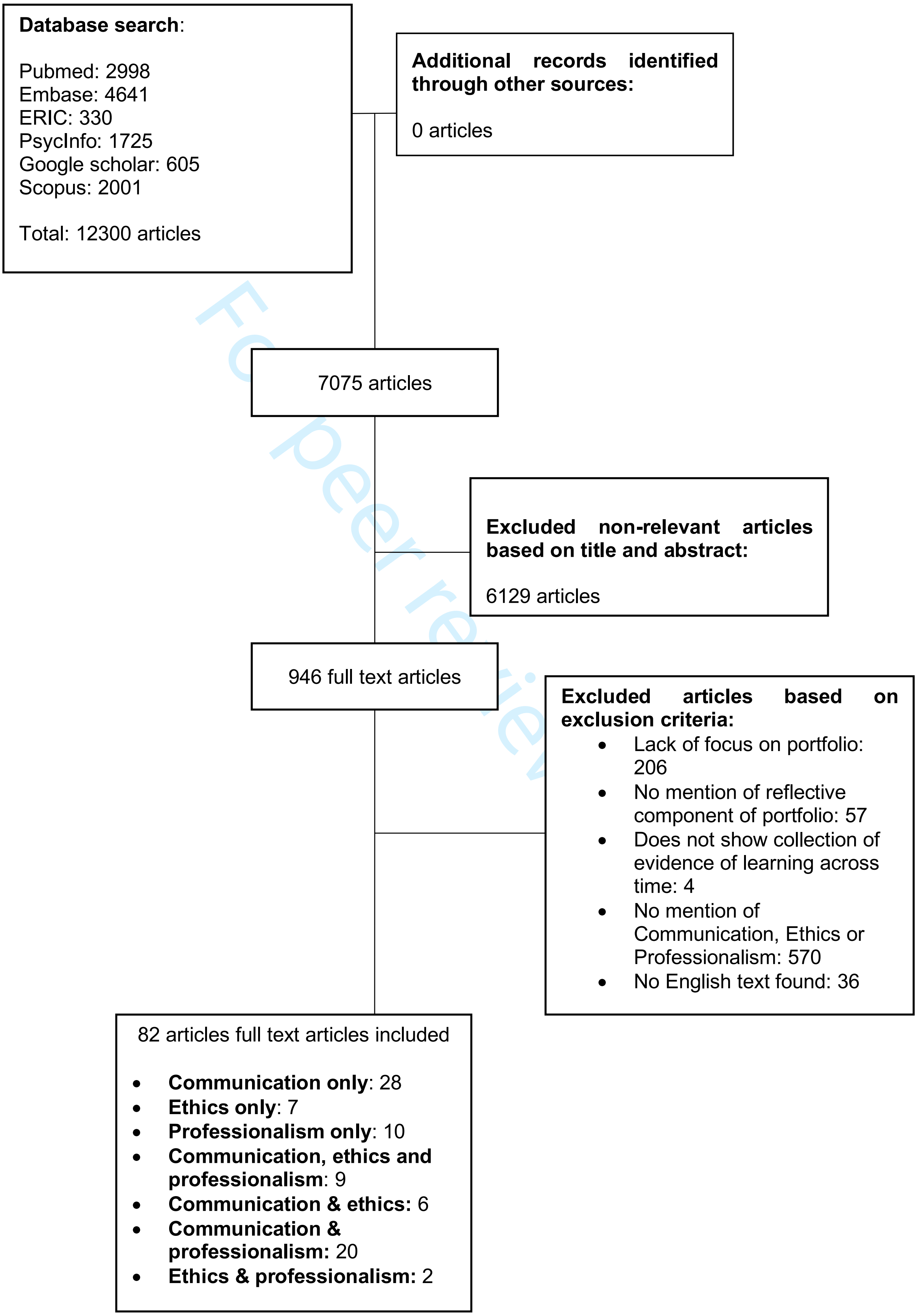

Appendices

Appendix A. SEBA Methodology

Theoretical lens

Krishna's Systematic Evidence-Based Approach (SEBA) is adopted to guide this systematic scoping review (SSR) (henceforth SSR in SEBA) (1-8). SEBA is composed of six stages- 1) Systematic Approach, 2) Split Approach, 3) Jigsaw Perspective, 4) Funnelling Process 5) Analysis of data and non-data driven literature, and 6) Discussion Synthesis (Figure 1). SEBA systematically map available data, structure the extraction of key characteristics of CEP education and its effects, synthesise and summarise actionable and applicable information across a diverse range of study formats and identify gaps in knowledge in current concepts. SEBA's constructivist approach (8-13) and relativist lens (14-17) acknowledges development of CEP competencies are individualised sociocultural constructs informed by the user's narratives, clinical competencies, contextual and environmental considerations.

Expert advice

An expert team consisting of a medical librarian from the Yong Loo Lin School of Medicine (YLLSoM) at the National University of Singapore (NUS), and local educational experts and clinicians at the National Cancer Centre Singapore (NCCS), the Palliative Care Unit, and the Institute of Population Health at the University of Liverpool, YLLSoM and Duke-NUS Medical School, ensured that the SEBA methodology was employed in a consistent manner within accepted practices.

Reflexivity

Personal reflexivity

Recognising that the research team are informed by their interests, narratives, clinical and research insights and contextual considerations, membership to the research and expert teams were made up of experienced physician-tutors, psychologists, methodologists, and educational scholars. Six members of these teams hold masters degrees in medical education, two hold masters degrees in ethics, two hold masters degrees in clinical research and two hold masters degrees in public health. Most of the expert and research team were experienced in quantitative research however five members of the team are experienced in qualitative methods. Six of the research and expert teams have published articles in peer-reviewed journals using the SEBA methodology. One member of the expert team is an experienced researcher at the Palliative Care Institute Liverpool and another is a member of the Health Data Science Department at the University of Liverpool. Both researchers from the University of Liverpool have

For peer review only

been collaborating with the research team on a number of studies pertaining to portfolio use, death and dying, moral distress, PIF and mentoring and are part of the team expanding the use of the SEBA methodology in medical education and palliative care.

Three senior members of the team are working on various projects on medical education and three others have already published articles in peer-reviewed journals in medical education. Six members of the research team were members of the PMI and have published SEBA guided reviews on mentoring, dignity and medical education. To ensure input from all members of the team, synchronous and asynchronous in-person and online meetings and Sandelowski and Barroso (18)'s approach to 'negotiated consensual validation' was used to reach consensus on the issues discussed.

Methodological reflexivity

Adopting a structured constructivist approach, we sought to build a holistic concept of current models of portfolios within medical education acknowledging however that we were limited by manpower and time constraints. We included articles featured in grey and bibliographic databases within the dates set in our selection criteria. Much of these theories required deeper consideration and discussions and we documented our discussions and decisions.

Iterative process

To ensure transparency and accountability, the expert team was involved in all stages of SEBA as part of the iterative process.

For peer review only

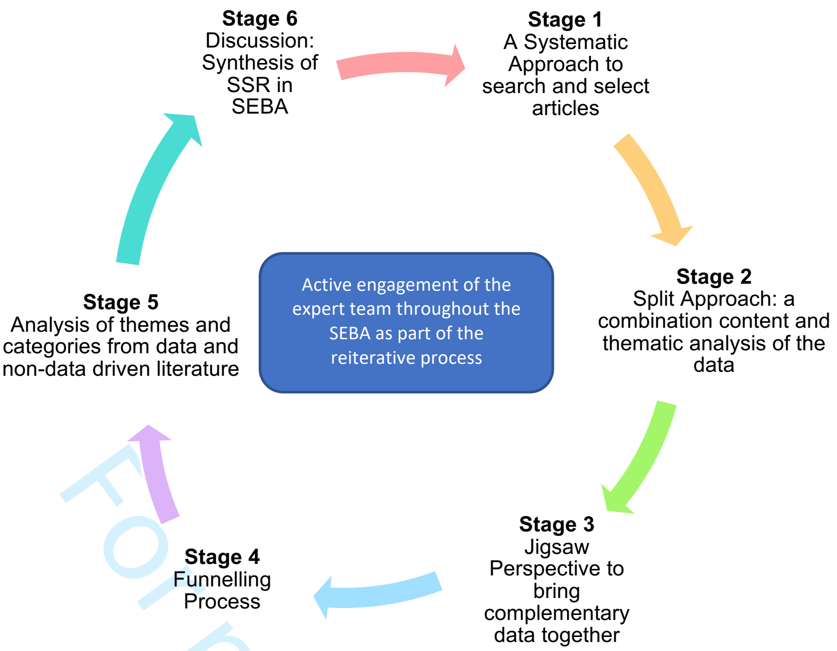

Figure 1. The SEBA Process

For peer review only

Stage 1 of SEBA: Systematic Approach

The SEBA methodology begins with the research and expert teams agreeing upon the research questions, the search terms and the databases to be scrutinised. In this case the research and expert team determined the primary research question: "*What is known about CEP portfolios?*". The secondary research questions were "*what role do CEP portfolios have in teaching and assessing CEP development?*".

Inclusion criteria

A PICOs (Population, Intervention, Comparison, Outcome, Study design) format was adopted to guide the research process (Table 1). There was no comparison group.

Table 1. *PICOS Inclusion and Exclusion Criteria*

PICOS	Inclusion criteria	Exclusion criteria
**Population**	• Undergraduate and postgraduate medical students<br>• Qualified medical doctors, physician or resident; medical officer, registrar, house officer, attending, consultant	• Allied health specialties such as Pharmacy, Dietetics, Chiropractic, Midwifery, Podiatry, Speech Therapy, Occupational and Physiotherapy<br>• Non-medical specialties such as Clinical and Translational Science, Alternative and Traditional Medicine, Veterinary, Dentistry
**Intervention**	• portfolios in undergraduate and postgraduate medical education for teaching and assessment of Communication, Ethics and Professionalism<br><br>Criteria of a portfolio:	Other documentation methods or learning tools that are:<br>• Not longitudinal or single timepoint<br>• Does not include personal intellectual engagement with the content and associated learning (for instance, curriculum vitae, logbooks

PICOS	Inclusion criteria	Exclusion criteria
	• longitudinal (more than a single timepoint) assessment data<br>• candidate's personal engagement with portfolio content and associated learning<br>• Interventions meeting the above criteria were included regardless of whether they were referred to as portfolios<br><br>All types of portfolios were included in the study:<br>• For instance: electronic & non-electronic; formative & summative or combined; clinical & non-clinical<br>• Portfolios with input from students and/or residents and/or doctors, and/or input from faculty members and other individuals<br>• Portfolios with different structures: extent by which the structure has been prescribed and/or left to individual discretion	and the use of personal digital assistants)
**Comparison/ Context**	NA	NA
**Outcome**	Papers that measured the following outcomes were included:<br>• Effectiveness of the use of portfolios to assess and teach Communication, Ethics and Professionalism<br>• Impact of the use of portfolios on medical students (both undergraduate and postgraduate)<br>• Impact of the use of portfolios on the faculty	NA
**Study design**	• Articles in English or translated to English<br>• Articles published from 1st January 2000 to 31st December 2020<br>• Databases: PsycINFO, EMBASE, PubMed, ERIC, Scopus, Google Scholar<br>• All study designs including:<br>• Mixed methods research, meta-analyses, systematic reviews, randomised controlled trials, cohort studies, case-control studies, cross-sectional studies, descriptive papers, grey literature, opinions, letters, commentaries and editorials	NA

Three members of the research team carried out independent ancestry searches of seven leading journals in medical education (Academic Medicine, Medical Education, Medical Teacher, Advances Health Sciences Education, BMC Medical Education, Teaching and Learning in Medicine and Perspectives on Medical Education) accessed through the NUS library portal. In keeping with Pham, Rajic (19)'s recommendations, the searches were restricted to articles published between 1st January 2000 and 31st December 2020 to accommodate to existing manpower and time constraints. Quantitative, mixed and qualitative research methodologies meeting the inclusion criteria were included.

Table 2. *Search Strategy for PubMed, Embase, PsycINFO, ERIC, Scopus, and Google Scholar databases.*

		Mesh Terms	tiab
		(1 OR 2 OR 3) AND 4	
**Population**	Medical Students OR Doctors	[1] "Physicians"[MeSH] OR "Students, Medical"[MeSH] OR "Clinical Clerkship"[MeSH] OR "Medicine"[Mesh] OR "Education, Medical"[Mesh] OR "Clinical Competence"[Mesh]	[2] Physician[tiab] OR Physicians[tiab] OR resident[tiab] OR residents[tiab] OR residency[tiab] OR residencies[tiab] OR practice[tiab] OR practitioner[tiab] OR practitioners[tiab] OR doctor[tiab] OR doctors[tiab] OR houseman[tiab] OR housemanship[tiab] OR housemen[tiab] OR medical[tiab] OR clinical[tiab] OR pre-clinical[tiab] OR preclinical[tiab] OR clinician*[tiab] OR surgery[tiab] OR surgical[tiab] OR surgeon*[tiab] OR clerkship*[tiab] OR specialist*[tiab]
			[3] ("Educational Measurement/methods"[Mesh] OR "Educational Measurement/standards"[Mesh] OR "Documentation/methods"[Mesh] OR "Benchmarking*"[MeSH] OR "Competency-based education/standards*"[MeSH] OR "Records*"[MeSH]) AND (medical[tiab] OR clinical[tiab] OR pre-clinical[tiab] OR preclinical[tiab] OR clinician*[tiab] OR surgery[tiab] OR surgical[tiab] OR surgeon*[tiab] OR clerkship*[tiab] OR specialist*[tiab])
**Intervention**	Portfolios		[4] Portfolio[tiab] OR portfolios[tiab] OR e-portfolio[tiab] OR e-portfolios[tiab] OR "curriculum vitae"[tiab] OR "personal statement"[tiab] OR "personal statements"[tiab]

Members of the research team carried out independent searches of databases. To facilitate this approach, the search process saw experienced senior researchers well-versed in carrying out systematic reviews and systematic scoping reviews each meet with a team of two to three medical students to guide them database searches. This approach was to enhance training of new researchers and to ensure that at least two teams were independently reviewing each database. Each team met regularly and discussed their findings. After a search of the first 100 articles in a particular database, the medical students and the senior researcher compared their findings at an online meeting.

Subsequently, the teams met at specific time points, often after reviewing a predetermined number of included articles to discuss their concerns, exchange opinions and advance their understanding of the research process and the area of study. Sandelowski and Barroso (18)'s 'negotiated consensual validation' was used to achieve consensus on the final list of titles to be reviewed. The teams repeated this process, independently studying all the

For peer review only

full text articles on the final list of titles, creating their own lists of articles to be included and discussing their findings at weekly online research meetings over the first 4 months of the project. Consensus on the final list of articles to be analysed was achieved following at least three online meetings when discrepancies were reviewed and discussed by each member of the research team.

As this was a training process for many of the participants who were medical students participating in the Palliative Medicine Initiative, senior mentors and peer-mentors were frequently involved in guiding and discussing individual findings over and above the online meetings especially at the start of each stage of the research process. Interrater reliability was not evaluated.

Stage 2 of SEBA: Split Approach

Krishna's 'Split Approach' (18-23) was employed to enhance the reliability of the data analyses. This saw three groups of researchers independently analysing the included articles.

The first team summarised and tabulated the included full-text articles in keeping with recommendations drawn from Wong, Greenhalgh (24)'s RAMESES publication standards: meta-narrative reviews and Popay, Roberts (25)'s "Guidance on the conduct of narrative synthesis in systematic reviews". The tabulated summaries served to ensure that key aspects of included articles were not lost.

Concurrently, the second team analysed the included articles using Braun and Clarke (26)'s approach to thematic analysis. In Phase 1, the research team carried out independent reviews, 'actively' reading the included articles to find meaning and patterns in the data. In Phase 2, 'codes' were constructed from the 'surface' meaning and collated into a code book to code and analyse the rest of the articles using an iterative step-by-step process. As new codes emerged, these were associated with previous codes and concepts. In Phase 3, the categories were organised into themes that best depict the data. An inductive approach allowed themes to be "defined from the raw data without any predetermined classification" (27). In Phase 4, the themes were refined to best represent the whole data set and discussed. In Phase 5, the research team discussed the results of their independent analysis online and at reviewer meetings. 'Negotiated consensual validation' was used to determine a final list of themes approach and ensure the final themes.

A third team of researchers employed Hsieh and Shannon (28)'s approach to directed content analysis to analyse the included articles. Analysis using the directed content analysis approach involved "identifying and operationalizing a priori coding categories". The categories employed in the content analysis for undergraduate communications were Rider et al. (29) 's *"A model for communication skills assessment across the undergraduate curriculum",* Goldie (30)'s *"Review of ethics curricula in undergraduate medical education"*, Duffy et al. (31)'s *"Assessing Competence in Communication and Interpersonal Skills: The Kalamazoo II Report"* and Hong et al. (32)'s *"Postgraduate Ethics Training Programs: A Systematic Scoping Review"*. Tay et al. (33)'s *"Assessing Professionalism in Medicine - A Scoping Review of Assessment Tools from 1990 to 2018"* was employed for codes for professionalism and David (34)'s article *"AMEE Medical Education Guide No. 24: Portfolios as a method of student assessment"* was then used to contextualise their use in portfolios. Any data not captured by these codes were assigned a new code.

By using directed content analysis, this "Split Approach" sought to address shortcomings int thematic analysis. This was done by ironing out disparities in data and increases the validity of the identified themes (35). It also limits inherent biases and condenses the interpretations of terminology used by each team's members. Consistency with existing literature is further offered by directed content analysis, by using existing data to identify codes and categories. The codes were used systematically and objectively, strengthening the validity and reliability of a positivist approach (35, 36). The transparency of this approach is enforced by clearly defined categories, together with references throughout the analytical process.

Stage 3 of SEBA: Jigsaw Perspective

The themes and categories identified in the Split Approach were viewed as pieces of a jigsaw puzzle where overlapping/complementary pieces were combined to create a bigger piece of the puzzle referred to as themes/categories. This process was guided by Phases 4 to 6 of France, Uny (37)'s adaptation of Noblit, Hare (38)'s seven phases of meta-ethnography. As per Phase 4, the themes and the categories identified in the Split Approach are grouped together according to their focus. These groupings of categories and themes were then contextualised through the review of the articles from which they were drawn from. Reciprocal translation was used to determine if the themes and categories could be used interchangeably. This allowed the themes and categories to be combined to form themes/categories.

France, Uny's adaptation:
Phase 1: Getting started, deciding on the focus of the synthesis.
Phase 2: Deciding what is relevant to the initial interest.
Phase 3: Reading the Studies.
Phase 4: Determining how the studies are related.
Phase 5: Translating the studies into one another.
Phase 6: Synthesising the translations.
Phase 7: Expressing the synthesis.

Codes derived from thematic analysis (TA): indications, characteristics and strengths and limitations

Codes derived from directed content analysis (DCA): indication for portfolio, portfolio content, portfolio design and strengths and limitations

Overlaps between indications and strengths and limitations allowed the combination of these categories and themes creating two themes/categories called indications and strengths and limitations. Careful review of the categories- portfolio content and portfolio design were subsets of the theme characteristics. For a more accurate presentation, the overlaps were conflated and presented together with the TA codes indicated under the DCA code. (Refer to Domain 4 table)

**Themes Identified**	**Categories Identified**	**Domains Created**

For peer review only

Indications	indications	Indications
characteristics	content	Characteristics
	Portfolio design	
Strengths and limitations	Strengths and limitations	Dignity-conserving Measures

For peer review only

Stage 4 of SEBA: Funnelling Process

The Funnelling Process employs Phases 3 to 5 of France, Uny's adaptation to juxtapose the themes/categories with key messages identified in the tabulated summaries to create domains. These domains form the basis for 'the line of argument' in Stage 6 of SEBA.

For peer review only

For peer review only

For peer review only - http://bmjopen.bmj.com/site/about/guidelines.xhtml

For peer review only

For peer review only - http://bmjopen.bmj.com/site/about/guidelines.xhtml

Domain 2. Relationship between Dignity, WTHD and Assisted Dying

Subdomains	Codes
**Increasing WTHD**	▪ Loss of dignity (39-81)<br>▪ Fear of loss of dignity (42, 44, 52, 59, 82, 83)<br>▪ Loss of autonomy (39, 40, 42, 45, 47, 50-53, 55, 59, 62, 65, 69, 70, 73, 74, 77, 79, 81, 82, 84-88)<br>▪ Fear of loss of autonomy (42, 54, 70, 89)
**Decreasing WTHD/protective factors**	▪ Addressing of dignity related issues improves health-related quality of life (46)<br>▪ Improving dignity reduces desire for euthanasia (50)
**Effect of assisted dying on dignity**	▪ Reclamation/preservation of dignity (45, 49, 58, 90-95)<br>▪ Lack of assisted dying interferes with dignity (50, 76, 96)<br>▪ Reclamation/preservation of autonomy (43, 45, 49, 51, 52, 67, 73, 74, 77, 85, 88, 92, 94, 97-100)<br>▪ Lack of assisted dying interferes with autonomy (49, 96)<br>▪ Violation of autonomy (100)

For peer review only

Domain 3. Stakeholder Perspectives on Dignity

Subdomains	Codes	
**Patient Perspectives**	**Positive Aspects**<br>▪ Respect for humanity (67)<br>▪ Right to die (99)<br>▪ Unaffected by fear of death (48)<br>▪ Good death (67, 73, 99, 101-103)<br>- Maintaining control of their death (67, 73, 99, 101-103)<br>- Ending suffering (67, 73)<br>▪ Perceived futility (73, 74)<br>▪ General support (73, 98)<br>▪ Wanting the option for themselves (98)	**Negative Aspects**<br>▪ Religious concerns (67, 73, 102)<br>▪ Ethical concerns (62, 67, 73)<br>- Inability to ascertain patient consent (62, 67)<br>- Equating to murder/suicide (67)<br>▪ Change in ideologies (50)<br>▪ Deterioration of patient-doctor relationship (58, 62, 74, 101)<br>- Loss of faith in palliative/hospice care (62, 101)<br>- Feeling of abandonment when WTHD is turned down (74)<br>- Poor communications (58)<br>▪ Against legislation (73)
**Healthcare Provider**<br><br>**Perspectives**	**Positive Aspects**<br>▪ Good death for patients (42, 47, 49, 50, 54, 56, 57, 61, 69, 70, 82, 87, 101, 103)<br>- Fulfilling patients' desire for control (47, 50, 56, 61, 82, 87, 101, 104)<br>- Ending suffering (42, 54, 57, 61, 69, 70, 103)<br>- Not requiring a long life (49)<br>▪ Feeling helpless (68)<br>▪ Non-abandonment of patients (90)<br>▪ Reducing patients' family burden (90)<br>▪ Avoiding unlawful hastening death (50)<br>▪ General support (40, 47, 62, 68-70, 75, 82, 90, 101, 103-105)<br>▪ Disregarding the need for alternatives (i.e. palliative care) (50)	**Negative Aspects**<br>▪ Religious concerns (106)<br>▪ Disrespecting patients' autonomy by not entertaining their wishes (104, 107)<br>▪ Emotional/moral distress (49, 50, 69, 91, 101, 108-113)<br>- Guilt (91, 112)<br>- Opposing medical principles (49, 69, 101, 108, 110, 111)<br>- Grief, avoidance after carrying out WTHD (113)<br>- Turning doctors into killers (111)<br>▪ No right to die (90)<br>▪ Unable to ascertain if patient's disease is incurable (109)<br>▪ Change in perceptions after disease (82)<br>▪ WTHD should not involve family members (113)<br>▪ "Slippery slope" (110)<br>▪ Societal pressure (55, 74)<br>▪ Disapproval of WTHD (62, 69, 75, 82, 101, 107, 113-115)<br>- Disagreement with the law (115)<br>- Reluctance to carry out WTHD (75, 101)<br>▪ Proposing alternatives (57, 62, 63, 90, 105, 111)<br>- Improving quality of life of dying patients (62, 111)<br>- Better palliative care (63, 90, 105)<br>- Healing (57)<br>▪ Death as an unknown (57)

For peer review only

		▪ Disapproval of 'tired of living' as a reason for WTHD (57)
**Lawmaker Perspectives**	▪ Belief in a right to die (76)<br>▪ Avoiding unlawful hastening death (96)<br>▪ Government's duty to consider patients' WTHD (74)<br>▪ Approval of WTHD (76, 100)	▪ "Slippery slope" (98, 114)<br>- Risk of abuse (114)<br>▪ Instigating social pressure (on the elderly) (74)<br>▪ Disapproval of WTHD (74, 83, 106, 114)

For peer review only

Domain 4. Dignity Conserving Measures

Subdomains	Codes
**Advantages to Dignity Conserving Measures**	▪ Palliative services<br>- Palliative care (40, 44, 47, 49, 50, 62, 63, 66, 73, 82, 90, 91, 103, 111, 116-118)<br>- Palliative sedation (47, 49, 91, 119)<br>- Palliative starvation (85, 109)<br>▪ Pain and symptom management<br>- Control of physical pain (58, 66, 75, 104, 107, 108, 119)<br>- Control of psychological symptoms (75, 102, 120)<br>▪ Hospice care (46, 47, 75, 100, 106, 107, 117)<br>▪ Holistic approach (43, 57, 105, 111, 112, 117, 121, 122)<br>▪ Ease of access to information (47, 57, 71, 87, 92, 100, 112, 117)<br>▪ Distributive justice (55)<br>▪ Social support<br>- Religious (75)<br>- Familial (45)<br>- Healthcare providers (47, 57, 58, 71, 87, 112)
**Limitations to Dignity Conserving Measures**	▪ Cannot completely address:<br>- Existential suffering (54)<br>- Pain (50)<br>- Complete loss of mobility (103)<br>- Extreme shortness of breath (103)<br>- Fear of sudden and rapid bleeding (103)<br>- Ongoing stress of losing autonomy and dignity (103)<br>▪ Side effects of pain medication (58)

For peer review only

Stage 5 of SEBA: Analysis of data and non-data driven literature

A novel aspect of the SEBA methodology has been its inclusion of position, perspective, conference, reflective and opinion papers, editorials, commentaries, letters, posters, oral presentations, forum discussions, interviews, blogs, governmental reports, policy statements and surveys from PubMed, Embase, PsycINFO, Cochrane Database of Systematic Reviews, CINAHL, Scopus databases and the Journal of Pain and Symptom Management, BMC Palliative Medicine, Death Studies, and Palliative Medicine. With many current survey and assessment tools not able to capture the intricate connections and personalised nature of wider concepts of dignity, assisted dying, WTHD, personhood and identity, these resources are a rich source of information. These resources also capture wider patient, HCP and lawmaker perspectives and offer information on ethical, existential, and societal considerations often excluded by traditional systematic reviews as evidenced by recent reviews into how physicians and patients deal with death and dying and moral distress (123-130)

The use of non-data driven articles such as position, perspective, conference, reflective and opinion papers, editorials, commentaries, letters, posters, oral presentations, forum discussions, interviews, blogs, governmental reports, policy statements and surveys is consistent with a constructivist approach that includes all forms of data. In addition, use of non-data driven articles is a valuable source of information for subject matter such as concepts of dignity which are notoriously difficult to study and open to sociocultural considerations.

However, with some of these sources neither peer-reviewed nor necessarily evidence-based, they were differentiated from primary research, compiled and thematically analysed. The themes identified from primary data sources and non-evidence based and/or tertiary data sources were then compared to enhance further the accountability and reproducibility.

With the themes from the two groups found to be similar, the expert and research teams were satisfied that their inclusion did not bias the overall data.

Stage 6 of SEBA: Synthesis of the Discussion

The Best Evidence Medical Education (BEME) Collaboration Guide (131) and the Structured approach to the Reporting In healthcare education of Evidence Synthesis (STORIES) (132) were used to guide the discussion.

For peer review only

References

1. Kow CS, Teo YH, Teo YN, Chua KZY, Quah ELY, Kamal NHBA, et al. A systematic scoping review of ethical issues in mentoring in medical schools. BMC Medical Education. 2020;20(1):1-10.
2. Ngiam LXL, Ong YT, Ng JX, Kuek JTY, Chia JL, Chan NPX, et al. Impact of Caring for Terminally Ill Children on Physicians: A Systematic Scoping Review. Am J Hosp Palliat Care. 2020:1049909120950301.
3. Krishna LKR, Tan LHE, Ong YT, Tay KT, Hee JM, Chiam M, et al. Enhancing Mentoring in Palliative Care: An Evidence Based Mentoring Framework. Journal of Medical Education and Curricular Development. 2020;7:2382120520957649.
4. Chia EWY, Huang H, Goh S, Peries MT, Lee CCY, Tan LHE, et al. A Systematic Scoping Review of Teaching and Evaluating Communications in The Intensive Care Unit. The Asia-Pacific Scholar. In Press.
5. Kuek JTY, Ngiam LXL, Kamal NHA, Chia JL, Chan NPX, Abdurrahman ABHM, et al. The impact of caring for dying patients in intensive care units on a physician's personhood: a systematic scoping review. 2020;15(1):1-16.
6. Hong DZ, Lim AJS, Tan R, Ong YT, Pisupati A, Chong EJX, et al. A Systematic Scoping Review on Portfolios of Medical Educators. Journal of Medical Education and Curricular Development. 2021;8:23821205211000356.
7. Goh S, Wong RSM, Quah ELY, Chua KZY, Lim WQ, Ng ADR, et al. Mentoring in palliative medicine in the time of covid-19: a systematic scoping review. BMC medical education. 2022;22(1):1-15.
8. Bok C, Ng CH, Koh JWH, Ong ZH, Ghazali HZB, Tan LHE, et al. Interprofessional communication (IPC) for medical students: a scoping review. BMC Med Educ. 2020;20(1):372.
9. Ng YX, Koh ZYK, Yap HW, Tay KT, Tan XH, Ong YT, et al. Assessing mentoring: A scoping review of mentoring assessment tools in internal medicine between 1990 and 2019. PloS one. 2020;15(5):e0232511.
10. Bousquet J, Schunemann HJ, Samolinski B, Demoly P, Baena-Cagnani CE, Bachert C, et al. Allergic Rhinitis and its Impact on Asthma (ARIA): achievements in 10 years and future needs. J Allergy Clin Immunol. 2012;130(5):1049-62.
11. Nur Haidah Ahmad Kamal LHET, Ruth Si Man Wong, Ryan Rui Song Ong, Ryan, Ern Wei Seow EKYL, Zheng Hui Mah, Min Chiam, Annelissa Mien Chew Chin, Jamie Xuelian Zhou, Gillian Li Gek Phua, Eng Koon Ong, Jin Wei Kwekc, Kiley Wei-Jen Loh and, Krishna LKR. Enhancing education in Palliative Medicine: the role of Systematic Scoping Reviews. Palliative Medicine & Care: Open Access. 2020;7(1):1-11.
12. Ryan Rui Song Ong REWS, Ruth Si Man Wong. A Systematic Scoping Review of Narrative Reviews in Palliative Medicine Education. Palliative Medicine & Care: Open Access. 2020;7(1):1-22.
13. Zheng Hui Mah RSMW, Ryan Ern Wei Seow Eleanor Kei Ying Loh, Nur Haidah, Ahmad Kamal RRSO, Lorraine Hui En Tan, Min Chiam, Annelissa Mien Chew Chin,, Jamie Xuelian Zhou GLGP, Yoke-Lim Soong, Jin Wei Kwek, and Lalit Kumar Radha Krishna. A Systematic Scoping Review of Systematic Reviews in Palliative Medicine Education. Palliative Medicine & Care: Open Access. 2020;7(1):1-12.
14. Pring R. The 'False Dualism' of Educational Research. Journal of Philosophy of Education. 2000;34(2):247-60.
15. Crotty M. The foundations of social research: Meaning and perspective in the research process: Sage; 1998 Oct 15.

For peer review only

16. Ford DW, Downey L, Engelberg R, Back AL, Curtis JR. Discussing religion and spirituality is an advanced communication skill: an exploratory structural equation model of physician trainee self-ratings. J Palliat Med. 2012;15(1):63-70.
17. Schick-Makaroff K, MacDonald M, Plummer M, Burgess J, Neander W. What synthesis methodology should I use? A review and analysis of approaches to research synthesis. AIMS public health. 2016;3(1):172.
18. Sandelowski M BJ. Handbook for synthesizing qualitative research. New York: Springer; 2007.
19. Pham MT, Rajic A, Greig JD, Sargeant JM, Papadopoulos A, McEwen SA. A scoping review of scoping reviews: advancing the approach and enhancing the consistency. Research synthesis methods. 2014;5(4):371-85.
20. Wen Jie Chua CWSC, Fion Qian Hui Lee, Eugene Yong Hian Koh, Ying Pin Toh, Stephen Mason, Lalit Kumar Radha Krishna. Structuring Mentoring in Medicine and Surgery. A Systematic Scoping Review of Mentoring Programs Between 2000 and 2019. Journal of Continuing Education in the Health Professions. 2020;40(3):158-68.
21. Yong Xiang Ng ZYKK, Hong Wei Yap, Kuang Teck Tay, Xiu Hui Tan, Yun Ting Ong, Lorraine Hui En Tan, Annelissa Mien Chew Chin, Ying Pin Toh, Sushma Shivananda, Scott Compton, Stephen Mason, Ravindran Kanesvaran, Lalit Krishna. Assessing mentoring: A scoping review of mentoring assessment tools in internal medicine between 1990 and 2019. . PLOS ONE. 2020;15(5):e0232511.
22. Peters MD, Godfrey CM, Khalil H, McInerney P, Parker D, Soares CB. Guidance for conducting systematic scoping reviews. Int J Evid Based Healthc. 2015;13(3):141-6.
23. Sambunjak D, Straus SE, Marusic A. A systematic review of qualitative research on the meaning and characteristics of mentoring in academic medicine. J Gen Intern Med. 2010;25(1):72-8.
24. Wong G, Greenhalgh T, Westhorp G, Buckingham J, Pawson R. RAMESES publication standards: meta-narrative reviews. BMC medicine. 2013;11(1):20.
25. Popay J, Roberts H, Sowden A, Petticrew M, Arai L, Rodgers M, et al. Guidance on the conduct of narrative synthesis in systematic reviews. A product from the ESRC methods programme Version. 2006;1:b92.
26. Braun V, Clarke V. Using thematic analysis in psychology. Qualitative research in psychology. 2006;3(2):77-101.
27. Cassol H, Pétré B, Degrange S, Martial C, Charland-Verville V, Lallier F, et al. Qualitative thematic analysis of the phenomenology of near-death experiences. PloS one. 2018;13(2):e0193001.
28. Hsieh H-F, Shannon SE. Three Approaches to Qualitative Content Analysis. Qualitative Health Research. 2005;15(9):1277-88.
29. Rider EA, Hinrichs MM, Lown BA. A model for communication skills assessment across the undergraduate curriculum. Med Teach. 2006;28(5):e127-34.
30. Goldie J. Review of ethics curricula in undergraduate medical education. Med Educ. 2000;34(2):108-19.
31. Duffy FD, Gordon GH, Whelan G, Cole-Kelly K, Frankel R, Buffone N, et al. Assessing competence in communication and interpersonal skills: the Kalamazoo II report. Acad Med. 2004;79(6):495-507.
32. Hong DZ, Goh JL, Ong ZY, Ting JJQ, Wong MK, Wu J, et al. Postgraduate ethics training programs: a systematic scoping review. BMC Medical Education. 2021;21(1):338.
33. Tay KT, Ng S, Hee JM, Chia EWY, Vythilingam D, Ong YT, et al. Assessing Professionalism in Medicine - A Scoping Review of Assessment Tools from 1990 to 2018. J Med Educ Curric Dev. 2020;7:2382120520955159.

For peer review only

34. David M, Davis M, Harden R, Howie P, Ker J, Pippard M. AMEE Medical Education Guide No. 24: Portfolios as a method of student assessment. Medical teacher. 2001;23:535-51.
35. Dixon-Woods M, Agarwal S, Young B, Jones D, Sutton A. Integrative Approaches to Qualitative and Quantitative Evidence. 2004.
36. Bryman A. Social Research Methods: Oxford University Press; 2016.
37. France EF, Uny I, Ring N, Turley RL, Maxwell M, Duncan EAS, et al. A methodological systematic review of meta-ethnography conduct to articulate the complex analytical phases. BMC Medical Research Methodology. 2019;19(1):35.
38. Noblit GW, Hare RD, Hare RD. Meta-ethnography: Synthesizing qualitative studies: sage; 1988.
39. Al Rabadi L, LeBlanc M, Bucy T, Ellis LM, Hershman DL, Meyskens FL, et al. Trends in Medical Aid in Dying in Oregon and Washington. JAMA Network Open. 2019;2(8):e198648-e.
40. Emanuel EJ, Onwuteaka-Philipsen BD, Urwin JW, Cohen J. Attitudes and practices of euthanasia and physician-assisted suicide in the United States, Canada, and Europe. JAMA: Journal of the American Medical Association. 2016;316(1):79-90.
41. Brinkman-Stoppelenburg A, Vergouwe Y, van der Heide A, Onwuteaka-Philipsen BD. Obligatory consultation of an independent physician on euthanasia requests in the Netherlands: What influences the SCEN physicians judgment of the legal requirements of due care? Health Policy. 2014;115(1):75-81.
42. Buiting H, van Delden J, Onwuteaka-Philpsen B, Rietjens J, Rurup M, van Tol D, et al. Reporting of euthanasia and physician-assisted suicide in the Netherlands: Descriptive study. BMC Medical Ethics Vol 10 2009, ArtID 18. 2009;10.
43. Konishi E, Davis A. The right-to-die and the duty-to-die: Perceptions of nurses in the West and in Japan. International Nursing Review. 2001;48(1):17-28.
44. Chapple A, Ziebland S, McPherson A, Herxheimer A. What People Close to Death Say About Euthanasia and Assisted Suicide: A Qualitative Study. Journal of Medical Ethics: Journal of the Institute of Medical Ethics. 2006;32(12):706-10.
45. Coyle N, Sculco L. Expressed desire for hastened death in seven patients living with advanced cancer: a phenomenologic inquiry. Oncol Nurs Forum. 2004;31(4):699-709.
46. Crespo I, Rodríguez-Prat A, Monforte-Royo C, Wilson KG, Porta-Sales J, Balaguer A. Health-related quality of life in patients with advanced cancer who express a wish to hasten death: A comparative study. Palliat Med. 2020;34(5):630-8.
47. Drum CE, White G, Taitano G, Horner-Johnson W. The Oregon Death with Dignity Act: Results of a literature review and naturalistic inquiry. Disability and Health Journal. 2010;3(1):3-15.
48. Ferrand E, Dreyfus JF, Chastrusse M, Ellien F, Lemaire F, Fischler M. Evolution of requests to hasten death among patients managed by palliative care teams in France: a multicentre cross-sectional survey (DemandE). Eur J Cancer. 2012;48(3):368-76.
49. Ramsey C. The right to die: beyond academia. Monash Bioeth Rev. 2016;34(1):70-87.
50. Reagan P, Hurst R, Cook L, Zylicz Z, Otlowski M, Veldink JH, et al. Physician-assisted death: Dying with dignity? Lancet Neurology. 2003;2(10):637-43.
51. Rodríguez-Prat A, Balaguer A, Booth A, Monforte-Royo C. Understanding patients' experiences of the wish to hasten death: an updated and expanded systematic review and meta-ethnography. BMJ Open. 2017;7(9):e016659.
52. Rodríguez-Prat A, van Leeuwen E. Assumptions and moral understanding of the wish to hasten death: a philosophical review of qualitative studies. Medicine, Health Care & Philosophy. 2018;21(1):63-75.

For peer review only

53. Wang L, Elliott M, Henson LJ, Gerena-Maldonado E, Strom S, Downing S, et al. Death with dignity in Washington and Oregon patients with amyotrophic lateral sclerosis. Neurology. 2016;86(16).
54. Fischer S, Huber CA, Furter M, Imhof L, Mahrer Imhof R, Schwarzenegger C, et al. Reasons why people in Switzerland seek assisted suicide: the view of patients and physicians. Swiss Med Wkly. 2009;139(23-24):333-8.
55. Freeman LM, Rose SL, Youngner SJ. Poverty: Not a Justification for Banning Physician-Assisted Death. Hastings Cent Rep. 2018;48(6):38-46.
56. Ganzini L, Goy ER, Dobscha SK. Why Oregon patients request assisted death: Family members' views. Journal of General Internal Medicine. 2008;23(2):154-7.
57. Goligher EC, Wesley Ely E, Sulmasy DP, Bakker J, Raphael J, Volandes AE, et al. Physician-Assisted Suicide and Euthanasia in the ICU: A Dialogue on Core Ethical Issues. Critical Care Medicine. 2017;45(2):149-55.
58. Hendry M, Pasterfield D, Lewis R, Carter B, Hodgson D, Wilkinson C. Why do we want the right to die? A systematic review of the international literature on the views of patients, carers and the public on assisted dying. Palliative Medicine. 2013;27(1):13-26.
59. Hiscox WE. Physician-assisted suicide in Oregon: The 'death with dignity' data. Medical Law International. 2007;8(3):197-220.
60. Kade WJ, Kade WJ. Death with dignity: a case study. Annals of Internal Medicine. 2000;132(6):504-6.
61. Kouwenhoven PS, van Thiel GJ, Raijmakers NJ, Rietjens JA, van der Heide A, van Delden JJ. Euthanasia or physician-assisted suicide? A survey from the Netherlands. Eur J Gen Pract. 2014;20(1):25-31.
62. Bailey F. "I am not afraid of dying. I just don't want to be there when it happens.". Medical Care. 2008;46(12):1195-7.
63. Barutta J, Vollmann J. Physician-assisted death with limited access to palliative care. Journal of Medical Ethics: Journal of the Institute of Medical Ethics. 2015;41(8):652-4.
64. Bolt EE, Pasman H, Deeg DJ, Onwuteaka-Philipsen BD. From advance euthanasia directive to euthanasia: Stable preference in older people? Journal of the American Geriatrics Society. 2016;64(8):1628-33.
65. Dees M, Vernooij-Dassen M, Dekkers W, van Weel C. Review unbearable suffering of patients with a request for euthanasia or physician-assisted suicide: An integrative review. Psycho-Oncology. 2010;19(4):339-52.
66. Duckett S. Pathos, death talk and palliative care in the assisted dying debate in Victoria, Australia. Mortality. 2020;25(2):151-66.
67. Eliott JA, Olver IN. Dying cancer patients talk about euthanasia. Social Science & Medicine. 2008;67(4):647-56.
68. McPherson T. My mum wanted assisted dying but we watched her die slowly and in pain. BMJ: British Medical Journal (Clinical Research Edition). 2012;344:e4007-e.
69. Mukhopadhyay S, Banerjee D. Physician assisted suicide in dementia: A critical review of global evidence and considerations from India. Asian J Psychiatr. 2021;64:102802.
70. Rowe Iii MJ. Beliefs. Chicago, Illinois: American Medical Association; 2015. p. 877-8.
71. Roy DJ. Palliative care and euthanasia: a continuing need to think again. J Palliat Care. 2002;18(1):3-5.
72. van Tol D, Rietjens J, van der Heide A. Judgment of unbearable suffering and willingness to grant a euthanasia request by Dutch general practitioners. Health Policy. 2010;97(2-3):166-72.

For peer review only

73. Wilson KG, Chochinov HM, McPherson CJ, Skirko MG, Allard P, Chary S, et al. Desire for euthanasia or physician-assisted suicide in palliative cancer care. Health Psychology. 2007;26(3):314-23.
74. Florijn BW. From Reciprocity to Autonomy in Physician-Assisted Death: An Ethical Analysis of the Dutch Supreme Court Ruling in the Albert Heringa Case. Am J Bioeth. 2022;22(2):51-8.
75. Ganzini L, Nelson HD, Schmidt TA, Kraemer DF, Delorit MA, Lee MA. Physicians' experiences with the Oregon Death with Dignity Act. N Engl J Med. 2000;342(8):557-63.
76. Kishore RR. Aruna Shanbaug and the right to die with dignity: the battle continues. Indian J Med Ethics. 2016;1(1):38-46.
77. Royo C, Villavicencio-Chávez C, Tomás-Sábado J, Mahtani V, Balaguer A. What Lies behind the Wish to Hasten Death? A Systematic Review and Meta-Ethnography from the Perspective of Patients. PloS one. 2012;7:e37117.
78. Bahník Š, Vranka MA, Trefná K. What makes euthanasia justifiable? The role of symptoms' characteristics and interindividual differences. Death Stud. 2021;45(3):226-37.
79. Hizo-Abes P, Siegel L, Schreier G. Exploring attitudes toward physician-assisted death in patients with life-limiting illnesses with varying experiences of palliative care: a pilot study. BMC Palliative Care. 2018;17(1):56.
80. Ohnsorge K, Gudat H, Rehmann-Sutter C. What a wish to die can mean: reasons, meanings and functions of wishes to die, reported from 30 qualitative case studies of terminally ill cancer patients in palliative care. BMC Palliat Care. 2014;13:38.
81. Ruijs CDM, van der Wal G, Kerkhof AJFM, Onwuteaka-Philipsen BD. Unbearable suffering and requests for euthanasia prospectively studied in end-of-life cancer patients in primary care. BMC Palliative Care. 2014;13(1):62.
82. Gómez-Vírseda C, Gastmans C. Euthanasia in persons with advanced dementia: a dignity-enhancing care approach. J Med Ethics. 2021.
83. Hale DB. A pretty pass: when is there a right to die? Common Law World Rev. 2003;32(1):1-14.
84. Cheung G, Sundram F. Who are the elderly who want to end their lives? [References]. Rational suicide in the elderly: Clinical, ethical, and sociocultural aspects. Cham, Switzerland: Springer International Publishing/Springer Nature; Switzerland; 2017. p. 113-28.
85. Dees MK, Vernooij-Dassen MJ, Dekkers WJ, Vissers KC, van Weel C. 'Unbearable suffering': A qualitative study on the perspectives of patients who request assistance in dying. Journal of Medical Ethics: Journal of the Institute of Medical Ethics. 2011;37(12):727-34.
86. Fontalis A, Prousali E, Kulkarni K. Euthanasia and assisted dying: what is the current position and what are the key arguments informing the debate? Journal of the Royal Society of Medicine. 2018;111(11):407-13.
87. Ganzini L, Dobscha SK, Heintz RT, Press N. Oregon physicians' perceptions of patients who request assisted suicide and their families. Journal of Palliative Medicine. 2003;6(3):381-90.
88. Karlsson M, Milberg A, Strang P. Dying cancer patients' own opinions on euthanasia: An expression of autonomy? A qualitative study. Palliative Medicine. 2012;26(1):34-42.
89. Gamester N, Van den Eynden B. The relationship between palliative care and legalized euthanasia in Belgium. Journal of Palliative Medicine. 2009;12(7):589-91.
90. Rodriquez E. The arguments for euthanasia and physician-assisted suicide: ethical reflection. Linacre Q. 2001;68(3):251-61.
91. Abrahm JL. Patient and family requests for hastened death. Hematology Am Soc Hematol Educ Program. 2008:475-80.

For peer review only

92. Gandsman A. Paradox of choice and the illusion of autonomy: The construction of ethical subjects in right-to-die activism. Death Studies. 2018;42(5):329-35.
93. Granda-Cameron C, Houldin A. Concept Analysis of Good Death in Terminally Ill Patients. American Journal of Hospice & Palliative Medicine. 2012;29(8):632-9.
94. Heintz AP. Euthanasia--or, death on request. J Psychosom Obstet Gynaecol. 2002;23(2):73-5.
95. Jiraphan A, Pitanupong J. General population-based study on preferences towards end-of-life care in Southern Thailand: a cross-sectional survey. BMC Palliative Care. 2022;21(1):36.
96. Dyer C. Two men plead with judges to let doctors end their life legally. BMJ: British Medical Journal (Clinical Research Edition). 2012;344:e4270-e.
97. Castelli Dransart DA, Lapierre S, Erlangsen A, Canetto SS, Heisel M, Draper B, et al. A systematic review of older adults' request for or attitude toward euthanasia or assisted-suicide. Aging & Mental Health. 2021;25(3):420-30.
98. Achille MA, Ogloff JR. Attitudes toward and desire for assisted suicide among persons with amyotrophic lateral sclerosis. Omega: Journal of Death and Dying. 2003;48(1):1-21.
99. Right to die--right or wrong? Lancet Neurol. 2003;2(10):583.
100. Friend ML. Physician-Assisted Suicide: Death With Dignity? Journal of Nursing Law. 2011;14(3/4):110-6.
101. Drury B. The doctors of mercy. Men's Health. 2006;21(8):180-7.
102. Wilson KG, Scott JF, Graham ID, Kozak JF, Chater S, Viola RA, et al. Attitudes of terminally ill patients toward euthanasia and physician-assisted suicide. Archives of Internal Medicine. 2000;160(16):2454-60.
103. Reid T. Reflections from a provider of medical assistance in dying. Canadian Family Physician. 2018;64(9):639-40.
104. Asai A, Ohnishi M, Nagata SK, Tanida N, Yamazaki Y. Doctors' and nurses' attitudes towards and experiences of voluntary euthanasia: survey of members of the Japanese Association of Palliative Medicine. J Med Ethics. 2001;27(5):324-30.
105. Eastaugh A. Choosing to die. Br J Gen Pract. 2012;62(595):96-7.
106. Salladay SA. Christian ethics. Death with dignity? Journal of Christian Nursing. 2010;27(3):232-.
107. Broom A. On euthanasia, resistance, and redemption: The moralities and politics of a hospice. Qualitative Health Research. 2012;22(2):226-37.
108. Mentzelopoulos SD, Haywood K, Cariou A, Mantzanas M, Bossaert L. Evolution of medical ethics in resuscitation and end of life. Trends in Anaesthesia and Critical Care. 2016;10:7-14.
109. Raus K, Sterckx S. Euthanasia for mental suffering. New directions in the ethics of assisted suicide and euthanasia. Cham, Switzerland: Springer International Publishing/Springer Nature; Switzerland; 2015. p. 79-96.
110. Robinson V, Clarke J, George R. Euthanasia: A caricature of care, not a cure for suffering. British Journal of Neuroscience Nursing. 2007;3(10):457-60.
111. Stephenson J. Assisted dying: a palliative care physician's view. Clin Med (Lond). 2006;6(4):374-7.
112. Finlay IG. Quality of life to the end. Commun Med. 2005;2(1):91-5.
113. Feigin S, Owens RG, Goodyear-Smith F. Helping a loved one die: the act of assisted dying in New Zealand. Mortality. 2019;24(1):95-110.
114. Dyer C. Dying woman loses her battle for assisted suicide. Bmj. 2002;324(7345):1055.

For peer review only

115. Fournier RR. Responses to life after death with dignity: the Oregon experience. Soc Work. 2000;45(5):467-8.
116. Comby M, Filbet M. The demand for euthanasia in palliative care units: A prospective study in seven units of the 'Rhone-Alpes' region. Palliative Medicine. 2005;19(8):587-93.
117. Simpson E. Harms to dignity, bioethics, and the scope of biolaw. Journal of Palliative Care. 2004;20(3):185-92.
118. Ganzini L, Back A. From the USA: Understanding requests for physician-assisted death. Palliative Medicine. 2003;17(2):113-4.
119. Gentzler J. What is a death with dignity? J Med Philos. 2003;28(4):461-87.
120. Robinson S, Kissane DW, Brooker J, Hempton C, Burney S. The Relationship Between Poor Quality of Life and Desire to Hasten Death: A Multiple Mediation Model Examining the Contributions of Depression, Demoralization, Loss of Control, and Low Self-worth. J Pain Symptom Manage. 2017;53(2):243-9.
121. Clark D. Cultural considerations in planning palliative and end of life care. Palliat Med. 2012;26(3):195-6.
122. Oczkowski SJW, Crawshaw DE, Austin P, Versluis D, Kalles-Chan G, Kekewich M, et al. How can we improve the experiences of patients and families who request medical assistance in dying? A multi-centre qualitative study. BMC Palliative Care. 2021;20(1):185.
123. Ong RSR, Wong RSM, Chee RCH, Quek CWN, Burla N, Loh CYL, et al. A systematic scoping review moral distress amongst medical students. BMC Medical Education. 2022;22(1):466.
124. Chua KZY, Quah ELY, Lim YX, Goh CK, Lim J, Wan DWJ, et al. A systematic scoping review on patients' perceptions of dignity. BMC Palliative Care. 2022;21(1):118.
125. Ho CY, Lim NA, Ong YT, Lee ASI, Chiam M, Gek GPL, et al. The impact of death and dying on the personhood of senior nurses at the National Cancer Centre Singapore (NCCS): a qualitative study. BMC Palliat Care. 2022;21(1):83.
126. Chiam M, Ho CY, Quah E, Chua KZY, Ng CWH, Lim EG, et al. Changing self-concept in the time of COVID-19: a close look at physician reflections on social media. Philos Ethics Humanit Med. 2022;17(1):1.
127. Chan NPX, Chia JL, Ho CY, Ngiam LXL, Kuek JTY, Ahmad Kamal NHB, et al. Extending the Ring Theory of Personhood to the Care of Dying Patients in Intensive Care Units. Asian Bioeth Rev. 2022;14(1):71-86.
128. Huang H, Toh RQE, Chiang CLL, Thenpandiyan AA, Vig PS, Lee RWL, et al. Impact of Dying Neonates on Doctors' and Nurses' Personhood: A Systematic Scoping Review. J Pain Symptom Manage. 2022;63(1):e59-e74.
129. Vig PS, Lim JY, Lee RWL, Huang H, Tan XH, Lim WQ, et al. Parental bereavement – impact of death of neonates and children under 12 years on personhood of parents: a systematic scoping review. BMC Palliative Care. 2021;20(1):136.
130. Ho CY, Kow CS, Chia CHJ, Low JY, Lai YHM, Lauw S-K, et al. The impact of death and dying on the personhood of medical students: a systematic scoping review. BMC Medical Education. 2020;20(1):516.
131. Haig A, Dozier M. BEME Guide no 3: systematic searching for evidence in medical education--Part 1: Sources of information. Medical teacher. 2003;25(4):352-63.
132. Gordon M, Gibbs T. STORIES statement: publication standards for healthcare education evidence synthesis. BMC medicine. 2014;12(1):143.

For peer review only

Appendix B. Search strategies
PubMed
Filters Applied: English, from 2000/1/1 – 2020/12/31

#1	("physicians"[MeSH Terms] OR "students, medical"[MeSH Terms] OR "physician*"[Title/Abstract] OR "doctor*"[Title/Abstract] OR "medical student*"[Title/Abstract] OR "medical trainee*"[Title/Abstract] OR "physician trainee"[Title/Abstract]) AND ((2000/1/1:2020/12/31[pdat]) AND (english[Filter]))	395,521
#2	("educational status"[MeSH Terms] OR "education"[MeSH Terms] OR "learning"[MeSH Terms] OR "educat*"[Title/Abstract] OR "learn*"[Title/Abstract] OR "teach*"[Title/Abstract] OR "mentor*"[Title/Abstract]) AND ((2000/1/1:2020/12/31[pdat]) AND (english[Filter]))	1,265,980
#3	("professional identity"[Title/Abstract] OR "portfolio*"[Title/Abstract] OR "appraisal*"[Title/Abstract] OR "assessment*"[Title/Abstract]) AND ((2000/1/1:2020/12/31[pdat]) AND (english[Filter]))	912,728
#4	("communication"[MeSH Terms] OR "communicat*"[Title/Abstract] OR "interpersonal skill*"[Title/Abstract]) AND ((2000/1/1:2020/12/31[pdat]) AND (english[Filter]))	418,990
#5	("ethics"[MeSH Terms] OR "morals"[MeSH Terms] OR "social values"[MeSH Terms] OR "ethic*"[Title/Abstract] OR "moral*"[Title/Abstract] OR "value*"[Title/Abstract] OR "Beliefs"[Title/Abstract] OR "Principles"[Title/Abstract] OR "ideals"[Title/Abstract]) AND ((2000/1/1:2020/12/31[pdat]) AND (english[Filter]))	1,628,161
#6	("Professionalism"[MeSH Terms] OR "Professionalism"[Title/Abstract] OR "Professional"[Title/Abstract]) AND ((2000/1/1:2020/12/31[pdat]) AND (english[Filter]))	130,203
#1 AND #2 AND #3 AND (#4 OR #5 OR #6)		7,617

For peer review only

Embase

#1	('physician'/exp OR 'medical student'/exp OR 'physician':ti,ab OR 'doctor':ti,ab OR 'medical student':ti,ab) AND [2000-2020]/py AND [embase]/lim NOT ([embase]/lim AND [medline]/lim) AND [english]/lim	482,177
#2	('education'/exp OR 'educat*':ti,ab OR 'learn*':ti,ab OR 'teach*':ti,ab OR 'mentor*':ti,ab) AND [2000-2020]/py AND [embase]/lim NOT ([embase]/lim AND [medline]/lim) AND [english]/lim	779,513
#3	('professional identity':ti,ab OR 'portfolio*':ti,ab OR 'appraisal*':ti,ab OR 'holistic assessment':ti,ab OR 'personal assessment':ti,ab OR 'feedback':ti,ab OR 'continuous assessment':ti,ab) AND [2000-2020]/py AND [embase]/lim NOT ([embase]/lim AND [medline]/lim) AND [english]/lim	73,761
#4	('interpersonal communication'/exp OR 'communication':ti,ab OR 'interpersonal skill':ti,ab) AND [2000-2020]/py AND [embase]/lim NOT ([embase]/lim AND [medline]/lim) AND [english]/lim	254,318
#5	('ethics'/exp OR 'morality'/exp OR 'social value'/exp OR 'ethic*':ti,ab OR 'moral*':ti,ab OR 'value*':ti,ab OR 'beliefs':ti,ab OR 'principles':ti,ab OR 'ideals':ti,ab) AND [2000-2020]/py AND [embase]/lim NOT ([embase]/lim AND [medline]/lim) AND [english]/lim	955,250
#6	('professionalism'/exp OR 'professionalism':ti,ab) AND [2000-2020]/py AND [embase]/lim NOT ([embase]/lim AND [medline]/lim) AND [english]/lim	3,740
#1 AND #2 AND #3 AND (#4 OR #5 OR #6)		3,751

For peer review only

PsycINFO

1	exp physicians/ or exp medical students/ or (physician* or doctor* or medical student* or physician trainee* or medical trainee*).ab,ti.	140,688
2	exp education/ or exp learning/ or (educat* or learn* or teach* or mentor*).ab,ti.	1,324,893
3	('professional identity' or portfolio* or appraisal* or assessment*).ab,ti.	410,796
4	1 and 2 and 3	6,780
5	exp communication/ or (communicat* or interpersonal skill*).ab,ti.	502,521
6	exp ethics/ or exp morality/ or exp social values/ or (ethic* or moral* or value* or beliefs or principles or ideals).ab,ti.	647,241
7	exp professionalism/ or (professionalism or professional).ab,ti.	130,203
8	5 or 6 or 7	1191406
9	4 and 8	3082
10	limit 9 to (english language and yr="2000 - 2020")	2507

For peer review only

ERIC
Limits applied: English Language, 01/01/2000-31/12/2020

S1	MAINSUBJECT.EXACT.EXPLODE("Physicians") OR MAINSUBJECT.EXACT.EXPLODE("Medical Students") OR title(physician* OR doctor* OR medical student* OR physician trainee* OR medical trainee*) AND abstract(physician* OR doctor* OR medical student* OR physician trainee* OR medical trainee*)	6,171
#2	(MAINSUBJECT.EXACT.EXPLODE("Informal Education") OR MAINSUBJECT.EXACT.EXPLODE("Adult Education") OR MAINSUBJECT.EXACT.EXPLODE("Medical Education") OR MAINSUBJECT.EXACT.EXPLODE("Individualized Education Programs")) OR title(educat* OR learn* OR teach* OR mentor*) OR abstract(educat* OR learn* OR teach* OR mentor*)	536,465
#3	(MAINSUBJECT.EXACT.EXPLODE("Portfolios (Background Materials)") OR MAINSUBJECT.EXACT.EXPLODE("Professional Identity") OR MAINSUBJECT.EXACT.EXPLODE("Portfolio Assessment")) OR abstract('professional identity' OR portfolio* OR appraisal* OR assessment*) OR title('professional identity' OR portfolio* OR appraisal* OR assessment*)	85,052
#4	(MAINSUBJECT.EXACT.EXPLODE("Communication Strategies") OR MAINSUBJECT.EXACT.EXPLODE("Communication (Thought Transfer)") OR MAINSUBJECT.EXACT.EXPLODE("Communication Skills")) OR abstract(communicat* OR 'interpersonal skill*') OR title(communicat* OR 'interpersonal skill*')	90,574
#5	(MAINSUBJECT.EXACT.EXPLODE("Social Values") OR MAINSUBJECT.EXACT.EXPLODE("Ethics")) OR abstract(ethic* OR moral* OR value* OR beliefs OR principles OR ideals) OR title(ethic* OR moral* OR value* OR beliefs OR principles OR ideals)	123,249
#6	MAINSUBJECT.EXACT.EXPLODE("Professionalism") OR abstract(professionalism OR professional) OR title(professionalism OR professional)	77,628
#1 AND #2 AND #3 AND (#4 OR #5 OR #6)		440

Google Scholar

(physicians OR medical students OR doctors) AND (education* OR learn* OR teach*) AND ("professional identity" OR portfolio*) AND ((communication* OR interpersonal skill*) OR (ethics OR values) OR professionalism)

For peer review only

Appendices

Appendix B. Tabulated Summaries.

No.	Title	Author/year	MERSQI	COREQ	Reason for inclusion			Undergraduate (UG)/Postgraduate (PG)	Methodology	Purpose of study	Key findings
					Communication	Ethics	Professionalism				
1	The CanMEDS resume: a useful educational portfolio tool for diagnostic radiology residents	Finlay, K. Probyn, L. Ho, S. 2012			✓		✓	PG	We developed a comprehensive resident CanMEDS resume for ongoing documentation of resident participation, education, and developing competencies in CanMEDS Roles for diagnostic radiology. This resume or portfolio summary is structured to high- light all 7 CanMEDS Roles. The resident is responsible for ongoing updates and submits this as part of his or her annual evaluation.	The objective of this article is to discuss the utility of this tool for inclusion in the educational portfolio for radiology residents.	The document increases awareness of the CanMEDS Roles and documents resident professional development, while also promoting reflection on the impor- tance of these Roles for radiology and future practice. The added advantage for program directors and assistants is up- to-date documentation of resident activities, which serves as a highly useful tool, particularly for program accreditation purposes.
2	A pilot evaluation of portfolios for quality attestation of clinical ethics consultants	Fins, Joseph J Kodish, Eric Cohn, Felicia Danis, Marion Derse, Arthur R Dubler, Nancy Neveloff Goulden, Barbara Kuczewski, Mark Mercer, Mary Beth Pearlman, Robert A 2016	7.5	14		✓		PG	The QAPTF committee decided on the key components of the portfolio, their respective weightages and scoring quality of each element. ASBH issued an open call to individuals practicing CEC, publicized on the ASBH website, for let- ters of intent (LOI) to submit portfolios for the piloting of this first step in a quality attestation process. The QAPTF wanted to ensure a	We describe the results of a pilot project utilizing portfolios as an evaluation tool. The goals of this quality attestation pilot project were to assess the feasibility of a process to evaluate whether CE consultants are competent to perform ethics consultations as a lead or solo consultant and to develop quality metrics for this assessment process.	We found that this approach is feasible and resulted in a reasonably wide distribution of scores among the 23 submitted portfolios that we evaluated. We discuss limitations and implications of these results, and suggest that this is a significant step on the pathway to an eventual certification process for clinical ethics consultants.

For peer review only

No.	Title	Author/year	MERSQI	COREQ	Reason for inclusion			Undergraduate (UG)/Postgraduate (PG)	Methodology	Purpose of study	Key findings
					Communication	Ethics	Professionalism				
									representative distribution of candidates for the first portfolio review and adopted a method for select- ing portfolios that was intentionally designed to minimize bias.Candidates needed to have performed CEC, have at least a master's degree in a relevant discipline, and have sufficient CEC experience to be able to provide all ele- ments of the portfolio. 32 out of 82 individuals were invited to submit portfo- lios. After candidates submitted their portfolios, they were surveyed to obtain feedback on their portfolio develop- ment and submission experience. QAPTF members were also asked about their experi- ence and perceptions of whether the portfolio elements and approach to scoring functioned effectively in identify- ing candidates who should or should not proceed with the next step of		

For peer review only

No.	Title	Author/year	MERSQI	COREQ	Reason for inclusion			Undergraduate (UG)/Postgraduate (PG)	Methodology	Purpose of study	Key findings
					Communication	Ethics	Professionalism				
									quality attestation. They also were asked about ways to improve future attestation activities. This feedback is reflected in the limitations and discussion sections that follow.		
3	Portfolios and 'the competent clinician': past, present and future assessment	Gavriel, J. 2016	-	-			✓	PG	NA	To describe the evolution of portfolios, including its advantages, challenges and the future of portfolios	Portfolios have been a part of medical education since the early 1990s and many educators use them frequently, even habitually. Portfolios are primarily based on a constructivist approach to learning and there are also strong links with adult learning principles.Competence based education, once under fire for being too simplistic, now forms a significant part of medical education including the development of portfolios.<br><br>In the med- ical education arena there are some advantages that are particularly relevant: triangulation amongst those working alongside learners, developing wider professional skills and demonstrating the potential for lifelong learning and excellence.<br><br>For some learners and educators the portfolio is a burden and a frustration: the volume of work, a hoop jumping approach and a subjective nature all providing challenges [8] that are at least partially a result of some key complex- ities of the portfolio assessment.

No.	Title	Author/year	MERSQI	COREQ	Reason for inclusion			Undergraduate (UG)/Postgraduate (PG)	Methodology	Purpose of study	Key findings
					Communication	Ethics	Professionalism				
											Portfolios are evolving assessments; they are subject to continuing developments but the ongoing challenge for portfolio designers is the balance between reliability and validity on one hand and tick-boxing on the other.
4	Diffusion of an e-portfolio to assist in the self-directed learning of physicians: An exploratory study	Goliath, Cheryl Lynn<br>2010	13	21	✓			PG	A single case study comprised of nine physicians was used to examine the factors involved when the diffusion of an e-portfolio was introduced. The participants were studied using interviews, the Stages of Concern (SoCQ) and the Self-Directed Learning Readiness Scale (SDLRS) questionnaires, four-week trial period of I-Webfolio and field notes based upon observations during the study.	This study examined the impact of implementing an e-portfolio system within an Internal Medicine physician practice as a means of developing a process of life-long learning and professional development for the participants.	The results were analyzed to identify patterns or themes that appeared. One such pattern emerged, in that; all entries in the e-portfolio were almost exclusively in the Medical Knowledge component. A theme that became apparent was that none of the physicians in the study have developed a self-directed learning plan. The results of this study suggested that adoption of technology is multifactoral and there was no consistent individual profile that predicted optimal adoption of the innovation; however, increased scores on the SDLRS would indicate that the diffusion of I-Webfolio was effective in improving the self-directed learning of the participants.
5	The attributes of a good medical trainee: how to build your portfolio	Joseph, S. R.<br>Sikkel, M. B.<br>2009	-	-	✓			PG	-	Review of the attributes of a good medical trainee based on the essential physician competencies, and how then to build a good portfolio	A comprehensive and contemporaneously completed procedural, operative, teaching and reflective practice logs should be included in the portfolio, and should be commenced as soon as possible. Compose your portfolio around the seven key roles of the Canadian Medical Education Directives for Specialists model, and be innovative when

For peer review only

No.	Title	Author/year	MERSQI	COREQ	Reason for inclusion			Undergraduate (UG)/Postgraduate (PG)	Methodology	Purpose of study	Key findings
					Communication	Ethics	Professionalism				
											filling each section with concrete examples. Use reflective practice of clinical cases also as further evidence.
6	Study skills course in medical education for postgraduate residents	Bhattarai, M. D. 2007	-	-	✓			PG	-	The learners have to take active parts in the teaching learning activities. To make them aware and to help them develop the skills required, the need of the study skills course in medical education early in the part of their training has been realized for the postgraduate residents	The important areas of the study skills course focusing in the requirement of clinical components of the postgraduate residents are 1.) Interpersonal and communications skills, 2.) Teaching, learning and presentation skills, 3.) Language, reading and computer use, 4.) Evidence based medicine and diagnosis and management, 5). Assessment principles and strategies, 6). Time management strategies to get the best out of the training, 7). Reflection, portfolio and self-directed lifelong learning, and 8). Follow-up presentation. The methodologies that could be used in such study skills course are interactive lectures, brainstorming, presentations by the trainees, demonstration to and by the trainees, small group discussion, group work and presentation, group and individual feedback, practice sessions, role play, short relevant video movies, video recording of the trainees and viewing with feedback. With their already tight training schedule and posting and other similar other mandatory courses required for the postgraduate residents, much time cannot be allocated for the study skills course in medical education alone. Similar study skills course in medical education

For peer review only

No.	Title	Author/year	MERSQI	COREQ	Reason for inclusion			Undergraduate (UG)/Postgraduate (PG)	Methodology	Purpose of study	Key findings
					Communication	Ethics	Professionalism				
											may need to be arranged for the undergraduate medical students as well.
7	Fellows' perceptions of a mandatory reflective electronic portfolio in a geriatric medicine fellowship program	Ruiz, Jorge G. Qadri, Syeda S. Karides, Marina Castillo, Carmen Milanez, Marcos Roos, Bernard A. 2009	9	15	✓		✓	PG	We conducted surveys and focus groups followed by quantitative and qualitative data analysis	Electronic portfolios (ePortfolios) can be useful for evaluating and documenting mastery of competencies. We investigated geriatric medicine fellows' perceptions of an ePortfolio	Our study revealed that fellows considered the ePortfolio acceptable and accessible. Fellows reflected on aspects relevant to postgraduate training and future practice and documented adherence to competencies. However, fellows expressed resentment about the self-reflection component, which interfered with the overall acceptance of the ePortfolio. Although ePortfolios can be powerful and reflective tools for competency assessment, care must be taken in their implementation.
8	Portfolios for assessment and learning: Guide Supplement 45.1 - Viewpoint	Burch, Vanessa 2011	-	-		✓		PG	NA	NA	The critical question to ask then is Should poorly resourced countries not find ways of adapting this valuable learning and assessment strategy to suit local realities? (Spandel 1997). Clearly, the ‘Rolls Royce’ version of portfolio implementation and assessment is beyond the reach of developing countries but a less resource-intensive strategy could be a feasible option. It was with this goal in mind that the University of Cape Town (UCT) in South Africa embarked on the task of adapting portfolios for use in less well-resourced settings (Burch & Seggie 2008). Medical students in a 6-year programme are introduced to portfolio-based learning and assessment in their third year of training and each subsequent year until graduation. Currently, clinical students write up,

For peer review only

No.	Title	Author/year	MERSQI	COREQ	Reason for inclusion			Undergraduate (UG)/Postgraduate (PG)	Methodology	Purpose of study	Key findings
					Communication	Ethics	Professionalism				
											and manage under supervision, between 18 and 32 cases in 6- to 10-week medicine clerkships These patients are presented to a specialist in training on admission and the attending consultant on the post-intake ward the following morning. This provides ample opportunity for students to look back on the action, reflect on their performance and receive feedback and mentoring from senior clinicians directly in the workplace setting. In this way, consultants and specialists in training act as mentors helping students identify alternative strategies and set goals to stimulate further learning. These distinct phases of the workplace-based mentoring process closely reflect the phases of spiral professional development described by Korthagen et al. (2001). The importance of this approach is that mentors provide ongoing guidance for students during the course of performing their routine clinical duties limiting the need for additional regular meetings between students and mentors. Students who are struggling are free to consult the course convener for assistance, but an introductory lecture clearly outlining the purpose, format and examination of portfolios, as well as a set of guidelines for completing portfolio tasks has significantly limited the need for further advice. In addition to writing up a series of patient case studies, students also

For peer review only

No.	Title	Author/year	MERSQI	COREQ	Reason for inclusion			Undergraduate (UG)/Postgraduate (PG)	Methodology	Purpose of study	Key findings
					Communication	Ethics	Professionalism				
											identify at least one clinical learning need based on each case discussion; these ‘question and answer’ tasks are also included in the portfolio. This provides a good opportunity for needs-driven, focused, self-directed learning. Students also complete a range of other written tasks using the index clinical cases in their portfolios. These case-based tasks (Harden et al. 2000) include written reflections on ethical issues relevant to selected patients, a bio-psycho-social analysis (mini-ethnography) (Kleinman & Benson 2006) of selected patients and the use of case material to explore the principles of Primary Health Care (Vivian et al. 2010) as they relate to patients in the portfolio. Finally, students also write up detailed treatment plans for a list of 32 common conditions as reflected by the cases in the portfolio. These tasks are all designed to promote self-directed, patient-centred learning.<br>At UCT it was recognised that marking of this volume of portfolio material was beyond the human resources capacity at departmental level and so a strategic decision was made to focus on an assessment strategy based on a series of structured interviews, focusing on different aspects of a sample of portfolio cases. Students rotate through four stations of 15 min each (OSCE style) at which single-examiner structured interviews are conducted based

For peer review only

No.	Title	Author/year	MERSQI	COREQ	Reason for inclusion			Undergraduate (UG)/Postgraduate (PG)	Methodology	Purpose of study	Key findings
					Communication	Ethics	Professionalism				
											on patient cases randomly selected from the portfolio. The case-based discussions focus on clinical reasoning, case management plans, bioethics, psychosocial aspects of illness and the principles of Primary Health Care. This approach assesses student performance on a 'biopsy' of at least 8–10 cases contained in the portfolio. Each case discussion is scored using a nine-point rubric (1–3 poor, 4–6 adequate, 7–9 good) and scores are totalled across all cases discussed to achieve an aggregate score for the examination. The key reasons for adopting this assessment strategy at UCT included limited human resources but, more importantly, the observation that English second language speakers, more than 50% of university admissions nationally (Council on Higher Education 2009), are better able to express themselves in oral rather than written form. Indeed, it has been asked whether portfolios provide educators with real insight into practitioners' clinical ability or simply show that they are good at writing about what they do? (Karlowicz 2000). Having developed the strategy, the key question is whether any other university in sub-Saharan Africa has successfully adopted it. To date, two other South African universities have adopted and adapted the approach to suit local needs and two other African universities have

For peer review only

No.	Title	Author/year	MERSQI	COREQ	Reason for inclusion			Undergraduate (UG)/Postgraduate (PG)	Methodology	Purpose of study	Key findings
					Communication	Ethics	Professionalism				
											successfully adopted the strategy at Makerere University (Mubuuke et al. 2010) and Kampala International University (Ezeala et al. 2010) both in Uganda. Clearly, the data are limited but it does suggest that portfolio-based learning and assessment are within reach of countries attempting to improve the quality of health sciences education using available resources. This, I believe, is one of the most important issues to be addressed in the twenty-first century – the adaptation and implementation of better teaching and assessment strategies in developing countries so as to keep abreast of educational advances developed world countries enjoy.
9	AMEE Guide No. 25: The assessment of learning outcomes for the competent and reflective physician	Shumway, J. M. Harden, R. M. 2003	-	-	✓	✓	✓	PG	-	To emphasize on the 2 important features of contemporary medical education: first is an emphasis on assessment as a tool to ensure quality in training programmes, to motivate students and to direct what they learn. The second is a move to outcome-based education where the learning outcomes are defined and decisions about the curriculum are based on these.	Portfolio assessments have a positive impact on the learning environment because they document what the learner has done and ask the learner to reflect on what she/he has accomplished. They ask the learner to put together large amounts of disparate information into a whole that tells a story about the learner's accomplishments. Portfolios are a valuable instru- ment for inclusion in the examiner's toolkit, if for no other reason than that they assess learning outcomes such as critical thinking and self-assessment, which are not easily assessed using other instruments. They also provide a record of the

For peer review only

No.	Title	Author/year	MERSQI	COREQ	Reason for inclusion			Undergraduate (UG)/Postgraduate (PG)	Methodology	Purpose of study	Key findings
					Communication	Ethics	Professionalism				
											student's performance over a period of time and are not just a snapshot at one specific point in time. The reliability of portfolios is at least in part due to the ability of raters to agree on standards and criteria for the content and assessment of portfolios.
10	A comprehensive collaborative patient safety residency curriculum to address the ACGME core competencies	Singh, R.<br>Naughton, B.<br>Taylor, J. S.<br>Koenigsberg, M. R.<br>Anderson, D. R.<br>McCausland, L. L.<br>Wahler, R. G.<br>Robinson, A.<br>Singh, G.<br><br>2005	-	-	✓			PG	-	To design and implement a new patient safety curriculum in collaboration with the Schools of Nursing and Pharmacy, in such a way as to address all 6 competencies.	Porfolios is used as one of the multiple assessment approaches in the patient safety programme. However, no evaluation made on this assessment mode.
11	ECLIPPx: an innovative model for reflective portfolios in life-long learning	Cheung, C. R.<br><br>2011	-	-			✓	PG	-	For healthcare professionals, the educational portfolio is the most widely used component of lifelong learning – a vital aspect of modern medical practice. When used effectively, portfolios provide evidence of continuous learning and promote reflective practice. But traditional portfolio models are in danger of becoming outmoded, in the face of changing expectations of health- care provider competences today.<br><br>Portfolios in health care have generally focused on competencies in clinical skills. How- ever, many other domains of professional	A new model for categorising the health care port- folios of professionals is proposed. The ECLIPPx model is based on personal practice, and divides the evidence of ongoing professional learning into four categories: educational development; clinical practice; leadership, innovation and professionalism; and personal experience.<br><br>The ECLIPPx model offers a new approach for personal reflection and longitudinal learn- ing, one that gives flexibility to the user whilst simultaneously encompassing the many relatively new areas of competence and expertise that are now required of a modern doctor.

For peer review only

No.	Title	Author/year	MERSQI	COREQ	Reason for inclusion			Undergraduate (UG)/Postgraduate (PG)	Methodology	Purpose of study	Key findings
					Communication	Ethics	Professionalism				
										development, such as professionalism and leadership skills, are increasingly important for doctors and health care professionals, and must be addressed in amassing evidence for training and revalidation. There is a need for modern health care learning portfolios to reflect this sea change.	
12	Development of a web-based, specialty specific portfolio	Clay, A. S., Petrusa, E., Harker, M. and Andolsek, K.<br><br>2007	-	-	✓	✓	✓	PG	Three different disciplines (anesthesiology, surgery and medicine) worked together to create a critical care medicine portfolio. We began by reviewing the curriculum requirements for critical care medicine and organise<br><br>d these requirements into the six ACGME core competencies. We then developed learner led exercises in each core competency that were specific to critical care. Each exercise includes assessment of resident knowledge and application, an evaluation of the exercise, a learner self-	This article illustrates the creation of a specialty specific portfolio that can be used by several different residency programs to document resident competence during a given rotation	Creation of specialty specific portfolio reduces redundancy between disciplines, allows for increased time to be spent on the development of exercises specific to rotation objectives, and aids program directors in the collection of portfolio entries for each resident over the course of a residency.<br><br>We describe the creation of a multidisciplinary learning portfolio that can be used by different training programs within one institution to assess trainee performance in each respective area of expertise. The strengths of the specialty specific portfolio include the ability to be specific to the learning objectives of that rotation/specialty and uniform in content across multiple disciplines within that specialty. Commitment to the portfolio from multiple disciplines allows for careful refining of the portfolio and reduction of duplicative efforts. These specialty specific portfolios could potentially be improved upon if professional

For peer review only

No.	Title	Author/year	MERSQI	COREQ	Reason for inclusion			Undergraduate (UG)/Postgraduate (PG)	Methodology	Purpose of study	Key findings
					Communication	Ethics	Professionalism				
									assessment of skill, and a review of performance by a faculty member. Portfolio entries are highlighted in a multi-disciplinary weekly conference and posted on a critical care web site at our University.		organizations were committed to their adaptation, perhaps as a component of maintenance of certification. The portfolio entries as we have described are also useful for prompting frequent self reflection/evaluation and for providing trainees with feedback on their self-perceptions. Self assessment, a skill which is not often taught or evaluated in medical education, is notoriously inaccurate (Gordon 1991). However, self assessment may be improved with deliberate practice, and frequent self assessment with feedback on that self assessment (Gordon 1992; Norman & Schmidt 1992; Ericsson 2004). If other specialties were to create similar portfolios, the logistics of creating a portfolio for each resident would be simplified: the portfolio could be built a rotation at a time, meeting the objectives of that rotation monthly, and meeting the ACGME requirements over the course of a residency. Furthermore, by simply requiring a certain number entries from each core competency by the end of each year or the end of a residency, the learner is given the discretion to choose those exercise which he/she feels is most relevant given the cases he/she has encountered.
13	A review of portfolio use in residency programs and	Colbert, C. Y., Ownby, A. R. and Butler, P. M.	-	-			✓	PG	Two searches of PubMed, OVID, JSTOR, SCOPUS, and	Portfolios, often described as collections of evi-dence, are discussed	There is wide varia- tion in how portfolios are utilized within U.S. residency programs. The challenge for

For peer review only

No.	Title	Author/year	MERSQI	COREQ	Reason for inclusion			Undergraduate (UG)/Postgraduate (PG)	Methodology	Purpose of study	Key findings
					Communication	Ethics	Professionalism				
	considerations before implementation	2008							FirstSearch Wilson Select were conducted between October 2006 and April 2007 to identify studies and articles related to portfolio usage. Thirty-nine articles met criteria and were reviewed.	as a means of teaching or assessing the Ac- creditation Council for Graduate Medical Education competencies. Yet, it is unclear how many residency programs utilize portfolios. The purpose of this article is to (a) review the literature on portfolio use in graduate medical education; (b) examine efficacy of portfolio use, based upon studies in the field; and (c) offer a discussion of con- siderations for implementing portfolios.	graduate medical education is to create consensus on the definition and purpose of portfolios, such that best practices in portfolio implementation and assessment can be achieved.<br><br>The case for utilizing portfolios in residency education is compelling. Portfolios can offer a diverse palette of learners' achievements and experiences. When assessing competence from a holistic standpoint, the range of behaviors captured in a portfolio may render it superior to more traditional assessment practices. Self-awareness and self-directed learning, thought to be critical to the development of competent physicians, can be enhanced through the act of portfolio development,19 as port- folios can force one to reflect upon strengths, weaknesses, and opportunities for growth.<br><br>Before implementing a portfolio system in a residency pro- gram, several issues need to be considered. Learners and faculty should be trained on the portfolio process including the purpose, structure, and content of portfolios. Mentoring may be required when it comes to content assembly and interpretation. If portfolios are to be used for summative assessment pur- poses, an evaluation rubric—a scoring template—will need to be

No.	Title	Author/year	MERSQI	COREQ	Reason for inclusion			Undergraduate (UG)/Postgraduate (PG)	Methodology	Purpose of study	Key findings
					Communication	Ethics	Professionalism				
											created.<br><br>Last, the promise offered by portfolios is tempered by diffi-culties associated with their use. The lack of a standard defini- tion across the field and the dearth of outcome studies to assess efficacy are pressing concerns. The challenge now is to create consensus on the definition and purpose of portfolios within res- idency programs, such that collaboration within the field may begin to offer best practices in the areas of implementation and assessment.
14	How we teach ethics and communication during a Canadian neonatal perinatal medicine residency: an interactive experience	Daboval, T., Moore, G. P. and Ferretti, E.<br><br>2013	6.5		✓	✓	✓	PG	A revised ethics program implemented in 2009 identified competencies that should be demonstrated at the end of the Neonatal-Perinatal Medicine (NPM) residency. Several seminars were refined while new workshops, problem-based learning in ethics, and a personal portfolio were added.	This article describes the process by which a well-delineated, interactive program to teach ethical reasoning and skillful communication with parents was implemented at the University of Ottawa, Canada.	All teaching strategies were well received based on the average level of satisfaction (5.8 out of 7, SD 0.4). We are now moving forward by formally assessing our program including the impact on knowledge acquisition and behavior.<br><br>A dedicated, interactive competency-based neonatal ethics teaching program is vital to support NPM trainees in learning how to integrate ethical thinking with competencies in communication.<br><br>A dedicated, interactive, competency-based neonatal ethics teaching program with well-identified expectations and eval-uation is vital so that all graduating NPM trainees will not only have knowledge of key ethical precepts and ethical reasoning, but will also have learned how to integrate ethical thinking when they communicate and interact

For peer review only

No.	Title	Author/year	MERSQI	COREQ	Reason for inclusion			Undergraduate (UG)/Postgraduate (PG)	Methodology	Purpose of study	Key findings
					Communication	Ethics	Professionalism				
											professionally with parents during ethically and emotionally charged clinical situations. In this manner, our teaching is supporting a paradigm shift in neonatology from a paternalistic to a shared decision-making model. To advance our methods of education in this area, formal assessments of such teaching programs must occur.
15	Portfolio as a tool to stimulate teachers' reflections	Tigelaar, D. E.<br>Dolmans, D. H.<br>de Grave, W. S.<br>Wolfhagen, I. H.<br>van der Vleuten, C. P.<br><br>2006	-	15	✓			PG	In this study, all the written portfolio assignments were approached as reflections on teacher functioning. In order to gain insight into the portfolio process, we collected the teachers' final portfolio assignments as well as data about the process of portfolio construction. In this way, we obtained portfolio data comprising written portfolio assignments and final reflective portfolio assignments. Firstly, the teachers were asked to describe the course of their teaching career. They were also asked to describe critical incidents in their development as a teacher, set learning goals,	Portfolios are increasingly being used to stimulate teachers' reflections. Frameworks for reflection on teaching often emphasize competencies and behaviours. However, other aspects of teacher functioning are also important, such as the teaching environment and individual teachers' beliefs, professional identity and mission. In a study among five medical school teachers, we explored how a portfolio stimulated reflections on the various aspects of teaching functioning.	The analysis yielded examples of reflections on all aspects of Korthagen's model, although reflections on competencies proved easiest to identify.

For peer review only

No.	Title	Author/year	MERSQI	COREQ	Reason for inclusion			Undergraduate (UG)/Postgraduate (PG)	Methodology	Purpose of study	Key findings
					Communication	Ethics	Professionalism				
									select artefacts in evidence of their teaching activities, compose a profile of a good teacher and reflect on that. The final assign- ment required them to compose a reflective portfolio, using the previous assignments as points of departure. The assignments were intended to stimulate reflection on all aspects of teacher functioning as defined in Korthagen's model. However, the critical incidents assignment was focused on the environment, behaviour, and competencies. Table 1 presents brief descriptions of the assignments.		
16	A method for defining competency-based promotion criteria for family medicine residents	Torbeck, L. Wrightson, A. S. 2005	-	-	✓		✓	PG	In 2003, both of us began the task of developing promotion criteria by searching the literature for studies identifying standards or criteria for resident promotion. Few studies were found related to residents' progress and	The Accreditation Council for Graduate Medical Education (ACGME) has mandated a shift from a structure- and process-based educational system to a competency-based system. The ACGME has not provided criteria (standards), preferring to leave that to the discretion	The value of promotion criteria Training residents to become competent health care providers has been and always will be an important and challenging endeavor. With the ACGME's new mandate, many program directors and house office staff are looking for useful guidance to make their residency programs compliant. We believe that developing a

For peer review only

No.	Title	Author/year	MERSQI	COREQ	Reason for inclusion			Undergraduate (UG)/Postgraduate (PG)	Methodology	Purpose of study	Key findings
					Communication	Ethics	Professionalism				
									promotion.[2,7] We then reviewed the information contained within the last three Residency Assistance Program workshops of the American Academy of Family Physicians, 2001–2003. Frey et al.'s[8] 2003 outpatient core competency presentation notes were earmarked as having specific core competencies that could easily be rewritten as promotion criteria. Next, we reviewed the residency curriculum of the Department of Family Practice and Community Medicine at the University of Kentucky College of Medicine and listed promotion criteria that we thought were important for Family Medicine residents at each level of that curriculum to achieve. Taking this list and extrapolating from Frey et al.'s work, we composed a list of working promotion criteria.	of the individual training programs. Such criteria and an overall strong evaluation process are essential for residents to attain the appropriate knowledge, skills, and attitudes. With this need in mind, the authors describe an evaluation process in which they developed ACGME-competency-based promotion criteria for family medicine residents at the University of Kentucky College of Medicine in 2004.	set number of promotion criteria that are competency-based should be among the first tasks residency programs should tackle, for several reasons.<br><br>First, having defined criteria will make the three to seven years of residency performance assessment more educationally sound. The education literature reminds us that two components are mandatory to a performance assessment: the task and the criteria.[11,12] Since continuous performance assessment is carried out during the majority of a resident's training, the awareness of what is expected of the resident can be raised for both the resident and the faculty by making the task and criteria clearer to them. Residents then are better able to demonstrate that which is expected of them, and faculty are better prepared to evaluate them.<br><br>Second, having defined promotion criteria can better target remediation in those residents failing to meet the criteria in a timely manner. Intervention during a time early on in the resident's development is much more beneficial than it is just before making a decision to promote or not promote.<br><br>Third, having criteria can improve faculty development, especially in terms of how to assess competence, how to give feedback, and how to account for faculty

For peer review only

No.	Title	Author/year	MERSQI	COREQ	Reason for inclusion			Undergraduate (UG)/Postgraduate (PG)	Methodology	Purpose of study	Key findings
					Communication	Ethics	Professionalism				
											responsibility in training and evaluating competent physicians.
17	Enhanced skills in global health and health equity: Guidelines for curriculum development	Dawe, R., Pike, A., Kidd, M., Janakiram, P., Nicolle, E. and Allison, J.<br><br>2017	10	10		✓		PG	A panel comprised of 34 experts in global health education and practice completed three rounds of a Delphi small group process.	To generate consensus on the essential components of a Global Health/Health Equity Enhanced Skills Program in family medicine.	Consensus (defined as ≥ 75% agreement) was achieved on program length (12 months), inclusion of both domestic and international components, importance of mentorship, methods of learner assessment (in-training evaluation report, portfolio), four program objectives (advocacy, sustainability, social justice, and an inclusive view of global health), importance of core content, and six specific core topics (social determinants of health, principles and ethics of health equity/global health, cultural humility and competency, pre and post-departure training, health systems, policy, and advocacy for change, and community engagement).<br><br>Panellists agreed on a number of program components forming the initial foundation for an evidence- informed, competency-based Global Health/Health Equity Enhanced Skills Program in family medicine.
18	Learning portfolios in radiology residency education: how do I get started?	Deitte, L.<br><br>2008	-	-	✓		✓	PG	-	The purpose of this article is three-fold: (1) to famil- iarize the reader with learning portfolios in medical edu-cation, (2) to outline the new radiology ACGME re- quirements for a portfolio, and (53) to discuss strategies for the implementation of a portfolio as a learning and	As medical education and continuous professional devel- opment shift toward an outcomes-based model, there is increasing focus on documentation of physician self-as- sessment and a commitment to lifelong learning. The ACGME program requirements for diagnostic radiology graduate medical education effective July 1, 2008, in- clude learning portfolios as

No.	Title	Author/year	MERSQI	COREQ	Reason for inclusion			Undergraduate (UG)/Postgraduate (PG)	Methodology	Purpose of study	Key findings
					Communication	Ethics	Professionalism				
										assessment tool in a residency education program and continued professional development..	one method for this docu- mentation. Although there are some challenges associ- ated with the implementation of learning portfolios, it is likely that portfolios will prove to be a valuable learning and assessment tool not only in graduate medical educa- tion but also throughout a physician’s career and recerti- fication process.
19	Evaluation of an established learning portfolio	Vance, G.<br>Williamson, A.<br>Frearson, R.<br>O'Connor, N.<br>Davison, J.<br>Steele, C.<br>Burford, B.<br><br>2013	9	-	✓			PG	Deanery-wide trainee attitudes were surveyed by an electronic questionnaire in 2009 and compared with perceptions recorded during the pilot phase (2004–2005).	The trainee-held learning portfolio is integral to the foundation programme in the UK. In the Northern Deanery, portfolio assessment is standar- dised through the Annual Review of Competence Progression (ARCP) process. In this study we aimed to establish how current trainees evaluate portfolio-based learning and ARCP, and how these attitudes may have changed since the foundation programme was first introduced.	Many trainees continue to view the e-portfolio negatively. Indeed, significantly fewer train- ees in 2009 thought that the e-portfolio was a ‘good idea’ or a ‘worthwhile investment of time’ than in 2005. Trainees remain unconvinced about the educa- tional value of the e-portfolio: fewer trainees in 2009 regarded it as a tool that might help focus on training or recognise individual strengths and weaknesses. Issues around unnecessary bureaucracy persist. Current trainees tend to understand how to use the e- portfolio, but many did not know how much, or what evidence to collect. Few supervisors were re- ported to provide useful guidance on the portfolio. ARCP encouraged portfolio completion but did not give meaningful feedback to drive future learning.<br><br>Continued support is needed for both trainees and supervisors in portfolio-building skills and in using the e-portfolio as an educational tool. Trainee-tailored feedback is needed to ensure that portfolio-

For peer review only

No.	Title	Author/year	MERSQI	COREQ	Reason for inclusion			Undergraduate (UG)/Postgraduate (PG)	Methodology	Purpose of study	Key findings
					Communication	Ethics	Professionalism				
											based assessment promotes lifelong, self-directed and reflective learners. Trainees remain unconvinced about the educational value of the e-portfolio
20	Reflective Practice for Patient Benefit: An Analysis of Doctors' Appraisal Portfolios in Scotland	Wakeling, J. Holmes, S. Boyd, A. Tredinnick-Rowe, J. Cameron, N. Marshall, M. Bryce, M. Archer, J. 2019	-	16	✓			PG	Using an in-depth case study approach, 18 online portfolios in Scotland were examined with a template developed to record the types of supporting information submitted and how far these showed reflection and/or changes to practice. Data from semistructured interviews with the doctors (n = 17) and their appraisers (n = 9) were used to contextualize and broaden our understanding of the portfolios.	Reflective practice has become the cornerstone of continuing professional development for doctors, with the expectation that it helps to develop and sustain the workforce for patient benefit. Annual appraisal is mandatory for all practicing doctors in the United Kingdom as part of medical revalidation. Doctors submit a portfolio of supporting information forming the basis of their appraisal discussion where reflection on the information is mandated and evaluated by a colleague, acting as an appraiser.	Portfolios generally showed little written reflection, and most doctors were unenthusiastic about documenting reflective practice. Appraisals provided a forum for verbal reflection, which was often detailed in the appraisal summary. Portfolio examples showed that reflecting on continued professional development, audits, significant events, and colleague multisource feedback were sometimes considered to be useful. Reflecting on patient feedback was seen as less valuable because feedback tended to be uncritical. The written reflection element of educational portfolios needs to be carefully considered because it is clear that many doctors do not find it a helpful exercise. Instead, using the portfolio to record topics covered by a reflective discussion with a facilitator would not only prove more amenable to many doctors but would also allay fears of documentary evidence being used in litigation.
21	Developing an Evaluation Tool for Assessing Clinical Ethics Consultation Skills in Simulation Based	Wasson, K. Parsi, K. McCarthy, M. Siddall, V. J. Kuczewski, M. 2016	-	-		✓		PG	At the beginning of the project development, four of the authors (who are bioethicists with experience in	The American Society for Bioethics and Humanities has created a quality attestation (129) process for clinical ethics consultants;	Given the move toward quality attestation by ASBH and the need to develop a way of assessing the interpersonal skills of clinical ethics consultants ''live'' we have developed the ACES

For peer review only

No.	Title	Author/year	MERSQI	COREQ	Reason for inclusion			Undergraduate (UG)/Postgraduate (PG)	Methodology	Purpose of study	Key findings
					Communication	Ethics	Professionalism				
	Education: The ACES Project								clinical ethics consultation) agreed that any tool created should be grounded in the ASBH core competencies, as they are the articulated professional standards in the field. After a review of the limited assessment tools available, we agreed to draw from the VA's Ethics Consultant Proficiency Assessment Tool (2014). It draws on the ASBH core competencies, including the categories of Interpersonal Skills, Process Skills, Analytic Skills, and Core Knowledge, is quite comprehensive, and has been in use for a number of years. In addition, we followed Stufflebeam's (2000) guidelines for creating a checklist evaluation tool.<br><br>Given the focused usage of the ACES tool—clinical ethics simulation in an educational setting—we met regularly to	the pilot phase of reviewing portfolios has begun. One aspect of the QA process which is particularly challenging is assessing the interpersonal skills of individual clinical ethics con- sultants. We propose that using case simulation to evaluate clinical ethics consul- tants is an approach that can meet this need provided clear standards for assessment are identified. To this end, we developed the Assessing Clinical Ethics Skills (ACES) tool, which identifies and specifies specific behaviors that a clinical ethics consultant should demonstrate in an ethics case simulation. The aim is for the clinical ethics consultant or student to use a videotaped case simulation, along with the ACES tool scored by a trained rater, to demonstrate their competence as part of their QA portfolio.	tool. Our tool provides a clear structure for key domains, behaviors, and interpersonal skills that can be observed and scored by a trained rater during simulated ethics consultations. Individuals completing the ACES course or using the tool can opt to include their evaluation and video as part of their QA portfolio as a means of demonstrating their interpersonal skills in a simulated ethics consultation. Our tool also provides a framework and structure for the content of an ethics consultation and helps translate the core competencies into practical areas, behaviors, and skills. We spent a great deal of time thinking about and debating which items from the VA tool to include in the final ACES tool. It is not intended to be comprehensive for all aspects of clinical ethics consultation, e.g., for simplicity in the pilot stage we did not examine the clinical ethics consultant's ability to review and decipher the medical chart or write a note, though these elements could be added to the simulations in future. Instead, it focuses on those elements we thought could be captured in simulated clinical ethics consultations in an educational setting in this pilot phase. In the future, other elements of ethics consultation and levels of complexity can be added to the simulations as well as additional cases.

For peer review only

No.	Title	Author/year	MERSQI	COREQ	Reason for inclusion			Undergraduate (UG)/Postgraduate (PG)	Methodology	Purpose of study	Key findings
					Communication	Ethics	Professionalism				
									determine which items from the VA tool would be necessary for this purpose. Our tool is not meant to be a comprehensive tool to assess every element of an ethics consultation or CEC's role, thus it is briefer than the VA tool. It is also designed to be used in ''real time'' by trained bioethics raters, not retrospectively self-administered by the ethics consultant. Once we agreed upon the primary items, we met weekly for 3–4 months to identify and determine the specific behaviors and skills we would be evaluating under each main item. This was a time consuming process involving in-depth discussion and debate. Before each weekly meeting, we viewed videos of simulated ethics consultations from past graduate bioethics courses to refine our tool, its specified items, and behaviors. We discussed and		For clarity of assessment, we developed the ACES tool to be used for individual consultants. We believe each individual ethics consultant needs to have this set of skills, even if they work within a small team model in practice. If the ACES tool was adapted to assess a small team of ethics consultants, the raters would have to rate each consultant individually or rate them all as one ''ethics consultant'' to assess whether the team covered all the necessary items on the evaluation. This approach would be more complicated to assess and, we suspect, much less straightforward to score. At this stage, we do not recommend using the ACES tool in this way.<br>One of the most challenging areas in the ACES tool development was how to view and rate students in a similar way—or inter-rater agreement. We spent a great deal of time discussing what qualifies as ''Done,'' ''Not Done'' or ''Done Incorrectly'' for each and every item. Rater agreement is an ongoing process which needs to be practiced and refreshed, and is not unique to our setting. We plan to assess and recalibrate rater agreement prior to each time a course is offered (1–2 per annum). What makes it more challenging in clinical ethics is the nature of some parts of the consultation; e.g., identifying and clarifying the ethical issues. What counts as ''Done'' for certain items in

For peer review only

No.	Title	Author/year	MERSQI	COREQ	Reason for inclusion			Undergraduate (UG)/Postgraduate (PG)	Methodology	Purpose of study	Key findings
					Communication	Ethics	Professionalism				
									agreed on all of the behaviors and skills listed under each item in the ACES tool. In the end, we agreed on 12 main items and 35 corresponding behaviors to be evaluated and scored.		our tool can be interpreted differently depending on the rater's background and potential biases. This type of item— communication and interpersonal skills in ethics—is not always as neat and clear cut as in other clinical areas such as surgical preparation checklists or putting in a central line. Further work needs to be done to train additional raters if we are to use the tool with more people over time. We plan to recruit additional students to take the ACES course and are working on refining the cases used and plan to evaluate the tool in other settings to obtain feedback on its reliability. When we used the ACES tool in the basic clinical ethics consultation course the feedback was overwhelmingly positive. Students appreciated a clear articulation of what was expected in an ethics consult. While recognizing the tool was not designed to be comprehensive, they valued having a structure and framework for thinking about the elements of an ethics consultation and the behaviors they should be exhibiting. It gave them a clear standard by which to assess themselves and others.
22	Evaluating practice-based learning and improvement: efforts to improve acceptance of portfolios	Fragneto, R. Y., Dilorenzo, A. N., Schell, R. M. and Bowe, E. A. 2010	8		✓		✓	PG	Intensive education about the goals and importance of portfolios began in January 2006, including presentations at departmental conferences and	To find out whether educational efforts improved acceptance of the portfolio and retrospectively audited the portfolio evaluation forms completed by faculty advisors.	Portfolios are considered a best methods techni- que by the ACGME for evaluation of practice-based learning and improvment. We have found that intensive education about the goals and importance of portfolios can enhance acceptance of this evaluation tool, resulting

For peer review only

No.	Title	Author/year	MERSQI	COREQ	Reason for inclusion			Undergraduate (UG)/Postgraduate (PG)	Methodology	Purpose of study	Key findings
					Communication	Ethics	Professionalism				
									one-on-one education sessions. Faculty advisors were instructed to evaluate each resident's portfolio and complete a review form. We retrospectively collected data to determine the percentage of review forms completed by faculty. The portfolio reviews also assessed the percentage of 10 required portfolio components residents had completed.		in improved compliance in completion and evaluation of portfolios.
23	Use of portfolios for assessment of global health residents: qualitative evaluation of design and implementation	Gibson, C., Chandratilake, M. and Hull, A.<br><br>2018	-	22	✓			PG	A qualitative study was conducted with academic experts in Canadian residency training, as well as directors and residents involved in Global Health study in order to assess the validity and benefit of such a tool. Through an online survey, interviews, and focus groups, views on the portfolio and intended content were collected and coded thematically.	To determine the robustness of portfolios as an assessment tool for Global Health Residents.	This qualitative evaluation validated the use of portfolios for this cohort of students while yielding comments and suggestions that will further enhance the interactive and flexible nature of this seldom used assessment tool. These findings contribute to the understanding of how Global Health assessment can remain individualized yet rigorous.
24	Assessment and Change: An Exploration of	Jocelyn M. Lockyer, PhD; Sanjeev Sockalingam, MD,	-	16	✓			PG	A total of 5000 entries from 2195 psychiatrists were	Fellows of the Royal College of Physicians and	There were 3841 entries for analysis; 1159 entries did not meet the criteria for

For peer review only

No.	Title	Author/year	MERSQI	COREQ	Reason for inclusion			Undergraduate (UG)/Postgraduate (PG)	Methodology	Purpose of study	Key findings
					Communication	Ethics	Professionalism				
	Documented Assessment Activities and Outcomes by Canadian Psychiatrists	MHPE, FRCPC; Craig Campbell, MD, FRCPC<br><br>2018							examined. A thematic analysis drawing on the framework analysis was undertaken of the 2016 entries.	Surgeons of Canada are required to participate in assessment activities for all new 5-year cycles beginning on or after January 2014 to meet the maintenance of certification program requirements. This study examined the assessment activities which psychiatrists reported in their maintenance of certification e-portfolios to determine the types and frequency of activities reported; the resultant learning, planned learning, and/or changes to the practice they planned or implemented; and the interrelationship between the types of assessment activities, learning that was affirmed or planned, and changes planned or implemented.	assessment. The most commonly reported activities were self-assessment programs, feedback on teaching, regular performance reviews, and chart reviews. Less frequent were direct observation, peer supervision, and reviews by provincial medical regulatory authorities. In response to the data, psychiatrists affirmed that their practices were appropriate, identified gaps they intended to address, planned future learning, and/ or planned or implemented changes. The assessment activities were internally or externally initiated and resulted in no or small changes (accommodations and adjustments) or redirections.<br><br>Psychiatrists reported participating in a variety of assessment activities that resulted in variable impact on learning and change. The study underscores the need to ensure that assessments being undertaken are purposeful, relevant, and designed to enable identification of outcomes that impact practice.
25	Development of a portfolio of learning for postgraduate family medicine training in South Africa: a Delphi study	Jenkins, L., Mash, B. and Derese, A.<br><br>2012	-	10	✓	✓		PG	A workshop held at the WONCA Africa Regional Conference in 2009 explored the purpose and broad contents of the portfolio. The 85 training outcomes, ideas from the WONCA workshop, the literature, and	This study aimed to achieve a consensus on the contents and principles of the first national portfolio for use in family medicine training in South Africa.	A portfolio was developed and distributed to the eight departments of Family Medicine in South Africa, and the CMSA, to be further tested in implementation. This was the first attempt to reach consensus on the development of a national portfolio for family medicine training in South Africa. Consensus was reached on 50 items to include, and 26

For peer review only

No.	Title	Author/year	MERSQI	COREQ	Reason for inclusion			Undergraduate (UG)/Postgraduate (PG)	Methodology	Purpose of study	Key findings
					Communication	Ethics	Professionalism				
									existing portfolios in the various universities were used to develop a questionnaire that was tested for content validity by a panel of 31 experts in family medicine in South Africa, via the Delphi technique in four rounds. Eighty five content items (national learning outcomes) and 27 principles were tested. Consensus was defined as 70% agreement. For those items that the panel thought should be included, they were also asked how to provide evidence for the specific item in the portfolio, and how to assess that evidence.		principles relating to the port- folio. A draft national portfolio and portfolio guide have been developed and distributed to all the medical schools in the country. Further revision and testing with registrars in training is underway, with the aim to deli- ver a final portfolio in the following year.
26	The national portfolio of learning for postgraduate family medicine training in South Africa: experiences of registrars and supervisors in clinical practice	Jenkins, L., Mash, B. and Derese, A.<br><br>2013	-	15	✓			PG	Semi-structured interviews were conducted with 17 purposively selected registrars and supervisors from all eight South African training programmes.	The aim of this study was to explore registrars' and supervisors' experience regarding the portfolio's educational impact, acceptability, and perceived usefulness for assessment of competence.	The learning portfolio had a significant educational impact in shaping work- place based supervision and training and providing formative assessment. Its acceptability and usefulness as a learning tool should increase over time as supervisors and registrars become more competent in its use. There is a need to clarify how it will be used in summative assessment.
27	Demonstration of Portfolios to Assess	PATRICIA S. O'SULLIVAN1,* , MARK D. RECKASE2, TINA	8	12	✓		✓	PG	22 participants in this demonstration project were all	Describes assessment of the reliability and validity of portfolios in a	We must continue to emphasize some important qualities of portfolios: res- ident choice and reflection.

For peer review only

No.	Title	Author/year	MERSQI	COREQ	Reason for inclusion			Undergraduate (UG)/Postgraduate (PG)	Methodology	Purpose of study	Key findings
					Communication	Ethics	Professionalism				
	Competency of Residents	MCCLAIN3, MILDRED A. SAVIDGE1 and JAMES A. CLARDY3<br><br>2004							residents in the psychiatry residency education program at the primary affiliate of a state university medical school.	psychiatry residency program.	These are important to the life-long learner. Portfolio has been able to aid the faculty in meeting ACGME expectations and has oriented programe to the increasingly important role of evaluation.
28	Developing and Successfully Implementing a Competency-Based Portfolio Assessment System in a Postgraduate Family Medicine Residency Program	Laura A. McEwen, PhD, Jane Griffiths, MD, CCFP, FCFP, and Karen Schultz, MD, CCFP, FCFP<br><br>2015	-	-	✓			PG	-	The use of portfolios in postgraduate medical residency education to support competency development is increasing; however, the processes by which these assessment systems are designed, implemented, and maintained are emergent. The authors describe the needs assessment, development, implementation, and continuing quality improvement processes that have shaped the Portfolio Assessment Support System (PASS) used by the postgraduate family medicine program at Queen's University since 2009.	The authors discuss the identification of impact measures at the individual, programmatic, and institutional levels and the ways the department uses these to monitor how PASS supports competency development, scaffolds residents' self- regulated learning skills, and promotes professional identity formation. They describe the "academic advisor" role and provide an appendix covering the portfolio elements. Reflection elements include learning plans, clinical question logs, confidence surveys, and reflections about continuity of care and significant incidents. Learning module elements cover the required, online bioethics, global health, and consult- request modules. Assessment elements cover each resident's research project, clinical audits, presentations, objective structured clinical exam and simulated office oral exam results, field notes, entrustable professional activities, multisource feedback, and in-training evaluation reports. Document elements are the resident's continuing medical education activities including procedures log, attendance log, and patient demographic summaries.

For peer review only

No.	Title	Author/year	MERSQI	COREQ	Reason for inclusion			Undergraduate (UG)/Postgraduate (PG)	Methodology	Purpose of study	Key findings
					Communication	Ethics	Professionalism				
29	Diving for PERLS Diving for PERLS Working and Performance Portfolios for Evaluation and Reflection on Learning	Linda E. Pinsky, MD, Kelly Fryer-Edwards, PhD<br><br>2004	-	-	✓		✓	PG	-	Professional competence requires a commitment to lifelong learning, self-assessment, and excellence. Complex skills such as these require flexible and comprehensive teaching and assessment measures. We describe a combination of working and performance portfolios that both foster and evaluate the development of professional competence. We explain the con- ceptual and practical underpinnings that maximize the effec- tiveness of these tools. Drawing on experience with University of Washington residents, we identify 5 criteria that can help promote successful use of portfolios: separate working and performance functions of portfolios, developing a supportive climate, developing skills in faculty and residents, observing progress over time, and fostering mentorship opportunities.	To incorporate portfolios successfully into a residency program, we recommend including 5 elements (Table 1). We had success in our program by introducing the program slowly, and dedicating time to faculty education on men- toring, reflection, goal setting, and feedback skills, recog- nizing that the use of portfolios requires new skills and a cultural transformation. We are pleased by the residents' appreciation of the process, their honesty in sharing and evaluating personal areas of challenge, and their incorpora- tion of this approach into other areas of their education. For example, a current inpatient chief resident, based on his ex- perience of the importance of goal setting to learning, revised the structure of morning report to include weekly goal-setting sessions as well as a method to evaluate its effectiveness. Residents seeking employment report finding components of the portfolio helpful in creating their CVs, crafting their applications, and as display examples at job applications.<br><br>To date, the literature reports few trials of the portfolio method for assessing professional competence. The Post Graduate Education Accreditation (PGEA) in Britain illus- trates sharing responsibility for learning between teachers and learners. PGEA experimented with portfolios

For peer review only

No.	Title	Author/year	MERSQI	COREQ	Reason for inclusion			Undergraduate (UG)/Postgraduate (PG)	Methodology	Purpose of study	Key findings
					Communication	Ethics	Professionalism				
											as a flex- ible, targeted means of meeting continuing medical edu- 18 –20 cation (CME) requirements. In their pilot study, physicians were responsible for identifying their own learning objectives and outlining how they were going to achieve and document those objectives. The physicians each met with a mentor to review appro- priate objectives and realistic means for achieving them. Structured reflection was built into the process and was seen as strength, explicitly moving away from the passive approach of most traditional lecture-based CME courses.<br>In the PGEA experience, the shortcomings identified were time and mentor availability. However, participants found the “deep learning” and personal mastery they achieved to be well worth it. In addition, accrediting bodies had something of substance to review in assessing the practitioners’ continuing education.<br>Portfolios fill a need within training programs to foster 21–23 and document professional competencies.<br>dents, training is a time of professional identity formation. The current format of most residencies — didactic confer- ences and time-consuming clinical work—emphasizes either a passive or unreflective learner role.<br>Through portfolios, residents can experience

For peer review only

No.	Title	Author/year	MERSQI	COREQ	Reason for inclusion			Undergraduate (UG)/Postgraduate (PG)	Methodology	Purpose of study	Key findings
					Communication	Ethics	Professionalism				
											and develop the skills of lifelong learning, critical thinking, self- assessment, and excellence that can be carried forward into their future careers and practices. They can complete residency with a clearer sense of their own strengths and limitations, and a sense of who they are as physicians.
30	How do postgraduate GP trainees regulate their learning and what helps and hinders them? A qualitative study	Sagasser, M. H., Kramer, A. W. and van der Vleuten, C. P.<br><br>2012	-	18	✓			PG	In a qualitative study with a phenomenologic approach we interviewed first- and third-year GP trainees from two universities in the Netherlands. Twenty-one verbatim transcripts were coded. Through iterative discussion the researchers agreed on the interpretation of the data and saturation was reached.	To explore how postgraduate trainees regulate their learning in the workplace, how external regulation promotes self-regulation and which elements facilitate or impede self-regulation and learning.	Trainees used a short and a long self-regulation loop. The short loop took one week at most and was focused on problems that were easy to resolve and needed minor learning activities. The long loop was focused on complex or recurring problems needing multiple and planned longitudinal learning activities. External assessments and formal training affected the long but not the short loop. The supervisor had a facilitating role in both loops.
31	First year specialist trainees' engagement with reflective practice in the e-portfolio	Helen McNeill • Jeremy M. Brown • Nigel J. Shaw<br><br>2010	7.5	12	✓			PG	A modified Delphi technique was used to develop a grading system to indentify the level of reflection recorded by participants in the e-portfolio. Transcripts of the reflective accounts were then analysed using a qualitative approach which involved coding and categorising	This study aims specifically to explore how they have engaged in reflection on their practice and how they utilise their learning portfolio to document evidence of this.	Findings indicate there is variation is the extent to which doctors both engage in and document evidence of reflection. Further research is needed to explore factors that enable or inhibit the use of the e-portfolio for reflection and whether recorded reflection is a true picture of the cognitive process involved.

For peer review only

No.	Title	Author/year	MERSQI	COREQ	Reason for inclusion			Undergraduate (UG)/Postgraduate (PG)	Methodology	Purpose of study	Key findings
					Communication	Ethics	Professionalism				
									the data. This study demonstrated a wide variation in both the quantity and quality of reflection. Of particular note in the qualitative data analysis were themes relating to clinical knowledge and skills, learning in practice, communication, feelings, types of learning experience reflected on and wider aspects of medical practice.		
32	Development of a learning portfolio to assess the competency of anesthesia residents in Thailand	Suraseranivongse, S., Chinachoti, T., Aroonpruksakul, N., Halilamien, P., Rushatamukayanunt, P., Raksamani, K., Sirivanasandha, B. and Mandee, S.<br><br>2011	12.5	8			✓	PG	Learning portfolio was developed from Thai Medical Council general competencies, academic activities, and performance assessment in several modalities. Twenty-four first year anesthesia residents and eight mentors were enrolled for this study. One staff mentored three residents and rated their competencies in portfolios, twice, four-months apart. Content validity was assessed by six content experts. Concurrent validity of portfolio was determined	Develop a portfolio for learning improvement in first year anesthesia residents in Thailand, and validate this portfolio as a competency evaluation and to identify strength and weakness of implementation.	All content experts accepted that this portfolio could assess general competencies of the first year anesthesia residents. The majority of mentors and residents (>70%) agreed with the benefit of portfolio based on learning development and competency assessment. However, half of residents were not satisfied with the burden from portfolio

For peer review only

No.	Title	Author/year	MERSQI	COREQ	Reason for inclusion			Undergraduate (UG)/Postgraduate (PG)	Methodology	Purpose of study	Key findings
					Communication	Ethics	Professionalism				
									by agreement with faculty global rating and in-training examination. Inter-rater reliability of portfolio was evaluated by five faculties that rated 24 residents. Practicality was commented upon by all mentors and residents in the questionnaire and semi-structure, open-ended questions.		
33	Education Research: Communication skills for neurology residents - Structured teaching and reflective practice	Watling, C. J. and Brown, J. B.<br><br>2007	8.5	10	✓	✓		PG	A group of 12 neurology residents participated in a series of six case-based communi-cation skills workshops. Each workshop focused on a particular clinical scenario, including break-ing bad news, discussing do-not-resuscitate orders, communicating with "difficult" patients, disclosing medical errors, obtaining informed consent for neurologic tests and procedures, and discussing life-and-death decisions with families of critically ill patients.	Despite the importance of communication skills for neurologists, specific training in this area at the residency level is often lacking. This study aimed to enhance learning of these skills and to encourage reflective practice around communication skills.	The program was well accepted, and residents rated the workshops as effective and relevant to their practice. Analysis of residents' portfolios revealed three themes relevant to patient–physician communication: 1) communication is more successful when adequate time is allowed, 2) the ability to empathize with patients and their families is essential to successful interactions, and 3) the develop- ment of specific approaches to challenging scenarios can facilitate effective interactions. The portfo- lios also demonstrated that residents would engage in reflective practice.<br><br>Targeting of communication skills training around specific clinical scenarios using neurologic cases was well accepted and was deemed relevant to practice. The use of portfolios may

For peer review only

No.	Title	Author/year	MERSQI	COREQ	Reason for inclusion			Undergraduate (UG)/Postgraduate (PG)	Methodology	Purpose of study	Key findings
					Communication	Ethics	Professionalism				
									Residents also kept reflective portfolios in which real examples of these interactions were recorded.		promote lifelong learning in this area.
34	What the HEC-C? An Analysis of the Healthcare Ethics Consultant-Certified Program: One Year in	Horner, C. Childress, A. Fantus, S. Malek, J.<br><br>2020			✓	✓		PG	-	In this paper, we explore the history that has led to this certification process, and evaluate the ability of the HEC-C Program to meet the goals it has set forth for HCECs. We describe the benefits and weak- nesses of the program and offer constructive feedback on how the process might be strengthened, as well as share our team's experience in preparing for the exam.	Ultimately, while we are generally supportive of efforts to professionalize the field and ensure competence among HCECs, we are skeptical that a limited process such as this provides much assurance of such compe- tence either for HCECs or their employers. We pro- pose that a more robust or two-tiered evaluation process is needed before such a credential could reli- ably attest to the competence and expertise of an HCEC.<br>Despite shortcomings in the Certification Process as it is currently designed, in general there are benefits of having some kind of certification for individual consultants, as well as HCEC programs and the broader HCEC field. For consultants, achieving and maintaining certification requires continued review of core materials to refresh ethics knowledge and pro- vides external validation of the consultant's ability to engage in consultation, boosting confidence that the individual is successfully engaged in continuing educa- tion. For HCEC programs, having credentialed con- sultants supports the program's mission to engage in quality consultation, taking a step toward ensuring individual consultants are performing their job in a manner

For peer review only

No.	Title	Author/year	MERSQI	COREQ	Reason for inclusion			Undergraduate (UG)/Postgraduate (PG)	Methodology	Purpose of study	Key findings
					Communication	Ethics	Professionalism				
											consistent with established standards in the field. Finally, for the HCEC community as a whole, having a singular credential helps to standardize the practice and validate ethics consultation as a defined and important healthcare specialty. This effort to legit- imize the field as a profession will support the inclu- sion of clinical ethics across healthcare, hopefully expanding the practice from a few select academic or larger healthcare systems to all health care settings. Reaching a point where a health care ethics credential signifies not only competence but also excellence in the ability to conduct a clinical ethics consultation, however, may still be a long way off.
35	Assessing Professionalism in Medicine – A Scoping Review of Assessment Tools from 1990 to 2018	Tay, K. T.<br>Ng, S.<br>Hee, J. M.<br>Chia, E. W. Y.<br>Vythilingam, D.<br>Ong, Y. T.<br>Chiam, M.<br>Chin, A. M. C.<br>Fong, W.<br>Wijaya, L.<br>Toh, Y. P.<br>Mason, S.<br>Krishna, L. K. R.<br><br>2020			✓		✓	UG and PG	Arksey and O'Malley's (2005) approach to scoping reviews was used to identify appropriate publications featured in four data- bases published between 1 January 1990 and 31 December 2018. Seven members of the research team employed thematic analysis to evaluate the selected articles.	Medical professionalism enhances doctor-patient relationships and advances patient-centric care. However, despite its pivotal role, the concept of medical professionalism remains diversely understood, taught and thus poorly assessed with Singapore lacking a linguistically sensitive, context specific and culturally appropriate assessment tool. A scoping review of assessments of professionalism in medicine was thus carried out to better guide its understanding	Prevailing assessments of professionalism in medicine must contend with differences in setting, context and levels of pro- fessional development as these explicate variances found in existing assessment criteria and approaches. However, acknowledging the sig- nificance of context-specific competency-based stages in medical professionalism will allow the forwarding of guiding principles to aid the design of a culturally-sensitive and practical approach to assessing professionalism.

For peer review only

No.	Title	Author/year	MERSQI	COREQ	Reason for inclusion			Undergraduate (UG)/Postgraduate (PG)	Methodology	Purpose of study	Key findings
					Communication	Ethics	Professionalism				
36	Content and outcomes of narrative medicine programmes: a systematic review of the literature through 2019	Remein, Christy DiFrances Childs, Ellen Pasco, John Carlo Trinquart, Ludovic Flynn, David B. Wingerter, Sarah L. Bhasin, Robina M. Demers, Lindsay B. Benjamin, Emelia J.<br><br>2020	-	-	✓			PG	We conducted a systematic review of literature published through 2019 using five major databases: PubMed, Embase, PsycINFO, ERIC and MedEdPORTAL. Eligible NM programming included textual analysis/close reading of published literature and creative/reflective writing. Qualifying participants comprised individuals from academic medicine and health sciences disciplines. We reviewed and categorised programme goals, content and evaluation activities to assess participant satisfaction and programme efficacy. Two members of the research team assessed the risk of bias, independently screening records via a two-round, iterative process to reach consensus on eligibility.	Narrative medicine (NM) incorporates stories into health sciences paradigms as fundamental aspects of the human experience. The aim of this systematic review is to answer the research question: how effective is the implementation and evaluation of NM programmes in academic medicine and health sciences? We documented objectives, content and evaluation outcomes of NM programming to provide recommendations for future narrative-based education.	Of 1569 original citations identified, we selected 55 unique programmes (described in 61 records). In all,<br>41 (75%) programmes reported a form of evaluation; evaluation methods lacked consistency. Twenty-two programmes used quantitative evaluation (13 well described), and 33 programmes used qualitative evaluation (27 well described). Well-described quantitative evaluations relied on 32 different measures (7 validated) and showed evidence of high participant satisfaction and pre-post improvement in competencies such as relationship- building, empathy, confidence/personal accomplishment, pedagogical skills and clinical skills. An average of 88.3% of participants agreed or strongly agreed that the programme had positive outcomes. Qualitative evaluation identified high participant satisfaction and improvement<br>in competencies such as relationship-building, empathy, perspective-taking/reflection, resilience and burnout detection/mitigation, confidence/personal accomplishment, narrative competence, and ethical inquiry.<br><br>Evaluation suggests that NM programming leads to high participant satisfaction and positive outcomes

No.	Title	Author/year	MERSQI	COREQ	Reason for inclusion			Undergraduate (UG)/Postgraduate (PG)	Methodology	Purpose of study	Key findings
					Communication	Ethics	Professionalism				
											across various competencies. We suggest best practices and innovative future directions for programme implementation and evaluation.
37	Use of portfolios in early undergraduate medical training	Driessen, Erik<br>Van Tartwijk, Jan<br>Vermunt, Jan<br>van der Vleuten, Cees<br><br>2003		10		✓		UG	During the academic year 2001–02 242 first-year medical students compiled a portfolio. Semi-structured interviews were held with a select group of students to explore the effect of the portfolio on reflection. Students from four random mentor groups, 39 in total, were approached and asked if they would be interviewed on the portfolio. With the exception of one student, all students were prepared to take part in the interview. The interview sched-ule focused on the process of compiling a portfolio and its possible effect on reflective ability. In addition to this, students were given the opportunity to offer suggestions for improvement.	This article describes the use of portfolios in **early** undergraduate medical training... In this article, we will describe the Maastricht portfolio as an illustration of an early undergraduate portfolio. We will discuss the reasons for the choices we made in designing the portfolio and report on a first evaluation, focusing on the effects on students' reflective ability.	The majority of students were of the opinion that analysing one's competences in a portfolio was instructive and meaningful. With regard to learning how to reflect and recognize learning needs, however, mentor coaching proved to be necessary. The results thus far show that the portfolio is a worthwhile addition to existing assessment and learning tools. Our preliminary findings show that the introduction of such a portfolio in the early stages of medical training seems to be an effective way for students to learn how to reflect on their learning and behaviour.
38	Conditions for reflective use of portfolios in	Driessen, E. W.<br>van Tartwijk, J.<br>Overeem, K.		18			✓	UG	We designed a portfolio that was aimed at	Portfolios are often used as an instrument with	The conditions for successful reflective use of portfolios that emerged from

For peer review only

No.	Title	Author/year	MERSQI	COREQ	Reason for inclusion			Undergraduate (UG)/Postgraduate (PG)	Methodology	Purpose of study	Key findings
					Communication	Ethics	Professionalism				
	undergraduate medical education	Vermunt, J. D. van der Vleuten, C. P. 2005							stimulating reflection in early undergraduate medical education, using experiences described in the medical educa- tion literature and elsewhere. Conditions for reflect- ive portfolio use were identified through interviews with 13 teachers (mentors), who were experienced in mentoring students in the process of developing their portfolios. The interviews were analysed according to the principles of grounded theory.	which to stimulate students to reflect on their experiences. Research has shown that working with portfolios does not automatically stimulate reflection. In this study we addressed the question: What are the conditions for successful reflective use of portfolios in undergraduate medical education?	the interviews fell into 4 categories: coaching; portfolio structure and guidelines; relevant experiences and materials, and summative assessment. According to the mentors, working with a portfolio designed to meet these conditions will stimulate students' reflective abilities...This study shows that portfolios are a potentially valuable method of assessing and develop- ing students' reflective skills in undergraduate med- ical training, provided certain conditions for effective portfolios are recognised and met. Portfolios have a strong potential for enhancing learning and assess- ment but they are very vulnerable and may easily lead to disappointment. Before implementing portfolios in education, one should first consider whether the necessary conditions can be fulfilled, including an appropriate portfolio structure, an appropriate assessment procedure, the provision of enough new experiences and materials, and sufficient teacher capacity for adequate coaching and assessment.
39	Portfolio Assessment Implementation in Clinical Year of Community Medicine Module: Students Perspective	Ekayanti, Fika Risahmawati, Risahmawati Fadhilah, Marita 2017	7				✓	UG	Nine portfolios were assigned to students within 5 weeks length module. The data was collected by questionnaires from 46 students after completing module in November 2016. Students were	This study aimed to identify the correlation of students' perspective for using portfolio and their portfolio scores.	Most students (36/78.3%) felt that the portfolio was not appropriate as their summative assessment. They preferred less portfolios, 30 (63%) students requested to decrease portfolios to 3-5, 28.3% to 6-8 and only 8.7% to <3. There was no significant correlation between students' satisfaction of using portfolio

No.	Title	Author/year	MERSQI	COREQ	Reason for inclusion			Undergraduate (UG)/Postgraduate (PG)	Methodology	Purpose of study	Key findings
					Communication	Ethics	Professionalism				
									asked about their satisfaction of using portfolio, then correlated to their portfolio scores. The reasons and suggestion for ideal portfolios were identified. Analysis was done by SPSS 20 using Rank Spearman correlation test. Students felt that creating portfolios were not comfortable.		to their portfolios score (p=0.262), while there was significant correlation between students' score to the number of portfolios to be submitted (p=0.017; r=0.349). Reasons for decreasing number of portfolios were inadequate time and many other tasks to be finished. Students in UIN Jakarta haven't used to retelling their experiences and reflecting them in writing as perceived in many students who were not used to portfolios. Doing portfolios need lots of work and time-consuming. Having lesser number of portfolios to be submitted would encourage students to create portfolios. Building familiarization, time management and good motivation for creating portfolios were important to successful portfolios. There was no significant correlation between students' satisfaction to their scores. Students should be encouraged to get comfortable in creating portfolios for the benefit as lifelong learning tool. Motivation to write and reflect should be nurtured to improve students' portfolio satisfaction and commitment.
40	Students' perception of portfolio as a learning tool at King Abdulaziz University Medical School	Fida, N. M. Hassanien, M. Shamim, M. S. Alafari, R. Zaini, R. Mufti, S. Al-Hayani, A. Farouq, M. Al-Zahrani, H. 2018	8	14	✓	✓	✓	UG	Portfolios were introduced in the 2nd through 5th years at King Abdulaziz University over a two-year period (2013–2015). At the end of each academic year, students completed a	Medical education has a longstanding tradition of using logbooks to record activities. The portfolio is an alter- native tool to document competence and promote reflective practice. This study assessed the	The results showed a difference in focus between basic and clinical years: in basic years students' focus was on acquiring practical skills, but in clinical years they focused more on acquiring complex skills, including identifying and man- aging problems. The questionnaire responses nonetheless revealed a

No.	Title	Author/year	MERSQI	COREQ	Reason for inclusion			Undergraduate (UG)/Postgraduate (PG)	Methodology	Purpose of study	Key findings
					Communication	Ethics	Professionalism				
									mixed questionnaire that included a self-assessment of skills learned through the use of portfolio.	acceptance of portfolio use among Saudi undergraduate medical students.	positive trend in acceptance (belief in the educational value) of portfolios among students and their mentors, across the years of the program. Using portfolios as a developmental learning and formative assessment tool in the early undergraduate years was found to contribute to students' ability to create their own clinical skills guidelines in later years, as well as to engage in and appreciate reflective learning.
41	The use of portfolios to foster professionalism: Attributes, outcomes, and recommendations	Renato Soleiman Franco, Camila Ament Giuliani dos Santos Franco,Olívia Pestana,Milton Severo &Maria Amélia Ferreira<br><br>2017		9		✓	✓	UG	A systematic review on the use of portfolios in teaching professionalism to medical students identified 1257 papers. Of these, 11 articles met all inclusion criteria	The main objective of this research was to review the characteristics of portfolios and their outcomes for teaching professionalism to undergraduate medical students	According to the papers, the use of portfolios for teaching professionalism shows versatility, supports learning strategies and has the potential to be used in different contexts, including for formative and summative purposes. The weaknesses were based on the artificiality of the reflections, deficient instructions, time-consuming processes and preference among students for other teaching methods. Students complained about feeling that the reflection was 'forced', and they tended to write based on social conventions rather than reveal their true thoughts. Reflection is a powerful component of the portfolio, but the method by which it is taught could easily ruin its potential to boost professionalism. Requiring reflection did not ensure its achievement; increased understanding by students regarding how and why they were doing it, the clarity of assessment methods and constructive feedback might strengthen the potential for success. A

For peer review only

No.	Title	Author/year	MERSQI	COREQ	Reason for inclusion			Undergraduate (UG)/Postgraduate (PG)	Methodology	Purpose of study	Key findings
					Communication	Ethics	Professionalism				
											framework was designed to support faculty members in developing and applying portfolios with a clear and broad view of this teaching strategy.
42	AMEE Medical Education Guide No. 24: Portfolios as a method of student assessment	Friedman Ben David, M.<br>Davis, M. H.<br>Harden, R. M.<br>Howie, P. W.<br>Ker, J.<br>Pippard, M. J.<br><br>2001	-	-	✓	✓	✓	UG	-	This guide is intended to inform medical teachers about the use of portfolios for student assessment. It provides a background to the topic, reviews the range of assessment purposes for which portfolios have been used, identifies possible portfolio contents and outlines the advantages of portfolio assess- ment with particular focus on assessing professionalism...The current state of understanding of the technical, psychometric issues relating to portfolio assessment is clarified. The final part of the paper provides a practical guide for those wishing to design and implement portfolio assessment in their own institutions.	-
43	Teaching professionalism in the early years of a medical curriculum: a qualitative study	Goldie, J.<br>Dowie, A.<br>Cotton, P.<br>Morrison, J.<br><br>2007		18	✓	✓		UG	A qualitative approach was adopted involving semistructured interviews, on a 1 in 6 sample of tutors involved in teaching in the early curricular years, and 3 student focus groups. The	This study investigated tutors' and students' perspectives of the delivery of profession- alism in the early years of Glasgow's learner-centred, problem-based learning (PBL), integrated medical curriculum.	Involvement in teaching raised students' and tutors' awareness of their professionalism. Learning activities promoting critical reflection were most effective. The integration of professionalism across the domains of Vocational Studies (135) was important for learning; however, it was not well integrated with the

For peer review only

No.	Title	Author/year	MERSQI	COREQ	Reason for inclusion			Undergraduate (UG)/Postgraduate (PG)	Methodology	Purpose of study	Key findings
					Communication	Ethics	Professionalism				
									findings were subjected to between-method triangulation.		PBL core. Integration was pro- moted by having the same tutor present throughout all VS sessions. Early patient contact experiences were found to be particularly important. The hidden curriculum provided both opportunities for, and threats to, learning. The small-group format provided a suitable environment for the examination of pre-existing perspectives. The portfolio was an effective learning tool, although its assessment should be formalised.<br><br>Reflection is integral to professional development. Early clinical contact is an important part of the process of socialisation, as it allows students to enter the community of practice that is the medical profession. Role models can contribute powerfully to students' learning and identity formation. As students move towards fuller participation, the clinical milieu should be controlled to maximise the influence of role models, and opportunities for guided reflection should be sustained.
44	Assessing students' personal and professional development using portfolios and interviews	Gordon, J.<br><br>2003	6.5			✓	✓	UG	The instruments used to assess Year 1 stu- dents in PPD are a portfolio and interview. This assessment format encourages students to explore ideas and values that are important to them	Medical schools are placing more emphasis on students' personal and professional development (PPD) and are seeking ways of assessing student progress towards meeting outcome goals in relation to professionalism. The	In 1997⁄98, 96% of students agreed that they had engaged in useful reflection on their approach to the course and 91% agreed that the experience was worthwhile. A further 76% of students agreed that they could see opportunities to modify their approach in some ways as result of this exercise.

No.	Title	Author/year	MERSQI	COREQ	Reason for inclusion			Undergraduate (UG)/Postgraduate (PG)	Methodology	Purpose of study	Key findings
					Communication	Ethics	Professionalism				
									and relevant to the PPD theme. A confidential interview, based on the PPD goals, is held with a faculty member who has read the student's portfolio.	Faculty of Medicine at the University of Sydney sought an assessment method that would demonstrate the value of reflection in attaining PPD, provide feedback and encourage stu- dents to take responsibility for setting and achieving high standards of performance.	
45	Developing a sustainable electronic portfolio (ePortfolio) program that fosters reflective practice and incorporates CanMEDS competencies into the undergraduate medical curriculum	Hall, P. Byszewski, A. Sutherland, S. Stodel, E. J.<br><br>2012	-	-	✓		✓	UG	-	In this article, the authors describe the development of an electronic Portfolio (ePortfolio) program that enables uOttawa medical students to document their activities and to demonstrate their development of competence in each of the eight roles	The authors reflect on the challenges they faced in the development and implementation of the ePortfolio program and share the lessons they have learned along the way to a successful and sustainable program. These lessons include switching from a complex information technology system to a user-friendly, Web- based blog platform; rethinking orientation sessions to ensure that faculty and students understand the value of the ePortfolio program; soliciting student input to improve the program and increase student buy-in; and providing faculty development opportunities and recognition.
46	The utility of reflective portfolios as a method of assessing first year medical students' personal and professional development	Rees, Charlotte E. Shepherd, Maggie Chamberlain, Suzanne<br><br>2005	8	17	✓			UG	The construct validity of the criteria was established by exploring its relationship with two other assessment methods and its reliability was determined using the	-	Themes emerging from the focus groups include students preferring the structured nature of the portfolios but assessors feel- ing that this reduced the uniqueness of the portfolios. Although students understood the importance of reflective practice, some disliked the process of reflection, particularly

No.	Title	Author/year	MERSQI	COREQ	Reason for inclusion			Undergraduate (UG)/Postgraduate (PG)	Methodology	Purpose of study	Key findings
					Communication	Ethics	Professionalism				
									generalizability (G) coefficient. Focus groups with assessors and students were convened to explore their views of the portfolios. Two portfolio analyses had a G coefficient of .42. Performance in the portfolio analyses was correlated with performance in personal and professional development judgements (r = −.512, p < .01) and scientific reports (r = .273, p = .002).		reflective writing. Educators should design assessment criteria that maximize reliability, validity and acceptability rather than simply focusing on reliability alone.<br><br>Despite these methodological limitations, this study provides support for the reli- ability and validity of the portfolio analyses assessment criteria at Peninsula Medical School.
47	Reflections: an inquiry into medical students' professional identity formation	Wong, A.<br>Trollope-Kumar, K.<br><br>2014		14	✓	✓		UG	Sixty-five medical students (46 women; 19 men) from a class of 194 consented to the study of their portfolios. In total, 604 reflections were analysed and coded using thematic narrative analysis. The codes were merged under subthemes and themes. Common or recurrent themes were identified in order to develop a descriptive framework of professional identity formation. Reflections were then analysed	The purpose of this study was to understand the major influences on medical students' professional identity formation.	Five major themes were associated with professional identity formation in medi- cal students: prior experiences, role models, patient encounters, curriculum (formal and hidden) and societal expectations. Our longi- tudinal analysis shows how these themes inter- act and shape pivotal moments, as well as the iterative nature of professional identity from the multiple ways in which individuals con- struct meaning from interactions with their environments.<br><br>Our study provides a window on the dynamic, discursive and constructed nature of professional identity formation. The five key themes associated with professional identity formation provide strategic

No.	Title	Author/year	MERSQI	COREQ	Reason for inclusion			Undergraduate (UG)/Postgraduate (PG)	Methodology	Purpose of study	Key findings
					Communication	Ethics	Professionalism				
									longitudinally within and across individual portfolios to examine the professional identity formation over time with respect to these themes.		opportuni- ties to enable positive development. This study also illustrates the power of reflective writing for students and tutors in the professional identity formation process.
48	Content validity of workplace-based portfolios: A multi-centre study	Nele R.M. Michels, Marijke Avonts, Griet Peeraer, Kris Ulenaers, Luc F. Van Gaal, Leo L. Bossaert, Erik W. Driessen, Arno M.M. Muijtjens & Benedicte Y. De Winter<br><br>2016	9.5		✓		✓	UG	We reviewed 120 workplace portfolios at three medical universities (Belgium and the Netherlands). To validate their content, we developed a Validity Inventory for Portfolio Assessment (VIPA) based on the CanMEDS roles. Two raters evaluated each portfolio and indicated for each VIPA item whether the portfolio provided sufficient information to enable satisfactory assessment of the item. We ran a descriptive analysis on the validation data and computed Cohen's Kappa to investigate interrater agreement.	This study sought to evaluate the content validity of portfolios as reflected in their capacity to adequately assess achieved competences of medical students during clerkships.	The portfolios adequately covered the items pertaining to the communicator (90%) and professional (87%) roles. Coverage of the medical expert, collaborator, scholar and manager roles ranged between 75% and 85%. The health advocate role, covering 59%, was clearly less well represented. This role also exhibited little interrater agreement (Kappa50.4).<br><br>This study lends further credence to the evidence that portfolios can indeed adequately assess the different CanMEDS roles during clerkships, the health advocate role, which was less well represented in the portfolio content, excepted.
49	Does a summative portfolio foster the development of capabilities such	Anthony J O'sullivan, Amanda C Howe, Susan Miles, Peter Harris, Chris S Hughes, Philip Jones, Helen	7.5		✓	✓	✓	UG	A questionnaire was designed to evaluate undergraduate medical students' experiences of	The aims of this study were (i) to determine whether preparing a portfolio helps promote students'	Students perceive portfolio preparation as an effective learning tool for the development of capabilities such as understanding ethical and legal principles

For peer review only

No.	Title	Author/year	MERSQI	COREQ	Reason for inclusion			Undergraduate (UG)/Postgraduate (PG)	Methodology	Purpose of study	Key findings
					Communication	Ethics	Professionalism				
	as reflective practice and understanding ethics? An evaluation from two medical schools	Scicluna & Sam J Leinster<br><br>2012							completing a portfolio at two medical schools. A total of 526 (45% response rate) students answered the on-line questionnaire. Students from both medical schools gave the highest ranking for the portfolio as a trigger for reflective practice. 63% of students agreed their portfolio helped them develop reflective practice skills (p < 0.001), whereas only 22% disagreed. 48% of students agreed portfolios helped them understand ethical and legal principles whereas 29% disagreed (p < 0.001). In contrast, only 34% of students thought the portfolio helped them to develop effective communication.	development in a range of capabilities including understanding ethical and legal principles, reflective practice and effective communication, and (ii) to determine to what extent the format of the portfolio affected the outcome by comparing the experiences of students at two different medical schools.<br><br>Therefore, the primary aim of this study was to determine whether the process of preparing a portfolio report or essay helped students appreciate and develop in a range of capabilities, such as understanding ethical and legal principles and reflective practice; capabilities which are not the focus of traditional assessments such as clinical or written examina- tions. The format of the portfolio assessment was different in each of the two medical schools in the study, and so a secondary aim was to determine to what extent the format of the assessment affected the outcome by comparing the student experiences with their portfolio	and reflective practice, whereas other capabilities such as effective communication require complementary techniques and other modes of assessment.

For peer review only

No.	Title	Author/year	MERSQI	COREQ	Reason for inclusion			Undergraduate (UG)/Postgraduate (PG)	Methodology	Purpose of study	Key findings
					Communication	Ethics	Professionalism				
										assessment in the two schools.	
50	Feasibility and Outcomes of Implementing a Portfolio Assessment System Alongside a Traditional Grading System	Celia Laird O'Brien, PhD, Sandra M. Sanguino, MD, MPH, John X. Thomas, PhD, and Marianne M. Green, MD<br><br>2016	7		✓		✓	UG	In 2009, the authors developed a portfolio system that served as a repository for all student assessments organized by competency domain. Five competencies were selected for a preclerkship summative portfolio review. Students submitted reflections on their performance. In 2014, four clinical faculty members participated in standard-setting activities and used expert judgment and holistic review to rate students' competency achievement as "progressing toward competence," "progressing toward competence with some concern," or "progressing toward competence pending remediation." Follow-up surveys measured students' and faculty members'	This study describes the development and implementation of a longitudinal competency-based electronic portfolio system alongside a graded curriculum at a relatively large U.S. medical school.	Faculty evaluated 156 portfolios and showed high levels of agreement in their ratings. The majority of students achieved the "progressing toward competence" benchmark in all competency areas. However, 31 students received at least one concerning rating, which was not reflected in their course grades. Students' perceptions of the system's ability to foster self-assessment were mixed.

For peer review only

No.	Title	Author/year	MERSQI	COREQ	Reason for inclusion			Undergraduate (UG)/Postgraduate (PG)	Methodology	Purpose of study	Key findings
					Communication	Ethics	Professionalism				
									perceptions of the process.		
51	Feelings related to first patient experiences in medical school A qualitative study on students' personal portfolios	Kaisu H. Pitkälä a,∗, Taina Mäntyranta b<br><br>2003? 2004		14	✓		✓	UG	Twenty-two volunteer third and fourth year medical students (15 women and 7 men) of the University of Helsinki participated in a portfolio course for 1 year. Their reflective learning diaries and writings on specific themes were analyzed by qualitative content analysis	Feelings and thoughts of medical students related to first patient experiences during the first clinical year were examined	First patient encoun- ters were strong emotional experiences for medical students. The first patient examination was often described as an anxiety-provoking and confusing incident. Other emotionally significant encounters included helplessness when faced with serious illness and death, and role confusion when examining patients of one's own age but opposite sex. Students felt guilty for using patients for their own learn- ing purposes. Portfolios as learning tools may help in recognizing key experiences and support professional development of medical students.
52	Linking assessment to undergraduate student capabilities through portfolio examination	Anthony J. O'Sullivan , Peter Harris , Chris S. Hughes , Susan M. Toohey , Chinthaka Balasooriya , Gary Velan , Rakesh K. Kumar & H. Patrick McNeil<br><br>2012	8.5		✓	✓	✓	UG	Students are required to complete assessments linked to graduate capabilities. In Year 3, a portfolio review occurs (205–248 students per year), focusing on students' grades and feedback from assessments and a reflective essay is submitted. In the essay, students reflect on their progress, identify areas of weakness and detail plans for improvement. Progress in each capability is summatively graded against	This article describes an e-portfolio which closely aligns learning and reflection to graduate capabilities, incorporating features that address concerns about portfolios	Portfolios are an established method of assessment, although concerns do exist around their validity for capabilities such as reflection and self-direction

For peer review only

No.	Title	Author/year	MERSQI	COREQ	Reason for inclusion			Undergraduate (UG)/Postgraduate (PG)	Methodology	Purpose of study	Key findings
					Communication	Ethics	Professionalism				
									specific criteria and feedback is provided.		
53	Medical student resilience strategies: A content analysis of medical students' portfolios	Richard A. Prayson · S. Beth Bierer · Elaine F. Dannefer<br><br>2016		12	✓	✓	✓	UG	We retrospectively reviewed a sampling of year 1, 2 and 5 portfolio essays focused on the Personal Develop- ment competency and performance milestones, written by 49 students from three different classes in a 5-year pro- gramme devoted to training physician investigators. Two medical educators used a framework established by Jensen and colleagues (2008) to identify the nature and prevalence of various resilience strategies (valuing the physician role, self-awareness, personal arena, professional arena, profes- sional support and personal support) medical students re- ported in portfolio essays.	A student's ability to employ resilience strategies to self-regulate behaviour is critical to the student's future career as a physician. (The aim is to examine this??)	All students documented at least one strategy in their essays each year. In all years, the most commonly documented strategies were in the personal arena (95.7% of year 1, 98% of year 2 and 87.8% of year 5 portfolios). The least frequently documented strategy in all years was professional support (42.8% of year 1, 38.8% of year 2, and 28.6% of year 5 portfolios). Year 5 portfolios discussed personal support strategies (79.6%) more frequently than year 1 (53.1%) and year 2 (59.2%) portfolios.<br><br>The results suggest that medical students can identify stressors and articulate resilience strategies that can be employed to potentially address them.
54	Portfolio Assessment during Medical Internships: How to Obtain a Reliable and Feasible	NRM Michels, EW Driessen, AMM Muijtjens, LF Van Gaal, LL Bossaert, BY De Winter<br><br>2009		9.5			✓	UG	The domain-oriented reliability of 61 double-rated portfolios was measured, using a generalisability	In this study, we investigated whether assessment during medical internship by a portfolio can combine reliability and feasibility.	We obtained reliability (Φ coefficient) of 0.87 with this internship portfolio containing 15 double-rated tasks. The generalisability analysis showed that an acceptable level of reliability

No.	Title	Author/year	MERSQI	COREQ	Reason for inclusion			Undergraduate (UG)/Postgraduate (PG)	Methodology	Purpose of study	Key findings
					Communication	Ethics	Professionalism				
	Assessment Procedure?								analysis with portfolio tasks and raters as sources of variation in measuring the performance of a student.		(Φ = 0.80) was maintained when the amount of portfolio tasks was decreased to 13 or 9 using one and two raters, respectively.<br><br>Our study shows that a portfolio can be a reliable method for the assessment of workplace learning. The possibility of reducing the amount of tasks or raters while maintaining a sufficient level of reliability suggests an increase in feasibility of portfolio use for both students and raters.
55	Twelve tips for introducing E-Portfolios with undergraduate students	Alis Moores & Maria Parks<br><br>2010	-	-	✓			UG	-	-	-
56	Use of Portfolios in Undergraduate Medical Training: First Meeting With a Patient	Nilgün ÖZÇAKAR, Vildan MEVSİM, Dilek GÜLDAL<br><br>2009		10	✓		✓	UG	We designed a portfolio that was aimed at making reflection in early undergraduate medical education. Conditions for portfolio use were identified through interviews with six trainers who were experienced in mentoring students in the process of developing their portfolios. Analysis of writings was per- formed using qualitative content analysis.	Portfolios, as learning tools, are becoming increasingly important in medical education. Our aim was to evaluate the contents of portfolios used in medical education.	The conditions for successful reflective use of portfolios that emerged from the interviews fell into catego- ries: practice evaluation, patient perspective and primary prevention/health promotion. According to the students, working with a portfolio designed to meet these conditions is very useful and will stimulate students' abilities. The stu- dents shared the same opinion that analyzing one's com- petences in a portfolio was instructive and meaningful.<br><br>According to our students, portfolios are a potentially valuable method of developing students' skills in undergraduate medical education, provided that certain conditions for effective portfolios are recognized and met. The portfolio is a worthwhile addition to existing

For peer review only

No.	Title	Author/year	MERSQI	COREQ	Reason for inclusion			Undergraduate (UG)/Postgraduate (PG)	Methodology	Purpose of study	Key findings
					Communication	Ethics	Professionalism				
											assessment and learning tools.
57	What Is the Relationship Between a Preclerkship Portfolio Review and Later Performance in Clerkships?	Celia Laird O’Brien, PhD, John X. Thomas Jr, PhD, and Marianne M. Green, MD<br><br>2018	9.5		✓		✓	UG	The authors divided students into two groups based on a summative preclerkship portfolio review in 2014: students who had concerning behavior in one or more competencies and students progressing satisfactorily. They compared how students in these groups later performed on two clerkship outcomes as of October 2015: final grades in required clerkships, and performance on a clerkship clinical composite score. They used Mann–Whitney tests and multiple linear regression to examine the relationship between portfolio review results and clerkship outcomes. They used USMLE Step 1 to control for knowledge acquisition.	This study examines whether a preclerkship portfolio review is a valid method of identifying problematic student behavior affecting later performance in clerkships.	Students with concerning behavior preclerkship received significantly lower clerkship grades than students progressing satisfactorily (P = .002). They also scored significantly lower on the clinical composite score (P < .001). Regression analysis indicated concerning behavior was associated with lower clinical composite scores, even after controlling for knowledge acquisition.<br><br>The results show a preclerkship portfolio review can identify behaviors that impact clerkship performance. A comprehensive portfolio system is a valid way to measure behavioral competencies.
58	Educating undergraduate medical students about oncology: A literature review	Cave, Judith<br><br>2006	-	-	✓			UG	MEDLINE, Psychinfo, ERIC, TIMELIT, EMBASE, CINAHL and the Cochrane CENTRAL Register of	This article is a review of the literature regarding teaching oncology to undergraduate medical students.	The main findings can be summarized as follows: the involvement of patients in teaching is popular with students and portfolio learning is a successful way of involving patients; the use of standardized patients to

For peer review only

No.	Title	Author/year	MERSQI	COREQ	Reason for inclusion: Communication	Reason for inclusion: Ethics	Reason for inclusion: Professionalism	Undergraduate (UG)/Postgraduate (PG)	Methodology	Purpose of study	Key findings
									Controlled Trials (CENTRAL) were searched, using the search terms cancer, oncology, education, undergraduate, and teaching.		teach breast examination improves students' performance in clinical assessment; the use of silicone models to teach breast examination improves students' sensitivity for detecting breast lumps; computer aided learning modules have a role, but are not superior to other types of learning; learning about cancer screening and prevention increases students' knowledge, improves their self rated skills, and changes their behavior; and cancer patients have an important role to play in teaching undergraduate communication skills.<br><br>We have found 48 articles on undergraduate teaching in oncology. Oncology teachers should consider adopting the evidence based approaches outlined in this review, and there should be more emphasis on educational research within the field of oncology.
59	Integrating the assessment of interprofessional education into the health care curriculum	Anderson, E. S. and Kinnair, D.<br><br>2016	-	-	✓	✓	✓	UG	-	Reflection on some of the challenges associated with assessment in IPE by describing the experiences at one UK-based medical school	In regard to written reflections, reading 240 student entries per learning event is time-consuming, making<br>it challenging to offer individual feedback. Some students have challenged the formative outcomes that were graded for progression across broad boundaries of unsatisfactory, satisfactory or excellent. Checking for consistency across a small numbers of marker illuminates the subjective perspectives and interpretations of individual

For peer review only

No.	Title	Author/year	MERSQI	COREQ	Reason for inclusion			Undergraduate (UG)/Postgraduate (PG)	Methodology	Purpose of study	Key findings
					Communication	Ethics	Professionalism				
											markers on what constitutes a meaningful reflection. Nevertheless, offering this early writing platform has proved to be extremely helpful and enables educators to determine if students can reflect critically and apply meaning
60	A model of engagement in reflective writing-based portfolios: Interactions between points of vulnerability and acts of adaptability	Arntfield, S., Parlett, B., Meston, C. N., Apramian, T. and Lingard, L.<br><br>2015	7	21			✓	UG	Using mixed-methods rooted in grounded theory, 139 students and 13 mentors completed questionnaires, 23 students participated in four focus groups and 9 mentors in individual interviews	The aim of this study was to explore conditions affecting the experience of teaching and learning from the perspective of both students and mentors in a reflective writing-based portfolio initiative.	The overarching theme in our data was student–mentor engagement. Our results confirm previous literature describing portfolio as a vulnerable method of learning, extend this concept by identifying and categorizing specific points of vulnerability, and contribute new knowledge regarding acts of adaptability, which serve to strengthen the student–mentor relationship.<br><br>Engagement is central to the success of portfolio and is shaped by a dynamic interaction between points of vulnerability and acts of adaptability. We propose a model of engagement in portfolio that can be used for faculty development to optimize student–mentor engagement.
61	Support for portfolio in the initial years of the undergraduate medical school curriculum: what do the tutors think?	Austin, C. and Braidman, I.<br><br>2008	7	18	✓		✓	UG	A mixed method approach was used with data collected from both questionnaires and a focus group meeting.	The aims of this study were to investigate the views of these tutor facilitators on the delivery and support of portfolios in this way.	Tutor facilitators were generally positive about their roles in the support of portfolio development in group sessions and identified several advantages to this type of tutoring – namely the value of group discussion and discussion between peers to encourage reflection, and the practical ability to integrate portfolio sessions more closely with clinical experiences – which would not be possible in one to one tutor/student meetings.

For peer review only

No.	Title	Author/year	MERSQI	COREQ	Reason for inclusion			Undergraduate (UG)/Postgraduate (PG)	Methodology	Purpose of study	Key findings
					Communication	Ethics	Professionalism				
											With the training and guidance notes provided tutors were confident that they understood the support they should give students and felt that they could encourage reflection.<br><br>Thus, tutors perceived that the support of portfolio in groups is an effective way of developing portfolios for large numbers of students.
62	Use of portfolios by medical students: significance of critical thinking	Azer, S. A.<br><br>2008	-	-	✓		✓	UG	-	The aims of this paper are to: (1) understand the uses of portfolios and the rationale for using reflection in the early years of a PBL curriculum; (2) discuss how to introduce portfolios and encourage students' critical thinking skills, not just reflection; and (53) provide students with tips that could enhance their skills in constructing good portfolios.	-
63	Time to Loosen the Apron Strings: Cohort-based Evaluation of a Learner-driven Remediation Model at One Medical School	Bierer, S. B., Dannefer, E. F. and Tetzlaff, J. E.<br><br>2015	10		✓			UG	We used a retrospective, cohort-based approach to examine our learner-driven remediation model.<br>Data sources: We consulted MSPRC records to identify all CCLCM students from six class cohorts (2009–2014) placed in remediation during medical school, and compared remedial students	To examine a remediation model where students, rather than faculty, develop remedial plans to improve performance.<br>We present an innovative, systematic approach to remediation purposefully designed to engage learners with the development and implementation of remedial plans to improve their performance. We first describe our approach to	From abstract -- participants included 177 students from six classes (2009–2014). Twenty-six were placed in remediation, with more referrals occurring during Years 1 or 2 ($n = 20$, 76 %). Unprofessional behavior represented the most common reason for referral in Years 3–5. Remedial students did not differ from classmates ($n = 151$) on baseline characteristics (Age, Gender, US citizenship, MCAT) or willingness to recommend their medical school to future students ($p < 0.05$). Two remedial students did not graduate and three did

For peer review only

No.	Title	Author/year	MERSQI	COREQ	Reason for inclusion			Undergraduate (UG)/Postgraduate (PG)	Methodology	Purpose of study	Key findings
					Communication	Ethics	Professionalism				
									to their classmates not placed in remediation on variables collected for program evaluation purposes: Baseline characteristics (gender, age, US citizenship, MCAT score), USMLE licensure exam performance (pass-rate for each student), National Residency Matching Program data (clinical specialty for each student), Satisfaction with medical school (collected from graduating students using questionnaire). Then, we read MSPRC correspondence to each remedial student to extract referral mechanism (portfolio-based decision or faculty-initiated), unsatisfactory competency domain(s), remediation duration (days), and completion of students' remediation goals. Finally, we read students' remedial plans to identify strategies they proposed to	remediation and then report outcomes for students who were and were not placed in remediation during medical school. We conclude with lessons learned and future directions.	not pass USLME licensure exams on first attempt. Most remedial students (92 %) generated appropriate plans to address performance deficits. Students can successfully design remedial interventions. This learner-driven remediation model promotes greater autonomy and reinforces self-regulated learning. From results: Participants include 177 of 187 medical students from six class cohorts (2009–2014) who consented (95 %) to release program evaluation data for research purposes. Of these, the MSPRC placed 26 students (17 men and nine women) in formal remediation. Half (13) were identified by the MSPRC for not meeting year-end performance standards assessed via portfolios. The dean of student affairs referred the remaining 13 students based on faculty recommendation. We did not detect significant differences between remedial students and their classmates for baseline characteristics (Table 1). Overall, three-quarters of remediation decisions occurred during the first (10, 38 %) or second (10, 38 %) year of medical school. With regard to specific domains in which students failed to meet year-end standards or were otherwise referred, professionalism (18, 69 %), communication (10, 38 %), research (9, 35 %), and reflective practice (5, 19 %) represent frequent domains of unsatisfactory performance. Fewer students did not meet

For peer review only

No.	Title	Author/year	MERSQI	COREQ	Reason for inclusion			Undergraduate (UG)/Postgraduate (PG)	Methodology	Purpose of study	Key findings
					Communication	Ethics	Professionalism				
									the MSPRC to address performance deficits in specific competency areas. We manually recorded a subset of these strategies to provide examples of students' approaches to remediation. All performance decisions, referral mechanisms, and outcomes were manually entered into SPSS. Chi-square and ANOVA tests were used to compare baseline characteristics and satisfaction scores of the two student groups (remediation vs. other), while the Mann Whitney U test was used to examine if remediation duration (days) differed by students' gender. Statistical significance was established at $p < 0.05$ for all comparisons.		performance standards for clinical reasoning (4, 15 %), medical knowledge (3, 11 %), clinical skills (2, 8 %), personal development (2, 8 %), or health care systems (2, 8 %). Unprofessional behavior (e.g., not logging patient encounters, unexcused absences, chronic tardiness, etc.) represented the most common reason for student remediation during Years 3–5 of medical school. Ten students had deficient performance in one competency, while others had unsatisfactory performance in two (7, 27 %) or more (9, 35 %) competencies. Three students were placed in remediation twice, with one repeating for unprofessional behavior. Time in remediation (determined by dates in MSPRC correspondence to students) ranged from 39 to 1696 days (Median = 448 days) and did not differ by student gender ($p > 0.05$). Remediation plans revealed various student-proposed strategies to address TAFIs for multiple competency domains (Table 2). We observed that students' remedial strategies resembled approaches faculty would recommend. We also observed the MSRPC's correspondence to students consistently had an encouraging, supportive tone for students' remedial efforts (See Appendices for MSPRC's correspondence to one remedial student where committee asks student to develop a remediation plan, comments

For peer review only - http://bmjopen.bmj.com/site/about/guidelines.xhtml

No.	Title	Author/year	MERSQI	COREQ	Reason for inclusion			Undergraduate (UG)/Postgraduate (PG)	Methodology	Purpose of study	Key findings
					Communication	Ethics	Professionalism				
											on student's progress report, and releases student from remediation). The MSPRC "prescribed" remediation plans for two students only after these students did not provide adequate assessment evidence in progress reports. The MSPRC determined that most students (24, 92 %) generated sufficient evidence (predominately obtained from faculty and peers) to document their competence. Table 1 reveals that some remedial students experienced negative outcomes, as two did not graduate and three did not pass USMLE licensure exams on first attempt. Proportionally, more remedial students (11, 46 %) pursued surgical specialties than their classmates (50, 33 %). Table 1 also shows graduating remedial students were just as likely as their classmates to recommend the CCLCM program to future students, even though fewer were as satisfied with their overall experience at CCLCM as their classmates. From discussion -- Most programs struggle with how to identify and assist learners requiring remediation. We explored if students, rather than faculty, can take ownership for remedial plan design and implementation. We review key findings and conclude with future directions. The locus of control for selecting remedial interventions and assessment evidence typically rests with faculty. In

For peer review only

No.	Title	Author/year	MERSQI	COREQ	Reason for inclusion			Undergraduate (UG)/Postgraduate (PG)	Methodology	Purpose of study	Key findings
					Communication	Ethics	Professionalism				
											our model, the MSPRC charged students to develop remediation plans, with PA guidance as needed, and obtain assessment evidence to document progress and achievement. Most remediating students (92 %) successfully met these expectations and graduated from medical school. Three-quarters of remedial students were identified in Years 1–2 of medical school. Early identification provides opportunities for multiple, tailored interventions to occur. Professionalism, communication skills, and research performance were frequent reasons the MSPRC placed students in remediation. Few students were identified for gaps in medical knowledge or clinical reasoning. The literature suggests some competencies, like professionalism, require direct observation and ongoing assessment for learner success.1,18 Our students' remedial strategies (Table 2) ranged from specific to holistic approaches and often cut across courses and contexts, thereby providing them with more opportunities to improve and document their performance in complex domains. This emphasis on competence may explain why students' time in remediation was longer (Median = 448 days) than normally needed for "more of the same," such as retaking a test or course.2 Some learners may not know how to navigate performance obstacles,

For peer review only

No.	Title	Author/year	MERSQI	COREQ	Reason for inclusion			Undergraduate (UG)/Postgraduate (PG)	Methodology	Purpose of study	Key findings
					Communication	Ethics	Professionalism				
											making it important to offer guidance and oversight.19,20 For this reason, we train a core group of faculty to help students apply the self-regulation cycle.17 PAs coach students in reflection, self-assessment, and goal-setting.2,20,21 They also form long-term relationships with students and frequently serve as student advocates. The MSPRC encourages students to identify measurable outcomes to document competence, while offering positive support in written correspondence to students (See Appendices for series of letters to one student throughout the remediation process). Both faculty groups work in tandem to support this learner-driven remediation model while ensuring student accountability. Our evaluation is limited to one program, at one school, with a small class size. Other programs may foster remedial learners' autonomy more than conveyed in the literature.5,6,8 Our student satisfaction measures originate from two questionnaire items obtained at graduation and do not capture students' satisfaction with specific academic referral policies or remedial processes.22 Remediation is complex and involves multiple stakeholders and contexts. Future studies should capture learners' perceptions of remediation to complement the faculty point-of-view currently reported in the literature.

For peer review only

No.	Title	Author/year	MERSQI	COREQ	Reason for inclusion			Undergraduate (UG)/Postgraduate (PG)	Methodology	Purpose of study	Key findings
					Communication	Ethics	Professionalism				
											We discovered that more men than women were identified for remediation, yet we found no research exploring this observation in depth. Additionally, few longitudinal or multi-institutional studies currently exist, suggesting an area ripe for research. We discovered, in our 10-year experience with this remediation model, that medical students can select appropriate remedial interventions with the support of well-trained faculty. Our learner-driven approach to remediation may transfer to graduate medical education (GME), where Clinical Competence Committees (CCCs) meet regularly to discuss and review resident performance. Having struggling residents create remediation plans and submit progress reports to CCCs may present few barriers, given GME's competency-based assessment framework and emphasis on providing residents with frequent, formative feedback. Our approach requires a learning environment where faculty must trust students have the motivation and capabilities to identify and implement appropriate remedial strategies. We have created a systematic process where students must reflect on their performance, develop plans tailored to meet learning needs, and collect assessment evidence to document competence. To conclude, we believe educators who wish to

For peer review only

No.	Title	Author/year	MERSQI	COREQ	Reason for inclusion			Undergraduate (UG)/Postgraduate (PG)	Methodology	Purpose of study	Key findings
					Communication	Ethics	Professionalism				
											adopt a learner-driven approach to remediation should view remediation as a growth opportunity rather than as a punishment.
64	Medical professionalism: conflicting values for tomorrow's doctors	Borgstrom, E., Cohn, S. and Barclay, S.<br><br>2010		14		✓		UG	From abstract: Qualitative study using interpretative discourse analysis of anonymized student reflective portfolios. One hundred twenty-three final year undergraduate medical students (64 male and 59 female) from the University of Cambridge School of Clinical Medicine supplied 116 portfolios from general practice and 118 from hospital settings about patients receiving palliative or end of life care.<br>From introduction: This study investigates how professionalism is understood and experienced via one such initiative for medical students training in Cambridge, England, and highlights the conflicts between 'new' and 'old' values.<br>From methods: Final year medical students in Cambridge meet patients approaching the	To investigate how final year medical students experience and interpret new values of professionalism as they emerge in relation to confronting dying patients and as they potentially conflict with older values that emerge through hidden dimensions of the curriculum.	From abstract -- Results: Professional values were prevalent in all the portfolios. Students emphasised patient-centered, holistic care, synonymous with a more contemporary idea of professionalism, in conjunction with values associated with the 'old' model of professionalism that had not be directly taught to them. Integrating 'new' professional values was at times problematic. Three main areas of potential conflict were identified: ethical considerations, doctor-patient interaction and subjective boundaries. Students explicitly and implicitly discussed several tensions and described strategies to resolve them. Conclusions: The conflicts outlined arise from the mix of values associated with different models of professionalism. Analysis indicates that 'new' models are not simply replacing existing elements. Whilst this analysis is of accounts from students within one UK medical school, the experience of conflict between different notions of professionalism and the three broad domains in which this conflict arises are relevant in other areas of medicine and in different national contexts.<br>From discussion -- All of the students encountered challenges that required

For peer review only

No.	Title	Author/year	MERSQI	COREQ	Reason for inclusion			Undergraduate (UG)/Postgraduate (PG)	Methodology	Purpose of study	Key findings
					Communication	Ethics	Professionalism				
									end of life during general practice (GP) and hospital attachments. As required coursework they write portfolio items on two patients, one from each attachment. Key to this exercise is integrating issues arising from the interview with reflections of their own personal experiences. This study focuses on how different ideas are received and reproduced by a relatively young (typically early 20s) cohort of soon-to-be qualified doctors during the academic year 2007–2008. The University of Cambridge Psychology Research Ethics committee approved the study, and 86% of students (n = 123; 64 male, 59 female) gave informed consent for 234 reflective items: 116 from the GP and 118 from hospital settings. Each student was assigned a random identification number (ID), which is included		them to try and balance values characteristics of 'old' and 'new' forms of medical professionalism. Ethics, interaction and managing subjective boundaries all generated areas of conflict that they wrestled with in their assignments. Students addressed these tensions in a variety of ways, with strategies drawn from both formally taught skills and personal resources. In parallel with shifts from 'old' to 'new' values of professionalism, conflicts between the formal and the hidden curricula may also generate tensions44. In our study, the formal curricula is explicitly designed to embrace the 'new', leaving the 'old' to be communicated through more informal and hidden aspects of teaching. Yet for the students trying to personally reflect on their experiences and summatively draw from their entire educational experience for the exercise, no distinction is made between these two domains. Large organisations like the University of Cambridge Clinical School and the National Health Service have institutional memories45—collective experiences and concepts. These are communicated imperceptively through teaching elements as well as the very structures and policies that shape the educational environment. This feature serves to complicate understandings of medical education; it

For peer review only - http://bmjopen.bmj.com/site/about/guidelines.xhtml

No.	Title	Author/year	MERSQI	COREQ	Reason for inclusion			Undergraduate (UG)/Postgraduate (PG)	Methodology	Purpose of study	Key findings
					Communication	Ethics	Professionalism				
									with the quotes to demonstrate the breadth of the dataset presented. Analysis focused on the topic of 'professionalism', viewing the content of the portfolios as representative of general values imparted to students throughout their education. All items were coded and analysed in NVivo 8 using an interpretative approach. Following the social science traditions of two of the authors (EB and SC), the methodology employed a hermeneutic approach, striving to find meaning beyond straightforward discrete references35. Accordingly, rather than assuming the meaning of isolated sections of text in a reductive manner, they were always analysed in context and in relation to one another. Given the difficulty many students had in addressing the topic of end of life care, and		emphasises the extent to which values are embedded in the routines and practices of the organisation, and cannot therefore be simply addressed through redesigning curricula or individuals championing change. Our study suggests that tensions arise primarily because of different values that underlie the concept of professionalism throughout their education, rather than anything that is specific to end of life care. Whilst topics central to the everyday practice of medicine are particularly evident in palliative care46, they are made highly visible by students who see themselves on the cusp of becoming members of the profession themselves. Whilst it is likely that the concept of professionalism always has contained a wide variety of underlying values and principles that are not always commensurate with each other, we have argued that a more widespread shift in values over recent years has generated greater variance and hence more contradictory positions around what it means to be a good doctor. Our findings add to the literature on medical professionalism, which is rich in doctors' anecdotal experiences47,48, highlights the stresses and conflicts doctors face44,49,50 and illustrates a current amalgamation of definitions with conflicting values51. Specific national and local contexts are likely to generate differently

For peer review only

No.	Title	Author/year	MERSQI	COREQ	Reason for inclusion			Undergraduate (UG)/Postgraduate (PG)	Methodology	Purpose of study	Key findings
					Communication	Ethics	Professionalism				
									consequently the very discursive descriptions of their observations, the authors were committed to code more than just those phrases in which a theme was referred to explicitly, and so included concerns that were expressed less directly. A coding scheme was derived from initial independent item analysis (EB, SC and SB) and adjusted through discussion. It was initially applicable to explicit statements by drawing on key words and then was augmented to include the more implicit references that potentially alluded more to the hidden dimensions of their teaching (see Box 2). The inclusion of these required careful interpretative readings of the entire text but provided an essential contribution to our overall analysis. The first author (193) then led on-going analysis, with regular meetings to		nuanced versions of these issues, which might only be identified through comparative work. From conclusion: The integration of 'new' professional values taught in medical school is at times problematic for students in any health care system that maintains elements, whether overtly or not, of the 'old' paradigm. The areas of potential conflicts outlined—ethics, patient interaction and managing subjective boundaries—are not limited to medical school or end of life care. Our analysis suggests that 'old' variants of professionalism are not simply being replaced by 'new' ones delivered by a redesigned curriculum, but rather that values from each can emerge in a range of medical contexts. As a result, professionalism does not consist of a set of fixed or abstract concepts, but rather surfaces through medical practice. If individual reflection is now heralded as an essential component of the 'new' professionalism, as indicated by the compulsory student exercise we have drawn on here, it should be acknowledged that it demands a dynamic engagement with the wide range of often contradictory and shifting ideas and beliefs from both formal and more hidden aspects of their education. This study illustrates that overt commitment to more empathic and patient-centered approaches to medical care do not

For peer review only

No.	Title	Author/year	MERSQI	COREQ	Reason for inclusion			Undergraduate (UG)/Postgraduate (PG)	Methodology	Purpose of study	Key findings
					Communication	Ethics	Professionalism				
									discuss emerging themes.		necessarily replace other more prescribed values and behaviours that remain part of a hidden curriculum embedded in institutional practices. Integration of 'new' core values and skills into good medical practice is not a smooth or simple transition. Additionally, it seems any simple attempt to communicate them through formal teaching is unlikely to prepare students for the reality of medical encounters. Instead, the experience of tension and the individual desire to seek balance and resolution across a wide range of issues may themselves be key and lasting features of medical professionalism.s
65	Facilitating students' reflective practice in a medical course: literature review	Chaffey, L. J., de Leeuw, E. J. and Finnigan, G. A.<br><br>2012		12			✓	UG	The literature search was undertaken using the online databases of CINAHL and Medline. Keyword searching was conducted using MeSH headings, Boolean operators, and the terms, 'medical students', 'medical education', 'reflection', 'reflect*' and 'medicine'. The search was limited to peer-reviewed published material in English and between the years 2001 and 2011 to maintain currency of	The aim of this review is to summarise the literature published around facilitating reflection in a medical course, and to answer the question : What is the current evidence regarding learning and development moments across the medical curriculum in developing students' reflective practice?	From abstract -- Discussion: A variety of reflective purposes was found in this literature review. Evidence indicates that, if students are unclear as to the purpose of reflection and do not see educators modelling reflective behaviours, they are likely to undervalue this important skill regardless of the associated learning and development opportunities embedded in the curriculum. From results: Thirty-six relevant articles were found. These articles were classified by methodology (see [Table 1]. Relevant references identified). As can be seen in [Table 1], this review included many opinion pieces, reviews and descriptions of methods of facilitating reflection, but little in regards to primary data in the area. Qualitative methodology was the main research output, providing largely descriptive data of

No.	Title	Author/year	MERSQI	COREQ	Reason for inclusion			Undergraduate (UG)/Postgraduate (PG)	Methodology	Purpose of study	Key findings
					Communication	Ethics	Professionalism				
									practice. The primary search yielded 129 references of eligible papers, including research papers, reviews, formal reviews and opinion pieces. Abstracts were read by the first author, and only articles pertaining to facilitating or teaching reflection in undergraduate or postgraduate medical courses were included. The first author read and classed all included articles, categorising them into construct sub-categories. At several stages in this process all authors reviewed the validity and legitimacy of the classifications. This led to the sub-headings and analysis presented below.		the facilitation of reflection. The quantitative research was also largely descriptive, with survey methodology being the most popular in the articles found. This review found only two case-control studies comparing facilitation methods of reflection. The literature presented enhancing factors and barriers to effectively teaching reflective practice within medical curricula, related to: The breadth of the meaning of reflection; Facilitating reflection by medical educators; Using written or web-based portfolios to facilitate reflection; and Assessing the reflective work of students.<br>- The breadth of the meaning of reflection: The term 'reflection' is widely mentioned within medical education literature, but the purposes and goals described indicate a breadth of purposes of reflection. [14] Mann et al.[10] concluded from their systematic review of reflective practice in the health professions that the nature of reflection makes it difficult to quantify. From this current review, we determined that the breadth of reflection covers the purposes of reflecting to improve judgements, to personally develop and, and to contextualise practice, and are explained in [Table 2]. Reflection to improve judgement centres on improving students' clinical skills, problem solving, [11] flexible thinking, lifelong learning and the development of expertise.

No.	Title	Author/year	MERSQI	COREQ	Reason for inclusion			Undergraduate (UG)/Postgraduate (PG)	Methodology	Purpose of study	Key findings
					Communication	Ethics	Professionalism				
											[1],[10],[14],[17] Reflection for this purpose usually has a problem focus, involves critical thinking, and is commonly indicated by hypothesis testing. Reflection to personally develop is essential in client-centred practice, as doctors need to consider they own values and assumptions, and those of the patient, while making clinical decisions. [18] From reviewing the literature, Sandars [14] asserted that the knowledge of one's values and beliefs is important for a doctor to develop therapeutic relationships with patients, as understanding oneself enables empathy and caring. Aukes et al.[11] reviewed literature in preparation for creating an assessment tool for reflection, and noted that personal reflection has often been overlooked in medical education. They suggested that this has occurred as personal development and has been seen as outside the domain of the problem-solving culture of medical practice, and thus, outside the domain of medical education. Reflection to contextualise practice helps students integrate theory with their own experience. Several authors have hypothesised that this aspect of reflection is essential in developing a sound medical practice, [5] with a lack of reflective ability resulting in poor self-awareness, and thus poor medical practice. [19] Howe et al.[8] found, from their study of the development of

For peer review only

No.	Title	Author/year	MERSQI	COREQ	Reason for inclusion			Undergraduate (UG)/Postgraduate (PG)	Methodology	Purpose of study	Key findings
					Communication	Ethics	Professionalism				
											professionalism, that reflecting on practice and developing self-awareness are important mechanisms of resilience in the face of a demanding profession. Although this breadth of reflective practice makes it an essential skill in medical practice, this very strength can also be a barrier in teaching facilitating reflective practice as there is often ambiguity of the educator's goals in regard to the use of a particular reflective task. [20],[21] Aukes et al.[11] noted that purposes of a reflective tasks were rarely articulated, and students were often unaware of the perspective, reflective purpose or role, they were expected to take. Sanders [14] concluded from a literature review of reflection in medical education that, for students to reflect effectively, they required clarity in determining the appropriate purpose of a reflective task.<br>- Facilitation reflection by medical educators:<br>Reflection is often provoked by uncertainty or unease resulting from an experience. [13],[22] According to Albanese, [22] in addition to this uncertainty or unease, a student requires knowledge of how to reflect, and time and motivation for reflection to occur. Supporting this opinion, Carr and Carmody [23] found in their qualitative study that some students could attain an acceptable level of reflection independently. However, reflection was greatly

For peer review only

No.	Title	Author/year	MERSQI	COREQ	Reason for inclusion			Undergraduate (UG)/Postgraduate (PG)	Methodology	Purpose of study	Key findings
					Communication	Ethics	Professionalism				
											enhanced through medical educators' facilitation. Medical educators' facilitation was found to be useful for reflection in one-on-one meetings with students and in small group tutorials. [9],[24] According to results of a study by Schaub-de Jong et al., [9] educators who wished to encourage reflection needed to model reflective practice by making their experiences more explicit, investigating emotions and stimulating interaction among students. Albanese [22] suggested that medical faculty staff should be trained to articulate their reflections to enhance modelling to students.<br>- Using written or web-based portfolios to facilitate reflection: Traditionally, reflective tasks have been paper based. Recently, web-based methods and social media have been adopted to facilitate reflective skills. Cook [29] asserted that there has been little study of the efficacy of these methods. Fischer et al. [30] compared content, depth of reflection and student preference for web-based versus traditional essay-style reflections in two medical schools in the USA. Ninety-five students were quasi-randomly assigned to one of two study groups, where one group wrote a traditional reflective essay and attended a small-group discussion, while the other group posted two writings in a group blog and commented on a peer's post. The writings were

For peer review only

No.	Title	Author/year	MERSQI	COREQ	Reason for inclusion			Undergraduate (UG)/Postgraduate (PG)	Methodology	Purpose of study	Key findings
					Communication	Ethics	Professionalism				
											coded for theme, and level of reflection, with the authors finding no difference between groups for either variable. From discussion: Traditionally, paper-based methods of reflection have been popular with educators, [7],[8],[25] but recently, web-based methods have been adopted. Driessen et al. [36] found that the method of reflection appears to be less important than the guidelines provided, which should be structured initially, and become more fluid as students' reflective skills progress. Ideally, students should be able to revisit and edit the reflection, hence web-based methods may be suitable for this purpose. - Assessing the reflective work of students: ... (see relevant columns) From discussion: Several authors noted that reflection was an essential skill in medical practice, [2],[5],[7],[10],[14],[34] and advocated for training in reflective processes. [13],[22] Training in reflective practice can be significantly enhanced with the facilitation of skilled mentors, providing guidance and modelling of reflective behaviours, as found by Carr and Carmody. [23] Several studies and literature reviews report inconsistencies regarding the purpose of reflection, which was found to be a significant barrier to the facilitation of reflective practice. [11],[20],[21],[35] The implication for educators is that students

For peer review only

No.	Title	Author/year	MERSQI	COREQ	Reason for inclusion			Undergraduate (UG)/Postgraduate (PG)	Methodology	Purpose of study	Key findings
					Communication	Ethics	Professionalism				
											need to be given clear guidelines as to the purpose for each individual reflective task. Traditionally, paper-based methods of reflection have been popular with educators, [7],[8],[25] but recently, web-based methods have been adopted. Driessen et al. [36] found that the method of reflection appears to be less important than the guidelines provided, which should be structured initially, and become more fluid as students' reflective skills progress. Ideally, students should be able to revisit and edit the reflection, hence web-based methods may be suitable for this purpose. Students need to have the designated time and motivation for reflective practice. A common method of increasing task motivation in the education setting is to include the task as an assessment piece. There was some debate in the literature about the efficacy of this method. Some authors found that students' quality of reflection would be diminished if undertaken for an assessment task, [28] whereas others reported that reflection simply would not happen if not attached to an assessable piece of work. [22],[33] It is apparent from this review that the research surrounding the teaching and facilitation of reflection is generally weak. Although there are some descriptive data, there is very little empirical evidence of the efficacy of facilitation methods. Additionally, there

For peer review only

No.	Title	Author/year	MERSQI	COREQ	Reason for inclusion			Undergraduate (UG)/Postgraduate (PG)	Methodology	Purpose of study	Key findings
					Communication	Ethics	Professionalism				
											is a lack of rigorous research investigating the impact of reflection on practice skills or patient outcomes. A major limitation of literature is the breadth of the construct of reflection. A more defined construct of reflection, with clear outcomes, could lead to the development of benchmarks useful in tracking student progress and as research outcome measures. From conclusion: Most authors cited in this review suggested that the emphasis on reflection is increasing in medical curricula. The construct of reflection was broad in the reviewed literature. This suggests that medical educators must be clear on the purpose of reflection, the intended outcome for the individual student and the process necessary to achieve these aims for students. Evidence indicated that, if students are unclear as to the purpose of reflection and do not see educators modelling reflective behaviours, they are likely to undervalue this skill regardless of the associated learning and development opportunities embedded in the curriculum.[40]
66	Beyond assessment of learning toward assessment for learning: educating tomorrow's physicians	Dannefer, E. F. 2013	-	-	✓			UG	From abstract: Information-rich assessment data, structured opportunities for reflection, and facilitated self-assessment using a portfolio approach are designed to support	From abstract: We describe a program (based at Cleveland Clinic Lerner College of Medicine) in which explicit performance standards align these components and provide a roadmap for students to manage their learning.	From abstract: Preliminary evidence suggests that the program directs students towards learning, rather than on achieving a grade for grade's sake.

No.	Title	Author/year	MERSQI	COREQ	Reason for inclusion			Undergraduate (UG)/Postgraduate (PG)	Methodology	Purpose of study	Key findings
					Communication	Ethics	Professionalism				
									development of habits of reflective practice. Promotion depends on the achievement of competencies rather than grades. From the essential building blocks: All components and processes of a system need to be designed to interact in a way that accomplishes the goal of enhancing learning and habits of reflective practice. Key features of the CCLCM assessment system are now presented by addressing issues of competency standards, ongoing formative assessment, student responsibility, and purposeful support.		
67	Evidence within a portfolio-based assessment program: what do medical students select to document their performance?	Dannefer, E. F., Bierer, S. B. and Gladding, S. P.<br><br>2012	7		✓		✓	UG	From abstract: We reviewed portfolios to examine the number, type, and source of assessments selected by students (n = 32) to document their performance in seven competencies. The quality of assessment data selected for each	From abstract: In this article, we examine the nature of evidence selected by first-year medical students to include in a portfolio used to make promotion decisions. From introduction: This article attempts to address this gap in the literature by examining the types and sources of assessment	From abstract -- Results: Findings indicate that students cited multiple types and sources of available assessments. The promotion committee rated evidence quality highest for competencies where the program provided sufficient evidence for students to cite a broad range of assessments. When assessments were not provided by the program, students cited self-generated evidence.

For peer review only

No.	Title	Author/year	MERSQI	COREQ	Reason for inclusion			Undergraduate (UG)/Postgraduate (PG)	Methodology	Purpose of study	Key findings
					Communication	Ethics	Professionalism				
									competency was rated by promotion committee members (n = 14). From methods: We used two procedures to explore the assessment evidence that one cohort of medical students cited in their Year 1 summative portfolios. First, we examined the evidence cited in portfolio essays to ascertain what types and sources were selected. Second, we obtained feedback from promotion committee members concerning the quality of evidence via a questionnaire. - Citation coding procedures and data: A research assistant extracted essays from the e-Portfolio database for seven of the nine competencies included in the Year 1 summative portfolios that students submitted during the 2009–2010 academic year. Essays for the competencies of Personal Development and	evidence first-year medical students select from their performance database to construct portfolios used for promotion decisions. We describe our assessment program's evidence database before presenting the organizational elements designed to help students purposefully select data representative of their performance. We then describe the methods and results of the study before discussing implications about students' ability to select evidence within a centralized assessment program.	Conclusion: We found that when student-constructed portfolios are part of an overall assessment system, students generally select evidence in proportion to the number and types of assessments available.

For peer review only

No.	Title	Author/year	MERSQI	COREQ	Reason for inclusion			Undergraduate (UG)/Postgraduate (PG)	Methodology	Purpose of study	Key findings
					Communication	Ethics	Professionalism				
									Reflective Practice were excluded to ensure student confidentiality. Students' names on essays were replaced with a unique six-digit identifier. Overall, 224 essays for 32 students were reviewed for these seven competencies. Sophia Gladding sorted the essays by competency. She then read each set of essays nested within a given competency and manually recorded on a data extraction form all college-generated assessments, work samples and personal evidence students cited to document their performance of Year 1 standards. Extraction form data were entered into SPSS version 16.0, and descriptive statistics were computed after averaging across students' portfolios.<br>- Quality ratings: Promotions committee members involved with portfolio reviews (n = 18 faculty)		

For peer review only

No.	Title	Author/year	MERSQI	COREQ	Reason for inclusion			Undergraduate (UG)/Postgraduate (PG)	Methodology	Purpose of study	Key findings
					Communication	Ethics	Professionalism				
									completed a 21-item, web-based questionnaire after reading students' Year 1 summative portfolios. The questionnaire had been designed (Beth Bierer and Elaine Dannefer) and piloted earlier to monitor the Year 1 portfolio review process. This instrument included seven Likert-scaled items ranging from 1 "poor" to 4 "excellent" which asked promotion committee members to rate the overall quality of the assessment evidence they read for each competency after judging students' summative portfolios. Percentages were computed for each competency to summarize the committee's global ratings ("excellent" and "good/excellent") of evidence quality.		
68	The portfolio approach to competency-based assessment at the Cleveland Clinic Lerner College of Medicine	Dannefer, E. F. and Henson, L. C. 2007	-	-	✓		✓	UG	-	From abstract: This article describes how the portfolio was developed to provide both formative and summative assessment of student achievement in relation to the program's nine	Design of the portfolio assessment system: The search for an assessment system consistent with our principles led us to the literature on portfolios for medical student assessment14 –15,21–23 and to firsthand investigation of experiences

For peer review only

No.	Title	Author/year	MERSQI	COREQ	Reason for inclusion			Undergraduate (UG)/Postgraduate (PG)	Methodology	Purpose of study	Key findings
					Communication	Ethics	Professionalism				
										competencies. From introduction: In this article we contribute to the literature on competency-based assessment by describing a portfolio assessment system developed as an integral component of the new Cleveland Clinic Lerner College of Medicine (CCLCM) of Case Western Reserve University In this article, we describe the design of CCLCM's portfolio approach for a comprehensive, competency-based assessment system that is fully integrated with the curriculum. To provide context, we describe the program's competencies, performance standards, core assessment principles, and unique assessment instrument before addressing the design of the overall assessment system.	with portfolio assessment within and beyond national, disciplinary, and professional borders. At the time (2002– 2003), the portfolio assessment approach was being implemented in the United States in grades K–12 and at the undergraduate college level24 –26 and in the United Kingdom and Europe at a number of medical schools. For example, at the University of Dundee College of Medicine, as a key component of the final examination process, each final-year medical student was required to submit a portfolio documenting his or her progress in achieving the outcomes of the curriculum.14,15 The first-year medical student portfolio at the University of Maastricht Faculty of Medicine included students' reflective essays on their own development in four identified roles of the doctor as a method of encouraging integration of competencies.22 An Answer to the Challenges of Competency-Based Assessment: In our opinion, this system and its components should be applied in other settings across the medical education continuum because major themes in the current debate about assessment in medical education, such as integration across competencies, competency-based assessment methods, sampling from multiple contexts and multiple sources,

For peer review only

No.	Title	Author/year	MERSQI	COREQ	Reason for inclusion			Undergraduate (UG)/Postgraduate (PG)	Methodology	Purpose of study	Key findings
					Communication	Ethics	Professionalism				
											triangulation of information, and training of learners in reflective practice are addressed in this innovative method of student assessment.
69	Supporting students in self-regulation: use of formative feedback and portfolios in a problem-based learning setting	Dannefer, E. F. and Prayson, R. A.<br><br>2013		11	✓		✓	UG	From abstract: Formative feedback on works habits and interpersonal skills provided by peers and tutors to a Year 1 cohort (n 1⁄4 32) over the course of a year-long PBL experience (5 blocks) was examined for comments on targeted areas for improvement (TAFIs) and observed improvements. We examined congruence between PBL feedback and students' self-reported TAFIs and behavioral improvements in their assessment portfolios.<br>From study design and methods:<br>- Study design: This study presents an analysis of (1) narrative feedback on professionalism provided to Year 1 medical students from PBL peers, tutors, and self and (2) summative portfolio essays to	From abstract: This study examines the extent to which students self-regulate professionalism behaviors related to work habits and interpersonal skills in a PBL setting.<br>From introduction: In 2004, Cleveland Clinic Lerner College of Medicine (CCLCM) implemented a program designed specifically to promote the development of self-regulated learners (Dannefer & Henson 2007). ... We confront the question of whether or not students use CCLCM's learning environment to self-regulate performance by focusing on the core competency of professionalism.<br>We selected for this study of SR the content of PBL feedback on professionalism and its use by students to identify learning needs (self-assess) and document improvement (regulate). We asked these questions:<br>(1) To what extent did the content of peer and tutor PBL	From abstract -- Results: Both PBL peer and faculty feedback and portfolio self-assessments targeted Interpersonal Skills TAFIs more frequently than Work Habit-related issues. TAFIs were more frequently identified midway in PBL blocks versus the end. Students reported TAFIs in their portfolio essays, citing feedback from both peers and tutors, and provided evidence of improved performance over time.; Conclusions: Students utilized external formative feedback to document their portfolio self-assessment in a system designed to support self-regulation of PBL professionalism-related behaviors. A decrease in TAFIs identified at the end of PBL blocks suggests students made use of mid-block feedback to self-regulate behaviors.<br>From results:<br>- (1) To what extent did the content of peer and tutor PBL narrative feedback provide information relevant to the professionalism competency standards? -- Table 1 summarizes the content of aggregate PBL tutor and peer feedback as well as PBL self and portfolio self- assessments. Of all TAFIs identified by tutors, Interpersonal Skills account for 63%. Interpersonal Skills also dominated peer statements (57%). The majority of tutor

For peer review only

No.	Title	Author/year	MERSQI	COREQ	Reason for inclusion			Undergraduate (UG)/Postgraduate (PG)	Methodology	Purpose of study	Key findings
					Communication	Ethics	Professionalism				
									determine how students used PBL feedback to support their self-assessments. We limited the study to year one the time period reported by students, and supported by advisors' observations, as requiring significant adjustments in terms of taking responsibility for their own learning.<br>- Measures: Faculty defined measurable, behavioral competency standards developmentally appropriate for Year 1 students and observ- able across multiple settings. ... Self-regulation, as a process, consists of overlapping and recursive activities of which we targeted self-assessments that identified areas needing improvement and cited evidence that documented improved performance.<br>- Data sources: We collected peer and tutor PBL assessments electronically at mid and end of Block 1 problem-	narrative feedback provide information relevant to the professionalism competency standards?<br>(2) To what extent did students self-report and document professionalism learning needs and improvements consistent with peer and tutor PBL feedback in a portfolio used for promotion decisions?	and peer TAFI statements about Interpersonal Skills indicated a concern with ''quietness'' (e.g. lack of participation). In the area of Work Habits, peers more frequently identified ''preparedness'' (e.g. disorganized presentations) as a TAFI in fellow students as compared with tutors, who more frequently documented behaviors associated with being ''distracted'' (e.g. carrying on side conversations). Peers were also more likely to give feedback on TAFIs than tutors; of 2016 peer assessments, 41% contained feedback on TAFIs. Overall, tutor feedback tended to be less detailed and only 28% of the 288 possible assessments contained feedback on TAFIs. Similar to tutor and peer evaluations, students self- identified Interpersonal Skills issues as TAFIs more often than Work Habit-related issues (60% vs. 40% respectively). Also similar to peer assessments, students frequently self-identified ''ill-preparedness'' as the Work Habit most needing improvement. In contrast to peer and tutor feedback, however, students more frequently self-identified ''dominating'' behavior issues rather than ''quietness''. All students, except for one who did not complete self-assessments, self-identified TAFIs also reported by their peers and tutors. Interestingly, 95% of students at some point during the year also self-identified issues not documented by peers and

For peer review only

No.	Title	Author/year	MERSQI	COREQ	Reason for inclusion			Undergraduate (UG)/Postgraduate (PG)	Methodology	Purpose of study	Key findings
					Communication	Ethics	Professionalism				
									solving sessions, and from peers, tutors and self at mid and end of Blocks 2-4 and end of Block 5 case-based sessions. Because professionalism cannot be compartmen- talized, students address standards not only in the Professionalism Competency essay but also in the Communication, Personal Development, and Reflective Practice competency essays. Thus, data used in this study included essays and citations for these four competencies. - Coding and analysis of PBL narrative feedback: A trained research assistant extracted the electronic competency report of all PBL feedback for professionalism for all first-year students (n ¼ 32) for the 2009 academic year, and replaced students' names with a unique six-digit identifier. We		tutors. Table 1 shows that peer and tutor feedback and self-assessments documented improvements less frequently than TAFIs, and statements noting improvement in Interpersonal Skills were more fre- quent than statements of improvement in Work Habits. Figure 1 documents peer and tutor feedback statements for students at mid and end of the four PBL blocks. In aggregate, more TAFI statements were made by peers and tutors for mid-block assessments than at the end of the block with an overall decrease across the year. Regarding individual student data, 29 of the 32 students had more TAFIs documented at mid-block by peers as compared with end-of-block, and early in the year (Blocks 1–2) versus later in the year (Blocks 3–4). Similarly, more evidence with regard to improvements was documented for students by peers in end-of-block and late year feedback versus mid block and early year feedback, respectively. Similar trends were noted in the tutor feedback for mid- and end-of- block TAFIs and improvement statements. - (2) To what extent did students self-report and document professionalism learning needs and improvements consistent with peer and tutor PBL feedback in a portfolio used for promotion decisions? -- Student self-assessments presented in their summative portfolio essays are required to be a

For peer review only

No.	Title	Author/year	MERSQI	COREQ	Reason for inclusion			Undergraduate (UG)/Postgraduate (PG)	Methodology	Purpose of study	Key findings
					Communication	Ethics	Professionalism				
									developed a rubric using the PBL assessment form criteria for Interpersonal Skills and Work Habits to individually code five competency reports. The authors then met and reached consensus on coded professionalism statements about TAFIs and observed improvements. The refined Professionalism Rubric has three unprofessional behavior criteria each for Interpersonal Skills (Dominating, Quiet, Inappropriate) and for Work Habits (Late, Ill-prepared, Distracted). Each author coded the remaining reports before meeting to reach consen-sus and in rare instances of coding differences; these discrepancies were reconciled. The authors tallied type and number of professionalism statements for each student for each block from peers, tutors and self.		representative and balanced account of their professional behavior across the year. As an indicator of ‘‘representative’’, we identified behaviors that were observed by tutors in at least two different groups, which suggest stability of behavior across time and group dynamics. For the 16 students where two different tutors identified the same TAFI, peers identified the same TAFIs as the tutors in five PBL Blocks for seven students, four PBL blocks for six students, three PBL blocks for two students, two PBL blocks for one student. All 16 students reported the TAFI in their summative portfolio, citing both tutor and peer feedback. All cited evidence of improvement from peers and/or tutors. A strong majority (91%) of portfolio essays followed a pattern of citing feedback from PBL peers and/or faculty to identify at least one TAFI, followed by citations indicating improved performance. To illustrate student’s use of PBL feedback to document performance, we provide the portfolio self-assessment of one student who received ten TAFIs during the first half of the year and three in the second half, indicating a tendency to dominate. The tendency to dominate was mentioned by peers in four of the five PBL groups but never mentioned by tutors. These comments included statements such as ‘‘talking over people,’’ ‘‘can push ideas a little bit too hard ‘‘sometime can seem

No.	Title	Author/year	MERSQI	COREQ	Reason for inclusion			Undergraduate (UG)/Postgraduate (PG)	Methodology	Purpose of study	Key findings
					Communication	Ethics	Professionalism				
									- Coding and analysis of summative portfolio essays: A research assistant extracted the summative portfolio essays from the database for the four competencies (Professionalism, Communication, Personal Development, and Reflective Practice) that 32 students submitted at the end of the 2009 academic year, for a total of 124 essays. The authors used the Professionalism Rubric to analyze the portfolio essays for the aforementioned competencies, where professionalism issues were likely to be addressed. The authors independently coded professionalism statements before meeting to reach consensus in the rare instances where there were differences in coding. All PBL citations used to document the coded statements were tallied by source (tutor, peer, self) and block time period.		argumentative/defensive'' and needs to ''listen to other viewpoints even though they might not coincide with personal views.'' Three other TAFIs were mentioned three or less times and only in the first half of the year. Table 2 provides quotes from this one student's cited PBL feedback, documenting a TAFI (tendency to dominate) and evidence of improvement over the course of year 1. This student acknowledged and reflected on this TAFI in the portfolio essay and provided evidence of improvement, noting that this behavior may need to be ''reestablished'' with each new working group. See Table 3 for excerpt from this student's portfolio. The essay provides evidence relevant to each of our questions. The student identified and acknowledged a prob- lematic pattern of behavior, reflected on the feedback, and used suggestions to ''amend'' behavior, which resulted in improved performance. Not every student described the self- regulation process in such detail, but all acknowledged a behavior identified by peers and/or tutors that was problem- atic and presented evidence of improvement.<br>From discussion: ... While this study suggests that students are using the supports of this educational program to self-regulate, we need to better understand how students process and use feedback, how external feedback triggers and shapes intern- ally

For peer review only - http://bmjopen.bmj.com/site/about/guidelines.xhtml

No.	Title	Author/year	MERSQI	COREQ	Reason for inclusion			Undergraduate (UG)/Postgraduate (PG)	Methodology	Purpose of study	Key findings
					Communication	Ethics	Professionalism				
											generated feedback, what role advisors play in helping students reflect on and use feedback, and what processes students use to make judgments and develop plans for improvement, questions being addressed by the work of Sargeant et al. (2008, 2009, 2011). As future physicians, today's students will need to be able to self-regulate their behaviors in settings where feedback may not be as structured or robust and facilitation of reflection lacking. To support development of SR skills, we need medical school programs designed to provide formative feedback and clear standards, opportunities to improve, and facilitated self-assessment. The PBL-portfolio model provides one strategy by which students can gain skills in self-regulation and develop habits useful for future practice.
70	Portfolio assessment in medical students' final examinations	Davis, MH, Ben-David, M Friedman, Harden, RM, Howie, Peter, Ker, Jean, McGhee, C, Pippard, MJ and Snadden, David<br><br>2009	7		✓	✓		UG	From abstract: Portfolio assessment has been developed for this purpose and has been adopted for the summative assessment of students in their final examination in Dundee. The contents of the portfolio and the assessment process have been defined and the first cohort of students to be assessed in this	This paper describes a new approach to the final examination based on portfolios, which has been piloted in Dundee Medical School. This paper describes our first year's experience with this new approach.	From abstract: The evaluation of the approach demonstrated strong staff support. Students were also positive although with some reservations. It is concluded that portfolio assessment is a powerful approach to assessing a range of curriculum outcomes not easily assessed by other methods and is worthy of inclusion in the assessor's toolkit.<br>Results: Of the 129 candidates for the portfolio assessment, three were withdrawn for the reasons given above. Of the 126 remaining, 108 (86%)

For peer review only

No.	Title	Author/year	MERSQI	COREQ	Reason for inclusion			Undergraduate (UG)/Postgraduate (PG)	Methodology	Purpose of study	Key findings
					Communication	Ethics	Professionalism				
									way has been studied. The final examination: The final examination was redesigned to meet the needs of the new curriculum, in particular the moves to an inte- grated, task-based approach and an outcome-based education model. The examination is in two parts. Part 1 is at the end of year 4 and comprises a multiple-choice paper of extended matching items (134) designed to assess knowledge and its application; a constructed response question (CRQ) paper to assess higher-order thinking such as problem solving and critical analysis as well as knowl- edge; and an OSCE to assess clinical skills. The use of an OSCE as part of the final examination has been reported (Preece et al., 1992). Part 2 of the final examination takes place towards the end of year 5 and adopts a more innova- tive		passed, 13 (10%) received a condi- tional pass and five (4%) failed. No students fell into the bad fail category. Individual student profiles, in terms of the 22 grades for the learning outcomes, showed variation. Three students (2%) were awarded 22 A grades. One student (0.8%) was awarded 22 B grades. No student was awarded 22 C, D, E or F grades. Forty-four students (35%) were awarded 22 grades at either A or B levels. Two students (1.5%) were awarded 22 grades at either C or D levels. No students were awarded 22 grades at either E or F levels. Over 60% of the students were not consistent in their ability across the learning outcomes, but had individual strengths and weaknesses. The student gradings awarded by both pairs of exam- iners in each outcome are given in Figure 1. Non-pass gradings are confined to outcomes 2, 7, 8, 11 and 12. Evaluation of portfolio assessment: - (1) Analysis of student results: Numeric values from one to six were allocated to the students' outcome grades in the portfolio assessment. An overall mark was then calculated for every student. This overall mark was correlated, using the Spearman correlation, with the mark for each of the three compo- nents of Part 1 of the final examination (EMI, 0.42; CRQ, 0.42; OSCE, 0.47) and with the mark allocated to students

For peer review only

No.	Title	Author/year	MERSQI	COREQ	Reason for inclusion			Undergraduate (UG)/Postgraduate (PG)	Methodology	Purpose of study	Key findings
					Communication	Ethics	Professionalism				
									approach – portfolio assessment. The approach was adopted as it offered the potential of assessing the students' achievement in each of the curriculum outcomes including those not easily assessed by more traditional approaches. It also offered the potential of reviewing the development of student competence over time and presenting feedback to students. Implementation of the portfolio assessment: Work for the portfolio assessment was carried out in three stages:<br>- Stage 1: Work carried out before the portfolio review -- Towards the end of year 5, before the PRHO preparation block, students submitted their completed portfolios to administrative staff in the medical school office. Following a check for completeness,		in the end-of-block examination for one phase 3 core block attachment (psychiatry, 0.34). These results show low/moderate correlation.<br>- (2) Observer documentation: Three observers, two internal and one external, were appointed to document any issues that arose during the portfolio assessment process. They reported that the port- folio submission procedures worked well. The countersigned receipt given to students was an essential step to avoid disputes over submitted material. Entering the pre-marked grades for various portfolio components into the PASS was a major task for administrative staff and required to be streamlined for future years. ... (see under issues related to implementation of portfolio) The PASS system worked well and provided signed sheets to be retained as a permanent record of the examination.<br>- (53) Examiners' evaluation questionnaire: The examiners' evaluation questionnaire was developed following discussion with members of the working group that introduced portfolio assessment and with both the external and internal examiners who participated in the portfolio examination. It was designed to gather the exam- iners' opinions regarding the: effectiveness of the portfolio approach to student assessment; usefulness of the components of the portfolio

For peer review only

No.	Title	Author/year	MERSQI	COREQ	Reason for inclusion			Undergraduate (UG)/Postgraduate (PG)	Methodology	Purpose of study	Key findings
					Communication	Ethics	Professionalism				
									they received and countersigned a receipt. The administrative staff transcribed from student records, onto an assessment summary sheet called the Port-folio Assessment and Summary Sheet (PASS), the grades awarded for the portfolio contents in respect of each of the curriculum outcomes. ... Three of the students were identified as ineligible to proceed to the portfolio review with the examiners at this stage, two for reasons of illness and non-completion of course work and one pending a disciplinary hearing. All three, however, were allowed to complete the portfolio review process for formative purposes.<br>- Stage 2: Portfolio review with the examiners -- ... (see under deciding how to mark/assess)<br>- Stage 3: Work completed after the portfolio review -- ... (see		in providing information about the students' abilities; conduct of the examination; standards achieved by the students; strengths and weaknesses of the portfolio approach to assessment; suggestions for improvement. Examiners were asked to rate a number of statements on a Likert-type five-point scale from 5—strongly agree to 1— strongly disagree. There were also free-response questions. The questionnaire was distributed and completed by the examiners at the end of the examiners' meeting on day 2. Thirty-three examiners' questionnaires were distributed and returned, giving a response rate of 100%. Responses were collated using the Excel computer software package by Microsoft. The percentage distribution of examiners' ratings with the number that responded to each statement (N) the calculated average score (AV) and the standard deviation (SD) are provided in Table 1. ... (see the rest under perspective of examiners)<br>- (4) Student evaluation questionnaire: The student evaluation questionnaire was developed following periodic feedback from the students during their phase 3 studies and from a student group discussion with the external observer that took place immediately after the portfolio review. Students were given the opportunity for free and frank expression of their opinions during this discussion. The

For peer review only

No.	Title	Author/year	MERSQI	COREQ	Reason for inclusion			Undergraduate (UG)/Postgraduate (PG)	Methodology	Purpose of study	Key findings
					Communication	Ethics	Professionalism				
									under deciding how to mark/assess) Evaluation of the portfolio assessment: Five methods were employed to evaluate the use of portfo- lios as the basis for the final examination: (1) analysis of student results; (2) observer documentation; (53) examiners' evaluation questionnaire; (4) student evaluation questionnaire; (5) verbal report from student representatives. (see details under results of study).; From discussion - evaluation methodology: Rossi & Freeman (1993a) (p. 13) define evaluation research as 'a robust arena of activity directed at collecting, analysing and interpreting information on the need for, implementa- tion of, and effectiveness and efficiency of intervention efforts'. Education evaluation is more specifically identified as 'the process of making judgement about		student questionnaire was designed to gather opinion about building the portfolio, about its focus around the curriculum outcomes and about events during the portfolio assessment. The questionnaire was distributed and completed 22 days after the examination, at a time when all students were aware of whether or not they had passed the examina- tion. Those students who were unsuccessful had received counselling. The rating scale, data entry and scoring system were similar to those used in the examiners' ques- tionnaire. A total of 107 student questionnaires were returned from 129 students who sat the examination—a response rate of 83%. The percentage distribution of students' ratings, the number that responded to each statement (N), the calcu- lated average score (AV) and the standard deviation (SD) are provided in Table 2 for building the portfolio and in Table 3 for the portfolio assessment. ... (see the rest under perspective of students)<br>- (5) Student representatives' comments: ... (see the rest under perspective of students)

For peer review only

No.	Title	Author/year	MERSQI	COREQ	Reason for inclusion			Undergraduate (UG)/Postgraduate (PG)	Methodology	Purpose of study	Key findings
					Communication	Ethics	Professionalism				
									the merit, value or worth of educational programmes' (Gall et al., 1996). It is recognized that it is often impossible to conduct educational evaluations according to the best possible design and Rossi & Freeman (1993b) advocate the concept of the 'good enough' evaluation. The five methods employed to evaluate the use of portfolios as the basis for the final examination followed the 'good enough' approach.		
71	A Qualitative Analysis of Narrative Preclerkship Assessment Data to Evaluate Teamwork Skills	Dolan, B. M., O'Brien, C. L., Cameron, K. A. and Green, M. M.<br><br>2018		15	✓		✓	UG	From abstract: We performed a qualitative analysis of narrative data in 15 assessment portfolios. Student portfolios were randomly selected from 3 groups stratified by quantitative ratings of teamwork performance gathered from small-group and clinical preceptor assessment forms. Narrative data included peer and faculty feedback from these same forms. Data were	We sought to understand if and how teamwork behaviors are described in the narrative feedback given by faculty and peers in preclerkship years and how this feedback may vary based on students' competency achievement level. We expect that review of assessment data collected in an electronic portfolio and judged by experienced faculty may fill the gap in teamwork assessment by providing a method to evaluate	From abstract -- Results: Eight codes related to teamwork emerged: attitude and demeanor, information facilitation, leadership, preparation and dependability, professionalism, team orientation, values team member contributions, and nonspecific teamwork comments. The frequency of codes and valence varied across the 3 performance groups, with students in the low-performing group receiving more suggestions for improvement across all teamwork codes. Conclusions: Narrative data from assessment portfolios included specific descriptions of teamwork behavior, with important contributions provided by

For peer review only

No.	Title	Author/year	MERSQI	COREQ	Reason for inclusion			Undergraduate (UG)/Postgraduate (PG)	Methodology	Purpose of study	Key findings
					Communication	Ethics	Professionalism				
									coded for teamwork-related behaviors using a constant comparative approach combined with an identification of the valence of the coded statements as either "positive observation" or "suggestion for improvement." Study approach and setting: We conducted a qualitative study using a constant comparative approach to better characterize the narrative assessments that students receive regarding teamwork.17 ... We excluded assessments that do not include students' teamwork skills, including assessments from observed structured clinical exams, assessments of written assignments, medical knowledge assessments, assessments of groups as a whole (where no individual feedback was given), ethics oral examinations, direct observations of	longitudinal, behaviorally based narrative feedback regarding student teamwork knowledge, skills, and attitudes.	both faculty and peers. A variety of teamwork domains were represented. Such feedback as collected in an assessment portfolio can be used for longitudinal assessment of preclerkship student teamwork skills and attitudes. From discussion: This analysis demonstrates that narrative feedback in assessment portfolios provides a longitudinal view of preclerkship students' teamwork behaviors. Although similarities exist between performance groups, we observed differences between groups in content, frequency, and valence of feedback. These differences could assist mentor/coaches and competency committee members in identifying both students who excel at teamwork and those needing additional support with teamwork behaviors prior to starting clerkships. Although our coded categories were identified without the use of a predetermined framework, they align with key teamwork and leadership attributes identified in the literature. Critical teamwork skills and attitudes have previously been described that share similarity with our codes: leadership, team orientation, preparation/dependability.21 Physician leadership competencies highlight the importance of showing integrity, listening actively, promoting team building, and nurturing/recognizing others' accomplishments.22–24

For peer review only

No.	Title	Author/year	MERSQI	COREQ	Reason for inclusion			Undergraduate (UG)/Postgraduate (PG)	Methodology	Purpose of study	Key findings
					Communication	Ethics	Professionalism				
									clinical skills, and scholarly project oral presentations (which focus solely on presentation skills). Sampling strategy: Students were notified of the study via e-mail and were encouraged to e-mail an author not involved in grading students (CLO) if they did not wish to participate. Students were given 1 week to respond. Four students chose to opt out of the study. We divided the remaining student portfolios into three groups. We first identified the students who received progressing towards competence with concern or progressing towards competence pending additional development for any of the three competencies most closely related to teamwork—teamwork, professionalism, and communication—during the competency review. We		These attributes arose in our analysis, lending credibility to the argument that narrative data in assessment portfolios can be used to understand students' teamwork behaviors in the preclerkship years and can help students reflect on how their behaviors promote or detract from their teams' overall performance. Review of the preclerkship narrative data provides mentors or coaches the ability to identify students in need of remediation before full-time immersion in clinical practice.25 Our analysis demonstrates that low-performing students share similar characteristics that reviewers can look for poor attitude in small groups, lack of preparation for group activities, or dismissive behavior toward others' contributions. It also demonstrates that the absence of certain comments may be telling, as evidenced by the sharp reduction in even nonspecific comments such as "great team member" or the lack of leadership observations in the low-performing student portfolios. When reviewing assessment data with their students, mentors may choose to highlight these absences to help students improve. Notably, peers provided the majority of feedback in the codes of team orientation and values' contributions of others. Due to their close working relationships during preclerkship years, peers may be in the best position

For peer review only

No.	Title	Author/year	MERSQI	COREQ	Reason for inclusion			Undergraduate (UG)/Postgraduate (PG)	Methodology	Purpose of study	Key findings
					Communication	Ethics	Professionalism				
									designated these 19 students as the low-performing group. As our competency review does not have a comparable high-performance designation, we employed an alternative strategy using the composite grading system. To identify a high-performing cohort of students for comparison, we identified the 19 students receiving the highest numerical ratings for the group of assessments included in Table 1. We designated this to be the high-performing group. The middle-performing group comprised all remaining students (n = 114) who did not meet criteria for the high- or low-performing groups. Four students opted out of the study. To protect privacy, each student portfolio received a random numerical identifier. A key was created linking each identifier to each student's ID number; an		to make such judgments. Furthermore, when peers are given formalized instruction in providing feedback, as they are at our institution, including their insight and feedback is critical when assessing student performance. Our results suggest that assessment systems that do not include peer feedback may miss the opportunity to identify students in need of support for developing teamwork skills. Prior studies demonstrate student acceptance of peer feedback, particularly when used for formative purposes.26 Additional studies that specifically compare faculty versus student feedback may further illuminate the best ways to employ the differing perspectives of peers and faculty when assessing student performance. The use of assessment portfolio data may continue to increase as institutions implement entrustable professional activities, competency committees, and other assessment strategies that require a systems-based approach.27 We believe that the findings in this analysis can assist both in teamwork assessment and as a resource to aid faculty and students in identifying preclerkship goals for teamwork and leadership skills development. Additional study is needed to determine if preclerkship assessment of teamwork correlates with later clinical performance. In conclusion, our analysis of narrative

For peer review only

No.	Title	Author/year	MERSQI	COREQ	Reason for inclusion			Undergraduate (UG)/Postgraduate (PG)	Methodology	Purpose of study	Key findings
					Communication	Ethics	Professionalism				
									author not involved in direct teaching of students (CLO) deidentified all data before analysis. We randomly selected three student portfolios from each group (high, middle, low) to analyze, then further intentionally sampled two additional portfolios in each group to allow for adequate gender inclusion and to confirm emergence of no new codes (see next); these 15 portfolios composed our sample. Data analysis: To better understand how teamwork performance is observed and described by peers and faculty, we used constant comparative analysis techniques. We selected this method as a framework to explore teamwork given that comparison between groups of students may better delineate the similarities and/or differences in how behaviors were described among variably		assessment data from across the preclerkship curriculum demonstrates that these data capture a number of key teamwork skills and attitudes with behaviorally based feedback, thus filling a gap in the longitudinal assessment of teamwork. Providing students and faculty the opportunity to critique and further reflect on behaviorally based narrative feedback may assist students in improving these skills prior to the beginning of clerkships.

For peer review only

No.	Title	Author/year	MERSQI	COREQ	Reason for inclusion			Undergraduate (UG)/Postgraduate (PG)	Methodology	Purpose of study	Key findings
					Communication	Ethics	Professionalism				
									performing students. Constant comparative methodology has been described as an ideal way to "conceptualise the variety that exists within the subject under study.17(p393) After using this methodology to complete the coding, we undertook a content analysis to better understand the prevalence and valence of identified themes across the three performance groups.19 Three research team members (BMD, CLO, MG) with expertise in medical student assessment performed the primary analysis of portfolio comments; two are practicing clinicians with experience on medical teams (BMD, MG). The fourth team member, an expert in qualitative research (KAC), provided guidance and oversight in methodology. Narrative data was uploaded for analysis into		

For peer review only

No.	Title	Author/year	MERSQI	COREQ	Reason for inclusion			Undergraduate (UG)/Postgraduate (PG)	Methodology	Purpose of study	Key findings
					Communication	Ethics	Professionalism				
									NVivo (QSR, Mac version 10.2.0) after deidentification.20 We selected the middle-performing group as the first group for analysis, as it represents the typical student who is neither struggling nor exceptionally high achieving. Consistent with conventional content analysis19 and to best explore what teamwork categories faculty and students choose to comment on in their narratives, we did not use an a priori framework for coding. However, one author (BMD) is familiar with medical teamwork literature,21,22 which has informed the way medical students approach teamwork in our institution and which may have influenced her approach to coding. The second coder has a background in education. She had no specific expertise with the teamwork literature and arrived at the same codes. Two		

For peer review only

No.	Title	Author/year	MERSQI	COREQ	Reason for inclusion			Undergraduate (UG)/Postgraduate (PG)	Methodology	Purpose of study	Key findings
					Communication	Ethics	Professionalism				
									authors (BMD and CLO) initially reviewed three portfolios from the middle-performing group, assigning a code to all narrative statements. These authors then met and achieved consensus regarding the coding framework. The remaining portfolios in the middle-performing group were analyzed using the identified codes; all statements were assigned a valence of either “positive observation” or “suggestion for improvement.” Three authors (BMD, CLO, MG) met to review the statements' assigned codes and come to consensus on any differences, both with respect to code and valence. Differences were resolved via group discussion. Using the codes identified, we then analyzed the low- and high-performing groups, representing the extremes of performance.		

For peer review only

No.	Title	Author/year	MERSQI	COREQ	Reason for inclusion			Undergraduate (UG)/Postgraduate (PG)	Methodology	Purpose of study	Key findings
					Communication	Ethics	Professionalism				
									Although no new codes were identified in the high-performing or low-performing groups, the narrative from these groups helped to refine the coding set to reflect nuances that emerged from the other groups. For example, after reviewing the high-performing narrative data, the original code "information sharing" was revised to "information facilitation" to better reflect the variety of ways that students mediated the flow of information in small groups. The code "values contributions of team members" evolved from "values team members" after review of the additional suggestions for improvement found in both the high- and low-performing groups. We reviewed three portfolios in each performance group but identified few new codes after the second student's portfolio in each group. However,		

For peer review only

No.	Title	Author/year	MERSQI	COREQ	Reason for inclusion			Undergraduate (UG)/Postgraduate (PG)	Methodology	Purpose of study	Key findings
					Communication	Ethics	Professionalism				
									we continued with two additional portfolios per group for adequate gender inclusion and to confirm the emergence of no new codes; no new codes were found with the addition of these six portfolios. Upon completion of the coding process, NVivo software was used to quantify the number of comments per code and valence. Techniques to ensure trustworthiness of data included the use of two coders followed by additional review of the codes by a third author. Codes and examples were also discussed at a medical education group research meeting where additional faculty members with less connection to the portfolio process provided feedback to the research team.		
72	Portfolio as a learning tool: students' perspective	Elango, S., Jutti, R. C. and Lee, L. K.<br><br>2005	6.5		✓			UG	From abstract: A questionnaire survey was conducted among 143 medical students to find out their perceptions of the	This study was formulated to find out students' perspectives on portfolio learning.	From abstract: ... (see the rest under perspective of students) The study indicates that students need appropriate guidance from the academic staff for the system to succeed.<br>From introduction: In the

For peer review only

No.	Title	Author/year	MERSQI	COREQ	Reason for inclusion			Undergraduate (UG)/Postgraduate (PG)	Methodology	Purpose of study	Key findings
					Communication	Ethics	Professionalism				
									portfolio as a learning tool. From materials and methods: Feedback was obtained from an entire batch of Semester 8 students by asking them to write down what they felt about writing a portfolio. Based on their feedback, a questionnaire consisting of 23 questions was designed. The entire student population (259) in the clinical school (from Semester 6 to Semester 10) was given an information sheet with details about this survey and was given the option of participating in the study. The students who volunteered for the study were asked to sign a written consent form. The students who gave their consent for the survey were requested to complete the questionnaire anonymously. For each of the 23 questions, students had to respond on a 4-point Likert scale, ranging from		last decade, there have been numerous innovations in educational theory and practice. Education has moved from being a traditional teacher-centred process to one that is student-centred. Similarly, in the area of assessment, there has been a shift away from assessing knowledge towards a more competency/performance-based assessment. There is now a wide variety of methods of education and training to choose from and portfolio-based learning is an increasingly popular option among educationists.1<br>From results: One hundred and forty-three students completed and returned the questionnaire (Table1). There were 76 (53.1%) females and 64 (44.8%) males. In order to facilitate the analysis, the 4 levels of perception in the questionnaire – strongly agree, agree, disagree and strongly disagree – were regrouped; strongly agree and agree were grouped as one, and strongly disagree and disagree were grouped as the other. ... (see the rest under perspective of students)<br>From discussion: A study has shown that students using portfolios obtain higher marks in factual knowledge compared to the control group of students not using portfolios11<br>From conclusion: The study demonstrates that any medical school intending to introduce the portfolio as a new learning method must take into consideration an

For peer review only

No.	Title	Author/year	MERSQI	COREQ	Reason for inclusion			Undergraduate (UG)/Postgraduate (PG)	Methodology	Purpose of study	Key findings
					Communication	Ethics	Professionalism				
									strongly agree to strongly disagree. The responses to the questionnaire were analysed to determine the overall views of the students on the different aspects of portfolio writing.		effective orientation programme for both the learners and the teachers. Motivated students, and trained and motivated staff are essential for portfolio-based learning to succeed.
73	Students' reflections in a portfolio pilot: highlighting professional issues	Haffling, A. C., Beckman, A., Pahlmblad, A. and Edgren, G.<br><br>2010		14	✓	✓	✓	UG	From abstract: Thirty-five voluntary final-year medical students piloted a standardized portfolio in a general practice (GP) attachment at Lund University, Sweden. Students' portfolio reflections were based upon documentary evidence from practice, and aimed to demonstrate students' learning. The reflections were qualitatively analysed, using a framework approach. Students' evaluations of the portfolio were subjected to quantitative and qualitative analysis. Pilot: Students of two consecutive classes, of 76 and 71 students, respectively, were approached. Twenty students in each class	From abstract: To investigate whether students' reflections include sufficient dimensions of professional competence, notwithstanding a standardized portfolio format, and to evaluate students' satisfaction with the portfolio<br>From introduction: We aimed to develop a portfolio for the assessment of final-year students' professional development during their general practice (GP) attachment. ... We designed a pilot study with the principal aim of investigating whether themes of students' reflections would include satisfactory dimensions of professional competence, notwithstanding the standardized format. This article qualitatively explores the emergent themes of students' reflections, and also reports on students' evaluation of the portfolio.	From abstract -- Results: Among professional issues, an integration of cognitive, affective and practical dimensions in clinical practice was provided by students' reflections. The findings suggested an emphasis on affective issues, particularly on self-awareness of feelings, attitudes and concerns. In addition, ethical problems, clinical reasoning strategies and future communication skills training were subjects of several reflective commentaries. Students' reflections on their consultation skills demonstrated their endeavour to achieve structure in the medical interview by negotiation of an agenda for the consultation, keeping the interview on track, and using internal summarizing. The importance of active listening and exploration of patient's perspective was also emphasized. In students' case summaries, illustrating characteristic attributes of GP, the dominating theme was 'patient-centred care', including the patient–doctor relationship, holistic modelling and longitudinal continuity. Students were satisfied with the portfolio,

For peer review only

No.	Title	Author/year	MERSQI	COREQ	Reason for inclusion			Undergraduate (UG)/Postgraduate (PG)	Methodology	Purpose of study	Key findings
					Communication	Ethics	Professionalism				
									were offered to voluntarily pilot a portfolio in GP as an addition to the written test, which for the participants would be reduced in the number of GP questions. A total of 35 students, 16 from the first and 19 from the second class, accepted. Twenty-four (69%) of them were women, as compared to 59% of all students. At the time of the study, consent from the University's ethics committee was not required for projects involving students' assessments. Participating students were provided with both verbal and written guidelines on the purpose, format and assessment of their portfolios. Three of the authors (ACH, AP, AB) and a fourth teacher, all involved in teaching, acted as mentors to support their students in compiling the portfolio. Qualitative analysis of students' reflections: To be		but improved instructions were needed.; Conclusions: A standardized portfolio in a defined course with a limited timeframe provided ample opportunities for reflections on professional issues. Support by mentors and a final examiner interview contributed to the success of the portfolio with students. The interview also allowed students to deepen their reflections and to receive feedback. Students' evaluation questionnaire: see under perspective of students From discussion: Firstly, students' reflections on their consultation skills showed that the most important learning issue in the theme of 'process skills' was how to achieve structure in the medical interview. Patient-centred communication can cause genuine concerns in students about losing control of the consultation and getting caught in a flow of less ordered information from the patient (Silverman et al. 2004). When students learned how to obtain structure, they also experienced better time management, enhanced self-confidence and an improved rapport with the patient. Secondly, the theme of 'patient-centred care' was emphasized in students' case summaries. Patient-centred care is a major principle in GP, but is of essential interest to several other medical disciplines as well. Achieving shared decision making, understanding the patient as

For peer review only

No.	Title	Author/year	MERSQI	COREQ	Reason for inclusion			Undergraduate (UG)/Postgraduate (PG)	Methodology	Purpose of study	Key findings
					Communication	Ethics	Professionalism				
									able to conduct the qualitative analysis, students' reflections were written verbatim into a word processor by one of the authors (173). Each student was allocated a code number to prevent identification of individual students' comments. The text of the reflections constructed two separate units of analysis; 'consultation skills' and 'case summaries'. The transcripts were read through several times to get a sense of the whole, and then analysed using a framework approach (Crabtree & Miller 1999). For 'consultation skills', the text was mapped against the Calgary Cambridge Guides (Silverman et al. 2004). For 'case summaries', the statement of WONCA Europe (2005), (Box 2), was used as a template for organizing the data. In each of		a whole person and exploring the patient's perspective are aspects shown to positively influence patients' satisfaction and enablement, reduce their symptom burden and discomfort, and increase efficiency of care (Stewart et al. 2000; Little et al. 2001). We believe that these central dimensions of patient-centred care ought to be highlighted earlier in hospital clerkships. Thirdly, the findings suggested integration of the cognitive, affective and practical dimensions of professional competence that can be acquired in a clinical learning environment (Mann et al. 2007; van Tartwijk & Driessen 2009; Passi et al. 2010). As expected, students reflected on their 'content skills' and provided evidence for their identification of deficits in knowledge and skills, their integration of new understanding, and their plans for development. However, 'perception skills' was a more prominent theme in both units of analysis. In their self-awareness of feelings, attitudes and concerns, students were unexpectedly open and frank about their deficiencies and about how they had tried to remedy them. They also acknowledged the importance of ethical issues and additional communication skills training, and openly reflected on their clinical reasoning. However, we found it remarkable that

For peer review only

No.	Title	Author/year	MERSQI	COREQ	Reason for inclusion			Undergraduate (UG)/Postgraduate (PG)	Methodology	Purpose of study	Key findings
					Communication	Ethics	Professionalism				
									the two units of analysis, related segments of text were identified and indexed to the appropriate part of the thematic framework. Some codes were modified and new ones added to reflect as many nuances in the data as possible. 'Chunks' of related text under each code were examined together and distilled summaries made; this process involved a considerable amount of abstraction. The chunk summaries provided an analytical tool from which connections were made and sub-themes and themes identified. ACH undertook the mapping of text and carried out the primary analysis of the data. The four teachers, involved in the pilot, examined in recurrent meetings whether the interpretation of the data was plausible. The codes and sub-themes were compared based on differences		several students, in their final year in medical school, reflected on their initial problems in transferring theory to practice; a skill supposed to be frequently trained during previous hospital clerkships.<br>From conclusion: In summary, this study demonstrated that a structured portfolio in a short timeframe provided abundant opportunities for students to reflect on personal and professional issues, notwithstanding the standardized format. We strongly recommend a final examiner interview to deepen students' reflections, to supply feedback and to raise the importance of reflective ability as a skill in future learning from practice.

For peer review only

No.	Title	Author/year	MERSQI	COREQ	Reason for inclusion			Undergraduate (UG)/Postgraduate (PG)	Methodology	Purpose of study	Key findings
					Communication	Ethics	Professionalism				
									and similarities, and some were revised during this process of corroboration. Students' evaluation questionnaire: The short student evaluation form was supplied directly after the examiner interview, asking five questions about the portfolio instructions, the mentor contact and the examiner interview, using a 3-point scale ('yes', 'to some extent', 'no'). Students were also requested to provide free comments on positive and negative experiences of the portfolio, and to give suggestions for improvements. The results were subjected to quantitative and qualitative (content) analyses.		
74	Undergraduate medical students' views about a reflective portfolio assessment of their communication skills learning	Rees, C. and Sheard, C.<br><br>2004	10		✓			UG	From abstract: 178 second-year medical students at the University of Nottingham completed the 18-item reflective portfolio questionnaire (RPQ) (α = 0.716) and a personal details questionnaire	this study explores second-year medical students' views about a reflective portfolio assessment of their communication skills. The research questions for this study are: 1) What are students' views of a reflective portfolio assessment	From abstract -- Results: Total scores on the RPQ ranged from 40 to 75 (mean 58.28, SD 7.08). Significant relationships existed between RPQ total scores and students' ratings of their reflection skills (r s = 0.322, P < 0.001), RPQ total scores and students' confidence building another portfolio (T = 4.381, d.f. = 176, P

For peer review only

No.	Title	Author/year	MERSQI	COREQ	Reason for inclusion			Undergraduate (UG)/Postgraduate (PG)	Methodology	Purpose of study	Key findings
					Communication	Ethics	Professionalism				
									three days before submitting their portfolio assessment for communication skills. Data were analysed using univariate and multivariate statistics on SPSS Version 10.0. From methods: Participants -- Second-year medical students at the University of Nottingham completed a personal details questionnaire (PDQ) and the reflective portfolio questionnaire (RPQ) three days before submitting their portfolio assessment for communication skills.; Measures -- The PDQ contains 13 demographic and education-related items. The RPQ was designed by CR to explore medical students' views of portfolios and reflection. Eighteen items were generated using previous research literature,1-4 e.g. ‘I have felt anxious about sharing my weaknesses in my reflective portfolio’ (item 16). Each item	of their communication skills? 2) Do relationships exist between students' views of reflective portfolios and their perceived and actual reflection skills and their confidence building portfolios?	< 0.001), and RPQ total scores and students' marks for their reflective portfolio assessment (r s = 0.167, P = 0.029). Students with more positive views about reflective portfolios were more likely to rate their reflection skills as good, receive better marks for their portfolio assessment, and be more confident building another portfolio. Discussion: This study begins to highlight preclinical medical students' views about reflective portfolios. However, further research is required using qualitative studies to explore students' views in depth. Medical educators should be encouraged to consider introducing portfolios as a method of formative and summative assessment earlier in the medical curriculum. From results: - Participant characteristics: Of the 206 students invited to participate in this study, 178 (86.4%) completed the reflective portfolio questionnaires satisfactorily. Students ranged in age from 18 to 30 years (median = 20, interquartile range = 19–20). The majority were female (n = 105, 59%), white (n = 130, 73.4%) and came from nonmanual socio-economic classes (n = 170, 95.5%). Students' ratings of their communication skills ranged from 1 to 5 (median = 3, interquartile range = 3–3) and nearly all (n = 173, 97.2%) thought their communication skills needed improving. Students' ratings of their

For peer review only

No.	Title	Author/year	MERSQI	COREQ	Reason for inclusion			Undergraduate (UG)/Postgraduate (PG)	Methodology	Purpose of study	Key findings
					Communication	Ethics	Professionalism				
									was written in the form of a statement and was accompanied by a 5-point Likert scale from 1 (strongly disagree) to 5 (strongly agree). Six medical educators with a range of experience of reflective portfolios commented on the language used in the preliminary draft of the questionnaire, which resulted in some minor alterations in language. The RPQ is scored by reversing the scores for the negative items and then summing the scores for all 18 items. The total score ranges from 18 to 90, with higher scores indicating more positive views about reflective portfolios. This scale was found to have good internal consistency in this study (Cronbach's α= 0.716) and the study results presented in this paper support the construct validity of the tool (copies of the RPQ can		reflection skills ranged from 1 to 5 (median = 3, interquartile range = 2–3) and most (n = 155, 87.6%) thought their reflection skills needed improving. ... (see the rest under perspective of students) Students' scores for their reflective portfolio coursework ranged from 33 to 100% (median = 67, interquartile range = 60–73%).<br>- Students' views about reflective portfolios: see the rest under perspective of students<br>From discussion: ... (see the rest under perspective of students) These findings have implications for further research and educational practice. Qualitative studies are needed to explore in depth the reasons why students possess the views they have about reflective portfolios. Qualitative research may also shed some light on how medical educators could increase students' confidence in building reflective portfolios. This qualitative research could help medical educators improve educational programs using portfolios, so that they are more acceptable to students. In the meantime, this study indicates that the act of constructing a reflective portfolio could increase students' confidence in building another portfolio. Despite portfolios being seen to have weaknesses (e.g. lacking reliability4), medical educators should be encouraged to consider introducing portfolios as a

No.	Title	Author/year	MERSQI	COREQ	Reason for inclusion			Undergraduate (UG)/Postgraduate (PG)	Methodology	Purpose of study	Key findings
					Communication	Ethics	Professionalism				
									be requested by contacting the author). Students' percentage marks for their reflective portfolio assessments were collected at a later date.; Data analysis -- After checking the distribution of continuous variables, data were analysed using univariate (i.e. Spearman's correlation coefficients and T -tests) and multivariate statistics (i.e. stepwise multiple regression) on SPSS Version 10.0.		method of formative and summative assessment earlier in the medical curriculum. Students may then be better placed to negotiate this type of assessment in higher stakes examinations.
75	The reliability of assessment criteria for undergraduate medical students' communication skills portfolios: the Nottingham experience	Rees, C. E. and Sheard, C. E.<br><br>2004	8.5		✓			UG	From abstract: Two independent analysts assessed a random sample of portfolios (n = 100, 49.5%) using criterion-referenced assessment. Students' performances were examined against subjective items in five areas: 1) portfolio structure, 2) level of critical reflection, 3) level of skills development, 4) use of documentary evidence, and 5) use of relevant literature. These	From abstract: This study aims to determine the reliability of assessment criteria used for a portfolio at the University of Nottingham.	From abstract -- Results: The level of agreement between the two raters for the total percentage score was 0.771 (95% CI = 0.678, 0.840), as measured by an intraclass correlation coefficient. The levels of agreement between the two raters for the individual items of the assessment criteria ranged from κ = 0.359 (item 3) to κ = 0.693 (item 4). Discussion: This study provides some support for the summative assessment of portfolios. The findings suggest that discussion and negotiation between independent assessors can enhance the reliability of assessment criteria. Therefore, medical educators are encouraged to use such procedures in the summative assessment

For peer review only

No.	Title	Author/year	MERSQI	COREQ	Reason for inclusion			Undergraduate (UG)/Postgraduate (PG)	Methodology	Purpose of study	Key findings
					Communication	Ethics	Professionalism				
									subjective judgements were later converted into quantitative scales ranging from 0 to 3 so that interrater reliability could be established. The level of agreement between the two analysts for the total percentage score was established using an intraclass correlation coefficient and for the individual items using weighted kappa coefficients. From methods: Sample -- Of the 202 portfolios submitted in March 2002, a random sample of 100 (49.5%) were photocopied for second marking. This random sample of 100 portfolios (median = 66.7%, interquartile range = 53.3–73.3%) did not differ significantly from the portfolios (n = 102) not chosen for second marking (median = 66.7%, interquartile range = 53.3–73.3%) in terms of their total percentage scores (Z = −0.155, P = 0.877).; Assessment		of portfolios. From results: The level of agreement between the two raters for the total percentage scores, as measured by an intraclass correlation coefficient, was 0.771 (95% CI = 0.678, 0.840). The levels of agreement between the two raters for the individual items, as measured by weighted kappa coefficients, ranged from 0.359 (item 3) to 0.693 (item 4) (see Table 1).

No.	Title	Author/year	MERSQI	COREQ	Reason for inclusion			Undergraduate (UG)/Postgraduate (PG)	Methodology	Purpose of study	Key findings
					Communication	Ethics	Professionalism				
									procedures -- One hundred portfolios were assessed ... Although students were aware of these five areas, they were not give the full assessment criteria outlined in Appendix 1. ... (see the rest under deciding how to mark/assess); Statistical procedures: An intraclass correlation coefficient on SPSS (Version 10.0) was used to establish the level of agreement between the two raters for the total percentage scores. Weighted kappa coefficients on SAS (Release 6.12) were used to determine the levels of agreement between the two raters for the individual items of the assessment criteria.		
76	Portfolio as a tool to evaluate clinical competences of traumatology in medical students	Santonja-Medina, F., Garcia-Sanz, M. P., Martinez-Martinez, F., Bo, D. and Garcia-Estan, J.<br><br>2016	10.5		✓			UG	From abstract: A total of 131 students used the portfolio during their clinical rotation of traumatology. The students' portfolios were blind evaluated by four professors who annotated the existence	From abstract: This article investigates whether a reflexive portfolio is instrumental in determining the level of acquisition of clinical competences in traumatology, a subject in the 5th year of the degree of medicine.<br>From introduction: ...	From abstract: The reliability of the portfolio was moderate, according to the kappa index (0.48), but the evaluation scores between evaluators were very similar. Considering the mean percentage, 59.8% of the students obtained all the competences established and only 13 of the 23 learning outcomes (56.5%) were fulfilled by >50% of the

No.	Title	Author/year	MERSQI	COREQ	Reason for inclusion			Undergraduate (UG)/Postgraduate (PG)	Methodology	Purpose of study	Key findings
					Communication	Ethics	Professionalism				
									(yes/no) of 23 learning outcomes. From methods: A portfolio was designed to assess the level of acquisition of clinical competences, ... - Academic structure: Traumatology is a compulsory subject in the 5th year of the degree, consisting of 55 hours of theoretical classes, of which 28 hours take place in the classroom, 14 hours are of a seminar type, 10 hours are about clinical cases, and 3 hours are about cooperative learning; 21 hours of classes on laboratory skills in seven sessions; and 2 weeks of hospital rotation (50 hours, 10 days). Of these 50 hours, students rotate in the traumatology service of the university hospitals between hospital ward, medical consultation, operating rooms, and emergency services. Only one student is assigned to one professor in the	Thus, in the current article, we report the application of a portfolio tool to assess the level of acquisition of clinical skills and competences in medical students of the Medical School of the University of Murcia (Spain), during the clinical rotations of traumatology and orthopedic surgery. An important part of the study is also devoted to the analysis of the reliability of the portfolio.29	students. Our study suggests that the portfolio may be an important tool to quantitatively analyze the acquisition of traumatology competences of medical students, thus allowing the implementation of methods to improve its teaching. From results: A total of 138 comparisons were made to obtain the kappa index among the four evaluators for each of the 23 learning outcomes. The distribution of frequencies and percentages of agreement, according to the classification of Landis and Koch,29 are shown in Table 3. As it can be seen, out of 138 comparisons, 83 had kappa indexes >0.4 and 44 of them had values >0.6, which indicates that in a 31.9% of the occasions, the agreements between evaluators have been very substantial. Table 4 shows the mean and standard deviation of the kappa index of the four evaluators. It can be seen that the global kappa is 0.48, indicating that the portfolio has a moderate reliability, according to Landis and Koch.29 As an additional way of assessing the reliability of the portfolio, 6 months after the initial evaluation, two of the evaluators revised again the portfolios (second revision or reference). Table 5 shows the level of agreement obtained by each evaluator when both revisions were analyzed. Globally, the differences among evaluators are not very important because kappa indexes show that

No.	Title	Author/year	MERSQI	COREQ	Reason for inclusion			Undergraduate (UG)/Postgraduate (PG)	Methodology	Purpose of study	Key findings
					Communication	Ethics	Professionalism				
									hospital. Table 2 shows the number of days students spent during these rotations. - Portfolio structure: The portfolio was given to all the students during the course (n=131) in a booklet format. After the identification of data, the students read about legal and ethical issues in relation to the need for keeping medical secret and signed conformity. Each day the students were expected to fill up their reflections before leaving the hospital, writing about the clinical activities observed during the day. In addition, the portfolio had to be signed by the assigned professor every day. The students were given enough space to include everything needed, but there were three main paragraphs: ... (see under content of portfolio) - Portfolio evaluation: The portfolios were evaluated by four		evaluators 1 and 4 have a substantial agreement in both revisions, whereas evaluators 2 and 3 have a moderate level of agreement. The level of achievement of the traumatology competences is shown in Table 1. The data show the mean percentage of the four evaluations as well as the number of times the procedures were performed. A total of 59.8% of the students have acquired the competences as expected during their hospital stay. In all, 13 of the 23 learning outcomes (56.5%) have been acquired by >50% of the students. Competence 5 (87.4%) and competence 1 (81.4%) show the greater values, whereas the rest of them show an acceptable level, except competence 7, showing only a value of 26%. From discussion: The current article shows the results obtained with the application of a portfolio to medical students as a tool to obtain information regarding the level of acquisition of the clinical competences of the topic traumatology. One of the important aspects of any portfolio is its reliability, the consistency and accuracy of the assessment tool in measuring students' performance. Our results quite agree with those found by other authors;30,31 although in some studies of nurse students, higher numbers (0.8) have been described.32 To prove that the results were not obtained by chance, the

No.	Title	Author/year	MERSQI	COREQ	Reason for inclusion			Undergraduate (UG)/Postgraduate (PG)	Methodology	Purpose of study	Key findings
					Communication	Ethics	Professionalism				
									professors, three of them professors of traumatology and one a colleague professor from a different area, that is, physiology; all of them were medical doctors. ... (see the rest under deciding how to mark/assess) All the portfolios underwent blind evaluation. All the students agreed and participated enthusiastically in the study.<br>- Statistical analysis: Data analysis was performed using the Statistical Package for the Social Sciences, Version 19. The Cohen's kappa coefficient was used as a statistical measure of interevaluator agreement for qualitative items.29 Frequencies, percentages, means, and standard deviations were also obtained to describe the results.		second review of the portfolios took place 6 months after the initial evaluation in order to see whether the reliability index could be improved. As observed in Table 5, the maximum level of agreement among evaluators is found in competence 6 (observe and assist in surgical treatments). A moderate agreement exists in competences 2, 3, and 4, whereas competences 1, 5, and 7 show a low level of agreement among evaluators. The results of Table 1 show important information regarding the level of acquisition of the competences analyzed. Two of them, competences 1 and 5, related to diagnostic and treatment obtained the greatest value, with an important frequency of occurrence. Next competence in terms of the level of acquisition is number 3 (70%), probably due to the high number of radiographic images viewed in contrast to magnetic resonance images, where it is likely that doctors rely more on the radiologist report, without actually interpreting them. Competence 6 has also a moderate level of acquisition (55.8%), in spite of the fact that almost all students (99.2%) report attendance to operating rooms (2.6 + 1.3 days). This is likely due to the fact that the surgeons do not invite all the students to wash and be prepared to help, an activity we recommend to all our professors. A similar

For peer review only

No.	Title	Author/year	MERSQI	COREQ	Reason for inclusion			Undergraduate (UG)/Postgraduate (PG)	Methodology	Purpose of study	Key findings
					Communication	Ethics	Professionalism				
											reason may be behind the moderate level achieved in competences 2 (47.1%) and 4 (51.5%), showing that not all the students are able to explore or perform maneuvers with the patients and that this may be due to a time limitation problem while in the ward or consultation. We believe that this should be corrected and doctors should allow more time for the students to perform, under the appropriate supervision, exploration, or treatment of the patients. Finally, competence 7 has the lowest level. It is likely related to the fact that students are not allowed to sign discharge reports or other forms, although it is important that they learn from their professors how to do it. In any case, this is the first time that we obtained this quantitative information regarding the clinical stays of the medical students and this will help us to analyze our teaching results and to develop new strategies to enhance the level of clinical competence the students should reach. A problem learned from the application of this portfolio is that many of the learning outcomes were checked without taking into account the number of times these were carried out. The rest of the learning outcomes were quantitative, so we could obtain the number of times the student performed them. For instance, they attended the operating rooms 5.5 + 3.3 times (range, 1–26), and viewed a mean of 2.3 magnetic resonance images

For peer review only

No.	Title	Author/year	MERSQI	COREQ	Reason for inclusion			Undergraduate (UG)/Postgraduate (PG)	Methodology	Purpose of study	Key findings
					Communication	Ethics	Professionalism				
											(range, 1–14). We believe that it is important that the portfolios are designed to collect also the quantitative information, since both quantitative and qualitative information give the real essence of the learning process. A drawback we have seen with the application of the portfolio to clinical students is that there is a random factor affecting the acquisition of the competences. This is due to the fact that the students rotate between different hospitals and at different times of the year. Thus, it depends on the type of medical problems available on the days they are assigned to the hospital; thus they may not see all the problems selected in the portfolio. Clearly, the greater the number of days of hospital stay, the better it is. However, this is not always possible. A better selection of the learning outcomes will also be helpful. Moreover, the portfolio is designed to be an evaluation tool that should be used in conjunction with others (written or oral exams, laboratory evaluations) because it is not possible to find just one tool that is able to assess all the competences.33,34<br>From conclusion: In conclusion, the portfolio is an important tool that has allowed to obtain qualitative and quantitative data regarding the degree of acquisition of traumatology competences by medical students. Although some factors need to be improved, such as the

For peer review only

No.	Title	Author/year	MERSQI	COREQ	Reason for inclusion			Undergraduate (UG)/Postgraduate (PG)	Methodology	Purpose of study	Key findings
					Communication	Ethics	Professionalism				
											inclusion of more quantitative elements, a more comprehensive selection of learning outcomes, and combination with other tools, we believe that the use of this kind of tool by clinical teachers will be useful to ascertain the degree of practical competence reached by their students.
77	Assessing the Development of Medical Students' Personal and Professional Skills by Portfolio	Yielder, J. and Moir, F. 2016		6			✓	UG	The implementation process employed a cyclical model that was designed to evaluate and improve the use of the portfolio, that could form the basis of the first cycle of action research that will be continued in the future to improve the initiative (Fig. 1). During 2013, the introduction of the portfolio was monitored, and changes were made in 2014. In 2014, feedback from students indicated the need for further modifications, which are currently being implemented, along with the introduction of an electronic platform. Student feedback was ascertained from the generic end-of-year course evaluation, which included	From abstract: We present the portfolio format along with the process for its introduction and appraise the challenges, strengths, and limitations of the approach within the context of the current literature. We then outline a cyclical model of evaluation used to monitor and fine-tune the portfolio tasks and implementation process, in response to student and assessor feedback. From introduction: This document outlines the learning outcomes that are to be substantively completed by the end of the second postgraduate year in practice. ... This article presents the process for introducing the portfolio, including the challenges, strengths and limitations, evaluation of data to date, and the ways in which student	From abstract: The portfolios have illustrated the level of insight, maturity, and synthesis of personal and professional qualities that students are capable of achieving. The Auckland medical program strives to foster these qualities in its students, and the portfolio provides an opportunity for students to demonstrate their reflective abilities. Moreover, the creation of a Personal and Professional Skills domain with the portfolio as its key assessment emphasizes the importance of reflective practice and personal and professional development and gives a clear message that these are fundamental longitudinal elements of the program. From conclusion: The implementation of a portfolio for the assessment of the PPS domain in the medical program at the University of Auckland has proved to be an important part of the redeveloped curriculum. It has highlighted the importance of PPS topics and has provided an opportunity and incentive for students to reflect on aspects of their personal and professional development, with the aim

No.	Title	Author/year	MERSQI	COREQ	Reason for inclusion			Undergraduate (UG)/Postgraduate (PG)	Methodology	Purpose of study	Key findings
					Communication	Ethics	Professionalism				
									questions relating to the PPS domain. The number of students in each year varied from 199–260 and there was an average response rate of 50%. In addition, the Auckland University Medical Students Association conducted a student evaluation for the purpose of writing a report for the ongoing medical program accreditation, from which key issues were collated and forwarded to the PPS domain coordinators. The eight portfolio assessors also provided suggestions for improvement at the end of 2013 and 2014. Evaluation feedback, modifications, and qualitative data derived from the portfolios are presented in the Findings section. Due to the qualitative nature of the feedback, it was analyzed inductively, through a cross-sectional thematic analysis that sought to	feedback has been addressed.	of improving their practice as doctors. In the process, we have learned several aspects about students that previously may have remained unknown, which has led in some cases to conversations with students that will hopefully make a difference to their learning and development trajectories. We have also seen many high-quality and insightful portfolios that demonstrate a synthesis of personal and professional qualities that are inspiring and which we hope are an indication that these students will develop into excellent doctors. With the increasing use of, and requirement for, the compilation of professional portfolios in the medical profession internationally, especially at postgraduate level, there is real benefit for students in becoming familiar with this process of pregraduation.<br>It has also been a learning process for the domain coordinators introducing the assessment. In particular, we learned the value of a cyclic, flexible, and ongoing process of evaluation that has allowed us to continuously reflect on what is working and what needs to change in order to be responsive to student needs and at the same time create a curriculum change that aims to create better doctors. ... We hope that as students come to trust our responsiveness, they will become increasingly open to the importance of taking on the responsibility for their

For peer review only - http://bmjopen.bmj.com/site/about/guidelines.xhtml

No.	Title	Author/year	MERSQI	COREQ	Reason for inclusion			Undergraduate (UG)/Postgraduate (PG)	Methodology	Purpose of study	Key findings
					Communication	Ethics	Professionalism				
									ascertain the strongest themes from across the three sources of feedback.		own learning in the PPS domain.
78	Construct Validity of an Instrument for Assessment of Reflective Writing-Based Portfolios of Medical Students	Kassab, Salah Bidmos, Mubarak Nomikos, Michail Daher-Nashif, Suhad Kane, Tanya Sarangi, Srikant Abu-Hijleh, Marwan<br><br>2020	7		✓		✓	UG	After an extensive literature review and pilot testing of the instrument, two raters assessed the reflective writing-based portfolios from years 2 and 3 medical students (n=135) on three occasions. The instrument consists of three criteria: organization, description of an experience and reflection on the experience. We calculated the reliability of scores using generalizability theory with a fully crossed design and two facets (raters and occasions). In addition, we measured criterion validity by testing correlations with students' scores using other assessment methods.	Assessment of reflective writing for medical students is challenging, and there is lack of an available instrument with good psychometric properties. The authors developed a new instrument for assessment of reflective writing-based portfolios and examined the construct validity of this instrument.	The dependability (Φ) coefficient of the portfolio scores was 0.75 using two raters on three occasions. Students' portfolio scores represented 46.6% of the total variance across all score comparisons. The variance due to occasions was negligible, while the student– occasion interaction was small. The variance due to student–rater interaction represented 17.7%, and the remaining 27.7% of the variance was due to unexplained sources of error. The decision (D) study suggested that an acceptable dependability (Φ = 0.70 and 0.72) can be achieved by using two raters for one and two occasions, respectively. Finally, we found moderate to large effect-size correlations between students' scores in reflective writing-based portfolios and communication skills (r = 0.47) and PBL tutorials (r = 0.50).<br><br>We demonstrated the presence of different sources of evidence that support construct validity of the study instrument. Further studies are warranted before utilizing this instrument for summative assessment of students' reflective writing-based portfolios in other medical schools.
79	Communication skills teaching	Douglas, A. H. Acharya, S. P.		19	✓			UG	This study is a qualitative	Communication skills (194) are vital for	The two main themes: 1. Positivity

For peer review only

No.	Title	Author/year	MERSQI	COREQ	Reason for inclusion			Undergraduate (UG)/Postgraduate (PG)	Methodology	Purpose of study	Key findings
					Communication	Ethics	Professionalism				
	and learning in Nepal; what are medical students' perceptions and experiences? A qualitative study	Allery, L. A.<br><br>2020							evaluation of a CS course in Nepal, exploring the experiences and perceptions of participants. The study aims to also identify aspects that were helpful or not for student learning and areas for potential development.<br><br>A purposive sample of twenty: second, fourth and Intern year students was selected for interview. Data were collected through audio recorded semi-structured interviews following a piloted schedule. Interview transcripts were manually coded and thematically analysed. Codes were arranged into themes and subthemes.	doctors. Indeed, as the most important element of consultations, are highly valued by patients. CS are core, teachable skills, however, have not been widely taught in South Asian medical schools, unlike their western counterparts.<br><br>Patan Academy of Health Sciences, is one of the first in Nepal to have CS central to its' aims and curriculum. CS are taught from the first weeks of medical school and re-enforced during preclinical study (first 2 years). Our study seeks to explore students' perceptions and experiences of CS teaching in this South Asian, Nepal context.	2. Experiential learning. Results demonstrate participants' positive perceptions of CS teaching: believing it is important, effective, relevant and valuable for personal development. Participants identified experiential learning features as valuable for CS acquisition. Intern students recognised CS relevance and requested expanding teaching to clinical years, incorporating challenging communication scenarios.<br><br>This study shows that PAHS' CS course is well perceived and valuable to learners. Experiential learning is powerful for CS development. Expansion of formal, structured CS teaching through all years in a spiral curriculum, should be considered. Violence towards doctors in South Asia is increasing. Students recognised CS teaching's significance in addressing this.<br><br>CS teaching,still in its' infancy in South Asia, is a pressing issue for medical educators here. Our study provides evidence it is well perceived with positive impacts in this context, particularly when employing experiential learning. Medical schools in south Asia should be encouraged to incorporate and strengthen their CS teaching curriculum.
80	Assessment practices in undergraduate	Brits, H.<br>Bezuidenhout, J.<br>Van der Merwe, L. J.	5.5	12	✓			UG	A descriptive cross-sectional study design was	Assessment should form an integral part of curriculum design	All disciplines in the undergraduate medical programme are

For peer review only - http://bmjopen.bmj.com/site/about/guidelines.xhtml

No.	Title	Author/year	MERSQI	COREQ	Reason for inclusion			Undergraduate (UG)/Postgraduate (PG)	Methodology	Purpose of study	Key findings
					Communication	Ethics	Professionalism				
	clinical medicine training: What do we do and how can we improve?	Joubert, G.<br><br>2020							used. Qualitative and quantitative data were gathered by means of open- and closed-ended questions in a self-administered questionnaire, which was completed by teaching and learning coordinators in 13 disciplines.	in higher education and should be robust enough to ensure clinical competence.<br><br>This article reports on current assessment practices and makes recommendations to improve clinical assessment in the undergraduate medical programme at the University of the Free State.	represented. They used different assessment methods to assess the competencies required of entry-level healthcare professionals. Workplace-based assessment was performed by 30.1% of disciplines, while multiple-choice questions (MCQs) (76.9%) and objective structured clinical examinations (OSCEs) (53.6%) were the main methods used during formative assessment. Not all assessors were well prepared for assessment, with 38.5% never having received any formal training on assessment. Few disciplines (15.4%) made use of post-assessment moderation as a standard practice, and few disciplines always gave feedback after assessments.<br><br>The current assessment practices for clinical students in the undergraduate medical programme at the University of the Free State cover the spectrum that is necessary to assess all the different competencies required. Multiple-choice questions and OSCEs, which are valid and reliable assessment methods, are used frequently. Poor feedback and moderation practices should be addressed. More formative assessments, and less emphasis on summative assessment, should be considered. Workplace-based and continuous assessments may be good ways to assess clinical competence.

For peer review only

No.	Title	Author/year	MERSQI	COREQ	Reason for inclusion			Undergraduate (UG)/Postgraduate (PG)	Methodology	Purpose of study	Key findings
					Communication	Ethics	Professionalism				
81	**Added value of assessing medical students' reflective writings in communication skills training: a longitudinal study in four academic centres**	Ament Giuliani Franco, C.<br>Franco, R. S.<br>Cecilio-Fernandes, D.<br>Severo, M.<br>Ferreira, M. A.<br>de Carvalho-Filho, M. A.<br><br>2020	10.5		✓			UG	Third-year and fourth-year medical students enrolled in an elective course on clinical communication skills development were assessed using different assessment methods.<br><br>The communication skills course was offered at four universities (three in Brazil and one in Portugal) and included 69 students.<br><br>The students were assessed by a Multiple-Choice Questionnaire (MCQ), an objective structured clinical examination (OSCE) and reflective writing narratives. The Cronbach's alpha, dimensionality and the person's correlation were applied to evaluate the reliability of the assessment methods and their correlations. Reflective witting was assessed by applying the Reflection Evaluation for Enhanced Competencies Tool Rubric	This study describes the development and implementation of a model to assess students' communication skills highlighting the use of reflective writing. We aimed to evaluate the usefulness of the students' reflections in the assessment of communication skills.	The Cronbach alpha for the MCQ, OSCE global score, TS and RS were, respectively, 0.697, 0.633, 0.784 and 0.850. The interobserver correlation for the TS and RS were, respectively, 0.907 and 0.816. The assessment of reflection using the TS was significantly correlated with the MCQ (r=0.412; p=0.019), OSCE (0.439; p=0.012) and RS (0.410; p=0.020). The RS did not correlate with the MCQ and OSCE.<br><br>Assessing reflection through mapping the themes and analysing the depth of reflective writing expands the assessment of communication skills. While the assessment of reflective themes is related to the cognitive and behavioural domains of learning, the reflective depth seems to be a specific competence, not correlated with other assessment methods—possibly a metacognitive domain.

For peer review only

No.	Title	Author/year	MERSQI	COREQ	Reason for inclusion			Undergraduate (UG)/Postgraduate (PG)	Methodology	Purpose of study	Key findings
					Communication	Ethics	Professionalism				
									(Reflect Score (RS)) to measure reflections' depth, and the Thematic Score (TS) to map and grade reflections' themes.		
82	Contextual Analysis of Stakeholder Opinion on Management and Leadership Competencies for Undergraduate Medical Education: Informing Course Design	Rajeh, N.<br>Grant, J.<br>Farsi, J.<br>Tekian, A.<br><br>2020		16	✓			UG	This was a cross-sectional study conducted at King Abdulaziz University (KAU), Saudi Arabia, during 2019. An exploratory qualitative approach, utilizing systematic content analysis, was used. Data were collected using semi-structured interviews that were con- ducted with 10 leaders who were stakeholders at KAU, health service providers at KAU hospital, and stakeholders in the Ministry of Health.	The study aimed to conduct a contextual analysis of interviews intended to assist with the future design of a feasible and relevant leadership and management course for undergraduate medical students at King Abdulaziz University (KAU), Saudi Arabia.	This study revealed critical findings that highlighted the areas in which KAU could instill better and adequate leadership and management skills in their undergraduate medical students. Multiple core categories for a leadership and management curriculum emerged with many interrelated themes. Most participants mentioned that leadership can be taught and that early exposure is beneficial for develop- ing skills. Additionally, they stated that leaders should have a vision and the ability to articulate that vision.<br><br>Different implementation challenges were described in relation to the availability of human resources, the current short supply of suitable teachers, and program design. Teaching methods recommended included simulations, lectures, and a project-based approach. Assessment methods that were recommended included objective structured clinical examination (OSCE), formative and summa- tive assessments, self-assessments, and portfolios.

For peer review only

Appendix C

Portfolio Content (Competencies Assessed and Assessment Modalities)

Subthemes	Content		
	**Communication**	**Ethics**	**Professionalism**
**Competencies Assessed**	• "Effective" communication (65, 67, 68, 81, 86, 107, 127, 131-133)<br>• Professional behaviour and moral reasoning (71)<br>• Systems awareness and team-based care (71)<br>• Continuous learning and quality improvement (71)<br>• Patient-centred medical care (71)<br>• Teamwork (70)<br><br>Oral communication skills<br>• Attentive, active listening (84, 139, 140)<br>• Explanation, information giving (127, 139)<br>• History taking (93)<br>• Consultation (141)<br>• Breaking bad news (24, 139)<br>• Shared decision making (142)<br>• Empathetic communication (142)<br>• Taking history and clinical examination (84, 93)<br>• Discerning appropriate language to ensure patient understands (24, 127)<br>• Conflict management (139)<br>• Adaptability to different settings (67)<br><br>Written communication skills<br>• Proper grammar, spelling, logical and coherent structure (103)<br>• Patient cases (93)<br>• Scientific research (93)<br><br>Communication with:<br>• Patients (24, 68, 84, 93, 127, 139, 143)<br>• Children (127)	• Ethics in interprofessional practice (24, 67, 86, 128, 130, 145, 146)<br>• Doctor-patient relationship (81, 141, 145)<br>• Values/ ethics (67, 70, 86, 146)<br>• Ethical and legal responsibilities (65, 86)<br>• Moral reasoning (89)<br>• Ethics is assessed as a component of professionalism (85, 87, 88)<br>• Empathy (84)<br>• Showing respect and commitment (84)<br>• Respect for privacy (84)<br>• How to express ethical views appropriately (82)<br>• Identifying unethical moments of practice and grey areas (85, 89, 90)<br><br>Reflection:<br>• Quality of student's reflections (82, 84, 87)<br>• Authenticity of reflection (87)	• Learning from clinical experience (24, 81)<br>• Working in groups/ teamwork (24, 67, 70, 81, 86, 128, 130, 134, 145)<br>• Doctor-patient relationship (81, 141, 145)<br>• Becoming a doctor/roles and responsibilities (81, 86, 128, 130, 134, 145, 146)<br>• Patient care (67, 70, 128, 130)<br>• Continuous learning (67, 70)<br>• Values/ ethics (67, 70, 86, 146)<br>• Interpersonal skills/ effective Communication, (24, 67, 128, 145, 146)<br><br>Reflection:<br>• Clinical experience (81)<br>• Teaching (81)<br>• Learning needs in becoming a doctor<br>• Strengths and developmental areas (65, 86)<br>• Understanding of concepts addressed (86)<br>• Dilemmas and doubts (133)

For peer review only

	• Family (68, 84, 139, 144)<br>• Colleagues, interprofessional team (24, 68, 84, 93, 130, 139)<br>• Public (68)		
**Assessment Modalities (Portfolio items)**	Formative only (67, 84, 87, 92, 119, 126, 130, 146)<br>Summative only (65, 70, 71, 81-83, 87, 104, 116, 131, 134)<br>Both formative and summative (24, 67, 83, 86, 87, 117, 122, 128, 130, 134)<br>MCQs (65, 68, 87, 95, 126, 141, 143, 144, 146)<br>OSCEs (65, 67, 70, 71, 87, 126, 127, 130, 141, 143, 144, 146)<br>Essays – topical (67, 81, 93, 126)<br>Mini clinical examination exercise (Mini-CEX) (65, 67, 70, 87, 93, 130, 139, 143, 146)<br>Medical/patient presentations/research (67, 68, 80, 86, 93, 121, 126, 129)<br>Workplace-based clinical (67, 70, 71, 80, 83, 93, 107, 112, 122, 126, 127, 130, 139, 143)<br>Case-based discussions, summaries (65, 68, 83, 85, 89, 92, 129)(19)(81, 129) (83, 126, 130)<br>Simulated patient assessment (65, 68, 84, 103, 112)<br>Team-based assessment (65, 83, 86, 93)<br>Logbooks/ Portfolio interview (65, 67, 68, 70, 81-85, 87, 88, 103, 121, 126) (67, 121, 126)<br>Peer evaluation (67, 70, 71, 80, 85, 93, 107, 126, 127)<br>Faculty evaluation (67, 68, 70, 71, 80, 81, 83-86, 117, 126, 131, 147)<br>Feedback reports (75, 77, 80, 86, 91, 93, 109, 112, 122, 126, 130, 134, 139, 143) – multisource feedback (75, 77, 81, 82, 85, 86, 91, 93, 103, 109, 127, 130)<br>Self-assessment/appraisal (67, 68, 80, 82, 93, 104, 126, 130, 134)<br>Self-reflections – written/video/diaries (65, 67, 68, 71, 80-83, 85, 87, 88, 90, 93, 103, 107, 121, 127-130)<br>Learning plans and contracts (67, 68, 71, 80-83, 86, 92, 93, 103, 133)<br>Letters of recommendation (65, 81, 103)<br>Curriculum vitae (65, 67, 70, 83, 86, 122, 126)		

For peer review only

Preferred Reporting Items for Systematic reviews and Meta-Analyses extension for Scoping Reviews (PRISMA-ScR) Checklist

SECTION	ITEM	PRISMA-ScR CHECKLIST ITEM	REPORTED ON PAGE #
**TITLE**			
Title	1	Identify the report as a scoping review.	1
**ABSTRACT**			
Structured summary	2	Provide a structured summary that includes (as applicable): background, objectives, eligibility criteria, sources of evidence, charting methods, results, and conclusions that relate to the review questions and objectives.	2
**INTRODUCTION**			
Rationale	3	Describe the rationale for the review in the context of what is already known. Explain why the review questions/objectives lend themselves to a scoping review approach.	4
Objectives	4	Provide an explicit statement of the questions and objectives being addressed with reference to their key elements (e.g., population or participants, concepts, and context) or other relevant key elements used to conceptualize the review questions and/or objectives.	4
**METHODS**			
Protocol and registration	5	Indicate whether a review protocol exists; state if and where it can be accessed (e.g., a Web address); and if available, provide registration information, including the registration number.	5
Eligibility criteria	6	Specify characteristics of the sources of evidence used as eligibility criteria (e.g., years considered, language, and publication status), and provide a rationale.	5
Information sources*	7	Describe all information sources in the search (e.g., databases with dates of coverage and contact with authors to identify additional sources), as well as the date the most recent search was executed.	5
Search	8	Present the full electronic search strategy for at least 1 database, including any limits used, such that it could be repeated.	5
Selection of sources of evidence†	9	State the process for selecting sources of evidence (i.e., screening and eligibility) included in the scoping review.	5
Data charting process‡	10	Describe the methods of charting data from the included sources of evidence (e.g., calibrated forms or forms that have been tested by the team before their use, and whether data charting was done independently or in duplicate) and any processes for obtaining and confirming data from investigators.	6
Data items	11	List and define all variables for which data were sought and any assumptions and simplifications made.	7
Critical appraisal of individual sources of evidence§	12	If done, provide a rationale for conducting a critical appraisal of included sources of evidence; describe the methods used and how this information was used in any data synthesis (if appropriate).	7

For peer review only - http://bmjopen.bmj.com/site/about/guidelines.xhtml

SECTION	ITEM	PRISMA-ScR CHECKLIST ITEM	REPORTED ON PAGE #
Synthesis of results	13	Describe the methods of handling and summarizing the data that were charted.	8
**RESULTS**			
Selection of sources of evidence	14	Give numbers of sources of evidence screened, assessed for eligibility, and included in the review, with reasons for exclusions at each stage, ideally using a flow diagram.	8
Characteristics of sources of evidence	15	For each source of evidence, present characteristics for which data were charted and provide the citations.	8
Critical appraisal within sources of evidence	16	If done, present data on critical appraisal of included sources of evidence (see item 12).	Appendix B
Results of individual sources of evidence	17	For each included source of evidence, present the relevant data that were charted that relate to the review questions and objectives.	Appendix B
Synthesis of results	18	Summarize and/or present the charting results as they relate to the review questions and objectives.	8
**DISCUSSION**			
Summary of evidence	19	Summarize the main results (including an overview of concepts, themes, and types of evidence available), link to the review questions and objectives, and consider the relevance to key groups.	9-13
Limitations	20	Discuss the limitations of the scoping review process.	15
Conclusions	21	Provide a general interpretation of the results with respect to the review questions and objectives, as well as potential implications and/or next steps.	15
**FUNDING**			
Funding	22	Describe sources of funding for the included sources of evidence, as well as sources of funding for the scoping review. Describe the role of the funders of the scoping review.	None

JBI = Joanna Briggs Institute; PRISMA-ScR = Preferred Reporting Items for Systematic reviews and Meta-Analyses extension for Scoping Reviews.
* Where *sources of evidence* (see second footnote) are compiled from, such as bibliographic databases, social media platforms, and Web sites.
† A more inclusive/heterogeneous term used to account for the different types of evidence or data sources (e.g., quantitative and/or qualitative research, expert opinion, and policy documents) that may be eligible in a scoping review as opposed to only studies. This is not to be confused with *information sources* (see first footnote).
‡ The frameworks by Arksey and O'Malley (6) and Levac and colleagues (7) and the JBI guidance (4, 5) refer to the process of data extraction in a scoping review as data charting.
§ The process of systematically examining research evidence to assess its validity, results, and relevance before using it to inform a decision. This term is used for items 12 and 19 instead of "risk of bias" (which is more applicable to systematic reviews of interventions) to include and acknowledge the various sources of evidence that may be used in a scoping review (e.g., quantitative and/or qualitative research, expert opinion, and policy document).

From: Tricco AC, Lillie E, Zarin W, O'Brien KK, Colquhoun H, Levac D, et al. PRISMA Extension for Scoping Reviews (PRISMAScR): Checklist and Explanation. Ann Intern Med. 2018;169:467–473. doi: 10.7326/M18-0850.

For peer review only - http://bmjopen.bmj.com/site/about/guidelines.xhtml
